# Supplementary material for: Alkaliphilic/Alkali-Tolerant Fungi: Molecular, Biochemical, and Biotechnological Aspects
Source: J Fungi (Basel). 2023 Jun 9;9(6):652. doi: 10.3390/jof9060652 (PMC10301932; doi:10.3390/jof9060652)
Supplement: Supplementary file 1 [file jof-09-00652-s001.zip › S2/html/input.path1.gene3_searchgtr.html]

Search Query


## **SEARCHGTr input**

**Sequence name:**    
FASTA-formatted sequence:   
>input.path1.gene3
MMNDGMTRVTRLLWPGRVFPDFEVYEVQVRRGEWYEAKHDINHHGSETCPSSALSDISALRPRVESVKTVRNERLSQIDVATMAPPPSRRRSSVATSAYRFRKMSIMHEQGNTFGPSLTTLYCGISAVFADDQTAVVAIAIHDTVYLIDYSVKHVVLGDALHMGQDAIADYVLSTLEVYEHDNFSKFIGAGLPITLKYMSPTLCSRLWLDMDIVPVVTRPDEDGKRENFWDIKRVDEQADSMARKCIMNFGPSLVPHLQVGFRGVVQTDAGFRVHLVTPQNHKDACGRPTWDSMAHYATKLRRNKIRVAFFSSTPQGGGVALMRHALVRFARLMGVDLTWYVPKPRPGVFRITKTMHNILQGVSHPEQRISAEEKQSIHDWITDNAYRYWLSEGGPLRPVEEGGAHVVIMPGLIPLIKKLTPNRPVLYRSHIQIRSDLTAMAGSPQADVWDFLWNNIQKADMFISHPIPLFVPHNVPHEKVAYMPATTDWLDGLNKPLSTWDMGYYGHIYNTACLHQRMAELQWPTRKYIVQVARFDPSKGIPTVIDAYGEFRRLLEEAGVEDSPQLVVCGNGSVDDPDASMIFDETMTQLETFYPHLVDDCSVMRLDPNDQLLNTLIRSSHVTLQLSTREGFEVKVSEALHAGRPVIASRAGGIPLQIKDQVNGFLVEPGDWKAVAQHLMDLFTDDELHARMSHAAATGVSDEVGTAGNALCWFYLASKWAEVGVKPGLPGNERWVNDMAREEAGKPYNESENRLPRHFTQRKERPVVVVKVERGEDGEKAENGENGE  
  
