# Supplementary material for: Alkaliphilic/Alkali-Tolerant Fungi: Molecular, Biochemical, and Biotechnological Aspects
Source: J Fungi (Basel). 2023 Jun 9;9(6):652. doi: 10.3390/jof9060652 (PMC10301932; doi:10.3390/jof9060652)
Supplement: Supplementary file 1 [file jof-09-00652-s001.zip › S2/index.html]

NITX01 - 24 region(s) - antiSMASH results


antiSMASH version 7.0.0beta2-86685d9d

Download

- Download all results
- Download GenBank summary file
- Download log file

About

Help

Contact

Select genomic region:

Overview

26.1

61.1

90.1

104.1

113.1

125.1

139.1

151.1

158.1

170.1

191.1

191.2

205.1

233.1

351.1

352.1

393.1

453.1

496.1

498.1

526.1

531.1

571.1

596.1

### Identified secondary metabolite regions using strictness 'relaxed'

**ENANITX01000026NITX01000026.1** (original name was: ENA|NITX01000026|NITX01000026.1)

| Region | Type | From | To | Most similar known cluster | | Similarity |
| --- | --- | --- | --- | --- | --- | --- |
| Region&nbsp26.1 | T3PKS | 1 | 35,100 |  | | |

**ENANITX01000062NITX01000062.1** (original name was: ENA|NITX01000062|NITX01000062.1)

| Region | Type | From | To | Most similar known cluster | | Similarity |
| --- | --- | --- | --- | --- | --- | --- |
| Region&nbsp61.1 | T1PKS | 124,154 | 192,305 |  | | |

**ENANITX01000091NITX01000091.1** (original name was: ENA|NITX01000091|NITX01000091.1)

| Region | Type | From | To | Most similar known cluster | | Similarity |
| --- | --- | --- | --- | --- | --- | --- |
| Region&nbsp90.1 | NRPS | 24,062 | 99,877 |  | | |

**ENANITX01000105NITX01000105.1** (original name was: ENA|NITX01000105|NITX01000105.1)

| Region | Type | From | To | Most similar known cluster | | Similarity |
| --- | --- | --- | --- | --- | --- | --- |
| Region&nbsp104.1 | fungal-RiPP-like | 195,570 | 242,827 |  | | |

**ENANITX01000114NITX01000114.1** (original name was: ENA|NITX01000114|NITX01000114.1)

| Region | Type | From | To | Most similar known cluster | | Similarity |
| --- | --- | --- | --- | --- | --- | --- |
| Region&nbsp113.1 | NRPS | 7,235 | 47,523 | metachelin C/metachelin A/metachelin A-CE/metachelin B/dimerumic acid 11-mannoside/dimerumic acid | NRP | 87% |

**ENANITX01000126NITX01000126.1** (original name was: ENA|NITX01000126|NITX01000126.1)

| Region | Type | From | To | Most similar known cluster | | Similarity |
| --- | --- | --- | --- | --- | --- | --- |
| Region&nbsp125.1 | terpene | 67,494 | 98,976 |  | | |

**ENANITX01000140NITX01000140.1** (original name was: ENA|NITX01000140|NITX01000140.1)

| Region | Type | From | To | Most similar known cluster | | Similarity |
| --- | --- | --- | --- | --- | --- | --- |
| Region&nbsp139.1 | T1PKS | 1 | 50,812 | solanapyrone A | Polyketide | 60% |

**ENANITX01000152NITX01000152.1** (original name was: ENA|NITX01000152|NITX01000152.1)

| Region | Type | From | To | Most similar known cluster | | Similarity |
| --- | --- | --- | --- | --- | --- | --- |
| Region&nbsp151.1 | T1PKS | 1 | 28,408 | depudecin | Polyketide:Iterative type I polyketide | 33% |

**ENANITX01000159NITX01000159.1** (original name was: ENA|NITX01000159|NITX01000159.1)

| Region | Type | From | To | Most similar known cluster | | Similarity |
| --- | --- | --- | --- | --- | --- | --- |
| Region&nbsp158.1 | T1PKS | 1 | 65,417 |  | | |

**ENANITX01000171NITX01000171.1** (original name was: ENA|NITX01000171|NITX01000171.1)

| Region | Type | From | To | Most similar known cluster | | Similarity |
| --- | --- | --- | --- | --- | --- | --- |
| Region&nbsp170.1 | NRPS,T1PKS | 1 | 21,602 |  | | |

**ENANITX01000192NITX01000192.1** (original name was: ENA|NITX01000192|NITX01000192.1)

| Region | Type | From | To | Most similar known cluster | | Similarity |
| --- | --- | --- | --- | --- | --- | --- |
| Region&nbsp191.1 | NRPS,T1PKS | 36,437 | 107,835 |  | | |
| Region&nbsp191.2 | NRPS-like | 125,340 | 188,211 |  | | |

**ENANITX01000206NITX01000206.1** (original name was: ENA|NITX01000206|NITX01000206.1)

| Region | Type | From | To | Most similar known cluster | | Similarity |
| --- | --- | --- | --- | --- | --- | --- |
| Region&nbsp205.1 | terpene | 13,911 | 47,643 | squalestatin S1 | Terpene | 40% |

**ENANITX01000234NITX01000234.1** (original name was: ENA|NITX01000234|NITX01000234.1)

| Region | Type | From | To | Most similar known cluster | | Similarity |
| --- | --- | --- | --- | --- | --- | --- |
| Region&nbsp233.1 | T1PKS | 4,133 | 54,076 | flavoglaucin | Polyketide | 25% |

**ENANITX01000352NITX01000352.1** (original name was: ENA|NITX01000352|NITX01000352.1)

| Region | Type | From | To | Most similar known cluster | | Similarity |
| --- | --- | --- | --- | --- | --- | --- |
| Region&nbsp351.1 | T1PKS | 132,661 | 201,094 | HEx-pks1 polyketide | Polyketide | 80% |

**ENANITX01000353NITX01000353.1** (original name was: ENA|NITX01000353|NITX01000353.1)

| Region | Type | From | To | Most similar known cluster | | Similarity |
| --- | --- | --- | --- | --- | --- | --- |
| Region&nbsp352.1 | NRPS | 3,059 | 71,736 |  | | |

**ENANITX01000394NITX01000394.1** (original name was: ENA|NITX01000394|NITX01000394.1)

| Region | Type | From | To | Most similar known cluster | | Similarity |
| --- | --- | --- | --- | --- | --- | --- |
| Region&nbsp393.1 | NRPS-like | 142,565 | 203,866 | choline | NRP | 100% |

**ENANITX01000454NITX01000454.1** (original name was: ENA|NITX01000454|NITX01000454.1)

| Region | Type | From | To | Most similar known cluster | | Similarity |
| --- | --- | --- | --- | --- | --- | --- |
| Region&nbsp453.1 | NRPS,T1PKS | 40,840 | 114,672 |  | | |

**ENANITX01000499NITX01000499.1** (original name was: ENA|NITX01000499|NITX01000499.1)

| Region | Type | From | To | Most similar known cluster | | Similarity |
| --- | --- | --- | --- | --- | --- | --- |
| Region&nbsp496.1 | NRPS | 5,178 | 77,072 | penicillin | NRP | 12% |

**ENANITX01000501NITX01000501.1** (original name was: ENA|NITX01000501|NITX01000501.1)

| Region | Type | From | To | Most similar known cluster | | Similarity |
| --- | --- | --- | --- | --- | --- | --- |
| Region&nbsp498.1 | terpene | 1 | 17,212 |  | | |

**ENANITX01000529NITX01000529.1** (original name was: ENA|NITX01000529|NITX01000529.1)

| Region | Type | From | To | Most similar known cluster | | Similarity |
| --- | --- | --- | --- | --- | --- | --- |
| Region&nbsp526.1 | T1PKS | 1 | 43,426 |  | | |

**ENANITX01000534NITX01000534.1** (original name was: ENA|NITX01000534|NITX01000534.1)

| Region | Type | From | To | Most similar known cluster | | Similarity |
| --- | --- | --- | --- | --- | --- | --- |
| Region&nbsp531.1 | T1PKS,NRPS-like | 106,513 | 158,980 |  | | |

**ENANITX01000574NITX01000574.1** (original name was: ENA|NITX01000574|NITX01000574.1)

| Region | Type | From | To | Most similar known cluster | | Similarity |
| --- | --- | --- | --- | --- | --- | --- |
| Region&nbsp571.1 | T1PKS | 26,928 | 72,293 |  | | |

**ENANITX01000600NITX01000600.1** (original name was: ENA|NITX01000600|NITX01000600.1)

| Region | Type | From | To | Most similar known cluster | | Similarity |
| --- | --- | --- | --- | --- | --- | --- |
| Region&nbsp596.1 | T1PKS | 69,838 | 105,701 | cryptosporioptide B/cryptosporioptide A/cryptosporioptide C | Polyketide:Iterative type I polyketide | 19% |

| Region | Type | From | To | Most similar known cluster | | Similarity |
| --- | --- | --- | --- | --- | --- | --- |
| Region&nbsp26.1 | T3PKS | 1 | 35,100 |  | | |
| Region&nbsp61.1 | T1PKS | 124,154 | 192,305 |  | | |
| Region&nbsp90.1 | NRPS | 24,062 | 99,877 |  | | |
| Region&nbsp104.1 | fungal-RiPP-like | 195,570 | 242,827 |  | | |
| Region&nbsp113.1 | NRPS | 7,235 | 47,523 | metachelin C/metachelin A/metachelin A-CE/metachelin B/dimerumic acid 11-mannoside/dimerumic acid | NRP | 87% |
| Region&nbsp125.1 | terpene | 67,494 | 98,976 |  | | |
| Region&nbsp139.1 | T1PKS | 1 | 50,812 | solanapyrone A | Polyketide | 60% |
| Region&nbsp151.1 | T1PKS | 1 | 28,408 | depudecin | Polyketide:Iterative type I polyketide | 33% |
| Region&nbsp158.1 | T1PKS | 1 | 65,417 |  | | |
| Region&nbsp170.1 | NRPS,T1PKS | 1 | 21,602 |  | | |
| Region&nbsp191.1 | NRPS,T1PKS | 36,437 | 107,835 |  | | |
| Region&nbsp191.2 | NRPS-like | 125,340 | 188,211 |  | | |
| Region&nbsp205.1 | terpene | 13,911 | 47,643 | squalestatin S1 | Terpene | 40% |
| Region&nbsp233.1 | T1PKS | 4,133 | 54,076 | flavoglaucin | Polyketide | 25% |
| Region&nbsp351.1 | T1PKS | 132,661 | 201,094 | HEx-pks1 polyketide | Polyketide | 80% |
| Region&nbsp352.1 | NRPS | 3,059 | 71,736 |  | | |
| Region&nbsp393.1 | NRPS-like | 142,565 | 203,866 | choline | NRP | 100% |
| Region&nbsp453.1 | NRPS,T1PKS | 40,840 | 114,672 |  | | |
| Region&nbsp496.1 | NRPS | 5,178 | 77,072 | penicillin | NRP | 12% |
| Region&nbsp498.1 | terpene | 1 | 17,212 |  | | |
| Region&nbsp526.1 | T1PKS | 1 | 43,426 |  | | |
| Region&nbsp531.1 | T1PKS,NRPS-like | 106,513 | 158,980 |  | | |
| Region&nbsp571.1 | T1PKS | 26,928 | 72,293 |  | | |
| Region&nbsp596.1 | T1PKS | 69,838 | 105,701 | cryptosporioptide B/cryptosporioptide A/cryptosporioptide C | Polyketide:Iterative type I polyketide | 19% |

No secondary metabolite regions were found in these records:
:   **ENANITX01000001NITX01000001.1** (original name was: ENA|NITX01000001|NITX01000001.1)
:   **ENANITX01000002NITX01000002.1** (original name was: ENA|NITX01000002|NITX01000002.1)
:   **ENANITX01000003NITX01000003.1** (original name was: ENA|NITX01000003|NITX01000003.1)
:   **ENANITX01000004NITX01000004.1** (original name was: ENA|NITX01000004|NITX01000004.1)
:   **ENANITX01000005NITX01000005.1** (original name was: ENA|NITX01000005|NITX01000005.1)
:   **ENANITX01000006NITX01000006.1** (original name was: ENA|NITX01000006|NITX01000006.1)
:   **ENANITX01000007NITX01000007.1** (original name was: ENA|NITX01000007|NITX01000007.1)
:   **ENANITX01000008NITX01000008.1** (original name was: ENA|NITX01000008|NITX01000008.1)
:   **ENANITX01000009NITX01000009.1** (original name was: ENA|NITX01000009|NITX01000009.1)
:   **ENANITX01000010NITX01000010.1** (original name was: ENA|NITX01000010|NITX01000010.1)
:   **ENANITX01000011NITX01000011.1** (original name was: ENA|NITX01000011|NITX01000011.1)
:   **ENANITX01000012NITX01000012.1** (original name was: ENA|NITX01000012|NITX01000012.1)
:   **ENANITX01000013NITX01000013.1** (original name was: ENA|NITX01000013|NITX01000013.1)
:   **ENANITX01000014NITX01000014.1** (original name was: ENA|NITX01000014|NITX01000014.1)
:   **ENANITX01000015NITX01000015.1** (original name was: ENA|NITX01000015|NITX01000015.1)
:   **ENANITX01000016NITX01000016.1** (original name was: ENA|NITX01000016|NITX01000016.1)
:   **ENANITX01000017NITX01000017.1** (original name was: ENA|NITX01000017|NITX01000017.1)
:   **ENANITX01000018NITX01000018.1** (original name was: ENA|NITX01000018|NITX01000018.1)
:   **ENANITX01000019NITX01000019.1** (original name was: ENA|NITX01000019|NITX01000019.1)
:   **ENANITX01000020NITX01000020.1** (original name was: ENA|NITX01000020|NITX01000020.1)
:   **ENANITX01000021NITX01000021.1** (original name was: ENA|NITX01000021|NITX01000021.1)
:   **ENANITX01000022NITX01000022.1** (original name was: ENA|NITX01000022|NITX01000022.1)
:   **ENANITX01000023NITX01000023.1** (original name was: ENA|NITX01000023|NITX01000023.1)
:   **ENANITX01000024NITX01000024.1** (original name was: ENA|NITX01000024|NITX01000024.1)
:   **ENANITX01000025NITX01000025.1** (original name was: ENA|NITX01000025|NITX01000025.1)
:   **ENANITX01000027NITX01000027.1** (original name was: ENA|NITX01000027|NITX01000027.1)
:   **ENANITX01000028NITX01000028.1** (original name was: ENA|NITX01000028|NITX01000028.1)
:   **ENANITX01000029NITX01000029.1** (original name was: ENA|NITX01000029|NITX01000029.1)
:   **ENANITX01000030NITX01000030.1** (original name was: ENA|NITX01000030|NITX01000030.1)
:   **ENANITX01000031NITX01000031.1** (original name was: ENA|NITX01000031|NITX01000031.1)
:   **ENANITX01000032NITX01000032.1** (original name was: ENA|NITX01000032|NITX01000032.1)
:   **ENANITX01000033NITX01000033.1** (original name was: ENA|NITX01000033|NITX01000033.1)
:   **ENANITX01000034NITX01000034.1** (original name was: ENA|NITX01000034|NITX01000034.1)
:   **ENANITX01000035NITX01000035.1** (original name was: ENA|NITX01000035|NITX01000035.1)
:   **ENANITX01000036NITX01000036.1** (original name was: ENA|NITX01000036|NITX01000036.1)
:   **ENANITX01000037NITX01000037.1** (original name was: ENA|NITX01000037|NITX01000037.1)
:   **ENANITX01000039NITX01000039.1** (original name was: ENA|NITX01000039|NITX01000039.1)
:   **ENANITX01000040NITX01000040.1** (original name was: ENA|NITX01000040|NITX01000040.1)
:   **ENANITX01000041NITX01000041.1** (original name was: ENA|NITX01000041|NITX01000041.1)
:   **ENANITX01000042NITX01000042.1** (original name was: ENA|NITX01000042|NITX01000042.1)
:   **ENANITX01000043NITX01000043.1** (original name was: ENA|NITX01000043|NITX01000043.1)
:   **ENANITX01000044NITX01000044.1** (original name was: ENA|NITX01000044|NITX01000044.1)
:   **ENANITX01000045NITX01000045.1** (original name was: ENA|NITX01000045|NITX01000045.1)
:   **ENANITX01000046NITX01000046.1** (original name was: ENA|NITX01000046|NITX01000046.1)
:   **ENANITX01000047NITX01000047.1** (original name was: ENA|NITX01000047|NITX01000047.1)
:   **ENANITX01000048NITX01000048.1** (original name was: ENA|NITX01000048|NITX01000048.1)
:   **ENANITX01000049NITX01000049.1** (original name was: ENA|NITX01000049|NITX01000049.1)
:   **ENANITX01000050NITX01000050.1** (original name was: ENA|NITX01000050|NITX01000050.1)
:   **ENANITX01000051NITX01000051.1** (original name was: ENA|NITX01000051|NITX01000051.1)
:   **ENANITX01000052NITX01000052.1** (original name was: ENA|NITX01000052|NITX01000052.1)
:   **ENANITX01000053NITX01000053.1** (original name was: ENA|NITX01000053|NITX01000053.1)
:   **ENANITX01000054NITX01000054.1** (original name was: ENA|NITX01000054|NITX01000054.1)
:   **ENANITX01000055NITX01000055.1** (original name was: ENA|NITX01000055|NITX01000055.1)
:   **ENANITX01000056NITX01000056.1** (original name was: ENA|NITX01000056|NITX01000056.1)
:   **ENANITX01000057NITX01000057.1** (original name was: ENA|NITX01000057|NITX01000057.1)
:   **ENANITX01000058NITX01000058.1** (original name was: ENA|NITX01000058|NITX01000058.1)
:   **ENANITX01000059NITX01000059.1** (original name was: ENA|NITX01000059|NITX01000059.1)
:   **ENANITX01000060NITX01000060.1** (original name was: ENA|NITX01000060|NITX01000060.1)
:   **ENANITX01000061NITX01000061.1** (original name was: ENA|NITX01000061|NITX01000061.1)
:   **ENANITX01000063NITX01000063.1** (original name was: ENA|NITX01000063|NITX01000063.1)
:   **ENANITX01000064NITX01000064.1** (original name was: ENA|NITX01000064|NITX01000064.1)
:   **ENANITX01000065NITX01000065.1** (original name was: ENA|NITX01000065|NITX01000065.1)
:   **ENANITX01000066NITX01000066.1** (original name was: ENA|NITX01000066|NITX01000066.1)
:   **ENANITX01000067NITX01000067.1** (original name was: ENA|NITX01000067|NITX01000067.1)
:   **ENANITX01000068NITX01000068.1** (original name was: ENA|NITX01000068|NITX01000068.1)
:   **ENANITX01000069NITX01000069.1** (original name was: ENA|NITX01000069|NITX01000069.1)
:   **ENANITX01000070NITX01000070.1** (original name was: ENA|NITX01000070|NITX01000070.1)
:   **ENANITX01000071NITX01000071.1** (original name was: ENA|NITX01000071|NITX01000071.1)
:   **ENANITX01000072NITX01000072.1** (original name was: ENA|NITX01000072|NITX01000072.1)
:   **ENANITX01000073NITX01000073.1** (original name was: ENA|NITX01000073|NITX01000073.1)
:   **ENANITX01000074NITX01000074.1** (original name was: ENA|NITX01000074|NITX01000074.1)
:   **ENANITX01000075NITX01000075.1** (original name was: ENA|NITX01000075|NITX01000075.1)
:   **ENANITX01000076NITX01000076.1** (original name was: ENA|NITX01000076|NITX01000076.1)
:   **ENANITX01000077NITX01000077.1** (original name was: ENA|NITX01000077|NITX01000077.1)
:   **ENANITX01000078NITX01000078.1** (original name was: ENA|NITX01000078|NITX01000078.1)
:   **ENANITX01000079NITX01000079.1** (original name was: ENA|NITX01000079|NITX01000079.1)
:   **ENANITX01000080NITX01000080.1** (original name was: ENA|NITX01000080|NITX01000080.1)
:   **ENANITX01000081NITX01000081.1** (original name was: ENA|NITX01000081|NITX01000081.1)
:   **ENANITX01000082NITX01000082.1** (original name was: ENA|NITX01000082|NITX01000082.1)
:   **ENANITX01000083NITX01000083.1** (original name was: ENA|NITX01000083|NITX01000083.1)
:   **ENANITX01000084NITX01000084.1** (original name was: ENA|NITX01000084|NITX01000084.1)
:   **ENANITX01000085NITX01000085.1** (original name was: ENA|NITX01000085|NITX01000085.1)
:   **ENANITX01000086NITX01000086.1** (original name was: ENA|NITX01000086|NITX01000086.1)
:   **ENANITX01000087NITX01000087.1** (original name was: ENA|NITX01000087|NITX01000087.1)
:   **ENANITX01000088NITX01000088.1** (original name was: ENA|NITX01000088|NITX01000088.1)
:   **ENANITX01000089NITX01000089.1** (original name was: ENA|NITX01000089|NITX01000089.1)
:   **ENANITX01000090NITX01000090.1** (original name was: ENA|NITX01000090|NITX01000090.1)
:   **ENANITX01000092NITX01000092.1** (original name was: ENA|NITX01000092|NITX01000092.1)
:   **ENANITX01000093NITX01000093.1** (original name was: ENA|NITX01000093|NITX01000093.1)
:   **ENANITX01000094NITX01000094.1** (original name was: ENA|NITX01000094|NITX01000094.1)
:   **ENANITX01000095NITX01000095.1** (original name was: ENA|NITX01000095|NITX01000095.1)
:   **ENANITX01000096NITX01000096.1** (original name was: ENA|NITX01000096|NITX01000096.1)
:   **ENANITX01000097NITX01000097.1** (original name was: ENA|NITX01000097|NITX01000097.1)
:   **ENANITX01000098NITX01000098.1** (original name was: ENA|NITX01000098|NITX01000098.1)
:   **ENANITX01000099NITX01000099.1** (original name was: ENA|NITX01000099|NITX01000099.1)
:   **ENANITX01000100NITX01000100.1** (original name was: ENA|NITX01000100|NITX01000100.1)
:   **ENANITX01000101NITX01000101.1** (original name was: ENA|NITX01000101|NITX01000101.1)
:   **ENANITX01000102NITX01000102.1** (original name was: ENA|NITX01000102|NITX01000102.1)
:   **ENANITX01000103NITX01000103.1** (original name was: ENA|NITX01000103|NITX01000103.1)
:   **ENANITX01000104NITX01000104.1** (original name was: ENA|NITX01000104|NITX01000104.1)
:   **ENANITX01000106NITX01000106.1** (original name was: ENA|NITX01000106|NITX01000106.1)
:   **ENANITX01000107NITX01000107.1** (original name was: ENA|NITX01000107|NITX01000107.1)
:   **ENANITX01000108NITX01000108.1** (original name was: ENA|NITX01000108|NITX01000108.1)
:   **ENANITX01000109NITX01000109.1** (original name was: ENA|NITX01000109|NITX01000109.1)
:   **ENANITX01000110NITX01000110.1** (original name was: ENA|NITX01000110|NITX01000110.1)
:   **ENANITX01000111NITX01000111.1** (original name was: ENA|NITX01000111|NITX01000111.1)
:   **ENANITX01000112NITX01000112.1** (original name was: ENA|NITX01000112|NITX01000112.1)
:   **ENANITX01000113NITX01000113.1** (original name was: ENA|NITX01000113|NITX01000113.1)
:   **ENANITX01000115NITX01000115.1** (original name was: ENA|NITX01000115|NITX01000115.1)
:   **ENANITX01000116NITX01000116.1** (original name was: ENA|NITX01000116|NITX01000116.1)
:   **ENANITX01000117NITX01000117.1** (original name was: ENA|NITX01000117|NITX01000117.1)
:   **ENANITX01000118NITX01000118.1** (original name was: ENA|NITX01000118|NITX01000118.1)
:   **ENANITX01000119NITX01000119.1** (original name was: ENA|NITX01000119|NITX01000119.1)
:   **ENANITX01000120NITX01000120.1** (original name was: ENA|NITX01000120|NITX01000120.1)
:   **ENANITX01000121NITX01000121.1** (original name was: ENA|NITX01000121|NITX01000121.1)
:   **ENANITX01000122NITX01000122.1** (original name was: ENA|NITX01000122|NITX01000122.1)
:   **ENANITX01000123NITX01000123.1** (original name was: ENA|NITX01000123|NITX01000123.1)
:   **ENANITX01000124NITX01000124.1** (original name was: ENA|NITX01000124|NITX01000124.1)
:   **ENANITX01000125NITX01000125.1** (original name was: ENA|NITX01000125|NITX01000125.1)
:   **ENANITX01000127NITX01000127.1** (original name was: ENA|NITX01000127|NITX01000127.1)
:   **ENANITX01000128NITX01000128.1** (original name was: ENA|NITX01000128|NITX01000128.1)
:   **ENANITX01000129NITX01000129.1** (original name was: ENA|NITX01000129|NITX01000129.1)
:   **ENANITX01000130NITX01000130.1** (original name was: ENA|NITX01000130|NITX01000130.1)
:   **ENANITX01000131NITX01000131.1** (original name was: ENA|NITX01000131|NITX01000131.1)
:   **ENANITX01000132NITX01000132.1** (original name was: ENA|NITX01000132|NITX01000132.1)
:   **ENANITX01000133NITX01000133.1** (original name was: ENA|NITX01000133|NITX01000133.1)
:   **ENANITX01000134NITX01000134.1** (original name was: ENA|NITX01000134|NITX01000134.1)
:   **ENANITX01000135NITX01000135.1** (original name was: ENA|NITX01000135|NITX01000135.1)
:   **ENANITX01000136NITX01000136.1** (original name was: ENA|NITX01000136|NITX01000136.1)
:   **ENANITX01000137NITX01000137.1** (original name was: ENA|NITX01000137|NITX01000137.1)
:   **ENANITX01000138NITX01000138.1** (original name was: ENA|NITX01000138|NITX01000138.1)
:   **ENANITX01000139NITX01000139.1** (original name was: ENA|NITX01000139|NITX01000139.1)
:   **ENANITX01000141NITX01000141.1** (original name was: ENA|NITX01000141|NITX01000141.1)
:   **ENANITX01000142NITX01000142.1** (original name was: ENA|NITX01000142|NITX01000142.1)
:   **ENANITX01000143NITX01000143.1** (original name was: ENA|NITX01000143|NITX01000143.1)
:   **ENANITX01000144NITX01000144.1** (original name was: ENA|NITX01000144|NITX01000144.1)
:   **ENANITX01000145NITX01000145.1** (original name was: ENA|NITX01000145|NITX01000145.1)
:   **ENANITX01000146NITX01000146.1** (original name was: ENA|NITX01000146|NITX01000146.1)
:   **ENANITX01000147NITX01000147.1** (original name was: ENA|NITX01000147|NITX01000147.1)
:   **ENANITX01000148NITX01000148.1** (original name was: ENA|NITX01000148|NITX01000148.1)
:   **ENANITX01000149NITX01000149.1** (original name was: ENA|NITX01000149|NITX01000149.1)
:   **ENANITX01000150NITX01000150.1** (original name was: ENA|NITX01000150|NITX01000150.1)
:   **ENANITX01000151NITX01000151.1** (original name was: ENA|NITX01000151|NITX01000151.1)
:   **ENANITX01000153NITX01000153.1** (original name was: ENA|NITX01000153|NITX01000153.1)
:   **ENANITX01000154NITX01000154.1** (original name was: ENA|NITX01000154|NITX01000154.1)
:   **ENANITX01000155NITX01000155.1** (original name was: ENA|NITX01000155|NITX01000155.1)
:   **ENANITX01000156NITX01000156.1** (original name was: ENA|NITX01000156|NITX01000156.1)
:   **ENANITX01000157NITX01000157.1** (original name was: ENA|NITX01000157|NITX01000157.1)
:   **ENANITX01000158NITX01000158.1** (original name was: ENA|NITX01000158|NITX01000158.1)
:   **ENANITX01000160NITX01000160.1** (original name was: ENA|NITX01000160|NITX01000160.1)
:   **ENANITX01000161NITX01000161.1** (original name was: ENA|NITX01000161|NITX01000161.1)
:   **ENANITX01000162NITX01000162.1** (original name was: ENA|NITX01000162|NITX01000162.1)
:   **ENANITX01000163NITX01000163.1** (original name was: ENA|NITX01000163|NITX01000163.1)
:   **ENANITX01000164NITX01000164.1** (original name was: ENA|NITX01000164|NITX01000164.1)
:   **ENANITX01000165NITX01000165.1** (original name was: ENA|NITX01000165|NITX01000165.1)
:   **ENANITX01000166NITX01000166.1** (original name was: ENA|NITX01000166|NITX01000166.1)
:   **ENANITX01000167NITX01000167.1** (original name was: ENA|NITX01000167|NITX01000167.1)
:   **ENANITX01000168NITX01000168.1** (original name was: ENA|NITX01000168|NITX01000168.1)
:   **ENANITX01000169NITX01000169.1** (original name was: ENA|NITX01000169|NITX01000169.1)
:   **ENANITX01000170NITX01000170.1** (original name was: ENA|NITX01000170|NITX01000170.1)
:   **ENANITX01000172NITX01000172.1** (original name was: ENA|NITX01000172|NITX01000172.1)
:   **ENANITX01000173NITX01000173.1** (original name was: ENA|NITX01000173|NITX01000173.1)
:   **ENANITX01000174NITX01000174.1** (original name was: ENA|NITX01000174|NITX01000174.1)
:   **ENANITX01000175NITX01000175.1** (original name was: ENA|NITX01000175|NITX01000175.1)
:   **ENANITX01000176NITX01000176.1** (original name was: ENA|NITX01000176|NITX01000176.1)
:   **ENANITX01000177NITX01000177.1** (original name was: ENA|NITX01000177|NITX01000177.1)
:   **ENANITX01000178NITX01000178.1** (original name was: ENA|NITX01000178|NITX01000178.1)
:   **ENANITX01000179NITX01000179.1** (original name was: ENA|NITX01000179|NITX01000179.1)
:   **ENANITX01000180NITX01000180.1** (original name was: ENA|NITX01000180|NITX01000180.1)
:   **ENANITX01000181NITX01000181.1** (original name was: ENA|NITX01000181|NITX01000181.1)
:   **ENANITX01000182NITX01000182.1** (original name was: ENA|NITX01000182|NITX01000182.1)
:   **ENANITX01000183NITX01000183.1** (original name was: ENA|NITX01000183|NITX01000183.1)
:   **ENANITX01000184NITX01000184.1** (original name was: ENA|NITX01000184|NITX01000184.1)
:   **ENANITX01000185NITX01000185.1** (original name was: ENA|NITX01000185|NITX01000185.1)
:   **ENANITX01000186NITX01000186.1** (original name was: ENA|NITX01000186|NITX01000186.1)
:   **ENANITX01000187NITX01000187.1** (original name was: ENA|NITX01000187|NITX01000187.1)
:   **ENANITX01000188NITX01000188.1** (original name was: ENA|NITX01000188|NITX01000188.1)
:   **ENANITX01000189NITX01000189.1** (original name was: ENA|NITX01000189|NITX01000189.1)
:   **ENANITX01000190NITX01000190.1** (original name was: ENA|NITX01000190|NITX01000190.1)
:   **ENANITX01000191NITX01000191.1** (original name was: ENA|NITX01000191|NITX01000191.1)
:   **ENANITX01000193NITX01000193.1** (original name was: ENA|NITX01000193|NITX01000193.1)
:   **ENANITX01000194NITX01000194.1** (original name was: ENA|NITX01000194|NITX01000194.1)
:   **ENANITX01000195NITX01000195.1** (original name was: ENA|NITX01000195|NITX01000195.1)
:   **ENANITX01000196NITX01000196.1** (original name was: ENA|NITX01000196|NITX01000196.1)
:   **ENANITX01000197NITX01000197.1** (original name was: ENA|NITX01000197|NITX01000197.1)
:   **ENANITX01000198NITX01000198.1** (original name was: ENA|NITX01000198|NITX01000198.1)
:   **ENANITX01000199NITX01000199.1** (original name was: ENA|NITX01000199|NITX01000199.1)
:   **ENANITX01000200NITX01000200.1** (original name was: ENA|NITX01000200|NITX01000200.1)
:   **ENANITX01000201NITX01000201.1** (original name was: ENA|NITX01000201|NITX01000201.1)
:   **ENANITX01000202NITX01000202.1** (original name was: ENA|NITX01000202|NITX01000202.1)
:   **ENANITX01000203NITX01000203.1** (original name was: ENA|NITX01000203|NITX01000203.1)
:   **ENANITX01000204NITX01000204.1** (original name was: ENA|NITX01000204|NITX01000204.1)
:   **ENANITX01000205NITX01000205.1** (original name was: ENA|NITX01000205|NITX01000205.1)
:   **ENANITX01000207NITX01000207.1** (original name was: ENA|NITX01000207|NITX01000207.1)
:   **ENANITX01000208NITX01000208.1** (original name was: ENA|NITX01000208|NITX01000208.1)
:   **ENANITX01000209NITX01000209.1** (original name was: ENA|NITX01000209|NITX01000209.1)
:   **ENANITX01000210NITX01000210.1** (original name was: ENA|NITX01000210|NITX01000210.1)
:   **ENANITX01000211NITX01000211.1** (original name was: ENA|NITX01000211|NITX01000211.1)
:   **ENANITX01000212NITX01000212.1** (original name was: ENA|NITX01000212|NITX01000212.1)
:   **ENANITX01000213NITX01000213.1** (original name was: ENA|NITX01000213|NITX01000213.1)
:   **ENANITX01000214NITX01000214.1** (original name was: ENA|NITX01000214|NITX01000214.1)
:   **ENANITX01000215NITX01000215.1** (original name was: ENA|NITX01000215|NITX01000215.1)
:   **ENANITX01000216NITX01000216.1** (original name was: ENA|NITX01000216|NITX01000216.1)
:   **ENANITX01000217NITX01000217.1** (original name was: ENA|NITX01000217|NITX01000217.1)
:   **ENANITX01000218NITX01000218.1** (original name was: ENA|NITX01000218|NITX01000218.1)
:   **ENANITX01000219NITX01000219.1** (original name was: ENA|NITX01000219|NITX01000219.1)
:   **ENANITX01000220NITX01000220.1** (original name was: ENA|NITX01000220|NITX01000220.1)
:   **ENANITX01000221NITX01000221.1** (original name was: ENA|NITX01000221|NITX01000221.1)
:   **ENANITX01000222NITX01000222.1** (original name was: ENA|NITX01000222|NITX01000222.1)
:   **ENANITX01000223NITX01000223.1** (original name was: ENA|NITX01000223|NITX01000223.1)
:   **ENANITX01000224NITX01000224.1** (original name was: ENA|NITX01000224|NITX01000224.1)
:   **ENANITX01000225NITX01000225.1** (original name was: ENA|NITX01000225|NITX01000225.1)
:   **ENANITX01000226NITX01000226.1** (original name was: ENA|NITX01000226|NITX01000226.1)
:   **ENANITX01000227NITX01000227.1** (original name was: ENA|NITX01000227|NITX01000227.1)
:   **ENANITX01000228NITX01000228.1** (original name was: ENA|NITX01000228|NITX01000228.1)
:   **ENANITX01000229NITX01000229.1** (original name was: ENA|NITX01000229|NITX01000229.1)
:   **ENANITX01000230NITX01000230.1** (original name was: ENA|NITX01000230|NITX01000230.1)
:   **ENANITX01000231NITX01000231.1** (original name was: ENA|NITX01000231|NITX01000231.1)
:   **ENANITX01000232NITX01000232.1** (original name was: ENA|NITX01000232|NITX01000232.1)
:   **ENANITX01000233NITX01000233.1** (original name was: ENA|NITX01000233|NITX01000233.1)
:   **ENANITX01000235NITX01000235.1** (original name was: ENA|NITX01000235|NITX01000235.1)
:   **ENANITX01000236NITX01000236.1** (original name was: ENA|NITX01000236|NITX01000236.1)
:   **ENANITX01000237NITX01000237.1** (original name was: ENA|NITX01000237|NITX01000237.1)
:   **ENANITX01000238NITX01000238.1** (original name was: ENA|NITX01000238|NITX01000238.1)
:   **ENANITX01000239NITX01000239.1** (original name was: ENA|NITX01000239|NITX01000239.1)
:   **ENANITX01000240NITX01000240.1** (original name was: ENA|NITX01000240|NITX01000240.1)
:   **ENANITX01000241NITX01000241.1** (original name was: ENA|NITX01000241|NITX01000241.1)
:   **ENANITX01000242NITX01000242.1** (original name was: ENA|NITX01000242|NITX01000242.1)
:   **ENANITX01000243NITX01000243.1** (original name was: ENA|NITX01000243|NITX01000243.1)
:   **ENANITX01000244NITX01000244.1** (original name was: ENA|NITX01000244|NITX01000244.1)
:   **ENANITX01000245NITX01000245.1** (original name was: ENA|NITX01000245|NITX01000245.1)
:   **ENANITX01000246NITX01000246.1** (original name was: ENA|NITX01000246|NITX01000246.1)
:   **ENANITX01000247NITX01000247.1** (original name was: ENA|NITX01000247|NITX01000247.1)
:   **ENANITX01000248NITX01000248.1** (original name was: ENA|NITX01000248|NITX01000248.1)
:   **ENANITX01000249NITX01000249.1** (original name was: ENA|NITX01000249|NITX01000249.1)
:   **ENANITX01000250NITX01000250.1** (original name was: ENA|NITX01000250|NITX01000250.1)
:   **ENANITX01000251NITX01000251.1** (original name was: ENA|NITX01000251|NITX01000251.1)
:   **ENANITX01000252NITX01000252.1** (original name was: ENA|NITX01000252|NITX01000252.1)
:   **ENANITX01000253NITX01000253.1** (original name was: ENA|NITX01000253|NITX01000253.1)
:   **ENANITX01000254NITX01000254.1** (original name was: ENA|NITX01000254|NITX01000254.1)
:   **ENANITX01000255NITX01000255.1** (original name was: ENA|NITX01000255|NITX01000255.1)
:   **ENANITX01000256NITX01000256.1** (original name was: ENA|NITX01000256|NITX01000256.1)
:   **ENANITX01000257NITX01000257.1** (original name was: ENA|NITX01000257|NITX01000257.1)
:   **ENANITX01000258NITX01000258.1** (original name was: ENA|NITX01000258|NITX01000258.1)
:   **ENANITX01000259NITX01000259.1** (original name was: ENA|NITX01000259|NITX01000259.1)
:   **ENANITX01000260NITX01000260.1** (original name was: ENA|NITX01000260|NITX01000260.1)
:   **ENANITX01000261NITX01000261.1** (original name was: ENA|NITX01000261|NITX01000261.1)
:   **ENANITX01000262NITX01000262.1** (original name was: ENA|NITX01000262|NITX01000262.1)
:   **ENANITX01000263NITX01000263.1** (original name was: ENA|NITX01000263|NITX01000263.1)
:   **ENANITX01000264NITX01000264.1** (original name was: ENA|NITX01000264|NITX01000264.1)
:   **ENANITX01000265NITX01000265.1** (original name was: ENA|NITX01000265|NITX01000265.1)
:   **ENANITX01000266NITX01000266.1** (original name was: ENA|NITX01000266|NITX01000266.1)
:   **ENANITX01000267NITX01000267.1** (original name was: ENA|NITX01000267|NITX01000267.1)
:   **ENANITX01000268NITX01000268.1** (original name was: ENA|NITX01000268|NITX01000268.1)
:   **ENANITX01000269NITX01000269.1** (original name was: ENA|NITX01000269|NITX01000269.1)
:   **ENANITX01000270NITX01000270.1** (original name was: ENA|NITX01000270|NITX01000270.1)
:   **ENANITX01000271NITX01000271.1** (original name was: ENA|NITX01000271|NITX01000271.1)
:   **ENANITX01000272NITX01000272.1** (original name was: ENA|NITX01000272|NITX01000272.1)
:   **ENANITX01000273NITX01000273.1** (original name was: ENA|NITX01000273|NITX01000273.1)
:   **ENANITX01000274NITX01000274.1** (original name was: ENA|NITX01000274|NITX01000274.1)
:   **ENANITX01000275NITX01000275.1** (original name was: ENA|NITX01000275|NITX01000275.1)
:   **ENANITX01000276NITX01000276.1** (original name was: ENA|NITX01000276|NITX01000276.1)
:   **ENANITX01000277NITX01000277.1** (original name was: ENA|NITX01000277|NITX01000277.1)
:   **ENANITX01000278NITX01000278.1** (original name was: ENA|NITX01000278|NITX01000278.1)
:   **ENANITX01000279NITX01000279.1** (original name was: ENA|NITX01000279|NITX01000279.1)
:   **ENANITX01000280NITX01000280.1** (original name was: ENA|NITX01000280|NITX01000280.1)
:   **ENANITX01000281NITX01000281.1** (original name was: ENA|NITX01000281|NITX01000281.1)
:   **ENANITX01000282NITX01000282.1** (original name was: ENA|NITX01000282|NITX01000282.1)
:   **ENANITX01000283NITX01000283.1** (original name was: ENA|NITX01000283|NITX01000283.1)
:   **ENANITX01000284NITX01000284.1** (original name was: ENA|NITX01000284|NITX01000284.1)
:   **ENANITX01000285NITX01000285.1** (original name was: ENA|NITX01000285|NITX01000285.1)
:   **ENANITX01000286NITX01000286.1** (original name was: ENA|NITX01000286|NITX01000286.1)
:   **ENANITX01000287NITX01000287.1** (original name was: ENA|NITX01000287|NITX01000287.1)
:   **ENANITX01000288NITX01000288.1** (original name was: ENA|NITX01000288|NITX01000288.1)
:   **ENANITX01000289NITX01000289.1** (original name was: ENA|NITX01000289|NITX01000289.1)
:   **ENANITX01000290NITX01000290.1** (original name was: ENA|NITX01000290|NITX01000290.1)
:   **ENANITX01000291NITX01000291.1** (original name was: ENA|NITX01000291|NITX01000291.1)
:   **ENANITX01000292NITX01000292.1** (original name was: ENA|NITX01000292|NITX01000292.1)
:   **ENANITX01000293NITX01000293.1** (original name was: ENA|NITX01000293|NITX01000293.1)
:   **ENANITX01000294NITX01000294.1** (original name was: ENA|NITX01000294|NITX01000294.1)
:   **ENANITX01000295NITX01000295.1** (original name was: ENA|NITX01000295|NITX01000295.1)
:   **ENANITX01000296NITX01000296.1** (original name was: ENA|NITX01000296|NITX01000296.1)
:   **ENANITX01000297NITX01000297.1** (original name was: ENA|NITX01000297|NITX01000297.1)
:   **ENANITX01000298NITX01000298.1** (original name was: ENA|NITX01000298|NITX01000298.1)
:   **ENANITX01000299NITX01000299.1** (original name was: ENA|NITX01000299|NITX01000299.1)
:   **ENANITX01000300NITX01000300.1** (original name was: ENA|NITX01000300|NITX01000300.1)
:   **ENANITX01000301NITX01000301.1** (original name was: ENA|NITX01000301|NITX01000301.1)
:   **ENANITX01000302NITX01000302.1** (original name was: ENA|NITX01000302|NITX01000302.1)
:   **ENANITX01000303NITX01000303.1** (original name was: ENA|NITX01000303|NITX01000303.1)
:   **ENANITX01000304NITX01000304.1** (original name was: ENA|NITX01000304|NITX01000304.1)
:   **ENANITX01000305NITX01000305.1** (original name was: ENA|NITX01000305|NITX01000305.1)
:   **ENANITX01000306NITX01000306.1** (original name was: ENA|NITX01000306|NITX01000306.1)
:   **ENANITX01000307NITX01000307.1** (original name was: ENA|NITX01000307|NITX01000307.1)
:   **ENANITX01000308NITX01000308.1** (original name was: ENA|NITX01000308|NITX01000308.1)
:   **ENANITX01000309NITX01000309.1** (original name was: ENA|NITX01000309|NITX01000309.1)
:   **ENANITX01000310NITX01000310.1** (original name was: ENA|NITX01000310|NITX01000310.1)
:   **ENANITX01000311NITX01000311.1** (original name was: ENA|NITX01000311|NITX01000311.1)
:   **ENANITX01000312NITX01000312.1** (original name was: ENA|NITX01000312|NITX01000312.1)
:   **ENANITX01000313NITX01000313.1** (original name was: ENA|NITX01000313|NITX01000313.1)
:   **ENANITX01000314NITX01000314.1** (original name was: ENA|NITX01000314|NITX01000314.1)
:   **ENANITX01000315NITX01000315.1** (original name was: ENA|NITX01000315|NITX01000315.1)
:   **ENANITX01000316NITX01000316.1** (original name was: ENA|NITX01000316|NITX01000316.1)
:   **ENANITX01000317NITX01000317.1** (original name was: ENA|NITX01000317|NITX01000317.1)
:   **ENANITX01000318NITX01000318.1** (original name was: ENA|NITX01000318|NITX01000318.1)
:   **ENANITX01000319NITX01000319.1** (original name was: ENA|NITX01000319|NITX01000319.1)
:   **ENANITX01000320NITX01000320.1** (original name was: ENA|NITX01000320|NITX01000320.1)
:   **ENANITX01000321NITX01000321.1** (original name was: ENA|NITX01000321|NITX01000321.1)
:   **ENANITX01000322NITX01000322.1** (original name was: ENA|NITX01000322|NITX01000322.1)
:   **ENANITX01000323NITX01000323.1** (original name was: ENA|NITX01000323|NITX01000323.1)
:   **ENANITX01000324NITX01000324.1** (original name was: ENA|NITX01000324|NITX01000324.1)
:   **ENANITX01000325NITX01000325.1** (original name was: ENA|NITX01000325|NITX01000325.1)
:   **ENANITX01000326NITX01000326.1** (original name was: ENA|NITX01000326|NITX01000326.1)
:   **ENANITX01000327NITX01000327.1** (original name was: ENA|NITX01000327|NITX01000327.1)
:   **ENANITX01000328NITX01000328.1** (original name was: ENA|NITX01000328|NITX01000328.1)
:   **ENANITX01000329NITX01000329.1** (original name was: ENA|NITX01000329|NITX01000329.1)
:   **ENANITX01000330NITX01000330.1** (original name was: ENA|NITX01000330|NITX01000330.1)
:   **ENANITX01000331NITX01000331.1** (original name was: ENA|NITX01000331|NITX01000331.1)
:   **ENANITX01000332NITX01000332.1** (original name was: ENA|NITX01000332|NITX01000332.1)
:   **ENANITX01000333NITX01000333.1** (original name was: ENA|NITX01000333|NITX01000333.1)
:   **ENANITX01000334NITX01000334.1** (original name was: ENA|NITX01000334|NITX01000334.1)
:   **ENANITX01000335NITX01000335.1** (original name was: ENA|NITX01000335|NITX01000335.1)
:   **ENANITX01000336NITX01000336.1** (original name was: ENA|NITX01000336|NITX01000336.1)
:   **ENANITX01000337NITX01000337.1** (original name was: ENA|NITX01000337|NITX01000337.1)
:   **ENANITX01000338NITX01000338.1** (original name was: ENA|NITX01000338|NITX01000338.1)
:   **ENANITX01000339NITX01000339.1** (original name was: ENA|NITX01000339|NITX01000339.1)
:   **ENANITX01000340NITX01000340.1** (original name was: ENA|NITX01000340|NITX01000340.1)
:   **ENANITX01000341NITX01000341.1** (original name was: ENA|NITX01000341|NITX01000341.1)
:   **ENANITX01000342NITX01000342.1** (original name was: ENA|NITX01000342|NITX01000342.1)
:   **ENANITX01000343NITX01000343.1** (original name was: ENA|NITX01000343|NITX01000343.1)
:   **ENANITX01000344NITX01000344.1** (original name was: ENA|NITX01000344|NITX01000344.1)
:   **ENANITX01000345NITX01000345.1** (original name was: ENA|NITX01000345|NITX01000345.1)
:   **ENANITX01000346NITX01000346.1** (original name was: ENA|NITX01000346|NITX01000346.1)
:   **ENANITX01000347NITX01000347.1** (original name was: ENA|NITX01000347|NITX01000347.1)
:   **ENANITX01000348NITX01000348.1** (original name was: ENA|NITX01000348|NITX01000348.1)
:   **ENANITX01000349NITX01000349.1** (original name was: ENA|NITX01000349|NITX01000349.1)
:   **ENANITX01000350NITX01000350.1** (original name was: ENA|NITX01000350|NITX01000350.1)
:   **ENANITX01000351NITX01000351.1** (original name was: ENA|NITX01000351|NITX01000351.1)
:   **ENANITX01000354NITX01000354.1** (original name was: ENA|NITX01000354|NITX01000354.1)
:   **ENANITX01000355NITX01000355.1** (original name was: ENA|NITX01000355|NITX01000355.1)
:   **ENANITX01000356NITX01000356.1** (original name was: ENA|NITX01000356|NITX01000356.1)
:   **ENANITX01000357NITX01000357.1** (original name was: ENA|NITX01000357|NITX01000357.1)
:   **ENANITX01000358NITX01000358.1** (original name was: ENA|NITX01000358|NITX01000358.1)
:   **ENANITX01000359NITX01000359.1** (original name was: ENA|NITX01000359|NITX01000359.1)
:   **ENANITX01000360NITX01000360.1** (original name was: ENA|NITX01000360|NITX01000360.1)
:   **ENANITX01000361NITX01000361.1** (original name was: ENA|NITX01000361|NITX01000361.1)
:   **ENANITX01000362NITX01000362.1** (original name was: ENA|NITX01000362|NITX01000362.1)
:   **ENANITX01000363NITX01000363.1** (original name was: ENA|NITX01000363|NITX01000363.1)
:   **ENANITX01000364NITX01000364.1** (original name was: ENA|NITX01000364|NITX01000364.1)
:   **ENANITX01000365NITX01000365.1** (original name was: ENA|NITX01000365|NITX01000365.1)
:   **ENANITX01000366NITX01000366.1** (original name was: ENA|NITX01000366|NITX01000366.1)
:   **ENANITX01000367NITX01000367.1** (original name was: ENA|NITX01000367|NITX01000367.1)
:   **ENANITX01000368NITX01000368.1** (original name was: ENA|NITX01000368|NITX01000368.1)
:   **ENANITX01000369NITX01000369.1** (original name was: ENA|NITX01000369|NITX01000369.1)
:   **ENANITX01000370NITX01000370.1** (original name was: ENA|NITX01000370|NITX01000370.1)
:   **ENANITX01000371NITX01000371.1** (original name was: ENA|NITX01000371|NITX01000371.1)
:   **ENANITX01000372NITX01000372.1** (original name was: ENA|NITX01000372|NITX01000372.1)
:   **ENANITX01000373NITX01000373.1** (original name was: ENA|NITX01000373|NITX01000373.1)
:   **ENANITX01000374NITX01000374.1** (original name was: ENA|NITX01000374|NITX01000374.1)
:   **ENANITX01000375NITX01000375.1** (original name was: ENA|NITX01000375|NITX01000375.1)
:   **ENANITX01000376NITX01000376.1** (original name was: ENA|NITX01000376|NITX01000376.1)
:   **ENANITX01000377NITX01000377.1** (original name was: ENA|NITX01000377|NITX01000377.1)
:   **ENANITX01000378NITX01000378.1** (original name was: ENA|NITX01000378|NITX01000378.1)
:   **ENANITX01000379NITX01000379.1** (original name was: ENA|NITX01000379|NITX01000379.1)
:   **ENANITX01000380NITX01000380.1** (original name was: ENA|NITX01000380|NITX01000380.1)
:   **ENANITX01000381NITX01000381.1** (original name was: ENA|NITX01000381|NITX01000381.1)
:   **ENANITX01000382NITX01000382.1** (original name was: ENA|NITX01000382|NITX01000382.1)
:   **ENANITX01000383NITX01000383.1** (original name was: ENA|NITX01000383|NITX01000383.1)
:   **ENANITX01000384NITX01000384.1** (original name was: ENA|NITX01000384|NITX01000384.1)
:   **ENANITX01000385NITX01000385.1** (original name was: ENA|NITX01000385|NITX01000385.1)
:   **ENANITX01000386NITX01000386.1** (original name was: ENA|NITX01000386|NITX01000386.1)
:   **ENANITX01000387NITX01000387.1** (original name was: ENA|NITX01000387|NITX01000387.1)
:   **ENANITX01000388NITX01000388.1** (original name was: ENA|NITX01000388|NITX01000388.1)
:   **ENANITX01000389NITX01000389.1** (original name was: ENA|NITX01000389|NITX01000389.1)
:   **ENANITX01000390NITX01000390.1** (original name was: ENA|NITX01000390|NITX01000390.1)
:   **ENANITX01000391NITX01000391.1** (original name was: ENA|NITX01000391|NITX01000391.1)
:   **ENANITX01000392NITX01000392.1** (original name was: ENA|NITX01000392|NITX01000392.1)
:   **ENANITX01000393NITX01000393.1** (original name was: ENA|NITX01000393|NITX01000393.1)
:   **ENANITX01000395NITX01000395.1** (original name was: ENA|NITX01000395|NITX01000395.1)
:   **ENANITX01000396NITX01000396.1** (original name was: ENA|NITX01000396|NITX01000396.1)
:   **ENANITX01000397NITX01000397.1** (original name was: ENA|NITX01000397|NITX01000397.1)
:   **ENANITX01000398NITX01000398.1** (original name was: ENA|NITX01000398|NITX01000398.1)
:   **ENANITX01000399NITX01000399.1** (original name was: ENA|NITX01000399|NITX01000399.1)
:   **ENANITX01000400NITX01000400.1** (original name was: ENA|NITX01000400|NITX01000400.1)
:   **ENANITX01000401NITX01000401.1** (original name was: ENA|NITX01000401|NITX01000401.1)
:   **ENANITX01000402NITX01000402.1** (original name was: ENA|NITX01000402|NITX01000402.1)
:   **ENANITX01000403NITX01000403.1** (original name was: ENA|NITX01000403|NITX01000403.1)
:   **ENANITX01000404NITX01000404.1** (original name was: ENA|NITX01000404|NITX01000404.1)
:   **ENANITX01000405NITX01000405.1** (original name was: ENA|NITX01000405|NITX01000405.1)
:   **ENANITX01000406NITX01000406.1** (original name was: ENA|NITX01000406|NITX01000406.1)
:   **ENANITX01000407NITX01000407.1** (original name was: ENA|NITX01000407|NITX01000407.1)
:   **ENANITX01000408NITX01000408.1** (original name was: ENA|NITX01000408|NITX01000408.1)
:   **ENANITX01000409NITX01000409.1** (original name was: ENA|NITX01000409|NITX01000409.1)
:   **ENANITX01000410NITX01000410.1** (original name was: ENA|NITX01000410|NITX01000410.1)
:   **ENANITX01000411NITX01000411.1** (original name was: ENA|NITX01000411|NITX01000411.1)
:   **ENANITX01000412NITX01000412.1** (original name was: ENA|NITX01000412|NITX01000412.1)
:   **ENANITX01000413NITX01000413.1** (original name was: ENA|NITX01000413|NITX01000413.1)
:   **ENANITX01000414NITX01000414.1** (original name was: ENA|NITX01000414|NITX01000414.1)
:   **ENANITX01000415NITX01000415.1** (original name was: ENA|NITX01000415|NITX01000415.1)
:   **ENANITX01000416NITX01000416.1** (original name was: ENA|NITX01000416|NITX01000416.1)
:   **ENANITX01000417NITX01000417.1** (original name was: ENA|NITX01000417|NITX01000417.1)
:   **ENANITX01000418NITX01000418.1** (original name was: ENA|NITX01000418|NITX01000418.1)
:   **ENANITX01000419NITX01000419.1** (original name was: ENA|NITX01000419|NITX01000419.1)
:   **ENANITX01000420NITX01000420.1** (original name was: ENA|NITX01000420|NITX01000420.1)
:   **ENANITX01000421NITX01000421.1** (original name was: ENA|NITX01000421|NITX01000421.1)
:   **ENANITX01000422NITX01000422.1** (original name was: ENA|NITX01000422|NITX01000422.1)
:   **ENANITX01000423NITX01000423.1** (original name was: ENA|NITX01000423|NITX01000423.1)
:   **ENANITX01000424NITX01000424.1** (original name was: ENA|NITX01000424|NITX01000424.1)
:   **ENANITX01000425NITX01000425.1** (original name was: ENA|NITX01000425|NITX01000425.1)
:   **ENANITX01000426NITX01000426.1** (original name was: ENA|NITX01000426|NITX01000426.1)
:   **ENANITX01000427NITX01000427.1** (original name was: ENA|NITX01000427|NITX01000427.1)
:   **ENANITX01000428NITX01000428.1** (original name was: ENA|NITX01000428|NITX01000428.1)
:   **ENANITX01000429NITX01000429.1** (original name was: ENA|NITX01000429|NITX01000429.1)
:   **ENANITX01000430NITX01000430.1** (original name was: ENA|NITX01000430|NITX01000430.1)
:   **ENANITX01000431NITX01000431.1** (original name was: ENA|NITX01000431|NITX01000431.1)
:   **ENANITX01000432NITX01000432.1** (original name was: ENA|NITX01000432|NITX01000432.1)
:   **ENANITX01000433NITX01000433.1** (original name was: ENA|NITX01000433|NITX01000433.1)
:   **ENANITX01000434NITX01000434.1** (original name was: ENA|NITX01000434|NITX01000434.1)
:   **ENANITX01000435NITX01000435.1** (original name was: ENA|NITX01000435|NITX01000435.1)
:   **ENANITX01000436NITX01000436.1** (original name was: ENA|NITX01000436|NITX01000436.1)
:   **ENANITX01000437NITX01000437.1** (original name was: ENA|NITX01000437|NITX01000437.1)
:   **ENANITX01000438NITX01000438.1** (original name was: ENA|NITX01000438|NITX01000438.1)
:   **ENANITX01000439NITX01000439.1** (original name was: ENA|NITX01000439|NITX01000439.1)
:   **ENANITX01000440NITX01000440.1** (original name was: ENA|NITX01000440|NITX01000440.1)
:   **ENANITX01000441NITX01000441.1** (original name was: ENA|NITX01000441|NITX01000441.1)
:   **ENANITX01000442NITX01000442.1** (original name was: ENA|NITX01000442|NITX01000442.1)
:   **ENANITX01000443NITX01000443.1** (original name was: ENA|NITX01000443|NITX01000443.1)
:   **ENANITX01000444NITX01000444.1** (original name was: ENA|NITX01000444|NITX01000444.1)
:   **ENANITX01000445NITX01000445.1** (original name was: ENA|NITX01000445|NITX01000445.1)
:   **ENANITX01000446NITX01000446.1** (original name was: ENA|NITX01000446|NITX01000446.1)
:   **ENANITX01000447NITX01000447.1** (original name was: ENA|NITX01000447|NITX01000447.1)
:   **ENANITX01000448NITX01000448.1** (original name was: ENA|NITX01000448|NITX01000448.1)
:   **ENANITX01000449NITX01000449.1** (original name was: ENA|NITX01000449|NITX01000449.1)
:   **ENANITX01000450NITX01000450.1** (original name was: ENA|NITX01000450|NITX01000450.1)
:   **ENANITX01000451NITX01000451.1** (original name was: ENA|NITX01000451|NITX01000451.1)
:   **ENANITX01000452NITX01000452.1** (original name was: ENA|NITX01000452|NITX01000452.1)
:   **ENANITX01000453NITX01000453.1** (original name was: ENA|NITX01000453|NITX01000453.1)
:   **ENANITX01000455NITX01000455.1** (original name was: ENA|NITX01000455|NITX01000455.1)
:   **ENANITX01000456NITX01000456.1** (original name was: ENA|NITX01000456|NITX01000456.1)
:   **ENANITX01000457NITX01000457.1** (original name was: ENA|NITX01000457|NITX01000457.1)
:   **ENANITX01000458NITX01000458.1** (original name was: ENA|NITX01000458|NITX01000458.1)
:   **ENANITX01000459NITX01000459.1** (original name was: ENA|NITX01000459|NITX01000459.1)
:   **ENANITX01000460NITX01000460.1** (original name was: ENA|NITX01000460|NITX01000460.1)
:   **ENANITX01000461NITX01000461.1** (original name was: ENA|NITX01000461|NITX01000461.1)
:   **ENANITX01000462NITX01000462.1** (original name was: ENA|NITX01000462|NITX01000462.1)
:   **ENANITX01000463NITX01000463.1** (original name was: ENA|NITX01000463|NITX01000463.1)
:   **ENANITX01000464NITX01000464.1** (original name was: ENA|NITX01000464|NITX01000464.1)
:   **ENANITX01000466NITX01000466.1** (original name was: ENA|NITX01000466|NITX01000466.1)
:   **ENANITX01000467NITX01000467.1** (original name was: ENA|NITX01000467|NITX01000467.1)
:   **ENANITX01000468NITX01000468.1** (original name was: ENA|NITX01000468|NITX01000468.1)
:   **ENANITX01000469NITX01000469.1** (original name was: ENA|NITX01000469|NITX01000469.1)
:   **ENANITX01000470NITX01000470.1** (original name was: ENA|NITX01000470|NITX01000470.1)
:   **ENANITX01000471NITX01000471.1** (original name was: ENA|NITX01000471|NITX01000471.1)
:   **ENANITX01000472NITX01000472.1** (original name was: ENA|NITX01000472|NITX01000472.1)
:   **ENANITX01000474NITX01000474.1** (original name was: ENA|NITX01000474|NITX01000474.1)
:   **ENANITX01000475NITX01000475.1** (original name was: ENA|NITX01000475|NITX01000475.1)
:   **ENANITX01000476NITX01000476.1** (original name was: ENA|NITX01000476|NITX01000476.1)
:   **ENANITX01000477NITX01000477.1** (original name was: ENA|NITX01000477|NITX01000477.1)
:   **ENANITX01000478NITX01000478.1** (original name was: ENA|NITX01000478|NITX01000478.1)
:   **ENANITX01000479NITX01000479.1** (original name was: ENA|NITX01000479|NITX01000479.1)
:   **ENANITX01000480NITX01000480.1** (original name was: ENA|NITX01000480|NITX01000480.1)
:   **ENANITX01000481NITX01000481.1** (original name was: ENA|NITX01000481|NITX01000481.1)
:   **ENANITX01000482NITX01000482.1** (original name was: ENA|NITX01000482|NITX01000482.1)
:   **ENANITX01000483NITX01000483.1** (original name was: ENA|NITX01000483|NITX01000483.1)
:   **ENANITX01000484NITX01000484.1** (original name was: ENA|NITX01000484|NITX01000484.1)
:   **ENANITX01000485NITX01000485.1** (original name was: ENA|NITX01000485|NITX01000485.1)
:   **ENANITX01000486NITX01000486.1** (original name was: ENA|NITX01000486|NITX01000486.1)
:   **ENANITX01000487NITX01000487.1** (original name was: ENA|NITX01000487|NITX01000487.1)
:   **ENANITX01000488NITX01000488.1** (original name was: ENA|NITX01000488|NITX01000488.1)
:   **ENANITX01000489NITX01000489.1** (original name was: ENA|NITX01000489|NITX01000489.1)
:   **ENANITX01000490NITX01000490.1** (original name was: ENA|NITX01000490|NITX01000490.1)
:   **ENANITX01000491NITX01000491.1** (original name was: ENA|NITX01000491|NITX01000491.1)
:   **ENANITX01000492NITX01000492.1** (original name was: ENA|NITX01000492|NITX01000492.1)
:   **ENANITX01000493NITX01000493.1** (original name was: ENA|NITX01000493|NITX01000493.1)
:   **ENANITX01000494NITX01000494.1** (original name was: ENA|NITX01000494|NITX01000494.1)
:   **ENANITX01000495NITX01000495.1** (original name was: ENA|NITX01000495|NITX01000495.1)
:   **ENANITX01000496NITX01000496.1** (original name was: ENA|NITX01000496|NITX01000496.1)
:   **ENANITX01000497NITX01000497.1** (original name was: ENA|NITX01000497|NITX01000497.1)
:   **ENANITX01000498NITX01000498.1** (original name was: ENA|NITX01000498|NITX01000498.1)
:   **ENANITX01000500NITX01000500.1** (original name was: ENA|NITX01000500|NITX01000500.1)
:   **ENANITX01000502NITX01000502.1** (original name was: ENA|NITX01000502|NITX01000502.1)
:   **ENANITX01000503NITX01000503.1** (original name was: ENA|NITX01000503|NITX01000503.1)
:   **ENANITX01000504NITX01000504.1** (original name was: ENA|NITX01000504|NITX01000504.1)
:   **ENANITX01000505NITX01000505.1** (original name was: ENA|NITX01000505|NITX01000505.1)
:   **ENANITX01000506NITX01000506.1** (original name was: ENA|NITX01000506|NITX01000506.1)
:   **ENANITX01000507NITX01000507.1** (original name was: ENA|NITX01000507|NITX01000507.1)
:   **ENANITX01000508NITX01000508.1** (original name was: ENA|NITX01000508|NITX01000508.1)
:   **ENANITX01000509NITX01000509.1** (original name was: ENA|NITX01000509|NITX01000509.1)
:   **ENANITX01000510NITX01000510.1** (original name was: ENA|NITX01000510|NITX01000510.1)
:   **ENANITX01000511NITX01000511.1** (original name was: ENA|NITX01000511|NITX01000511.1)
:   **ENANITX01000512NITX01000512.1** (original name was: ENA|NITX01000512|NITX01000512.1)
:   **ENANITX01000513NITX01000513.1** (original name was: ENA|NITX01000513|NITX01000513.1)
:   **ENANITX01000514NITX01000514.1** (original name was: ENA|NITX01000514|NITX01000514.1)
:   **ENANITX01000515NITX01000515.1** (original name was: ENA|NITX01000515|NITX01000515.1)
:   **ENANITX01000516NITX01000516.1** (original name was: ENA|NITX01000516|NITX01000516.1)
:   **ENANITX01000517NITX01000517.1** (original name was: ENA|NITX01000517|NITX01000517.1)
:   **ENANITX01000518NITX01000518.1** (original name was: ENA|NITX01000518|NITX01000518.1)
:   **ENANITX01000519NITX01000519.1** (original name was: ENA|NITX01000519|NITX01000519.1)
:   **ENANITX01000520NITX01000520.1** (original name was: ENA|NITX01000520|NITX01000520.1)
:   **ENANITX01000521NITX01000521.1** (original name was: ENA|NITX01000521|NITX01000521.1)
:   **ENANITX01000522NITX01000522.1** (original name was: ENA|NITX01000522|NITX01000522.1)
:   **ENANITX01000523NITX01000523.1** (original name was: ENA|NITX01000523|NITX01000523.1)
:   **ENANITX01000524NITX01000524.1** (original name was: ENA|NITX01000524|NITX01000524.1)
:   **ENANITX01000525NITX01000525.1** (original name was: ENA|NITX01000525|NITX01000525.1)
:   **ENANITX01000526NITX01000526.1** (original name was: ENA|NITX01000526|NITX01000526.1)
:   **ENANITX01000527NITX01000527.1** (original name was: ENA|NITX01000527|NITX01000527.1)
:   **ENANITX01000528NITX01000528.1** (original name was: ENA|NITX01000528|NITX01000528.1)
:   **ENANITX01000530NITX01000530.1** (original name was: ENA|NITX01000530|NITX01000530.1)
:   **ENANITX01000531NITX01000531.1** (original name was: ENA|NITX01000531|NITX01000531.1)
:   **ENANITX01000532NITX01000532.1** (original name was: ENA|NITX01000532|NITX01000532.1)
:   **ENANITX01000533NITX01000533.1** (original name was: ENA|NITX01000533|NITX01000533.1)
:   **ENANITX01000535NITX01000535.1** (original name was: ENA|NITX01000535|NITX01000535.1)
:   **ENANITX01000536NITX01000536.1** (original name was: ENA|NITX01000536|NITX01000536.1)
:   **ENANITX01000537NITX01000537.1** (original name was: ENA|NITX01000537|NITX01000537.1)
:   **ENANITX01000538NITX01000538.1** (original name was: ENA|NITX01000538|NITX01000538.1)
:   **ENANITX01000539NITX01000539.1** (original name was: ENA|NITX01000539|NITX01000539.1)
:   **ENANITX01000540NITX01000540.1** (original name was: ENA|NITX01000540|NITX01000540.1)
:   **ENANITX01000541NITX01000541.1** (original name was: ENA|NITX01000541|NITX01000541.1)
:   **ENANITX01000542NITX01000542.1** (original name was: ENA|NITX01000542|NITX01000542.1)
:   **ENANITX01000543NITX01000543.1** (original name was: ENA|NITX01000543|NITX01000543.1)
:   **ENANITX01000544NITX01000544.1** (original name was: ENA|NITX01000544|NITX01000544.1)
:   **ENANITX01000545NITX01000545.1** (original name was: ENA|NITX01000545|NITX01000545.1)
:   **ENANITX01000546NITX01000546.1** (original name was: ENA|NITX01000546|NITX01000546.1)
:   **ENANITX01000547NITX01000547.1** (original name was: ENA|NITX01000547|NITX01000547.1)
:   **ENANITX01000548NITX01000548.1** (original name was: ENA|NITX01000548|NITX01000548.1)
:   **ENANITX01000549NITX01000549.1** (original name was: ENA|NITX01000549|NITX01000549.1)
:   **ENANITX01000550NITX01000550.1** (original name was: ENA|NITX01000550|NITX01000550.1)
:   **ENANITX01000551NITX01000551.1** (original name was: ENA|NITX01000551|NITX01000551.1)
:   **ENANITX01000552NITX01000552.1** (original name was: ENA|NITX01000552|NITX01000552.1)
:   **ENANITX01000553NITX01000553.1** (original name was: ENA|NITX01000553|NITX01000553.1)
:   **ENANITX01000554NITX01000554.1** (original name was: ENA|NITX01000554|NITX01000554.1)
:   **ENANITX01000555NITX01000555.1** (original name was: ENA|NITX01000555|NITX01000555.1)
:   **ENANITX01000556NITX01000556.1** (original name was: ENA|NITX01000556|NITX01000556.1)
:   **ENANITX01000557NITX01000557.1** (original name was: ENA|NITX01000557|NITX01000557.1)
:   **ENANITX01000558NITX01000558.1** (original name was: ENA|NITX01000558|NITX01000558.1)
:   **ENANITX01000559NITX01000559.1** (original name was: ENA|NITX01000559|NITX01000559.1)
:   **ENANITX01000560NITX01000560.1** (original name was: ENA|NITX01000560|NITX01000560.1)
:   **ENANITX01000561NITX01000561.1** (original name was: ENA|NITX01000561|NITX01000561.1)
:   **ENANITX01000562NITX01000562.1** (original name was: ENA|NITX01000562|NITX01000562.1)
:   **ENANITX01000563NITX01000563.1** (original name was: ENA|NITX01000563|NITX01000563.1)
:   **ENANITX01000564NITX01000564.1** (original name was: ENA|NITX01000564|NITX01000564.1)
:   **ENANITX01000565NITX01000565.1** (original name was: ENA|NITX01000565|NITX01000565.1)
:   **ENANITX01000566NITX01000566.1** (original name was: ENA|NITX01000566|NITX01000566.1)
:   **ENANITX01000567NITX01000567.1** (original name was: ENA|NITX01000567|NITX01000567.1)
:   **ENANITX01000568NITX01000568.1** (original name was: ENA|NITX01000568|NITX01000568.1)
:   **ENANITX01000569NITX01000569.1** (original name was: ENA|NITX01000569|NITX01000569.1)
:   **ENANITX01000570NITX01000570.1** (original name was: ENA|NITX01000570|NITX01000570.1)
:   **ENANITX01000571NITX01000571.1** (original name was: ENA|NITX01000571|NITX01000571.1)
:   **ENANITX01000572NITX01000572.1** (original name was: ENA|NITX01000572|NITX01000572.1)
:   **ENANITX01000573NITX01000573.1** (original name was: ENA|NITX01000573|NITX01000573.1)
:   **ENANITX01000575NITX01000575.1** (original name was: ENA|NITX01000575|NITX01000575.1)
:   **ENANITX01000576NITX01000576.1** (original name was: ENA|NITX01000576|NITX01000576.1)
:   **ENANITX01000578NITX01000578.1** (original name was: ENA|NITX01000578|NITX01000578.1)
:   **ENANITX01000579NITX01000579.1** (original name was: ENA|NITX01000579|NITX01000579.1)
:   **ENANITX01000580NITX01000580.1** (original name was: ENA|NITX01000580|NITX01000580.1)
:   **ENANITX01000581NITX01000581.1** (original name was: ENA|NITX01000581|NITX01000581.1)
:   **ENANITX01000582NITX01000582.1** (original name was: ENA|NITX01000582|NITX01000582.1)
:   **ENANITX01000583NITX01000583.1** (original name was: ENA|NITX01000583|NITX01000583.1)
:   **ENANITX01000584NITX01000584.1** (original name was: ENA|NITX01000584|NITX01000584.1)
:   **ENANITX01000585NITX01000585.1** (original name was: ENA|NITX01000585|NITX01000585.1)
:   **ENANITX01000586NITX01000586.1** (original name was: ENA|NITX01000586|NITX01000586.1)
:   **ENANITX01000587NITX01000587.1** (original name was: ENA|NITX01000587|NITX01000587.1)
:   **ENANITX01000588NITX01000588.1** (original name was: ENA|NITX01000588|NITX01000588.1)
:   **ENANITX01000589NITX01000589.1** (original name was: ENA|NITX01000589|NITX01000589.1)
:   **ENANITX01000590NITX01000590.1** (original name was: ENA|NITX01000590|NITX01000590.1)
:   **ENANITX01000591NITX01000591.1** (original name was: ENA|NITX01000591|NITX01000591.1)
:   **ENANITX01000592NITX01000592.1** (original name was: ENA|NITX01000592|NITX01000592.1)
:   **ENANITX01000593NITX01000593.1** (original name was: ENA|NITX01000593|NITX01000593.1)
:   **ENANITX01000594NITX01000594.1** (original name was: ENA|NITX01000594|NITX01000594.1)
:   **ENANITX01000595NITX01000595.1** (original name was: ENA|NITX01000595|NITX01000595.1)
:   **ENANITX01000596NITX01000596.1** (original name was: ENA|NITX01000596|NITX01000596.1)
:   **ENANITX01000597NITX01000597.1** (original name was: ENA|NITX01000597|NITX01000597.1)
:   **ENANITX01000598NITX01000598.1** (original name was: ENA|NITX01000598|NITX01000598.1)
:   **ENANITX01000599NITX01000599.1** (original name was: ENA|NITX01000599|NITX01000599.1)
:   **ENANITX01000601NITX01000601.1** (original name was: ENA|NITX01000601|NITX01000601.1)
:   **ENANITX01000602NITX01000602.1** (original name was: ENA|NITX01000602|NITX01000602.1)
:   **ENANITX01000603NITX01000603.1** (original name was: ENA|NITX01000603|NITX01000603.1)
:   **ENANITX01000604NITX01000604.1** (original name was: ENA|NITX01000604|NITX01000604.1)
:   **ENANITX01000605NITX01000605.1** (original name was: ENA|NITX01000605|NITX01000605.1)
:   **ENANITX01000606NITX01000606.1** (original name was: ENA|NITX01000606|NITX01000606.1)
:   **ENANITX01000607NITX01000607.1** (original name was: ENA|NITX01000607|NITX01000607.1)
:   **ENANITX01000608NITX01000608.1** (original name was: ENA|NITX01000608|NITX01000608.1)
:   **ENANITX01000609NITX01000609.1** (original name was: ENA|NITX01000609|NITX01000609.1)
:   **ENANITX01000610NITX01000610.1** (original name was: ENA|NITX01000610|NITX01000610.1)
:   **ENANITX01000611NITX01000611.1** (original name was: ENA|NITX01000611|NITX01000611.1)

Compact view

ENANITX01000026NITX01000026.1 - Region 1 - T3PKS

Shows the layout of the region, marking coding sequences and areas of interest. Clicking a gene will select it and show any relevant details. Clicking an area feature (e.g. a candidate cluster) will select all coding sequences within that area. Double clicking an area feature will zoom to that area. Multiple genes and area features can be selected by clicking them while holding the Ctrl key.  
More detailed help is available here.

Download region GenBank file

Download region SVG

Location: 1 - 35,100 nt. (total: 35,100 nt)
Show pHMM detection rules used

Region on contig edge.

T3PKS: (Chal\_sti\_synt\_C or Chal\_sti\_synt\_N)

#### Legend:

core biosynthetic genes

additional biosynthetic genes

transport-related genes

regulatory genes

other genes

resistance

reset view

zoom to selection

Gene details

Shows details of the most recently selected gene, including names, products, location, and other annotations.

Select a gene to view the details available for it

Gene overview

MIBiG comparison

ClusterBlast

KnownClusterBlast

SubClusterBlast

Pfam domains

TIGRFAM domains

Gene/CDS overview

A brief tabular summary of genes/CDS features within the region.  
Filtering the table will also search biosynthetic profiles and gene function data. If enabled, the overview will then zoom to show the area covered by the filtered selection.  
Genes selected in the region drawing above will be marked in the table with an indicator to the left of the gene name.

Filter:

Automatically zoom to filtered/selected features

| Identifier | Product | Length | | Function | Sequence | | NCBI Blast | Filter details |
| --- | --- | --- | --- | --- | --- | --- | --- | --- |
|  |  | NT | AA |  | NT | AA |  |  |

No genes match the given filter

Similar gene clusters

Shows careas that are similar to the current region to a reference database.  
Mouseover a score cell in the table to get a breakdown of how the score was calculated.The MIBiG database.  
  
Click on an accession to open that entry in the MIBiG database.

Analysis type:

Protocluster to Region
Region to Region

| Reference | T3PKS | Similarity score | Type | Compound(s) | Organism |
| --- | --- | --- | --- | --- | --- |
| BGC0002403 |  | 0.20 | Polyketide | β-Diketones | Hordeum vulgare subsp. vulgare |
| BGC0002362 |  | 0.10 | Polyketide | loseolamycin A1, loseolamycin A2 | Micromonospora endolithica |
| BGC0001878 |  | 0.08 | NRP, Polyketide | detoxin N2, detoxin N3 | Streptomyces sp. NRRL B-1347 |
| BGC0002262 |  | 0.08 | Polyketide | trienylfuranol A, sporothriolide, dihydrosporothriolide, deoxysporothric acid, dihydroisosporothric acid, sporothric acid, isosporothric acid, sporochartine B | Hypomontagnella monticulosa |
| BGC0000678 |  | 0.07 | Terpene | pentalenolactone | Streptomyces avermitilis MA-4680 = NBRC 14893 |
| BGC0001841 |  | 0.07 | NRP, Polyketide | detoxin P1, detoxin P2, detoxin P3 | Amycolatopsis jejuensis |
| BGC0001996 |  | 0.06 | Other | oryzine A, oryzine B | Aspergillus oryzae RIB40 |
| BGC0001760 |  | 0.06 | NRP | rimosamide | Streptomyces rimosus subsp. rimosus ATCC 10970 |
| BGC0001528 |  | 0.05 | Terpene | betaestacin | Phoma betae |
| BGC0001840 |  | 0.05 | NRP, Polyketide | detoxin S1 | Streptomyces sp. NRRL S-325 |

| Reference | Aggregated | Similarity score | Type | Compound(s) | Organism |
| --- | --- | --- | --- | --- | --- |
| BGC0002403 |  | 0.53 | Polyketide | β-Diketones | Hordeum vulgare subsp. vulgare |
| BGC0002362 |  | 0.37 | Polyketide | loseolamycin A1, loseolamycin A2 | Micromonospora endolithica |
| BGC0001878 |  | 0.32 | NRP, Polyketide | detoxin N2, detoxin N3 | Streptomyces sp. NRRL B-1347 |
| BGC0002262 |  | 0.30 | Polyketide | trienylfuranol A, sporothriolide, dihydrosporothriolide, deoxysporothric acid, dihydroisosporothric acid, sporothric acid, isosporothric acid, sporochartine B | Hypomontagnella monticulosa |
| BGC0000678 |  | 0.28 | Terpene | pentalenolactone | Streptomyces avermitilis MA-4680 = NBRC 14893 |
| BGC0001841 |  | 0.27 | NRP, Polyketide | detoxin P1, detoxin P2, detoxin P3 | Amycolatopsis jejuensis |
| BGC0001996 |  | 0.27 | Other | oryzine A, oryzine B | Aspergillus oryzae RIB40 |
| BGC0001760 |  | 0.25 | NRP | rimosamide | Streptomyces rimosus subsp. rimosus ATCC 10970 |
| BGC0001528 |  | 0.23 | Terpene | betaestacin | Phoma betae |
| BGC0001840 |  | 0.22 | NRP, Polyketide | detoxin S1 | Streptomyces sp. NRRL S-325 |

Similar gene clusters

Shows regions from the antiSMASH database that are similar to the current region. Genes marked with the same colour are interrelated. White genes have no relationship.  
Click on reference genes to show details of similarities to genes within the current region.  
Click on an accession to open that entry in the antiSMASH database (if applicable).

All hits

NW\_021167079 (2642961-2685463): Sodiomyces alkalinus F11 unplaced genomic sca... (47% of genes show similarity), T3PKS

NW\_009276935 (727151-768605): Verticillium dahliae VdLs.17 supercont1.16 geno... (36% of genes show similarity), T3PKS

NC\_030957 (3708969-3750425): Colletotrichum higginsianum IMI 349063 chromosom... (26% of genes show similarity), T3PKS

NW\_023336263 (1625065-1666515): Colletotrichum scovillei strain TJNH1 chromos... (30% of genes show similarity), T3PKS

CM000596 (2483961-2525418): Fusarium oxysporum f. sp. lycopersici 4287 chromo... (16% of genes show similarity), T3PKS

NC\_030993 (2483961-2525418): Fusarium oxysporum f. sp. lycopersici 4287 chrom... (16% of genes show similarity), T3PKS

CM000575 (2059829-2101313): Fusarium graminearum PH-1 chromosome 2, whole gen... (25% of genes show similarity), T3PKS

NC\_026475 (2059829-2101313): Fusarium graminearum PH-1 chromosome 2, whole ge... (25% of genes show similarity), T3PKS

NW\_022194795 (789395-830853): Fusarium proliferatum ET1 genome assembly, cont... (21% of genes show similarity), T3PKS

NC\_038013 (1473588-1515077): Fusarium venenatum strain A3/5 genome assembly, ... (25% of genes show similarity), T3PKS
Download graphic

Similar known gene clusters from MIBiG 3.1

Shows clusters from the MiBIG database that are similar to the current region. Genes marked with the same colour are interrelated. White genes have no relationship.  
Click on reference genes to show details of similarities to genes within the current region.  
Click on an accession to open that entry in the MiBIG database.

No matches found.

Similar subclusters

Shows sub-cluster units that are similar to the current region. Genes marked with the same colour are interrelated. White genes have no relationship.  
Click on reference genes to show details of similarities to genes within the current region.

No matches found.

Detailed Pfam domain annotation

Shows Pfam domains found in each gene within the region. Click on each domain for more information about the domain's accession, location, and description. Domains with a bold border have Gene Ontology information.

Selected features only

Expand to show all names

Detailed TIGRFAM domain annotation

Shows TIGRFAM domains found in each gene within the region. Click on each domain for more information about the domain's accession, location, and description.

Selected features only

Expand to show all names

ENANITX01000062NITX01000062.1 - Region 1 - T1PKS

Shows the layout of the region, marking coding sequences and areas of interest. Clicking a gene will select it and show any relevant details. Clicking an area feature (e.g. a candidate cluster) will select all coding sequences within that area. Double clicking an area feature will zoom to that area. Multiple genes and area features can be selected by clicking them while holding the Ctrl key.  
More detailed help is available here.

Download region GenBank file

Download region SVG

Location: 124,154 - 192,305 nt. (total: 68,152 nt)
Show pHMM detection rules used

T1PKS: cds(PKS\_AT and (PKS\_KS or ene\_KS or mod\_KS or hyb\_KS or itr\_KS or tra\_KS))

#### Legend:

core biosynthetic genes

additional biosynthetic genes

transport-related genes

regulatory genes

other genes

resistance

reset view

zoom to selection

Gene details

Shows details of the most recently selected gene, including names, products, location, and other annotations.

Select a gene to view the details available for it

Gene overview

NRPS/PKS domains

MIBiG comparison

ClusterBlast

KnownClusterBlast

SubClusterBlast

NRPS/PKS modules

Pfam domains

TIGRFAM domains

Gene/CDS overview

A brief tabular summary of genes/CDS features within the region.  
Filtering the table will also search biosynthetic profiles and gene function data. If enabled, the overview will then zoom to show the area covered by the filtered selection.  
Genes selected in the region drawing above will be marked in the table with an indicator to the left of the gene name.

Filter:

Automatically zoom to filtered/selected features

| Identifier | Product | Length | | Function | Sequence | | NCBI Blast | Filter details |
| --- | --- | --- | --- | --- | --- | --- | --- | --- |
|  |  | NT | AA |  | NT | AA |  |  |

No genes match the given filter

Detailed domain annotation

Shows NRPS- and PKS-related domains for each feature that contains them. Click on each domain for more information about the domain's location, consensus monomer prediction, and other details.  
A domain glossary is available here, and an explanation of the visualisation is available here.

Selected features only

Show module domains

Similar gene clusters

Shows careas that are similar to the current region to a reference database.  
Mouseover a score cell in the table to get a breakdown of how the score was calculated.The MIBiG database.  
  
Click on an accession to open that entry in the MIBiG database.

Analysis type:

Protocluster to Region
Region to Region

| Reference | T1PKS | Similarity score | Type | Compound(s) | Organism |
| --- | --- | --- | --- | --- | --- |
| BGC0002525 |  | 0.33 | Polyketide | fusarubin, 1233A, 1233B, NG-391, lucilactaene | Fusarium sp. |
| BGC0002404 |  | 0.33 | Other | falcarindiol | Solanum lycopersicum |
| BGC0002191 |  | 0.33 | Polyketide | prolipyrone B, gibepyrone D | Fusarium graminearum PH-1 |
| BGC0002515 |  | 0.31 | Polyketide | solanapyrone A | Ascochyta rabiei |
| BGC0001252 |  | 0.30 | Polyketide | UNII-YC2Q1O94PT | Alternaria alternata |
| BGC0001606 |  | 0.30 | Polyketide | gibepyrone-A | Fusarium fujikuroi IMI 58289 |
| BGC0002155 |  | 0.29 | Polyketide | nectriapyrone C, nectriapyrone D, nectriapyrone | Pyricularia oryzae 70-15 |
| BGC0000146 |  | 0.28 | Polyketide | solanapyrone D | Alternaria solani |
| BGC0002224 |  | 0.28 | Polyketide | zopfiellin | Diffractella curvata |
| BGC0001899 |  | 0.28 | Polyketide | abscisic acid | Leptosphaeria maculans JN3 |

| Reference | Aggregated | Similarity score | Type | Compound(s) | Organism |
| --- | --- | --- | --- | --- | --- |
| BGC0002525 |  | 0.67 | Polyketide | fusarubin, 1233A, 1233B, NG-391, lucilactaene | Fusarium sp. |
| BGC0002404 |  | 0.66 | Other | falcarindiol | Solanum lycopersicum |
| BGC0002191 |  | 0.66 | Polyketide | prolipyrone B, gibepyrone D | Fusarium graminearum PH-1 |
| BGC0002515 |  | 0.64 | Polyketide | solanapyrone A | Ascochyta rabiei |
| BGC0001252 |  | 0.64 | Polyketide | UNII-YC2Q1O94PT | Alternaria alternata |
| BGC0001606 |  | 0.64 | Polyketide | gibepyrone-A | Fusarium fujikuroi IMI 58289 |
| BGC0002155 |  | 0.63 | Polyketide | nectriapyrone C, nectriapyrone D, nectriapyrone | Pyricularia oryzae 70-15 |
| BGC0000146 |  | 0.62 | Polyketide | solanapyrone D | Alternaria solani |
| BGC0002224 |  | 0.62 | Polyketide | zopfiellin | Diffractella curvata |
| BGC0001899 |  | 0.61 | Polyketide | abscisic acid | Leptosphaeria maculans JN3 |

Similar gene clusters

Shows regions from the antiSMASH database that are similar to the current region. Genes marked with the same colour are interrelated. White genes have no relationship.  
Click on reference genes to show details of similarities to genes within the current region.  
Click on an accession to open that entry in the antiSMASH database (if applicable).

All hits

NW\_021167079 (4764547-4812699): Sodiomyces alkalinus F11 unplaced genomic sca... (84% of genes show similarity), T1PKS

NW\_022194791 (4116991-4170575): Fusarium proliferatum ET1 genome assembly, co... (18% of genes show similarity), NRPS,T1PKS

NW\_022983866 (974232-1001357): Arthroderma uncinatum strain CBS 119779 chromo... (33% of genes show similarity), T1PKS

NW\_023336280 (75664-136253): Aspergillus tubingensis WU-2223L DNA, scaffold 6... (11% of genes show similarity), NRPS,T1PKS,terpene

NC\_007196 (3877250-3924985): Aspergillus fumigatus Af293 chromosome 3, whole ... (10% of genes show similarity), T1PKS

NW\_003315094 (177129-224833): Trichophyton benhamiae CBS 112371 chromosome Un... (11% of genes show similarity), T1PKS

NW\_003345201 (80944-123730): Nannizzia gypsea CBS 118893 supercont1.1 genomic... (11% of genes show similarity), T1PKS

NW\_017264199 (1083767-1131168): Xylona heveae TC161 unplaced genomic scaffold... (12% of genes show similarity), T1PKS

NW\_003456428 (3937600-3983953): Trichophyton rubrum CBS 118892 genomic scaffo... (11% of genes show similarity), T1PKS

NW\_022984628 (4194508-4271225): Aspergillus tanneri strain NIH1004 chromosome... (15% of genes show similarity), T1PKS
Download graphic

Similar known gene clusters from MIBiG 3.1

Shows clusters from the MiBIG database that are similar to the current region. Genes marked with the same colour are interrelated. White genes have no relationship.  
Click on reference genes to show details of similarities to genes within the current region.  
Click on an accession to open that entry in the MiBIG database.

No matches found.

Similar subclusters

Shows sub-cluster units that are similar to the current region. Genes marked with the same colour are interrelated. White genes have no relationship.  
Click on reference genes to show details of similarities to genes within the current region.

No matches found.

Module view

Shows module structures for each candidate cluster in NRPS and PKS regions.   
Genes are shown in predicted order, and are only present when containing at least one complete module.   
A domain glossary is available here, and an explanation of the visualisation is available here.

Candidate 1 (124153 - 192305): single T1PKS

Legend

Detailed Pfam domain annotation

Shows Pfam domains found in each gene within the region. Click on each domain for more information about the domain's accession, location, and description. Domains with a bold border have Gene Ontology information.

Selected features only

Expand to show all names

Detailed TIGRFAM domain annotation

Shows TIGRFAM domains found in each gene within the region. Click on each domain for more information about the domain's accession, location, and description.

Selected features only

Expand to show all names

NRPS/PKS products

NRPS/PKS monomers

Predicted core structure(s)

Shows estimated product structure and polymer for each candidate cluster in the region. To show the product, click on the expander or the candidate cluster feature drawn in the overview.

For candidate cluster 1, location 124153 - 192305:

Rough prediction of core scaffold based on assumed PKS/NRPS colinearity; tailoring reactions not taken into account

**Polymer prediction:**
:   (pk)

  
Direct lookup in NORINE database:
strict
or
relaxed

Link to NORINE database query form

NRPS/PKS monomer predictions

Shows the predicted monomers for each adynelation domain and acyltransferase within genes. Each gene prediction can be expanded to view detailed predictions of each domain. Each prediction can be expanded to view the predictions by tool (and, for some tools, further expanded for extra details).

**input.path1.gene54**: pk

:   **PKS\_AT (631..934)**: pk

    ATSignature: Malonyl-CoA

    Top 3 matches:
    :   Malonyl-CoA: 70.8%
    :   Methylmalonyl-CoA: 58.3%
    :   Isobutyryl-CoA: 58.3%

      
    minowa: Methylmalonyl-CoA

    Prediction, score:
    :   Methylmalonyl-CoA: 83.3


        Methoxymalonyl-CoA: 70.8


        Propionyl-CoA: 54.2


        Malonyl-CoA: 44.8


        Isobutyryl-CoA: 44.6


        2-Methylbutyryl-CoA: 39.1


        Ethylmalonyl-CoA: 34.2


        fatty\_acid: 27.6


        trans-1,2-CPDA: 25.7


        Benzoyl-CoA: 20.6


        CHC-CoA: 20.3


        inactive: 11.4


        3-Methylbutyryl-CoA: 10.3


        Acetyl-CoA: 0.0

ENANITX01000091NITX01000091.1 - Region 1 - NRPS

Shows the layout of the region, marking coding sequences and areas of interest. Clicking a gene will select it and show any relevant details. Clicking an area feature (e.g. a candidate cluster) will select all coding sequences within that area. Double clicking an area feature will zoom to that area. Multiple genes and area features can be selected by clicking them while holding the Ctrl key.  
More detailed help is available here.

Download region GenBank file

Download region SVG

Location: 24,062 - 99,877 nt. (total: 75,816 nt)
Show pHMM detection rules used

NRPS: cds(Condensation and (AMP-binding or A-OX))

#### Legend:

core biosynthetic genes

additional biosynthetic genes

transport-related genes

regulatory genes

other genes

resistance

reset view

zoom to selection

Gene details

Shows details of the most recently selected gene, including names, products, location, and other annotations.

Select a gene to view the details available for it

Gene overview

NRPS/PKS domains

MIBiG comparison

ClusterBlast

KnownClusterBlast

SubClusterBlast

NRPS/PKS modules

Pfam domains

TIGRFAM domains

Gene/CDS overview

A brief tabular summary of genes/CDS features within the region.  
Filtering the table will also search biosynthetic profiles and gene function data. If enabled, the overview will then zoom to show the area covered by the filtered selection.  
Genes selected in the region drawing above will be marked in the table with an indicator to the left of the gene name.

Filter:

Automatically zoom to filtered/selected features

| Identifier | Product | Length | | Function | Sequence | | NCBI Blast | Filter details |
| --- | --- | --- | --- | --- | --- | --- | --- | --- |
|  |  | NT | AA |  | NT | AA |  |  |

No genes match the given filter

Detailed domain annotation

Shows NRPS- and PKS-related domains for each feature that contains them. Click on each domain for more information about the domain's location, consensus monomer prediction, and other details.  
A domain glossary is available here, and an explanation of the visualisation is available here.

Selected features only

Show module domains

Similar gene clusters

Shows careas that are similar to the current region to a reference database.  
Mouseover a score cell in the table to get a breakdown of how the score was calculated.The MIBiG database.  
  
Click on an accession to open that entry in the MIBiG database.

Analysis type:

Protocluster to Region
Region to Region

| Reference | NRPS | Similarity score | Type | Compound(s) | Organism |
| --- | --- | --- | --- | --- | --- |
| BGC0002286 |  | 0.20 | NRP | ririwpeptide A, ririwpeptide B, ririwpeptide C | Photorhabdus laumondii subsp. laumondii TTO1 |
| BGC0001641 |  | 0.20 | NRP | kolossin | Photorhabdus laumondii subsp. laumondii TTO1 |
| BGC0001825 |  | 0.20 | NRP | xenematide | Xenorhabdus nematophila AN6/1 |
| BGC0001240 |  | 0.20 | NRP | serinocyclin A, serinocyclin B | Metarhizium robertsii |
| BGC0001844 |  | 0.20 | NRP | holrhizin | Paraburkholderia rhizoxinica HKI 454 |
| BGC0001128 |  | 0.20 | NRP | gamexpeptide C | Photorhabdus laumondii subsp. laumondii TTO1 |
| BGC0002135 |  | 0.20 | NRP | bovienimide A | Xenorhabdus bovienii SS-2004 |
| BGC0001166 |  | 0.20 | NRP | HC-toxin | Alternaria jesenskae |
| BGC0002164 |  | 0.18 | NRP | peramine | Epichloe festucae |
| BGC0002157 |  | 0.18 | NRP, Alkaloid | (-)-ditryptophenaline | Aspergillus flavus |

| Reference | Aggregated | Similarity score | Type | Compound(s) | Organism |
| --- | --- | --- | --- | --- | --- |
| BGC0002286 |  | 0.53 | NRP | ririwpeptide A, ririwpeptide B, ririwpeptide C | Photorhabdus laumondii subsp. laumondii TTO1 |
| BGC0001641 |  | 0.52 | NRP | kolossin | Photorhabdus laumondii subsp. laumondii TTO1 |
| BGC0001825 |  | 0.52 | NRP | xenematide | Xenorhabdus nematophila AN6/1 |
| BGC0001240 |  | 0.52 | NRP | serinocyclin A, serinocyclin B | Metarhizium robertsii |
| BGC0001844 |  | 0.52 | NRP | holrhizin | Paraburkholderia rhizoxinica HKI 454 |
| BGC0001128 |  | 0.52 | NRP | gamexpeptide C | Photorhabdus laumondii subsp. laumondii TTO1 |
| BGC0002135 |  | 0.52 | NRP | bovienimide A | Xenorhabdus bovienii SS-2004 |
| BGC0001166 |  | 0.52 | NRP | HC-toxin | Alternaria jesenskae |
| BGC0002164 |  | 0.49 | NRP | peramine | Epichloe festucae |
| BGC0002157 |  | 0.49 | NRP, Alkaloid | (-)-ditryptophenaline | Aspergillus flavus |

Similar gene clusters

Shows regions from the antiSMASH database that are similar to the current region. Genes marked with the same colour are interrelated. White genes have no relationship.  
Click on reference genes to show details of similarities to genes within the current region.  
Click on an accession to open that entry in the antiSMASH database (if applicable).

All hits

NW\_021167079 (7466543-7521186): Sodiomyces alkalinus F11 unplaced genomic sca... (75% of genes show similarity), NRPS

NW\_009276922 (45361-100400): Verticillium dahliae VdLs.17 supercont1.10 genom... (62% of genes show similarity), NRPS

NW\_003315021 (40390-95422): Verticillium alfalfae VaMs.102 supercont1.18 geno... (55% of genes show similarity), NRPS

NW\_011942149 (5543357-5594041): Metarhizium robertsii ARSEF 23 MAA Scf 1, who... (46% of genes show similarity), NRPS

NW\_014013632 (105644-160356): Trichoderma atroviride IMI 206040 chromosome Un... (42% of genes show similarity), NRPS

NW\_014574715 (349608-404260): Metarhizium brunneum ARSEF 3297 chromosome Unkn... (41% of genes show similarity), NRPS

NC\_016457 (6139731->6190500): Thermothielavioides terrestris NRRL 8126 chromo... (35% of genes show similarity), NRPS

NW\_001914850 (2298019-2353277): Podospora anserina S mat+ genomic DNA chromos... (33% of genes show similarity), NRPS

NC\_035790 (6874142-6923850): Pochonia chlamydosporia 170 chromosome 1, whole ... (36% of genes show similarity), NRPS

NT\_165981 (3746214-3801488): Chaetomium globosum CBS 148.51 scaffold 6 genomi... (30% of genes show similarity), NRPS
Download graphic

Similar known gene clusters from MIBiG 3.1

Shows clusters from the MiBIG database that are similar to the current region. Genes marked with the same colour are interrelated. White genes have no relationship.  
Click on reference genes to show details of similarities to genes within the current region.  
Click on an accession to open that entry in the MiBIG database.

No matches found.

Similar subclusters

Shows sub-cluster units that are similar to the current region. Genes marked with the same colour are interrelated. White genes have no relationship.  
Click on reference genes to show details of similarities to genes within the current region.

No matches found.

Module view

Shows module structures for each candidate cluster in NRPS and PKS regions.   
Genes are shown in predicted order, and are only present when containing at least one complete module.   
A domain glossary is available here, and an explanation of the visualisation is available here.

Candidate 1 (24061 - 99877): single NRPS

Legend

Detailed Pfam domain annotation

Shows Pfam domains found in each gene within the region. Click on each domain for more information about the domain's accession, location, and description. Domains with a bold border have Gene Ontology information.

Selected features only

Expand to show all names

Detailed TIGRFAM domain annotation

Shows TIGRFAM domains found in each gene within the region. Click on each domain for more information about the domain's accession, location, and description.

Selected features only

Expand to show all names

NRPS/PKS products

NRPS/PKS monomers

Predicted core structure(s)

Shows estimated product structure and polymer for each candidate cluster in the region. To show the product, click on the expander or the candidate cluster feature drawn in the overview.

For candidate cluster 1, location 24061 - 99877:

Rough prediction of core scaffold based on assumed PKS/NRPS colinearity; tailoring reactions not taken into account

**Polymer prediction:**
:   (X - X - X)

  
Direct lookup in NORINE database:
strict
or
relaxed

Link to NORINE database query form

NRPS/PKS monomer predictions

Shows the predicted monomers for each adynelation domain and acyltransferase within genes. Each gene prediction can be expanded to view detailed predictions of each domain. Each prediction can be expanded to view the predictions by tool (and, for some tools, further expanded for extra details).

**input.path1.gene17**: X - X - X

:   **AMP-binding (115..514)**: X

    nrpys: (unknown)

    SVM prediction details:
    :   Predicted physicochemical class:
        :   N/A

        Large clusters prediction:
        :   N/A

        Small clusters prediction:
        :   N/A

        Single AA prediction:
        :   N/A

    Stachelhaus prediction details:
    :   Stachelhaus sequence:
        :   DVFELIMIHK

        Nearest Stachelhaus code(s):
        :   Orn DVMELSSITK (56% 8Å match)
        :   Ser DLFNLGLIHK (44% 8Å match)

        Stachelhaus code match:
        :   60% (weak)
:   **AMP-binding (1163..1390)**: X

    nrpys: (unknown)

    SVM prediction details:
    :   **NOTE: uncertain match**  

        Predicted physicochemical class:
        :   hydrophobic-aliphatic (Ala, Gly, Val, Leu, Ile, Abu, Ival, Ser, Thr, Hpg, Dhpg, Cys, Pro, Hpr)

        Large clusters prediction:
        :   N/A

        Small clusters prediction:
        :   N/A

        Single AA prediction:
        :   N/A

    Stachelhaus prediction details:
    :   Stachelhaus sequence:
        :   DVF------K

        Nearest Stachelhaus code(s):

        Stachelhaus code match:
        :   0% (weak)
:   **AMP-binding (2711..3109)**: X

    nrpys: (unknown)

    SVM prediction details:
    :   Predicted physicochemical class:
        :   N/A

        Large clusters prediction:
        :   N/A

        Small clusters prediction:
        :   N/A

        Single AA prediction:
        :   N/A

    Stachelhaus prediction details:
    :   Stachelhaus sequence:
        :   DVLDIGAIGK

        Nearest Stachelhaus code(s):
        :   Val DVLSFGAIIK (68% 8Å match)
        :   Phe or Trp DVLSIGAVVK (62% 8Å match)
        :   Arg DVESIGAIAK (56% 8Å match)

        Stachelhaus code match:
        :   70% (weak)

ENANITX01000105NITX01000105.1 - Region 1 - fungal-RiPP-like

Shows the layout of the region, marking coding sequences and areas of interest. Clicking a gene will select it and show any relevant details. Clicking an area feature (e.g. a candidate cluster) will select all coding sequences within that area. Double clicking an area feature will zoom to that area. Multiple genes and area features can be selected by clicking them while holding the Ctrl key.  
More detailed help is available here.

Download region GenBank file

Download region SVG

Location: 195,570 - 242,827 nt. (total: 47,258 nt)
Show pHMM detection rules used

Region on contig edge.

fungal-RiPP-like: DUF3328

#### Legend:

core biosynthetic genes

additional biosynthetic genes

transport-related genes

regulatory genes

other genes

resistance

reset view

zoom to selection

Gene details

Shows details of the most recently selected gene, including names, products, location, and other annotations.

Select a gene to view the details available for it

Gene overview

MIBiG comparison

ClusterBlast

KnownClusterBlast

SubClusterBlast

Pfam domains

TIGRFAM domains

Gene/CDS overview

A brief tabular summary of genes/CDS features within the region.  
Filtering the table will also search biosynthetic profiles and gene function data. If enabled, the overview will then zoom to show the area covered by the filtered selection.  
Genes selected in the region drawing above will be marked in the table with an indicator to the left of the gene name.

Filter:

Automatically zoom to filtered/selected features

| Identifier | Product | Length | | Function | Sequence | | NCBI Blast | Filter details |
| --- | --- | --- | --- | --- | --- | --- | --- | --- |
|  |  | NT | AA |  | NT | AA |  |  |

No genes match the given filter

Similar gene clusters

Shows careas that are similar to the current region to a reference database.  
Mouseover a score cell in the table to get a breakdown of how the score was calculated.The MIBiG database.  
  
Click on an accession to open that entry in the MIBiG database.

Analysis type:

Protocluster to Region
Region to Region

| Reference | fungal-RiPP-like | Similarity score | Type | Compound(s) | Organism |
| --- | --- | --- | --- | --- | --- |
| BGC0001720 |  | 0.08 | Polyketide | oosporein | Beauveria bassiana ARSEF 2860 |
| BGC0002213 |  | 0.07 | Polyketide | HEx-pks16 polyketide | Punctularia strigosozonata HHB-11173 SS5 |
| BGC0001587 |  | 0.05 | Other | ethylenediaminesuccinic acid hydroxyarginine (EDHA) | Streptomyces avermitilis MA-4680 = NBRC 14893 |
| BGC0000943 |  | 0.05 | Other | staphyloferrin B | Staphylococcus aureus subsp. aureus NCTC 8325 |
| BGC0002568 |  | 0.03 | Other | EDHA | Streptomyces scabiei 87.22 |

| Reference | Aggregated | Similarity score | Type | Compound(s) | Organism |
| --- | --- | --- | --- | --- | --- |
| BGC0001720 |  | 0.31 | Polyketide | oosporein | Beauveria bassiana ARSEF 2860 |
| BGC0002213 |  | 0.28 | Polyketide | HEx-pks16 polyketide | Punctularia strigosozonata HHB-11173 SS5 |
| BGC0001587 |  | 0.21 | Other | ethylenediaminesuccinic acid hydroxyarginine (EDHA) | Streptomyces avermitilis MA-4680 = NBRC 14893 |
| BGC0000943 |  | 0.21 | Other | staphyloferrin B | Staphylococcus aureus subsp. aureus NCTC 8325 |
| BGC0002568 |  | 0.17 | Other | EDHA | Streptomyces scabiei 87.22 |

Similar gene clusters

Shows regions from the antiSMASH database that are similar to the current region. Genes marked with the same colour are interrelated. White genes have no relationship.  
Click on reference genes to show details of similarities to genes within the current region.  
Click on an accession to open that entry in the antiSMASH database (if applicable).

No significant ClusterBlast hits found.

Similar known gene clusters from MIBiG 3.1

Shows clusters from the MiBIG database that are similar to the current region. Genes marked with the same colour are interrelated. White genes have no relationship.  
Click on reference genes to show details of similarities to genes within the current region.  
Click on an accession to open that entry in the MiBIG database.

No matches found.

Similar subclusters

Shows sub-cluster units that are similar to the current region. Genes marked with the same colour are interrelated. White genes have no relationship.  
Click on reference genes to show details of similarities to genes within the current region.

No matches found.

Detailed Pfam domain annotation

Shows Pfam domains found in each gene within the region. Click on each domain for more information about the domain's accession, location, and description. Domains with a bold border have Gene Ontology information.

Selected features only

Expand to show all names

Detailed TIGRFAM domain annotation

Shows TIGRFAM domains found in each gene within the region. Click on each domain for more information about the domain's accession, location, and description.

Selected features only

Expand to show all names

ENANITX01000114NITX01000114.1 - Region 1 - NRPS

Shows the layout of the region, marking coding sequences and areas of interest. Clicking a gene will select it and show any relevant details. Clicking an area feature (e.g. a candidate cluster) will select all coding sequences within that area. Double clicking an area feature will zoom to that area. Multiple genes and area features can be selected by clicking them while holding the Ctrl key.  
More detailed help is available here.

Download region GenBank file

Download region SVG

Location: 7,235 - 47,523 nt. (total: 40,289 nt)
Show pHMM detection rules used

Region on contig edge.

NRPS: cds(Condensation and (AMP-binding or A-OX))

#### Legend:

core biosynthetic genes

additional biosynthetic genes

transport-related genes

regulatory genes

other genes

resistance

reset view

zoom to selection

Gene details

Shows details of the most recently selected gene, including names, products, location, and other annotations.

Select a gene to view the details available for it

Gene overview

NRPS/PKS domains

MIBiG comparison

ClusterBlast

KnownClusterBlast

SubClusterBlast

NRPS/PKS modules

Pfam domains

TIGRFAM domains

Gene/CDS overview

A brief tabular summary of genes/CDS features within the region.  
Filtering the table will also search biosynthetic profiles and gene function data. If enabled, the overview will then zoom to show the area covered by the filtered selection.  
Genes selected in the region drawing above will be marked in the table with an indicator to the left of the gene name.

Filter:

Automatically zoom to filtered/selected features

| Identifier | Product | Length | | Function | Sequence | | NCBI Blast | Filter details |
| --- | --- | --- | --- | --- | --- | --- | --- | --- |
|  |  | NT | AA |  | NT | AA |  |  |

No genes match the given filter

Detailed domain annotation

Shows NRPS- and PKS-related domains for each feature that contains them. Click on each domain for more information about the domain's location, consensus monomer prediction, and other details.  
A domain glossary is available here, and an explanation of the visualisation is available here.

Selected features only

Show module domains

Similar gene clusters

Shows careas that are similar to the current region to a reference database.  
Mouseover a score cell in the table to get a breakdown of how the score was calculated.The MIBiG database.  
  
Click on an accession to open that entry in the MIBiG database.

Analysis type:

Protocluster to Region
Region to Region

| Reference | NRPS | Similarity score | Type | Compound(s) | Organism |
| --- | --- | --- | --- | --- | --- |
| BGC0002710 |  | 0.40 | NRP | metachelin C, metachelin A, metachelin A-CE, metachelin B, dimerumic acid 11-mannoside, dimerumic acid | Metarhizium robertsii ARSEF 23 |
| BGC0001249 |  | 0.38 | NRP | dimethylcoprogen | Alternaria alternata |
| BGC0000900 |  | 0.35 | Other | ferrichrome | Aspergillus oryzae |
| BGC0000348 |  | 0.27 | NRP | ergovaline | Epichloe festucae var. lolii |
| BGC0001261 |  | 0.26 | NRP | AM-toxin | Alternaria alternata |
| BGC0001517 |  | 0.26 | NRP | asperphenamate | Aspergillus terreus NIH2624 |
| BGC0002166 |  | 0.26 | NRP | peramine, intermediate 1, intermediate 2 | Metarhizium rileyi |
| BGC0002164 |  | 0.25 | NRP | peramine | Epichloe festucae |
| BGC0002157 |  | 0.24 | NRP, Alkaloid | (-)-ditryptophenaline | Aspergillus flavus |
| BGC0000980 |  | 0.24 | NRP, Polyketide | cylindrospermopsin | Aphanizomenon sp. 22D11 |

| Reference | Aggregated | Similarity score | Type | Compound(s) | Organism |
| --- | --- | --- | --- | --- | --- |
| BGC0002710 |  | 0.72 | NRP | metachelin C, metachelin A, metachelin A-CE, metachelin B, dimerumic acid 11-mannoside, dimerumic acid | Metarhizium robertsii ARSEF 23 |
| BGC0001249 |  | 0.71 | NRP | dimethylcoprogen | Alternaria alternata |
| BGC0000900 |  | 0.68 | Other | ferrichrome | Aspergillus oryzae |
| BGC0000348 |  | 0.61 | NRP | ergovaline | Epichloe festucae var. lolii |
| BGC0001261 |  | 0.60 | NRP | AM-toxin | Alternaria alternata |
| BGC0001517 |  | 0.59 | NRP | asperphenamate | Aspergillus terreus NIH2624 |
| BGC0002166 |  | 0.59 | NRP | peramine, intermediate 1, intermediate 2 | Metarhizium rileyi |
| BGC0002164 |  | 0.59 | NRP | peramine | Epichloe festucae |
| BGC0002157 |  | 0.58 | NRP, Alkaloid | (-)-ditryptophenaline | Aspergillus flavus |
| BGC0000980 |  | 0.57 | NRP, Polyketide | cylindrospermopsin | Aphanizomenon sp. 22D11 |

Similar gene clusters

Shows regions from the antiSMASH database that are similar to the current region. Genes marked with the same colour are interrelated. White genes have no relationship.  
Click on reference genes to show details of similarities to genes within the current region.  
Click on an accession to open that entry in the antiSMASH database (if applicable).

All hits

NW\_021167080 (1279846-1323576): Sodiomyces alkalinus F11 unplaced genomic sca... (60% of genes show similarity), NRPS

NC\_035791 (4607802-4652963): Pochonia chlamydosporia 170 chromosome 2, whole ... (58% of genes show similarity), NRPS

NW\_011942160 (3776462-3826079): Metarhizium robertsii ARSEF 23 MAA Scf 2, who... (50% of genes show similarity), NRPS

NW\_014574716 (6269401-6319031): Metarhizium brunneum ARSEF 3297 chromosome Un... (50% of genes show similarity), NRPS

NW\_014013633 (76225-119313): Trichoderma atroviride IMI 206040 chromosome Unk... (50% of genes show similarity), NRPS

NW\_006271973 (1002774-1052288): Cordyceps militaris CM01 unplaced genomic sca... (46% of genes show similarity), NRPS

NW\_009276926 (1339309-1390245): Verticillium dahliae VdLs.17 supercont1.6 gen... (37% of genes show similarity), NRPS

NW\_003315036 (1786754-1837693): Verticillium alfalfae VaMs.102 supercont1.3 g... (35% of genes show similarity), NRPS

CM004175 (8516549-8567297): Drechmeria coniospora strain ARSEF 6962 chromosom... (31% of genes show similarity), NRPS

NW\_013562494 (26196-92392): Exophiala xenobiotica strain CBS 118157 unplaced ... (28% of genes show similarity), NRPS
Download graphic

Similar known gene clusters from MIBiG 3.1

Shows clusters from the MiBIG database that are similar to the current region. Genes marked with the same colour are interrelated. White genes have no relationship.  
Click on reference genes to show details of similarities to genes within the current region.  
Click on an accession to open that entry in the MiBIG database.

All hits

metachelin C/metachelin A/metachelin A-CE/metachelin B/dimerumic acid 11-mannoside/dimerumic acid

dimethylcoprogen
Download graphic

Similar subclusters

Shows sub-cluster units that are similar to the current region. Genes marked with the same colour are interrelated. White genes have no relationship.  
Click on reference genes to show details of similarities to genes within the current region.

No matches found.

Module view

Shows module structures for each candidate cluster in NRPS and PKS regions.   
Genes are shown in predicted order, and are only present when containing at least one complete module.   
A domain glossary is available here, and an explanation of the visualisation is available here.

Candidate 1 (7234 - 47523): single NRPS

Legend

Detailed Pfam domain annotation

Shows Pfam domains found in each gene within the region. Click on each domain for more information about the domain's accession, location, and description. Domains with a bold border have Gene Ontology information.

Selected features only

Expand to show all names

Detailed TIGRFAM domain annotation

Shows TIGRFAM domains found in each gene within the region. Click on each domain for more information about the domain's accession, location, and description.

Selected features only

Expand to show all names

NRPS/PKS products

NRPS/PKS monomers

Predicted core structure(s)

Shows estimated product structure and polymer for each candidate cluster in the region. To show the product, click on the expander or the candidate cluster feature drawn in the overview.

For candidate cluster 1, location 7234 - 47523:

Rough prediction of core scaffold based on assumed PKS/NRPS colinearity; tailoring reactions not taken into account

**Polymer prediction:**
:   (X)

  
Direct lookup in NORINE database:
strict
or
relaxed

Link to NORINE database query form

NRPS/PKS monomer predictions

Shows the predicted monomers for each adynelation domain and acyltransferase within genes. Each gene prediction can be expanded to view detailed predictions of each domain. Each prediction can be expanded to view the predictions by tool (and, for some tools, further expanded for extra details).

**input.path1.gene4**: X

:   **AMP-binding (55..471)**: X

    nrpys: Z-ahmohOrn

    SVM prediction details:
    :   Predicted physicochemical class:
        :   hydrophobic-aromatic (Phe, Tyr, diOH-Bz, Ph-Gly, bOH-Tyr)

        Large clusters prediction:
        :   N/A

        Small clusters prediction:
        :   N/A

        Single AA prediction:
        :   N/A

    Stachelhaus prediction details:
    :   Stachelhaus sequence:
        :   FGLHIASTCK

        Nearest Stachelhaus code(s):
        :   Z-ahmohOrn FGLHIASTCK (97% 8Å match)

        Stachelhaus code match:
        :   100% (strong)

  
**input.path1.gene5**: X

:   **AMP-binding (5..412)**: X

    nrpys: (unknown)

    SVM prediction details:
    :   Predicted physicochemical class:
        :   N/A

        Large clusters prediction:
        :   N/A

        Small clusters prediction:
        :   N/A

        Single AA prediction:
        :   N/A

    Stachelhaus prediction details:
    :   Stachelhaus sequence:
        :   DVDHGGAVGK

        Nearest Stachelhaus code(s):
        :   Z-ahmohOrn DVDGGGGIGK (74% 8Å match)

        Stachelhaus code match:
        :   70% (weak)

ENANITX01000126NITX01000126.1 - Region 1 - terpene

Shows the layout of the region, marking coding sequences and areas of interest. Clicking a gene will select it and show any relevant details. Clicking an area feature (e.g. a candidate cluster) will select all coding sequences within that area. Double clicking an area feature will zoom to that area. Multiple genes and area features can be selected by clicking them while holding the Ctrl key.  
More detailed help is available here.

Download region GenBank file

Download region SVG

Location: 67,494 - 98,976 nt. (total: 31,483 nt)
Show pHMM detection rules used

terpene: (Terpene\_synth or Terpene\_synth\_C or phytoene\_synt or Lycopene\_cycl or terpene\_cyclase or NapT7 or fung\_ggpps or fung\_ggpps2 or trichodiene\_synth or TRI5)

#### Legend:

core biosynthetic genes

additional biosynthetic genes

transport-related genes

regulatory genes

other genes

resistance

reset view

zoom to selection

Gene details

Shows details of the most recently selected gene, including names, products, location, and other annotations.

Select a gene to view the details available for it

Gene overview

MIBiG comparison

ClusterBlast

KnownClusterBlast

SubClusterBlast

Pfam domains

TIGRFAM domains

Gene/CDS overview

A brief tabular summary of genes/CDS features within the region.  
Filtering the table will also search biosynthetic profiles and gene function data. If enabled, the overview will then zoom to show the area covered by the filtered selection.  
Genes selected in the region drawing above will be marked in the table with an indicator to the left of the gene name.

Filter:

Automatically zoom to filtered/selected features

| Identifier | Product | Length | | Function | Sequence | | NCBI Blast | Filter details |
| --- | --- | --- | --- | --- | --- | --- | --- | --- |
|  |  | NT | AA |  | NT | AA |  |  |

No genes match the given filter

Similar gene clusters

Shows careas that are similar to the current region to a reference database.  
Mouseover a score cell in the table to get a breakdown of how the score was calculated.The MIBiG database.  
  
Click on an accession to open that entry in the MIBiG database.

Analysis type:

Protocluster to Region
Region to Region

| Reference | terpene | Similarity score | Type | Compound(s) | Organism |
| --- | --- | --- | --- | --- | --- |
| BGC0002176 |  | 0.33 | Terpene | culmorin, (+)-juniperol(longiborneol), 15-acetyldeoxynivalenol | Fusarium graminearum PH-1 |
| BGC0000286 |  | 0.08 | Polyketide | viguiepinol | Streptomyces sp. KO-3988 |
| BGC0002561 |  | 0.08 | Alkaloid | phenazine SA, phenazine SB, phenazine SC | Streptomyces sp. |
| BGC0001080 |  | 0.05 | Other (Phenazine) | endophenazine A, endophenazine B | Streptomyces anulatus |
| BGC0000934 |  | 0.05 | Other (Phenazine) | 5-acetyl-5,10-dihydrophenazine-1-carboxylic acid, 5-(2-hydroxyacetyl)-5,10-dihydrophenazine-1-carboxylic acid, endophenazine A1, endophenazine F, endophenazine G | Kitasatospora sp. HKI 714 |
| BGC0001078 |  | 0.04 | Terpene, Polyketide | furaquinocin B | Streptomyces sp. KO-3988 |
| BGC0000679 |  | 0.03 | Terpene | diazepinomicin | Micromonospora sp. M42 |
| BGC0001079 |  | 0.03 | Polyketide, Terpene | napyradiomycin A80915C, napyradiomycin 2, napyradiomycin 3, napyradiomycin 4 | Streptomyces aculeolatus |
| BGC0001848 |  | 0.03 | Other | diazaquinomycin H, diazaquinomycin J | Micromonospora sp. B006 |

| Reference | Aggregated | Similarity score | Type | Compound(s) | Organism |
| --- | --- | --- | --- | --- | --- |
| BGC0002176 |  | 0.66 | Terpene | culmorin, (+)-juniperol(longiborneol), 15-acetyldeoxynivalenol | Fusarium graminearum PH-1 |
| BGC0000286 |  | 0.30 | Polyketide | viguiepinol | Streptomyces sp. KO-3988 |
| BGC0002561 |  | 0.30 | Alkaloid | phenazine SA, phenazine SB, phenazine SC | Streptomyces sp. |
| BGC0001080 |  | 0.24 | Other (Phenazine) | endophenazine A, endophenazine B | Streptomyces anulatus |
| BGC0000934 |  | 0.23 | Other (Phenazine) | 5-acetyl-5,10-dihydrophenazine-1-carboxylic acid, 5-(2-hydroxyacetyl)-5,10-dihydrophenazine-1-carboxylic acid, endophenazine A1, endophenazine F, endophenazine G | Kitasatospora sp. HKI 714 |
| BGC0001078 |  | 0.18 | Terpene, Polyketide | furaquinocin B | Streptomyces sp. KO-3988 |
| BGC0000679 |  | 0.17 | Terpene | diazepinomicin | Micromonospora sp. M42 |
| BGC0001079 |  | 0.16 | Polyketide, Terpene | napyradiomycin A80915C, napyradiomycin 2, napyradiomycin 3, napyradiomycin 4 | Streptomyces aculeolatus |
| BGC0001848 |  | 0.15 | Other | diazaquinomycin H, diazaquinomycin J | Micromonospora sp. B006 |

Similar gene clusters

Shows regions from the antiSMASH database that are similar to the current region. Genes marked with the same colour are interrelated. White genes have no relationship.  
Click on reference genes to show details of similarities to genes within the current region.  
Click on an accession to open that entry in the antiSMASH database (if applicable).

All hits

NW\_021167080 (2152204-2173522): Sodiomyces alkalinus F11 unplaced genomic sca... (85% of genes show similarity), terpene

NC\_030957 (2261520-2278481): Colletotrichum higginsianum IMI 349063 chromosom... (33% of genes show similarity), terpene

NW\_023336260 (1757882-1779297): Colletotrichum scovillei strain TJNH1 chromos... (33% of genes show similarity), terpene

NW\_023336263 (3001072-3022140): Colletotrichum scovillei strain TJNH1 chromos... (33% of genes show similarity), terpene

NW\_014574617 (215752-266030): Penicillium digitatum Pd1 unplaced genomic scaf... (11% of genes show similarity), NRPS
Download graphic

Similar known gene clusters from MIBiG 3.1

Shows clusters from the MiBIG database that are similar to the current region. Genes marked with the same colour are interrelated. White genes have no relationship.  
Click on reference genes to show details of similarities to genes within the current region.  
Click on an accession to open that entry in the MiBIG database.

No matches found.

Similar subclusters

Shows sub-cluster units that are similar to the current region. Genes marked with the same colour are interrelated. White genes have no relationship.  
Click on reference genes to show details of similarities to genes within the current region.

No matches found.

Detailed Pfam domain annotation

Shows Pfam domains found in each gene within the region. Click on each domain for more information about the domain's accession, location, and description. Domains with a bold border have Gene Ontology information.

Selected features only

Expand to show all names

Detailed TIGRFAM domain annotation

Shows TIGRFAM domains found in each gene within the region. Click on each domain for more information about the domain's accession, location, and description.

Selected features only

Expand to show all names

ENANITX01000140NITX01000140.1 - Region 1 - T1PKS

Shows the layout of the region, marking coding sequences and areas of interest. Clicking a gene will select it and show any relevant details. Clicking an area feature (e.g. a candidate cluster) will select all coding sequences within that area. Double clicking an area feature will zoom to that area. Multiple genes and area features can be selected by clicking them while holding the Ctrl key.  
More detailed help is available here.

Download region GenBank file

Download region SVG

Location: 1 - 50,812 nt. (total: 50,812 nt)
Show pHMM detection rules used

Region on contig edge.

T1PKS: cds(PKS\_AT and (PKS\_KS or ene\_KS or mod\_KS or hyb\_KS or itr\_KS or tra\_KS))

#### Legend:

core biosynthetic genes

additional biosynthetic genes

transport-related genes

regulatory genes

other genes

resistance

reset view

zoom to selection

Gene details

Shows details of the most recently selected gene, including names, products, location, and other annotations.

Select a gene to view the details available for it

Gene overview

NRPS/PKS domains

MIBiG comparison

ClusterBlast

KnownClusterBlast

SubClusterBlast

NRPS/PKS modules

Pfam domains

TIGRFAM domains

Gene/CDS overview

A brief tabular summary of genes/CDS features within the region.  
Filtering the table will also search biosynthetic profiles and gene function data. If enabled, the overview will then zoom to show the area covered by the filtered selection.  
Genes selected in the region drawing above will be marked in the table with an indicator to the left of the gene name.

Filter:

Automatically zoom to filtered/selected features

| Identifier | Product | Length | | Function | Sequence | | NCBI Blast | Filter details |
| --- | --- | --- | --- | --- | --- | --- | --- | --- |
|  |  | NT | AA |  | NT | AA |  |  |

No genes match the given filter

Detailed domain annotation

Shows NRPS- and PKS-related domains for each feature that contains them. Click on each domain for more information about the domain's location, consensus monomer prediction, and other details.  
A domain glossary is available here, and an explanation of the visualisation is available here.

Selected features only

Show module domains

Similar gene clusters

Shows careas that are similar to the current region to a reference database.  
Mouseover a score cell in the table to get a breakdown of how the score was calculated.The MIBiG database.  
  
Click on an accession to open that entry in the MIBiG database.

Analysis type:

Protocluster to Region
Region to Region

| Reference | T1PKS | Similarity score | Type | Compound(s) | Organism |
| --- | --- | --- | --- | --- | --- |
| BGC0002515 |  | 0.34 | Polyketide | solanapyrone A | Ascochyta rabiei |
| BGC0002191 |  | 0.32 | Polyketide | prolipyrone B, gibepyrone D | Fusarium graminearum PH-1 |
| BGC0002525 |  | 0.30 | Polyketide | fusarubin, 1233A, 1233B, NG-391, lucilactaene | Fusarium sp. |
| BGC0002155 |  | 0.29 | Polyketide | nectriapyrone C, nectriapyrone D, nectriapyrone | Pyricularia oryzae 70-15 |
| BGC0001252 |  | 0.29 | Polyketide | UNII-YC2Q1O94PT | Alternaria alternata |
| BGC0000146 |  | 0.29 | Polyketide | solanapyrone D | Alternaria solani |
| BGC0001899 |  | 0.28 | Polyketide | abscisic acid | Leptosphaeria maculans JN3 |
| BGC0002194 |  | 0.26 | Polyketide | epipyrone A | Epicoccum nigrum |
| BGC0002224 |  | 0.26 | Polyketide | zopfiellin | Diffractella curvata |
| BGC0002223 |  | 0.25 | Polyketide | scytalidin | Scytalidium album |

| Reference | Aggregated | Similarity score | Type | Compound(s) | Organism |
| --- | --- | --- | --- | --- | --- |
| BGC0002515 |  | 0.67 | Polyketide | solanapyrone A | Ascochyta rabiei |
| BGC0002191 |  | 0.65 | Polyketide | prolipyrone B, gibepyrone D | Fusarium graminearum PH-1 |
| BGC0002525 |  | 0.64 | Polyketide | fusarubin, 1233A, 1233B, NG-391, lucilactaene | Fusarium sp. |
| BGC0002155 |  | 0.63 | Polyketide | nectriapyrone C, nectriapyrone D, nectriapyrone | Pyricularia oryzae 70-15 |
| BGC0001252 |  | 0.63 | Polyketide | UNII-YC2Q1O94PT | Alternaria alternata |
| BGC0000146 |  | 0.63 | Polyketide | solanapyrone D | Alternaria solani |
| BGC0001899 |  | 0.61 | Polyketide | abscisic acid | Leptosphaeria maculans JN3 |
| BGC0002194 |  | 0.60 | Polyketide | epipyrone A | Epicoccum nigrum |
| BGC0002224 |  | 0.59 | Polyketide | zopfiellin | Diffractella curvata |
| BGC0002223 |  | 0.59 | Polyketide | scytalidin | Scytalidium album |

Similar gene clusters

Shows regions from the antiSMASH database that are similar to the current region. Genes marked with the same colour are interrelated. White genes have no relationship.  
Click on reference genes to show details of similarities to genes within the current region.  
Click on an accession to open that entry in the antiSMASH database (if applicable).

All hits

NW\_021167080 (3138044-3169744): Sodiomyces alkalinus F11 unplaced genomic sca... (58% of genes show similarity), T1PKS

NW\_006917091 (1569923-1612376): Pestalotiopsis fici W106-1 unplaced genomic s... (30% of genes show similarity), T1PKS

NW\_001914850 (107734-155722): Podospora anserina S mat+ genomic DNA chromosom... (17% of genes show similarity), T1PKS

NW\_003613579 (666561-714714): Podospora anserina S mat+ genomic DNA chromosom... (21% of genes show similarity), T1PKS

NT\_165982 (3368274-3416284): Chaetomium globosum CBS 148.51 scaffold 7 genomi... (25% of genes show similarity), T1PKS

NC\_026504 (1967-36181): Neurospora crassa OR74A linkage group IV, whole genom... (30% of genes show similarity), T1PKS

NZ\_CP041061 (3092951-3173303): Micromonospora sp. HM134 chromosome, complete ... (13% of genes show similarity), T1PKS

NZ\_CP022961 (4041696-4308611): Plantactinospora sp. KBS50 chromosome, complet... (4% of genes show similarity), NRPS,T1PKS,bacteriocin,blactam,transAT-PKS

CM002801 (1232608-1273325): Penicillium chrysogenum strain P2niaD18 chromosom... (15% of genes show similarity), T1PKS

NW\_023336277 (3526136-3573625): Aspergillus tubingensis WU-2223L DNA, scaffol... (11% of genes show similarity), T1PKS
Download graphic

Similar known gene clusters from MIBiG 3.1

Shows clusters from the MiBIG database that are similar to the current region. Genes marked with the same colour are interrelated. White genes have no relationship.  
Click on reference genes to show details of similarities to genes within the current region.  
Click on an accession to open that entry in the MiBIG database.

All hits

solanapyrone A

solanapyrone D
Download graphic

Similar subclusters

Shows sub-cluster units that are similar to the current region. Genes marked with the same colour are interrelated. White genes have no relationship.  
Click on reference genes to show details of similarities to genes within the current region.

No matches found.

Module view

Shows module structures for each candidate cluster in NRPS and PKS regions.   
Genes are shown in predicted order, and are only present when containing at least one complete module.   
A domain glossary is available here, and an explanation of the visualisation is available here.

Candidate 1 (0 - 50812): single T1PKS

Legend

Detailed Pfam domain annotation

Shows Pfam domains found in each gene within the region. Click on each domain for more information about the domain's accession, location, and description. Domains with a bold border have Gene Ontology information.

Selected features only

Expand to show all names

Detailed TIGRFAM domain annotation

Shows TIGRFAM domains found in each gene within the region. Click on each domain for more information about the domain's accession, location, and description.

Selected features only

Expand to show all names

NRPS/PKS products

NRPS/PKS monomers

Predicted core structure(s)

Shows estimated product structure and polymer for each candidate cluster in the region. To show the product, click on the expander or the candidate cluster feature drawn in the overview.

For candidate cluster 1, location 0 - 50812:

Rough prediction of core scaffold based on assumed PKS/NRPS colinearity; tailoring reactions not taken into account

**Polymer prediction:**
:   (pk)

  
Direct lookup in NORINE database:
strict
or
relaxed

Link to NORINE database query form

NRPS/PKS monomer predictions

Shows the predicted monomers for each adynelation domain and acyltransferase within genes. Each gene prediction can be expanded to view detailed predictions of each domain. Each prediction can be expanded to view the predictions by tool (and, for some tools, further expanded for extra details).

**input.path1.gene5**: pk

:   **PKS\_AT (600..939)**: pk

    ATSignature: Malonyl-CoA

    Top 3 matches:
    :   Malonyl-CoA: 70.8%
    :   Methylmalonyl-CoA: 62.5%
    :   Propionyl-CoA: 58.3%

      
    minowa: Methoxymalonyl-CoA

    Prediction, score:
    :   Methoxymalonyl-CoA: 64.5


        Methylmalonyl-CoA: 60.9


        Malonyl-CoA: 44.0


        Propionyl-CoA: 30.2


        Ethylmalonyl-CoA: 26.8


        Isobutyryl-CoA: 22.0


        fatty\_acid: 11.3


        CHC-CoA: 5.6


        trans-1,2-CPDA: 0.0


        inactive: 0.0


        Benzoyl-CoA: 0.0


        Acetyl-CoA: 0.0


        3-Methylbutyryl-CoA: 0.0


        2-Methylbutyryl-CoA: -5.5

ENANITX01000152NITX01000152.1 - Region 1 - T1PKS

Shows the layout of the region, marking coding sequences and areas of interest. Clicking a gene will select it and show any relevant details. Clicking an area feature (e.g. a candidate cluster) will select all coding sequences within that area. Double clicking an area feature will zoom to that area. Multiple genes and area features can be selected by clicking them while holding the Ctrl key.  
More detailed help is available here.

Download region GenBank file

Download region SVG

Location: 1 - 28,408 nt. (total: 28,408 nt)
Show pHMM detection rules used

Region on contig edge.

T1PKS: cds(PKS\_AT and (PKS\_KS or ene\_KS or mod\_KS or hyb\_KS or itr\_KS or tra\_KS))

#### Legend:

core biosynthetic genes

additional biosynthetic genes

transport-related genes

regulatory genes

other genes

resistance

reset view

zoom to selection

Gene details

Shows details of the most recently selected gene, including names, products, location, and other annotations.

Select a gene to view the details available for it

Gene overview

NRPS/PKS domains

MIBiG comparison

ClusterBlast

KnownClusterBlast

SubClusterBlast

Pfam domains

Gene/CDS overview

A brief tabular summary of genes/CDS features within the region.  
Filtering the table will also search biosynthetic profiles and gene function data. If enabled, the overview will then zoom to show the area covered by the filtered selection.  
Genes selected in the region drawing above will be marked in the table with an indicator to the left of the gene name.

Filter:

Automatically zoom to filtered/selected features

| Identifier | Product | Length | | Function | Sequence | | NCBI Blast | Filter details |
| --- | --- | --- | --- | --- | --- | --- | --- | --- |
|  |  | NT | AA |  | NT | AA |  |  |

No genes match the given filter

Detailed domain annotation

Shows NRPS- and PKS-related domains for each feature that contains them. Click on each domain for more information about the domain's location, consensus monomer prediction, and other details.  
A domain glossary is available here, and an explanation of the visualisation is available here.

Selected features only

Show module domains

Similar gene clusters

Shows careas that are similar to the current region to a reference database.  
Mouseover a score cell in the table to get a breakdown of how the score was calculated.The MIBiG database.  
  
Click on an accession to open that entry in the MIBiG database.

Analysis type:

Protocluster to Region
Region to Region

| Reference | T1PKS | Similarity score | Type | Compound(s) | Organism |
| --- | --- | --- | --- | --- | --- |
| BGC0002191 |  | 0.20 | Polyketide | prolipyrone B, gibepyrone D | Fusarium graminearum PH-1 |
| BGC0002240 |  | 0.20 | Polyketide | BAB, BAA | Metarhizium anisopliae |
| BGC0001254 |  | 0.20 | Polyketide | ACT-Toxin II | Alternaria alternata |
| BGC0002429 |  | 0.20 | Terpene, Polyketide | higginsianin B | Colletotrichum higginsianum IMI 349063 |
| BGC0000107 |  | 0.19 | Polyketide | naphthopyrone | Aspergillus nidulans FGSC A4 |
| BGC0002175 |  | 0.19 | Polyketide | YWA1 | Aspergillus oryzae RIB40 |
| BGC0001284 |  | 0.19 | Polyketide | alternariol | Parastagonospora nodorum SN15 |
| BGC0002745 |  | 0.19 | Polyketide | verrucosidin | Penicillium polonicum |
| BGC0001906 |  | 0.18 | Polyketide | naphthalene | Daldinia eschscholzii IFB-TL01 |
| BGC0001257 |  | 0.18 | Polyketide | 1,3,6,8-tetrahydroxynaphthalene | Nodulisporium sp. ATCC74245 |

| Reference | Aggregated | Similarity score | Type | Compound(s) | Organism |
| --- | --- | --- | --- | --- | --- |
| BGC0002191 |  | 0.53 | Polyketide | prolipyrone B, gibepyrone D | Fusarium graminearum PH-1 |
| BGC0002240 |  | 0.53 | Polyketide | BAB, BAA | Metarhizium anisopliae |
| BGC0001254 |  | 0.53 | Polyketide | ACT-Toxin II | Alternaria alternata |
| BGC0002429 |  | 0.52 | Terpene, Polyketide | higginsianin B | Colletotrichum higginsianum IMI 349063 |
| BGC0000107 |  | 0.51 | Polyketide | naphthopyrone | Aspergillus nidulans FGSC A4 |
| BGC0002175 |  | 0.51 | Polyketide | YWA1 | Aspergillus oryzae RIB40 |
| BGC0001284 |  | 0.50 | Polyketide | alternariol | Parastagonospora nodorum SN15 |
| BGC0002745 |  | 0.50 | Polyketide | verrucosidin | Penicillium polonicum |
| BGC0001906 |  | 0.50 | Polyketide | naphthalene | Daldinia eschscholzii IFB-TL01 |
| BGC0001257 |  | 0.50 | Polyketide | 1,3,6,8-tetrahydroxynaphthalene | Nodulisporium sp. ATCC74245 |

Similar gene clusters

Shows regions from the antiSMASH database that are similar to the current region. Genes marked with the same colour are interrelated. White genes have no relationship.  
Click on reference genes to show details of similarities to genes within the current region.  
Click on an accession to open that entry in the antiSMASH database (if applicable).

All hits

NW\_021167080 (4142387-4206356): Sodiomyces alkalinus F11 unplaced genomic sca... (38% of genes show similarity), T1PKS

CM002799 (5638577-5691101): Penicillium chrysogenum strain P2niaD18 chromosom... (50% of genes show similarity), T1PKS

NC\_049565 (2708272-2802515): Talaromyces rugulosus chromosome V, complete seq... (14% of genes show similarity), T1PKS

NW\_006917095 (2028347-2112797): Pestalotiopsis fici W106-1 unplaced genomic s... (20% of genes show similarity), T1PKS

NW\_003052500 (3389432-3427416): Uncinocarpus reesii 1704 scaffold 1 genomic s... (21% of genes show similarity), T1PKS

NC\_030957 (3306461-3350412): Colletotrichum higginsianum IMI 349063 chromosom... (21% of genes show similarity), T1PKS

NW\_003345195 (84932-125002): Nannizzia gypsea CBS 118893 supercont1.7 genomic... (20% of genes show similarity), T1PKS

NT\_165926 (<43-42903): Aspergillus terreus NIH2624 scaffold 3 genomic scaffol... (30% of genes show similarity), T1PKS

NC\_016475 (1389263-1436461): Thermothelomyces thermophilus ATCC 42464 chromos... (16% of genes show similarity), T1PKS

NW\_003299163 (808981-852528): Microsporum canis CBS 113480 supercont1.7 genom... (21% of genes show similarity), T1PKS
Download graphic

Similar known gene clusters from MIBiG 3.1

Shows clusters from the MiBIG database that are similar to the current region. Genes marked with the same colour are interrelated. White genes have no relationship.  
Click on reference genes to show details of similarities to genes within the current region.  
Click on an accession to open that entry in the MiBIG database.

All hits

depudecin
Download graphic

Similar subclusters

Shows sub-cluster units that are similar to the current region. Genes marked with the same colour are interrelated. White genes have no relationship.  
Click on reference genes to show details of similarities to genes within the current region.

No matches found.

Detailed Pfam domain annotation

Shows Pfam domains found in each gene within the region. Click on each domain for more information about the domain's accession, location, and description. Domains with a bold border have Gene Ontology information.

Selected features only

Expand to show all names

NRPS/PKS monomers

NRPS/PKS monomer predictions

Shows the predicted monomers for each adynelation domain and acyltransferase within genes. Each gene prediction can be expanded to view detailed predictions of each domain. Each prediction can be expanded to view the predictions by tool (and, for some tools, further expanded for extra details).

**input.path1.gene6**: mal

:   **PKS\_AT (585..910)**: mal

    ATSignature: Malonyl-CoA

    Top 3 matches:
    :   Malonyl-CoA: 70.8%
    :   Methylmalonyl-CoA: 58.3%

      
    minowa: Malonyl-CoA

    Prediction, score:
    :   Malonyl-CoA: 64.4


        Methoxymalonyl-CoA: 58.5


        Methylmalonyl-CoA: 58.4


        Isobutyryl-CoA: 32.9


        Propionyl-CoA: 14.5


        Benzoyl-CoA: 14.5


        inactive: 11.3


        Ethylmalonyl-CoA: 1.2


        trans-1,2-CPDA: 0.0


        fatty\_acid: 0.0


        CHC-CoA: 0.0


        Acetyl-CoA: 0.0


        3-Methylbutyryl-CoA: 0.0


        2-Methylbutyryl-CoA: 0.0

ENANITX01000159NITX01000159.1 - Region 1 - T1PKS

Shows the layout of the region, marking coding sequences and areas of interest. Clicking a gene will select it and show any relevant details. Clicking an area feature (e.g. a candidate cluster) will select all coding sequences within that area. Double clicking an area feature will zoom to that area. Multiple genes and area features can be selected by clicking them while holding the Ctrl key.  
More detailed help is available here.

Download region GenBank file

Download region SVG

Location: 1 - 65,417 nt. (total: 65,417 nt)
Show pHMM detection rules used

Region on contig edge.

T1PKS: cds(PKS\_AT and (PKS\_KS or ene\_KS or mod\_KS or hyb\_KS or itr\_KS or tra\_KS))

#### Legend:

core biosynthetic genes

additional biosynthetic genes

transport-related genes

regulatory genes

other genes

resistance

reset view

zoom to selection

Gene details

Shows details of the most recently selected gene, including names, products, location, and other annotations.

Select a gene to view the details available for it

Gene overview

NRPS/PKS domains

MIBiG comparison

ClusterBlast

KnownClusterBlast

SubClusterBlast

NRPS/PKS modules

Pfam domains

TIGRFAM domains

Gene/CDS overview

A brief tabular summary of genes/CDS features within the region.  
Filtering the table will also search biosynthetic profiles and gene function data. If enabled, the overview will then zoom to show the area covered by the filtered selection.  
Genes selected in the region drawing above will be marked in the table with an indicator to the left of the gene name.

Filter:

Automatically zoom to filtered/selected features

| Identifier | Product | Length | | Function | Sequence | | NCBI Blast | Filter details |
| --- | --- | --- | --- | --- | --- | --- | --- | --- |
|  |  | NT | AA |  | NT | AA |  |  |

No genes match the given filter

Detailed domain annotation

Shows NRPS- and PKS-related domains for each feature that contains them. Click on each domain for more information about the domain's location, consensus monomer prediction, and other details.  
A domain glossary is available here, and an explanation of the visualisation is available here.

Selected features only

Show module domains

Similar gene clusters

Shows careas that are similar to the current region to a reference database.  
Mouseover a score cell in the table to get a breakdown of how the score was calculated.The MIBiG database.  
  
Click on an accession to open that entry in the MIBiG database.

Analysis type:

Protocluster to Region
Region to Region

| Reference | T1PKS | Similarity score | Type | Compound(s) | Organism |
| --- | --- | --- | --- | --- | --- |
| BGC0002191 |  | 0.32 | Polyketide | prolipyrone B, gibepyrone D | Fusarium graminearum PH-1 |
| BGC0002155 |  | 0.31 | Polyketide | nectriapyrone C, nectriapyrone D, nectriapyrone | Pyricularia oryzae 70-15 |
| BGC0001606 |  | 0.29 | Polyketide | gibepyrone-A | Fusarium fujikuroi IMI 58289 |
| BGC0001252 |  | 0.28 | Polyketide | UNII-YC2Q1O94PT | Alternaria alternata |
| BGC0002222 |  | 0.26 | Polyketide | zopfiellin | Diffractella curvata |
| BGC0002525 |  | 0.26 | Polyketide | fusarubin, 1233A, 1233B, NG-391, lucilactaene | Fusarium sp. |
| BGC0002194 |  | 0.25 | Polyketide | epipyrone A | Epicoccum nigrum |
| BGC0002224 |  | 0.24 | Polyketide | zopfiellin | Diffractella curvata |
| BGC0000056 |  | 0.23 | Polyketide | esperamicin | Actinomadura verrucosospora |
| BGC0002515 |  | 0.22 | Polyketide | solanapyrone A | Ascochyta rabiei |

| Reference | Aggregated | Similarity score | Type | Compound(s) | Organism |
| --- | --- | --- | --- | --- | --- |
| BGC0002191 |  | 0.65 | Polyketide | prolipyrone B, gibepyrone D | Fusarium graminearum PH-1 |
| BGC0002155 |  | 0.64 | Polyketide | nectriapyrone C, nectriapyrone D, nectriapyrone | Pyricularia oryzae 70-15 |
| BGC0001606 |  | 0.63 | Polyketide | gibepyrone-A | Fusarium fujikuroi IMI 58289 |
| BGC0001252 |  | 0.61 | Polyketide | UNII-YC2Q1O94PT | Alternaria alternata |
| BGC0002222 |  | 0.60 | Polyketide | zopfiellin | Diffractella curvata |
| BGC0002525 |  | 0.59 | Polyketide | fusarubin, 1233A, 1233B, NG-391, lucilactaene | Fusarium sp. |
| BGC0002194 |  | 0.59 | Polyketide | epipyrone A | Epicoccum nigrum |
| BGC0002224 |  | 0.58 | Polyketide | zopfiellin | Diffractella curvata |
| BGC0000056 |  | 0.56 | Polyketide | esperamicin | Actinomadura verrucosospora |
| BGC0002515 |  | 0.55 | Polyketide | solanapyrone A | Ascochyta rabiei |

Similar gene clusters

Shows regions from the antiSMASH database that are similar to the current region. Genes marked with the same colour are interrelated. White genes have no relationship.  
Click on reference genes to show details of similarities to genes within the current region.  
Click on an accession to open that entry in the antiSMASH database (if applicable).

All hits

NW\_021167080 (4553879-4600163): Sodiomyces alkalinus F11 unplaced genomic sca... (92% of genes show similarity), T1PKS

NW\_006917101 (1419941-1468387): Pestalotiopsis fici W106-1 unplaced genomic s... (46% of genes show similarity), T1PKS

NW\_023336282 (3888174-3936319): Aspergillus tubingensis WU-2223L DNA, scaffol... (20% of genes show similarity), T1PKS

NW\_007360988 (15349-57851): Glarea lozoyensis ATCC 20868 chromosome Unknown G... (15% of genes show similarity), T1PKS

NW\_022984628 (39095-101054): Aspergillus tanneri strain NIH1004 chromosome Un... (11% of genes show similarity), NRPS,T1PKS

NW\_022474215 (800980-849315): Venustampulla echinocandica strain BP 5553 chro... (16% of genes show similarity), T1PKS

NC\_049563 (621195-675338): Talaromyces rugulosus chromosome III, complete seq... (10% of genes show similarity), NRPS-like,T1PKS

NW\_020167523 (1556917-1605272): Pseudogymnoascus destructans isolate 20631-21... (33% of genes show similarity), T1PKS

NW\_022474205 (5404643-5449301): Venustampulla echinocandica strain BP 5553 ch... (13% of genes show similarity), T1PKS

NW\_023336282 (927218-974229): Aspergillus tubingensis WU-2223L DNA, scaffold ... (11% of genes show similarity), T1PKS
Download graphic

Similar known gene clusters from MIBiG 3.1

Shows clusters from the MiBIG database that are similar to the current region. Genes marked with the same colour are interrelated. White genes have no relationship.  
Click on reference genes to show details of similarities to genes within the current region.  
Click on an accession to open that entry in the MiBIG database.

No matches found.

Similar subclusters

Shows sub-cluster units that are similar to the current region. Genes marked with the same colour are interrelated. White genes have no relationship.  
Click on reference genes to show details of similarities to genes within the current region.

No matches found.

Module view

Shows module structures for each candidate cluster in NRPS and PKS regions.   
Genes are shown in predicted order, and are only present when containing at least one complete module.   
A domain glossary is available here, and an explanation of the visualisation is available here.

Candidate 1 (0 - 65417): single T1PKS

Legend

Detailed Pfam domain annotation

Shows Pfam domains found in each gene within the region. Click on each domain for more information about the domain's accession, location, and description. Domains with a bold border have Gene Ontology information.

Selected features only

Expand to show all names

Detailed TIGRFAM domain annotation

Shows TIGRFAM domains found in each gene within the region. Click on each domain for more information about the domain's accession, location, and description.

Selected features only

Expand to show all names

NRPS/PKS products

NRPS/PKS monomers

Predicted core structure(s)

Shows estimated product structure and polymer for each candidate cluster in the region. To show the product, click on the expander or the candidate cluster feature drawn in the overview.

For candidate cluster 1, location 0 - 65417:

Rough prediction of core scaffold based on assumed PKS/NRPS colinearity; tailoring reactions not taken into account

**Polymer prediction:**
:   (pk)

  
Direct lookup in NORINE database:
strict
or
relaxed

Link to NORINE database query form

NRPS/PKS monomer predictions

Shows the predicted monomers for each adynelation domain and acyltransferase within genes. Each gene prediction can be expanded to view detailed predictions of each domain. Each prediction can be expanded to view the predictions by tool (and, for some tools, further expanded for extra details).

**input.path1.gene10**: pk

:   **PKS\_AT (656..962)**: pk

    ATSignature: Malonyl-CoA

    Top 3 matches:
    :   Malonyl-CoA: 62.5%
    :   Isobutyryl-CoA: 54.2%

      
    minowa: Methoxymalonyl-CoA

    Prediction, score:
    :   Methoxymalonyl-CoA: 65.5


        Malonyl-CoA: 54.5


        Isobutyryl-CoA: 46.1


        Ethylmalonyl-CoA: 37.7


        Propionyl-CoA: 26.7


        inactive: 22.7


        trans-1,2-CPDA: 16.8


        Benzoyl-CoA: 16.4


        CHC-CoA: 15.3


        fatty\_acid: 13.3


        2-Methylbutyryl-CoA: 10.0


        3-Methylbutyryl-CoA: 3.5


        Acetyl-CoA: 0.0


        Methylmalonyl-CoA: -2.9

ENANITX01000171NITX01000171.1 - Region 1 - NRPS,T1PKS

Shows the layout of the region, marking coding sequences and areas of interest. Clicking a gene will select it and show any relevant details. Clicking an area feature (e.g. a candidate cluster) will select all coding sequences within that area. Double clicking an area feature will zoom to that area. Multiple genes and area features can be selected by clicking them while holding the Ctrl key.  
More detailed help is available here.

Download region GenBank file

Download region SVG

Location: 1 - 21,602 nt. (total: 21,602 nt)
Show pHMM detection rules used

Region on contig edge.

NRPS: cds(Condensation and (AMP-binding or A-OX))  
T1PKS: cds(PKS\_AT and (PKS\_KS or ene\_KS or mod\_KS or hyb\_KS or itr\_KS or tra\_KS))

#### Legend:

core biosynthetic genes

additional biosynthetic genes

transport-related genes

regulatory genes

other genes

resistance

reset view

zoom to selection

Gene details

Shows details of the most recently selected gene, including names, products, location, and other annotations.

Select a gene to view the details available for it

Gene overview

NRPS/PKS domains

MIBiG comparison

ClusterBlast

KnownClusterBlast

SubClusterBlast

NRPS/PKS modules

Pfam domains

TIGRFAM domains

Gene/CDS overview

A brief tabular summary of genes/CDS features within the region.  
Filtering the table will also search biosynthetic profiles and gene function data. If enabled, the overview will then zoom to show the area covered by the filtered selection.  
Genes selected in the region drawing above will be marked in the table with an indicator to the left of the gene name.

Filter:

Automatically zoom to filtered/selected features

| Identifier | Product | Length | | Function | Sequence | | NCBI Blast | Filter details |
| --- | --- | --- | --- | --- | --- | --- | --- | --- |
|  |  | NT | AA |  | NT | AA |  |  |

No genes match the given filter

Detailed domain annotation

Shows NRPS- and PKS-related domains for each feature that contains them. Click on each domain for more information about the domain's location, consensus monomer prediction, and other details.  
A domain glossary is available here, and an explanation of the visualisation is available here.

Selected features only

Show module domains

Similar gene clusters

Shows careas that are similar to the current region to a reference database.  
Mouseover a score cell in the table to get a breakdown of how the score was calculated.The MIBiG database.  
  
Click on an accession to open that entry in the MIBiG database.

Analysis type:

Protocluster to Region
Region to Region

| Reference | NRPS | T1PKS | Similarity score | Type | Compound(s) | Organism |
| --- | --- | --- | --- | --- | --- | --- |
| BGC0000046 |  |  | 0.53 | Polyketide | depudecin | Alternaria brassicicola |
| BGC0001124 |  |  | 0.53 | Polyketide | pyranonigrin E | Aspergillus niger ATCC 1015 |
| BGC0001340 |  |  | 0.50 | Polyketide | byssochlamic acid | Byssochlamys fulva |
| BGC0002227 |  |  | 0.47 | NRP | AKML B, AKML D, AKML A, AKML C | Aspergillus luchuensis IFO 4308 |
| BGC0001913 |  |  | 0.45 | Polyketide | phomoidride | fungal sp. ATCC 74256 |
| BGC0001749 |  |  | 0.45 | Polyketide | epipyriculol | Pyricularia oryzae 70-15 |
| BGC0001187 |  |  | 0.44 | NRP, Polyketide | xenolozoyenone | Glarea lozoyensis |
| BGC0002192 |  |  | 0.44 | Polyketide | FR901512 | fungal sp. No.14919 |
| BGC0002191 |  |  | 0.43 | Polyketide | prolipyrone B, gibepyrone D | Fusarium graminearum PH-1 |
| BGC0002228 |  |  | 0.42 | NRP | CIML B, CIML A, CIML D, CIML C | Colletotrichum incanum |

| Reference | Aggregated | Similarity score | Type | Compound(s) | Organism |
| --- | --- | --- | --- | --- | --- |
| BGC0000046 |  | 0.60 | Polyketide | depudecin | Alternaria brassicicola |
| BGC0001124 |  | 0.60 | Polyketide | pyranonigrin E | Aspergillus niger ATCC 1015 |
| BGC0001340 |  | 0.58 | Polyketide | byssochlamic acid | Byssochlamys fulva |
| BGC0002227 |  | 0.56 | NRP | AKML B, AKML D, AKML A, AKML C | Aspergillus luchuensis IFO 4308 |
| BGC0001913 |  | 0.56 | Polyketide | phomoidride | fungal sp. ATCC 74256 |
| BGC0001749 |  | 0.56 | Polyketide | epipyriculol | Pyricularia oryzae 70-15 |
| BGC0001187 |  | 0.55 | NRP, Polyketide | xenolozoyenone | Glarea lozoyensis |
| BGC0002192 |  | 0.55 | Polyketide | FR901512 | fungal sp. No.14919 |
| BGC0002191 |  | 0.54 | Polyketide | prolipyrone B, gibepyrone D | Fusarium graminearum PH-1 |
| BGC0002228 |  | 0.54 | NRP | CIML B, CIML A, CIML D, CIML C | Colletotrichum incanum |

Similar gene clusters

Shows regions from the antiSMASH database that are similar to the current region. Genes marked with the same colour are interrelated. White genes have no relationship.  
Click on reference genes to show details of similarities to genes within the current region.  
Click on an accession to open that entry in the antiSMASH database (if applicable).

All hits

NW\_021167080 (5330881-5382708): Sodiomyces alkalinus F11 unplaced genomic sca... (23% of genes show similarity), NRPS,T1PKS

NW\_023336259 (1062896-1110407): Colletotrichum scovillei strain TJNH1 chromos... (27% of genes show similarity), NRPS,T1PKS

NW\_011942160 (2682302-2729789): Metarhizium robertsii ARSEF 23 MAA Scf 2, who... (20% of genes show similarity), NRPS,T1PKS

NW\_014574716 (5184553-5236629): Metarhizium brunneum ARSEF 3297 chromosome Un... (20% of genes show similarity), NRPS,T1PKS

NW\_009276921 (659963-706705): Verticillium dahliae VdLs.17 supercont1.1 genom... (18% of genes show similarity), T1PKS
Download graphic

Similar known gene clusters from MIBiG 3.1

Shows clusters from the MiBIG database that are similar to the current region. Genes marked with the same colour are interrelated. White genes have no relationship.  
Click on reference genes to show details of similarities to genes within the current region.  
Click on an accession to open that entry in the MiBIG database.

No matches found.

Similar subclusters

Shows sub-cluster units that are similar to the current region. Genes marked with the same colour are interrelated. White genes have no relationship.  
Click on reference genes to show details of similarities to genes within the current region.

No matches found.

Module view

Shows module structures for each candidate cluster in NRPS and PKS regions.   
Genes are shown in predicted order, and are only present when containing at least one complete module.   
A domain glossary is available here, and an explanation of the visualisation is available here.

Candidate 1 (0 - 21602): chemical hybrid NRPS-T1PKS

Legend

Detailed Pfam domain annotation

Shows Pfam domains found in each gene within the region. Click on each domain for more information about the domain's accession, location, and description. Domains with a bold border have Gene Ontology information.

Selected features only

Expand to show all names

Detailed TIGRFAM domain annotation

Shows TIGRFAM domains found in each gene within the region. Click on each domain for more information about the domain's accession, location, and description.

Selected features only

Expand to show all names

NRPS/PKS products

NRPS/PKS monomers

Predicted core structure(s)

Shows estimated product structure and polymer for each candidate cluster in the region. To show the product, click on the expander or the candidate cluster feature drawn in the overview.

For candidate cluster 1, location 0 - 21602:

Rough prediction of core scaffold based on assumed PKS/NRPS colinearity; tailoring reactions not taken into account

**Polymer prediction:**
:   (pk - Gly)

  
Direct lookup in NORINE database:
strict
or
relaxed

Link to NORINE database query form

NRPS/PKS monomer predictions

Shows the predicted monomers for each adynelation domain and acyltransferase within genes. Each gene prediction can be expanded to view detailed predictions of each domain. Each prediction can be expanded to view the predictions by tool (and, for some tools, further expanded for extra details).

**input.path1.gene4**: pk - Gly

:   Search NORINE for peptide:
    strict
    or
    relaxed
  
:   **PKS\_AT (544..855)**: pk

    ATSignature: Malonyl-CoA

    Top 3 matches:
    :   Malonyl-CoA: 79.2%
    :   Methylmalonyl-CoA: 62.5%
    :   inactive: 58.3%

      
    minowa: Methoxymalonyl-CoA

    Prediction, score:
    :   Methoxymalonyl-CoA: 80.7


        Methylmalonyl-CoA: 80.4


        Malonyl-CoA: 73.4


        Isobutyryl-CoA: 71.6


        Ethylmalonyl-CoA: 42.6


        inactive: 31.0


        Propionyl-CoA: 29.4


        trans-1,2-CPDA: 24.7


        fatty\_acid: 24.2


        Benzoyl-CoA: 20.8


        Acetyl-CoA: 13.2


        3-Methylbutyryl-CoA: 12.0


        2-Methylbutyryl-CoA: 9.5


        CHC-CoA: 0.0
:   **AMP-binding (2829..3243)**: Gly

    nrpys: Gly

    SVM prediction details:
    :   Predicted physicochemical class:
        :   hydrophobic-aliphatic (Ala, Gly, Val, Leu, Ile, Abu, Ival, Ser, Thr, Hpg, Dhpg, Cys, Pro, Hpr)

        Large clusters prediction:
        :   N/A

        Small clusters prediction:
        :   N/A

        Single AA prediction:
        :   N/A

    Stachelhaus prediction details:
    :   Stachelhaus sequence:
        :   DLVYYATVQK

        Nearest Stachelhaus code(s):
        :   Gly DLVYFATVQK (82% 8Å match)

        Stachelhaus code match:
        :   90% (moderate)

ENANITX01000192NITX01000192.1 - Region 1 - NRPS,T1PKS

Shows the layout of the region, marking coding sequences and areas of interest. Clicking a gene will select it and show any relevant details. Clicking an area feature (e.g. a candidate cluster) will select all coding sequences within that area. Double clicking an area feature will zoom to that area. Multiple genes and area features can be selected by clicking them while holding the Ctrl key.  
More detailed help is available here.

Download region GenBank file

Download region SVG

Location: 36,437 - 107,835 nt. (total: 71,399 nt)
Show pHMM detection rules used

NRPS: cds(Condensation and (AMP-binding or A-OX))  
T1PKS: cds(PKS\_AT and (PKS\_KS or ene\_KS or mod\_KS or hyb\_KS or itr\_KS or tra\_KS))

#### Legend:

core biosynthetic genes

additional biosynthetic genes

transport-related genes

regulatory genes

other genes

resistance

reset view

zoom to selection

Gene details

Shows details of the most recently selected gene, including names, products, location, and other annotations.

Select a gene to view the details available for it

Gene overview

NRPS/PKS domains

MIBiG comparison

ClusterBlast

KnownClusterBlast

SubClusterBlast

NRPS/PKS modules

Pfam domains

TIGRFAM domains

Gene/CDS overview

A brief tabular summary of genes/CDS features within the region.  
Filtering the table will also search biosynthetic profiles and gene function data. If enabled, the overview will then zoom to show the area covered by the filtered selection.  
Genes selected in the region drawing above will be marked in the table with an indicator to the left of the gene name.

Filter:

Automatically zoom to filtered/selected features

| Identifier | Product | Length | | Function | Sequence | | NCBI Blast | Filter details |
| --- | --- | --- | --- | --- | --- | --- | --- | --- |
|  |  | NT | AA |  | NT | AA |  |  |

No genes match the given filter

Detailed domain annotation

Shows NRPS- and PKS-related domains for each feature that contains them. Click on each domain for more information about the domain's location, consensus monomer prediction, and other details.  
A domain glossary is available here, and an explanation of the visualisation is available here.

Selected features only

Show module domains

Similar gene clusters

Shows careas that are similar to the current region to a reference database.  
Mouseover a score cell in the table to get a breakdown of how the score was calculated.The MIBiG database.  
  
Click on an accession to open that entry in the MIBiG database.

Analysis type:

Protocluster to Region
Region to Region

| Reference | NRPS | T1PKS | Similarity score | Type | Compound(s) | Organism |
| --- | --- | --- | --- | --- | --- | --- |
| BGC0000120 |  |  | 0.47 | Polyketide | patulin | Penicillium expansum |
| BGC0001627 |  |  | 0.44 | NRP | isonitrile lipopeptides | Mycobacterium tuberculosis H37Rv |
| BGC0001085 |  |  | 0.42 | NRP, Terpene | teleocidin B1 | Streptomyces blastmyceticus |
| BGC0001218 |  |  | 0.42 | NRP, Polyketide | fumosorinone | Cordyceps fumosorosea |
| BGC0001844 |  |  | 0.41 | NRP | holrhizin | Paraburkholderia rhizoxinica HKI 454 |
| BGC0002135 |  |  | 0.41 | NRP | bovienimide A | Xenorhabdus bovienii SS-2004 |
| BGC0001758 |  |  | 0.41 | NRP | rhizomide A, rhizomide B, rhizomide C | Paraburkholderia rhizoxinica HKI 454 |
| BGC0002437 |  |  | 0.40 | NRP | thermoactinoamide A | Thermoactinomyces sp. AS95 |
| BGC0001128 |  |  | 0.40 | NRP | gamexpeptide C | Photorhabdus laumondii subsp. laumondii TTO1 |
| BGC0002286 |  |  | 0.40 | NRP | ririwpeptide A, ririwpeptide B, ririwpeptide C | Photorhabdus laumondii subsp. laumondii TTO1 |

| Reference | Aggregated | Similarity score | Type | Compound(s) | Organism |
| --- | --- | --- | --- | --- | --- |
| BGC0000120 |  | 0.57 | Polyketide | patulin | Penicillium expansum |
| BGC0001627 |  | 0.55 | NRP | isonitrile lipopeptides | Mycobacterium tuberculosis H37Rv |
| BGC0001085 |  | 0.54 | NRP, Terpene | teleocidin B1 | Streptomyces blastmyceticus |
| BGC0001218 |  | 0.53 | NRP, Polyketide | fumosorinone | Cordyceps fumosorosea |
| BGC0001844 |  | 0.53 | NRP | holrhizin | Paraburkholderia rhizoxinica HKI 454 |
| BGC0002135 |  | 0.53 | NRP | bovienimide A | Xenorhabdus bovienii SS-2004 |
| BGC0001758 |  | 0.53 | NRP | rhizomide A, rhizomide B, rhizomide C | Paraburkholderia rhizoxinica HKI 454 |
| BGC0002437 |  | 0.53 | NRP | thermoactinoamide A | Thermoactinomyces sp. AS95 |
| BGC0001128 |  | 0.52 | NRP | gamexpeptide C | Photorhabdus laumondii subsp. laumondii TTO1 |
| BGC0002286 |  | 0.52 | NRP | ririwpeptide A, ririwpeptide B, ririwpeptide C | Photorhabdus laumondii subsp. laumondii TTO1 |

Similar gene clusters

Shows regions from the antiSMASH database that are similar to the current region. Genes marked with the same colour are interrelated. White genes have no relationship.  
Click on reference genes to show details of similarities to genes within the current region.  
Click on an accession to open that entry in the antiSMASH database (if applicable).

All hits

NW\_021167081 (277870-329989): Sodiomyces alkalinus F11 unplaced genomic scaff... (78% of genes show similarity), NRPS,T1PKS

NW\_003315034 (2149935-2225558): Verticillium alfalfae VaMs.102 supercont1.5 g... (33% of genes show similarity), NRPS,T1PKS

NW\_017971433 (600583-652209): Talaromyces atroroseus strain IBT 11181 chromos... (20% of genes show similarity), NRPS,T1PKS

NZ\_CP024190 (4464568-4554379): Mycobacterium marinum strain CCUG20998 chromos... (12% of genes show similarity), PKS-like,T1PKS

NC\_010612 (4639983-4740494): Mycobacterium marinum M, complete genome (9% of genes show similarity), NRPS,PKS-like,T1PKS

NW\_017264199 (1083767-1131168): Xylona heveae TC161 unplaced genomic scaffold... (12% of genes show similarity), T1PKS

NC\_021191 (2626192-2710257): Actinoplanes sp. N902-109, complete genome (9% of genes show similarity), T1PKS

NW\_003345199 (1003727-1098367): Nannizzia gypsea CBS 118893 supercont1.3 geno... (11% of genes show similarity), NRPS,T1PKS

NZ\_VNHJ01000012 (23780-71316): Oceanicella actignis strain DSM 22673 LY05DRAF... (6% of genes show similarity), T1PKS

NZ\_FOHL01000004 (223021-270629): Oceanicella actignis strain DSM 24423, whole... (6% of genes show similarity), T1PKS
Download graphic

Similar known gene clusters from MIBiG 3.1

Shows clusters from the MiBIG database that are similar to the current region. Genes marked with the same colour are interrelated. White genes have no relationship.  
Click on reference genes to show details of similarities to genes within the current region.  
Click on an accession to open that entry in the MiBIG database.

No matches found.

Similar subclusters

Shows sub-cluster units that are similar to the current region. Genes marked with the same colour are interrelated. White genes have no relationship.  
Click on reference genes to show details of similarities to genes within the current region.

No matches found.

Module view

Shows module structures for each candidate cluster in NRPS and PKS regions.   
Genes are shown in predicted order, and are only present when containing at least one complete module.   
A domain glossary is available here, and an explanation of the visualisation is available here.

Candidate 1 (36436 - 107835): chemical hybrid NRPS-T1PKS

Legend

Detailed Pfam domain annotation

Shows Pfam domains found in each gene within the region. Click on each domain for more information about the domain's accession, location, and description. Domains with a bold border have Gene Ontology information.

Selected features only

Expand to show all names

Detailed TIGRFAM domain annotation

Shows TIGRFAM domains found in each gene within the region. Click on each domain for more information about the domain's accession, location, and description.

Selected features only

Expand to show all names

NRPS/PKS products

NRPS/PKS monomers

Predicted core structure(s)

Shows estimated product structure and polymer for each candidate cluster in the region. To show the product, click on the expander or the candidate cluster feature drawn in the overview.

For candidate cluster 1, location 36436 - 107835:

Rough prediction of core scaffold based on assumed PKS/NRPS colinearity; tailoring reactions not taken into account

**Polymer prediction:**
:   (pk - X)

  
Direct lookup in NORINE database:
strict
or
relaxed

Link to NORINE database query form

NRPS/PKS monomer predictions

Shows the predicted monomers for each adynelation domain and acyltransferase within genes. Each gene prediction can be expanded to view detailed predictions of each domain. Each prediction can be expanded to view the predictions by tool (and, for some tools, further expanded for extra details).

**input.path1.gene15**: pk - X

:   **PKS\_AT (533..846)**: pk

    ATSignature: Malonyl-CoA

    Top 3 matches:
    :   Malonyl-CoA: 75.0%
    :   Isobutyryl-CoA: 66.7%
    :   Methylmalonyl-CoA: 62.5%

      
    minowa: Methylmalonyl-CoA

    Prediction, score:
    :   Methylmalonyl-CoA: 96.6


        Isobutyryl-CoA: 94.5


        Methoxymalonyl-CoA: 82.2


        Malonyl-CoA: 77.7


        Propionyl-CoA: 53.4


        trans-1,2-CPDA: 43.0


        2-Methylbutyryl-CoA: 36.2


        Benzoyl-CoA: 35.2


        inactive: 29.7


        3-Methylbutyryl-CoA: 23.3


        fatty\_acid: 18.8


        CHC-CoA: 16.3


        Acetyl-CoA: 16.0


        Ethylmalonyl-CoA: 2.7
:   **AMP-binding (2686..3055)**: X

    nrpys: Ala, Gly, Val, Leu, Ile, Abu, Ival, Ser, Thr, Hpg, Dhpg, Cys, Pro, Hpr

    SVM prediction details:
    :   Predicted physicochemical class:
        :   hydrophobic-aliphatic (Ala, Gly, Val, Leu, Ile, Abu, Ival, Ser, Thr, Hpg, Dhpg, Cys, Pro, Hpr)

        Large clusters prediction:
        :   N/A

        Small clusters prediction:
        :   N/A

        Single AA prediction:
        :   N/A

    Stachelhaus prediction details:
    :   Stachelhaus sequence:
        :   DMH-TGGIIK

        Nearest Stachelhaus code(s):
        :   Leu DMHMLGVIIK (41% 8Å match)

        Stachelhaus code match:
        :   70% (weak)

ENANITX01000192NITX01000192.1 - Region 2 - NRPS-like

Shows the layout of the region, marking coding sequences and areas of interest. Clicking a gene will select it and show any relevant details. Clicking an area feature (e.g. a candidate cluster) will select all coding sequences within that area. Double clicking an area feature will zoom to that area. Multiple genes and area features can be selected by clicking them while holding the Ctrl key.  
More detailed help is available here.

Download region GenBank file

Download region SVG

Location: 125,340 - 188,211 nt. (total: 62,872 nt)
Show pHMM detection rules used

NRPS-like: cds((PP-binding or NAD\_binding\_4) and (AMP-binding or A-OX))

#### Legend:

core biosynthetic genes

additional biosynthetic genes

transport-related genes

regulatory genes

other genes

resistance

reset view

zoom to selection

Gene details

Shows details of the most recently selected gene, including names, products, location, and other annotations.

Select a gene to view the details available for it

Gene overview

NRPS/PKS domains

MIBiG comparison

ClusterBlast

KnownClusterBlast

SubClusterBlast

NRPS/PKS modules

Pfam domains

Gene/CDS overview

A brief tabular summary of genes/CDS features within the region.  
Filtering the table will also search biosynthetic profiles and gene function data. If enabled, the overview will then zoom to show the area covered by the filtered selection.  
Genes selected in the region drawing above will be marked in the table with an indicator to the left of the gene name.

Filter:

Automatically zoom to filtered/selected features

| Identifier | Product | Length | | Function | Sequence | | NCBI Blast | Filter details |
| --- | --- | --- | --- | --- | --- | --- | --- | --- |
|  |  | NT | AA |  | NT | AA |  |  |

No genes match the given filter

Detailed domain annotation

Shows NRPS- and PKS-related domains for each feature that contains them. Click on each domain for more information about the domain's location, consensus monomer prediction, and other details.  
A domain glossary is available here, and an explanation of the visualisation is available here.

Selected features only

Show module domains

Similar gene clusters

Shows careas that are similar to the current region to a reference database.  
Mouseover a score cell in the table to get a breakdown of how the score was calculated.The MIBiG database.  
  
Click on an accession to open that entry in the MIBiG database.

Analysis type:

Protocluster to Region
Region to Region

| Reference | NRPS-like | Similarity score | Type | Compound(s) | Organism |
| --- | --- | --- | --- | --- | --- |
| BGC0002274 |  | 0.36 | NRP | aspulvinone H, aspulvinone B1 | Aspergillus terreus NIH2624 |
| BGC0002193 |  | 0.35 | Other | phenguignardic acid | Aspergillus terreus NIH2624 |
| BGC0002348 |  | 0.34 | NRP | aspulvinone E | Aspergillus terreus NIH2624 |
| BGC0002277 |  | 0.34 | NRP | atromentin | Suillus grevillei |
| BGC0001668 |  | 0.31 | NRP | microperfuranone | Aspergillus nidulans FGSC A4 |
| BGC0000442 |  | 0.27 | NRP | terrequinone A | Aspergillus nidulans FGSC A4 |
| BGC0000894 |  | 0.23 | Other | citrinin | Monascus aurantiacus |
| BGC0001712 |  | 0.22 | Other | ochrindole A | Aspergillus steynii IBT 23096 |
| BGC0001909 |  | 0.21 | Polyketide | strobilurin A | Strobilurus tenacellus |
| BGC0002271 |  | 0.18 | NRP | asterriquinone CT5 | Aspergillus terreus NIH2624 |

| Reference | Aggregated | Similarity score | Type | Compound(s) | Organism |
| --- | --- | --- | --- | --- | --- |
| BGC0002274 |  | 0.69 | NRP | aspulvinone H, aspulvinone B1 | Aspergillus terreus NIH2624 |
| BGC0002193 |  | 0.68 | Other | phenguignardic acid | Aspergillus terreus NIH2624 |
| BGC0002348 |  | 0.67 | NRP | aspulvinone E | Aspergillus terreus NIH2624 |
| BGC0002277 |  | 0.67 | NRP | atromentin | Suillus grevillei |
| BGC0001668 |  | 0.64 | NRP | microperfuranone | Aspergillus nidulans FGSC A4 |
| BGC0000442 |  | 0.60 | NRP | terrequinone A | Aspergillus nidulans FGSC A4 |
| BGC0000894 |  | 0.56 | Other | citrinin | Monascus aurantiacus |
| BGC0001712 |  | 0.54 | Other | ochrindole A | Aspergillus steynii IBT 23096 |
| BGC0001909 |  | 0.54 | Polyketide | strobilurin A | Strobilurus tenacellus |
| BGC0002271 |  | 0.49 | NRP | asterriquinone CT5 | Aspergillus terreus NIH2624 |

Similar gene clusters

Shows regions from the antiSMASH database that are similar to the current region. Genes marked with the same colour are interrelated. White genes have no relationship.  
Click on reference genes to show details of similarities to genes within the current region.  
Click on an accession to open that entry in the antiSMASH database (if applicable).

All hits

NW\_021167081 (367009-410018): Sodiomyces alkalinus F11 unplaced genomic scaff... (93% of genes show similarity), NRPS-like

NW\_011942151 (202104-242443): Metarhizium robertsii ARSEF 23 MAA Scf 11, whol... (28% of genes show similarity), NRPS-like

NW\_003299167 (2939649-2983011): Microsporum canis CBS 113480 supercont1.3 gen... (21% of genes show similarity), NRPS-like

NW\_003456427 (964233-999652): Trichophyton rubrum CBS 118892 genomic scaffold... (25% of genes show similarity), NRPS-like

NW\_014574694 (187294-230297): Metarhizium brunneum ARSEF 3297 chromosome Unkn... (30% of genes show similarity), NRPS-like

NT\_165977 (540278-580968): Chaetomium globosum CBS 148.51 scaffold 2 genomic ... (33% of genes show similarity), NRPS-like

NW\_003315109 (394166-437247): Trichophyton benhamiae CBS 112371 chromosome Un... (20% of genes show similarity), NRPS-like

NC\_049563 (2324897-2362519): Talaromyces rugulosus chromosome III, complete s... (23% of genes show similarity), NRPS-like

NW\_001914834 (578519-621534): Podospora anserina S mat+ genomic DNA chromosom... (20% of genes show similarity), NRPS-like

NC\_049561 (735278-788133): Talaromyces rugulosus chromosome I, complete sequence (13% of genes show similarity), NRPS-like,T1PKS
Download graphic

Similar known gene clusters from MIBiG 3.1

Shows clusters from the MiBIG database that are similar to the current region. Genes marked with the same colour are interrelated. White genes have no relationship.  
Click on reference genes to show details of similarities to genes within the current region.  
Click on an accession to open that entry in the MiBIG database.

No matches found.

Similar subclusters

Shows sub-cluster units that are similar to the current region. Genes marked with the same colour are interrelated. White genes have no relationship.  
Click on reference genes to show details of similarities to genes within the current region.

No matches found.

Module view

Shows module structures for each candidate cluster in NRPS and PKS regions.   
Genes are shown in predicted order, and are only present when containing at least one complete module.   
A domain glossary is available here, and an explanation of the visualisation is available here.

Candidate 2 (125339 - 188211): single NRPS-like

Legend

Detailed Pfam domain annotation

Shows Pfam domains found in each gene within the region. Click on each domain for more information about the domain's accession, location, and description. Domains with a bold border have Gene Ontology information.

Selected features only

Expand to show all names

NRPS/PKS products

NRPS/PKS monomers

Predicted core structure(s)

Shows estimated product structure and polymer for each candidate cluster in the region. To show the product, click on the expander or the candidate cluster feature drawn in the overview.

For candidate cluster 2, location 125339 - 188211:

Rough prediction of core scaffold based on assumed PKS/NRPS colinearity; tailoring reactions not taken into account

**Polymer prediction:**
:   (X)

  
Direct lookup in NORINE database:
strict
or
relaxed

Link to NORINE database query form

NRPS/PKS monomer predictions

Shows the predicted monomers for each adynelation domain and acyltransferase within genes. Each gene prediction can be expanded to view detailed predictions of each domain. Each prediction can be expanded to view the predictions by tool (and, for some tools, further expanded for extra details).

**input.path1.gene41**: X

:   **AMP-binding (76..486)**: X

    nrpys: 4-ohPpa

    SVM prediction details:
    :   Predicted physicochemical class:
        :   N/A

        Large clusters prediction:
        :   N/A

        Small clusters prediction:
        :   glu,gln (Glu, Gln)

        Single AA prediction:
        :   N/A

    Stachelhaus prediction details:
    :   Stachelhaus sequence:
        :   VAVFAGGAGK

        Nearest Stachelhaus code(s):
        :   4-ohPpa VAVFAGGAGK (76% 8Å match)

        Stachelhaus code match:
        :   100% (strong)

ENANITX01000206NITX01000206.1 - Region 1 - terpene

Shows the layout of the region, marking coding sequences and areas of interest. Clicking a gene will select it and show any relevant details. Clicking an area feature (e.g. a candidate cluster) will select all coding sequences within that area. Double clicking an area feature will zoom to that area. Multiple genes and area features can be selected by clicking them while holding the Ctrl key.  
More detailed help is available here.

Download region GenBank file

Download region SVG

Location: 13,911 - 47,643 nt. (total: 33,733 nt)
Show pHMM detection rules used

terpene: (Terpene\_synth or Terpene\_synth\_C or phytoene\_synt or Lycopene\_cycl or terpene\_cyclase or NapT7 or fung\_ggpps or fung\_ggpps2 or trichodiene\_synth or TRI5)

#### Legend:

core biosynthetic genes

additional biosynthetic genes

transport-related genes

regulatory genes

other genes

resistance

reset view

zoom to selection

Gene details

Shows details of the most recently selected gene, including names, products, location, and other annotations.

Select a gene to view the details available for it

Gene overview

MIBiG comparison

ClusterBlast

KnownClusterBlast

SubClusterBlast

Pfam domains

TIGRFAM domains

Gene/CDS overview

A brief tabular summary of genes/CDS features within the region.  
Filtering the table will also search biosynthetic profiles and gene function data. If enabled, the overview will then zoom to show the area covered by the filtered selection.  
Genes selected in the region drawing above will be marked in the table with an indicator to the left of the gene name.

Filter:

Automatically zoom to filtered/selected features

| Identifier | Product | Length | | Function | Sequence | | NCBI Blast | Filter details |
| --- | --- | --- | --- | --- | --- | --- | --- | --- |
|  |  | NT | AA |  | NT | AA |  |  |

No genes match the given filter

Similar gene clusters

Shows careas that are similar to the current region to a reference database.  
Mouseover a score cell in the table to get a breakdown of how the score was calculated.The MIBiG database.  
  
Click on an accession to open that entry in the MIBiG database.

Analysis type:

Protocluster to Region
Region to Region

| Reference | terpene | Similarity score | Type | Compound(s) | Organism |
| --- | --- | --- | --- | --- | --- |
| BGC0000622 |  | 0.31 | RiPP | megacin | Bacillus megaterium |
| BGC0001839 |  | 0.27 | Terpene | squalestatin S1 | Aspergillus sp. Z5 |
| BGC0000595 |  | 0.13 | RiPP | SCO-2138 | Streptomyces coelicolor A3(2) |
| BGC0000596 |  | 0.13 | RiPP | SLI-2138 | Streptomyces lividans TK24 |
| BGC0000597 |  | 0.10 | RiPP | SWA-2138 | Streptomyces sp. e14 |
| BGC0000842 |  | 0.07 | Other (Non-NRP beta-lactam) | carbapenem MM4550 | Streptomyces argenteolus |
| BGC0000865 |  | 0.06 | Other | eicoseicosapentaenoic acid | Photobacterium profundum SS9 |
| BGC0001339 |  | 0.05 | Polyketide | squalestatin S1 | Phoma sp. MF5453 |
| BGC0002122 |  | 0.05 | NRP | gramicidin S | Aneurinibacillus migulanus |
| BGC0002338 |  | 0.04 | Polyketide | sorangipyranone | Myxococcales bacterium |

| Reference | Aggregated | Similarity score | Type | Compound(s) | Organism |
| --- | --- | --- | --- | --- | --- |
| BGC0000622 |  | 0.64 | RiPP | megacin | Bacillus megaterium |
| BGC0001839 |  | 0.60 | Terpene | squalestatin S1 | Aspergillus sp. Z5 |
| BGC0000595 |  | 0.41 | RiPP | SCO-2138 | Streptomyces coelicolor A3(2) |
| BGC0000596 |  | 0.41 | RiPP | SLI-2138 | Streptomyces lividans TK24 |
| BGC0000597 |  | 0.36 | RiPP | SWA-2138 | Streptomyces sp. e14 |
| BGC0000842 |  | 0.28 | Other (Non-NRP beta-lactam) | carbapenem MM4550 | Streptomyces argenteolus |
| BGC0000865 |  | 0.26 | Other | eicoseicosapentaenoic acid | Photobacterium profundum SS9 |
| BGC0001339 |  | 0.23 | Polyketide | squalestatin S1 | Phoma sp. MF5453 |
| BGC0002122 |  | 0.22 | NRP | gramicidin S | Aneurinibacillus migulanus |
| BGC0002338 |  | 0.18 | Polyketide | sorangipyranone | Myxococcales bacterium |

Similar gene clusters

Shows regions from the antiSMASH database that are similar to the current region. Genes marked with the same colour are interrelated. White genes have no relationship.  
Click on reference genes to show details of similarities to genes within the current region.  
Click on an accession to open that entry in the antiSMASH database (if applicable).

All hits

NC\_016459 (1654781-1676459): Thermothielavioides terrestris NRRL 8126 chromos... (87% of genes show similarity), terpene

NW\_021167081 (1616604-1638292): Sodiomyces alkalinus F11 unplaced genomic sca... (83% of genes show similarity), terpene

NC\_026507 (1953450-1975103): Neurospora crassa OR74A linkage group VII, whole... (100% of genes show similarity), terpene

NW\_001914847 (163106-184737): Podospora anserina S mat+ genomic DNA chromosom... (71% of genes show similarity), terpene

NW\_009276944 (141519-163106): Verticillium dahliae VdLs.17 supercont1.13 geno... (100% of genes show similarity), terpene

NC\_016476 (3082694-3104368): Thermothelomyces thermophilus ATCC 42464 chromos... (100% of genes show similarity), terpene

NW\_015971145 (2878443-2899944): Sporothrix schenckii 1099-18 chromosome Unkno... (100% of genes show similarity), terpene

NT\_165983 (459288-480903): Chaetomium globosum CBS 148.51 scaffold 8 genomic ... (71% of genes show similarity), terpene

NW\_014013627 (1443836-1465419): Trichoderma atroviride IMI 206040 chromosome ... (100% of genes show similarity), terpene

NW\_003315030 (1140705-1161916): Verticillium alfalfae VaMs.102 supercont1.9 g... (100% of genes show similarity), terpene
Download graphic

Similar known gene clusters from MIBiG 3.1

Shows clusters from the MiBIG database that are similar to the current region. Genes marked with the same colour are interrelated. White genes have no relationship.  
Click on reference genes to show details of similarities to genes within the current region.  
Click on an accession to open that entry in the MiBIG database.

All hits

squalestatin S1
Download graphic

Similar subclusters

Shows sub-cluster units that are similar to the current region. Genes marked with the same colour are interrelated. White genes have no relationship.  
Click on reference genes to show details of similarities to genes within the current region.

No matches found.

Detailed Pfam domain annotation

Shows Pfam domains found in each gene within the region. Click on each domain for more information about the domain's accession, location, and description. Domains with a bold border have Gene Ontology information.

Selected features only

Expand to show all names

Detailed TIGRFAM domain annotation

Shows TIGRFAM domains found in each gene within the region. Click on each domain for more information about the domain's accession, location, and description.

Selected features only

Expand to show all names

ENANITX01000234NITX01000234.1 - Region 1 - T1PKS

Shows the layout of the region, marking coding sequences and areas of interest. Clicking a gene will select it and show any relevant details. Clicking an area feature (e.g. a candidate cluster) will select all coding sequences within that area. Double clicking an area feature will zoom to that area. Multiple genes and area features can be selected by clicking them while holding the Ctrl key.  
More detailed help is available here.

Download region GenBank file

Download region SVG

Location: 4,133 - 54,076 nt. (total: 49,944 nt)
Show pHMM detection rules used

Region on contig edge.

T1PKS: cds(PKS\_AT and (PKS\_KS or ene\_KS or mod\_KS or hyb\_KS or itr\_KS or tra\_KS))

#### Legend:

core biosynthetic genes

additional biosynthetic genes

transport-related genes

regulatory genes

other genes

resistance

reset view

zoom to selection

Gene details

Shows details of the most recently selected gene, including names, products, location, and other annotations.

Select a gene to view the details available for it

Gene overview

NRPS/PKS domains

MIBiG comparison

ClusterBlast

KnownClusterBlast

SubClusterBlast

NRPS/PKS modules

Pfam domains

Gene/CDS overview

A brief tabular summary of genes/CDS features within the region.  
Filtering the table will also search biosynthetic profiles and gene function data. If enabled, the overview will then zoom to show the area covered by the filtered selection.  
Genes selected in the region drawing above will be marked in the table with an indicator to the left of the gene name.

Filter:

Automatically zoom to filtered/selected features

| Identifier | Product | Length | | Function | Sequence | | NCBI Blast | Filter details |
| --- | --- | --- | --- | --- | --- | --- | --- | --- |
|  |  | NT | AA |  | NT | AA |  |  |

No genes match the given filter

Detailed domain annotation

Shows NRPS- and PKS-related domains for each feature that contains them. Click on each domain for more information about the domain's location, consensus monomer prediction, and other details.  
A domain glossary is available here, and an explanation of the visualisation is available here.

Selected features only

Show module domains

Similar gene clusters

Shows careas that are similar to the current region to a reference database.  
Mouseover a score cell in the table to get a breakdown of how the score was calculated.The MIBiG database.  
  
Click on an accession to open that entry in the MIBiG database.

Analysis type:

Protocluster to Region
Region to Region

| Reference | T1PKS | Similarity score | Type | Compound(s) | Organism |
| --- | --- | --- | --- | --- | --- |
| BGC0000046 |  | 0.27 | Polyketide | depudecin | Alternaria brassicicola |
| BGC0002227 |  | 0.25 | NRP | AKML B, AKML D, AKML A, AKML C | Aspergillus luchuensis IFO 4308 |
| BGC0002228 |  | 0.24 | NRP | CIML B, CIML A, CIML D, CIML C | Colletotrichum incanum |
| BGC0001913 |  | 0.23 | Polyketide | phomoidride | fungal sp. ATCC 74256 |
| BGC0001124 |  | 0.22 | Polyketide | pyranonigrin E | Aspergillus niger ATCC 1015 |
| BGC0001340 |  | 0.22 | Polyketide | byssochlamic acid | Byssochlamys fulva |
| BGC0002266 |  | 0.22 | Terpene, Polyketide | calidoustene A, calidoustene B, calidoustene C | Aspergillus calidoustus |
| BGC0002180 |  | 0.22 | Polyketide | (+)-asperlin, (2Z,4Z,6E)-octa-2,4,6-trienoic acid | Aspergillus nidulans FGSC A4 |
| BGC0002211 |  | 0.21 | Polyketide | HEx-pks1 polyketide | Paraphaeosphaeria sporulosa |
| BGC0002234 |  | 0.20 | Polyketide | flavoglaucin | Aspergillus ruber CBS 135680 |

| Reference | Aggregated | Similarity score | Type | Compound(s) | Organism |
| --- | --- | --- | --- | --- | --- |
| BGC0000046 |  | 0.60 | Polyketide | depudecin | Alternaria brassicicola |
| BGC0002227 |  | 0.59 | NRP | AKML B, AKML D, AKML A, AKML C | Aspergillus luchuensis IFO 4308 |
| BGC0002228 |  | 0.57 | NRP | CIML B, CIML A, CIML D, CIML C | Colletotrichum incanum |
| BGC0001913 |  | 0.56 | Polyketide | phomoidride | fungal sp. ATCC 74256 |
| BGC0001124 |  | 0.55 | Polyketide | pyranonigrin E | Aspergillus niger ATCC 1015 |
| BGC0001340 |  | 0.55 | Polyketide | byssochlamic acid | Byssochlamys fulva |
| BGC0002266 |  | 0.55 | Terpene, Polyketide | calidoustene A, calidoustene B, calidoustene C | Aspergillus calidoustus |
| BGC0002180 |  | 0.55 | Polyketide | (+)-asperlin, (2Z,4Z,6E)-octa-2,4,6-trienoic acid | Aspergillus nidulans FGSC A4 |
| BGC0002211 |  | 0.53 | Polyketide | HEx-pks1 polyketide | Paraphaeosphaeria sporulosa |
| BGC0002234 |  | 0.52 | Polyketide | flavoglaucin | Aspergillus ruber CBS 135680 |

Similar gene clusters

Shows regions from the antiSMASH database that are similar to the current region. Genes marked with the same colour are interrelated. White genes have no relationship.  
Click on reference genes to show details of similarities to genes within the current region.  
Click on an accession to open that entry in the antiSMASH database (if applicable).

All hits

NW\_021167082 (107498-155745): Sodiomyces alkalinus F11 unplaced genomic scaff... (84% of genes show similarity), T1PKS

NW\_022194797 (2177790-2225517): Fusarium proliferatum ET1 genome assembly, co... (28% of genes show similarity), T1PKS

NC\_036631 (2096864-2144579): Fusarium fujikuroi IMI 58289 draft genome, chrom... (27% of genes show similarity), T1PKS

NC\_030956 (1267040-1325778): Colletotrichum higginsianum IMI 349063 chromosom... (26% of genes show similarity), T1PKS,indole

NW\_014013635 (717478-764870): Trichoderma atroviride IMI 206040 chromosome Un... (27% of genes show similarity), T1PKS

NW\_022984633 (1531709-1573817): Aspergillus tanneri strain NIH1004 chromosome... (21% of genes show similarity), T1PKS

NW\_007361002 (1736341-1775942): Glarea lozoyensis ATCC 20868 chromosome Unkno... (13% of genes show similarity), T1PKS

NW\_001820810 (191380-239137): Sclerotinia sclerotiorum 1980 UF-70 scaffold 26... (12% of genes show similarity), NRPS,T1PKS

NC\_026501 (8672541-8719834): Neurospora crassa OR74A linkage group I, whole g... (26% of genes show similarity), T1PKS

NW\_023336285 (511329-554581): Aspergillus tubingensis WU-2223L DNA, scaffold ... (21% of genes show similarity), T1PKS
Download graphic

Similar known gene clusters from MIBiG 3.1

Shows clusters from the MiBIG database that are similar to the current region. Genes marked with the same colour are interrelated. White genes have no relationship.  
Click on reference genes to show details of similarities to genes within the current region.  
Click on an accession to open that entry in the MiBIG database.

All hits

flavoglaucin

trichoxide
Download graphic

Similar subclusters

Shows sub-cluster units that are similar to the current region. Genes marked with the same colour are interrelated. White genes have no relationship.  
Click on reference genes to show details of similarities to genes within the current region.

No matches found.

Module view

Shows module structures for each candidate cluster in NRPS and PKS regions.   
Genes are shown in predicted order, and are only present when containing at least one complete module.   
A domain glossary is available here, and an explanation of the visualisation is available here.

Candidate 1 (4132 - 54076): single T1PKS

Legend

Detailed Pfam domain annotation

Shows Pfam domains found in each gene within the region. Click on each domain for more information about the domain's accession, location, and description. Domains with a bold border have Gene Ontology information.

Selected features only

Expand to show all names

NRPS/PKS products

NRPS/PKS monomers

Predicted core structure(s)

Shows estimated product structure and polymer for each candidate cluster in the region. To show the product, click on the expander or the candidate cluster feature drawn in the overview.

For candidate cluster 1, location 4132 - 54076:

Rough prediction of core scaffold based on assumed PKS/NRPS colinearity; tailoring reactions not taken into account

**Polymer prediction:**
:   (pk)

  
Direct lookup in NORINE database:
strict
or
relaxed

Link to NORINE database query form

NRPS/PKS monomer predictions

Shows the predicted monomers for each adynelation domain and acyltransferase within genes. Each gene prediction can be expanded to view detailed predictions of each domain. Each prediction can be expanded to view the predictions by tool (and, for some tools, further expanded for extra details).

**input.path1.gene6**: pk

:   **PKS\_AT (573..898)**: pk

    ATSignature: Malonyl-CoA

    Top 3 matches:
    :   Malonyl-CoA: 70.8%
    :   Ethylmalonyl-CoA: 58.3%
    :   Methylmalonyl-CoA: 54.2%

      
    minowa: Methylmalonyl-CoA

    Prediction, score:
    :   Methylmalonyl-CoA: 61.5


        Methoxymalonyl-CoA: 52.5


        Malonyl-CoA: 42.1


        Ethylmalonyl-CoA: 25.2


        Propionyl-CoA: 24.6


        trans-1,2-CPDA: 17.6


        fatty\_acid: 16.2


        CHC-CoA: 16.0


        Isobutyryl-CoA: 15.6


        Benzoyl-CoA: 14.7


        Acetyl-CoA: 11.6


        inactive: 0.0


        3-Methylbutyryl-CoA: 0.0


        2-Methylbutyryl-CoA: 0.0

ENANITX01000352NITX01000352.1 - Region 1 - T1PKS

Shows the layout of the region, marking coding sequences and areas of interest. Clicking a gene will select it and show any relevant details. Clicking an area feature (e.g. a candidate cluster) will select all coding sequences within that area. Double clicking an area feature will zoom to that area. Multiple genes and area features can be selected by clicking them while holding the Ctrl key.  
More detailed help is available here.

Download region GenBank file

Download region SVG

Location: 132,661 - 201,094 nt. (total: 68,434 nt)
Show pHMM detection rules used

T1PKS: cds(PKS\_AT and (PKS\_KS or ene\_KS or mod\_KS or hyb\_KS or itr\_KS or tra\_KS))

#### Legend:

core biosynthetic genes

additional biosynthetic genes

transport-related genes

regulatory genes

other genes

resistance

reset view

zoom to selection

Gene details

Shows details of the most recently selected gene, including names, products, location, and other annotations.

Select a gene to view the details available for it

Gene overview

NRPS/PKS domains

MIBiG comparison

ClusterBlast

KnownClusterBlast

SubClusterBlast

Pfam domains

TIGRFAM domains

Gene/CDS overview

A brief tabular summary of genes/CDS features within the region.  
Filtering the table will also search biosynthetic profiles and gene function data. If enabled, the overview will then zoom to show the area covered by the filtered selection.  
Genes selected in the region drawing above will be marked in the table with an indicator to the left of the gene name.

Filter:

Automatically zoom to filtered/selected features

| Identifier | Product | Length | | Function | Sequence | | NCBI Blast | Filter details |
| --- | --- | --- | --- | --- | --- | --- | --- | --- |
|  |  | NT | AA |  | NT | AA |  |  |

No genes match the given filter

Detailed domain annotation

Shows NRPS- and PKS-related domains for each feature that contains them. Click on each domain for more information about the domain's location, consensus monomer prediction, and other details.  
A domain glossary is available here, and an explanation of the visualisation is available here.

Selected features only

Show module domains

Similar gene clusters

Shows careas that are similar to the current region to a reference database.  
Mouseover a score cell in the table to get a breakdown of how the score was calculated.The MIBiG database.  
  
Click on an accession to open that entry in the MIBiG database.

Analysis type:

Protocluster to Region
Region to Region

| Reference | T1PKS | Similarity score | Type | Compound(s) | Organism |
| --- | --- | --- | --- | --- | --- |
| BGC0002610 |  | 0.34 | Terpene | stellatic acid | Aspergillus stellatus |
| BGC0001659 |  | 0.28 | Terpene | mangicol A | Fusarium equiseti |
| BGC0002228 |  | 0.25 | NRP | CIML B, CIML A, CIML D, CIML C | Colletotrichum incanum |
| BGC0002245 |  | 0.25 | Terpene | terpestacin, preterpestacin 3, preterpestacin 2, preterpestacin 1 | Bipolaris maydis C5 |
| BGC0002320 |  | 0.25 | Terpene | conidiogenone | Penicillium rubens Wisconsin 54-1255 |
| BGC0001909 |  | 0.24 | Polyketide | strobilurin A | Strobilurus tenacellus |
| BGC0002211 |  | 0.23 | Polyketide | HEx-pks1 polyketide | Paraphaeosphaeria sporulosa |
| BGC0000894 |  | 0.23 | Other | citrinin | Monascus aurantiacus |
| BGC0000677 |  | 0.23 | Terpene | cyclooctatin | Streptomyces melanosporofaciens |
| BGC0001528 |  | 0.22 | Terpene | betaestacin | Phoma betae |

| Reference | Aggregated | Similarity score | Type | Compound(s) | Organism |
| --- | --- | --- | --- | --- | --- |
| BGC0002610 |  | 0.67 | Terpene | stellatic acid | Aspergillus stellatus |
| BGC0001659 |  | 0.62 | Terpene | mangicol A | Fusarium equiseti |
| BGC0002228 |  | 0.59 | NRP | CIML B, CIML A, CIML D, CIML C | Colletotrichum incanum |
| BGC0002245 |  | 0.59 | Terpene | terpestacin, preterpestacin 3, preterpestacin 2, preterpestacin 1 | Bipolaris maydis C5 |
| BGC0002320 |  | 0.58 | Terpene | conidiogenone | Penicillium rubens Wisconsin 54-1255 |
| BGC0001909 |  | 0.58 | Polyketide | strobilurin A | Strobilurus tenacellus |
| BGC0002211 |  | 0.56 | Polyketide | HEx-pks1 polyketide | Paraphaeosphaeria sporulosa |
| BGC0000894 |  | 0.56 | Other | citrinin | Monascus aurantiacus |
| BGC0000677 |  | 0.56 | Terpene | cyclooctatin | Streptomyces melanosporofaciens |
| BGC0001528 |  | 0.55 | Terpene | betaestacin | Phoma betae |

Similar gene clusters

Shows regions from the antiSMASH database that are similar to the current region. Genes marked with the same colour are interrelated. White genes have no relationship.  
Click on reference genes to show details of similarities to genes within the current region.  
Click on an accession to open that entry in the antiSMASH database (if applicable).

All hits

NW\_021167084 (1847567-1897553): Sodiomyces alkalinus F11 unplaced genomic sca... (68% of genes show similarity), T1PKS

NW\_022983863 (624230-690958): Arthroderma uncinatum strain CBS 119779 chromos... (20% of genes show similarity), T1PKS

NC\_049565 (2708272-2802515): Talaromyces rugulosus chromosome V, complete seq... (25% of genes show similarity), T1PKS

NW\_020194484 (12340-73776): Amorphotheca resinae ATCC 22711 unplaced genomic ... (41% of genes show similarity), T1PKS,terpene

NW\_022474205 (7029281-7077036): Venustampulla echinocandica strain BP 5553 ch... (35% of genes show similarity), T1PKS

NW\_022984632 (251759-297619): Aspergillus tanneri strain NIH1004 chromosome U... (35% of genes show similarity), T1PKS

NW\_017971434 (336405-382902): Talaromyces atroroseus strain IBT 11181 chromos... (25% of genes show similarity), T1PKS

NW\_006271974 (2331315-2395136): Cordyceps militaris CM01 unplaced genomic sca... (40% of genes show similarity), NRPS,T1PKS

NW\_006917095 (2028347-2112797): Pestalotiopsis fici W106-1 unplaced genomic s... (20% of genes show similarity), T1PKS

NW\_023336268 (2893694-2960350): Colletotrichum scovillei strain TJNH1 chromos... (33% of genes show similarity), T1PKS
Download graphic

Similar known gene clusters from MIBiG 3.1

Shows clusters from the MiBIG database that are similar to the current region. Genes marked with the same colour are interrelated. White genes have no relationship.  
Click on reference genes to show details of similarities to genes within the current region.  
Click on an accession to open that entry in the MiBIG database.

All hits

HEx-pks1 polyketide
Download graphic

Similar subclusters

Shows sub-cluster units that are similar to the current region. Genes marked with the same colour are interrelated. White genes have no relationship.  
Click on reference genes to show details of similarities to genes within the current region.

No matches found.

Detailed Pfam domain annotation

Shows Pfam domains found in each gene within the region. Click on each domain for more information about the domain's accession, location, and description. Domains with a bold border have Gene Ontology information.

Selected features only

Expand to show all names

Detailed TIGRFAM domain annotation

Shows TIGRFAM domains found in each gene within the region. Click on each domain for more information about the domain's accession, location, and description.

Selected features only

Expand to show all names

NRPS/PKS monomers

NRPS/PKS monomer predictions

Shows the predicted monomers for each adynelation domain and acyltransferase within genes. Each gene prediction can be expanded to view detailed predictions of each domain. Each prediction can be expanded to view the predictions by tool (and, for some tools, further expanded for extra details).

**input.path1.gene41**: pk

:   **PKS\_AT (697..1020)**: pk

    ATSignature: Malonyl-CoA

    Top 3 matches:
    :   Malonyl-CoA: 79.2%
    :   Methylmalonyl-CoA: 66.7%
    :   2-Methylbutyryl-CoA: 58.3%

      
    minowa: Methylmalonyl-CoA

    Prediction, score:
    :   Methylmalonyl-CoA: 74.8


        Methoxymalonyl-CoA: 62.4


        Malonyl-CoA: 52.5


        Isobutyryl-CoA: 33.2


        Ethylmalonyl-CoA: 30.5


        2-Methylbutyryl-CoA: 29.1


        Propionyl-CoA: 28.2


        fatty\_acid: 16.8


        Benzoyl-CoA: 16.1


        inactive: 15.5


        3-Methylbutyryl-CoA: 13.7


        trans-1,2-CPDA: 12.9


        CHC-CoA: 12.1


        Acetyl-CoA: -1.3

ENANITX01000353NITX01000353.1 - Region 1 - NRPS

Shows the layout of the region, marking coding sequences and areas of interest. Clicking a gene will select it and show any relevant details. Clicking an area feature (e.g. a candidate cluster) will select all coding sequences within that area. Double clicking an area feature will zoom to that area. Multiple genes and area features can be selected by clicking them while holding the Ctrl key.  
More detailed help is available here.

Download region GenBank file

Download region SVG

Location: 3,059 - 71,736 nt. (total: 68,678 nt)
Show pHMM detection rules used

NRPS: cds(Condensation and (AMP-binding or A-OX))

#### Legend:

core biosynthetic genes

additional biosynthetic genes

transport-related genes

regulatory genes

other genes

resistance

reset view

zoom to selection

Gene details

Shows details of the most recently selected gene, including names, products, location, and other annotations.

Select a gene to view the details available for it

Gene overview

NRPS/PKS domains

MIBiG comparison

ClusterBlast

KnownClusterBlast

SubClusterBlast

Pfam domains

Gene/CDS overview

A brief tabular summary of genes/CDS features within the region.  
Filtering the table will also search biosynthetic profiles and gene function data. If enabled, the overview will then zoom to show the area covered by the filtered selection.  
Genes selected in the region drawing above will be marked in the table with an indicator to the left of the gene name.

Filter:

Automatically zoom to filtered/selected features

| Identifier | Product | Length | | Function | Sequence | | NCBI Blast | Filter details |
| --- | --- | --- | --- | --- | --- | --- | --- | --- |
|  |  | NT | AA |  | NT | AA |  |  |

No genes match the given filter

Detailed domain annotation

Shows NRPS- and PKS-related domains for each feature that contains them. Click on each domain for more information about the domain's location, consensus monomer prediction, and other details.  
A domain glossary is available here, and an explanation of the visualisation is available here.

Selected features only

Show module domains

Similar gene clusters

Shows careas that are similar to the current region to a reference database.  
Mouseover a score cell in the table to get a breakdown of how the score was calculated.The MIBiG database.  
  
Click on an accession to open that entry in the MIBiG database.

Analysis type:

Protocluster to Region
Region to Region

| Reference | NRPS | Similarity score | Type | Compound(s) | Organism |
| --- | --- | --- | --- | --- | --- |
| BGC0001699 |  | 0.18 | NRP | nidulanin A | Aspergillus nidulans FGSC A4 |
| BGC0000357 |  | 0.17 | NRP | cyclo-(D-Phe-L-Phe-D-Val-L-Val), cyclo-(D-Tyr-L-Phe-D-Val-L-Val), cyclo-(D-Tyr-L-Trp-D-Val-L-Val), cyclo-(D-Phe-L-Trp-D-Val-L-Val), cyclo-(D-Phe-L-Phe-D-Val-L-Ile), cyclo-(D-Phe-L-Phe-D-Ile-L-Val), cyclo-(D-Tyr-L-Trp-D-Val-L-Ile), cyclo-(D-Tyr-L-Trp-D-Ile-L-Val), cyclo-(D-Tyr-L-Phe-D-Val-L-Ile), cyclo-(D-Tyr-L-Phe-D-Ile-L-Val) | Penicillium rubens Wisconsin 54-1255 |
| BGC0001240 |  | 0.17 | NRP | serinocyclin A, serinocyclin B | Metarhizium robertsii |
| BGC0001166 |  | 0.17 | NRP | HC-toxin | Alternaria jesenskae |
| BGC0001249 |  | 0.17 | NRP | dimethylcoprogen | Alternaria alternata |
| BGC0001758 |  | 0.17 | NRP | rhizomide A, rhizomide B, rhizomide C | Paraburkholderia rhizoxinica HKI 454 |
| BGC0001811 |  | 0.16 | Terpene | trichodiene-11-one | Fusarium asiaticum |
| BGC0000930 |  | 0.15 | Terpene | deoxynivalenol | Fusarium graminearum |
| BGC0002164 |  | 0.15 | NRP | peramine | Epichloe festucae |
| BGC0002208 |  | 0.15 | NRP | oxepinamide F | Aspergillus ustus |

| Reference | Aggregated | Similarity score | Type | Compound(s) | Organism |
| --- | --- | --- | --- | --- | --- |
| BGC0001699 |  | 0.49 | NRP | nidulanin A | Aspergillus nidulans FGSC A4 |
| BGC0000357 |  | 0.49 | NRP | cyclo-(D-Phe-L-Phe-D-Val-L-Val), cyclo-(D-Tyr-L-Phe-D-Val-L-Val), cyclo-(D-Tyr-L-Trp-D-Val-L-Val), cyclo-(D-Phe-L-Trp-D-Val-L-Val), cyclo-(D-Phe-L-Phe-D-Val-L-Ile), cyclo-(D-Phe-L-Phe-D-Ile-L-Val), cyclo-(D-Tyr-L-Trp-D-Val-L-Ile), cyclo-(D-Tyr-L-Trp-D-Ile-L-Val), cyclo-(D-Tyr-L-Phe-D-Val-L-Ile), cyclo-(D-Tyr-L-Phe-D-Ile-L-Val) | Penicillium rubens Wisconsin 54-1255 |
| BGC0001240 |  | 0.48 | NRP | serinocyclin A, serinocyclin B | Metarhizium robertsii |
| BGC0001166 |  | 0.48 | NRP | HC-toxin | Alternaria jesenskae |
| BGC0001249 |  | 0.48 | NRP | dimethylcoprogen | Alternaria alternata |
| BGC0001758 |  | 0.48 | NRP | rhizomide A, rhizomide B, rhizomide C | Paraburkholderia rhizoxinica HKI 454 |
| BGC0001811 |  | 0.46 | Terpene | trichodiene-11-one | Fusarium asiaticum |
| BGC0000930 |  | 0.46 | Terpene | deoxynivalenol | Fusarium graminearum |
| BGC0002164 |  | 0.46 | NRP | peramine | Epichloe festucae |
| BGC0002208 |  | 0.46 | NRP | oxepinamide F | Aspergillus ustus |

Similar gene clusters

Shows regions from the antiSMASH database that are similar to the current region. Genes marked with the same colour are interrelated. White genes have no relationship.  
Click on reference genes to show details of similarities to genes within the current region.  
Click on an accession to open that entry in the antiSMASH database (if applicable).

All hits

NW\_021167084 (2272829-2321507): Sodiomyces alkalinus F11 unplaced genomic sca... (53% of genes show similarity), NRPS

NW\_006917095 (3620764-3668979): Pestalotiopsis fici W106-1 unplaced genomic s... (33% of genes show similarity), NRPS

NW\_009276965 (48451-96630): Verticillium dahliae VdLs.17 supercont1.33 genomi... (33% of genes show similarity), NRPS

NW\_003315026 (1094542-1142723): Verticillium alfalfae VaMs.102 supercont1.13 ... (37% of genes show similarity), NRPS

NC\_030961 (304923-347797): Colletotrichum higginsianum IMI 349063 chromosome ... (25% of genes show similarity), NRPS

NW\_023336263 (3292078-3340175): Colletotrichum scovillei strain TJNH1 chromos... (20% of genes show similarity), NRPS

CM014983 (3661571-3710214): Pyricularia sp. CBS 133598 strain NI919 chromosom... (15% of genes show similarity), NRPS

CM000595 (588120-635277): Fusarium oxysporum f. sp. lycopersici 4287 chromoso... (16% of genes show similarity), NRPS

NC\_030992 (588120-635277): Fusarium oxysporum f. sp. lycopersici 4287 chromos... (16% of genes show similarity), NRPS

NW\_022194796 (2099127-2146596): Fusarium proliferatum ET1 genome assembly, co... (16% of genes show similarity), NRPS
Download graphic

Similar known gene clusters from MIBiG 3.1

Shows clusters from the MiBIG database that are similar to the current region. Genes marked with the same colour are interrelated. White genes have no relationship.  
Click on reference genes to show details of similarities to genes within the current region.  
Click on an accession to open that entry in the MiBIG database.

No matches found.

Similar subclusters

Shows sub-cluster units that are similar to the current region. Genes marked with the same colour are interrelated. White genes have no relationship.  
Click on reference genes to show details of similarities to genes within the current region.

No matches found.

Detailed Pfam domain annotation

Shows Pfam domains found in each gene within the region. Click on each domain for more information about the domain's accession, location, and description. Domains with a bold border have Gene Ontology information.

Selected features only

Expand to show all names

NRPS/PKS monomers

NRPS/PKS monomer predictions

Shows the predicted monomers for each adynelation domain and acyltransferase within genes. Each gene prediction can be expanded to view detailed predictions of each domain. Each prediction can be expanded to view the predictions by tool (and, for some tools, further expanded for extra details).

**input.path1.gene6**: Ala

:   Search NORINE for peptide:
    strict
    or
    relaxed
  
:   **AMP-binding (1229..1570)**: Ala

    nrpys: Ala

    SVM prediction details:
    :   Predicted physicochemical class:
        :   hydrophobic-aliphatic (Ala, Gly, Val, Leu, Ile, Abu, Ival, Ser, Thr, Hpg, Dhpg, Cys, Pro, Hpr)

        Large clusters prediction:
        :   Apolar, aliphatic (Gly, Ala, Val, Leu, Ile, Abu, Ival)

        Small clusters prediction:
        :   Tiny, hydrophilic, transition to aliphatic (Gly, Ala)

        Single AA prediction:
        :   ala (Ala)

    Stachelhaus prediction details:
    :   Stachelhaus sequence:
        :   DIFYCRAPLK

        Nearest Stachelhaus code(s):
        :   Ala DLFFCGGPLK (44% 8Å match)
        :   bAla DIFYAIATAK (38% 8Å match)

        Stachelhaus code match:
        :   60% (weak)

ENANITX01000394NITX01000394.1 - Region 1 - NRPS-like

Shows the layout of the region, marking coding sequences and areas of interest. Clicking a gene will select it and show any relevant details. Clicking an area feature (e.g. a candidate cluster) will select all coding sequences within that area. Double clicking an area feature will zoom to that area. Multiple genes and area features can be selected by clicking them while holding the Ctrl key.  
More detailed help is available here.

Download region GenBank file

Download region SVG

Location: 142,565 - 203,866 nt. (total: 61,302 nt)
Show pHMM detection rules used

Region on contig edge.

NRPS-like: cds((PP-binding or NAD\_binding\_4) and (AMP-binding or A-OX))

#### Legend:

core biosynthetic genes

additional biosynthetic genes

transport-related genes

regulatory genes

other genes

resistance

reset view

zoom to selection

Gene details

Shows details of the most recently selected gene, including names, products, location, and other annotations.

Select a gene to view the details available for it

Gene overview

NRPS/PKS domains

MIBiG comparison

ClusterBlast

KnownClusterBlast

SubClusterBlast

NRPS/PKS modules

Pfam domains

TIGRFAM domains

Gene/CDS overview

A brief tabular summary of genes/CDS features within the region.  
Filtering the table will also search biosynthetic profiles and gene function data. If enabled, the overview will then zoom to show the area covered by the filtered selection.  
Genes selected in the region drawing above will be marked in the table with an indicator to the left of the gene name.

Filter:

Automatically zoom to filtered/selected features

| Identifier | Product | Length | | Function | Sequence | | NCBI Blast | Filter details |
| --- | --- | --- | --- | --- | --- | --- | --- | --- |
|  |  | NT | AA |  | NT | AA |  |  |

No genes match the given filter

Detailed domain annotation

Shows NRPS- and PKS-related domains for each feature that contains them. Click on each domain for more information about the domain's location, consensus monomer prediction, and other details.  
A domain glossary is available here, and an explanation of the visualisation is available here.

Selected features only

Show module domains

Similar gene clusters

Shows careas that are similar to the current region to a reference database.  
Mouseover a score cell in the table to get a breakdown of how the score was calculated.The MIBiG database.  
  
Click on an accession to open that entry in the MIBiG database.

Analysis type:

Protocluster to Region
Region to Region

| Reference | NRPS-like | NRPS-like | Similarity score | Type | Compound(s) | Organism |
| --- | --- | --- | --- | --- | --- | --- |
| BGC0002276 |  |  | 0.72 | NRP | choline | Aspergillus nidulans FGSC A4 |
| BGC0000465 |  |  | 0.43 | NRP | xenortide A, xenortide B, xenortide C, xenortide D | Xenorhabdus nematophila ATCC 19061 |
| BGC0000426 |  |  | 0.42 | NRP | sevadicin | Paenibacillus larvae |
| BGC0001133 |  |  | 0.42 | NRP | taxlllaid A | Xenorhabdus bovienii SS-2004 |
| BGC0001168 |  |  | 0.42 | NRP | livipeptin | Streptomyces lividans 1326 |
| BGC0002071 |  |  | 0.41 | NRP | virginiafactin A, virginiafactin B, virginiafactin C, virginiafactin D | Pseudomonas sp. QS1027 |
| BGC0000893 |  |  | 0.41 | NRP | chloramphenicol | Streptomyces venezuelae ATCC 10712 |
| BGC0002167 |  |  | 0.38 | NRP | actinopolymorphol C, morpholine containing hemiacetal piperazine compound, piperazine compound 2, piperazine compound 1, 3-(p-hydroxyphenyl)-1,2-propanediol, N,N-dioxide containing derivate, O-sulfonated actinopolymorphol C, C-3 sulfonylated derivative | Aspergillus flavus NRRL3357 |
| BGC0002166 |  |  | 0.36 | NRP | peramine, intermediate 1, intermediate 2 | Metarhizium rileyi |
| BGC0001844 |  |  | 0.36 | NRP | holrhizin | Paraburkholderia rhizoxinica HKI 454 |

| Reference | Aggregated | Similarity score | Type | Compound(s) | Organism |
| --- | --- | --- | --- | --- | --- |
| BGC0002276 |  | 0.69 | NRP | choline | Aspergillus nidulans FGSC A4 |
| BGC0000465 |  | 0.54 | NRP | xenortide A, xenortide B, xenortide C, xenortide D | Xenorhabdus nematophila ATCC 19061 |
| BGC0000426 |  | 0.54 | NRP | sevadicin | Paenibacillus larvae |
| BGC0001133 |  | 0.54 | NRP | taxlllaid A | Xenorhabdus bovienii SS-2004 |
| BGC0001168 |  | 0.54 | NRP | livipeptin | Streptomyces lividans 1326 |
| BGC0002071 |  | 0.53 | NRP | virginiafactin A, virginiafactin B, virginiafactin C, virginiafactin D | Pseudomonas sp. QS1027 |
| BGC0000893 |  | 0.53 | NRP | chloramphenicol | Streptomyces venezuelae ATCC 10712 |
| BGC0002167 |  | 0.51 | NRP | actinopolymorphol C, morpholine containing hemiacetal piperazine compound, piperazine compound 2, piperazine compound 1, 3-(p-hydroxyphenyl)-1,2-propanediol, N,N-dioxide containing derivate, O-sulfonated actinopolymorphol C, C-3 sulfonylated derivative | Aspergillus flavus NRRL3357 |
| BGC0000351 |  | 0.50 | NRP | exochelin | Mycolicibacterium smegmatis MC2 155 |
| BGC0002166 |  | 0.50 | NRP | peramine, intermediate 1, intermediate 2 | Metarhizium rileyi |

Similar gene clusters

Shows regions from the antiSMASH database that are similar to the current region. Genes marked with the same colour are interrelated. White genes have no relationship.  
Click on reference genes to show details of similarities to genes within the current region.  
Click on an accession to open that entry in the antiSMASH database (if applicable).

All hits

NW\_021167086 (151848-211942): Sodiomyces alkalinus F11 unplaced genomic scaff... (57% of genes show similarity), NRPS-like

NC\_019695 (2077572-2120425): Chroococcidiopsis thermalis PCC 7203, complete s... (6% of genes show similarity), NRPS-like

NZ\_JACHOW010000014 (25963-134244): Longimicrobium terrae strain CECT 8660 Ga0... (10% of genes show similarity), NRPS,T1PKS

NZ\_FNOG01000006 (25918-84197): Lysobacter enzymogenes strain ATCC 29487, whol... (7% of genes show similarity), NRPS,bacteriocin

NZ\_FNPT01000001 (409958-467709): Lysobacter sp. yr284, whole genome shotgun s... (7% of genes show similarity), NRPS,bacteriocin

NZ\_KB913032 (7146893-7280545): Amycolatopsis alba DSM 44262 scaffold1, whole ... (4% of genes show similarity), NRPS,blactam

NZ\_CP011509 (4941731-5061842): Archangium gephyra strain DSM 2261 chromosome,... (5% of genes show similarity), NRPS,T1PKS

NC\_010162 (11466108-11549102): Sorangium cellulosum So ce56, complete genome (8% of genes show similarity), NRPS,T1PKS

NZ\_FPJB01000001 (348284-438096): Pseudomonas sp. NFACC10-1, whole genome shot... (8% of genes show similarity), NRPS

NZ\_LT222319 (3655590-3742143): Pseudomonas cerasi isolate Sour cherry (Prunus... (8% of genes show similarity), NRPS
Download graphic

Similar known gene clusters from MIBiG 3.1

Shows clusters from the MiBIG database that are similar to the current region. Genes marked with the same colour are interrelated. White genes have no relationship.  
Click on reference genes to show details of similarities to genes within the current region.  
Click on an accession to open that entry in the MiBIG database.

All hits

choline
Download graphic

Similar subclusters

Shows sub-cluster units that are similar to the current region. Genes marked with the same colour are interrelated. White genes have no relationship.  
Click on reference genes to show details of similarities to genes within the current region.

No matches found.

Module view

Shows module structures for each candidate cluster in NRPS and PKS regions.   
Genes are shown in predicted order, and are only present when containing at least one complete module.   
A domain glossary is available here, and an explanation of the visualisation is available here.

Candidate 1 (142564 - 203866): neighbouring NRPS-like

Candidate 2 (142564 - 202187): single NRPS-like

Candidate 3 (157886 - 203866): single NRPS-like

Legend

Detailed Pfam domain annotation

Shows Pfam domains found in each gene within the region. Click on each domain for more information about the domain's accession, location, and description. Domains with a bold border have Gene Ontology information.

Selected features only

Expand to show all names

Detailed TIGRFAM domain annotation

Shows TIGRFAM domains found in each gene within the region. Click on each domain for more information about the domain's accession, location, and description.

Selected features only

Expand to show all names

NRPS/PKS products

NRPS/PKS monomers

Predicted core structure(s)

Shows estimated product structure and polymer for each candidate cluster in the region. To show the product, click on the expander or the candidate cluster feature drawn in the overview.

For candidate cluster 1, location 142564 - 203866:

Rough prediction of core scaffold based on assumed PKS/NRPS colinearity; tailoring reactions not taken into account

**Polymer prediction:**
:   (Aad) + (X)

  
Direct lookup in NORINE database:
strict
or
relaxed

---

For candidate cluster 2, location 142564 - 202187:

Rough prediction of core scaffold based on assumed PKS/NRPS colinearity; tailoring reactions not taken into account

**Polymer prediction:**
:   (Aad) + (X)

  
Direct lookup in NORINE database:
strict
or
relaxed

---

For candidate cluster 3, location 157886 - 203866:

Rough prediction of core scaffold based on assumed PKS/NRPS colinearity; tailoring reactions not taken into account

**Polymer prediction:**
:   (Aad) + (X)

  
Direct lookup in NORINE database:
strict
or
relaxed

Link to NORINE database query form

NRPS/PKS monomer predictions

Shows the predicted monomers for each adynelation domain and acyltransferase within genes. Each gene prediction can be expanded to view detailed predictions of each domain. Each prediction can be expanded to view the predictions by tool (and, for some tools, further expanded for extra details).

**input.path1.gene36**: X

:   **AMP-binding (13..416)**: X

    nrpys: Ala, Gly, Val, Leu, Ile, Abu, Ival, Ser, Thr, Hpg, Dhpg, Cys, Pro, Hpr

    SVM prediction details:
    :   Predicted physicochemical class:
        :   hydrophobic-aliphatic (Ala, Gly, Val, Leu, Ile, Abu, Ival, Ser, Thr, Hpg, Dhpg, Cys, Pro, Hpr)

        Large clusters prediction:
        :   N/A

        Small clusters prediction:
        :   N/A

        Single AA prediction:
        :   N/A

    Stachelhaus prediction details:
    :   Stachelhaus sequence:
        :   --WLWNLDVK

        Nearest Stachelhaus code(s):

        Stachelhaus code match:
        :   0% (weak)

  
**input.path1.gene41**: Aad

:   Search NORINE for peptide:
    strict
    or
    relaxed
  
:   **AMP-binding (24..512)**: Aad

    nrpys: Aad

    SVM prediction details:
    :   Predicted physicochemical class:
        :   hydrophilic (Arg, Asp, Glu, Asn, Lys, Gln, Orn, Aad)

        Large clusters prediction:
        :   Aliphatic chain with H-bond donor (Asp, Asn, Glu, Gln, Aad)

        Small clusters prediction:
        :   N/A

        Single AA prediction:
        :   N/A

    Stachelhaus prediction details:
    :   Stachelhaus sequence:
        :   DPRHFVMRSK

        Nearest Stachelhaus code(s):
        :   Aad DPRHFVMRSK (97% 8Å match)

        Stachelhaus code match:
        :   100% (strong)

ENANITX01000454NITX01000454.1 - Region 1 - NRPS,T1PKS

Shows the layout of the region, marking coding sequences and areas of interest. Clicking a gene will select it and show any relevant details. Clicking an area feature (e.g. a candidate cluster) will select all coding sequences within that area. Double clicking an area feature will zoom to that area. Multiple genes and area features can be selected by clicking them while holding the Ctrl key.  
More detailed help is available here.

Download region GenBank file

Download region SVG

Location: 40,840 - 114,672 nt. (total: 73,833 nt)
Show pHMM detection rules used

NRPS: cds(Condensation and (AMP-binding or A-OX))  
T1PKS: cds(PKS\_AT and (PKS\_KS or ene\_KS or mod\_KS or hyb\_KS or itr\_KS or tra\_KS))

#### Legend:

core biosynthetic genes

additional biosynthetic genes

transport-related genes

regulatory genes

other genes

resistance

reset view

zoom to selection

Gene details

Shows details of the most recently selected gene, including names, products, location, and other annotations.

Select a gene to view the details available for it

Gene overview

NRPS/PKS domains

MIBiG comparison

ClusterBlast

KnownClusterBlast

SubClusterBlast

NRPS/PKS modules

Pfam domains

Gene/CDS overview

A brief tabular summary of genes/CDS features within the region.  
Filtering the table will also search biosynthetic profiles and gene function data. If enabled, the overview will then zoom to show the area covered by the filtered selection.  
Genes selected in the region drawing above will be marked in the table with an indicator to the left of the gene name.

Filter:

Automatically zoom to filtered/selected features

| Identifier | Product | Length | | Function | Sequence | | NCBI Blast | Filter details |
| --- | --- | --- | --- | --- | --- | --- | --- | --- |
|  |  | NT | AA |  | NT | AA |  |  |

No genes match the given filter

Detailed domain annotation

Shows NRPS- and PKS-related domains for each feature that contains them. Click on each domain for more information about the domain's location, consensus monomer prediction, and other details.  
A domain glossary is available here, and an explanation of the visualisation is available here.

Selected features only

Show module domains

Similar gene clusters

Shows careas that are similar to the current region to a reference database.  
Mouseover a score cell in the table to get a breakdown of how the score was calculated.The MIBiG database.  
  
Click on an accession to open that entry in the MIBiG database.

Analysis type:

Protocluster to Region
Region to Region

| Reference | NRPS | T1PKS | Similarity score | Type | Compound(s) | Organism |
| --- | --- | --- | --- | --- | --- | --- |
| BGC0001400 |  |  | 0.68 | Polyketide | citreoviridin | Aspergillus terreus NIH2624 |
| BGC0001998 |  |  | 0.67 | Polyketide | aspernidgulene A1, aspernidgulene A2, aspernidgulene B1 | Aspergillus nidulans FGSC A4 |
| BGC0001268 |  |  | 0.64 | NRP, Polyketide | fusarin C | Fusarium fujikuroi |
| BGC0000064 |  |  | 0.64 | NRP, Polyketide | fusarin | Fusarium verticillioides |
| BGC0001254 |  |  | 0.63 | Polyketide | ACT-Toxin II | Alternaria alternata |
| BGC0002192 |  |  | 0.62 | Polyketide | FR901512 | fungal sp. No.14919 |
| BGC0001218 |  |  | 0.61 | NRP, Polyketide | fumosorinone | Cordyceps fumosorosea |
| BGC0002258 |  |  | 0.61 | Polyketide | shimalactone A, shimalactone B | Aspergillus stellatus |
| BGC0002230 |  |  | 0.60 | Polyketide, NRP | tolypyridone C | Tolypocladium sp. 49Y |
| BGC0001136 |  |  | 0.59 | NRP, Polyketide | desmethylbassianin | Beauveria bassiana |

| Reference | Aggregated | Similarity score | Type | Compound(s) | Organism |
| --- | --- | --- | --- | --- | --- |
| BGC0001400 |  | 0.67 | Polyketide | citreoviridin | Aspergillus terreus NIH2624 |
| BGC0001998 |  | 0.67 | Polyketide | aspernidgulene A1, aspernidgulene A2, aspernidgulene B1 | Aspergillus nidulans FGSC A4 |
| BGC0001268 |  | 0.65 | NRP, Polyketide | fusarin C | Fusarium fujikuroi |
| BGC0000064 |  | 0.65 | NRP, Polyketide | fusarin | Fusarium verticillioides |
| BGC0001254 |  | 0.65 | Polyketide | ACT-Toxin II | Alternaria alternata |
| BGC0002192 |  | 0.65 | Polyketide | FR901512 | fungal sp. No.14919 |
| BGC0001218 |  | 0.64 | NRP, Polyketide | fumosorinone | Cordyceps fumosorosea |
| BGC0002258 |  | 0.64 | Polyketide | shimalactone A, shimalactone B | Aspergillus stellatus |
| BGC0002230 |  | 0.64 | Polyketide, NRP | tolypyridone C | Tolypocladium sp. 49Y |
| BGC0001136 |  | 0.63 | NRP, Polyketide | desmethylbassianin | Beauveria bassiana |

Similar gene clusters

Shows regions from the antiSMASH database that are similar to the current region. Genes marked with the same colour are interrelated. White genes have no relationship.  
Click on reference genes to show details of similarities to genes within the current region.  
Click on an accession to open that entry in the antiSMASH database (if applicable).

All hits

NW\_021167087 (1196598-1249641): Sodiomyces alkalinus F11 unplaced genomic sca... (68% of genes show similarity), NRPS,T1PKS

NC\_030955 (5505539-5558376): Colletotrichum higginsianum IMI 349063 chromosom... (20% of genes show similarity), NRPS,T1PKS

NT\_165976 (3817736-3873747): Chaetomium globosum CBS 148.51 scaffold 1 genomi... (21% of genes show similarity), NRPS,T1PKS

NW\_023336259 (4272523-4375263): Colletotrichum scovillei strain TJNH1 chromos... (10% of genes show similarity), NRPS,T1PKS

NW\_023336267 (5170209-5222154): Colletotrichum scovillei strain TJNH1 chromos... (15% of genes show similarity), NRPS,T1PKS

NW\_009276923 (402667-455430): Verticillium dahliae VdLs.17 supercont1.18 geno... (18% of genes show similarity), NRPS,T1PKS

NW\_003345199 (1572647-1624975): Nannizzia gypsea CBS 118893 supercont1.3 geno... (10% of genes show similarity), NRPS,T1PKS

CM002798 (1208-46571): Penicillium chrysogenum strain P2niaD18 chromosome I, ... (13% of genes show similarity), NRPS,T1PKS

NW\_003299167 (489-39896): Microsporum canis CBS 113480 supercont1.3 genomic s... (11% of genes show similarity), NRPS,T1PKS

NW\_022474207 (2348451-2400505): Venustampulla echinocandica strain BP 5553 ch... (13% of genes show similarity), NRPS,T1PKS
Download graphic

Similar known gene clusters from MIBiG 3.1

Shows clusters from the MiBIG database that are similar to the current region. Genes marked with the same colour are interrelated. White genes have no relationship.  
Click on reference genes to show details of similarities to genes within the current region.  
Click on an accession to open that entry in the MiBIG database.

No matches found.

Similar subclusters

Shows sub-cluster units that are similar to the current region. Genes marked with the same colour are interrelated. White genes have no relationship.  
Click on reference genes to show details of similarities to genes within the current region.

No matches found.

Module view

Shows module structures for each candidate cluster in NRPS and PKS regions.   
Genes are shown in predicted order, and are only present when containing at least one complete module.   
A domain glossary is available here, and an explanation of the visualisation is available here.

Candidate 1 (40839 - 114672): chemical hybrid NRPS-T1PKS

Legend

Detailed Pfam domain annotation

Shows Pfam domains found in each gene within the region. Click on each domain for more information about the domain's accession, location, and description. Domains with a bold border have Gene Ontology information.

Selected features only

Expand to show all names

NRPS/PKS products

NRPS/PKS monomers

Predicted core structure(s)

Shows estimated product structure and polymer for each candidate cluster in the region. To show the product, click on the expander or the candidate cluster feature drawn in the overview.

For candidate cluster 1, location 40839 - 114672:

Rough prediction of core scaffold based on assumed PKS/NRPS colinearity; tailoring reactions not taken into account

**Polymer prediction:**
:   (pk - X)

  
Direct lookup in NORINE database:
strict
or
relaxed

Link to NORINE database query form

NRPS/PKS monomer predictions

Shows the predicted monomers for each adynelation domain and acyltransferase within genes. Each gene prediction can be expanded to view detailed predictions of each domain. Each prediction can be expanded to view the predictions by tool (and, for some tools, further expanded for extra details).

**input.path1.gene20**: pk - X

:   **PKS\_AT (678..999)**: pk

    ATSignature: Malonyl-CoA

    Top 3 matches:
    :   Malonyl-CoA: 75.0%
    :   Methylmalonyl-CoA: 66.7%
    :   2-Methylbutyryl-CoA: 62.5%

      
    minowa: Methylmalonyl-CoA

    Prediction, score:
    :   Methylmalonyl-CoA: 91.3


        Methoxymalonyl-CoA: 74.9


        Ethylmalonyl-CoA: 69.4


        Malonyl-CoA: 55.2


        Propionyl-CoA: 35.8


        Isobutyryl-CoA: 30.6


        Benzoyl-CoA: 30.5


        fatty\_acid: 25.6


        trans-1,2-CPDA: 23.3


        Acetyl-CoA: 17.3


        CHC-CoA: 16.5


        2-Methylbutyryl-CoA: 11.7


        inactive: 9.7


        3-Methylbutyryl-CoA: 8.0
:   **AMP-binding (3294..3715)**: X

    nrpys: Arg, Asp, Glu, Asn, Lys, Gln, Orn, Aad

    SVM prediction details:
    :   Predicted physicochemical class:
        :   hydrophilic (Arg, Asp, Glu, Asn, Lys, Gln, Orn, Aad)

        Large clusters prediction:
        :   N/A

        Small clusters prediction:
        :   N/A

        Single AA prediction:
        :   N/A

    Stachelhaus prediction details:
    :   Stachelhaus sequence:
        :   DMDCHGALGK

        Nearest Stachelhaus code(s):
        :   Tyr DMNSTGALSK (56% 8Å match)

        Stachelhaus code match:
        :   60% (weak)

ENANITX01000499NITX01000499.1 - Region 1 - NRPS

Shows the layout of the region, marking coding sequences and areas of interest. Clicking a gene will select it and show any relevant details. Clicking an area feature (e.g. a candidate cluster) will select all coding sequences within that area. Double clicking an area feature will zoom to that area. Multiple genes and area features can be selected by clicking them while holding the Ctrl key.  
More detailed help is available here.

Download region GenBank file

Download region SVG

Location: 5,178 - 77,072 nt. (total: 71,895 nt)
Show pHMM detection rules used

NRPS: cds(Condensation and (AMP-binding or A-OX))

#### Legend:

core biosynthetic genes

additional biosynthetic genes

transport-related genes

regulatory genes

other genes

resistance

reset view

zoom to selection

Gene details

Shows details of the most recently selected gene, including names, products, location, and other annotations.

Select a gene to view the details available for it

Gene overview

NRPS/PKS domains

MIBiG comparison

ClusterBlast

KnownClusterBlast

SubClusterBlast

NRPS/PKS modules

Pfam domains

TIGRFAM domains

Gene/CDS overview

A brief tabular summary of genes/CDS features within the region.  
Filtering the table will also search biosynthetic profiles and gene function data. If enabled, the overview will then zoom to show the area covered by the filtered selection.  
Genes selected in the region drawing above will be marked in the table with an indicator to the left of the gene name.

Filter:

Automatically zoom to filtered/selected features

| Identifier | Product | Length | | Function | Sequence | | NCBI Blast | Filter details |
| --- | --- | --- | --- | --- | --- | --- | --- | --- |
|  |  | NT | AA |  | NT | AA |  |  |

No genes match the given filter

Detailed domain annotation

Shows NRPS- and PKS-related domains for each feature that contains them. Click on each domain for more information about the domain's location, consensus monomer prediction, and other details.  
A domain glossary is available here, and an explanation of the visualisation is available here.

Selected features only

Show module domains

Similar gene clusters

Shows careas that are similar to the current region to a reference database.  
Mouseover a score cell in the table to get a breakdown of how the score was calculated.The MIBiG database.  
  
Click on an accession to open that entry in the MIBiG database.

Analysis type:

Protocluster to Region
Region to Region

| Reference | NRPS | Similarity score | Type | Compound(s) | Organism |
| --- | --- | --- | --- | --- | --- |
| BGC0001825 |  | 0.27 | NRP | xenematide | Xenorhabdus nematophila AN6/1 |
| BGC0001132 |  | 0.27 | NRP | xenotetrapeptide | Xenorhabdus nematophila ATCC 19061 |
| BGC0000404 |  | 0.26 | NRP | penicillin | Penicillium chrysogenum |
| BGC0000313 |  | 0.26 | NRP | beauvericin | Beauveria bassiana |
| BGC0001824 |  | 0.25 | NRP | xefoampeptides A-G | Xenorhabdus beddingii |
| BGC0001135 |  | 0.25 | NRP | bicornutin A1, bicornutin A2 | Xenorhabdus budapestensis |
| BGC0000342 |  | 0.25 | NRP | enniatin | Fusarium equiseti |
| BGC0001128 |  | 0.25 | NRP | gamexpeptide C | Photorhabdus laumondii subsp. laumondii TTO1 |
| BGC0002286 |  | 0.25 | NRP | ririwpeptide A, ririwpeptide B, ririwpeptide C | Photorhabdus laumondii subsp. laumondii TTO1 |
| BGC0001561 |  | 0.25 | NRP | curacomycin | Streptomyces curacoi |

| Reference | Aggregated | Similarity score | Type | Compound(s) | Organism |
| --- | --- | --- | --- | --- | --- |
| BGC0001825 |  | 0.60 | NRP | xenematide | Xenorhabdus nematophila AN6/1 |
| BGC0001132 |  | 0.60 | NRP | xenotetrapeptide | Xenorhabdus nematophila ATCC 19061 |
| BGC0000404 |  | 0.59 | NRP | penicillin | Penicillium chrysogenum |
| BGC0000313 |  | 0.59 | NRP | beauvericin | Beauveria bassiana |
| BGC0001824 |  | 0.59 | NRP | xefoampeptides A-G | Xenorhabdus beddingii |
| BGC0001135 |  | 0.59 | NRP | bicornutin A1, bicornutin A2 | Xenorhabdus budapestensis |
| BGC0000342 |  | 0.59 | NRP | enniatin | Fusarium equiseti |
| BGC0001128 |  | 0.58 | NRP | gamexpeptide C | Photorhabdus laumondii subsp. laumondii TTO1 |
| BGC0002286 |  | 0.58 | NRP | ririwpeptide A, ririwpeptide B, ririwpeptide C | Photorhabdus laumondii subsp. laumondii TTO1 |
| BGC0001561 |  | 0.58 | NRP | curacomycin | Streptomyces curacoi |

Similar gene clusters

Shows regions from the antiSMASH database that are similar to the current region. Genes marked with the same colour are interrelated. White genes have no relationship.  
Click on reference genes to show details of similarities to genes within the current region.  
Click on an accession to open that entry in the antiSMASH database (if applicable).

All hits

NW\_021167089 (8954-74367): Sodiomyces alkalinus F11 unplaced genomic scaffold... (77% of genes show similarity), NRPS

NC\_035793 (4210482-4273534): Pochonia chlamydosporia 170 chromosome 4, whole ... (21% of genes show similarity), NRPS

NC\_036440 (2147708-2212689): Aspergillus oryzae RIB40 DNA, chromosome 6 (11% of genes show similarity), NRPS

CM002799 (9738816-9788231): Penicillium chrysogenum strain P2niaD18 chromosom... (15% of genes show similarity), NRPS

CM002799 (9842192-9893523): Penicillium chrysogenum strain P2niaD18 chromosom... (15% of genes show similarity), NRPS

NZ\_JAASQX010000001 (1652597-1726507): Xanthomonas sp. F4 Ga0372548 01, whole ... (8% of genes show similarity), NRPS,T1PKS

NT\_107013 (13261-64574): Aspergillus nidulans FGSC A4 chromosome VI map unloc... (20% of genes show similarity), NRPS

NW\_003315089 (439011-490372): Trichophyton benhamiae CBS 112371 chromosome Un... (12% of genes show similarity), NRPS

NW\_003456417 (380872-432230): Trichophyton rubrum CBS 118892 genomic scaffold... (9% of genes show similarity), NRPS

NZ\_CP015163 (627391-677232): Amycolatopsis albispora strain WP1 chromosome, c... (4% of genes show similarity), NRPS
Download graphic

Similar known gene clusters from MIBiG 3.1

Shows clusters from the MiBIG database that are similar to the current region. Genes marked with the same colour are interrelated. White genes have no relationship.  
Click on reference genes to show details of similarities to genes within the current region.  
Click on an accession to open that entry in the MiBIG database.

All hits

penicillin

δ-(L-α-aminoadipyl)-L-cysteine-D-valine/isopenicillin N/benzylpenicillin/phenoxymethylpenicillin
Download graphic

Similar subclusters

Shows sub-cluster units that are similar to the current region. Genes marked with the same colour are interrelated. White genes have no relationship.  
Click on reference genes to show details of similarities to genes within the current region.

No matches found.

Module view

Shows module structures for each candidate cluster in NRPS and PKS regions.   
Genes are shown in predicted order, and are only present when containing at least one complete module.   
A domain glossary is available here, and an explanation of the visualisation is available here.

Candidate 1 (5177 - 77072): single NRPS

Legend

Detailed Pfam domain annotation

Shows Pfam domains found in each gene within the region. Click on each domain for more information about the domain's accession, location, and description. Domains with a bold border have Gene Ontology information.

Selected features only

Expand to show all names

Detailed TIGRFAM domain annotation

Shows TIGRFAM domains found in each gene within the region. Click on each domain for more information about the domain's accession, location, and description.

Selected features only

Expand to show all names

NRPS/PKS products

NRPS/PKS monomers

Predicted core structure(s)

Shows estimated product structure and polymer for each candidate cluster in the region. To show the product, click on the expander or the candidate cluster feature drawn in the overview.

For candidate cluster 1, location 5177 - 77072:

Rough prediction of core scaffold based on assumed PKS/NRPS colinearity; tailoring reactions not taken into account

**Polymer prediction:**
:   (Aad - Cys - D-Val)

  
Direct lookup in NORINE database:
strict
or
relaxed

Link to NORINE database query form

NRPS/PKS monomer predictions

Shows the predicted monomers for each adynelation domain and acyltransferase within genes. Each gene prediction can be expanded to view detailed predictions of each domain. Each prediction can be expanded to view the predictions by tool (and, for some tools, further expanded for extra details).

**input.path1.gene8**: X

:   **AMP-binding (47..397)**: X

    nrpys: (unknown)

    SVM prediction details:
    :   **NOTE: uncertain match**  

        Predicted physicochemical class:
        :   hydrophobic-aromatic (Phe, Tyr, diOH-Bz, Ph-Gly, bOH-Tyr)

        Large clusters prediction:
        :   N/A

        Small clusters prediction:
        :   N/A

        Single AA prediction:
        :   N/A

    Stachelhaus prediction details:
    :   Stachelhaus sequence:
        :   ----W-FLTK

        Nearest Stachelhaus code(s):

        Stachelhaus code match:
        :   0% (weak)

  
**input.path1.gene11**: Aad - Cys - Val

:   Search NORINE for peptide:
    strict
    or
    relaxed
  
:   **AMP-binding (348..773)**: Aad

    nrpys: Aad

    SVM prediction details:
    :   Predicted physicochemical class:
        :   hydrophilic (Arg, Asp, Glu, Asn, Lys, Gln, Orn, Aad)

        Large clusters prediction:
        :   Aliphatic chain with H-bond donor (Asp, Asn, Glu, Gln, Aad)

        Small clusters prediction:
        :   aad (Aad)

        Single AA prediction:
        :   aad (Aad)

    Stachelhaus prediction details:
    :   Stachelhaus sequence:
        :   EPRNIVEFVK

        Nearest Stachelhaus code(s):
        :   Aad EPRNIVEFVK (97% 8Å match)

        Stachelhaus code match:
        :   100% (strong)
:   **AMP-binding (1497..1911)**: Cys

    nrpys: Cys

    SVM prediction details:
    :   Predicted physicochemical class:
        :   hydrophobic-aliphatic (Ala, Gly, Val, Leu, Ile, Abu, Ival, Ser, Thr, Hpg, Dhpg, Cys, Pro, Hpr)

        Large clusters prediction:
        :   Polar, uncharged (aliphatic with -SH) (Cys)

        Small clusters prediction:
        :   Polar, uncharged (aliphatic with -SH) (Cys)

        Single AA prediction:
        :   Polar, uncharged (aliphatic with -SH) (Cys)

    Stachelhaus prediction details:
    :   Stachelhaus sequence:
        :   DHESDVGITK

        Nearest Stachelhaus code(s):
        :   Cys DHESDVGITK (100% 8Å match)

        Stachelhaus code match:
        :   100% (strong)
:   **AMP-binding (2612..3028)**: Val

    nrpys: Val

    SVM prediction details:
    :   Predicted physicochemical class:
        :   hydrophobic-aliphatic (Ala, Gly, Val, Leu, Ile, Abu, Ival, Ser, Thr, Hpg, Dhpg, Cys, Pro, Hpr)

        Large clusters prediction:
        :   Apolar, aliphatic (Gly, Ala, Val, Leu, Ile, Abu, Ival)

        Small clusters prediction:
        :   Aliphatic, branched hydrophobic (Val, Leu, Ile, Abu, Ival)

        Single AA prediction:
        :   val (Val)

    Stachelhaus prediction details:
    :   Stachelhaus sequence:
        :   DFESTAAVYK

        Nearest Stachelhaus code(s):
        :   Val DFESTAAVYK (85% 8Å match)

        Stachelhaus code match:
        :   100% (strong)

ENANITX01000501NITX01000501.1 - Region 1 - terpene

Shows the layout of the region, marking coding sequences and areas of interest. Clicking a gene will select it and show any relevant details. Clicking an area feature (e.g. a candidate cluster) will select all coding sequences within that area. Double clicking an area feature will zoom to that area. Multiple genes and area features can be selected by clicking them while holding the Ctrl key.  
More detailed help is available here.

Download region GenBank file

Download region SVG

Location: 1 - 17,212 nt. (total: 17,212 nt)
Show pHMM detection rules used

Region on contig edge.

terpene: (Terpene\_synth or Terpene\_synth\_C or phytoene\_synt or Lycopene\_cycl or terpene\_cyclase or NapT7 or fung\_ggpps or fung\_ggpps2 or trichodiene\_synth or TRI5)

#### Legend:

core biosynthetic genes

additional biosynthetic genes

transport-related genes

regulatory genes

other genes

resistance

reset view

zoom to selection

Gene details

Shows details of the most recently selected gene, including names, products, location, and other annotations.

Select a gene to view the details available for it

Gene overview

MIBiG comparison

ClusterBlast

KnownClusterBlast

SubClusterBlast

Pfam domains

TIGRFAM domains

Gene/CDS overview

A brief tabular summary of genes/CDS features within the region.  
Filtering the table will also search biosynthetic profiles and gene function data. If enabled, the overview will then zoom to show the area covered by the filtered selection.  
Genes selected in the region drawing above will be marked in the table with an indicator to the left of the gene name.

Filter:

Automatically zoom to filtered/selected features

| Identifier | Product | Length | | Function | Sequence | | NCBI Blast | Filter details |
| --- | --- | --- | --- | --- | --- | --- | --- | --- |
|  |  | NT | AA |  | NT | AA |  |  |

No genes match the given filter

Similar gene clusters

Shows careas that are similar to the current region to a reference database.  
Mouseover a score cell in the table to get a breakdown of how the score was calculated.The MIBiG database.  
  
Click on an accession to open that entry in the MIBiG database.

Analysis type:

Protocluster to Region
Region to Region

| Reference | terpene | Similarity score | Type | Compound(s) | Organism |
| --- | --- | --- | --- | --- | --- |
| BGC0000673 |  | 0.18 | Terpene | pimara-8(14),15-diene | Aspergillus nidulans FGSC A4 |
| BGC0002320 |  | 0.17 | Terpene | conidiogenone | Penicillium rubens Wisconsin 54-1255 |
| BGC0000688 |  | 0.16 | Terpene | copalyl diphosphate | Diaporthe amygdali |
| BGC0000676 |  | 0.16 | Terpene | aphidicolin, aphidicolan-16β-ol, 3-deoxyaphidicolin, 17-deoxyaphidicolin | Phoma betae |
| BGC0002427 |  | 0.12 | Terpene, Polyketide | subglutinol A, subglutinol B | Metarhizium robertsii ARSEF 23 |
| BGC0001969 |  | 0.12 | Terpene | asperterpenoid A | Talaromyces wortmannii |
| BGC0001082 |  | 0.11 | Terpene | paxilline, paspaline, 13-dehydroxypaxilline, paspaline B | Penicillium paxilli |
| BGC0002604 |  | 0.10 | Polyketide | sartorypyrone A | Aspergillus felis |
| BGC0002610 |  | 0.10 | Terpene | stellatic acid | Aspergillus stellatus |
| BGC0002736 |  | 0.09 | Terpene | preaspterpenacid I | Aspergillus terreus NIH2624 |

| Reference | Aggregated | Similarity score | Type | Compound(s) | Organism |
| --- | --- | --- | --- | --- | --- |
| BGC0000673 |  | 0.50 | Terpene | pimara-8(14),15-diene | Aspergillus nidulans FGSC A4 |
| BGC0002320 |  | 0.49 | Terpene | conidiogenone | Penicillium rubens Wisconsin 54-1255 |
| BGC0000688 |  | 0.47 | Terpene | copalyl diphosphate | Diaporthe amygdali |
| BGC0000676 |  | 0.46 | Terpene | aphidicolin, aphidicolan-16β-ol, 3-deoxyaphidicolin, 17-deoxyaphidicolin | Phoma betae |
| BGC0002427 |  | 0.40 | Terpene, Polyketide | subglutinol A, subglutinol B | Metarhizium robertsii ARSEF 23 |
| BGC0001969 |  | 0.40 | Terpene | asperterpenoid A | Talaromyces wortmannii |
| BGC0001082 |  | 0.38 | Terpene | paxilline, paspaline, 13-dehydroxypaxilline, paspaline B | Penicillium paxilli |
| BGC0002604 |  | 0.35 | Polyketide | sartorypyrone A | Aspergillus felis |
| BGC0002610 |  | 0.35 | Terpene | stellatic acid | Aspergillus stellatus |
| BGC0002736 |  | 0.34 | Terpene | preaspterpenacid I | Aspergillus terreus NIH2624 |

Similar gene clusters

Shows regions from the antiSMASH database that are similar to the current region. Genes marked with the same colour are interrelated. White genes have no relationship.  
Click on reference genes to show details of similarities to genes within the current region.  
Click on an accession to open that entry in the antiSMASH database (if applicable).

All hits

NW\_021167089 (225979-247224): Sodiomyces alkalinus F11 unplaced genomic scaff... (66% of genes show similarity), terpene

NW\_009276924 (225128-246337): Verticillium dahliae VdLs.17 supercont1.21 geno... (66% of genes show similarity), terpene

NW\_023336259 (2624055-2647290): Colletotrichum scovillei strain TJNH1 chromos... (66% of genes show similarity), terpene

NW\_007360994 (84151-105327): Glarea lozoyensis ATCC 20868 chromosome Unknown ... (36% of genes show similarity), terpene

NC\_030959 (3057828-3079184): Colletotrichum higginsianum IMI 349063 chromosom... (66% of genes show similarity), terpene

CM014984 (1464921-1486163): Pyricularia sp. CBS 133598 strain NI919 chromosom... (42% of genes show similarity), terpene

NW\_015971150 (2029137-2050652): Sporothrix schenckii 1099-18 chromosome Unkno... (42% of genes show similarity), terpene

NW\_022474206 (2960715-2981897): Venustampulla echinocandica strain BP 5553 ch... (50% of genes show similarity), terpene

NC\_016457 (673447-692443): Thermothielavioides terrestris NRRL 8126 chromosom... (50% of genes show similarity), terpene

NW\_015622511 (178074-199244): Exophiala mesophila strain CBS 40295 unplaced g... (37% of genes show similarity), terpene
Download graphic

Similar known gene clusters from MIBiG 3.1

Shows clusters from the MiBIG database that are similar to the current region. Genes marked with the same colour are interrelated. White genes have no relationship.  
Click on reference genes to show details of similarities to genes within the current region.  
Click on an accession to open that entry in the MiBIG database.

No matches found.

Similar subclusters

Shows sub-cluster units that are similar to the current region. Genes marked with the same colour are interrelated. White genes have no relationship.  
Click on reference genes to show details of similarities to genes within the current region.

No matches found.

Detailed Pfam domain annotation

Shows Pfam domains found in each gene within the region. Click on each domain for more information about the domain's accession, location, and description. Domains with a bold border have Gene Ontology information.

Selected features only

Expand to show all names

Detailed TIGRFAM domain annotation

Shows TIGRFAM domains found in each gene within the region. Click on each domain for more information about the domain's accession, location, and description.

Selected features only

Expand to show all names

ENANITX01000529NITX01000529.1 - Region 1 - T1PKS

Shows the layout of the region, marking coding sequences and areas of interest. Clicking a gene will select it and show any relevant details. Clicking an area feature (e.g. a candidate cluster) will select all coding sequences within that area. Double clicking an area feature will zoom to that area. Multiple genes and area features can be selected by clicking them while holding the Ctrl key.  
More detailed help is available here.

Download region GenBank file

Download region SVG

Location: 1 - 43,426 nt. (total: 43,426 nt)
Show pHMM detection rules used

Region on contig edge.

T1PKS: cds(PKS\_AT and (PKS\_KS or ene\_KS or mod\_KS or hyb\_KS or itr\_KS or tra\_KS))

#### Legend:

core biosynthetic genes

additional biosynthetic genes

transport-related genes

regulatory genes

other genes

resistance

reset view

zoom to selection

Gene details

Shows details of the most recently selected gene, including names, products, location, and other annotations.

Select a gene to view the details available for it

Gene overview

NRPS/PKS domains

MIBiG comparison

ClusterBlast

KnownClusterBlast

SubClusterBlast

NRPS/PKS modules

Pfam domains

Gene/CDS overview

A brief tabular summary of genes/CDS features within the region.  
Filtering the table will also search biosynthetic profiles and gene function data. If enabled, the overview will then zoom to show the area covered by the filtered selection.  
Genes selected in the region drawing above will be marked in the table with an indicator to the left of the gene name.

Filter:

Automatically zoom to filtered/selected features

| Identifier | Product | Length | | Function | Sequence | | NCBI Blast | Filter details |
| --- | --- | --- | --- | --- | --- | --- | --- | --- |
|  |  | NT | AA |  | NT | AA |  |  |

No genes match the given filter

Detailed domain annotation

Shows NRPS- and PKS-related domains for each feature that contains them. Click on each domain for more information about the domain's location, consensus monomer prediction, and other details.  
A domain glossary is available here, and an explanation of the visualisation is available here.

Selected features only

Show module domains

Similar gene clusters

Shows careas that are similar to the current region to a reference database.  
Mouseover a score cell in the table to get a breakdown of how the score was calculated.The MIBiG database.  
  
Click on an accession to open that entry in the MIBiG database.

Analysis type:

Protocluster to Region
Region to Region

| Reference | T1PKS | Similarity score | Type | Compound(s) | Organism |
| --- | --- | --- | --- | --- | --- |
| BGC0002227 |  | 0.26 | NRP | AKML B, AKML D, AKML A, AKML C | Aspergillus luchuensis IFO 4308 |
| BGC0000046 |  | 0.26 | Polyketide | depudecin | Alternaria brassicicola |
| BGC0002228 |  | 0.26 | NRP | CIML B, CIML A, CIML D, CIML C | Colletotrichum incanum |
| BGC0001340 |  | 0.24 | Polyketide | byssochlamic acid | Byssochlamys fulva |
| BGC0001913 |  | 0.23 | Polyketide | phomoidride | fungal sp. ATCC 74256 |
| BGC0000056 |  | 0.23 | Polyketide | esperamicin | Actinomadura verrucosospora |
| BGC0001749 |  | 0.23 | Polyketide | epipyriculol | Pyricularia oryzae 70-15 |
| BGC0002729 |  | 0.23 | Polyketide | sordarial | Neurospora crassa OR74A |
| BGC0002266 |  | 0.22 | Terpene, Polyketide | calidoustene A, calidoustene B, calidoustene C | Aspergillus calidoustus |
| BGC0001254 |  | 0.22 | Polyketide | ACT-Toxin II | Alternaria alternata |

| Reference | Aggregated | Similarity score | Type | Compound(s) | Organism |
| --- | --- | --- | --- | --- | --- |
| BGC0002227 |  | 0.60 | NRP | AKML B, AKML D, AKML A, AKML C | Aspergillus luchuensis IFO 4308 |
| BGC0000046 |  | 0.60 | Polyketide | depudecin | Alternaria brassicicola |
| BGC0002228 |  | 0.59 | NRP | CIML B, CIML A, CIML D, CIML C | Colletotrichum incanum |
| BGC0001340 |  | 0.57 | Polyketide | byssochlamic acid | Byssochlamys fulva |
| BGC0001913 |  | 0.56 | Polyketide | phomoidride | fungal sp. ATCC 74256 |
| BGC0000056 |  | 0.56 | Polyketide | esperamicin | Actinomadura verrucosospora |
| BGC0001749 |  | 0.56 | Polyketide | epipyriculol | Pyricularia oryzae 70-15 |
| BGC0002729 |  | 0.56 | Polyketide | sordarial | Neurospora crassa OR74A |
| BGC0002266 |  | 0.55 | Terpene, Polyketide | calidoustene A, calidoustene B, calidoustene C | Aspergillus calidoustus |
| BGC0001254 |  | 0.55 | Polyketide | ACT-Toxin II | Alternaria alternata |

Similar gene clusters

Shows regions from the antiSMASH database that are similar to the current region. Genes marked with the same colour are interrelated. White genes have no relationship.  
Click on reference genes to show details of similarities to genes within the current region.  
Click on an accession to open that entry in the antiSMASH database (if applicable).

All hits

NW\_021167090 (680283-725597): Sodiomyces alkalinus F11 unplaced genomic scaff... (50% of genes show similarity), T1PKS

NW\_006271969 (4877275-4917236): Cordyceps militaris CM01 unplaced genomic sca... (23% of genes show similarity), T1PKS

NW\_023336265 (1662394-1719576): Colletotrichum scovillei strain TJNH1 chromos... (21% of genes show similarity), T1PKS

NC\_030962 (1405271-1459471): Colletotrichum higginsianum IMI 349063 chromosom... (17% of genes show similarity), T1PKS

NW\_022474213 (391991-435464): Venustampulla echinocandica strain BP 5553 chro... (20% of genes show similarity), T1PKS

NC\_049565 (1728455-1770196): Talaromyces rugulosus chromosome V, complete seq... (21% of genes show similarity), T1PKS

NW\_022984635 (3145916-3193220): Aspergillus tanneri strain NIH1004 chromosome... (20% of genes show similarity), T1PKS

NW\_001914837 (<303538-347736): Podospora anserina S mat+ genomic DNA chromoso... (20% of genes show similarity), T1PKS

NW\_003345199 (1003727-1098367): Nannizzia gypsea CBS 118893 supercont1.3 geno... (7% of genes show similarity), NRPS,T1PKS

NW\_003299163 (1364680-1417022): Microsporum canis CBS 113480 supercont1.7 gen... (11% of genes show similarity), T1PKS,T3PKS
Download graphic

Similar known gene clusters from MIBiG 3.1

Shows clusters from the MiBIG database that are similar to the current region. Genes marked with the same colour are interrelated. White genes have no relationship.  
Click on reference genes to show details of similarities to genes within the current region.  
Click on an accession to open that entry in the MiBIG database.

No matches found.

Similar subclusters

Shows sub-cluster units that are similar to the current region. Genes marked with the same colour are interrelated. White genes have no relationship.  
Click on reference genes to show details of similarities to genes within the current region.

No matches found.

Module view

Shows module structures for each candidate cluster in NRPS and PKS regions.   
Genes are shown in predicted order, and are only present when containing at least one complete module.   
A domain glossary is available here, and an explanation of the visualisation is available here.

Candidate 1 (0 - 43426): single T1PKS

Legend

Detailed Pfam domain annotation

Shows Pfam domains found in each gene within the region. Click on each domain for more information about the domain's accession, location, and description. Domains with a bold border have Gene Ontology information.

Selected features only

Expand to show all names

NRPS/PKS products

NRPS/PKS monomers

Predicted core structure(s)

Shows estimated product structure and polymer for each candidate cluster in the region. To show the product, click on the expander or the candidate cluster feature drawn in the overview.

For candidate cluster 1, location 0 - 43426:

Rough prediction of core scaffold based on assumed PKS/NRPS colinearity; tailoring reactions not taken into account

**Polymer prediction:**
:   (pk)

  
Direct lookup in NORINE database:
strict
or
relaxed

Link to NORINE database query form

NRPS/PKS monomer predictions

Shows the predicted monomers for each adynelation domain and acyltransferase within genes. Each gene prediction can be expanded to view detailed predictions of each domain. Each prediction can be expanded to view the predictions by tool (and, for some tools, further expanded for extra details).

**input.path1.gene2**: pk

:   **PKS\_AT (551..876)**: pk

    ATSignature: Malonyl-CoA

    Top 3 matches:
    :   Malonyl-CoA: 75.0%
    :   Methylmalonyl-CoA: 62.5%
    :   2-Methylbutyryl-CoA: 58.3%

      
    minowa: Methylmalonyl-CoA

    Prediction, score:
    :   Methylmalonyl-CoA: 90.4


        Methoxymalonyl-CoA: 81.2


        Malonyl-CoA: 64.9


        Isobutyryl-CoA: 61.5


        Ethylmalonyl-CoA: 55.6


        Propionyl-CoA: 37.3


        trans-1,2-CPDA: 35.5


        2-Methylbutyryl-CoA: 22.2


        CHC-CoA: 19.6


        3-Methylbutyryl-CoA: 19.1


        fatty\_acid: 19.0


        Benzoyl-CoA: 15.0


        inactive: 0.0


        Acetyl-CoA: 0.0

ENANITX01000534NITX01000534.1 - Region 1 - NRPS-like,T1PKS

Shows the layout of the region, marking coding sequences and areas of interest. Clicking a gene will select it and show any relevant details. Clicking an area feature (e.g. a candidate cluster) will select all coding sequences within that area. Double clicking an area feature will zoom to that area. Multiple genes and area features can be selected by clicking them while holding the Ctrl key.  
More detailed help is available here.

Download region GenBank file

Download region SVG

Location: 106,513 - 158,980 nt. (total: 52,468 nt)
Show pHMM detection rules used

Region on contig edge.

T1PKS: cds(PKS\_AT and (PKS\_KS or ene\_KS or mod\_KS or hyb\_KS or itr\_KS or tra\_KS))  
NRPS-like: cds((PP-binding or NAD\_binding\_4) and (AMP-binding or A-OX))

#### Legend:

core biosynthetic genes

additional biosynthetic genes

transport-related genes

regulatory genes

other genes

resistance

reset view

zoom to selection

Gene details

Shows details of the most recently selected gene, including names, products, location, and other annotations.

Select a gene to view the details available for it

Gene overview

NRPS/PKS domains

MIBiG comparison

ClusterBlast

KnownClusterBlast

SubClusterBlast

NRPS/PKS modules

Pfam domains

TIGRFAM domains

Gene/CDS overview

A brief tabular summary of genes/CDS features within the region.  
Filtering the table will also search biosynthetic profiles and gene function data. If enabled, the overview will then zoom to show the area covered by the filtered selection.  
Genes selected in the region drawing above will be marked in the table with an indicator to the left of the gene name.

Filter:

Automatically zoom to filtered/selected features

| Identifier | Product | Length | | Function | Sequence | | NCBI Blast | Filter details |
| --- | --- | --- | --- | --- | --- | --- | --- | --- |
|  |  | NT | AA |  | NT | AA |  |  |

No genes match the given filter

Detailed domain annotation

Shows NRPS- and PKS-related domains for each feature that contains them. Click on each domain for more information about the domain's location, consensus monomer prediction, and other details.  
A domain glossary is available here, and an explanation of the visualisation is available here.

Selected features only

Show module domains

Similar gene clusters

Shows careas that are similar to the current region to a reference database.  
Mouseover a score cell in the table to get a breakdown of how the score was calculated.The MIBiG database.  
  
Click on an accession to open that entry in the MIBiG database.

Analysis type:

Protocluster to Region
Region to Region

| Reference | T1PKS | NRPS-like | Similarity score | Type | Compound(s) | Organism |
| --- | --- | --- | --- | --- | --- | --- |
| BGC0002253 |  |  | 0.57 | Polyketide | waikikiamide A, waikikiamide B, waikikiamide C, (+)-semivioxanthin, semivioxanthin | Aspergillus sp. FM242 |
| BGC0000013 |  |  | 0.57 | Polyketide | alternariol | Aspergillus nidulans FGSC A4 |
| BGC0002507 |  |  | 0.56 | Polyketide | sporandol | Chrysosporium merdarium |
| BGC0001219 |  |  | 0.56 | Polyketide | shanorellin | Chaetomium globosum CBS 148.51 |
| BGC0002236 |  |  | 0.56 | Polyketide | 8-methyldiaporthin | Aspergillus oryzae RIB40 |
| BGC0001144 |  |  | 0.56 | Polyketide | neosartoricin B | Trichophyton tonsurans CBS 112818 |
| BGC0002596 |  |  | 0.55 | Polyketide | lecanoric acid, orsellinic acid | Claviceps purpurea 20.1 |
| BGC0002237 |  |  | 0.53 | Polyketide | dichlorodiaporthin | Aspergillus oryzae RIB40 |
| BGC0002646 |  |  | 0.53 | Polyketide | hancockinone A | Aspergillus hancockii |
| BGC0001542 |  |  | 0.51 | Polyketide | cercosporin | Cercospora zeina |

| Reference | Aggregated | Similarity score | Type | Compound(s) | Organism |
| --- | --- | --- | --- | --- | --- |
| BGC0002253 |  | 0.62 | Polyketide | waikikiamide A, waikikiamide B, waikikiamide C, (+)-semivioxanthin, semivioxanthin | Aspergillus sp. FM242 |
| BGC0000013 |  | 0.62 | Polyketide | alternariol | Aspergillus nidulans FGSC A4 |
| BGC0002507 |  | 0.62 | Polyketide | sporandol | Chrysosporium merdarium |
| BGC0001219 |  | 0.62 | Polyketide | shanorellin | Chaetomium globosum CBS 148.51 |
| BGC0002236 |  | 0.62 | Polyketide | 8-methyldiaporthin | Aspergillus oryzae RIB40 |
| BGC0001144 |  | 0.61 | Polyketide | neosartoricin B | Trichophyton tonsurans CBS 112818 |
| BGC0002596 |  | 0.61 | Polyketide | lecanoric acid, orsellinic acid | Claviceps purpurea 20.1 |
| BGC0002237 |  | 0.60 | Polyketide | dichlorodiaporthin | Aspergillus oryzae RIB40 |
| BGC0002646 |  | 0.60 | Polyketide | hancockinone A | Aspergillus hancockii |
| BGC0001542 |  | 0.59 | Polyketide | cercosporin | Cercospora zeina |

Similar gene clusters

Shows regions from the antiSMASH database that are similar to the current region. Genes marked with the same colour are interrelated. White genes have no relationship.  
Click on reference genes to show details of similarities to genes within the current region.  
Click on an accession to open that entry in the antiSMASH database (if applicable).

All hits

NW\_009276941 (357077-408070): Verticillium dahliae VdLs.17 supercont1.26 geno... (50% of genes show similarity), NRPS-like,T1PKS

NW\_001939254 (1234647-1284505): Pyrenophora tritici-repentis Pt-1C-BFP superc... (50% of genes show similarity), NRPS-like,T1PKS

NW\_003315023 (387992-434620): Verticillium alfalfae VaMs.102 supercont1.16 ge... (41% of genes show similarity), T1PKS

NT\_165933 (1427101-1535424): Aspergillus terreus NIH2624 scaffold 10 genomic ... (19% of genes show similarity), NRPS,NRPS-like,T1PKS,terpene

NC\_016476 (830278-878421): Thermothelomyces thermophilus ATCC 42464 chromosom... (23% of genes show similarity), NRPS-like,T1PKS

NW\_022984628 (4194508-4271225): Aspergillus tanneri strain NIH1004 chromosome... (31% of genes show similarity), T1PKS

NW\_020939749 (1903727-1956364): Sparassis crispa DNA, contig 000002F, whole g... (20% of genes show similarity), NRPS-like,T1PKS

NT\_165928 (153206-202357): Aspergillus terreus NIH2624 scaffold 5 genomic sca... (16% of genes show similarity), NRPS-like,T1PKS

NZ\_JPLW01000002 (1677186-1766744): Amycolatopsis sp. MJM2582 contig00002, who... (7% of genes show similarity), T1PKS,hglE-KS

NZ\_KB913022 (3244266-3288793): Salinispora pacifica DSM 45543 = CNS-863 strai... (15% of genes show similarity), T1PKS
Download graphic

Similar known gene clusters from MIBiG 3.1

Shows clusters from the MiBIG database that are similar to the current region. Genes marked with the same colour are interrelated. White genes have no relationship.  
Click on reference genes to show details of similarities to genes within the current region.  
Click on an accession to open that entry in the MiBIG database.

No matches found.

Similar subclusters

Shows sub-cluster units that are similar to the current region. Genes marked with the same colour are interrelated. White genes have no relationship.  
Click on reference genes to show details of similarities to genes within the current region.

No matches found.

Module view

Shows module structures for each candidate cluster in NRPS and PKS regions.   
Genes are shown in predicted order, and are only present when containing at least one complete module.   
A domain glossary is available here, and an explanation of the visualisation is available here.

Candidate 1 (106512 - 158980): single T1PKS

Candidate 2 (106512 - 158980): neighbouring T1PKS-NRPS-like

Candidate 3 (114703 - 158980): single NRPS-like

Legend

Detailed Pfam domain annotation

Shows Pfam domains found in each gene within the region. Click on each domain for more information about the domain's accession, location, and description. Domains with a bold border have Gene Ontology information.

Selected features only

Expand to show all names

Detailed TIGRFAM domain annotation

Shows TIGRFAM domains found in each gene within the region. Click on each domain for more information about the domain's accession, location, and description.

Selected features only

Expand to show all names

NRPS/PKS products

NRPS/PKS monomers

Predicted core structure(s)

Shows estimated product structure and polymer for each candidate cluster in the region. To show the product, click on the expander or the candidate cluster feature drawn in the overview.

For candidate cluster 1, location 106512 - 158980:

Rough prediction of core scaffold based on assumed PKS/NRPS colinearity; tailoring reactions not taken into account

**Polymer prediction:**
:   (X) + (mal)

  
Direct lookup in NORINE database:
strict
or
relaxed

---

For candidate cluster 2, location 106512 - 158980:

Rough prediction of core scaffold based on assumed PKS/NRPS colinearity; tailoring reactions not taken into account

**Polymer prediction:**
:   (X) + (mal)

  
Direct lookup in NORINE database:
strict
or
relaxed

---

For candidate cluster 3, location 114703 - 158980:

Rough prediction of core scaffold based on assumed PKS/NRPS colinearity; tailoring reactions not taken into account

**Polymer prediction:**
:   (X) + (mal)

  
Direct lookup in NORINE database:
strict
or
relaxed

Link to NORINE database query form

NRPS/PKS monomer predictions

Shows the predicted monomers for each adynelation domain and acyltransferase within genes. Each gene prediction can be expanded to view detailed predictions of each domain. Each prediction can be expanded to view the predictions by tool (and, for some tools, further expanded for extra details).

**input.path1.gene45**: mal

:   **PKS\_AT (554..849)**: mal

    ATSignature: Malonyl-CoA

    Top 3 matches:
    :   Malonyl-CoA: 66.7%
    :   Ethylmalonyl-CoA: 54.2%
    :   Methylmalonyl-CoA: 54.2%

      
    minowa: Malonyl-CoA

    Prediction, score:
    :   Malonyl-CoA: 88.8


        Methylmalonyl-CoA: 68.3


        Methoxymalonyl-CoA: 55.6


        Isobutyryl-CoA: 42.8


        inactive: 41.7


        Benzoyl-CoA: 31.5


        Propionyl-CoA: 27.5


        Ethylmalonyl-CoA: 26.0


        2-Methylbutyryl-CoA: 22.0


        fatty\_acid: 19.4


        CHC-CoA: 17.1


        trans-1,2-CPDA: 12.7


        Acetyl-CoA: 9.1


        3-Methylbutyryl-CoA: 0.0

  
**input.path1.gene46**: X

:   **AMP-binding (5..264)**: X

    nrpys: Gly, Ala, Val, Leu, Ile, Abu, Ival

    SVM prediction details:
    :   Predicted physicochemical class:
        :   hydrophobic-aliphatic (Ala, Gly, Val, Leu, Ile, Abu, Ival, Ser, Thr, Hpg, Dhpg, Cys, Pro, Hpr)

        Large clusters prediction:
        :   Apolar, aliphatic (Gly, Ala, Val, Leu, Ile, Abu, Ival)

        Small clusters prediction:
        :   N/A

        Single AA prediction:
        :   N/A

    Stachelhaus prediction details:
    :   Stachelhaus sequence:
        :   GFLMAGHAIK

        Nearest Stachelhaus code(s):
        :   meOrs GFVMLGHLGK (56% 8Å match)

        Stachelhaus code match:
        :   60% (weak)

ENANITX01000574NITX01000574.1 - Region 1 - T1PKS

Shows the layout of the region, marking coding sequences and areas of interest. Clicking a gene will select it and show any relevant details. Clicking an area feature (e.g. a candidate cluster) will select all coding sequences within that area. Double clicking an area feature will zoom to that area. Multiple genes and area features can be selected by clicking them while holding the Ctrl key.  
More detailed help is available here.

Download region GenBank file

Download region SVG

Location: 26,928 - 72,293 nt. (total: 45,366 nt)
Show pHMM detection rules used

Region on contig edge.

T1PKS: cds(PKS\_AT and (PKS\_KS or ene\_KS or mod\_KS or hyb\_KS or itr\_KS or tra\_KS))

#### Legend:

core biosynthetic genes

additional biosynthetic genes

transport-related genes

regulatory genes

other genes

resistance

reset view

zoom to selection

Gene details

Shows details of the most recently selected gene, including names, products, location, and other annotations.

Select a gene to view the details available for it

Gene overview

NRPS/PKS domains

MIBiG comparison

ClusterBlast

KnownClusterBlast

SubClusterBlast

Pfam domains

TIGRFAM domains

Gene/CDS overview

A brief tabular summary of genes/CDS features within the region.  
Filtering the table will also search biosynthetic profiles and gene function data. If enabled, the overview will then zoom to show the area covered by the filtered selection.  
Genes selected in the region drawing above will be marked in the table with an indicator to the left of the gene name.

Filter:

Automatically zoom to filtered/selected features

| Identifier | Product | Length | | Function | Sequence | | NCBI Blast | Filter details |
| --- | --- | --- | --- | --- | --- | --- | --- | --- |
|  |  | NT | AA |  | NT | AA |  |  |

No genes match the given filter

Detailed domain annotation

Shows NRPS- and PKS-related domains for each feature that contains them. Click on each domain for more information about the domain's location, consensus monomer prediction, and other details.  
A domain glossary is available here, and an explanation of the visualisation is available here.

Selected features only

Show module domains

Similar gene clusters

Shows careas that are similar to the current region to a reference database.  
Mouseover a score cell in the table to get a breakdown of how the score was calculated.The MIBiG database.  
  
Click on an accession to open that entry in the MIBiG database.

Analysis type:

Protocluster to Region
Region to Region

| Reference | T1PKS | Similarity score | Type | Compound(s) | Organism |
| --- | --- | --- | --- | --- | --- |
| BGC0001254 |  | 0.19 | Polyketide | ACT-Toxin II | Alternaria alternata |
| BGC0002191 |  | 0.19 | Polyketide | prolipyrone B, gibepyrone D | Fusarium graminearum PH-1 |
| BGC0002429 |  | 0.18 | Terpene, Polyketide | higginsianin B | Colletotrichum higginsianum IMI 349063 |
| BGC0002240 |  | 0.18 | Polyketide | BAB, BAA | Metarhizium anisopliae |
| BGC0001284 |  | 0.17 | Polyketide | alternariol | Parastagonospora nodorum SN15 |
| BGC0002155 |  | 0.17 | Polyketide | nectriapyrone C, nectriapyrone D, nectriapyrone | Pyricularia oryzae 70-15 |
| BGC0002192 |  | 0.17 | Polyketide | FR901512 | fungal sp. No.14919 |
| BGC0002745 |  | 0.17 | Polyketide | verrucosidin | Penicillium polonicum |
| BGC0001264 |  | 0.16 | Polyketide | betaenone A, betaenone B, betaenone C | Phoma betae |
| BGC0001068 |  | 0.16 | Terpene, Polyketide | pyripyropene A | unidentified unclassified sequences. |

| Reference | Aggregated | Similarity score | Type | Compound(s) | Organism |
| --- | --- | --- | --- | --- | --- |
| BGC0001254 |  | 0.51 | Polyketide | ACT-Toxin II | Alternaria alternata |
| BGC0002191 |  | 0.50 | Polyketide | prolipyrone B, gibepyrone D | Fusarium graminearum PH-1 |
| BGC0002429 |  | 0.50 | Terpene, Polyketide | higginsianin B | Colletotrichum higginsianum IMI 349063 |
| BGC0002240 |  | 0.49 | Polyketide | BAB, BAA | Metarhizium anisopliae |
| BGC0001284 |  | 0.48 | Polyketide | alternariol | Parastagonospora nodorum SN15 |
| BGC0002155 |  | 0.48 | Polyketide | nectriapyrone C, nectriapyrone D, nectriapyrone | Pyricularia oryzae 70-15 |
| BGC0002192 |  | 0.48 | Polyketide | FR901512 | fungal sp. No.14919 |
| BGC0002745 |  | 0.48 | Polyketide | verrucosidin | Penicillium polonicum |
| BGC0001264 |  | 0.47 | Polyketide | betaenone A, betaenone B, betaenone C | Phoma betae |
| BGC0001068 |  | 0.47 | Terpene, Polyketide | pyripyropene A | unidentified unclassified sequences. |

Similar gene clusters

Shows regions from the antiSMASH database that are similar to the current region. Genes marked with the same colour are interrelated. White genes have no relationship.  
Click on reference genes to show details of similarities to genes within the current region.  
Click on an accession to open that entry in the antiSMASH database (if applicable).

All hits

NW\_021167094 (217435-265256): Sodiomyces alkalinus F11 unplaced genomic scaff... (35% of genes show similarity), T1PKS

NW\_009276940 (706502-754236): Verticillium dahliae VdLs.17 supercont1.3 genom... (18% of genes show similarity), T1PKS

NW\_003315037 (717958-759991): Verticillium alfalfae VaMs.102 supercont1.2 gen... (22% of genes show similarity), T1PKS

NZ\_CP020809 (3745995-3834119): Mycobacterium dioxanotrophicus strain PH-06 ch... (6% of genes show similarity), NRPS,T1PKS

NZ\_CP024087 (6765982-6907073): Micromonospora tulbaghiae strain CNY-010 chrom... (6% of genes show similarity), T1PKS

NW\_013550603 (4312478-4353657): Rhinocladiella mackenziei CBS 650.93 unplaced... (15% of genes show similarity), T1PKS

NZ\_LZNS01000001 (535763-606162): Streptomyces sp. MP131-18 scaf 01, whole gen... (4% of genes show similarity), T1PKS,butyrolactone

NZ\_CP010071 (806855-865200): Mycobacterium sp. QIA-37, complete genome (4% of genes show similarity), NRPS,T1PKS

NZ\_CP031516 (866040-923701): [Mycobacterium] chelonae subsp. gwanakae strain ... (4% of genes show similarity), NRPS,T1PKS

NZ\_KQ949024 (607928-695233): Streptomyces sp. DSM 15324 PRJNA299231 s002, who... (2% of genes show similarity), NRPS,T1PKS,transAT-PKS-like
Download graphic

Similar known gene clusters from MIBiG 3.1

Shows clusters from the MiBIG database that are similar to the current region. Genes marked with the same colour are interrelated. White genes have no relationship.  
Click on reference genes to show details of similarities to genes within the current region.  
Click on an accession to open that entry in the MiBIG database.

No matches found.

Similar subclusters

Shows sub-cluster units that are similar to the current region. Genes marked with the same colour are interrelated. White genes have no relationship.  
Click on reference genes to show details of similarities to genes within the current region.

No matches found.

Detailed Pfam domain annotation

Shows Pfam domains found in each gene within the region. Click on each domain for more information about the domain's accession, location, and description. Domains with a bold border have Gene Ontology information.

Selected features only

Expand to show all names

Detailed TIGRFAM domain annotation

Shows TIGRFAM domains found in each gene within the region. Click on each domain for more information about the domain's accession, location, and description.

Selected features only

Expand to show all names

NRPS/PKS monomers

NRPS/PKS monomer predictions

Shows the predicted monomers for each adynelation domain and acyltransferase within genes. Each gene prediction can be expanded to view detailed predictions of each domain. Each prediction can be expanded to view the predictions by tool (and, for some tools, further expanded for extra details).

**input.path1.gene16**: pk

:   **PKS\_AT (510..827)**: pk

    ATSignature: Malonyl-CoA

    Top 3 matches:
    :   Malonyl-CoA: 79.2%
    :   Methylmalonyl-CoA: 70.8%
    :   Ethylmalonyl-CoA: 62.5%

      
    minowa: Methylmalonyl-CoA

    Prediction, score:
    :   Methylmalonyl-CoA: 83.6


        Methoxymalonyl-CoA: 75.9


        Isobutyryl-CoA: 58.2


        Ethylmalonyl-CoA: 46.9


        Malonyl-CoA: 45.3


        Propionyl-CoA: 38.4


        2-Methylbutyryl-CoA: 29.1


        trans-1,2-CPDA: 28.0


        Benzoyl-CoA: 19.4


        CHC-CoA: 10.4


        fatty\_acid: 9.8


        inactive: 9.2


        3-Methylbutyryl-CoA: 5.0


        Acetyl-CoA: 2.6

ENANITX01000600NITX01000600.1 - Region 1 - T1PKS

Shows the layout of the region, marking coding sequences and areas of interest. Clicking a gene will select it and show any relevant details. Clicking an area feature (e.g. a candidate cluster) will select all coding sequences within that area. Double clicking an area feature will zoom to that area. Multiple genes and area features can be selected by clicking them while holding the Ctrl key.  
More detailed help is available here.

Download region GenBank file

Download region SVG

Location: 69,838 - 105,701 nt. (total: 35,864 nt)
Show pHMM detection rules used

Region on contig edge.

T1PKS: cds(PKS\_AT and (PKS\_KS or ene\_KS or mod\_KS or hyb\_KS or itr\_KS or tra\_KS))

#### Legend:

core biosynthetic genes

additional biosynthetic genes

transport-related genes

regulatory genes

other genes

resistance

reset view

zoom to selection

Gene details

Shows details of the most recently selected gene, including names, products, location, and other annotations.

Select a gene to view the details available for it

Gene overview

NRPS/PKS domains

MIBiG comparison

ClusterBlast

KnownClusterBlast

SubClusterBlast

Pfam domains

TIGRFAM domains

Gene/CDS overview

A brief tabular summary of genes/CDS features within the region.  
Filtering the table will also search biosynthetic profiles and gene function data. If enabled, the overview will then zoom to show the area covered by the filtered selection.  
Genes selected in the region drawing above will be marked in the table with an indicator to the left of the gene name.

Filter:

Automatically zoom to filtered/selected features

| Identifier | Product | Length | | Function | Sequence | | NCBI Blast | Filter details |
| --- | --- | --- | --- | --- | --- | --- | --- | --- |
|  |  | NT | AA |  | NT | AA |  |  |

No genes match the given filter

Detailed domain annotation

Shows NRPS- and PKS-related domains for each feature that contains them. Click on each domain for more information about the domain's location, consensus monomer prediction, and other details.  
A domain glossary is available here, and an explanation of the visualisation is available here.

Selected features only

Show module domains

Similar gene clusters

Shows careas that are similar to the current region to a reference database.  
Mouseover a score cell in the table to get a breakdown of how the score was calculated.The MIBiG database.  
  
Click on an accession to open that entry in the MIBiG database.

Analysis type:

Protocluster to Region
Region to Region

| Reference | T1PKS | Similarity score | Type | Compound(s) | Organism |
| --- | --- | --- | --- | --- | --- |
| BGC0000056 |  | 0.23 | Polyketide | esperamicin | Actinomadura verrucosospora |
| BGC0002507 |  | 0.20 | Polyketide | sporandol | Chrysosporium merdarium |
| BGC0000156 |  | 0.19 | Polyketide | TAN-1612, 1-(2,3,5,10-tetrahydroxy-7-methoxy-4-oxo-1,2,3,4-tetrahydroanthracen-2-yl)pentane-2,4-dione, desmethyl TAN-1612 | Aspergillus niger |
| BGC0001257 |  | 0.19 | Polyketide | 1,3,6,8-tetrahydroxynaphthalene | Nodulisporium sp. ATCC74245 |
| BGC0001284 |  | 0.19 | Polyketide | alternariol | Parastagonospora nodorum SN15 |
| BGC0001906 |  | 0.19 | Polyketide | naphthalene | Daldinia eschscholzii IFB-TL01 |
| BGC0001258 |  | 0.19 | Polyketide | 1,3,6,8-tetrahydroxynaphthalene | Glarea lozoyensis |
| BGC0002154 |  | 0.19 | Polyketide | 1,3,5,8-tetrahydroxynapthalene, 1-(alpha-l-(2-O-methyl)-6-deoxymannopyranosyloxy)-3,6,8-trimethoxynaphthalene | Pyricularia oryzae 70-15 |
| BGC0000013 |  | 0.19 | Polyketide | alternariol | Aspergillus nidulans FGSC A4 |
| BGC0002236 |  | 0.19 | Polyketide | 8-methyldiaporthin | Aspergillus oryzae RIB40 |

| Reference | Aggregated | Similarity score | Type | Compound(s) | Organism |
| --- | --- | --- | --- | --- | --- |
| BGC0000056 |  | 0.56 | Polyketide | esperamicin | Actinomadura verrucosospora |
| BGC0002507 |  | 0.52 | Polyketide | sporandol | Chrysosporium merdarium |
| BGC0000156 |  | 0.52 | Polyketide | TAN-1612, 1-(2,3,5,10-tetrahydroxy-7-methoxy-4-oxo-1,2,3,4-tetrahydroanthracen-2-yl)pentane-2,4-dione, desmethyl TAN-1612 | Aspergillus niger |
| BGC0001257 |  | 0.51 | Polyketide | 1,3,6,8-tetrahydroxynaphthalene | Nodulisporium sp. ATCC74245 |
| BGC0001284 |  | 0.51 | Polyketide | alternariol | Parastagonospora nodorum SN15 |
| BGC0001906 |  | 0.51 | Polyketide | naphthalene | Daldinia eschscholzii IFB-TL01 |
| BGC0001258 |  | 0.51 | Polyketide | 1,3,6,8-tetrahydroxynaphthalene | Glarea lozoyensis |
| BGC0002154 |  | 0.51 | Polyketide | 1,3,5,8-tetrahydroxynapthalene, 1-(alpha-l-(2-O-methyl)-6-deoxymannopyranosyloxy)-3,6,8-trimethoxynaphthalene | Pyricularia oryzae 70-15 |
| BGC0000013 |  | 0.51 | Polyketide | alternariol | Aspergillus nidulans FGSC A4 |
| BGC0002236 |  | 0.51 | Polyketide | 8-methyldiaporthin | Aspergillus oryzae RIB40 |

Similar gene clusters

Shows regions from the antiSMASH database that are similar to the current region. Genes marked with the same colour are interrelated. White genes have no relationship.  
Click on reference genes to show details of similarities to genes within the current region.  
Click on an accession to open that entry in the antiSMASH database (if applicable).

All hits

CP051141 (2205984-2251512): Peltaster fructicola strain LNHT1506 chromosome 3 (36% of genes show similarity), T1PKS

NW\_011942151 (90937-172848): Metarhizium robertsii ARSEF 23 MAA Scf 11, whole... (14% of genes show similarity), NRPS,T1PKS,terpene

NW\_014574694 (86125-153989): Metarhizium brunneum ARSEF 3297 chromosome Unkno... (15% of genes show similarity), NRPS,T1PKS

NW\_022984630 (495088-539069): Aspergillus tanneri strain NIH1004 chromosome U... (35% of genes show similarity), T1PKS

NW\_006917098 (414284-449249): Pestalotiopsis fici W106-1 unplaced genomic sca... (42% of genes show similarity), T1PKS

NC\_007197 (3816930-3862461): Aspergillus fumigatus Af293 chromosome 4, whole ... (28% of genes show similarity), T1PKS

NW\_022984634 (4255890-4301503): Aspergillus tanneri strain NIH1004 chromosome... (26% of genes show similarity), T1PKS

NT\_165935 (1259087-1319359): Aspergillus terreus NIH2624 scaffold 12 genomic ... (20% of genes show similarity), NRPS,T1PKS

NC\_016458 (5151387-5191687): Thermothielavioides terrestris NRRL 8126 chromos... (25% of genes show similarity), T1PKS

NW\_019154043 (172137-217646): Pochonia chlamydosporia 170 chromosome Unknown ... (25% of genes show similarity), T1PKS
Download graphic

Similar known gene clusters from MIBiG 3.1

Shows clusters from the MiBIG database that are similar to the current region. Genes marked with the same colour are interrelated. White genes have no relationship.  
Click on reference genes to show details of similarities to genes within the current region.  
Click on an accession to open that entry in the MiBIG database.

All hits

cryptosporioptide B/cryptosporioptide A/cryptosporioptide C

geodin

3'-methoxy-1,2-dehydropenicillide/pestalotiollide B/pestalotiollide C/1,2-dehydropenicillide/3-methoxy-1,2-dehydropenicillide/1,2-epoxy-3,4-didehydropenicillide

trypacidin

RES-1214-2

agnestin A/agnestin B

secalonic acids

neosartorin

4-chloropinselin/pinselin/chloromonilinic acid B/chloromonilinic acid D/4-hydroxyvertixanthone

chrysoxanthone A/chrysoxanthone B/chrysoxanthone C
Download graphic

Similar subclusters

Shows sub-cluster units that are similar to the current region. Genes marked with the same colour are interrelated. White genes have no relationship.  
Click on reference genes to show details of similarities to genes within the current region.

No matches found.

Detailed Pfam domain annotation

Shows Pfam domains found in each gene within the region. Click on each domain for more information about the domain's accession, location, and description. Domains with a bold border have Gene Ontology information.

Selected features only

Expand to show all names

Detailed TIGRFAM domain annotation

Shows TIGRFAM domains found in each gene within the region. Click on each domain for more information about the domain's accession, location, and description.

Selected features only

Expand to show all names

NRPS/PKS monomers

NRPS/PKS monomer predictions

Shows the predicted monomers for each adynelation domain and acyltransferase within genes. Each gene prediction can be expanded to view detailed predictions of each domain. Each prediction can be expanded to view the predictions by tool (and, for some tools, further expanded for extra details).

**input.path1.gene33**: mal

:   **PKS\_AT (947..1238)**: mal

    ATSignature: Malonyl-CoA

    Top 3 matches:
    :   Malonyl-CoA: 70.8%
    :   inactive: 62.5%
    :   Methylmalonyl-CoA: 58.3%

      
    minowa: Malonyl-CoA

    Prediction, score:
    :   Malonyl-CoA: 99.9


        inactive: 64.7


        Methylmalonyl-CoA: 61.4


        Methoxymalonyl-CoA: 52.9


        Propionyl-CoA: 46.2


        Isobutyryl-CoA: 33.4


        Benzoyl-CoA: 24.9


        3-Methylbutyryl-CoA: 22.5


        Ethylmalonyl-CoA: 20.4


        2-Methylbutyryl-CoA: 19.9


        CHC-CoA: 18.7


        trans-1,2-CPDA: 14.8


        Acetyl-CoA: 13.6


        fatty\_acid: 11.9

If you have found antiSMASH useful, please cite us.
