# Supplementary material for: Alkaliphilic/Alkali-Tolerant Fungi: Molecular, Biochemical, and Biotechnological Aspects
Source: J Fungi (Basel). 2023 Jun 9;9(6):652. doi: 10.3390/jof9060652 (PMC10301932; doi:10.3390/jof9060652)
Supplement: Supplementary file 1 [file jof-09-00652-s001.zip › S2/knownclusterblast/region1/input.path1.gene10_mibig_hits.html]

| MIBiG Protein | Description | MIBiG Cluster | MiBiG Product | % ID | % Coverage | BLAST Score | E-value |
| --- | --- | --- | --- | --- | --- | --- | --- |
| ABA70583.1 | isopenicillin\_N\_synthetase | BGC0000404 | NRP | 76.0 | 100.0 | 539.0 | 7.66e-195 |
| ABR12616.1 | IPN\_synthase | BGC0000405 | NRP:Beta-lactam | 76.0 | 100.0 | 539.0 | 7.66e-195 |
| EDY47119.1 | isopenicillin\_N\_synthetase | BGC0000319 | NRP:Beta-lactam | 58.0 | 99.1 | 385.0 | 3.46e-134 |
| AMM63174.1 | AniF | BGC0001371 | NRP | 29.0 | 45.1 | 59.0 | 2.48e-09 |
| QBE85645.1 | BuaE | BGC0001857 | Alkaloid+NRP+Polyketide:Iterative type I polyketide | 28.0 | 62.7 | 57.0 | 5.7e-09 |
