# Supplementary material for: Alkaliphilic/Alkali-Tolerant Fungi: Molecular, Biochemical, and Biotechnological Aspects
Source: J Fungi (Basel). 2023 Jun 9;9(6):652. doi: 10.3390/jof9060652 (PMC10301932; doi:10.3390/jof9060652)
Supplement: Supplementary file 1 [file jof-09-00652-s001.zip › S2/knownclusterblast/region1/input.path1.gene11_mibig_hits.html]

| MIBiG Protein | Description | MIBiG Cluster | MiBiG Product | % ID | % Coverage | BLAST Score | E-value |
| --- | --- | --- | --- | --- | --- | --- | --- |
| ACH72904.1 | AflH | BGC0000011 | Polyketide | 33.0 | 76.4 | 125.0 | 1.02e-32 |
| AAS90028.1 | AdhA | BGC0000008 | Polyketide | 34.0 | 62.4 | 122.0 | 9.11e-32 |
| AAS90073.1 | AdhA | BGC0000010 | Polyketide | 34.0 | 62.4 | 122.0 | 9.11e-32 |
| AAS90097.1 | AdhA | BGC0000006 | Polyketide | 34.0 | 62.4 | 121.0 | 1.27e-31 |
| BAE71320.1 | short\_chain\_alcohol\_dehydrogenase | BGC0000004 | Polyketide | 34.0 | 62.4 | 120.0 | 2.45e-31 |
| AAS90053.1 | AdhA | BGC0000009 | Polyketide | 34.0 | 63.2 | 118.0 | 1.44e-30 |
| AAS90005.1 | AdhA | BGC0000007 | Polyketide | 34.0 | 62.4 | 116.0 | 8.66e-30 |
| AVY05515.1 | dehydrogenase | BGC0001571 | Terpene | 31.0 | 59.3 | 95.0 | 9.44e-22 |
| OBR09785.1 | Short\_chain\_dehydrogenase | BGC0002429 | Terpene+Polyketide | 29.0 | 63.0 | 74.0 | 1.93e-14 |
| CAM34370.1 | putative\_3-oxoacyl-ACP\_reductase | BGC0000242 | Polyketide | 30.0 | 63.5 | 68.0 | 1.35e-12 |
| EEF48747.1 | short\_chain\_alcohol\_dehydrogenase,\_putative | BGC0002393 | Terpene | 27.0 | 62.2 | 66.0 | 1.25e-11 |
| ABS75820.1 | BacC | BGC0001184 | Other | 24.0 | 57.3 | 62.0 | 1e-10 |
| AIL50182.1 | putative\_ketoreductase | BGC0000213 | Polyketide:Type II polyketide | 25.0 | 58.5 | 61.0 | 3.6e-10 |
| ACX35428.1 | BacC | BGC0000888 | Other | 24.0 | 57.3 | 61.0 | 4.45e-10 |
| ADI58639.1 | Ketoreductase | BGC0000187 | Polyketide:Type II polyketide | 27.0 | 57.8 | 60.0 | 4.86e-10 |
| QCT05738.1 | Tri5 | BGC0001983 | Other | 26.0 | 63.2 | 60.0 | 6.55e-10 |
| ARS01471.1 | NcmD | BGC0001702 | NRP+Polyketide | 30.0 | 59.8 | 56.0 | 1.82e-08 |
| BAK64641.1 | dihydroxy\_ketone\_synthase | BGC0000135 | Polyketide | 28.0 | 63.0 | 56.0 | 2.48e-08 |
| BAJ52686.1 | putative\_3-oxoacyl-ACP\_reductase | BGC0000222 | Polyketide | 29.0 | 58.0 | 55.0 | 2.73e-08 |
| BCN13450.1 | glucose\_1-dehydrogenase | BGC0002457 | NRP | 25.0 | 58.5 | 54.0 | 7.04e-08 |
| ABX71095.1 | Lct12 | BGC0000238 | Polyketide | 28.0 | 57.5 | 54.0 | 8.91e-08 |
| ADE34504.1 | ssfK | BGC0000269 | Polyketide:Type II polyketide+Saccharide:Hybrid/tailoring saccharide | 40.0 | 23.8 | 53.0 | 1.17e-07 |
| AMK51287.1 | Hex30 | BGC0001376 | Polyketide | 25.0 | 59.3 | 52.0 | 2.8e-07 |
| WP\_051970762.1 | SDR\_family\_oxidoreductase | BGC0002536 | NRP | 38.0 | 24.1 | 52.0 | 3.11e-07 |
| OWA01602.1 | short-chain\_dehydrogenase | BGC0001439 | Polyketide+Saccharide:Hybrid/tailoring saccharide | 26.0 | 58.0 | 50.0 | 9.68e-07 |
| WP\_035850920.1 | glucose\_1-dehydrogenase | BGC0002536 | NRP | 27.0 | 58.5 | 50.0 | 1.35e-06 |
| EYE95337.1 | NAD(P)-binding\_protein | BGC0002234 | Polyketide | 25.0 | 59.6 | 50.0 | 1.95e-06 |
| ACZ87047.1 | short-chain\_dehydrogenase/reductase\_SDR | BGC0002732 | Polyketide | 25.0 | 59.3 | 49.0 | 2.2e-06 |
| ADE22321.1 | 3-oxoacyl-ACP\_reductase | BGC0000279 | Polyketide | 28.0 | 58.8 | 49.0 | 2.3e-06 |
| ACM68692.1 | putative\_oxidoreductase | BGC0000298 | NRP | 33.0 | 27.2 | 49.0 | 2.41e-06 |
| WP\_037817213.1 | SDR\_family\_oxidoreductase | BGC0002137 | Polyketide | 30.0 | 35.0 | 49.0 | 4.13e-06 |
| CAK50776.1 | ketoreductase | BGC0000247 | Polyketide:Type II polyketide+Saccharide:Oligosaccharide | 25.0 | 61.9 | 48.0 | 5.6e-06 |
| AYU66263.1 | TjhD5 | BGC0002461 | Polyketide | 25.0 | 60.9 | 48.0 | 5.6e-06 |
