# Supplementary material for: Alkaliphilic/Alkali-Tolerant Fungi: Molecular, Biochemical, and Biotechnological Aspects
Source: J Fungi (Basel). 2023 Jun 9;9(6):652. doi: 10.3390/jof9060652 (PMC10301932; doi:10.3390/jof9060652)
Supplement: Supplementary file 1 [file jof-09-00652-s001.zip › S2/knownclusterblast/region1/input.path1.gene12_mibig_hits.html]

| MIBiG Protein | Description | MIBiG Cluster | MiBiG Product | % ID | % Coverage | BLAST Score | E-value |
| --- | --- | --- | --- | --- | --- | --- | --- |
| CAD62204.1 | Ata10\_protein | BGC0000873 | Other | 27.0 | 67.8 | 109.0 | 9.74e-25 |
| BAE62226.1 |  | BGC0002237 | Polyketide | 27.0 | 68.5 | 103.0 | 1.11e-22 |
| EIN09539.1 | pyranose\_dehydrogenase | BGC0002213 | Polyketide | 25.0 | 70.3 | 102.0 | 5.36e-22 |
| AAS90106.1 | VBS | BGC0000006 | Polyketide | 26.0 | 71.8 | 100.0 | 2.54e-21 |
| BAE71331.1 | versicolorin\_B\_synthase | BGC0000004 | Polyketide | 26.0 | 71.8 | 98.0 | 1.03e-20 |
| AAS90019.1 | VBS | BGC0000007 | Polyketide | 26.0 | 71.8 | 98.0 | 1.03e-20 |
| AAS90042.1 | VBS | BGC0000008 | Polyketide | 26.0 | 71.8 | 97.0 | 3.13e-20 |
| AAS90088.1 | VBS | BGC0000010 | Polyketide | 26.0 | 71.8 | 97.0 | 3.13e-20 |
| ctg1\_orf10 |  | BGC0000846 | Other | 26.0 | 69.0 | 96.0 | 4.64e-20 |
| EAU32818.1 | predicted\_protein | BGC0000160 | Polyketide | 24.0 | 71.4 | 92.0 | 7.79e-19 |
| ATV82114.1 | GMC\_oxidoreductase/oxidase/dehydrogenase | BGC0001909 | Polyketide | 24.0 | 73.1 | 92.0 | 1.09e-18 |
| AAS90066.1 | VBS | BGC0000009 | Polyketide | 26.0 | 71.8 | 91.0 | 2.02e-18 |
| AVT42378.1 | glucose-methanol-choline\_oxidoreductase | BGC0001476 | Other:Nucleoside | 27.0 | 69.6 | 89.0 | 4.48e-18 |
| AJI44177.1 | glucose-methanol-choline\_oxidoreductase | BGC0001193 | NRP | 26.0 | 65.3 | 89.0 | 5.48e-18 |
| ACH72898.1 | AflK | BGC0000011 | Polyketide | 25.0 | 73.3 | 86.0 | 9.75e-17 |
| AEF33092.1 | choline\_dehydrogenase | BGC0001039 | NRP+Polyketide | 26.0 | 67.6 | 84.0 | 2.11e-16 |
| EHK18384.1 | hypothetical\_protein | BGC0002216 | Terpene | 24.0 | 75.0 | 81.0 | 1.88e-15 |
| KNA98285.1 | hypothetical\_protein | BGC0002670 | Other | 22.0 | 70.3 | 80.0 | 4.45e-15 |
| KAF7526514.1 | hypothetical\_protein | BGC0002244 | Polyketide | 25.0 | 71.8 | 79.0 | 9.5e-15 |
| CBF83141.1 | conserved\_hypothetical\_protein | BGC0001722 | Polyketide | 23.0 | 72.9 | 79.0 | 1.03e-14 |
| BAQ25461.1 | putative\_dehydrogenase | BGC0001264 | Polyketide | 25.0 | 76.7 | 78.0 | 1.79e-14 |
| EEP98515.1 | Glucose-methanol-choline\_oxidoreductase | BGC0002091 | NRP | 23.0 | 66.4 | 75.0 | 1.4e-13 |
| KDM89831.1 | glucose-methanol-choline\_oxidoreductase | BGC0002412 | NRP | 23.0 | 66.5 | 75.0 | 1.45e-13 |
| AET51867.1 | oxidoreductase | BGC0001138 | Other:Nucleoside | 26.0 | 66.7 | 72.0 | 9.69e-13 |
| MCB8905710.1 | GMC\_family\_oxidoreductase\_N-terminal\_domain-containing\_protein | BGC0002340 | NRP+Other | 26.0 | 66.5 | 71.0 | 3.06e-12 |
| ACA34720.1 | CtnD | BGC0000894 | Other | 24.0 | 75.6 | 66.0 | 1.25e-10 |
| ALI92648.1 | CitC\_oxidoreductase | BGC0001338 | Polyketide:Iterative type I polyketide | 24.0 | 75.6 | 66.0 | 1.25e-10 |
| EFG10343.1 | Glucose-methanol-choline\_oxidoreductase | BGC0000373 | NRP | 23.0 | 69.3 | 64.0 | 3.45e-10 |
| AIG62134.1 | patulin\_synthase | BGC0000120 | Polyketide:Iterative type I polyketide | 22.0 | 73.3 | 64.0 | 4.98e-10 |
| CAM56763.1 | hypothetical\_protein | BGC0000354 | NRP | 26.0 | 34.3 | 64.0 | 5.06e-10 |
| FAA01296.1 | glucose-methanol-choline\_family\_oxidoreductase\_PyvF | BGC0002210 | Polyketide+NRP | 22.0 | 70.7 | 61.0 | 3.42e-09 |
| ACH85567.1 | CetG | BGC0000283 | Other:Cyclitol | 24.0 | 58.7 | 59.0 | 1.04e-08 |
| AGK82821.1 | alcohol\_oxidase | BGC0001324 | Terpene | 21.0 | 68.8 | 58.0 | 3.35e-08 |
| AGK82806.1 | alcohol\_oxidase | BGC0001322 | Terpene | 21.0 | 68.8 | 57.0 | 5.8e-08 |
| BBD84647.1 | putative\_GMC\_oxidoreductase | BGC0001775 | Terpene | 24.0 | 71.5 | 56.0 | 1.55e-07 |
| AGK82832.1 | alcohol\_oxidase | BGC0001321 | Terpene | 23.0 | 32.9 | 56.0 | 2.28e-07 |
| AGK82825.1 | alcohol\_oxidase | BGC0001323 | Terpene | 23.0 | 32.9 | 56.0 | 2.28e-07 |
