# Supplementary material for: Alkaliphilic/Alkali-Tolerant Fungi: Molecular, Biochemical, and Biotechnological Aspects
Source: J Fungi (Basel). 2023 Jun 9;9(6):652. doi: 10.3390/jof9060652 (PMC10301932; doi:10.3390/jof9060652)
Supplement: Supplementary file 1 [file jof-09-00652-s001.zip › S2/knownclusterblast/region1/input.path1.gene13_mibig_hits.html]

| MIBiG Protein | Description | MIBiG Cluster | MiBiG Product | % ID | % Coverage | BLAST Score | E-value |
| --- | --- | --- | --- | --- | --- | --- | --- |
| AAK33071.1 | trichothecene\_efflux\_pump | BGC0001278 | Terpene | 27.0 | 37.7 | 204.0 | 3.73e-55 |
| AAK33081.1 | trichothecene\_efflux\_pump | BGC0001277 | Terpene | 29.0 | 38.1 | 192.0 | 5.84e-51 |
| BAX01966.1 | trichothecene\_efflux\_pump | BGC0001811 | Terpene | 28.0 | 38.3 | 176.0 | 6.39e-46 |
| AAK53581.1 | trichothecene\_efflux\_pump | BGC0000930 | Terpene | 28.0 | 38.3 | 175.0 | 1.55e-45 |
| ATU31811.1 | MFS\_transporter | BGC0001814 | NRP | 25.0 | 27.4 | 87.0 | 4.38e-17 |
| ADG86335.1 | transporter | BGC0000190 | Polyketide | 26.0 | 26.6 | 84.0 | 4.84e-16 |
| AAQ08935.1 | putative\_membrane\_transporter | BGC0000224 | Polyketide:Type II polyketide | 26.0 | 26.5 | 77.0 | 5.95e-14 |
| QEU90619.1 | MFS\_transporter | BGC0000703 | Saccharide | 26.0 | 20.8 | 75.0 | 4.73e-13 |
| CAF60521.1 | putative\_efflux\_protein | BGC0000704 | Saccharide | 26.0 | 20.8 | 75.0 | 4.73e-13 |
| CAF31575.1 | putative\_kanamycin\_efflux\_protein | BGC0000705 | Saccharide | 26.0 | 20.8 | 75.0 | 4.73e-13 |
| UMP03494.1 | NmvH | BGC0002649 | NRP+Polyketide | 24.0 | 27.9 | 68.0 | 6.19e-11 |
| PPT15949.1 | MFS\_transporter | BGC0001468 | Other:tRNA-derived | 24.0 | 26.9 | 65.0 | 4.79e-10 |
| WP\_016640227.1 | DHA2\_family\_efflux\_MFS\_transporter\_permease\_subunit | BGC0002000 | Polyketide | 24.0 | 28.3 | 54.0 | 8.78e-07 |
