# Supplementary material for: Alkaliphilic/Alkali-Tolerant Fungi: Molecular, Biochemical, and Biotechnological Aspects
Source: J Fungi (Basel). 2023 Jun 9;9(6):652. doi: 10.3390/jof9060652 (PMC10301932; doi:10.3390/jof9060652)
Supplement: Supplementary file 1 [file jof-09-00652-s001.zip › S2/knownclusterblast/region1/input.path1.gene14_mibig_hits.html]

| MIBiG Protein | Description | MIBiG Cluster | MiBiG Product | % ID | % Coverage | BLAST Score | E-value |
| --- | --- | --- | --- | --- | --- | --- | --- |
| AAK53485.1 | cystathionine\_beta-lyase | BGC0000774 | Saccharide:Lipopolysaccharide | 38.0 | 29.6 | 194.0 | 2.71e-53 |
| EIN09545.1 | cystathionine\_beta-synthase | BGC0002213 | Polyketide | 37.0 | 32.0 | 173.0 | 1.07e-46 |
| QRN75750.1 | 3-Aminoalanin\_synthase | BGC0002114 | NRP+Polyketide | 33.0 | 31.1 | 158.0 | 4.17e-42 |
| SQF72445.1 | Siderophore\_staphylobactin\_biosynthesis\_protein\_SbnA | BGC0000943 | Other | 30.0 | 30.6 | 147.0 | 1.78e-38 |
| BAC73327.1 | putative\_cysteine\_synthase | BGC0001587 | Other | 32.0 | 30.5 | 139.0 | 1.54e-35 |
| AFV25494.1 | hypothetical\_protein | BGC0000469 | RiPP:Bottromycin | 34.0 | 28.3 | 137.0 | 2.31e-35 |
| BAI70378.1 | cysteine\_synthase | BGC0000896 | Other | 34.0 | 28.0 | 138.0 | 2.54e-35 |
| CBG67729.1 | putative\_cystathionine\_beta-synthase | BGC0002568 | Other | 35.0 | 31.1 | 136.0 | 2.16e-34 |
| QMX85610.1 | SapC | BGC0002510 | Polyketide | 31.0 | 27.7 | 119.0 | 1.2e-28 |
| QRG35018.1 | 2,3-diaminopropionate\_biosynthesis\_protein\_SbnA | BGC0002378 | NRP | 30.0 | 27.7 | 115.0 | 1.82e-27 |
| AQX14447.1 | SbnA | BGC0001671 | NRP | 28.0 | 28.1 | 108.0 | 3.33e-25 |
| AOZ21315.1 | SulH | BGC0001790 | NRP | 27.0 | 27.7 | 97.0 | 2.98e-21 |
