# Supplementary material for: Alkaliphilic/Alkali-Tolerant Fungi: Molecular, Biochemical, and Biotechnological Aspects
Source: J Fungi (Basel). 2023 Jun 9;9(6):652. doi: 10.3390/jof9060652 (PMC10301932; doi:10.3390/jof9060652)
Supplement: Supplementary file 1 [file jof-09-00652-s001.zip › S2/knownclusterblast/region1/input.path1.gene15_mibig_hits.html]

| MIBiG Protein | Description | MIBiG Cluster | MiBiG Product | % ID | % Coverage | BLAST Score | E-value |
| --- | --- | --- | --- | --- | --- | --- | --- |
| FAA01291.1 | polyketide\_synthase-nonribosomal\_peptide\_synthetase\_PyvA | BGC0002210 | Polyketide+NRP | 30.0 | 108.0 | 1360.0 | 0.0 |
| AAT28740.1 | FUSS | BGC0000064 | NRP+Polyketide | 29.0 | 106.2 | 1148.0 | 0.0 |
| AFP73394.1 | FusA | BGC0001268 | NRP+Polyketide | 28.0 | 105.5 | 1139.0 | 0.0 |
| QJY30853.1 | PKS-NRPS\_hybrid\_protein | BGC0002539 | Alkaloid | 25.0 | 103.6 | 784.0 | 7.7e-229 |
| EHA19289.1 | hypothetical\_protein | BGC0001124 | Polyketide | 34.0 | 43.6 | 716.0 | 5.47e-212 |
| BCA42568.1 | polyketide\_synthase\_GrgA | BGC0002185 | Polyketide | 43.0 | 27.2 | 691.0 | 4.22e-203 |
| ACS68554.1 | hybrid\_PKS-NRPS\_protein | BGC0001026 | NRP+Polyketide | 39.0 | 30.3 | 682.0 | 5.23e-197 |
| CCT72377.1 | probable\_polyketide\_synthase | BGC0001305 | Polyketide | 41.0 | 27.7 | 672.0 | 1.03e-196 |
| BAQ25466.1 | polyketide\_synthase | BGC0001264 | Polyketide | 39.0 | 29.3 | 672.0 | 4.56e-195 |
| ATZ45182.1 | Bcboa6 | BGC0001892 | Polyketide | 40.0 | 27.1 | 663.0 | 5.38e-194 |
| AEO57481.1 | PKS-NRPSs | BGC0001449 | NRP+Alkaloid+Polyketide:Iterative type I polyketide | 41.0 | 27.2 | 670.0 | 4.3e-193 |
| BBQ09587.1 | PKS-NRPS\_hybrid | BGC0002261 | Polyketide | 40.0 | 28.9 | 664.0 | 2.51e-191 |
| EAW09117.1 | hybrid\_NRPS/PKS\_enzyme,\_putative | BGC0000983 | NRP+Polyketide:Iterative type I polyketide | 41.0 | 28.8 | 664.0 | 3.64e-191 |
| KKP04599.1 | Non-ribosomal\_peptide\_synthetase\_-\_Polyketide\_synthase | BGC0002066 | NRP+Polyketide:Iterative type I polyketide | 39.0 | 27.1 | 655.0 | 1.03e-188 |
| OJJ98486.1 | hypothetical\_protein | BGC0002169 | Polyketide+NRP | 41.0 | 26.8 | 645.0 | 2.14e-188 |
| AKL78824.1 | GLPKS3 | BGC0001187 | NRP:Lipopeptide+Polyketide:Iterative type I polyketide | 39.0 | 26.9 | 650.0 | 7.21e-188 |
| ADN43685.1 | PKS-NRPS | BGC0001136 | NRP+Polyketide:Iterative type I polyketide | 37.0 | 30.4 | 652.0 | 1.83e-187 |
| EPS29069.1 | hypothetical\_protein | BGC0001724 | NRP+Polyketide | 40.0 | 28.2 | 646.0 | 1.01e-185 |
| iliA |  | BGC0002035 | NRP+Polyketide | 39.0 | 28.3 | 645.0 | 2.22e-185 |
| QPC57090.1 | polyketide\_synthase-nonribosomal\_peptide\_synthetase | BGC0002230 | Polyketide+NRP | 39.0 | 28.1 | 645.0 | 2.22e-185 |
| CAL69597.1 | PKS-NRPS | BGC0001049 | NRP+Polyketide:Iterative type I polyketide | 34.0 | 36.0 | 644.0 | 5.7e-185 |
| AAS98783.1 | polyketide\_synthase/nonribosomal\_peptide\_synthase\_hybrid | BGC0001001 | NRP+Polyketide | 25.0 | 84.1 | 643.0 | 6.08e-185 |
| EED49862.1 | hybrid\_PKS/NRPS\_enzyme,\_putative | BGC0001445 | NRP+Polyketide:Iterative type I polyketide | 40.0 | 27.3 | 642.0 | 1.06e-184 |
| QHD43130.1 | NRPS/PKS\_hybrid\_protein | BGC0002546 | NRP+Polyketide | 42.0 | 26.5 | 642.0 | 1.95e-184 |
| QBQ83704.1 | polyketide\_synthase-nonribosomal\_peptide\_synthetase | BGC0002093 | Polyketide+NRP | 38.0 | 29.2 | 639.0 | 9.78e-184 |
| EAU38971.1 | PKS-NRPS\_hybrid | BGC0001122 | NRP+Polyketide:Iterative type I polyketide | 39.0 | 28.1 | 639.0 | 1.53e-183 |
| CBF80487.1 | hybrid\_PKS-NRPS\_(Eurofung) | BGC0000959 | NRP+Polyketide:Iterative type I polyketide | 39.0 | 27.3 | 638.0 | 2.72e-183 |
| EHA55860.1 | polyketide\_synthase/peptide\_synthetase | BGC0002235 | Polyketide+NRP | 40.0 | 26.6 | 635.0 | 2.58e-182 |
| AGO86662.1 | equisetin\_synthetase | BGC0001255 | NRP+Polyketide | 40.0 | 27.1 | 634.0 | 5.47e-182 |
| BAZ95823.1 | PKS-NRPS\_hybrid\_cpaA | BGC0001563 | NRP+Polyketide | 39.0 | 28.1 | 633.0 | 8.85e-182 |
| EPS34234.1 | nonribosomal\_peptide\_synthatase-polyketide\_synthase | BGC0002067 | NRP+Polyketide:Iterative type I polyketide | 39.0 | 27.2 | 632.0 | 1.26e-181 |
| ARP51711.1 | PKS-NRPS\_hybrid\_protein | BGC0001741 | NRP+Polyketide | 40.0 | 27.5 | 630.0 | 5.11e-181 |
| KKP00963.1 | fatty\_acid\_synthase\_S-acetyltransferase | BGC0001854 | Polyketide:Iterative type I polyketide | 38.0 | 28.9 | 622.0 | 5.25e-181 |
| AZZ09613.1 | PvhA | BGC0002304 | Polyketide+NRP | 39.0 | 27.0 | 630.0 | 8.72e-181 |
| EAT85332.2 | hypothetical\_protein | BGC0002165 | Polyketide | 39.0 | 27.8 | 624.0 | 3.13e-180 |
| ABA02239.1 | polyketide\_synthase | BGC0000098 | Polyketide | 37.0 | 29.7 | 625.0 | 4.95e-180 |
| EDU47082.1 | lovastatin\_nonaketide\_synthase | BGC0002250 | Polyketide+NRP | 40.0 | 27.1 | 627.0 | 6.85e-180 |
| AKC54422.1 | fumosorinone\_biosynthesis\_polyketide\_synthase | BGC0001218 | NRP+Polyketide | 37.0 | 31.2 | 626.0 | 1.08e-179 |
| KAF5858310.1 | HR-PKS | BGC0002139 | Polyketide | 39.0 | 29.1 | 619.0 | 1.97e-179 |
| QOJ72663.1 | XenE | BGC0002505 | Polyketide+NRP | 38.0 | 27.0 | 625.0 | 2.22e-179 |
| EHA55875.1 | polyketide\_synthase | BGC0002235 | Polyketide+NRP | 40.0 | 27.0 | 625.0 | 2.4e-179 |
| QBK15049.1 | PKS-NRPS\_hybrid\_TraA | BGC0002197 | Polyketide+NRP | 39.0 | 27.2 | 625.0 | 2.62e-179 |
| BAJ14522.1 | polyketide\_synthase | BGC0001254 | Polyketide | 37.0 | 27.1 | 617.0 | 3.46e-179 |
| EWG54266.1 | hypothetical\_protein | BGC0001190 | Polyketide | 37.0 | 29.9 | 615.0 | 1.19e-178 |
| EHA52508.1 | mycocerosic\_acid\_synthase | BGC0001749 | Polyketide | 39.0 | 27.8 | 616.0 | 1.69e-178 |
| AQW44889.1 | polyketide\_synthase | BGC0001737 | NRP+Polyketide | 28.0 | 61.7 | 609.0 | 4.26e-178 |
| EAA36364.1 | polyketide\_synthase\_6 | BGC0002729 | Polyketide | 37.0 | 30.8 | 612.0 | 8.93e-178 |
| CAO91861.1 | PKS-NRPS\_hybrid | BGC0000968 | NRP+Polyketide:Iterative type I polyketide | 37.0 | 28.4 | 619.0 | 1.31e-177 |
| BAV32159.1 | polyketide\_synthase | BGC0001373 | Polyketide | 37.0 | 28.4 | 616.0 | 2.9e-177 |
| ctg1\_orf0002 |  | BGC0001068 | Terpene+Polyketide | 38.0 | 27.1 | 615.0 | 3.66e-177 |
| BAK26562.1 | PKS-NRPS\_hybrid | BGC0000977 | NRP+Polyketide | 37.0 | 27.2 | 617.0 | 8.14e-177 |
| AFA26384.1 | polyketide\_synthase\_A | BGC0001874 | NRP+Polyketide | 37.0 | 27.2 | 616.0 | 1.07e-176 |
| QOG08944.1 | FfsA | BGC0002204 | Polyketide+NRP | 37.0 | 30.0 | 616.0 | 1.17e-176 |
| QQW45467.1 | polyketide\_synthase\_CalA' | BGC0002168 | Polyketide | 37.0 | 29.0 | 609.0 | 2.06e-175 |
| BBM05082.1 | polyketide\_synthase | BGC0002170 | Polyketide | 37.0 | 29.0 | 609.0 | 2.06e-175 |
| CEF75886.1 |  | BGC0001600 | Polyketide | 38.0 | 27.8 | 606.0 | 5.9e-175 |
| XP\_659388.1 | hypothetical\_protein | BGC0001998 | Polyketide | 38.0 | 28.4 | 604.0 | 8.8e-175 |
| AAV66110.2 | fusaridione\_A\_synthetase | BGC0000992 | NRP+Polyketide | 38.0 | 29.0 | 609.0 | 1.97e-174 |
| ASK38717.1 | polyketide\_synthase | BGC0001436 | Polyketide:Iterative type I polyketide | 37.0 | 29.1 | 602.0 | 2.18e-174 |
| BBC43184.1 | PKS-NRPS\_hybrid | BGC0001738 | NRP+Polyketide | 40.0 | 27.0 | 608.0 | 3.29e-174 |
| AVI26390.1 | polyketide\_synthase\_/\_nonribosomal\_peptide\_synthase\_hybrid | BGC0001800 | NRP+Polyketide | 25.0 | 93.4 | 606.0 | 1.86e-173 |
| BBU42026.1 | putative\_polyketide\_synthase | BGC0002222 | Polyketide | 36.0 | 29.8 | 601.0 | 1.96e-173 |
| QTE75992.1 | ZopPKS | BGC0002224 | Polyketide | 36.0 | 29.8 | 601.0 | 1.96e-173 |
| BAC20564.1 | polyketide\_synthase | BGC0000039 | Polyketide | 36.0 | 29.7 | 603.0 | 2.16e-173 |
| QBC19710.1 | TwmB | BGC0001954 | NRP+Polyketide | 38.0 | 28.3 | 602.0 | 7.17e-173 |
| QCS37521.1 | pyiS | BGC0001881 | NRP+Polyketide:Iterative type I polyketide | 39.0 | 27.8 | 602.0 | 3.51e-172 |
| EAL89230.2 | LovB-like\_polyketide\_synthase,\_putative | BGC0000129 | Polyketide | 37.0 | 28.9 | 593.0 | 1.42e-171 |
| OQD69647.1 | hypothetical\_protein | BGC0002745 | Polyketide | 35.0 | 32.5 | 594.0 | 1.6e-171 |
| GAW21479.1 | hypothetical\_protein | BGC0002192 | Polyketide | 36.0 | 30.5 | 597.0 | 2.84e-171 |
| ANF07288.1 | hrPKS | BGC0001340 | Polyketide:Iterative type I polyketide | 35.0 | 30.8 | 593.0 | 4.03e-171 |
| KGO40478.1 | Acyl\_transferase/acyl\_hydrolase/lysophospholipase | BGC0001205 | Polyketide | 38.0 | 27.5 | 590.0 | 4.92e-171 |
| AMJ52080.1 | lijA | BGC0002255 | Polyketide | 37.0 | 28.6 | 592.0 | 1.16e-170 |
| BAC20566.1 | polyketide\_synthase | BGC0000039 | Polyketide | 37.0 | 29.3 | 591.0 | 1.97e-170 |
| QXF14600.1 | PydA | BGC0002239 | Polyketide+NRP | 38.0 | 27.1 | 595.0 | 2.85e-170 |
| APZ78742.1 | polyketide\_synthase | BGC0001422 | NRP:Cyclic depsipeptide+Polyketide:Iterative type I polyketide | 27.0 | 61.7 | 585.0 | 3.66e-170 |
| AGC45624.1 | polyketide\_synthase | BGC0001394 | NRP+Polyketide | 28.0 | 60.4 | 584.0 | 6.58e-170 |
| QBE85649.1 | BuaA | BGC0001857 | Alkaloid+NRP+Polyketide:Iterative type I polyketide | 39.0 | 26.2 | 594.0 | 7.53e-170 |
| CBF87072.1 | polyketide\_synthase,\_putative\_(Eurofung) | BGC0001290 | NRP | 37.0 | 28.9 | 586.0 | 6.21e-169 |
| CAQ18830.1 | polyketide\_synthase | BGC0000954 | NRP+Polyketide:Modular type I polyketide | 27.0 | 62.3 | 578.0 | 1.1e-167 |
| QNH68024.1 | PfpA | BGC0002268 | Polyketide+NRP | 38.0 | 27.2 | 585.0 | 3.89e-167 |
| CAP95405.1 |  | BGC0001404 | Polyketide | 37.0 | 27.8 | 580.0 | 4.39e-167 |
| ESU15174.1 | hypothetical\_protein | BGC0002186 | NRP+Polyketide | 35.0 | 30.0 | 580.0 | 6.44e-167 |
| OSS48297.1 | hypothetical\_protein | BGC0002194 | Polyketide | 36.0 | 29.0 | 579.0 | 1.01e-166 |
| EGX96624.1 | polyketide\_synthase,\_putative | BGC0002259 | Polyketide+NRP | 38.0 | 28.8 | 577.0 | 3.77e-166 |
| OPB37944.1 | putative\_polyketide\_synthase | BGC0002206 | Polyketide | 35.0 | 30.6 | 577.0 | 6.83e-166 |
| XP\_028481819.1 | highly-reducing\_poluketide\_synthase | BGC0001866 | Polyketide | 27.0 | 62.9 | 572.0 | 7.91e-166 |
| BAD83684.1 | PKSN\_polyketide\_synthase\_for\_alternapyrone\_biosynthesis | BGC0000012 | Polyketide | 35.0 | 30.5 | 575.0 | 1.83e-165 |
| AHV78245.1 | LasS1 | BGC0001245 | Polyketide | 37.0 | 27.6 | 572.0 | 3.97e-165 |
| EYE95336.1 | polyketide\_synthase | BGC0002234 | Polyketide | 38.0 | 27.0 | 569.0 | 5.64e-164 |
| EAL85113.2 | hybrid\_PKS-NRPS\_enzyme | BGC0001037 | NRP+Polyketide:Iterative type I polyketide | 37.0 | 27.6 | 574.0 | 7.48e-164 |
| BAE61265.1 |  | BGC0002238 | Polyketide | 37.0 | 27.9 | 568.0 | 9.18e-164 |
| CBF82304.1 | polyketide\_synthase,\_putative\_(JCVI) | BGC0002180 | Polyketide | 35.0 | 30.0 | 567.0 | 2.66e-163 |
| KAF9708863.1 | hypothetical\_protein | BGC0002515 | Polyketide | 35.0 | 30.6 | 568.0 | 4.28e-163 |
| ADB12491.1 | EpoD | BGC0000990 | NRP+Polyketide | 26.0 | 61.7 | 571.0 | 1.64e-162 |
| QVV57687.1 | polyketide\_synthase | BGC0002338 | Polyketide | 27.0 | 61.6 | 569.0 | 2.12e-162 |
| AIT55262.1 | polyketide\_synthase | BGC0000072 | Polyketide:Modular type I polyketide | 38.0 | 23.5 | 536.0 | 2.58e-162 |
| AQM37582.1 | polyketide\_synthase | BGC0001424 | NRP:Cyclic depsipeptide+Polyketide:Iterative type I polyketide | 26.0 | 61.4 | 560.0 | 2.71e-162 |
| BAJ09789.1 | polyketide\_synthase | BGC0000146 | Polyketide | 34.0 | 31.2 | 565.0 | 3.37e-162 |
| AAF62883.1 | epoD | BGC0000991 | NRP+Polyketide | 26.0 | 61.0 | 569.0 | 4.82e-162 |
| AAD34559.1 | polyketide\_synthase | BGC0000088 | Polyketide | 37.0 | 28.0 | 564.0 | 5.51e-162 |
| EAQ86385.1 | hypothetical\_protein | BGC0001405 | Polyketide | 38.0 | 25.4 | 562.0 | 5.88e-162 |
| APZ78727.1 | polyketide\_synthase | BGC0001421 | NRP:Cyclic depsipeptide+Polyketide:Iterative type I polyketide | 27.0 | 61.5 | 558.0 | 1.14e-161 |
| ACD39758.1 | reducing\_polyketide\_synthase | BGC0000076 | Polyketide | 36.0 | 28.2 | 560.0 | 1.83e-161 |
| ACD39767.1 | reducing\_polyketide\_synthase | BGC0000077 | Polyketide | 36.0 | 28.2 | 560.0 | 1.83e-161 |
| ABA02240.1 | polyketide\_synthase | BGC0000098 | Polyketide | 35.0 | 28.5 | 562.0 | 1.85e-161 |
| MBV7329454.1 | type\_I\_polyketide\_synthase | BGC0002131 | Polyketide+NRP:Glycopeptide+Saccharide:Hybrid/tailoring saccharide | 37.0 | 27.8 | 553.0 | 2.15e-161 |
| ACB46195.1 | polyketide\_synthase | BGC0000989 | NRP+Polyketide | 27.0 | 61.8 | 566.0 | 4.16e-161 |
| KFG78606.1 | polyketide\_synthase | BGC0002240 | Polyketide | 37.0 | 27.1 | 564.0 | 4.35e-161 |
| EAU31921.1 | hypothetical\_protein | BGC0002267 | Polyketide | 35.0 | 29.6 | 561.0 | 8.13e-161 |
| KFH44396.1 | Lovastatin\_nonaketide\_synthase-like\_protein | BGC0002190 | Polyketide | 35.0 | 30.2 | 560.0 | 1.01e-160 |
| EJP62832.1 | polyketide\_synthase,\_putative | BGC0002203 | NRP+Polyketide+Other | 36.0 | 28.2 | 560.0 | 1.13e-160 |
| APZ78780.1 | polyketide\_synthase | BGC0001426 | NRP:Cyclic depsipeptide+Polyketide:Iterative type I polyketide | 26.0 | 60.3 | 555.0 | 2.05e-160 |
| AAF26921.1 | polyketide\_synthase | BGC0000988 | NRP+Polyketide | 26.0 | 61.4 | 563.0 | 2.74e-160 |
| APZ78793.1 | polyketide\_synthase | BGC0001427 | NRP:Cyclic depsipeptide+Polyketide:Iterative type I polyketide | 26.0 | 60.8 | 554.0 | 2.93e-160 |
| APZ78807.1 | polyketide\_synthase | BGC0001428 | NRP:Cyclic depsipeptide+Polyketide:Iterative type I polyketide | 26.0 | 62.2 | 553.0 | 6.44e-160 |
| BBI47418.1 | polyketide\_synthase | BGC0002258 | Polyketide | 36.0 | 28.1 | 556.0 | 2.09e-159 |
| CAJ46690.1 | polyketide\_synthase | BGC0000969 | NRP:Cyclic depsipeptide+Polyketide:Modular type I polyketide | 26.0 | 62.4 | 552.0 | 2.43e-159 |
| APZ78754.1 | polyketide\_synthase | BGC0001423 | NRP:Cyclic depsipeptide+Polyketide:Iterative type I polyketide | 26.0 | 61.5 | 550.0 | 4.73e-159 |
| EHA28244.1 | hypothetical\_protein | BGC0001143 | Polyketide | 35.0 | 27.5 | 554.0 | 6.04e-159 |
| CAD19091.1 | StiG\_protein | BGC0000153 | NRP+Polyketide:Modular type I polyketide | 35.0 | 29.7 | 534.0 | 1.12e-158 |
| EAT91803.2 | hypothetical\_protein | BGC0002205 | Polyketide+NRP | 36.0 | 29.1 | 557.0 | 1.46e-158 |
| QJX57338.1 | ChaA | BGC0002538 | Polyketide | 35.0 | 28.9 | 556.0 | 2.41e-158 |
| ACD39774.1 | reducing\_polyketide\_synthase | BGC0000134 | Polyketide | 35.0 | 26.9 | 550.0 | 3.71e-158 |
| AHV78252.1 | ResS1 | BGC0001246 | Polyketide | 36.0 | 27.6 | 550.0 | 5.56e-158 |
| EAA65604.1 | hypothetical\_protein | BGC0000022 | Polyketide | 35.0 | 28.8 | 551.0 | 7.2e-158 |
| ACZ57548.1 | polyketide\_synthase | BGC0000046 | Polyketide:Iterative type I polyketide | 36.0 | 28.1 | 548.0 | 2.53e-157 |
| CEN60541.1 | hypothetical\_protein | BGC0002266 | Terpene+Polyketide | 35.0 | 29.1 | 546.0 | 6.89e-157 |
| KZL86691.1 | polyketide\_synthase | BGC0002228 | NRP | 37.0 | 26.3 | 546.0 | 1.06e-156 |
| AMY15057.1 | tetraketide\_synthase\_MF-SQTKS | BGC0001339 | Polyketide:Iterative type I polyketide | 35.0 | 28.3 | 547.0 | 1.33e-156 |
| ACB12550.1 | Fum1 | BGC0000063 | Polyketide | 35.0 | 28.1 | 545.0 | 4.79e-156 |
| OAQ63050.2 | polyketide\_synthase | BGC0002187 | Polyketide | 35.0 | 26.8 | 543.0 | 7.8e-156 |
| AGC95324.1 | CurS1 | BGC0000045 | Polyketide | 35.0 | 28.3 | 542.0 | 1.41e-155 |
| BBG28498.1 | putative\_polyketide\_synthase | BGC0001913 | Polyketide | 35.0 | 29.0 | 542.0 | 4.19e-155 |
| CBX99534.1 | similar\_to\_polyketide\_synthase | BGC0001899 | Polyketide | 34.0 | 28.5 | 542.0 | 5.46e-155 |
| gene4 |  | BGC0001907 | Polyketide | 34.0 | 28.9 | 536.0 | 9.96e-155 |
| APZ78767.1 | polyketide\_synthase | BGC0001425 | NRP:Cyclic depsipeptide+Polyketide:Iterative type I polyketide | 26.0 | 61.0 | 537.0 | 1.05e-154 |
| CAD19090.1 | StiF\_protein | BGC0000153 | NRP+Polyketide:Modular type I polyketide | 27.0 | 61.5 | 537.0 | 1.46e-154 |
| AAD43562.2 | Fum1p | BGC0000062 | Polyketide | 33.0 | 30.3 | 540.0 | 2.37e-154 |
| ABB90283.1 | polyketide\_synthase | BGC0001057 | NRP+Polyketide | 36.0 | 26.2 | 538.0 | 3.06e-154 |
| AAK57186.1 | MxaB2 | BGC0001022 | NRP+Polyketide | 36.0 | 23.4 | 506.0 | 3.32e-154 |
| EHK18438.1 | putative\_polyketide\_synthase | BGC0002233 | Polyketide | 36.0 | 26.8 | 532.0 | 2e-152 |
| CCE88376.1 | polyketide\_synthase | BGC0001034 | NRP+Polyketide:Modular type I polyketide | 26.0 | 62.3 | 533.0 | 3.51e-151 |
| WP\_036342114.1 | type\_I\_polyketide\_synthase | BGC0001327 | NRP:Cyclic depsipeptide+Polyketide:Modular type I polyketide | 27.0 | 61.9 | 531.0 | 3.73e-151 |
| AEA35016.1 | hypothetical\_protein | BGC0002502 | Polyketide | 35.0 | 28.1 | 529.0 | 7.14e-151 |
| WP\_042799407.1 | SDR\_family\_NAD(P)-dependent\_oxidoreductase | BGC0001283 | Polyketide | 27.0 | 58.8 | 526.0 | 1.16e-150 |
| ATZ45185.1 | Bcboa9 | BGC0001892 | Polyketide | 29.0 | 45.3 | 526.0 | 1.68e-150 |
| BBG28484.1 | polyketide\_synthase\_CdmE | BGC0001926 | Polyketide | 34.0 | 29.6 | 528.0 | 1.72e-150 |
| QBK15047.1 | polyketide\_synthase\_ClaI | BGC0002196 | Polyketide | 35.0 | 27.2 | 525.0 | 2.48e-150 |
| CAQ43075.1 | polyketide\_synthase | BGC0000970 | NRP+Polyketide:Modular type I polyketide | 27.0 | 61.8 | 528.0 | 4.5e-150 |
| ABK32287.1 | JerA | BGC0000080 | Polyketide | 38.0 | 23.5 | 498.0 | 1.3e-149 |
| XP\_001220460.1 | uncharacterized\_protein | BGC0001182 | NRP+Polyketide:Iterative type I polyketide | 41.0 | 22.5 | 527.0 | 2.5e-149 |
| GAA85575.1 | polyketide\_synthase | BGC0002227 | NRP | 36.0 | 26.6 | 522.0 | 3.2e-149 |
| AIT55259.1 | polyketide\_synthase | BGC0000072 | Polyketide:Modular type I polyketide | 37.0 | 24.6 | 509.0 | 7.24e-149 |
| QGW49095.1 | putative\_polyketide\_synthase | BGC0002731 | Polyketide | 34.0 | 27.1 | 521.0 | 9.07e-149 |
| BBU37368.1 | polyketide\_synthase | BGC0002525 | Polyketide | 34.0 | 27.4 | 519.0 | 7.04e-148 |
| AAK57188.1 | MxaD | BGC0001022 | NRP+Polyketide | 35.0 | 26.4 | 511.0 | 9.63e-148 |
| AZH23788.1 | MgcR | BGC0001970 | NRP+Polyketide | 25.0 | 58.5 | 518.0 | 1.23e-147 |
| CBD77732.1 | polyketide\_synthase | BGC0000974 | NRP+Polyketide | 37.0 | 23.8 | 518.0 | 2.23e-147 |
| QCP68972.1 | VatE | BGC0002296 | NRP+Polyketide | 37.0 | 23.5 | 506.0 | 5.8e-147 |
| AGC45621.1 | polyketide\_synthase | BGC0001394 | NRP+Polyketide | 35.0 | 26.5 | 508.0 | 8.52e-147 |
| OAG05545.1 | PKSKA1\_protein | BGC0002211 | Polyketide | 35.0 | 27.4 | 514.0 | 9.27e-147 |
| ABK32255.1 | AmbA | BGC0000014 | Polyketide | 37.0 | 23.5 | 489.0 | 1.01e-146 |
| AZH23789.1 | MgcI | BGC0001970 | NRP+Polyketide | 26.0 | 58.1 | 511.0 | 1.93e-146 |
| AIA58899.1 | HRPKS | BGC0001141 | Polyketide:Iterative type I polyketide | 33.0 | 29.4 | 513.0 | 3e-146 |
| AQW44891.1 | polyketide\_synthase | BGC0001737 | NRP+Polyketide | 35.0 | 26.6 | 506.0 | 3.85e-146 |
| AQW44890.1 | polyketide\_synthase | BGC0001737 | NRP+Polyketide | 34.0 | 29.0 | 509.0 | 8.06e-146 |
| QCP68968.1 | VatM | BGC0002296 | NRP+Polyketide | 25.0 | 61.2 | 509.0 | 8.11e-146 |
| EAU29808.1 | hypothetical\_protein | BGC0001400 | Polyketide | 33.0 | 29.2 | 511.0 | 1.29e-145 |
| AIT55263.1 | polyketide\_synthase | BGC0000072 | Polyketide:Modular type I polyketide | 38.0 | 22.5 | 510.0 | 2.66e-145 |
| CCT75967.1 | polyketide\_synthase | BGC0001606 | Polyketide | 33.0 | 27.6 | 511.0 | 2.69e-145 |
| CAD19086.1 | StiB\_protein | BGC0000153 | NRP+Polyketide:Modular type I polyketide | 35.0 | 24.8 | 498.0 | 4.75e-145 |
| AKD43522.1 | Type\_I\_polyketide\_synthase | BGC0001409 | Polyketide | 26.0 | 61.8 | 507.0 | 5.38e-145 |
| AZH23818.1 | MgiI | BGC0001971 | NRP+Polyketide | 26.0 | 58.1 | 506.0 | 7.59e-145 |
| QCC63000.1 | BII-rafflesfungin\_polyketide\_synthase | BGC0001966 | NRP+Polyketide | 31.0 | 33.1 | 509.0 | 8.14e-145 |
| AEE88279.1 | CurK | BGC0000976 | NRP+Polyketide:Modular type I polyketide | 24.0 | 62.6 | 507.0 | 8.16e-145 |
| AAT70106.1 | CurK | BGC0001165 | NRP+Polyketide:Modular type I polyketide | 24.0 | 62.6 | 507.0 | 8.16e-145 |
| QDA77058.1 | polyketide\_synthase | BGC0002026 | NRP+Polyketide | 25.0 | 62.5 | 510.0 | 8.83e-145 |
| AZF85932.1 | type\_I\_polyketide\_synthase | BGC0001963 | NRP+Polyketide | 37.0 | 24.1 | 494.0 | 1.02e-144 |
| AAF19810.1 | MtaB | BGC0001024 | NRP+Polyketide:Modular type I polyketide | 35.0 | 26.7 | 511.0 | 1.67e-144 |
| AAZ77696.1 | ChlA3 | BGC0000036 | Polyketide:Modular type I polyketide+Polyketide:Iterative type I polyketide+Saccharide:Oligosaccharide | 26.0 | 62.0 | 511.0 | 1.86e-144 |
| AAW03328.1 | CtaE | BGC0000982 | NRP+Polyketide | 36.0 | 24.9 | 501.0 | 2.96e-144 |
| CAD19089.1 | StiE\_protein | BGC0000153 | NRP+Polyketide:Modular type I polyketide | 35.0 | 23.8 | 501.0 | 4.13e-144 |
| CAD89776.1 | MelE\_protein | BGC0001010 | NRP+Polyketide:Modular type I polyketide | 36.0 | 24.7 | 501.0 | 4.17e-144 |
| CCE88381.1 | polyketide\_synthase | BGC0001034 | NRP+Polyketide:Modular type I polyketide | 37.0 | 24.7 | 499.0 | 9.29e-144 |
| AAS98777.1 | polyketide\_synthetase | BGC0001001 | NRP+Polyketide | 36.0 | 23.4 | 496.0 | 1.02e-143 |
| WP\_053065267.1 | type\_I\_polyketide\_synthase | BGC0001330 | NRP:Cyclic depsipeptide+Polyketide:Modular type I polyketide | 37.0 | 23.6 | 503.0 | 1.48e-143 |
| EKJ70677.1 | PKS6 | BGC0002188 | NRP+Polyketide | 34.0 | 29.3 | 505.0 | 1.82e-143 |
| CAD19088.1 | StiD\_protein | BGC0000153 | NRP+Polyketide:Modular type I polyketide | 37.0 | 23.8 | 498.0 | 2.89e-143 |
| ADN13832.1 | Polyketide\_Synthase | BGC0001164 | Polyketide:Modular type I polyketide | 37.0 | 22.3 | 505.0 | 3.26e-143 |
| AQW44893.1 | polyketide\_synthase | BGC0001737 | NRP+Polyketide | 36.0 | 23.6 | 497.0 | 3.84e-143 |
| AMY15068.1 | hexaketide\_synthase\_MF-SQHKS | BGC0001339 | Polyketide:Iterative type I polyketide | 34.0 | 29.3 | 503.0 | 5.42e-143 |
| AAF26922.1 | polyketide\_synthase | BGC0000988 | NRP+Polyketide | 34.0 | 26.9 | 505.0 | 8.75e-143 |
| WP\_019032757.1 | type\_I\_polyketide\_synthase | BGC0001331 | NRP:Cyclic depsipeptide+Polyketide:Modular type I polyketide | 36.0 | 23.6 | 500.0 | 1.44e-142 |
| ACA99172.1 | polyketide\_synthase | BGC0001160 | Polyketide:Modular type I polyketide | 38.0 | 22.1 | 503.0 | 1.48e-142 |
| QLJ99331.2 | acyltransferase\_domain-containing\_protein | BGC0002088 | Polyketide+Saccharide:Oligosaccharide | 37.0 | 24.6 | 482.0 | 1.85e-142 |
| AAK57189.1 | MxaE | BGC0001022 | NRP+Polyketide | 36.0 | 24.4 | 494.0 | 3.13e-142 |
| AVI26388.1 | polyketide\_synthase | BGC0001800 | NRP+Polyketide | 27.0 | 58.7 | 501.0 | 5.94e-142 |
| ACB46196.1 | polyketide\_synthase | BGC0000989 | NRP+Polyketide | 34.0 | 26.9 | 501.0 | 9.92e-142 |
| ADB12492.1 | EpoE | BGC0000990 | NRP+Polyketide | 34.0 | 26.9 | 501.0 | 9.92e-142 |
| EFL02193.1 | amino\_acid\_adenylation\_domain-containing\_protein | BGC0000996 | NRP+Polyketide:Iterative type I polyketide | 36.0 | 26.9 | 500.0 | 1.23e-141 |
| AAF62884.1 | EpoE | BGC0000991 | NRP+Polyketide | 34.0 | 26.9 | 501.0 | 1.3e-141 |
| QLD23491.1 | Polyketide\_synthase | BGC0002085 | Saccharide:Oligosaccharide | 36.0 | 24.7 | 479.0 | 1.46e-141 |
| BAJ16468.1 | polyketide\_synthase | BGC0000058 | Polyketide | 26.0 | 64.2 | 501.0 | 1.52e-141 |
| CAD19085.1 | StiA\_protein | BGC0000153 | NRP+Polyketide:Modular type I polyketide | 26.0 | 63.0 | 498.0 | 1.56e-141 |
| ADZ24998.1 | polyketide\_synthase | BGC0000380 | NRP+Polyketide:Modular type I polyketide | 26.0 | 63.0 | 496.0 | 1.58e-141 |
| EAL85129.1 | polyketide\_synthase | BGC0001067 | Terpene+Polyketide:Iterative type I polyketide | 30.0 | 38.6 | 498.0 | 1.77e-141 |
| AAF19813.1 | MtaE | BGC0001024 | NRP+Polyketide:Modular type I polyketide | 35.0 | 24.5 | 493.0 | 1.89e-141 |
| WP\_018540604.1 | type\_I\_polyketide\_synthase | BGC0001332 | NRP+Polyketide | 36.0 | 22.5 | 474.0 | 1.98e-141 |
| AQW44892.1 | polyketide\_synthase | BGC0001737 | NRP+Polyketide | 34.0 | 26.7 | 491.0 | 2.89e-141 |
| EPE34340.1 | polyketide\_synthase | BGC0001035 | Polyketide+NRP | 32.0 | 29.5 | 498.0 | 3.04e-141 |
| ADF88280.1 | polyketide\_synthase | BGC0000981 | NRP+Polyketide | 37.0 | 22.4 | 491.0 | 4.21e-141 |
| CCE88380.1 | polyketide\_synthase | BGC0001034 | NRP+Polyketide:Modular type I polyketide | 33.0 | 29.6 | 491.0 | 4.58e-141 |
| CCE88378.1 | polyketide\_synthase | BGC0001034 | NRP+Polyketide:Modular type I polyketide | 25.0 | 62.4 | 496.0 | 5.16e-141 |
| AEE88282.1 | CurH | BGC0000976 | NRP+Polyketide:Modular type I polyketide | 26.0 | 62.3 | 494.0 | 5.23e-141 |
| AAT70103.1 | CurH | BGC0001165 | NRP+Polyketide:Modular type I polyketide | 26.0 | 62.3 | 494.0 | 5.23e-141 |
| AGC45620.1 | polyketide\_synthase | BGC0001394 | NRP+Polyketide | 33.0 | 29.4 | 499.0 | 7.24e-141 |
| AGC45622.1 | polyketide\_synthase | BGC0001394 | NRP+Polyketide | 33.0 | 29.4 | 489.0 | 1.25e-140 |
| ATV95616.1 | 6-methylsalicylic\_acid\_synthase | BGC0001503 | Polyketide | 36.0 | 26.6 | 487.0 | 1.25e-140 |
| CAQ18838.1 | polyketide\_synthase | BGC0000954 | NRP+Polyketide:Modular type I polyketide | 37.0 | 23.3 | 489.0 | 1.28e-140 |
| AQW44888.1 | polyketide\_synthase | BGC0001737 | NRP+Polyketide | 32.0 | 29.2 | 498.0 | 2.12e-140 |
| ABX60162.1 | polyketide\_synthase | BGC0000978 | NRP+Alkaloid+Polyketide:Modular type I polyketide | 37.0 | 22.4 | 489.0 | 2.36e-140 |
| ANI24099.1 | polyketide\_synthase | BGC0001235 | NRP+Polyketide | 35.0 | 27.4 | 497.0 | 3.29e-140 |
| QTE76000.1 | ScyPKS | BGC0002223 | Polyketide | 33.0 | 29.6 | 494.0 | 3.65e-140 |
| AVI26389.1 | polyketide\_synthase | BGC0001800 | NRP+Polyketide | 34.0 | 27.4 | 487.0 | 4.97e-140 |
| TXD00034.1 | SDR\_family\_NAD(P)-dependent\_oxidoreductase | BGC0001877 | Polyketide | 35.0 | 24.8 | 496.0 | 5.07e-140 |
| CAQ18834.1 | polyketide\_synthase | BGC0000954 | NRP+Polyketide:Modular type I polyketide | 37.0 | 23.8 | 495.0 | 5.15e-140 |
| CAO98847.1 | polyketide\_synthase\_AufC | BGC0000023 | Polyketide:Modular type I polyketide | 36.0 | 22.4 | 487.0 | 7.85e-140 |
| CAQ43076.1 | polyketide\_synthase | BGC0000970 | NRP+Polyketide:Modular type I polyketide | 33.0 | 27.2 | 488.0 | 8.01e-140 |
| AAK57187.1 | MxaC | BGC0001022 | NRP+Polyketide | 32.0 | 29.6 | 496.0 | 8.16e-140 |
| APZ78832.1 | polyketide\_synthase | BGC0001430 | NRP:Cyclic depsipeptide+Polyketide:Iterative type I polyketide | 34.0 | 28.2 | 490.0 | 9.03e-140 |
| AIW82279.1 | PuwB | BGC0001125 | NRP+Polyketide | 33.0 | 28.0 | 493.0 | 1.1e-139 |
| BAG17643.1 | putative\_NRPS-type-I\_PKS\_fusion\_protein | BGC0001043 | NRP+Polyketide | 35.0 | 27.0 | 493.0 | 2.52e-139 |
| CAQ18829.1 | polyketide\_synthase | BGC0000954 | NRP+Polyketide:Modular type I polyketide | 38.0 | 22.2 | 493.0 | 3.99e-139 |
| ADF88276.1 | polyketide\_synthase | BGC0000981 | NRP+Polyketide | 35.0 | 24.7 | 484.0 | 4.9e-139 |
| MCF2150415.1 | Polyketide\_synthase | BGC0002625 | NRP+Polyketide | 35.0 | 23.5 | 486.0 | 6.88e-139 |
| AIT55260.1 | polyketide\_synthase | BGC0000072 | Polyketide:Modular type I polyketide | 32.0 | 28.9 | 484.0 | 6.92e-139 |
| APD26279.1 | PtmA | BGC0001726 | NRP+Polyketide | 39.0 | 22.8 | 491.0 | 7.15e-139 |
| QNS30807.1 | hybrid\_non-ribosomal\_peptide\_synthetase/type\_I\_polyketide\_syn-thase | BGC0002509 | NRP | 35.0 | 27.0 | 491.0 | 7.5e-139 |
| APZ78854.1 | polyketide\_synthase | BGC0001432 | NRP:Cyclic depsipeptide+Polyketide:Iterative type I polyketide | 34.0 | 28.3 | 487.0 | 8.62e-139 |
| APZ78690.1 | polyketide\_synthase | BGC0001418 | NRP:Cyclic depsipeptide+Polyketide:Iterative type I polyketide | 34.0 | 28.3 | 487.0 | 8.68e-139 |
| APZ78714.1 | polyketide\_synthase | BGC0001420 | NRP:Cyclic depsipeptide+Polyketide:Iterative type I polyketide | 34.0 | 28.3 | 487.0 | 8.68e-139 |
| QBF51754.1 | type\_I\_polyketide\_synthase | BGC0001856 | Polyketide:Modular type I polyketide | 35.0 | 25.0 | 492.0 | 9.74e-139 |
| OAQ83765.1 | KR\_domain-containing\_protein | BGC0001358 | NRP+Polyketide | 25.0 | 66.7 | 488.0 | 1.32e-138 |
| CCE88379.1 | polyketide\_synthase | BGC0001034 | NRP+Polyketide:Modular type I polyketide | 34.0 | 26.7 | 483.0 | 1.63e-138 |
| AGC45619.1 | polyketide\_synthase | BGC0001394 | NRP+Polyketide | 33.0 | 28.8 | 486.0 | 1.72e-138 |
| CAG28678.1 | polyketide\_synthase | BGC0001023 | NRP+Polyketide:Modular type I polyketide | 33.0 | 28.3 | 486.0 | 2.67e-138 |
| APZ78820.1 | polyketide\_synthase | BGC0001429 | NRP:Cyclic depsipeptide+Polyketide:Iterative type I polyketide | 33.0 | 28.3 | 486.0 | 2.67e-138 |
| ARM20280.1 | polyketide\_synthase | BGC0001523 | Polyketide | 27.0 | 61.1 | 491.0 | 2.69e-138 |
| QVV57685.1 | malonyl\_CoA-acyl\_carrier\_protein\_transacylase | BGC0002338 | Polyketide | 36.0 | 22.2 | 482.0 | 3.58e-138 |
| BCK51644.1 | modular\_polyketide\_synthase | BGC0002520 | Polyketide | 26.0 | 61.5 | 489.0 | 4.12e-138 |
| ctg1\_13 |  | BGC0001931 | Polyketide | 27.0 | 60.8 | 489.0 | 4.24e-138 |
| APZ78702.1 | polyketide\_synthase | BGC0001419 | NRP:Cyclic depsipeptide+Polyketide:Iterative type I polyketide | 34.0 | 28.3 | 484.0 | 6.2e-138 |
| WP\_035121546.1 | type\_I\_polyketide\_synthase | BGC0001467 | NRP:Cyclic depsipeptide+Polyketide:Modular type I polyketide | 36.0 | 25.4 | 482.0 | 1.03e-137 |
| CBD77738.1 | polyketide\_synthase | BGC0000974 | NRP+Polyketide | 35.0 | 23.1 | 458.0 | 1.16e-137 |
| ABY66019.1 | 6-methylsalicylic\_acid\_synthase | BGC0001008 | Polyketide:Iterative type I polyketide+Polyketide:Enediyne type I polyketide | 37.0 | 23.6 | 478.0 | 1.43e-137 |
| AAF62885.1 | EpoF | BGC0000991 | NRP+Polyketide | 25.0 | 61.7 | 485.0 | 1.47e-137 |
| DAB41916.1 | ArzN\_-\_PKS\_(KS,\_AT,\_OMT,\_KR,\_ACP) | BGC0001884 | NRP+Polyketide | 37.0 | 22.8 | 480.0 | 1.74e-137 |
| AAM70355.1 | CalO5 | BGC0000033 | Polyketide | 35.0 | 24.7 | 467.0 | 2.24e-137 |
| QWM97862.1 | hybrid\_non-ribosomal\_peptide\_synthetase/type\_I\_polyketide\_synthase | BGC0002434 | Polyketide+NRP | 34.0 | 27.6 | 486.0 | 2.56e-137 |
| AEP40940.1 | polyketide\_synthase\_type\_I | BGC0000021 | Polyketide | 37.0 | 24.4 | 488.0 | 2.64e-137 |
| AXM42950.1 | polyketide\_synthase | BGC0001941 | NRP+Polyketide | 35.0 | 23.6 | 480.0 | 2.69e-137 |
| ESU09893.1 | hypothetical\_protein | BGC0002191 | Polyketide | 32.0 | 27.9 | 485.0 | 2.93e-137 |
| APZ78844.1 | polyketide\_synthase | BGC0001431 | NRP:Cyclic depsipeptide+Polyketide:Iterative type I polyketide | 33.0 | 28.5 | 482.0 | 3.34e-137 |
| DAB41915.1 | ArzM\_-\_PKS\_(KS,\_AT,\_DH,\_MT,\_ER,\_KR,\_ACP) | BGC0001884 | NRP+Polyketide | 37.0 | 22.8 | 484.0 | 4.38e-137 |
| ABX60152.1 | polyketide\_synthase | BGC0000978 | NRP+Alkaloid+Polyketide:Modular type I polyketide | 35.0 | 24.7 | 478.0 | 4.78e-137 |
| AFV96138.1 | polyketide\_synthase | BGC0001064 | Polyketide:Modular type I polyketide+Polyketide:Type III polyketide | 36.0 | 22.3 | 465.0 | 5.55e-137 |
| ARU81118.1 | CylD | BGC0001566 | Polyketide | 36.0 | 22.3 | 465.0 | 5.55e-137 |
| AUO16401.1 | polyketide\_synthase | BGC0001700 | Polyketide | 26.0 | 60.3 | 486.0 | 6.76e-137 |
| MBE8994630.1 | amino\_acid\_adenylation\_domain-containing\_protein | BGC0002623 | NRP+Polyketide | 36.0 | 23.0 | 484.0 | 7.43e-137 |
| ACB46197.1 | polyketide\_synthase | BGC0000989 | NRP+Polyketide | 25.0 | 61.5 | 483.0 | 1.01e-136 |
| ADB12493.1 | EpoF | BGC0000990 | NRP+Polyketide | 25.0 | 61.5 | 483.0 | 1.01e-136 |
| AGC45623.1 | polyketide\_synthase | BGC0001394 | NRP+Polyketide | 32.0 | 29.5 | 477.0 | 1.18e-136 |
| AP234\_RS37235 | type\_I\_polyketide\_synthase | BGC0001653 | Polyketide | 35.0 | 23.7 | 452.0 | 1.2e-136 |
| ABM21570.1 | crpB | BGC0000975 | NRP+Polyketide | 34.0 | 24.8 | 484.0 | 1.21e-136 |
| AHH34186.1 | polyketide\_synthase | BGC0001161 | Polyketide:Modular type I polyketide | 35.0 | 23.1 | 481.0 | 1.53e-136 |
| AAZ77673.1 | ChlB1 | BGC0000036 | Polyketide:Modular type I polyketide+Polyketide:Iterative type I polyketide+Saccharide:Oligosaccharide | 35.0 | 25.8 | 475.0 | 1.62e-136 |
| AEZ54375.1 | PieA2 | BGC0000124 | Polyketide | 35.0 | 23.1 | 484.0 | 1.96e-136 |
| QGU18619.1 | polyketide\_synthase/non-ribosomal\_peptide\_synthetase | BGC0002365 | Other+Polyketide | 37.0 | 22.6 | 483.0 | 2.14e-136 |
| AEE88280.1 | CurJ | BGC0000976 | NRP+Polyketide:Modular type I polyketide | 26.0 | 58.3 | 481.0 | 2.2e-136 |
| AAT70105.1 | CurJ | BGC0001165 | NRP+Polyketide:Modular type I polyketide | 26.0 | 58.3 | 481.0 | 2.2e-136 |
| AEE88281.1 | CurI | BGC0000976 | NRP+Polyketide:Modular type I polyketide | 35.0 | 23.9 | 473.0 | 2.5e-136 |
| AAT70104.1 | CurI | BGC0001165 | NRP+Polyketide:Modular type I polyketide | 35.0 | 23.9 | 473.0 | 2.5e-136 |
| AAF26923.1 | polyketide\_synthase | BGC0000988 | NRP+Polyketide | 25.0 | 61.5 | 481.0 | 3.05e-136 |
| AFY58525.1 | polyketide\_synthase\_family\_protein | BGC0002411 | NRP+Polyketide | 36.0 | 22.3 | 470.0 | 4.68e-136 |
| AQM58285.1 | polyketide\_synthase | BGC0001816 | NRP+Polyketide | 34.0 | 27.0 | 480.0 | 6.93e-136 |
| BCB17033.1 | modular\_polyketide\_synthase | BGC0002523 | NRP | 26.0 | 60.9 | 482.0 | 8.31e-136 |
| APZ78678.1 | polyketide\_synthase | BGC0001417 | NRP:Cyclic depsipeptide+Polyketide:Iterative type I polyketide | 31.0 | 31.7 | 478.0 | 9.66e-136 |
| QVV57686.1 | hypothetical\_protein | BGC0002338 | Polyketide | 32.0 | 28.0 | 481.0 | 9.81e-136 |
| ACN64831.1 | PokM1 | BGC0001061 | Polyketide:Iterative type I polyketide+Polyketide:Type II polyketide+Saccharide:Hybrid/tailoring saccharide | 37.0 | 22.4 | 472.0 | 9.9e-136 |
| CBD77734.1 | polyketide\_synthase | BGC0000974 | NRP+Polyketide | 35.0 | 23.8 | 474.0 | 1.45e-135 |
| QBM78312.1 | polyketide\_synthase | BGC0002542 | Polyketide+NRP | 25.0 | 58.5 | 480.0 | 1.54e-135 |
| AHB82053.1 | polyketide\_synthase | BGC0001019 | NRP+Polyketide:Modular type I polyketide | 35.0 | 23.8 | 468.0 | 1.8e-135 |
| BAQ25507.1 | type\_I\_polyketide\_synthase | BGC0001288 | Polyketide | 34.0 | 26.7 | 472.0 | 2.07e-135 |
| AAK83194.1 | polyketide\_synthase | BGC0000026 | Saccharide:Oligosaccharide | 36.0 | 22.7 | 462.0 | 2.25e-135 |
| EPH46608.1 | putative\_Erythronolide\_synthase,\_modules\_3\_and\_4 | BGC0001519 | NRP+Polyketide | 34.0 | 22.6 | 454.0 | 2.75e-135 |
| ABB05102.1 | LipPks1 | BGC0001003 | NRP:Lipopeptide+Polyketide:Modular type I polyketide+Saccharide:Hybrid/tailoring saccharide | 35.0 | 24.9 | 477.0 | 2.9e-135 |
| AAW03325.1 | CtaB | BGC0000982 | NRP+Polyketide | 36.0 | 23.6 | 454.0 | 3.58e-135 |
| CAQ18832.1 | polyketide\_synthase | BGC0000954 | NRP+Polyketide:Modular type I polyketide | 25.0 | 61.7 | 476.0 | 4.23e-135 |
| TGZ15167.1 | polyketide\_synthase | BGC0002032 | Polyketide | 36.0 | 23.8 | 479.0 | 4.34e-135 |
| CAQ43078.1 | polyketide\_synthase | BGC0000970 | NRP+Polyketide:Modular type I polyketide | 32.0 | 28.6 | 472.0 | 4.52e-135 |
| ABK32263.1 | AmbH | BGC0000014 | Polyketide | 34.0 | 24.8 | 470.0 | 6.32e-135 |
| CAQ34919.1 | polyketide\_synthase | BGC0000986 | NRP+Polyketide | 36.0 | 22.6 | 471.0 | 6.64e-135 |
| WP\_033261453.1 | type\_I\_polyketide\_synthase | BGC0002009 | Polyketide | 34.0 | 27.7 | 471.0 | 7e-135 |
| AEE88278.1 | CurL | BGC0000976 | NRP+Polyketide:Modular type I polyketide | 35.0 | 23.8 | 473.0 | 7.56e-135 |
| AAT70107.1 | CurL | BGC0001165 | NRP+Polyketide:Modular type I polyketide | 35.0 | 23.8 | 473.0 | 7.56e-135 |
| AVI57433.1 | AbmB1 | BGC0001694 | Polyketide | 37.0 | 23.5 | 479.0 | 7.6e-135 |
| CAL58684.1 | polyketide\_synthase | BGC0000149 | Polyketide:Modular type I polyketide | 36.0 | 22.7 | 479.0 | 8.8e-135 |
| AWS21279.1 | type\_I\_polyketide\_synthase | BGC0001934 | Polyketide | 36.0 | 24.4 | 464.0 | 8.95e-135 |
| AZY91989.1 | polyketide\_synthase | BGC0002022 | Polyketide | 36.0 | 24.4 | 464.0 | 8.95e-135 |
| CAD89773.1 | MelB\_protein | BGC0001010 | NRP+Polyketide:Modular type I polyketide | 35.0 | 23.8 | 453.0 | 1.17e-134 |
| AHA12079.1 | polyketide\_synthase\_type\_1 | BGC0001172 | NRP+Polyketide:Modular type I polyketide | 36.0 | 24.4 | 462.0 | 1.6e-134 |
| ADC79637.1 | TamAI | BGC0001052 | NRP+Polyketide:Modular type I polyketide | 37.0 | 22.5 | 478.0 | 1.6e-134 |
| AAY42396.1 | Polyketide\_synthase | BGC0001000 | NRP:Lipopeptide+Polyketide:Modular type I polyketide | 36.0 | 22.4 | 471.0 | 1.76e-134 |
| ACR33079.1 | polyketide\_synthase | BGC0000017 | Alkaloid+Polyketide:Modular type I polyketide | 34.0 | 22.8 | 466.0 | 1.91e-134 |
| AIT55264.1 | polyketide\_synthase | BGC0000072 | Polyketide:Modular type I polyketide | 36.0 | 23.8 | 475.0 | 2.05e-134 |
| AEU11006.1 | NpnB | BGC0001029 | NRP+Polyketide | 37.0 | 22.7 | 478.0 | 2.36e-134 |
| BAW35654.1 | modular\_polyketide\_synthase | BGC0002355 | Polyketide+Other | 34.0 | 24.8 | 467.0 | 2.39e-134 |
| ctg1\_orf16 |  | BGC0001457 | NRP | 31.0 | 28.5 | 469.0 | 2.72e-134 |
| CAD19087.1 | StiC\_protein | BGC0000153 | NRP+Polyketide:Modular type I polyketide | 35.0 | 26.9 | 469.0 | 3.77e-134 |
| CAQ18835.1 | polyketide\_synthase | BGC0000954 | NRP+Polyketide:Modular type I polyketide | 32.0 | 29.5 | 464.0 | 6.83e-134 |
| ctg1\_orf15 |  | BGC0001457 | NRP | 36.0 | 23.5 | 471.0 | 7e-134 |
| CAQ64686.1 | lasalocid\_modular\_polyketide\_synthase | BGC0000087 | Polyketide | 33.0 | 26.5 | 476.0 | 7.18e-134 |
| CAQ18833.1 | polyketide\_synthase | BGC0000954 | NRP+Polyketide:Modular type I polyketide | 35.0 | 23.6 | 463.0 | 9.26e-134 |
| AZH23817.1 | MgiQ | BGC0001971 | NRP+Polyketide | 25.0 | 58.1 | 474.0 | 9.41e-134 |
| AMB48442.1 | polyketide\_synthase | BGC0001357 | Polyketide | 36.0 | 22.4 | 459.0 | 1e-133 |
| AAY89049.1 | polyketide\_synthase | BGC0001069 | NRP+Polyketide:Trans-AT type I polyketide | 36.0 | 24.5 | 475.0 | 1.38e-133 |
| AJD77023.1 | IkaA | BGC0001435 | NRP+Polyketide:Iterative type I polyketide | 36.0 | 22.4 | 474.0 | 1.4e-133 |
| AAF19814.1 | MtaF | BGC0001024 | NRP+Polyketide:Modular type I polyketide | 35.0 | 25.0 | 458.0 | 1.55e-133 |
| CAD19092.1 | StiH\_protein | BGC0000153 | NRP+Polyketide:Modular type I polyketide | 36.0 | 22.3 | 463.0 | 1.75e-133 |
| BAG85026.1 | putative\_polyketide\_synthase | BGC0000086 | Polyketide | 33.0 | 26.5 | 474.0 | 2.07e-133 |
| CAO98850.1 | polyketide\_synthase\_AufG | BGC0000023 | Polyketide:Modular type I polyketide | 35.0 | 23.6 | 469.0 | 2.87e-133 |
| APZ78858.1 | polyketide\_synthase | BGC0001432 | NRP:Cyclic depsipeptide+Polyketide:Iterative type I polyketide | 35.0 | 23.3 | 471.0 | 3.01e-133 |
| AAW03329.1 | CtaF | BGC0000982 | NRP+Polyketide | 36.0 | 23.9 | 457.0 | 3.77e-133 |
| CAD19093.1 | StiJ\_protein | BGC0000153 | NRP+Polyketide:Modular type I polyketide | 35.0 | 23.8 | 454.0 | 4.44e-133 |
| AUS29495.1 | polyketide\_synthase | BGC0001030 | NRP+Polyketide | 33.0 | 30.2 | 471.0 | 5.22e-133 |
| QCQ67875.1 | hybrid\_peptide\_synthetase/polyketide\_synthase | BGC0002297 | NRP+Polyketide | 35.0 | 23.4 | 473.0 | 5.24e-133 |
| AKL71649.1 | NocP | BGC0001703 | Other | 32.0 | 27.9 | 456.0 | 5.29e-133 |
| AQA28562.1 | type\_I\_polyketide\_synthase | BGC0001663 | Polyketide | 31.0 | 28.3 | 471.0 | 5.63e-133 |
| QBM78307.1 | polyketide\_synthase | BGC0002542 | Polyketide+NRP | 34.0 | 24.9 | 472.0 | 5.97e-133 |
| BAG84248.1 | putative\_polyketide\_synthase | BGC0000257 | Polyketide | 35.0 | 23.9 | 470.0 | 5.98e-133 |
| ADM46358.1 | polyketide\_synthase | BGC0000106 | Polyketide | 33.0 | 29.2 | 473.0 | 6.5e-133 |
| QTX15955.1 | polyketide\_synthase | BGC0002598 | Polyketide | 32.0 | 30.2 | 471.0 | 7.08e-133 |
| AIT55261.1 | polyketide\_synthase | BGC0000072 | Polyketide:Modular type I polyketide | 36.0 | 22.5 | 460.0 | 7.24e-133 |
| BBM96639.1 | modular\_polyketide\_synthase | BGC0002452 | Polyketide | 36.0 | 23.4 | 472.0 | 1.12e-132 |
| AEZ53945.1 | polyketide\_synthase | BGC0000144 | Polyketide:Modular type I polyketide | 37.0 | 22.3 | 472.0 | 1.37e-132 |
| ABC84458.1 | NigAIII | BGC0000114 | Polyketide:Modular type I polyketide | 26.0 | 62.8 | 471.0 | 1.48e-132 |
| CAJ46689.1 | polyketide\_synthase | BGC0000969 | NRP:Cyclic depsipeptide+Polyketide:Modular type I polyketide | 26.0 | 59.7 | 471.0 | 1.53e-132 |
| ABK32288.1 | JerB | BGC0000080 | Polyketide | 36.0 | 23.3 | 471.0 | 1.68e-132 |
| ADF88275.1 | polyketide\_synthase | BGC0000981 | NRP+Polyketide | 35.0 | 22.5 | 454.0 | 2.11e-132 |
| QEA08907.1 | JenA8 | BGC0002559 | Polyketide | 26.0 | 62.4 | 471.0 | 2.26e-132 |
| QCP68970.1 | VatU | BGC0002296 | NRP+Polyketide | 33.0 | 25.2 | 464.0 | 2.79e-132 |
| AEZ54377.1 | PieA4 | BGC0000124 | Polyketide | 33.0 | 26.8 | 466.0 | 3.74e-132 |
| AVI57434.1 | AbmB2 | BGC0001694 | Polyketide | 27.0 | 61.5 | 470.0 | 4.32e-132 |
| AXN93610.1 | PuwB | BGC0001953 | NRP | 32.0 | 28.8 | 468.0 | 4.78e-132 |
| ACR33078.1 | polyketide\_synthase | BGC0000017 | Alkaloid+Polyketide:Modular type I polyketide | 34.0 | 23.8 | 462.0 | 5.28e-132 |
| AQA28563.1 | type\_I\_polyketide\_synthase | BGC0001663 | Polyketide | 33.0 | 23.8 | 454.0 | 5.66e-132 |
| AXN93597.1 | PuwB | BGC0001952 | NRP | 33.0 | 28.4 | 468.0 | 6.1e-132 |
| QCP68973.1 | VatL | BGC0002296 | NRP+Polyketide | 34.0 | 25.4 | 459.0 | 6.74e-132 |
| AUS29500.1 | polyketide\_synthase | BGC0002607 | NRP+Polyketide | 32.0 | 30.1 | 467.0 | 7.98e-132 |
| AHH34189.1 | polyketide\_synthase | BGC0001162 | Polyketide:Modular type I polyketide | 35.0 | 23.5 | 466.0 | 8.14e-132 |
| AUA09467.1 | Erythronolide\_synthase,\_modules\_1\_and\_2 | BGC0002291 | Polyketide | 33.0 | 27.2 | 469.0 | 8.22e-132 |
| QCP68974.1 | VatV | BGC0002296 | NRP+Polyketide | 33.0 | 23.9 | 458.0 | 8.37e-132 |
| AEU11005.1 | NpnA | BGC0001029 | NRP+Polyketide | 34.0 | 23.7 | 469.0 | 9.3e-132 |
| CAQ18828.1 | polyketide\_synthase | BGC0000954 | NRP+Polyketide:Modular type I polyketide | 36.0 | 23.5 | 465.0 | 1.02e-131 |
| AWC08658.1 | polyketide\_synthase\_type\_I | BGC0001662 | Polyketide | 35.0 | 23.3 | 458.0 | 1.08e-131 |
| AAG23264.1 | polyketide\_synthase\_loading\_and\_extender\_module\_1 | BGC0000148 | Polyketide | 36.0 | 23.2 | 467.0 | 1.2e-131 |
| QDA77059.1 | polyketide\_synthase/nonribosomal\_peptide\_synthetase | BGC0002026 | NRP+Polyketide | 35.0 | 24.5 | 469.0 | 1.27e-131 |
| CAD89777.1 | MelF\_protein | BGC0001010 | NRP+Polyketide:Modular type I polyketide | 35.0 | 24.3 | 452.0 | 1.32e-131 |
| ABX60163.1 | polyketide\_synthase | BGC0000978 | NRP+Alkaloid+Polyketide:Modular type I polyketide | 34.0 | 25.0 | 458.0 | 1.33e-131 |
| QPP46758.1 | polyketide\_synthase | BGC0002500 | Polyketide | 36.0 | 23.2 | 468.0 | 1.4e-131 |
| WP\_051206794.1 | type\_I\_polyketide\_synthase | BGC0002624 | NRP+Polyketide | 34.0 | 22.9 | 439.0 | 1.57e-131 |
| ABL86391.1 | hybrid\_polyketide\_synthase\_and\_nonribosomal\_peptide\_synthetase | BGC0000999 | NRP+Polyketide | 37.0 | 22.6 | 467.0 | 1.82e-131 |
| BAW35638.1 | modular\_polyketide\_synthase | BGC0002356 | Polyketide+Other | 35.0 | 23.3 | 457.0 | 1.94e-131 |
| ATY46594.1 | polyketide\_synthase | BGC0001666 | Polyketide | 35.0 | 22.9 | 443.0 | 1.98e-131 |
| MBA0053739.1 | acyltransferase\_domain-containing\_protein | BGC0002096 | Polyketide | 37.0 | 23.0 | 455.0 | 2.18e-131 |
| AAC01712.2 | RifC | BGC0000136 | Polyketide | 34.0 | 26.7 | 459.0 | 2.38e-131 |
| TMU97099.1 | SDR\_family\_NAD(P)-dependent\_oxidoreductase | BGC0002038 | Polyketide | 27.0 | 64.1 | 467.0 | 2.85e-131 |
| ABO15860.1 | polyketide\_synthase | BGC0000130 | Polyketide | 37.0 | 22.9 | 464.0 | 3.21e-131 |
| AGZ15475.1 | putative\_type\_1\_modular\_polyketide\_synthase | BGC0001036 | NRP+Polyketide | 33.0 | 23.4 | 442.0 | 3.23e-131 |
| QIE07124.1 | OvmK2 | BGC0001719 | Polyketide | 35.0 | 23.9 | 457.0 | 3.35e-131 |
| AEE88289.1 | CurA | BGC0000976 | NRP+Polyketide:Modular type I polyketide | 35.0 | 22.8 | 464.0 | 3.49e-131 |
| AAT70096.1 | CurA | BGC0001165 | NRP+Polyketide:Modular type I polyketide | 35.0 | 22.8 | 464.0 | 3.49e-131 |
| AHB82064.1 | polyketide\_synthase | BGC0001231 | NRP+Polyketide:Modular type I polyketide | 35.0 | 24.7 | 455.0 | 3.62e-131 |
| CAE02602.1 | polyketide\_synthase\_type\_I | BGC0000024 | Polyketide:Modular type I polyketide | 34.0 | 23.4 | 460.0 | 4.29e-131 |
| AZH23819.1 | MgiR | BGC0001971 | NRP+Polyketide | 33.0 | 23.7 | 461.0 | 4.3e-131 |
| AAZ77698.1 | ChlA5 | BGC0000036 | Polyketide:Modular type I polyketide+Polyketide:Iterative type I polyketide+Saccharide:Oligosaccharide | 26.0 | 61.9 | 466.0 | 4.7e-131 |
| CAL58682.1 | polyketide\_synthase | BGC0000149 | Polyketide:Modular type I polyketide | 35.0 | 25.8 | 466.0 | 4.98e-131 |
| EGJ35088.1 | Polyketide\_synthase | BGC0001163 | Polyketide:Modular type I polyketide | 35.0 | 23.7 | 463.0 | 5.25e-131 |
| AAC69332.1 | type\_I\_polyketide\_synthase\_PikAIV | BGC0000094 | Polyketide:Modular type I polyketide+Saccharide:Hybrid/tailoring saccharide | 33.0 | 24.3 | 449.0 | 8.12e-131 |
| BAK64649.1 | polyketide\_synthase | BGC0000135 | Polyketide | 37.0 | 22.8 | 466.0 | 9.12e-131 |
| AKJ15837.1 | acyl\_transferase | BGC0002735 | Polyketide+NRP | 33.0 | 22.6 | 441.0 | 9.6e-131 |
| IF55\_RS32375 | beta-ketoacyl\_synthase | BGC0001348 | Polyketide:Modular type I polyketide | 34.0 | 26.8 | 465.0 | 1.05e-130 |
| QBG82532.1 | Polyketide\_synthase | BGC0002587 | Polyketide | 35.0 | 24.6 | 465.0 | 1.21e-130 |
| AAZ94389.1 | modular\_polyketide\_synthase | BGC0000040 | Polyketide | 26.0 | 61.5 | 465.0 | 1.4e-130 |
| CAO98879.1 | polyketide\_synthase\_AufD | BGC0000023 | Polyketide:Modular type I polyketide | 33.0 | 29.4 | 464.0 | 1.44e-130 |
| AAK57190.1 | MxaF | BGC0001022 | NRP+Polyketide | 25.0 | 62.9 | 462.0 | 1.72e-130 |
| AUO16422.1 | polyketide\_synthase | BGC0001700 | Polyketide | 34.0 | 24.0 | 464.0 | 1.95e-130 |
| AEK75503.1 | type\_1\_polyketide\_synthase | BGC0000001 | Polyketide:Modular type I polyketide | 34.0 | 27.0 | 464.0 | 2.13e-130 |
| ABK32256.1 | AmbB | BGC0000014 | Polyketide | 35.0 | 24.7 | 464.0 | 2.17e-130 |
| BAF02922.1 | type\_I\_polyketide\_synthase | BGC0000073 | Polyketide | 32.0 | 26.9 | 465.0 | 2.17e-130 |
| ACC80699.1 | beta-ketoacyl\_synthase | BGC0002677 | Other | 33.0 | 25.5 | 458.0 | 2.18e-130 |
| ABX60153.1 | polyketide\_synthase | BGC0000978 | NRP+Alkaloid+Polyketide:Modular type I polyketide | 35.0 | 22.5 | 448.0 | 2.4e-130 |
| ABC84460.1 | NigAV | BGC0000114 | Polyketide:Modular type I polyketide | 27.0 | 63.7 | 464.0 | 2.45e-130 |
| ASZ00151.1 | polyketide\_synthase | BGC0001785 | Polyketide | 34.0 | 26.5 | 464.0 | 2.69e-130 |
| ADM46357.1 | polyketide\_synthase | BGC0000106 | Polyketide | 33.0 | 26.6 | 464.0 | 3.16e-130 |
| ACC80701.1 | beta-ketoacyl\_synthase | BGC0002677 | Other | 35.0 | 23.5 | 457.0 | 3.29e-130 |
| ADH04657.1 | TugA | BGC0001342 | NRP+Polyketide | 36.0 | 23.4 | 464.0 | 3.49e-130 |
| BAJ16470.1 | polyketide\_synthase | BGC0000058 | Polyketide | 32.0 | 30.1 | 463.0 | 3.64e-130 |
| CAQ34928.1 | polyketide\_synthase | BGC0000986 | NRP+Polyketide | 37.0 | 21.9 | 457.0 | 3.77e-130 |
| AWC08661.1 | polyketide\_synthase\_type\_I | BGC0001662 | Polyketide | 35.0 | 23.4 | 463.0 | 3.79e-130 |
| ADF88277.1 | polyketide\_synthase | BGC0000981 | NRP+Polyketide | 34.0 | 25.1 | 454.0 | 4.3e-130 |
| CAQ43077.1 | polyketide\_synthase | BGC0000970 | NRP+Polyketide:Modular type I polyketide | 32.0 | 28.8 | 449.0 | 4.54e-130 |
| AAC69329.1 | type\_I\_polyketide\_synthase\_PikAI | BGC0000094 | Polyketide:Modular type I polyketide+Saccharide:Hybrid/tailoring saccharide | 37.0 | 22.6 | 463.0 | 4.81e-130 |
| AUS29485.1 | polyketide\_synthase | BGC0002605 | NRP+Polyketide | 32.0 | 30.1 | 461.0 | 4.89e-130 |
| AAZ77694.1 | ChlA2 | BGC0000036 | Polyketide:Modular type I polyketide+Polyketide:Iterative type I polyketide+Saccharide:Oligosaccharide | 25.0 | 60.7 | 463.0 | 5.34e-130 |
| WP\_235558179.1 | SDR\_family\_NAD(P)-dependent\_oxidoreductase | BGC0001653 | Polyketide | 33.0 | 26.5 | 462.0 | 6.18e-130 |
| ANC94964.1 | AlmHIII | BGC0001396 | Polyketide | 26.0 | 60.5 | 462.0 | 6.37e-130 |
| AAC01710.1 | RifA | BGC0000136 | Polyketide | 36.0 | 24.7 | 463.0 | 6.45e-130 |
| ctg1\_orf3 |  | BGC0001329 | Polyketide+NRP:Cyclic depsipeptide | 35.0 | 22.7 | 452.0 | 6.49e-130 |
| AZH23791.1 | MgcH | BGC0001970 | NRP+Polyketide | 34.0 | 23.9 | 453.0 | 6.83e-130 |
| AWH12670.1 | RmpA2 | BGC0001759 | Polyketide | 35.0 | 24.6 | 451.0 | 6.85e-130 |
| ADC79618.1 | BafAIII | BGC0000028 | Polyketide:Modular type I polyketide | 33.0 | 27.2 | 462.0 | 7.02e-130 |
| MCF2150414.1 | Polyketide\_synthase | BGC0002625 | NRP+Polyketide | 34.0 | 23.8 | 461.0 | 7.09e-130 |
| ADZ24997.1 | polyketide\_synthase | BGC0000380 | NRP+Polyketide:Modular type I polyketide | 31.0 | 28.9 | 456.0 | 9.03e-130 |
| WP\_083502114.1 | type\_I\_polyketide\_synthase | BGC0001653 | Polyketide | 36.0 | 24.8 | 461.0 | 1.02e-129 |
| AEZ54379.1 | PieA6 | BGC0000124 | Polyketide | 36.0 | 23.3 | 460.0 | 1.03e-129 |
| ABC84456.1 | NigAI | BGC0000114 | Polyketide:Modular type I polyketide | 36.0 | 22.6 | 460.0 | 1.05e-129 |
| BAW35635.1 | modular\_polyketide\_synthase | BGC0002356 | Polyketide+Other | 35.0 | 23.4 | 461.0 | 1.11e-129 |
| BAW35657.1 | modular\_polyketide\_synthase | BGC0002355 | Polyketide+Other | 34.0 | 24.8 | 461.0 | 1.13e-129 |
| AEZ54374.1 | PieA1 | BGC0000124 | Polyketide | 32.0 | 27.0 | 460.0 | 1.16e-129 |
| ctg1\_orf30 |  | BGC0000096 | Polyketide | 35.0 | 22.7 | 451.0 | 1.18e-129 |
| ADH04640.1 | TgaB | BGC0001051 | NRP+Polyketide:Modular type I polyketide | 36.0 | 22.2 | 461.0 | 2.18e-129 |
| WP\_020636817.1 | type\_I\_polyketide\_synthase | BGC0002011 | Polyketide | 35.0 | 23.5 | 449.0 | 2.19e-129 |
| CAD29794.1 | peptide\_synthetase | BGC0001015 | NRP+Polyketide | 35.0 | 23.5 | 461.0 | 2.24e-129 |
| BAC68128.1 | modular\_polyketide\_synthase | BGC0000059 | Polyketide | 32.0 | 27.1 | 461.0 | 2.32e-129 |
| OAQ83760.1 | polyketide\_synthase | BGC0001358 | NRP+Polyketide | 31.0 | 31.7 | 459.0 | 2.32e-129 |
| CAM00065.1 | EryAIII\_Erythromycin\_polyketide\_synthase\_modules\_5\_and\_6 | BGC0000055 | Polyketide:Modular type I polyketide+Saccharide:Hybrid/tailoring saccharide | 35.0 | 23.0 | 460.0 | 2.44e-129 |
| ASZ00150.1 | polyketide\_synthase | BGC0001785 | Polyketide | 33.0 | 26.8 | 454.0 | 2.52e-129 |
| ARM20277.1 | polyketide\_synthase | BGC0001523 | Polyketide | 34.0 | 23.5 | 461.0 | 2.66e-129 |
| AFI57006.1 | QmnA2 | BGC0000133 | Polyketide | 36.0 | 22.4 | 452.0 | 3.42e-129 |
| ctg1\_orf29 |  | BGC0000096 | Polyketide | 35.0 | 23.3 | 460.0 | 4.2e-129 |
| QBG82527.1 | Polyketide\_synthase | BGC0002587 | Polyketide | 35.0 | 23.0 | 460.0 | 4.2e-129 |
| MBA0053740.1 | acyltransferase\_domain-containing\_protein | BGC0002096 | Polyketide | 35.0 | 24.1 | 459.0 | 4.96e-129 |
| QDA77044.1 | polyketide\_synthase | BGC0002025 | NRP+Polyketide | 35.0 | 23.4 | 460.0 | 5.16e-129 |
| AGI99497.1 | type\_I\_polyketide\_synthase | BGC0001004 | Polyketide:Modular type I polyketide | 36.0 | 23.6 | 460.0 | 5.17e-129 |
| QFU80902.1 | PKS | BGC0002550 | Polyketide | 36.0 | 23.6 | 460.0 | 5.17e-129 |
| AVI57435.1 | AbmB3 | BGC0001694 | Polyketide | 34.0 | 22.7 | 436.0 | 5.29e-129 |
| ARM20283.1 | polyketide\_synthase | BGC0001523 | Polyketide | 34.0 | 23.7 | 456.0 | 5.85e-129 |
| AAS98782.1 | polyketide\_synthase | BGC0001001 | NRP+Polyketide | 35.0 | 22.8 | 451.0 | 6.25e-129 |
| WP\_055469549.1 | type\_I\_polyketide\_synthase | BGC0001537 | Polyketide | 32.0 | 27.4 | 459.0 | 6.51e-129 |
| AHA38201.1 | GphH | BGC0000069 | Polyketide | 36.0 | 22.0 | 449.0 | 7.1e-129 |
| WP\_033261454.1 | type\_I\_polyketide\_synthase | BGC0002009 | Polyketide | 34.0 | 26.8 | 459.0 | 7.65e-129 |
| BAN19720.1 | polyketide\_synthase | BGC0001252 | Polyketide | 32.0 | 28.2 | 457.0 | 9.25e-129 |
| ADU85988.1 | putative\_iterative\_type\_I\_polyketide\_synthase | BGC0000165 | Polyketide:Modular type I polyketide | 34.0 | 26.7 | 450.0 | 9.59e-129 |
| AIW82282.1 | PuwE | BGC0001125 | NRP+Polyketide | 34.0 | 24.8 | 458.0 | 1.15e-128 |
| AGC09484.1 | LobS1 | BGC0001183 | Polyketide | 37.0 | 22.7 | 459.0 | 1.16e-128 |
| AGC09487.1 | LobS5 | BGC0001183 | Polyketide | 35.0 | 23.6 | 448.0 | 1.22e-128 |
| BCB17030.1 | modular\_polyketide\_synthase | BGC0002523 | NRP | 32.0 | 27.0 | 459.0 | 1.35e-128 |
| AUO16399.1 | polyketide\_synthase | BGC0001700 | Polyketide | 34.0 | 23.4 | 458.0 | 1.4e-128 |
| BAD08358.1 | polyketide\_synthase\_modules\_4 | BGC0000167 | Polyketide | 26.0 | 63.1 | 455.0 | 1.63e-128 |
| ASZ00147.1 | polyketide\_synthase | BGC0001785 | Polyketide | 33.0 | 27.3 | 458.0 | 1.83e-128 |
| ATG32075.1 | polyketide\_synthase | BGC0001750 | NRP+Polyketide | 33.0 | 28.2 | 451.0 | 2.28e-128 |
| QQZ01626.1 | PKS | BGC0002497 | Other | 33.0 | 26.9 | 458.0 | 2.53e-128 |
| QGJ79645.1 | Polyketide\_synthase | BGC0002552 | Polyketide | 35.0 | 23.5 | 446.0 | 2.9e-128 |
| AQH32481.1 | hybrid\_polyketide\_synthase/peptide\_synthetase | BGC0001667 | NRP+Polyketide | 32.0 | 28.2 | 457.0 | 3.36e-128 |
| QRI43530.1 | type\_I\_polyketide\_synthase | BGC0002454 | Polyketide | 26.0 | 60.8 | 457.0 | 3.7e-128 |
| AFI57007.1 | QmnA3 | BGC0000133 | Polyketide | 35.0 | 22.4 | 440.0 | 3.71e-128 |
| CCE88377.1 | non-ribosomal\_peptide\_synthetase/polyketide\_synthase | BGC0001034 | NRP+Polyketide:Modular type I polyketide | 37.0 | 22.3 | 457.0 | 4.03e-128 |
| AGC24270.1 | prlP | BGC0001038 | NRP+Polyketide:Modular type I polyketide | 37.0 | 22.5 | 448.0 | 4.69e-128 |
| AWO77084.1 | hybrid\_non-ribosomal\_peptide\_synthetase/type\_I\_polyketide\_synthase | BGC0001556 | NRP+Polyketide | 33.0 | 28.8 | 456.0 | 5.27e-128 |
| ARW71486.1 | type\_I\_PKS\_module\_6 | BGC0001812 | Polyketide | 34.0 | 24.8 | 446.0 | 5.62e-128 |
| ASZ00148.1 | polyketide\_synthase | BGC0001785 | Polyketide | 34.0 | 26.7 | 456.0 | 5.96e-128 |
| ADB23403.1 | polyketide\_synthase\_type\_I | BGC0001062 | Polyketide | 35.0 | 23.4 | 455.0 | 5.98e-128 |
| AXN93601.1 | PuwE | BGC0001952 | NRP | 35.0 | 22.4 | 456.0 | 5.99e-128 |
| ctg1\_15 |  | BGC0001931 | Polyketide | 35.0 | 22.4 | 456.0 | 6.02e-128 |
| BAQ25483.1 | type\_I\_polyketide\_synthase | BGC0001288 | Polyketide | 35.0 | 22.7 | 435.0 | 6.52e-128 |
| BAG85027.1 | putative\_polyketide\_synthase | BGC0000086 | Polyketide | 35.0 | 22.2 | 456.0 | 6.92e-128 |
| CAQ64687.1 | lasalocid\_modular\_polyketide\_synthase | BGC0000087 | Polyketide | 35.0 | 22.2 | 456.0 | 6.95e-128 |
| QBG82518.1 | Polyketide\_synthase | BGC0002587 | Polyketide | 34.0 | 24.5 | 453.0 | 7.01e-128 |
| FS847\_01985 | type\_I\_polyketide\_synthase | BGC0001877 | Polyketide | 35.0 | 22.5 | 429.0 | 7.65e-128 |
| UHY14126.1 | PKS\_I | BGC0002671 | Polyketide | 33.0 | 27.4 | 452.0 | 8.58e-128 |
| ARW71483.1 | type\_I\_PKS\_loading\_module,\_module\_1,\_module\_2 | BGC0001812 | Polyketide | 35.0 | 23.8 | 456.0 | 1.01e-127 |
| CRI73799.1 | CongC\_protein | BGC0001215 | NRP | 35.0 | 23.9 | 456.0 | 1.01e-127 |
| CAL58681.1 | polyketide\_synthase | BGC0000149 | Polyketide:Modular type I polyketide | 35.0 | 22.7 | 456.0 | 1.03e-127 |
| AUS29490.1 | polyketide\_synthase | BGC0002606 | NRP+Polyketide | 32.0 | 30.4 | 454.0 | 1.09e-127 |
| BAW35633.1 | modular\_polyketide\_synthase | BGC0002356 | Polyketide+Other | 33.0 | 26.9 | 455.0 | 1.12e-127 |
| QKG20147.1 | type\_I\_polyketide\_synthase | BGC0002124 | Polyketide | 33.0 | 29.0 | 456.0 | 1.21e-127 |
| AAF00958.1 | mcyE | BGC0001017 | NRP+Polyketide:Modular type I polyketide | 34.0 | 23.8 | 455.0 | 1.28e-127 |
| QLD23837.1 | SDR\_family\_NAD(P)-dependent\_oxidoreductase | BGC0002086 | Polyketide | 34.0 | 24.8 | 444.0 | 1.34e-127 |
| ABK32257.1 | AmbC | BGC0000014 | Polyketide | 35.0 | 22.5 | 455.0 | 1.38e-127 |
| EWM63000.1 | non-ribosomal\_peptide\_synthetase | BGC0001328 | NRP:Cyclic depsipeptide+Polyketide:Modular type I polyketide | 35.0 | 22.5 | 439.0 | 1.43e-127 |
| ADM46359.1 | polyketide\_synthase | BGC0000106 | Polyketide | 34.0 | 27.1 | 454.0 | 1.78e-127 |
| ctg1\_orf7 |  | BGC0000053 | Polyketide | 33.0 | 27.2 | 454.0 | 2.23e-127 |
| ARM20284.1 | polyketide\_synthase | BGC0001523 | Polyketide | 26.0 | 56.5 | 452.0 | 2.28e-127 |
| ADZ24996.1 | polyketide\_synthase | BGC0000380 | NRP+Polyketide:Modular type I polyketide | 27.0 | 59.0 | 451.0 | 2.59e-127 |
| CAO98852.1 | polyketide\_synthase\_AufI | BGC0000023 | Polyketide:Modular type I polyketide | 35.0 | 23.7 | 453.0 | 2.64e-127 |
| QPP46757.1 | polyketide\_synthase | BGC0002500 | Polyketide | 36.0 | 23.7 | 451.0 | 2.85e-127 |
| AZH23821.1 | MgiH | BGC0001971 | NRP+Polyketide | 34.0 | 22.8 | 445.0 | 2.93e-127 |
| CBD77736.1 | polyketide\_synthase | BGC0000974 | NRP+Polyketide | 34.0 | 23.3 | 448.0 | 2.97e-127 |
| AQW44873.1 | polyketide\_synthase | BGC0001761 | Polyketide | 31.0 | 29.4 | 447.0 | 3.29e-127 |
| ABK32259.1 | AmbE | BGC0000014 | Polyketide | 35.0 | 23.4 | 453.0 | 3.8e-127 |
| QES95474.1 | type\_I\_polyketide\_synthase | BGC0002453 | Polyketide | 36.0 | 23.0 | 454.0 | 4.54e-127 |
| BAW35616.1 | modular\_polyketide\_synthase | BGC0002357 | Polyketide+Other | 32.0 | 27.0 | 454.0 | 5.34e-127 |
| AWW87422.1 | type\_I\_polyketide\_synthase | BGC0001755 | Polyketide | 35.0 | 23.5 | 453.0 | 5.6e-127 |
| AGI99494.1 | Type\_I\_polyketide\_synthase | BGC0001004 | Polyketide:Modular type I polyketide | 35.0 | 23.6 | 442.0 | 5.67e-127 |
| QFU80898.1 | PKS | BGC0002550 | Polyketide | 35.0 | 23.6 | 442.0 | 5.67e-127 |
| AAK19883.1 | soraphen\_polyketide\_synthase\_A | BGC0000147 | Polyketide:Modular type I polyketide | 34.0 | 23.6 | 453.0 | 6.49e-127 |
| CQR60495.1 | Polyketide\_synthase,\_type\_I,\_module\_7 | BGC0001287 | Polyketide | 33.0 | 26.4 | 446.0 | 7.25e-127 |
| QCP68966.1 | VatW | BGC0002296 | NRP+Polyketide | 35.0 | 23.8 | 452.0 | 7.38e-127 |
| AXN93613.1 | PuwE | BGC0001953 | NRP | 35.0 | 22.4 | 452.0 | 8.84e-127 |
| AAC46026.1 | polyketide\_synthase\_modules\_4\_and\_5 | BGC0000113 | Polyketide | 35.0 | 22.7 | 452.0 | 9.07e-127 |
| TMU97101.1 | SDR\_family\_NAD(P)-dependent\_oxidoreductase | BGC0002038 | Polyketide | 25.0 | 61.6 | 452.0 | 1.03e-126 |
| AWH12668.1 | RmpC | BGC0001759 | Polyketide | 34.0 | 26.7 | 444.0 | 1.08e-126 |
| ARM20282.1 | polyketide\_synthase | BGC0001523 | Polyketide | 35.0 | 22.7 | 452.0 | 1.09e-126 |
| BAT51065.1 | type\_I\_polyketide\_synthase | BGC0001296 | Polyketide | 32.0 | 26.7 | 452.0 | 1.16e-126 |
| QSV12659.1 | AvmB | BGC0002456 | Polyketide+NRP | 35.0 | 22.6 | 451.0 | 1.27e-126 |
| QGJ79676.1 | Polyketide\_synthase | BGC0002552 | Polyketide | 33.0 | 26.6 | 451.0 | 1.37e-126 |
| BBM96638.1 | modular\_polyketide\_synthase | BGC0002452 | Polyketide | 34.0 | 23.6 | 441.0 | 1.42e-126 |
| QLD23835.2 | SDR\_family\_NAD(P)-dependent\_oxidoreductase | BGC0002086 | Polyketide | 35.0 | 23.6 | 452.0 | 1.5e-126 |
| CAA16183.1 | polyketide\_synthase | BGC0001063 | NRP+Polyketide | 35.0 | 23.4 | 449.0 | 1.56e-126 |
| AAC01711.1 | RifB | BGC0000136 | Polyketide | 34.0 | 26.7 | 452.0 | 1.66e-126 |
| AAA79984.2 | soraphen\_polyketide\_synthase\_B | BGC0000147 | Polyketide:Modular type I polyketide | 35.0 | 22.2 | 452.0 | 1.67e-126 |
| ABB88523.1 | polyketide\_synthase\_type\_I | BGC0000050 | Polyketide | 35.0 | 24.0 | 446.0 | 1.7e-126 |
| ATY46595.1 | polyketide\_synthase | BGC0001666 | Polyketide | 33.0 | 26.8 | 444.0 | 1.9e-126 |
| AEZ53946.1 | polyketide\_synthase | BGC0000144 | Polyketide:Modular type I polyketide | 35.0 | 22.8 | 451.0 | 2.23e-126 |
| PAU45552.1 | Iterative\_polyketide\_synthase | BGC0002138 | Polyketide | 35.0 | 23.6 | 436.0 | 2.28e-126 |
| ACB46485.1 | polyketide\_synthase | BGC0000082 | Polyketide | 34.0 | 22.6 | 439.0 | 2.4e-126 |
| CAQ64691.1 | lasalocid\_modular\_polyketide\_synthase | BGC0000087 | Polyketide | 32.0 | 27.1 | 444.0 | 3.78e-126 |
| BAW35659.1 | modular\_polyketide\_synthase | BGC0002355 | Polyketide+Other | 33.0 | 27.1 | 450.0 | 3.79e-126 |
| WP\_051137606.1 | type\_I\_polyketide\_synthase | BGC0002011 | Polyketide | 33.0 | 26.6 | 449.0 | 4.06e-126 |
| QUQ72347.1 | 3-ketoacyl-CoA\_thiolase | BGC0002349 | Polyketide+Saccharide | 34.0 | 24.3 | 451.0 | 4.6e-126 |
| AUD08663.1 | iPKS-NRPS | BGC0001553 | NRP+Polyketide | 33.0 | 28.5 | 449.0 | 5.03e-126 |
| BAF02924.1 | type\_I\_polyketide\_synthase | BGC0000073 | Polyketide | 33.0 | 26.7 | 450.0 | 5.04e-126 |
| QKG20145.1 | type\_I\_polyketide\_synthase | BGC0002124 | Polyketide | 36.0 | 22.3 | 449.0 | 5.2e-126 |
| ADM46356.1 | polyketide\_synthase | BGC0000106 | Polyketide | 33.0 | 24.4 | 450.0 | 5.26e-126 |
| QFU19826.1 | PKS | BGC0002431 | Polyketide+Saccharide | 34.0 | 23.8 | 446.0 | 5.57e-126 |
| CBD77748.1 | polyketide\_synthase | BGC0000974 | NRP+Polyketide | 35.0 | 23.4 | 444.0 | 5.8e-126 |
| EHK80163.1 | acyl\_transferase | BGC0001447 | Polyketide | 34.0 | 26.6 | 450.0 | 6.44e-126 |
| ACB37755.1 | putative\_type\_I\_polyketide\_synthase | BGC0000162 | Polyketide | 35.0 | 22.5 | 449.0 | 6.62e-126 |
| ATP76239.1 | NdaF | BGC0001705 | NRP+Polyketide | 34.0 | 23.4 | 449.0 | 7.29e-126 |
| AAU93807.2 | polyketide\_synthase\_modules\_1\_and\_2 | BGC0000054 | Polyketide | 35.0 | 24.7 | 449.0 | 7.44e-126 |
| ADH04660.1 | TugD | BGC0001342 | NRP+Polyketide | 35.0 | 23.8 | 450.0 | 7.46e-126 |
| ASZ00149.1 | polyketide\_synthase | BGC0001785 | Polyketide | 35.0 | 24.9 | 449.0 | 7.57e-126 |
| OJJ98497.1 | hypothetical\_protein | BGC0002169 | Polyketide+NRP | 29.0 | 37.2 | 439.0 | 7.8e-126 |
| QCQ67874.1 | type\_I\_polyketide\_synthase | BGC0002297 | NRP+Polyketide | 33.0 | 23.4 | 449.0 | 8.55e-126 |
| AAM77986.1 | iterative\_type\_I\_polyketide\_synthase | BGC0000112 | Polyketide:Iterative type I polyketide+Polyketide:Enediyne type I polyketide | 34.0 | 25.6 | 442.0 | 8.61e-126 |
| CAJ88187.2 | Type\_I\_modular\_polyketide\_synthase | BGC0000151 | Polyketide:Modular type I polyketide+Saccharide:Hybrid/tailoring saccharide | 36.0 | 22.6 | 449.0 | 8.76e-126 |
| UHH90010.1 | VicP2 | BGC0002634 | Polyketide+NRP+Other | 26.0 | 61.8 | 446.0 | 1.26e-125 |
| ACB37743.1 | putative\_type\_I\_polyketide\_synthase | BGC0000162 | Polyketide | 36.0 | 22.5 | 437.0 | 1.52e-125 |
| ANR02551.1 | LodJ | BGC0001648 | Polyketide | 33.0 | 23.0 | 428.0 | 1.53e-125 |
| BAW35611.1 | modular\_polyketide\_synthase | BGC0002357 | Polyketide+Other | 35.0 | 22.6 | 439.0 | 1.61e-125 |
| AEZ64505.1 | Herb | BGC0001065 | Polyketide | 37.0 | 21.4 | 449.0 | 1.64e-125 |
| AGY62754.1 | EbeB | BGC0000051 | Polyketide | 34.0 | 23.4 | 445.0 | 1.71e-125 |
| SCN11950.1 | EbeB-type\_I\_polyketide\_synthase | BGC0001580 | Polyketide | 34.0 | 23.4 | 445.0 | 1.71e-125 |
| AXG22406.1 | type\_I\_polyketide\_synthase | BGC0002024 | Polyketide | 36.0 | 22.6 | 448.0 | 1.78e-125 |
| QQZ01584.1 | PKS | BGC0002498 | Other | 34.0 | 22.3 | 439.0 | 1.81e-125 |
| AJW65409.1 | type\_I\_modular\_polyketide\_synthase | BGC0001195 | NRP+Polyketide | 35.0 | 23.5 | 448.0 | 1.83e-125 |
| QQZ01587.1 | PKS | BGC0002498 | Other | 34.0 | 24.6 | 447.0 | 1.85e-125 |
| BBA66511.1 | type\_I\_polyketide\_synthase | BGC0001495 | Polyketide | 35.0 | 23.3 | 448.0 | 1.95e-125 |
| QPP46749.1 | polyketide\_synthase | BGC0002500 | Polyketide | 32.0 | 27.1 | 442.0 | 1.96e-125 |
| AWS21278.1 | type\_I\_polyketide\_synthase | BGC0001934 | Polyketide | 34.0 | 24.8 | 447.0 | 2.01e-125 |
| AZY91987.1 | polyketide\_synthase | BGC0002022 | Polyketide | 34.0 | 24.8 | 447.0 | 2.01e-125 |
| WP\_052165465.1 | type\_I\_polyketide\_synthase | BGC0001327 | NRP:Cyclic depsipeptide+Polyketide:Modular type I polyketide | 34.0 | 22.5 | 438.0 | 2.12e-125 |
| AWH12669.1 | RmpB | BGC0001759 | Polyketide | 33.0 | 26.8 | 448.0 | 2.43e-125 |
| AWC08663.1 | polyketide\_synthase\_type\_I | BGC0001662 | Polyketide | 33.0 | 26.9 | 447.0 | 2.45e-125 |
| AGZ15472.1 | putative\_modular\_polyketide\_synthase | BGC0001036 | NRP+Polyketide | 34.0 | 24.3 | 425.0 | 2.66e-125 |
| AXG22407.1 | type\_I\_polyketide\_synthase | BGC0002024 | Polyketide | 34.0 | 23.1 | 448.0 | 2.71e-125 |
| QES95477.1 | type\_I\_polyketide\_synthase | BGC0002453 | Polyketide | 35.0 | 22.9 | 427.0 | 2.72e-125 |
| AXI91546.1 | FunP7 | BGC0001944 | Polyketide | 34.0 | 24.7 | 448.0 | 2.97e-125 |
| QKW94285.1 | short-chain\_dehydrogenase/reductase\_SDR | BGC0002342 | NRP+Polyketide | 34.0 | 23.7 | 446.0 | 2.97e-125 |
| QIE07127.1 | OvmL1 | BGC0001719 | Polyketide | 25.0 | 62.2 | 444.0 | 3.03e-125 |
| AHH99925.1 | PKS\_I | BGC0000002 | Polyketide | 35.0 | 23.2 | 447.0 | 3.16e-125 |
| ARV85760.1 | PieA1\_type\_I\_PKS | BGC0001742 | Polyketide | 35.0 | 22.5 | 446.0 | 3.33e-125 |
| AKA59088.1 | type-I\_PKS | BGC0001619 | Polyketide | 33.0 | 26.1 | 447.0 | 3.42e-125 |
| BAG85031.1 | putative\_polyketide\_synthase | BGC0000086 | Polyketide | 32.0 | 27.3 | 442.0 | 3.43e-125 |
| ACF35445.1 | mbcAI | BGC0000090 | Polyketide | 33.0 | 27.3 | 447.0 | 3.78e-125 |
| AUO16402.1 | polyketide\_synthase | BGC0001700 | Polyketide | 34.0 | 22.6 | 446.0 | 4.16e-125 |
| BAF02926.1 | type\_I\_polyketide\_synthase | BGC0000073 | Polyketide | 34.0 | 27.0 | 447.0 | 4.28e-125 |
| AAC38076.1 | polyketide\_synthase\_type\_I | BGC0000127 | Polyketide | 34.0 | 23.4 | 440.0 | 4.45e-125 |
| SCN11951.1 | ebeC-type\_I\_polyketide\_synthase | BGC0001580 | Polyketide | 34.0 | 23.5 | 446.0 | 4.97e-125 |
| AXN93580.1 | PuwE | BGC0001950 | NRP | 33.0 | 25.8 | 446.0 | 5.16e-125 |
| AXN93589.1 | PuwE | BGC0001951 | NRP | 33.0 | 25.8 | 446.0 | 5.16e-125 |
| BCK51645.1 | modular\_polyketide\_synthase | BGC0002520 | Polyketide | 34.0 | 23.0 | 447.0 | 5.17e-125 |
| AAB66506.1 | tylactone\_synthase\_modules\_4\_&\_5 | BGC0000166 | Polyketide | 34.0 | 24.1 | 446.0 | 5.31e-125 |
| AAQ82565.1 | FscB | BGC0000034 | NRP+Polyketide | 35.0 | 23.8 | 447.0 | 5.81e-125 |
| AAD03047.1 | type\_I\_polyketide\_synthase | BGC0000041 | Polyketide | 34.0 | 23.2 | 445.0 | 6.19e-125 |
| WP\_157358234.1 | SDR\_family\_NAD(P)-dependent\_oxidoreductase | BGC0002011 | Polyketide | 35.0 | 23.5 | 446.0 | 6.28e-125 |
| BCK51638.1 | modular\_polyketide\_synthase | BGC0002520 | Polyketide | 34.0 | 24.7 | 447.0 | 6.3e-125 |
| MBV7329455.1 | amino\_acid\_adenylation\_domain-containing\_protein | BGC0002131 | Polyketide+NRP:Glycopeptide+Saccharide:Hybrid/tailoring saccharide | 35.0 | 22.7 | 445.0 | 6.68e-125 |
| QKV49765.1 | PKS | BGC0002526 | Polyketide | 35.0 | 22.4 | 445.0 | 7.13e-125 |
| ANR02555.1 | LodN | BGC0001648 | Polyketide | 31.0 | 26.5 | 446.0 | 7.51e-125 |
| SCN11949.1 | ebeA-type\_I\_polyketide\_synthase\_KSQ-ATa-ACP | BGC0001580 | Polyketide | 36.0 | 22.0 | 424.0 | 8.32e-125 |
| ABY21538.1 | AngAI | BGC0000018 | Polyketide | 34.0 | 24.4 | 446.0 | 8.41e-125 |
| AVV61982.1 | type\_I\_modular\_PKS | BGC0001477 | NRP+Polyketide:Modular type I polyketide | 32.0 | 26.9 | 440.0 | 8.49e-125 |
| SCO70309.1 | Type\_I\_polyketide\_synthase | BGC0001433 | Polyketide:Modular type I polyketide | 34.0 | 24.3 | 446.0 | 8.58e-125 |
| AUO16403.1 | polyketide\_synthase | BGC0001700 | Polyketide | 33.0 | 23.6 | 444.0 | 8.6e-125 |
| SCN11952.1 | ebeD-type\_I\_polyketide\_synthase | BGC0001580 | Polyketide | 32.0 | 27.0 | 446.0 | 9.72e-125 |
| CAI94682.1 | putative\_polyketide\_synthase | BGC0000141 | Polyketide | 35.0 | 24.4 | 446.0 | 9.73e-125 |
| AUO16423.1 | polyketide\_synthase | BGC0001700 | Polyketide | 25.0 | 57.1 | 443.0 | 1e-124 |
| ALD82523.1 | polyketide\_synthase | BGC0001212 | NRP+Polyketide | 35.0 | 23.8 | 440.0 | 1.02e-124 |
| ADC45538.1 | modular\_polyketide\_synthase | BGC0000093 | Polyketide | 34.0 | 23.5 | 446.0 | 1.02e-124 |
| AHA12078.1 | polyketide\_synthase\_type\_1 | BGC0001172 | NRP+Polyketide:Modular type I polyketide | 34.0 | 25.2 | 445.0 | 1.03e-124 |
| AGY30676.1 | Ann4 | BGC0001298 | Polyketide | 34.0 | 22.8 | 446.0 | 1.06e-124 |
| QIQ28616.1 | Nbc20 | BGC0002541 | Other | 34.0 | 23.7 | 446.0 | 1.06e-124 |
| ARV85761.1 | PieA2\_type\_I\_PKS | BGC0001742 | Polyketide | 35.0 | 22.7 | 445.0 | 1.07e-124 |
| ATX68116.1 | malonyl\_CoA-acyl\_carrier\_protein\_transacylase | BGC0001772 | Polyketide | 35.0 | 22.9 | 443.0 | 1.16e-124 |
| AAX98187.1 | polyketide\_synthase\_type\_I | BGC0000052 | Polyketide | 34.0 | 23.6 | 437.0 | 1.25e-124 |
| ARV85765.1 | PieA6\_type\_I\_PKS | BGC0001742 | Polyketide | 35.0 | 22.7 | 443.0 | 1.28e-124 |
| AFU82617.1 | polyketide\_synthase | BGC0000998 | NRP+Polyketide | 35.0 | 22.5 | 441.0 | 1.34e-124 |
| QKV49771.1 | PKS | BGC0002526 | Polyketide | 33.0 | 24.4 | 437.0 | 1.44e-124 |
| ACR50782.1 | polyketide\_synthase | BGC0000163 | Polyketide | 34.0 | 23.8 | 435.0 | 1.44e-124 |
| AUA09465.1 | Erythronolide\_synthase,\_modules\_5\_and\_6 | BGC0002291 | Polyketide | 34.0 | 23.5 | 436.0 | 1.52e-124 |
| ADC45515.1 | modular\_polyketide\_synthase | BGC0000093 | Polyketide | 35.0 | 22.9 | 440.0 | 1.55e-124 |
| AAS79461.1 | polyketide\_synthase\_subunit | BGC0000035 | Polyketide | 26.0 | 61.0 | 445.0 | 1.59e-124 |
| AFU82616.1 | polyketide\_synthase | BGC0000998 | NRP+Polyketide | 35.0 | 22.7 | 441.0 | 1.65e-124 |
| BAC76491.1 | lankamycin\_synthase\_LkmAIII | BGC0000085 | Polyketide | 34.0 | 23.4 | 444.0 | 1.69e-124 |
| QQZ01588.1 | PKS | BGC0002498 | Other | 33.0 | 26.7 | 445.0 | 1.74e-124 |
| TMU97102.1 | SDR\_family\_NAD(P)-dependent\_oxidoreductase | BGC0002038 | Polyketide | 34.0 | 25.1 | 442.0 | 1.85e-124 |
| WP\_159041997.1 | SDR\_family\_NAD(P)-dependent\_oxidoreductase | BGC0002033 | Polyketide | 34.0 | 26.9 | 444.0 | 2.04e-124 |
| WP\_051206795.1 | type\_I\_polyketide\_synthase | BGC0002624 | NRP+Polyketide | 34.0 | 23.5 | 424.0 | 2.12e-124 |
| BCK51641.1 | modular\_polyketide\_synthase | BGC0002520 | Polyketide | 32.0 | 26.6 | 445.0 | 2.17e-124 |
| AGY62755.1 | EbeC | BGC0000051 | Polyketide | 33.0 | 23.5 | 435.0 | 2.23e-124 |
| AWH12936.1 | StmA | BGC0001784 | Polyketide | 34.0 | 23.5 | 445.0 | 2.33e-124 |
| AUO16398.1 | polyketide\_synthase | BGC0001700 | Polyketide | 35.0 | 22.8 | 444.0 | 2.4e-124 |
| BAO98805.1 | putative\_polyketide\_synthase | BGC0001002 | NRP+Polyketide | 34.0 | 22.2 | 431.0 | 2.67e-124 |
| AAM81586.2 | putative\_type\_I\_polyketide\_synthase | BGC0000047 | Polyketide | 25.0 | 60.9 | 444.0 | 2.68e-124 |
| ARV85763.1 | PieA4\_type\_I\_PKS | BGC0001742 | Polyketide | 35.0 | 22.6 | 441.0 | 2.86e-124 |
| AFI57005.1 | QmnA1 | BGC0000133 | Polyketide | 37.0 | 22.2 | 444.0 | 3.03e-124 |
| AAO62582.1 | polyketide\_synthase\_peptide\_sythetase\_fusion\_protein | BGC0001016 | NRP+Polyketide | 34.0 | 23.4 | 444.0 | 3.18e-124 |
| BAQ21948.1 | putative\_type\_I\_polyketide\_synthase | BGC0001204 | Polyketide | 35.0 | 22.8 | 434.0 | 3.27e-124 |
| QCF28927.1 | type\_I\_polyketide\_synthase | BGC0002308 | Alkaloid+Polyketide | 36.0 | 22.5 | 444.0 | 3.34e-124 |
| AZH23787.1 | MgcQ | BGC0001970 | NRP+Polyketide | 24.0 | 59.2 | 443.0 | 3.38e-124 |
| CQR60497.1 | Polyketide\_synthase,\_type\_I,\_modules:\_loading,\_1,\_2\_and\_3 | BGC0001287 | Polyketide | 35.0 | 22.5 | 444.0 | 3.84e-124 |
| BAF02921.1 | type\_I\_polyketide\_synthase | BGC0000073 | Polyketide | 34.0 | 24.9 | 444.0 | 4e-124 |
| AAM81584.2 | putative\_type\_I\_polyketide\_synthase | BGC0000047 | Polyketide | 34.0 | 25.0 | 444.0 | 4.18e-124 |
| BCK51640.1 | modular\_polyketide\_synthase | BGC0002520 | Polyketide | 35.0 | 23.0 | 444.0 | 4.43e-124 |
| AHH99921.1 | PKS\_I | BGC0000002 | Polyketide | 34.0 | 23.5 | 444.0 | 4.5e-124 |
| BAW35615.1 | modular\_polyketide\_synthase | BGC0002357 | Polyketide+Other | 33.0 | 26.7 | 444.0 | 5.08e-124 |
| QQZ01629.1 | PKS | BGC0002497 | Other | 33.0 | 27.1 | 444.0 | 5.12e-124 |
| QHZ99323.1 | nargenicin\_PKS | BGC0001875 | Polyketide | 35.0 | 22.4 | 444.0 | 5.2e-124 |
| AKL64832.1 | polyketide\_synthase | BGC0002072 | Polyketide:Modular type I polyketide | 34.0 | 22.8 | 434.0 | 5.25e-124 |
| ACB46488.1 | polyketide\_synthase | BGC0000082 | Polyketide | 35.0 | 22.4 | 444.0 | 5.33e-124 |
| AJY78092.1 | polyketide\_synthase | BGC0001902 | NRP+Polyketide | 34.0 | 22.8 | 434.0 | 5.51e-124 |
| CQR60494.1 | Polyketide\_synthase,\_type\_I,\_module\_8 | BGC0001287 | Polyketide | 32.0 | 26.5 | 436.0 | 5.75e-124 |
| ADH04639.1 | TgaA | BGC0001051 | NRP+Polyketide:Modular type I polyketide | 26.0 | 63.1 | 444.0 | 6.07e-124 |
| ABK32290.1 | JerD | BGC0000080 | Polyketide | 34.0 | 22.7 | 437.0 | 6.35e-124 |
| AVV61980.1 | type\_I\_modular\_PKS | BGC0001477 | NRP+Polyketide:Modular type I polyketide | 32.0 | 26.7 | 443.0 | 7.39e-124 |
| WP\_003060229.1 | type\_I\_polyketide\_synthase | BGC0002009 | Polyketide | 33.0 | 24.8 | 443.0 | 7.67e-124 |
| ABY83164.1 | Azi26 | BGC0000960 | NRP+Polyketide | 32.0 | 26.6 | 436.0 | 8.11e-124 |
| CAQ64688.1 | lasalocid\_modular\_polyketide\_synthase | BGC0000087 | Polyketide | 32.0 | 23.0 | 422.0 | 8.7e-124 |
| ctg1\_14 |  | BGC0001931 | Polyketide | 34.0 | 22.6 | 438.0 | 8.85e-124 |
| AID65222.1 | putative\_aspartate\_racemase | BGC0000335 | NRP+Polyketide | 35.0 | 23.5 | 442.0 | 8.95e-124 |
| ATX68115.1 | malonyl\_CoA-acyl\_carrier\_protein\_transacylase | BGC0001772 | Polyketide | 34.0 | 24.8 | 437.0 | 9.56e-124 |
| BAG85028.1 | putative\_polyketide\_synthase | BGC0000086 | Polyketide | 32.0 | 23.0 | 422.0 | 9.67e-124 |
| CAD55506.1 | CpkA;\_Polyketide\_synthase\_loading\_module,\_and\_modules\_1\_and\_2 | BGC0000038 | Polyketide:Modular type I polyketide | 32.0 | 26.7 | 442.0 | 9.7e-124 |
| AAC46027.1 | polyketide\_synthase\_module\_6 | BGC0000113 | Polyketide | 35.0 | 23.4 | 432.0 | 1.02e-123 |
| AWH12665.1 | RmpE1 | BGC0001759 | Polyketide | 33.0 | 26.7 | 420.0 | 1.03e-123 |
| CBW54671.1 | polyketide\_synthase/non\_ribosomal\_peptide\_synthetase | BGC0000971 | NRP+Polyketide:Modular type I polyketide | 35.0 | 23.6 | 438.0 | 1.1e-123 |
| ACO94456.1 | polyketide\_synthase\_type\_I | BGC0000029 | Polyketide:Modular type I polyketide | 34.0 | 23.7 | 442.0 | 1.12e-123 |
| QKG20146.1 | Type\_I\_polyketide\_synthase | BGC0002124 | Polyketide | 32.0 | 27.1 | 442.0 | 1.13e-123 |
| AVV61984.1 | type\_I\_modular\_polyketide\_synthase | BGC0001477 | NRP+Polyketide:Modular type I polyketide | 35.0 | 22.5 | 442.0 | 1.31e-123 |
| CAQ34920.1 | polyketide\_synthase | BGC0000986 | NRP+Polyketide | 36.0 | 22.2 | 436.0 | 1.38e-123 |
| QKG20136.1 | type\_I\_polyketide\_synthase | BGC0002124 | Polyketide | 35.0 | 22.8 | 442.0 | 1.45e-123 |
| CCP20048.1 | divL1\_protein | BGC0001119 | Polyketide:Modular type I polyketide | 25.0 | 62.8 | 439.0 | 1.48e-123 |
| AWC08662.1 | polyketide\_synthase\_type\_I | BGC0001662 | Polyketide | 33.0 | 26.8 | 442.0 | 1.49e-123 |
| QBG82531.1 | cytochrome\_P450 | BGC0002587 | Polyketide | 33.0 | 24.5 | 441.0 | 1.6e-123 |
| SCO70308.1 | Type\_I\_polyketide\_synthase | BGC0001433 | Polyketide:Modular type I polyketide | 36.0 | 22.4 | 442.0 | 1.68e-123 |
| BAF92601.1 | iterative\_type\_I\_PKS | BGC0000118 | Polyketide | 34.0 | 24.8 | 436.0 | 1.69e-123 |
| ACJ24875.1 | 6-methylsalicylic\_acid\_synthase | BGC0000119 | Polyketide:Iterative type I polyketide+Saccharide:Hybrid/tailoring saccharide | 34.0 | 24.8 | 436.0 | 1.69e-123 |
| SAI82896.1 | HrnA2;\_Starter\_unit\_polyketide\_synthase\_type\_I;\_module\_4\_(partial) | BGC0002101 | Polyketide | 34.0 | 22.8 | 420.0 | 1.73e-123 |
| ANZ22995.1 | ZinA | BGC0001828 | Polyketide | 35.0 | 23.3 | 441.0 | 1.75e-123 |
| AHA38199.1 | GphF | BGC0000069 | Polyketide | 32.0 | 26.8 | 442.0 | 1.77e-123 |
| AAY28226.1 | HbmAII | BGC0000074 | Polyketide | 34.0 | 23.8 | 441.0 | 2.05e-123 |
| ABV97154.1 | Beta-ketoacyl\_synthase | BGC0000137 | Polyketide | 32.0 | 26.8 | 435.0 | 2.1e-123 |
| BCB17028.1 | modular\_polyketide\_synthase | BGC0002523 | NRP | 34.0 | 23.9 | 441.0 | 2.36e-123 |
| QFU19839.1 | PKS | BGC0002431 | Polyketide+Saccharide | 25.0 | 62.6 | 441.0 | 2.53e-123 |
| ctg1\_orf27 |  | BGC0000096 | Polyketide | 35.0 | 22.5 | 441.0 | 2.79e-123 |
| BAO66519.1 | type\_I\_polyketide\_synthase | BGC0000042 | Polyketide | 32.0 | 27.0 | 441.0 | 2.86e-123 |
| AGM05533.1 | type\_I\_polyketide\_synthase | BGC0002098 | Polyketide | 33.0 | 26.7 | 434.0 | 2.91e-123 |
| AAG13919.1 | megalomicin\_6-deoxyerythronolide\_B\_synthase\_3 | BGC0000092 | Polyketide | 33.0 | 24.5 | 440.0 | 3.12e-123 |
| ABV97152.1 | Beta-ketoacyl\_synthase | BGC0000137 | Polyketide | 31.0 | 29.1 | 441.0 | 3.17e-123 |
| ACO94483.1 | polyketide\_synthase\_type\_I | BGC0000097 | Polyketide:Modular type I polyketide | 34.0 | 23.3 | 441.0 | 3.54e-123 |
| ADC45586.1 | modular\_polyketide\_synthase | BGC0000093 | Polyketide | 35.0 | 22.5 | 441.0 | 3.55e-123 |
| AFY58526.1 | polyketide\_synthase\_family\_protein | BGC0002411 | NRP+Polyketide | 32.0 | 23.4 | 419.0 | 3.72e-123 |
| ANZ22985.1 | ZinB | BGC0001828 | Polyketide | 35.0 | 22.4 | 441.0 | 4.08e-123 |
| AGM05531.1 | type\_I\_polyketide\_synthase | BGC0002098 | Polyketide | 36.0 | 22.6 | 440.0 | 4.09e-123 |
| AMB20393.1 | polyketide\_synthase | BGC0002072 | Polyketide:Modular type I polyketide | 34.0 | 22.6 | 441.0 | 4.31e-123 |
| QCF28928.1 | type\_I\_polyketide\_synthase | BGC0002308 | Alkaloid+Polyketide | 37.0 | 20.3 | 440.0 | 4.89e-123 |
| QPP46750.1 | polyketide\_synthase | BGC0002500 | Polyketide | 35.0 | 22.6 | 439.0 | 4.92e-123 |
| QHZ99322.1 | nargenicin\_biosynthesis\_PKS | BGC0001875 | Polyketide | 35.0 | 23.2 | 440.0 | 5.02e-123 |
| AKA59090.1 | type-I\_PKS | BGC0001619 | Polyketide | 36.0 | 22.5 | 440.0 | 5.07e-123 |
| ABV97151.1 | AMP-dependent\_synthetase\_and\_ligase | BGC0000137 | Polyketide | 33.0 | 27.5 | 440.0 | 5.35e-123 |
| BAQ25511.1 | type\_I\_polyketide\_synthase | BGC0001288 | Polyketide | 34.0 | 23.6 | 441.0 | 5.44e-123 |
| QWF78547.1 | 3-ketoacyl-CoA\_thiolase | BGC0002142 | Polyketide | 35.0 | 22.7 | 440.0 | 5.48e-123 |
| AAT70108.1 | CurM | BGC0001165 | NRP+Polyketide:Modular type I polyketide | 34.0 | 23.5 | 437.0 | 5.87e-123 |
| AVV61989.1 | beta-ketoacyl\_synthase | BGC0001477 | NRP+Polyketide:Modular type I polyketide | 35.0 | 23.0 | 435.0 | 6.38e-123 |
| BAF85844.1 | modular\_polyketide\_synthase | BGC0000109 | Polyketide | 34.0 | 27.1 | 440.0 | 7.01e-123 |
| BAW35634.1 | modular\_polyketide\_synthase | BGC0002356 | Polyketide+Other | 33.0 | 26.8 | 440.0 | 7.45e-123 |
| UHY14127.1 | PKS\_I | BGC0002671 | Polyketide | 34.0 | 23.7 | 439.0 | 7.57e-123 |
| BAD08359.1 | polyketide\_synthase\_modules\_5-6 | BGC0000167 | Polyketide | 33.0 | 24.1 | 439.0 | 7.63e-123 |
| ACV42478.1 | polyketide\_synthase | BGC0000043 | Polyketide | 34.0 | 23.5 | 437.0 | 7.68e-123 |
| AEE88277.1 | CurM | BGC0000976 | NRP+Polyketide:Modular type I polyketide | 34.0 | 23.5 | 437.0 | 7.68e-123 |
| BAB69195.1 | modular\_polyketide\_synthase | BGC0000117 | Polyketide | 34.0 | 22.4 | 439.0 | 8.53e-123 |
| AAZ77693.1 | ChlA1 | BGC0000036 | Polyketide:Modular type I polyketide+Polyketide:Iterative type I polyketide+Saccharide:Oligosaccharide | 35.0 | 22.7 | 439.0 | 8.55e-123 |
| ABC84471.1 | NigAVII | BGC0000114 | Polyketide:Modular type I polyketide | 34.0 | 22.9 | 439.0 | 8.68e-123 |
| ctg1\_12 |  | BGC0001931 | Polyketide | 33.0 | 23.4 | 430.0 | 9.11e-123 |
| ANR02556.1 | LodO | BGC0001648 | Polyketide | 34.0 | 22.6 | 434.0 | 9.45e-123 |
| ACY06286.1 | polyketide\_synthase | BGC0001042 | NRP+Polyketide | 34.0 | 23.6 | 436.0 | 1.04e-122 |
| AWW87423.1 | type\_I\_polyketide\_synthase | BGC0001755 | Polyketide | 34.0 | 23.8 | 439.0 | 1.04e-122 |
| AAC46024.1 | polyketide\_synthase\_modules\_1\_and\_2 | BGC0000113 | Polyketide | 36.0 | 22.2 | 439.0 | 1.04e-122 |
| AEU17899.1 | putative\_type\_I\_PKS | BGC0001072 | Saccharide+Polyketide:Modular type I polyketide+Polyketide:Type II polyketide+Other:Aminocoumarin | 34.0 | 24.5 | 439.0 | 1.05e-122 |
| BAT51066.1 | type\_I\_polyketide\_synthase | BGC0001296 | Polyketide | 34.0 | 22.5 | 439.0 | 1.11e-122 |
| QBF51758.1 | type\_I\_polyketide\_synthase | BGC0001856 | Polyketide:Modular type I polyketide | 31.0 | 27.3 | 439.0 | 1.16e-122 |
| ALP32046.1 | CycF | BGC0001293 | Polyketide | 34.0 | 23.4 | 437.0 | 1.17e-122 |
| AFP87523.1 | type\_I\_polyketide\_synthase | BGC0001159 | NRP+Polyketide:Modular type I polyketide | 35.0 | 22.5 | 436.0 | 1.19e-122 |
| ACB46471.1 | polyketide\_synthase | BGC0000082 | Polyketide | 34.0 | 23.5 | 439.0 | 1.23e-122 |
| QNN81297.1 | IonAI | BGC0002446 | Polyketide | 26.0 | 61.0 | 439.0 | 1.27e-122 |
| CAD17792.1 | probable\_non\_ribosomal\_peptide\_synthetase\_protein | BGC0001363 | NRP+Polyketide | 35.0 | 22.6 | 439.0 | 1.4e-122 |
| ABC84457.1 | NigAII | BGC0000114 | Polyketide:Modular type I polyketide | 33.0 | 24.8 | 436.0 | 1.45e-122 |
| ANH11412.1 | SceQ | BGC0001770 | Polyketide | 33.0 | 27.5 | 432.0 | 1.58e-122 |
| ADC45534.1 | modular\_polyketide\_synthase | BGC0000093 | Polyketide | 31.0 | 27.2 | 439.0 | 1.72e-122 |
| QBF51769.1 | type\_I\_polyketide\_synthase | BGC0001856 | Polyketide:Modular type I polyketide | 33.0 | 27.7 | 436.0 | 1.77e-122 |
| UMP03508.1 | NmvAIII | BGC0002649 | NRP+Polyketide | 34.0 | 24.1 | 428.0 | 1.88e-122 |
| BAG23202.1 | putative\_type-I\_PKS | BGC0002673 | Polyketide+Alkaloid | 33.0 | 22.6 | 437.0 | 1.92e-122 |
| BAC76493.1 | lankamycin\_synthase\_LkmAI | BGC0000085 | Polyketide | 35.0 | 22.6 | 438.0 | 1.93e-122 |
| QEA08906.1 | JenA11 | BGC0002559 | Polyketide | 35.0 | 22.5 | 435.0 | 1.94e-122 |
| QKV49766.1 | PKS | BGC0002526 | Polyketide | 35.0 | 22.3 | 436.0 | 1.97e-122 |
| AAF71775.1 | nysB | BGC0000115 | Polyketide:Modular type I polyketide+Saccharide:Hybrid/tailoring saccharide | 34.0 | 24.7 | 437.0 | 2.05e-122 |
| ACN69990.1 | polyketide\_synthase | BGC0000079 | Polyketide | 35.0 | 22.8 | 438.0 | 2.07e-122 |
| ABB05105.1 | LipPks4 | BGC0001003 | NRP:Lipopeptide+Polyketide:Modular type I polyketide+Saccharide:Hybrid/tailoring saccharide | 33.0 | 27.4 | 437.0 | 2.15e-122 |
| AGC24271.1 | prlQ | BGC0001038 | NRP+Polyketide:Modular type I polyketide | 34.0 | 23.6 | 422.0 | 2.15e-122 |
| QWF78550.1 | 3-ketoacyl-CoA\_thiolase | BGC0002142 | Polyketide | 33.0 | 24.5 | 438.0 | 2.28e-122 |
| QES95476.1 | type\_I\_polyketide\_synthase | BGC0002453 | Polyketide | 34.0 | 23.1 | 432.0 | 2.31e-122 |
| QVV57684.1 | beta-ketoacyl\_synthase | BGC0002338 | Polyketide | 34.0 | 23.4 | 437.0 | 2.37e-122 |
| ctg1\_orf255 |  | BGC0001200 | Polyketide | 33.0 | 23.7 | 435.0 | 2.66e-122 |
| BAW35655.1 | modular\_polyketide\_synthase | BGC0002355 | Polyketide+Other | 32.0 | 26.9 | 438.0 | 2.72e-122 |
| AQH32482.1 | type\_1\_polyketide\_synthase | BGC0001667 | NRP+Polyketide | 34.0 | 22.4 | 437.0 | 2.72e-122 |
| ATP76241.1 | NdaD | BGC0001705 | NRP+Polyketide | 34.0 | 22.3 | 437.0 | 2.75e-122 |
| AAP42873.1 | NanA11 | BGC0000105 | Polyketide | 33.0 | 26.9 | 435.0 | 2.78e-122 |
| ACO94460.1 | polyketide\_synthase\_type\_I | BGC0000029 | Polyketide:Modular type I polyketide | 33.0 | 26.8 | 437.0 | 3.16e-122 |
| QFU19843.1 | PKS | BGC0002431 | Polyketide+Saccharide | 37.0 | 21.3 | 436.0 | 3.35e-122 |
| ACO94484.1 | polyketide\_synthase\_type\_I | BGC0000097 | Polyketide:Modular type I polyketide | 34.0 | 22.3 | 416.0 | 3.49e-122 |
| BAW35651.1 | modular\_polyketide\_synthase | BGC0002355 | Polyketide+Other | 35.0 | 22.7 | 438.0 | 3.57e-122 |
| ACC80700.1 | beta-ketoacyl\_synthase | BGC0002677 | Other | 33.0 | 24.7 | 433.0 | 3.62e-122 |
| ABB88521.1 | polyketide\_synthase\_type\_I | BGC0000050 | Polyketide | 35.0 | 22.5 | 434.0 | 3.63e-122 |
| BAR73007.1 | putative\_PKS\_(ACP-KS-AT-DH-KR-ACP-KS-AT-DH-ER-KR-ACP) | BGC0001194 | Polyketide | 36.0 | 22.6 | 437.0 | 3.68e-122 |
| TXD00261.1 | AMP-binding\_protein | BGC0001877 | Polyketide | 33.0 | 27.2 | 435.0 | 3.97e-122 |
| ABK32289.1 | JerC | BGC0000080 | Polyketide | 33.0 | 22.6 | 437.0 | 4.34e-122 |
| BAD08373.1 | polyketide\_synthase\_modules\_1-3 | BGC0000167 | Polyketide | 35.0 | 22.7 | 437.0 | 4.95e-122 |
| ARE67853.1 | AbsB1 | BGC0001492 | Polyketide | 34.0 | 23.8 | 437.0 | 5.21e-122 |
| QGA70078.1 | type\_I\_polyketide\_synthase | BGC0002517 | Polyketide | 32.0 | 26.7 | 436.0 | 5.36e-122 |
| AHH99923.1 | PKS\_I | BGC0000002 | Polyketide | 35.0 | 22.2 | 436.0 | 6.11e-122 |
| AAS98781.1 | polyketide\_synthase | BGC0001001 | NRP+Polyketide | 33.0 | 24.0 | 436.0 | 6.4e-122 |
| AEK75504.1 | type\_1\_polyketide\_synthase | BGC0000001 | Polyketide:Modular type I polyketide | 34.0 | 24.7 | 414.0 | 6.72e-122 |
| QHZ99321.1 | polyketide\_synthaase | BGC0001875 | Polyketide | 36.0 | 22.2 | 436.0 | 7.43e-122 |
| ctg1\_orf9 |  | BGC0000053 | Polyketide | 33.0 | 23.6 | 428.0 | 7.91e-122 |
| AAU93805.2 | polyketide\_synthase\_modules\_5\_and\_6 | BGC0000054 | Polyketide | 33.0 | 24.9 | 436.0 | 7.91e-122 |
| WP\_018540593.1 | type\_I\_polyketide\_synthase | BGC0001332 | NRP+Polyketide | 34.0 | 23.4 | 435.0 | 8.09e-122 |
| AWR88404.1 | putative\_beta-ketoacyl\_synthase | BGC0001522 | Polyketide | 33.0 | 27.7 | 436.0 | 8.12e-122 |
| TXD00265.1 | SDR\_family\_NAD(P)-dependent\_oxidoreductase | BGC0001877 | Polyketide | 33.0 | 24.0 | 436.0 | 8.39e-122 |
| ABK32258.1 | AmbD | BGC0000014 | Polyketide | 34.0 | 23.0 | 431.0 | 8.49e-122 |
| ADC79638.1 | TamAII | BGC0001052 | NRP+Polyketide:Modular type I polyketide | 33.0 | 26.6 | 436.0 | 8.65e-122 |
| AEC13070.1 | fosD | BGC0000060 | Polyketide | 34.0 | 23.6 | 429.0 | 9.26e-122 |
| AUO16397.1 | polyketide\_synthase | BGC0001700 | Polyketide | 32.0 | 26.8 | 436.0 | 1.07e-121 |
| ACC40921.1 | polyketide\_synthase\_Pks7 | BGC0001665 | Polyketide | 34.0 | 22.3 | 432.0 | 1.09e-121 |
| SAI82895.1 | HrnA1;\_Starter\_unit\_polyketide\_synthase\_type\_I;\_modules\_loading,\_1-3 | BGC0002101 | Polyketide | 34.0 | 23.5 | 436.0 | 1.16e-121 |
| BBM95963.1 | modular\_polyketide\_synthase | BGC0002558 | Polyketide | 34.0 | 25.2 | 435.0 | 1.2e-121 |
| QNN81301.1 | IonAV | BGC0002446 | Polyketide | 32.0 | 26.8 | 435.0 | 1.21e-121 |
| AZH23820.1 | MgiG | BGC0001971 | NRP+Polyketide | 31.0 | 23.9 | 431.0 | 1.21e-121 |
| AEZ53950.1 | polyketide\_synthase | BGC0000144 | Polyketide:Modular type I polyketide | 31.0 | 28.0 | 424.0 | 1.28e-121 |
| AAO62584.1 | polyketide\_synthase\_type\_1 | BGC0001016 | NRP+Polyketide | 34.0 | 22.4 | 435.0 | 1.37e-121 |
| BAO66528.1 | type\_I\_polyketide\_synthase | BGC0000042 | Polyketide | 35.0 | 22.8 | 414.0 | 1.6e-121 |
| ADB12490.1 | EpoC | BGC0000990 | NRP+Polyketide | 31.0 | 27.5 | 429.0 | 1.63e-121 |
| AAF62882.1 | EpoC | BGC0000991 | NRP+Polyketide | 31.0 | 27.4 | 429.0 | 1.63e-121 |
| BAE93728.1 | type\_I\_polyketide\_synthase | BGC0000164 | Polyketide | 35.0 | 23.1 | 427.0 | 1.68e-121 |
| BAF02927.1 | type\_I\_polyketide\_synthase | BGC0000073 | Polyketide | 34.0 | 23.5 | 432.0 | 1.72e-121 |
| QOD94997.1 | PldAII | BGC0002102 | Polyketide | 34.0 | 23.1 | 433.0 | 1.99e-121 |
| simG |  | BGC0000334 | NRP | 31.0 | 30.2 | 433.0 | 2.1e-121 |
| CAM00064.1 | EryAII\_Erythromycin\_polyketide\_synthase\_modules\_3\_and\_4 | BGC0000055 | Polyketide:Modular type I polyketide+Saccharide:Hybrid/tailoring saccharide | 33.0 | 22.2 | 434.0 | 2.12e-121 |
| ABB05103.1 | LipPks2 | BGC0001003 | NRP:Lipopeptide+Polyketide:Modular type I polyketide+Saccharide:Hybrid/tailoring saccharide | 32.0 | 26.7 | 434.0 | 2.13e-121 |
| AAU04878.1 | polyketide\_synthase | BGC0000365 | NRP | 34.0 | 22.4 | 434.0 | 2.19e-121 |
| TMV00153.1 | acyltransferase\_domain-containing\_protein | BGC0002038 | Polyketide | 36.0 | 20.7 | 433.0 | 2.26e-121 |
| QKG20159.1 | type\_I\_polyketide\_synthase | BGC0002124 | Polyketide | 34.0 | 23.8 | 426.0 | 2.36e-121 |
| AFV30251.1 | polyketide\_synthase | BGC0000075 | Polyketide | 33.0 | 25.1 | 434.0 | 2.36e-121 |
| BAQ25482.1 | type\_I\_polyketide\_synthase | BGC0001288 | Polyketide | 26.0 | 61.5 | 434.0 | 2.4e-121 |
| AKJ15895.1 | modular\_polyketide\_synthase | BGC0002735 | Polyketide+NRP | 33.0 | 23.4 | 413.0 | 2.71e-121 |
| CAL58686.1 | polyketide\_synthase | BGC0000149 | Polyketide:Modular type I polyketide | 35.0 | 22.9 | 434.0 | 2.71e-121 |
| QWF78553.1 | 3-ketoacyl-CoA\_thiolase | BGC0002142 | Polyketide | 32.0 | 26.5 | 434.0 | 2.76e-121 |
| TXD00024.1 | SDR\_family\_NAD(P)-dependent\_oxidoreductase | BGC0001877 | Polyketide | 34.0 | 24.4 | 434.0 | 2.85e-121 |
| AOE23578.1 | FoxBII | BGC0001598 | NRP+Polyketide | 34.0 | 22.8 | 434.0 | 2.87e-121 |
| BAH02271.1 | polyketide\_synthase | BGC0000126 | Polyketide | 33.0 | 23.5 | 428.0 | 3.12e-121 |
| QOD95000.1 | PldAV | BGC0002102 | Polyketide | 33.0 | 23.5 | 428.0 | 3.12e-121 |
| AAP85336.1 | type\_I\_PKS | BGC0000233 | Polyketide | 35.0 | 23.4 | 411.0 | 3.48e-121 |
| KFL51883.1 | amino\_acid\_adenylation\_protein | BGC0001711 | NRP+Polyketide | 37.0 | 22.2 | 434.0 | 3.54e-121 |
| AAB66505.1 | tylactone\_synthase\_module\_3 | BGC0000166 | Polyketide | 34.0 | 22.8 | 429.0 | 3.72e-121 |
| ACN69988.1 | polyketide\_synthase | BGC0000079 | Polyketide | 35.0 | 22.8 | 434.0 | 3.74e-121 |
| ACC40923.1 | polyketide\_synthase\_Pks9 | BGC0001665 | Polyketide | 35.0 | 23.1 | 412.0 | 3.82e-121 |
| AZF85945.1 | type\_I\_polyketide\_synthase | BGC0001963 | NRP+Polyketide | 32.0 | 24.7 | 434.0 | 3.86e-121 |
| AAC46025.1 | polyketide\_synthase\_module\_3 | BGC0000113 | Polyketide | 34.0 | 22.3 | 428.0 | 4.05e-121 |
| AEP40935.1 | polyketide\_synthase\_type\_I | BGC0000021 | Polyketide | 34.0 | 23.4 | 433.0 | 4.26e-121 |
| ADM46360.1 | polyketide\_synthase | BGC0000106 | Polyketide | 33.0 | 27.3 | 433.0 | 4.69e-121 |
| EPH46606.1 | putative\_Phenolphthiocerol\_synthesis\_polyketide\_synthase\_type\_I\_Pks15/1 | BGC0001519 | NRP+Polyketide | 34.0 | 23.0 | 429.0 | 4.85e-121 |
| WP\_063764078.1 | polyketide\_synthase | BGC0001348 | Polyketide:Modular type I polyketide | 33.0 | 26.7 | 431.0 | 4.97e-121 |
| AAG23263.1 | polyketide\_synthase\_extender\_modules\_5-7 | BGC0000148 | Polyketide | 35.0 | 22.5 | 434.0 | 5e-121 |
| AEH42490.1 | polyketide\_synthase | BGC0000032 | Polyketide | 34.0 | 22.6 | 425.0 | 5.66e-121 |
| CAQ64692.1 | lasalocid\_modular\_polyketide\_synthase | BGC0000087 | Polyketide | 34.0 | 24.4 | 419.0 | 6.02e-121 |
| ctg1\_orf28 |  | BGC0000096 | Polyketide | 32.0 | 24.0 | 429.0 | 6.63e-121 |
| BCB17026.1 | modular\_polyketide\_synthase | BGC0002523 | NRP | 33.0 | 23.7 | 433.0 | 6.82e-121 |
| AVV61983.1 | type\_I\_modular\_polyketide\_synthase | BGC0001477 | NRP+Polyketide:Modular type I polyketide | 32.0 | 27.5 | 433.0 | 7.19e-121 |
| QQZ01590.1 | PKS | BGC0002498 | Other | 34.0 | 24.5 | 429.0 | 8.1e-121 |
| QQZ01581.1 | PKS | BGC0002498 | Other | 35.0 | 22.7 | 433.0 | 8.43e-121 |
| AXG22405.1 | type\_I\_polyketide\_synthase | BGC0002024 | Polyketide | 35.0 | 22.4 | 432.0 | 8.43e-121 |
| QFU19842.1 | PKS | BGC0002431 | Polyketide+Saccharide | 35.0 | 22.5 | 430.0 | 8.53e-121 |
| UHY14125.1 | PKS\_I | BGC0002671 | Polyketide | 32.0 | 27.0 | 432.0 | 8.98e-121 |
| QFU19840.1 | PKS | BGC0002431 | Polyketide+Saccharide | 34.0 | 22.7 | 432.0 | 9.39e-121 |
| BAW35608.1 | modular\_polyketide\_synthase | BGC0002357 | Polyketide+Other | 33.0 | 24.8 | 433.0 | 9.46e-121 |
| AAM54077.1 | polyketide\_synthase | BGC0000020 | Polyketide | 34.0 | 24.5 | 424.0 | 1.01e-120 |
| BAH02269.1 | polyketide\_synthase | BGC0000126 | Polyketide | 34.0 | 23.1 | 433.0 | 1.04e-120 |
| IF55\_RS36525 | polyketide\_synthase | BGC0001348 | Polyketide:Modular type I polyketide | 33.0 | 24.4 | 429.0 | 1.07e-120 |
| AVV61979.1 | beta-ketoacyl\_synthase | BGC0001477 | NRP+Polyketide:Modular type I polyketide | 33.0 | 23.1 | 432.0 | 1.08e-120 |
| AAC01713.1 | RifD | BGC0000136 | Polyketide | 33.0 | 24.5 | 426.0 | 1.1e-120 |
| ABC84461.1 | NigAVI | BGC0000114 | Polyketide:Modular type I polyketide | 34.0 | 24.1 | 425.0 | 1.11e-120 |
| ADC79616.1 | BafAI | BGC0000028 | Polyketide:Modular type I polyketide | 34.0 | 23.4 | 432.0 | 1.11e-120 |
| UMP03507.1 | NmvAIV | BGC0002649 | NRP+Polyketide | 34.0 | 24.3 | 426.0 | 1.12e-120 |
| AEZ64503.1 | Herd | BGC0001065 | Polyketide | 35.0 | 22.4 | 429.0 | 1.12e-120 |
| CAL58685.1 | polyketide\_synthase | BGC0000149 | Polyketide:Modular type I polyketide | 34.0 | 22.9 | 432.0 | 1.13e-120 |
| ARM20281.1 | polyketide\_synthase | BGC0001523 | Polyketide | 33.0 | 23.5 | 432.0 | 1.18e-120 |
| WP\_055480219.1 | type\_I\_polyketide\_synthase | BGC0001653 | Polyketide | 36.0 | 22.5 | 432.0 | 1.19e-120 |
| AGY62759.1 | EbeG | BGC0000051 | Polyketide | 34.0 | 23.8 | 429.0 | 1.28e-120 |
| SCN11953.1 | ebeE-type\_I\_polyketide\_synthase | BGC0001580 | Polyketide | 34.0 | 23.8 | 429.0 | 1.28e-120 |
| BCK51647.1 | modular\_polyketide\_synthase | BGC0002520 | Polyketide | 34.0 | 25.0 | 424.0 | 1.34e-120 |
| AAG13917.1 | megalomicin\_6-deoxyerythronolide\_B\_synthase\_1 | BGC0000092 | Polyketide | 33.0 | 24.6 | 432.0 | 1.38e-120 |
| ATQ39432.1 | PKS | BGC0001565 | NRP | 32.0 | 28.7 | 431.0 | 1.49e-120 |
| WP\_102919232.1 | type\_I\_polyketide\_synthase | BGC0002104 | NRP+Polyketide | 35.0 | 22.2 | 432.0 | 1.51e-120 |
| AAF26920.1 | polyketide\_synthase | BGC0000988 | NRP+Polyketide | 31.0 | 27.5 | 426.0 | 1.57e-120 |
| QCP68965.1 | VatK | BGC0002296 | NRP+Polyketide | 33.0 | 24.2 | 431.0 | 1.61e-120 |
| ADC79620.1 | BafAV | BGC0000028 | Polyketide:Modular type I polyketide | 35.0 | 23.1 | 429.0 | 1.62e-120 |
| AHH99922.1 | PKS\_I | BGC0000002 | Polyketide | 36.0 | 22.5 | 432.0 | 1.76e-120 |
| ABV97155.1 | Acyl\_transferase | BGC0000137 | Polyketide | 32.0 | 26.5 | 431.0 | 1.78e-120 |
| CAO85896.1 | protein\_modular\_polyketide\_synthase\_NorA' | BGC0000110 | Polyketide:Modular type I polyketide | 34.0 | 23.3 | 431.0 | 1.87e-120 |
| ANH11414.1 | SceS | BGC0001770 | Polyketide | 32.0 | 26.7 | 432.0 | 1.98e-120 |
| AKG06378.1 | polyketide\_synthase\_type\_1 | BGC0001830 | Polyketide | 33.0 | 24.7 | 422.0 | 2.01e-120 |
| ADC45535.1 | modular\_polyketide\_synthase | BGC0000093 | Polyketide | 35.0 | 22.8 | 432.0 | 2.01e-120 |
| AZH23790.1 | MgcG | BGC0001970 | NRP+Polyketide | 31.0 | 23.9 | 427.0 | 2.04e-120 |
| AGI99482.1 | Type\_I\_polyketide\_synthase | BGC0001004 | Polyketide:Modular type I polyketide | 35.0 | 24.5 | 431.0 | 2.06e-120 |
| QFU80887.1 | PKS | BGC0002550 | Polyketide | 35.0 | 24.5 | 431.0 | 2.06e-120 |
| BBA66512.1 | type\_I\_polyketide\_synthase | BGC0001495 | Polyketide | 34.0 | 23.2 | 432.0 | 2.24e-120 |
| BBM96641.1 | modular\_polyketide\_synthase | BGC0002452 | Polyketide | 35.0 | 22.4 | 431.0 | 2.28e-120 |
| QDA77045.1 | polyketide\_synthase/nonribosomal\_peptide\_synthetase | BGC0002025 | NRP+Polyketide | 34.0 | 22.3 | 432.0 | 2.36e-120 |
| ARE67852.1 | AbsB2 | BGC0001492 | Polyketide | 33.0 | 27.4 | 431.0 | 2.46e-120 |
| AVV61985.1 | beta-ketoacyl\_synthase | BGC0001477 | NRP+Polyketide:Modular type I polyketide | 33.0 | 23.5 | 431.0 | 2.74e-120 |
| BAB69198.1 | modular\_polyketide\_synthase | BGC0000117 | Polyketide | 31.0 | 28.9 | 426.0 | 2.78e-120 |
| ANC94966.1 | AlmHI | BGC0001396 | Polyketide | 34.0 | 24.8 | 431.0 | 2.8e-120 |
| QKV49770.1 | PKS | BGC0002526 | Polyketide | 33.0 | 24.7 | 431.0 | 2.82e-120 |
| AFV30250.1 | polyketide\_synthase | BGC0000075 | Polyketide | 32.0 | 26.8 | 428.0 | 3.11e-120 |
| AAX98189.1 | polyketide\_synthase\_type\_I | BGC0000052 | Polyketide | 34.0 | 22.5 | 431.0 | 3.5e-120 |
| BBA84068.1 | type\_I\_polyketide\_synthase | BGC0001649 | Polyketide | 34.0 | 22.6 | 421.0 | 4.12e-120 |
| AAZ94388.1 | nodular\_polyketide\_synthase | BGC0000040 | Polyketide | 32.0 | 27.7 | 431.0 | 4.32e-120 |
| AVX51107.1 | nysB | BGC0001709 | Polyketide | 35.0 | 22.7 | 430.0 | 4.52e-120 |
| QWF78548.1 | 3-ketoacyl-CoA\_thiolase | BGC0002142 | Polyketide | 34.0 | 23.7 | 430.0 | 5.28e-120 |
| AAM54075.1 | polyketide\_synthase | BGC0000020 | Polyketide | 33.0 | 26.4 | 430.0 | 5.41e-120 |
| QBF51760.1 | type\_I\_polyketide\_synthase | BGC0001856 | Polyketide:Modular type I polyketide | 34.0 | 23.7 | 430.0 | 5.51e-120 |
| AAZ94387.1 | modular\_polyketide\_synthase | BGC0000040 | Polyketide | 33.0 | 24.9 | 430.0 | 5.54e-120 |
| ARS01476.1 | NcmAIV | BGC0001702 | NRP+Polyketide | 34.0 | 23.3 | 424.0 | 5.85e-120 |
| AEZ53953.1 | polyketide\_synthase | BGC0000144 | Polyketide:Modular type I polyketide | 34.0 | 23.4 | 428.0 | 5.96e-120 |
| AAF00959.1 | mcyD | BGC0001017 | NRP+Polyketide:Modular type I polyketide | 34.0 | 22.3 | 430.0 | 5.99e-120 |
| QBF51756.1 | type\_I\_polyketide\_synthase | BGC0001856 | Polyketide:Modular type I polyketide | 34.0 | 22.8 | 430.0 | 6.03e-120 |
| AXI91547.1 | FunP6 | BGC0001944 | Polyketide | 34.0 | 24.5 | 429.0 | 6.13e-120 |
| ANR02557.1 | LodP | BGC0001648 | Polyketide | 33.0 | 24.5 | 416.0 | 6.15e-120 |
| AEW95639.1 | type\_I\_polyketide\_synthase | BGC0002697 | NRP+Polyketide | 31.0 | 27.0 | 430.0 | 6.31e-120 |
| ACF35446.1 | mbcAII | BGC0000090 | Polyketide | 35.0 | 22.9 | 429.0 | 6.57e-120 |
| BAW35636.1 | modular\_polyketide\_synthase | BGC0002356 | Polyketide+Other | 33.0 | 27.0 | 430.0 | 6.85e-120 |
| ADH04680.1 | hybrid\_polyketide\_synthase/non-ribosomal\_peptide\_synthetase | BGC0001344 | NRP+Polyketide | 23.0 | 93.7 | 429.0 | 6.88e-120 |
| BAW35639.1 | modular\_polyketide\_synthase | BGC0002356 | Polyketide+Other | 35.0 | 22.5 | 430.0 | 7.27e-120 |
| BAF85838.1 | modular\_polyketide\_synthase | BGC0000109 | Polyketide | 34.0 | 22.6 | 429.0 | 7.42e-120 |
| BAG85032.1 | putative\_polyketide\_synthase | BGC0000086 | Polyketide | 33.0 | 24.2 | 415.0 | 7.63e-120 |
| AAX98192.1 | polyketide\_synthase\_type\_I | BGC0000052 | Polyketide | 35.0 | 22.7 | 429.0 | 7.77e-120 |
| CAO85893.1 | modular\_polyketide\_synthase\_NorA | BGC0000110 | Polyketide:Modular type I polyketide | 34.0 | 22.6 | 425.0 | 7.98e-120 |
| QUQ72353.1 | type\_I\_polyketide\_synthase | BGC0002349 | Polyketide+Saccharide | 36.0 | 22.1 | 429.0 | 8.01e-120 |
| UMP03509.1 | NmvAII | BGC0002649 | NRP+Polyketide | 35.0 | 23.2 | 422.0 | 9.1e-120 |
| ABB05104.1 | LipPks3 | BGC0001003 | NRP:Lipopeptide+Polyketide:Modular type I polyketide+Saccharide:Hybrid/tailoring saccharide | 32.0 | 27.6 | 429.0 | 9.35e-120 |
| QRI43531.1 | type\_I\_polyketide\_synthase | BGC0002454 | Polyketide | 33.0 | 23.4 | 429.0 | 9.5e-120 |
| ATY46587.1 | polyketide\_synthase | BGC0001666 | Polyketide | 35.0 | 22.2 | 429.0 | 9.63e-120 |
| AAC69330.1 | type\_I\_polyketide\_synthase\_PikAII | BGC0000094 | Polyketide:Modular type I polyketide+Saccharide:Hybrid/tailoring saccharide | 34.0 | 23.8 | 429.0 | 9.76e-120 |
| CQR60496.1 | Polyketide\_synthase,\_type\_I,\_modules:\_4,\_5\_and\_6 | BGC0001287 | Polyketide | 34.0 | 26.7 | 429.0 | 1.02e-119 |
| ANY10590.1 | polyketide\_synthase | BGC0001773 | Polyketide | 34.0 | 23.2 | 429.0 | 1.02e-119 |
| AKL64830.1 | polyketide\_synthase | BGC0002072 | Polyketide:Modular type I polyketide | 33.0 | 23.0 | 429.0 | 1.02e-119 |
| QWF78551.1 | hypothetical\_protein | BGC0002142 | Polyketide | 32.0 | 26.7 | 429.0 | 1.02e-119 |
| CAD29793.1 | polyketide\_synthase\_type\_I | BGC0001015 | NRP+Polyketide | 34.0 | 22.4 | 429.0 | 1.03e-119 |
| WP\_081238290.1 | type\_I\_polyketide\_synthase | BGC0002105 | Polyketide | 35.0 | 23.3 | 423.0 | 1.06e-119 |
| BAR73017.1 | putative\_PKS\_(KS-AT-KR-ACP-KS-AT-DH-KR-ACP) | BGC0001194 | Polyketide | 35.0 | 22.9 | 429.0 | 1.09e-119 |
| QIZ24099.1 | type\_I\_polyketide\_synthase | BGC0002540 | Polyketide | 33.0 | 27.1 | 423.0 | 1.1e-119 |
| ACY13414.1 | amino\_acid\_adenylation\_domain\_protein | BGC0001367 | NRP+Polyketide | 33.0 | 22.4 | 429.0 | 1.1e-119 |
| WP\_245661582.1 | polyketide\_synthase | BGC0001348 | Polyketide:Modular type I polyketide | 33.0 | 24.4 | 429.0 | 1.16e-119 |
| CBA11583.1 | polyketide\_synthase\_type\_I | BGC0001046 | NRP+Polyketide:Modular type I polyketide+Saccharide:Hybrid/tailoring saccharide | 31.0 | 26.9 | 429.0 | 1.16e-119 |
| AEP40934.1 | polyketide\_synthase\_type\_I | BGC0000021 | Polyketide | 34.0 | 23.3 | 429.0 | 1.17e-119 |
| AKL64829.1 | polyketide\_synthase | BGC0002072 | Polyketide:Modular type I polyketide | 34.0 | 22.6 | 429.0 | 1.2e-119 |
| AWC08655.1 | polyketide\_synthase\_type\_I | BGC0001662 | Polyketide | 35.0 | 22.5 | 429.0 | 1.23e-119 |
| AAS98784.1 | polyketide\_synthase | BGC0001001 | NRP+Polyketide | 32.0 | 24.9 | 407.0 | 1.31e-119 |
| WP\_051137607.1 | type\_I\_polyketide\_synthase | BGC0002011 | Polyketide | 36.0 | 22.0 | 428.0 | 1.36e-119 |
| QFU19841.1 | PKS | BGC0002431 | Polyketide+Saccharide | 34.0 | 25.2 | 429.0 | 1.39e-119 |
| ADB12488.1 | EpoA | BGC0000990 | NRP+Polyketide | 32.0 | 23.5 | 417.0 | 1.49e-119 |
| CAL58683.1 | polyketide\_synthase | BGC0000149 | Polyketide:Modular type I polyketide | 34.0 | 23.5 | 421.0 | 1.52e-119 |
| AAZ94391.1 | modular\_polyketide\_synthase | BGC0000040 | Polyketide | 34.0 | 23.4 | 425.0 | 1.57e-119 |
| AWC08657.1 | polyketide\_synthase\_type\_I | BGC0001662 | Polyketide | 35.0 | 22.5 | 429.0 | 1.63e-119 |
| QKG20163.1 | type\_I\_polyketide\_synthase | BGC0002124 | Polyketide | 35.0 | 22.3 | 429.0 | 1.63e-119 |
| BAW35614.1 | modular\_polyketide\_synthase | BGC0002357 | Polyketide+Other | 34.0 | 22.2 | 428.0 | 1.75e-119 |
| ABC84459.1 | NigAIV | BGC0000114 | Polyketide:Modular type I polyketide | 25.0 | 61.5 | 428.0 | 1.83e-119 |
| AAF62880.1 | EpoA | BGC0000991 | NRP+Polyketide | 32.0 | 23.5 | 417.0 | 2e-119 |
| AWC08660.1 | polyketide\_synthase\_type\_I | BGC0001662 | Polyketide | 32.0 | 27.2 | 429.0 | 2.01e-119 |
| AEZ54378.1 | PieA5 | BGC0000124 | Polyketide | 34.0 | 22.7 | 423.0 | 2.07e-119 |
| AEE88284.1 | CurF | BGC0000976 | NRP+Polyketide:Modular type I polyketide | 33.0 | 24.1 | 427.0 | 2.28e-119 |
| AUA09463.1 | Phenolphthiocerol\_synthesis\_polyketide\_synthase\_type\_I\_Pks15/1 | BGC0002291 | Polyketide | 33.0 | 27.0 | 425.0 | 2.36e-119 |
| UHY14130.1 | PKS\_I | BGC0002671 | Polyketide | 35.0 | 22.7 | 420.0 | 2.42e-119 |
| AKJ15835.1 | type\_I\_polyketide\_synthase | BGC0002735 | Polyketide+NRP | 34.0 | 22.7 | 423.0 | 2.45e-119 |
| TGZ15165.1 | hypothetical\_protein | BGC0002032 | Polyketide | 33.0 | 22.8 | 428.0 | 2.46e-119 |
| AAC01714.1 | RifE | BGC0000136 | Polyketide | 35.0 | 22.3 | 427.0 | 2.54e-119 |
| BAW35610.1 | modular\_polyketide\_synthase | BGC0002357 | Polyketide+Other | 34.0 | 22.6 | 428.0 | 2.56e-119 |
| AWH12937.1 | StmB | BGC0001784 | Polyketide | 35.0 | 22.2 | 428.0 | 2.76e-119 |
| QIQ28634.1 | Nbc38 | BGC0002541 | Other | 34.0 | 22.5 | 427.0 | 2.86e-119 |
| AAP42856.1 | NanA2 | BGC0000105 | Polyketide | 34.0 | 22.5 | 425.0 | 3.11e-119 |
| QSV12663.1 | AvmE | BGC0002456 | Polyketide+NRP | 25.0 | 62.4 | 427.0 | 3.35e-119 |
| UHY14129.1 | PKS\_I | BGC0002671 | Polyketide | 34.0 | 24.8 | 427.0 | 3.44e-119 |
| QSV12655.1 | AvmA | BGC0002456 | Polyketide+NRP | 34.0 | 23.8 | 427.0 | 3.73e-119 |
| AEC13080.1 | fosB | BGC0000060 | Polyketide | 33.0 | 24.8 | 422.0 | 3.82e-119 |
| AHE80995.1 | PieA5 | BGC0001169 | Polyketide:Modular type I polyketide | 36.0 | 22.9 | 422.0 | 3.85e-119 |
| sipP2 | Type\_I\_Modular\_PKS | BGC0001452 | Polyketide | 32.0 | 27.8 | 427.0 | 3.97e-119 |
| ABB88522.1 | polyketide\_synthase\_type\_I | BGC0000050 | Polyketide | 36.0 | 23.0 | 427.0 | 4.13e-119 |
| QNN81302.1 | IonAVI | BGC0002446 | Polyketide | 31.0 | 29.5 | 427.0 | 4.33e-119 |
| BAE93729.1 | type\_I\_polyketide\_synthase | BGC0000164 | Polyketide | 34.0 | 22.7 | 427.0 | 4.34e-119 |
| ABB52544.1 | putative\_type\_I\_polyketide\_synthase | BGC0000047 | Polyketide | 34.0 | 24.9 | 419.0 | 4.5e-119 |
| AQV04230.1 | SwnK | BGC0001794 | NRP+Polyketide | 32.0 | 23.6 | 426.0 | 4.61e-119 |
| ACB46194.1 | polyketide\_synthase | BGC0000989 | NRP+Polyketide | 31.0 | 27.5 | 422.0 | 4.68e-119 |
| BAC76492.1 | lankamycin\_synthase\_LkmAII | BGC0000085 | Polyketide | 32.0 | 27.0 | 427.0 | 4.78e-119 |
| AGZ15473.1 | putative\_type\_I\_polyketide\_synthase | BGC0001036 | NRP+Polyketide | 33.0 | 23.9 | 422.0 | 4.78e-119 |
| ACB46192.1 | polyketide\_synthase | BGC0000989 | NRP+Polyketide | 33.0 | 23.7 | 416.0 | 4.8e-119 |
| AAZ94390.1 | modular\_polyketide\_synthase | BGC0000040 | Polyketide | 33.0 | 23.4 | 427.0 | 4.88e-119 |
| ANZ22987.1 | ZinD | BGC0001828 | Polyketide | 33.0 | 25.0 | 426.0 | 5.11e-119 |
| AAG23262.1 | polyketide\_synthase\_extender\_modules\_8-10 | BGC0000148 | Polyketide | 31.0 | 27.2 | 427.0 | 5.23e-119 |
| AAZ94386.1 | modular\_polyketide\_synthase | BGC0000040 | Polyketide | 34.0 | 23.3 | 426.0 | 5.27e-119 |
| AKG06376.1 | polyketide\_synthase\_type\_1 | BGC0001830 | Polyketide | 34.0 | 22.8 | 422.0 | 5.4e-119 |
| AJW65407.1 | type\_I\_modular\_polyketide\_synthase | BGC0001195 | NRP+Polyketide | 33.0 | 24.6 | 427.0 | 5.61e-119 |
| ACB37741.1 | putative\_type\_I\_polyketide\_synthase | BGC0000162 | Polyketide | 34.0 | 22.4 | 427.0 | 5.83e-119 |
| BAJ16467.1 | polyketide\_synthase | BGC0000058 | Polyketide | 34.0 | 22.3 | 427.0 | 5.98e-119 |
| BCK51646.1 | modular\_polyketide\_synthase | BGC0002520 | Polyketide | 34.0 | 24.8 | 426.0 | 6.24e-119 |
| CRI73798.1 | CongD\_protein | BGC0001215 | NRP | 34.0 | 23.0 | 424.0 | 6.49e-119 |
| BAA20102.2 | 6-methylsalicylic\_acid\_synthase | BGC0001276 | Polyketide | 33.0 | 25.4 | 421.0 | 6.53e-119 |
| QEA08889.1 | JenA3 | BGC0002559 | Polyketide | 35.0 | 22.5 | 426.0 | 6.93e-119 |
| ACR33077.1 | polyketide\_synthase | BGC0000017 | Alkaloid+Polyketide:Modular type I polyketide | 29.0 | 29.5 | 424.0 | 7.09e-119 |
| AFV96142.1 | polyketide\_synthase | BGC0001064 | Polyketide:Modular type I polyketide+Polyketide:Type III polyketide | 32.0 | 23.5 | 424.0 | 7.13e-119 |
| ARU81122.1 | CylH | BGC0001566 | Polyketide | 32.0 | 23.5 | 424.0 | 7.13e-119 |
| AFU82615.1 | polyketide\_synthase | BGC0000998 | NRP+Polyketide | 35.0 | 22.1 | 416.0 | 7.18e-119 |
| BBA84067.1 | type\_I\_polyketide\_synthase | BGC0001649 | Polyketide | 35.0 | 24.1 | 422.0 | 7.2e-119 |
| AKG06375.1 | polyketide\_synthase\_type\_1 | BGC0001830 | Polyketide | 33.0 | 23.9 | 426.0 | 7.61e-119 |
| TXD00026.1 | SDR\_family\_NAD(P)-dependent\_oxidoreductase | BGC0001877 | Polyketide | 34.0 | 22.6 | 426.0 | 8.62e-119 |
| BCB17032.1 | modular\_polyketide\_synthase | BGC0002523 | NRP | 32.0 | 23.8 | 426.0 | 8.77e-119 |
| AAT70101.1 | CurF | BGC0001165 | NRP+Polyketide:Modular type I polyketide | 33.0 | 24.1 | 426.0 | 8.8e-119 |
| BAQ25513.1 | type\_I\_polyketide\_synthase | BGC0001288 | Polyketide | 33.0 | 22.6 | 426.0 | 8.84e-119 |
| AHH99926.1 | PKS\_I | BGC0000002 | Polyketide | 32.0 | 26.8 | 426.0 | 8.89e-119 |
| QNN81300.1 | IonAIV | BGC0002446 | Polyketide | 32.0 | 26.7 | 426.0 | 9.69e-119 |
| ABC87510.1 | polyketide\_synthase | BGC0001011 | NRP+Polyketide | 34.0 | 22.3 | 426.0 | 1e-118 |
| ctg1\_orf21 |  | BGC0001013 | NRP+Polyketide | 34.0 | 22.3 | 426.0 | 1e-118 |
| QQZ01583.1 | PKS | BGC0002498 | Other | 32.0 | 26.7 | 426.0 | 1.07e-118 |
| BAQ21939.1 | putative\_type\_I\_polyketide\_synthase | BGC0001204 | Polyketide | 35.0 | 23.0 | 426.0 | 1.08e-118 |
| BAO66541.1 | type\_I\_polyketide\_synthase | BGC0000042 | Polyketide | 32.0 | 27.6 | 422.0 | 1.1e-118 |
| AAX98190.1 | polyketide\_synthase\_type\_I | BGC0000052 | Polyketide | 32.0 | 24.3 | 425.0 | 1.17e-118 |
| AHH25595.1 | PKS | BGC0000957 | NRP+Polyketide | 32.0 | 26.8 | 425.0 | 1.18e-118 |
| AAP42857.1 | NanA3 | BGC0000105 | Polyketide | 35.0 | 22.5 | 426.0 | 1.2e-118 |
| AXI91551.1 | FunP2 | BGC0001944 | Polyketide | 33.0 | 25.5 | 416.0 | 1.22e-118 |
| ABI91470.1 | beta-ketoacyl\_synthase | BGC0001094 | NRP+Polyketide | 34.0 | 22.2 | 424.0 | 1.27e-118 |
| QSE03602.1 | LcmA | BGC0002333 | Polyketide | 35.0 | 23.4 | 425.0 | 1.38e-118 |
| CAQ43079.1 | polyketide\_synthase | BGC0000970 | NRP+Polyketide:Modular type I polyketide | 34.0 | 22.9 | 421.0 | 1.43e-118 |
| BAG85029.1 | putative\_polyketide\_synthase | BGC0000086 | Polyketide | 33.0 | 23.0 | 418.0 | 1.46e-118 |
| AJW65410.1 | type\_I\_modular\_polyketide\_synthase | BGC0001195 | NRP+Polyketide | 34.0 | 22.7 | 420.0 | 1.5e-118 |
| AAF26919.1 | polyketide\_synthase | BGC0000988 | NRP+Polyketide | 33.0 | 23.7 | 414.0 | 1.54e-118 |
| AKD43769.1 | HerA2 | BGC0001349 | NRP+Polyketide | 33.0 | 22.7 | 405.0 | 1.73e-118 |
| ARM20279.1 | polyketide\_synthase | BGC0001523 | Polyketide | 33.0 | 23.6 | 425.0 | 1.81e-118 |
| CAQ64689.1 | lasalocid\_modular\_polyketide\_synthase | BGC0000087 | Polyketide | 33.0 | 23.0 | 417.0 | 1.95e-118 |
| BAC68127.1 | modular\_polyketide\_synthase | BGC0000059 | Polyketide | 31.0 | 29.1 | 420.0 | 1.97e-118 |
| BAC57032.1 | protomycinolide\_IV\_synthase\_5 | BGC0000102 | Polyketide | 35.0 | 22.8 | 422.0 | 2.04e-118 |
| ALP32042.1 | CycB | BGC0001293 | Polyketide | 35.0 | 22.3 | 422.0 | 2.07e-118 |
| QGJ79675.1 | Polyketide\_synthase | BGC0002552 | Polyketide | 35.0 | 22.0 | 425.0 | 2.22e-118 |
| AEZ53947.1 | polyketide\_synthase | BGC0000144 | Polyketide:Modular type I polyketide | 33.0 | 22.8 | 424.0 | 2.31e-118 |
| CAM00062.1 | EryAI\_Erythromycin\_polyketide\_synthase\_modules\_1\_and\_2 | BGC0000055 | Polyketide:Modular type I polyketide+Saccharide:Hybrid/tailoring saccharide | 33.0 | 23.2 | 424.0 | 2.31e-118 |
| AAQ84145.1 | Plm5 | BGC0000123 | Polyketide | 34.0 | 23.7 | 420.0 | 2.36e-118 |
| AKL64831.1 | polyketide\_synthase | BGC0002072 | Polyketide:Modular type I polyketide | 34.0 | 22.4 | 425.0 | 2.57e-118 |
| AQT01384.1 | SgnS0 | BGC0001690 | Polyketide | 33.0 | 23.9 | 419.0 | 2.59e-118 |
| BAB69194.1 | modular\_polyketide\_synthase | BGC0000117 | Polyketide | 35.0 | 22.7 | 424.0 | 2.62e-118 |
| AGM05536.1 | type\_I\_polyketide\_synthase | BGC0002098 | Polyketide | 32.0 | 26.7 | 420.0 | 2.73e-118 |
| BAH02268.1 | polyketide\_synthase | BGC0000126 | Polyketide | 34.0 | 22.5 | 425.0 | 2.84e-118 |
| QOD94996.1 | PldAI | BGC0002102 | Polyketide | 34.0 | 22.5 | 425.0 | 2.84e-118 |
| BAJ16469.1 | polyketide\_synthase | BGC0000058 | Polyketide | 31.0 | 27.0 | 422.0 | 2.85e-118 |
| AZF85917.1 | type\_I\_polyketide\_synthase | BGC0001963 | NRP+Polyketide | 33.0 | 25.1 | 424.0 | 3.56e-118 |
| AZF85946.1 | type\_I\_polyketide\_synthase | BGC0001963 | NRP+Polyketide | 33.0 | 22.9 | 424.0 | 3.7e-118 |
| ACY06290.1 | type\_I\_polyketide\_synthase | BGC0001042 | NRP+Polyketide | 33.0 | 23.1 | 424.0 | 3.75e-118 |
| AAF71774.1 | nysA | BGC0000115 | Polyketide:Modular type I polyketide+Saccharide:Hybrid/tailoring saccharide | 31.0 | 27.0 | 412.0 | 3.91e-118 |
| AAZ77697.1 | ChlA4 | BGC0000036 | Polyketide:Modular type I polyketide+Polyketide:Iterative type I polyketide+Saccharide:Oligosaccharide | 34.0 | 22.3 | 419.0 | 3.96e-118 |
| AKD43753.1 | HerB | BGC0001349 | NRP+Polyketide | 31.0 | 26.8 | 424.0 | 3.98e-118 |
| BCK51637.1 | modular\_modular\_polyketide\_synthase | BGC0002520 | Polyketide | 35.0 | 22.5 | 419.0 | 3.99e-118 |
| AAU93806.2 | polyketide\_synthase\_modules\_3\_and\_4 | BGC0000054 | Polyketide | 33.0 | 24.2 | 424.0 | 4e-118 |
| ACN69989.1 | polyketide\_synthase | BGC0000079 | Polyketide | 34.0 | 23.0 | 424.0 | 4.04e-118 |
| AWR88405.1 | putative\_phosphopantetheine-binding\_domain-\_containing\_prot\_ein | BGC0001522 | Polyketide | 34.0 | 23.9 | 424.0 | 4.3e-118 |
| ABB88519.1 | polyketide\_synthase\_type\_I | BGC0000050 | Polyketide | 33.0 | 26.8 | 424.0 | 4.88e-118 |
| AEZ64504.1 | Herc | BGC0001065 | Polyketide | 34.0 | 22.1 | 424.0 | 5.03e-118 |
| BAC68129.1 | modular\_polyketide\_synthase | BGC0000059 | Polyketide | 34.0 | 22.1 | 424.0 | 5.19e-118 |
| AEP40939.1 | polyketide\_synthase\_type\_I | BGC0000021 | Polyketide | 34.0 | 22.8 | 418.0 | 5.51e-118 |
| QKV49767.1 | PKS | BGC0002526 | Polyketide | 25.0 | 60.5 | 423.0 | 5.96e-118 |
| BAB69192.1 | modular\_polyketide\_synthase | BGC0000117 | Polyketide | 35.0 | 22.5 | 424.0 | 6.17e-118 |
| BCK51642.1 | modular\_polyketide\_synthase | BGC0002520 | Polyketide | 35.0 | 23.1 | 423.0 | 6.25e-118 |
| ctg1\_orf8 |  | BGC0001109 | NRP+Polyketide | 28.0 | 29.9 | 324.0 | 7.41e-88 |
| CAC20930.1 | PimS0\_protein | BGC0000125 | Polyketide | 33.0 | 23.9 | 418.0 | 6.66e-118 |
| ACO94488.1 | polyketide\_synthase\_type\_I | BGC0000097 | Polyketide:Modular type I polyketide | 32.0 | 26.8 | 423.0 | 6.75e-118 |
| BAP34740.1 | type\_I\_polyketide\_synthase | BGC0000078 | Polyketide | 32.0 | 24.7 | 417.0 | 7.35e-118 |
| ABK32291.1 | JerE | BGC0000080 | Polyketide | 34.0 | 22.7 | 422.0 | 8.06e-118 |
| ALV82341.1 | borrelidin\_type\_I\_polyketide\_synthase | BGC0001533 | Polyketide | 32.0 | 24.5 | 415.0 | 8.14e-118 |
| BAW35653.1 | modular\_polyketide\_synthase | BGC0002355 | Polyketide+Other | 34.0 | 23.0 | 423.0 | 8.42e-118 |
| AAB66504.1 | tylactone\_synthase\_starter\_module\_and\_modules\_1\_&\_2 | BGC0000166 | Polyketide | 34.0 | 24.1 | 423.0 | 8.63e-118 |
| QQZ01586.1 | PKS | BGC0002498 | Other | 31.0 | 26.7 | 423.0 | 8.7e-118 |
| AAX98191.1 | polyketide\_synthase\_type\_I | BGC0000052 | Polyketide | 35.0 | 22.6 | 423.0 | 8.78e-118 |
| CAE45670.1 | borrelidin\_polyketide\_synthase,\_type\_I | BGC0000031 | Polyketide:Modular type I polyketide | 32.0 | 24.5 | 415.0 | 9.4e-118 |
| CCP20051.1 | divM\_protein | BGC0001119 | Polyketide:Modular type I polyketide | 35.0 | 22.3 | 422.0 | 9.47e-118 |
| WP\_048832936.1 | polyketide\_synthase | BGC0001348 | Polyketide:Modular type I polyketide | 34.0 | 22.7 | 423.0 | 9.51e-118 |
| ABB86409.1 | GelB | BGC0000067 | Polyketide | 33.0 | 23.7 | 422.0 | 1.11e-117 |
| QKV49790.1 | PKS | BGC0002526 | Polyketide | 34.0 | 22.4 | 419.0 | 1.15e-117 |
| TGZ15168.1 | hypothetical\_protein | BGC0002032 | Polyketide | 34.0 | 22.9 | 423.0 | 1.17e-117 |
| EHK80171.1 | modular\_polyketide\_synthase | BGC0001447 | Polyketide | 32.0 | 23.2 | 417.0 | 1.34e-117 |
| ANC94963.1 | AlmHIV | BGC0001396 | Polyketide | 34.0 | 24.7 | 414.0 | 1.41e-117 |
| BBA66513.1 | type\_I\_polyketide\_synthase | BGC0001495 | Polyketide | 34.0 | 23.6 | 422.0 | 1.43e-117 |
| ctg1\_orf11 |  | BGC0000053 | Polyketide | 35.0 | 22.6 | 419.0 | 1.54e-117 |
| AUO16400.1 | polyketide\_synthase | BGC0001700 | Polyketide | 34.0 | 22.8 | 422.0 | 1.55e-117 |
| QQZ01628.1 | PKS | BGC0002497 | Other | 33.0 | 23.5 | 422.0 | 1.58e-117 |
| QIE07131.1 | OvmM | BGC0001719 | Polyketide | 35.0 | 22.3 | 421.0 | 1.63e-117 |
| TXD00266.1 | SDR\_family\_NAD(P)-dependent\_oxidoreductase | BGC0001877 | Polyketide | 31.0 | 27.3 | 422.0 | 1.73e-117 |
| CAE46843.1 | Type\_I\_modular\_polyketide\_synthase | BGC0000103 | Polyketide | 33.0 | 23.4 | 422.0 | 1.76e-117 |
| AAQ82568.1 | FscD | BGC0000034 | NRP+Polyketide | 35.0 | 22.7 | 422.0 | 2.12e-117 |
| ACR50773.1 | polyketide\_synthase | BGC0000163 | Polyketide | 34.0 | 22.2 | 422.0 | 2.14e-117 |
| AAX98188.1 | polyketide\_synthase\_type\_I | BGC0000052 | Polyketide | 34.0 | 22.7 | 422.0 | 2.16e-117 |
| QKW94294.1 | short-chain\_dehydrogenase/reductase\_SDR | BGC0002342 | NRP+Polyketide | 33.0 | 23.7 | 398.0 | 2.21e-117 |
| AAG23266.1 | polyketide\_synthase\_extender\_modules\_3-4 | BGC0000148 | Polyketide | 34.0 | 22.6 | 421.0 | 2.21e-117 |
| QCF28926.1 | type\_I\_polyketide\_synthase | BGC0002308 | Alkaloid+Polyketide | 31.0 | 26.8 | 419.0 | 2.28e-117 |
| CAE46850.1 | Type\_I\_modular\_polyketide\_synthase | BGC0000103 | Polyketide | 32.0 | 23.7 | 420.0 | 2.31e-117 |
| QWF78544.1 | 3-ketoacyl-CoA\_thiolase | BGC0002142 | Polyketide | 34.0 | 22.3 | 422.0 | 2.32e-117 |
| AAS79462.1 | polyketide\_synthase\_subunit | BGC0000035 | Polyketide | 34.0 | 22.7 | 414.0 | 2.35e-117 |
| EAU32819.1 | 6-methylsalicylic\_acid\_synthase | BGC0000160 | Polyketide | 33.0 | 25.4 | 416.0 | 2.58e-117 |
| AKD43768.1 | HerA1 | BGC0001349 | NRP+Polyketide | 34.0 | 22.6 | 421.0 | 2.6e-117 |
| AAG13918.1 | megalomicin\_6-deoxyerythronolide\_B\_synthase\_2 | BGC0000092 | Polyketide | 32.0 | 26.7 | 421.0 | 2.63e-117 |
| QGA70079.1 | type\_I\_polyketide\_synthase | BGC0002517 | Polyketide | 34.0 | 22.5 | 421.0 | 2.65e-117 |
| WP\_102918845.1 | type\_I\_polyketide\_synthase | BGC0002104 | NRP+Polyketide | 33.0 | 26.6 | 422.0 | 2.68e-117 |
| AGM05532.1 | beta-ketoacyl\_synthase | BGC0002098 | Polyketide | 35.0 | 22.7 | 421.0 | 2.98e-117 |
| ANZ22991.1 | ZinG | BGC0001828 | Polyketide | 33.0 | 22.7 | 419.0 | 3.04e-117 |
| AAS79459.1 | polyketide\_synthase\_subunit | BGC0000035 | Polyketide | 34.0 | 22.7 | 421.0 | 3.29e-117 |
| CQR60493.1 | Polyketide\_synthase,\_type\_I,\_modules:\_9\_and\_10 | BGC0001287 | Polyketide | 32.0 | 26.7 | 421.0 | 3.34e-117 |
| BAQ21946.1 | putative\_type\_I\_polyketide\_synthase | BGC0001204 | Polyketide | 34.0 | 22.6 | 421.0 | 3.47e-117 |
| AHF22854.1 | MarL | BGC0000091 | Polyketide | 33.0 | 23.3 | 419.0 | 3.63e-117 |
| BCK51648.1 | modular\_polyketide\_synthase | BGC0002520 | Polyketide | 34.0 | 24.6 | 420.0 | 3.78e-117 |
| AWH12664.1 | RmpE2 | BGC0001759 | Polyketide | 33.0 | 26.9 | 419.0 | 3.8e-117 |
| ANZ22986.1 | ZinC | BGC0001828 | Polyketide | 35.0 | 22.7 | 418.0 | 3.83e-117 |
| AFL48525.1 | laidlomycin\_polyketide\_synthase\_(loading\_module\_and\_module\_1) | BGC0000084 | Polyketide | 34.0 | 22.3 | 419.0 | 4.14e-117 |
| ATL73033.1 | type\_I\_modular\_polyketide\_synthase | BGC0001807 | NRP+Polyketide | 32.0 | 27.0 | 421.0 | 4.15e-117 |
| BAQ21940.1 | putative\_Type\_I\_polyketide\_synthase | BGC0001204 | Polyketide | 31.0 | 29.4 | 421.0 | 4.4e-117 |
| MBA5221219.1 | aminotransferase\_class\_I/II-fold\_pyridoxal\_phosphate-dependent\_enzyme | BGC0002090 | NRP+Polyketide:Modular type I polyketide | 35.0 | 22.4 | 419.0 | 4.53e-117 |
| BAB69196.1 | modular\_polyketide\_synthase | BGC0000117 | Polyketide | 32.0 | 27.2 | 420.0 | 4.58e-117 |
| ARS01473.1 | NcmAI | BGC0001702 | NRP+Polyketide | 34.0 | 23.2 | 421.0 | 4.65e-117 |
| AQZ37094.1 | polyketide\_synthase | BGC0001511 | Polyketide | 34.0 | 24.6 | 412.0 | 4.71e-117 |
| ANC94962.1 | AlmHV | BGC0001396 | Polyketide | 33.0 | 23.3 | 408.0 | 4.76e-117 |
| AAQ82561.1 | FscA | BGC0000034 | NRP+Polyketide | 35.0 | 22.4 | 414.0 | 4.85e-117 |
| QKG20144.1 | type\_I\_polyketide\_synthase | BGC0002124 | Polyketide | 32.0 | 26.8 | 415.0 | 5.15e-117 |
| CAE02606.1 | polyketide\_synthase\_type\_I | BGC0000024 | Polyketide:Modular type I polyketide | 33.0 | 22.6 | 418.0 | 5.37e-117 |
| WP\_102918843.1 | SDR\_family\_NAD(P)-dependent\_oxidoreductase | BGC0002104 | NRP+Polyketide | 32.0 | 27.0 | 417.0 | 5.49e-117 |
| AAS79460.1 | polyketide\_synthase\_subunit | BGC0000035 | Polyketide | 33.0 | 23.0 | 416.0 | 6.14e-117 |
| ABB52545.1 | putative\_type\_I\_polyketide\_synthase | BGC0000047 | Polyketide | 33.0 | 23.0 | 408.0 | 6.38e-117 |
| ACO94498.1 | polyketide\_synthase\_type\_I | BGC0000097 | Polyketide:Modular type I polyketide | 33.0 | 26.6 | 416.0 | 6.77e-117 |
| AAZ77699.1 | ChlA6 | BGC0000036 | Polyketide:Modular type I polyketide+Polyketide:Iterative type I polyketide+Saccharide:Oligosaccharide | 33.0 | 22.4 | 412.0 | 7.16e-117 |
| ABV97153.1 | Beta-ketoacyl\_synthase | BGC0000137 | Polyketide | 31.0 | 26.8 | 415.0 | 7.47e-117 |
| OAP25820.1 | Erythronolide\_synthase,\_modules\_1\_and\_2 | BGC0001658 | Polyketide | 34.0 | 22.2 | 412.0 | 7.49e-117 |
| WP\_102918844.1 | type\_I\_polyketide\_synthase | BGC0002104 | NRP+Polyketide | 32.0 | 26.8 | 419.0 | 8.13e-117 |
| QBL56210.1 | PKS | BGC0002376 | Polyketide | 33.0 | 26.8 | 420.0 | 8.48e-117 |
| QWF78545.1 | 3-ketoacyl-CoA\_thiolase | BGC0002142 | Polyketide | 35.0 | 22.3 | 420.0 | 8.68e-117 |
| QNN81303.1 | IonAVII | BGC0002446 | Polyketide | 32.0 | 27.0 | 418.0 | 9.28e-117 |
| BAW35652.1 | modular\_polyketide\_synthase | BGC0002355 | Polyketide+Other | 32.0 | 23.8 | 419.0 | 9.88e-117 |
| SAI82900.1 | HrnB;\_Macrolactam\_polyketide\_synthase\_type\_I;\_modules\_loading,\_1-2 | BGC0002101 | Polyketide | 32.0 | 27.4 | 419.0 | 9.96e-117 |
| WP\_055480220.1 | type\_I\_polyketide\_synthase | BGC0001653 | Polyketide | 35.0 | 23.0 | 419.0 | 9.97e-117 |
| BAW35641.1 | modular\_polyketide\_synthase | BGC0002356 | Polyketide+Other | 34.0 | 22.7 | 420.0 | 1.01e-116 |
| ARW71484.1 | type\_I\_PKS\_module\_3 | BGC0001812 | Polyketide | 33.0 | 23.5 | 415.0 | 1.05e-116 |
| ABJ97439.1 | MerC | BGC0001012 | NRP+Polyketide | 32.0 | 27.2 | 420.0 | 1.11e-116 |
| CAE46851.1 | Type\_I\_modular\_polyketide\_synthase | BGC0000103 | Polyketide | 32.0 | 23.6 | 420.0 | 1.18e-116 |
| QGA70100.1 | type\_I\_polyketide\_synthase | BGC0002517 | Polyketide | 34.0 | 23.3 | 411.0 | 1.26e-116 |
| QWF78546.1 | Narbonolide/10-deoxymethynolide\_synthase\_PikA2,\_modules\_3\_and\_4 | BGC0002142 | Polyketide | 31.0 | 26.5 | 419.0 | 1.35e-116 |
| AEZ53951.1 | polyketide\_synthase | BGC0000144 | Polyketide:Modular type I polyketide | 33.0 | 23.3 | 412.0 | 1.39e-116 |
| QLD23836.1 | SDR\_family\_NAD(P)-dependent\_oxidoreductase | BGC0002086 | Polyketide | 33.0 | 23.5 | 415.0 | 1.41e-116 |
| ANC94965.1 | AlmHII | BGC0001396 | Polyketide | 32.0 | 23.1 | 415.0 | 1.43e-116 |
| AGY30677.1 | Ann5 | BGC0001298 | Polyketide | 34.0 | 24.2 | 419.0 | 1.44e-116 |
| ctg1\_orf254 |  | BGC0001200 | Polyketide | 34.0 | 22.2 | 419.0 | 1.46e-116 |
| UHH90012.1 | VicP4 | BGC0002634 | Polyketide+NRP+Other | 34.0 | 22.9 | 419.0 | 1.47e-116 |
| ADX66470.1 | ScnS0 | BGC0000108 | Polyketide | 33.0 | 23.7 | 413.0 | 1.67e-116 |
| AAO23334.1 | NcpB | BGC0000397 | NRP | 26.0 | 39.6 | 419.0 | 1.75e-116 |
| AMB48441.1 | polyketide\_synthase | BGC0001357 | Polyketide | 33.0 | 23.5 | 416.0 | 1.77e-116 |
| AAC69331.1 | type\_I\_polyketide\_synthase\_PikAIII | BGC0000094 | Polyketide:Modular type I polyketide+Saccharide:Hybrid/tailoring saccharide | 33.0 | 23.4 | 410.0 | 1.78e-116 |
| AWC08659.1 | polyketide\_synthase\_type\_I | BGC0001662 | Polyketide | 34.0 | 22.6 | 419.0 | 1.84e-116 |
| AFL48526.1 | laidlomycin\_polyketide\_synthase\_(module\_2) | BGC0000084 | Polyketide | 33.0 | 22.6 | 416.0 | 1.92e-116 |
| CBA11584.1 | polyketide\_synthase\_type\_I | BGC0001046 | NRP+Polyketide:Modular type I polyketide+Saccharide:Hybrid/tailoring saccharide | 34.0 | 22.6 | 419.0 | 2.03e-116 |
| WP\_015031691.1 | type\_I\_polyketide\_synthase | BGC0001819 | Polyketide | 34.0 | 23.1 | 405.0 | 2.25e-116 |
| ALJ49910.1 | TlmH | BGC0001237 | Polyketide | 34.0 | 22.4 | 416.0 | 2.26e-116 |
| QRI43527.1 | type\_I\_polyketide\_synthase | BGC0002454 | Polyketide | 34.0 | 24.5 | 418.0 | 2.31e-116 |
| AXI91549.1 | FunP4 | BGC0001944 | Polyketide | 35.0 | 22.3 | 418.0 | 2.32e-116 |
| AFL48532.1 | laidlomycin\_polyketide\_synthase\_(module\_11\_and\_module\_12) | BGC0000084 | Polyketide | 35.0 | 22.4 | 418.0 | 2.34e-116 |
| QBF51755.1 | type\_I\_polyketide\_synthase | BGC0001856 | Polyketide:Modular type I polyketide | 34.0 | 22.0 | 418.0 | 2.34e-116 |
| QWF78552.1 | 3-ketoacyl-CoA\_thiolase | BGC0002142 | Polyketide | 34.0 | 22.5 | 419.0 | 2.39e-116 |
| BAK64638.1 | polyketide\_synthase | BGC0000135 | Polyketide | 32.0 | 25.5 | 418.0 | 2.39e-116 |
| ABY21540.1 | AngAIII | BGC0000018 | Polyketide | 34.0 | 23.3 | 418.0 | 2.48e-116 |
| BAC68126.1 | modular\_polyketide\_synthase | BGC0000059 | Polyketide | 35.0 | 22.5 | 418.0 | 2.65e-116 |
| ACB37740.1 | putative\_type\_I\_polyketide\_synthase | BGC0000162 | Polyketide | 33.0 | 23.4 | 418.0 | 2.68e-116 |
| BAW35640.1 | modular\_polyketide\_synthase | BGC0002356 | Polyketide+Other | 32.0 | 27.1 | 417.0 | 2.76e-116 |
| SCO70310.1 | Type\_I\_polyketide\_synthase | BGC0001433 | Polyketide:Modular type I polyketide | 32.0 | 27.2 | 416.0 | 2.81e-116 |
| ANR02553.1 | LodL | BGC0001648 | Polyketide | 32.0 | 26.7 | 417.0 | 3.1e-116 |
| AGM05535.1 | modular\_polyketide\_synthase | BGC0002098 | Polyketide | 32.0 | 26.7 | 418.0 | 3.13e-116 |
| AZH23793.1 | MgcK | BGC0001970 | NRP+Polyketide | 31.0 | 24.4 | 403.0 | 3.25e-116 |
| AEH42491.1 | polyketide\_synthase | BGC0000032 | Polyketide | 33.0 | 22.5 | 417.0 | 3.34e-116 |
| AAP42874.1 | NanA8 | BGC0000105 | Polyketide | 34.0 | 22.4 | 417.0 | 3.73e-116 |
| ANH11409.1 | SceN | BGC0001770 | Polyketide | 34.0 | 23.3 | 417.0 | 3.97e-116 |
| QFU19827.1 | PKS | BGC0002431 | Polyketide+Saccharide | 33.0 | 23.1 | 413.0 | 3.97e-116 |
| WP\_245661588.1 | hypothetical\_protein | BGC0001348 | Polyketide:Modular type I polyketide | 33.0 | 25.2 | 417.0 | 4.28e-116 |
| CAQ52623.1 | type\_I\_polyketide\_synthase,\_module\_6 | BGC0001066 | Polyketide:Modular type I polyketide | 31.0 | 27.1 | 415.0 | 4.41e-116 |
| AEU17898.1 | putative\_type\_I\_PKS | BGC0001072 | Saccharide+Polyketide:Modular type I polyketide+Polyketide:Type II polyketide+Other:Aminocoumarin | 32.0 | 23.3 | 405.0 | 4.44e-116 |
| CAQ52626.1 | type\_I\_polyketide\_synthase,\_loading\_module\_and\_modules\_1-3 | BGC0001066 | Polyketide:Modular type I polyketide | 34.0 | 23.2 | 417.0 | 4.53e-116 |
| ACY06287.1 | type\_I\_polyketide\_synthase | BGC0001042 | NRP+Polyketide | 35.0 | 22.5 | 417.0 | 4.63e-116 |
| AWC08656.1 | polyketide\_synthase\_type\_I | BGC0001662 | Polyketide | 32.0 | 27.1 | 417.0 | 4.73e-116 |
| AHN85651.1 | Phn2 | BGC0000122 | Polyketide:Modular type I polyketide | 34.0 | 24.5 | 417.0 | 4.89e-116 |
| BAE93722.1 | type\_I\_polyketide\_synthase | BGC0000164 | Polyketide | 34.0 | 23.5 | 417.0 | 5.19e-116 |
| ABV83229.1 | CppB | BGC0000116 | Polyketide | 34.0 | 22.7 | 416.0 | 5.64e-116 |
| QCP68967.1 | VatT | BGC0002296 | NRP+Polyketide | 24.0 | 59.0 | 415.0 | 5.73e-116 |
| QQZ01627.1 | PKS | BGC0002497 | Other | 33.0 | 22.9 | 417.0 | 6.12e-116 |
| ACC40922.1 | polyketide\_synthase,\_Pks8 | BGC0001665 | Polyketide | 32.0 | 26.7 | 414.0 | 6.21e-116 |
| CAQ34929.1 | putative\_polyketide\_synthase | BGC0000986 | NRP+Polyketide | 34.0 | 22.5 | 395.0 | 6.39e-116 |
| QQZ01582.1 | PKS | BGC0002498 | Other | 34.0 | 22.7 | 416.0 | 6.4e-116 |
| CAL58687.1 | polyketide\_synthase | BGC0000149 | Polyketide:Modular type I polyketide | 34.0 | 22.7 | 416.0 | 6.69e-116 |
| BAO66542.1 | type\_I\_polyketide\_synthase | BGC0000042 | Polyketide | 31.0 | 27.2 | 417.0 | 7.09e-116 |
| BAW35613.1 | modular\_polyketide\_synthase | BGC0002357 | Polyketide+Other | 32.0 | 27.2 | 417.0 | 7.22e-116 |
| AAM81585.1 | putative\_type\_I\_polyketide\_synthase | BGC0000047 | Polyketide | 32.0 | 23.1 | 413.0 | 7.62e-116 |
| AKL64833.1 | polyketide\_synthase | BGC0002072 | Polyketide:Modular type I polyketide | 31.0 | 26.5 | 417.0 | 8.05e-116 |
| WP\_032929422.1 | type\_I\_polyketide\_synthase | BGC0002106 | Polyketide | 34.0 | 23.1 | 417.0 | 8.08e-116 |
| TMU97090.1 | SDR\_family\_NAD(P)-dependent\_oxidoreductase | BGC0002038 | Polyketide | 33.0 | 22.8 | 412.0 | 8.25e-116 |
| AAO06917.1 | GdmAII | BGC0000066 | Polyketide | 33.0 | 23.7 | 416.0 | 8.3e-116 |
| QGA70098.1 | type\_I\_polyketide\_synthase | BGC0002517 | Polyketide | 35.0 | 22.6 | 403.0 | 9.06e-116 |
| QGA70099.1 | type\_I\_polyketide\_synthase | BGC0002517 | Polyketide | 33.0 | 23.2 | 416.0 | 9.2e-116 |
| QBL56181.1 | PKS | BGC0002376 | Polyketide | 32.0 | 26.9 | 416.0 | 9.22e-116 |
| AKG06379.1 | polyketide\_synthase\_type\_1 | BGC0001830 | Polyketide | 34.0 | 23.0 | 411.0 | 9.62e-116 |
| AZF85941.1 | type\_I\_polyketide\_synthase | BGC0001963 | NRP+Polyketide | 30.0 | 26.8 | 416.0 | 1.03e-115 |
| WP\_033261452.1 | type\_I\_polyketide\_synthase | BGC0002009 | Polyketide | 32.0 | 27.2 | 416.0 | 1.09e-115 |
| BAW35658.1 | modular\_polyketide\_synthase | BGC0002355 | Polyketide+Other | 34.0 | 22.7 | 416.0 | 1.1e-115 |
| AAF86393.1 | FkbB | BGC0000994 | NRP+Polyketide | 33.0 | 23.4 | 416.0 | 1.1e-115 |
| BAG23200.1 | putative\_type-I\_PKS | BGC0002673 | Polyketide+Alkaloid | 34.0 | 23.4 | 416.0 | 1.12e-115 |
| CAJ88186.1 | Type\_I\_modular\_polyketide\_synthase | BGC0000151 | Polyketide:Modular type I polyketide+Saccharide:Hybrid/tailoring saccharide | 34.0 | 23.3 | 416.0 | 1.14e-115 |
| ARS01477.1 | NcmAV | BGC0001702 | NRP+Polyketide | 34.0 | 23.1 | 415.0 | 1.15e-115 |
| ALP32043.1 | CycC | BGC0001293 | Polyketide | 32.0 | 26.7 | 416.0 | 1.2e-115 |
| AEW98134.1 | polyketide\_synthase | BGC0002642 | Alkaloid | 33.0 | 22.3 | 414.0 | 1.23e-115 |
| AXI91545.1 | FunP8 | BGC0001944 | Polyketide | 33.0 | 26.9 | 412.0 | 1.34e-115 |
| ABP73645.1 | SalA | BGC0000145 | NRP+Polyketide | 34.0 | 23.4 | 412.0 | 1.46e-115 |
| CAI94713.1 | putative\_polyketide\_synthase | BGC0000141 | Polyketide | 32.0 | 24.2 | 416.0 | 1.56e-115 |
| ADH04658.1 | TugB | BGC0001342 | NRP+Polyketide | 34.0 | 22.2 | 415.0 | 1.59e-115 |
| QSE03604.1 | LcmD | BGC0002333 | Polyketide | 34.0 | 22.7 | 410.0 | 1.6e-115 |
| AFV30247.1 | polyketide\_synthase | BGC0000075 | Polyketide | 33.0 | 24.4 | 416.0 | 1.64e-115 |
| ABB86410.1 | GelC | BGC0000067 | Polyketide | 31.0 | 27.0 | 415.0 | 1.65e-115 |
| ACR50774.1 | polyketide\_synthase | BGC0000163 | Polyketide | 31.0 | 27.0 | 416.0 | 1.66e-115 |
| AEK75502.1 | type\_1\_polyketide\_synthase | BGC0000001 | Polyketide:Modular type I polyketide | 35.0 | 22.2 | 416.0 | 1.68e-115 |
| AAP42859.1 | NanA5 | BGC0000105 | Polyketide | 31.0 | 28.3 | 415.0 | 1.69e-115 |
| AAB66508.1 | tylactone\_synthase\_module\_7 | BGC0000166 | Polyketide | 34.0 | 22.7 | 410.0 | 1.78e-115 |
| AXI91548.1 | FunP5 | BGC0001944 | Polyketide | 36.0 | 22.4 | 416.0 | 1.82e-115 |
| BAO66539.1 | type\_I\_polyketide\_synthase | BGC0000042 | Polyketide | 34.0 | 22.8 | 414.0 | 1.93e-115 |
| ABI93779.1 | GdmPKS | BGC0000068 | Polyketide | 32.0 | 24.6 | 414.0 | 2.03e-115 |
| BAH02270.1 | polyketide\_synthase | BGC0000126 | Polyketide | 33.0 | 22.4 | 415.0 | 2.05e-115 |
| QOD94999.1 | PldAIV | BGC0002102 | Polyketide | 33.0 | 22.4 | 415.0 | 2.05e-115 |
| AEZ53952.1 | polyketide\_synthase | BGC0000144 | Polyketide:Modular type I polyketide | 33.0 | 22.7 | 415.0 | 2.06e-115 |
| BAW35612.1 | modular\_polyketide\_synthase | BGC0002357 | Polyketide+Other | 33.0 | 22.8 | 415.0 | 2.07e-115 |
| AEZ53949.1 | polyketide\_synthase | BGC0000144 | Polyketide:Modular type I polyketide | 32.0 | 25.0 | 415.0 | 2.07e-115 |
| QRI43529.1 | type\_I\_polyketide\_synthase | BGC0002454 | Polyketide | 35.0 | 23.1 | 415.0 | 2.15e-115 |
| QGJ79644.1 | Polyketide\_synthase | BGC0002552 | Polyketide | 36.0 | 22.2 | 414.0 | 2.17e-115 |
| TMU97100.1 | SDR\_family\_NAD(P)-dependent\_oxidoreductase | BGC0002038 | Polyketide | 34.0 | 22.6 | 415.0 | 2.28e-115 |
| AJO72743.1 | Type\_I\_modular\_polyketide\_synthase | BGC0001381 | Polyketide | 35.0 | 22.2 | 414.0 | 2.44e-115 |
| WP\_053138504.1 | type\_I\_polyketide\_synthase | BGC0002033 | Polyketide | 33.0 | 22.7 | 415.0 | 2.48e-115 |
| AUA09466.1 | Erythronolide\_synthase,\_modules\_1\_and\_2 | BGC0002291 | Polyketide | 32.0 | 25.5 | 408.0 | 2.54e-115 |
| AWW87424.1 | type\_I\_polyketide\_synthase | BGC0001755 | Polyketide | 31.0 | 26.8 | 415.0 | 2.65e-115 |
| QQZ01585.1 | PKS | BGC0002498 | Other | 33.0 | 23.8 | 415.0 | 2.72e-115 |
| ACB46486.1 | polyketide\_synthase | BGC0000082 | Polyketide | 32.0 | 23.4 | 409.0 | 2.89e-115 |
| WP\_053138519.1 | type\_I\_polyketide\_synthase | BGC0002033 | Polyketide | 33.0 | 23.5 | 407.0 | 2.91e-115 |
| ADH04659.1 | TugC | BGC0001342 | NRP+Polyketide | 34.0 | 23.4 | 414.0 | 3.23e-115 |
| AHA38203.1 | GphJ | BGC0000069 | Polyketide | 33.0 | 24.9 | 409.0 | 3.28e-115 |
| TXD00033.1 | SDR\_family\_NAD(P)-dependent\_oxidoreductase | BGC0001877 | Polyketide | 34.0 | 22.1 | 414.0 | 3.44e-115 |
| AKA59089.1 | type-I\_PKS | BGC0001619 | Polyketide | 35.0 | 22.7 | 414.0 | 3.48e-115 |
| ABW96542.1 | type\_I\_modular\_polyketide\_synthase | BGC0000159 | Polyketide:Modular type I polyketide | 32.0 | 27.5 | 415.0 | 3.58e-115 |
| ANY10599.1 | polyketide\_synthase | BGC0001773 | Polyketide | 32.0 | 26.9 | 412.0 | 3.63e-115 |
| ctg1\_orf256 |  | BGC0001200 | Polyketide | 34.0 | 22.2 | 414.0 | 3.66e-115 |
| QIZ24104.1 | type\_I\_polyketide\_synthase | BGC0002540 | Polyketide | 33.0 | 24.0 | 408.0 | 4.24e-115 |
| ACO94470.1 | polyketide\_synthase\_type\_I | BGC0000029 | Polyketide:Modular type I polyketide | 34.0 | 22.3 | 410.0 | 4.26e-115 |
| TMU97098.1 | acyltransferase\_domain-containing\_protein | BGC0002038 | Polyketide | 33.0 | 22.6 | 407.0 | 4.28e-115 |
| ACR50785.1 | polyketide\_synthase | BGC0000163 | Polyketide | 33.0 | 25.1 | 414.0 | 4.55e-115 |
| AXM42951.1 | polyketide\_synthase | BGC0001941 | NRP+Polyketide | 35.0 | 22.4 | 402.0 | 5.07e-115 |
| AAO65807.1 | monensin\_polyketide\_synthase\_module\_10 | BGC0000100 | Polyketide | 35.0 | 22.5 | 407.0 | 5.54e-115 |
| ANZ52470.1 | MonAVII | BGC0001670 | Polyketide | 35.0 | 22.5 | 407.0 | 5.54e-115 |
| QWF78549.1 | 3-ketoacyl-CoA\_thiolase | BGC0002142 | Polyketide | 34.0 | 22.5 | 414.0 | 5.66e-115 |
| ABP53498.1 | PKS\_(ACP-AT-AT-KS-ACP-C) | BGC0001041 | NRP+Polyketide | 33.0 | 23.4 | 410.0 | 5.83e-115 |
| SAI82910.1 | HrnG;\_Macrolactam\_polyketide\_synthase\_type\_I;\_module\_8 | BGC0002101 | Polyketide | 34.0 | 22.8 | 410.0 | 6.06e-115 |
| AMB20394.1 | polyketide\_synthase | BGC0002072 | Polyketide:Modular type I polyketide | 31.0 | 27.7 | 414.0 | 6.09e-115 |
| AAF71776.1 | nysC | BGC0000115 | Polyketide:Modular type I polyketide+Saccharide:Hybrid/tailoring saccharide | 31.0 | 26.6 | 414.0 | 6.15e-115 |
| sipP1 | Type\_I\_Modular\_PKS | BGC0001452 | Polyketide | 33.0 | 25.0 | 413.0 | 6.2e-115 |
| BCK51649.1 | modular\_polyketide\_synthase | BGC0002520 | Polyketide | 35.0 | 22.4 | 409.0 | 6.21e-115 |
| WP\_015031692.1 | type\_I\_polyketide\_synthase | BGC0001819 | Polyketide | 33.0 | 23.7 | 411.0 | 6.32e-115 |
| ARS01474.1 | NcmAII | BGC0001702 | NRP+Polyketide | 35.0 | 22.3 | 408.0 | 6.43e-115 |
| QEA08887.1 | JenA1 | BGC0002559 | Polyketide | 34.0 | 22.4 | 412.0 | 6.96e-115 |
| ALP32045.1 | CycE | BGC0001293 | Polyketide | 33.0 | 24.3 | 413.0 | 7.58e-115 |
| AJW65408.1 | type\_I\_modular\_polyketide\_synthase | BGC0001195 | NRP+Polyketide | 34.0 | 22.6 | 413.0 | 7.92e-115 |
| QBF51757.1 | type\_I\_polyketide\_synthase | BGC0001856 | Polyketide:Modular type I polyketide | 33.0 | 22.7 | 413.0 | 8.03e-115 |
| AQV04224.1 | SwnK | BGC0001793 | NRP+Polyketide | 33.0 | 22.8 | 412.0 | 8.11e-115 |
| TGZ15164.1 | hypothetical\_protein | BGC0002032 | Polyketide | 35.0 | 22.6 | 412.0 | 9.07e-115 |
| ARM20278.1 | polyketide\_synthase | BGC0001523 | Polyketide | 33.0 | 22.9 | 412.0 | 9.13e-115 |
| QFU80901.1 | PKS | BGC0002550 | Polyketide | 34.0 | 22.5 | 412.0 | 9.14e-115 |
| BAP34733.1 | type\_I\_polyketide\_synthase | BGC0000078 | Polyketide | 33.0 | 23.9 | 412.0 | 1.01e-114 |
| ANR02552.1 | LodK | BGC0001648 | Polyketide | 33.0 | 22.2 | 406.0 | 1.07e-114 |
| TGZ15166.1 | hypothetical\_protein | BGC0002032 | Polyketide | 34.0 | 22.7 | 408.0 | 1.12e-114 |
| BAC68125.1 | modular\_polyketide\_synthase | BGC0000059 | Polyketide | 35.0 | 23.0 | 412.0 | 1.19e-114 |
| BAK64650.1 | polyketide\_synthase | BGC0000135 | Polyketide | 35.0 | 23.0 | 413.0 | 1.2e-114 |
| QRI43528.1 | type\_I\_polyketide\_synthase | BGC0002454 | Polyketide | 33.0 | 23.8 | 410.0 | 1.23e-114 |
| AAS79463.1 | polyketide\_synthase\_subunit | BGC0000035 | Polyketide | 32.0 | 23.1 | 400.0 | 1.49e-114 |
| AHE80994.1 | PieA4 | BGC0001169 | Polyketide:Modular type I polyketide | 32.0 | 26.6 | 410.0 | 1.59e-114 |
| WP\_030180235.1 | type\_I\_polyketide\_synthase | BGC0002106 | Polyketide | 32.0 | 23.6 | 412.0 | 1.73e-114 |
| ADX66472.1 | ScnS1 | BGC0000108 | Polyketide | 34.0 | 23.6 | 412.0 | 2.04e-114 |
| ABJ97438.1 | MerB | BGC0001012 | NRP+Polyketide | 33.0 | 23.0 | 412.0 | 2.08e-114 |
| ANR02554.1 | LodM | BGC0001648 | Polyketide | 34.0 | 22.6 | 411.0 | 2.1e-114 |
| ADC79619.1 | BafAIV | BGC0000028 | Polyketide:Modular type I polyketide | 33.0 | 23.9 | 411.0 | 2.16e-114 |
| AAX98184.1 | polyketide\_synthase\_type\_I | BGC0000052 | Polyketide | 34.0 | 22.9 | 412.0 | 2.17e-114 |
| BAO66529.1 | type\_I\_polyketide\_synthase | BGC0000042 | Polyketide | 34.0 | 22.5 | 412.0 | 2.34e-114 |
| AKL64834.1 | polyketide\_synthase | BGC0002072 | Polyketide:Modular type I polyketide | 34.0 | 22.7 | 412.0 | 2.37e-114 |
| EPH46605.1 | putative\_Oleandomycin\_polyketide\_synthase,\_modules\_5\_and\_6 | BGC0001519 | NRP+Polyketide | 33.0 | 22.8 | 392.0 | 2.52e-114 |
| ACR50775.1 | polyketide\_synthase | BGC0000163 | Polyketide | 34.0 | 22.4 | 412.0 | 2.52e-114 |
| QKV49769.1 | PKS | BGC0002526 | Polyketide | 25.0 | 61.4 | 411.0 | 2.64e-114 |
| AQT01382.1 | SgnS1 | BGC0001690 | Polyketide | 34.0 | 23.4 | 412.0 | 2.66e-114 |
| QBL56182.1 | PKS | BGC0002376 | Polyketide | 33.0 | 22.9 | 411.0 | 2.9e-114 |
| AAQ84147.1 | Plm7 | BGC0000123 | Polyketide | 35.0 | 23.0 | 406.0 | 2.97e-114 |
| QBL56191.1 | PKS | BGC0002376 | Polyketide | 34.0 | 22.5 | 405.0 | 3.14e-114 |
| BAP34739.1 | type\_I\_polyketide\_synthase | BGC0000078 | Polyketide | 32.0 | 26.7 | 411.0 | 3.16e-114 |
| AAO65797.1 | monensin\_polyketide\_synthase\_module\_2 | BGC0000100 | Polyketide | 34.0 | 22.5 | 409.0 | 3.28e-114 |
| ANZ52460.1 | MonAII | BGC0001670 | Polyketide | 34.0 | 22.5 | 409.0 | 3.28e-114 |
| BBM96640.1 | modular\_polyketide\_synthase | BGC0002452 | Polyketide | 33.0 | 22.5 | 407.0 | 3.39e-114 |
| AJO72737.1 | Type\_I\_modular\_polyketide\_synthase | BGC0001381 | Polyketide | 33.0 | 23.0 | 411.0 | 3.46e-114 |
| QQZ01589.1 | PKS | BGC0002498 | Other | 33.0 | 23.4 | 407.0 | 3.52e-114 |
| AXN93577.1 | PuwB | BGC0001950 | NRP | 30.0 | 29.7 | 410.0 | 3.55e-114 |
| AXN93586.1 | PuwB | BGC0001951 | NRP | 30.0 | 29.7 | 410.0 | 3.55e-114 |
| ALJ49921.1 | TtmH | BGC0001236 | Polyketide | 32.0 | 23.3 | 409.0 | 3.67e-114 |
| ABY21541.1 | AngAIV | BGC0000018 | Polyketide | 33.0 | 23.4 | 403.0 | 3.69e-114 |
| ABB88520.1 | polyketide\_synthase\_type\_I | BGC0000050 | Polyketide | 32.0 | 27.0 | 410.0 | 3.76e-114 |
| AVV61981.1 | type\_I\_modular\_PKS | BGC0001477 | NRP+Polyketide:Modular type I polyketide | 31.0 | 27.7 | 410.0 | 3.78e-114 |
| AAQ84146.1 | Plm6 | BGC0000123 | Polyketide | 33.0 | 23.1 | 403.0 | 3.94e-114 |
| AKU20507.1 | polyketide\_synthase | BGC0002687 | Polyketide+NRP | 35.0 | 22.7 | 410.0 | 4.57e-114 |
| BAW35609.1 | modular\_polyketide\_synthase | BGC0002357 | Polyketide+Other | 33.0 | 22.9 | 410.0 | 5.2e-114 |
| BAJ16471.1 | polyketide\_synthase | BGC0000058 | Polyketide | 33.0 | 23.7 | 410.0 | 5.31e-114 |
| ACF35447.1 | mbcAIII | BGC0000090 | Polyketide | 34.0 | 23.8 | 410.0 | 5.42e-114 |
| CAC20920.1 | PimS3\_protein | BGC0000125 | Polyketide | 34.0 | 22.5 | 405.0 | 5.45e-114 |
| AQT01394.1 | SgnS3 | BGC0001690 | Polyketide | 34.0 | 22.5 | 405.0 | 5.45e-114 |
| WP\_240490790.1 | type\_I\_polyketide\_synthase | BGC0002009 | Polyketide | 31.0 | 27.3 | 405.0 | 6.05e-114 |
| CAF05649.1 | TubD\_protein | BGC0001053 | NRP+Polyketide | 23.0 | 93.8 | 410.0 | 6.36e-114 |
| AGY62758.1 | EbeF | BGC0000051 | Polyketide | 31.0 | 26.5 | 409.0 | 6.81e-114 |
| ALA09358.1 | type\_I\_modular\_PKS | BGC0001303 | Polyketide | 31.0 | 26.7 | 401.0 | 7.03e-114 |
| ABG02264.1 | SalB | BGC0000143 | Polyketide | 34.0 | 23.1 | 409.0 | 7.24e-114 |
| EHK80167.1 | modular\_polyketide\_synthase | BGC0001447 | Polyketide | 33.0 | 23.9 | 410.0 | 7.29e-114 |
| CCP20050.1 | divL3\_protein | BGC0001119 | Polyketide:Modular type I polyketide | 30.0 | 27.7 | 407.0 | 8.85e-114 |
| BAW35637.1 | modular\_polyketide\_synthase | BGC0002356 | Polyketide+Other | 34.0 | 22.7 | 410.0 | 8.89e-114 |
| AGI99495.1 | Type\_I\_polyketide\_synthase | BGC0001004 | Polyketide:Modular type I polyketide | 33.0 | 22.6 | 405.0 | 8.9e-114 |
| QFU80899.1 | PKS | BGC0002550 | Polyketide | 33.0 | 22.6 | 405.0 | 8.9e-114 |
| AAQ82566.1 | FscF | BGC0000034 | NRP+Polyketide | 31.0 | 26.9 | 407.0 | 9.11e-114 |
| QEA08888.1 | JenA2 | BGC0002559 | Polyketide | 34.0 | 22.3 | 407.0 | 9.78e-114 |
| AAO65798.1 | monensin\_polyketide\_synthase\_modules\_3\_and\_4 | BGC0000100 | Polyketide | 34.0 | 22.4 | 409.0 | 9.87e-114 |
| ANZ52461.1 | MonAIII | BGC0001670 | Polyketide | 34.0 | 22.4 | 409.0 | 9.87e-114 |
| AWW87425.1 | polyketide\_synthase | BGC0001755 | Polyketide | 33.0 | 23.5 | 410.0 | 1.04e-113 |
| QSV12662.1 | AvmD | BGC0002456 | Polyketide+NRP | 33.0 | 23.8 | 410.0 | 1.06e-113 |
| OAP25821.1 | Phenolphthiocerol\_synthesis\_polyketide\_synthase\_type\_I\_Pks15/1 | BGC0001658 | Polyketide | 34.0 | 22.3 | 409.0 | 1.07e-113 |
| AAQ82567.1 | FscE | BGC0000034 | NRP+Polyketide | 32.0 | 27.3 | 410.0 | 1.07e-113 |
| ACN69991.1 | polyketide\_synthase | BGC0000079 | Polyketide | 33.0 | 22.8 | 407.0 | 1.08e-113 |
| AFL48520.1 | laidlomycin\_polyketide\_synthase\_(module\_9) | BGC0000084 | Polyketide | 34.0 | 23.0 | 402.0 | 1.09e-113 |
| CAC22145.1 | CpkB;\_Polyketide\_synthase\_modules\_3\_and\_4 | BGC0000038 | Polyketide:Modular type I polyketide | 32.0 | 23.6 | 409.0 | 1.11e-113 |
| ADX66461.1 | ScnS2 | BGC0000108 | Polyketide | 34.0 | 22.3 | 410.0 | 1.13e-113 |
| QIQ28638.1 | Nbc42 | BGC0002541 | Other | 33.0 | 22.5 | 408.0 | 1.2e-113 |
| AKA59093.1 | type-I\_PKS | BGC0001619 | Polyketide | 35.0 | 22.5 | 409.0 | 1.24e-113 |
| BAB69199.1 | modular\_polyketide\_synthase | BGC0000117 | Polyketide | 34.0 | 22.7 | 409.0 | 1.25e-113 |
| ABP55221.1 | acyl\_transferase\_domain\_protein | BGC0000142 | Polyketide | 33.0 | 24.7 | 402.0 | 1.27e-113 |
| QES95475.1 | type\_I\_polyketide\_synthase | BGC0002453 | Polyketide | 33.0 | 23.1 | 405.0 | 1.27e-113 |
| ONK09689.1 | Beta-ketoacyl-acyl-carrier-protein\_synthase\_I | BGC0001647 | Polyketide | 34.0 | 22.7 | 408.0 | 1.35e-113 |
| QUQ72345.1 | 3-ketoacyl-CoA\_thiolase | BGC0002349 | Polyketide+Saccharide | 33.0 | 24.7 | 409.0 | 1.38e-113 |
| QIE07128.1 | OvmL2 | BGC0001719 | Polyketide | 34.0 | 22.8 | 404.0 | 1.4e-113 |
| QGA70101.1 | type\_I\_polyketide\_synthase | BGC0002517 | Polyketide | 34.0 | 23.0 | 404.0 | 1.52e-113 |
| CCP20049.1 | divL2\_protein | BGC0001119 | Polyketide:Modular type I polyketide | 34.0 | 22.8 | 404.0 | 1.53e-113 |
| AAO06918.1 | GdmAIII | BGC0000066 | Polyketide | 30.0 | 27.0 | 409.0 | 1.6e-113 |
| QBG82528.1 | Polyketide\_synthase | BGC0002587 | Polyketide | 35.0 | 22.6 | 409.0 | 1.75e-113 |
| EYT83439.1 | beta-ketoacyl\_synthase | BGC0001213 | Polyketide | 32.0 | 25.0 | 402.0 | 1.75e-113 |
| CAJ88176.1 | Type\_I\_modular\_polyketide\_synthase | BGC0000151 | Polyketide:Modular type I polyketide+Saccharide:Hybrid/tailoring saccharide | 32.0 | 25.0 | 408.0 | 1.96e-113 |
| CAC20919.1 | PimS4\_protein | BGC0000125 | Polyketide | 32.0 | 26.8 | 405.0 | 2.48e-113 |
| AQT01395.1 | SgnS4 | BGC0001690 | Polyketide | 32.0 | 26.8 | 405.0 | 2.48e-113 |
| WP\_081238284.1 | type\_I\_polyketide\_synthase | BGC0002105 | Polyketide | 34.0 | 24.2 | 407.0 | 2.58e-113 |
| AKG06377.1 | polyketide\_synthase\_type\_1 | BGC0001830 | Polyketide | 33.0 | 24.2 | 408.0 | 2.61e-113 |
| BAB69193.1 |  | BGC0000117 | Polyketide | 33.0 | 22.6 | 408.0 | 3.27e-113 |
| ctg1\_orf253 |  | BGC0001200 | Polyketide | 34.0 | 22.2 | 407.0 | 3.56e-113 |
| WP\_234353270.1 | SDR\_family\_NAD(P)-dependent\_oxidoreductase | BGC0001537 | Polyketide | 34.0 | 22.2 | 408.0 | 3.71e-113 |
| ctg1\_orf23 |  | BGC0001013 | NRP+Polyketide | 31.0 | 27.0 | 407.0 | 3.73e-113 |
| AZF85947.1 | type\_I\_polyketide\_synthase | BGC0001963 | NRP+Polyketide | 32.0 | 25.4 | 408.0 | 3.81e-113 |
| CAC20931.1 | PimS1\_protein | BGC0000125 | Polyketide | 34.0 | 22.4 | 408.0 | 3.9e-113 |
| BAV56011.1 | PKS\_(KS-AT-DH-ER-KR-ACP-KS-AT-DH-ER-KR-ACP) | BGC0001597 | Polyketide | 32.0 | 24.1 | 407.0 | 3.91e-113 |
| ABC87512.1 | polyketide\_synthase | BGC0001011 | NRP+Polyketide | 31.0 | 27.0 | 407.0 | 4.06e-113 |
| AAP42855.1 | NanA1 | BGC0000105 | Polyketide | 34.0 | 22.6 | 407.0 | 4.07e-113 |
| ADX66459.1 | ScnS4 | BGC0000108 | Polyketide | 32.0 | 26.9 | 404.0 | 4.25e-113 |
| AFL48533.1 | laidlomycin\_polyketide\_synthase\_(module\_10) | BGC0000084 | Polyketide | 33.0 | 22.6 | 400.0 | 4.7e-113 |
| ABC84469.1 | NigAIX | BGC0000114 | Polyketide:Modular type I polyketide | 33.0 | 22.8 | 404.0 | 5.07e-113 |
| EHA27898.1 | hypothetical\_protein | BGC0002171 | NRP+Polyketide | 32.0 | 23.1 | 403.0 | 5.32e-113 |
| ALA09356.1 | type\_I\_modular\_PKS | BGC0001303 | Polyketide | 34.0 | 24.3 | 400.0 | 5.43e-113 |
| AEH42474.1 | polyketide\_synthase | BGC0000032 | Polyketide | 32.0 | 24.3 | 404.0 | 6.66e-113 |
| BBM96637.1 | modular\_polyketide\_synthase | BGC0002452 | Polyketide | 32.0 | 25.3 | 402.0 | 7e-113 |
| AHH99920.1 | PKS\_I | BGC0000002 | Polyketide | 32.0 | 22.4 | 406.0 | 7.29e-113 |
| ctg1\_orf522 |  | BGC0001199 | Polyketide | 33.0 | 23.3 | 406.0 | 7.39e-113 |
| AAO65806.1 | monensin\_polyketide\_synthase\_modules\_11\_and\_12 | BGC0000100 | Polyketide | 34.0 | 22.7 | 406.0 | 7.74e-113 |
| ANZ52469.1 | MonAVIII | BGC0001670 | Polyketide | 34.0 | 22.7 | 406.0 | 7.74e-113 |
| ATY12793.1 | type\_I\_polyketide\_synthase | BGC0001504 | Polyketide | 34.0 | 22.3 | 404.0 | 8.16e-113 |
| EHA48594.1 | hypothetical\_protein | BGC0002155 | Polyketide | 29.0 | 31.5 | 405.0 | 8.2e-113 |
| AAQ84144.1 | Plm4 | BGC0000123 | Polyketide | 33.0 | 21.6 | 399.0 | 8.28e-113 |
| BCB17027.1 | modular\_polyketide\_synthase | BGC0002523 | NRP | 33.0 | 22.8 | 406.0 | 8.65e-113 |
| QNN81298.1 | IonAII | BGC0002446 | Polyketide | 31.0 | 25.0 | 400.0 | 9.06e-113 |
| CBA11582.1 | polyketide\_synthase\_type\_I | BGC0001046 | NRP+Polyketide:Modular type I polyketide+Saccharide:Hybrid/tailoring saccharide | 34.0 | 22.7 | 406.0 | 9.17e-113 |
| PLB34720.1 | polyketide\_synthase | BGC0002749 | NRP+Polyketide | 33.0 | 22.5 | 405.0 | 1.07e-112 |
| ACO94471.1 | polyketide\_synthase\_type\_I | BGC0000029 | Polyketide:Modular type I polyketide | 30.0 | 26.7 | 405.0 | 1.17e-112 |
| QOD94998.1 | PldAIII | BGC0002102 | Polyketide | 31.0 | 22.4 | 406.0 | 1.21e-112 |
| ABV83222.1 | CppJ | BGC0000116 | Polyketide | 33.0 | 24.5 | 406.0 | 1.34e-112 |
| AAF71767.1 | nysJ | BGC0000115 | Polyketide:Modular type I polyketide+Saccharide:Hybrid/tailoring saccharide | 34.0 | 22.3 | 406.0 | 1.34e-112 |
| ctg1\_orf524 |  | BGC0001199 | Polyketide | 34.0 | 22.5 | 394.0 | 1.42e-112 |
| BCB17031.1 | modular\_polyketide\_synthase | BGC0002523 | NRP | 31.0 | 26.8 | 405.0 | 1.57e-112 |
| ATL73034.1 | type\_I\_modular\_polyketide\_synthase | BGC0001807 | NRP+Polyketide | 34.0 | 23.0 | 405.0 | 1.73e-112 |
| CAJ88177.1 | Type\_I\_modular\_polyketide\_synthase | BGC0000151 | Polyketide:Modular type I polyketide+Saccharide:Hybrid/tailoring saccharide | 34.0 | 23.4 | 405.0 | 1.81e-112 |
| QIZ24100.1 | type\_I\_polyketide\_synthase | BGC0002540 | Polyketide | 32.0 | 26.8 | 404.0 | 1.89e-112 |
| ctg1\_orf31 |  | BGC0000096 | Polyketide | 33.0 | 22.7 | 401.0 | 2.05e-112 |
| BAY02135.1 | putative\_beta-ketoacyl\_synthase | BGC0002532 | NRP+Polyketide | 34.0 | 22.4 | 397.0 | 2.09e-112 |
| ACY06289.1 | type\_I\_polyketide\_synthase | BGC0001042 | NRP+Polyketide | 34.0 | 22.5 | 405.0 | 2.09e-112 |
| QFU19825.1 | PKS | BGC0002431 | Polyketide+Saccharide | 33.0 | 22.6 | 405.0 | 2.12e-112 |
| ANH11413.1 | SceR | BGC0001770 | Polyketide | 34.0 | 22.9 | 398.0 | 2.18e-112 |
| BAR73021.1 | putative\_PKS\_(KS-AT-KR-ACP) | BGC0001194 | Polyketide | 33.0 | 23.5 | 398.0 | 2.23e-112 |
| AAM54076.1 | polyketide\_synthase | BGC0000020 | Polyketide | 34.0 | 22.3 | 404.0 | 2.28e-112 |
| QIQ28639.1 | Nbc43 | BGC0002541 | Other | 32.0 | 28.1 | 405.0 | 2.3e-112 |
| AFL48528.1 | laidlomycin\_polyketide\_synthase\_(module\_7\_and\_module\_8) | BGC0000084 | Polyketide | 32.0 | 24.7 | 405.0 | 2.36e-112 |
| ABW96541.1 | type\_I\_modular\_polyketide\_synthase | BGC0000159 | Polyketide:Modular type I polyketide | 31.0 | 24.6 | 405.0 | 2.36e-112 |
| ABW96540.1 | type\_I\_modular\_polyketide\_synthase | BGC0000159 | Polyketide:Modular type I polyketide | 33.0 | 22.7 | 405.0 | 2.37e-112 |
| AAO65801.1 | monensin\_polyketide\_synthase\_module\_9 | BGC0000100 | Polyketide | 33.0 | 23.2 | 399.0 | 2.84e-112 |
| ANZ52464.1 | MonAVI | BGC0001670 | Polyketide | 33.0 | 23.2 | 399.0 | 2.84e-112 |
| ADC79617.1 | BafAII | BGC0000028 | Polyketide:Modular type I polyketide | 33.0 | 23.3 | 405.0 | 2.92e-112 |
| AAQ90174.1 | polyketide\_synthase\_type\_I | BGC0000128 | Polyketide | 33.0 | 22.7 | 399.0 | 2.95e-112 |
| ANH11415.1 | SceT | BGC0001770 | Polyketide | 34.0 | 23.7 | 392.0 | 2.97e-112 |
| ACB37742.1 | putative\_type\_I\_polyketide\_synthase | BGC0000162 | Polyketide | 34.0 | 22.4 | 400.0 | 2.99e-112 |
| AGC09499.1 | LobS4 | BGC0001183 | Polyketide | 33.0 | 23.7 | 404.0 | 3.23e-112 |
| AAC68815.1 | FK506\_polyketide\_synthase | BGC0000353 | NRP | 33.0 | 23.0 | 405.0 | 3.47e-112 |
| AEZ53948.1 | polyketide\_synthase | BGC0000144 | Polyketide:Modular type I polyketide | 32.0 | 24.0 | 398.0 | 3.97e-112 |
| AAY28227.1 | HbmAIII | BGC0000074 | Polyketide | 30.0 | 27.1 | 404.0 | 4.01e-112 |
| AFV30248.1 | polyketide\_synthase | BGC0000075 | Polyketide | 32.0 | 23.7 | 402.0 | 4.29e-112 |
| OAP25819.1 | Phenolphthiocerol\_synthesis\_polyketide\_synthase\_type\_I\_Pks15/1 | BGC0001658 | Polyketide | 34.0 | 22.4 | 399.0 | 4.33e-112 |
| ANZ22988.1 | ZinE | BGC0001828 | Polyketide | 34.0 | 22.8 | 398.0 | 4.6e-112 |
| CAA60459.1 | polyketide\_synthase | BGC0001040 | NRP+Polyketide | 32.0 | 26.8 | 404.0 | 4.94e-112 |
| AAO65796.1 | monensin\_polyketide\_synthase\_loading\_module\_and\_module\_1 | BGC0000100 | Polyketide | 33.0 | 22.3 | 403.0 | 4.99e-112 |
| ANZ52459.1 | MonAI | BGC0001670 | Polyketide | 33.0 | 22.3 | 403.0 | 4.99e-112 |
| BAK64637.1 | polyketide\_synthase | BGC0000135 | Polyketide | 32.0 | 23.9 | 404.0 | 5.03e-112 |
| AAP42858.1 | NanA4 | BGC0000105 | Polyketide | 31.0 | 27.3 | 404.0 | 5.36e-112 |
| AGY62753.1 | EbeA | BGC0000051 | Polyketide | 35.0 | 21.3 | 385.0 | 5.55e-112 |
| ACZ65476.1 | type\_I\_modular\_polyketide\_synthase | BGC0000140 | Polyketide | 33.0 | 22.5 | 385.0 | 5.55e-112 |
| AVX51106.1 | nysA | BGC0001709 | Polyketide | 31.0 | 26.7 | 393.0 | 5.61e-112 |
| BAC57029.1 | protomycinolide\_IV\_synthase\_2 | BGC0000102 | Polyketide | 33.0 | 23.1 | 399.0 | 6.18e-112 |
| BAG85030.1 | putative\_polyketide\_synthase | BGC0000086 | Polyketide | 35.0 | 22.3 | 403.0 | 6.52e-112 |
| CAQ64690.1 | lasalocid\_modular\_polyketide\_synthase | BGC0000087 | Polyketide | 35.0 | 22.3 | 403.0 | 6.52e-112 |
| AFL48527.1 | laidlomycin\_polyketide\_synthase\_(module\_3\_and\_module\_4) | BGC0000084 | Polyketide | 33.0 | 22.7 | 403.0 | 7.03e-112 |
| AGM05534.1 | modular\_polyketide\_synthase | BGC0002098 | Polyketide | 31.0 | 27.3 | 403.0 | 7.36e-112 |
| ALA09371.1 | type\_I\_modular\_PKS | BGC0001303 | Polyketide | 34.0 | 22.7 | 404.0 | 7.59e-112 |
| CAA60460.1 | polyketide\_synthase | BGC0001040 | NRP+Polyketide | 32.0 | 27.2 | 404.0 | 8.08e-112 |
| PYH50506.1 | hypothetical\_protein | BGC0002275 | NRP+Polyketide | 32.0 | 23.1 | 402.0 | 8.32e-112 |
| ACY06288.1 | type\_I\_polyketide\_synthase | BGC0001042 | NRP+Polyketide | 34.0 | 22.7 | 403.0 | 8.4e-112 |
| BAW32323.1 | hybrid\_cis-AT\_polyketide\_synthase\_-\_nonribosomal\_peptide\_synthetase | BGC0001630 | NRP+Polyketide | 33.0 | 22.5 | 403.0 | 8.57e-112 |
| AIG62146.1 | 6-methylsalicylic\_acid\_synthase | BGC0000120 | Polyketide:Iterative type I polyketide | 32.0 | 25.5 | 398.0 | 9.11e-112 |
| AAP85335.1 | type\_I\_PKS | BGC0000233 | Polyketide | 31.0 | 26.9 | 400.0 | 9.87e-112 |
| ABV99085.1 | thioester\_reductase\_domain | BGC0001007 | Polyketide+NRP | 33.0 | 22.2 | 401.0 | 1.02e-111 |
| ctg1\_orf521 |  | BGC0001199 | Polyketide | 33.0 | 23.4 | 403.0 | 1.09e-111 |
| EFY95969.1 | polyketide\_synthase | BGC0002270 | NRP+Polyketide | 32.0 | 23.8 | 401.0 | 1.21e-111 |
| AAO65800.1 | monensin\_polyketide\_synthase\_modules\_7\_and\_8 | BGC0000100 | Polyketide | 31.0 | 27.0 | 402.0 | 1.24e-111 |
| ANZ52463.1 | MonAV | BGC0001670 | Polyketide | 31.0 | 27.0 | 402.0 | 1.24e-111 |
| QKV49791.1 | PKS | BGC0002526 | Polyketide | 33.0 | 22.3 | 402.0 | 1.4e-111 |
| AAF86392.1 | FkbC | BGC0000994 | NRP+Polyketide | 35.0 | 22.3 | 402.0 | 1.42e-111 |
| EHK80166.1 | beta-ketoacyl\_synthase | BGC0001447 | Polyketide | 31.0 | 24.8 | 402.0 | 1.47e-111 |
| QBF51759.1 | type\_I\_polyketide\_synthase | BGC0001856 | Polyketide:Modular type I polyketide | 32.0 | 23.7 | 402.0 | 1.48e-111 |
| ACO94500.1 | polyketide\_synthase\_type\_I | BGC0000097 | Polyketide:Modular type I polyketide | 35.0 | 22.8 | 396.0 | 1.57e-111 |
| AAP42867.1 | NanA7 | BGC0000105 | Polyketide | 33.0 | 23.8 | 396.0 | 1.71e-111 |
| ANH11410.1 | SceO | BGC0001770 | Polyketide | 32.0 | 27.0 | 402.0 | 1.81e-111 |
| QTT72113.1 | type\_I\_polyketide\_synthase | BGC0002350 | NRP+Polyketide+Saccharide | 31.0 | 27.2 | 401.0 | 1.92e-111 |
| AVX51108.1 | nysC | BGC0001709 | Polyketide | 33.0 | 22.6 | 402.0 | 1.92e-111 |
| BAW35656.1 | modular\_polyketide\_synthase | BGC0002355 | Polyketide+Other | 32.0 | 22.5 | 402.0 | 1.99e-111 |
| BBM95964.1 | modular\_polyketide\_synthase | BGC0002558 | Polyketide | 34.0 | 23.7 | 395.0 | 2.06e-111 |
| CAO98849.1 | polyketide\_synthase\_AufF | BGC0000023 | Polyketide:Modular type I polyketide | 33.0 | 22.5 | 401.0 | 2.25e-111 |
| AUA09464.1 | Erythronolide\_synthase,\_modules\_3\_and\_4 | BGC0002291 | Polyketide | 33.0 | 23.5 | 401.0 | 2.28e-111 |
| ABP55493.1 | thioester\_reductase\_domain | BGC0001006 | NRP+Polyketide | 33.0 | 22.3 | 400.0 | 2.32e-111 |
| QEA08891.1 | JenA5 | BGC0002559 | Polyketide | 33.0 | 22.7 | 401.0 | 2.69e-111 |
| ABC87511.1 | polyketide\_synthase | BGC0001011 | NRP+Polyketide | 34.0 | 22.3 | 402.0 | 3.02e-111 |
| ctg1\_orf22 |  | BGC0001013 | NRP+Polyketide | 34.0 | 22.3 | 402.0 | 3.02e-111 |
| AJO72736.1 | Type\_I\_modular\_polyketide\_synthase | BGC0001381 | Polyketide | 34.0 | 22.7 | 402.0 | 3.05e-111 |
| CAN89636.1 | putative\_polyketide\_synthase | BGC0001070 | NRP+Polyketide:Modular type I polyketide+Polyketide:Trans-AT type I polyketide | 33.0 | 22.7 | 400.0 | 3.05e-111 |
| BCB17029.1 | modular\_polyketide\_synthase | BGC0002523 | NRP | 32.0 | 22.7 | 401.0 | 3.14e-111 |
| ALA09357.1 | type\_I\_modular\_PKS | BGC0001303 | Polyketide | 34.0 | 22.5 | 400.0 | 3.21e-111 |
| QIQ28617.1 | Nbc21 | BGC0002541 | Other | 32.0 | 23.9 | 401.0 | 3.25e-111 |
| ARW71485.1 | type\_I\_PKS\_module\_4,\_module\_5 | BGC0001812 | Polyketide | 33.0 | 24.4 | 401.0 | 3.33e-111 |
| AXI91552.1 | FunP1 | BGC0001944 | Polyketide | 34.0 | 23.0 | 402.0 | 3.44e-111 |
| AKJ15836.1 | Type\_I\_polyketide\_synthase | BGC0002735 | Polyketide+NRP | 34.0 | 22.6 | 399.0 | 3.6e-111 |
| AAY28225.1 | HbmAI | BGC0000074 | Polyketide | 30.0 | 27.0 | 401.0 | 3.75e-111 |
| BAF85837.1 | modular\_polyketide\_synthase | BGC0000109 | Polyketide | 33.0 | 22.5 | 399.0 | 3.77e-111 |
| BAT51067.1 | type\_I\_polyketide\_synthase | BGC0001296 | Polyketide | 32.0 | 24.4 | 399.0 | 3.91e-111 |
| ALV82345.1 | borrelidin\_type\_I\_polyketide\_synthase | BGC0001533 | Polyketide | 32.0 | 23.4 | 399.0 | 3.96e-111 |
| sipP4 | Type\_I\_Modular\_PKS | BGC0001452 | Polyketide | 33.0 | 22.3 | 394.0 | 4.43e-111 |
| ABV83228.1 | CppA | BGC0000116 | Polyketide | 31.0 | 23.6 | 384.0 | 4.73e-111 |
| UMP03506.1 | NmvAV | BGC0002649 | NRP+Polyketide | 34.0 | 22.0 | 399.0 | 5.29e-111 |
| CAC20921.1 | PimS2\_protein | BGC0000125 | Polyketide | 34.0 | 22.3 | 401.0 | 5.44e-111 |
| AQT01393.1 | SgnS2 | BGC0001690 | Polyketide | 34.0 | 22.3 | 401.0 | 5.44e-111 |
| AAG23265.1 | polyketide\_synthase\_extender\_module\_2 | BGC0000148 | Polyketide | 33.0 | 22.5 | 398.0 | 5.56e-111 |
| BAE93730.1 | type\_I\_polyketide\_synthase | BGC0000164 | Polyketide | 32.0 | 24.1 | 400.0 | 5.9e-111 |
| QIQ28636.1 | Nbc40 | BGC0002541 | Other | 32.0 | 23.6 | 400.0 | 6.85e-111 |
| BAD08360.1 | polyketide\_synthase\_modules\_7-8 | BGC0000167 | Polyketide | 32.0 | 22.7 | 400.0 | 7.61e-111 |
| AKA59091.1 | type-I\_PKS | BGC0001619 | Polyketide | 31.0 | 26.6 | 400.0 | 8.27e-111 |
| AOC89001.1 | putative\_nonribosomal\_peptide\_synthetase | BGC0001652 | NRP | 27.0 | 39.6 | 392.0 | 8.33e-111 |
| ADX66460.1 | ScnS3 | BGC0000108 | Polyketide | 31.0 | 27.5 | 395.0 | 8.4e-111 |
| WP\_244927023.1 | type\_I\_polyketide\_synthase | BGC0002104 | NRP+Polyketide | 32.0 | 23.6 | 384.0 | 8.97e-111 |
| AEC13069.1 | fosC | BGC0000060 | Polyketide | 34.0 | 22.6 | 399.0 | 9.22e-111 |
| QEA08892.1 | JenA6 | BGC0002559 | Polyketide | 32.0 | 22.6 | 394.0 | 9.6e-111 |
| AKD43765.1 | HerE | BGC0001349 | NRP+Polyketide | 34.0 | 23.0 | 394.0 | 9.69e-111 |
| AAX98186.1 | polyketide\_synthase\_type\_I | BGC0000052 | Polyketide | 33.0 | 22.8 | 400.0 | 1.09e-110 |
| MBE8994631.1 | amino\_acid\_adenylation\_domain-containing\_protein | BGC0002623 | NRP+Polyketide | 28.0 | 30.6 | 384.0 | 1.16e-110 |
| CAJ88184.1 | Type\_I\_modular\_polyketide\_synthase | BGC0000151 | Polyketide:Modular type I polyketide+Saccharide:Hybrid/tailoring saccharide | 35.0 | 22.3 | 399.0 | 1.39e-110 |
| QBG82517.1 | Polyketide\_synthase | BGC0002587 | Polyketide | 33.0 | 22.6 | 397.0 | 1.4e-110 |
| QIZ24102.1 | type\_I\_polyketide\_synthase | BGC0002540 | Polyketide | 31.0 | 23.6 | 399.0 | 1.43e-110 |
| ARS01475.1 | NcmAIII | BGC0001702 | NRP+Polyketide | 33.0 | 23.3 | 392.0 | 1.59e-110 |
| AAX35547.1 | polyketide\_syntase\_2 | BGC0001275 | Polyketide | 32.0 | 23.5 | 394.0 | 1.68e-110 |
| ACO94499.1 | polyketide\_synthase\_type\_I | BGC0000097 | Polyketide:Modular type I polyketide | 30.0 | 26.9 | 398.0 | 1.95e-110 |
| SAI82911.1 | HrnF;\_Macrolactam\_polyketide\_synthase\_type\_I;\_modules\_6-7 | BGC0002101 | Polyketide | 31.0 | 27.0 | 398.0 | 1.96e-110 |
| QSV12661.1 | AvmC | BGC0002456 | Polyketide+NRP | 32.0 | 23.4 | 398.0 | 2.19e-110 |
| CAQ52622.1 | type\_I\_polyketide\_synthase,\_modules\_4-5 | BGC0001066 | Polyketide:Modular type I polyketide | 31.0 | 26.4 | 398.0 | 2.32e-110 |
| CAE45668.1 | borrelidin\_polyketide\_synthase,\_type\_I | BGC0000031 | Polyketide:Modular type I polyketide | 34.0 | 22.3 | 391.0 | 2.53e-110 |
| ABG02263.1 | SalA | BGC0000143 | Polyketide | 33.0 | 23.1 | 393.0 | 3.25e-110 |
| ALV82346.1 | borrelidin\_type\_I\_polyketide\_synthase | BGC0001533 | Polyketide | 34.0 | 22.3 | 390.0 | 4.58e-110 |
| ANY10600.1 | polyketide\_synthase | BGC0001773 | Polyketide | 32.0 | 26.8 | 398.0 | 4.64e-110 |
| ABV83230.1 | CppC | BGC0000116 | Polyketide | 32.0 | 26.8 | 398.0 | 4.8e-110 |
| QIQ28637.1 | Nbc41 | BGC0002541 | Other | 34.0 | 23.1 | 390.0 | 5.17e-110 |
| QPP46760.1 | polyketide\_synthase | BGC0002500 | Polyketide | 33.0 | 23.0 | 396.0 | 5.18e-110 |
| CAJ88185.2 | Type\_I\_modular\_polyketide\_synthase | BGC0000151 | Polyketide:Modular type I polyketide+Saccharide:Hybrid/tailoring saccharide | 34.0 | 22.4 | 397.0 | 5.23e-110 |
| QFU80900.1 | PKS | BGC0002550 | Polyketide | 33.0 | 24.4 | 397.0 | 5.64e-110 |
| ABV83221.1 | CppI | BGC0000116 | Polyketide | 34.0 | 22.6 | 397.0 | 6.08e-110 |
| ANZ22989.1 | ZinF | BGC0001828 | Polyketide | 34.0 | 22.7 | 397.0 | 6.42e-110 |
| ATP76242.1 | NdaC | BGC0001705 | NRP+Polyketide | 33.0 | 24.0 | 396.0 | 6.44e-110 |
| QLD28380.2 | SDR\_family\_NAD(P)-dependent\_oxidoreductase | BGC0002086 | Polyketide | 32.0 | 24.1 | 397.0 | 6.45e-110 |
| AIW00670.1 | mellein\_synthase | BGC0001244 | Polyketide | 33.0 | 22.3 | 392.0 | 6.59e-110 |
| ABJ97437.1 | MerA | BGC0001012 | NRP+Polyketide | 34.0 | 22.2 | 397.0 | 6.65e-110 |
| CAE02605.1 | polyketide\_synthase\_type\_I | BGC0000024 | Polyketide:Modular type I polyketide | 32.0 | 23.0 | 395.0 | 6.76e-110 |
| AAO06916.1 | GdmAI | BGC0000066 | Polyketide | 30.0 | 27.0 | 397.0 | 7.19e-110 |
| AGI99496.1 | Type\_I\_polyketide\_synthase | BGC0001004 | Polyketide:Modular type I polyketide | 33.0 | 24.4 | 397.0 | 7.3e-110 |
| CBZ41586.1 | Type\_I\_modular\_polyketide\_synthase | BGC0000151 | Polyketide:Modular type I polyketide+Saccharide:Hybrid/tailoring saccharide | 33.0 | 22.1 | 390.0 | 7.79e-110 |
| ALA09354.1 | type\_I\_modular\_PKS | BGC0001303 | Polyketide | 34.0 | 22.9 | 392.0 | 7.87e-110 |
| CAE45671.1 | borrelidin\_polyketide\_synthase,\_type\_I | BGC0000031 | Polyketide:Modular type I polyketide | 32.0 | 23.4 | 394.0 | 8.71e-110 |
| BAE93731.1 | type\_I\_polyketide\_synthase | BGC0000164 | Polyketide | 31.0 | 24.0 | 397.0 | 8.87e-110 |
| AWR88398.1 | putative\_beta-ketoacyl\_synthase | BGC0001522 | Polyketide | 33.0 | 22.4 | 390.0 | 8.92e-110 |
| ADC45516.1 | modular\_polyketide\_synthase | BGC0000093 | Polyketide | 35.0 | 22.4 | 395.0 | 8.98e-110 |
| OJF16266.1 | AceP4 | BGC0001491 | Polyketide | 35.0 | 22.4 | 397.0 | 1.03e-109 |
| QIZ24098.1 | type\_I\_polyketide\_synthase | BGC0002540 | Polyketide | 34.0 | 22.4 | 397.0 | 1.04e-109 |
| QIE07126.1 | OvmK4 | BGC0001719 | Polyketide | 33.0 | 24.4 | 395.0 | 1.04e-109 |
| AAO62585.1 | peptide\_sythetase\_polyketide\_synthase\_fusion\_protein | BGC0001016 | NRP+Polyketide | 33.0 | 24.0 | 395.0 | 1.09e-109 |
| AEP40936.1 | polyketide\_synthase\_type\_I | BGC0000021 | Polyketide | 32.0 | 22.8 | 396.0 | 1.09e-109 |
| ctg1\_orf20 |  | BGC0001767 | NRP | 27.0 | 32.6 | 319.0 | 2.26e-86 |
| ABC87509.1 | polyketide\_synthase | BGC0001011 | NRP+Polyketide | 34.0 | 22.3 | 396.0 | 1.14e-109 |
| AAO65799.1 | monensin\_polyketide\_synthase\_modules\_5\_and\_6 | BGC0000100 | Polyketide | 32.0 | 23.0 | 396.0 | 1.18e-109 |
| ANZ52462.1 | MonAIV | BGC0001670 | Polyketide | 32.0 | 23.0 | 396.0 | 1.18e-109 |
| AQH32483.1 | hybrid\_peptide\_synthetase/polyketide\_synthase | BGC0001667 | NRP+Polyketide | 33.0 | 23.1 | 395.0 | 1.43e-109 |
| SAI82912.1 | HrnE;\_Macrolactam\_polyketide\_synthase\_type\_I;\_module\_5 | BGC0002101 | Polyketide | 34.0 | 22.8 | 390.0 | 1.51e-109 |
| ADH04641.1 | TgaC | BGC0001051 | NRP+Polyketide:Modular type I polyketide | 32.0 | 23.3 | 396.0 | 1.59e-109 |
| ADU85981.1 | putative\_modular\_polyketide\_synthase | BGC0000165 | Polyketide:Modular type I polyketide | 33.0 | 23.7 | 388.0 | 1.59e-109 |
| ACB46487.1 | polyketide\_synthase | BGC0000082 | Polyketide | 33.0 | 22.2 | 396.0 | 1.61e-109 |
| QES95478.1 | type\_I\_polyketide\_synthase | BGC0002453 | Polyketide | 33.0 | 23.0 | 392.0 | 1.71e-109 |
| AKL69764.1 | polyketide\_synthase | BGC0002072 | Polyketide:Modular type I polyketide | 30.0 | 28.1 | 395.0 | 1.72e-109 |
| QES95479.1 | type\_I\_polyketide\_synthase | BGC0002453 | Polyketide | 32.0 | 23.0 | 391.0 | 1.87e-109 |
| ARV85764.1 | PieA5\_type\_I\_PKS | BGC0001742 | Polyketide | 31.0 | 24.6 | 392.0 | 1.92e-109 |
| QBG82529.1 | Polyketide\_synthase | BGC0002587 | Polyketide | 31.0 | 28.0 | 395.0 | 2.07e-109 |
| BAP27942.1 | nonribosomal\_peptide\_synthetase | BGC0001085 | NRP+Terpene | 26.0 | 44.8 | 394.0 | 2.14e-109 |
| AKD43764.1 | HerF | BGC0001349 | NRP+Polyketide | 33.0 | 23.3 | 395.0 | 2.21e-109 |
| QSE03591.1 | LcmC | BGC0002333 | Polyketide | 33.0 | 22.4 | 395.0 | 2.31e-109 |
| CCP20047.1 | divK\_protein | BGC0001119 | Polyketide:Modular type I polyketide | 32.0 | 24.5 | 395.0 | 2.43e-109 |
| AZH23823.1 | MgiK | BGC0001971 | NRP+Polyketide | 31.0 | 24.5 | 382.0 | 2.52e-109 |
| QEA08890.1 | JenA4 | BGC0002559 | Polyketide | 31.0 | 27.1 | 395.0 | 2.57e-109 |
| ANY10588.1 | polyketide\_synthase | BGC0001773 | Polyketide | 34.0 | 22.4 | 393.0 | 2.84e-109 |
| CAJ88175.1 | Type\_I\_modular\_polyketide\_synthase | BGC0000151 | Polyketide:Modular type I polyketide+Saccharide:Hybrid/tailoring saccharide | 33.0 | 22.6 | 395.0 | 2.92e-109 |
| AAS98787.1 | polyketide\_synthase/thioesterase | BGC0001001 | NRP+Polyketide | 34.0 | 22.5 | 390.0 | 3.11e-109 |
| ALA09355.1 | type\_I\_modular\_PKS | BGC0001303 | Polyketide | 34.0 | 22.5 | 394.0 | 3.21e-109 |
| AFL48529.1 | laidlomycin\_polyketide\_synthase\_(module\_5\_and\_module\_6) | BGC0000084 | Polyketide | 32.0 | 22.7 | 394.0 | 3.36e-109 |
| QIQ28635.1 | Nbc39 | BGC0002541 | Other | 33.0 | 22.8 | 394.0 | 3.49e-109 |
| BAF02923.1 | type\_I\_polyketide\_synthase | BGC0000073 | Polyketide | 31.0 | 27.0 | 394.0 | 4.15e-109 |
| EHK80169.1 | acyl\_transferase | BGC0001447 | Polyketide | 33.0 | 22.5 | 394.0 | 4.57e-109 |
| AAN32979.1 | BarE | BGC0000962 | NRP+Polyketide:Modular type I polyketide | 33.0 | 24.4 | 389.0 | 4.68e-109 |
| BAD38873.1 | polyketide\_synthase | BGC0000111 | Polyketide | 34.0 | 23.3 | 392.0 | 5.13e-109 |
| ctg1\_orf523 |  | BGC0001199 | Polyketide | 34.0 | 22.4 | 393.0 | 5.15e-109 |
| UHH90025.1 | VicP1 | BGC0002634 | Polyketide+NRP+Other | 34.0 | 22.8 | 394.0 | 5.78e-109 |
| QBL56183.1 | PKS | BGC0002376 | Polyketide | 33.0 | 22.7 | 387.0 | 7.25e-109 |
| CAA60462.1 | polyketide\_synthase | BGC0001040 | NRP+Polyketide | 34.0 | 22.9 | 394.0 | 7.77e-109 |
| AAF71766.1 | nysI | BGC0000115 | Polyketide:Modular type I polyketide+Saccharide:Hybrid/tailoring saccharide | 33.0 | 22.4 | 394.0 | 8.89e-109 |
| AWH12938.1 | StmC | BGC0001784 | Polyketide | 33.0 | 22.2 | 393.0 | 9.29e-109 |
| BAP34734.1 | type\_I\_polyketide\_synthase | BGC0000078 | Polyketide | 31.0 | 27.2 | 393.0 | 9.42e-109 |
| ACC80698.1 | beta-ketoacyl\_synthase | BGC0002677 | Other | 30.0 | 25.2 | 377.0 | 1.31e-108 |
| AAC46028.1 | polyketide\_synthase\_module\_7 | BGC0000113 | Polyketide | 32.0 | 22.3 | 389.0 | 1.43e-108 |
| QLD23838.1 | SDR\_family\_NAD(P)-dependent\_oxidoreductase | BGC0002086 | Polyketide | 32.0 | 23.3 | 388.0 | 1.43e-108 |
| ctg1\_11 |  | BGC0001931 | Polyketide | 32.0 | 22.8 | 389.0 | 1.51e-108 |
| AVX51098.1 | nysI | BGC0001709 | Polyketide | 33.0 | 22.5 | 393.0 | 1.52e-108 |
| BAF85843.1 | modular\_polyketide\_synthase | BGC0000109 | Polyketide | 34.0 | 23.0 | 392.0 | 1.66e-108 |
| AAS98200.1 | MSAS-type\_polyketide\_synthase | BGC0001273 | Polyketide | 32.0 | 23.8 | 388.0 | 1.68e-108 |
| ARW71487.1 | type\_I\_PKS\_module\_7 | BGC0001812 | Polyketide | 32.0 | 23.3 | 388.0 | 1.9e-108 |
| QUQ72344.1 | 3-ketoacyl-CoA\_thiolase | BGC0002349 | Polyketide+Saccharide | 34.0 | 22.4 | 391.0 | 2.26e-108 |
| ACO94472.1 | polyketide\_synthase\_type\_I | BGC0000029 | Polyketide:Modular type I polyketide | 33.0 | 23.0 | 386.0 | 2.43e-108 |
| AKD43761.1 | HerD | BGC0001349 | NRP+Polyketide | 34.0 | 23.0 | 391.0 | 2.48e-108 |
| AEU17897.1 | putative\_type\_I\_PKS | BGC0001072 | Saccharide+Polyketide:Modular type I polyketide+Polyketide:Type II polyketide+Other:Aminocoumarin | 32.0 | 22.7 | 390.0 | 2.57e-108 |
| ALV82320.1 | borrelidin\_type\_I\_polyketide\_synthase | BGC0001533 | Polyketide | 32.0 | 26.8 | 391.0 | 2.59e-108 |
| ABC84470.1 | NIGAVIII | BGC0000114 | Polyketide:Modular type I polyketide | 32.0 | 23.8 | 390.0 | 2.62e-108 |
| QCQ67877.1 | hybrid\_peptide\_synthetase/polyketide\_synthase | BGC0002297 | NRP+Polyketide | 33.0 | 23.1 | 390.0 | 2.82e-108 |
| WP\_226048588.1 | AMP-binding\_protein | BGC0002106 | Polyketide | 32.0 | 23.4 | 387.0 | 3e-108 |
| ABB86408.1 | GelA | BGC0000067 | Polyketide | 30.0 | 27.0 | 392.0 | 3.08e-108 |
| AAQ84156.1 | Plm1 | BGC0000123 | Polyketide | 33.0 | 22.5 | 390.0 | 3.89e-108 |
| ctg1\_orf10 |  | BGC0000053 | Polyketide | 31.0 | 23.4 | 390.0 | 4.27e-108 |
| ESU09199.1 | hypothetical\_protein | BGC0002594 | Polyketide | 31.0 | 23.8 | 388.0 | 4.76e-108 |
| BAF85839.1 | modular\_polyketide\_synthase | BGC0000109 | Polyketide | 34.0 | 22.6 | 391.0 | 5.01e-108 |
| ADU86003.1 | putative\_modular\_polyketide\_synthase | BGC0000165 | Polyketide:Modular type I polyketide | 34.0 | 22.4 | 390.0 | 5.21e-108 |
| QBL56184.1 | PKS | BGC0002376 | Polyketide | 33.0 | 22.9 | 390.0 | 5.88e-108 |
| TMU97089.1 | SDR\_family\_NAD(P)-dependent\_oxidoreductase | BGC0002038 | Polyketide | 32.0 | 24.0 | 389.0 | 5.95e-108 |
| WP\_053138522.1 | type\_I\_polyketide\_synthase | BGC0002033 | Polyketide | 33.0 | 23.1 | 387.0 | 5.98e-108 |
| CAD15508.1 | polyketide\_synthase/non-ribosomal\_peptide\_synthetase | BGC0001014 | NRP:NRP siderophore+Polyketide:Modular type I polyketide+Polyketide:Iterative type I polyketide | 34.0 | 22.8 | 390.0 | 6.99e-108 |
| ADC79639.1 | TamAIII | BGC0001052 | NRP+Polyketide:Modular type I polyketide | 34.0 | 22.6 | 389.0 | 7.17e-108 |
| ADU86004.1 | putative\_modular\_polyketide\_synthase | BGC0000165 | Polyketide:Modular type I polyketide | 34.0 | 22.3 | 390.0 | 7.51e-108 |
| BAR73020.1 | putative\_PKS\_(KS-AT-DH-KR-ACP-KS-AT-DH-KR-ACP-KS-AT-DH-KR-ACP) | BGC0001194 | Polyketide | 31.0 | 27.2 | 390.0 | 7.94e-108 |
| AQZ37113.1 | polyketide\_synthase | BGC0001511 | Polyketide | 34.0 | 22.8 | 390.0 | 9.62e-108 |
| AAR16521.1 | RimA | BGC0000138 | Polyketide | 32.0 | 23.4 | 385.0 | 9.86e-108 |
| CAE45669.1 | borrelidin\_polyketide\_synthase,\_type\_I | BGC0000031 | Polyketide:Modular type I polyketide | 32.0 | 26.8 | 389.0 | 9.96e-108 |
| BAG23201.1 | putative\_type-I\_PKS | BGC0002673 | Polyketide+Alkaloid | 34.0 | 23.4 | 390.0 | 9.98e-108 |
| BAQ21947.1 | putative\_type\_I\_polyketide\_synthase | BGC0001204 | Polyketide | 30.0 | 23.9 | 389.0 | 1.03e-107 |
| AVX51099.1 | NysJ | BGC0001709 | Polyketide | 33.0 | 22.8 | 390.0 | 1.06e-107 |
| QKV49789.1 | PKS | BGC0002526 | Polyketide | 31.0 | 23.5 | 382.0 | 1.19e-107 |
| AAO23333.1 | NcpA | BGC0000397 | NRP | 28.0 | 31.5 | 389.0 | 1.4e-107 |
| OJJ97578.1 | hypothetical\_protein | BGC0002229 | Polyketide | 32.0 | 25.5 | 385.0 | 1.42e-107 |
| ATD51278.1 | nonribosomal\_peptide\_synthase | BGC0001650 | NRP | 27.0 | 40.2 | 389.0 | 1.62e-107 |
| CAQ52624.1 | type\_I\_polyketide\_synthase,\_modules\_7-8 | BGC0001066 | Polyketide:Modular type I polyketide | 32.0 | 23.6 | 389.0 | 1.68e-107 |
| TXD00025.1 | SDR\_family\_NAD(P)-dependent\_oxidoreductase | BGC0001877 | Polyketide | 32.0 | 23.8 | 389.0 | 1.79e-107 |
| QFU19838.1 | PKS | BGC0002431 | Polyketide+Saccharide | 32.0 | 23.1 | 383.0 | 2.43e-107 |
| AAB66507.1 | tylactone\_synthase\_module\_6 | BGC0000166 | Polyketide | 33.0 | 23.8 | 382.0 | 2.62e-107 |
| OJF16272.1 | AceP1 | BGC0001491 | Polyketide | 33.0 | 23.4 | 382.0 | 2.72e-107 |
| WP\_055469548.1 | type\_I\_polyketide\_synthase | BGC0001537 | Polyketide | 31.0 | 27.4 | 388.0 | 2.79e-107 |
| ABP55222.1 | beta-ketoacyl\_synthase | BGC0000142 | Polyketide | 33.0 | 24.7 | 387.0 | 3.17e-107 |
| WP\_081238291.1 | type\_I\_polyketide\_synthase | BGC0002105 | Polyketide | 32.0 | 24.0 | 388.0 | 3.78e-107 |
| WP\_035122279.1 | type\_I\_polyketide\_synthase | BGC0001467 | NRP:Cyclic depsipeptide+Polyketide:Modular type I polyketide | 32.0 | 23.0 | 384.0 | 3.83e-107 |
| AKA59092.1 | type-I\_PKS | BGC0001619 | Polyketide | 33.0 | 22.6 | 387.0 | 4.95e-107 |
| BAR73019.1 | putative\_PKS\_(KS-AT-DH-KR-ACP-TE) | BGC0001194 | Polyketide | 32.0 | 27.0 | 385.0 | 5.19e-107 |
| BAF02925.1 | type\_I\_polyketide\_synthase | BGC0000073 | Polyketide | 33.0 | 22.7 | 387.0 | 5.46e-107 |
| CAQ18839.1 | hybrid\_polyketide\_synthase/nonribosomal\_polypetide\_synthetase | BGC0000954 | NRP+Polyketide:Modular type I polyketide | 33.0 | 23.7 | 387.0 | 5.69e-107 |
| ABP55210.1 | beta-ketoacyl\_synthase | BGC0000142 | Polyketide | 33.0 | 22.5 | 387.0 | 6.01e-107 |
| AAQ84157.1 | Plm2-3 | BGC0000123 | Polyketide | 33.0 | 22.7 | 386.0 | 8.19e-107 |
| AEE88283.1 | CurG | BGC0000976 | NRP+Polyketide:Modular type I polyketide | 32.0 | 24.0 | 380.0 | 8.43e-107 |
| AAT70102.1 | CurG | BGC0001165 | NRP+Polyketide:Modular type I polyketide | 32.0 | 24.0 | 380.0 | 8.43e-107 |
| AAQ82564.1 | FscC | BGC0000034 | NRP+Polyketide | 33.0 | 23.7 | 387.0 | 8.73e-107 |
| OJF16268.1 | AceP6 | BGC0001491 | Polyketide | 33.0 | 22.4 | 386.0 | 1.16e-106 |
| AEC13072.1 | fosF | BGC0000060 | Polyketide | 31.0 | 25.0 | 383.0 | 1.17e-106 |
| DAB41918.1 | ArzP\_-\_PKS\_(KS,\_AT,\_OMT,\_ACP,\_TE) | BGC0001884 | NRP+Polyketide | 32.0 | 25.0 | 381.0 | 1.42e-106 |
| AGC09486.1 | LobS3 | BGC0001183 | Polyketide | 31.0 | 24.1 | 382.0 | 1.52e-106 |
| ARE67851.1 | AbsB3 | BGC0001492 | Polyketide | 32.0 | 23.1 | 370.0 | 1.52e-106 |
| BAO66543.1 | type\_I\_polyketide\_synthase | BGC0000042 | Polyketide | 32.0 | 22.7 | 380.0 | 1.63e-106 |
| AAF71768.1 | nysK | BGC0000115 | Polyketide:Modular type I polyketide+Saccharide:Hybrid/tailoring saccharide | 32.0 | 22.9 | 383.0 | 1.91e-106 |
| BAC57031.1 | protomycinolide\_IV\_synthase\_4 | BGC0000102 | Polyketide | 32.0 | 23.7 | 379.0 | 1.96e-106 |
| QUQ72348.1 | type\_I\_polyketide\_synthase | BGC0002349 | Polyketide+Saccharide | 33.0 | 22.4 | 385.0 | 2.13e-106 |
| AHH99919.1 | PKS\_I | BGC0000002 | Polyketide | 33.0 | 22.4 | 385.0 | 2.68e-106 |
| BAG23199.1 | putative\_type-I\_PKS | BGC0002673 | Polyketide+Alkaloid | 31.0 | 26.8 | 383.0 | 2.78e-106 |
| ANY10589.1 | polyketide\_synthase | BGC0001773 | Polyketide | 34.0 | 22.5 | 385.0 | 2.97e-106 |
| AAP42860.1 | NanA6 | BGC0000105 | Polyketide | 31.0 | 23.4 | 380.0 | 2.99e-106 |
| ACO94468.1 | polyketide\_synthase\_type\_I | BGC0000029 | Polyketide:Modular type I polyketide | 33.0 | 22.9 | 384.0 | 3.16e-106 |
| ALD82524.1 | polyketide\_synthase | BGC0001212 | NRP+Polyketide | 29.0 | 31.0 | 381.0 | 3.74e-106 |
| QEA08899.1 | JenA7 | BGC0002559 | Polyketide | 31.0 | 25.1 | 379.0 | 3.76e-106 |
| AVX51100.1 | nysK | BGC0001709 | Polyketide | 32.0 | 22.8 | 382.0 | 4.22e-106 |
| ADU86002.1 | putative\_modular\_polyketide\_synthase | BGC0000165 | Polyketide:Modular type I polyketide | 33.0 | 22.6 | 384.0 | 5.86e-106 |
| WP\_079030450.1 | type\_I\_polyketide\_synthase | BGC0002033 | Polyketide | 32.0 | 23.3 | 381.0 | 7.92e-106 |
| ESK96613.1 | polyketide\_synthase | BGC0002212 | Polyketide | 33.0 | 22.5 | 382.0 | 7.97e-106 |
| AFV30249.1 | polyketide\_synthase | BGC0000075 | Polyketide | 32.0 | 22.5 | 380.0 | 9.77e-106 |
| AAX98185.1 | polyketide\_synthase\_type\_I | BGC0000052 | Polyketide | 30.0 | 27.1 | 382.0 | 1.24e-105 |
| BBM95965.1 | modular\_polyketide\_synthase | BGC0002558 | Polyketide | 32.0 | 23.0 | 379.0 | 1.35e-105 |
| ABI94380.1 | tautomycetin\_biosynthetic\_PKS | BGC0000157 | Polyketide | 30.0 | 26.5 | 383.0 | 1.53e-105 |
| AGC45618.1 | non-ribosomal\_peptide\_synthetase | BGC0001394 | NRP+Polyketide | 28.0 | 37.7 | 375.0 | 1.65e-105 |
| CBW75451.1 | Non-ribosomal\_peptide\_synthetase\_modules | BGC0002048 | NRP:Cyclic depsipeptide | 28.0 | 32.8 | 382.0 | 1.68e-105 |
| QIH29229.1 | endopyrrole\_NRPS\_B | BGC0002326 | NRP | 28.0 | 32.8 | 382.0 | 1.68e-105 |
| CBZ41585.1 | Type\_I\_modular\_polyketide\_synthase | BGC0000151 | Polyketide:Modular type I polyketide+Saccharide:Hybrid/tailoring saccharide | 33.0 | 22.3 | 382.0 | 1.68e-105 |
| AHB82070.1 | polyketide\_synthase | BGC0001231 | NRP+Polyketide:Modular type I polyketide | 32.0 | 22.5 | 375.0 | 1.71e-105 |
| ABP55223.1 | beta-ketoacyl\_synthase | BGC0000142 | Polyketide | 32.0 | 26.8 | 382.0 | 2.14e-105 |
| QNN81299.1 | IonAIII | BGC0002446 | Polyketide | 31.0 | 27.7 | 379.0 | 2.25e-105 |
| ABP55220.1 | beta-ketoacyl\_synthase | BGC0000142 | Polyketide | 32.0 | 27.0 | 382.0 | 2.3e-105 |
| BAC57030.1 | protomycinolide\_IV\_synthase\_3 | BGC0000102 | Polyketide | 32.0 | 25.0 | 381.0 | 2.96e-105 |
| AEC13079.1 | fosA | BGC0000060 | Polyketide | 33.0 | 23.4 | 381.0 | 3.48e-105 |
| ABV83223.1 | CppK | BGC0000116 | Polyketide | 32.0 | 26.8 | 379.0 | 3.52e-105 |
| AHD05619.1 | putative\_polyketide\_synthase\_subunit | BGC0001033 | NRP+Polyketide | 32.0 | 22.6 | 375.0 | 3.55e-105 |
| AFU82614.1 | mixed\_NRPS\_PKS | BGC0000998 | NRP+Polyketide | 31.0 | 25.3 | 380.0 | 4.56e-105 |
| QSE03601.1 | LcmB | BGC0002333 | Polyketide | 34.0 | 22.2 | 380.0 | 7.23e-105 |
| WP\_041754829.1 | non-ribosomal\_peptide\_synthetase | BGC0001844 | NRP:Lipopeptide | 29.0 | 32.6 | 380.0 | 7.28e-105 |
| AEC13071.1 | fosE | BGC0000060 | Polyketide | 30.0 | 27.7 | 380.0 | 8.42e-105 |
| CAO85897.1 | modular\_polyketide\_synthase\_NorB | BGC0000110 | Polyketide:Modular type I polyketide | 32.0 | 22.6 | 378.0 | 8.77e-105 |
| AAD03048.1 | type\_I\_polyketide\_synthase | BGC0000041 | Polyketide | 33.0 | 22.4 | 378.0 | 8.88e-105 |
| sipP5 | Type\_I\_Modular\_PKS | BGC0001452 | Polyketide | 33.0 | 22.6 | 380.0 | 9.05e-105 |
| AWR88393.1 | putative\_beta-ketoacyl\_synthase | BGC0001522 | Polyketide | 33.0 | 22.7 | 380.0 | 9.07e-105 |
| AWH12671.1 | RmpA1 | BGC0001759 | Polyketide | 32.0 | 22.8 | 379.0 | 9.54e-105 |
| ALV82335.1 | borrelidin\_type\_I\_polyketide\_synthase | BGC0001533 | Polyketide | 34.0 | 22.4 | 375.0 | 1.14e-104 |
| BAP34763.1 | type\_I\_polyketide\_synthase | BGC0000078 | Polyketide | 31.0 | 25.1 | 379.0 | 1.21e-104 |
| OJF16270.1 | AceP3 | BGC0001491 | Polyketide | 34.0 | 22.1 | 380.0 | 1.4e-104 |
| SAI82908.1 | HrnD;\_Macrolactam\_polyketidesynthase\_type\_I;\_modules\_3-4 | BGC0002101 | Polyketide | 33.0 | 22.8 | 379.0 | 1.79e-104 |
| BCK51633.1 | modular\_polyketide\_synthase | BGC0002520 | Polyketide | 33.0 | 23.3 | 379.0 | 1.87e-104 |
| CAC22144.1 | CpkC;\_Polyketide\_synthase\_module\_5 | BGC0000038 | Polyketide:Modular type I polyketide | 32.0 | 22.2 | 377.0 | 2.49e-104 |
| AHA38202.1 | GphI | BGC0000069 | Polyketide | 34.0 | 22.9 | 377.0 | 2.82e-104 |
| CAE45672.1 | Borrelidin\_polyketide\_synthase,\_type\_I | BGC0000031 | Polyketide:Modular type I polyketide | 34.0 | 22.3 | 374.0 | 3.49e-104 |
| BAQ25512.1 | type\_I\_polyketide\_synthase | BGC0001288 | Polyketide | 31.0 | 25.1 | 378.0 | 3.8e-104 |
| BAE93725.1 | type\_I\_polyketide\_synthase | BGC0000164 | Polyketide | 32.0 | 22.4 | 372.0 | 5.39e-104 |
| ABI94379.1 | tautomycetin\_biosynthetic\_PKS | BGC0000157 | Polyketide | 32.0 | 22.6 | 377.0 | 6.98e-104 |
| ABV91286.1 | type\_I\_modular\_polyketide\_synthase | BGC0000158 | Polyketide:Modular type I polyketide | 32.0 | 22.6 | 377.0 | 1.19e-103 |
| AEU11003.1 | NpnC | BGC0001029 | NRP+Polyketide | 27.0 | 31.2 | 376.0 | 1.62e-103 |
| AXI91550.1 | FunP3 | BGC0001944 | Polyketide | 32.0 | 24.2 | 375.0 | 1.78e-103 |
| ABX60161.1 | mixed\_NRPS/PKS | BGC0000978 | NRP+Alkaloid+Polyketide:Modular type I polyketide | 24.0 | 59.5 | 375.0 | 2.18e-103 |
| CCA29203.1 | non-ribosomal\_peptide\_synthetase/polyketide\_synthase | BGC0000955 | NRP+Polyketide:Modular type I polyketide | 33.0 | 22.5 | 375.0 | 2.2e-103 |
| AAF19812.1 | MtaD | BGC0001024 | NRP+Polyketide:Modular type I polyketide | 29.0 | 28.2 | 374.0 | 3.37e-103 |
| AAK57184.1 | MxaA | BGC0001022 | NRP+Polyketide | 26.0 | 43.7 | 367.0 | 7.17e-103 |
| ABV91287.1 | type\_I\_modular\_polyketide\_synthase | BGC0000158 | Polyketide:Modular type I polyketide | 30.0 | 26.6 | 374.0 | 7.32e-103 |
| ADH04682.1 | polyketide\_synthase | BGC0001344 | NRP+Polyketide | 34.0 | 22.7 | 373.0 | 8.07e-103 |
| SJZ83675.1 | non-ribosomal\_peptide\_synthase\_domain\_TIGR01720/amino\_acid\_adenylation\_domain-containing\_protein/thioester\_reductase\_domain-containing\_protein | BGC0002660 | NRP | 27.0 | 37.0 | 374.0 | 8.9e-103 |
| WP\_234353271.1 | SDR\_family\_NAD(P)-dependent\_oxidoreductase | BGC0001537 | Polyketide | 31.0 | 27.4 | 371.0 | 1.29e-102 |
| CBW75453.1 | Non-ribosomal\_peptide\_synthetase\_modules\_(EC\_6.3.2.-) | BGC0002048 | NRP:Cyclic depsipeptide | 28.0 | 33.3 | 372.0 | 1.83e-102 |
| ACO78737.1 | Non-ribosomal\_peptide\_synthase,\_PvdD/PvdJ-like\_protein | BGC0002433 | NRP | 27.0 | 32.6 | 372.0 | 2.42e-102 |
| ATD51280.1 | nonribosomal\_peptide\_synthase | BGC0001650 | NRP | 26.0 | 37.6 | 366.0 | 2.43e-102 |
| EPH46607.1 | putative\_Phenolphthiocerol\_synthesis\_polyketide\_synthase\_type\_I\_Pks15/1 | BGC0001519 | NRP+Polyketide | 32.0 | 23.0 | 370.0 | 2.81e-102 |
| QIH29228.1 | endopyrrole\_NRPS\_A | BGC0002326 | NRP | 28.0 | 33.3 | 372.0 | 3.16e-102 |
| CDG12864.1 | non-ribosomal\_peptide\_synthetase | BGC0001415 | NRP+Polyketide | 32.0 | 22.9 | 372.0 | 3.2e-102 |
| AEP40932.1 | polyketide\_synthase\_type\_I | BGC0000021 | Polyketide | 32.0 | 22.6 | 369.0 | 3.43e-102 |
| WP\_030498975.1 | type\_I\_polyketide\_synthase | BGC0001327 | NRP:Cyclic depsipeptide+Polyketide:Modular type I polyketide | 33.0 | 22.8 | 369.0 | 3.58e-102 |
| ACO94496.1 | polyketide\_synthase\_type\_I | BGC0000097 | Polyketide:Modular type I polyketide | 33.0 | 22.8 | 371.0 | 3.89e-102 |
| ADF88279.1 | mixed\_NRPS/PKS | BGC0000981 | NRP+Polyketide | 23.0 | 63.2 | 370.0 | 4.24e-102 |
| AQZ37114.1 | polyketide\_synthase | BGC0001511 | Polyketide | 32.0 | 22.2 | 370.0 | 4.7e-102 |
| QBC75448.1 | MacA | BGC0002615 | Terpene | 31.0 | 23.8 | 367.0 | 5.08e-102 |
| CAD17793.1 | probable\_non\_ribosomal\_peptide\_synthetase\_protein | BGC0001363 | NRP+Polyketide | 28.0 | 32.7 | 371.0 | 5.75e-102 |
| WP\_081238289.1 | type\_I\_polyketide\_synthase | BGC0002105 | Polyketide | 32.0 | 22.2 | 368.0 | 6.86e-102 |
| WP\_013428324.1 | non-ribosomal\_peptide\_synthetase | BGC0001758 | NRP | 27.0 | 33.5 | 370.0 | 8.22e-102 |
| QSJ20139.1 | non-ribosomal\_peptide\_synthase/polyketide\_synthase | BGC0002572 | NRP+Polyketide | 27.0 | 31.3 | 370.0 | 9.08e-102 |
| AHZ20784.1 | non-ribosomal\_peptide\_synthase | BGC0000369 | NRP+Saccharide:Hybrid/tailoring saccharide | 26.0 | 33.2 | 369.0 | 9.16e-102 |
| ABY21542.1 | AngAV | BGC0000018 | Polyketide | 31.0 | 23.4 | 368.0 | 9.27e-102 |
| OJF16269.1 | AceP2 | BGC0001491 | Polyketide | 32.0 | 23.2 | 370.0 | 1.18e-101 |
| AWR88399.1 | putative\_beta-ketoacyl\_synthase | BGC0001522 | Polyketide | 32.0 | 23.6 | 369.0 | 1.23e-101 |
| BAW32334.1 | hybrid\_cis-AT\_polyketide\_synthase\_-\_nonribosomal\_peptide\_synthetase | BGC0001631 | NRP+Polyketide | 33.0 | 22.5 | 369.0 | 1.37e-101 |
| CAD29795.1 | peptide\_synthetase | BGC0001015 | NRP+Polyketide | 32.0 | 24.0 | 369.0 | 1.37e-101 |
| EHK80165.1 | beta-ketoacyl\_synthase | BGC0001447 | Polyketide | 32.0 | 22.7 | 367.0 | 1.58e-101 |
| AGZ15474.1 | putative\_type\_I\_polyketide\_synthase | BGC0001036 | NRP+Polyketide | 32.0 | 23.8 | 367.0 | 1.89e-101 |
| EHA22196.1 | polyketide\_synthase | BGC0000170 | Polyketide | 31.0 | 24.5 | 365.0 | 2e-101 |
| CBD77746.1 | non-ribosomal\_peptide\_synthetase/polyketide\_synthase | BGC0000974 | NRP+Polyketide | 33.0 | 22.6 | 368.0 | 2.86e-101 |
| AAW03327.1 | CtaD | BGC0000982 | NRP+Polyketide | 30.0 | 28.0 | 368.0 | 3.26e-101 |
| PKY07881.1 | hypothetical\_protein | BGC0001544 | NRP+Polyketide | 29.0 | 27.8 | 367.0 | 4.38e-101 |
| ACO78738.1 | Non-ribosomal\_peptide\_synthase,\_PvdJ(2)-like\_protein | BGC0002433 | NRP | 27.0 | 31.4 | 368.0 | 4.4e-101 |
| OAP25815.1 | Phenolphthiocerol\_synthesis\_polyketide\_synthase\_type\_I\_Pks15/1 | BGC0001658 | Polyketide | 32.0 | 22.6 | 367.0 | 4.53e-101 |
| WP\_012408786.1 | non-ribosomal\_peptide\_synthetase | BGC0002061 | NRP:Cyclic depsipeptide+Polyketide:Modular type I polyketide | 27.0 | 30.4 | 366.0 | 6.39e-101 |
| WP\_039806854.1 | type\_I\_polyketide\_synthase | BGC0002001 | NRP+Polyketide | 33.0 | 22.7 | 361.0 | 7.16e-101 |
| EJP62792.1 | polyketide\_synthase | BGC0001720 | Polyketide | 31.0 | 22.7 | 365.0 | 7.49e-101 |
| AHH99924.1 | PKS\_I | BGC0000002 | Polyketide | 33.0 | 22.3 | 363.0 | 9.32e-101 |
| WP\_039806856.1 | non-ribosomal\_peptide\_synthetase | BGC0002001 | NRP+Polyketide | 28.0 | 31.9 | 363.0 | 9.88e-101 |
| QSE03603.1 | LcmE | BGC0002333 | Polyketide | 31.0 | 22.4 | 364.0 | 1.05e-100 |
| QUQ72349.1 | type\_I\_polyketide\_synthase | BGC0002349 | Polyketide+Saccharide | 31.0 | 24.5 | 364.0 | 1.08e-100 |
| AAF17281.1 | nosD | BGC0001028 | Polyketide+NRP:Cyclic depsipeptide | 27.0 | 29.8 | 365.0 | 1.74e-100 |
| ABM21569.1 | crpA | BGC0000975 | NRP+Polyketide | 23.0 | 62.2 | 365.0 | 1.87e-100 |
| ABI26077.1 | OciA | BGC0000331 | NRP | 26.0 | 29.7 | 365.0 | 2.28e-100 |
| AJF34464.1 | Txo2 | BGC0001207 | NRP | 29.0 | 30.1 | 365.0 | 3.31e-100 |
| WP\_018540607.1 | non-ribosomal\_peptide\_synthetase | BGC0001332 | NRP+Polyketide | 28.0 | 29.8 | 364.0 | 3.45e-100 |
| UHH90011.1 | VicP3 | BGC0002634 | Polyketide+NRP+Other | 33.0 | 23.2 | 364.0 | 3.83e-100 |
| WP\_106731933.1 | type\_I\_polyketide\_synthase | BGC0001332 | NRP+Polyketide | 33.0 | 22.5 | 362.0 | 4.34e-100 |
| AHB82057.1 | polyketide\_synthase | BGC0001019 | NRP+Polyketide:Modular type I polyketide | 31.0 | 22.6 | 359.0 | 5.02e-100 |
| AHE80996.1 | PieA6 | BGC0001169 | Polyketide:Modular type I polyketide | 31.0 | 25.2 | 362.0 | 8.36e-100 |
| sipP3 | Type\_I\_Modular\_PKS | BGC0001452 | Polyketide | 31.0 | 23.4 | 363.0 | 1.19e-99 |
| QCQ67881.1 | non-ribosomal\_peptide\_synthetase | BGC0002297 | NRP+Polyketide | 28.0 | 29.7 | 354.0 | 1.44e-99 |
| ABD14711.1 | cesA | BGC0000320 | NRP:Cyclic depsipeptide | 23.0 | 41.6 | 362.0 | 1.46e-99 |
| AIW82284.1 | PuwG | BGC0001125 | NRP+Polyketide | 27.0 | 31.1 | 362.0 | 1.51e-99 |
| CAF05651.1 | TubF\_protein | BGC0001053 | NRP+Polyketide | 33.0 | 22.8 | 361.0 | 2.62e-99 |
| WP\_144411596.1 | non-ribosomal\_peptide\_synthetase | BGC0002001 | NRP+Polyketide | 28.0 | 29.4 | 353.0 | 3.98e-99 |
| AHB82051.1 | polyketide\_synthase | BGC0001019 | NRP+Polyketide:Modular type I polyketide | 30.0 | 29.0 | 361.0 | 4.13e-99 |
| AAK89721.2 | polyketide\_synthetase,\_siderophore\_biosynthesis\_protein | BGC0002107 | NRP+Polyketide | 32.0 | 23.5 | 355.0 | 6.09e-99 |
| AAF00957.1 | mcyG | BGC0001017 | NRP+Polyketide:Modular type I polyketide | 30.0 | 23.9 | 360.0 | 6.8e-99 |
| AWX24483.1 | type\_I\_polyketide\_synthase | BGC0001695 | NRP | 32.0 | 23.9 | 354.0 | 7.09e-99 |
| AJO72735.1 | Type\_I\_modular\_polyketide\_synthase | BGC0001381 | Polyketide | 32.0 | 22.4 | 360.0 | 7.36e-99 |
| KPN90369.1 | NunE | BGC0001416 | NRP | 28.0 | 29.9 | 360.0 | 1.21e-98 |
| AFP87549.1 | NrpS | BGC0001135 | NRP | 26.0 | 33.4 | 359.0 | 1.76e-98 |
| ABI26079.1 | OciC | BGC0000331 | NRP | 28.0 | 30.0 | 352.0 | 1.87e-98 |
| AEO14744.1 | NdaB | BGC0000396 | NRP | 27.0 | 29.3 | 350.0 | 2.13e-98 |
| WP\_030498974.1 | tyrocidine\_synthase\_3 | BGC0001327 | NRP:Cyclic depsipeptide+Polyketide:Modular type I polyketide | 29.0 | 29.8 | 347.0 | 2.21e-98 |
| KPN90376.1 | NupC | BGC0001416 | NRP | 27.0 | 31.5 | 358.0 | 3.61e-98 |
| WP\_039806852.1 | non-ribosomal\_peptide\_synthetase | BGC0002001 | NRP+Polyketide | 28.0 | 29.4 | 346.0 | 4.18e-98 |
| OJF16267.1 | AceP5 | BGC0001491 | Polyketide | 32.0 | 22.2 | 357.0 | 4.52e-98 |
| WP\_100939443.1 | non-ribosomal\_peptide\_synthetase | BGC0002071 | NRP:Lipopeptide | 28.0 | 32.1 | 358.0 | 5.27e-98 |
| MBE8994632.1 | amino\_acid\_adenylation\_domain-containing\_protein | BGC0002623 | NRP+Polyketide | 26.0 | 33.1 | 357.0 | 5.3e-98 |
| ABI26078.1 | OciB | BGC0000331 | NRP | 27.0 | 31.9 | 357.0 | 6.2e-98 |
| ACR50791.1 | putative\_polyketide\_synthase | BGC0000163 | Polyketide | 33.0 | 22.5 | 355.0 | 7.24e-98 |
| QMS47800.1 | JesC | BGC0001629 | NRP:Lipopeptide | 28.0 | 31.9 | 357.0 | 8.07e-98 |
| AJK49757.1 | non-ribosomal\_peptide\_synthase | BGC0002565 | NRP | 28.0 | 29.7 | 356.0 | 1.04e-97 |
| AAF17280.1 | nosC | BGC0001028 | Polyketide+NRP:Cyclic depsipeptide | 26.0 | 31.9 | 356.0 | 1.06e-97 |
| ABV79988.1 | ApnD | BGC0000301 | NRP | 27.0 | 29.5 | 350.0 | 1.07e-97 |
| EWM63002.1 | non-ribosomal\_peptide\_synthetase | BGC0001328 | NRP:Cyclic depsipeptide+Polyketide:Modular type I polyketide | 29.0 | 29.8 | 345.0 | 1.29e-97 |
| AEF33078.1 | dimodular\_nonribosomal\_peptide\_synthetase | BGC0001039 | NRP+Polyketide | 28.0 | 29.5 | 355.0 | 1.36e-97 |
| CAD89775.1 | MelD\_protein | BGC0001010 | NRP+Polyketide:Modular type I polyketide | 28.0 | 29.3 | 356.0 | 1.37e-97 |
| AHB82062.1 | polyketide\_synthase | BGC0001231 | NRP+Polyketide:Modular type I polyketide | 32.0 | 24.8 | 356.0 | 1.47e-97 |
| AQW44894.1 | non-ribosomal\_peptide\_synthetase | BGC0001737 | NRP+Polyketide | 27.0 | 37.6 | 350.0 | 1.93e-97 |
| AXN93581.1 | PuwF-G | BGC0001950 | NRP | 26.0 | 31.6 | 356.0 | 2.01e-97 |
| EED57518.1 | polyketide\_synthase,\_putative | BGC0001446 | Polyketide:Iterative type I polyketide | 32.0 | 25.0 | 353.0 | 2.55e-97 |
| AJK49758.1 | non-ribosomal\_peptide\_synthase | BGC0002565 | NRP | 29.0 | 29.9 | 355.0 | 2.57e-97 |
| ADA69241.1 | cis-AT\_polyketide\_synthase | BGC0001071 | NRP+Polyketide:Modular type I polyketide+Polyketide:Trans-AT type I polyketide | 31.0 | 22.5 | 354.0 | 2.57e-97 |
| EJK79843.1 | amino\_acid\_adenylation\_enzyme/thioester\_reductase\_family\_protein | BGC0000436 | NRP | 32.0 | 22.5 | 355.0 | 2.87e-97 |
| BBD17742.1 | polyketide\_synthase | BGC0001918 | NRP+Polyketide | 33.0 | 22.4 | 351.0 | 2.94e-97 |
| BAY02139.1 | nonribosomal\_protein\_synthetase | BGC0002532 | NRP+Polyketide | 26.0 | 37.7 | 350.0 | 3.55e-97 |
| UHJ79953.1 | non-ribosomal\_peptide\_synthetase | BGC0002654 | NRP | 28.0 | 32.1 | 354.0 | 3.85e-97 |
| AZM57022.1 | non-ribosomal\_peptide\_synthetase | BGC0002314 | NRP | 29.0 | 32.5 | 354.0 | 4.55e-97 |
| CDG17982.1 | Non-ribosomal\_peptide\_synthetase | BGC0000464 | NRP:Cyclic depsipeptide | 28.0 | 29.9 | 354.0 | 5.44e-97 |
| MCF2151708.1 | Non-ribosomal\_peptide\_synthetase | BGC0002625 | NRP+Polyketide | 27.0 | 31.5 | 354.0 | 6.14e-97 |
| ABV79987.1 | ApnC | BGC0000301 | NRP | 27.0 | 29.0 | 353.0 | 6.43e-97 |
| WP\_019032754.1 | type\_I\_polyketide\_synthase | BGC0001331 | NRP:Cyclic depsipeptide+Polyketide:Modular type I polyketide | 33.0 | 22.4 | 351.0 | 6.78e-97 |
| CZT62794.1 | Non-ribosomal\_peptide\_synthase\_involved\_in\_Hassallidin\_biosynthesis | BGC0001614 | NRP | 26.0 | 32.3 | 353.0 | 7.5e-97 |
| AID65225.1 | nonribosomal\_peptide\_synthetase | BGC0000335 | NRP+Polyketide | 27.0 | 31.5 | 353.0 | 7.7e-97 |
| ADZ24995.1 | non-ribosomal\_peptide\_synthase/polyketide\_synthase | BGC0000380 | NRP+Polyketide:Modular type I polyketide | 31.0 | 22.2 | 353.0 | 8.13e-97 |
| BAH22765.1 | nonribosomal\_peptide\_synthetase | BGC0001018 | NRP | 27.0 | 29.7 | 347.0 | 9.95e-97 |
| CAQ34918.1 | nonribosomal\_peptide\_synthetase/\_polyketide\_synthase | BGC0000986 | NRP+Polyketide | 30.0 | 22.6 | 353.0 | 1.05e-96 |
| ACZ55942.1 | non-ribosomal\_peptide\_synthetase | BGC0000302 | NRP | 27.0 | 32.5 | 352.0 | 1.25e-96 |
| AHE80992.1 | PieA2 | BGC0001169 | Polyketide:Modular type I polyketide | 30.0 | 24.9 | 352.0 | 1.58e-96 |
| CBG67541.1 | putative\_non-ribosomal\_peptide\_synthetase | BGC0002367 | NRP | 27.0 | 38.3 | 353.0 | 1.68e-96 |
| ACC81024.1 | non-ribosomal\_peptide\_synthetase | BGC0001479 | NRP | 27.0 | 30.1 | 346.0 | 2.17e-96 |
| AGU50952.1 | putative\_polyketide\_synthase | BGC0002417 | NRP+Polyketide | 32.0 | 22.9 | 347.0 | 2.59e-96 |
| BAY02137.1 | amino\_acid\_adenylation\_domain-containing\_protein | BGC0002532 | NRP+Polyketide | 27.0 | 30.5 | 351.0 | 2.65e-96 |
| AAO62588.1 | peptide\_sythetase | BGC0001016 | NRP+Polyketide | 26.0 | 29.5 | 343.0 | 3.09e-96 |
| ATG32077.1 | polyketide\_synthase | BGC0001750 | NRP+Polyketide | 30.0 | 26.2 | 350.0 | 3.12e-96 |
| AKA54627.1 | PKS | BGC0001216 | NRP+Polyketide | 32.0 | 22.4 | 343.0 | 4.79e-96 |
| CCJ67648.1 | JagD | BGC0001127 | NRP | 28.0 | 31.9 | 350.0 | 5.19e-96 |
| AGD80618.1 | non-ribosomal\_peptide\_synthetase | BGC0000394 | NRP | 26.0 | 38.2 | 345.0 | 5.98e-96 |
| WA1\_15565 | non-ribosomal\_peptide\_synthetase | BGC0002484 | NRP+Polyketide | 27.0 | 31.7 | 350.0 | 6.7e-96 |
| WP\_047890614.1 | type\_I\_polyketide\_synthase | BGC0001330 | NRP:Cyclic depsipeptide+Polyketide:Modular type I polyketide | 32.0 | 22.5 | 348.0 | 8.22e-96 |
| QTT72106.1 | amino\_acid\_adenylation\_domain-containing\_protein | BGC0002350 | NRP+Polyketide+Saccharide | 27.0 | 32.4 | 350.0 | 8.46e-96 |
| AAG02349.1 | peptide\_synthetase\_NRPS11-10 | BGC0000963 | NRP:Glycopeptide+Polyketide:Modular type I polyketide+Saccharide:Hybrid/tailoring saccharide | 27.0 | 32.4 | 350.0 | 8.55e-96 |
| AFO85453.1 | non-ribosomal\_peptide\_synthetase | BGC0000391 | NRP | 28.0 | 37.4 | 350.0 | 8.68e-96 |
| CAD29799.1 | microcystin\_synthetase | BGC0001015 | NRP+Polyketide | 27.0 | 29.8 | 342.0 | 8.72e-96 |
| AQM37584.1 | nonribosomal\_peptide\_synthetase | BGC0001424 | NRP:Cyclic depsipeptide+Polyketide:Iterative type I polyketide | 27.0 | 33.4 | 350.0 | 1.06e-95 |
| QVQ62868.1 | nonribosomal\_peptide\_synthase | BGC0002373 | NRP | 29.0 | 29.7 | 350.0 | 1.33e-95 |
| BAC57028.1 | protomycinolide\_IV\_synthase\_1 | BGC0000102 | Polyketide | 32.0 | 23.3 | 349.0 | 2.16e-95 |
| AHB82059.1 | non\_ribosomal\_peptide\_synthetase/polyketide\_synthase | BGC0001019 | NRP+Polyketide:Modular type I polyketide | 32.0 | 22.6 | 348.0 | 2.58e-95 |
| AGU50951.1 | putative\_non-ribosomal\_peptide\_synthetase | BGC0002417 | NRP+Polyketide | 27.0 | 30.0 | 337.0 | 2.93e-95 |
| ACN39727.1 | SibD | BGC0000428 | NRP | 26.0 | 40.3 | 343.0 | 3.83e-95 |
| ATP76244.1 | NdaB | BGC0001705 | NRP+Polyketide | 27.0 | 29.6 | 340.0 | 4.17e-95 |
| KYC42612.1 | hypothetical\_protein | BGC0002484 | NRP+Polyketide | 27.0 | 31.4 | 348.0 | 4.27e-95 |
| AXN93590.1 | PuwF-G | BGC0001951 | NRP | 27.0 | 29.5 | 348.0 | 5.63e-95 |
| CCM44338.1 | Polyketide\_synthase | BGC0001056 | NRP+Polyketide:Modular type I polyketide+Polyketide:PUFA synthase or related polyketide | 32.0 | 22.6 | 343.0 | 6.32e-95 |
| AAF15892.2 | nosB | BGC0001028 | Polyketide+NRP:Cyclic depsipeptide | 29.0 | 22.6 | 338.0 | 8.02e-95 |
| ACS20361.1 | KR\_domain\_protein | BGC0002420 | NRP+Polyketide | 32.0 | 22.9 | 342.0 | 1.02e-94 |
| AED90003.1 | non-ribosomal\_peptide\_synthetase\_ThaB | BGC0000443 | NRP:Beta-lactam | 27.0 | 29.6 | 347.0 | 1.12e-94 |
| ACZ55946.1 | non-ribosomal\_peptide\_synthetase | BGC0000302 | NRP | 27.0 | 29.5 | 340.0 | 1.15e-94 |
| AAM54078.1 | polyketide\_synthase | BGC0000020 | Polyketide | 31.0 | 24.4 | 346.0 | 1.16e-94 |
| AAO72425.1 | syringopeptin\_synthetase\_C | BGC0000438 | NRP | 26.0 | 31.7 | 347.0 | 1.18e-94 |
| AAY37655.1 | Amino\_acid\_adenylation | BGC0000437 | NRP | 26.0 | 31.7 | 347.0 | 1.18e-94 |
| AED90002.1 | non-ribosomal\_peptide\_synthetase\_ThaA | BGC0000443 | NRP:Beta-lactam | 27.0 | 29.9 | 347.0 | 1.22e-94 |
| AEZ51520.1 | pmxE | BGC0001153 | NRP:Lipopeptide | 26.0 | 32.1 | 346.0 | 1.69e-94 |
| KFA69335.1 | hypothetical\_protein | BGC0001626 | Polyketide | 32.0 | 22.6 | 345.0 | 1.79e-94 |
| CCC55921.1 | non-ribosomal\_peptide\_synthetase/polyketide\_synthase\_hybrid\_protein | BGC0000973 | NRP+Polyketide:Modular type I polyketide | 33.0 | 22.6 | 345.0 | 1.84e-94 |
| ACS20360.1 | amino\_acid\_adenylation\_domain\_protein | BGC0002420 | NRP+Polyketide | 27.0 | 29.9 | 335.0 | 2.18e-94 |
| QWP75304.1 | non-ribosomal\_peptide\_synthase | BGC0002126 | NRP:Cyclic depsipeptide | 26.0 | 33.5 | 345.0 | 2.22e-94 |
| AAY93355.1 | non-ribosomal\_peptide\_synthetase\_PvdJ | BGC0000413 | NRP | 26.0 | 31.4 | 344.0 | 2.23e-94 |
| CAJ76291.1 | putative\_polyketide\_synthase | BGC0000972 | NRP+Polyketide:Modular type I polyketide+Polyketide:Trans-AT type I polyketide | 30.0 | 23.7 | 332.0 | 2.39e-94 |
| APZ78856.1 | nonribosomal\_peptide\_synthetase | BGC0001432 | NRP:Cyclic depsipeptide+Polyketide:Iterative type I polyketide | 27.0 | 31.6 | 345.0 | 2.48e-94 |
| KPN93065.1 | NunD | BGC0001416 | NRP | 27.0 | 32.1 | 345.0 | 2.62e-94 |
| AQH32486.1 | peptide\_synthetase | BGC0001667 | NRP+Polyketide | 26.0 | 29.4 | 337.0 | 3.11e-94 |
| AKP45395.1 | CysG | BGC0001413 | NRP | 28.0 | 31.7 | 343.0 | 3.6e-94 |
| BAC67536.1 | arthrofactin\_synthetase\_C | BGC0000305 | NRP:Lipopeptide | 27.0 | 31.6 | 345.0 | 3.7e-94 |
| AHE80991.1 | PieA1 | BGC0001169 | Polyketide:Modular type I polyketide | 30.0 | 24.8 | 343.0 | 4.94e-94 |
| BAH43870.1 | putative\_linear\_pentadecapeptide\_gramicidin\_synthetase\_LgrB | BGC0000367 | NRP | 27.0 | 29.6 | 345.0 | 5.28e-94 |
| ctg1\_orf1264 |  | BGC0001752 | NRP | 28.0 | 30.1 | 344.0 | 5.42e-94 |
| QKF54436.1 | nonribosomal\_peptide\_synthetase | BGC0002581 | NRP | 28.0 | 32.2 | 344.0 | 5.6e-94 |
| ALV82356.1 | CDA\_peptide\_synthetase\_I | BGC0001370 | NRP | 28.0 | 33.1 | 344.0 | 6.78e-94 |
| BAV19379.1 | polyketide\_synthase | BGC0001390 | NRP+Polyketide | 32.0 | 22.6 | 342.0 | 8.97e-94 |
| UEF20578.1 | nonribosomal\_peptide\_synthetase | BGC0002360 | NRP | 28.0 | 32.2 | 343.0 | 9.59e-94 |
| ACA97580.1 | PmxE | BGC0000408 | NRP | 26.0 | 32.1 | 343.0 | 1.11e-93 |
| APZ78680.1 | nonribosomal\_peptide\_synthetase | BGC0001417 | NRP:Cyclic depsipeptide+Polyketide:Iterative type I polyketide | 27.0 | 31.6 | 343.0 | 1.13e-93 |
| EAT86855.2 | hypothetical\_protein | BGC0001858 | Polyketide | 33.0 | 21.6 | 342.0 | 1.17e-93 |
| APZ78729.1 | nonribosomal\_peptide\_synthetase | BGC0001421 | NRP:Cyclic depsipeptide+Polyketide:Iterative type I polyketide | 27.0 | 31.9 | 343.0 | 1.38e-93 |
| WP\_064118559.1 | non-ribosomal\_peptide\_synthase/polyketide\_synthase | BGC0001509 | NRP | 27.0 | 31.7 | 343.0 | 1.42e-93 |
| CAJ34381.1 | NRPS\_protein | BGC0000445 | NRP:Cyclic depsipeptide | 29.0 | 30.0 | 340.0 | 1.45e-93 |
| AFV52200.1 | polyketide\_synthase\_module | BGC0000081 | NRP+Polyketide:Iterative type I polyketide+Polyketide:Enediyne type I polyketide | 31.0 | 23.0 | 340.0 | 1.9e-93 |
| ATJ34002.1 | non-ribosomal\_peptide\_synthetase | BGC0001442 | NRP | 26.0 | 38.3 | 338.0 | 2.73e-93 |
| AAZ03554.1 | McnE | BGC0000332 | NRP | 26.0 | 29.8 | 336.0 | 3.05e-93 |
| AVI26393.1 | nonribosomal\_peptide\_synthase | BGC0001800 | NRP+Polyketide | 27.0 | 31.8 | 342.0 | 3.41e-93 |
| XP\_028481820.1 | non-reducing\_polyketide\_synthase | BGC0001866 | Polyketide | 30.0 | 24.9 | 340.0 | 3.7e-93 |
| WP\_012408784.1 | acyltransferase\_domain-containing\_protein | BGC0002061 | NRP:Cyclic depsipeptide+Polyketide:Modular type I polyketide | 29.0 | 22.8 | 333.0 | 4.13e-93 |
| AJF34463.1 | Txo1 | BGC0001207 | NRP | 28.0 | 31.4 | 341.0 | 5.58e-93 |
| AXA91302.1 | non-ribosomal\_peptide\_synthetase | BGC0002044 | NRP | 27.0 | 31.9 | 340.0 | 8.88e-93 |
| QBC75022.1 | non-ribosomal\_peptide\_synthetase | BGC0001968 | NRP | 27.0 | 35.5 | 340.0 | 9.14e-93 |
| ADZ24999.1 | non-ribosomal\_peptide\_synthase | BGC0000380 | NRP+Polyketide:Modular type I polyketide | 27.0 | 30.9 | 335.0 | 9.45e-93 |
| WP\_012988806.1 | non-ribosomal\_peptide\_synthetase | BGC0002135 | NRP:Lipopeptide | 28.0 | 30.0 | 340.0 | 9.65e-93 |
| AHB82072.1 | non\_ribosomal\_peptide\_synthetase/polyketide\_synthase | BGC0001231 | NRP+Polyketide:Modular type I polyketide | 33.0 | 22.8 | 340.0 | 9.92e-93 |
| ACM68684.1 | AerB | BGC0000298 | NRP | 27.0 | 29.9 | 336.0 | 1.15e-92 |
| AAY93354.1 | non-ribosomal\_peptide\_synthetase\_PvdD | BGC0000413 | NRP | 27.0 | 29.6 | 340.0 | 1.38e-92 |
| WP\_011146892.1 | non-ribosomal\_peptide\_synthetase | BGC0001641 | NRP | 28.0 | 29.6 | 340.0 | 1.5e-92 |
| BBA21073.1 | putative\_non-ribosomal\_peptide\_synthetase | BGC0001740 | NRP+Polyketide | 28.0 | 29.7 | 339.0 | 1.55e-92 |
| QPI18727.1 | nonribosomal\_peptide\_synthetase | BGC0002125 | NRP:Cyclic depsipeptide | 28.0 | 29.9 | 339.0 | 1.74e-92 |
| AEW31020.1 | plipastatin\_synthetase | BGC0000407 | NRP | 26.0 | 31.8 | 338.0 | 1.81e-92 |
| QYA95663.1 | acyltransferase\_domain-containing\_protein | BGC0002676 | NRP | 31.0 | 22.5 | 334.0 | 2.01e-92 |
| QMN69934.1 | PsoC | BGC0002521 | NRP | 27.0 | 31.6 | 339.0 | 2.14e-92 |
| CCT67991.1 | bikaverin\_cluster-polyketide\_synthase | BGC0000030 | Polyketide | 29.0 | 25.2 | 337.0 | 3.06e-92 |
| ALV86866.1 | Tlo20 | BGC0001406 | NRP | 29.0 | 29.7 | 338.0 | 3.23e-92 |
| AIW82283.1 | PuwF | BGC0001125 | NRP+Polyketide | 27.0 | 29.7 | 337.0 | 3.53e-92 |
| AFJ23826.1 | WLIP\_synthetase\_C | BGC0001838 | NRP | 27.0 | 31.8 | 338.0 | 3.68e-92 |
| AXN93614.1 | PuwF | BGC0001953 | NRP | 27.0 | 29.3 | 338.0 | 3.7e-92 |
| KYC42747.1 | hypothetical\_protein | BGC0002484 | NRP+Polyketide | 26.0 | 32.1 | 338.0 | 3.7e-92 |
| AHD05615.1 | putative\_non-ribosomal\_peptide\_ligase/\_polyketide\_synthase\_hybrid | BGC0001033 | NRP+Polyketide | 24.0 | 41.5 | 338.0 | 3.89e-92 |
| ABB90282.1 | polyketide\_synthase | BGC0001057 | NRP+Polyketide | 29.0 | 24.6 | 337.0 | 4.02e-92 |
| AAY91421.3 | non-ribosomal\_peptide\_synthetase\_OfaC | BGC0000399 | NRP:Cyclic depsipeptide | 27.0 | 32.5 | 338.0 | 4.28e-92 |
| ABY83163.1 | Azi25 | BGC0000960 | NRP+Polyketide | 26.0 | 37.0 | 334.0 | 4.4e-92 |
| ABX37383.1 | amino\_acid\_adenylation\_domain\_protein | BGC0000984 | NRP+Polyketide | 27.0 | 30.2 | 337.0 | 5.65e-92 |
| PHM49485.1 | Amino\_acid\_adenylation | BGC0001131 | NRP | 27.0 | 32.4 | 338.0 | 6.31e-92 |
| AHB82069.1 | non\_ribosomal\_peptide\_synthetase | BGC0001231 | NRP+Polyketide:Modular type I polyketide | 28.0 | 32.6 | 336.0 | 6.6e-92 |
| WP\_053065270.1 | non-ribosomal\_peptide\_synthetase | BGC0001330 | NRP:Cyclic depsipeptide+Polyketide:Modular type I polyketide | 29.0 | 25.7 | 335.0 | 6.68e-92 |
| KYC42613.1 | non-ribosomal\_peptide\_synthetase | BGC0002484 | NRP+Polyketide | 28.0 | 31.5 | 337.0 | 7.43e-92 |
| WP\_126241403.1 | non-ribosomal\_peptide\_synthetase | BGC0002336 | NRP | 27.0 | 32.9 | 337.0 | 9.22e-92 |
| BAP05597.1 | calI | BGC0000967 | NRP+Polyketide:Trans-AT type I polyketide | 27.0 | 32.9 | 336.0 | 9.8e-92 |
| AAO56329.1 | non-ribosomal\_peptide\_synthetase\_SyfB | BGC0000435 | NRP | 27.0 | 32.1 | 337.0 | 1.04e-91 |
| AEI70245.1 | nonribosomal\_peptide\_synthetase\_NRPS | BGC0000401 | NRP | 28.0 | 33.1 | 336.0 | 1.04e-91 |
| AJM89738.1 | PmxE | BGC0001192 | NRP | 26.0 | 32.0 | 337.0 | 1.06e-91 |
| BBD17760.1 | polyketide\_synthase | BGC0001919 | NRP+Polyketide | 31.0 | 24.7 | 331.0 | 1.16e-91 |
| BAV57443.1 | NRPS\_(C-A-PCP-TE) | BGC0001818 | NRP | 27.0 | 29.5 | 331.0 | 1.26e-91 |
| QNL14925.1 | AptD | BGC0002512 | NRP | 27.0 | 29.3 | 331.0 | 1.36e-91 |
| EED21099.1 | polyketide\_synthase,\_putative | BGC0001578 | Polyketide | 30.0 | 24.8 | 335.0 | 1.43e-91 |
| ACC81021.1 | non-ribosomal\_peptide\_synthetase | BGC0001479 | NRP | 26.0 | 32.5 | 335.0 | 1.47e-91 |
| AEA30273.1 | peptide\_synthetase | BGC0000429 | Polyketide+NRP:Cyclic depsipeptide | 28.0 | 29.4 | 336.0 | 1.61e-91 |
| AWI62628.1 | nonribosomal\_peptide\_synthetase | BGC0001822 | NRP | 28.0 | 32.4 | 336.0 | 1.65e-91 |
| ADI24926.1 | VrtA | BGC0000168 | Polyketide:Iterative type I polyketide | 30.0 | 22.9 | 334.0 | 1.66e-91 |
| AXN93592.1 | PuwA | BGC0001952 | NRP | 27.0 | 29.8 | 335.0 | 1.84e-91 |
| AFH75322.1 | nonribosomal\_peptide\_synthetase | BGC0000425 | NRP:Cyclic depsipeptide | 27.0 | 29.8 | 336.0 | 1.88e-91 |
| WP\_019032753.1 | non-ribosomal\_peptide\_synthetase | BGC0001331 | NRP:Cyclic depsipeptide+Polyketide:Modular type I polyketide | 30.0 | 25.6 | 334.0 | 2e-91 |
| ALG65339.1 | Var4 | BGC0002416 | NRP+Polyketide | 31.0 | 23.5 | 335.0 | 2.29e-91 |
| AXN93602.1 | PuwF-G | BGC0001952 | NRP | 27.0 | 29.4 | 336.0 | 2.31e-91 |
| AJD47482.1 | erythronolide\_synthase | BGC0002418 | NRP+Polyketide | 32.0 | 23.4 | 334.0 | 2.63e-91 |
| ABE35421.1 | Non-ribosomal\_peptide\_synthetase | BGC0002421 | NRP | 27.0 | 29.5 | 332.0 | 2.85e-91 |
| AAP92491.1 | nonribosomal\_peptide\_synthetase | BGC0000458 | NRP | 28.0 | 30.1 | 334.0 | 3.08e-91 |
| CCJ67640.1 | TaaE | BGC0000447 | NRP:Lipopeptide | 27.0 | 31.3 | 335.0 | 3.21e-91 |
| AGM16414.1 | paenibacterin\_synthetase\_C | BGC0000400 | NRP | 26.0 | 32.1 | 335.0 | 3.54e-91 |
| QYA95681.1 | amino\_acid\_adenylation\_domain-containing\_protein | BGC0002676 | NRP | 28.0 | 29.4 | 335.0 | 3.6e-91 |
| BAW27693.1 | NRPS(C-A-T-TE) | BGC0001764 | NRP | 28.0 | 29.7 | 329.0 | 3.61e-91 |
| ABD65957.1 | nonribosomal\_peptide\_synthetase | BGC0000341 | NRP | 30.0 | 29.6 | 335.0 | 4.18e-91 |
| AHJ31215.1 | Long-chain-fatty-acid--CoA\_ligase | BGC0000430 | NRP+Polyketide:Modular type I polyketide | 27.0 | 29.6 | 335.0 | 4.39e-91 |
| ACZ55945.1 | non-ribosomal\_peptide\_synthetase | BGC0000302 | NRP | 27.0 | 30.0 | 333.0 | 4.43e-91 |
| AEF33079.1 | polyketide\_synthase | BGC0001039 | NRP+Polyketide | 31.0 | 22.3 | 329.0 | 4.79e-91 |
| WP\_010639241.1 | type\_I\_polyketide\_synthase | BGC0000958 | NRP:Cyclic depsipeptide+Polyketide:Modular type I polyketide | 31.0 | 22.4 | 327.0 | 5e-91 |
| CAJ14039.1 | peptide\_synthetase | BGC0000406 | NRP | 27.0 | 29.8 | 324.0 | 5.57e-91 |
| AFD30954.1 | CrmA | BGC0000966 | NRP+Polyketide | 32.0 | 23.8 | 333.0 | 5.76e-91 |
| EWM63005.1 | linear\_gramicidin\_synthetase\_LgrC | BGC0001328 | NRP:Cyclic depsipeptide+Polyketide:Modular type I polyketide | 29.0 | 26.0 | 322.0 | 6.1e-91 |
| ALV82384.1 | CDA\_peptide\_synthetase\_II | BGC0001370 | NRP | 28.0 | 32.0 | 334.0 | 6.84e-91 |
| AGI89789.1 | Nonribosomal\_peptide\_synthetase | BGC0001792 | NRP | 28.0 | 29.4 | 334.0 | 7.36e-91 |
| SDF67386.1 | non-ribosomal\_peptide\_synthase\_domain\_TIGR01720/amino\_acid\_adenylation\_domain-containing\_protein | BGC0002422 | NRP | 28.0 | 31.8 | 334.0 | 7.98e-91 |
| QRI43520.1 | NRPS/PKS\_hybrid | BGC0002454 | Polyketide | 31.0 | 22.4 | 333.0 | 8.89e-91 |
| QNH85840.1 | BolH | BGC0002327 | NRP | 27.0 | 32.7 | 333.0 | 1.03e-90 |
| QDF82259.1 | non-ribosomal\_peptide\_synthetase | BGC0001980 | NRP | 27.0 | 31.8 | 333.0 | 1.16e-90 |
| ATP76246.1 | SpuB | BGC0001748 | NRP+Polyketide | 27.0 | 29.7 | 333.0 | 1.28e-90 |
| ABX37382.1 | amino\_acid\_adenylation\_domain\_protein | BGC0000984 | NRP+Polyketide | 27.0 | 30.8 | 333.0 | 1.54e-90 |
| mycH | polyketide\_synthase | BGC0002055 | NRP+Polyketide:Trans-AT type I polyketide | 25.0 | 33.2 | 333.0 | 1.6e-90 |
| AKJ15828.1 | peptide\_synthetase | BGC0002735 | Polyketide+NRP | 28.0 | 29.7 | 326.0 | 2.02e-90 |
| WP\_055469550.1 | type\_I\_polyketide\_synthase | BGC0001537 | Polyketide | 31.0 | 23.0 | 332.0 | 2.3e-90 |
| AEP40925.1 | polyketide\_synthase\_type\_I | BGC0000021 | Polyketide | 31.0 | 23.3 | 328.0 | 2.74e-90 |
| QBQ12465.1 | amino\_acid\_adenylation\_domain-containing\_protein | BGC0002693 | NRP | 27.0 | 29.7 | 332.0 | 2.86e-90 |
| AKC91856.1 | nonribosomal\_peptide\_synthetase | BGC0001414 | NRP | 28.0 | 33.1 | 332.0 | 2.88e-90 |
| AAC06347.1 | bacitracin\_synthetase\_2 | BGC0000310 | NRP | 26.0 | 29.5 | 331.0 | 3.14e-90 |
| AMK48226.1 | nonribosomal\_peptide\_synthetase | BGC0001351 | NRP | 27.0 | 32.9 | 331.0 | 3.18e-90 |
| AZF85940.1 | non-ribosomal\_peptide\_synthase | BGC0001963 | NRP+Polyketide | 27.0 | 31.6 | 332.0 | 3.45e-90 |
| AXN93605.1 | PuwA | BGC0001953 | NRP | 27.0 | 30.6 | 331.0 | 3.56e-90 |
| QSJ20140.1 | amino\_acid\_adenylation\_domain-containing\_protein | BGC0002572 | NRP+Polyketide | 27.0 | 29.7 | 328.0 | 4.33e-90 |
| ABX37384.1 | Beta-ketoacyl\_synthase | BGC0000984 | NRP+Polyketide | 31.0 | 23.0 | 328.0 | 4.4e-90 |
| APU91751.1 | Non-Ribosomal\_Peptide\_Synthetase | BGC0001806 | NRP | 27.0 | 29.6 | 332.0 | 4.58e-90 |
| TRX17523.1 | non-ribosomal\_peptide\_synthetase | BGC0002329 | NRP | 27.0 | 31.1 | 329.0 | 5.47e-90 |
| AEH59099.1 | amino\_acid\_adenylation\_domain-containing\_protein/NRPS | BGC0000385 | NRP | 28.0 | 31.4 | 331.0 | 6.1e-90 |
| AIW82277.1 | PuwA | BGC0001125 | NRP+Polyketide | 26.0 | 29.7 | 330.0 | 6.13e-90 |
| AXN93575.1 | PuwA | BGC0001950 | NRP | 26.0 | 29.6 | 330.0 | 8.03e-90 |
| AXN93584.1 | PuwA | BGC0001951 | NRP | 26.0 | 29.6 | 330.0 | 8.03e-90 |
| CBF74114.1 | Conidial\_yellow\_pigment\_biosynthesis\_polyketide\_synthase\_(PKS)(EC\_2.3.1.-)\_[Source:UniProtKB/Swiss-Prot;Acc:Q03149] | BGC0000107 | Polyketide | 31.0 | 22.8 | 329.0 | 8.34e-90 |
| ABV79985.1 | ApnA | BGC0000301 | NRP | 25.0 | 30.1 | 329.0 | 8.43e-90 |
| ACY06285.1 | non-ribosomal\_peptide\_synthetase | BGC0001042 | NRP+Polyketide | 28.0 | 32.9 | 330.0 | 1e-89 |
| ABL74938.1 | PKS | BGC0001048 | NRP:Glycopeptide+Polyketide:Modular type I polyketide+Saccharide:Hybrid/tailoring saccharide | 31.0 | 22.5 | 326.0 | 1.14e-89 |
| UEF20580.1 | nonribosomal\_peptide\_synthetase | BGC0002360 | NRP | 27.0 | 29.9 | 330.0 | 1.39e-89 |
| ATY37590.1 | BogC | BGC0001532 | NRP | 26.0 | 31.6 | 330.0 | 1.53e-89 |
| BBF25315.1 | polyketide\_synthase | BGC0001923 | Terpene+Polyketide | 32.0 | 20.7 | 328.0 | 1.79e-89 |
| QKF54438.1 | nonribosomal\_peptide\_synthetase | BGC0002581 | NRP | 28.0 | 29.9 | 329.0 | 1.82e-89 |
| QTT72099.1 | type\_I\_polyketide\_synthase | BGC0002350 | NRP+Polyketide+Saccharide | 31.0 | 22.5 | 327.0 | 1.84e-89 |
| AFY58523.1 | amino\_acid\_adenylation\_enzyme/thioester\_reductase\_family\_protein | BGC0002411 | NRP+Polyketide | 25.0 | 31.0 | 329.0 | 1.85e-89 |
| ALG65342.1 | Var7 | BGC0002416 | NRP+Polyketide | 27.0 | 30.1 | 328.0 | 1.85e-89 |
| AUW31184.1 | putative\_type\_I\_PKS | BGC0001489 | Polyketide | 31.0 | 22.5 | 328.0 | 1.87e-89 |
| AXN93615.1 | PuwG | BGC0001953 | NRP | 26.0 | 32.1 | 329.0 | 1.88e-89 |
| AHZ34241.1 | CipD | BGC0001389 | NRP | 27.0 | 29.6 | 329.0 | 1.92e-89 |
| CCP42826.1 | Probable\_peptide\_synthetase\_Nrp\_(peptide\_synthase) | BGC0001627 | NRP | 27.0 | 41.0 | 328.0 | 1.96e-89 |
| CDG17986.1 | Non-ribosomal\_peptide\_synthetase | BGC0000464 | NRP:Cyclic depsipeptide | 27.0 | 29.6 | 328.0 | 2.06e-89 |
| AAU39359.1 | lichenysin\_synthase\_LchAA | BGC0000381 | NRP | 26.0 | 31.8 | 329.0 | 2.23e-89 |
| EAU38791.1 | hypothetical\_protein | BGC0000161 | Polyketide:Iterative type I polyketide | 30.0 | 22.5 | 328.0 | 2.24e-89 |
| AAF86395.1 | FkbP | BGC0000994 | NRP+Polyketide | 27.0 | 30.3 | 325.0 | 2.73e-89 |
| CAE15637.1 |  | BGC0001128 | NRP | 26.0 | 31.9 | 329.0 | 2.76e-89 |
| AXG48275.1 | non-ribosomal\_peptide\_synthetase | BGC0002716 | NRP | 26.0 | 31.9 | 329.0 | 2.76e-89 |
| AQX14493.1 | monobactam\_NRPS\_scaffold\_2 | BGC0001672 | NRP | 25.0 | 29.8 | 325.0 | 3.86e-89 |
| AHB38498.1 | polyketide\_synthase | BGC0000346 | NRP+Polyketide:Modular type I polyketide | 33.0 | 24.3 | 327.0 | 4.46e-89 |
| AGU50953.1 | putative\_non-ribosomal\_peptide\_synthetase | BGC0002417 | NRP+Polyketide | 27.0 | 31.8 | 326.0 | 4.51e-89 |
| AHB82056.1 | non\_ribosomal\_peptide\_synthetase | BGC0001019 | NRP+Polyketide:Modular type I polyketide | 29.0 | 32.9 | 327.0 | 4.79e-89 |
| UKO95748.1 | amino\_acid\_adenylation\_domain-containing\_protein | BGC0002632 | NRP | 26.0 | 29.4 | 327.0 | 5.25e-89 |
| BAV69313.1 | PrhL | BGC0001729 | Polyketide+Terpene | 29.0 | 25.5 | 327.0 | 5.63e-89 |
| DAB41484.1 | nonribosomal\_peptide\_synthetase/polyketide\_synthase\_type\_I | BGC0001230 | NRP:Cyclic depsipeptide+Polyketide:Modular type I polyketide | 27.0 | 33.1 | 327.0 | 5.93e-89 |
| CAK15814.1 | putative\_non-ribosomal\_peptide\_synthetase,\_terminal\_component | BGC0000344 | NRP | 27.0 | 32.9 | 328.0 | 5.98e-89 |
| WP\_052165466.1 | non-ribosomal\_peptide\_synthetase | BGC0001327 | NRP:Cyclic depsipeptide+Polyketide:Modular type I polyketide | 27.0 | 30.6 | 323.0 | 7.39e-89 |
| CEK23366.1 | putative\_Ornithine\_racemase | BGC0001716 | NRP | 26.0 | 29.3 | 327.0 | 7.93e-89 |
| EAA59563.1 | polyketide\_synthase | BGC0000057 | Polyketide:Iterative type I polyketide | 29.0 | 25.0 | 325.0 | 1.17e-88 |
| ART41209.1 | AdrD | BGC0001508 | Polyketide | 30.0 | 22.6 | 326.0 | 1.28e-88 |
| CAY48789.1 | putative\_non-ribosomal\_peptide\_synthetase | BGC0001312 | NRP | 26.0 | 32.8 | 326.0 | 1.52e-88 |
| EFE73313.1 | nonribosomal\_peptide\_synthetase | BGC0000431 | NRP:Cyclic depsipeptide | 27.0 | 30.4 | 326.0 | 1.55e-88 |
| CAB38518.1 | CDA\_peptide\_synthetase\_I\_(CdaPs1) | BGC0000315 | NRP:Lipopeptide:Ca+-dependent lipopeptide | 27.0 | 33.6 | 327.0 | 1.56e-88 |
| AEH59100.1 | amino\_acid\_adenylation\_domain-containing\_protein/NRPS | BGC0000385 | NRP | 27.0 | 32.5 | 327.0 | 1.61e-88 |
| WP\_051700112.1 | non-ribosomal\_peptide\_synthetase | BGC0001368 | NRP | 26.0 | 29.7 | 317.0 | 1.73e-88 |
| KYQ85937.1 | hypothetical\_protein | BGC0002437 | NRP | 26.0 | 31.3 | 326.0 | 1.89e-88 |
| ARF06222.1 | non-ribosomal\_peptide\_synthetase | BGC0001593 | NRP | 27.0 | 29.9 | 321.0 | 1.94e-88 |
| AAS90093.1 | PksA | BGC0000006 | Polyketide | 30.0 | 22.8 | 325.0 | 2.03e-88 |
| WP\_053065269.1 | non-ribosomal\_peptide\_synthetase | BGC0001330 | NRP:Cyclic depsipeptide+Polyketide:Modular type I polyketide | 28.0 | 29.5 | 325.0 | 2.12e-88 |
| AAG02357.1 | polyketide\_synthase | BGC0000963 | NRP:Glycopeptide+Polyketide:Modular type I polyketide+Saccharide:Hybrid/tailoring saccharide | 31.0 | 22.5 | 324.0 | 2.16e-88 |
| ABL74937.1 | NRPS | BGC0001048 | NRP:Glycopeptide+Polyketide:Modular type I polyketide+Saccharide:Hybrid/tailoring saccharide | 28.0 | 30.7 | 316.0 | 2.69e-88 |
| BBA20967.1 | nonribosomal\_peptide\_synthetase | BGC0001763 | NRP+Polyketide | 27.0 | 32.8 | 326.0 | 2.71e-88 |
| QPB41097.1 | non-ribosomal\_peptide\_synthetase | BGC0002503 | NRP+Polyketide | 28.0 | 29.8 | 325.0 | 2.91e-88 |
| PKX88487.1 | polyketide\_synthase | BGC0001708 | Polyketide+Terpene | 31.0 | 22.8 | 324.0 | 4.1e-88 |
| AAS90022.1 | PksA | BGC0000008 | Polyketide | 30.0 | 22.8 | 323.0 | 4.59e-88 |
| EAL84397.1 | polyketide\_synthase | BGC0001118 | Polyketide:Iterative type I polyketide | 29.0 | 24.8 | 322.0 | 5.4e-88 |
| AJW65406.1 | nonribosomal\_peptide\_synthetase | BGC0001195 | NRP+Polyketide | 26.0 | 30.0 | 321.0 | 6.13e-88 |
| AAS47562.1 | mixed\_type\_I\_polyketide\_synthase\_-\_peptide\_synthetase | BGC0001108 | NRP+Polyketide:Trans-AT type I polyketide | 28.0 | 29.9 | 324.0 | 7.41e-88 |
| WP\_018540603.1 | non-ribosomal\_peptide\_synthetase | BGC0001332 | NRP+Polyketide | 28.0 | 29.5 | 315.0 | 8.53e-88 |
| BAE71314.1 | polyketide\_synthase | BGC0000004 | Polyketide | 30.0 | 22.8 | 322.0 | 9e-88 |
| AXF14775.1 | non-ribosomal\_peptide\_synthetase | BGC0002563 | NRP | 27.0 | 30.7 | 322.0 | 9.93e-88 |
| CAD70195.1 | non-ribosomal\_peptide\_synthetase | BGC0001047 | NRP+Polyketide | 26.0 | 31.6 | 323.0 | 1.06e-87 |
| KON97028.1 | phenylalanine\_racemase | BGC0002122 | NRP | 27.0 | 29.3 | 323.0 | 1.1e-87 |
| AAS89999.1 | PksA | BGC0000007 | Polyketide | 30.0 | 22.7 | 322.0 | 1.36e-87 |
| AAK89720.1 | non-ribosomal\_peptide\_synthetase,\_siderophore\_biosynthesis\_protein | BGC0002107 | NRP+Polyketide | 26.0 | 29.7 | 322.0 | 1.36e-87 |
| DAC80541.1 | NRPS/PKS | BGC0001840 | NRP+Polyketide | 26.0 | 30.4 | 318.0 | 1.37e-87 |
| QUS58938.1 | amino\_acid\_adenylation\_domain-containing\_protein | BGC0002123 | NRP+Polyketide | 26.0 | 30.0 | 323.0 | 1.6e-87 |
| ASX95241.1 | IlaS | BGC0001620 | NRP+Polyketide | 27.0 | 29.9 | 323.0 | 1.77e-87 |
| AKJ15829.1 | peptide\_synthetase | BGC0002735 | Polyketide+NRP | 27.0 | 29.5 | 316.0 | 1.85e-87 |
| APO47822.1 | non-ribosomal\_peptide\_synthetase | BGC0002653 | NRP | 25.0 | 31.8 | 323.0 | 2.17e-87 |
| CAL17540.1 | peptide\_synthetase,\_putative | BGC0002465 | NRP | 27.0 | 29.3 | 320.0 | 2.43e-87 |
| ETS82099.1 | hypothetical\_protein | BGC0002161 | Polyketide | 28.0 | 24.8 | 321.0 | 2.46e-87 |
| AEA30272.1 | peptide\_synthetase | BGC0000429 | Polyketide+NRP:Cyclic depsipeptide | 26.0 | 30.0 | 322.0 | 2.54e-87 |
| ABA73956.1 | putative\_non-ribosomal\_peptide\_synthetase | BGC0001842 | NRP:Lipopeptide | 26.0 | 31.8 | 322.0 | 2.57e-87 |
| ALG65341.1 | Var6 | BGC0002416 | NRP+Polyketide | 27.0 | 29.5 | 322.0 | 2.63e-87 |
| NHN68325.1 | amino\_acid\_adenylation\_domain-containing\_protein | BGC0002719 | NRP | 26.0 | 33.6 | 322.0 | 2.78e-87 |
| ABS74206.1 | fengycin\_synthetase\_D | BGC0001095 | NRP | 25.0 | 31.7 | 322.0 | 2.82e-87 |
| AKA59437.1 | polyketide\_synthase | BGC0001202 | NRP+Polyketide | 31.0 | 22.4 | 305.0 | 3.8e-87 |
| PHM26614.1 | Phthiocerol\_synthesis\_polyketide\_synthase\_type\_I\_PpsE | BGC0001130 | NRP+Polyketide | 30.0 | 23.5 | 319.0 | 4.47e-87 |
| CBL93730.1 | NRPS | BGC0000360 | NRP | 28.0 | 30.4 | 314.0 | 4.74e-87 |
| BAH43765.1 | tyrocidine\_synthetase\_II | BGC0000452 | NRP | 26.0 | 29.3 | 321.0 | 4.82e-87 |
| ACS20362.1 | amino\_acid\_adenylation\_domain\_protein | BGC0002420 | NRP+Polyketide | 28.0 | 31.8 | 319.0 | 4.86e-87 |
| ABX37385.1 | amino\_acid\_adenylation\_domain\_protein | BGC0000984 | NRP+Polyketide | 26.0 | 31.8 | 319.0 | 5.04e-87 |
| AXG49819.1 | hybrid\_non-ribosomal\_peptide\_synthetase/type\_I\_polyketide\_synthase | BGC0000383 | NRP+Polyketide:Modular type I polyketide | 28.0 | 29.9 | 321.0 | 5.29e-87 |
| CAE52334.1 | non-ribosomal\_peptide\_synthase | BGC0001088 | NRP+Polyketide | 26.0 | 30.7 | 320.0 | 5.56e-87 |
| CAJ21198.2 | non-ribosomal\_peptide\_synthetase | BGC0000297 | NRP:Glycopeptide+Polyketide:Other polyketide+Saccharide:Hybrid/tailoring saccharide | 26.0 | 29.7 | 318.0 | 5.72e-87 |
| AXM42949.1 | hybrid\_type\_1\_PKS/NRPS | BGC0001941 | NRP+Polyketide | 28.0 | 29.6 | 321.0 | 5.78e-87 |
| ADH01663.1 | putative\_polyketide\_synthase\_PKS3 | BGC0000099 | Polyketide | 29.0 | 23.4 | 320.0 | 6.21e-87 |
| ABM34278.1 | amino\_acid\_adenylation\_domain\_protein | BGC0002419 | NRP+Polyketide | 26.0 | 31.4 | 312.0 | 6.74e-87 |
| CAQ34921.1 | nonribosomal\_peptide\_synthetase | BGC0000986 | NRP+Polyketide | 26.0 | 29.5 | 317.0 | 7e-87 |
| AAF00962.1 | mcyC | BGC0001017 | NRP+Polyketide:Modular type I polyketide | 25.0 | 29.5 | 315.0 | 7.88e-87 |
| BAH43766.1 | tyrocidine\_synthetase\_III | BGC0000452 | NRP | 25.0 | 32.1 | 321.0 | 8.37e-87 |
| ABD14712.1 | cesB | BGC0000320 | NRP:Cyclic depsipeptide | 23.0 | 41.3 | 320.0 | 1.06e-86 |
| AAU39360.1 | lichenysin\_synthase\_LchAB | BGC0000381 | NRP | 27.0 | 28.9 | 320.0 | 1.08e-86 |
| CAB38517.1 | CDA\_peptide\_synthetase\_II\_(CdaPs2) | BGC0000315 | NRP:Lipopeptide:Ca+-dependent lipopeptide | 27.0 | 32.4 | 320.0 | 1.1e-86 |
| gene6 |  | BGC0001906 | Polyketide | 29.0 | 22.7 | 319.0 | 1.33e-86 |
| QLY89264.1 | pseudodesmin\_synthetase | BGC0002522 | NRP | 26.0 | 31.7 | 320.0 | 1.48e-86 |
| AHH53508.1 | non-ribosomal\_peptide\_synthetase | BGC0000439 | NRP:Lipopeptide:Ca+-dependent lipopeptide | 27.0 | 33.4 | 319.0 | 1.53e-86 |
| AAD38786.1 | polyketide\_synthase | BGC0001257 | Polyketide | 29.0 | 22.4 | 318.0 | 1.65e-86 |
| AIW58892.1 | non-ribosomal\_peptide\_synthetase | BGC0001582 | NRP | 28.0 | 32.3 | 320.0 | 1.68e-86 |
| ALK27914.1 | non-ribosomal\_peptide\_synthase | BGC0001233 | NRP | 28.0 | 33.1 | 320.0 | 1.76e-86 |
| ABM34279.1 | beta-ketoacyl\_synthase | BGC0002419 | NRP+Polyketide | 31.0 | 23.0 | 316.0 | 2.19e-86 |
| AFH75330.1 | nonribosomal\_peptide\_synthetase | BGC0000398 | NRP:Cyclic depsipeptide | 26.0 | 31.8 | 319.0 | 2.89e-86 |
| ALK27915.1 | non-ribosomal\_peptide\_synthase | BGC0001233 | NRP | 28.0 | 31.9 | 319.0 | 3.38e-86 |
| BGRAMDRAFT\_RS22640 | amino\_acid\_adenylation\_domain-containing\_protein | BGC0001999 | NRP | 26.0 | 32.2 | 312.0 | 3.41e-86 |
| ADF88262.1 | mixed\_nonribosomal\_peptide\_synthetase/\_polyketide\_synthase | BGC0000979 | NRP+Polyketide | 31.0 | 20.2 | 314.0 | 3.49e-86 |
| ADF88265.1 | mixed\_nonribosomal\_peptide\_synthetase/\_polyketide\_synthase | BGC0000980 | NRP+Polyketide | 31.0 | 20.2 | 314.0 | 3.49e-86 |
| AJD47481.1 | amino\_acid\_adenylation\_domain-containing\_protein | BGC0002418 | NRP+Polyketide | 26.0 | 29.7 | 309.0 | 3.5e-86 |
| BAO84866.1 | putative\_non-ribosomal\_peptide\_synthetase | BGC0000414 | NRP | 25.0 | 38.2 | 315.0 | 3.8e-86 |
| AKP45399.1 | CysK | BGC0001413 | NRP | 26.0 | 34.1 | 318.0 | 4.77e-86 |
| BCD52390.1 | polyketide\_synthase\_SptM | BGC0002537 | Polyketide+Terpene | 32.0 | 20.7 | 317.0 | 6.09e-86 |
| AJD47485.1 | PpsD | BGC0002418 | NRP+Polyketide | 27.0 | 29.7 | 317.0 | 6.67e-86 |
| CDG17980.1 | Putative\_Ornithine\_racemase\_(fragment) | BGC0000464 | NRP:Cyclic depsipeptide | 27.0 | 32.0 | 318.0 | 7.48e-86 |
| NKI69296.1 | amino\_acid\_adenylation\_domain-containing\_protein | BGC0002408 | NRP | 27.0 | 30.5 | 315.0 | 7.64e-86 |
| ABS74207.1 | fengycin\_synthetase\_C | BGC0001095 | NRP | 26.0 | 31.5 | 317.0 | 8.58e-86 |
| AAS90047.1 | PksA | BGC0000009 | Polyketide | 30.0 | 22.7 | 316.0 | 1.03e-85 |
| BAP81867.1 | AndM | BGC0002612 | Terpene | 32.0 | 20.7 | 316.0 | 1.05e-85 |
| BAH43871.1 | truncated\_linear\_pentadecapeptide\_gramicidin\_synthetase\_LgrC | BGC0000367 | NRP | 26.0 | 28.4 | 317.0 | 1.22e-85 |
| CDM36726.1 | Beta-ketoacyl\_synthase | BGC0001360 | Polyketide | 29.0 | 22.7 | 316.0 | 1.41e-85 |
| CAJ76298.1 | putative\_hybrid\_polyketide-non-ribosomal\_peptide\_synthetase | BGC0000972 | NRP+Polyketide:Modular type I polyketide+Polyketide:Trans-AT type I polyketide | 30.0 | 24.6 | 316.0 | 1.45e-85 |
| AEH41793.1 | HrmO | BGC0000374 | NRP:Cyclic depsipeptide | 28.0 | 30.1 | 317.0 | 1.49e-85 |
| AAY93445.1 | non-ribosomal\_peptide\_synthetase\_PvdL | BGC0000413 | NRP | 27.0 | 29.3 | 316.0 | 1.78e-85 |
| AAF15891.2 | nosA | BGC0001028 | Polyketide+NRP:Cyclic depsipeptide | 26.0 | 30.2 | 316.0 | 1.79e-85 |
| ACC81023.1 | non-ribosomal\_peptide\_synthetase | BGC0001479 | NRP | 28.0 | 25.6 | 315.0 | 1.96e-85 |
| CDG17981.1 | Non-ribosomal\_peptide\_synthetase | BGC0000464 | NRP:Cyclic depsipeptide | 27.0 | 30.2 | 315.0 | 2.04e-85 |
| AHD05621.1 | non-ribosomal\_peptide\_ligase\_domain\_protein | BGC0001033 | NRP+Polyketide | 25.0 | 30.0 | 315.0 | 2.12e-85 |
| AHZ34243.1 | CipF | BGC0001389 | NRP | 26.0 | 32.0 | 316.0 | 2.15e-85 |
| ADY00130.1 | polyketide\_synthase | BGC0000104 | Terpene+Polyketide:Iterative type I polyketide | 28.0 | 23.9 | 315.0 | 2.38e-85 |
| QEO74904.1 | AMP-dependent\_synthetase\_and\_ligase | BGC0002588 | Other | 26.0 | 31.5 | 315.0 | 2.41e-85 |
| QGA70148.1 | nonribosomal\_peptide\_synthetase | BGC0002293 | NRP | 28.0 | 28.8 | 316.0 | 2.43e-85 |
| ERF77221.1 | hypothetical\_protein | BGC0002215 | Polyketide | 30.0 | 22.9 | 315.0 | 2.48e-85 |
| QED88055.1 | nonribosomal\_peptide\_synthetase | BGC0001967 | NRP+Polyketide | 26.0 | 33.1 | 315.0 | 2.97e-85 |
| EHA28237.1 | hypothetical\_protein | BGC0001143 | Polyketide | 29.0 | 22.9 | 315.0 | 3.39e-85 |
| BAE61567.1 |  | BGC0002175 | Polyketide | 30.0 | 25.1 | 314.0 | 4.16e-85 |
| AQX14499.1 | monobactam\_NRPS\_scaffold\_1 | BGC0001672 | NRP | 27.0 | 29.6 | 312.0 | 4.24e-85 |
| AAZ03550.1 | McnA | BGC0000332 | NRP | 26.0 | 29.9 | 306.0 | 4.59e-85 |
| QUJ09167.1 | Lon20 | BGC0002440 | NRP | 27.0 | 30.5 | 315.0 | 5.11e-85 |
| QIE08737.1 | non-ribosomal\_peptide\_synthetase | BGC0002544 | NRP | 27.0 | 32.4 | 314.0 | 5.14e-85 |
| QBA57737.1 | NRPS | BGC0002377 | NRP | 27.0 | 33.8 | 314.0 | 5.16e-85 |
| EHA55627.1 | conidial\_yellow\_pigment\_biosynthesis\_polyketide\_synthase | BGC0002154 | Polyketide | 28.0 | 22.7 | 313.0 | 5.68e-85 |
| AAZ95017.1 | polyketide\_synthase | BGC0000048 | Polyketide | 30.0 | 23.3 | 313.0 | 6.77e-85 |
| APZ78679.1 | nonribosomal\_peptide\_synthetase | BGC0001417 | NRP:Cyclic depsipeptide+Polyketide:Iterative type I polyketide | 27.0 | 31.4 | 314.0 | 6.96e-85 |
| AAZ23076.1 | peptide\_synthetase | BGC0000291 | NRP | 27.0 | 30.2 | 313.0 | 7.16e-85 |
| QYA95682.1 | amino\_acid\_adenylation\_domain-containing\_protein | BGC0002676 | NRP | 26.0 | 29.9 | 314.0 | 9.33e-85 |
| WP\_019032755.1 | non-ribosomal\_peptide\_synthetase | BGC0001331 | NRP:Cyclic depsipeptide+Polyketide:Modular type I polyketide | 27.0 | 29.5 | 313.0 | 9.39e-85 |
| ATY37589.1 | BogB | BGC0001532 | NRP | 26.0 | 31.2 | 313.0 | 9.61e-85 |
| ABD65958.1 | nonribosomal\_peptide\_synthetase | BGC0000341 | NRP | 29.0 | 30.2 | 314.0 | 1.13e-84 |
| QNH67551.1 | Cip23 | BGC0002108 | NRP | 27.0 | 32.9 | 313.0 | 1.6e-84 |
| AAO56104.1 | yersiniabactin\_polyketide/non-ribosomal\_peptide\_synthetase | BGC0002570 | NRP+Polyketide | 30.0 | 22.5 | 313.0 | 1.62e-84 |
| CAE02631.1 | surfactin\_synthetase\_B\_ | BGC0000433 | NRP:Lipopeptide | 27.0 | 29.2 | 313.0 | 1.78e-84 |
| QST87270.1 | amino\_acid\_adenylation\_domain-containing\_protein | BGC0002572 | NRP+Polyketide | 28.0 | 24.6 | 313.0 | 1.88e-84 |
| EPH46598.1 | putative\_Dimodular\_nonribosomal\_peptide\_synthase | BGC0001519 | NRP+Polyketide | 27.0 | 29.7 | 307.0 | 2.03e-84 |
| AXM43052.1 | non-ribosomal\_peptide\_synthetase | BGC0001945 | NRP | 28.0 | 29.3 | 312.0 | 2.26e-84 |
| MCF2150416.1 | Non-ribosomal\_peptide\_synthetase | BGC0002625 | NRP+Polyketide | 26.0 | 30.0 | 313.0 | 2.33e-84 |
| QBA57736.1 | NRPS | BGC0002377 | NRP | 26.0 | 32.6 | 311.0 | 2.91e-84 |
| FIS9431\_RS32925 | non-ribosomal\_peptide\_synthetase | BGC0001467 | NRP:Cyclic depsipeptide+Polyketide:Modular type I polyketide | 28.0 | 24.4 | 308.0 | 3.46e-84 |
| ALG65318.1 | Cal18 | BGC0001297 | NRP | 27.0 | 31.8 | 311.0 | 3.79e-84 |
| AAC82550.1 | FxbC | BGC0000351 | NRP | 27.0 | 31.9 | 311.0 | 4.77e-84 |
| AAN85493.1 | nonribosomal\_peptide\_synthetase | BGC0001101 | NRP+Polyketide:Modular type I polyketide+Polyketide:Trans-AT type I polyketide | 27.0 | 29.7 | 303.0 | 5.18e-84 |
| APZ78781.1 | nonribosomal\_peptide\_synthetase | BGC0001426 | NRP:Cyclic depsipeptide+Polyketide:Iterative type I polyketide | 27.0 | 31.3 | 311.0 | 5.99e-84 |
| AEW31022.1 | plipastatin\_synthetase | BGC0000407 | NRP | 25.0 | 31.5 | 311.0 | 6.87e-84 |
| AJK49766.1 | non-ribosomal\_peptide\_synthase | BGC0002565 | NRP | 28.0 | 29.6 | 311.0 | 7e-84 |
| KIA75596.1 | polyketide\_synthase | BGC0002209 | Polyketide | 31.0 | 20.2 | 310.0 | 7.26e-84 |
| AAC44129.1 | saframycin\_Mx1\_synthetase\_A | BGC0002706 | NRP | 25.0 | 38.2 | 310.0 | 8.64e-84 |
| ESU07748.1 | hypothetical\_protein | BGC0002709 | Polyketide | 30.0 | 25.4 | 309.0 | 9.87e-84 |
| ABM34280.1 | amino\_acid\_adenylation\_domain\_protein | BGC0002419 | NRP+Polyketide | 27.0 | 29.6 | 308.0 | 1.38e-83 |
| AID65224.1 | nonribosomal\_peptide\_synthetase | BGC0000335 | NRP+Polyketide | 26.0 | 32.0 | 309.0 | 1.44e-83 |
| ABL74939.1 | NRPS | BGC0001048 | NRP:Glycopeptide+Polyketide:Modular type I polyketide+Saccharide:Hybrid/tailoring saccharide | 27.0 | 31.0 | 302.0 | 1.47e-83 |
| QWT72292.1 | putative\_non-ribosomal\_peptide\_synthetase | BGC0002430 | NRP+Saccharide | 27.0 | 29.8 | 310.0 | 1.48e-83 |
| AJV88375.1 | MfnC | BGC0001214 | NRP | 26.0 | 33.8 | 310.0 | 1.55e-83 |
| BAH22764.1 | nonribosomal\_peptide\_synthetase | BGC0001018 | NRP | 28.0 | 24.5 | 310.0 | 1.78e-83 |
| AKJ29410.1 | peptide\_synthetase | BGC0001608 | NRP | 26.0 | 32.1 | 309.0 | 1.85e-83 |
| CAJ14037.1 | peptide\_synthetase | BGC0000406 | NRP | 28.0 | 32.2 | 303.0 | 1.94e-83 |
| ATD51279.1 | nonribosomal\_peptide\_synthase | BGC0001650 | NRP | 27.0 | 31.6 | 307.0 | 2.04e-83 |
| BAH43869.1 | linear\_pentadecapeptide\_gramicidin\_synthetase\_LgrA | BGC0000367 | NRP | 26.0 | 29.3 | 308.0 | 2.07e-83 |
| ABD65956.1 | nonribosomal\_peptide\_synthetase | BGC0000341 | NRP | 27.0 | 33.4 | 308.0 | 2.31e-83 |
| CAQ71828.1 | non\_ribosomal\_peptide\_synthase,\_antibiotic\_synthesis;\_contains\_3\_condensation\_domains,\_2\_AMP-acid\_ligases\_II\_domains,\_2\_PP-binding,\_Phosphopantetheine\_attachment\_site | BGC0001189 | NRP | 28.0 | 30.2 | 308.0 | 2.53e-83 |
| ADA69239.2 | trans-AT\_hybrid\_polyketide\_synthase-NRPS | BGC0001071 | NRP+Polyketide:Modular type I polyketide+Polyketide:Trans-AT type I polyketide | 25.0 | 32.0 | 309.0 | 2.78e-83 |
| ABC34108.1 | JamP | BGC0000961 | NRP+Polyketide | 31.0 | 22.4 | 306.0 | 2.89e-83 |
| CAA73127.1 | HMWP1\_protein | BGC0000467 | NRP | 30.0 | 22.3 | 308.0 | 3.11e-83 |
| CAJ87591.1 | putative\_peptide/polyketide\_synthetase | BGC0001055 | NRP+Polyketide | 30.0 | 22.4 | 308.0 | 3.11e-83 |
| QIE07364.1 | polyketide\_synthase\_NecE | BGC0002050 | NRP+Polyketide:Trans-AT type I polyketide | 24.0 | 43.2 | 308.0 | 3.66e-83 |
| QED55422.1 | nonribosomal\_peptide\_synthetase | BGC0001984 | NRP | 28.0 | 33.4 | 308.0 | 3.77e-83 |
| WP\_082191961.1 | non-ribosomal\_peptide\_synthetase | BGC0001451 | NRP | 28.0 | 30.0 | 308.0 | 3.9e-83 |
| ACS20358.1 | amino\_acid\_adenylation\_domain\_protein | BGC0002420 | NRP+Polyketide | 28.0 | 29.5 | 304.0 | 4.1e-83 |
| AAZ23077.1 | peptide\_synthetase | BGC0000291 | NRP | 27.0 | 32.8 | 308.0 | 4.17e-83 |
| QEO74982.1 | omn7 | BGC0002078 | NRP:Cyclic depsipeptide | 26.0 | 32.3 | 308.0 | 4.49e-83 |
| ACO78745.1 | Non-ribosomal\_peptide\_synthase:Amino\_acid\_adenylation | BGC0002433 | NRP | 26.0 | 34.1 | 308.0 | 5e-83 |
| WP\_012408783.1 | non-ribosomal\_peptide\_synthetase | BGC0002061 | NRP:Cyclic depsipeptide+Polyketide:Modular type I polyketide | 25.0 | 30.2 | 308.0 | 5.04e-83 |
| ATY37608.1 | BreC | BGC0001536 | NRP | 26.0 | 30.9 | 308.0 | 5.08e-83 |
| ALK27916.1 | non-ribosomal\_peptide\_synthase | BGC0001233 | NRP | 27.0 | 32.6 | 308.0 | 5.65e-83 |
| AKJ15827.1 | peptide\_synthetase | BGC0002735 | Polyketide+NRP | 27.0 | 29.6 | 307.0 | 5.65e-83 |
| AHI59108.1 | locillomycin\_synthase\_A | BGC0001005 | NRP+Polyketide | 24.0 | 29.6 | 307.0 | 6.33e-83 |
| AGI89788.1 | Nonribosomal\_peptide\_synthetase | BGC0001792 | NRP | 28.0 | 29.8 | 308.0 | 6.37e-83 |
| WP\_013310342.1 | non-ribosomal\_peptide\_synthetase | BGC0001728 | NRP+Polyketide | 25.0 | 32.3 | 307.0 | 6.56e-83 |
| AAK89719.2 | peptide\_synthetase,\_siderophore\_biosynthesis\_protein | BGC0002107 | NRP+Polyketide | 26.0 | 29.2 | 307.0 | 7.31e-83 |
| UHJ79948.1 | non-ribosomal\_peptide\_synthetase | BGC0002654 | NRP | 27.0 | 31.7 | 306.0 | 7.63e-83 |
| QUS58937.1 | non-ribosomal\_peptide\_synthetase | BGC0002123 | NRP+Polyketide | 24.0 | 29.9 | 303.0 | 8.02e-83 |
| QBQ12463.1 | amino\_acid\_adenylation\_domain-containing\_protein | BGC0002693 | NRP | 26.0 | 32.1 | 307.0 | 8.33e-83 |
| CAL80821.1 | sylD-like\_NRPS/PKS | BGC0000997 | NRP+Polyketide | 26.0 | 30.3 | 307.0 | 8.41e-83 |
| APZ78715.1 | nonribosomal\_peptide\_synthetase | BGC0001420 | NRP:Cyclic depsipeptide+Polyketide:Iterative type I polyketide | 26.0 | 31.6 | 307.0 | 8.82e-83 |
| AAZ03552.1 | McnC | BGC0000332 | NRP | 28.0 | 24.4 | 307.0 | 8.92e-83 |
| AGN71604.1 | conidial\_yellow\_pigment\_biosynthesis\_polyketide\_synthase | BGC0000027 | Polyketide:Iterative type I polyketide | 29.0 | 22.7 | 306.0 | 1.02e-82 |
| AGP37410.1 | peptide\_synthetase | BGC0002386 | NRP+Polyketide | 27.0 | 27.1 | 307.0 | 1.03e-82 |
| APZ78768.1 | nonribosomal\_peptide\_synthetase | BGC0001425 | NRP:Cyclic depsipeptide+Polyketide:Iterative type I polyketide | 27.0 | 31.3 | 306.0 | 1.16e-82 |
| KDB16994.1 | polyketide\_synthetase\_PksP | BGC0002177 | Polyketide | 30.0 | 22.6 | 306.0 | 1.2e-82 |
| ABC37099.1 | non-ribosomal\_peptide\_synthetase,\_putative | BGC0000386 | NRP:NRP siderophore | 26.0 | 32.4 | 305.0 | 1.2e-82 |
| AAZ55900.1 | non-ribosomal\_peptide\_synthase:Amino\_acid\_adenylation | BGC0000359 | NRP | 27.0 | 33.2 | 306.0 | 1.32e-82 |
| ARU08075.1 | mlcM | BGC0001448 | NRP:Lipopeptide:Ca+-dependent lipopeptide | 26.0 | 32.6 | 306.0 | 1.38e-82 |
| CAJ96470.1 | non-ribosomal\_peptide\_synthetase | BGC0000330 | NRP:NRP siderophore | 27.0 | 29.4 | 305.0 | 1.38e-82 |
| APZ78703.1 | nonribosomal\_peptide\_synthetase | BGC0001419 | NRP:Cyclic depsipeptide+Polyketide:Iterative type I polyketide | 26.0 | 31.6 | 306.0 | 1.51e-82 |
| WP\_078586793.1 | non-ribosomal\_peptide\_synthetase | BGC0001760 | NRP | 26.0 | 29.4 | 302.0 | 1.65e-82 |
| AGE11898.1 | nonribosomal\_peptide\_synthetase | BGC0000366 | NRP | 27.0 | 29.0 | 306.0 | 1.66e-82 |
| QDQ83033.1 | amino\_acid\_adenylation\_domain-containing\_protein | BGC0002564 | NRP | 27.0 | 29.3 | 305.0 | 1.86e-82 |
| EFL06865.1 | hypothetical\_protein | BGC0000300 | NRP | 27.0 | 29.0 | 305.0 | 1.91e-82 |
| WP\_019634550.1 | type\_I\_polyketide\_synthase | BGC0001443 | NRP+Polyketide | 31.0 | 22.4 | 303.0 | 2.27e-82 |
| EDY47118.1 | N-(5-amino-5-carboxypentanoyl)-L-cysteinyl-D-\_valine\_synthase | BGC0000319 | NRP:Beta-lactam | 25.0 | 32.4 | 306.0 | 2.31e-82 |
| AIG26883.1 | NRPS\_domain-containing\_protein | BGC0002432 | NRP | 23.0 | 31.5 | 306.0 | 2.47e-82 |
| QKG86295.1 | non-reducing\_polyketide\_synthase | BGC0002253 | Polyketide | 28.0 | 24.9 | 305.0 | 2.57e-82 |
| ATW47208.1 | non-ribosomal\_peptide\_synthetase | BGC0002466 | NRP | 26.0 | 32.7 | 306.0 | 2.71e-82 |
| AHB38509.1 | polyketide\_synthase | BGC0000345 | NRP+Polyketide:Modular type I polyketide | 32.0 | 23.5 | 304.0 | 3.16e-82 |
| EWS95124.1 | hypothetical\_protein | BGC0000306 | NRP:Lipopeptide | 27.0 | 29.3 | 305.0 | 3.27e-82 |
| QPB41098.1 | non-ribosomal\_peptide\_synthetase | BGC0002503 | NRP+Polyketide | 26.0 | 29.9 | 301.0 | 3.53e-82 |
| CAJ34382.1 | NRPS\_protein | BGC0000445 | NRP:Cyclic depsipeptide | 27.0 | 31.0 | 301.0 | 3.56e-82 |
| AEW31021.1 | plipastatin\_synthetase | BGC0000407 | NRP | 25.0 | 31.4 | 305.0 | 3.67e-82 |
| QEO75075.1 | condensation\_domain-containing\_protein | BGC0002079 | NRP:Cyclic depsipeptide | 26.0 | 32.5 | 305.0 | 4.75e-82 |
| BCP96883.1 | non-reducing\_polyketide\_synthase | BGC0002614 | NRP+Polyketide | 28.0 | 22.2 | 304.0 | 4.95e-82 |
| DAC80524.1 | peptide\_synthetase | BGC0001841 | NRP+Polyketide | 26.0 | 30.1 | 299.0 | 5.34e-82 |
| ABW71853.1 | nonribosomal\_peptide\_synthetase | BGC0000303 | NRP | 26.0 | 37.4 | 301.0 | 5.36e-82 |
| AOZ21320.1 | SulM | BGC0001790 | NRP | 27.0 | 32.4 | 304.0 | 5.69e-82 |
| APZ78808.1 | nonribosomal\_peptide\_synthetase | BGC0001428 | NRP:Cyclic depsipeptide+Polyketide:Iterative type I polyketide | 26.0 | 31.3 | 304.0 | 5.8e-82 |
| ALG65317.1 | Cal19 | BGC0001297 | NRP | 27.0 | 33.5 | 303.0 | 5.96e-82 |
| QNT61260.1 | polyketide\_synthase | BGC0002507 | Polyketide | 29.0 | 22.8 | 303.0 | 6.06e-82 |
| ARU08074.1 | mlcL | BGC0001448 | NRP:Lipopeptide:Ca+-dependent lipopeptide | 27.0 | 31.1 | 305.0 | 6.26e-82 |
| WP\_012408785.1 | non-ribosomal\_peptide\_synthetase | BGC0002061 | NRP:Cyclic depsipeptide+Polyketide:Modular type I polyketide | 26.0 | 29.9 | 304.0 | 6.92e-82 |
| MBN3579113.1 | amino\_acid\_adenylation\_domain-containing\_protein | BGC0002613 | NRP+Polyketide | 29.0 | 22.4 | 304.0 | 7.03e-82 |
| ACZ55944.1 | non-ribosomal\_peptide\_synthetase | BGC0000302 | NRP | 27.0 | 26.5 | 303.0 | 8.27e-82 |
| QCE43603.1 | nonribosomal\_peptide\_synthetase\_(NRPS),\_subunit\_2 | BGC0001834 | NRP | 25.0 | 32.5 | 304.0 | 8.33e-82 |
| AAS98786.1 | nonribosomal\_peptide\_synthetase | BGC0001001 | NRP+Polyketide | 26.0 | 30.0 | 297.0 | 1.03e-81 |
| CBA63680.1 | nonribosomal\_peptide\_synthetase\_NRPS | BGC0000368 | NRP | 27.0 | 32.3 | 303.0 | 1.03e-81 |
| QUJ09168.1 | Lon21 | BGC0002440 | NRP | 27.0 | 29.4 | 301.0 | 1.1e-81 |
| CAE02630.1 | surfactin\_synthetase\_A | BGC0000433 | NRP:Lipopeptide | 26.0 | 29.3 | 303.0 | 1.13e-81 |
| CUX96955.1 | TmcH | BGC0001829 | NRP+Polyketide | 31.0 | 23.9 | 302.0 | 1.27e-81 |
| NPC94426.1 | amino\_acid\_adenylation\_domain-containing\_protein | BGC0002695 | NRP | 25.0 | 29.9 | 303.0 | 1.28e-81 |
| APZ78691.1 | nonribosomal\_peptide\_synthetase | BGC0001418 | NRP:Cyclic depsipeptide+Polyketide:Iterative type I polyketide | 25.0 | 31.6 | 303.0 | 1.3e-81 |
| QCQ67880.1 | non-ribosomal\_peptide\_synthetase | BGC0002297 | NRP+Polyketide | 26.0 | 29.8 | 302.0 | 1.38e-81 |
| AHH53507.1 | non-ribosomal\_peptide\_synthetase | BGC0000439 | NRP:Lipopeptide:Ca+-dependent lipopeptide | 27.0 | 32.6 | 303.0 | 1.51e-81 |
| AXG47411.1 | hybrid\_non-ribosomal\_peptide\_synthetase/type\_I\_polyketide\_synthase | BGC0002715 | NRP+Polyketide | 30.0 | 22.5 | 303.0 | 1.55e-81 |
| AGZ15459.1 | putative\_non-ribosomal\_peptide\_synthetase | BGC0001036 | NRP+Polyketide | 27.0 | 29.7 | 303.0 | 1.57e-81 |
| AHF21228.1 | TriD | BGC0000449 | NRP | 26.0 | 32.2 | 303.0 | 1.69e-81 |
| CCJ67636.1 | TaaA | BGC0000447 | NRP:Lipopeptide | 25.0 | 29.8 | 303.0 | 1.78e-81 |
| AUW31052.1 | putative\_type\_I\_PKS | BGC0002483 | Polyketide | 31.0 | 20.4 | 302.0 | 1.84e-81 |
| ABW00331.1 | amino\_acid\_adenylation\_domain | BGC0000333 | NRP | 29.0 | 26.1 | 303.0 | 2e-81 |
| CZT62785.1 | Non-ribosomal\_peptide\_synthase,\_involved\_in\_Hassallidin\_biosynthesis | BGC0001614 | NRP | 26.0 | 29.3 | 302.0 | 2.21e-81 |
| QDK64760.1 | AshP | BGC0002301 | Polyketide | 28.0 | 25.3 | 301.0 | 2.32e-81 |
| antaD | Type\_I\_PKS | BGC0001455 | NRP+Polyketide | 30.0 | 22.9 | 297.0 | 2.46e-81 |
| AAU34203.1 | mannopeptimycin\_peptide\_synthetase\_MppB | BGC0000388 | NRP | 27.0 | 29.6 | 302.0 | 2.56e-81 |
| ANS62968.1 | actinomycin\_synthetase\_II | BGC0001567 | NRP | 27.0 | 33.1 | 301.0 | 3.16e-81 |
| EPH46597.1 | putative\_Linear\_gramicidin\_synthase\_subunit\_C | BGC0001519 | NRP+Polyketide | 26.0 | 29.9 | 301.0 | 4.19e-81 |
| AJY78094.1 | nonribosomal\_peptide\_synthetase | BGC0001902 | NRP+Polyketide | 26.0 | 29.9 | 297.0 | 4.73e-81 |
| BAE65965.1 |  | BGC0002236 | Polyketide | 28.0 | 24.9 | 300.0 | 5.29e-81 |
| AKQ52532.1 | nonribosomal\_peptide\_synthetase | BGC0002533 | NRP+Polyketide | 29.0 | 22.5 | 301.0 | 6.03e-81 |
| CCE33500.1 | polyketide\_synthase\_that\_catalyse\_the\_condensation\_of\_one\_acetyl-CoA\_and\_six\_malonyl-CoA\_resulting\_in\_formation\_of\_nor-rubrofusarin | BGC0002596 | Polyketide | 27.0 | 25.8 | 300.0 | 7.6e-81 |
| AAX31558.1 | peptide\_synthetase\_2 | BGC0000336 | NRP | 27.0 | 29.7 | 301.0 | 9.87e-81 |
| AIG26884.1 | NRPS\_domain-containing\_protein | BGC0002432 | NRP | 26.0 | 28.9 | 300.0 | 1.41e-80 |
| CAM56771.1 |  | BGC0000354 | NRP | 27.0 | 29.9 | 299.0 | 1.44e-80 |
| ATY37591.1 | BogD | BGC0001532 | NRP | 26.0 | 30.6 | 300.0 | 1.45e-80 |
| BAH04161.1 | putative\_non-ribosomal\_peptide\_synthetase | BGC0000450 | NRP | 28.0 | 25.4 | 300.0 | 1.51e-80 |
| CAR51995.1 | ornibactin\_biosynthesis\_non-ribosomal\_peptide\_synthase | BGC0002569 | NRP | 26.0 | 29.5 | 298.0 | 1.67e-80 |
| KFL51887.1 | amino\_acid\_adenylation\_protein | BGC0001711 | NRP+Polyketide | 26.0 | 33.8 | 299.0 | 1.98e-80 |
| AXG46163.1 | non-ribosomal\_peptide\_synthetase | BGC0002713 | NRP | 26.0 | 30.0 | 293.0 | 2.05e-80 |
| QBK15044.1 | clavatol\_synthase\_ClaF | BGC0002196 | Polyketide | 28.0 | 25.2 | 299.0 | 2.12e-80 |
| WP\_039806850.1 | non-ribosomal\_peptide\_synthetase | BGC0002001 | NRP+Polyketide | 27.0 | 29.8 | 298.0 | 2.8e-80 |
| DAC76734.1 | type\_I\_polyketide\_synthase/non-ribosomal\_peptide\_synthetase | BGC0001885 | NRP+Polyketide | 27.0 | 32.7 | 299.0 | 2.97e-80 |
| WP\_068925909.1 | non-ribosomal\_peptide\_synthetase | BGC0002688 | NRP | 26.0 | 31.8 | 298.0 | 3.26e-80 |
| AJG44381.1 | MpaC' | BGC0002619 | Polyketide | 29.0 | 22.2 | 298.0 | 3.73e-80 |
| ACG60773.1 | NRPS(C/A/PCP) | BGC0001058 | NRP:Glycopeptide+Polyketide:Modular type I polyketide+Saccharide:Hybrid/tailoring saccharide | 25.0 | 30.1 | 291.0 | 4.16e-80 |
| QCP68976.1 | VatQ | BGC0002296 | NRP+Polyketide | 26.0 | 28.9 | 296.0 | 4.98e-80 |
| AUD11993.1 | OrbJ | BGC0001721 | NRP | 27.0 | 30.5 | 295.0 | 9e-80 |
| ABS75232.1 | DhbF | BGC0001185 | NRP:NRP siderophore | 27.0 | 29.9 | 296.0 | 9.55e-80 |
| WP\_245566645.1 | amino\_acid\_adenylation\_domain-containing\_protein | BGC0002467 | NRP | 26.0 | 32.2 | 297.0 | 1.01e-79 |
| WA1\_15570 | hypothetical\_protein | BGC0002484 | NRP+Polyketide | 28.0 | 25.4 | 297.0 | 1.06e-79 |
| AGU50950.1 | putative\_non-ribosomal\_peptide\_synthetase | BGC0002417 | NRP+Polyketide | 27.0 | 29.6 | 296.0 | 1.09e-79 |
| AJK49765.1 | non-ribosomal\_peptide\_synthase | BGC0002565 | NRP | 27.0 | 32.5 | 295.0 | 1.4e-79 |
| QED55421.1 | nonribosomal\_peptide\_synthetase | BGC0001984 | NRP | 27.0 | 27.2 | 296.0 | 1.49e-79 |
| ATL73036.1 | amino\_acid\_adenylation\_protein | BGC0001807 | NRP+Polyketide | 27.0 | 30.3 | 296.0 | 1.81e-79 |
| QRG35014.1 | NRPS | BGC0002378 | NRP | 26.0 | 32.1 | 296.0 | 2.19e-79 |
| AEC14348.1 | nonribosomal\_peptide\_synthetase | BGC0000377 | NRP | 24.0 | 31.4 | 295.0 | 2.38e-79 |
| ACS20359.1 | amino\_acid\_adenylation\_domain\_protein | BGC0002420 | NRP+Polyketide | 26.0 | 29.6 | 295.0 | 2.44e-79 |
| CEK23364.1 | putative\_Phenylalanine\_racemase\_(ATP-hydrolyzing) | BGC0001716 | NRP | 25.0 | 29.7 | 296.0 | 2.45e-79 |
| QBG38783.1 | Atr22 | BGC0001975 | NRP | 27.0 | 33.1 | 296.0 | 2.47e-79 |
| ATU31795.1 | NRPS | BGC0001814 | NRP | 28.0 | 29.7 | 295.0 | 2.48e-79 |
| AKC91849.1 | nonribosomal\_peptide\_synthetase | BGC0001414 | NRP | 28.0 | 28.1 | 296.0 | 2.5e-79 |
| AAK89727.1 | peptide\_synthetase,\_siderophore\_biosynthesis\_protein | BGC0002107 | NRP+Polyketide | 25.0 | 37.9 | 293.0 | 2.69e-79 |
| EAU29529.1 | hypothetical\_protein | BGC0000682 | Terpene | 31.0 | 20.3 | 295.0 | 2.88e-79 |
| AAC44128.1 | saframycin\_Mx1\_synthetase\_B | BGC0002706 | NRP | 26.0 | 32.2 | 294.0 | 3.28e-79 |
| CCE67070.1 | polyketide\_synthase | BGC0001242 | Polyketide | 28.0 | 25.1 | 294.0 | 4.55e-79 |
| BBG67008.1 | polyketide\_synthase\_Sre6 | BGC0002604 | Polyketide | 29.0 | 22.3 | 293.0 | 4.9e-79 |
| ABA59547.1 | NRPS | BGC0000453 | NRP:Cyclic depsipeptide | 24.0 | 44.8 | 295.0 | 5.28e-79 |
| AEA29644.1 | putative\_nonribosomal\_peptide\_synthetase\_and\_kinurenine\_monooxygenase | BGC0000409 | NRP | 26.0 | 37.9 | 293.0 | 5.65e-79 |
| AAF08795.1 | MycA | BGC0001103 | NRP+Polyketide | 24.0 | 29.4 | 295.0 | 5.77e-79 |
| APZ78855.1 | nonribosomal\_peptide\_synthetase | BGC0001432 | NRP:Cyclic depsipeptide+Polyketide:Iterative type I polyketide | 27.0 | 25.2 | 294.0 | 6.34e-79 |
| ALV86867.1 | Tlo21 | BGC0001406 | NRP | 26.0 | 32.1 | 295.0 | 6.77e-79 |
| ALG65336.1 | Var3 | BGC0002416 | NRP+Polyketide | 25.0 | 31.5 | 293.0 | 7.01e-79 |
| CAM56770.1 |  | BGC0000354 | NRP | 28.0 | 29.9 | 295.0 | 7.03e-79 |
| KUM80513.1 | hypothetical\_protein | BGC0001562 | NRP | 27.0 | 30.6 | 294.0 | 7.16e-79 |
| ABI22132.1 | putative\_non-ribosomal\_peptide\_synthetase | BGC0000422 | NRP | 27.0 | 29.7 | 287.0 | 7.57e-79 |
| BBG28471.1 | polyketide\_synthase\_Cle1 | BGC0002603 | Polyketide | 28.0 | 23.9 | 293.0 | 7.97e-79 |
| AGU50949.1 | putative\_non-ribosomal\_peptide\_synthetase | BGC0002417 | NRP+Polyketide | 27.0 | 29.6 | 290.0 | 1.04e-78 |
| AEG64698.1 | LpmD | BGC0000379 | NRP | 26.0 | 29.8 | 293.0 | 1.44e-78 |
| CDE97356.1 | plipastatin\_synthase\_subunit\_C | BGC0001686 | NRP | 22.0 | 40.0 | 293.0 | 1.49e-78 |
| AJV88377.1 | MfnE | BGC0001214 | NRP | 27.0 | 33.2 | 293.0 | 1.61e-78 |
| CBJ89760.1 | Polyketide\_synthase\_involved\_in\_xenocoumacin\_synthesis | BGC0001054 | NRP+Polyketide:Modular type I polyketide | 28.0 | 22.2 | 290.0 | 1.62e-78 |
| AZH29360.1 | amino\_acid\_adenylation\_domain-containing\_protein | BGC0001843 | NRP | 25.0 | 32.7 | 293.0 | 1.79e-78 |
| CDG76959.1 | non-ribosomal\_peptide\_synthetase,\_terminal\_component | BGC0000446 | NRP:Pyrrolobenzodiazepine | 24.0 | 37.8 | 290.0 | 1.84e-78 |
| CBJ90288.1 | Peptide\_synthetase | BGC0000416 | NRP | 28.0 | 24.6 | 290.0 | 2.13e-78 |
| WP\_013184318.1 | non-ribosomal\_peptide\_synthetase | BGC0001692 | NRP | 28.0 | 24.6 | 290.0 | 2.13e-78 |
| CBF83139.1 | polyketide\_synthase,\_putative\_(JCVI) | BGC0001722 | Polyketide | 28.0 | 23.1 | 292.0 | 2.15e-78 |
| QEO75073.1 | condensation\_domain-containing\_protein | BGC0002079 | NRP:Cyclic depsipeptide | 25.0 | 32.4 | 292.0 | 2.54e-78 |
| ANG60379.1 | nonribosomal\_peptide\_synthetase\_BudA | BGC0001434 | NRP | 25.0 | 29.7 | 286.0 | 2.72e-78 |
| QCO93110.1 | polyketide\_synthase | BGC0001976 | Terpene | 27.0 | 23.5 | 292.0 | 2.82e-78 |
| ABM21571.1 | crpC | BGC0000975 | NRP+Polyketide | 27.0 | 24.5 | 291.0 | 2.89e-78 |
| BAP05596.1 | calH | BGC0000967 | NRP+Polyketide:Trans-AT type I polyketide | 24.0 | 32.9 | 292.0 | 3.16e-78 |
| NHN68324.1 | amino\_acid\_adenylation\_domain-containing\_protein | BGC0002719 | NRP | 26.0 | 29.9 | 292.0 | 3.28e-78 |
| BAD55611.1 | putative\_non-ribosomal\_peptide\_synthetase | BGC0001027 | NRP+Polyketide | 26.0 | 29.4 | 290.0 | 3.3e-78 |
| AGN74885.1 | nonribosomal\_peptide\_synthetase | BGC0000459 | NRP:Cyclic depsipeptide+Polyketide:Trans-AT type I polyketide | 26.0 | 29.8 | 292.0 | 4.4e-78 |
| BAY02138.1 | peptide\_synthetase | BGC0002532 | NRP+Polyketide | 26.0 | 29.5 | 284.0 | 4.95e-78 |
| DAB41477.1 | nonribosomal\_peptide\_synthetase | BGC0001230 | NRP:Cyclic depsipeptide+Polyketide:Modular type I polyketide | 27.0 | 33.6 | 291.0 | 5.01e-78 |
| ABS90473.1 | NRPS | BGC0001106 | NRP+Polyketide | 27.0 | 29.3 | 290.0 | 5.28e-78 |
| AAC06346.1 | bacitracin\_synthetase\_1 | BGC0000310 | NRP | 25.0 | 31.1 | 291.0 | 7.31e-78 |
| KMO93435.1 | NRPS/PKS | BGC0002095 | NRP | 27.0 | 29.9 | 283.0 | 8.72e-78 |
| ALI92655.1 | CitS\_citrinin\_polyketide\_synthase | BGC0001338 | Polyketide:Iterative type I polyketide | 27.0 | 22.7 | 290.0 | 1.04e-77 |
| UEF20591.1 | nonribosomal\_peptide\_synthetase | BGC0002360 | NRP | 28.0 | 26.2 | 287.0 | 1.22e-77 |
| BCD33690.1 | non-ribosomal\_peptide\_synthetase | BGC0002448 | NRP | 27.0 | 28.8 | 290.0 | 1.32e-77 |
| ABF87031.1 | non-ribosomal\_peptide\_synthetase/polyketide\_synthase | BGC0000393 | NRP+Polyketide:Modular type I polyketide | 25.0 | 31.9 | 290.0 | 1.53e-77 |
| AQM37583.1 | nonribosomal\_peptide\_synthetase | BGC0001424 | NRP:Cyclic depsipeptide+Polyketide:Iterative type I polyketide | 26.0 | 26.9 | 290.0 | 1.59e-77 |
| CBJ89766.1 | Polyketide\_synthase\_involved\_in\_xenocoumacin\_synthesis | BGC0001054 | NRP+Polyketide:Modular type I polyketide | 29.0 | 22.3 | 290.0 | 1.74e-77 |
| DAC80528.1 | peptide\_synthetase | BGC0001878 | NRP+Polyketide | 25.0 | 30.0 | 285.0 | 1.83e-77 |
| CAJ46692.1 | non-ribosomal\_peptide\_synthase | BGC0000969 | NRP:Cyclic depsipeptide+Polyketide:Modular type I polyketide | 27.0 | 25.1 | 290.0 | 1.88e-77 |
| ABS74209.1 | fengycin\_synthetase\_A | BGC0001095 | NRP | 25.0 | 32.0 | 289.0 | 2.3e-77 |
| ACH72912.1 | AflC | BGC0000011 | Polyketide | 28.0 | 25.0 | 288.0 | 2.46e-77 |
| EIN09536.1 | polyketide\_synthase | BGC0002213 | Polyketide | 30.0 | 22.0 | 288.0 | 2.53e-77 |
| QNL14923.1 | AptC | BGC0002512 | NRP | 27.0 | 26.9 | 288.0 | 3.06e-77 |
| AGI89790.1 | ATP-dependent\_valine\_adenylase | BGC0001792 | NRP | 28.0 | 31.5 | 289.0 | 3.36e-77 |
| WP\_028678148.1 | non-ribosomal\_peptide\_synthetase | BGC0001228 | NRP:Cyclic depsipeptide | 28.0 | 25.2 | 288.0 | 3.64e-77 |
| CAJ96468.1 | non-ribosomal\_peptide\_synthetase | BGC0000330 | NRP:NRP siderophore | 26.0 | 30.0 | 281.0 | 4e-77 |
| QYA95662.1 | amino\_acid\_adenylation\_domain-containing\_protein | BGC0002676 | NRP | 25.0 | 30.3 | 283.0 | 4.02e-77 |
| KIJ60886.1 | polyketide\_synthase | BGC0002214 | Polyketide | 31.0 | 21.1 | 288.0 | 4.11e-77 |
| CBD77749.1 | non-ribosomal\_peptide\_synthetase | BGC0000974 | NRP+Polyketide | 26.0 | 29.8 | 286.0 | 4.15e-77 |
| AFK57219.1 | DidH | BGC0000985 | Polyketide+NRP:Cyclic depsipeptide | 27.0 | 29.2 | 284.0 | 4.27e-77 |
| ATJ04411.1 | NRPS,\_TomB\_binding | BGC0001637 | NRP | 23.0 | 37.7 | 285.0 | 5.17e-77 |
| AFJ14794.1 | PlpE | BGC0000403 | NRP | 26.0 | 31.4 | 288.0 | 5.42e-77 |
| AWI62629.1 | nonribosomal\_peptide\_synthetase | BGC0001822 | NRP | 28.0 | 24.7 | 286.0 | 6.07e-77 |
| AAC06348.1 | bacitracin\_synthetase\_3 | BGC0000310 | NRP | 26.0 | 29.1 | 288.0 | 6.63e-77 |
| AHD05679.1 | putative\_non-ribosomal\_peptide\_ligase/\_polyketide\_synthase\_hybrid | BGC0000402 | NRP | 24.0 | 31.8 | 288.0 | 7.27e-77 |
| CEK23605.1 | Non-ribosomal\_peptide\_synthase\_involved\_in\_xenematides\_synthesis | BGC0001825 | NRP | 24.0 | 32.0 | 288.0 | 8e-77 |
| ANZ15839.1 | peptide\_synthetase\_ScpsB | BGC0001569 | NRP | 27.0 | 29.9 | 287.0 | 9.15e-77 |
| BAH22763.1 | nonribosomal\_peptide\_synthetase | BGC0001018 | NRP | 24.0 | 30.4 | 281.0 | 1.01e-76 |
| KFH44362.1 | Conidial\_yellow\_pigment\_biosynthesis\_polyketide\_synthase-like\_protein | BGC0002190 | Polyketide | 28.0 | 23.1 | 286.0 | 1.21e-76 |
| APZ78845.1 | nonribosomal\_peptide\_synthetase | BGC0001431 | NRP:Cyclic depsipeptide+Polyketide:Iterative type I polyketide | 26.0 | 32.4 | 286.0 | 1.37e-76 |
| AAZ23078.1 | peptide\_synthetase | BGC0000291 | NRP | 27.0 | 30.2 | 286.0 | 1.83e-76 |
| CAF05648.1 | TubC\_protein | BGC0001053 | NRP+Polyketide | 26.0 | 29.4 | 286.0 | 2.05e-76 |
| QIE08736.1 | non-ribosomal\_peptide\_synthetase | BGC0002544 | NRP | 25.0 | 33.5 | 286.0 | 2.08e-76 |
| OBR09781.1 | Polyketide\_synthase | BGC0002429 | Terpene+Polyketide | 29.0 | 22.5 | 285.0 | 2.17e-76 |
| AGZ15460.1 | putative\_non-ribosomal\_peptide\_synthetase | BGC0001036 | NRP+Polyketide | 27.0 | 26.0 | 284.0 | 3.13e-76 |
| AWN90\_15505 | non-ribosomal\_peptide\_synthetase | BGC0002352 | Other | 26.0 | 30.1 | 284.0 | 3.42e-76 |
| AET98905.1 | putative\_non-ribosomal\_peptide\_synthetase | BGC0000415 | NRP | 28.0 | 25.4 | 285.0 | 4.12e-76 |
| AXN93591.1 | PuwH | BGC0001951 | NRP | 26.0 | 30.1 | 279.0 | 4.14e-76 |
| QCX41945.1 | Amc8 | BGC0001957 | Polyketide | 30.0 | 23.0 | 277.0 | 5e-76 |
| QHW08554.1 | polyketide\_synthase | BGC0002054 | Polyketide+NRP+Saccharide | 30.0 | 23.0 | 277.0 | 5e-76 |
| ABG94125.1 | non-ribosomal\_peptide\_synthetase | BGC0000417 | NRP | 25.0 | 32.9 | 284.0 | 5.32e-76 |
| ABY83142.1 | Azi3 | BGC0000960 | NRP+Polyketide | 26.0 | 29.3 | 278.0 | 5.58e-76 |
| CAL17541.1 | peptide\_synthetase,\_putative | BGC0002465 | NRP | 25.0 | 32.9 | 285.0 | 5.89e-76 |
| AAF00961.1 | mcyB | BGC0001017 | NRP+Polyketide:Modular type I polyketide | 25.0 | 29.6 | 284.0 | 5.94e-76 |
| AJY78093.1 | polyketide\_synthase | BGC0001902 | NRP+Polyketide | 30.0 | 20.6 | 283.0 | 8.16e-76 |
| DAB41478.1 | nonribosomal\_peptide\_synthetase | BGC0001230 | NRP:Cyclic depsipeptide+Polyketide:Modular type I polyketide | 26.0 | 29.9 | 282.0 | 9.13e-76 |
| ARU80380.1 | polyketide\_synthase | BGC0001542 | Polyketide | 27.0 | 24.8 | 283.0 | 1.07e-75 |
| XP\_001798923.1 | polyketide\_synthase | BGC0001865 | Polyketide:Iterative type I polyketide | 28.0 | 24.8 | 283.0 | 1.08e-75 |
| EED18001.1 | NR-PKS | BGC0000154 | Polyketide:Iterative type I polyketide | 29.0 | 23.0 | 283.0 | 1.37e-75 |
| AXN93582.1 | PuwH | BGC0001950 | NRP | 25.0 | 30.0 | 277.0 | 1.37e-75 |
| QCP68975.1 | VatS | BGC0002296 | NRP+Polyketide | 27.0 | 24.9 | 281.0 | 1.74e-75 |
| AWM95789.1 | non-reduciing\_polyketide\_synthase\_methylorcinaldehyde\_synthase | BGC0001827 | Polyketide | 27.0 | 22.8 | 283.0 | 1.83e-75 |
| CAG29031.1 | nonribosomal\_peptide\_synthetase\_(modules\_1\_and\_2) | BGC0001023 | NRP+Polyketide:Modular type I polyketide | 26.0 | 26.3 | 282.0 | 2.64e-75 |
| APZ78821.1 | nonribosomal\_peptide\_synthetase | BGC0001429 | NRP:Cyclic depsipeptide+Polyketide:Iterative type I polyketide | 26.0 | 26.3 | 282.0 | 2.64e-75 |
| BAI63289.1 | putative\_non-ribosomal\_peptide\_synthetase | BGC0000434 | NRP | 28.0 | 25.3 | 282.0 | 3.55e-75 |
| AEC14347.1 | nonribosomal\_peptide\_synthetase | BGC0000377 | NRP | 25.0 | 29.5 | 280.0 | 3.78e-75 |
| QXJ21807.1 | amino\_acid\_adenylation\_domain-containing\_protein | BGC0002370 | NRP | 26.0 | 32.2 | 281.0 | 4.53e-75 |
| AGC09528.1 | NRPS | BGC0001183 | Polyketide | 29.0 | 28.3 | 281.0 | 4.65e-75 |
| ABM34276.1 | amino\_acid\_adenylation\_domain\_protein | BGC0002419 | NRP+Polyketide | 26.0 | 30.2 | 278.0 | 4.93e-75 |
| AJW76709.1 | DsaG | BGC0001196 | NRP | 26.0 | 30.7 | 281.0 | 6.82e-75 |
| AKL78825.1 | GLNRPS7 | BGC0001187 | NRP:Lipopeptide+Polyketide:Iterative type I polyketide | 28.0 | 24.6 | 271.0 | 7.35e-75 |
| CBJ90287.1 | peptide\_synthetase | BGC0000416 | NRP | 27.0 | 25.7 | 279.0 | 8.38e-75 |
| WP\_013184317.1 | non-ribosomal\_peptide\_synthetase | BGC0001692 | NRP | 27.0 | 25.7 | 279.0 | 8.38e-75 |
| AAZ03551.1 | McnB | BGC0000332 | NRP | 24.0 | 30.4 | 275.0 | 1.01e-74 |
| EFL06867.1 | predicted\_protein | BGC0000300 | NRP | 26.0 | 29.6 | 280.0 | 1.01e-74 |
| KJY94239.1 | thioester\_reductase | BGC0002691 | NRP | 25.0 | 29.9 | 279.0 | 1.08e-74 |
| EAU35431.1 | hypothetical\_protein | BGC0002734 | Polyketide | 27.0 | 22.6 | 280.0 | 1.15e-74 |
| APZ78794.1 | nonribosomal\_peptide\_synthetase | BGC0001427 | NRP:Cyclic depsipeptide+Polyketide:Iterative type I polyketide | 25.0 | 26.7 | 280.0 | 1.32e-74 |
| AIW82285.1 | PuwH | BGC0001125 | NRP+Polyketide | 26.0 | 30.1 | 274.0 | 1.74e-74 |
| WP\_051700111.1 | non-ribosomal\_peptide\_synthetase | BGC0001368 | NRP | 25.0 | 30.0 | 276.0 | 1.94e-74 |
| AAU34202.1 | mannopeptimycin\_peptide\_synthetase\_MppA | BGC0000388 | NRP | 27.0 | 31.7 | 279.0 | 2.08e-74 |
| BAO84861.1 | putative\_non-ribosomal\_peptide\_synthetase | BGC0000414 | NRP | 24.0 | 30.1 | 273.0 | 2.48e-74 |
| PIB02405.1 | CTB1 | BGC0001541 | Polyketide | 27.0 | 22.7 | 278.0 | 2.76e-74 |
| ACM79806.1 | ZmaB | BGC0001059 | NRP+Polyketide | 24.0 | 31.8 | 278.0 | 2.78e-74 |
| ATV82110.1 | PKS | BGC0001909 | Polyketide | 30.0 | 22.5 | 279.0 | 2.79e-74 |
| ABK36076.1 | nonribosomal\_peptide\_synthetase | BGC0001502 | NRP | 27.0 | 29.2 | 278.0 | 3.25e-74 |
| BAH22762.1 | nonribosomal\_peptide\_synthetase | BGC0001018 | NRP | 25.0 | 29.8 | 273.0 | 3.44e-74 |
| ADL64235.1 | aureusimine\_non-ribosomal\_peptide\_synthetase | BGC0000308 | NRP | 25.0 | 29.3 | 278.0 | 5.29e-74 |
| AXN93603.1 | PuwH | BGC0001952 | NRP | 26.0 | 31.2 | 272.0 | 6.7e-74 |
| XP\_011392701.1 | uncharacterized\_protein | BGC0001281 | Polyketide | 27.0 | 25.4 | 277.0 | 6.72e-74 |
| ALD82526.1 | non-ribosomal\_peptide\_synthase | BGC0001212 | NRP+Polyketide | 28.0 | 29.4 | 278.0 | 7.25e-74 |
| AXG46164.1 | non-ribosomal\_peptide\_synthetase | BGC0002713 | NRP | 27.0 | 25.4 | 276.0 | 7.91e-74 |
| APZ78743.1 | nonribosomal\_peptide\_synthetase | BGC0001422 | NRP:Cyclic depsipeptide+Polyketide:Iterative type I polyketide | 27.0 | 25.5 | 277.0 | 8.7e-74 |
| APZ78833.1 | nonribosomal\_peptide\_synthetase | BGC0001430 | NRP:Cyclic depsipeptide+Polyketide:Iterative type I polyketide | 25.0 | 26.2 | 277.0 | 8.72e-74 |
| AGD80623.1 | non-ribosomal\_peptide\_synthetase | BGC0000394 | NRP | 25.0 | 32.1 | 277.0 | 8.8e-74 |
| CBJ90289.1 | peptide\_synthetase | BGC0000416 | NRP | 28.0 | 24.8 | 276.0 | 9.04e-74 |
| WP\_013184319.1 | non-ribosomal\_peptide\_synthetase | BGC0001692 | NRP | 28.0 | 24.8 | 276.0 | 9.04e-74 |
| QED55423.1 | nonribosomal\_peptide\_synthetase | BGC0001984 | NRP | 25.0 | 32.7 | 277.0 | 1.24e-73 |
| CAP95404.1 |  | BGC0001404 | Polyketide | 27.0 | 23.0 | 276.0 | 1.33e-73 |
| EFY96950.1 | BcPKS18,\_polyketide\_synthase | BGC0002427 | Terpene+Polyketide | 29.0 | 22.4 | 276.0 | 1.37e-73 |
| AXN93616.1 | PuwH | BGC0001953 | NRP | 26.0 | 31.2 | 271.0 | 1.58e-73 |
| MBD2892722.1 | D-alanine--D-alanyl\_carrier\_protein\_ligase | BGC0002718 | NRP | 26.0 | 30.1 | 276.0 | 1.76e-73 |
| QEO74981.1 | omn6 | BGC0002078 | NRP:Cyclic depsipeptide | 25.0 | 33.2 | 276.0 | 1.85e-73 |
| QEO75077.1 | condensation\_domain-containing\_protein | BGC0002079 | NRP:Cyclic depsipeptide | 26.0 | 29.8 | 276.0 | 1.94e-73 |
| ABQ96384.2 | fusaricidin\_synthetase | BGC0001152 | Polyketide+NRP:Lipopeptide | 25.0 | 31.1 | 276.0 | 2.17e-73 |
| AEC14346.1 | nonribosomal\_peptide\_synthetase | BGC0000377 | NRP | 23.0 | 31.4 | 275.0 | 2.91e-73 |
| AAF08796.1 | MycB | BGC0001103 | NRP+Polyketide | 25.0 | 31.2 | 276.0 | 3.37e-73 |
| AAF63833.1 | PstD | BGC0000362 | NRP | 27.0 | 29.1 | 272.0 | 4.99e-73 |
| AGZ03651.1 | sevB | BGC0000426 | NRP | 24.0 | 29.1 | 275.0 | 5.33e-73 |
| PHM26612.1 | pvdj | BGC0001130 | NRP+Polyketide | 23.0 | 31.8 | 275.0 | 5.34e-73 |
| AAT09804.1 | NocA | BGC0000395 | NRP | 25.0 | 31.9 | 275.0 | 6.45e-73 |
| WP\_054234643.1 | non-ribosomal\_peptide\_synthetase | BGC0002014 | NRP+Polyketide | 26.0 | 25.0 | 275.0 | 6.56e-73 |
| ACG60776.1 | NRPS(AL/ACP/C/A/PCP/C/A) | BGC0001058 | NRP:Glycopeptide+Polyketide:Modular type I polyketide+Saccharide:Hybrid/tailoring saccharide | 27.0 | 33.4 | 274.0 | 6.8e-73 |
| ANG60380.1 | nonribosomal\_peptide\_synthetase\_BudB | BGC0001434 | NRP | 26.0 | 25.5 | 273.0 | 6.89e-73 |
| RLV71193.1 | non-ribosomal\_peptide\_synthetase | BGC0001846 | NRP+Saccharide:Hybrid/tailoring saccharide | 23.0 | 41.4 | 273.0 | 1.35e-72 |
| ANG60381.1 | nonribosomal\_peptide\_synthetase\_BudC | BGC0001434 | NRP | 27.0 | 24.8 | 273.0 | 1.36e-72 |
| AQH32485.1 | peptide\_synthetase | BGC0001667 | NRP+Polyketide | 24.0 | 32.2 | 273.0 | 1.51e-72 |
| APZ78728.1 | nonribosomal\_peptide\_synthetase | BGC0001421 | NRP:Cyclic depsipeptide+Polyketide:Iterative type I polyketide | 26.0 | 25.5 | 273.0 | 1.67e-72 |
| ACM79810.1 | ZmaO | BGC0001059 | NRP+Polyketide | 23.0 | 29.7 | 271.0 | 1.68e-72 |
| CBJ90358.1 | putative\_Peptide\_synthetase | BGC0000465 | NRP | 26.0 | 24.9 | 272.0 | 1.77e-72 |
| QUJ09166.1 | Lon19 | BGC0002440 | NRP | 26.0 | 29.6 | 272.0 | 2e-72 |
| UHJ79951.1 | non-ribosomal\_peptide\_synthetase | BGC0002654 | NRP | 26.0 | 30.2 | 273.0 | 2.21e-72 |
| APZ78756.1 | nonribosomal\_peptide\_synthetase | BGC0001423 | NRP:Cyclic depsipeptide+Polyketide:Iterative type I polyketide | 27.0 | 29.2 | 273.0 | 2.37e-72 |
| AXG46165.1 | non-ribosomal\_peptide\_synthetase | BGC0002713 | NRP | 28.0 | 24.6 | 271.0 | 3.07e-72 |
| CUX79060.1 | Octapeptin\_synthase\_subunit\_A | BGC0001715 | NRP | 25.0 | 29.5 | 272.0 | 3.4e-72 |
| CAA60461.1 | pipecolate\_incorporating\_enzyme | BGC0001040 | NRP+Polyketide | 26.0 | 29.3 | 270.0 | 4.37e-72 |
| ctg1\_orf17 |  | BGC0001457 | NRP | 25.0 | 30.1 | 266.0 | 4.72e-72 |
| APZ78755.1 | nonribosomal\_peptide\_synthetase | BGC0001423 | NRP:Cyclic depsipeptide+Polyketide:Iterative type I polyketide | 26.0 | 25.5 | 271.0 | 4.91e-72 |
| ANS62966.1 | non-ribosomal\_peptide\_synthetase | BGC0001567 | NRP | 27.0 | 29.0 | 269.0 | 4.97e-72 |
| ADC79642.1 | TamD | BGC0001052 | NRP+Polyketide:Modular type I polyketide | 27.0 | 30.3 | 265.0 | 5.56e-72 |
| AZH29361.1 | amino\_acid\_adenylation\_domain-containing\_protein | BGC0001843 | NRP | 26.0 | 29.9 | 271.0 | 5.57e-72 |
| QWT72279.1 | non-ribosomal\_peptide\_synthetase | BGC0002430 | NRP+Saccharide | 27.0 | 33.5 | 271.0 | 7.18e-72 |
| QBC75023.1 | non-ribosomal\_peptide\_synthetase | BGC0001968 | NRP | 27.0 | 24.9 | 271.0 | 7.83e-72 |
| AEW31015.1 | plipastatin\_synthetase | BGC0000407 | NRP | 25.0 | 29.6 | 267.0 | 9.71e-72 |
| ABL74940.1 | NRPS | BGC0001048 | NRP:Glycopeptide+Polyketide:Modular type I polyketide+Saccharide:Hybrid/tailoring saccharide | 26.0 | 34.1 | 270.0 | 1.01e-71 |
| AAC49191.1 | putative\_polyketide\_synthase | BGC0000152 | Polyketide | 27.0 | 22.8 | 270.0 | 1.03e-71 |
| EAQ86392.1 | hypothetical\_protein | BGC0001405 | Polyketide | 29.0 | 20.7 | 266.0 | 1.14e-71 |
| EAU31923.1 | hypothetical\_protein | BGC0002267 | Polyketide | 27.0 | 25.4 | 270.0 | 1.73e-71 |
| ACA97576.1 | PmxA | BGC0000408 | NRP | 26.0 | 30.4 | 270.0 | 1.84e-71 |
| AAL33758.1 | putative\_non-ribosomal\_peptide\_synthetase | BGC0000421 | NRP | 25.0 | 38.2 | 267.0 | 2.75e-71 |
| BAE98156.1 | putative\_non-ribosomal\_peptide\_synthetase | BGC0000339 | NRP | 27.0 | 25.0 | 269.0 | 3.29e-71 |
| CBF69451.1 | polyketide\_synthase,\_putative\_(JCVI) | BGC0000037 | Polyketide | 29.0 | 20.4 | 268.0 | 3.59e-71 |
| QCP68969.1 | VatN | BGC0002296 | NRP+Polyketide | 25.0 | 24.5 | 268.0 | 3.89e-71 |
| AEZ51516.1 | pmxA | BGC0001153 | NRP:Lipopeptide | 26.0 | 30.3 | 269.0 | 4.09e-71 |
| ACA97577.1 | PmxB | BGC0000408 | NRP | 27.0 | 24.9 | 263.0 | 5.33e-71 |
| CUX79061.1 | Octapeptin\_synthase\_subunit\_B | BGC0001715 | NRP | 25.0 | 30.9 | 268.0 | 5.35e-71 |
| AQX14441.1 | EM5400\_NRPS\_scaffold | BGC0001671 | NRP | 26.0 | 29.9 | 268.0 | 6.89e-71 |
| ADH04679.1 | non-ribosomal\_peptide\_synthetase | BGC0001344 | NRP+Polyketide | 26.0 | 30.5 | 267.0 | 8.38e-71 |
| CCM44337.1 | Nonribosomal\_peptide\_synthetase | BGC0001056 | NRP+Polyketide:Modular type I polyketide+Polyketide:PUFA synthase or related polyketide | 25.0 | 30.3 | 267.0 | 8.9e-71 |
| QEO74983.1 | omn8 | BGC0002078 | NRP:Cyclic depsipeptide | 26.0 | 29.8 | 267.0 | 9.52e-71 |
| AEH42487.1 | polyketide\_synthase | BGC0000032 | Polyketide | 29.0 | 21.6 | 266.0 | 1.07e-70 |
| AYJ71720.1 | non-ribosomal\_peptide\_synthetase | BGC0001942 | NRP+Polyketide | 26.0 | 29.5 | 266.0 | 1.14e-70 |
| ACO79122.1 | Type\_I\_fatty\_acid\_synthase\_ArsA | BGC0000284 | Polyketide | 27.0 | 23.7 | 266.0 | 1.37e-70 |
| AHZ20774.1 | non-ribosomal\_peptide\_synthase | BGC0000369 | NRP+Saccharide:Hybrid/tailoring saccharide | 25.0 | 30.2 | 266.0 | 1.8e-70 |
| AAN85501.1 | nonribosomal\_peptide\_synthetase | BGC0001101 | NRP+Polyketide:Modular type I polyketide+Polyketide:Trans-AT type I polyketide | 26.0 | 30.2 | 261.0 | 2.25e-70 |
| AZH23822.1 | MgiJ | BGC0001971 | NRP+Polyketide | 25.0 | 24.9 | 265.0 | 2.26e-70 |
| RLV71192.1 | non-ribosomal\_peptide\_synthetase | BGC0001846 | NRP+Saccharide:Hybrid/tailoring saccharide | 25.0 | 42.5 | 266.0 | 2.48e-70 |
| CAJ76290.1 | putative\_non-ribosomal\_peptide\_synthase | BGC0000972 | NRP+Polyketide:Modular type I polyketide+Polyketide:Trans-AT type I polyketide | 24.0 | 30.9 | 265.0 | 2.6e-70 |
| APO47826.1 | hypothetical\_protein | BGC0002653 | NRP | 26.0 | 29.5 | 266.0 | 2.69e-70 |
| WP\_054234617.1 | non-ribosomal\_peptide\_synthetase | BGC0002014 | NRP+Polyketide | 25.0 | 30.8 | 265.0 | 5.28e-70 |
| AIG79241.1 | Hypothetical\_protein | BGC0000419 | Saccharide+NRP:Glycopeptide | 26.0 | 29.1 | 265.0 | 5.52e-70 |
| RAT94091.1 | NRPS | BGC0001469 | NRP | 25.0 | 30.6 | 259.0 | 6.59e-70 |
| QBQ12464.1 | amino\_acid\_adenylation\_domain-containing\_protein | BGC0002693 | NRP | 27.0 | 28.7 | 264.0 | 9.08e-70 |
| AIE77059.1 | peptide\_synthetase | BGC0000418 | NRP | 27.0 | 29.1 | 264.0 | 9.44e-70 |
| CEK23365.1 | conserved\_hypothetical\_protein | BGC0001716 | NRP | 24.0 | 29.9 | 259.0 | 9.66e-70 |
| MBE3200466.1 | non-ribosomal\_peptide\_synthetase | BGC0002409 | NRP | 26.0 | 31.7 | 264.0 | 1.11e-69 |
| CCM44336.1 | Nonribosomal\_peptide\_synthetase | BGC0001056 | NRP+Polyketide:Modular type I polyketide+Polyketide:PUFA synthase or related polyketide | 25.0 | 31.7 | 264.0 | 1.26e-69 |
| CAJ34375.1 | NRPS | BGC0000445 | NRP:Cyclic depsipeptide | 26.0 | 25.1 | 263.0 | 1.85e-69 |
| UEF20592.1 | nonribosomal\_peptide\_synthetase | BGC0002360 | NRP | 26.0 | 29.7 | 259.0 | 2.37e-69 |
| QIE07359.1 | dimodular\_nonribosomal\_peptide\_synthase\_NecA | BGC0002050 | NRP+Polyketide:Trans-AT type I polyketide | 25.0 | 29.8 | 260.0 | 2.78e-69 |
| AHD05627.1 | putative\_non-ribosomal\_peptide\_ligase\_domain\_protein | BGC0001033 | NRP+Polyketide | 23.0 | 29.2 | 261.0 | 2.79e-69 |
| APZ78782.1 | nonribosomal\_peptide\_synthetase | BGC0001426 | NRP:Cyclic depsipeptide+Polyketide:Iterative type I polyketide | 26.0 | 29.5 | 263.0 | 2.89e-69 |
| AAY37653.1 | Amino\_acid\_adenylation | BGC0000437 | NRP | 24.0 | 30.0 | 263.0 | 3.08e-69 |
| AFY58521.1 | amino\_acid\_adenylation\_enzyme/thioester\_reductase\_family\_protein | BGC0002411 | NRP+Polyketide | 25.0 | 29.7 | 261.0 | 3.12e-69 |
| AKA59448.1 | polyketide\_synthase | BGC0001203 | NRP+Polyketide | 30.0 | 22.3 | 261.0 | 3.27e-69 |
| OTA20325.1 | peptide\_synthase | BGC0001824 | NRP | 24.0 | 32.2 | 262.0 | 3.55e-69 |
| EAA65602.1 | hypothetical\_protein | BGC0000022 | Polyketide | 27.0 | 25.6 | 262.0 | 3.82e-69 |
| ORC16618.1 | hypothetical\_protein | BGC0001341 | NRP | 23.0 | 41.4 | 262.0 | 4.33e-69 |
| AAF99707.2 | syringopeptin\_synthetase | BGC0000438 | NRP | 24.0 | 29.9 | 262.0 | 5.27e-69 |
| WP\_013310343.1 | non-ribosomal\_peptide\_synthetase | BGC0001728 | NRP+Polyketide | 25.0 | 29.7 | 259.0 | 7.1e-69 |
| CAB15186.3 | siderophore\_2,3-dihydroxybenzoate-glycine-threonine\_trimeric\_ester\_bacillibactin\_synthetase | BGC0000309 | NRP | 25.0 | 29.8 | 261.0 | 7.39e-69 |
| ABS74179.1 | bacillomycin\_D\_synthetase\_C | BGC0001090 | Polyketide+NRP:Lipopeptide | 24.0 | 28.9 | 261.0 | 8.11e-69 |
| AMJ52084.1 | lijE | BGC0002255 | Polyketide | 25.0 | 22.8 | 261.0 | 8.29e-69 |
| QNH67550.1 | Cip22 | BGC0002108 | NRP | 26.0 | 30.2 | 261.0 | 9.69e-69 |
| AQX14497.1 | monobactam\_NRPS\_scaffold\_4 | BGC0001672 | NRP | 23.0 | 29.8 | 260.0 | 1e-68 |
| AZH23792.1 | MgcJ | BGC0001970 | NRP+Polyketide | 25.0 | 25.0 | 259.0 | 1.03e-68 |
| BAX90000.1 | Non-ribosomal\_peptide\_synthetase | BGC0001628 | NRP | 26.0 | 29.3 | 261.0 | 1.04e-68 |
| QKF54435.2 | nonribosomal\_peptide\_synthetase | BGC0002581 | NRP | 26.0 | 29.7 | 260.0 | 1.06e-68 |
| KYC41483.1 | hypothetical\_protein | BGC0002484 | NRP+Polyketide | 27.0 | 24.2 | 256.0 | 1.14e-68 |
| CDG17987.1 | Putative\_Ornithine\_racemase\_(fragment) | BGC0000464 | NRP:Cyclic depsipeptide | 26.0 | 30.0 | 261.0 | 1.15e-68 |
| AJM89735.1 | PmxA | BGC0001192 | NRP | 26.0 | 30.0 | 261.0 | 1.15e-68 |
| CBJ79916.1 | putative\_Ornithine\_racemase | BGC0001133 | NRP | 24.0 | 32.1 | 260.0 | 1.47e-68 |
| CCJ67646.1 | JagB | BGC0001127 | NRP | 25.0 | 29.4 | 260.0 | 1.52e-68 |
| QBG38782.1 | Atr21 | BGC0001975 | NRP | 26.0 | 29.7 | 260.0 | 1.8e-68 |
| QCX41916.1 | Mhr10 | BGC0001956 | Polyketide | 29.0 | 22.8 | 254.0 | 1.82e-68 |
| QHD26313.1 | polyketide\_synthase | BGC0002479 | Polyketide+NRP+Saccharide | 29.0 | 22.8 | 254.0 | 1.82e-68 |
| AZM51140.1 | non-ribosomal\_peptide\_synthetase | BGC0002702 | NRP | 26.0 | 29.5 | 258.0 | 1.98e-68 |
| AJM89734.1 | PmxB | BGC0001192 | NRP | 27.0 | 23.0 | 255.0 | 2.14e-68 |
| AVV61987.1 | putative\_non-ribosomal\_peptide\_synthetase | BGC0001477 | NRP+Polyketide:Modular type I polyketide | 26.0 | 29.9 | 254.0 | 2.5e-68 |
| NAO96318.1 | amino\_acid\_adenylation\_domain-containing\_protein | BGC0002117 | NRP | 25.0 | 29.5 | 259.0 | 2.55e-68 |
| CBJ79915.1 | putative\_Phenylalanine\_racemase\_(ATP-hydrolyzing) | BGC0001133 | NRP | 24.0 | 31.6 | 259.0 | 3.08e-68 |
| QSJ20135.1 | non-ribosomal\_peptide\_synthase/polyketide\_synthase | BGC0002572 | NRP+Polyketide | 25.0 | 29.5 | 259.0 | 4.02e-68 |
| APZ78809.1 | nonribosomal\_peptide\_synthetase | BGC0001428 | NRP:Cyclic depsipeptide+Polyketide:Iterative type I polyketide | 26.0 | 29.4 | 259.0 | 4.23e-68 |
| ACM68690.1 | AerG1 | BGC0000298 | NRP | 24.0 | 29.4 | 254.0 | 4.59e-68 |
| CAJ77716.1 | Mps2\_protein | BGC0000364 | NRP | 25.0 | 38.1 | 258.0 | 5.27e-68 |
| AHD05677.1 | nonribosomal\_peptide\_ligase\_subunit | BGC0000402 | NRP | 25.0 | 29.5 | 258.0 | 5.3e-68 |
| APZ78744.1 | nonribosomal\_peptide\_synthetase | BGC0001422 | NRP:Cyclic depsipeptide+Polyketide:Iterative type I polyketide | 27.0 | 28.9 | 258.0 | 5.54e-68 |
| CBZ42146.1 | putative\_non-ribosomal\_peptide\_synthetase | BGC0001117 | NRP | 27.0 | 29.5 | 258.0 | 5.79e-68 |
| AAL33757.1 | putative\_non-ribosomal\_peptide\_synthetase | BGC0000421 | NRP | 25.0 | 29.2 | 253.0 | 6.17e-68 |
| APZ78795.1 | nonribosomal\_peptide\_synthetase | BGC0001427 | NRP:Cyclic depsipeptide+Polyketide:Iterative type I polyketide | 26.0 | 29.4 | 258.0 | 7.24e-68 |
| OLZ52456.1 | non-ribosomal\_peptide\_synthetase | BGC0001462 | NRP:Glycopeptide | 27.0 | 29.3 | 257.0 | 7.79e-68 |
| QDQ83031.1 | amino\_acid\_adenylation\_domain-containing\_protein | BGC0002564 | NRP | 26.0 | 29.6 | 256.0 | 8.16e-68 |
| AAK89731.2 | siderophore\_biosynthesis\_protein | BGC0002107 | NRP+Polyketide | 25.0 | 30.7 | 253.0 | 8.34e-68 |
| QTT72098.1 | non-ribosomal\_peptide\_synthetase | BGC0002350 | NRP+Polyketide+Saccharide | 26.0 | 29.7 | 254.0 | 8.35e-68 |
| APZ78769.1 | nonribosomal\_peptide\_synthetase | BGC0001425 | NRP:Cyclic depsipeptide+Polyketide:Iterative type I polyketide | 26.0 | 29.5 | 258.0 | 9.47e-68 |
| AAT01807.1 | non-ribosomal\_peptide\_synthetase | BGC0000365 | NRP | 24.0 | 37.9 | 256.0 | 1.53e-67 |
| NAO96320.1 | amino\_acid\_adenylation\_domain-containing\_protein | BGC0002117 | NRP | 25.0 | 30.0 | 257.0 | 1.63e-67 |
| NAO96319.1 | amino\_acid\_adenylation\_domain-containing\_protein | BGC0002117 | NRP | 25.0 | 29.7 | 256.0 | 1.71e-67 |
| AHB82071.1 | non\_ribosomal\_peptide\_synthetase | BGC0001231 | NRP+Polyketide:Modular type I polyketide | 27.0 | 29.5 | 256.0 | 2.05e-67 |
| CAD29798.1 | peptide\_synthetase | BGC0001015 | NRP+Polyketide | 24.0 | 30.1 | 256.0 | 2.18e-67 |
| AFY58522.1 | non-ribosomal\_peptide\_synthase/amino\_acid\_adenylation\_enzyme | BGC0002411 | NRP+Polyketide | 24.0 | 29.9 | 254.0 | 3.12e-67 |
| AAG06715.1 | probable\_non-ribosomal\_peptide\_synthetase | BGC0002037 | NRP | 27.0 | 30.0 | 255.0 | 3.17e-67 |
| OLZ50886.1 | non-ribosomal\_peptide\_synthetase | BGC0001461 | NRP:Glycopeptide | 27.0 | 29.7 | 254.0 | 5.19e-67 |
| AYA22333.1 | KerD | BGC0001955 | NRP | 27.0 | 29.7 | 254.0 | 5.19e-67 |
| CAE02633.1 | surfactin\_synthetase\_C\_ | BGC0000433 | NRP:Lipopeptide | 25.0 | 29.7 | 252.0 | 5.22e-67 |
| AAF08797.1 | MycC | BGC0001103 | NRP+Polyketide | 23.0 | 28.7 | 254.0 | 5.99e-67 |
| AAG02358.1 | peptide\_synthetase\_NRPS6 | BGC0000963 | NRP:Glycopeptide+Polyketide:Modular type I polyketide+Saccharide:Hybrid/tailoring saccharide | 26.0 | 29.7 | 251.0 | 6.51e-67 |
| EFE73312.1 | nonribosomal\_peptide\_synthetase | BGC0000431 | NRP:Cyclic depsipeptide | 26.0 | 33.3 | 255.0 | 6.8e-67 |
| AGS77309.1 | NRPS\_modules\_4-6 | BGC0001178 | NRP:Glycopeptide | 25.0 | 29.5 | 254.0 | 7.82e-67 |
| QKW60393.1 | amino\_acid\_adenylation\_domain-containing\_protein | BGC0002288 | NRP | 25.0 | 29.4 | 252.0 | 8.83e-67 |
| QYC40290.1 | A50926\_NRPS,\_module\_7 | BGC0002344 | NRP | 27.0 | 29.7 | 253.0 | 8.97e-67 |
| AHD05618.1 | putative\_non-ribosomal\_peptide\_ligase\_domain\_protein | BGC0001033 | NRP+Polyketide | 24.0 | 30.8 | 249.0 | 1.12e-66 |
| QWM97320.1 | non-ribosomal\_peptide\_synthetase | BGC0002384 | NRP | 25.0 | 29.6 | 254.0 | 1.18e-66 |
| AAY37647.1 | Amino\_acid\_adenylation | BGC0000437 | NRP | 25.0 | 29.9 | 254.0 | 1.27e-66 |
| AIG79240.1 | Hypothetical\_protein | BGC0000419 | Saccharide+NRP:Glycopeptide | 27.0 | 29.3 | 253.0 | 1.52e-66 |
| ANY58984.1 | non-ribosomal\_synthetase | BGC0001615 | NRP | 24.0 | 29.3 | 251.0 | 1.52e-66 |
| WP\_064118616.1 | non-ribosomal\_peptide\_synthetase | BGC0002075 | Alkaloid+NRP:Lipopeptide | 26.0 | 27.4 | 251.0 | 1.74e-66 |
| AHB38515.1 | non-ribosomal\_peptide\_synthetase | BGC0000345 | NRP+Polyketide:Modular type I polyketide | 24.0 | 31.7 | 253.0 | 1.88e-66 |
| AEZ51517.1 | pmxB | BGC0001153 | NRP:Lipopeptide | 26.0 | 25.0 | 249.0 | 2.02e-66 |
| CAA11795.1 | PCZA363.4 | BGC0000322 | NRP | 26.0 | 29.5 | 253.0 | 2.29e-66 |
| ABS74180.1 | bacillomycin\_D\_synthetase\_B | BGC0001090 | Polyketide+NRP:Lipopeptide | 24.0 | 32.2 | 253.0 | 3.29e-66 |
| ABS74205.1 | fengycin\_synthetase\_E | BGC0001095 | NRP | 25.0 | 29.7 | 249.0 | 3.47e-66 |
| WP\_043882190.1 | non-ribosomal\_peptide\_synthetase | BGC0001728 | NRP+Polyketide | 25.0 | 29.3 | 253.0 | 3.51e-66 |
| AHI59109.1 | locillomycin\_synthase\_B | BGC0001005 | NRP+Polyketide | 23.0 | 30.0 | 252.0 | 3.83e-66 |
| BAP16697.1 | nonribosomal\_peptide\_synthetase | BGC0000376 | NRP | 27.0 | 26.8 | 250.0 | 4.11e-66 |
| QYC40289.1 | A50926\_NRPS,\_modules\_4-5-6 | BGC0002344 | NRP | 26.0 | 29.1 | 252.0 | 5.12e-66 |
| BAB69700.1 | iturin\_A\_synthetase\_C | BGC0001098 | NRP+Polyketide | 24.0 | 30.4 | 251.0 | 5.17e-66 |
| ORC16617.1 | hypothetical\_protein | BGC0001341 | NRP | 24.0 | 37.9 | 251.0 | 5.23e-66 |
| AAO62587.1 | peptide\_sythetase | BGC0001016 | NRP+Polyketide | 24.0 | 29.6 | 251.0 | 5.51e-66 |
| CCP45168.1 | Peptide\_synthetase\_MbtE\_(peptide\_synthase) | BGC0001021 | NRP+Polyketide | 25.0 | 29.5 | 250.0 | 6.33e-66 |
| AHB82058.1 | non\_ribosomal\_peptide\_synthetase | BGC0001019 | NRP+Polyketide:Modular type I polyketide | 26.0 | 30.2 | 251.0 | 6.74e-66 |
| AYA44686.1 | icosalide\_NRPS | BGC0001833 | NRP:Lipopeptide | 26.0 | 29.7 | 251.0 | 7.21e-66 |
| UKO95756.1 | amino\_acid\_adenylation\_domain-containing\_protein | BGC0002632 | NRP | 23.0 | 29.9 | 250.0 | 7.5e-66 |
| ACO78736.1 | Non-ribosomal\_peptide\_synthase,\_PvdD-like\_protein | BGC0002433 | NRP | 24.0 | 30.9 | 251.0 | 9.51e-66 |
| APO47825.1 | non-ribosomal\_peptide\_synthetase | BGC0002653 | NRP | 26.0 | 22.9 | 246.0 | 1.02e-65 |
| CDN62030.1 | Peptide\_synthetase | BGC0001599 | NRP | 28.0 | 29.0 | 247.0 | 1.18e-65 |
| AAU39361.1 | lichenysin\_synthase\_LchAC | BGC0000381 | NRP | 25.0 | 29.4 | 248.0 | 1.5e-65 |
| ALG65319.1 | Cal17 | BGC0001297 | NRP | 25.0 | 30.2 | 249.0 | 1.95e-65 |
| ABM21572.1 | crpD | BGC0000975 | NRP+Polyketide | 23.0 | 31.1 | 249.0 | 2.34e-65 |
| BAW32324.1 | nonribosomal\_peptide\_synthetase | BGC0001630 | NRP+Polyketide | 25.0 | 28.9 | 249.0 | 2.42e-65 |
| AKJ15826.1 | peptide\_synthetase | BGC0002735 | Polyketide+NRP | 26.0 | 29.3 | 249.0 | 2.58e-65 |
| AKJ29411.1 | peptide\_synthetase | BGC0001608 | NRP | 26.0 | 29.4 | 249.0 | 2.63e-65 |
| QPI18729.1 | nonribosomal\_peptide\_synthetase | BGC0002125 | NRP:Cyclic depsipeptide | 26.0 | 26.2 | 249.0 | 2.95e-65 |
| WP\_100939442.1 | non-ribosomal\_peptide\_synthetase | BGC0002071 | NRP:Lipopeptide | 24.0 | 29.7 | 249.0 | 2.96e-65 |
| WP\_084702182.1 | non-ribosomal\_peptide\_synthetase | BGC0001211 | NRP | 27.0 | 29.2 | 249.0 | 3.03e-65 |
| AGI87382.1 | Peptide\_synthase | BGC0002358 | Polyketide | 26.0 | 24.3 | 249.0 | 3.09e-65 |
| AGI87384.1 | Peptide\_synthase | BGC0002358 | Polyketide | 25.0 | 32.1 | 249.0 | 3.38e-65 |
| AXA91301.1 | non-ribosomal\_peptide\_synthetase | BGC0002044 | NRP | 25.0 | 31.0 | 249.0 | 3.53e-65 |
| ATQ39428.1 | cyclosporin\_C\_synthetase | BGC0001565 | NRP | 25.0 | 28.8 | 249.0 | 4.34e-65 |
| ATY72525.1 | non-ribosomal\_peptide\_synthetase | BGC0001574 | NRP | 24.0 | 37.7 | 247.0 | 4.43e-65 |
| AKA59447.1 | non-ribosomal\_peptide\_synthetase | BGC0001203 | NRP+Polyketide | 29.0 | 22.8 | 249.0 | 4.62e-65 |
| QBA57735.1 | NRPS | BGC0002377 | NRP | 25.0 | 30.3 | 248.0 | 5.73e-65 |
| ABS74208.1 | fengycin\_synthetase\_B | BGC0001095 | NRP | 24.0 | 29.9 | 248.0 | 5.73e-65 |
| BAB69699.1 | iturin\_A\_synthetase\_B | BGC0001098 | NRP+Polyketide | 24.0 | 32.2 | 248.0 | 6.28e-65 |
| ACM79812.1 | ZmaQ | BGC0001059 | NRP+Polyketide | 25.0 | 24.5 | 248.0 | 6.39e-65 |
| AGM16413.1 | paenibacterin\_synthetase\_B | BGC0000400 | NRP | 24.0 | 32.4 | 248.0 | 6.57e-65 |
| APZ78704.1 | nonribosomal\_peptide\_synthetase | BGC0001419 | NRP:Cyclic depsipeptide+Polyketide:Iterative type I polyketide | 25.0 | 28.8 | 248.0 | 7.76e-65 |
| QNL14921.1 | AptB | BGC0002512 | NRP | 24.0 | 29.9 | 243.0 | 8.14e-65 |
| QLY89262.1 | pseudodesmin\_synthetase | BGC0002522 | NRP | 25.0 | 29.9 | 247.0 | 8.21e-65 |
| ABC36203.1 | Gonyol-Synthetase\_(NRPS-PKS\_hybrid) | BGC0001102 | NRP+Polyketide:Modular type I polyketide+Polyketide:Trans-AT type I polyketide | 28.0 | 23.7 | 247.0 | 1.05e-64 |
| MQQ32958.1 | amino\_acid\_adenylation\_domain-containing\_protein | BGC0002518 | NRP | 25.0 | 29.3 | 247.0 | 1.14e-64 |
| OKA09424.1 | non-ribosomal\_peptide\_synthetase | BGC0001459 | NRP:Glycopeptide | 26.0 | 28.9 | 247.0 | 1.28e-64 |
| CAE53352.1 | non-ribosomal\_peptide\_synthetase | BGC0000440 | NRP:Glycopeptide | 26.0 | 29.1 | 247.0 | 1.28e-64 |
| CDF96614.1 | NRPS | BGC0001149 | NRP:Lipopeptide+Saccharide:Hybrid/tailoring saccharide | 25.0 | 29.8 | 246.0 | 1.6e-64 |
| CZT62784.1 | Non-ribosomal\_peptide\_synthase,\_involved\_in\_Hassallidin\_biosynthesis | BGC0001614 | NRP | 26.0 | 30.2 | 247.0 | 1.61e-64 |
| AFR69334.1 | nonribosomal\_peptide\_synthetase\_SpiDE1 | BGC0001045 | NRP:Cyclic depsipeptide+Polyketide:Modular type I polyketide | 25.0 | 30.8 | 247.0 | 1.68e-64 |
| APZ78846.1 | nonribosomal\_peptide\_synthetase | BGC0001431 | NRP:Cyclic depsipeptide+Polyketide:Iterative type I polyketide | 27.0 | 29.1 | 247.0 | 1.74e-64 |
| OPB37950.1 | hypothetical\_protein | BGC0002206 | Polyketide | 27.0 | 23.1 | 246.0 | 1.76e-64 |
| QWP75305.1 | non-ribosomal\_peptide\_synthase | BGC0002126 | NRP:Cyclic depsipeptide | 26.0 | 29.7 | 246.0 | 3.29e-64 |
| KJY94240.1 | peptide\_synthetase | BGC0002691 | NRP | 24.0 | 29.6 | 246.0 | 3.58e-64 |
| AHF21229.1 | TriE | BGC0000449 | NRP | 25.0 | 29.8 | 246.0 | 3.62e-64 |
| CAD91212.1 | putative\_non-ribosomal\_peptide\_synthetase,\_modules\_4-6 | BGC0000289 | NRP:Glycopeptide+Saccharide:Hybrid/tailoring saccharide | 25.0 | 29.0 | 246.0 | 3.75e-64 |
| AEW31019.1 | plipastatin\_synthetase | BGC0000407 | NRP | 23.0 | 29.8 | 245.0 | 3.76e-64 |
| AIE77060.1 | peptide\_synthetase\_module\_7 | BGC0000418 | NRP | 26.0 | 29.8 | 244.0 | 4.44e-64 |
| ABC36450.1 | peptide\_synthetase-like\_protein | BGC0000386 | NRP:NRP siderophore | 25.0 | 33.2 | 245.0 | 4.45e-64 |
| QEO74905.1 | condensation\_domain-containing\_protein | BGC0002588 | Other | 27.0 | 23.1 | 245.0 | 6.05e-64 |
| UMP03490.1 | NmvB | BGC0002649 | NRP+Polyketide | 26.0 | 29.7 | 240.0 | 6.14e-64 |
| ABW17377.1 | PsoC | BGC0000411 | NRP | 25.0 | 29.4 | 245.0 | 6.22e-64 |
| AAM80536.1 | StaD | BGC0000290 | NRP:Glycopeptide | 26.0 | 29.2 | 244.0 | 7.64e-64 |
| CAC48361.1 | peptide\_synthetase | BGC0000311 | NRP | 26.0 | 28.5 | 244.0 | 8.39e-64 |
| CAJ77696.1 | MPS2\_protein | BGC0000363 | NRP | 24.0 | 39.0 | 244.0 | 8.48e-64 |
| AKC91857.1 | nonribosomal\_peptide\_synthetase | BGC0001414 | NRP | 25.0 | 29.2 | 242.0 | 8.63e-64 |
| AGJ76601.1 | HglE | BGC0000869 | Other | 28.0 | 21.4 | 243.0 | 9.65e-64 |
| MCC5036786.1 | amino\_acid\_adenylation\_domain-containing\_protein | BGC0002638 | NRP | 26.0 | 29.2 | 244.0 | 9.74e-64 |
| CAA11796.1 | PCZA363.5 | BGC0000322 | NRP | 26.0 | 30.7 | 243.0 | 1.01e-63 |
| AEI58866.1 | peptide\_synthetase | BGC0000455 | NRP | 25.0 | 28.4 | 244.0 | 1.1e-63 |
| APZ78716.1 | nonribosomal\_peptide\_synthetase | BGC0001420 | NRP:Cyclic depsipeptide+Polyketide:Iterative type I polyketide | 25.0 | 28.8 | 244.0 | 1.14e-63 |
| SDF67417.1 | amino\_acid\_adenylation\_domain-containing\_protein | BGC0002422 | NRP | 25.0 | 30.0 | 244.0 | 1.24e-63 |
| AAZ23075.1 | peptide\_synthetase | BGC0000291 | NRP | 25.0 | 29.1 | 244.0 | 1.25e-63 |
| ATO51563.1 | non-ribosomal\_peptide\_synthetase | BGC0001796 | NRP | 24.0 | 30.2 | 244.0 | 1.25e-63 |
| AJW76710.1 | DsaH | BGC0001196 | NRP | 24.0 | 30.0 | 243.0 | 1.29e-63 |
| ABI22133.1 | putative\_non-ribosomal\_peptide\_synthetase | BGC0000422 | NRP | 24.0 | 37.5 | 242.0 | 1.37e-63 |
| QDJ74275.1 | non-ribosomal\_peptide\_synthetase | BGC0002109 | NRP | 27.0 | 29.3 | 244.0 | 1.54e-63 |
| ABW17376.1 | PsoB | BGC0000411 | NRP | 25.0 | 29.1 | 244.0 | 1.69e-63 |
| EWS95122.1 | hypothetical\_protein | BGC0000306 | NRP:Lipopeptide | 26.0 | 30.3 | 243.0 | 1.75e-63 |
| AZM51141.1 | non-ribosomal\_peptide\_synthetase | BGC0002702 | NRP | 26.0 | 29.6 | 242.0 | 2.78e-63 |
| CAD91211.1 | putative\_non-ribosomal\_peptide\_synthetase,\_module\_7 | BGC0000289 | NRP:Glycopeptide+Saccharide:Hybrid/tailoring saccharide | 27.0 | 29.6 | 242.0 | 2.97e-63 |
| AAY93356.2 | non-ribosomal\_peptide\_synthetase\_PvdI | BGC0000413 | NRP | 26.0 | 29.7 | 243.0 | 3.09e-63 |
| TRX17524.1 | amino\_acid\_adenylation\_domain-containing\_protein | BGC0002329 | NRP | 26.0 | 29.9 | 243.0 | 3.51e-63 |
| QMS47798.1 | JesB | BGC0001629 | NRP:Lipopeptide | 26.0 | 29.5 | 243.0 | 3.53e-63 |
| AAX31557.1 | peptide\_synthetase\_1 | BGC0000336 | NRP | 25.0 | 29.6 | 243.0 | 3.59e-63 |
| DMA15\_34345 | non-ribosomal\_peptide\_synthetase | BGC0002314 | NRP | 26.0 | 29.4 | 240.0 | 4.05e-63 |
| AJD47484.1 | protein\_PvdD | BGC0002418 | NRP+Polyketide | 26.0 | 29.7 | 242.0 | 4.16e-63 |
| CAG15011.1 | peptide\_synthetase,\_module\_4-6 | BGC0000441 | NRP | 25.0 | 29.1 | 242.0 | 4.2e-63 |
| CAA11794.1 | PCZA363.3 | BGC0000322 | NRP | 25.0 | 31.7 | 242.0 | 4.88e-63 |
| AEP18655.1 | WAPS2 | BGC0000461 | NRP | 25.0 | 29.6 | 242.0 | 4.93e-63 |
| AEA30274.1 | peptide\_synthetase | BGC0000429 | Polyketide+NRP:Cyclic depsipeptide | 26.0 | 29.7 | 242.0 | 5.4e-63 |
| AJI44167.1 | long-chain-fatty-acid-CoA\_ligase | BGC0001193 | NRP | 28.0 | 24.5 | 241.0 | 5.6e-63 |
| CUX96954.1 | TmcG | BGC0001829 | NRP+Polyketide | 25.0 | 31.1 | 241.0 | 6.21e-63 |
| QMN69933.1 | PsoB | BGC0002521 | NRP | 25.0 | 32.1 | 241.0 | 8.43e-63 |
| CAE15497.1 |  | BGC0002286 | NRP | 26.0 | 30.0 | 241.0 | 1.03e-62 |
| QBC75021.1 | non-ribosomal\_peptide\_synthetase | BGC0001968 | NRP | 25.0 | 29.1 | 241.0 | 1.1e-62 |
| QCP68971.1 | VatR | BGC0002296 | NRP+Polyketide | 25.0 | 24.9 | 239.0 | 1.54e-62 |
| AGS77308.1 | NRPS\_module\_3 | BGC0001178 | NRP:Glycopeptide | 26.0 | 29.1 | 238.0 | 1.57e-62 |
| APZ78692.1 | nonribosomal\_peptide\_synthetase | BGC0001418 | NRP:Cyclic depsipeptide+Polyketide:Iterative type I polyketide | 25.0 | 28.8 | 240.0 | 2.17e-62 |
| AAM80537.1 | StaC | BGC0000290 | NRP:Glycopeptide | 25.0 | 29.1 | 239.0 | 2.75e-62 |
| CAC48362.1 | peptide\_synthetase | BGC0000311 | NRP | 27.0 | 29.2 | 238.0 | 3.37e-62 |
| QQZ01635.1 | PKS | BGC0002497 | Other | 28.0 | 23.8 | 239.0 | 3.42e-62 |
| CAG29032.1 | nonribosomal\_peptide\_synthetase\_(modules\_3\_to\_6) | BGC0001023 | NRP+Polyketide:Modular type I polyketide | 26.0 | 29.4 | 239.0 | 3.72e-62 |
| APZ78822.1 | nonribosomal\_peptide\_synthetase | BGC0001429 | NRP:Cyclic depsipeptide+Polyketide:Iterative type I polyketide | 26.0 | 29.4 | 239.0 | 3.72e-62 |
| ARO38317.1 | nonribosomal\_peptide\_synthetase | BGC0001560 | NRP+Polyketide | 24.0 | 29.5 | 239.0 | 4.1e-62 |
| ATY37592.1 | BogE | BGC0001532 | NRP | 24.0 | 29.2 | 238.0 | 4.64e-62 |
| WP\_010369430.1 | non-ribosomal\_peptide\_synthetase | BGC0000314 | Polyketide+NRP:Cyclic depsipeptide+Other:Aminocoumarin | 25.0 | 29.6 | 238.0 | 5.59e-62 |
| AAR87760.2 | ZmaK | BGC0001059 | NRP+Polyketide | 23.0 | 29.5 | 238.0 | 6.25e-62 |
| AGM16412.1 | paenibacterin\_synthetase\_A | BGC0000400 | NRP | 23.0 | 29.4 | 238.0 | 7.04e-62 |
| EME52989.1 | amino\_acid\_adenylation\_protein | BGC0001460 | NRP:Glycopeptide | 25.0 | 28.7 | 238.0 | 8.03e-62 |
| CCJ67638.1 | TaaC | BGC0000447 | NRP:Lipopeptide | 25.0 | 29.5 | 238.0 | 9.62e-62 |
| PHM26613.1 | pyoverdine\_synthetase\_D | BGC0001130 | NRP+Polyketide | 23.0 | 30.7 | 237.0 | 9.65e-62 |
| AFJ23825.1 | WLIP\_synthetase\_B | BGC0001838 | NRP | 25.0 | 29.7 | 238.0 | 1.07e-61 |
| AQZ69229.1 | hypothetical\_protein | BGC0001635 | NRP+Polyketide | 25.0 | 29.0 | 236.0 | 1.2e-61 |
| CAG15010.1 | peptide\_synthetase,\_module\_3 | BGC0000441 | NRP | 26.0 | 29.6 | 233.0 | 1.22e-61 |
| OLZ50885.1 | non-ribosomal\_peptide\_synthetase | BGC0001461 | NRP:Glycopeptide | 25.0 | 28.2 | 237.0 | 1.37e-61 |
| AHD05614.1 | putative\_non-ribosomal\_peptide\_ligase/\_polyketide\_synthase\_hybrid | BGC0001033 | NRP+Polyketide | 23.0 | 29.3 | 237.0 | 1.41e-61 |
| AFD30953.1 | CrmB | BGC0000966 | NRP+Polyketide | 24.0 | 29.6 | 233.0 | 1.48e-61 |
| WP\_050383082.1 | non-ribosomal\_peptide\_synthetase | BGC0001451 | NRP | 25.0 | 26.4 | 237.0 | 1.6e-61 |
| EME52988.1 | amino\_acid\_adenylation\_protein | BGC0001460 | NRP:Glycopeptide | 26.0 | 29.7 | 236.0 | 1.67e-61 |
| KPN93064.1 | NupB | BGC0001416 | NRP | 25.0 | 29.1 | 237.0 | 1.85e-61 |
| ABW17375.1 | PsoA | BGC0000411 | NRP | 23.0 | 29.8 | 236.0 | 2.02e-61 |
| WP\_013184322.1 | non-ribosomal\_peptide\_synthetase | BGC0001692 | NRP | 25.0 | 25.8 | 235.0 | 2.04e-61 |
| RSO11555.1 | non-ribosomal\_peptide\_synthetase | BGC0002637 | NRP | 25.0 | 30.1 | 235.0 | 2.06e-61 |
| ACC81022.1 | non-ribosomal\_peptide\_synthetase | BGC0001479 | NRP | 24.0 | 30.2 | 233.0 | 2.41e-61 |
| PHM26606.1 | malonyl\_CoA-acyl\_carrier\_protein\_transacylase | BGC0001130 | NRP+Polyketide | 25.0 | 24.5 | 236.0 | 2.5e-61 |
| QDQ83032.1 | amino\_acid\_adenylation\_domain-containing\_protein | BGC0002564 | NRP | 25.0 | 29.2 | 236.0 | 2.56e-61 |
| ABE35422.1 | Non-ribosomal\_peptide\_synthase | BGC0002421 | NRP | 25.0 | 32.6 | 236.0 | 2.75e-61 |
| OKA09425.1 | non-ribosomal\_peptide\_synthetase | BGC0001459 | NRP:Glycopeptide | 26.0 | 29.5 | 235.0 | 2.93e-61 |
| extra\_gene | NRPS/PKS | BGC0002095 | NRP | 24.0 | 33.4 | 236.0 | 3.02e-61 |
| EOY45602.1 | Adenylation\_and\_reductase\_domains\_containing\_protein | BGC0001168 | NRP | 27.0 | 26.6 | 231.0 | 3.57e-61 |
| AEG64696.1 | LpmB | BGC0000379 | NRP | 25.0 | 30.0 | 236.0 | 3.61e-61 |
| WP\_050383084.1 | non-ribosomal\_peptide\_synthetase | BGC0001451 | NRP | 25.0 | 29.0 | 235.0 | 4.06e-61 |
| BAH04162.1 | trsJ | BGC0000450 | NRP | 26.0 | 29.3 | 235.0 | 4.25e-61 |
| AHZ34238.1 | CipA | BGC0001389 | NRP | 25.0 | 29.2 | 236.0 | 4.28e-61 |
| APZ78852.1 | polyketide\_synthase | BGC0001432 | NRP:Cyclic depsipeptide+Polyketide:Iterative type I polyketide | 26.0 | 23.9 | 234.0 | 4.46e-61 |
| CAE53351.1 | non-ribosomal\_peptide\_synthetase | BGC0000440 | NRP:Glycopeptide | 26.0 | 29.6 | 231.0 | 5.02e-61 |
| AYJ71721.1 | non-ribosomal\_peptide\_synthetase | BGC0001942 | NRP+Polyketide | 24.0 | 30.1 | 235.0 | 5.04e-61 |
| AYA22334.1 | KerC | BGC0001955 | NRP | 25.0 | 28.2 | 235.0 | 5.26e-61 |
| AHB38497.1 | non-ribosomal\_peptide\_synthetase | BGC0000346 | NRP+Polyketide:Modular type I polyketide | 28.0 | 23.9 | 235.0 | 7.39e-61 |
| ACG60782.1 | NRPS(C/A/PCP/C/A/PCP) | BGC0001058 | NRP:Glycopeptide+Polyketide:Modular type I polyketide+Saccharide:Hybrid/tailoring saccharide | 25.0 | 30.3 | 234.0 | 7.81e-61 |
| CAJ18237.2 | non-ribosomal\_peptide\_synthetase\_B | BGC0000354 | NRP | 25.0 | 29.4 | 234.0 | 8.01e-61 |
| CBJ82077.1 | hypothetical\_protein | BGC0001872 | Polyketide | 27.0 | 24.0 | 234.0 | 8.33e-61 |
| AGS77310.1 | NRPS\_module\_7 | BGC0001178 | NRP:Glycopeptide | 26.0 | 29.6 | 234.0 | 8.72e-61 |
| QRG35013.1 | NRPS | BGC0002378 | NRP | 25.0 | 30.2 | 234.0 | 1.04e-60 |
| AXF16146.1 | non-ribosomal\_peptide\_synthetase | BGC0002563 | NRP | 25.0 | 28.9 | 234.0 | 1.3e-60 |
| AHZ20773.1 | non-ribosomal\_peptide\_synthase | BGC0000369 | NRP+Saccharide:Hybrid/tailoring saccharide | 23.0 | 29.7 | 234.0 | 1.35e-60 |
| CAE53353.1 | non-ribosomal\_peptide\_synthetase | BGC0000440 | NRP:Glycopeptide | 25.0 | 30.8 | 233.0 | 1.49e-60 |
| CCJ67639.1 | TaaD | BGC0000447 | NRP:Lipopeptide | 25.0 | 30.3 | 233.0 | 1.85e-60 |
| AIE77058.1 | peptide\_synthetase\_module\_3 | BGC0000418 | NRP | 25.0 | 30.0 | 229.0 | 1.88e-60 |
| CAG15012.1 | peptide\_synthetase,\_module\_7 | BGC0000441 | NRP | 25.0 | 30.7 | 233.0 | 1.95e-60 |
| simA |  | BGC0000334 | NRP | 25.0 | 26.1 | 234.0 | 1.96e-60 |
| ADQ55475.1 | NRPS | BGC0000350 | NRP:Beta-lactam | 23.0 | 32.5 | 232.0 | 1.99e-60 |
| AAY91420.2 | non-ribosomal\_peptide\_synthetase\_OfaB | BGC0000399 | NRP:Cyclic depsipeptide | 25.0 | 30.2 | 233.0 | 2.06e-60 |
| AGZ15458.1 | putative\_non-ribosomal\_peptide\_synthetase | BGC0001036 | NRP+Polyketide | 25.0 | 29.8 | 233.0 | 2.08e-60 |
| MCC5036785.1 | amino\_acid\_adenylation\_domain-containing\_protein | BGC0002638 | NRP | 26.0 | 29.4 | 233.0 | 2.75e-60 |
| AZM50111.1 | non-ribosomal\_peptide\_synthetase | BGC0002702 | NRP | 26.0 | 28.4 | 233.0 | 3.59e-60 |
| BAV56271.1 |  | BGC0001657 | NRP | 25.0 | 29.5 | 233.0 | 3.91e-60 |
| BCJ07532.1 | hypothetical\_protein | BGC0002379 | NRP | 25.0 | 28.6 | 232.0 | 5.01e-60 |
| QUF98525.1 | non-ribosomal\_peptide\_synthetase | BGC0002582 | NRP | 28.0 | 24.6 | 228.0 | 5.3e-60 |
| BAF50711.1 | non\_ribosomal\_peptide\_synthetase\_for\_virginiamycin\_S | BGC0001116 | NRP+Polyketide | 25.0 | 31.0 | 231.0 | 6.23e-60 |
| QED88054.1 | nonribosomal\_peptide\_synthetase | BGC0001967 | NRP+Polyketide | 24.0 | 30.0 | 231.0 | 8.06e-60 |
| AAG02355.1 | peptide\_synthetase\_NRPS9-8 | BGC0000963 | NRP:Glycopeptide+Polyketide:Modular type I polyketide+Saccharide:Hybrid/tailoring saccharide | 26.0 | 29.9 | 231.0 | 8.97e-60 |
| MCC5036784.1 | amino\_acid\_adenylation\_domain-containing\_protein | BGC0002638 | NRP | 25.0 | 30.4 | 230.0 | 9.23e-60 |
| AXA94654.1 | hypothetical\_protein | BGC0002044 | NRP | 25.0 | 30.2 | 228.0 | 9.6e-60 |
| AEI58867.1 | peptide\_synthetase | BGC0000455 | NRP | 26.0 | 29.5 | 230.0 | 9.82e-60 |
| CAY48788.1 | putative\_non-ribosomal\_peptide\_synthetase | BGC0001312 | NRP | 25.0 | 29.5 | 231.0 | 1.03e-59 |
| CBL93718.1 | NRPS\_didomain\_PCP-C | BGC0000360 | NRP | 25.0 | 31.2 | 230.0 | 1.29e-59 |
| QPI18728.1 | nonribosomal\_peptide\_synthetase | BGC0002125 | NRP:Cyclic depsipeptide | 25.0 | 29.6 | 230.0 | 1.34e-59 |
| AWI62626.1 | nonribosomal\_peptide\_synthetase | BGC0001822 | NRP | 26.0 | 30.1 | 231.0 | 1.4e-59 |
| QDJ74273.1 | non-ribosomal\_peptide\_synthetase | BGC0002109 | NRP | 24.0 | 30.1 | 231.0 | 1.45e-59 |
| QTT72101.1 | non-ribosomal\_peptide\_synthetase | BGC0002350 | NRP+Polyketide+Saccharide | 26.0 | 29.9 | 230.0 | 1.52e-59 |
| AKJ29412.1 | peptide\_synthetase | BGC0001608 | NRP | 26.0 | 28.7 | 230.0 | 1.92e-59 |
| AFJ14793.1 | PlpD | BGC0000403 | NRP | 23.0 | 29.8 | 229.0 | 1.98e-59 |
| DAB41476.1 | nonribosomal\_peptide\_synthetase | BGC0001230 | NRP:Cyclic depsipeptide+Polyketide:Modular type I polyketide | 25.0 | 29.1 | 228.0 | 2.33e-59 |
| AAK81827.1 | peptide\_synthetase | BGC0000326 | NRP | 25.0 | 29.7 | 229.0 | 2.64e-59 |
| CAF32362.1 | putative\_non-ribosomal\_peptide\_synthetase | BGC0000712 | Saccharide | 27.0 | 24.5 | 226.0 | 2.74e-59 |
| AEW95634.1 | non-ribosomal\_peptide\_synthetase | BGC0002697 | NRP+Polyketide | 26.0 | 29.4 | 229.0 | 2.88e-59 |
| AIG79242.1 | Non-ribosomal\_peptide\_synthetase | BGC0000419 | Saccharide+NRP:Glycopeptide | 26.0 | 29.0 | 226.0 | 3.21e-59 |
| BAW32333.1 | nonribosomal\_peptide\_synthetase | BGC0001631 | NRP+Polyketide | 24.0 | 32.8 | 229.0 | 3.39e-59 |
| QPB41096.1 | non-ribosomal\_peptide\_synthetase | BGC0002503 | NRP+Polyketide | 26.0 | 30.1 | 229.0 | 4.19e-59 |
| QRN75755.1 | Amino\_acid\_adenylation\_domain\_protein | BGC0002114 | NRP+Polyketide | 25.0 | 29.8 | 228.0 | 4.43e-59 |
| AHZ34232.1 | CifA | BGC0000323 | NRP:Lipopeptide | 24.0 | 29.5 | 229.0 | 4.52e-59 |
| AAK81825.1 | peptide\_synthetase | BGC0000326 | NRP | 25.0 | 29.2 | 228.0 | 4.64e-59 |
| QDF82255.1 | non-ribosomal\_peptide\_synthetase | BGC0001980 | NRP | 25.0 | 29.5 | 229.0 | 5.15e-59 |
| RSO11554.1 | non-ribosomal\_peptide\_synthetase | BGC0002637 | NRP | 25.0 | 33.2 | 229.0 | 5.36e-59 |
| ABP57748.1 | DepD | BGC0000993 | NRP:Cyclic depsipeptide+Polyketide:Modular type I polyketide | 26.0 | 29.0 | 228.0 | 5.81e-59 |
| QMS47799.1 | JesA | BGC0001629 | NRP:Lipopeptide | 25.0 | 29.1 | 228.0 | 7.16e-59 |
| KUM80514.1 | hypothetical\_protein | BGC0001562 | NRP | 25.0 | 28.7 | 224.0 | 8.09e-59 |
| ACZ55943.1 | non-ribosomal\_peptide\_synthetase | BGC0000302 | NRP | 23.0 | 30.4 | 224.0 | 8.56e-59 |
| BAX64247.1 | NRPS | BGC0001623 | NRP+Polyketide | 25.0 | 29.8 | 227.0 | 1.13e-58 |
| AIZ66879.1 | nonribosomal\_peptide\_synthetase | BGC0002666 | NRP+Alkaloid | 25.0 | 32.2 | 227.0 | 1.14e-58 |
| CAL80824.1 | NRPS\_module\_protein | BGC0000997 | NRP+Polyketide | 25.0 | 30.4 | 224.0 | 1.24e-58 |
| WP\_064118561.1 | non-ribosomal\_peptide\_synthetase | BGC0001509 | NRP | 26.0 | 29.8 | 227.0 | 1.31e-58 |
| APZ78834.1 | nonribosomal\_peptide\_synthetase | BGC0001430 | NRP:Cyclic depsipeptide+Polyketide:Iterative type I polyketide | 25.0 | 29.8 | 227.0 | 1.52e-58 |
| AKA59436.1 | non-ribosomal\_peptide\_synthetase | BGC0001202 | NRP+Polyketide | 24.0 | 34.3 | 227.0 | 1.52e-58 |
| AAZ55899.1 | amino\_acid\_adenylation | BGC0000359 | NRP | 25.0 | 30.0 | 224.0 | 1.55e-58 |
| WP\_006051170.1 | non-ribosomal\_peptide\_synthetase | BGC0001999 | NRP | 26.0 | 29.9 | 227.0 | 1.61e-58 |
| QMN69932.1 | PsoA | BGC0002521 | NRP | 24.0 | 29.7 | 226.0 | 1.7e-58 |
| ATY37609.1 | BreD | BGC0001536 | NRP | 26.0 | 23.3 | 227.0 | 1.74e-58 |
| CAK15815.1 | putative\_non\_ribosomal\_peptide\_synthetase | BGC0000344 | NRP | 24.0 | 28.8 | 227.0 | 1.75e-58 |
| WP\_004571779.1 | non-ribosomal\_peptide\_synthetase | BGC0001760 | NRP | 26.0 | 29.8 | 223.0 | 2.18e-58 |
| AME18003.1 | enediyne\_polyketie\_synthase | BGC0001378 | Polyketide:Enediyne type I polyketide | 27.0 | 25.2 | 226.0 | 2.22e-58 |
| ACU36654.1 | amino\_acid\_adenylation\_domain\_protein | BGC0000392 | NRP | 26.0 | 29.1 | 226.0 | 2.35e-58 |
| CCM44330.1 | Polyketide\_synthase | BGC0001056 | NRP+Polyketide:Modular type I polyketide+Polyketide:PUFA synthase or related polyketide | 25.0 | 24.1 | 226.0 | 3.09e-58 |
| QEO75074.1 | condensation\_domain-containing\_protein | BGC0002079 | NRP:Cyclic depsipeptide | 26.0 | 26.2 | 224.0 | 3.2e-58 |
| AOA33123.1 | Nonribosomal\_peptide\_synthetase | BGC0001346 | NRP:Cyclic depsipeptide | 24.0 | 30.6 | 226.0 | 3.6e-58 |
| QBG38784.1 | Atr23 | BGC0001975 | NRP | 25.0 | 29.7 | 226.0 | 4.56e-58 |
| ABV79986.1 | ApnB | BGC0000301 | NRP | 24.0 | 29.7 | 222.0 | 4.91e-58 |
| UMM61373.1 | Tsk12 | BGC0002661 | NRP | 25.0 | 29.8 | 223.0 | 5.75e-58 |
| QGQ63519.1 | nonribosomal\_peptide\_synthetase\_modules\_B | BGC0002548 | NRP | 25.0 | 29.1 | 225.0 | 5.76e-58 |
| BAX89998.1 | Non-ribosomal\_peptide\_synthetase | BGC0001628 | NRP | 26.0 | 29.2 | 225.0 | 5.86e-58 |
| BAV56270.1 |  | BGC0001657 | NRP | 25.0 | 29.3 | 225.0 | 6.74e-58 |
| ACY06292.1 | modular\_polyketide\_synthase | BGC0001042 | NRP+Polyketide | 27.0 | 23.4 | 224.0 | 6.86e-58 |
| CBJ90082.1 | Non\_Ribosomal\_peptide\_synthetase\_(-succinylbenzoate--CoA\_ligase) | BGC0001132 | NRP | 26.0 | 28.9 | 225.0 | 7.42e-58 |
| ARU08073.1 | mlcK | BGC0001448 | NRP:Lipopeptide:Ca+-dependent lipopeptide | 25.0 | 29.2 | 224.0 | 8.54e-58 |
| CAQ71829.1 | non\_ribosomal\_peptide\_synthase,\_antibiotic\_synthesis;\_contains\_4\_condensation\_domains,\_3\_AMP-acid\_ligases\_II\_domains,\_3\_PP-binding,\_Phosphopantetheine\_attachment\_site\_and\_a\_putative\_thioesterase\_domain | BGC0001189 | NRP | 25.0 | 28.7 | 224.0 | 9.55e-58 |
| AOA33122.1 | Nonribosomal\_peptide\_synthetase | BGC0001346 | NRP:Cyclic depsipeptide | 24.0 | 29.5 | 224.0 | 9.85e-58 |
| PVC99865.1 | non-ribosomal\_peptide\_synthetase | BGC0002100 | NRP+Other | 24.0 | 29.5 | 224.0 | 1.22e-57 |
| OLZ52457.1 | non-ribosomal\_peptide\_synthetase | BGC0001462 | NRP:Glycopeptide | 24.0 | 29.0 | 224.0 | 1.26e-57 |
| BAJ19066.1 | L-lysine\_activating\_non-ribosomal\_peptide\_synthetase | BGC0000288 | NRP | 24.0 | 29.9 | 221.0 | 1.33e-57 |
| ADJ63842.1 | Serobactin\_synthetase | BGC0000424 | NRP:NRP siderophore | 25.0 | 30.8 | 224.0 | 1.5e-57 |
| AHZ34242.1 | CipE | BGC0001389 | NRP | 26.0 | 29.3 | 223.0 | 1.92e-57 |
| CZT62792.1 | non-ribosomal\_peptide\_synthase\_involved\_in\_Hassallidin\_biosynthesis | BGC0001614 | NRP | 24.0 | 26.5 | 223.0 | 1.95e-57 |
| QTT72097.1 | amino\_acid\_adenylation\_domain-containing\_protein | BGC0002350 | NRP+Polyketide+Saccharide | 25.0 | 33.7 | 223.0 | 2.32e-57 |
| AFJ14795.1 | PlpF | BGC0000403 | NRP | 26.0 | 21.7 | 220.0 | 2.34e-57 |
| NKI69295.1 | amino\_acid\_adenylation\_domain-containing\_protein | BGC0002408 | NRP | 25.0 | 29.9 | 223.0 | 2.56e-57 |
| AVI26392.1 | putative\_nonribosomal\_peptide\_synthase | BGC0001800 | NRP+Polyketide | 25.0 | 30.6 | 222.0 | 2.74e-57 |
| CAD55498.1 | CDA\_peptide\_synthetase\_III\_(CdaPs3) | BGC0000315 | NRP:Lipopeptide:Ca+-dependent lipopeptide | 25.0 | 29.9 | 223.0 | 2.82e-57 |
| AFH75320.1 | nonribosomal\_peptide\_synthetase | BGC0000425 | NRP:Cyclic depsipeptide | 26.0 | 28.7 | 223.0 | 3.18e-57 |
| CAD91221.1 | putative\_non-ribosomal\_peptide\_synthetase,\_module\_3 | BGC0000289 | NRP:Glycopeptide+Saccharide:Hybrid/tailoring saccharide | 25.0 | 30.4 | 219.0 | 3.18e-57 |
| AEG64697.1 | LpmC | BGC0000379 | NRP | 25.0 | 29.1 | 223.0 | 3.21e-57 |
| AZM57023.1 | non-ribosomal\_peptide\_synthetase | BGC0002314 | NRP | 25.0 | 28.1 | 221.0 | 3.61e-57 |
| WP\_080679150.1 | non-ribosomal\_peptide\_synthetase | BGC0001228 | NRP:Cyclic depsipeptide | 25.0 | 29.4 | 222.0 | 3.87e-57 |
| AET98906.1 | putative\_non-ribosomal\_peptide\_synthetase | BGC0000415 | NRP | 25.0 | 29.4 | 222.0 | 3.95e-57 |
| ABA73955.1 | putative\_non-ribosomal\_peptide\_synthetase | BGC0001842 | NRP:Lipopeptide | 25.0 | 30.0 | 223.0 | 4.01e-57 |
| AHH53506.1 | non-ribosomal\_peptide\_synthetase | BGC0000439 | NRP:Lipopeptide:Ca+-dependent lipopeptide | 26.0 | 30.2 | 223.0 | 4.06e-57 |
| AHZ34239.1 | CipB | BGC0001389 | NRP | 25.0 | 28.7 | 222.0 | 4.35e-57 |
| ctg1\_orf1265 |  | BGC0001752 | NRP | 27.0 | 29.1 | 222.0 | 4.41e-57 |
| AZM58102.1 | non-ribosomal\_peptide\_synthetase | BGC0002314 | NRP | 25.0 | 30.0 | 222.0 | 4.49e-57 |
| BAC67535.1 | arthrofactin\_synthetase\_B | BGC0000305 | NRP:Lipopeptide | 25.0 | 29.0 | 222.0 | 4.93e-57 |
| CAJ34374.1 | NRPS\_protein | BGC0000445 | NRP:Cyclic depsipeptide | 24.0 | 29.3 | 222.0 | 5.08e-57 |
| QGQ63520.1 | nonribosomal\_peptide\_synthetase\_modules\_C | BGC0002548 | NRP | 25.0 | 28.9 | 221.0 | 9.06e-57 |
| QCQ67879.1 | non-ribosomal\_peptide\_synthetase | BGC0002297 | NRP+Polyketide | 24.0 | 31.5 | 221.0 | 9.09e-57 |
| AEP18656.1 | WAPS1 | BGC0000461 | NRP | 25.0 | 30.4 | 221.0 | 9.84e-57 |
| EAL89049.1 | nonribosomal\_peptide\_synthetase | BGC0000355 | NRP | 24.0 | 30.4 | 221.0 | 1.07e-56 |
| QKM21620.1 | non-ribosomal\_peptide\_synthetase | BGC0002351 | NRP | 25.0 | 29.2 | 221.0 | 1.19e-56 |
| AFH75321.1 | nonribosomal\_peptide\_synthetase | BGC0000425 | NRP:Cyclic depsipeptide | 25.0 | 29.4 | 221.0 | 1.22e-56 |
| ALV82388.1 | CDA\_peptide\_synthetase\_III | BGC0001370 | NRP | 25.0 | 29.8 | 220.0 | 1.41e-56 |
| QKM21619.1 | non-ribosomal\_peptide\_synthetase | BGC0002351 | NRP | 25.0 | 29.9 | 220.0 | 1.68e-56 |
| UMM61372.1 | Tsk11 | BGC0002661 | NRP | 24.0 | 30.2 | 219.0 | 1.86e-56 |
| ABM34277.1 | amino\_acid\_adenylation\_domain\_protein | BGC0002419 | NRP+Polyketide | 25.0 | 29.2 | 219.0 | 2.59e-56 |
| AAY37654.1 | Amino\_acid\_adenylation | BGC0000437 | NRP | 25.0 | 29.3 | 220.0 | 2.62e-56 |
| APU91750.1 | Non-Ribosomal\_Peptide\_Synthetase | BGC0001806 | NRP | 25.0 | 29.3 | 220.0 | 2.97e-56 |
| KZM69124.1 | non-ribosomal\_peptide\_synthetase | BGC0002352 | Other | 26.0 | 31.1 | 218.0 | 3.27e-56 |
| AGN74876.1 | nonribosomal\_peptide\_synthetase | BGC0000459 | NRP:Cyclic depsipeptide+Polyketide:Trans-AT type I polyketide | 25.0 | 26.5 | 219.0 | 3.37e-56 |
| WP\_051872436.1 | non-ribosomal\_peptide\_synthetase | BGC0001771 | NRP | 23.0 | 30.2 | 216.0 | 3.58e-56 |
| ABU70377.1 | hypothetical\_protein | BGC0001890 | NRP | 24.0 | 29.7 | 218.0 | 3.68e-56 |
| RSO11553.1 | non-ribosomal\_peptide\_synthetase | BGC0002637 | NRP | 24.0 | 30.2 | 219.0 | 3.84e-56 |
| QWM97319.1 | non-ribosomal\_peptide\_synthetase | BGC0002384 | NRP | 24.0 | 29.5 | 219.0 | 4.91e-56 |
| QWT72293.1 | non-ribosomal\_peptide\_synthetase | BGC0002430 | NRP+Saccharide | 25.0 | 30.3 | 218.0 | 5.43e-56 |
| AAG02359.1 | peptide\_synthetase\_NRPS5-4-3 | BGC0000963 | NRP:Glycopeptide+Polyketide:Modular type I polyketide+Saccharide:Hybrid/tailoring saccharide | 25.0 | 33.6 | 218.0 | 5.83e-56 |
| CCJ67637.1 | TaaB | BGC0000447 | NRP:Lipopeptide | 24.0 | 29.9 | 218.0 | 6.49e-56 |
| AAM80538.1 | StaB | BGC0000290 | NRP:Glycopeptide | 26.0 | 29.5 | 217.0 | 6.71e-56 |
| AGE11899.1 | nonribosomal\_peptide\_synthetase | BGC0000366 | NRP | 26.0 | 30.0 | 218.0 | 6.97e-56 |
| OKJ61999.1 | peptide\_synthetase | BGC0002147 | NRP | 24.0 | 29.8 | 218.0 | 7.12e-56 |
| AJV88376.1 | MfnD | BGC0001214 | NRP | 24.0 | 30.7 | 217.0 | 7.15e-56 |
| CUX79062.1 | Octapeptin\_synthase\_subunit\_C | BGC0001715 | NRP | 25.0 | 24.4 | 215.0 | 7.84e-56 |
| ADH01485.1 | putative\_mixed\_polyketide\_synthase/non-ribosomal\_peptide\_synthetase | BGC0000995 | NRP+Polyketide | 25.0 | 30.0 | 217.0 | 8.97e-56 |
| AZM50110.1 | non-ribosomal\_peptide\_synthetase | BGC0002702 | NRP | 25.0 | 29.3 | 217.0 | 9.45e-56 |
| ABP57749.1 | DepE | BGC0000993 | NRP:Cyclic depsipeptide+Polyketide:Modular type I polyketide | 25.0 | 30.0 | 217.0 | 1.28e-55 |
| AAO72424.1 | syringopeptin\_synthetase\_B | BGC0000438 | NRP | 25.0 | 29.3 | 218.0 | 1.31e-55 |
| BAX89999.1 | Non-ribosomal\_peptide\_synthetase | BGC0001628 | NRP | 26.0 | 29.8 | 217.0 | 1.65e-55 |
| ctg4\_5 |  | BGC0002017 | NRP | 25.0 | 30.2 | 217.0 | 1.77e-55 |
| KPN93063.1 | NupA | BGC0001416 | NRP | 25.0 | 30.1 | 217.0 | 2.46e-55 |
| AQZ69227.1 | hypothetical\_protein | BGC0001635 | NRP+Polyketide | 27.0 | 29.2 | 215.0 | 2.52e-55 |
| AHZ34240.1 | CipC | BGC0001389 | NRP | 25.0 | 29.1 | 215.0 | 2.78e-55 |
| ACJ04424.1 | aureobasidin\_A1\_biosynthesis\_complex | BGC0000307 | NRP | 25.0 | 26.1 | 216.0 | 3.28e-55 |
| BCJ07529.1 | hypothetical\_protein | BGC0002379 | NRP | 25.0 | 22.2 | 216.0 | 3.32e-55 |
| QNL34618.1 | SteC | BGC0002092 | NRP:Cyclic depsipeptide | 25.0 | 29.9 | 216.0 | 3.9e-55 |
| CAR51994.1 | ornibactin\_biosynthesis\_non-ribosomal\_peptide\_synthase | BGC0002569 | NRP | 25.0 | 28.9 | 216.0 | 4.22e-55 |
| AEC14349.1 | nonribosomal\_peptide\_synthetase | BGC0000377 | NRP | 25.0 | 24.2 | 211.0 | 5.74e-55 |
| ADG27359.1 | peptide\_synthetase | BGC0000296 | NRP | 25.0 | 30.7 | 215.0 | 6.12e-55 |
| RSO11556.1 | non-ribosomal\_peptide\_synthetase | BGC0002637 | NRP | 25.0 | 30.0 | 215.0 | 7.11e-55 |
| ADG27358.1 | peptide\_synthetase | BGC0000296 | NRP | 24.0 | 30.2 | 214.0 | 1.11e-54 |
| WP\_020993844.1 | non-ribosomal\_peptide\_synthetase | BGC0001575 | NRP | 23.0 | 29.0 | 214.0 | 1.19e-54 |
| ANS62967.1 | non-ribosomal\_peptide\_synthase/amino\_acid\_adenylation\_enzyme | BGC0001567 | NRP | 25.0 | 29.9 | 214.0 | 1.43e-54 |
| CBG75492.1 | putative\_NRPS/siderophore\_biosynthesis\_protein | BGC0000423 | NRP | 25.0 | 33.3 | 214.0 | 1.51e-54 |
| AEH41794.1 | HrmP | BGC0000374 | NRP:Cyclic depsipeptide | 25.0 | 29.4 | 214.0 | 1.67e-54 |
| AXB34356.1 | non-ribosomal\_peptide\_synthetase | BGC0002415 | NRP | 24.0 | 30.4 | 213.0 | 1.98e-54 |
| ABL74936.1 | NRPS | BGC0001048 | NRP:Glycopeptide+Polyketide:Modular type I polyketide+Saccharide:Hybrid/tailoring saccharide | 24.0 | 30.2 | 213.0 | 2.13e-54 |
| ABA70582.1 | alpha-aminoadypil-cysteinyl-valine\_synthetase | BGC0000404 | NRP | 23.0 | 32.8 | 213.0 | 2.25e-54 |
| AOA33121.1 | Nonribosomal\_peptide\_synthetase | BGC0001346 | NRP:Cyclic depsipeptide | 25.0 | 30.1 | 213.0 | 2.8e-54 |
| AHZ20781.1 | non-ribosomal\_peptide\_synthase | BGC0000369 | NRP+Saccharide:Hybrid/tailoring saccharide | 23.0 | 29.8 | 213.0 | 2.81e-54 |
| AHZ34233.1 | CifB | BGC0000323 | NRP:Lipopeptide | 24.0 | 28.2 | 213.0 | 3.32e-54 |
| QYC40288.1 | A50926\_NRPS,\_module\_3 | BGC0002344 | NRP | 25.0 | 29.1 | 210.0 | 3.51e-54 |
| AAY42398.1 | Nonribosomal\_peptide\_synthetase | BGC0001000 | NRP:Lipopeptide+Polyketide:Modular type I polyketide | 24.0 | 27.5 | 213.0 | 3.91e-54 |
| ALV86868.1 | Tlo22 | BGC0001406 | NRP | 25.0 | 29.7 | 212.0 | 4.01e-54 |
| QLY89263.1 | pseudodesmin\_synthetase | BGC0002522 | NRP | 27.0 | 22.7 | 213.0 | 4.04e-54 |
| ABR12615.1 | ACV\_synthetase | BGC0000405 | NRP:Beta-lactam | 23.0 | 32.8 | 212.0 | 5.05e-54 |
| WP\_010369428.1 | non-ribosomal\_peptide\_synthetase | BGC0000314 | Polyketide+NRP:Cyclic depsipeptide+Other:Aminocoumarin | 23.0 | 29.6 | 212.0 | 5.55e-54 |
| AYA22335.1 | KerB | BGC0001955 | NRP | 25.0 | 29.1 | 209.0 | 5.87e-54 |
| AAD44233.1 | PstA | BGC0000362 | NRP | 25.0 | 29.0 | 212.0 | 6.34e-54 |
| KIA75688.1 | nonribosomal\_peptide\_synthase | BGC0002242 | NRP | 25.0 | 29.6 | 211.0 | 8.72e-54 |
| CAQ46279.1 | putative\_enterobactin\_synthetase\_component\_F | BGC0002689 | NRP | 25.0 | 29.5 | 209.0 | 1.1e-53 |
| QNL34617.1 | SteB | BGC0002092 | NRP:Cyclic depsipeptide | 23.0 | 29.3 | 211.0 | 1.18e-53 |
| AFH75329.1 | nonribosomal\_peptide\_synthetase | BGC0000398 | NRP:Cyclic depsipeptide | 24.0 | 29.1 | 211.0 | 1.18e-53 |
| AGN74886.1 | nonribosomal\_peptide\_synthetase | BGC0000459 | NRP:Cyclic depsipeptide+Polyketide:Trans-AT type I polyketide | 25.0 | 29.1 | 210.0 | 1.28e-53 |
| KFL51886.1 | amino\_acid\_adenylation\_protein | BGC0001711 | NRP+Polyketide | 25.0 | 30.3 | 211.0 | 1.34e-53 |
| OAL11435.1 | non-ribosomal\_peptide\_synthetase | BGC0001570 | NRP | 25.0 | 30.5 | 208.0 | 1.41e-53 |
| AQZ69228.1 | hypothetical\_protein | BGC0001635 | NRP+Polyketide | 25.0 | 29.2 | 211.0 | 1.45e-53 |
| BAI23334.1 | putative\_non-ribosomal\_peptide\_synthetase | BGC0000949 | NRP | 24.0 | 26.0 | 208.0 | 1.61e-53 |
| BS330\_28385 | non-ribosomal\_peptide\_synthetase | BGC0001461 | NRP:Glycopeptide | 26.0 | 28.0 | 210.0 | 1.8e-53 |
| AMK48228.1 | nonribosomal\_peptide\_synthetase | BGC0001351 | NRP | 25.0 | 29.4 | 207.0 | 2.4e-53 |
| BAC67534.2 | arthrofactin\_synthetase\_A | BGC0000305 | NRP:Lipopeptide | 24.0 | 29.0 | 209.0 | 2.41e-53 |
| CAN89638.1 | putative\_non-ribosomal\_peptide\_synthetase | BGC0001070 | NRP+Polyketide:Modular type I polyketide+Polyketide:Trans-AT type I polyketide | 25.0 | 29.9 | 207.0 | 2.77e-53 |
| CAM02313.1 | putative\_non-ribosomal\_peptide\_synthetase | BGC0000349 | NRP | 26.0 | 29.7 | 209.0 | 3.65e-53 |
| AYA22336.1 | KerA | BGC0001955 | NRP | 26.0 | 28.0 | 209.0 | 4.04e-53 |
| AAX31559.1 | peptide\_synthetase\_3 | BGC0000336 | NRP | 25.0 | 29.2 | 209.0 | 4.49e-53 |
| CCC55922.1 | putative\_non-ribosomal\_peptide\_synthetase | BGC0000973 | NRP+Polyketide:Modular type I polyketide | 25.0 | 29.7 | 206.0 | 4.98e-53 |
| QDJ74274.1 | non-ribosomal\_peptide\_synthetase | BGC0002109 | NRP | 25.0 | 26.8 | 205.0 | 8.84e-53 |
| QNL34616.1 | SteA | BGC0002092 | NRP:Cyclic depsipeptide | 25.0 | 29.8 | 207.0 | 1.59e-52 |
| AAO56328.1 | non-ribosomal\_peptide\_synthetase\_SyfA | BGC0000435 | NRP | 25.0 | 29.4 | 207.0 | 2.02e-52 |
| AFK57214.1 | DidC | BGC0000985 | Polyketide+NRP:Cyclic depsipeptide | 26.0 | 29.7 | 205.0 | 2.37e-52 |
| BCD33691.1 | non-ribosomal\_peptide\_synthetase | BGC0002448 | NRP | 24.0 | 29.4 | 205.0 | 3.01e-52 |
| WP\_099111429.1 | non-ribosomal\_peptide\_synthetase | BGC0001826 | NRP | 23.0 | 29.5 | 204.0 | 4.51e-52 |
| BCJ07533.1 | hypothetical\_protein | BGC0002379 | NRP | 25.0 | 29.8 | 205.0 | 5.32e-52 |
| UMM61371.1 | Tsk10 | BGC0002661 | NRP | 26.0 | 29.6 | 205.0 | 6.21e-52 |
| ABI22131.1 | putative\_non-ribosomal\_peptide\_synthetase | BGC0000422 | NRP | 24.0 | 32.1 | 204.0 | 6.94e-52 |
| CBF87069.1 | nonribosomal\_peptide\_synthase,\_putative\_(Eurofung) | BGC0001290 | NRP | 23.0 | 29.4 | 204.0 | 1.25e-51 |
| EPH46596.1 | putative\_Linear\_gramicidin\_synthase\_subunit\_C | BGC0001519 | NRP+Polyketide | 24.0 | 24.8 | 204.0 | 1.26e-51 |
| AAY91419.3 | non-ribosomal\_peptide\_synthetase\_OfaA | BGC0000399 | NRP:Cyclic depsipeptide | 25.0 | 29.6 | 203.0 | 1.78e-51 |
| ABS75103.1 | non-ribosomal\_peptide\_synthetase | BGC0002641 | NRP | 23.0 | 29.8 | 203.0 | 2.33e-51 |
| AFL68053.1 | amino\_acid\_adenylation\_enzyme/thioester\_reductase\_family\_protein | BGC0001524 | NRP+Polyketide | 23.0 | 29.6 | 202.0 | 3.15e-51 |
| QYI86762.1 | non-ribosomal\_peptide\_synthetase | BGC0002424 | NRP | 25.0 | 29.4 | 203.0 | 3.32e-51 |
| AHI59110.1 | locillomycin\_synthase\_C | BGC0001005 | NRP+Polyketide | 24.0 | 24.6 | 202.0 | 4.01e-51 |
| AAL15600.1 | SimH | BGC0000270 | Polyketide | 25.0 | 22.8 | 199.0 | 4.59e-51 |
| ctg1\_orf00001 |  | BGC0000901 | Other | 23.0 | 29.7 | 202.0 | 5.07e-51 |
| BAH23995.1 | nonribosomal\_peptide\_synthetase | BGC0000356 | NRP+Alkaloid | 22.0 | 33.1 | 202.0 | 5.41e-51 |
| CBJ90359.1 | putative\_Peptide\_synthetase | BGC0000465 | NRP | 25.0 | 21.8 | 201.0 | 6.43e-51 |
| AZM51139.1 | non-ribosomal\_peptide\_synthetase | BGC0002702 | NRP | 24.0 | 29.8 | 201.0 | 8.73e-51 |
| KJY85279.1 | long-chain\_fatty\_acid--CoA\_ligase | BGC0002491 | NRP | 24.0 | 36.2 | 200.0 | 1.27e-50 |
| MBE3202942.1 | non-ribosomal\_peptide\_synthetase | BGC0002410 | NRP | 25.0 | 29.5 | 200.0 | 1.31e-50 |
| AXG47007.1 | non-ribosomal\_peptide\_synthetase | BGC0000383 | NRP+Polyketide:Modular type I polyketide | 23.0 | 29.8 | 198.0 | 1.51e-50 |
| CCJ67645.1 | JagA | BGC0001127 | NRP | 24.0 | 29.8 | 200.0 | 1.53e-50 |
| BAX64246.1 | NRPS | BGC0001623 | NRP+Polyketide | 24.0 | 30.6 | 200.0 | 2.35e-50 |
| CAJ76292.1 | putative\_non-ribosomal\_peptide\_synthase | BGC0000972 | NRP+Polyketide:Modular type I polyketide+Polyketide:Trans-AT type I polyketide | 24.0 | 30.2 | 199.0 | 2.57e-50 |
| ABC36785.1 | peptide\_synthetase,\_putative | BGC0000964 | NRP:Cyclic depsipeptide+Polyketide:Trans-AT type I polyketide | 24.0 | 28.8 | 200.0 | 2.67e-50 |
| EFL06866.1 | predicted\_protein | BGC0000300 | NRP | 24.0 | 28.3 | 199.0 | 2.72e-50 |
| AGI89791.1 | Nonribosomal\_peptide\_synthetase | BGC0001792 | NRP | 24.0 | 29.9 | 200.0 | 3e-50 |
| OKA09423.1 | non-ribosomal\_peptide\_synthetase | BGC0001459 | NRP:Glycopeptide | 25.0 | 29.1 | 199.0 | 3.31e-50 |
| WP\_051462298.1 | non-ribosomal\_peptide\_synthetase | BGC0001873 | NRP:Lipopeptide | 23.0 | 27.9 | 199.0 | 3.84e-50 |
| AUD11994.1 | OrbI | BGC0001721 | NRP | 24.0 | 29.1 | 199.0 | 4.32e-50 |
| QYA95680.1 | amino\_acid\_adenylation\_domain-containing\_protein | BGC0002676 | NRP | 24.0 | 29.4 | 199.0 | 4.67e-50 |
| AAK81826.1 | peptide\_synthetase | BGC0000326 | NRP | 26.0 | 24.1 | 199.0 | 4.97e-50 |
| AHD05617.1 | putative\_non-ribosomal\_peptide\_ligase\_domain\_protein | BGC0001033 | NRP+Polyketide | 24.0 | 24.7 | 197.0 | 5.54e-50 |
| AIG79243.1 | Non-ribosomal\_peptide\_synthetase | BGC0000419 | Saccharide+NRP:Glycopeptide | 25.0 | 29.1 | 198.0 | 5.78e-50 |
| CCJ67647.1 | JagC | BGC0001127 | NRP | 25.0 | 28.6 | 199.0 | 5.98e-50 |
| ADY16697.1 | TqaA | BGC0001142 | NRP | 24.0 | 30.3 | 199.0 | 6.19e-50 |
| XP\_020058100.1 | uncharacterized\_protein | BGC0001220 | NRP | 23.0 | 35.0 | 199.0 | 7e-50 |
| CAJ45639.1 | vanchrobactin\_non\_ribosomal\_peptide\_synthetase | BGC0000454 | NRP | 23.0 | 26.4 | 198.0 | 7.06e-50 |
| AMK92560.1 | enediyne\_polyketide\_synthase | BGC0001377 | Polyketide | 26.0 | 23.5 | 198.0 | 7.21e-50 |
| BCJ07599.1 | hypothetical\_protein | BGC0002379 | NRP | 24.0 | 29.6 | 196.0 | 7.99e-50 |
| AZM57024.1 | non-ribosomal\_peptide\_synthetase | BGC0002314 | NRP | 25.0 | 28.5 | 198.0 | 8.41e-50 |
| ABA73954.1 | putative\_non-ribosomal\_peptide\_synthetase | BGC0001842 | NRP:Lipopeptide | 24.0 | 30.1 | 197.0 | 1.02e-49 |
| WP\_153044786.1 | non-ribosomal\_peptide\_synthetase | BGC0001826 | NRP | 23.0 | 30.5 | 196.0 | 1.07e-49 |
| QOE83923.1 | tyrocidine\_synthase\_3 | BGC0002051 | NRP | 24.0 | 30.0 | 195.0 | 1.39e-49 |
| BAY02129.1 | barbamide\_biosynthesis\_protein\_BarG | BGC0002532 | NRP+Polyketide | 23.0 | 28.7 | 194.0 | 3.37e-49 |
| AQM58286.1 | non-ribosomal\_peptide\_synthase | BGC0001816 | NRP+Polyketide | 23.0 | 30.5 | 196.0 | 3.41e-49 |
| QDF82254.1 | non-ribosomal\_peptide\_synthetase | BGC0001980 | NRP | 24.0 | 29.3 | 196.0 | 3.9e-49 |
| BS329\_14150 | non-ribosomal\_peptide\_synthetase | BGC0001462 | NRP:Glycopeptide | 25.0 | 28.9 | 195.0 | 5.02e-49 |
| BCJ07600.1 | hypothetical\_protein | BGC0002379 | NRP | 26.0 | 20.2 | 189.0 | 6.24e-49 |
| ESU15173.1 | hypothetical\_protein | BGC0002186 | NRP+Polyketide | 24.0 | 29.3 | 195.0 | 7.08e-49 |
| MBX9445647.1 | amino\_acid\_adenylation\_domain-containing\_protein | BGC0002414 | NRP | 25.0 | 26.8 | 195.0 | 7.92e-49 |
| AEI58865.1 | peptide\_synthetase | BGC0000455 | NRP | 24.0 | 29.7 | 195.0 | 8.29e-49 |
| DAB41479.1 | nonribosomal\_peptide\_synthetase | BGC0001230 | NRP:Cyclic depsipeptide+Polyketide:Modular type I polyketide | 25.0 | 29.6 | 193.0 | 1.42e-48 |
| QGQ63518.1 | nonribosomal\_peptide\_synthetase\_modules\_A | BGC0002548 | NRP | 24.0 | 29.9 | 194.0 | 1.5e-48 |
| ESU17760.1 | hypothetical\_protein | BGC0002172 | NRP | 24.0 | 28.8 | 194.0 | 1.83e-48 |
| AGS77307.1 | NRPS\_modules\_1-2 | BGC0001178 | NRP:Glycopeptide | 25.0 | 28.9 | 193.0 | 1.93e-48 |
| QBM78313.1 | non-ribosomal\_peptide\_synthatase | BGC0002542 | Polyketide+NRP | 23.0 | 29.9 | 191.0 | 4.16e-48 |
| AAM80539.1 | StaA | BGC0000290 | NRP:Glycopeptide | 26.0 | 28.8 | 192.0 | 4.31e-48 |
| WP\_064118560.1 | non-ribosomal\_peptide\_synthetase | BGC0001509 | NRP | 22.0 | 29.5 | 192.0 | 4.6e-48 |
| AGE11891.1 | nonribosomal\_peptide\_synthetase | BGC0000366 | NRP | 25.0 | 29.8 | 191.0 | 4.76e-48 |
| CBF76038.1 | nonribosomal\_peptide\_synthase,\_putative\_(Eurofung) | BGC0001399 | NRP | 22.0 | 34.9 | 191.0 | 6.77e-48 |
| AQZ71347.1 | hypothetical\_protein | BGC0001635 | NRP+Polyketide | 26.0 | 28.6 | 191.0 | 9.05e-48 |
| CAE53350.1 | non-ribosomal\_peptide\_synthetase | BGC0000440 | NRP:Glycopeptide | 25.0 | 28.8 | 191.0 | 9.69e-48 |
| CAG15009.1 | peptide\_synthetase,\_module\_1-2 | BGC0000441 | NRP | 25.0 | 28.8 | 190.0 | 1.66e-47 |
| AGQ43600.1 | HC-toxin\_synthetase | BGC0001166 | NRP | 24.0 | 27.7 | 191.0 | 1.84e-47 |
| EME52990.1 | amino\_acid\_adenylation\_protein | BGC0001460 | NRP:Glycopeptide | 24.0 | 29.5 | 190.0 | 2.08e-47 |
| ABV56588.1 | KtzH | BGC0000378 | NRP | 24.0 | 27.1 | 190.0 | 2.41e-47 |
| EXU96269.1 | nonribosomal\_peptide\_synthetase,\_serinocyclin\_synthetase\_NPS1 | BGC0001240 | NRP | 22.0 | 29.4 | 190.0 | 3.42e-47 |
| QCC62999.1 | BII-rafflesfungin\_nonribosomal\_protein\_synthetase | BGC0001966 | NRP+Polyketide | 24.0 | 29.9 | 190.0 | 3.47e-47 |
| BAD55612.1 | non-ribosomal\_peptide\_synthetase | BGC0001027 | NRP+Polyketide | 24.0 | 30.0 | 189.0 | 3.58e-47 |
| QKW60392.1 | amino\_acid\_adenylation\_domain-containing\_protein | BGC0002288 | NRP | 26.0 | 24.7 | 187.0 | 3.83e-47 |
| EPE34341.1 | non-ribosomal\_peptide\_synthetase | BGC0001035 | Polyketide+NRP | 23.0 | 28.9 | 189.0 | 4.33e-47 |
| QNN94286.1 | EmeB | BGC0002555 | NRP+Polyketide | 23.0 | 29.9 | 189.0 | 4.7e-47 |
| WP\_050383088.1 | non-ribosomal\_peptide\_synthetase | BGC0001451 | NRP | 24.0 | 29.9 | 187.0 | 5.23e-47 |
| QYC40287.1 | A50926\_NRPS,\_modules\_1-2 | BGC0002344 | NRP | 24.0 | 28.9 | 188.0 | 6.42e-47 |
| AWI62627.1 | nonribosomal\_peptide\_synthetase | BGC0001822 | NRP | 24.0 | 28.8 | 188.0 | 6.56e-47 |
| CBW75452.1 | Non-ribosomal\_peptide\_synthetase\_modules\_(EC\_6.3.2.-) | BGC0002048 | NRP:Cyclic depsipeptide | 24.0 | 27.0 | 188.0 | 6.9e-47 |
| AAZ55898.1 | amino\_acid\_adenylation | BGC0000359 | NRP | 25.0 | 29.1 | 187.0 | 8.7e-47 |
| ACZ66258.1 | APS1 | BGC0000304 | NRP | 25.0 | 26.7 | 188.0 | 9.15e-47 |
| ABC94347.1 | vicibactin\_biosynthesis\_non-ribosomal\_peptide\_synthase\_protein | BGC0000457 | NRP | 25.0 | 21.6 | 186.0 | 1.47e-46 |
| ARS01470.1 | NcmB | BGC0001702 | NRP+Polyketide | 25.0 | 31.3 | 186.0 | 1.59e-46 |
| XP\_001217690.1 | hypothetical\_protein | BGC0001517 | NRP | 22.0 | 35.2 | 187.0 | 2.01e-46 |
| EAU29302.1 | hypothetical\_protein | BGC0002272 | NRP | 23.0 | 29.6 | 187.0 | 2.02e-46 |
| AKJ70942.1 | non-ribosomal\_peptide\_synthetase | BGC0002611 | NRP | 24.0 | 29.3 | 187.0 | 2.16e-46 |
| AFH75328.1 | nonribosomal\_peptide\_synthetase | BGC0000398 | NRP:Cyclic depsipeptide | 23.0 | 29.7 | 186.0 | 2.49e-46 |
| AKJ15896.1 | non\_ribosomal\_peptide\_synthetase | BGC0002735 | Polyketide+NRP | 24.0 | 29.6 | 184.0 | 2.96e-46 |
| CAJ77695.1 | MPS1\_protein | BGC0000363 | NRP | 23.0 | 30.1 | 186.0 | 3.15e-46 |
| AIE77057.1 | peptide\_synthetase | BGC0000418 | NRP | 24.0 | 29.4 | 186.0 | 3.19e-46 |
| WP\_050383094.1 | non-ribosomal\_peptide\_synthetase | BGC0001451 | NRP | 24.0 | 26.6 | 184.0 | 5.1e-46 |
| EAW16180.1 | nonribosomal\_peptide\_synthase,\_putative | BGC0000293 | NRP | 23.0 | 29.5 | 185.0 | 5.96e-46 |
| AQZ26587.1 | obafluorin\_dimodular\_nonribosomal\_peptide\_synthetase | BGC0001437 | NRP | 23.0 | 28.9 | 185.0 | 6.76e-46 |
| BAE98155.1 | putative\_non-ribosomal\_peptide\_synthetase | BGC0000339 | NRP | 25.0 | 29.4 | 185.0 | 8.23e-46 |
| BAI63288.1 | putative\_non-ribosomal\_peptide\_synthetase | BGC0000434 | NRP | 25.0 | 30.1 | 184.0 | 1.07e-45 |
| QCE43602.1 | nonribosomal\_peptide\_synthetase\_(NRPS),\_subunit\_1 | BGC0001834 | NRP | 24.0 | 24.4 | 184.0 | 1.21e-45 |
| CAJ77715.1 | Mps1\_protein | BGC0000364 | NRP | 24.0 | 29.9 | 184.0 | 1.58e-45 |
| CAJ96471.1 | non-ribosomal\_peptide\_synthetase | BGC0000330 | NRP:NRP siderophore | 24.0 | 28.9 | 181.0 | 2.58e-45 |
| AAN32981.1 | BarG | BGC0000962 | NRP+Polyketide:Modular type I polyketide | 23.0 | 32.6 | 183.0 | 3.3e-45 |
| WP\_044618979.1 | non-ribosomal\_peptide\_synthetase | BGC0001791 | NRP | 22.0 | 29.9 | 183.0 | 3.65e-45 |
| AAQ59905.1 | synthetase\_CbsF | BGC0002680 | NRP | 23.0 | 32.8 | 182.0 | 4.3e-45 |
| ALG65340.1 | Var5 | BGC0002416 | NRP+Polyketide | 23.0 | 29.3 | 180.0 | 7.97e-45 |
| DMH02\_027320 | amino\_acid\_adenylation\_domain-containing\_protein | BGC0002638 | NRP | 25.0 | 27.0 | 179.0 | 9.72e-45 |
| CAC48360.1 | peptide\_synthetase | BGC0000311 | NRP | 25.0 | 28.8 | 181.0 | 1e-44 |
| AGZ20183.1 | non-ribosomal\_peptide\_synthetase | BGC0002618 | Terpene | 24.0 | 30.1 | 181.0 | 1.36e-44 |
| QIW91877.1 | NRPS | BGC0002543 | NRP | 23.0 | 30.2 | 181.0 | 1.58e-44 |
| AAT01806.1 | non-ribosomal\_peptide\_synthetase | BGC0000365 | NRP | 24.0 | 29.8 | 180.0 | 2.3e-44 |
| ADN26248.1 | peptide\_synthetase | BGC0000951 | NRP | 26.0 | 21.5 | 177.0 | 2.81e-44 |
| BBD17759.1 | non-ribosomal\_peptide\_synthetase | BGC0001919 | NRP+Polyketide | 26.0 | 21.5 | 179.0 | 7.29e-44 |
| QRD90553.1 | non-ribosomal\_peptide\_synthetase\_module | BGC0002157 | NRP+Alkaloid | 23.0 | 31.4 | 178.0 | 7.95e-44 |
| EHK22005.1 | putative\_non-ribosomal\_peptide\_synthetase\_GliP | BGC0001609 | NRP | 24.0 | 24.5 | 177.0 | 1.18e-43 |
| CAD91220.1 | putative\_non-ribosomal\_peptide\_synthetase,\_modules\_1-2 | BGC0000289 | NRP:Glycopeptide+Saccharide:Hybrid/tailoring saccharide | 24.0 | 28.9 | 177.0 | 1.21e-43 |
| XP\_003044554.1 | uncharacterized\_protein | BGC0001768 | NRP | 23.0 | 30.8 | 177.0 | 1.71e-43 |
| ESU05145.1 | hypothetical\_protein | BGC0002178 | NRP | 23.0 | 29.6 | 177.0 | 2.77e-43 |
| OJJ96433.1 | hypothetical\_protein | BGC0002226 | NRP | 23.0 | 31.9 | 176.0 | 5.01e-43 |
| NSC23530.1 | amino\_acid\_adenylation\_domain-containing\_protein | BGC0002359 | NRP | 25.0 | 31.8 | 175.0 | 5.29e-43 |
| QMS79067.1 | nonribosomal\_peptide\_synthetase\_12 | BGC0002198 | NRP | 23.0 | 30.1 | 175.0 | 6.69e-43 |
| CBF73453.1 | nonribosomal\_peptide\_synthase,\_putative\_(JCVI) | BGC0001515 | NRP | 23.0 | 30.1 | 176.0 | 6.71e-43 |
| AGK15447.1 | Non-ribosomal\_peptide\_synthetase,\_with\_condensation,\_AMP\_binding\_and\_thioesterase\_modules | BGC0002529 | NRP | 23.0 | 29.1 | 173.0 | 1.59e-42 |
| KGA48739.1 | amino\_acid\_adenylation\_domain\_protein | BGC0002413 | NRP | 22.0 | 30.1 | 174.0 | 1.92e-42 |
| WP\_051700122.1 | non-ribosomal\_peptide\_synthetase | BGC0001368 | NRP | 24.0 | 29.1 | 172.0 | 2.31e-42 |
| CAQ71827.1 | non\_ribosomal\_peptide\_synthase,\_antibiotic\_synthesis;\_contains\_1\_condensation\_domain,\_1\_AMP-acid\_ligases\_II\_domain | BGC0001189 | NRP | 24.0 | 26.5 | 172.0 | 2.76e-42 |
| AFK57220.1 | DidI | BGC0000985 | Polyketide+NRP:Cyclic depsipeptide | 25.0 | 24.3 | 171.0 | 2.97e-42 |
| AAS92545.1 | SirP | BGC0001044 | NRP | 22.0 | 32.2 | 173.0 | 3.11e-42 |
| BAX64244.1 | NRPS | BGC0001623 | NRP+Polyketide | 26.0 | 23.4 | 172.0 | 7.55e-42 |
| BCJ07531.1 | hypothetical\_protein | BGC0002379 | NRP | 24.0 | 27.8 | 169.0 | 1.57e-41 |
| AAL33756.1 | putative\_non-ribosomal\_peptide\_synthetase | BGC0000421 | NRP | 24.0 | 28.9 | 168.0 | 3.68e-41 |
| OAQ83772.1 | nonribosomal\_peptide\_synthase | BGC0001358 | NRP+Polyketide | 23.0 | 29.5 | 170.0 | 3.91e-41 |
| WP\_069848004.1 | non-ribosomal\_peptide\_synthetase | BGC0002472 | NRP | 22.0 | 23.8 | 167.0 | 1.08e-40 |
| AMK48225.1 | nonribosomal\_peptide\_synthetase | BGC0001351 | NRP | 25.0 | 29.7 | 167.0 | 1.2e-40 |
| QRD93053.1 | putative\_nonribosomal\_peptide\_synthase | BGC0002160 | NRP | 24.0 | 26.4 | 168.0 | 1.44e-40 |
| AJD47483.1 | amino\_acid\_adenylation\_domain-containing\_protein | BGC0002418 | NRP+Polyketide | 26.0 | 21.2 | 166.0 | 1.6e-40 |
| CAJ76286.1 | putative\_non-ribosomal\_peptide\_synthetase | BGC0000972 | NRP+Polyketide:Modular type I polyketide+Polyketide:Trans-AT type I polyketide | 21.0 | 27.5 | 167.0 | 1.68e-40 |
| ABW70808.1 | PchF | BGC0002475 | NRP | 25.0 | 23.6 | 167.0 | 2.05e-40 |
| EGX96627.1 | non-ribosomal\_peptide\_synthase,\_putative | BGC0002259 | Polyketide+NRP | 22.0 | 29.5 | 167.0 | 2.99e-40 |
| EFL06864.1 | non-ribosomal\_peptide\_synthetase | BGC0000300 | NRP | 24.0 | 29.2 | 165.0 | 3.31e-40 |
| ABK39646.1 | nonribosomal\_peptide\_synthetase | BGC0001502 | NRP | 23.0 | 29.7 | 165.0 | 3.6e-40 |
| EJP62835.1 | nonribosomal\_peptide\_synthase,\_putative | BGC0002203 | NRP+Polyketide+Other | 23.0 | 29.9 | 166.0 | 3.92e-40 |
| EKJ70673.1 | hypothetical\_protein | BGC0002188 | NRP+Polyketide | 23.0 | 30.1 | 166.0 | 4.26e-40 |
| QTT72092.1 | amino\_acid\_adenylation\_domain-containing\_protein | BGC0002350 | NRP+Polyketide+Saccharide | 24.0 | 29.3 | 166.0 | 4.32e-40 |
| BAD55613.1 | putative\_non-ribosomal\_peptide\_synthetase | BGC0001027 | NRP+Polyketide | 23.0 | 33.2 | 164.0 | 8.17e-40 |
| AAG02364.1 | peptide\_synthetase\_NRPS2-1 | BGC0000963 | NRP:Glycopeptide+Polyketide:Modular type I polyketide+Saccharide:Hybrid/tailoring saccharide | 23.0 | 29.7 | 165.0 | 9.66e-40 |
| WP\_035121683.1 | non-ribosomal\_peptide\_synthetase | BGC0002624 | NRP+Polyketide | 24.0 | 23.8 | 164.0 | 1.08e-39 |
| BAC76476.1 | multifunctional\_polyketide-peptide\_synthase\_LkcA | BGC0001100 | NRP+Polyketide | 27.0 | 22.8 | 164.0 | 1.24e-39 |
| UOH28374.1 | AceN | BGC0002149 | NRP+Terpene | 24.0 | 22.5 | 164.0 | 1.26e-39 |
| WP\_141576286.1 | non-ribosomal\_peptide\_synthetase | BGC0002686 | NRP | 25.0 | 28.5 | 163.0 | 1.3e-39 |
| KAF7597140.1 | hypothetical\_protein | BGC0002264 | NRP | 23.0 | 24.8 | 163.0 | 1.37e-39 |
| RAT94090.1 | NRPS | BGC0001469 | NRP | 21.0 | 32.3 | 164.0 | 1.55e-39 |
| CBF76036.1 | putative\_nonribosomal\_peptide\_synthetase\_(Eurofung) | BGC0001399 | NRP | 22.0 | 34.5 | 164.0 | 1.57e-39 |
| ATG32076.1 | nonribosomal\_peptide\_synthetase | BGC0001750 | NRP+Polyketide | 23.0 | 27.2 | 163.0 | 1.83e-39 |
| AGZ15476.1 | putative\_non\_ribosomal\_peptide\_synthetase | BGC0001036 | NRP+Polyketide | 24.0 | 30.2 | 162.0 | 3.59e-39 |
| AAF19811.1 | mtaC | BGC0001024 | NRP+Polyketide:Modular type I polyketide | 22.0 | 29.4 | 162.0 | 3.73e-39 |
| BAP16693.1 | nonribosomal\_peptide\_synthetase | BGC0000376 | NRP | 22.0 | 28.8 | 162.0 | 3.92e-39 |
| ABC34305.1 | peptide\_synthetase,\_putative | BGC0000961 | NRP+Polyketide | 24.0 | 26.1 | 162.0 | 5.61e-39 |
| MAA\_10043 | non-ribosomal\_peptide\_synthetase | BGC0000337 | NRP | 22.0 | 29.7 | 162.0 | 8.01e-39 |
| KIA75458.1 | hypothetical\_protein | BGC0002208 | NRP | 23.0 | 29.6 | 162.0 | 9.36e-39 |
| AMM63162.1 | AniA | BGC0001371 | NRP | 22.0 | 29.6 | 161.0 | 1.77e-38 |
| CCP45171.1 | Phenyloxazoline\_synthase\_MbtB\_(phenyloxazoline\_synthetase) | BGC0001021 | NRP+Polyketide | 24.0 | 29.6 | 160.0 | 2.12e-38 |
| QIE08738.1 | non-ribosomal\_peptide\_synthetase | BGC0002544 | NRP | 25.0 | 25.3 | 159.0 | 2.64e-38 |
| AAW03326.1 | CtaC | BGC0000982 | NRP+Polyketide | 23.0 | 29.8 | 156.0 | 2.9e-37 |
| WP\_069848010.1 | non-ribosomal\_peptide\_synthetase | BGC0002472 | NRP | 22.0 | 27.5 | 156.0 | 4.89e-37 |
| AAQ59161.2 | enterobactin\_synthetase\_component\_F | BGC0002679 | NRP | 24.0 | 22.3 | 154.0 | 8.43e-37 |
| AAO56106.1 | yersiniabactin\_non-ribosomal\_peptide\_synthetase | BGC0002570 | NRP+Polyketide | 23.0 | 28.2 | 155.0 | 9.36e-37 |
| EHA53213.1 | D-alanine-poly(phosphoribitol)\_ligase\_subunit\_1 | BGC0002158 | NRP+Polyketide | 23.0 | 31.7 | 154.0 | 1.1e-36 |
| QXJ21809.1 | amino\_acid\_adenylation\_domain-containing\_protein | BGC0002370 | NRP | 23.0 | 28.6 | 154.0 | 1.38e-36 |
| CAD89774.1 | MelC\_protein | BGC0001010 | NRP+Polyketide:Modular type I polyketide | 22.0 | 29.5 | 154.0 | 1.49e-36 |
| CAQ18831.1 | nonribosomal\_polypetide\_synthetase | BGC0000954 | NRP+Polyketide:Modular type I polyketide | 24.0 | 24.3 | 152.0 | 6.15e-36 |
| ABM91454.1 | lysergyl\_peptide\_synthetase\_LpsB | BGC0000348 | NRP | 23.0 | 25.2 | 151.0 | 7.73e-36 |
| MBD2892727.1 | Phenyloxazoline\_synthase\_MbtB | BGC0002718 | NRP | 25.0 | 28.4 | 150.0 | 1.05e-35 |
| CAA79245.2 | enniatin\_synthetase | BGC0000342 | NRP | 21.0 | 29.2 | 150.0 | 2.12e-35 |
| CRG85572.1 | nonribosomal\_peptide\_synthase,\_putative | BGC0001402 | NRP | 23.0 | 25.8 | 150.0 | 3.12e-35 |
| QYA95659.1 | amino\_acid\_adenylation\_domain-containing\_protein | BGC0002676 | NRP | 24.0 | 29.6 | 149.0 | 4.38e-35 |
| AUS29484.1 | non-ribosomal\_peptide\_synthetase | BGC0002605 | NRP+Polyketide | 23.0 | 29.2 | 149.0 | 5.04e-35 |
| QTX15956.1 | nonribosomal\_peptide\_synthase | BGC0002598 | Polyketide | 23.0 | 27.3 | 149.0 | 5.05e-35 |
| MBN3579112.1 | amino\_acid\_adenylation\_domain-containing\_protein | BGC0002613 | NRP+Polyketide | 23.0 | 24.8 | 149.0 | 5.28e-35 |
| AAC83656.1 | dihydroaeruginoic\_acid\_synthetase | BGC0000412 | NRP | 25.0 | 24.2 | 149.0 | 5.45e-35 |
| AUS29499.1 | non-ribosomal\_peptide\_synthetase | BGC0002607 | NRP+Polyketide | 23.0 | 27.4 | 148.0 | 8.65e-35 |
| MAA\_10036 | nonribosomal\_peptide\_synthase\_GliP-like,\_putative | BGC0000337 | NRP | 22.0 | 32.2 | 148.0 | 9.23e-35 |
| DAB41917.1 | ArzO\_-\_NRPS\_(Cy,\_A,\_Ox,\_PCP) | BGC0001884 | NRP+Polyketide | 23.0 | 24.7 | 147.0 | 1.59e-34 |
| BAE56606.1 |  | BGC0001123 | NRP | 23.0 | 32.3 | 147.0 | 1.77e-34 |
| MCC5026027.1 | amino\_acid\_adenylation\_domain-containing\_protein | BGC0002118 | NRP+Polyketide | 24.0 | 20.4 | 145.0 | 9.57e-34 |
| CAC17500.1 | putative\_non-ribosomal\_peptide\_synthase | BGC0000324 | NRP | 24.0 | 28.0 | 145.0 | 9.64e-34 |
| AAY42397.1 | Nonribosomal\_peptide\_synthetase | BGC0001000 | NRP:Lipopeptide+Polyketide:Modular type I polyketide | 22.0 | 37.3 | 145.0 | 1.21e-33 |
| AUS29489.1 | non-ribosomal\_peptide\_synthetase | BGC0002606 | NRP+Polyketide | 23.0 | 30.5 | 142.0 | 6.41e-33 |
| KDM89832.1 | peptide\_synthetase | BGC0002412 | NRP | 22.0 | 29.8 | 140.0 | 1.23e-32 |
| QXJ21808.1 | amino\_acid\_adenylation\_domain-containing\_protein | BGC0002370 | NRP | 24.0 | 29.3 | 141.0 | 1.79e-32 |
| AAF62881.1 | EpoB | BGC0000991 | NRP+Polyketide | 22.0 | 24.0 | 138.0 | 1.03e-31 |
| CCE28989.1 | non-ribosomal\_peptide\_synthetase | BGC0001365 | NRP | 22.0 | 26.4 | 138.0 | 1.29e-31 |
| BAE60013.1 |  | BGC0001518 | Terpene | 24.0 | 20.4 | 137.0 | 1.29e-31 |
| AAF26925.1 | nonribosomal\_peptide\_synthetase | BGC0000988 | NRP+Polyketide | 23.0 | 21.4 | 137.0 | 1.35e-31 |
| CBK62746.1 |  | BGC0001115 | NRP+Polyketide | 22.0 | 29.8 | 137.0 | 1.41e-31 |
| ACB46193.1 | nonribosomal\_peptide\_synthetase | BGC0000989 | NRP+Polyketide | 22.0 | 24.0 | 137.0 | 2.32e-31 |
| ADB12489.1 | EpoB | BGC0000990 | NRP+Polyketide | 22.0 | 24.0 | 136.0 | 3.98e-31 |
| CBJ89771.1 | Non-ribosomal\_peptide\_synthase\_involved\_in\_Xenocoumacin\_synthesis | BGC0001054 | NRP+Polyketide:Modular type I polyketide | 23.0 | 25.4 | 136.0 | 4.16e-31 |
| ACY02015.1 | enterobactin\_synthetase\_component\_F | BGC0000343 | NRP | 23.0 | 24.2 | 135.0 | 4.34e-31 |
| AXG47410.1 | non-ribosomal\_peptide\_synthetase | BGC0002715 | NRP+Polyketide | 25.0 | 25.1 | 136.0 | 4.95e-31 |
| ACG60761.1 | NRPS(C/A) | BGC0001058 | NRP:Glycopeptide+Polyketide:Modular type I polyketide+Saccharide:Hybrid/tailoring saccharide | 22.0 | 30.9 | 135.0 | 5.56e-31 |
| CAJ76289.1 | putative\_hybrid\_non-ribosomal\_peptide-polyketide\_synthetase | BGC0000972 | NRP+Polyketide:Modular type I polyketide+Polyketide:Trans-AT type I polyketide | 23.0 | 23.7 | 135.0 | 8.65e-31 |
| ALK21569.1 | non-ribosomal\_peptide\_synthetase | BGC0002678 | NRP | 25.0 | 21.9 | 134.0 | 1.58e-30 |
| CAJ87590.1 | putative\_peptide\_synthase | BGC0001055 | NRP+Polyketide | 24.0 | 24.7 | 132.0 | 9.5e-30 |
| AUS29494.1 | non-ribosomal\_peptide\_synthetase | BGC0001030 | NRP+Polyketide | 22.0 | 29.2 | 130.0 | 2.06e-29 |
| AJI44176.1 | nonribosomal\_peptide\_synthetase | BGC0001193 | NRP | 23.0 | 28.6 | 130.0 | 2.24e-29 |
| CCA29202.1 | non-ribosomal\_peptide\_synthetase | BGC0000955 | NRP+Polyketide:Modular type I polyketide | 22.0 | 29.2 | 129.0 | 8.66e-29 |
| AET79179.1 | lysergyl\_peptide\_synthetase\_subunit\_2 | BGC0001241 | Terpene | 22.0 | 24.3 | 127.0 | 1.86e-28 |
| CCE30235.1 | related\_to\_non-ribosomal\_peptide\_synthetase | BGC0002232 | Alkaloid | 22.0 | 24.3 | 127.0 | 1.86e-28 |
| CCA53799.1 | iron\_aquisition\_yersiniabactin\_synthesis\_enzyme | BGC0001801 | NRP | 25.0 | 23.8 | 127.0 | 2.71e-28 |
| ARB50206.1 | lysergyl\_peptide\_synthetase\_2 | BGC0001573 | Alkaloid | 22.0 | 25.7 | 122.0 | 4.86e-27 |
| AKQ52531.1 | nonribosomal\_peptide\_synthetase | BGC0002533 | NRP+Polyketide | 23.0 | 24.4 | 119.0 | 5.1e-26 |
| CAD15513.1 | non-ribosomal\_peptide\_synthetase | BGC0001014 | NRP:NRP siderophore+Polyketide:Modular type I polyketide+Polyketide:Iterative type I polyketide | 21.0 | 26.5 | 117.0 | 2.59e-25 |
| EWM62998.1 | mycocerosic\_acid\_synthase | BGC0001328 | NRP:Cyclic depsipeptide+Polyketide:Modular type I polyketide | 23.0 | 39.8 | 112.0 | 7.43e-24 |
