# Supplementary material for: Alkaliphilic/Alkali-Tolerant Fungi: Molecular, Biochemical, and Biotechnological Aspects
Source: J Fungi (Basel). 2023 Jun 9;9(6):652. doi: 10.3390/jof9060652 (PMC10301932; doi:10.3390/jof9060652)
Supplement: Supplementary file 1 [file jof-09-00652-s001.zip › S2/knownclusterblast/region1/input.path1.gene17_mibig_hits.html]

| MIBiG Protein | Description | MIBiG Cluster | MiBiG Product | % ID | % Coverage | BLAST Score | E-value |
| --- | --- | --- | --- | --- | --- | --- | --- |
| AGA37263.1 | short\_chain\_dehydrogenase | BGC0000816 | NRP+Alkaloid | 38.0 | 103.7 | 179.0 | 1.1e-55 |
| AGA37272.1 | short\_chain\_dehydrogenase | BGC0000819 | NRP+Alkaloid | 39.0 | 102.8 | 176.0 | 3.61e-54 |
| CCE67074.1 | dehydrogenase | BGC0001242 | Polyketide | 36.0 | 105.7 | 152.0 | 3.89e-45 |
| ABB69767.1 | PlaZ | BGC0000654 | Terpene+Saccharide:Hybrid/tailoring saccharide | 32.0 | 96.7 | 87.0 | 3.74e-20 |
| EWM63058.1 | oxidoreductase,\_short\_chain\_dehydrogenase/reductase\_family | BGC0000679 | Terpene | 27.0 | 99.6 | 58.0 | 7.48e-10 |
| AXO35178.1 | short-chain\_dehydrogenase/reductase\_SDR | BGC0001848 | Other | 26.0 | 99.6 | 58.0 | 7.48e-10 |
| AAM33685.1 | putative\_bifunctional\_cyclase/3-oxoacyl-ACP\_reductase | BGC0000230 | Polyketide:Type II polyketide | 28.0 | 103.3 | 57.0 | 2.93e-09 |
| CAM34370.1 | putative\_3-oxoacyl-ACP\_reductase | BGC0000242 | Polyketide | 27.0 | 102.8 | 49.0 | 1.68e-06 |
