# Supplementary material for: Alkaliphilic/Alkali-Tolerant Fungi: Molecular, Biochemical, and Biotechnological Aspects
Source: J Fungi (Basel). 2023 Jun 9;9(6):652. doi: 10.3390/jof9060652 (PMC10301932; doi:10.3390/jof9060652)
Supplement: Supplementary file 1 [file jof-09-00652-s001.zip › S2/knownclusterblast/region1/input.path1.gene18_mibig_hits.html]

| MIBiG Protein | Description | MIBiG Cluster | MiBiG Product | % ID | % Coverage | BLAST Score | E-value |
| --- | --- | --- | --- | --- | --- | --- | --- |
| ATZ45180.1 | Bcboa4 | BGC0001892 | Polyketide | 27.0 | 89.8 | 147.0 | 6.67e-39 |
| EHK21999.1 | hypothetical\_protein | BGC0001609 | NRP | 27.0 | 93.9 | 140.0 | 2.28e-36 |
| ANV81297.1 | GA14\_synthase | BGC0001604 | Terpene | 28.0 | 81.2 | 129.0 | 1.3e-32 |
| BBD84646.1 | putative\_cytochrome\_P450 | BGC0001775 | Terpene | 28.0 | 74.7 | 120.0 | 3e-29 |
| EHA55872.1 | cytochrome\_P450 | BGC0002235 | Polyketide+NRP | 28.0 | 74.9 | 116.0 | 1.05e-27 |
| QCS37514.1 | pyiG | BGC0001881 | NRP+Polyketide:Iterative type I polyketide | 31.0 | 74.2 | 113.0 | 8.2e-27 |
| EAW09122.1 | cytochrome\_P450 | BGC0000983 | NRP+Polyketide:Iterative type I polyketide | 26.0 | 75.1 | 108.0 | 2.98e-25 |
| QHD43134.1 | P450\_monooxygenase | BGC0002546 | NRP+Polyketide | 27.0 | 84.6 | 103.0 | 1.56e-23 |
| BAV32146.1 | cytochrome\_P450\_monooxygenase | BGC0001373 | Polyketide | 23.0 | 80.5 | 103.0 | 2.4e-23 |
