# Supplementary material for: Alkaliphilic/Alkali-Tolerant Fungi: Molecular, Biochemical, and Biotechnological Aspects
Source: J Fungi (Basel). 2023 Jun 9;9(6):652. doi: 10.3390/jof9060652 (PMC10301932; doi:10.3390/jof9060652)
Supplement: Supplementary file 1 [file jof-09-00652-s001.zip › S2/knownclusterblast/region1/input.path1.gene1_mibig_hits.html]

| MIBiG Protein | Description | MIBiG Cluster | MiBiG Product | % ID | % Coverage | BLAST Score | E-value |
| --- | --- | --- | --- | --- | --- | --- | --- |
| CBF82302.1 | conserved\_hypothetical\_protein | BGC0002180 | Polyketide | 33.0 | 63.1 | 139.0 | 1.84e-38 |
| KGO40477.1 | Serine\_hydrolase\_FSH | BGC0001205 | Polyketide | 28.0 | 63.7 | 88.0 | 9.92e-20 |
| BBU42027.1 | putative\_hydrolase | BGC0002222 | Polyketide | 27.0 | 66.5 | 71.0 | 6.89e-14 |
| QTE75993.1 | ZopR1 | BGC0002224 | Polyketide | 27.0 | 66.5 | 71.0 | 6.89e-14 |
| ANF07287.1 | hydrolase\_341 | BGC0001340 | Polyketide:Iterative type I polyketide | 39.0 | 26.3 | 67.0 | 1.1e-12 |
| EWG54261.1 | hypothetical\_protein | BGC0001190 | Polyketide | 29.0 | 56.4 | 68.0 | 1.48e-12 |
| QTE76001.1 | ScyR1 | BGC0002223 | Polyketide | 25.0 | 66.5 | 61.0 | 9.78e-11 |
| ASK38716.1 | hydrolase\_341 | BGC0001436 | Polyketide:Iterative type I polyketide | 39.0 | 26.5 | 59.0 | 1.32e-09 |
