# Supplementary material for: Alkaliphilic/Alkali-Tolerant Fungi: Molecular, Biochemical, and Biotechnological Aspects
Source: J Fungi (Basel). 2023 Jun 9;9(6):652. doi: 10.3390/jof9060652 (PMC10301932; doi:10.3390/jof9060652)
Supplement: Supplementary file 1 [file jof-09-00652-s001.zip › S2/knownclusterblast/region1/input.path1.gene20_mibig_hits.html]

| MIBiG Protein | Description | MIBiG Cluster | MiBiG Product | % ID | % Coverage | BLAST Score | E-value |
| --- | --- | --- | --- | --- | --- | --- | --- |
| EPS29069.1 | hypothetical\_protein | BGC0001724 | NRP+Polyketide | 37.0 | 98.4 | 2320.0 | 0.0 |
| BBQ09587.1 | PKS-NRPS\_hybrid | BGC0002261 | Polyketide | 37.0 | 97.3 | 2316.0 | 0.0 |
| EAW09117.1 | hybrid\_NRPS/PKS\_enzyme,\_putative | BGC0000983 | NRP+Polyketide:Iterative type I polyketide | 36.0 | 98.9 | 2290.0 | 0.0 |
| QOJ72663.1 | XenE | BGC0002505 | Polyketide+NRP | 36.0 | 98.4 | 2273.0 | 0.0 |
| QPC57090.1 | polyketide\_synthase-nonribosomal\_peptide\_synthetase | BGC0002230 | Polyketide+NRP | 36.0 | 95.8 | 2254.0 | 0.0 |
| EPS34234.1 | nonribosomal\_peptide\_synthatase-polyketide\_synthase | BGC0002067 | NRP+Polyketide:Iterative type I polyketide | 35.0 | 96.9 | 2254.0 | 0.0 |
| ACS68554.1 | hybrid\_PKS-NRPS\_protein | BGC0001026 | NRP+Polyketide | 35.0 | 97.8 | 2253.0 | 0.0 |
| QOG08944.1 | FfsA | BGC0002204 | Polyketide+NRP | 36.0 | 97.1 | 2242.0 | 0.0 |
| QXF14600.1 | PydA | BGC0002239 | Polyketide+NRP | 36.0 | 98.4 | 2226.0 | 0.0 |
| CBF80487.1 | hybrid\_PKS-NRPS\_(Eurofung) | BGC0000959 | NRP+Polyketide:Iterative type I polyketide | 36.0 | 96.1 | 2207.0 | 0.0 |
| QHD43130.1 | NRPS/PKS\_hybrid\_protein | BGC0002546 | NRP+Polyketide | 36.0 | 96.4 | 2206.0 | 0.0 |
| KKP04599.1 | Non-ribosomal\_peptide\_synthetase\_-\_Polyketide\_synthase | BGC0002066 | NRP+Polyketide:Iterative type I polyketide | 36.0 | 92.9 | 2203.0 | 0.0 |
| QCS37521.1 | pyiS | BGC0001881 | NRP+Polyketide:Iterative type I polyketide | 36.0 | 99.1 | 2200.0 | 0.0 |
| ARP51711.1 | PKS-NRPS\_hybrid\_protein | BGC0001741 | NRP+Polyketide | 35.0 | 97.0 | 2182.0 | 0.0 |
| EED49862.1 | hybrid\_PKS/NRPS\_enzyme,\_putative | BGC0001445 | NRP+Polyketide:Iterative type I polyketide | 36.0 | 93.2 | 2172.0 | 0.0 |
| QJX57338.1 | ChaA | BGC0002538 | Polyketide | 35.0 | 98.3 | 2171.0 | 0.0 |
| EHA55875.1 | polyketide\_synthase | BGC0002235 | Polyketide+NRP | 35.0 | 98.3 | 2159.0 | 0.0 |
| EHA55860.1 | polyketide\_synthase/peptide\_synthetase | BGC0002235 | Polyketide+NRP | 37.0 | 98.6 | 2156.0 | 0.0 |
| QBE85649.1 | BuaA | BGC0001857 | Alkaloid+NRP+Polyketide:Iterative type I polyketide | 35.0 | 97.5 | 2148.0 | 0.0 |
| AAV66110.2 | fusaridione\_A\_synthetase | BGC0000992 | NRP+Polyketide | 35.0 | 96.3 | 2147.0 | 0.0 |
| EAT91803.2 | hypothetical\_protein | BGC0002205 | Polyketide+NRP | 35.0 | 97.2 | 2139.0 | 0.0 |
| AFP73394.1 | FusA | BGC0001268 | NRP+Polyketide | 35.0 | 97.8 | 2138.0 | 0.0 |
| AAT28740.1 | FUSS | BGC0000064 | NRP+Polyketide | 34.0 | 97.5 | 2122.0 | 0.0 |
| QNH68024.1 | PfpA | BGC0002268 | Polyketide+NRP | 35.0 | 98.8 | 2118.0 | 0.0 |
| iliA |  | BGC0002035 | NRP+Polyketide | 35.0 | 98.1 | 2110.0 | 0.0 |
| AGO86662.1 | equisetin\_synthetase | BGC0001255 | NRP+Polyketide | 35.0 | 93.1 | 2073.0 | 0.0 |
| AEO57481.1 | PKS-NRPSs | BGC0001449 | NRP+Alkaloid+Polyketide:Iterative type I polyketide | 35.0 | 96.5 | 2062.0 | 0.0 |
| BBC43184.1 | PKS-NRPS\_hybrid | BGC0001738 | NRP+Polyketide | 35.0 | 93.2 | 2060.0 | 0.0 |
| QBQ83704.1 | polyketide\_synthase-nonribosomal\_peptide\_synthetase | BGC0002093 | Polyketide+NRP | 35.0 | 93.4 | 2047.0 | 0.0 |
| XP\_001220460.1 | uncharacterized\_protein | BGC0001182 | NRP+Polyketide:Iterative type I polyketide | 36.0 | 92.9 | 2030.0 | 0.0 |
| CAO91861.1 | PKS-NRPS\_hybrid | BGC0000968 | NRP+Polyketide:Iterative type I polyketide | 34.0 | 96.7 | 2023.0 | 0.0 |
| ADN43685.1 | PKS-NRPS | BGC0001136 | NRP+Polyketide:Iterative type I polyketide | 34.0 | 100.4 | 2013.0 | 0.0 |
| QBK15049.1 | PKS-NRPS\_hybrid\_TraA | BGC0002197 | Polyketide+NRP | 34.0 | 96.0 | 1986.0 | 0.0 |
| EAL85113.2 | hybrid\_PKS-NRPS\_enzyme | BGC0001037 | NRP+Polyketide:Iterative type I polyketide | 33.0 | 98.4 | 1971.0 | 0.0 |
| CAL69597.1 | PKS-NRPS | BGC0001049 | NRP+Polyketide:Iterative type I polyketide | 34.0 | 101.5 | 1967.0 | 0.0 |
| BAZ95823.1 | PKS-NRPS\_hybrid\_cpaA | BGC0001563 | NRP+Polyketide | 34.0 | 96.8 | 1961.0 | 0.0 |
| EDU47082.1 | lovastatin\_nonaketide\_synthase | BGC0002250 | Polyketide+NRP | 34.0 | 96.5 | 1941.0 | 0.0 |
| AKC54422.1 | fumosorinone\_biosynthesis\_polyketide\_synthase | BGC0001218 | NRP+Polyketide | 34.0 | 99.8 | 1934.0 | 0.0 |
| AZZ09613.1 | PvhA | BGC0002304 | Polyketide+NRP | 34.0 | 93.7 | 1909.0 | 0.0 |
| EAU38971.1 | PKS-NRPS\_hybrid | BGC0001122 | NRP+Polyketide:Iterative type I polyketide | 33.0 | 98.5 | 1859.0 | 0.0 |
| CCT72377.1 | probable\_polyketide\_synthase | BGC0001305 | Polyketide | 42.0 | 60.0 | 1847.0 | 0.0 |
| BCA42568.1 | polyketide\_synthase\_GrgA | BGC0002185 | Polyketide | 42.0 | 59.6 | 1840.0 | 0.0 |
| QJY30853.1 | PKS-NRPS\_hybrid\_protein | BGC0002539 | Alkaloid | 33.0 | 96.0 | 1801.0 | 0.0 |
| AFA26384.1 | polyketide\_synthase\_A | BGC0001874 | NRP+Polyketide | 33.0 | 93.4 | 1757.0 | 0.0 |
| BAK26562.1 | PKS-NRPS\_hybrid | BGC0000977 | NRP+Polyketide | 32.0 | 93.4 | 1730.0 | 0.0 |
| ATZ45182.1 | Bcboa6 | BGC0001892 | Polyketide | 39.0 | 60.0 | 1658.0 | 0.0 |
| OJJ98486.1 | hypothetical\_protein | BGC0002169 | Polyketide+NRP | 39.0 | 60.0 | 1595.0 | 0.0 |
| BAJ14522.1 | polyketide\_synthase | BGC0001254 | Polyketide | 38.0 | 60.4 | 1573.0 | 0.0 |
| KAF5858310.1 | HR-PKS | BGC0002139 | Polyketide | 37.0 | 60.3 | 1519.0 | 0.0 |
| ABA02239.1 | polyketide\_synthase | BGC0000098 | Polyketide | 34.0 | 74.9 | 1511.0 | 0.0 |
| GAW21479.1 | hypothetical\_protein | BGC0002192 | Polyketide | 34.0 | 73.7 | 1480.0 | 0.0 |
| BAC20564.1 | polyketide\_synthase | BGC0000039 | Polyketide | 33.0 | 74.0 | 1479.0 | 0.0 |
| CEF75886.1 |  | BGC0001600 | Polyketide | 37.0 | 62.7 | 1473.0 | 0.0 |
| QBC19710.1 | TwmB | BGC0001954 | NRP+Polyketide | 33.0 | 73.2 | 1457.0 | 0.0 |
| BBM05082.1 | polyketide\_synthase | BGC0002170 | Polyketide | 37.0 | 60.4 | 1454.0 | 0.0 |
| QQW45467.1 | polyketide\_synthase\_CalA' | BGC0002168 | Polyketide | 37.0 | 60.4 | 1453.0 | 0.0 |
| OQD69647.1 | hypothetical\_protein | BGC0002745 | Polyketide | 36.0 | 61.3 | 1403.0 | 0.0 |
| EAT85332.2 | hypothetical\_protein | BGC0002165 | Polyketide | 36.0 | 60.6 | 1401.0 | 0.0 |
| BAQ25466.1 | polyketide\_synthase | BGC0001264 | Polyketide | 36.0 | 60.0 | 1400.0 | 0.0 |
| XP\_659388.1 | hypothetical\_protein | BGC0001998 | Polyketide | 35.0 | 59.9 | 1293.0 | 0.0 |
| BBI47418.1 | polyketide\_synthase | BGC0002258 | Polyketide | 34.0 | 61.1 | 1274.0 | 0.0 |
| EAU29808.1 | hypothetical\_protein | BGC0001400 | Polyketide | 36.0 | 57.5 | 1233.0 | 0.0 |
| BAE61265.1 |  | BGC0002238 | Polyketide | 35.0 | 47.9 | 1048.0 | 0.0 |
| ctg1\_orf0002 |  | BGC0001068 | Terpene+Polyketide | 30.0 | 59.8 | 1014.0 | 7.75e-308 |
| OSS48297.1 | hypothetical\_protein | BGC0002194 | Polyketide | 30.0 | 62.7 | 939.0 | 7.45e-285 |
| AMY15057.1 | tetraketide\_synthase\_MF-SQTKS | BGC0001339 | Polyketide:Iterative type I polyketide | 29.0 | 62.6 | 930.0 | 1.81e-281 |
| BAC20566.1 | polyketide\_synthase | BGC0000039 | Polyketide | 29.0 | 61.6 | 929.0 | 1.99e-281 |
| ABA02240.1 | polyketide\_synthase | BGC0000098 | Polyketide | 29.0 | 62.2 | 902.0 | 1.69e-272 |
| EHA28244.1 | hypothetical\_protein | BGC0001143 | Polyketide | 29.0 | 62.0 | 902.0 | 1.74e-272 |
| AAD34559.1 | polyketide\_synthase | BGC0000088 | Polyketide | 29.0 | 60.9 | 894.0 | 5.24e-270 |
| EAL89230.2 | LovB-like\_polyketide\_synthase,\_putative | BGC0000129 | Polyketide | 36.0 | 37.5 | 886.0 | 7.37e-268 |
| BAD83684.1 | PKSN\_polyketide\_synthase\_for\_alternapyrone\_biosynthesis | BGC0000012 | Polyketide | 29.0 | 63.4 | 869.0 | 2.16e-261 |
| AMJ52080.1 | lijA | BGC0002255 | Polyketide | 28.0 | 63.0 | 870.0 | 3.28e-261 |
| EAU31921.1 | hypothetical\_protein | BGC0002267 | Polyketide | 29.0 | 63.1 | 866.0 | 6.81e-260 |
| EAA65604.1 | hypothetical\_protein | BGC0000022 | Polyketide | 29.0 | 60.6 | 851.0 | 2.31e-255 |
| EJP62832.1 | polyketide\_synthase,\_putative | BGC0002203 | NRP+Polyketide+Other | 30.0 | 61.2 | 848.0 | 2.28e-254 |
| EGX96624.1 | polyketide\_synthase,\_putative | BGC0002259 | Polyketide+NRP | 29.0 | 60.5 | 833.0 | 1.9e-249 |
| BBU42026.1 | putative\_polyketide\_synthase | BGC0002222 | Polyketide | 28.0 | 63.8 | 832.0 | 3.04e-248 |
| QTE75992.1 | ZopPKS | BGC0002224 | Polyketide | 28.0 | 63.8 | 832.0 | 3.04e-248 |
| BBU37368.1 | polyketide\_synthase | BGC0002525 | Polyketide | 29.0 | 61.3 | 826.0 | 4.08e-247 |
| CBF87072.1 | polyketide\_synthase,\_putative\_(Eurofung) | BGC0001290 | NRP | 29.0 | 61.9 | 805.0 | 5.08e-240 |
| OPB37944.1 | putative\_polyketide\_synthase | BGC0002206 | Polyketide | 28.0 | 64.7 | 795.0 | 7.93e-236 |
| EPE34340.1 | polyketide\_synthase | BGC0001035 | Polyketide+NRP | 28.0 | 62.7 | 791.0 | 2.56e-235 |
| ANF07288.1 | hrPKS | BGC0001340 | Polyketide:Iterative type I polyketide | 28.0 | 64.2 | 792.0 | 4.05e-235 |
| CAP95405.1 |  | BGC0001404 | Polyketide | 28.0 | 61.9 | 789.0 | 2.41e-234 |
| KFH44396.1 | Lovastatin\_nonaketide\_synthase-like\_protein | BGC0002190 | Polyketide | 28.0 | 62.7 | 785.0 | 5.98e-233 |
| ESU15174.1 | hypothetical\_protein | BGC0002186 | NRP+Polyketide | 28.0 | 61.5 | 783.0 | 9.62e-233 |
| CBX99534.1 | similar\_to\_polyketide\_synthase | BGC0001899 | Polyketide | 28.0 | 62.8 | 775.0 | 5.62e-230 |
| ASK38717.1 | polyketide\_synthase | BGC0001436 | Polyketide:Iterative type I polyketide | 28.0 | 62.8 | 770.0 | 4.08e-229 |
| ACB12550.1 | Fum1 | BGC0000063 | Polyketide | 28.0 | 62.9 | 754.0 | 6.33e-223 |
| EKJ70677.1 | PKS6 | BGC0002188 | NRP+Polyketide | 27.0 | 62.1 | 753.0 | 8.67e-223 |
| AAD43562.2 | Fum1p | BGC0000062 | Polyketide | 28.0 | 63.2 | 749.0 | 2.89e-221 |
| AEA35016.1 | hypothetical\_protein | BGC0002502 | Polyketide | 28.0 | 62.8 | 747.0 | 1.52e-220 |
| EAQ86385.1 | hypothetical\_protein | BGC0001405 | Polyketide | 27.0 | 62.3 | 737.0 | 1.24e-218 |
| OAQ83760.1 | polyketide\_synthase | BGC0001358 | NRP+Polyketide | 28.0 | 64.1 | 736.0 | 1.41e-217 |
| QTE76000.1 | ScyPKS | BGC0002223 | Polyketide | 26.0 | 63.3 | 735.0 | 4.93e-217 |
| QCC63000.1 | BII-rafflesfungin\_polyketide\_synthase | BGC0001966 | NRP+Polyketide | 27.0 | 64.0 | 729.0 | 1.63e-214 |
| EHA48594.1 | hypothetical\_protein | BGC0002155 | Polyketide | 27.0 | 65.8 | 731.0 | 2.59e-214 |
| AUS29485.1 | polyketide\_synthase | BGC0002605 | NRP+Polyketide | 28.0 | 62.6 | 716.0 | 1.08e-210 |
| BBG28484.1 | polyketide\_synthase\_CdmE | BGC0001926 | Polyketide | 27.0 | 61.4 | 717.0 | 1.16e-210 |
| ATQ39432.1 | PKS | BGC0001565 | NRP | 27.0 | 63.7 | 715.0 | 3.69e-210 |
| FAA01291.1 | polyketide\_synthase-nonribosomal\_peptide\_synthetase\_PyvA | BGC0002210 | Polyketide+NRP | 37.0 | 30.6 | 723.0 | 4.83e-209 |
| AMY15068.1 | hexaketide\_synthase\_MF-SQHKS | BGC0001339 | Polyketide:Iterative type I polyketide | 27.0 | 60.9 | 709.0 | 2.55e-208 |
| BAD97694.1 | Aft9-1 | BGC0000003 | Polyketide | 27.0 | 60.1 | 714.0 | 5.6e-208 |
| BBG28498.1 | putative\_polyketide\_synthase | BGC0001913 | Polyketide | 26.0 | 63.5 | 703.0 | 2.95e-206 |
| KAF9708863.1 | hypothetical\_protein | BGC0002515 | Polyketide | 27.0 | 63.9 | 701.0 | 2.93e-205 |
| EHA19289.1 | hypothetical\_protein | BGC0001124 | Polyketide | 36.0 | 30.3 | 696.0 | 3.64e-205 |
| CCT75967.1 | polyketide\_synthase | BGC0001606 | Polyketide | 27.0 | 61.4 | 696.0 | 2.8e-204 |
| OJJ98497.1 | hypothetical\_protein | BGC0002169 | Polyketide+NRP | 33.0 | 35.7 | 672.0 | 7.22e-204 |
| BAJ09789.1 | polyketide\_synthase | BGC0000146 | Polyketide | 26.0 | 64.6 | 695.0 | 2.07e-203 |
| ESU09893.1 | hypothetical\_protein | BGC0002191 | Polyketide | 27.0 | 61.1 | 689.0 | 8.68e-202 |
| AUS29500.1 | polyketide\_synthase | BGC0002607 | NRP+Polyketide | 27.0 | 63.3 | 682.0 | 1.09e-199 |
| QTX15955.1 | polyketide\_synthase | BGC0002598 | Polyketide | 27.0 | 63.3 | 682.0 | 1.31e-199 |
| BAN19720.1 | polyketide\_synthase | BGC0001252 | Polyketide | 28.0 | 60.7 | 681.0 | 1.49e-199 |
| AUS29490.1 | polyketide\_synthase | BGC0002606 | NRP+Polyketide | 28.0 | 60.4 | 674.0 | 3.75e-197 |
| AKL78824.1 | GLPKS3 | BGC0001187 | NRP:Lipopeptide+Polyketide:Iterative type I polyketide | 36.0 | 30.2 | 675.0 | 1.21e-195 |
| gene4 |  | BGC0001907 | Polyketide | 30.0 | 42.9 | 660.0 | 4.57e-195 |
| simG |  | BGC0000334 | NRP | 27.0 | 63.0 | 667.0 | 5.75e-195 |
| AIW82279.1 | PuwB | BGC0001125 | NRP+Polyketide | 27.0 | 60.7 | 665.0 | 3.33e-194 |
| QCP68965.1 | VatK | BGC0002296 | NRP+Polyketide | 26.0 | 60.9 | 667.0 | 1.19e-192 |
| DAB41915.1 | ArzM\_-\_PKS\_(KS,\_AT,\_DH,\_MT,\_ER,\_KR,\_ACP) | BGC0001884 | NRP+Polyketide | 26.0 | 61.3 | 659.0 | 9.9e-192 |
| AQM58285.1 | polyketide\_synthase | BGC0001816 | NRP+Polyketide | 26.0 | 62.0 | 657.0 | 1.19e-191 |
| AUS29495.1 | polyketide\_synthase | BGC0001030 | NRP+Polyketide | 27.0 | 63.0 | 653.0 | 2.5e-190 |
| AXN93577.1 | PuwB | BGC0001950 | NRP | 26.0 | 61.9 | 637.0 | 8.93e-185 |
| AAS98781.1 | polyketide\_synthase | BGC0001001 | NRP+Polyketide | 26.0 | 62.8 | 640.0 | 5.03e-184 |
| AXN93586.1 | PuwB | BGC0001951 | NRP | 26.0 | 61.9 | 634.0 | 6.52e-184 |
| EAT86855.2 | hypothetical\_protein | BGC0001858 | Polyketide | 29.0 | 55.2 | 628.0 | 7.25e-184 |
| KKP00963.1 | fatty\_acid\_synthase\_S-acetyltransferase | BGC0001854 | Polyketide:Iterative type I polyketide | 34.0 | 31.2 | 630.0 | 1.36e-183 |
| OAQ83765.1 | KR\_domain-containing\_protein | BGC0001358 | NRP+Polyketide | 26.0 | 62.0 | 625.0 | 4.24e-182 |
| AQA28562.1 | type\_I\_polyketide\_synthase | BGC0001663 | Polyketide | 26.0 | 62.4 | 627.0 | 1.17e-181 |
| EHA52508.1 | mycocerosic\_acid\_synthase | BGC0001749 | Polyketide | 27.0 | 63.4 | 625.0 | 3.7e-181 |
| XP\_028481819.1 | highly-reducing\_poluketide\_synthase | BGC0001866 | Polyketide | 32.0 | 37.5 | 619.0 | 4.26e-181 |
| AXN93597.1 | PuwB | BGC0001952 | NRP | 26.0 | 60.4 | 624.0 | 6.39e-181 |
| BAV32159.1 | polyketide\_synthase | BGC0001373 | Polyketide | 39.0 | 25.3 | 627.0 | 1.63e-180 |
| EAA36364.1 | polyketide\_synthase\_6 | BGC0002729 | Polyketide | 35.0 | 26.6 | 620.0 | 3.13e-180 |
| AXN93610.1 | PuwB | BGC0001953 | NRP | 26.0 | 60.3 | 618.0 | 5.03e-179 |
| CBF82304.1 | polyketide\_synthase,\_putative\_(JCVI) | BGC0002180 | Polyketide | 35.0 | 29.2 | 608.0 | 4.72e-176 |
| KGO40478.1 | Acyl\_transferase/acyl\_hydrolase/lysophospholipase | BGC0001205 | Polyketide | 39.0 | 24.4 | 597.0 | 7.29e-173 |
| KFG78606.1 | polyketide\_synthase | BGC0002240 | Polyketide | 36.0 | 28.7 | 600.0 | 4.67e-172 |
| AGC95324.1 | CurS1 | BGC0000045 | Polyketide | 35.0 | 27.8 | 586.0 | 2.68e-169 |
| AHV78252.1 | ResS1 | BGC0001246 | Polyketide | 37.0 | 25.1 | 584.0 | 1.25e-168 |
| EYE95336.1 | polyketide\_synthase | BGC0002234 | Polyketide | 37.0 | 25.7 | 581.0 | 9.33e-168 |
| ACD39758.1 | reducing\_polyketide\_synthase | BGC0000076 | Polyketide | 37.0 | 25.1 | 573.0 | 1.68e-165 |
| ACD39767.1 | reducing\_polyketide\_synthase | BGC0000077 | Polyketide | 37.0 | 25.1 | 573.0 | 2.23e-165 |
| AHV78245.1 | LasS1 | BGC0001245 | Polyketide | 36.0 | 28.1 | 573.0 | 4.26e-165 |
| AEE88280.1 | CurJ | BGC0000976 | NRP+Polyketide:Modular type I polyketide | 29.0 | 38.7 | 568.0 | 4.22e-164 |
| AAT70105.1 | CurJ | BGC0001165 | NRP+Polyketide:Modular type I polyketide | 29.0 | 38.7 | 568.0 | 4.22e-164 |
| QBM78312.1 | polyketide\_synthase | BGC0002542 | Polyketide+NRP | 29.0 | 38.5 | 573.0 | 9.14e-164 |
| QCL09089.1 | dmxL2 | BGC0002063 | Polyketide:Iterative type I polyketide | 34.0 | 30.2 | 565.0 | 1.85e-162 |
| AAF62884.1 | EpoE | BGC0000991 | NRP+Polyketide | 31.0 | 36.9 | 561.0 | 1.06e-159 |
| ACD39774.1 | reducing\_polyketide\_synthase | BGC0000134 | Polyketide | 36.0 | 24.4 | 555.0 | 2.37e-159 |
| EWG54266.1 | hypothetical\_protein | BGC0001190 | Polyketide | 35.0 | 25.2 | 553.0 | 8.9e-159 |
| OAQ63050.2 | polyketide\_synthase | BGC0002187 | Polyketide | 35.0 | 24.4 | 550.0 | 6.98e-158 |
| ADB12492.1 | EpoE | BGC0000990 | NRP+Polyketide | 31.0 | 36.9 | 555.0 | 1.04e-157 |
| AAF26922.1 | polyketide\_synthase | BGC0000988 | NRP+Polyketide | 30.0 | 36.7 | 554.0 | 1.78e-157 |
| AZH23817.1 | MgiQ | BGC0001971 | NRP+Polyketide | 28.0 | 38.8 | 550.0 | 4.33e-157 |
| CEN60541.1 | hypothetical\_protein | BGC0002266 | Terpene+Polyketide | 33.0 | 27.2 | 545.0 | 2.27e-156 |
| ACB46196.1 | polyketide\_synthase | BGC0000989 | NRP+Polyketide | 31.0 | 36.8 | 550.0 | 2.65e-156 |
| ABB90283.1 | polyketide\_synthase | BGC0001057 | NRP+Polyketide | 35.0 | 25.5 | 544.0 | 3.58e-156 |
| ADZ24996.1 | polyketide\_synthase | BGC0000380 | NRP+Polyketide:Modular type I polyketide | 29.0 | 44.1 | 543.0 | 5.53e-156 |
| AZH23787.1 | MgcQ | BGC0001970 | NRP+Polyketide | 28.0 | 38.0 | 538.0 | 3.98e-153 |
| ADZ24998.1 | polyketide\_synthase | BGC0000380 | NRP+Polyketide:Modular type I polyketide | 30.0 | 37.4 | 524.0 | 3.42e-150 |
| GAA85575.1 | polyketide\_synthase | BGC0002227 | NRP | 35.0 | 24.6 | 526.0 | 3.66e-150 |
| AIA58899.1 | HRPKS | BGC0001141 | Polyketide:Iterative type I polyketide | 35.0 | 23.9 | 525.0 | 4.16e-150 |
| EHK18438.1 | putative\_polyketide\_synthase | BGC0002233 | Polyketide | 34.0 | 26.2 | 523.0 | 1.77e-149 |
| ACZ57548.1 | polyketide\_synthase | BGC0000046 | Polyketide:Iterative type I polyketide | 33.0 | 26.2 | 518.0 | 8.61e-148 |
| QCP68967.1 | VatT | BGC0002296 | NRP+Polyketide | 29.0 | 37.6 | 515.0 | 2.83e-147 |
| QGW49095.1 | putative\_polyketide\_synthase | BGC0002731 | Polyketide | 32.0 | 27.7 | 516.0 | 3.65e-147 |
| EAL85129.1 | polyketide\_synthase | BGC0001067 | Terpene+Polyketide:Iterative type I polyketide | 32.0 | 29.7 | 512.0 | 1.18e-145 |
| ABX60161.1 | mixed\_NRPS/PKS | BGC0000978 | NRP+Alkaloid+Polyketide:Modular type I polyketide | 29.0 | 37.9 | 514.0 | 1.2e-145 |
| QBK15047.1 | polyketide\_synthase\_ClaI | BGC0002196 | Polyketide | 33.0 | 27.5 | 510.0 | 1.92e-145 |
| APZ78754.1 | polyketide\_synthase | BGC0001423 | NRP:Cyclic depsipeptide+Polyketide:Iterative type I polyketide | 36.0 | 24.6 | 508.0 | 1.94e-145 |
| APZ78727.1 | polyketide\_synthase | BGC0001421 | NRP:Cyclic depsipeptide+Polyketide:Iterative type I polyketide | 36.0 | 24.5 | 508.0 | 2.55e-145 |
| ADF88279.1 | mixed\_NRPS/PKS | BGC0000981 | NRP+Polyketide | 29.0 | 37.9 | 511.0 | 6.12e-145 |
| AQW44889.1 | polyketide\_synthase | BGC0001737 | NRP+Polyketide | 36.0 | 23.7 | 506.0 | 1.24e-144 |
| ABM21569.1 | crpA | BGC0000975 | NRP+Polyketide | 28.0 | 38.4 | 510.0 | 1.44e-144 |
| APZ78702.1 | polyketide\_synthase | BGC0001419 | NRP:Cyclic depsipeptide+Polyketide:Iterative type I polyketide | 36.0 | 24.2 | 506.0 | 1.59e-144 |
| BAG17643.1 | putative\_NRPS-type-I\_PKS\_fusion\_protein | BGC0001043 | NRP+Polyketide | 35.0 | 24.6 | 510.0 | 2.51e-144 |
| APZ78854.1 | polyketide\_synthase | BGC0001432 | NRP:Cyclic depsipeptide+Polyketide:Iterative type I polyketide | 36.0 | 24.3 | 504.0 | 3.68e-144 |
| APZ78690.1 | polyketide\_synthase | BGC0001418 | NRP:Cyclic depsipeptide+Polyketide:Iterative type I polyketide | 36.0 | 24.1 | 504.0 | 3.71e-144 |
| APZ78714.1 | polyketide\_synthase | BGC0001420 | NRP:Cyclic depsipeptide+Polyketide:Iterative type I polyketide | 36.0 | 24.1 | 504.0 | 3.71e-144 |
| QGU18619.1 | polyketide\_synthase/non-ribosomal\_peptide\_synthetase | BGC0002365 | Other+Polyketide | 35.0 | 25.4 | 508.0 | 7.23e-144 |
| APZ78678.1 | polyketide\_synthase | BGC0001417 | NRP:Cyclic depsipeptide+Polyketide:Iterative type I polyketide | 36.0 | 24.1 | 503.0 | 1.52e-143 |
| KZL86691.1 | polyketide\_synthase | BGC0002228 | NRP | 34.0 | 25.6 | 504.0 | 1.87e-143 |
| APZ78780.1 | polyketide\_synthase | BGC0001426 | NRP:Cyclic depsipeptide+Polyketide:Iterative type I polyketide | 37.0 | 22.4 | 499.0 | 1.3e-142 |
| APZ78742.1 | polyketide\_synthase | BGC0001422 | NRP:Cyclic depsipeptide+Polyketide:Iterative type I polyketide | 37.0 | 22.6 | 499.0 | 1.71e-142 |
| APD26279.1 | PtmA | BGC0001726 | NRP+Polyketide | 35.0 | 27.2 | 504.0 | 1.82e-142 |
| CAD19089.1 | StiE\_protein | BGC0000153 | NRP+Polyketide:Modular type I polyketide | 37.0 | 20.6 | 495.0 | 5.42e-142 |
| APZ78844.1 | polyketide\_synthase | BGC0001431 | NRP:Cyclic depsipeptide+Polyketide:Iterative type I polyketide | 35.0 | 27.2 | 497.0 | 1.04e-141 |
| AQM37582.1 | polyketide\_synthase | BGC0001424 | NRP:Cyclic depsipeptide+Polyketide:Iterative type I polyketide | 37.0 | 22.6 | 496.0 | 1.63e-141 |
| APZ78807.1 | polyketide\_synthase | BGC0001428 | NRP:Cyclic depsipeptide+Polyketide:Iterative type I polyketide | 36.0 | 23.8 | 496.0 | 2.19e-141 |
| AQW44891.1 | polyketide\_synthase | BGC0001737 | NRP+Polyketide | 37.0 | 22.2 | 491.0 | 2.93e-141 |
| MBV7329454.1 | type\_I\_polyketide\_synthase | BGC0002131 | Polyketide+NRP:Glycopeptide+Saccharide:Hybrid/tailoring saccharide | 33.0 | 29.2 | 491.0 | 4.78e-141 |
| AGC45624.1 | polyketide\_synthase | BGC0001394 | NRP+Polyketide | 37.0 | 23.5 | 494.0 | 8.07e-141 |
| APZ78832.1 | polyketide\_synthase | BGC0001430 | NRP:Cyclic depsipeptide+Polyketide:Iterative type I polyketide | 36.0 | 24.1 | 493.0 | 1.3e-140 |
| ATZ45185.1 | Bcboa9 | BGC0001892 | Polyketide | 31.0 | 30.0 | 494.0 | 1.61e-140 |
| APZ78793.1 | polyketide\_synthase | BGC0001427 | NRP:Cyclic depsipeptide+Polyketide:Iterative type I polyketide | 37.0 | 22.3 | 493.0 | 1.66e-140 |
| APZ78767.1 | polyketide\_synthase | BGC0001425 | NRP:Cyclic depsipeptide+Polyketide:Iterative type I polyketide | 37.0 | 22.4 | 488.0 | 8.1e-139 |
| AJD77023.1 | IkaA | BGC0001435 | NRP+Polyketide:Iterative type I polyketide | 34.0 | 25.2 | 489.0 | 4.08e-138 |
| CAG28678.1 | polyketide\_synthase | BGC0001023 | NRP+Polyketide:Modular type I polyketide | 35.0 | 24.1 | 485.0 | 6.39e-138 |
| APZ78820.1 | polyketide\_synthase | BGC0001429 | NRP:Cyclic depsipeptide+Polyketide:Iterative type I polyketide | 35.0 | 24.1 | 485.0 | 6.39e-138 |
| QWM97862.1 | hybrid\_non-ribosomal\_peptide\_synthetase/type\_I\_polyketide\_synthase | BGC0002434 | Polyketide+NRP | 34.0 | 25.3 | 489.0 | 7.37e-138 |
| AAK57188.1 | MxaD | BGC0001022 | NRP+Polyketide | 35.0 | 23.8 | 481.0 | 7.6e-138 |
| OAG05545.1 | PKSKA1\_protein | BGC0002211 | Polyketide | 31.0 | 30.8 | 486.0 | 1.07e-137 |
| EFL02193.1 | amino\_acid\_adenylation\_domain-containing\_protein | BGC0000996 | NRP+Polyketide:Iterative type I polyketide | 34.0 | 25.2 | 488.0 | 1.08e-137 |
| AHA38202.1 | GphI | BGC0000069 | Polyketide | 28.0 | 38.7 | 484.0 | 1.53e-137 |
| ABL86391.1 | hybrid\_polyketide\_synthase\_and\_nonribosomal\_peptide\_synthetase | BGC0000999 | NRP+Polyketide | 35.0 | 23.2 | 488.0 | 1.57e-137 |
| AIT55263.1 | polyketide\_synthase | BGC0000072 | Polyketide:Modular type I polyketide | 34.0 | 27.3 | 483.0 | 9.85e-137 |
| QNS30807.1 | hybrid\_non-ribosomal\_peptide\_synthetase/type\_I\_polyketide\_syn-thase | BGC0002509 | NRP | 35.0 | 23.5 | 483.0 | 4.18e-136 |
| AGC45619.1 | polyketide\_synthase | BGC0001394 | NRP+Polyketide | 36.0 | 22.7 | 478.0 | 1.13e-135 |
| CAJ46690.1 | polyketide\_synthase | BGC0000969 | NRP:Cyclic depsipeptide+Polyketide:Modular type I polyketide | 33.0 | 26.5 | 476.0 | 4.93e-135 |
| AAK57187.1 | MxaC | BGC0001022 | NRP+Polyketide | 35.0 | 22.6 | 479.0 | 1.26e-134 |
| AAK57189.1 | MxaE | BGC0001022 | NRP+Polyketide | 35.0 | 23.0 | 470.0 | 2.96e-134 |
| AGC45621.1 | polyketide\_synthase | BGC0001394 | NRP+Polyketide | 37.0 | 22.3 | 468.0 | 1.12e-133 |
| QDA77058.1 | polyketide\_synthase | BGC0002026 | NRP+Polyketide | 37.0 | 22.5 | 474.0 | 2.06e-133 |
| AWO77084.1 | hybrid\_non-ribosomal\_peptide\_synthetase/type\_I\_polyketide\_synthase | BGC0001556 | NRP+Polyketide | 32.0 | 28.2 | 474.0 | 3.08e-133 |
| AVI26388.1 | polyketide\_synthase | BGC0001800 | NRP+Polyketide | 28.0 | 38.0 | 471.0 | 1.1e-132 |
| QVV57685.1 | malonyl\_CoA-acyl\_carrier\_protein\_transacylase | BGC0002338 | Polyketide | 35.0 | 22.2 | 463.0 | 4.35e-132 |
| AQW44890.1 | polyketide\_synthase | BGC0001737 | NRP+Polyketide | 36.0 | 22.8 | 466.0 | 5.3e-132 |
| CAQ18830.1 | polyketide\_synthase | BGC0000954 | NRP+Polyketide:Modular type I polyketide | 32.0 | 29.0 | 464.0 | 4.19e-131 |
| AUD08663.1 | iPKS-NRPS | BGC0001553 | NRP+Polyketide | 34.0 | 24.9 | 466.0 | 6.55e-131 |
| CCE88380.1 | polyketide\_synthase | BGC0001034 | NRP+Polyketide:Modular type I polyketide | 32.0 | 27.1 | 459.0 | 7.17e-131 |
| AMB48442.1 | polyketide\_synthase | BGC0001357 | Polyketide | 32.0 | 24.0 | 450.0 | 1.13e-130 |
| AKL71649.1 | NocP | BGC0001703 | Other | 30.0 | 28.7 | 449.0 | 1.38e-130 |
| CBD77732.1 | polyketide\_synthase | BGC0000974 | NRP+Polyketide | 37.0 | 21.0 | 464.0 | 1.48e-130 |
| AHA38199.1 | GphF | BGC0000069 | Polyketide | 28.0 | 41.3 | 465.0 | 1.91e-130 |
| CAQ34919.1 | polyketide\_synthase | BGC0000986 | NRP+Polyketide | 31.0 | 28.1 | 458.0 | 1.94e-130 |
| CCE88378.1 | polyketide\_synthase | BGC0001034 | NRP+Polyketide:Modular type I polyketide | 34.0 | 24.6 | 462.0 | 2.39e-130 |
| CAD19091.1 | StiG\_protein | BGC0000153 | NRP+Polyketide:Modular type I polyketide | 32.0 | 26.7 | 449.0 | 3.43e-130 |
| CCE88381.1 | polyketide\_synthase | BGC0001034 | NRP+Polyketide:Modular type I polyketide | 32.0 | 27.2 | 457.0 | 4.65e-130 |
| CAQ43076.1 | polyketide\_synthase | BGC0000970 | NRP+Polyketide:Modular type I polyketide | 32.0 | 26.7 | 453.0 | 1.08e-128 |
| AGC45622.1 | polyketide\_synthase | BGC0001394 | NRP+Polyketide | 35.0 | 22.9 | 450.0 | 5.89e-128 |
| CCE88379.1 | polyketide\_synthase | BGC0001034 | NRP+Polyketide:Modular type I polyketide | 33.0 | 23.5 | 449.0 | 1.73e-127 |
| AEE88284.1 | CurF | BGC0000976 | NRP+Polyketide:Modular type I polyketide | 29.0 | 28.4 | 453.0 | 4.53e-127 |
| AAT70101.1 | CurF | BGC0001165 | NRP+Polyketide:Modular type I polyketide | 29.0 | 28.4 | 452.0 | 1.02e-126 |
| CAD19088.1 | StiD\_protein | BGC0000153 | NRP+Polyketide:Modular type I polyketide | 37.0 | 20.4 | 447.0 | 1.08e-126 |
| QVV57686.1 | hypothetical\_protein | BGC0002338 | Polyketide | 36.0 | 21.5 | 452.0 | 1.47e-126 |
| QLJ99331.2 | acyltransferase\_domain-containing\_protein | BGC0002088 | Polyketide+Saccharide:Oligosaccharide | 35.0 | 22.2 | 434.0 | 2.63e-126 |
| AIT55260.1 | polyketide\_synthase | BGC0000072 | Polyketide:Modular type I polyketide | 33.0 | 24.7 | 445.0 | 3.12e-126 |
| AAS98783.1 | polyketide\_synthase/nonribosomal\_peptide\_synthase\_hybrid | BGC0001001 | NRP+Polyketide | 32.0 | 25.5 | 449.0 | 9.31e-126 |
| BCB17033.1 | modular\_polyketide\_synthase | BGC0002523 | NRP | 32.0 | 26.5 | 449.0 | 1.74e-125 |
| AGC45623.1 | polyketide\_synthase | BGC0001394 | NRP+Polyketide | 34.0 | 23.0 | 441.0 | 4.94e-125 |
| ACR33078.1 | polyketide\_synthase | BGC0000017 | Alkaloid+Polyketide:Modular type I polyketide | 33.0 | 22.3 | 441.0 | 5.78e-125 |
| AKD43522.1 | Type\_I\_polyketide\_synthase | BGC0001409 | Polyketide | 35.0 | 23.7 | 443.0 | 1.08e-124 |
| AAF62882.1 | EpoC | BGC0000991 | NRP+Polyketide | 32.0 | 27.1 | 439.0 | 2.37e-124 |
| CAD89776.1 | MelE\_protein | BGC0001010 | NRP+Polyketide:Modular type I polyketide | 36.0 | 20.5 | 439.0 | 2.64e-124 |
| CCE88376.1 | polyketide\_synthase | BGC0001034 | NRP+Polyketide:Modular type I polyketide | 32.0 | 27.0 | 444.0 | 3.14e-124 |
| AZH23788.1 | MgcR | BGC0001970 | NRP+Polyketide | 33.0 | 23.5 | 442.0 | 4.59e-124 |
| ADB12490.1 | EpoC | BGC0000990 | NRP+Polyketide | 32.0 | 27.1 | 437.0 | 5.56e-124 |
| CAQ18829.1 | polyketide\_synthase | BGC0000954 | NRP+Polyketide:Modular type I polyketide | 37.0 | 21.3 | 443.0 | 5.63e-124 |
| QVV57687.1 | polyketide\_synthase | BGC0002338 | Polyketide | 36.0 | 21.3 | 443.0 | 6.42e-124 |
| AAF26920.1 | polyketide\_synthase | BGC0000988 | NRP+Polyketide | 32.0 | 27.1 | 437.0 | 7.38e-124 |
| AQW44893.1 | polyketide\_synthase | BGC0001737 | NRP+Polyketide | 34.0 | 24.2 | 437.0 | 9.02e-124 |
| WP\_036342114.1 | type\_I\_polyketide\_synthase | BGC0001327 | NRP:Cyclic depsipeptide+Polyketide:Modular type I polyketide | 33.0 | 27.1 | 442.0 | 9.09e-124 |
| AQW44892.1 | polyketide\_synthase | BGC0001737 | NRP+Polyketide | 34.0 | 22.5 | 437.0 | 1.2e-123 |
| CAQ43075.1 | polyketide\_synthase | BGC0000970 | NRP+Polyketide:Modular type I polyketide | 32.0 | 27.3 | 442.0 | 1.21e-123 |
| CBD77748.1 | polyketide\_synthase | BGC0000974 | NRP+Polyketide | 32.0 | 27.2 | 434.0 | 8.98e-123 |
| AEE88279.1 | CurK | BGC0000976 | NRP+Polyketide:Modular type I polyketide | 30.0 | 27.7 | 437.0 | 1.13e-122 |
| AAT70106.1 | CurK | BGC0001165 | NRP+Polyketide:Modular type I polyketide | 30.0 | 27.7 | 437.0 | 1.13e-122 |
| AAS98777.1 | polyketide\_synthetase | BGC0001001 | NRP+Polyketide | 33.0 | 21.6 | 432.0 | 1.5e-122 |
| WP\_042799407.1 | SDR\_family\_NAD(P)-dependent\_oxidoreductase | BGC0001283 | Polyketide | 33.0 | 24.5 | 436.0 | 2.93e-122 |
| ADM46358.1 | polyketide\_synthase | BGC0000106 | Polyketide | 32.0 | 26.6 | 438.0 | 2.94e-122 |
| ANI24099.1 | polyketide\_synthase | BGC0001235 | NRP+Polyketide | 34.0 | 22.7 | 438.0 | 3.32e-122 |
| CAQ43077.1 | polyketide\_synthase | BGC0000970 | NRP+Polyketide:Modular type I polyketide | 32.0 | 27.5 | 425.0 | 3.49e-122 |
| QCP68968.1 | VatM | BGC0002296 | NRP+Polyketide | 32.0 | 23.5 | 434.0 | 4.95e-122 |
| AQW44873.1 | polyketide\_synthase | BGC0001761 | Polyketide | 34.0 | 23.3 | 431.0 | 6.62e-122 |
| BAB69198.1 | modular\_polyketide\_synthase | BGC0000117 | Polyketide | 31.0 | 26.5 | 432.0 | 7.14e-122 |
| QSV12662.1 | AvmD | BGC0002456 | Polyketide+NRP | 33.0 | 26.6 | 437.0 | 1.03e-121 |
| ACN64831.1 | PokM1 | BGC0001061 | Polyketide:Iterative type I polyketide+Polyketide:Type II polyketide+Saccharide:Hybrid/tailoring saccharide | 34.0 | 22.3 | 429.0 | 1.22e-121 |
| ACB46194.1 | polyketide\_synthase | BGC0000989 | NRP+Polyketide | 31.0 | 27.1 | 429.0 | 2.14e-121 |
| QCP68974.1 | VatV | BGC0002296 | NRP+Polyketide | 34.0 | 20.2 | 426.0 | 3.95e-121 |
| CAE46843.1 | Type\_I\_modular\_polyketide\_synthase | BGC0000103 | Polyketide | 31.0 | 25.8 | 434.0 | 7.87e-121 |
| CAE46851.1 | Type\_I\_modular\_polyketide\_synthase | BGC0000103 | Polyketide | 31.0 | 25.8 | 434.0 | 8.1e-121 |
| BAF02922.1 | type\_I\_polyketide\_synthase | BGC0000073 | Polyketide | 31.0 | 27.7 | 434.0 | 8.54e-121 |
| ALD82524.1 | polyketide\_synthase | BGC0001212 | NRP+Polyketide | 31.0 | 28.1 | 428.0 | 9.83e-121 |
| QGA70099.1 | type\_I\_polyketide\_synthase | BGC0002517 | Polyketide | 32.0 | 27.1 | 433.0 | 1.23e-120 |
| ADH04641.1 | TgaC | BGC0001051 | NRP+Polyketide:Modular type I polyketide | 32.0 | 23.2 | 432.0 | 1.86e-120 |
| QQZ01583.1 | PKS | BGC0002498 | Other | 31.0 | 27.7 | 432.0 | 2.04e-120 |
| BAW35616.1 | modular\_polyketide\_synthase | BGC0002357 | Polyketide+Other | 32.0 | 25.3 | 432.0 | 2.54e-120 |
| CAQ18828.1 | polyketide\_synthase | BGC0000954 | NRP+Polyketide:Modular type I polyketide | 34.0 | 22.5 | 428.0 | 3.86e-120 |
| CAQ43078.1 | polyketide\_synthase | BGC0000970 | NRP+Polyketide:Modular type I polyketide | 35.0 | 22.4 | 426.0 | 5.02e-120 |
| AAF19810.1 | MtaB | BGC0001024 | NRP+Polyketide:Modular type I polyketide | 34.0 | 22.7 | 430.0 | 6.54e-120 |
| BBM96639.1 | modular\_polyketide\_synthase | BGC0002452 | Polyketide | 31.0 | 28.0 | 430.0 | 6.62e-120 |
| AOE23578.1 | FoxBII | BGC0001598 | NRP+Polyketide | 34.0 | 22.9 | 430.0 | 7.69e-120 |
| ADN13832.1 | Polyketide\_Synthase | BGC0001164 | Polyketide:Modular type I polyketide | 33.0 | 21.9 | 429.0 | 7.85e-120 |
| AZH23789.1 | MgcI | BGC0001970 | NRP+Polyketide | 32.0 | 23.4 | 427.0 | 9.24e-120 |
| AAM70355.1 | CalO5 | BGC0000033 | Polyketide | 35.0 | 22.6 | 415.0 | 9.73e-120 |
| ABV91286.1 | type\_I\_modular\_polyketide\_synthase | BGC0000158 | Polyketide:Modular type I polyketide | 33.0 | 22.1 | 430.0 | 1.05e-119 |
| ADH04660.1 | TugD | BGC0001342 | NRP+Polyketide | 34.0 | 23.1 | 430.0 | 1.21e-119 |
| AZH23818.1 | MgiI | BGC0001971 | NRP+Polyketide | 31.0 | 24.5 | 426.0 | 1.6e-119 |
| AVV61979.1 | beta-ketoacyl\_synthase | BGC0001477 | NRP+Polyketide:Modular type I polyketide | 31.0 | 26.5 | 429.0 | 1.69e-119 |
| AAZ77673.1 | ChlB1 | BGC0000036 | Polyketide:Modular type I polyketide+Polyketide:Iterative type I polyketide+Saccharide:Oligosaccharide | 34.0 | 22.3 | 422.0 | 1.82e-119 |
| ABI94379.1 | tautomycetin\_biosynthetic\_PKS | BGC0000157 | Polyketide | 33.0 | 22.1 | 429.0 | 2.36e-119 |
| AIT55261.1 | polyketide\_synthase | BGC0000072 | Polyketide:Modular type I polyketide | 34.0 | 21.8 | 418.0 | 5.26e-119 |
| AEE88289.1 | CurA | BGC0000976 | NRP+Polyketide:Modular type I polyketide | 34.0 | 21.9 | 425.0 | 7.21e-119 |
| AAT70096.1 | CurA | BGC0001165 | NRP+Polyketide:Modular type I polyketide | 34.0 | 21.9 | 425.0 | 7.21e-119 |
| ADC79618.1 | BafAIII | BGC0000028 | Polyketide:Modular type I polyketide | 34.0 | 22.6 | 427.0 | 7.28e-119 |
| BAW35655.1 | modular\_polyketide\_synthase | BGC0002355 | Polyketide+Other | 33.0 | 22.1 | 426.0 | 1.57e-118 |
| BCB17030.1 | modular\_polyketide\_synthase | BGC0002523 | NRP | 31.0 | 26.6 | 426.0 | 1.57e-118 |
| AAQ84145.1 | Plm5 | BGC0000123 | Polyketide | 31.0 | 26.9 | 420.0 | 3.1e-118 |
| BAF85844.1 | modular\_polyketide\_synthase | BGC0000109 | Polyketide | 32.0 | 27.9 | 425.0 | 3.47e-118 |
| AGY62754.1 | EbeB | BGC0000051 | Polyketide | 32.0 | 26.6 | 422.0 | 3.56e-118 |
| SCN11950.1 | EbeB-type\_I\_polyketide\_synthase | BGC0001580 | Polyketide | 32.0 | 26.6 | 422.0 | 3.56e-118 |
| BAQ21940.1 | putative\_Type\_I\_polyketide\_synthase | BGC0001204 | Polyketide | 30.0 | 27.5 | 425.0 | 4.17e-118 |
| QNN81301.1 | IonAV | BGC0002446 | Polyketide | 32.0 | 25.3 | 424.0 | 4.2e-118 |
| BAF02926.1 | type\_I\_polyketide\_synthase | BGC0000073 | Polyketide | 32.0 | 26.8 | 424.0 | 4.57e-118 |
| ABW96540.1 | type\_I\_modular\_polyketide\_synthase | BGC0000159 | Polyketide:Modular type I polyketide | 31.0 | 26.4 | 424.0 | 6.33e-118 |
| BAW35633.1 | modular\_polyketide\_synthase | BGC0002356 | Polyketide+Other | 32.0 | 26.3 | 423.0 | 7.88e-118 |
| AEP40934.1 | polyketide\_synthase\_type\_I | BGC0000021 | Polyketide | 33.0 | 22.1 | 423.0 | 9.22e-118 |
| AKD43753.1 | HerB | BGC0001349 | NRP+Polyketide | 31.0 | 26.2 | 423.0 | 9.41e-118 |
| CAE02602.1 | polyketide\_synthase\_type\_I | BGC0000024 | Polyketide:Modular type I polyketide | 31.0 | 25.1 | 418.0 | 1.33e-117 |
| CAQ64691.1 | lasalocid\_modular\_polyketide\_synthase | BGC0000087 | Polyketide | 31.0 | 27.9 | 418.0 | 1.44e-117 |
| CAO98852.1 | polyketide\_synthase\_AufI | BGC0000023 | Polyketide:Modular type I polyketide | 25.0 | 63.4 | 421.0 | 1.64e-117 |
| CAQ34928.1 | polyketide\_synthase | BGC0000986 | NRP+Polyketide | 32.0 | 27.4 | 417.0 | 1.74e-117 |
| BAF02927.1 | type\_I\_polyketide\_synthase | BGC0000073 | Polyketide | 32.0 | 24.3 | 419.0 | 2.16e-117 |
| AWR88404.1 | putative\_beta-ketoacyl\_synthase | BGC0001522 | Polyketide | 31.0 | 27.8 | 422.0 | 3.06e-117 |
| QBM78307.1 | polyketide\_synthase | BGC0002542 | Polyketide+NRP | 34.0 | 20.9 | 421.0 | 3.44e-117 |
| ABY66019.1 | 6-methylsalicylic\_acid\_synthase | BGC0001008 | Polyketide:Iterative type I polyketide+Polyketide:Enediyne type I polyketide | 34.0 | 22.8 | 415.0 | 4.92e-117 |
| ACB37755.1 | putative\_type\_I\_polyketide\_synthase | BGC0000162 | Polyketide | 31.0 | 27.4 | 421.0 | 5.35e-117 |
| QKW94285.1 | short-chain\_dehydrogenase/reductase\_SDR | BGC0002342 | NRP+Polyketide | 32.0 | 25.5 | 419.0 | 5.94e-117 |
| ABI94380.1 | tautomycetin\_biosynthetic\_PKS | BGC0000157 | Polyketide | 34.0 | 22.3 | 421.0 | 6.12e-117 |
| ABK32256.1 | AmbB | BGC0000014 | Polyketide | 36.0 | 20.9 | 420.0 | 6.48e-117 |
| ABW96541.1 | type\_I\_modular\_polyketide\_synthase | BGC0000159 | Polyketide:Modular type I polyketide | 32.0 | 23.8 | 421.0 | 7.04e-117 |
| AAC38075.1 | polyketide\_synthase\_type\_I | BGC0000127 | Polyketide | 34.0 | 20.6 | 419.0 | 7.77e-117 |
| ACR33079.1 | polyketide\_synthase | BGC0000017 | Alkaloid+Polyketide:Modular type I polyketide | 34.0 | 20.8 | 412.0 | 8.98e-117 |
| BAW35656.1 | modular\_polyketide\_synthase | BGC0002355 | Polyketide+Other | 30.0 | 27.3 | 420.0 | 1.18e-116 |
| CAD19086.1 | StiB\_protein | BGC0000153 | NRP+Polyketide:Modular type I polyketide | 34.0 | 20.5 | 411.0 | 1.25e-116 |
| ACB46487.1 | polyketide\_synthase | BGC0000082 | Polyketide | 34.0 | 22.5 | 420.0 | 1.31e-116 |
| BCK51640.1 | modular\_polyketide\_synthase | BGC0002520 | Polyketide | 34.0 | 22.4 | 420.0 | 1.36e-116 |
| ABV91287.1 | type\_I\_modular\_polyketide\_synthase | BGC0000158 | Polyketide:Modular type I polyketide | 34.0 | 22.3 | 420.0 | 1.36e-116 |
| QEA08907.1 | JenA8 | BGC0002559 | Polyketide | 30.0 | 26.6 | 419.0 | 1.48e-116 |
| AWC08663.1 | polyketide\_synthase\_type\_I | BGC0001662 | Polyketide | 32.0 | 26.3 | 419.0 | 1.98e-116 |
| AMB20393.1 | polyketide\_synthase | BGC0002072 | Polyketide:Modular type I polyketide | 30.0 | 26.3 | 419.0 | 2.04e-116 |
| BAG85031.1 | putative\_polyketide\_synthase | BGC0000086 | Polyketide | 31.0 | 28.0 | 414.0 | 2.16e-116 |
| ADB12493.1 | EpoF | BGC0000990 | NRP+Polyketide | 33.0 | 22.8 | 417.0 | 2.23e-116 |
| QKG20136.1 | type\_I\_polyketide\_synthase | BGC0002124 | Polyketide | 31.0 | 26.4 | 418.0 | 2.7e-116 |
| BAF02921.1 | type\_I\_polyketide\_synthase | BGC0000073 | Polyketide | 35.0 | 20.1 | 418.0 | 2.84e-116 |
| ASZ00148.1 | polyketide\_synthase | BGC0001785 | Polyketide | 30.0 | 26.2 | 418.0 | 3.14e-116 |
| sipP2 | Type\_I\_Modular\_PKS | BGC0001452 | Polyketide | 31.0 | 27.6 | 418.0 | 3.46e-116 |
| AAF62885.1 | EpoF | BGC0000991 | NRP+Polyketide | 33.0 | 22.8 | 416.0 | 3.84e-116 |
| AVI26389.1 | polyketide\_synthase | BGC0001800 | NRP+Polyketide | 34.0 | 22.4 | 413.0 | 4.66e-116 |
| ACC80699.1 | beta-ketoacyl\_synthase | BGC0002677 | Other | 32.0 | 23.8 | 413.0 | 4.78e-116 |
| ACO94471.1 | polyketide\_synthase\_type\_I | BGC0000029 | Polyketide:Modular type I polyketide | 32.0 | 23.7 | 417.0 | 4.97e-116 |
| ACO94484.1 | polyketide\_synthase\_type\_I | BGC0000097 | Polyketide:Modular type I polyketide | 32.0 | 22.4 | 398.0 | 5.68e-116 |
| AVV61982.1 | type\_I\_modular\_PKS | BGC0001477 | NRP+Polyketide:Modular type I polyketide | 33.0 | 22.5 | 412.0 | 5.79e-116 |
| AKL64829.1 | polyketide\_synthase | BGC0002072 | Polyketide:Modular type I polyketide | 31.0 | 27.4 | 417.0 | 6.79e-116 |
| QGJ79676.1 | Polyketide\_synthase | BGC0002552 | Polyketide | 31.0 | 26.2 | 416.0 | 7.31e-116 |
| WP\_033261453.1 | type\_I\_polyketide\_synthase | BGC0002009 | Polyketide | 31.0 | 23.5 | 412.0 | 7.78e-116 |
| QPP46758.1 | polyketide\_synthase | BGC0002500 | Polyketide | 31.0 | 26.5 | 416.0 | 9.21e-116 |
| ADM46357.1 | polyketide\_synthase | BGC0000106 | Polyketide | 32.0 | 23.0 | 417.0 | 9.92e-116 |
| AHH34186.1 | polyketide\_synthase | BGC0001161 | Polyketide:Modular type I polyketide | 33.0 | 21.6 | 414.0 | 1.23e-115 |
| BAJ16468.1 | polyketide\_synthase | BGC0000058 | Polyketide | 31.0 | 27.0 | 416.0 | 1.33e-115 |
| ARW71485.1 | type\_I\_PKS\_module\_4,\_module\_5 | BGC0001812 | Polyketide | 30.0 | 27.3 | 416.0 | 1.66e-115 |
| AAZ94388.1 | nodular\_polyketide\_synthase | BGC0000040 | Polyketide | 31.0 | 27.6 | 416.0 | 2.12e-115 |
| AAZ94389.1 | modular\_polyketide\_synthase | BGC0000040 | Polyketide | 30.0 | 27.6 | 415.0 | 2.32e-115 |
| QQZ01629.1 | PKS | BGC0002497 | Other | 31.0 | 26.3 | 416.0 | 2.32e-115 |
| AXM42950.1 | polyketide\_synthase | BGC0001941 | NRP+Polyketide | 35.0 | 20.8 | 411.0 | 2.4e-115 |
| BAJ16470.1 | polyketide\_synthase | BGC0000058 | Polyketide | 32.0 | 23.5 | 415.0 | 2.75e-115 |
| AVI57433.1 | AbmB1 | BGC0001694 | Polyketide | 38.0 | 20.0 | 415.0 | 3.2e-115 |
| CQR60495.1 | Polyketide\_synthase,\_type\_I,\_module\_7 | BGC0001287 | Polyketide | 31.0 | 24.3 | 410.0 | 3.51e-115 |
| BAW35615.1 | modular\_polyketide\_synthase | BGC0002357 | Polyketide+Other | 33.0 | 22.3 | 415.0 | 3.94e-115 |
| QQZ01590.1 | PKS | BGC0002498 | Other | 32.0 | 25.9 | 411.0 | 4.03e-115 |
| QNN81300.1 | IonAIV | BGC0002446 | Polyketide | 31.0 | 26.6 | 414.0 | 4.25e-115 |
| AWS21279.1 | type\_I\_polyketide\_synthase | BGC0001934 | Polyketide | 35.0 | 20.3 | 404.0 | 4.26e-115 |
| AZY91989.1 | polyketide\_synthase | BGC0002022 | Polyketide | 35.0 | 20.3 | 404.0 | 4.26e-115 |
| AAF26923.1 | polyketide\_synthase | BGC0000988 | NRP+Polyketide | 33.0 | 22.8 | 413.0 | 4.43e-115 |
| QQZ01585.1 | PKS | BGC0002498 | Other | 32.0 | 23.2 | 414.0 | 4.89e-115 |
| ARV85763.1 | PieA4\_type\_I\_PKS | BGC0001742 | Polyketide | 31.0 | 26.7 | 412.0 | 5e-115 |
| AAP42873.1 | NanA11 | BGC0000105 | Polyketide | 32.0 | 26.8 | 412.0 | 5.59e-115 |
| QGJ79675.1 | Polyketide\_synthase | BGC0002552 | Polyketide | 34.0 | 22.9 | 414.0 | 5.67e-115 |
| WP\_051137606.1 | type\_I\_polyketide\_synthase | BGC0002011 | Polyketide | 31.0 | 26.2 | 413.0 | 6.35e-115 |
| ctg1\_13 |  | BGC0001931 | Polyketide | 31.0 | 27.1 | 414.0 | 6.46e-115 |
| AGY30677.1 | Ann5 | BGC0001298 | Polyketide | 33.0 | 22.7 | 414.0 | 6.54e-115 |
| ACB46471.1 | polyketide\_synthase | BGC0000082 | Polyketide | 31.0 | 26.9 | 414.0 | 6.69e-115 |
| BAQ25507.1 | type\_I\_polyketide\_synthase | BGC0001288 | Polyketide | 34.0 | 22.5 | 409.0 | 7.1e-115 |
| ACB46197.1 | polyketide\_synthase | BGC0000989 | NRP+Polyketide | 33.0 | 22.8 | 412.0 | 7.63e-115 |
| ACC40921.1 | polyketide\_synthase\_Pks7 | BGC0001665 | Polyketide | 33.0 | 22.2 | 411.0 | 7.71e-115 |
| TXD00033.1 | SDR\_family\_NAD(P)-dependent\_oxidoreductase | BGC0001877 | Polyketide | 33.0 | 23.2 | 414.0 | 8.09e-115 |
| SAI82896.1 | HrnA2;\_Starter\_unit\_polyketide\_synthase\_type\_I;\_module\_4\_(partial) | BGC0002101 | Polyketide | 31.0 | 23.8 | 394.0 | 8.4e-115 |
| AHH99919.1 | PKS\_I | BGC0000002 | Polyketide | 31.0 | 26.5 | 413.0 | 1.08e-114 |
| BAW35634.1 | modular\_polyketide\_synthase | BGC0002356 | Polyketide+Other | 33.0 | 22.4 | 413.0 | 1.51e-114 |
| AWC08659.1 | polyketide\_synthase\_type\_I | BGC0001662 | Polyketide | 31.0 | 26.6 | 412.0 | 1.86e-114 |
| AMB20394.1 | polyketide\_synthase | BGC0002072 | Polyketide:Modular type I polyketide | 32.0 | 25.9 | 412.0 | 2.45e-114 |
| ABY21540.1 | AngAIII | BGC0000018 | Polyketide | 31.0 | 27.9 | 412.0 | 2.52e-114 |
| AFV30247.1 | polyketide\_synthase | BGC0000075 | Polyketide | 30.0 | 27.2 | 412.0 | 2.53e-114 |
| AWC08662.1 | polyketide\_synthase\_type\_I | BGC0001662 | Polyketide | 33.0 | 22.4 | 412.0 | 2.58e-114 |
| WP\_157358234.1 | SDR\_family\_NAD(P)-dependent\_oxidoreductase | BGC0002011 | Polyketide | 33.0 | 22.9 | 412.0 | 2.81e-114 |
| AUO16399.1 | polyketide\_synthase | BGC0001700 | Polyketide | 32.0 | 23.3 | 411.0 | 2.81e-114 |
| AHN85651.1 | Phn2 | BGC0000122 | Polyketide:Modular type I polyketide | 31.0 | 26.8 | 412.0 | 2.88e-114 |
| AFI57006.1 | QmnA2 | BGC0000133 | Polyketide | 31.0 | 27.4 | 406.0 | 3.06e-114 |
| AWR88405.1 | putative\_phosphopantetheine-binding\_domain-\_containing\_prot\_ein | BGC0001522 | Polyketide | 33.0 | 23.6 | 411.0 | 3.24e-114 |
| CAO98847.1 | polyketide\_synthase\_AufC | BGC0000023 | Polyketide:Modular type I polyketide | 36.0 | 20.9 | 407.0 | 3.48e-114 |
| ABK32259.1 | AmbE | BGC0000014 | Polyketide | 32.0 | 26.3 | 411.0 | 3.87e-114 |
| CAO85896.1 | protein\_modular\_polyketide\_synthase\_NorA' | BGC0000110 | Polyketide:Modular type I polyketide | 31.0 | 26.5 | 411.0 | 4.03e-114 |
| ACC80701.1 | beta-ketoacyl\_synthase | BGC0002677 | Other | 34.0 | 20.4 | 407.0 | 4.12e-114 |
| ASZ00150.1 | polyketide\_synthase | BGC0001785 | Polyketide | 30.0 | 26.4 | 407.0 | 4.23e-114 |
| AAM77986.1 | iterative\_type\_I\_polyketide\_synthase | BGC0000112 | Polyketide:Iterative type I polyketide+Polyketide:Enediyne type I polyketide | 33.0 | 22.2 | 405.0 | 4.65e-114 |
| CAM00064.1 | EryAII\_Erythromycin\_polyketide\_synthase\_modules\_3\_and\_4 | BGC0000055 | Polyketide:Modular type I polyketide+Saccharide:Hybrid/tailoring saccharide | 33.0 | 22.7 | 410.0 | 5.17e-114 |
| AEZ64503.1 | Herd | BGC0001065 | Polyketide | 33.0 | 22.3 | 407.0 | 6.41e-114 |
| AVV61989.1 | beta-ketoacyl\_synthase | BGC0001477 | NRP+Polyketide:Modular type I polyketide | 30.0 | 27.9 | 407.0 | 6.49e-114 |
| AAG23262.1 | polyketide\_synthase\_extender\_modules\_8-10 | BGC0000148 | Polyketide | 30.0 | 27.4 | 410.0 | 7.45e-114 |
| QQZ01586.1 | PKS | BGC0002498 | Other | 30.0 | 26.3 | 410.0 | 8.41e-114 |
| BAO66542.1 | type\_I\_polyketide\_synthase | BGC0000042 | Polyketide | 31.0 | 26.5 | 410.0 | 9.36e-114 |
| AZH23793.1 | MgcK | BGC0001970 | NRP+Polyketide | 33.0 | 21.1 | 396.0 | 1.11e-113 |
| ATG32075.1 | polyketide\_synthase | BGC0001750 | NRP+Polyketide | 31.0 | 27.9 | 405.0 | 1.23e-113 |
| CQR60496.1 | Polyketide\_synthase,\_type\_I,\_modules:\_4,\_5\_and\_6 | BGC0001287 | Polyketide | 30.0 | 25.5 | 410.0 | 1.25e-113 |
| BAW35659.1 | modular\_polyketide\_synthase | BGC0002355 | Polyketide+Other | 31.0 | 27.2 | 409.0 | 1.28e-113 |
| BAB69194.1 | modular\_polyketide\_synthase | BGC0000117 | Polyketide | 32.0 | 27.4 | 409.0 | 1.69e-113 |
| CAE45669.1 | borrelidin\_polyketide\_synthase,\_type\_I | BGC0000031 | Polyketide:Modular type I polyketide | 33.0 | 24.4 | 409.0 | 1.94e-113 |
| BCK51645.1 | modular\_polyketide\_synthase | BGC0002520 | Polyketide | 36.0 | 20.2 | 409.0 | 1.96e-113 |
| AHH34189.1 | polyketide\_synthase | BGC0001162 | Polyketide:Modular type I polyketide | 32.0 | 21.9 | 407.0 | 1.98e-113 |
| AAF71775.1 | nysB | BGC0000115 | Polyketide:Modular type I polyketide+Saccharide:Hybrid/tailoring saccharide | 35.0 | 20.8 | 408.0 | 2.23e-113 |
| AAX98191.1 | polyketide\_synthase\_type\_I | BGC0000052 | Polyketide | 30.0 | 27.4 | 409.0 | 2.48e-113 |
| AAK83194.1 | polyketide\_synthase | BGC0000026 | Saccharide:Oligosaccharide | 32.0 | 24.2 | 396.0 | 2.61e-113 |
| BCK51646.1 | modular\_polyketide\_synthase | BGC0002520 | Polyketide | 33.0 | 22.2 | 408.0 | 2.67e-113 |
| CAQ34920.1 | polyketide\_synthase | BGC0000986 | NRP+Polyketide | 34.0 | 22.9 | 404.0 | 2.69e-113 |
| ANH11412.1 | SceQ | BGC0001770 | Polyketide | 32.0 | 22.4 | 404.0 | 3.26e-113 |
| ADC79620.1 | BafAV | BGC0000028 | Polyketide:Modular type I polyketide | 33.0 | 22.6 | 405.0 | 3.42e-113 |
| AAM81586.2 | putative\_type\_I\_polyketide\_synthase | BGC0000047 | Polyketide | 30.0 | 27.7 | 408.0 | 3.58e-113 |
| QQZ01588.1 | PKS | BGC0002498 | Other | 29.0 | 26.8 | 408.0 | 3.79e-113 |
| ABV97154.1 | Beta-ketoacyl\_synthase | BGC0000137 | Polyketide | 32.0 | 23.8 | 403.0 | 4.44e-113 |
| ASZ00151.1 | polyketide\_synthase | BGC0001785 | Polyketide | 33.0 | 22.3 | 407.0 | 4.45e-113 |
| sipP1 | Type\_I\_Modular\_PKS | BGC0001452 | Polyketide | 32.0 | 24.2 | 407.0 | 4.8e-113 |
| ADH04659.1 | TugC | BGC0001342 | NRP+Polyketide | 31.0 | 24.6 | 407.0 | 5.61e-113 |
| AWC08657.1 | polyketide\_synthase\_type\_I | BGC0001662 | Polyketide | 32.0 | 23.1 | 408.0 | 5.75e-113 |
| AGI99482.1 | Type\_I\_polyketide\_synthase | BGC0001004 | Polyketide:Modular type I polyketide | 30.0 | 26.9 | 407.0 | 6.47e-113 |
| QFU80887.1 | PKS | BGC0002550 | Polyketide | 30.0 | 26.9 | 407.0 | 6.47e-113 |
| ALV82320.1 | borrelidin\_type\_I\_polyketide\_synthase | BGC0001533 | Polyketide | 33.0 | 24.4 | 407.0 | 7.45e-113 |
| ARM20280.1 | polyketide\_synthase | BGC0001523 | Polyketide | 33.0 | 22.7 | 407.0 | 7.48e-113 |
| BAQ21948.1 | putative\_type\_I\_polyketide\_synthase | BGC0001204 | Polyketide | 35.0 | 20.6 | 399.0 | 7.9e-113 |
| AKL64834.1 | polyketide\_synthase | BGC0002072 | Polyketide:Modular type I polyketide | 35.0 | 20.1 | 407.0 | 8.12e-113 |
| AAZ77698.1 | ChlA5 | BGC0000036 | Polyketide:Modular type I polyketide+Polyketide:Iterative type I polyketide+Saccharide:Oligosaccharide | 31.0 | 26.4 | 407.0 | 8.47e-113 |
| QIQ28617.1 | Nbc21 | BGC0002541 | Other | 33.0 | 21.1 | 407.0 | 1.03e-112 |
| AFV30250.1 | polyketide\_synthase | BGC0000075 | Polyketide | 32.0 | 22.7 | 404.0 | 1.04e-112 |
| BAF85839.1 | modular\_polyketide\_synthase | BGC0000109 | Polyketide | 31.0 | 25.4 | 407.0 | 1.13e-112 |
| ARO38317.1 | nonribosomal\_peptide\_synthetase | BGC0001560 | NRP+Polyketide | 27.0 | 39.1 | 407.0 | 1.17e-112 |
| QNN81302.1 | IonAVI | BGC0002446 | Polyketide | 30.0 | 26.2 | 406.0 | 1.2e-112 |
| QRI43530.1 | type\_I\_polyketide\_synthase | BGC0002454 | Polyketide | 31.0 | 26.6 | 406.0 | 1.38e-112 |
| AEP40936.1 | polyketide\_synthase\_type\_I | BGC0000021 | Polyketide | 33.0 | 22.2 | 406.0 | 1.38e-112 |
| AAY42396.1 | Polyketide\_synthase | BGC0001000 | NRP:Lipopeptide+Polyketide:Modular type I polyketide | 33.0 | 20.8 | 402.0 | 1.4e-112 |
| ALV82345.1 | borrelidin\_type\_I\_polyketide\_synthase | BGC0001533 | Polyketide | 34.0 | 22.3 | 404.0 | 1.49e-112 |
| AFV96142.1 | polyketide\_synthase | BGC0001064 | Polyketide:Modular type I polyketide+Polyketide:Type III polyketide | 33.0 | 20.1 | 404.0 | 1.49e-112 |
| ARU81122.1 | CylH | BGC0001566 | Polyketide | 33.0 | 20.1 | 404.0 | 1.49e-112 |
| QDA77044.1 | polyketide\_synthase | BGC0002025 | NRP+Polyketide | 35.0 | 20.7 | 406.0 | 1.52e-112 |
| ANR02556.1 | LodO | BGC0001648 | Polyketide | 30.0 | 27.0 | 402.0 | 1.62e-112 |
| AMB48441.1 | polyketide\_synthase | BGC0001357 | Polyketide | 32.0 | 20.6 | 403.0 | 1.62e-112 |
| QQZ01628.1 | PKS | BGC0002497 | Other | 32.0 | 22.7 | 405.0 | 1.75e-112 |
| AAD03047.1 | type\_I\_polyketide\_synthase | BGC0000041 | Polyketide | 33.0 | 22.2 | 405.0 | 1.87e-112 |
| ADC79619.1 | BafAIV | BGC0000028 | Polyketide:Modular type I polyketide | 31.0 | 26.8 | 405.0 | 2.19e-112 |
| ctg1\_orf522 |  | BGC0001199 | Polyketide | 31.0 | 26.6 | 405.0 | 2.26e-112 |
| OJF16269.1 | AceP2 | BGC0001491 | Polyketide | 32.0 | 23.2 | 405.0 | 2.28e-112 |
| AAX98188.1 | polyketide\_synthase\_type\_I | BGC0000052 | Polyketide | 34.0 | 22.1 | 405.0 | 2.34e-112 |
| AAO65806.1 | monensin\_polyketide\_synthase\_modules\_11\_and\_12 | BGC0000100 | Polyketide | 30.0 | 27.3 | 405.0 | 2.37e-112 |
| ANZ52469.1 | MonAVIII | BGC0001670 | Polyketide | 30.0 | 27.3 | 405.0 | 2.37e-112 |
| BAW35613.1 | modular\_polyketide\_synthase | BGC0002357 | Polyketide+Other | 30.0 | 26.9 | 405.0 | 2.39e-112 |
| AUO16397.1 | polyketide\_synthase | BGC0001700 | Polyketide | 31.0 | 26.3 | 405.0 | 2.49e-112 |
| CAE45671.1 | borrelidin\_polyketide\_synthase,\_type\_I | BGC0000031 | Polyketide:Modular type I polyketide | 34.0 | 22.3 | 403.0 | 2.79e-112 |
| AAZ77693.1 | ChlA1 | BGC0000036 | Polyketide:Modular type I polyketide+Polyketide:Iterative type I polyketide+Saccharide:Oligosaccharide | 30.0 | 26.7 | 405.0 | 2.87e-112 |
| EGJ35088.1 | Polyketide\_synthase | BGC0001163 | Polyketide:Modular type I polyketide | 32.0 | 22.1 | 403.0 | 2.92e-112 |
| BAW35637.1 | modular\_polyketide\_synthase | BGC0002356 | Polyketide+Other | 33.0 | 22.4 | 405.0 | 3.05e-112 |
| AKG06375.1 | polyketide\_synthase\_type\_1 | BGC0001830 | Polyketide | 34.0 | 21.5 | 405.0 | 3.59e-112 |
| EYT83439.1 | beta-ketoacyl\_synthase | BGC0001213 | Polyketide | 34.0 | 22.1 | 399.0 | 3.89e-112 |
| AEZ54375.1 | PieA2 | BGC0000124 | Polyketide | 32.0 | 23.7 | 404.0 | 4.68e-112 |
| ADH04640.1 | TgaB | BGC0001051 | NRP+Polyketide:Modular type I polyketide | 34.0 | 20.4 | 404.0 | 4.77e-112 |
| CAQ18834.1 | polyketide\_synthase | BGC0000954 | NRP+Polyketide:Modular type I polyketide | 35.0 | 20.4 | 404.0 | 4.93e-112 |
| QRI43527.1 | type\_I\_polyketide\_synthase | BGC0002454 | Polyketide | 31.0 | 26.5 | 404.0 | 5.09e-112 |
| AVV61984.1 | type\_I\_modular\_polyketide\_synthase | BGC0001477 | NRP+Polyketide:Modular type I polyketide | 33.0 | 22.3 | 404.0 | 5.15e-112 |
| ARV85760.1 | PieA1\_type\_I\_PKS | BGC0001742 | Polyketide | 32.0 | 22.6 | 403.0 | 5.22e-112 |
| BAR73020.1 | putative\_PKS\_(KS-AT-DH-KR-ACP-KS-AT-DH-KR-ACP-KS-AT-DH-KR-ACP) | BGC0001194 | Polyketide | 31.0 | 26.5 | 404.0 | 6.84e-112 |
| WP\_051137607.1 | type\_I\_polyketide\_synthase | BGC0002011 | Polyketide | 33.0 | 22.5 | 403.0 | 7.55e-112 |
| ARM20278.1 | polyketide\_synthase | BGC0001523 | Polyketide | 30.0 | 27.3 | 403.0 | 7.98e-112 |
| ASZ00149.1 | polyketide\_synthase | BGC0001785 | Polyketide | 32.0 | 22.3 | 403.0 | 8.58e-112 |
| CQR60494.1 | Polyketide\_synthase,\_type\_I,\_module\_8 | BGC0001287 | Polyketide | 32.0 | 22.1 | 399.0 | 8.75e-112 |
| QFU19826.1 | PKS | BGC0002431 | Polyketide+Saccharide | 30.0 | 25.8 | 401.0 | 8.91e-112 |
| ABV97152.1 | Beta-ketoacyl\_synthase | BGC0000137 | Polyketide | 31.0 | 26.6 | 404.0 | 9.02e-112 |
| QGJ79644.1 | Polyketide\_synthase | BGC0002552 | Polyketide | 33.0 | 22.6 | 403.0 | 9.6e-112 |
| ARE67852.1 | AbsB2 | BGC0001492 | Polyketide | 30.0 | 27.3 | 403.0 | 1.15e-111 |
| BAD08373.1 | polyketide\_synthase\_modules\_1-3 | BGC0000167 | Polyketide | 33.0 | 22.8 | 403.0 | 1.25e-111 |
| BAG23202.1 | putative\_type-I\_PKS | BGC0002673 | Polyketide+Alkaloid | 31.0 | 26.1 | 402.0 | 1.28e-111 |
| QEA08887.1 | JenA1 | BGC0002559 | Polyketide | 33.0 | 22.2 | 402.0 | 1.39e-111 |
| ADF88276.1 | polyketide\_synthase | BGC0000981 | NRP+Polyketide | 33.0 | 20.1 | 399.0 | 1.49e-111 |
| CAI94713.1 | putative\_polyketide\_synthase | BGC0000141 | Polyketide | 33.0 | 22.6 | 403.0 | 1.51e-111 |
| AWH12664.1 | RmpE2 | BGC0001759 | Polyketide | 32.0 | 22.1 | 401.0 | 1.85e-111 |
| QBF51755.1 | type\_I\_polyketide\_synthase | BGC0001856 | Polyketide:Modular type I polyketide | 34.0 | 20.4 | 402.0 | 1.95e-111 |
| ARM20284.1 | polyketide\_synthase | BGC0001523 | Polyketide | 30.0 | 26.8 | 400.0 | 2.06e-111 |
| AFI57005.1 | QmnA1 | BGC0000133 | Polyketide | 32.0 | 24.0 | 402.0 | 2.15e-111 |
| AVV61981.1 | type\_I\_modular\_PKS | BGC0001477 | NRP+Polyketide:Modular type I polyketide | 32.0 | 22.8 | 402.0 | 2.51e-111 |
| ACN69990.1 | polyketide\_synthase | BGC0000079 | Polyketide | 35.0 | 20.1 | 402.0 | 2.63e-111 |
| WP\_159041997.1 | SDR\_family\_NAD(P)-dependent\_oxidoreductase | BGC0002033 | Polyketide | 33.0 | 23.2 | 402.0 | 2.64e-111 |
| TGZ15167.1 | polyketide\_synthase | BGC0002032 | Polyketide | 30.0 | 26.8 | 401.0 | 3.34e-111 |
| ctg1\_orf29 |  | BGC0000096 | Polyketide | 30.0 | 27.1 | 401.0 | 3.42e-111 |
| BAE93731.1 | type\_I\_polyketide\_synthase | BGC0000164 | Polyketide | 31.0 | 25.5 | 402.0 | 3.69e-111 |
| ctg1\_orf521 |  | BGC0001199 | Polyketide | 31.0 | 26.2 | 401.0 | 4.33e-111 |
| ALP32045.1 | CycE | BGC0001293 | Polyketide | 33.0 | 23.1 | 401.0 | 4.34e-111 |
| AUO16402.1 | polyketide\_synthase | BGC0001700 | Polyketide | 34.0 | 20.4 | 400.0 | 4.9e-111 |
| BAW35610.1 | modular\_polyketide\_synthase | BGC0002357 | Polyketide+Other | 32.0 | 23.2 | 401.0 | 5.08e-111 |
| AGZ15473.1 | putative\_type\_I\_polyketide\_synthase | BGC0001036 | NRP+Polyketide | 33.0 | 22.6 | 397.0 | 5.17e-111 |
| QSV12663.1 | AvmE | BGC0002456 | Polyketide+NRP | 32.0 | 24.0 | 401.0 | 5.2e-111 |
| ABV97153.1 | Beta-ketoacyl\_synthase | BGC0000137 | Polyketide | 31.0 | 24.2 | 397.0 | 5.32e-111 |
| ADM46359.1 | polyketide\_synthase | BGC0000106 | Polyketide | 30.0 | 26.1 | 400.0 | 5.73e-111 |
| CAO85893.1 | modular\_polyketide\_synthase\_NorA | BGC0000110 | Polyketide:Modular type I polyketide | 31.0 | 26.1 | 397.0 | 5.96e-111 |
| ADC45538.1 | modular\_polyketide\_synthase | BGC0000093 | Polyketide | 30.0 | 26.7 | 401.0 | 6.23e-111 |
| BAE93730.1 | type\_I\_polyketide\_synthase | BGC0000164 | Polyketide | 30.0 | 26.6 | 400.0 | 8.03e-111 |
| ADC45534.1 | modular\_polyketide\_synthase | BGC0000093 | Polyketide | 30.0 | 26.6 | 400.0 | 8.26e-111 |
| QQZ01626.1 | PKS | BGC0002497 | Other | 30.0 | 26.3 | 400.0 | 8.31e-111 |
| ABB05105.1 | LipPks4 | BGC0001003 | NRP:Lipopeptide+Polyketide:Modular type I polyketide+Saccharide:Hybrid/tailoring saccharide | 33.0 | 22.2 | 400.0 | 8.66e-111 |
| AJW65409.1 | type\_I\_modular\_polyketide\_synthase | BGC0001195 | NRP+Polyketide | 30.0 | 27.2 | 400.0 | 9.72e-111 |
| ATY12793.1 | type\_I\_polyketide\_synthase | BGC0001504 | Polyketide | 32.0 | 25.4 | 397.0 | 1.16e-110 |
| QGA70078.1 | type\_I\_polyketide\_synthase | BGC0002517 | Polyketide | 30.0 | 26.3 | 399.0 | 1.24e-110 |
| QEA08906.1 | JenA11 | BGC0002559 | Polyketide | 34.0 | 22.8 | 397.0 | 1.34e-110 |
| AKJ15835.1 | type\_I\_polyketide\_synthase | BGC0002735 | Polyketide+NRP | 33.0 | 22.3 | 395.0 | 1.45e-110 |
| BCK51638.1 | modular\_polyketide\_synthase | BGC0002520 | Polyketide | 32.0 | 22.4 | 400.0 | 1.46e-110 |
| ANC94964.1 | AlmHIII | BGC0001396 | Polyketide | 30.0 | 27.7 | 399.0 | 1.71e-110 |
| ARE67853.1 | AbsB1 | BGC0001492 | Polyketide | 34.0 | 22.8 | 399.0 | 1.9e-110 |
| QKG20145.1 | type\_I\_polyketide\_synthase | BGC0002124 | Polyketide | 33.0 | 22.5 | 399.0 | 2e-110 |
| BAW35639.1 | modular\_polyketide\_synthase | BGC0002356 | Polyketide+Other | 32.0 | 23.2 | 399.0 | 2.09e-110 |
| AAZ94391.1 | modular\_polyketide\_synthase | BGC0000040 | Polyketide | 32.0 | 22.8 | 396.0 | 2.14e-110 |
| BAG85029.1 | putative\_polyketide\_synthase | BGC0000086 | Polyketide | 34.0 | 20.3 | 393.0 | 2.16e-110 |
| BAT51066.1 | type\_I\_polyketide\_synthase | BGC0001296 | Polyketide | 31.0 | 24.4 | 399.0 | 2.18e-110 |
| ATX68116.1 | malonyl\_CoA-acyl\_carrier\_protein\_transacylase | BGC0001772 | Polyketide | 32.0 | 21.3 | 397.0 | 2.25e-110 |
| AQT01382.1 | SgnS1 | BGC0001690 | Polyketide | 33.0 | 22.4 | 399.0 | 2.55e-110 |
| QLD23491.1 | Polyketide\_synthase | BGC0002085 | Saccharide:Oligosaccharide | 33.0 | 22.3 | 386.0 | 2.66e-110 |
| QWF78548.1 | 3-ketoacyl-CoA\_thiolase | BGC0002142 | Polyketide | 34.0 | 20.0 | 399.0 | 2.69e-110 |
| QOD94998.1 | PldAIII | BGC0002102 | Polyketide | 30.0 | 26.3 | 399.0 | 2.71e-110 |
| TXD00261.1 | AMP-binding\_protein | BGC0001877 | Polyketide | 31.0 | 26.8 | 397.0 | 2.79e-110 |
| ABB05104.1 | LipPks3 | BGC0001003 | NRP:Lipopeptide+Polyketide:Modular type I polyketide+Saccharide:Hybrid/tailoring saccharide | 32.0 | 23.7 | 398.0 | 2.87e-110 |
| CAQ64689.1 | lasalocid\_modular\_polyketide\_synthase | BGC0000087 | Polyketide | 34.0 | 20.3 | 392.0 | 2.88e-110 |
| AEH42491.1 | polyketide\_synthase | BGC0000032 | Polyketide | 30.0 | 26.3 | 398.0 | 3.14e-110 |
| BAH02269.1 | polyketide\_synthase | BGC0000126 | Polyketide | 30.0 | 26.3 | 399.0 | 3.37e-110 |
| QCP68966.1 | VatW | BGC0002296 | NRP+Polyketide | 32.0 | 20.8 | 397.0 | 3.55e-110 |
| AEZ53951.1 | polyketide\_synthase | BGC0000144 | Polyketide:Modular type I polyketide | 33.0 | 21.3 | 392.0 | 3.72e-110 |
| QRI43529.1 | type\_I\_polyketide\_synthase | BGC0002454 | Polyketide | 31.0 | 26.8 | 398.0 | 3.96e-110 |
| QBG82532.1 | Polyketide\_synthase | BGC0002587 | Polyketide | 34.0 | 20.2 | 397.0 | 4.88e-110 |
| UHY14125.1 | PKS\_I | BGC0002671 | Polyketide | 34.0 | 22.2 | 397.0 | 5.26e-110 |
| AVI57434.1 | AbmB2 | BGC0001694 | Polyketide | 33.0 | 22.3 | 397.0 | 5.5e-110 |
| ATY46595.1 | polyketide\_synthase | BGC0001666 | Polyketide | 30.0 | 27.3 | 393.0 | 5.96e-110 |
| QIQ28639.1 | Nbc43 | BGC0002541 | Other | 33.0 | 20.6 | 397.0 | 6.76e-110 |
| AUO16423.1 | polyketide\_synthase | BGC0001700 | Polyketide | 30.0 | 27.2 | 395.0 | 7.2e-110 |
| QBG82518.1 | Polyketide\_synthase | BGC0002587 | Polyketide | 33.0 | 22.2 | 395.0 | 7.78e-110 |
| BAC68128.1 | modular\_polyketide\_synthase | BGC0000059 | Polyketide | 33.0 | 22.9 | 397.0 | 8.28e-110 |
| QBF51769.1 | type\_I\_polyketide\_synthase | BGC0001856 | Polyketide:Modular type I polyketide | 31.0 | 27.5 | 395.0 | 8.32e-110 |
| BAW35612.1 | modular\_polyketide\_synthase | BGC0002357 | Polyketide+Other | 30.0 | 26.8 | 397.0 | 8.56e-110 |
| AEH42474.1 | polyketide\_synthase | BGC0000032 | Polyketide | 33.0 | 21.7 | 394.0 | 8.68e-110 |
| QPP46757.1 | polyketide\_synthase | BGC0002500 | Polyketide | 30.0 | 26.4 | 395.0 | 8.91e-110 |
| AAC01712.2 | RifC | BGC0000136 | Polyketide | 31.0 | 22.1 | 392.0 | 9.74e-110 |
| ANZ22985.1 | ZinB | BGC0001828 | Polyketide | 35.0 | 22.4 | 397.0 | 1.11e-109 |
| BAD08358.1 | polyketide\_synthase\_modules\_4 | BGC0000167 | Polyketide | 32.0 | 23.3 | 395.0 | 1.13e-109 |
| SCN11952.1 | ebeD-type\_I\_polyketide\_synthase | BGC0001580 | Polyketide | 30.0 | 27.0 | 396.0 | 1.2e-109 |
| QFU19841.1 | PKS | BGC0002431 | Polyketide+Saccharide | 30.0 | 27.7 | 396.0 | 1.23e-109 |
| CAN89636.1 | putative\_polyketide\_synthase | BGC0001070 | NRP+Polyketide:Modular type I polyketide+Polyketide:Trans-AT type I polyketide | 33.0 | 22.4 | 395.0 | 1.38e-109 |
| ACN69989.1 | polyketide\_synthase | BGC0000079 | Polyketide | 30.0 | 27.2 | 396.0 | 1.43e-109 |
| TXD00024.1 | SDR\_family\_NAD(P)-dependent\_oxidoreductase | BGC0001877 | Polyketide | 32.0 | 22.2 | 396.0 | 1.45e-109 |
| AEZ54377.1 | PieA4 | BGC0000124 | Polyketide | 32.0 | 22.6 | 394.0 | 1.47e-109 |
| AAP42874.1 | NanA8 | BGC0000105 | Polyketide | 30.0 | 27.9 | 395.0 | 1.79e-109 |
| ANY10590.1 | polyketide\_synthase | BGC0001773 | Polyketide | 34.0 | 22.1 | 395.0 | 2.12e-109 |
| ctg1\_orf28 |  | BGC0000096 | Polyketide | 30.0 | 27.4 | 392.0 | 2.12e-109 |
| BBA66513.1 | type\_I\_polyketide\_synthase | BGC0001495 | Polyketide | 34.0 | 21.3 | 395.0 | 2.21e-109 |
| BAH02270.1 | polyketide\_synthase | BGC0000126 | Polyketide | 29.0 | 26.6 | 395.0 | 2.54e-109 |
| QOD94999.1 | PldAIV | BGC0002102 | Polyketide | 29.0 | 26.6 | 395.0 | 2.54e-109 |
| ABK32258.1 | AmbD | BGC0000014 | Polyketide | 31.0 | 23.9 | 392.0 | 2.86e-109 |
| ARM20283.1 | polyketide\_synthase | BGC0001523 | Polyketide | 31.0 | 23.5 | 393.0 | 3.05e-109 |
| AHH99926.1 | PKS\_I | BGC0000002 | Polyketide | 30.0 | 26.2 | 395.0 | 3.39e-109 |
| BAJ16471.1 | polyketide\_synthase | BGC0000058 | Polyketide | 30.0 | 27.1 | 395.0 | 3.4e-109 |
| ACY13414.1 | amino\_acid\_adenylation\_domain\_protein | BGC0001367 | NRP+Polyketide | 33.0 | 22.9 | 395.0 | 3.7e-109 |
| QRI43528.1 | type\_I\_polyketide\_synthase | BGC0002454 | Polyketide | 30.0 | 26.4 | 393.0 | 3.71e-109 |
| SCO70309.1 | Type\_I\_polyketide\_synthase | BGC0001433 | Polyketide:Modular type I polyketide | 31.0 | 26.7 | 394.0 | 4.17e-109 |
| BBA66512.1 | type\_I\_polyketide\_synthase | BGC0001495 | Polyketide | 33.0 | 22.4 | 395.0 | 4.77e-109 |
| WP\_032929422.1 | type\_I\_polyketide\_synthase | BGC0002106 | Polyketide | 33.0 | 22.5 | 395.0 | 4.85e-109 |
| AWH12668.1 | RmpC | BGC0001759 | Polyketide | 32.0 | 22.1 | 389.0 | 6.18e-109 |
| AXI91546.1 | FunP7 | BGC0001944 | Polyketide | 32.0 | 24.7 | 394.0 | 6.49e-109 |
| UMP03507.1 | NmvAIV | BGC0002649 | NRP+Polyketide | 32.0 | 22.6 | 389.0 | 6.82e-109 |
| AAO65797.1 | monensin\_polyketide\_synthase\_module\_2 | BGC0000100 | Polyketide | 33.0 | 24.1 | 392.0 | 7.28e-109 |
| ANZ52460.1 | MonAII | BGC0001670 | Polyketide | 33.0 | 24.1 | 392.0 | 7.28e-109 |
| BAF85843.1 | modular\_polyketide\_synthase | BGC0000109 | Polyketide | 32.0 | 22.3 | 394.0 | 7.69e-109 |
| AVV61983.1 | type\_I\_modular\_polyketide\_synthase | BGC0001477 | NRP+Polyketide:Modular type I polyketide | 30.0 | 26.5 | 394.0 | 7.72e-109 |
| AAZ94390.1 | modular\_polyketide\_synthase | BGC0000040 | Polyketide | 33.0 | 21.1 | 394.0 | 9.4e-109 |
| QQZ01589.1 | PKS | BGC0002498 | Other | 32.0 | 22.9 | 390.0 | 9.67e-109 |
| AAC69329.1 | type\_I\_polyketide\_synthase\_PikAI | BGC0000094 | Polyketide:Modular type I polyketide+Saccharide:Hybrid/tailoring saccharide | 30.0 | 28.0 | 393.0 | 1.17e-108 |
| WP\_102918845.1 | type\_I\_polyketide\_synthase | BGC0002104 | NRP+Polyketide | 33.0 | 22.3 | 394.0 | 1.17e-108 |
| BCB17026.1 | modular\_polyketide\_synthase | BGC0002523 | NRP | 33.0 | 22.8 | 393.0 | 1.26e-108 |
| AAQ82564.1 | FscC | BGC0000034 | NRP+Polyketide | 31.0 | 26.2 | 393.0 | 1.62e-108 |
| BCK51637.1 | modular\_modular\_polyketide\_synthase | BGC0002520 | Polyketide | 30.0 | 26.9 | 389.0 | 1.63e-108 |
| ACR50774.1 | polyketide\_synthase | BGC0000163 | Polyketide | 30.0 | 26.7 | 393.0 | 1.71e-108 |
| ABB86410.1 | GelC | BGC0000067 | Polyketide | 31.0 | 23.5 | 392.0 | 1.74e-108 |
| AAO06918.1 | GdmAIII | BGC0000066 | Polyketide | 30.0 | 23.4 | 392.0 | 1.74e-108 |
| ACR50775.1 | polyketide\_synthase | BGC0000163 | Polyketide | 30.0 | 26.7 | 393.0 | 1.77e-108 |
| WP\_055469549.1 | type\_I\_polyketide\_synthase | BGC0001537 | Polyketide | 33.0 | 22.6 | 392.0 | 2.02e-108 |
| QKG20147.1 | type\_I\_polyketide\_synthase | BGC0002124 | Polyketide | 31.0 | 22.3 | 392.0 | 2.24e-108 |
| QKG20146.1 | Type\_I\_polyketide\_synthase | BGC0002124 | Polyketide | 31.0 | 23.3 | 392.0 | 2.25e-108 |
| ADX66472.1 | ScnS1 | BGC0000108 | Polyketide | 32.0 | 22.5 | 392.0 | 2.44e-108 |
| AAX98184.1 | polyketide\_synthase\_type\_I | BGC0000052 | Polyketide | 33.0 | 20.5 | 392.0 | 2.59e-108 |
| ACC40922.1 | polyketide\_synthase,\_Pks8 | BGC0001665 | Polyketide | 33.0 | 22.3 | 390.0 | 2.75e-108 |
| BAR73007.1 | putative\_PKS\_(ACP-KS-AT-DH-KR-ACP-KS-AT-DH-ER-KR-ACP) | BGC0001194 | Polyketide | 32.0 | 23.6 | 392.0 | 3.05e-108 |
| ctg1\_orf255 |  | BGC0001200 | Polyketide | 30.0 | 26.6 | 390.0 | 3.25e-108 |
| AEP40935.1 | polyketide\_synthase\_type\_I | BGC0000021 | Polyketide | 34.0 | 20.3 | 391.0 | 3.28e-108 |
| QKV49790.1 | PKS | BGC0002526 | Polyketide | 30.0 | 25.8 | 389.0 | 3.32e-108 |
| AAG23266.1 | polyketide\_synthase\_extender\_modules\_3-4 | BGC0000148 | Polyketide | 34.0 | 21.2 | 391.0 | 4.04e-108 |
| AGC09485.1 | LobS2 | BGC0001183 | Polyketide | 31.0 | 24.1 | 392.0 | 4.3e-108 |
| AKD43764.1 | HerF | BGC0001349 | NRP+Polyketide | 34.0 | 22.4 | 391.0 | 4.41e-108 |
| CBD77736.1 | polyketide\_synthase | BGC0000974 | NRP+Polyketide | 30.0 | 27.2 | 387.0 | 5.66e-108 |
| ctg1\_14 |  | BGC0001931 | Polyketide | 32.0 | 22.7 | 388.0 | 5.73e-108 |
| QIQ28634.1 | Nbc38 | BGC0002541 | Other | 30.0 | 27.4 | 390.0 | 6.42e-108 |
| CAC20931.1 | PimS1\_protein | BGC0000125 | Polyketide | 33.0 | 22.4 | 391.0 | 7.11e-108 |
| CBA11583.1 | polyketide\_synthase\_type\_I | BGC0001046 | NRP+Polyketide:Modular type I polyketide+Saccharide:Hybrid/tailoring saccharide | 35.0 | 20.2 | 390.0 | 7.79e-108 |
| AAX98192.1 | polyketide\_synthase\_type\_I | BGC0000052 | Polyketide | 33.0 | 22.3 | 390.0 | 8.7e-108 |
| ATG32078.1 | polyketide\_synthase | BGC0001750 | NRP+Polyketide | 31.0 | 25.6 | 383.0 | 9.2e-108 |
| ACF35445.1 | mbcAI | BGC0000090 | Polyketide | 32.0 | 22.9 | 390.0 | 9.28e-108 |
| CAQ18832.1 | polyketide\_synthase | BGC0000954 | NRP+Polyketide:Modular type I polyketide | 30.0 | 27.5 | 388.0 | 9.73e-108 |
| AEZ54374.1 | PieA1 | BGC0000124 | Polyketide | 32.0 | 22.2 | 389.0 | 1.03e-107 |
| CAM00062.1 | EryAI\_Erythromycin\_polyketide\_synthase\_modules\_1\_and\_2 | BGC0000055 | Polyketide:Modular type I polyketide+Saccharide:Hybrid/tailoring saccharide | 33.0 | 20.8 | 390.0 | 1.04e-107 |
| ATG32077.1 | polyketide\_synthase | BGC0001750 | NRP+Polyketide | 33.0 | 23.5 | 387.0 | 1.05e-107 |
| CRI73798.1 | CongD\_protein | BGC0001215 | NRP | 30.0 | 26.5 | 388.0 | 1.11e-107 |
| IF55\_RS32375 | beta-ketoacyl\_synthase | BGC0001348 | Polyketide:Modular type I polyketide | 32.0 | 22.2 | 390.0 | 1.15e-107 |
| UHY14126.1 | PKS\_I | BGC0002671 | Polyketide | 34.0 | 22.5 | 388.0 | 1.17e-107 |
| AFL48526.1 | laidlomycin\_polyketide\_synthase\_(module\_2) | BGC0000084 | Polyketide | 32.0 | 22.5 | 388.0 | 1.26e-107 |
| BAW35658.1 | modular\_polyketide\_synthase | BGC0002355 | Polyketide+Other | 29.0 | 26.3 | 390.0 | 1.26e-107 |
| AHE80994.1 | PieA4 | BGC0001169 | Polyketide:Modular type I polyketide | 32.0 | 23.4 | 388.0 | 1.27e-107 |
| AKJ15836.1 | Type\_I\_polyketide\_synthase | BGC0002735 | Polyketide+NRP | 32.0 | 22.4 | 388.0 | 1.33e-107 |
| ARW71486.1 | type\_I\_PKS\_module\_6 | BGC0001812 | Polyketide | 33.0 | 21.4 | 383.0 | 1.35e-107 |
| AEE88283.1 | CurG | BGC0000976 | NRP+Polyketide:Modular type I polyketide | 33.0 | 20.5 | 383.0 | 1.47e-107 |
| AAT70102.1 | CurG | BGC0001165 | NRP+Polyketide:Modular type I polyketide | 33.0 | 20.5 | 383.0 | 1.47e-107 |
| BAH02271.1 | polyketide\_synthase | BGC0000126 | Polyketide | 33.0 | 21.4 | 385.0 | 1.54e-107 |
| QOD95000.1 | PldAV | BGC0002102 | Polyketide | 33.0 | 21.4 | 385.0 | 1.54e-107 |
| QIQ28636.1 | Nbc40 | BGC0002541 | Other | 30.0 | 27.3 | 390.0 | 1.7e-107 |
| QOD94997.1 | PldAII | BGC0002102 | Polyketide | 30.0 | 26.7 | 388.0 | 1.84e-107 |
| QUQ72348.1 | type\_I\_polyketide\_synthase | BGC0002349 | Polyketide+Saccharide | 32.0 | 24.0 | 389.0 | 1.93e-107 |
| AFL48525.1 | laidlomycin\_polyketide\_synthase\_(loading\_module\_and\_module\_1) | BGC0000084 | Polyketide | 30.0 | 26.8 | 388.0 | 2e-107 |
| AKL64831.1 | polyketide\_synthase | BGC0002072 | Polyketide:Modular type I polyketide | 31.0 | 26.4 | 389.0 | 2.45e-107 |
| ASZ00147.1 | polyketide\_synthase | BGC0001785 | Polyketide | 34.0 | 21.8 | 389.0 | 2.49e-107 |
| UHH90025.1 | VicP1 | BGC0002634 | Polyketide+NRP+Other | 33.0 | 22.5 | 389.0 | 2.56e-107 |
| ADZ24997.1 | polyketide\_synthase | BGC0000380 | NRP+Polyketide:Modular type I polyketide | 31.0 | 27.6 | 385.0 | 2.82e-107 |
| ABK32290.1 | JerD | BGC0000080 | Polyketide | 31.0 | 23.9 | 385.0 | 3.03e-107 |
| AKG06376.1 | polyketide\_synthase\_type\_1 | BGC0001830 | Polyketide | 32.0 | 22.7 | 385.0 | 3.17e-107 |
| WP\_055480219.1 | type\_I\_polyketide\_synthase | BGC0001653 | Polyketide | 32.0 | 22.5 | 389.0 | 3.2e-107 |
| CAE46850.1 | Type\_I\_modular\_polyketide\_synthase | BGC0000103 | Polyketide | 29.0 | 25.7 | 387.0 | 3.34e-107 |
| AEP40940.1 | polyketide\_synthase\_type\_I | BGC0000021 | Polyketide | 34.0 | 20.3 | 389.0 | 3.44e-107 |
| BAW35636.1 | modular\_polyketide\_synthase | BGC0002356 | Polyketide+Other | 31.0 | 24.2 | 389.0 | 3.62e-107 |
| AEZ53950.1 | polyketide\_synthase | BGC0000144 | Polyketide:Modular type I polyketide | 30.0 | 27.2 | 380.0 | 3.93e-107 |
| CAL58687.1 | polyketide\_synthase | BGC0000149 | Polyketide:Modular type I polyketide | 32.0 | 22.4 | 388.0 | 4.03e-107 |
| BAG85027.1 | putative\_polyketide\_synthase | BGC0000086 | Polyketide | 33.0 | 22.6 | 388.0 | 4.21e-107 |
| CAQ64687.1 | lasalocid\_modular\_polyketide\_synthase | BGC0000087 | Polyketide | 33.0 | 22.6 | 388.0 | 4.22e-107 |
| AEZ53946.1 | polyketide\_synthase | BGC0000144 | Polyketide:Modular type I polyketide | 30.0 | 27.1 | 388.0 | 4.41e-107 |
| AAZ77694.1 | ChlA2 | BGC0000036 | Polyketide:Modular type I polyketide+Polyketide:Iterative type I polyketide+Saccharide:Oligosaccharide | 30.0 | 27.2 | 388.0 | 4.44e-107 |
| QKV49770.1 | PKS | BGC0002526 | Polyketide | 32.0 | 22.4 | 388.0 | 4.59e-107 |
| AAO06916.1 | GdmAI | BGC0000066 | Polyketide | 30.0 | 26.9 | 388.0 | 4.66e-107 |
| AAQ84157.1 | Plm2-3 | BGC0000123 | Polyketide | 34.0 | 20.4 | 387.0 | 4.91e-107 |
| QCF28927.1 | type\_I\_polyketide\_synthase | BGC0002308 | Alkaloid+Polyketide | 31.0 | 27.6 | 387.0 | 5.32e-107 |
| QTT72113.1 | type\_I\_polyketide\_synthase | BGC0002350 | NRP+Polyketide+Saccharide | 33.0 | 22.4 | 387.0 | 5.71e-107 |
| WP\_079030450.1 | type\_I\_polyketide\_synthase | BGC0002033 | Polyketide | 29.0 | 27.8 | 385.0 | 6.64e-107 |
| AUO16403.1 | polyketide\_synthase | BGC0001700 | Polyketide | 34.0 | 20.2 | 386.0 | 7e-107 |
| ANH11409.1 | SceN | BGC0001770 | Polyketide | 33.0 | 22.6 | 387.0 | 7e-107 |
| ADC45515.1 | modular\_polyketide\_synthase | BGC0000093 | Polyketide | 32.0 | 22.1 | 384.0 | 7.42e-107 |
| AJW65407.1 | type\_I\_modular\_polyketide\_synthase | BGC0001195 | NRP+Polyketide | 33.0 | 20.3 | 387.0 | 7.76e-107 |
| AKD43763.1 | HerG | BGC0001349 | NRP+Polyketide | 34.0 | 22.2 | 384.0 | 8.78e-107 |
| AFL48529.1 | laidlomycin\_polyketide\_synthase\_(module\_5\_and\_module\_6) | BGC0000084 | Polyketide | 31.0 | 27.3 | 387.0 | 9.8e-107 |
| TMU97102.1 | SDR\_family\_NAD(P)-dependent\_oxidoreductase | BGC0002038 | Polyketide | 31.0 | 27.3 | 385.0 | 9.97e-107 |
| AEZ53945.1 | polyketide\_synthase | BGC0000144 | Polyketide:Modular type I polyketide | 31.0 | 26.3 | 387.0 | 1.17e-106 |
| ACF35447.1 | mbcAIII | BGC0000090 | Polyketide | 31.0 | 22.6 | 386.0 | 1.28e-106 |
| AGM05536.1 | type\_I\_polyketide\_synthase | BGC0002098 | Polyketide | 33.0 | 22.7 | 383.0 | 1.47e-106 |
| PAU45552.1 | Iterative\_polyketide\_synthase | BGC0002138 | Polyketide | 33.0 | 23.6 | 376.0 | 1.57e-106 |
| BAK64637.1 | polyketide\_synthase | BGC0000135 | Polyketide | 32.0 | 22.8 | 386.0 | 1.57e-106 |
| AAX98189.1 | polyketide\_synthase\_type\_I | BGC0000052 | Polyketide | 32.0 | 22.5 | 386.0 | 1.61e-106 |
| ADX66459.1 | ScnS4 | BGC0000108 | Polyketide | 31.0 | 23.7 | 384.0 | 1.66e-106 |
| AZH23823.1 | MgiK | BGC0001971 | NRP+Polyketide | 31.0 | 21.9 | 374.0 | 2.02e-106 |
| AJW65408.1 | type\_I\_modular\_polyketide\_synthase | BGC0001195 | NRP+Polyketide | 34.0 | 20.2 | 386.0 | 2.05e-106 |
| BAP34734.1 | type\_I\_polyketide\_synthase | BGC0000078 | Polyketide | 30.0 | 27.7 | 386.0 | 2.09e-106 |
| ANR02555.1 | LodN | BGC0001648 | Polyketide | 31.0 | 22.2 | 386.0 | 2.11e-106 |
| ctg1\_orf256 |  | BGC0001200 | Polyketide | 31.0 | 25.4 | 386.0 | 2.11e-106 |
| ctg1\_orf254 |  | BGC0001200 | Polyketide | 30.0 | 26.9 | 385.0 | 2.87e-106 |
| AXI91547.1 | FunP6 | BGC0001944 | Polyketide | 34.0 | 20.9 | 385.0 | 3.1e-106 |
| AUO16422.1 | polyketide\_synthase | BGC0001700 | Polyketide | 33.0 | 22.6 | 385.0 | 3.33e-106 |
| ABB88521.1 | polyketide\_synthase\_type\_I | BGC0000050 | Polyketide | 33.0 | 22.2 | 383.0 | 3.43e-106 |
| QBL56182.1 | PKS | BGC0002376 | Polyketide | 33.0 | 22.5 | 385.0 | 3.46e-106 |
| ABB05103.1 | LipPks2 | BGC0001003 | NRP:Lipopeptide+Polyketide:Modular type I polyketide+Saccharide:Hybrid/tailoring saccharide | 32.0 | 22.4 | 385.0 | 3.47e-106 |
| EHK80163.1 | acyl\_transferase | BGC0001447 | Polyketide | 30.0 | 23.3 | 385.0 | 3.5e-106 |
| AEZ54378.1 | PieA5 | BGC0000124 | Polyketide | 31.0 | 27.9 | 382.0 | 3.57e-106 |
| WP\_234353270.1 | SDR\_family\_NAD(P)-dependent\_oxidoreductase | BGC0001537 | Polyketide | 33.0 | 22.3 | 385.0 | 3.79e-106 |
| WP\_240490790.1 | type\_I\_polyketide\_synthase | BGC0002009 | Polyketide | 30.0 | 26.3 | 381.0 | 3.81e-106 |
| QLD23837.1 | SDR\_family\_NAD(P)-dependent\_oxidoreductase | BGC0002086 | Polyketide | 33.0 | 21.4 | 379.0 | 4.12e-106 |
| AQZ37113.1 | polyketide\_synthase | BGC0001511 | Polyketide | 32.0 | 23.0 | 385.0 | 4.26e-106 |
| AAP42859.1 | NanA5 | BGC0000105 | Polyketide | 29.0 | 27.5 | 384.0 | 5.01e-106 |
| WP\_102919232.1 | type\_I\_polyketide\_synthase | BGC0002104 | NRP+Polyketide | 30.0 | 26.5 | 385.0 | 5.73e-106 |
| BAP34740.1 | type\_I\_polyketide\_synthase | BGC0000078 | Polyketide | 32.0 | 22.0 | 380.0 | 5.93e-106 |
| TMV00153.1 | acyltransferase\_domain-containing\_protein | BGC0002038 | Polyketide | 30.0 | 27.3 | 383.0 | 6.75e-106 |
| QPP46749.1 | polyketide\_synthase | BGC0002500 | Polyketide | 29.0 | 27.2 | 380.0 | 6.86e-106 |
| UHY14127.1 | PKS\_I | BGC0002671 | Polyketide | 33.0 | 20.5 | 384.0 | 7.2e-106 |
| QHZ99322.1 | nargenicin\_biosynthesis\_PKS | BGC0001875 | Polyketide | 32.0 | 22.1 | 384.0 | 7.45e-106 |
| BAP34733.1 | type\_I\_polyketide\_synthase | BGC0000078 | Polyketide | 30.0 | 26.6 | 384.0 | 7.92e-106 |
| AEZ53949.1 | polyketide\_synthase | BGC0000144 | Polyketide:Modular type I polyketide | 29.0 | 27.2 | 384.0 | 8.13e-106 |
| ANZ22986.1 | ZinC | BGC0001828 | Polyketide | 32.0 | 23.1 | 382.0 | 1.02e-105 |
| AKD43769.1 | HerA2 | BGC0001349 | NRP+Polyketide | 31.0 | 22.4 | 367.0 | 1.1e-105 |
| AFL48527.1 | laidlomycin\_polyketide\_synthase\_(module\_3\_and\_module\_4) | BGC0000084 | Polyketide | 30.0 | 27.1 | 383.0 | 1.12e-105 |
| ABB88519.1 | polyketide\_synthase\_type\_I | BGC0000050 | Polyketide | 32.0 | 22.2 | 383.0 | 1.19e-105 |
| AKA59090.1 | type-I\_PKS | BGC0001619 | Polyketide | 32.0 | 21.0 | 383.0 | 1.28e-105 |
| ACR50773.1 | polyketide\_synthase | BGC0000163 | Polyketide | 29.0 | 27.0 | 383.0 | 1.34e-105 |
| AAC01713.1 | RifD | BGC0000136 | Polyketide | 31.0 | 24.0 | 379.0 | 1.43e-105 |
| AGM05533.1 | type\_I\_polyketide\_synthase | BGC0002098 | Polyketide | 32.0 | 22.6 | 379.0 | 1.49e-105 |
| QNN81303.1 | IonAVII | BGC0002446 | Polyketide | 30.0 | 25.2 | 382.0 | 1.51e-105 |
| EPH46606.1 | putative\_Phenolphthiocerol\_synthesis\_polyketide\_synthase\_type\_I\_Pks15/1 | BGC0001519 | NRP+Polyketide | 33.0 | 20.0 | 380.0 | 1.61e-105 |
| CAQ52624.1 | type\_I\_polyketide\_synthase,\_modules\_7-8 | BGC0001066 | Polyketide:Modular type I polyketide | 33.0 | 22.6 | 382.0 | 1.67e-105 |
| QWF78551.1 | hypothetical\_protein | BGC0002142 | Polyketide | 28.0 | 26.3 | 383.0 | 1.8e-105 |
| ADB23403.1 | polyketide\_synthase\_type\_I | BGC0001062 | Polyketide | 32.0 | 22.1 | 382.0 | 1.86e-105 |
| ABC84461.1 | NigAVI | BGC0000114 | Polyketide:Modular type I polyketide | 33.0 | 20.4 | 378.0 | 2.07e-105 |
| QFU80901.1 | PKS | BGC0002550 | Polyketide | 31.0 | 24.1 | 382.0 | 2.23e-105 |
| ABB88520.1 | polyketide\_synthase\_type\_I | BGC0000050 | Polyketide | 31.0 | 23.2 | 382.0 | 2.26e-105 |
| QBG82528.1 | Polyketide\_synthase | BGC0002587 | Polyketide | 34.0 | 20.2 | 382.0 | 2.67e-105 |
| AAC01711.1 | RifB | BGC0000136 | Polyketide | 30.0 | 22.5 | 382.0 | 2.96e-105 |
| QBF51760.1 | type\_I\_polyketide\_synthase | BGC0001856 | Polyketide:Modular type I polyketide | 32.0 | 22.2 | 382.0 | 3.03e-105 |
| AQH32482.1 | type\_1\_polyketide\_synthase | BGC0001667 | NRP+Polyketide | 33.0 | 20.1 | 382.0 | 3.21e-105 |
| AGM05532.1 | beta-ketoacyl\_synthase | BGC0002098 | Polyketide | 31.0 | 23.6 | 382.0 | 4.13e-105 |
| SCO70310.1 | Type\_I\_polyketide\_synthase | BGC0001433 | Polyketide:Modular type I polyketide | 31.0 | 22.7 | 380.0 | 4.14e-105 |
| QFU19840.1 | PKS | BGC0002431 | Polyketide+Saccharide | 30.0 | 27.6 | 381.0 | 4.34e-105 |
| ABC84457.1 | NigAII | BGC0000114 | Polyketide:Modular type I polyketide | 29.0 | 27.8 | 380.0 | 4.39e-105 |
| ACB37741.1 | putative\_type\_I\_polyketide\_synthase | BGC0000162 | Polyketide | 32.0 | 23.4 | 382.0 | 4.48e-105 |
| AGI99496.1 | Type\_I\_polyketide\_synthase | BGC0001004 | Polyketide:Modular type I polyketide | 31.0 | 24.1 | 382.0 | 4.51e-105 |
| AAQ82566.1 | FscF | BGC0000034 | NRP+Polyketide | 31.0 | 25.0 | 379.0 | 4.89e-105 |
| ADM46360.1 | polyketide\_synthase | BGC0000106 | Polyketide | 31.0 | 27.0 | 381.0 | 5.03e-105 |
| AEC13071.1 | fosE | BGC0000060 | Polyketide | 30.0 | 26.6 | 381.0 | 5.03e-105 |
| EPH46607.1 | putative\_Phenolphthiocerol\_synthesis\_polyketide\_synthase\_type\_I\_Pks15/1 | BGC0001519 | NRP+Polyketide | 32.0 | 22.4 | 379.0 | 5.4e-105 |
| QIZ24102.1 | type\_I\_polyketide\_synthase | BGC0002540 | Polyketide | 33.0 | 22.2 | 381.0 | 5.79e-105 |
| WP\_102918843.1 | SDR\_family\_NAD(P)-dependent\_oxidoreductase | BGC0002104 | NRP+Polyketide | 32.0 | 22.2 | 379.0 | 6.07e-105 |
| BBM96640.1 | modular\_polyketide\_synthase | BGC0002452 | Polyketide | 30.0 | 26.8 | 378.0 | 6.21e-105 |
| ABI93779.1 | GdmPKS | BGC0000068 | Polyketide | 30.0 | 23.5 | 380.0 | 7.18e-105 |
| TXD00265.1 | SDR\_family\_NAD(P)-dependent\_oxidoreductase | BGC0001877 | Polyketide | 29.0 | 27.0 | 380.0 | 7.46e-105 |
| AAM54075.1 | polyketide\_synthase | BGC0000020 | Polyketide | 32.0 | 23.0 | 380.0 | 8.28e-105 |
| AQT01393.1 | SgnS2 | BGC0001690 | Polyketide | 32.0 | 22.4 | 381.0 | 8.37e-105 |
| ALD82523.1 | polyketide\_synthase | BGC0001212 | NRP+Polyketide | 35.0 | 20.4 | 377.0 | 8.5e-105 |
| BAF02924.1 | type\_I\_polyketide\_synthase | BGC0000073 | Polyketide | 34.0 | 20.1 | 380.0 | 8.91e-105 |
| ARV85764.1 | PieA5\_type\_I\_PKS | BGC0001742 | Polyketide | 30.0 | 27.8 | 377.0 | 9.38e-105 |
| ACB37742.1 | putative\_type\_I\_polyketide\_synthase | BGC0000162 | Polyketide | 31.0 | 22.6 | 377.0 | 9.97e-105 |
| AXG22406.1 | type\_I\_polyketide\_synthase | BGC0002024 | Polyketide | 31.0 | 22.2 | 380.0 | 1.09e-104 |
| EHK80166.1 | beta-ketoacyl\_synthase | BGC0001447 | Polyketide | 32.0 | 21.5 | 380.0 | 1.18e-104 |
| EHK80169.1 | acyl\_transferase | BGC0001447 | Polyketide | 31.0 | 24.4 | 380.0 | 1.26e-104 |
| UHH90010.1 | VicP2 | BGC0002634 | Polyketide+NRP+Other | 33.0 | 22.6 | 378.0 | 1.38e-104 |
| QRI43526.1 | type\_I\_polyketide\_synthase | BGC0002454 | Polyketide | 33.0 | 22.5 | 379.0 | 1.38e-104 |
| ACN69991.1 | polyketide\_synthase | BGC0000079 | Polyketide | 32.0 | 22.1 | 378.0 | 1.53e-104 |
| TMU97101.1 | SDR\_family\_NAD(P)-dependent\_oxidoreductase | BGC0002038 | Polyketide | 31.0 | 26.3 | 379.0 | 1.67e-104 |
| AEC13069.1 | fosC | BGC0000060 | Polyketide | 33.0 | 22.9 | 379.0 | 1.93e-104 |
| WP\_102918844.1 | type\_I\_polyketide\_synthase | BGC0002104 | NRP+Polyketide | 32.0 | 22.1 | 379.0 | 2.03e-104 |
| ALA09371.1 | type\_I\_modular\_PKS | BGC0001303 | Polyketide | 32.0 | 22.4 | 379.0 | 2.25e-104 |
| CAJ88176.1 | Type\_I\_modular\_polyketide\_synthase | BGC0000151 | Polyketide:Modular type I polyketide+Saccharide:Hybrid/tailoring saccharide | 32.0 | 22.4 | 379.0 | 2.61e-104 |
| AFV30248.1 | polyketide\_synthase | BGC0000075 | Polyketide | 31.0 | 23.5 | 377.0 | 2.87e-104 |
| WP\_033261452.1 | type\_I\_polyketide\_synthase | BGC0002009 | Polyketide | 31.0 | 24.1 | 378.0 | 3.22e-104 |
| BAC68127.1 | modular\_polyketide\_synthase | BGC0000059 | Polyketide | 32.0 | 22.3 | 375.0 | 3.31e-104 |
| BAO98805.1 | putative\_polyketide\_synthase | BGC0001002 | NRP+Polyketide | 29.0 | 28.9 | 370.0 | 3.34e-104 |
| QWF78547.1 | 3-ketoacyl-CoA\_thiolase | BGC0002142 | Polyketide | 29.0 | 27.1 | 379.0 | 3.42e-104 |
| QPP46750.1 | polyketide\_synthase | BGC0002500 | Polyketide | 30.0 | 26.3 | 378.0 | 3.64e-104 |
| QEA08891.1 | JenA5 | BGC0002559 | Polyketide | 32.0 | 22.4 | 378.0 | 3.66e-104 |
| TMU97099.1 | SDR\_family\_NAD(P)-dependent\_oxidoreductase | BGC0002038 | Polyketide | 31.0 | 26.4 | 378.0 | 3.74e-104 |
| QKV49766.1 | PKS | BGC0002526 | Polyketide | 30.0 | 27.5 | 377.0 | 3.93e-104 |
| BAV56011.1 | PKS\_(KS-AT-DH-ER-KR-ACP-KS-AT-DH-ER-KR-ACP) | BGC0001597 | Polyketide | 29.0 | 26.3 | 378.0 | 5.11e-104 |
| AUA09463.1 | Phenolphthiocerol\_synthesis\_polyketide\_synthase\_type\_I\_Pks15/1 | BGC0002291 | Polyketide | 29.0 | 27.4 | 375.0 | 6.33e-104 |
| CAJ88175.1 | Type\_I\_modular\_polyketide\_synthase | BGC0000151 | Polyketide:Modular type I polyketide+Saccharide:Hybrid/tailoring saccharide | 33.0 | 20.2 | 378.0 | 6.86e-104 |
| BAQ21947.1 | putative\_type\_I\_polyketide\_synthase | BGC0001204 | Polyketide | 27.0 | 26.4 | 377.0 | 7.57e-104 |
| BAK64650.1 | polyketide\_synthase | BGC0000135 | Polyketide | 32.0 | 23.1 | 377.0 | 8.57e-104 |
| AWC08660.1 | polyketide\_synthase\_type\_I | BGC0001662 | Polyketide | 31.0 | 22.4 | 377.0 | 8.61e-104 |
| AGM05535.1 | modular\_polyketide\_synthase | BGC0002098 | Polyketide | 32.0 | 22.2 | 377.0 | 9.74e-104 |
| BAD08359.1 | polyketide\_synthase\_modules\_5-6 | BGC0000167 | Polyketide | 33.0 | 20.1 | 376.0 | 1.2e-103 |
| ANZ22989.1 | ZinF | BGC0001828 | Polyketide | 34.0 | 20.6 | 376.0 | 1.34e-103 |
| AXI91548.1 | FunP5 | BGC0001944 | Polyketide | 31.0 | 25.4 | 377.0 | 1.45e-103 |
| AEZ64504.1 | Herc | BGC0001065 | Polyketide | 30.0 | 26.3 | 377.0 | 1.47e-103 |
| BAF92601.1 | iterative\_type\_I\_PKS | BGC0000118 | Polyketide | 33.0 | 23.2 | 373.0 | 1.51e-103 |
| ACJ24875.1 | 6-methylsalicylic\_acid\_synthase | BGC0000119 | Polyketide:Iterative type I polyketide+Saccharide:Hybrid/tailoring saccharide | 33.0 | 23.2 | 373.0 | 1.51e-103 |
| QBF51758.1 | type\_I\_polyketide\_synthase | BGC0001856 | Polyketide:Modular type I polyketide | 29.0 | 26.4 | 377.0 | 1.55e-103 |
| BAO66528.1 | type\_I\_polyketide\_synthase | BGC0000042 | Polyketide | 32.0 | 22.8 | 361.0 | 1.6e-103 |
| BBA66511.1 | type\_I\_polyketide\_synthase | BGC0001495 | Polyketide | 33.0 | 20.4 | 376.0 | 1.72e-103 |
| AWW87424.1 | type\_I\_polyketide\_synthase | BGC0001755 | Polyketide | 32.0 | 22.4 | 376.0 | 2.09e-103 |
| AXI91549.1 | FunP4 | BGC0001944 | Polyketide | 32.0 | 23.7 | 376.0 | 2.13e-103 |
| WP\_245661582.1 | polyketide\_synthase | BGC0001348 | Polyketide:Modular type I polyketide | 32.0 | 23.5 | 375.0 | 2.13e-103 |
| AXI91545.1 | FunP8 | BGC0001944 | Polyketide | 34.0 | 22.4 | 373.0 | 2.39e-103 |
| AAC69332.1 | type\_I\_polyketide\_synthase\_PikAIV | BGC0000094 | Polyketide:Modular type I polyketide+Saccharide:Hybrid/tailoring saccharide | 32.0 | 20.3 | 367.0 | 2.41e-103 |
| AAO62585.1 | peptide\_sythetase\_polyketide\_synthase\_fusion\_protein | BGC0001016 | NRP+Polyketide | 31.0 | 22.4 | 375.0 | 2.42e-103 |
| AVV61980.1 | type\_I\_modular\_PKS | BGC0001477 | NRP+Polyketide:Modular type I polyketide | 29.0 | 26.5 | 376.0 | 2.51e-103 |
| AAC46027.1 | polyketide\_synthase\_module\_6 | BGC0000113 | Polyketide | 35.0 | 20.9 | 370.0 | 2.73e-103 |
| QBG82529.1 | Polyketide\_synthase | BGC0002587 | Polyketide | 30.0 | 27.0 | 375.0 | 3.35e-103 |
| ACY06289.1 | type\_I\_polyketide\_synthase | BGC0001042 | NRP+Polyketide | 32.0 | 22.5 | 375.0 | 3.44e-103 |
| QFU19838.1 | PKS | BGC0002431 | Polyketide+Saccharide | 33.0 | 20.5 | 370.0 | 3.45e-103 |
| ARW71484.1 | type\_I\_PKS\_module\_3 | BGC0001812 | Polyketide | 29.0 | 28.5 | 372.0 | 4.49e-103 |
| BAV56006.1 | PKS\_(ACP-KS-AT-DH-ER-KR-ACP-KS-AT-KR-ACP) | BGC0001597 | Polyketide | 28.0 | 28.9 | 374.0 | 5.35e-103 |
| AAP42858.1 | NanA4 | BGC0000105 | Polyketide | 30.0 | 27.4 | 374.0 | 5.38e-103 |
| CAQ52622.1 | type\_I\_polyketide\_synthase,\_modules\_4-5 | BGC0001066 | Polyketide:Modular type I polyketide | 30.0 | 27.1 | 374.0 | 5.4e-103 |
| ABC84470.1 | NIGAVIII | BGC0000114 | Polyketide:Modular type I polyketide | 28.0 | 27.3 | 373.0 | 5.59e-103 |
| AFP87523.1 | type\_I\_polyketide\_synthase | BGC0001159 | NRP+Polyketide:Modular type I polyketide | 33.0 | 20.4 | 373.0 | 6.09e-103 |
| QBF51756.1 | type\_I\_polyketide\_synthase | BGC0001856 | Polyketide:Modular type I polyketide | 32.0 | 22.2 | 374.0 | 7e-103 |
| CAC20919.1 | PimS4\_protein | BGC0000125 | Polyketide | 32.0 | 22.1 | 372.0 | 8.31e-103 |
| AQT01395.1 | SgnS4 | BGC0001690 | Polyketide | 32.0 | 22.1 | 372.0 | 8.31e-103 |
| TGZ15166.1 | hypothetical\_protein | BGC0002032 | Polyketide | 31.0 | 22.9 | 370.0 | 8.75e-103 |
| QCQ67874.1 | type\_I\_polyketide\_synthase | BGC0002297 | NRP+Polyketide | 33.0 | 20.1 | 374.0 | 9.12e-103 |
| QEA08889.1 | JenA3 | BGC0002559 | Polyketide | 34.0 | 20.1 | 374.0 | 9.3e-103 |
| UHH90012.1 | VicP4 | BGC0002634 | Polyketide+NRP+Other | 31.0 | 22.9 | 373.0 | 1.19e-102 |
| ANH11410.1 | SceO | BGC0001770 | Polyketide | 32.0 | 22.7 | 373.0 | 1.41e-102 |
| CAC22145.1 | CpkB;\_Polyketide\_synthase\_modules\_3\_and\_4 | BGC0000038 | Polyketide:Modular type I polyketide | 29.0 | 26.3 | 373.0 | 1.44e-102 |
| AAY28227.1 | HbmAIII | BGC0000074 | Polyketide | 28.0 | 27.0 | 373.0 | 1.55e-102 |
| QFU19839.1 | PKS | BGC0002431 | Polyketide+Saccharide | 30.0 | 27.6 | 373.0 | 1.6e-102 |
| TMU97100.1 | SDR\_family\_NAD(P)-dependent\_oxidoreductase | BGC0002038 | Polyketide | 29.0 | 27.4 | 373.0 | 1.63e-102 |
| QBL56181.1 | PKS | BGC0002376 | Polyketide | 33.0 | 22.3 | 372.0 | 1.96e-102 |
| AKA59093.1 | type-I\_PKS | BGC0001619 | Polyketide | 31.0 | 22.3 | 372.0 | 2.07e-102 |
| BAB69199.1 | modular\_polyketide\_synthase | BGC0000117 | Polyketide | 31.0 | 23.0 | 372.0 | 2.71e-102 |
| AAF19812.1 | MtaD | BGC0001024 | NRP+Polyketide:Modular type I polyketide | 32.0 | 23.3 | 372.0 | 2.95e-102 |
| QEA08888.1 | JenA2 | BGC0002559 | Polyketide | 32.0 | 23.8 | 370.0 | 3.02e-102 |
| ADX66461.1 | ScnS2 | BGC0000108 | Polyketide | 32.0 | 22.3 | 372.0 | 3.04e-102 |
| CAC20921.1 | PimS2\_protein | BGC0000125 | Polyketide | 32.0 | 22.3 | 372.0 | 3.04e-102 |
| OAP25820.1 | Erythronolide\_synthase,\_modules\_1\_and\_2 | BGC0001658 | Polyketide | 32.0 | 20.4 | 367.0 | 3.08e-102 |
| CAA60459.1 | polyketide\_synthase | BGC0001040 | NRP+Polyketide | 32.0 | 22.1 | 372.0 | 3.09e-102 |
| CQR60493.1 | Polyketide\_synthase,\_type\_I,\_modules:\_9\_and\_10 | BGC0001287 | Polyketide | 32.0 | 22.4 | 372.0 | 3.15e-102 |
| BAQ25482.1 | type\_I\_polyketide\_synthase | BGC0001288 | Polyketide | 29.0 | 27.4 | 372.0 | 3.53e-102 |
| WP\_030180235.1 | type\_I\_polyketide\_synthase | BGC0002106 | Polyketide | 32.0 | 22.4 | 372.0 | 3.97e-102 |
| QHZ99321.1 | polyketide\_synthaase | BGC0001875 | Polyketide | 32.0 | 22.3 | 371.0 | 4.3e-102 |
| QBG82517.1 | Polyketide\_synthase | BGC0002587 | Polyketide | 30.0 | 26.6 | 370.0 | 4.79e-102 |
| ctg1\_orf10 |  | BGC0000053 | Polyketide | 33.0 | 20.5 | 371.0 | 5.22e-102 |
| IF55\_RS36525 | polyketide\_synthase | BGC0001348 | Polyketide:Modular type I polyketide | 34.0 | 20.1 | 369.0 | 5.62e-102 |
| BAR73019.1 | putative\_PKS\_(KS-AT-DH-KR-ACP-TE) | BGC0001194 | Polyketide | 34.0 | 22.5 | 369.0 | 7.02e-102 |
| ABV97155.1 | Acyl\_transferase | BGC0000137 | Polyketide | 31.0 | 24.1 | 370.0 | 7.06e-102 |
| AAC46028.1 | polyketide\_synthase\_module\_7 | BGC0000113 | Polyketide | 33.0 | 20.4 | 368.0 | 7.84e-102 |
| AHE80995.1 | PieA5 | BGC0001169 | Polyketide:Modular type I polyketide | 32.0 | 23.1 | 367.0 | 1.04e-101 |
| CAQ18839.1 | hybrid\_polyketide\_synthase/nonribosomal\_polypetide\_synthetase | BGC0000954 | NRP+Polyketide:Modular type I polyketide | 32.0 | 22.1 | 370.0 | 1.06e-101 |
| WP\_081238291.1 | type\_I\_polyketide\_synthase | BGC0002105 | Polyketide | 31.0 | 26.9 | 370.0 | 1.15e-101 |
| QIZ24100.1 | type\_I\_polyketide\_synthase | BGC0002540 | Polyketide | 32.0 | 22.1 | 369.0 | 1.36e-101 |
| AKA59091.1 | type-I\_PKS | BGC0001619 | Polyketide | 33.0 | 20.2 | 370.0 | 1.37e-101 |
| AHB82070.1 | polyketide\_synthase | BGC0001231 | NRP+Polyketide:Modular type I polyketide | 33.0 | 20.7 | 363.0 | 1.52e-101 |
| AAM54077.1 | polyketide\_synthase | BGC0000020 | Polyketide | 34.0 | 21.0 | 364.0 | 1.71e-101 |
| ABC84469.1 | NigAIX | BGC0000114 | Polyketide:Modular type I polyketide | 30.0 | 27.0 | 367.0 | 1.73e-101 |
| QBG82531.1 | cytochrome\_P450 | BGC0002587 | Polyketide | 32.0 | 21.7 | 369.0 | 1.74e-101 |
| TMU97089.1 | SDR\_family\_NAD(P)-dependent\_oxidoreductase | BGC0002038 | Polyketide | 29.0 | 27.3 | 368.0 | 1.92e-101 |
| ANR02552.1 | LodK | BGC0001648 | Polyketide | 32.0 | 20.2 | 365.0 | 2.05e-101 |
| BAD38873.1 | polyketide\_synthase | BGC0000111 | Polyketide | 32.0 | 22.8 | 367.0 | 2.07e-101 |
| OAP25821.1 | Phenolphthiocerol\_synthesis\_polyketide\_synthase\_type\_I\_Pks15/1 | BGC0001658 | Polyketide | 31.0 | 23.5 | 369.0 | 2.68e-101 |
| QSV12664.1 | AvmF | BGC0002456 | Polyketide+NRP | 32.0 | 21.0 | 369.0 | 2.81e-101 |
| QLD23836.1 | SDR\_family\_NAD(P)-dependent\_oxidoreductase | BGC0002086 | Polyketide | 29.0 | 28.4 | 366.0 | 2.82e-101 |
| AAZ94386.1 | modular\_polyketide\_synthase | BGC0000040 | Polyketide | 30.0 | 26.9 | 368.0 | 3.56e-101 |
| BAD08360.1 | polyketide\_synthase\_modules\_7-8 | BGC0000167 | Polyketide | 30.0 | 22.7 | 368.0 | 3.84e-101 |
| CAQ52623.1 | type\_I\_polyketide\_synthase,\_module\_6 | BGC0001066 | Polyketide:Modular type I polyketide | 32.0 | 22.0 | 367.0 | 4.08e-101 |
| BAE93722.1 | type\_I\_polyketide\_synthase | BGC0000164 | Polyketide | 31.0 | 25.2 | 368.0 | 4.55e-101 |
| CBZ41585.1 | Type\_I\_modular\_polyketide\_synthase | BGC0000151 | Polyketide:Modular type I polyketide+Saccharide:Hybrid/tailoring saccharide | 31.0 | 25.4 | 368.0 | 4.71e-101 |
| QSE03604.1 | LcmD | BGC0002333 | Polyketide | 32.0 | 22.4 | 365.0 | 4.76e-101 |
| CAA60462.1 | polyketide\_synthase | BGC0001040 | NRP+Polyketide | 30.0 | 24.3 | 368.0 | 5.12e-101 |
| AHH99925.1 | PKS\_I | BGC0000002 | Polyketide | 32.0 | 22.6 | 368.0 | 6.03e-101 |
| BAO66519.1 | type\_I\_polyketide\_synthase | BGC0000042 | Polyketide | 34.0 | 22.1 | 367.0 | 6.22e-101 |
| CAC20920.1 | PimS3\_protein | BGC0000125 | Polyketide | 31.0 | 22.4 | 364.0 | 6.9e-101 |
| AQT01394.1 | SgnS3 | BGC0001690 | Polyketide | 31.0 | 22.4 | 364.0 | 6.9e-101 |
| ABC84459.1 | NigAIV | BGC0000114 | Polyketide:Modular type I polyketide | 30.0 | 27.3 | 367.0 | 6.9e-101 |
| AAC68815.1 | FK506\_polyketide\_synthase | BGC0000353 | NRP | 31.0 | 22.5 | 368.0 | 7.14e-101 |
| AAB66505.1 | tylactone\_synthase\_module\_3 | BGC0000166 | Polyketide | 30.0 | 27.3 | 364.0 | 8.85e-101 |
| QCF28926.1 | type\_I\_polyketide\_synthase | BGC0002308 | Alkaloid+Polyketide | 32.0 | 22.4 | 365.0 | 9.54e-101 |
| CAA60460.1 | polyketide\_synthase | BGC0001040 | NRP+Polyketide | 31.0 | 23.2 | 367.0 | 9.66e-101 |
| AXG22405.1 | type\_I\_polyketide\_synthase | BGC0002024 | Polyketide | 31.0 | 23.5 | 367.0 | 1.09e-100 |
| TGZ15168.1 | hypothetical\_protein | BGC0002032 | Polyketide | 32.0 | 22.3 | 367.0 | 1.23e-100 |
| WP\_081238284.1 | type\_I\_polyketide\_synthase | BGC0002105 | Polyketide | 32.0 | 22.1 | 366.0 | 1.51e-100 |
| AAO65800.1 | monensin\_polyketide\_synthase\_modules\_7\_and\_8 | BGC0000100 | Polyketide | 31.0 | 26.1 | 366.0 | 1.56e-100 |
| ANZ52463.1 | MonAV | BGC0001670 | Polyketide | 31.0 | 26.1 | 366.0 | 1.56e-100 |
| ADX66460.1 | ScnS3 | BGC0000108 | Polyketide | 31.0 | 22.4 | 363.0 | 1.62e-100 |
| AGC09486.1 | LobS3 | BGC0001183 | Polyketide | 33.0 | 20.9 | 363.0 | 1.64e-100 |
| AAF86392.1 | FkbC | BGC0000994 | NRP+Polyketide | 32.0 | 22.1 | 366.0 | 1.82e-100 |
| ABC84458.1 | NigAIII | BGC0000114 | Polyketide:Modular type I polyketide | 30.0 | 26.8 | 366.0 | 2.03e-100 |
| AFL48532.1 | laidlomycin\_polyketide\_synthase\_(module\_11\_and\_module\_12) | BGC0000084 | Polyketide | 33.0 | 20.3 | 365.0 | 2.43e-100 |
| BAR73021.1 | putative\_PKS\_(KS-AT-KR-ACP) | BGC0001194 | Polyketide | 34.0 | 21.3 | 361.0 | 2.6e-100 |
| AAC01714.1 | RifE | BGC0000136 | Polyketide | 30.0 | 26.5 | 365.0 | 2.97e-100 |
| BCK51643.1 | modular\_polyketide\_synthase | BGC0002520 | Polyketide | 31.0 | 26.6 | 365.0 | 2.98e-100 |
| ABJ97439.1 | MerC | BGC0001012 | NRP+Polyketide | 30.0 | 23.3 | 366.0 | 3.01e-100 |
| AGY62758.1 | EbeF | BGC0000051 | Polyketide | 29.0 | 26.9 | 364.0 | 3.08e-100 |
| QGA70079.1 | type\_I\_polyketide\_synthase | BGC0002517 | Polyketide | 32.0 | 22.5 | 365.0 | 3.12e-100 |
| AAO65799.1 | monensin\_polyketide\_synthase\_modules\_5\_and\_6 | BGC0000100 | Polyketide | 31.0 | 27.5 | 365.0 | 3.45e-100 |
| ANZ52462.1 | MonAIV | BGC0001670 | Polyketide | 31.0 | 27.5 | 365.0 | 3.45e-100 |
| BAV56012.1 | PKS\_(KS-AT-DH-ER-KR-ACP-TE) | BGC0001597 | Polyketide | 28.0 | 28.4 | 364.0 | 4.07e-100 |
| QNN81299.1 | IonAIII | BGC0002446 | Polyketide | 29.0 | 27.0 | 362.0 | 4.19e-100 |
| CAD89775.1 | MelD\_protein | BGC0001010 | NRP+Polyketide:Modular type I polyketide | 32.0 | 23.1 | 364.0 | 4.88e-100 |
| UMP03509.1 | NmvAII | BGC0002649 | NRP+Polyketide | 31.0 | 23.1 | 361.0 | 5.03e-100 |
| BAQ25512.1 | type\_I\_polyketide\_synthase | BGC0001288 | Polyketide | 30.0 | 22.8 | 365.0 | 6.06e-100 |
| BAB69193.1 |  | BGC0000117 | Polyketide | 34.0 | 20.1 | 364.0 | 6.63e-100 |
| WP\_235558179.1 | SDR\_family\_NAD(P)-dependent\_oxidoreductase | BGC0001653 | Polyketide | 30.0 | 24.1 | 364.0 | 7.04e-100 |
| ARS01476.1 | NcmAIV | BGC0001702 | NRP+Polyketide | 32.0 | 22.4 | 361.0 | 7.53e-100 |
| ABC84460.1 | NigAV | BGC0000114 | Polyketide:Modular type I polyketide | 30.0 | 27.0 | 364.0 | 7.73e-100 |
| ATP76242.1 | NdaC | BGC0001705 | NRP+Polyketide | 31.0 | 22.5 | 363.0 | 8.04e-100 |
| AEZ54379.1 | PieA6 | BGC0000124 | Polyketide | 33.0 | 20.6 | 363.0 | 8.56e-100 |
| AGC45618.1 | non-ribosomal\_peptide\_synthetase | BGC0001394 | NRP+Polyketide | 28.0 | 33.4 | 358.0 | 1.06e-99 |
| AAF71776.1 | nysC | BGC0000115 | Polyketide:Modular type I polyketide+Saccharide:Hybrid/tailoring saccharide | 34.0 | 20.4 | 364.0 | 1.14e-99 |
| AWR88393.1 | putative\_beta-ketoacyl\_synthase | BGC0001522 | Polyketide | 30.0 | 26.1 | 363.0 | 1.26e-99 |
| ABJ97438.1 | MerB | BGC0001012 | NRP+Polyketide | 31.0 | 22.6 | 363.0 | 1.34e-99 |
| QIZ24099.1 | type\_I\_polyketide\_synthase | BGC0002540 | Polyketide | 31.0 | 22.2 | 360.0 | 1.36e-99 |
| ANY10599.1 | polyketide\_synthase | BGC0001773 | Polyketide | 31.0 | 22.3 | 361.0 | 1.75e-99 |
| ADC45586.1 | modular\_polyketide\_synthase | BGC0000093 | Polyketide | 31.0 | 22.5 | 363.0 | 1.81e-99 |
| ANH11414.1 | SceS | BGC0001770 | Polyketide | 31.0 | 23.2 | 363.0 | 2.01e-99 |
| ctg1\_orf11 |  | BGC0000053 | Polyketide | 28.0 | 27.5 | 361.0 | 2.05e-99 |
| QLD28380.2 | SDR\_family\_NAD(P)-dependent\_oxidoreductase | BGC0002086 | Polyketide | 34.0 | 20.1 | 362.0 | 2.13e-99 |
| QSE03591.1 | LcmC | BGC0002333 | Polyketide | 30.0 | 27.0 | 363.0 | 2.45e-99 |
| AEU17897.1 | putative\_type\_I\_PKS | BGC0001072 | Saccharide+Polyketide:Modular type I polyketide+Polyketide:Type II polyketide+Other:Aminocoumarin | 31.0 | 22.9 | 361.0 | 2.52e-99 |
| ADC45535.1 | modular\_polyketide\_synthase | BGC0000093 | Polyketide | 29.0 | 27.5 | 362.0 | 2.65e-99 |
| QWF78544.1 | 3-ketoacyl-CoA\_thiolase | BGC0002142 | Polyketide | 33.0 | 20.1 | 362.0 | 2.79e-99 |
| BAO66539.1 | type\_I\_polyketide\_synthase | BGC0000042 | Polyketide | 34.0 | 20.5 | 361.0 | 4.06e-99 |
| ACO94488.1 | polyketide\_synthase\_type\_I | BGC0000097 | Polyketide:Modular type I polyketide | 31.0 | 22.5 | 361.0 | 4.52e-99 |
| CAQ52626.1 | type\_I\_polyketide\_synthase,\_loading\_module\_and\_modules\_1-3 | BGC0001066 | Polyketide:Modular type I polyketide | 31.0 | 23.8 | 362.0 | 4.84e-99 |
| AAF86396.1 | FkbA | BGC0000994 | NRP+Polyketide | 31.0 | 22.5 | 362.0 | 4.92e-99 |
| ALP32046.1 | CycF | BGC0001293 | Polyketide | 30.0 | 22.3 | 360.0 | 6.67e-99 |
| QIZ24098.1 | type\_I\_polyketide\_synthase | BGC0002540 | Polyketide | 32.0 | 22.7 | 361.0 | 7.21e-99 |
| AAF71774.1 | nysA | BGC0000115 | Polyketide:Modular type I polyketide+Saccharide:Hybrid/tailoring saccharide | 32.0 | 22.1 | 353.0 | 7.6e-99 |
| AEW98134.1 | polyketide\_synthase | BGC0002642 | Alkaloid | 31.0 | 22.4 | 359.0 | 7.93e-99 |
| QBL56183.1 | PKS | BGC0002376 | Polyketide | 34.0 | 20.1 | 356.0 | 8.24e-99 |
| AVX51099.1 | NysJ | BGC0001709 | Polyketide | 32.0 | 22.2 | 360.0 | 1.03e-98 |
| AFL48528.1 | laidlomycin\_polyketide\_synthase\_(module\_7\_and\_module\_8) | BGC0000084 | Polyketide | 30.0 | 26.2 | 360.0 | 1.44e-98 |
| ABP55210.1 | beta-ketoacyl\_synthase | BGC0000142 | Polyketide | 29.0 | 27.0 | 360.0 | 1.5e-98 |
| BAQ25481.1 | type\_I\_polyketide\_synthase | BGC0001288 | Polyketide | 29.0 | 29.0 | 360.0 | 1.5e-98 |
| CAE02605.1 | polyketide\_synthase\_type\_I | BGC0000024 | Polyketide:Modular type I polyketide | 33.0 | 20.2 | 358.0 | 1.59e-98 |
| BAQ25513.1 | type\_I\_polyketide\_synthase | BGC0001288 | Polyketide | 31.0 | 22.6 | 359.0 | 1.89e-98 |
| AAO65796.1 | monensin\_polyketide\_synthase\_loading\_module\_and\_module\_1 | BGC0000100 | Polyketide | 29.0 | 27.3 | 359.0 | 1.91e-98 |
| ANZ52459.1 | MonAI | BGC0001670 | Polyketide | 29.0 | 27.3 | 359.0 | 1.91e-98 |
| EHK80165.1 | beta-ketoacyl\_synthase | BGC0001447 | Polyketide | 30.0 | 23.8 | 357.0 | 1.94e-98 |
| CCP20050.1 | divL3\_protein | BGC0001119 | Polyketide:Modular type I polyketide | 31.0 | 23.4 | 358.0 | 2.22e-98 |
| QIE07127.1 | OvmL1 | BGC0001719 | Polyketide | 31.0 | 23.1 | 357.0 | 2.95e-98 |
| QBL56184.1 | PKS | BGC0002376 | Polyketide | 29.0 | 27.1 | 358.0 | 2.97e-98 |
| AJO72742.1 | Type\_I\_modular\_polyketide\_synthase | BGC0001381 | Polyketide | 34.0 | 20.5 | 358.0 | 3.04e-98 |
| ONK09689.1 | Beta-ketoacyl-acyl-carrier-protein\_synthase\_I | BGC0001647 | Polyketide | 32.0 | 22.2 | 358.0 | 3.32e-98 |
| ACR33077.1 | polyketide\_synthase | BGC0000017 | Alkaloid+Polyketide:Modular type I polyketide | 30.0 | 22.5 | 357.0 | 3.49e-98 |
| AAU93805.2 | polyketide\_synthase\_modules\_5\_and\_6 | BGC0000054 | Polyketide | 32.0 | 20.9 | 358.0 | 3.5e-98 |
| AVI26390.1 | polyketide\_synthase\_/\_nonribosomal\_peptide\_synthase\_hybrid | BGC0001800 | NRP+Polyketide | 33.0 | 21.3 | 358.0 | 3.7e-98 |
| AAQ90174.1 | polyketide\_synthase\_type\_I | BGC0000128 | Polyketide | 35.0 | 20.1 | 355.0 | 3.84e-98 |
| QSE03601.1 | LcmB | BGC0002333 | Polyketide | 31.0 | 22.1 | 358.0 | 4.22e-98 |
| sipP5 | Type\_I\_Modular\_PKS | BGC0001452 | Polyketide | 30.0 | 26.1 | 358.0 | 5.42e-98 |
| AAW03327.1 | CtaD | BGC0000982 | NRP+Polyketide | 31.0 | 24.0 | 357.0 | 6.18e-98 |
| QKV49767.1 | PKS | BGC0002526 | Polyketide | 32.0 | 22.6 | 357.0 | 7.36e-98 |
| DAB41918.1 | ArzP\_-\_PKS\_(KS,\_AT,\_OMT,\_ACP,\_TE) | BGC0001884 | NRP+Polyketide | 32.0 | 21.8 | 354.0 | 7.84e-98 |
| SAI82900.1 | HrnB;\_Macrolactam\_polyketide\_synthase\_type\_I;\_modules\_loading,\_1-2 | BGC0002101 | Polyketide | 31.0 | 22.6 | 357.0 | 8.67e-98 |
| BAP34739.1 | type\_I\_polyketide\_synthase | BGC0000078 | Polyketide | 32.0 | 22.6 | 357.0 | 9.37e-98 |
| QBC75448.1 | MacA | BGC0002615 | Terpene | 29.0 | 23.5 | 354.0 | 1.09e-97 |
| CCM44338.1 | Polyketide\_synthase | BGC0001056 | NRP+Polyketide:Modular type I polyketide+Polyketide:PUFA synthase or related polyketide | 32.0 | 22.1 | 352.0 | 1.21e-97 |
| AAS98200.1 | MSAS-type\_polyketide\_synthase | BGC0001273 | Polyketide | 30.0 | 23.5 | 353.0 | 1.33e-97 |
| AQH32483.1 | hybrid\_peptide\_synthetase/polyketide\_synthase | BGC0001667 | NRP+Polyketide | 30.0 | 22.0 | 355.0 | 1.75e-97 |
| QBL56191.1 | PKS | BGC0002376 | Polyketide | 31.0 | 22.1 | 353.0 | 2.02e-97 |
| ATY46587.1 | polyketide\_synthase | BGC0001666 | Polyketide | 30.0 | 25.4 | 356.0 | 2.45e-97 |
| BCK51633.1 | modular\_polyketide\_synthase | BGC0002520 | Polyketide | 31.0 | 22.5 | 356.0 | 2.46e-97 |
| QUQ72349.1 | type\_I\_polyketide\_synthase | BGC0002349 | Polyketide+Saccharide | 31.0 | 22.2 | 353.0 | 3.01e-97 |
| WP\_081238290.1 | type\_I\_polyketide\_synthase | BGC0002105 | Polyketide | 32.0 | 22.3 | 352.0 | 3.4e-97 |
| AAQ84156.1 | Plm1 | BGC0000123 | Polyketide | 32.0 | 22.8 | 354.0 | 4.41e-97 |
| AEC13080.1 | fosB | BGC0000060 | Polyketide | 30.0 | 22.3 | 352.0 | 6.35e-97 |
| AJO72734.1 | Type\_I\_modular\_polyketide\_synthase | BGC0001381 | Polyketide | 35.0 | 20.1 | 354.0 | 6.57e-97 |
| ALA09354.1 | type\_I\_modular\_PKS | BGC0001303 | Polyketide | 29.0 | 26.5 | 351.0 | 9.44e-97 |
| BAG23200.1 | putative\_type-I\_PKS | BGC0002673 | Polyketide+Alkaloid | 29.0 | 23.4 | 353.0 | 9.73e-97 |
| ACO94460.1 | polyketide\_synthase\_type\_I | BGC0000029 | Polyketide:Modular type I polyketide | 31.0 | 22.6 | 353.0 | 9.74e-97 |
| ABP55220.1 | beta-ketoacyl\_synthase | BGC0000142 | Polyketide | 32.0 | 22.5 | 353.0 | 1.02e-96 |
| WP\_063764078.1 | polyketide\_synthase | BGC0001348 | Polyketide:Modular type I polyketide | 32.0 | 22.3 | 352.0 | 1.2e-96 |
| TXD00266.1 | SDR\_family\_NAD(P)-dependent\_oxidoreductase | BGC0001877 | Polyketide | 31.0 | 22.7 | 353.0 | 1.4e-96 |
| QKV49769.1 | PKS | BGC0002526 | Polyketide | 31.0 | 22.6 | 352.0 | 2.7e-96 |
| ALJ49921.1 | TtmH | BGC0001236 | Polyketide | 29.0 | 25.4 | 351.0 | 3.26e-96 |
| QBL56210.1 | PKS | BGC0002376 | Polyketide | 30.0 | 24.2 | 352.0 | 3.76e-96 |
| AEP40932.1 | polyketide\_synthase\_type\_I | BGC0000021 | Polyketide | 30.0 | 24.9 | 349.0 | 4.3e-96 |
| CAF05651.1 | TubF\_protein | BGC0001053 | NRP+Polyketide | 33.0 | 20.6 | 351.0 | 5e-96 |
| AWW87425.1 | polyketide\_synthase | BGC0001755 | Polyketide | 31.0 | 22.6 | 352.0 | 5.46e-96 |
| ABY21539.1 | AngAII | BGC0000018 | Polyketide | 28.0 | 29.6 | 350.0 | 5.5e-96 |
| ABP55223.1 | beta-ketoacyl\_synthase | BGC0000142 | Polyketide | 32.0 | 23.9 | 351.0 | 6.29e-96 |
| AAF71767.1 | nysJ | BGC0000115 | Polyketide:Modular type I polyketide+Saccharide:Hybrid/tailoring saccharide | 32.0 | 22.6 | 351.0 | 8.41e-96 |
| ADC79639.1 | TamAIII | BGC0001052 | NRP+Polyketide:Modular type I polyketide | 32.0 | 20.4 | 350.0 | 1.1e-95 |
| AAF86393.1 | FkbB | BGC0000994 | NRP+Polyketide | 31.0 | 22.1 | 350.0 | 1.23e-95 |
| QIQ28637.1 | Nbc41 | BGC0002541 | Other | 30.0 | 22.6 | 345.0 | 1.47e-95 |
| WP\_245661588.1 | hypothetical\_protein | BGC0001348 | Polyketide:Modular type I polyketide | 32.0 | 20.7 | 350.0 | 1.55e-95 |
| QIE07129.1 | OvmL3 | BGC0001719 | Polyketide | 29.0 | 28.7 | 347.0 | 2.03e-95 |
| BAC57032.1 | protomycinolide\_IV\_synthase\_5 | BGC0000102 | Polyketide | 32.0 | 22.3 | 348.0 | 2.08e-95 |
| AAF00957.1 | mcyG | BGC0001017 | NRP+Polyketide:Modular type I polyketide | 31.0 | 20.7 | 348.0 | 2.93e-95 |
| AFV30249.1 | polyketide\_synthase | BGC0000075 | Polyketide | 31.0 | 20.1 | 346.0 | 3.55e-95 |
| QFU80900.1 | PKS | BGC0002550 | Polyketide | 32.0 | 22.6 | 348.0 | 3.76e-95 |
| AVX51108.1 | nysC | BGC0001709 | Polyketide | 30.0 | 22.7 | 349.0 | 3.93e-95 |
| ALP32043.1 | CycC | BGC0001293 | Polyketide | 29.0 | 23.3 | 348.0 | 4.38e-95 |
| ALA09357.1 | type\_I\_modular\_PKS | BGC0001303 | Polyketide | 32.0 | 22.1 | 348.0 | 4.59e-95 |
| AIG62146.1 | 6-methylsalicylic\_acid\_synthase | BGC0000120 | Polyketide:Iterative type I polyketide | 30.0 | 22.8 | 345.0 | 4.66e-95 |
| CAQ43079.1 | polyketide\_synthase | BGC0000970 | NRP+Polyketide:Modular type I polyketide | 34.0 | 20.0 | 346.0 | 4.95e-95 |
| ABV83230.1 | CppC | BGC0000116 | Polyketide | 31.0 | 22.5 | 348.0 | 5.13e-95 |
| CCP20049.1 | divL2\_protein | BGC0001119 | Polyketide:Modular type I polyketide | 31.0 | 22.6 | 345.0 | 6.63e-95 |
| QCQ67877.1 | hybrid\_peptide\_synthetase/polyketide\_synthase | BGC0002297 | NRP+Polyketide | 30.0 | 22.0 | 347.0 | 6.63e-95 |
| BAT51067.1 | type\_I\_polyketide\_synthase | BGC0001296 | Polyketide | 32.0 | 20.5 | 346.0 | 7.86e-95 |
| AKA59088.1 | type-I\_PKS | BGC0001619 | Polyketide | 33.0 | 20.3 | 347.0 | 7.99e-95 |
| OAP25815.1 | Phenolphthiocerol\_synthesis\_polyketide\_synthase\_type\_I\_Pks15/1 | BGC0001658 | Polyketide | 30.0 | 24.1 | 347.0 | 9.55e-95 |
| AFO85453.1 | non-ribosomal\_peptide\_synthetase | BGC0000391 | NRP | 28.0 | 34.0 | 347.0 | 9.83e-95 |
| ARE67851.1 | AbsB3 | BGC0001492 | Polyketide | 31.0 | 20.4 | 335.0 | 9.89e-95 |
| ALD82522.1 | polyketide\_synthase | BGC0001212 | NRP+Polyketide | 28.0 | 29.3 | 347.0 | 1.02e-94 |
| AAF71768.1 | nysK | BGC0000115 | Polyketide:Modular type I polyketide+Saccharide:Hybrid/tailoring saccharide | 30.0 | 22.3 | 345.0 | 1.06e-94 |
| ADC79616.1 | BafAI | BGC0000028 | Polyketide:Modular type I polyketide | 32.0 | 21.2 | 347.0 | 1.17e-94 |
| BAC57031.1 | protomycinolide\_IV\_synthase\_4 | BGC0000102 | Polyketide | 34.0 | 20.2 | 342.0 | 1.53e-94 |
| ANY10588.1 | polyketide\_synthase | BGC0001773 | Polyketide | 32.0 | 22.1 | 345.0 | 1.98e-94 |
| CCP20048.1 | divL1\_protein | BGC0001119 | Polyketide:Modular type I polyketide | 30.0 | 23.3 | 344.0 | 2.99e-94 |
| QIE07128.1 | OvmL2 | BGC0001719 | Polyketide | 31.0 | 22.6 | 343.0 | 3.31e-94 |
| CCA89326.1 | mixed\_trans-AT\_type\_I\_polyketide\_synthase/nonribosomal\_peptide\_synthetase | BGC0001111 | NRP+Polyketide:Trans-AT type I polyketide | 27.0 | 38.2 | 346.0 | 3.41e-94 |
| AWR88399.1 | putative\_beta-ketoacyl\_synthase | BGC0001522 | Polyketide | 30.0 | 27.0 | 345.0 | 3.69e-94 |
| QSV12661.1 | AvmC | BGC0002456 | Polyketide+NRP | 30.0 | 23.8 | 345.0 | 3.77e-94 |
| ESU09199.1 | hypothetical\_protein | BGC0002594 | Polyketide | 30.0 | 20.2 | 343.0 | 3.84e-94 |
| ADA69241.1 | cis-AT\_polyketide\_synthase | BGC0001071 | NRP+Polyketide:Modular type I polyketide+Polyketide:Trans-AT type I polyketide | 32.0 | 20.6 | 344.0 | 3.89e-94 |
| ALA09355.1 | type\_I\_modular\_PKS | BGC0001303 | Polyketide | 32.0 | 20.1 | 344.0 | 6.87e-94 |
| ALA09358.1 | type\_I\_modular\_PKS | BGC0001303 | Polyketide | 30.0 | 22.1 | 339.0 | 8.48e-94 |
| ATD51280.1 | nonribosomal\_peptide\_synthase | BGC0001650 | NRP | 28.0 | 33.3 | 339.0 | 1.21e-93 |
| WP\_055469548.1 | type\_I\_polyketide\_synthase | BGC0001537 | Polyketide | 30.0 | 23.5 | 342.0 | 2.26e-93 |
| OAP25819.1 | Phenolphthiocerol\_synthesis\_polyketide\_synthase\_type\_I\_Pks15/1 | BGC0001658 | Polyketide | 31.0 | 22.7 | 340.0 | 2.66e-93 |
| AKD43761.1 | HerD | BGC0001349 | NRP+Polyketide | 30.0 | 22.8 | 342.0 | 3.89e-93 |
| BCD52390.1 | polyketide\_synthase\_SptM | BGC0002537 | Polyketide+Terpene | 25.0 | 40.4 | 341.0 | 4.26e-93 |
| BBA84070.1 | type\_I\_polyketide\_synthase | BGC0001649 | Polyketide | 32.0 | 22.7 | 342.0 | 5.34e-93 |
| ADZ24995.1 | non-ribosomal\_peptide\_synthase/polyketide\_synthase | BGC0000380 | NRP+Polyketide:Modular type I polyketide | 29.0 | 27.6 | 341.0 | 5.88e-93 |
| AVX51106.1 | nysA | BGC0001709 | Polyketide | 31.0 | 22.1 | 335.0 | 6.07e-93 |
| AVX51100.1 | nysK | BGC0001709 | Polyketide | 31.0 | 22.3 | 340.0 | 6.14e-93 |
| CBD77746.1 | non-ribosomal\_peptide\_synthetase/polyketide\_synthase | BGC0000974 | NRP+Polyketide | 31.0 | 22.5 | 340.0 | 7.58e-93 |
| BAP81867.1 | AndM | BGC0002612 | Terpene | 25.0 | 40.4 | 340.0 | 9.58e-93 |
| BAC57030.1 | protomycinolide\_IV\_synthase\_3 | BGC0000102 | Polyketide | 33.0 | 20.4 | 339.0 | 2.08e-92 |
| AGC09499.1 | LobS4 | BGC0001183 | Polyketide | 28.0 | 26.9 | 339.0 | 2.96e-92 |
| QGY73449.1 | Itm17 | BGC0002451 | Polyketide | 26.0 | 40.1 | 339.0 | 3.27e-92 |
| BAG23199.1 | putative\_type-I\_PKS | BGC0002673 | Polyketide+Alkaloid | 30.0 | 23.5 | 337.0 | 3.37e-92 |
| AHB82051.1 | polyketide\_synthase | BGC0001019 | NRP+Polyketide:Modular type I polyketide | 29.0 | 28.1 | 338.0 | 5.55e-92 |
| AOC89001.1 | putative\_nonribosomal\_peptide\_synthetase | BGC0001652 | NRP | 28.0 | 30.6 | 333.0 | 7.71e-92 |
| ALG65339.1 | Var4 | BGC0002416 | NRP+Polyketide | 33.0 | 20.6 | 337.0 | 7.72e-92 |
| BAV69313.1 | PrhL | BGC0001729 | Polyketide+Terpene | 26.0 | 40.5 | 336.0 | 1.13e-91 |
| AAO65798.1 | monensin\_polyketide\_synthase\_modules\_3\_and\_4 | BGC0000100 | Polyketide | 29.0 | 28.2 | 336.0 | 2.55e-91 |
| ANZ52461.1 | MonAIII | BGC0001670 | Polyketide | 29.0 | 28.2 | 336.0 | 2.55e-91 |
| CAC22144.1 | CpkC;\_Polyketide\_synthase\_module\_5 | BGC0000038 | Polyketide:Modular type I polyketide | 28.0 | 22.4 | 333.0 | 7.15e-91 |
| EJK79843.1 | amino\_acid\_adenylation\_enzyme/thioester\_reductase\_family\_protein | BGC0000436 | NRP | 33.0 | 20.1 | 334.0 | 9.79e-91 |
| OJF16270.1 | AceP3 | BGC0001491 | Polyketide | 33.0 | 20.4 | 334.0 | 1.01e-90 |
| AKD43765.1 | HerE | BGC0001349 | NRP+Polyketide | 30.0 | 22.5 | 330.0 | 2.25e-90 |
| ABV83223.1 | CppK | BGC0000116 | Polyketide | 31.0 | 22.1 | 331.0 | 3.05e-90 |
| AHE80992.1 | PieA2 | BGC0001169 | Polyketide:Modular type I polyketide | 30.0 | 22.7 | 331.0 | 5.49e-90 |
| AQZ37114.1 | polyketide\_synthase | BGC0001511 | Polyketide | 30.0 | 25.6 | 331.0 | 6.73e-90 |
| QKV49789.1 | PKS | BGC0002526 | Polyketide | 30.0 | 20.5 | 326.0 | 1.56e-89 |
| BAP34763.1 | type\_I\_polyketide\_synthase | BGC0000078 | Polyketide | 30.0 | 22.8 | 330.0 | 1.8e-89 |
| AFD30954.1 | CrmA | BGC0000966 | NRP+Polyketide | 34.0 | 20.8 | 328.0 | 2.5e-89 |
| QIE07126.1 | OvmK4 | BGC0001719 | Polyketide | 31.0 | 21.2 | 328.0 | 3.5e-89 |
| AHB82062.1 | polyketide\_synthase | BGC0001231 | NRP+Polyketide:Modular type I polyketide | 34.0 | 20.5 | 326.0 | 1.88e-88 |
| CAD29795.1 | peptide\_synthetase | BGC0001015 | NRP+Polyketide | 30.0 | 20.7 | 325.0 | 3.1e-88 |
| KAF7597159.1 | hypothetical\_protein | BGC0002646 | Polyketide | 29.0 | 24.5 | 320.0 | 3.85e-87 |
| PKY07881.1 | hypothetical\_protein | BGC0001544 | NRP+Polyketide | 28.0 | 28.0 | 321.0 | 4.85e-87 |
| RAT98527.1 | trans-acyltransferase\_polyketide\_synthase | BGC0001470 | Polyketide:Trans-AT type I polyketide | 25.0 | 41.0 | 321.0 | 7.7e-87 |
| ALD83687.1 | tAT\_polyketide\_synthase | BGC0001300 | Polyketide | 26.0 | 39.5 | 321.0 | 9.29e-87 |
| EGD99348.1 | polyketide\_synthase | BGC0001144 | Polyketide | 28.0 | 26.0 | 318.0 | 1.16e-86 |
| ADY00130.1 | polyketide\_synthase | BGC0000104 | Terpene+Polyketide:Iterative type I polyketide | 25.0 | 40.0 | 319.0 | 2.07e-86 |
| CDM36726.1 | Beta-ketoacyl\_synthase | BGC0001360 | Polyketide | 25.0 | 40.1 | 318.0 | 3.61e-86 |
| AQZ37095.1 | polyketide\_synthase | BGC0001511 | Polyketide | 31.0 | 20.1 | 317.0 | 1.13e-85 |
| AJF34464.1 | Txo2 | BGC0001207 | NRP | 29.0 | 26.4 | 316.0 | 2.69e-85 |
| CTQ34882.1 | AtcE;\_polyketide\_synthase,\_modules\_5-7 | BGC0001301 | Polyketide | 25.0 | 39.7 | 314.0 | 1.17e-84 |
| AAM54078.1 | polyketide\_synthase | BGC0000020 | Polyketide | 31.0 | 20.3 | 313.0 | 2.17e-84 |
| XP\_028481820.1 | non-reducing\_polyketide\_synthase | BGC0001866 | Polyketide | 27.0 | 24.7 | 312.0 | 2.34e-84 |
| EAU31624.1 | hypothetical\_protein | BGC0002592 | Polyketide | 30.0 | 20.8 | 311.0 | 2.54e-84 |
| QCL09091.1 | dmx-nrPKS | BGC0002063 | Polyketide:Iterative type I polyketide | 31.0 | 20.9 | 310.0 | 3.42e-84 |
| KAF7526531.1 | hypothetical\_protein | BGC0002244 | Polyketide | 30.0 | 21.3 | 310.0 | 3.75e-84 |
| ibo19 |  | BGC0001619 | Polyketide | 30.0 | 20.7 | 310.0 | 1.51e-83 |
| EAL89339.1 | polyketide\_synthase,\_putative | BGC0001403 | Polyketide | 27.0 | 25.4 | 308.0 | 1.77e-83 |
| AFV52200.1 | polyketide\_synthase\_module | BGC0000081 | NRP+Polyketide:Iterative type I polyketide+Polyketide:Enediyne type I polyketide | 33.0 | 20.1 | 308.0 | 1.85e-83 |
| CCE67070.1 | polyketide\_synthase | BGC0001242 | Polyketide | 28.0 | 23.8 | 308.0 | 3.51e-83 |
| EAA59563.1 | polyketide\_synthase | BGC0000057 | Polyketide:Iterative type I polyketide | 29.0 | 24.5 | 307.0 | 6.69e-83 |
| EWM63002.1 | non-ribosomal\_peptide\_synthetase | BGC0001328 | NRP:Cyclic depsipeptide+Polyketide:Modular type I polyketide | 28.0 | 26.8 | 300.0 | 8.26e-83 |
| AXN93602.1 | PuwF-G | BGC0001952 | NRP | 28.0 | 25.7 | 308.0 | 9.56e-83 |
| AGD80618.1 | non-ribosomal\_peptide\_synthetase | BGC0000394 | NRP | 27.0 | 32.9 | 303.0 | 2.08e-82 |
| WP\_030498974.1 | tyrocidine\_synthase\_3 | BGC0001327 | NRP:Cyclic depsipeptide+Polyketide:Modular type I polyketide | 28.0 | 26.7 | 298.0 | 3.51e-82 |
| PIB02405.1 | CTB1 | BGC0001541 | Polyketide | 29.0 | 20.1 | 303.0 | 1.1e-81 |
| ADM79459.1 | PKS16\_protein | BGC0001266 | Polyketide | 28.0 | 24.5 | 303.0 | 1.29e-81 |
| ADI24953.1 | GsfA | BGC0000070 | Polyketide:Iterative type I polyketide | 28.0 | 22.9 | 301.0 | 1.87e-81 |
| CCE33500.1 | polyketide\_synthase\_that\_catalyse\_the\_condensation\_of\_one\_acetyl-CoA\_and\_six\_malonyl-CoA\_resulting\_in\_formation\_of\_nor-rubrofusarin | BGC0002596 | Polyketide | 28.0 | 27.2 | 302.0 | 1.93e-81 |
| ARU80380.1 | polyketide\_synthase | BGC0001542 | Polyketide | 28.0 | 21.6 | 302.0 | 2.45e-81 |
| BAE62229.1 |  | BGC0002237 | Polyketide | 31.0 | 20.3 | 300.0 | 6.68e-81 |
| BAF50727.1 | hybrid\_polyketide\_synthase-non\_ribosomal\_peptide\_synthetase | BGC0001116 | NRP+Polyketide | 26.0 | 38.8 | 301.0 | 7.32e-81 |
| AHV78253.1 | ResS2 | BGC0001246 | Polyketide | 29.0 | 25.0 | 300.0 | 7.33e-81 |
| AAO23333.1 | NcpA | BGC0000397 | NRP | 26.0 | 26.0 | 301.0 | 9.73e-81 |
| OJF16267.1 | AceP5 | BGC0001491 | Polyketide | 30.0 | 21.7 | 300.0 | 1.14e-80 |
| ACD39770.1 | non-reducing\_polyketide\_synthase | BGC0000134 | Polyketide | 26.0 | 23.5 | 299.0 | 1.48e-80 |
| AGO59040.1 | PtaA | BGC0000121 | Polyketide | 30.0 | 20.9 | 298.0 | 1.5e-80 |
| AAK57184.1 | MxaA | BGC0001022 | NRP+Polyketide | 28.0 | 30.5 | 297.0 | 1.58e-80 |
| CBD77749.1 | non-ribosomal\_peptide\_synthetase | BGC0000974 | NRP+Polyketide | 27.0 | 25.9 | 297.0 | 2.29e-80 |
| ABW71853.1 | nonribosomal\_peptide\_synthetase | BGC0000303 | NRP | 27.0 | 32.6 | 295.0 | 7.78e-80 |
| AQW44894.1 | non-ribosomal\_peptide\_synthetase | BGC0001737 | NRP+Polyketide | 27.0 | 30.8 | 295.0 | 8.28e-80 |
| QDK64760.1 | AshP | BGC0002301 | Polyketide | 28.0 | 25.1 | 296.0 | 9.99e-80 |
| ATJ04411.1 | NRPS,\_TomB\_binding | BGC0001637 | NRP | 26.0 | 30.6 | 294.0 | 1.08e-79 |
| AJF34463.1 | Txo1 | BGC0001207 | NRP | 28.0 | 25.6 | 297.0 | 1.37e-79 |
| ABW17377.1 | PsoC | BGC0000411 | NRP | 27.0 | 25.9 | 297.0 | 1.43e-79 |
| ACN39727.1 | SibD | BGC0000428 | NRP | 26.0 | 30.3 | 294.0 | 1.81e-79 |
| UEF20578.1 | nonribosomal\_peptide\_synthetase | BGC0002360 | NRP | 29.0 | 26.1 | 296.0 | 2.19e-79 |
| OAQ63055.1 | polyketide\_synthase | BGC0002187 | Polyketide | 26.0 | 23.4 | 295.0 | 2.2e-79 |
| WP\_019032755.1 | non-ribosomal\_peptide\_synthetase | BGC0001331 | NRP:Cyclic depsipeptide+Polyketide:Modular type I polyketide | 29.0 | 25.4 | 295.0 | 2.32e-79 |
| AEA29644.1 | putative\_nonribosomal\_peptide\_synthetase\_and\_kinurenine\_monooxygenase | BGC0000409 | NRP | 27.0 | 32.5 | 295.0 | 2.43e-79 |
| APZ78704.1 | nonribosomal\_peptide\_synthetase | BGC0001419 | NRP:Cyclic depsipeptide+Polyketide:Iterative type I polyketide | 28.0 | 26.0 | 296.0 | 2.68e-79 |
| WP\_018540603.1 | non-ribosomal\_peptide\_synthetase | BGC0001332 | NRP+Polyketide | 29.0 | 25.2 | 289.0 | 2.99e-79 |
| AQM37584.1 | nonribosomal\_peptide\_synthetase | BGC0001424 | NRP:Cyclic depsipeptide+Polyketide:Iterative type I polyketide | 28.0 | 26.0 | 295.0 | 3.94e-79 |
| APZ78692.1 | nonribosomal\_peptide\_synthetase | BGC0001418 | NRP:Cyclic depsipeptide+Polyketide:Iterative type I polyketide | 28.0 | 26.0 | 294.0 | 1.02e-78 |
| AXA20096.1 | trans-AT\_PKS\_LgaG | BGC0001646 | NRP+Polyketide | 25.0 | 39.8 | 294.0 | 1.26e-78 |
| AAZ95017.1 | polyketide\_synthase | BGC0000048 | Polyketide | 30.0 | 20.4 | 293.0 | 1.83e-78 |
| CAJ34382.1 | NRPS\_protein | BGC0000445 | NRP:Cyclic depsipeptide | 28.0 | 25.1 | 289.0 | 2.1e-78 |
| AEH59100.1 | amino\_acid\_adenylation\_domain-containing\_protein/NRPS | BGC0000385 | NRP | 28.0 | 26.0 | 293.0 | 2.13e-78 |
| APZ78716.1 | nonribosomal\_peptide\_synthetase | BGC0001420 | NRP:Cyclic depsipeptide+Polyketide:Iterative type I polyketide | 28.0 | 26.0 | 293.0 | 2.29e-78 |
| AVI26393.1 | nonribosomal\_peptide\_synthase | BGC0001800 | NRP+Polyketide | 28.0 | 26.0 | 293.0 | 2.85e-78 |
| EED57518.1 | polyketide\_synthase,\_putative | BGC0001446 | Polyketide:Iterative type I polyketide | 28.0 | 23.2 | 291.0 | 3.15e-78 |
| QKF54436.1 | nonribosomal\_peptide\_synthetase | BGC0002581 | NRP | 29.0 | 26.1 | 292.0 | 4.21e-78 |
| WP\_053065269.1 | non-ribosomal\_peptide\_synthetase | BGC0001330 | NRP:Cyclic depsipeptide+Polyketide:Modular type I polyketide | 29.0 | 25.5 | 291.0 | 4.52e-78 |
| AAC44129.1 | saframycin\_Mx1\_synthetase\_A | BGC0002706 | NRP | 26.0 | 31.5 | 291.0 | 4.56e-78 |
| EAU29529.1 | hypothetical\_protein | BGC0000682 | Terpene | 25.0 | 40.8 | 291.0 | 5.45e-78 |
| AFH75322.1 | nonribosomal\_peptide\_synthetase | BGC0000425 | NRP:Cyclic depsipeptide | 28.0 | 24.4 | 291.0 | 7.8e-78 |
| ctg1\_orf19 |  | BGC0001013 | NRP+Polyketide | 27.0 | 26.0 | 289.0 | 8.2e-78 |
| ABC87508.1 | NRPS\_for\_pipecolate\_incorporation | BGC0001011 | NRP+Polyketide | 27.0 | 26.0 | 289.0 | 8.65e-78 |
| ABY83163.1 | Azi25 | BGC0000960 | NRP+Polyketide | 27.0 | 32.6 | 288.0 | 1.02e-77 |
| CAJ96468.1 | non-ribosomal\_peptide\_synthetase | BGC0000330 | NRP:NRP siderophore | 26.0 | 25.5 | 283.0 | 1.16e-77 |
| ESU07748.1 | hypothetical\_protein | BGC0002709 | Polyketide | 28.0 | 24.6 | 289.0 | 2.14e-77 |
| AFJ23826.1 | WLIP\_synthetase\_C | BGC0001838 | NRP | 28.0 | 24.1 | 289.0 | 3.09e-77 |
| BAO84866.1 | putative\_non-ribosomal\_peptide\_synthetase | BGC0000414 | NRP | 27.0 | 32.8 | 286.0 | 3.24e-77 |
| QNH85840.1 | BolH | BGC0002327 | NRP | 28.0 | 26.3 | 289.0 | 4.41e-77 |
| AAK89719.2 | peptide\_synthetase,\_siderophore\_biosynthesis\_protein | BGC0002107 | NRP+Polyketide | 28.0 | 25.7 | 288.0 | 5.05e-77 |
| ALV82356.1 | CDA\_peptide\_synthetase\_I | BGC0001370 | NRP | 28.0 | 26.0 | 288.0 | 6.7e-77 |
| AAY37647.1 | Amino\_acid\_adenylation | BGC0000437 | NRP | 28.0 | 26.0 | 288.0 | 7.06e-77 |
| WP\_126241403.1 | non-ribosomal\_peptide\_synthetase | BGC0002336 | NRP | 28.0 | 26.4 | 287.0 | 1.29e-76 |
| AAO23334.1 | NcpB | BGC0000397 | NRP | 27.0 | 26.8 | 287.0 | 1.32e-76 |
| AXA94654.1 | hypothetical\_protein | BGC0002044 | NRP | 29.0 | 24.9 | 281.0 | 1.51e-76 |
| BAH43869.1 | linear\_pentadecapeptide\_gramicidin\_synthetase\_LgrA | BGC0000367 | NRP | 26.0 | 25.5 | 286.0 | 1.66e-76 |
| AXG46163.1 | non-ribosomal\_peptide\_synthetase | BGC0002713 | NRP | 26.0 | 26.6 | 281.0 | 1.84e-76 |
| CDG76959.1 | non-ribosomal\_peptide\_synthetase,\_terminal\_component | BGC0000446 | NRP:Pyrrolobenzodiazepine | 26.0 | 30.7 | 284.0 | 1.97e-76 |
| QBG38783.1 | Atr22 | BGC0001975 | NRP | 28.0 | 26.0 | 286.0 | 2.6e-76 |
| CAB38518.1 | CDA\_peptide\_synthetase\_I\_(CdaPs1) | BGC0000315 | NRP:Lipopeptide:Ca+-dependent lipopeptide | 28.0 | 26.8 | 286.0 | 3.36e-76 |
| CAJ14037.1 | peptide\_synthetase | BGC0000406 | NRP | 28.0 | 25.5 | 280.0 | 3.99e-76 |
| ART41209.1 | AdrD | BGC0001508 | Polyketide | 29.0 | 24.6 | 285.0 | 4.22e-76 |
| AAK89727.1 | peptide\_synthetase,\_siderophore\_biosynthesis\_protein | BGC0002107 | NRP+Polyketide | 25.0 | 34.0 | 283.0 | 4.47e-76 |
| CAL69890.1 | RhiC\_protein | BGC0001112 | NRP+Polyketide:Trans-AT type I polyketide | 26.0 | 37.6 | 285.0 | 8.9e-76 |
| DAB41653.1 | polyketide\_synthase | BGC0001583 | Polyketide | 30.0 | 20.6 | 282.0 | 1.15e-75 |
| CBF74114.1 | Conidial\_yellow\_pigment\_biosynthesis\_polyketide\_synthase\_(PKS)(EC\_2.3.1.-)\_[Source:UniProtKB/Swiss-Prot;Acc:Q03149] | BGC0000107 | Polyketide | 27.0 | 24.5 | 283.0 | 1.74e-75 |
| QBG38888.1 | nr-PKS | BGC0002062 | Polyketide | 30.0 | 21.1 | 282.0 | 2.05e-75 |
| QWP75305.1 | non-ribosomal\_peptide\_synthase | BGC0002126 | NRP:Cyclic depsipeptide | 28.0 | 26.1 | 283.0 | 2.12e-75 |
| CAG29032.1 | nonribosomal\_peptide\_synthetase\_(modules\_3\_to\_6) | BGC0001023 | NRP+Polyketide:Modular type I polyketide | 27.0 | 25.8 | 283.0 | 2.45e-75 |
| APZ78822.1 | nonribosomal\_peptide\_synthetase | BGC0001429 | NRP:Cyclic depsipeptide+Polyketide:Iterative type I polyketide | 27.0 | 25.8 | 283.0 | 2.45e-75 |
| ABI22132.1 | putative\_non-ribosomal\_peptide\_synthetase | BGC0000422 | NRP | 27.0 | 24.9 | 276.0 | 4.07e-75 |
| ABC36203.1 | Gonyol-Synthetase\_(NRPS-PKS\_hybrid) | BGC0001102 | NRP+Polyketide:Modular type I polyketide+Polyketide:Trans-AT type I polyketide | 31.0 | 20.1 | 282.0 | 4.08e-75 |
| AAT12283.1 | LtxA | BGC0000384 | NRP | 26.0 | 34.6 | 281.0 | 4.72e-75 |
| AAY93445.1 | non-ribosomal\_peptide\_synthetase\_PvdL | BGC0000413 | NRP | 28.0 | 26.2 | 281.0 | 7.07e-75 |
| WP\_012408783.1 | non-ribosomal\_peptide\_synthetase | BGC0002061 | NRP:Cyclic depsipeptide+Polyketide:Modular type I polyketide | 27.0 | 25.1 | 281.0 | 7.11e-75 |
| AWI62626.1 | nonribosomal\_peptide\_synthetase | BGC0001822 | NRP | 27.0 | 25.7 | 281.0 | 7.44e-75 |
| ATY37609.1 | BreD | BGC0001536 | NRP | 24.0 | 25.8 | 281.0 | 7.76e-75 |
| AAY37655.1 | Amino\_acid\_adenylation | BGC0000437 | NRP | 27.0 | 26.1 | 281.0 | 9.2e-75 |
| APZ78834.1 | nonribosomal\_peptide\_synthetase | BGC0001430 | NRP:Cyclic depsipeptide+Polyketide:Iterative type I polyketide | 27.0 | 25.4 | 281.0 | 1.22e-74 |
| AAO72425.1 | syringopeptin\_synthetase\_C | BGC0000438 | NRP | 27.0 | 26.1 | 281.0 | 1.57e-74 |
| EAU38791.1 | hypothetical\_protein | BGC0000161 | Polyketide:Iterative type I polyketide | 28.0 | 21.4 | 279.0 | 1.86e-74 |
| MBE8994631.1 | amino\_acid\_adenylation\_domain-containing\_protein | BGC0002623 | NRP+Polyketide | 27.0 | 26.7 | 275.0 | 1.89e-74 |
| APZ78680.1 | nonribosomal\_peptide\_synthetase | BGC0001417 | NRP:Cyclic depsipeptide+Polyketide:Iterative type I polyketide | 28.0 | 26.1 | 280.0 | 1.96e-74 |
| AAF17280.1 | nosC | BGC0001028 | Polyketide+NRP:Cyclic depsipeptide | 27.0 | 25.8 | 280.0 | 2.34e-74 |
| CAQ71829.1 | non\_ribosomal\_peptide\_synthase,\_antibiotic\_synthesis;\_contains\_4\_condensation\_domains,\_3\_AMP-acid\_ligases\_II\_domains,\_3\_PP-binding,\_Phosphopantetheine\_attachment\_site\_and\_a\_putative\_thioesterase\_domain | BGC0001189 | NRP | 28.0 | 26.0 | 280.0 | 2.59e-74 |
| ATY37591.1 | BogD | BGC0001532 | NRP | 25.0 | 25.9 | 280.0 | 2.77e-74 |
| AHZ34243.1 | CipF | BGC0001389 | NRP | 26.0 | 26.1 | 279.0 | 4.14e-74 |
| AAF15891.2 | nosA | BGC0001028 | Polyketide+NRP:Cyclic depsipeptide | 27.0 | 26.7 | 279.0 | 4.64e-74 |
| AAY93354.1 | non-ribosomal\_peptide\_synthetase\_PvdD | BGC0000413 | NRP | 27.0 | 26.0 | 279.0 | 4.66e-74 |
| CUX96955.1 | TmcH | BGC0001829 | NRP+Polyketide | 30.0 | 20.1 | 277.0 | 6.99e-74 |
| CCP42826.1 | Probable\_peptide\_synthetase\_Nrp\_(peptide\_synthase) | BGC0001627 | NRP | 26.0 | 32.7 | 278.0 | 7.08e-74 |
| AEP40925.1 | polyketide\_synthase\_type\_I | BGC0000021 | Polyketide | 30.0 | 22.1 | 276.0 | 7.48e-74 |
| ATJ34002.1 | non-ribosomal\_peptide\_synthetase | BGC0001442 | NRP | 26.0 | 33.0 | 276.0 | 7.8e-74 |
| ACO78738.1 | Non-ribosomal\_peptide\_synthase,\_PvdJ(2)-like\_protein | BGC0002433 | NRP | 27.0 | 26.4 | 278.0 | 8.2e-74 |
| ABS90473.1 | NRPS | BGC0001106 | NRP+Polyketide | 29.0 | 22.7 | 277.0 | 8.69e-74 |
| XP\_001798923.1 | polyketide\_synthase | BGC0001865 | Polyketide:Iterative type I polyketide | 28.0 | 22.4 | 276.0 | 1.04e-73 |
| ALK27915.1 | non-ribosomal\_peptide\_synthase | BGC0001233 | NRP | 28.0 | 26.4 | 278.0 | 1.24e-73 |
| BAY02139.1 | nonribosomal\_protein\_synthetase | BGC0002532 | NRP+Polyketide | 25.0 | 30.6 | 275.0 | 1.45e-73 |
| QDF82259.1 | non-ribosomal\_peptide\_synthetase | BGC0001980 | NRP | 27.0 | 26.2 | 277.0 | 1.51e-73 |
| KDB16994.1 | polyketide\_synthetase\_PksP | BGC0002177 | Polyketide | 28.0 | 20.9 | 276.0 | 2.05e-73 |
| WP\_039806852.1 | non-ribosomal\_peptide\_synthetase | BGC0002001 | NRP+Polyketide | 27.0 | 26.3 | 271.0 | 2.28e-73 |
| ABI22133.1 | putative\_non-ribosomal\_peptide\_synthetase | BGC0000422 | NRP | 27.0 | 32.4 | 273.0 | 6.71e-73 |
| APZ78846.1 | nonribosomal\_peptide\_synthetase | BGC0001431 | NRP:Cyclic depsipeptide+Polyketide:Iterative type I polyketide | 27.0 | 25.8 | 275.0 | 6.84e-73 |
| AEA30273.1 | peptide\_synthetase | BGC0000429 | Polyketide+NRP:Cyclic depsipeptide | 28.0 | 26.2 | 275.0 | 7e-73 |
| QBG38784.1 | Atr23 | BGC0001975 | NRP | 27.0 | 26.5 | 275.0 | 7.07e-73 |
| ANG60379.1 | nonribosomal\_peptide\_synthetase\_BudA | BGC0001434 | NRP | 26.0 | 26.6 | 270.0 | 7.27e-73 |
| BAV56271.1 |  | BGC0001657 | NRP | 27.0 | 26.2 | 275.0 | 7.72e-73 |
| QGQ63520.1 | nonribosomal\_peptide\_synthetase\_modules\_C | BGC0002548 | NRP | 27.0 | 26.2 | 274.0 | 8.76e-73 |
| QMN69934.1 | PsoC | BGC0002521 | NRP | 27.0 | 25.8 | 274.0 | 1.42e-72 |
| MBE8994632.1 | amino\_acid\_adenylation\_domain-containing\_protein | BGC0002623 | NRP+Polyketide | 27.0 | 26.5 | 273.0 | 1.77e-72 |
| ALV82384.1 | CDA\_peptide\_synthetase\_II | BGC0001370 | NRP | 27.0 | 26.6 | 273.0 | 1.81e-72 |
| QLM00044.1 | polyketide\_synthase | BGC0002257 | Polyketide | 29.0 | 20.8 | 272.0 | 2.33e-72 |
| ACZ55944.1 | non-ribosomal\_peptide\_synthetase | BGC0000302 | NRP | 26.0 | 26.0 | 273.0 | 2.39e-72 |
| AKJ29411.1 | peptide\_synthetase | BGC0001608 | NRP | 27.0 | 26.4 | 273.0 | 2.59e-72 |
| AEH59099.1 | amino\_acid\_adenylation\_domain-containing\_protein/NRPS | BGC0000385 | NRP | 28.0 | 26.3 | 273.0 | 3.19e-72 |
| APZ78782.1 | nonribosomal\_peptide\_synthetase | BGC0001426 | NRP:Cyclic depsipeptide+Polyketide:Iterative type I polyketide | 26.0 | 25.7 | 273.0 | 3.42e-72 |
| APZ78795.1 | nonribosomal\_peptide\_synthetase | BGC0001427 | NRP:Cyclic depsipeptide+Polyketide:Iterative type I polyketide | 26.0 | 25.7 | 273.0 | 3.42e-72 |
| QYA95681.1 | amino\_acid\_adenylation\_domain-containing\_protein | BGC0002676 | NRP | 28.0 | 26.3 | 273.0 | 3.5e-72 |
| AJD47481.1 | amino\_acid\_adenylation\_domain-containing\_protein | BGC0002418 | NRP+Polyketide | 27.0 | 25.0 | 266.0 | 4.02e-72 |
| QKF54435.2 | nonribosomal\_peptide\_synthetase | BGC0002581 | NRP | 28.0 | 25.9 | 272.0 | 4.16e-72 |
| AGI89789.1 | Nonribosomal\_peptide\_synthetase | BGC0001792 | NRP | 28.0 | 25.8 | 272.0 | 5.23e-72 |
| QNL34618.1 | SteC | BGC0002092 | NRP:Cyclic depsipeptide | 26.0 | 26.2 | 271.0 | 8.41e-72 |
| APZ78744.1 | nonribosomal\_peptide\_synthetase | BGC0001422 | NRP:Cyclic depsipeptide+Polyketide:Iterative type I polyketide | 27.0 | 25.9 | 271.0 | 1e-71 |
| AHD05679.1 | putative\_non-ribosomal\_peptide\_ligase/\_polyketide\_synthase\_hybrid | BGC0000402 | NRP | 26.0 | 25.6 | 271.0 | 1.24e-71 |
| BAV57443.1 | NRPS\_(C-A-PCP-TE) | BGC0001818 | NRP | 26.0 | 26.9 | 268.0 | 1.66e-71 |
| ALG65317.1 | Cal19 | BGC0001297 | NRP | 28.0 | 26.5 | 269.0 | 1.75e-71 |
| BBD17760.1 | polyketide\_synthase | BGC0001919 | NRP+Polyketide | 30.0 | 20.0 | 268.0 | 1.82e-71 |
| AGU50953.1 | putative\_non-ribosomal\_peptide\_synthetase | BGC0002417 | NRP+Polyketide | 27.0 | 25.8 | 269.0 | 1.98e-71 |
| ANY58984.1 | non-ribosomal\_synthetase | BGC0001615 | NRP | 26.0 | 25.7 | 267.0 | 2.13e-71 |
| KKP00966.1 | RADS2\_nonreducing\_polyketide\_synthase | BGC0001854 | Polyketide:Iterative type I polyketide | 26.0 | 27.4 | 268.0 | 2.44e-71 |
| QST87270.1 | amino\_acid\_adenylation\_domain-containing\_protein | BGC0002572 | NRP+Polyketide | 26.0 | 25.8 | 270.0 | 2.62e-71 |
| WA1\_15565 | non-ribosomal\_peptide\_synthetase | BGC0002484 | NRP+Polyketide | 26.0 | 26.2 | 270.0 | 2.7e-71 |
| ABV79985.1 | ApnA | BGC0000301 | NRP | 26.0 | 26.7 | 269.0 | 2.89e-71 |
| AED90002.1 | non-ribosomal\_peptide\_synthetase\_ThaA | BGC0000443 | NRP:Beta-lactam | 28.0 | 25.9 | 270.0 | 3.12e-71 |
| AOA33123.1 | Nonribosomal\_peptide\_synthetase | BGC0001346 | NRP:Cyclic depsipeptide | 26.0 | 25.9 | 269.0 | 3.78e-71 |
| QED88054.1 | nonribosomal\_peptide\_synthetase | BGC0001967 | NRP+Polyketide | 28.0 | 26.5 | 269.0 | 3.89e-71 |
| ETS82099.1 | hypothetical\_protein | BGC0002161 | Polyketide | 29.0 | 20.1 | 268.0 | 4.92e-71 |
| AAG31130.1 | MxcG | BGC0001345 | NRP | 27.0 | 34.0 | 266.0 | 4.96e-71 |
| AJK49758.1 | non-ribosomal\_peptide\_synthase | BGC0002565 | NRP | 26.0 | 25.8 | 268.0 | 5.05e-71 |
| BAC67536.1 | arthrofactin\_synthetase\_C | BGC0000305 | NRP:Lipopeptide | 27.0 | 26.2 | 269.0 | 5.49e-71 |
| AAD44234.1 | PstB | BGC0000362 | NRP | 27.0 | 30.4 | 268.0 | 6.01e-71 |
| BAV56270.1 |  | BGC0001657 | NRP | 28.0 | 26.4 | 269.0 | 6.03e-71 |
| EFE73313.1 | nonribosomal\_peptide\_synthetase | BGC0000431 | NRP:Cyclic depsipeptide | 27.0 | 25.9 | 268.0 | 6.15e-71 |
| QEO74981.1 | omn6 | BGC0002078 | NRP:Cyclic depsipeptide | 28.0 | 26.2 | 268.0 | 6.55e-71 |
| AQH32486.1 | peptide\_synthetase | BGC0001667 | NRP+Polyketide | 25.0 | 25.3 | 265.0 | 6.65e-71 |
| AAO62588.1 | peptide\_sythetase | BGC0001016 | NRP+Polyketide | 26.0 | 25.8 | 265.0 | 6.69e-71 |
| BAH43766.1 | tyrocidine\_synthetase\_III | BGC0000452 | NRP | 24.0 | 25.8 | 268.0 | 7.34e-71 |
| AFJ14794.1 | PlpE | BGC0000403 | NRP | 26.0 | 25.8 | 268.0 | 7.76e-71 |
| gene6 |  | BGC0001906 | Polyketide | 28.0 | 24.3 | 267.0 | 8.77e-71 |
| AHZ20773.1 | non-ribosomal\_peptide\_synthase | BGC0000369 | NRP+Saccharide:Hybrid/tailoring saccharide | 26.0 | 25.7 | 267.0 | 1.56e-70 |
| AAD38786.1 | polyketide\_synthase | BGC0001257 | Polyketide | 28.0 | 23.0 | 266.0 | 1.9e-70 |
| ABS74208.1 | fengycin\_synthetase\_B | BGC0001095 | NRP | 25.0 | 25.8 | 266.0 | 3.04e-70 |
| CAF05648.1 | TubC\_protein | BGC0001053 | NRP+Polyketide | 27.0 | 22.6 | 266.0 | 3.1e-70 |
| KYC42612.1 | hypothetical\_protein | BGC0002484 | NRP+Polyketide | 26.0 | 26.1 | 266.0 | 3.55e-70 |
| CBW75451.1 | Non-ribosomal\_peptide\_synthetase\_modules | BGC0002048 | NRP:Cyclic depsipeptide | 27.0 | 25.8 | 266.0 | 3.84e-70 |
| QIH29229.1 | endopyrrole\_NRPS\_B | BGC0002326 | NRP | 27.0 | 25.8 | 266.0 | 3.84e-70 |
| QNL14923.1 | AptC | BGC0002512 | NRP | 26.0 | 26.0 | 265.0 | 4.01e-70 |
| ACO78745.1 | Non-ribosomal\_peptide\_synthase:Amino\_acid\_adenylation | BGC0002433 | NRP | 27.0 | 26.0 | 266.0 | 4.22e-70 |
| APZ78769.1 | nonribosomal\_peptide\_synthetase | BGC0001425 | NRP:Cyclic depsipeptide+Polyketide:Iterative type I polyketide | 26.0 | 25.7 | 266.0 | 4.27e-70 |
| QNL14922.1 | AptA | BGC0002512 | NRP | 27.0 | 26.5 | 265.0 | 4.38e-70 |
| MCF2151708.1 | Non-ribosomal\_peptide\_synthetase | BGC0002625 | NRP+Polyketide | 25.0 | 26.6 | 266.0 | 4.81e-70 |
| ACS20360.1 | amino\_acid\_adenylation\_domain\_protein | BGC0002420 | NRP+Polyketide | 27.0 | 26.3 | 260.0 | 5.24e-70 |
| ABI26078.1 | OciB | BGC0000331 | NRP | 26.0 | 26.1 | 265.0 | 5.71e-70 |
| CBF83139.1 | polyketide\_synthase,\_putative\_(JCVI) | BGC0001722 | Polyketide | 30.0 | 20.2 | 265.0 | 5.71e-70 |
| QKM21620.1 | non-ribosomal\_peptide\_synthetase | BGC0002351 | NRP | 28.0 | 25.0 | 265.0 | 6.13e-70 |
| ABJ97436.1 | MerP | BGC0001012 | NRP+Polyketide | 27.0 | 26.5 | 263.0 | 6.69e-70 |
| QBG38782.1 | Atr21 | BGC0001975 | NRP | 27.0 | 26.1 | 265.0 | 6.92e-70 |
| ABF87167.1 | non-ribosomal\_peptide\_synthase\_MxcG | BGC0002492 | NRP | 29.0 | 21.9 | 263.0 | 7.74e-70 |
| ALK27914.1 | non-ribosomal\_peptide\_synthase | BGC0001233 | NRP | 27.0 | 26.2 | 265.0 | 1.02e-69 |
| QWP75304.1 | non-ribosomal\_peptide\_synthase | BGC0002126 | NRP:Cyclic depsipeptide | 27.0 | 26.1 | 264.0 | 1.11e-69 |
| BAH22764.1 | nonribosomal\_peptide\_synthetase | BGC0001018 | NRP | 27.0 | 25.8 | 264.0 | 1.67e-69 |
| ALV86867.1 | Tlo21 | BGC0001406 | NRP | 27.0 | 26.7 | 264.0 | 1.78e-69 |
| AEO14744.1 | NdaB | BGC0000396 | NRP | 26.0 | 25.2 | 260.0 | 1.87e-69 |
| AAC68816.1 | FK506\_peptide\_synthetase | BGC0000353 | NRP | 26.0 | 26.1 | 261.0 | 2.06e-69 |
| ATP76246.1 | SpuB | BGC0001748 | NRP+Polyketide | 25.0 | 25.9 | 263.0 | 2.07e-69 |
| AFH75330.1 | nonribosomal\_peptide\_synthetase | BGC0000398 | NRP:Cyclic depsipeptide | 26.0 | 25.9 | 263.0 | 2.21e-69 |
| ACO78737.1 | Non-ribosomal\_peptide\_synthase,\_PvdD/PvdJ-like\_protein | BGC0002433 | NRP | 26.0 | 26.4 | 263.0 | 2.25e-69 |
| QSJ20135.1 | non-ribosomal\_peptide\_synthase/polyketide\_synthase | BGC0002572 | NRP+Polyketide | 27.0 | 26.2 | 263.0 | 2.67e-69 |
| KIJ60886.1 | polyketide\_synthase | BGC0002214 | Polyketide | 27.0 | 22.9 | 263.0 | 2.69e-69 |
| WP\_144411596.1 | non-ribosomal\_peptide\_synthetase | BGC0002001 | NRP+Polyketide | 27.0 | 26.1 | 260.0 | 2.75e-69 |
| CCJ67648.1 | JagD | BGC0001127 | NRP | 28.0 | 26.4 | 263.0 | 2.91e-69 |
| WP\_064118559.1 | non-ribosomal\_peptide\_synthase/polyketide\_synthase | BGC0001509 | NRP | 26.0 | 26.1 | 263.0 | 4e-69 |
| CAM56770.1 |  | BGC0000354 | NRP | 29.0 | 25.8 | 263.0 | 4.11e-69 |
| KYC42747.1 | hypothetical\_protein | BGC0002484 | NRP+Polyketide | 26.0 | 26.7 | 263.0 | 4.12e-69 |
| ALG65342.1 | Var7 | BGC0002416 | NRP+Polyketide | 28.0 | 25.7 | 262.0 | 4.25e-69 |
| AJV88375.1 | MfnC | BGC0001214 | NRP | 27.0 | 26.1 | 262.0 | 4.38e-69 |
| WP\_012408786.1 | non-ribosomal\_peptide\_synthetase | BGC0002061 | NRP:Cyclic depsipeptide+Polyketide:Modular type I polyketide | 26.0 | 27.4 | 261.0 | 5.79e-69 |
| AGE11898.1 | nonribosomal\_peptide\_synthetase | BGC0000366 | NRP | 27.0 | 26.3 | 261.0 | 7.71e-69 |
| ALG65341.1 | Var6 | BGC0002416 | NRP+Polyketide | 26.0 | 25.8 | 261.0 | 7.71e-69 |
| AEU11003.1 | NpnC | BGC0001029 | NRP+Polyketide | 25.0 | 25.8 | 261.0 | 8.54e-69 |
| AHB38509.1 | polyketide\_synthase | BGC0000345 | NRP+Polyketide:Modular type I polyketide | 30.0 | 20.1 | 260.0 | 9.87e-69 |
| AAG02349.1 | peptide\_synthetase\_NRPS11-10 | BGC0000963 | NRP:Glycopeptide+Polyketide:Modular type I polyketide+Saccharide:Hybrid/tailoring saccharide | 29.0 | 22.7 | 261.0 | 1.09e-68 |
| QUJ09168.1 | Lon21 | BGC0002440 | NRP | 27.0 | 26.6 | 259.0 | 1.14e-68 |
| WP\_018540607.1 | non-ribosomal\_peptide\_synthetase | BGC0001332 | NRP+Polyketide | 26.0 | 26.5 | 261.0 | 1.17e-68 |
| BAE61567.1 |  | BGC0002175 | Polyketide | 27.0 | 24.7 | 260.0 | 1.41e-68 |
| AKJ15828.1 | peptide\_synthetase | BGC0002735 | Polyketide+NRP | 26.0 | 26.1 | 258.0 | 1.44e-68 |
| QSJ20139.1 | non-ribosomal\_peptide\_synthase/polyketide\_synthase | BGC0002572 | NRP+Polyketide | 27.0 | 24.9 | 261.0 | 1.46e-68 |
| AAZ23077.1 | peptide\_synthetase | BGC0000291 | NRP | 29.0 | 26.7 | 261.0 | 1.48e-68 |
| AXM43052.1 | non-ribosomal\_peptide\_synthetase | BGC0001945 | NRP | 27.0 | 26.2 | 260.0 | 1.62e-68 |
| AAN85512.1 | nonribosomal\_peptide\_synthetase | BGC0001101 | NRP+Polyketide:Modular type I polyketide+Polyketide:Trans-AT type I polyketide | 28.0 | 24.4 | 259.0 | 1.68e-68 |
| CBL93730.1 | NRPS | BGC0000360 | NRP | 27.0 | 25.7 | 256.0 | 1.77e-68 |
| ACC81024.1 | non-ribosomal\_peptide\_synthetase | BGC0001479 | NRP | 25.0 | 25.9 | 258.0 | 1.78e-68 |
| QWM97320.1 | non-ribosomal\_peptide\_synthetase | BGC0002384 | NRP | 28.0 | 26.3 | 260.0 | 2.04e-68 |
| AEW31020.1 | plipastatin\_synthetase | BGC0000407 | NRP | 26.0 | 26.0 | 259.0 | 2.24e-68 |
| QSJ20140.1 | amino\_acid\_adenylation\_domain-containing\_protein | BGC0002572 | NRP+Polyketide | 25.0 | 25.9 | 258.0 | 2.32e-68 |
| BAY02137.1 | amino\_acid\_adenylation\_domain-containing\_protein | BGC0002532 | NRP+Polyketide | 26.0 | 25.8 | 259.0 | 2.33e-68 |
| QNH67551.1 | Cip23 | BGC0002108 | NRP | 27.0 | 26.3 | 260.0 | 2.45e-68 |
| CZT62785.1 | Non-ribosomal\_peptide\_synthase,\_involved\_in\_Hassallidin\_biosynthesis | BGC0001614 | NRP | 26.0 | 25.1 | 259.0 | 2.56e-68 |
| WP\_041754829.1 | non-ribosomal\_peptide\_synthetase | BGC0001844 | NRP:Lipopeptide | 26.0 | 25.8 | 260.0 | 2.68e-68 |
| ACC81023.1 | non-ribosomal\_peptide\_synthetase | BGC0001479 | NRP | 26.0 | 25.9 | 259.0 | 2.96e-68 |
| ERF77221.1 | hypothetical\_protein | BGC0002215 | Polyketide | 26.0 | 24.7 | 259.0 | 3.2e-68 |
| ACN39015.1 | putative\_nonribosomal\_peptide\_synthetase\_TomB | BGC0000448 | NRP | 28.0 | 27.5 | 258.0 | 3.49e-68 |
| ATD51278.1 | nonribosomal\_peptide\_synthase | BGC0001650 | NRP | 26.0 | 27.0 | 259.0 | 3.56e-68 |
| CAB15186.3 | siderophore\_2,3-dihydroxybenzoate-glycine-threonine\_trimeric\_ester\_bacillibactin\_synthetase | BGC0000309 | NRP | 26.0 | 26.0 | 259.0 | 3.58e-68 |
| AEF33078.1 | dimodular\_nonribosomal\_peptide\_synthetase | BGC0001039 | NRP+Polyketide | 27.0 | 26.9 | 258.0 | 4.15e-68 |
| NHN68325.1 | amino\_acid\_adenylation\_domain-containing\_protein | BGC0002719 | NRP | 27.0 | 25.6 | 259.0 | 4.46e-68 |
| AID65225.1 | nonribosomal\_peptide\_synthetase | BGC0000335 | NRP+Polyketide | 27.0 | 26.5 | 258.0 | 5.54e-68 |
| QDJ74275.1 | non-ribosomal\_peptide\_synthetase | BGC0002109 | NRP | 27.0 | 26.4 | 259.0 | 5.56e-68 |
| AHD05678.1 | nonribosomal\_peptide\_ligase\_subunit | BGC0000402 | NRP | 26.0 | 24.9 | 259.0 | 5.67e-68 |
| KIA75596.1 | polyketide\_synthase | BGC0002209 | Polyketide | 26.0 | 26.9 | 258.0 | 5.96e-68 |
| AYJ71721.1 | non-ribosomal\_peptide\_synthetase | BGC0001942 | NRP+Polyketide | 27.0 | 25.9 | 258.0 | 6.1e-68 |
| QED55423.1 | nonribosomal\_peptide\_synthetase | BGC0001984 | NRP | 27.0 | 26.5 | 258.0 | 7.92e-68 |
| BAP27942.1 | nonribosomal\_peptide\_synthetase | BGC0001085 | NRP+Terpene | 27.0 | 31.0 | 258.0 | 8.44e-68 |
| ACO78736.1 | Non-ribosomal\_peptide\_synthase,\_PvdD-like\_protein | BGC0002433 | NRP | 26.0 | 26.4 | 258.0 | 9.47e-68 |
| CBG67541.1 | putative\_non-ribosomal\_peptide\_synthetase | BGC0002367 | NRP | 26.0 | 25.7 | 258.0 | 9.77e-68 |
| QKF54438.1 | nonribosomal\_peptide\_synthetase | BGC0002581 | NRP | 28.0 | 26.1 | 258.0 | 1.13e-67 |
| AGU50951.1 | putative\_non-ribosomal\_peptide\_synthetase | BGC0002417 | NRP+Polyketide | 27.0 | 26.1 | 253.0 | 1.2e-67 |
| CBW75453.1 | Non-ribosomal\_peptide\_synthetase\_modules\_(EC\_6.3.2.-) | BGC0002048 | NRP:Cyclic depsipeptide | 26.0 | 26.3 | 257.0 | 1.24e-67 |
| AAT01807.1 | non-ribosomal\_peptide\_synthetase | BGC0000365 | NRP | 26.0 | 30.3 | 257.0 | 1.47e-67 |
| AAC49191.1 | putative\_polyketide\_synthase | BGC0000152 | Polyketide | 27.0 | 20.3 | 256.0 | 1.63e-67 |
| QIH29228.1 | endopyrrole\_NRPS\_A | BGC0002326 | NRP | 26.0 | 26.3 | 257.0 | 1.64e-67 |
| CEK23366.1 | putative\_Ornithine\_racemase | BGC0001716 | NRP | 25.0 | 25.9 | 257.0 | 1.76e-67 |
| SDF67386.1 | non-ribosomal\_peptide\_synthase\_domain\_TIGR01720/amino\_acid\_adenylation\_domain-containing\_protein | BGC0002422 | NRP | 27.0 | 26.0 | 257.0 | 2.07e-67 |
| ATL73036.1 | amino\_acid\_adenylation\_protein | BGC0001807 | NRP+Polyketide | 27.0 | 25.1 | 257.0 | 2.26e-67 |
| ACY06285.1 | non-ribosomal\_peptide\_synthetase | BGC0001042 | NRP+Polyketide | 28.0 | 26.3 | 256.0 | 2.43e-67 |
| UEF20580.1 | nonribosomal\_peptide\_synthetase | BGC0002360 | NRP | 28.0 | 26.1 | 256.0 | 2.54e-67 |
| AEW95634.1 | non-ribosomal\_peptide\_synthetase | BGC0002697 | NRP+Polyketide | 27.0 | 25.1 | 256.0 | 3.09e-67 |
| QPB41098.1 | non-ribosomal\_peptide\_synthetase | BGC0002503 | NRP+Polyketide | 27.0 | 25.8 | 254.0 | 3.38e-67 |
| CAP95404.1 |  | BGC0001404 | Polyketide | 28.0 | 20.3 | 256.0 | 3.42e-67 |
| QNL14925.1 | AptD | BGC0002512 | NRP | 25.0 | 25.8 | 254.0 | 3.57e-67 |
| AEP18656.1 | WAPS1 | BGC0000461 | NRP | 28.0 | 26.3 | 256.0 | 4.16e-67 |
| ACO79122.1 | Type\_I\_fatty\_acid\_synthase\_ArsA | BGC0000284 | Polyketide | 28.0 | 20.1 | 255.0 | 4.24e-67 |
| KMO93435.1 | NRPS/PKS | BGC0002095 | NRP | 27.0 | 26.2 | 251.0 | 4.47e-67 |
| AHB82056.1 | non\_ribosomal\_peptide\_synthetase | BGC0001019 | NRP+Polyketide:Modular type I polyketide | 28.0 | 25.5 | 255.0 | 5.03e-67 |
| ALK27916.1 | non-ribosomal\_peptide\_synthase | BGC0001233 | NRP | 28.0 | 26.5 | 255.0 | 5.28e-67 |
| AAF17281.1 | nosD | BGC0001028 | Polyketide+NRP:Cyclic depsipeptide | 26.0 | 26.4 | 255.0 | 5.44e-67 |
| ABV79987.1 | ApnC | BGC0000301 | NRP | 24.0 | 26.0 | 255.0 | 5.69e-67 |
| WP\_051700112.1 | non-ribosomal\_peptide\_synthetase | BGC0001368 | NRP | 26.0 | 26.2 | 251.0 | 6.9e-67 |
| QBQ12463.1 | amino\_acid\_adenylation\_domain-containing\_protein | BGC0002693 | NRP | 27.0 | 26.1 | 255.0 | 7.55e-67 |
| CBA63680.1 | nonribosomal\_peptide\_synthetase\_NRPS | BGC0000368 | NRP | 27.0 | 26.0 | 254.0 | 9.38e-67 |
| AAZ03552.1 | McnC | BGC0000332 | NRP | 27.0 | 25.9 | 254.0 | 1.04e-66 |
| KPN93065.1 | NunD | BGC0001416 | NRP | 27.0 | 25.8 | 254.0 | 1.06e-66 |
| AHH53506.1 | non-ribosomal\_peptide\_synthetase | BGC0000439 | NRP:Lipopeptide:Ca+-dependent lipopeptide | 28.0 | 26.3 | 254.0 | 1.44e-66 |
| QWT72292.1 | putative\_non-ribosomal\_peptide\_synthetase | BGC0002430 | NRP+Saccharide | 27.0 | 26.2 | 254.0 | 1.45e-66 |
| CAE02631.1 | surfactin\_synthetase\_B\_ | BGC0000433 | NRP:Lipopeptide | 25.0 | 25.8 | 254.0 | 1.59e-66 |
| AEA30274.1 | peptide\_synthetase | BGC0000429 | Polyketide+NRP:Cyclic depsipeptide | 26.0 | 26.1 | 254.0 | 1.65e-66 |
| QCX41945.1 | Amc8 | BGC0001957 | Polyketide | 30.0 | 20.4 | 248.0 | 1.66e-66 |
| QHW08554.1 | polyketide\_synthase | BGC0002054 | Polyketide+NRP+Saccharide | 30.0 | 20.4 | 248.0 | 1.66e-66 |
| ACS20362.1 | amino\_acid\_adenylation\_domain\_protein | BGC0002420 | NRP+Polyketide | 27.0 | 25.8 | 253.0 | 1.75e-66 |
| ctg4\_5 |  | BGC0002017 | NRP | 29.0 | 24.3 | 253.0 | 1.81e-66 |
| AAF86395.1 | FkbP | BGC0000994 | NRP+Polyketide | 26.0 | 25.8 | 252.0 | 1.88e-66 |
| AUD11993.1 | OrbJ | BGC0001721 | NRP | 28.0 | 25.8 | 252.0 | 2.68e-66 |
| DAB41477.1 | nonribosomal\_peptide\_synthetase | BGC0001230 | NRP:Cyclic depsipeptide+Polyketide:Modular type I polyketide | 28.0 | 26.5 | 253.0 | 2.87e-66 |
| ABE35421.1 | Non-ribosomal\_peptide\_synthetase | BGC0002421 | NRP | 27.0 | 26.3 | 251.0 | 3.47e-66 |
| AJD47485.1 | PpsD | BGC0002418 | NRP+Polyketide | 27.0 | 24.9 | 252.0 | 3.75e-66 |
| QUS58937.1 | non-ribosomal\_peptide\_synthetase | BGC0002123 | NRP+Polyketide | 26.0 | 25.7 | 250.0 | 3.9e-66 |
| ABM34278.1 | amino\_acid\_adenylation\_domain\_protein | BGC0002419 | NRP+Polyketide | 27.0 | 26.7 | 248.0 | 4.15e-66 |
| ctg1\_orf1264 |  | BGC0001752 | NRP | 27.0 | 27.0 | 252.0 | 5.02e-66 |
| UKO95748.1 | amino\_acid\_adenylation\_domain-containing\_protein | BGC0002632 | NRP | 25.0 | 26.1 | 252.0 | 5.3e-66 |
| CAL17540.1 | peptide\_synthetase,\_putative | BGC0002465 | NRP | 26.0 | 26.0 | 251.0 | 5.45e-66 |
| CAR51995.1 | ornibactin\_biosynthesis\_non-ribosomal\_peptide\_synthase | BGC0002569 | NRP | 27.0 | 25.8 | 251.0 | 5.92e-66 |
| ATP76244.1 | NdaB | BGC0001705 | NRP+Polyketide | 25.0 | 25.6 | 249.0 | 6.18e-66 |
| CAD29799.1 | microcystin\_synthetase | BGC0001015 | NRP+Polyketide | 26.0 | 25.4 | 249.0 | 6.6e-66 |
| AXG49819.1 | hybrid\_non-ribosomal\_peptide\_synthetase/type\_I\_polyketide\_synthase | BGC0000383 | NRP+Polyketide:Modular type I polyketide | 26.0 | 26.0 | 251.0 | 8.47e-66 |
| AGI87384.1 | Peptide\_synthase | BGC0002358 | Polyketide | 26.0 | 26.1 | 251.0 | 8.48e-66 |
| ADH04679.1 | non-ribosomal\_peptide\_synthetase | BGC0001344 | NRP+Polyketide | 27.0 | 21.6 | 251.0 | 8.52e-66 |
| ABI26079.1 | OciC | BGC0000331 | NRP | 25.0 | 26.0 | 249.0 | 1.04e-65 |
| QBA57737.1 | NRPS | BGC0002377 | NRP | 27.0 | 26.3 | 251.0 | 1.22e-65 |
| QED88055.1 | nonribosomal\_peptide\_synthetase | BGC0001967 | NRP+Polyketide | 27.0 | 26.5 | 250.0 | 1.34e-65 |
| AAU39359.1 | lichenysin\_synthase\_LchAA | BGC0000381 | NRP | 24.0 | 24.0 | 251.0 | 1.36e-65 |
| WP\_011146892.1 | non-ribosomal\_peptide\_synthetase | BGC0001641 | NRP | 26.0 | 26.3 | 251.0 | 1.44e-65 |
| ANI24100.1 | nonribosomal\_peptide\_synthetase | BGC0001235 | NRP+Polyketide | 27.0 | 25.9 | 251.0 | 1.5e-65 |
| QPB41097.1 | non-ribosomal\_peptide\_synthetase | BGC0002503 | NRP+Polyketide | 29.0 | 24.4 | 251.0 | 1.5e-65 |
| AEZ51516.1 | pmxA | BGC0001153 | NRP:Lipopeptide | 25.0 | 26.0 | 251.0 | 1.54e-65 |
| AEH41794.1 | HrmP | BGC0000374 | NRP:Cyclic depsipeptide | 28.0 | 25.4 | 250.0 | 1.75e-65 |
| EPH46598.1 | putative\_Dimodular\_nonribosomal\_peptide\_synthase | BGC0001519 | NRP+Polyketide | 26.0 | 25.9 | 248.0 | 1.79e-65 |
| QTT72106.1 | amino\_acid\_adenylation\_domain-containing\_protein | BGC0002350 | NRP+Polyketide+Saccharide | 27.0 | 26.2 | 250.0 | 2.02e-65 |
| NKI69296.1 | amino\_acid\_adenylation\_domain-containing\_protein | BGC0002408 | NRP | 26.0 | 26.2 | 249.0 | 2.37e-65 |
| UEF20591.1 | nonribosomal\_peptide\_synthetase | BGC0002360 | NRP | 28.0 | 23.0 | 248.0 | 2.56e-65 |
| CAE15637.1 |  | BGC0001128 | NRP | 27.0 | 24.1 | 250.0 | 2.68e-65 |
| AXG48275.1 | non-ribosomal\_peptide\_synthetase | BGC0002716 | NRP | 27.0 | 24.1 | 250.0 | 2.68e-65 |
| QEO75073.1 | condensation\_domain-containing\_protein | BGC0002079 | NRP:Cyclic depsipeptide | 28.0 | 24.1 | 249.0 | 2.87e-65 |
| AHH53507.1 | non-ribosomal\_peptide\_synthetase | BGC0000439 | NRP:Lipopeptide:Ca+-dependent lipopeptide | 27.0 | 26.8 | 250.0 | 2.9e-65 |
| QEO74982.1 | omn7 | BGC0002078 | NRP:Cyclic depsipeptide | 27.0 | 26.0 | 249.0 | 3.71e-65 |
| QIE08736.1 | non-ribosomal\_peptide\_synthetase | BGC0002544 | NRP | 27.0 | 26.0 | 249.0 | 4.09e-65 |
| CCM44336.1 | Nonribosomal\_peptide\_synthetase | BGC0001056 | NRP+Polyketide:Modular type I polyketide+Polyketide:PUFA synthase or related polyketide | 28.0 | 22.8 | 249.0 | 4.24e-65 |
| CAD55498.1 | CDA\_peptide\_synthetase\_III\_(CdaPs3) | BGC0000315 | NRP:Lipopeptide:Ca+-dependent lipopeptide | 29.0 | 27.0 | 248.0 | 5.21e-65 |
| CAB38517.1 | CDA\_peptide\_synthetase\_II\_(CdaPs2) | BGC0000315 | NRP:Lipopeptide:Ca+-dependent lipopeptide | 27.0 | 27.9 | 249.0 | 5.26e-65 |
| EFL06867.1 | predicted\_protein | BGC0000300 | NRP | 26.0 | 25.7 | 248.0 | 6.59e-65 |
| UHJ79948.1 | non-ribosomal\_peptide\_synthetase | BGC0002654 | NRP | 27.0 | 26.1 | 248.0 | 7.03e-65 |
| ABV79988.1 | ApnD | BGC0000301 | NRP | 24.0 | 25.8 | 246.0 | 7.13e-65 |
| ACA97576.1 | PmxA | BGC0000408 | NRP | 25.0 | 26.7 | 248.0 | 7.71e-65 |
| AJM89735.1 | PmxA | BGC0001192 | NRP | 25.0 | 26.5 | 248.0 | 7.73e-65 |
| EWM63005.1 | linear\_gramicidin\_synthetase\_LgrC | BGC0001328 | NRP:Cyclic depsipeptide+Polyketide:Modular type I polyketide | 27.0 | 22.7 | 243.0 | 8.35e-65 |
| AAF63833.1 | PstD | BGC0000362 | NRP | 28.0 | 25.2 | 246.0 | 8.84e-65 |
| APZ78729.1 | nonribosomal\_peptide\_synthetase | BGC0001421 | NRP:Cyclic depsipeptide+Polyketide:Iterative type I polyketide | 26.0 | 26.0 | 248.0 | 1.36e-64 |
| BAX89999.1 | Non-ribosomal\_peptide\_synthetase | BGC0001628 | NRP | 28.0 | 26.2 | 247.0 | 1.6e-64 |
| QBC75023.1 | non-ribosomal\_peptide\_synthetase | BGC0001968 | NRP | 26.0 | 22.8 | 247.0 | 1.71e-64 |
| APO47826.1 | hypothetical\_protein | BGC0002653 | NRP | 25.0 | 26.8 | 247.0 | 1.73e-64 |
| ACG60773.1 | NRPS(C/A/PCP) | BGC0001058 | NRP:Glycopeptide+Polyketide:Modular type I polyketide+Saccharide:Hybrid/tailoring saccharide | 28.0 | 26.4 | 243.0 | 1.79e-64 |
| ACZ55945.1 | non-ribosomal\_peptide\_synthetase | BGC0000302 | NRP | 25.0 | 26.6 | 246.0 | 1.81e-64 |
| AAY93356.2 | non-ribosomal\_peptide\_synthetase\_PvdI | BGC0000413 | NRP | 26.0 | 25.5 | 247.0 | 2.02e-64 |
| CAJ77696.1 | MPS2\_protein | BGC0000363 | NRP | 26.0 | 30.5 | 246.0 | 2.11e-64 |
| WP\_013428324.1 | non-ribosomal\_peptide\_synthetase | BGC0001758 | NRP | 27.0 | 26.0 | 247.0 | 2.5e-64 |
| ABW00331.1 | amino\_acid\_adenylation\_domain | BGC0000333 | NRP | 28.0 | 23.9 | 247.0 | 2.5e-64 |
| ABI26077.1 | OciA | BGC0000331 | NRP | 25.0 | 26.4 | 246.0 | 2.56e-64 |
| QWM97319.1 | non-ribosomal\_peptide\_synthetase | BGC0002384 | NRP | 27.0 | 25.9 | 247.0 | 2.57e-64 |
| UMM61371.1 | Tsk10 | BGC0002661 | NRP | 28.0 | 26.2 | 246.0 | 2.6e-64 |
| QXJ21807.1 | amino\_acid\_adenylation\_domain-containing\_protein | BGC0002370 | NRP | 25.0 | 26.0 | 246.0 | 3.32e-64 |
| AAY91420.2 | non-ribosomal\_peptide\_synthetase\_OfaB | BGC0000399 | NRP:Cyclic depsipeptide | 28.0 | 25.3 | 246.0 | 3.69e-64 |
| AJW76709.1 | DsaG | BGC0001196 | NRP | 26.0 | 26.5 | 246.0 | 3.69e-64 |
| CAJ76290.1 | putative\_non-ribosomal\_peptide\_synthase | BGC0000972 | NRP+Polyketide:Modular type I polyketide+Polyketide:Trans-AT type I polyketide | 27.0 | 26.3 | 245.0 | 4.01e-64 |
| CEK23364.1 | putative\_Phenylalanine\_racemase\_(ATP-hydrolyzing) | BGC0001716 | NRP | 26.0 | 26.0 | 246.0 | 4.48e-64 |
| QCQ67881.1 | non-ribosomal\_peptide\_synthetase | BGC0002297 | NRP+Polyketide | 24.0 | 25.3 | 243.0 | 5.51e-64 |
| ALV82388.1 | CDA\_peptide\_synthetase\_III | BGC0001370 | NRP | 28.0 | 26.5 | 245.0 | 5.84e-64 |
| AID65224.1 | nonribosomal\_peptide\_synthetase | BGC0000335 | NRP+Polyketide | 26.0 | 25.6 | 245.0 | 6.14e-64 |
| QBA57736.1 | NRPS | BGC0002377 | NRP | 27.0 | 26.1 | 245.0 | 6.21e-64 |
| AAZ03550.1 | McnA | BGC0000332 | NRP | 24.0 | 26.0 | 241.0 | 6.98e-64 |
| AMK48226.1 | nonribosomal\_peptide\_synthetase | BGC0001351 | NRP | 27.0 | 25.9 | 244.0 | 8.23e-64 |
| AEP18655.1 | WAPS2 | BGC0000461 | NRP | 27.0 | 26.3 | 245.0 | 9.47e-64 |
| QED55421.1 | nonribosomal\_peptide\_synthetase | BGC0001984 | NRP | 27.0 | 26.2 | 244.0 | 1.03e-63 |
| AHB82071.1 | non\_ribosomal\_peptide\_synthetase | BGC0001231 | NRP+Polyketide:Modular type I polyketide | 26.0 | 21.9 | 244.0 | 1.05e-63 |
| CAY48788.1 | putative\_non-ribosomal\_peptide\_synthetase | BGC0001312 | NRP | 26.0 | 24.9 | 244.0 | 1.07e-63 |
| DAC80524.1 | peptide\_synthetase | BGC0001841 | NRP+Polyketide | 25.0 | 26.1 | 242.0 | 1.08e-63 |
| RSO11553.1 | non-ribosomal\_peptide\_synthetase | BGC0002637 | NRP | 27.0 | 26.3 | 244.0 | 1.19e-63 |
| ARF06222.1 | non-ribosomal\_peptide\_synthetase | BGC0001593 | NRP | 27.0 | 26.3 | 242.0 | 1.31e-63 |
| BAO84861.1 | putative\_non-ribosomal\_peptide\_synthetase | BGC0000414 | NRP | 25.0 | 26.4 | 240.0 | 1.44e-63 |
| WP\_019032753.1 | non-ribosomal\_peptide\_synthetase | BGC0001331 | NRP:Cyclic depsipeptide+Polyketide:Modular type I polyketide | 28.0 | 22.3 | 243.0 | 1.69e-63 |
| DAC80541.1 | NRPS/PKS | BGC0001840 | NRP+Polyketide | 26.0 | 26.1 | 242.0 | 1.7e-63 |
| QWT72293.1 | non-ribosomal\_peptide\_synthetase | BGC0002430 | NRP+Saccharide | 26.0 | 26.0 | 243.0 | 1.72e-63 |
| BAH22765.1 | nonribosomal\_peptide\_synthetase | BGC0001018 | NRP | 25.0 | 26.2 | 242.0 | 1.74e-63 |
| QUJ09166.1 | Lon19 | BGC0002440 | NRP | 27.0 | 25.4 | 243.0 | 2e-63 |
| ABD65958.1 | nonribosomal\_peptide\_synthetase | BGC0000341 | NRP | 27.0 | 26.2 | 244.0 | 2.18e-63 |
| ABY83142.1 | Azi3 | BGC0000960 | NRP+Polyketide | 27.0 | 25.8 | 239.0 | 2.25e-63 |
| CAA60461.1 | pipecolate\_incorporating\_enzyme | BGC0001040 | NRP+Polyketide | 26.0 | 25.2 | 242.0 | 2.5e-63 |
| APO47822.1 | non-ribosomal\_peptide\_synthetase | BGC0002653 | NRP | 24.0 | 26.2 | 243.0 | 2.68e-63 |
| MCF2150416.1 | Non-ribosomal\_peptide\_synthetase | BGC0002625 | NRP+Polyketide | 25.0 | 26.7 | 243.0 | 2.68e-63 |
| ACM68684.1 | AerB | BGC0000298 | NRP | 25.0 | 25.9 | 242.0 | 2.73e-63 |
| CCJ67640.1 | TaaE | BGC0000447 | NRP:Lipopeptide | 27.0 | 26.4 | 243.0 | 2.74e-63 |
| AJW65406.1 | nonribosomal\_peptide\_synthetase | BGC0001195 | NRP+Polyketide | 26.0 | 26.3 | 241.0 | 3.3e-63 |
| ABD65957.1 | nonribosomal\_peptide\_synthetase | BGC0000341 | NRP | 27.0 | 26.1 | 243.0 | 3.57e-63 |
| ctg1\_orf1265 |  | BGC0001752 | NRP | 27.0 | 25.8 | 243.0 | 3.62e-63 |
| QYA95680.1 | amino\_acid\_adenylation\_domain-containing\_protein | BGC0002676 | NRP | 26.0 | 25.8 | 243.0 | 3.95e-63 |
| CAQ34921.1 | nonribosomal\_peptide\_synthetase | BGC0000986 | NRP+Polyketide | 26.0 | 26.1 | 241.0 | 4.61e-63 |
| UHJ79953.1 | non-ribosomal\_peptide\_synthetase | BGC0002654 | NRP | 26.0 | 25.7 | 242.0 | 4.66e-63 |
| CAJ21198.2 | non-ribosomal\_peptide\_synthetase | BGC0000297 | NRP:Glycopeptide+Polyketide:Other polyketide+Saccharide:Hybrid/tailoring saccharide | 25.0 | 26.0 | 241.0 | 6.21e-63 |
| QDQ83033.1 | amino\_acid\_adenylation\_domain-containing\_protein | BGC0002564 | NRP | 26.0 | 26.3 | 241.0 | 6.3e-63 |
| ABD14711.1 | cesA | BGC0000320 | NRP:Cyclic depsipeptide | 24.0 | 25.8 | 242.0 | 6.34e-63 |
| AGE11899.1 | nonribosomal\_peptide\_synthetase | BGC0000366 | NRP | 27.0 | 26.6 | 241.0 | 6.44e-63 |
| ATY37589.1 | BogB | BGC0001532 | NRP | 24.0 | 25.8 | 241.0 | 6.87e-63 |
| AJK49757.1 | non-ribosomal\_peptide\_synthase | BGC0002565 | NRP | 28.0 | 25.4 | 241.0 | 7.16e-63 |
| PHM49485.1 | Amino\_acid\_adenylation | BGC0001131 | NRP | 27.0 | 25.2 | 242.0 | 7.91e-63 |
| CDG17986.1 | Non-ribosomal\_peptide\_synthetase | BGC0000464 | NRP:Cyclic depsipeptide | 26.0 | 25.7 | 241.0 | 8.04e-63 |
| CAJ77716.1 | Mps2\_protein | BGC0000364 | NRP | 25.0 | 30.5 | 241.0 | 9.13e-63 |
| CAD29798.1 | peptide\_synthetase | BGC0001015 | NRP+Polyketide | 26.0 | 26.5 | 241.0 | 1e-62 |
| AKJ29412.1 | peptide\_synthetase | BGC0001608 | NRP | 29.0 | 20.7 | 241.0 | 1.01e-62 |
| AAL33758.1 | putative\_non-ribosomal\_peptide\_synthetase | BGC0000421 | NRP | 27.0 | 26.7 | 239.0 | 1.02e-62 |
| ABL74937.1 | NRPS | BGC0001048 | NRP:Glycopeptide+Polyketide:Modular type I polyketide+Saccharide:Hybrid/tailoring saccharide | 27.0 | 26.4 | 237.0 | 1.09e-62 |
| CCP45168.1 | Peptide\_synthetase\_MbtE\_(peptide\_synthase) | BGC0001021 | NRP+Polyketide | 26.0 | 25.7 | 240.0 | 1.2e-62 |
| ACZ55946.1 | non-ribosomal\_peptide\_synthetase | BGC0000302 | NRP | 25.0 | 25.9 | 239.0 | 1.24e-62 |
| CDG17981.1 | Non-ribosomal\_peptide\_synthetase | BGC0000464 | NRP:Cyclic depsipeptide | 26.0 | 24.9 | 240.0 | 1.38e-62 |
| BAW27693.1 | NRPS(C-A-T-TE) | BGC0001764 | NRP | 26.0 | 25.8 | 239.0 | 1.4e-62 |
| WP\_051700111.1 | non-ribosomal\_peptide\_synthetase | BGC0001368 | NRP | 26.0 | 25.8 | 239.0 | 1.46e-62 |
| DAC80528.1 | peptide\_synthetase | BGC0001878 | NRP+Polyketide | 26.0 | 25.0 | 238.0 | 1.46e-62 |
| ANS62967.1 | non-ribosomal\_peptide\_synthase/amino\_acid\_adenylation\_enzyme | BGC0001567 | NRP | 27.0 | 26.4 | 240.0 | 1.56e-62 |
| EFL06865.1 | hypothetical\_protein | BGC0000300 | NRP | 27.0 | 23.3 | 240.0 | 1.9e-62 |
| AXF14775.1 | non-ribosomal\_peptide\_synthetase | BGC0002563 | NRP | 27.0 | 25.9 | 239.0 | 1.95e-62 |
| KUM80513.1 | hypothetical\_protein | BGC0001562 | NRP | 27.0 | 26.6 | 240.0 | 2.07e-62 |
| ADZ24999.1 | non-ribosomal\_peptide\_synthase | BGC0000380 | NRP+Polyketide:Modular type I polyketide | 26.0 | 26.1 | 238.0 | 2.23e-62 |
| AWI62628.1 | nonribosomal\_peptide\_synthetase | BGC0001822 | NRP | 26.0 | 27.0 | 240.0 | 2.4e-62 |
| KON97028.1 | phenylalanine\_racemase | BGC0002122 | NRP | 23.0 | 25.4 | 240.0 | 2.71e-62 |
| AOZ21320.1 | SulM | BGC0001790 | NRP | 25.0 | 26.0 | 239.0 | 2.96e-62 |
| AGC09528.1 | NRPS | BGC0001183 | Polyketide | 27.0 | 23.7 | 239.0 | 3.06e-62 |
| AED90003.1 | non-ribosomal\_peptide\_synthetase\_ThaB | BGC0000443 | NRP:Beta-lactam | 26.0 | 26.2 | 239.0 | 3.57e-62 |
| AHD05618.1 | putative\_non-ribosomal\_peptide\_ligase\_domain\_protein | BGC0001033 | NRP+Polyketide | 25.0 | 25.9 | 236.0 | 3.67e-62 |
| QYA95662.1 | amino\_acid\_adenylation\_domain-containing\_protein | BGC0002676 | NRP | 26.0 | 24.3 | 236.0 | 3.96e-62 |
| AAY93355.1 | non-ribosomal\_peptide\_synthetase\_PvdJ | BGC0000413 | NRP | 25.0 | 25.5 | 238.0 | 5.18e-62 |
| QUS58938.1 | amino\_acid\_adenylation\_domain-containing\_protein | BGC0002123 | NRP+Polyketide | 26.0 | 25.9 | 239.0 | 5.49e-62 |
| WP\_052165466.1 | non-ribosomal\_peptide\_synthetase | BGC0001327 | NRP:Cyclic depsipeptide+Polyketide:Modular type I polyketide | 27.0 | 22.7 | 237.0 | 6.29e-62 |
| AAZ03554.1 | McnE | BGC0000332 | NRP | 25.0 | 26.4 | 237.0 | 6.68e-62 |
| AHB82069.1 | non\_ribosomal\_peptide\_synthetase | BGC0001231 | NRP+Polyketide:Modular type I polyketide | 27.0 | 25.6 | 238.0 | 6.77e-62 |
| QCP68976.1 | VatQ | BGC0002296 | NRP+Polyketide | 25.0 | 26.0 | 237.0 | 6.99e-62 |
| QNH67550.1 | Cip22 | BGC0002108 | NRP | 27.0 | 26.3 | 238.0 | 8.95e-62 |
| AJK49765.1 | non-ribosomal\_peptide\_synthase | BGC0002565 | NRP | 27.0 | 26.0 | 237.0 | 9.83e-62 |
| AIG26883.1 | NRPS\_domain-containing\_protein | BGC0002432 | NRP | 24.0 | 25.5 | 238.0 | 1.02e-61 |
| ARU08074.1 | mlcL | BGC0001448 | NRP:Lipopeptide:Ca+-dependent lipopeptide | 27.0 | 26.3 | 238.0 | 1.11e-61 |
| QCE43603.1 | nonribosomal\_peptide\_synthetase\_(NRPS),\_subunit\_2 | BGC0001834 | NRP | 26.0 | 25.3 | 238.0 | 1.12e-61 |
| APZ78845.1 | nonribosomal\_peptide\_synthetase | BGC0001431 | NRP:Cyclic depsipeptide+Polyketide:Iterative type I polyketide | 27.0 | 23.0 | 238.0 | 1.15e-61 |
| QEO74983.1 | omn8 | BGC0002078 | NRP:Cyclic depsipeptide | 27.0 | 26.2 | 237.0 | 1.22e-61 |
| QXJ21808.1 | amino\_acid\_adenylation\_domain-containing\_protein | BGC0002370 | NRP | 27.0 | 25.1 | 238.0 | 1.24e-61 |
| BAW32324.1 | nonribosomal\_peptide\_synthetase | BGC0001630 | NRP+Polyketide | 26.0 | 26.2 | 238.0 | 1.25e-61 |
| WP\_078586793.1 | non-ribosomal\_peptide\_synthetase | BGC0001760 | NRP | 26.0 | 25.8 | 236.0 | 1.43e-61 |
| QMS47798.1 | JesB | BGC0001629 | NRP:Lipopeptide | 28.0 | 25.4 | 238.0 | 1.44e-61 |
| KPN90369.1 | NunE | BGC0001416 | NRP | 26.0 | 26.7 | 237.0 | 1.78e-61 |
| ADG27358.1 | peptide\_synthetase | BGC0000296 | NRP | 27.0 | 26.4 | 236.0 | 2.32e-61 |
| CZT62784.1 | Non-ribosomal\_peptide\_synthase,\_involved\_in\_Hassallidin\_biosynthesis | BGC0001614 | NRP | 26.0 | 26.3 | 236.0 | 2.82e-61 |
| CAJ96470.1 | non-ribosomal\_peptide\_synthetase | BGC0000330 | NRP:NRP siderophore | 26.0 | 25.9 | 236.0 | 3.09e-61 |
| CDG17985.1 | Putative\_Ornithine\_racemase\_(fragment) | BGC0000464 | NRP:Cyclic depsipeptide | 26.0 | 26.3 | 236.0 | 3.43e-61 |
| AZM58102.1 | non-ribosomal\_peptide\_synthetase | BGC0002314 | NRP | 26.0 | 25.7 | 236.0 | 3.44e-61 |
| WP\_004571779.1 | non-ribosomal\_peptide\_synthetase | BGC0001760 | NRP | 27.0 | 23.7 | 231.0 | 3.85e-61 |
| QUS58939.1 | amino\_acid\_adenylation\_domain-containing\_protein | BGC0002123 | NRP+Polyketide | 26.0 | 25.3 | 236.0 | 4.14e-61 |
| BAD55611.1 | putative\_non-ribosomal\_peptide\_synthetase | BGC0001027 | NRP+Polyketide | 27.0 | 25.8 | 235.0 | 4.14e-61 |
| ABS74206.1 | fengycin\_synthetase\_D | BGC0001095 | NRP | 25.0 | 25.9 | 236.0 | 4.77e-61 |
| ADG27359.1 | peptide\_synthetase | BGC0000296 | NRP | 28.0 | 23.1 | 236.0 | 5.1e-61 |
| AAO72424.1 | syringopeptin\_synthetase\_B | BGC0000438 | NRP | 27.0 | 25.0 | 236.0 | 5.5e-61 |
| AEG64698.1 | LpmD | BGC0000379 | NRP | 28.0 | 25.5 | 235.0 | 6.38e-61 |
| AAY37654.1 | Amino\_acid\_adenylation | BGC0000437 | NRP | 27.0 | 25.0 | 235.0 | 7.2e-61 |
| QMN69933.1 | PsoB | BGC0002521 | NRP | 26.0 | 26.1 | 235.0 | 7.68e-61 |
| WA1\_15570 | hypothetical\_protein | BGC0002484 | NRP+Polyketide | 26.0 | 22.7 | 234.0 | 1.05e-60 |
| AGU50949.1 | putative\_non-ribosomal\_peptide\_synthetase | BGC0002417 | NRP+Polyketide | 26.0 | 25.8 | 233.0 | 1.16e-60 |
| EPH46596.1 | putative\_Linear\_gramicidin\_synthase\_subunit\_C | BGC0001519 | NRP+Polyketide | 26.0 | 26.1 | 234.0 | 1.24e-60 |
| AEW31022.1 | plipastatin\_synthetase | BGC0000407 | NRP | 24.0 | 25.8 | 234.0 | 1.4e-60 |
| AXA91302.1 | non-ribosomal\_peptide\_synthetase | BGC0002044 | NRP | 27.0 | 25.4 | 234.0 | 1.6e-60 |
| BAH43870.1 | putative\_linear\_pentadecapeptide\_gramicidin\_synthetase\_LgrB | BGC0000367 | NRP | 25.0 | 26.3 | 234.0 | 1.73e-60 |
| ASX95241.1 | IlaS | BGC0001620 | NRP+Polyketide | 27.0 | 26.0 | 234.0 | 1.74e-60 |
| CAY48789.1 | putative\_non-ribosomal\_peptide\_synthetase | BGC0001312 | NRP | 27.0 | 25.1 | 234.0 | 1.86e-60 |
| WP\_006051170.1 | non-ribosomal\_peptide\_synthetase | BGC0001999 | NRP | 27.0 | 25.9 | 234.0 | 2.11e-60 |
| KPN93063.1 | NupA | BGC0001416 | NRP | 28.0 | 26.0 | 234.0 | 2.33e-60 |
| AAY91421.3 | non-ribosomal\_peptide\_synthetase\_OfaC | BGC0000399 | NRP:Cyclic depsipeptide | 25.0 | 25.7 | 233.0 | 2.67e-60 |
| AXN93575.1 | PuwA | BGC0001950 | NRP | 26.0 | 22.9 | 233.0 | 2.78e-60 |
| AXN93584.1 | PuwA | BGC0001951 | NRP | 26.0 | 22.9 | 233.0 | 2.78e-60 |
| AYJ71720.1 | non-ribosomal\_peptide\_synthetase | BGC0001942 | NRP+Polyketide | 26.0 | 25.0 | 233.0 | 2.87e-60 |
| CAG29031.1 | nonribosomal\_peptide\_synthetase\_(modules\_1\_and\_2) | BGC0001023 | NRP+Polyketide:Modular type I polyketide | 27.0 | 22.8 | 233.0 | 2.88e-60 |
| APZ78821.1 | nonribosomal\_peptide\_synthetase | BGC0001429 | NRP:Cyclic depsipeptide+Polyketide:Iterative type I polyketide | 27.0 | 22.8 | 233.0 | 2.88e-60 |
| AKC91849.1 | nonribosomal\_peptide\_synthetase | BGC0001414 | NRP | 28.0 | 22.2 | 233.0 | 2.95e-60 |
| CAL17541.1 | peptide\_synthetase,\_putative | BGC0002465 | NRP | 26.0 | 23.3 | 233.0 | 3.13e-60 |
| AIW82277.1 | PuwA | BGC0001125 | NRP+Polyketide | 26.0 | 23.0 | 233.0 | 3.64e-60 |
| QRG35013.1 | NRPS | BGC0002378 | NRP | 27.0 | 26.1 | 233.0 | 3.79e-60 |
| AEG64696.1 | LpmB | BGC0000379 | NRP | 26.0 | 26.4 | 233.0 | 3.86e-60 |
| BBA20967.1 | nonribosomal\_peptide\_synthetase | BGC0001763 | NRP+Polyketide | 27.0 | 26.0 | 233.0 | 3.88e-60 |
| KJY94239.1 | thioester\_reductase | BGC0002691 | NRP | 25.0 | 25.9 | 232.0 | 3.98e-60 |
| AAN85501.1 | nonribosomal\_peptide\_synthetase | BGC0001101 | NRP+Polyketide:Modular type I polyketide+Polyketide:Trans-AT type I polyketide | 27.0 | 26.0 | 229.0 | 4.15e-60 |
| ABA73956.1 | putative\_non-ribosomal\_peptide\_synthetase | BGC0001842 | NRP:Lipopeptide | 26.0 | 25.6 | 233.0 | 4.56e-60 |
| CDG17987.1 | Putative\_Ornithine\_racemase\_(fragment) | BGC0000464 | NRP:Cyclic depsipeptide | 26.0 | 26.4 | 233.0 | 4.58e-60 |
| AGI89788.1 | Nonribosomal\_peptide\_synthetase | BGC0001792 | NRP | 28.0 | 24.9 | 232.0 | 5.64e-60 |
| DAB41484.1 | nonribosomal\_peptide\_synthetase/polyketide\_synthase\_type\_I | BGC0001230 | NRP:Cyclic depsipeptide+Polyketide:Modular type I polyketide | 25.0 | 38.5 | 232.0 | 5.75e-60 |
| AHH53508.1 | non-ribosomal\_peptide\_synthetase | BGC0000439 | NRP:Lipopeptide:Ca+-dependent lipopeptide | 27.0 | 25.5 | 231.0 | 7.13e-60 |
| AFJ23825.1 | WLIP\_synthetase\_B | BGC0001838 | NRP | 28.0 | 20.6 | 232.0 | 7.49e-60 |
| ABS74180.1 | bacillomycin\_D\_synthetase\_B | BGC0001090 | Polyketide+NRP:Lipopeptide | 25.0 | 25.0 | 232.0 | 7.99e-60 |
| WP\_012408785.1 | non-ribosomal\_peptide\_synthetase | BGC0002061 | NRP:Cyclic depsipeptide+Polyketide:Modular type I polyketide | 24.0 | 26.9 | 231.0 | 9.43e-60 |
| CBJ90082.1 | Non\_Ribosomal\_peptide\_synthetase\_(-succinylbenzoate--CoA\_ligase) | BGC0001132 | NRP | 26.0 | 26.1 | 231.0 | 9.66e-60 |
| AEC14346.1 | nonribosomal\_peptide\_synthetase | BGC0000377 | NRP | 24.0 | 25.6 | 231.0 | 9.99e-60 |
| ABC37099.1 | non-ribosomal\_peptide\_synthetase,\_putative | BGC0000386 | NRP:NRP siderophore | 26.0 | 27.3 | 230.0 | 1.14e-59 |
| AHF21228.1 | TriD | BGC0000449 | NRP | 26.0 | 25.7 | 231.0 | 1.21e-59 |
| QLY89264.1 | pseudodesmin\_synthetase | BGC0002522 | NRP | 27.0 | 25.8 | 231.0 | 1.23e-59 |
| RAT94091.1 | NRPS | BGC0001469 | NRP | 24.0 | 26.4 | 227.0 | 1.76e-59 |
| ARU08073.1 | mlcK | BGC0001448 | NRP:Lipopeptide:Ca+-dependent lipopeptide | 26.0 | 26.1 | 230.0 | 1.89e-59 |
| CDG17980.1 | Putative\_Ornithine\_racemase\_(fragment) | BGC0000464 | NRP:Cyclic depsipeptide | 27.0 | 25.4 | 231.0 | 1.92e-59 |
| QNL34617.1 | SteB | BGC0002092 | NRP:Cyclic depsipeptide | 27.0 | 25.5 | 230.0 | 2.19e-59 |
| ALV86866.1 | Tlo20 | BGC0001406 | NRP | 27.0 | 26.4 | 230.0 | 2.26e-59 |
| BAY02138.1 | peptide\_synthetase | BGC0002532 | NRP+Polyketide | 26.0 | 25.4 | 226.0 | 2.59e-59 |
| QUF98525.1 | non-ribosomal\_peptide\_synthetase | BGC0002582 | NRP | 29.0 | 22.7 | 226.0 | 2.66e-59 |
| AZH29360.1 | amino\_acid\_adenylation\_domain-containing\_protein | BGC0001843 | NRP | 27.0 | 25.2 | 230.0 | 2.7e-59 |
| BAX89998.1 | Non-ribosomal\_peptide\_synthetase | BGC0001628 | NRP | 27.0 | 25.0 | 229.0 | 2.9e-59 |
| BAP16697.1 | nonribosomal\_peptide\_synthetase | BGC0000376 | NRP | 28.0 | 24.7 | 228.0 | 2.96e-59 |
| MAA\_10033 | polyketide\_synthase,\_putative | BGC0000337 | NRP | 25.0 | 29.3 | 228.0 | 3.01e-59 |
| BCJ07532.1 | hypothetical\_protein | BGC0002379 | NRP | 26.0 | 25.6 | 230.0 | 3.12e-59 |
| CAF32362.1 | putative\_non-ribosomal\_peptide\_synthetase | BGC0000712 | Saccharide | 30.0 | 22.6 | 226.0 | 3.37e-59 |
| BAH22762.1 | nonribosomal\_peptide\_synthetase | BGC0001018 | NRP | 24.0 | 26.4 | 226.0 | 3.41e-59 |
| AAG06715.1 | probable\_non-ribosomal\_peptide\_synthetase | BGC0002037 | NRP | 27.0 | 23.8 | 229.0 | 3.54e-59 |
| BAX64247.1 | NRPS | BGC0001623 | NRP+Polyketide | 26.0 | 26.9 | 229.0 | 3.66e-59 |
| AKJ15829.1 | peptide\_synthetase | BGC0002735 | Polyketide+NRP | 26.0 | 23.4 | 227.0 | 4.01e-59 |
| QMS47800.1 | JesC | BGC0001629 | NRP:Lipopeptide | 30.0 | 20.8 | 229.0 | 4.47e-59 |
| AQX14499.1 | monobactam\_NRPS\_scaffold\_1 | BGC0001672 | NRP | 25.0 | 25.7 | 228.0 | 5.02e-59 |
| WP\_013310342.1 | non-ribosomal\_peptide\_synthetase | BGC0001728 | NRP+Polyketide | 24.0 | 25.2 | 229.0 | 5.43e-59 |
| AEG64697.1 | LpmC | BGC0000379 | NRP | 27.0 | 26.2 | 229.0 | 5.49e-59 |
| AFK57219.1 | DidH | BGC0000985 | Polyketide+NRP:Cyclic depsipeptide | 26.0 | 26.1 | 227.0 | 5.73e-59 |
| AGI89790.1 | ATP-dependent\_valine\_adenylase | BGC0001792 | NRP | 28.0 | 24.6 | 229.0 | 6.38e-59 |
| BAB69699.1 | iturin\_A\_synthetase\_B | BGC0001098 | NRP+Polyketide | 26.0 | 25.0 | 229.0 | 6.82e-59 |
| ABX37383.1 | amino\_acid\_adenylation\_domain\_protein | BGC0000984 | NRP+Polyketide | 26.0 | 25.3 | 228.0 | 7.56e-59 |
| QED55422.1 | nonribosomal\_peptide\_synthetase | BGC0001984 | NRP | 26.0 | 26.3 | 228.0 | 8.31e-59 |
| AEW31015.1 | plipastatin\_synthetase | BGC0000407 | NRP | 25.0 | 26.1 | 226.0 | 9.71e-59 |
| WP\_053065270.1 | non-ribosomal\_peptide\_synthetase | BGC0001330 | NRP:Cyclic depsipeptide+Polyketide:Modular type I polyketide | 28.0 | 22.4 | 227.0 | 1.08e-58 |
| AXA91301.1 | non-ribosomal\_peptide\_synthetase | BGC0002044 | NRP | 27.0 | 26.3 | 228.0 | 1.16e-58 |
| NAO96320.1 | amino\_acid\_adenylation\_domain-containing\_protein | BGC0002117 | NRP | 26.0 | 26.2 | 228.0 | 1.46e-58 |
| CBG75492.1 | putative\_NRPS/siderophore\_biosynthesis\_protein | BGC0000423 | NRP | 26.0 | 26.1 | 228.0 | 1.58e-58 |
| KPN90376.1 | NupC | BGC0001416 | NRP | 26.0 | 25.7 | 228.0 | 1.7e-58 |
| AAZ23078.1 | peptide\_synthetase | BGC0000291 | NRP | 27.0 | 26.3 | 227.0 | 1.8e-58 |
| BAH43871.1 | truncated\_linear\_pentadecapeptide\_gramicidin\_synthetase\_LgrC | BGC0000367 | NRP | 25.0 | 24.9 | 227.0 | 2.07e-58 |
| APU91751.1 | Non-Ribosomal\_Peptide\_Synthetase | BGC0001806 | NRP | 29.0 | 20.7 | 227.0 | 2.08e-58 |
| DAB41478.1 | nonribosomal\_peptide\_synthetase | BGC0001230 | NRP:Cyclic depsipeptide+Polyketide:Modular type I polyketide | 26.0 | 26.2 | 226.0 | 2.31e-58 |
| NPC94426.1 | amino\_acid\_adenylation\_domain-containing\_protein | BGC0002695 | NRP | 26.0 | 26.3 | 226.0 | 2.35e-58 |
| QPI18728.1 | nonribosomal\_peptide\_synthetase | BGC0002125 | NRP:Cyclic depsipeptide | 29.0 | 22.9 | 226.0 | 2.45e-58 |
| AXN93592.1 | PuwA | BGC0001952 | NRP | 25.0 | 22.9 | 226.0 | 2.67e-58 |
| AAF00961.1 | mcyB | BGC0001017 | NRP+Polyketide:Modular type I polyketide | 25.0 | 26.6 | 226.0 | 2.78e-58 |
| NKI69295.1 | amino\_acid\_adenylation\_domain-containing\_protein | BGC0002408 | NRP | 26.0 | 25.4 | 226.0 | 2.85e-58 |
| extra\_gene | NRPS/PKS | BGC0002095 | NRP | 26.0 | 24.6 | 226.0 | 3.13e-58 |
| ACS20358.1 | amino\_acid\_adenylation\_domain\_protein | BGC0002420 | NRP+Polyketide | 26.0 | 25.9 | 224.0 | 3.65e-58 |
| AZH29361.1 | amino\_acid\_adenylation\_domain-containing\_protein | BGC0001843 | NRP | 25.0 | 24.7 | 226.0 | 3.98e-58 |
| WP\_043882190.1 | non-ribosomal\_peptide\_synthetase | BGC0001728 | NRP+Polyketide | 25.0 | 26.0 | 226.0 | 4.71e-58 |
| FIS9431\_RS32925 | non-ribosomal\_peptide\_synthetase | BGC0001467 | NRP:Cyclic depsipeptide+Polyketide:Modular type I polyketide | 26.0 | 22.2 | 224.0 | 4.76e-58 |
| AIW58892.1 | non-ribosomal\_peptide\_synthetase | BGC0001582 | NRP | 27.0 | 25.7 | 226.0 | 6.75e-58 |
| DAC76736.1 | non-ribosomal\_peptide\_synthetase | BGC0001885 | NRP+Polyketide | 26.0 | 21.9 | 224.0 | 7.83e-58 |
| AAF00962.1 | mcyC | BGC0001017 | NRP+Polyketide:Modular type I polyketide | 25.0 | 26.0 | 223.0 | 9.1e-58 |
| QEO75077.1 | condensation\_domain-containing\_protein | BGC0002079 | NRP:Cyclic depsipeptide | 27.0 | 26.3 | 224.0 | 1.15e-57 |
| ABS75232.1 | DhbF | BGC0001185 | NRP:NRP siderophore | 25.0 | 26.5 | 224.0 | 1.18e-57 |
| CAJ14039.1 | peptide\_synthetase | BGC0000406 | NRP | 26.0 | 26.1 | 221.0 | 1.95e-57 |
| WP\_012988806.1 | non-ribosomal\_peptide\_synthetase | BGC0002135 | NRP:Lipopeptide | 25.0 | 26.1 | 224.0 | 2.01e-57 |
| AJW76711.1 | DsaI | BGC0001196 | NRP | 26.0 | 26.6 | 223.0 | 2.02e-57 |
| WP\_100939442.1 | non-ribosomal\_peptide\_synthetase | BGC0002071 | NRP:Lipopeptide | 27.0 | 24.9 | 223.0 | 3.16e-57 |
| AAK89731.2 | siderophore\_biosynthesis\_protein | BGC0002107 | NRP+Polyketide | 25.0 | 25.9 | 220.0 | 3.27e-57 |
| AIG26884.1 | NRPS\_domain-containing\_protein | BGC0002432 | NRP | 25.0 | 24.7 | 223.0 | 4.31e-57 |
| AEH41793.1 | HrmO | BGC0000374 | NRP:Cyclic depsipeptide | 26.0 | 26.0 | 223.0 | 4.94e-57 |
| APZ78703.1 | nonribosomal\_peptide\_synthetase | BGC0001419 | NRP:Cyclic depsipeptide+Polyketide:Iterative type I polyketide | 25.0 | 22.9 | 222.0 | 5.31e-57 |
| BAB69700.1 | iturin\_A\_synthetase\_C | BGC0001098 | NRP+Polyketide | 25.0 | 25.3 | 222.0 | 6.35e-57 |
| ABG94125.1 | non-ribosomal\_peptide\_synthetase | BGC0000417 | NRP | 26.0 | 25.7 | 221.0 | 7.72e-57 |
| WP\_068925909.1 | non-ribosomal\_peptide\_synthetase | BGC0002688 | NRP | 28.0 | 20.6 | 221.0 | 7.76e-57 |
| APZ78781.1 | nonribosomal\_peptide\_synthetase | BGC0001426 | NRP:Cyclic depsipeptide+Polyketide:Iterative type I polyketide | 26.0 | 22.8 | 221.0 | 9.08e-57 |
| MQQ32958.1 | amino\_acid\_adenylation\_domain-containing\_protein | BGC0002518 | NRP | 27.0 | 26.0 | 221.0 | 9.25e-57 |
| AHI59109.1 | locillomycin\_synthase\_B | BGC0001005 | NRP+Polyketide | 25.0 | 23.3 | 221.0 | 1.01e-56 |
| CAD17793.1 | probable\_non\_ribosomal\_peptide\_synthetase\_protein | BGC0001363 | NRP+Polyketide | 27.0 | 20.9 | 221.0 | 1.14e-56 |
| AXN93605.1 | PuwA | BGC0001953 | NRP | 25.0 | 22.8 | 221.0 | 1.15e-56 |
| AAY91419.3 | non-ribosomal\_peptide\_synthetase\_OfaA | BGC0000399 | NRP:Cyclic depsipeptide | 28.0 | 26.3 | 221.0 | 1.2e-56 |
| AGZ15458.1 | putative\_non-ribosomal\_peptide\_synthetase | BGC0001036 | NRP+Polyketide | 26.0 | 26.2 | 221.0 | 1.41e-56 |
| ABQ96384.2 | fusaricidin\_synthetase | BGC0001152 | Polyketide+NRP:Lipopeptide | 27.0 | 25.2 | 221.0 | 1.56e-56 |
| AHI59108.1 | locillomycin\_synthase\_A | BGC0001005 | NRP+Polyketide | 22.0 | 25.7 | 220.0 | 1.71e-56 |
| CAM56771.1 |  | BGC0000354 | NRP | 27.0 | 25.5 | 220.0 | 1.73e-56 |
| WP\_245566645.1 | amino\_acid\_adenylation\_domain-containing\_protein | BGC0002467 | NRP | 27.0 | 24.3 | 221.0 | 1.9e-56 |
| KFL51886.1 | amino\_acid\_adenylation\_protein | BGC0001711 | NRP+Polyketide | 27.0 | 26.4 | 220.0 | 2.02e-56 |
| NAO96319.1 | amino\_acid\_adenylation\_domain-containing\_protein | BGC0002117 | NRP | 25.0 | 26.1 | 220.0 | 2.13e-56 |
| AKC91856.1 | nonribosomal\_peptide\_synthetase | BGC0001414 | NRP | 27.0 | 22.1 | 220.0 | 2.31e-56 |
| AEW31019.1 | plipastatin\_synthetase | BGC0000407 | NRP | 23.0 | 25.5 | 220.0 | 2.4e-56 |
| WP\_039806850.1 | non-ribosomal\_peptide\_synthetase | BGC0002001 | NRP+Polyketide | 26.0 | 26.1 | 220.0 | 2.42e-56 |
| AAF08796.1 | MycB | BGC0001103 | NRP+Polyketide | 25.0 | 24.9 | 220.0 | 2.48e-56 |
| WP\_100939443.1 | non-ribosomal\_peptide\_synthetase | BGC0002071 | NRP:Lipopeptide | 28.0 | 21.1 | 220.0 | 2.54e-56 |
| APZ78679.1 | nonribosomal\_peptide\_synthetase | BGC0001417 | NRP:Cyclic depsipeptide+Polyketide:Iterative type I polyketide | 25.0 | 22.9 | 220.0 | 2.66e-56 |
| APZ78833.1 | nonribosomal\_peptide\_synthetase | BGC0001430 | NRP:Cyclic depsipeptide+Polyketide:Iterative type I polyketide | 26.0 | 22.8 | 220.0 | 2.66e-56 |
| AAD44233.1 | PstA | BGC0000362 | NRP | 27.0 | 24.6 | 220.0 | 2.8e-56 |
| ATY37592.1 | BogE | BGC0001532 | NRP | 25.0 | 24.1 | 219.0 | 3.06e-56 |
| AQX14493.1 | monobactam\_NRPS\_scaffold\_2 | BGC0001672 | NRP | 24.0 | 24.9 | 219.0 | 3.22e-56 |
| ABD14712.1 | cesB | BGC0000320 | NRP:Cyclic depsipeptide | 24.0 | 25.6 | 219.0 | 3.23e-56 |
| AAZ23075.1 | peptide\_synthetase | BGC0000291 | NRP | 26.0 | 26.9 | 220.0 | 3.36e-56 |
| QRG35014.1 | NRPS | BGC0002378 | NRP | 27.0 | 24.5 | 219.0 | 4.16e-56 |
| KYQ85937.1 | hypothetical\_protein | BGC0002437 | NRP | 23.0 | 25.4 | 219.0 | 4.86e-56 |
| PHM26613.1 | pyoverdine\_synthetase\_D | BGC0001130 | NRP+Polyketide | 26.0 | 25.7 | 219.0 | 4.92e-56 |
| ATO51563.1 | non-ribosomal\_peptide\_synthetase | BGC0001796 | NRP | 25.0 | 25.1 | 219.0 | 5.75e-56 |
| APZ78755.1 | nonribosomal\_peptide\_synthetase | BGC0001423 | NRP:Cyclic depsipeptide+Polyketide:Iterative type I polyketide | 26.0 | 22.8 | 219.0 | 5.94e-56 |
| APZ78691.1 | nonribosomal\_peptide\_synthetase | BGC0001418 | NRP:Cyclic depsipeptide+Polyketide:Iterative type I polyketide | 25.0 | 22.7 | 219.0 | 5.95e-56 |
| AFH75328.1 | nonribosomal\_peptide\_synthetase | BGC0000398 | NRP:Cyclic depsipeptide | 27.0 | 25.2 | 218.0 | 6.07e-56 |
| QCP68969.1 | VatN | BGC0002296 | NRP+Polyketide | 26.0 | 22.9 | 218.0 | 6.14e-56 |
| AKJ15826.1 | peptide\_synthetase | BGC0002735 | Polyketide+NRP | 26.0 | 26.3 | 218.0 | 7.09e-56 |
| AGM16413.1 | paenibacterin\_synthetase\_B | BGC0000400 | NRP | 25.0 | 23.7 | 219.0 | 7.53e-56 |
| APZ78728.1 | nonribosomal\_peptide\_synthetase | BGC0001421 | NRP:Cyclic depsipeptide+Polyketide:Iterative type I polyketide | 26.0 | 22.7 | 218.0 | 7.77e-56 |
| AHY86403.1 | non\_ribosomal\_peptide\_synthetase | BGC0000329 | NRP | 26.0 | 26.0 | 218.0 | 8.07e-56 |
| AAN85493.1 | nonribosomal\_peptide\_synthetase | BGC0001101 | NRP+Polyketide:Modular type I polyketide+Polyketide:Trans-AT type I polyketide | 25.0 | 26.3 | 216.0 | 8.21e-56 |
| AQZ69228.1 | hypothetical\_protein | BGC0001635 | NRP+Polyketide | 26.0 | 25.5 | 218.0 | 8.39e-56 |
| AZM57022.1 | non-ribosomal\_peptide\_synthetase | BGC0002314 | NRP | 26.0 | 26.0 | 218.0 | 8.51e-56 |
| QIE08737.1 | non-ribosomal\_peptide\_synthetase | BGC0002544 | NRP | 27.0 | 23.8 | 218.0 | 8.76e-56 |
| UEF20592.1 | nonribosomal\_peptide\_synthetase | BGC0002360 | NRP | 26.0 | 25.2 | 216.0 | 8.77e-56 |
| WP\_064118560.1 | non-ribosomal\_peptide\_synthetase | BGC0001509 | NRP | 26.0 | 26.4 | 218.0 | 8.94e-56 |
| AGS77309.1 | NRPS\_modules\_4-6 | BGC0001178 | NRP:Glycopeptide | 26.0 | 25.0 | 218.0 | 1.15e-55 |
| ATW47208.1 | non-ribosomal\_peptide\_synthetase | BGC0002466 | NRP | 27.0 | 25.6 | 218.0 | 1.23e-55 |
| AXN93614.1 | PuwF | BGC0001953 | NRP | 25.0 | 25.8 | 218.0 | 1.29e-55 |
| ABA73955.1 | putative\_non-ribosomal\_peptide\_synthetase | BGC0001842 | NRP:Lipopeptide | 26.0 | 25.0 | 218.0 | 1.62e-55 |
| AHZ20774.1 | non-ribosomal\_peptide\_synthase | BGC0000369 | NRP+Saccharide:Hybrid/tailoring saccharide | 26.0 | 25.1 | 217.0 | 1.88e-55 |
| CCJ67647.1 | JagC | BGC0001127 | NRP | 26.0 | 25.8 | 217.0 | 1.88e-55 |
| ctg1\_orf17 |  | BGC0001457 | NRP | 27.0 | 25.2 | 214.0 | 1.97e-55 |
| EFE73312.1 | nonribosomal\_peptide\_synthetase | BGC0000431 | NRP:Cyclic depsipeptide | 27.0 | 25.2 | 217.0 | 2.17e-55 |
| AKJ29410.1 | peptide\_synthetase | BGC0001608 | NRP | 27.0 | 26.1 | 217.0 | 2.34e-55 |
| BGRAMDRAFT\_RS22640 | amino\_acid\_adenylation\_domain-containing\_protein | BGC0001999 | NRP | 26.0 | 27.5 | 215.0 | 2.7e-55 |
| TRX17523.1 | non-ribosomal\_peptide\_synthetase | BGC0002329 | NRP | 26.0 | 26.6 | 216.0 | 2.73e-55 |
| APZ78715.1 | nonribosomal\_peptide\_synthetase | BGC0001420 | NRP:Cyclic depsipeptide+Polyketide:Iterative type I polyketide | 25.0 | 22.7 | 216.0 | 2.98e-55 |
| APZ78808.1 | nonribosomal\_peptide\_synthetase | BGC0001428 | NRP:Cyclic depsipeptide+Polyketide:Iterative type I polyketide | 26.0 | 22.8 | 216.0 | 2.98e-55 |
| APZ78768.1 | nonribosomal\_peptide\_synthetase | BGC0001425 | NRP:Cyclic depsipeptide+Polyketide:Iterative type I polyketide | 26.0 | 22.9 | 216.0 | 2.98e-55 |
| APZ78794.1 | nonribosomal\_peptide\_synthetase | BGC0001427 | NRP:Cyclic depsipeptide+Polyketide:Iterative type I polyketide | 26.0 | 22.9 | 216.0 | 2.98e-55 |
| AUD11994.1 | OrbI | BGC0001721 | NRP | 26.0 | 24.6 | 216.0 | 3.02e-55 |
| WP\_010369430.1 | non-ribosomal\_peptide\_synthetase | BGC0000314 | Polyketide+NRP:Cyclic depsipeptide+Other:Aminocoumarin | 25.0 | 26.1 | 216.0 | 3.08e-55 |
| AHZ34241.1 | CipD | BGC0001389 | NRP | 26.0 | 25.3 | 216.0 | 3.41e-55 |
| AAC06347.1 | bacitracin\_synthetase\_2 | BGC0000310 | NRP | 24.0 | 24.4 | 216.0 | 3.57e-55 |
| APZ78855.1 | nonribosomal\_peptide\_synthetase | BGC0001432 | NRP:Cyclic depsipeptide+Polyketide:Iterative type I polyketide | 27.0 | 23.1 | 216.0 | 3.9e-55 |
| ARU08075.1 | mlcM | BGC0001448 | NRP:Lipopeptide:Ca+-dependent lipopeptide | 27.0 | 26.0 | 216.0 | 4.24e-55 |
| AHB82058.1 | non\_ribosomal\_peptide\_synthetase | BGC0001019 | NRP+Polyketide:Modular type I polyketide | 25.0 | 22.9 | 216.0 | 4.4e-55 |
| BAC67535.1 | arthrofactin\_synthetase\_B | BGC0000305 | NRP:Lipopeptide | 26.0 | 26.6 | 216.0 | 4.47e-55 |
| AWN90\_15505 | non-ribosomal\_peptide\_synthetase | BGC0002352 | Other | 26.0 | 26.8 | 215.0 | 4.56e-55 |
| ALG65318.1 | Cal18 | BGC0001297 | NRP | 26.0 | 25.5 | 216.0 | 4.63e-55 |
| AEC14347.1 | nonribosomal\_peptide\_synthetase | BGC0000377 | NRP | 23.0 | 25.8 | 215.0 | 4.67e-55 |
| AAS98786.1 | nonribosomal\_peptide\_synthetase | BGC0001001 | NRP+Polyketide | 24.0 | 27.0 | 213.0 | 5.58e-55 |
| CAJ46692.1 | non-ribosomal\_peptide\_synthase | BGC0000969 | NRP:Cyclic depsipeptide+Polyketide:Modular type I polyketide | 25.0 | 22.8 | 216.0 | 5.65e-55 |
| ANG60380.1 | nonribosomal\_peptide\_synthetase\_BudB | BGC0001434 | NRP | 25.0 | 23.1 | 214.0 | 6.11e-55 |
| APU91750.1 | Non-Ribosomal\_Peptide\_Synthetase | BGC0001806 | NRP | 25.0 | 26.0 | 216.0 | 7.02e-55 |
| OKJ61999.1 | peptide\_synthetase | BGC0002147 | NRP | 26.0 | 25.7 | 215.0 | 7.58e-55 |
| CBJ90287.1 | peptide\_synthetase | BGC0000416 | NRP | 25.0 | 22.4 | 214.0 | 7.94e-55 |
| WP\_013184317.1 | non-ribosomal\_peptide\_synthetase | BGC0001692 | NRP | 25.0 | 22.4 | 214.0 | 7.94e-55 |
| AAF08797.1 | MycC | BGC0001103 | NRP+Polyketide | 24.0 | 24.4 | 215.0 | 7.99e-55 |
| AXG46164.1 | non-ribosomal\_peptide\_synthetase | BGC0002713 | NRP | 25.0 | 22.5 | 214.0 | 8.1e-55 |
| AHZ20784.1 | non-ribosomal\_peptide\_synthase | BGC0000369 | NRP+Saccharide:Hybrid/tailoring saccharide | 24.0 | 26.0 | 215.0 | 8.41e-55 |
| QPI18727.1 | nonribosomal\_peptide\_synthetase | BGC0002125 | NRP:Cyclic depsipeptide | 26.0 | 25.9 | 215.0 | 8.67e-55 |
| CCJ67639.1 | TaaD | BGC0000447 | NRP:Lipopeptide | 26.0 | 25.0 | 215.0 | 9.08e-55 |
| AJV88377.1 | MfnE | BGC0001214 | NRP | 27.0 | 25.2 | 215.0 | 9.4e-55 |
| ABM34276.1 | amino\_acid\_adenylation\_domain\_protein | BGC0002419 | NRP+Polyketide | 26.0 | 25.9 | 213.0 | 1.03e-54 |
| QVQ62868.1 | nonribosomal\_peptide\_synthase | BGC0002373 | NRP | 27.0 | 26.2 | 214.0 | 1.34e-54 |
| AGU50950.1 | putative\_non-ribosomal\_peptide\_synthetase | BGC0002417 | NRP+Polyketide | 25.0 | 26.4 | 214.0 | 1.37e-54 |
| WP\_084702182.1 | non-ribosomal\_peptide\_synthetase | BGC0001211 | NRP | 27.0 | 24.7 | 214.0 | 1.47e-54 |
| CAR51994.1 | ornibactin\_biosynthesis\_non-ribosomal\_peptide\_synthase | BGC0002569 | NRP | 26.0 | 24.4 | 214.0 | 1.53e-54 |
| AAO62587.1 | peptide\_sythetase | BGC0001016 | NRP+Polyketide | 25.0 | 25.7 | 214.0 | 1.54e-54 |
| MCC5036785.1 | amino\_acid\_adenylation\_domain-containing\_protein | BGC0002638 | NRP | 24.0 | 26.2 | 214.0 | 1.74e-54 |
| AHD05677.1 | nonribosomal\_peptide\_ligase\_subunit | BGC0000402 | NRP | 24.0 | 25.8 | 214.0 | 1.79e-54 |
| BAF50711.1 | non\_ribosomal\_peptide\_synthetase\_for\_virginiamycin\_S | BGC0001116 | NRP+Polyketide | 26.0 | 26.6 | 214.0 | 1.82e-54 |
| AQX14441.1 | EM5400\_NRPS\_scaffold | BGC0001671 | NRP | 25.0 | 26.3 | 214.0 | 1.89e-54 |
| AQM37583.1 | nonribosomal\_peptide\_synthetase | BGC0001424 | NRP:Cyclic depsipeptide+Polyketide:Iterative type I polyketide | 26.0 | 22.7 | 214.0 | 1.95e-54 |
| RSO11556.1 | non-ribosomal\_peptide\_synthetase | BGC0002637 | NRP | 27.0 | 24.6 | 214.0 | 1.97e-54 |
| AEI58865.1 | peptide\_synthetase | BGC0000455 | NRP | 27.0 | 24.7 | 214.0 | 1.98e-54 |
| ADQ55475.1 | NRPS | BGC0000350 | NRP:Beta-lactam | 25.0 | 23.5 | 213.0 | 2.02e-54 |
| AQH32485.1 | peptide\_synthetase | BGC0001667 | NRP+Polyketide | 24.0 | 25.5 | 213.0 | 2.02e-54 |
| NAO96318.1 | amino\_acid\_adenylation\_domain-containing\_protein | BGC0002117 | NRP | 25.0 | 26.1 | 213.0 | 2.05e-54 |
| CAE53352.1 | non-ribosomal\_peptide\_synthetase | BGC0000440 | NRP:Glycopeptide | 27.0 | 25.4 | 214.0 | 2.19e-54 |
| QYC40290.1 | A50926\_NRPS,\_module\_7 | BGC0002344 | NRP | 27.0 | 26.1 | 213.0 | 2.32e-54 |
| DMA15\_34345 | non-ribosomal\_peptide\_synthetase | BGC0002314 | NRP | 25.0 | 25.7 | 212.0 | 2.61e-54 |
| QBC75022.1 | non-ribosomal\_peptide\_synthetase | BGC0001968 | NRP | 25.0 | 26.7 | 213.0 | 3.13e-54 |
| AAL33757.1 | putative\_non-ribosomal\_peptide\_synthetase | BGC0000421 | NRP | 24.0 | 26.2 | 210.0 | 3.25e-54 |
| CAJ18237.2 | non-ribosomal\_peptide\_synthetase\_B | BGC0000354 | NRP | 27.0 | 26.4 | 213.0 | 3.38e-54 |
| QCQ67880.1 | non-ribosomal\_peptide\_synthetase | BGC0002297 | NRP+Polyketide | 24.0 | 26.1 | 213.0 | 3.45e-54 |
| ATY37608.1 | BreC | BGC0001536 | NRP | 25.0 | 25.1 | 213.0 | 3.48e-54 |
| AIW82284.1 | PuwG | BGC0001125 | NRP+Polyketide | 25.0 | 26.6 | 213.0 | 3.54e-54 |
| KGA48739.1 | amino\_acid\_adenylation\_domain\_protein | BGC0002413 | NRP | 26.0 | 23.9 | 213.0 | 3.79e-54 |
| AZM51141.1 | non-ribosomal\_peptide\_synthetase | BGC0002702 | NRP | 27.0 | 25.1 | 212.0 | 4.6e-54 |
| QKW60392.1 | amino\_acid\_adenylation\_domain-containing\_protein | BGC0002288 | NRP | 26.0 | 26.1 | 210.0 | 4.94e-54 |
| CCM44337.1 | Nonribosomal\_peptide\_synthetase | BGC0001056 | NRP+Polyketide:Modular type I polyketide+Polyketide:PUFA synthase or related polyketide | 25.0 | 23.9 | 212.0 | 6.03e-54 |
| BAH43765.1 | tyrocidine\_synthetase\_II | BGC0000452 | NRP | 27.0 | 23.7 | 212.0 | 6.12e-54 |
| AJD47484.1 | protein\_PvdD | BGC0002418 | NRP+Polyketide | 27.0 | 23.6 | 212.0 | 6.35e-54 |
| AZM50110.1 | non-ribosomal\_peptide\_synthetase | BGC0002702 | NRP | 27.0 | 24.8 | 211.0 | 6.81e-54 |
| MBE3200466.1 | non-ribosomal\_peptide\_synthetase | BGC0002409 | NRP | 26.0 | 27.1 | 212.0 | 7.19e-54 |
| QEO74904.1 | AMP-dependent\_synthetase\_and\_ligase | BGC0002588 | Other | 28.0 | 25.2 | 212.0 | 7.53e-54 |
| QKW60393.1 | amino\_acid\_adenylation\_domain-containing\_protein | BGC0002288 | NRP | 25.0 | 26.3 | 210.0 | 8.44e-54 |
| CDG17982.1 | Non-ribosomal\_peptide\_synthetase | BGC0000464 | NRP:Cyclic depsipeptide | 24.0 | 25.6 | 212.0 | 8.84e-54 |
| QTT72092.1 | amino\_acid\_adenylation\_domain-containing\_protein | BGC0002350 | NRP+Polyketide+Saccharide | 26.0 | 25.7 | 211.0 | 9.02e-54 |
| QTT72097.1 | amino\_acid\_adenylation\_domain-containing\_protein | BGC0002350 | NRP+Polyketide+Saccharide | 26.0 | 25.7 | 211.0 | 9.12e-54 |
| BCJ07529.1 | hypothetical\_protein | BGC0002379 | NRP | 27.0 | 26.2 | 211.0 | 1.03e-53 |
| CAD91211.1 | putative\_non-ribosomal\_peptide\_synthetase,\_module\_7 | BGC0000289 | NRP:Glycopeptide+Saccharide:Hybrid/tailoring saccharide | 27.0 | 26.3 | 211.0 | 1.16e-53 |
| AAC06348.1 | bacitracin\_synthetase\_3 | BGC0000310 | NRP | 24.0 | 21.8 | 211.0 | 1.22e-53 |
| AGZ03651.1 | sevB | BGC0000426 | NRP | 24.0 | 25.0 | 211.0 | 1.22e-53 |
| KPN93064.1 | NupB | BGC0001416 | NRP | 26.0 | 25.3 | 211.0 | 1.46e-53 |
| AHJ31215.1 | Long-chain-fatty-acid--CoA\_ligase | BGC0000430 | NRP+Polyketide:Modular type I polyketide | 24.0 | 26.2 | 211.0 | 1.89e-53 |
| AZM51140.1 | non-ribosomal\_peptide\_synthetase | BGC0002702 | NRP | 26.0 | 24.6 | 209.0 | 1.94e-53 |
| QVQ62855.1 | nonribosomal\_peptide\_synthase | BGC0002373 | NRP | 26.0 | 26.6 | 210.0 | 2.33e-53 |
| WP\_013310343.1 | non-ribosomal\_peptide\_synthetase | BGC0001728 | NRP+Polyketide | 25.0 | 25.0 | 209.0 | 2.63e-53 |
| ANZ15839.1 | peptide\_synthetase\_ScpsB | BGC0001569 | NRP | 26.0 | 26.5 | 210.0 | 2.64e-53 |
| QCP68975.1 | VatS | BGC0002296 | NRP+Polyketide | 25.0 | 22.7 | 209.0 | 2.76e-53 |
| AXN93615.1 | PuwG | BGC0001953 | NRP | 25.0 | 25.7 | 210.0 | 2.82e-53 |
| APZ78743.1 | nonribosomal\_peptide\_synthetase | BGC0001422 | NRP:Cyclic depsipeptide+Polyketide:Iterative type I polyketide | 26.0 | 22.7 | 210.0 | 2.86e-53 |
| ABL74939.1 | NRPS | BGC0001048 | NRP:Glycopeptide+Polyketide:Modular type I polyketide+Saccharide:Hybrid/tailoring saccharide | 26.0 | 25.6 | 207.0 | 3.31e-53 |
| MCC5036784.1 | amino\_acid\_adenylation\_domain-containing\_protein | BGC0002638 | NRP | 25.0 | 26.5 | 209.0 | 3.45e-53 |
| SDF67417.1 | amino\_acid\_adenylation\_domain-containing\_protein | BGC0002422 | NRP | 25.0 | 25.8 | 209.0 | 3.73e-53 |
| AWI62627.1 | nonribosomal\_peptide\_synthetase | BGC0001822 | NRP | 25.0 | 26.4 | 209.0 | 3.94e-53 |
| AAO56329.1 | non-ribosomal\_peptide\_synthetase\_SyfB | BGC0000435 | NRP | 28.0 | 20.6 | 209.0 | 4.6e-53 |
| WP\_028678148.1 | non-ribosomal\_peptide\_synthetase | BGC0001228 | NRP:Cyclic depsipeptide | 26.0 | 23.1 | 209.0 | 4.95e-53 |
| AAG02359.1 | peptide\_synthetase\_NRPS5-4-3 | BGC0000963 | NRP:Glycopeptide+Polyketide:Modular type I polyketide+Saccharide:Hybrid/tailoring saccharide | 26.0 | 25.7 | 209.0 | 5.97e-53 |
| AAT01806.1 | non-ribosomal\_peptide\_synthetase | BGC0000365 | NRP | 26.0 | 25.2 | 209.0 | 6.73e-53 |
| AFK57221.1 | DidJ | BGC0000985 | Polyketide+NRP:Cyclic depsipeptide | 26.0 | 22.0 | 208.0 | 6.76e-53 |
| UMP03490.1 | NmvB | BGC0002649 | NRP+Polyketide | 27.0 | 25.3 | 206.0 | 6.83e-53 |
| QDQ83031.1 | amino\_acid\_adenylation\_domain-containing\_protein | BGC0002564 | NRP | 26.0 | 25.2 | 207.0 | 7.71e-53 |
| AXN93581.1 | PuwF-G | BGC0001950 | NRP | 25.0 | 26.1 | 209.0 | 7.84e-53 |
| AHZ34239.1 | CipB | BGC0001389 | NRP | 26.0 | 26.0 | 208.0 | 8.51e-53 |
| QMN69932.1 | PsoA | BGC0002521 | NRP | 28.0 | 20.5 | 208.0 | 8.68e-53 |
| QNN94286.1 | EmeB | BGC0002555 | NRP+Polyketide | 25.0 | 33.1 | 209.0 | 8.91e-53 |
| AAG02356.1 | peptide\_synthetase\_NRPS7 | BGC0000963 | NRP:Glycopeptide+Polyketide:Modular type I polyketide+Saccharide:Hybrid/tailoring saccharide | 27.0 | 26.5 | 206.0 | 9.42e-53 |
| QDF82255.1 | non-ribosomal\_peptide\_synthetase | BGC0001980 | NRP | 26.0 | 25.4 | 208.0 | 9.53e-53 |
| PHM26612.1 | pvdj | BGC0001130 | NRP+Polyketide | 26.0 | 25.0 | 208.0 | 9.55e-53 |
| AXN93590.1 | PuwF-G | BGC0001951 | NRP | 25.0 | 26.1 | 208.0 | 1.34e-52 |
| CBJ90289.1 | peptide\_synthetase | BGC0000416 | NRP | 25.0 | 22.5 | 207.0 | 1.85e-52 |
| WP\_013184319.1 | non-ribosomal\_peptide\_synthetase | BGC0001692 | NRP | 25.0 | 22.5 | 207.0 | 1.85e-52 |
| UKO95756.1 | amino\_acid\_adenylation\_domain-containing\_protein | BGC0002632 | NRP | 25.0 | 26.1 | 206.0 | 1.89e-52 |
| QLY89263.1 | pseudodesmin\_synthetase | BGC0002522 | NRP | 27.0 | 25.4 | 207.0 | 2.14e-52 |
| AAZ55899.1 | amino\_acid\_adenylation | BGC0000359 | NRP | 27.0 | 25.4 | 204.0 | 2.26e-52 |
| ABM21571.1 | crpC | BGC0000975 | NRP+Polyketide | 25.0 | 22.5 | 206.0 | 2.36e-52 |
| AAG02355.1 | peptide\_synthetase\_NRPS9-8 | BGC0000963 | NRP:Glycopeptide+Polyketide:Modular type I polyketide+Saccharide:Hybrid/tailoring saccharide | 24.0 | 25.8 | 206.0 | 2.59e-52 |
| AZL87944.1 | aspergillic\_acid\_non-ribosomal\_peptide\_sythetase-like\_(NRPS-like)\_(AsaC) | BGC0002602 | Alkaloid | 26.0 | 22.2 | 204.0 | 2.97e-52 |
| AGN74876.1 | nonribosomal\_peptide\_synthetase | BGC0000459 | NRP:Cyclic depsipeptide+Polyketide:Trans-AT type I polyketide | 28.0 | 26.0 | 206.0 | 2.97e-52 |
| AFH75321.1 | nonribosomal\_peptide\_synthetase | BGC0000425 | NRP:Cyclic depsipeptide | 25.0 | 26.2 | 207.0 | 3.05e-52 |
| CBJ90288.1 | Peptide\_synthetase | BGC0000416 | NRP | 25.0 | 22.8 | 206.0 | 3.05e-52 |
| WP\_013184318.1 | non-ribosomal\_peptide\_synthetase | BGC0001692 | NRP | 25.0 | 22.8 | 206.0 | 3.05e-52 |
| QTT72101.1 | non-ribosomal\_peptide\_synthetase | BGC0002350 | NRP+Polyketide+Saccharide | 24.0 | 25.8 | 206.0 | 3.37e-52 |
| AAU39360.1 | lichenysin\_synthase\_LchAB | BGC0000381 | NRP | 25.0 | 23.6 | 206.0 | 3.42e-52 |
| CUX79061.1 | Octapeptin\_synthase\_subunit\_B | BGC0001715 | NRP | 23.0 | 25.0 | 206.0 | 3.76e-52 |
| QMS47799.1 | JesA | BGC0001629 | NRP:Lipopeptide | 28.0 | 20.7 | 206.0 | 3.84e-52 |
| ATU31795.1 | NRPS | BGC0001814 | NRP | 26.0 | 26.5 | 206.0 | 3.91e-52 |
| BAH22763.1 | nonribosomal\_peptide\_synthetase | BGC0001018 | NRP | 25.0 | 26.3 | 204.0 | 4.54e-52 |
| QBQ12465.1 | amino\_acid\_adenylation\_domain-containing\_protein | BGC0002693 | NRP | 26.0 | 25.1 | 206.0 | 4.69e-52 |
| QUJ09167.1 | Lon20 | BGC0002440 | NRP | 26.0 | 25.0 | 206.0 | 4.71e-52 |
| AOA33122.1 | Nonribosomal\_peptide\_synthetase | BGC0001346 | NRP:Cyclic depsipeptide | 27.0 | 26.3 | 206.0 | 4.76e-52 |
| BCJ07533.1 | hypothetical\_protein | BGC0002379 | NRP | 25.0 | 25.9 | 206.0 | 5.02e-52 |
| ABM34277.1 | amino\_acid\_adenylation\_domain\_protein | BGC0002419 | NRP+Polyketide | 26.0 | 26.5 | 206.0 | 5.1e-52 |
| ACM79806.1 | ZmaB | BGC0001059 | NRP+Polyketide | 24.0 | 25.4 | 205.0 | 5.2e-52 |
| AEI58867.1 | peptide\_synthetase | BGC0000455 | NRP | 26.0 | 26.4 | 205.0 | 6.62e-52 |
| DAB41476.1 | nonribosomal\_peptide\_synthetase | BGC0001230 | NRP:Cyclic depsipeptide+Polyketide:Modular type I polyketide | 26.0 | 24.7 | 204.0 | 6.63e-52 |
| CAK15815.1 | putative\_non\_ribosomal\_peptide\_synthetase | BGC0000344 | NRP | 25.0 | 25.9 | 206.0 | 7.09e-52 |
| EWS95124.1 | hypothetical\_protein | BGC0000306 | NRP:Lipopeptide | 26.0 | 22.9 | 205.0 | 8.12e-52 |
| KYC42613.1 | non-ribosomal\_peptide\_synthetase | BGC0002484 | NRP+Polyketide | 26.0 | 26.9 | 205.0 | 8.47e-52 |
| AFJ14793.1 | PlpD | BGC0000403 | NRP | 24.0 | 23.8 | 204.0 | 8.58e-52 |
| AAG02364.1 | peptide\_synthetase\_NRPS2-1 | BGC0000963 | NRP:Glycopeptide+Polyketide:Modular type I polyketide+Saccharide:Hybrid/tailoring saccharide | 26.0 | 24.9 | 205.0 | 8.68e-52 |
| BCJ07599.1 | hypothetical\_protein | BGC0002379 | NRP | 27.0 | 25.8 | 202.0 | 8.69e-52 |
| AGM16412.1 | paenibacterin\_synthetase\_A | BGC0000400 | NRP | 25.0 | 23.7 | 205.0 | 8.93e-52 |
| ABW17376.1 | PsoB | BGC0000411 | NRP | 25.0 | 25.9 | 205.0 | 9.11e-52 |
| ACM68690.1 | AerG1 | BGC0000298 | NRP | 25.0 | 26.2 | 202.0 | 9.86e-52 |
| AAZ23076.1 | peptide\_synthetase | BGC0000291 | NRP | 25.0 | 26.6 | 204.0 | 9.89e-52 |
| AGS77310.1 | NRPS\_module\_7 | BGC0001178 | NRP:Glycopeptide | 25.0 | 26.0 | 204.0 | 1.14e-51 |
| AIW82283.1 | PuwF | BGC0001125 | NRP+Polyketide | 25.0 | 26.3 | 204.0 | 1.39e-51 |
| APZ78856.1 | nonribosomal\_peptide\_synthetase | BGC0001432 | NRP:Cyclic depsipeptide+Polyketide:Iterative type I polyketide | 26.0 | 25.5 | 204.0 | 1.4e-51 |
| QTT72098.1 | non-ribosomal\_peptide\_synthetase | BGC0002350 | NRP+Polyketide+Saccharide | 25.0 | 25.4 | 202.0 | 1.47e-51 |
| QTT72100.1 | non-ribosomal\_peptide\_synthetase | BGC0002350 | NRP+Polyketide+Saccharide | 26.0 | 26.5 | 202.0 | 1.52e-51 |
| WP\_050383082.1 | non-ribosomal\_peptide\_synthetase | BGC0001451 | NRP | 25.0 | 21.8 | 204.0 | 1.62e-51 |
| QGQ63518.1 | nonribosomal\_peptide\_synthetase\_modules\_A | BGC0002548 | NRP | 27.0 | 25.1 | 204.0 | 1.69e-51 |
| WP\_003981346.1 | non-ribosomal\_peptide\_synthetase | BGC0001813 | NRP | 26.0 | 26.3 | 204.0 | 1.71e-51 |
| CAG15011.1 | peptide\_synthetase,\_module\_4-6 | BGC0000441 | NRP | 26.0 | 25.4 | 204.0 | 1.79e-51 |
| OKA09425.1 | non-ribosomal\_peptide\_synthetase | BGC0001459 | NRP:Glycopeptide | 26.0 | 26.2 | 203.0 | 1.95e-51 |
| EME52990.1 | amino\_acid\_adenylation\_protein | BGC0001460 | NRP:Glycopeptide | 26.0 | 24.7 | 204.0 | 2.13e-51 |
| ALD82526.1 | non-ribosomal\_peptide\_synthase | BGC0001212 | NRP+Polyketide | 25.0 | 26.0 | 204.0 | 2.21e-51 |
| AGZ15460.1 | putative\_non-ribosomal\_peptide\_synthetase | BGC0001036 | NRP+Polyketide | 26.0 | 23.9 | 203.0 | 2.24e-51 |
| AYA22334.1 | KerC | BGC0001955 | NRP | 25.0 | 24.4 | 204.0 | 2.33e-51 |
| CDN62030.1 | Peptide\_synthetase | BGC0001599 | NRP | 27.0 | 22.9 | 202.0 | 2.43e-51 |
| QPI18729.1 | nonribosomal\_peptide\_synthetase | BGC0002125 | NRP:Cyclic depsipeptide | 25.0 | 22.1 | 203.0 | 2.78e-51 |
| OTA20325.1 | peptide\_synthase | BGC0001824 | NRP | 25.0 | 26.2 | 203.0 | 3e-51 |
| AQX14497.1 | monobactam\_NRPS\_scaffold\_4 | BGC0001672 | NRP | 24.0 | 25.4 | 203.0 | 3.2e-51 |
| AXN93591.1 | PuwH | BGC0001951 | NRP | 25.0 | 26.0 | 201.0 | 4.11e-51 |
| QMW33900.1 | hypothetical\_protein | BGC0002167 | NRP | 25.0 | 22.6 | 200.0 | 4.12e-51 |
| MBX9445647.1 | amino\_acid\_adenylation\_domain-containing\_protein | BGC0002414 | NRP | 27.0 | 26.1 | 202.0 | 4.54e-51 |
| AAZ03551.1 | McnB | BGC0000332 | NRP | 24.0 | 26.3 | 200.0 | 5.49e-51 |
| WP\_054234617.1 | non-ribosomal\_peptide\_synthetase | BGC0002014 | NRP+Polyketide | 27.0 | 22.5 | 202.0 | 5.55e-51 |
| AAU34202.1 | mannopeptimycin\_peptide\_synthetase\_MppA | BGC0000388 | NRP | 26.0 | 23.5 | 202.0 | 5.83e-51 |
| AAX31558.1 | peptide\_synthetase\_2 | BGC0000336 | NRP | 27.0 | 25.5 | 202.0 | 5.92e-51 |
| AHF21229.1 | TriE | BGC0000449 | NRP | 25.0 | 24.5 | 202.0 | 6.65e-51 |
| WP\_010369428.1 | non-ribosomal\_peptide\_synthetase | BGC0000314 | Polyketide+NRP:Cyclic depsipeptide+Other:Aminocoumarin | 25.0 | 25.7 | 202.0 | 7.43e-51 |
| QIE07359.1 | dimodular\_nonribosomal\_peptide\_synthase\_NecA | BGC0002050 | NRP+Polyketide:Trans-AT type I polyketide | 26.0 | 25.2 | 201.0 | 7.88e-51 |
| CAJ96472.1 | non-ribosomal\_peptide\_synthetase | BGC0000330 | NRP:NRP siderophore | 27.0 | 26.9 | 202.0 | 8.08e-51 |
| XP\_002373813.1 | NRPS-like\_enzyme,\_putative | BGC0001516 | NRP | 26.0 | 22.2 | 199.0 | 8.51e-51 |
| AXN93582.1 | PuwH | BGC0001950 | NRP | 25.0 | 25.3 | 199.0 | 8.64e-51 |
| AFH75329.1 | nonribosomal\_peptide\_synthetase | BGC0000398 | NRP:Cyclic depsipeptide | 28.0 | 20.7 | 202.0 | 9.11e-51 |
| AYA44686.1 | icosalide\_NRPS | BGC0001833 | NRP:Lipopeptide | 26.0 | 25.3 | 202.0 | 9.43e-51 |
| AAM80536.1 | StaD | BGC0000290 | NRP:Glycopeptide | 26.0 | 25.6 | 201.0 | 9.8e-51 |
| ACG60776.1 | NRPS(AL/ACP/C/A/PCP/C/A) | BGC0001058 | NRP:Glycopeptide+Polyketide:Modular type I polyketide+Saccharide:Hybrid/tailoring saccharide | 27.0 | 27.0 | 201.0 | 9.92e-51 |
| AAO56328.1 | non-ribosomal\_peptide\_synthetase\_SyfA | BGC0000435 | NRP | 27.0 | 24.7 | 201.0 | 1.07e-50 |
| QRN75755.1 | Amino\_acid\_adenylation\_domain\_protein | BGC0002114 | NRP+Polyketide | 25.0 | 25.9 | 201.0 | 1.11e-50 |
| MCC5036786.1 | amino\_acid\_adenylation\_domain-containing\_protein | BGC0002638 | NRP | 26.0 | 24.7 | 201.0 | 1.15e-50 |
| AWI62629.1 | nonribosomal\_peptide\_synthetase | BGC0001822 | NRP | 26.0 | 22.6 | 201.0 | 1.24e-50 |
| ABX37382.1 | amino\_acid\_adenylation\_domain\_protein | BGC0000984 | NRP+Polyketide | 28.0 | 20.2 | 201.0 | 1.29e-50 |
| AGZ15459.1 | putative\_non-ribosomal\_peptide\_synthetase | BGC0001036 | NRP+Polyketide | 27.0 | 24.6 | 201.0 | 1.32e-50 |
| CZT62794.1 | Non-ribosomal\_peptide\_synthase\_involved\_in\_Hassallidin\_biosynthesis | BGC0001614 | NRP | 25.0 | 22.3 | 201.0 | 1.73e-50 |
| WP\_044618979.1 | non-ribosomal\_peptide\_synthetase | BGC0001791 | NRP | 24.0 | 26.0 | 201.0 | 1.96e-50 |
| OLZ50885.1 | non-ribosomal\_peptide\_synthetase | BGC0001461 | NRP:Glycopeptide | 25.0 | 24.3 | 201.0 | 1.99e-50 |
| AFP87549.1 | NrpS | BGC0001135 | NRP | 26.0 | 25.1 | 201.0 | 2.16e-50 |
| BCD33690.1 | non-ribosomal\_peptide\_synthetase | BGC0002448 | NRP | 26.0 | 26.1 | 201.0 | 2.2e-50 |
| AAG02358.1 | peptide\_synthetase\_NRPS6 | BGC0000963 | NRP:Glycopeptide+Polyketide:Modular type I polyketide+Saccharide:Hybrid/tailoring saccharide | 25.0 | 25.4 | 199.0 | 2.37e-50 |
| OAQ83772.1 | nonribosomal\_peptide\_synthase | BGC0001358 | NRP+Polyketide | 27.0 | 20.3 | 201.0 | 2.39e-50 |
| AAZ55900.1 | non-ribosomal\_peptide\_synthase:Amino\_acid\_adenylation | BGC0000359 | NRP | 27.0 | 25.0 | 200.0 | 2.51e-50 |
| AGI89791.1 | Nonribosomal\_peptide\_synthetase | BGC0001792 | NRP | 27.0 | 24.0 | 200.0 | 2.83e-50 |
| CAJ34374.1 | NRPS\_protein | BGC0000445 | NRP:Cyclic depsipeptide | 26.0 | 25.2 | 200.0 | 2.83e-50 |
| EME52988.1 | amino\_acid\_adenylation\_protein | BGC0001460 | NRP:Glycopeptide | 26.0 | 25.2 | 199.0 | 2.85e-50 |
| AIW82285.1 | PuwH | BGC0001125 | NRP+Polyketide | 26.0 | 26.0 | 198.0 | 2.85e-50 |
| AAQ59905.1 | synthetase\_CbsF | BGC0002680 | NRP | 26.0 | 22.9 | 200.0 | 2.98e-50 |
| RSO11555.1 | non-ribosomal\_peptide\_synthetase | BGC0002637 | NRP | 25.0 | 25.6 | 199.0 | 3.01e-50 |
| CAA11794.1 | PCZA363.3 | BGC0000322 | NRP | 25.0 | 25.2 | 200.0 | 3.12e-50 |
| QGQ63519.1 | nonribosomal\_peptide\_synthetase\_modules\_B | BGC0002548 | NRP | 25.0 | 25.8 | 200.0 | 3.48e-50 |
| CBZ42146.1 | putative\_non-ribosomal\_peptide\_synthetase | BGC0001117 | NRP | 26.0 | 25.5 | 200.0 | 3.61e-50 |
| CAM02313.1 | putative\_non-ribosomal\_peptide\_synthetase | BGC0000349 | NRP | 26.0 | 25.4 | 200.0 | 3.67e-50 |
| OLZ52456.1 | non-ribosomal\_peptide\_synthetase | BGC0001462 | NRP:Glycopeptide | 26.0 | 26.1 | 199.0 | 3.79e-50 |
| AJY78094.1 | nonribosomal\_peptide\_synthetase | BGC0001902 | NRP+Polyketide | 25.0 | 25.6 | 198.0 | 4.07e-50 |
| AAK81826.1 | peptide\_synthetase | BGC0000326 | NRP | 25.0 | 25.7 | 199.0 | 4.68e-50 |
| AEC14348.1 | nonribosomal\_peptide\_synthetase | BGC0000377 | NRP | 22.0 | 25.3 | 199.0 | 4.82e-50 |
| QDJ74273.1 | non-ribosomal\_peptide\_synthetase | BGC0002109 | NRP | 26.0 | 24.8 | 199.0 | 4.83e-50 |
| AKJ15827.1 | peptide\_synthetase | BGC0002735 | Polyketide+NRP | 26.0 | 25.3 | 199.0 | 4.85e-50 |
| AHZ34233.1 | CifB | BGC0000323 | NRP:Lipopeptide | 27.0 | 20.9 | 199.0 | 4.86e-50 |
| QGY73453.1 | Itm21 | BGC0002451 | Polyketide | 27.0 | 21.5 | 199.0 | 5.22e-50 |
| AET98905.1 | putative\_non-ribosomal\_peptide\_synthetase | BGC0000415 | NRP | 25.0 | 23.6 | 199.0 | 5.33e-50 |
| EWS95122.1 | hypothetical\_protein | BGC0000306 | NRP:Lipopeptide | 26.0 | 24.6 | 199.0 | 5.54e-50 |
| QYC40289.1 | A50926\_NRPS,\_modules\_4-5-6 | BGC0002344 | NRP | 25.0 | 25.0 | 199.0 | 5.84e-50 |
| WP\_051872436.1 | non-ribosomal\_peptide\_synthetase | BGC0001771 | NRP | 26.0 | 20.9 | 196.0 | 6.79e-50 |
| BAI63289.1 | putative\_non-ribosomal\_peptide\_synthetase | BGC0000434 | NRP | 27.0 | 22.5 | 199.0 | 6.98e-50 |
| ANS62966.1 | non-ribosomal\_peptide\_synthetase | BGC0001567 | NRP | 26.0 | 25.3 | 197.0 | 7.09e-50 |
| ORC16618.1 | hypothetical\_protein | BGC0001341 | NRP | 25.0 | 25.1 | 199.0 | 7.15e-50 |
| AXG47007.1 | non-ribosomal\_peptide\_synthetase | BGC0000383 | NRP+Polyketide:Modular type I polyketide | 26.0 | 23.9 | 196.0 | 7.41e-50 |
| OKA09424.1 | non-ribosomal\_peptide\_synthetase | BGC0001459 | NRP:Glycopeptide | 25.0 | 25.4 | 198.0 | 9.97e-50 |
| ADH04678.1 | non-ribosomal\_peptide\_synthetase | BGC0001344 | NRP+Polyketide | 26.0 | 21.8 | 197.0 | 1.14e-49 |
| CBJ90358.1 | putative\_Peptide\_synthetase | BGC0000465 | NRP | 24.0 | 22.6 | 197.0 | 1.19e-49 |
| CBF87069.1 | nonribosomal\_peptide\_synthase,\_putative\_(Eurofung) | BGC0001290 | NRP | 24.0 | 25.1 | 198.0 | 1.46e-49 |
| AFY58521.1 | amino\_acid\_adenylation\_enzyme/thioester\_reductase\_family\_protein | BGC0002411 | NRP+Polyketide | 25.0 | 25.3 | 197.0 | 1.52e-49 |
| WP\_050383084.1 | non-ribosomal\_peptide\_synthetase | BGC0001451 | NRP | 27.0 | 23.3 | 197.0 | 1.83e-49 |
| AFH75320.1 | nonribosomal\_peptide\_synthetase | BGC0000425 | NRP:Cyclic depsipeptide | 25.0 | 25.4 | 197.0 | 1.89e-49 |
| AHZ34232.1 | CifA | BGC0000323 | NRP:Lipopeptide | 26.0 | 25.2 | 197.0 | 2.04e-49 |
| AZH23822.1 | MgiJ | BGC0001971 | NRP+Polyketide | 25.0 | 22.7 | 196.0 | 2.08e-49 |
| CAG15012.1 | peptide\_synthetase,\_module\_7 | BGC0000441 | NRP | 26.0 | 25.3 | 196.0 | 3.27e-49 |
| CAA11796.1 | PCZA363.5 | BGC0000322 | NRP | 25.0 | 24.9 | 196.0 | 4.28e-49 |
| AFY58522.1 | non-ribosomal\_peptide\_synthase/amino\_acid\_adenylation\_enzyme | BGC0002411 | NRP+Polyketide | 23.0 | 25.0 | 195.0 | 4.41e-49 |
| AVV61987.1 | putative\_non-ribosomal\_peptide\_synthetase | BGC0001477 | NRP+Polyketide:Modular type I polyketide | 27.0 | 26.1 | 194.0 | 4.43e-49 |
| ACC81022.1 | non-ribosomal\_peptide\_synthetase | BGC0001479 | NRP | 24.0 | 25.4 | 194.0 | 5.39e-49 |
| CAE53353.1 | non-ribosomal\_peptide\_synthetase | BGC0000440 | NRP:Glycopeptide | 25.0 | 25.5 | 195.0 | 5.62e-49 |
| APZ78756.1 | nonribosomal\_peptide\_synthetase | BGC0001423 | NRP:Cyclic depsipeptide+Polyketide:Iterative type I polyketide | 26.0 | 25.2 | 196.0 | 6.16e-49 |
| AEI70245.1 | nonribosomal\_peptide\_synthetase\_NRPS | BGC0000401 | NRP | 27.0 | 20.5 | 195.0 | 6.76e-49 |
| CAL80824.1 | NRPS\_module\_protein | BGC0000997 | NRP+Polyketide | 25.0 | 23.5 | 193.0 | 7.3e-49 |
| AQZ26587.1 | obafluorin\_dimodular\_nonribosomal\_peptide\_synthetase | BGC0001437 | NRP | 25.0 | 26.1 | 195.0 | 7.52e-49 |
| AKC91857.1 | nonribosomal\_peptide\_synthetase | BGC0001414 | NRP | 25.0 | 25.2 | 194.0 | 7.77e-49 |
| AAT09804.1 | NocA | BGC0000395 | NRP | 27.0 | 24.9 | 195.0 | 8.27e-49 |
| ATY37590.1 | BogC | BGC0001532 | NRP | 22.0 | 23.5 | 195.0 | 8.81e-49 |
| AGD80623.1 | non-ribosomal\_peptide\_synthetase | BGC0000394 | NRP | 26.0 | 24.8 | 195.0 | 1.01e-48 |
| ANG60381.1 | nonribosomal\_peptide\_synthetase\_BudC | BGC0001434 | NRP | 24.0 | 22.7 | 194.0 | 1.03e-48 |
| AXN93603.1 | PuwH | BGC0001952 | NRP | 24.0 | 26.6 | 193.0 | 1.1e-48 |
| AGN74885.1 | nonribosomal\_peptide\_synthetase | BGC0000459 | NRP:Cyclic depsipeptide+Polyketide:Trans-AT type I polyketide | 27.0 | 24.4 | 195.0 | 1.21e-48 |
| BAE98155.1 | putative\_non-ribosomal\_peptide\_synthetase | BGC0000339 | NRP | 27.0 | 23.7 | 194.0 | 1.22e-48 |
| CAJ77715.1 | Mps1\_protein | BGC0000364 | NRP | 26.0 | 23.1 | 194.0 | 1.38e-48 |
| WP\_054234643.1 | non-ribosomal\_peptide\_synthetase | BGC0002014 | NRP+Polyketide | 27.0 | 22.6 | 194.0 | 1.43e-48 |
| ADC79642.1 | TamD | BGC0001052 | NRP+Polyketide:Modular type I polyketide | 26.0 | 24.4 | 192.0 | 1.47e-48 |
| ABV56588.1 | KtzH | BGC0000378 | NRP | 26.0 | 20.5 | 194.0 | 1.55e-48 |
| ABS74179.1 | bacillomycin\_D\_synthetase\_C | BGC0001090 | Polyketide+NRP:Lipopeptide | 23.0 | 25.0 | 194.0 | 1.6e-48 |
| AIZ66879.1 | nonribosomal\_peptide\_synthetase | BGC0002666 | NRP+Alkaloid | 25.0 | 26.5 | 194.0 | 1.67e-48 |
| AFK57214.1 | DidC | BGC0000985 | Polyketide+NRP:Cyclic depsipeptide | 27.0 | 24.3 | 193.0 | 1.77e-48 |
| WP\_082191961.1 | non-ribosomal\_peptide\_synthetase | BGC0001451 | NRP | 25.0 | 25.8 | 194.0 | 2.24e-48 |
| ABE35422.1 | Non-ribosomal\_peptide\_synthase | BGC0002421 | NRP | 26.0 | 24.2 | 194.0 | 2.29e-48 |
| WP\_013184322.1 | non-ribosomal\_peptide\_synthetase | BGC0001692 | NRP | 25.0 | 22.2 | 192.0 | 2.58e-48 |
| AAF99707.2 | syringopeptin\_synthetase | BGC0000438 | NRP | 28.0 | 20.7 | 194.0 | 2.66e-48 |
| NAO96317.1 | amino\_acid\_adenylation\_domain-containing\_protein | BGC0002117 | NRP | 25.0 | 25.4 | 191.0 | 2.67e-48 |
| ACS20359.1 | amino\_acid\_adenylation\_domain\_protein | BGC0002420 | NRP+Polyketide | 26.0 | 25.4 | 193.0 | 2.75e-48 |
| AAK81825.1 | peptide\_synthetase | BGC0000326 | NRP | 26.0 | 25.5 | 192.0 | 2.96e-48 |
| AZM57023.1 | non-ribosomal\_peptide\_synthetase | BGC0002314 | NRP | 25.0 | 26.1 | 192.0 | 2.98e-48 |
| ABM21572.1 | crpD | BGC0000975 | NRP+Polyketide | 24.0 | 26.3 | 193.0 | 3.06e-48 |
| ABW17375.1 | PsoA | BGC0000411 | NRP | 25.0 | 25.2 | 193.0 | 3.15e-48 |
| CAC48362.1 | peptide\_synthetase | BGC0000311 | NRP | 26.0 | 26.3 | 192.0 | 4.84e-48 |
| UMM61373.1 | Tsk12 | BGC0002661 | NRP | 27.0 | 24.7 | 191.0 | 5.01e-48 |
| OLZ50886.1 | non-ribosomal\_peptide\_synthetase | BGC0001461 | NRP:Glycopeptide | 26.0 | 26.3 | 192.0 | 6.35e-48 |
| AYA22333.1 | KerD | BGC0001955 | NRP | 26.0 | 26.3 | 192.0 | 6.35e-48 |
| NHN68324.1 | amino\_acid\_adenylation\_domain-containing\_protein | BGC0002719 | NRP | 26.0 | 25.3 | 192.0 | 6.71e-48 |
| AAK81827.1 | peptide\_synthetase | BGC0000326 | NRP | 25.0 | 26.2 | 192.0 | 7.16e-48 |
| QBC75021.1 | non-ribosomal\_peptide\_synthetase | BGC0001968 | NRP | 26.0 | 25.5 | 192.0 | 8.18e-48 |
| AFK57215.1 | DidD | BGC0000985 | Polyketide+NRP:Cyclic depsipeptide | 27.0 | 21.0 | 192.0 | 9.37e-48 |
| CAD91212.1 | putative\_non-ribosomal\_peptide\_synthetase,\_modules\_4-6 | BGC0000289 | NRP:Glycopeptide+Saccharide:Hybrid/tailoring saccharide | 25.0 | 25.1 | 192.0 | 9.53e-48 |
| QRD93053.1 | putative\_nonribosomal\_peptide\_synthase | BGC0002160 | NRP | 24.0 | 21.1 | 192.0 | 1.08e-47 |
| ATY72525.1 | non-ribosomal\_peptide\_synthetase | BGC0001574 | NRP | 25.0 | 28.3 | 190.0 | 1.35e-47 |
| CAA11795.1 | PCZA363.4 | BGC0000322 | NRP | 25.0 | 24.5 | 191.0 | 1.63e-47 |
| AXN93616.1 | PuwH | BGC0001953 | NRP | 24.0 | 26.6 | 189.0 | 1.73e-47 |
| CAQ71828.1 | non\_ribosomal\_peptide\_synthase,\_antibiotic\_synthesis;\_contains\_3\_condensation\_domains,\_2\_AMP-acid\_ligases\_II\_domains,\_2\_PP-binding,\_Phosphopantetheine\_attachment\_site | BGC0001189 | NRP | 26.0 | 25.2 | 191.0 | 1.79e-47 |
| AAN32981.1 | BarG | BGC0000962 | NRP+Polyketide:Modular type I polyketide | 23.0 | 22.3 | 191.0 | 1.88e-47 |
| AAL06699.1 | polyketide\_synthase | BGC0000965 | Polyketide:Iterative type I polyketide+Polyketide:Enediyne type I polyketide | 26.0 | 20.0 | 190.0 | 1.93e-47 |
| ALU98461.1 | erythronolide\_synthase | BGC0001397 | NRP+Polyketide | 26.0 | 20.0 | 190.0 | 1.93e-47 |
| CAK15814.1 | putative\_non-ribosomal\_peptide\_synthetase,\_terminal\_component | BGC0000344 | NRP | 27.0 | 20.8 | 191.0 | 2.22e-47 |
| CAB53322.1 | putative\_peptide\_synthetase | BGC0000325 | NRP | 25.0 | 26.4 | 190.0 | 2.69e-47 |
| AEI58866.1 | peptide\_synthetase | BGC0000455 | NRP | 26.0 | 24.5 | 190.0 | 2.78e-47 |
| AEW31021.1 | plipastatin\_synthetase | BGC0000407 | NRP | 24.0 | 26.3 | 190.0 | 3.04e-47 |
| AIG79240.1 | Hypothetical\_protein | BGC0000419 | Saccharide+NRP:Glycopeptide | 26.0 | 26.4 | 189.0 | 3.18e-47 |
| ABC36450.1 | peptide\_synthetase-like\_protein | BGC0000386 | NRP:NRP siderophore | 25.0 | 26.1 | 190.0 | 3.4e-47 |
| AAS92545.1 | SirP | BGC0001044 | NRP | 25.0 | 24.3 | 189.0 | 4.73e-47 |
| AAM80537.1 | StaC | BGC0000290 | NRP:Glycopeptide | 26.0 | 26.3 | 189.0 | 4.76e-47 |
| CAC48361.1 | peptide\_synthetase | BGC0000311 | NRP | 25.0 | 24.3 | 189.0 | 4.76e-47 |
| BAE98156.1 | putative\_non-ribosomal\_peptide\_synthetase | BGC0000339 | NRP | 25.0 | 22.8 | 189.0 | 5.71e-47 |
| AHZ34238.1 | CipA | BGC0001389 | NRP | 25.0 | 25.4 | 189.0 | 6.51e-47 |
| AQZ69229.1 | hypothetical\_protein | BGC0001635 | NRP+Polyketide | 24.0 | 25.0 | 188.0 | 6.82e-47 |
| AXG46165.1 | non-ribosomal\_peptide\_synthetase | BGC0002713 | NRP | 24.0 | 23.1 | 188.0 | 7.64e-47 |
| CZT62792.1 | non-ribosomal\_peptide\_synthase\_involved\_in\_Hassallidin\_biosynthesis | BGC0001614 | NRP | 23.0 | 25.2 | 188.0 | 8.02e-47 |
| ABP57749.1 | DepE | BGC0000993 | NRP:Cyclic depsipeptide+Polyketide:Modular type I polyketide | 26.0 | 24.8 | 188.0 | 9.53e-47 |
| CAC48360.1 | peptide\_synthetase | BGC0000311 | NRP | 25.0 | 25.2 | 188.0 | 9.8e-47 |
| AZH23792.1 | MgcJ | BGC0001970 | NRP+Polyketide | 23.0 | 22.4 | 187.0 | 1.04e-46 |
| CEK23367.1 | putative\_Ornithine\_racemase | BGC0001716 | NRP | 24.0 | 26.3 | 188.0 | 1.21e-46 |
| AIE77060.1 | peptide\_synthetase\_module\_7 | BGC0000418 | NRP | 26.0 | 26.2 | 187.0 | 1.22e-46 |
| EME52989.1 | amino\_acid\_adenylation\_protein | BGC0001460 | NRP:Glycopeptide | 25.0 | 24.8 | 188.0 | 1.39e-46 |
| APZ78809.1 | nonribosomal\_peptide\_synthetase | BGC0001428 | NRP:Cyclic depsipeptide+Polyketide:Iterative type I polyketide | 25.0 | 25.2 | 188.0 | 1.42e-46 |
| CAE15497.1 |  | BGC0002286 | NRP | 27.0 | 25.9 | 188.0 | 1.49e-46 |
| ADN26248.1 | peptide\_synthetase | BGC0000951 | NRP | 27.0 | 20.3 | 184.0 | 1.57e-46 |
| ADJ63842.1 | Serobactin\_synthetase | BGC0000424 | NRP:NRP siderophore | 26.0 | 25.2 | 188.0 | 1.6e-46 |
| QNL14921.1 | AptB | BGC0002512 | NRP | 24.0 | 25.0 | 185.0 | 2.29e-46 |
| BAC67534.2 | arthrofactin\_synthetase\_A | BGC0000305 | NRP:Lipopeptide | 25.0 | 25.2 | 187.0 | 2.35e-46 |
| ABS74207.1 | fengycin\_synthetase\_C | BGC0001095 | NRP | 25.0 | 26.1 | 187.0 | 2.6e-46 |
| CAF05647.1 | TubB\_protein | BGC0001053 | NRP+Polyketide | 25.0 | 22.4 | 186.0 | 2.96e-46 |
| ABL74936.1 | NRPS | BGC0001048 | NRP:Glycopeptide+Polyketide:Modular type I polyketide+Saccharide:Hybrid/tailoring saccharide | 23.0 | 25.9 | 186.0 | 3.06e-46 |
| CUX79060.1 | Octapeptin\_synthase\_subunit\_A | BGC0001715 | NRP | 25.0 | 20.3 | 187.0 | 3.11e-46 |
| ADL64235.1 | aureusimine\_non-ribosomal\_peptide\_synthetase | BGC0000308 | NRP | 23.0 | 28.0 | 186.0 | 3.3e-46 |
| ABS74209.1 | fengycin\_synthetase\_A | BGC0001095 | NRP | 25.0 | 23.9 | 186.0 | 3.41e-46 |
| BAH04161.1 | putative\_non-ribosomal\_peptide\_synthetase | BGC0000450 | NRP | 25.0 | 23.1 | 186.0 | 3.74e-46 |
| ABA70582.1 | alpha-aminoadypil-cysteinyl-valine\_synthetase | BGC0000404 | NRP | 23.0 | 26.3 | 186.0 | 3.98e-46 |
| ctg1\_orf20 |  | BGC0001767 | NRP | 25.0 | 26.9 | 186.0 | 4.3e-46 |
| AIG79241.1 | Hypothetical\_protein | BGC0000419 | Saccharide+NRP:Glycopeptide | 26.0 | 25.3 | 186.0 | 6.91e-46 |
| AJM89738.1 | PmxE | BGC0001192 | NRP | 23.0 | 22.4 | 186.0 | 7.61e-46 |
| ABR12615.1 | ACV\_synthetase | BGC0000405 | NRP:Beta-lactam | 23.0 | 26.3 | 185.0 | 8.9e-46 |
| QOE83923.1 | tyrocidine\_synthase\_3 | BGC0002051 | NRP | 25.0 | 26.5 | 183.0 | 8.92e-46 |
| BCD33691.1 | non-ribosomal\_peptide\_synthetase | BGC0002448 | NRP | 25.0 | 26.4 | 184.0 | 8.95e-46 |
| DAB41479.1 | nonribosomal\_peptide\_synthetase | BGC0001230 | NRP:Cyclic depsipeptide+Polyketide:Modular type I polyketide | 25.0 | 24.9 | 184.0 | 9.03e-46 |
| ACA97580.1 | PmxE | BGC0000408 | NRP | 24.0 | 22.6 | 185.0 | 9.95e-46 |
| AGI87382.1 | Peptide\_synthase | BGC0002358 | Polyketide | 27.0 | 23.0 | 185.0 | 1.13e-45 |
| AAC82550.1 | FxbC | BGC0000351 | NRP | 25.0 | 25.2 | 185.0 | 1.25e-45 |
| CAJ34375.1 | NRPS | BGC0000445 | NRP:Cyclic depsipeptide | 25.0 | 23.1 | 184.0 | 1.43e-45 |
| QEO74905.1 | condensation\_domain-containing\_protein | BGC0002588 | Other | 25.0 | 20.3 | 184.0 | 1.49e-45 |
| UMM61372.1 | Tsk11 | BGC0002661 | NRP | 26.0 | 26.5 | 184.0 | 1.51e-45 |
| CEK23365.1 | conserved\_hypothetical\_protein | BGC0001716 | NRP | 24.0 | 24.8 | 182.0 | 1.92e-45 |
| ABK36076.1 | nonribosomal\_peptide\_synthetase | BGC0001502 | NRP | 25.0 | 26.2 | 184.0 | 1.97e-45 |
| AZM57024.1 | non-ribosomal\_peptide\_synthetase | BGC0002314 | NRP | 26.0 | 21.8 | 184.0 | 2.1e-45 |
| SJZ83675.1 | non-ribosomal\_peptide\_synthase\_domain\_TIGR01720/amino\_acid\_adenylation\_domain-containing\_protein/thioester\_reductase\_domain-containing\_protein | BGC0002660 | NRP | 24.0 | 23.4 | 184.0 | 2.21e-45 |
| CAM59606.1 | non-ribosomal\_peptide\_synthetase | BGC0000297 | NRP:Glycopeptide+Polyketide:Other polyketide+Saccharide:Hybrid/tailoring saccharide | 24.0 | 26.2 | 182.0 | 3.58e-45 |
| RSO11554.1 | non-ribosomal\_peptide\_synthetase | BGC0002637 | NRP | 25.0 | 24.6 | 183.0 | 3.64e-45 |
| ABV79986.1 | ApnB | BGC0000301 | NRP | 24.0 | 25.0 | 181.0 | 3.7e-45 |
| ACG60761.1 | NRPS(C/A) | BGC0001058 | NRP:Glycopeptide+Polyketide:Modular type I polyketide+Saccharide:Hybrid/tailoring saccharide | 25.0 | 26.4 | 181.0 | 4.36e-45 |
| AGN74886.1 | nonribosomal\_peptide\_synthetase | BGC0000459 | NRP:Cyclic depsipeptide+Polyketide:Trans-AT type I polyketide | 25.0 | 25.6 | 182.0 | 4.51e-45 |
| BAI63288.1 | putative\_non-ribosomal\_peptide\_synthetase | BGC0000434 | NRP | 25.0 | 23.5 | 182.0 | 5.04e-45 |
| WP\_080679150.1 | non-ribosomal\_peptide\_synthetase | BGC0001228 | NRP:Cyclic depsipeptide | 25.0 | 23.7 | 182.0 | 6.58e-45 |
| KUM80514.1 | hypothetical\_protein | BGC0001562 | NRP | 26.0 | 25.5 | 180.0 | 9.24e-45 |
| ABP57748.1 | DepD | BGC0000993 | NRP:Cyclic depsipeptide+Polyketide:Modular type I polyketide | 25.0 | 25.0 | 182.0 | 9.27e-45 |
| AFY58519.1 | amino\_acid\_adenylation\_enzyme/thioester\_reductase\_family\_protein,thioester\_reductase-like\_protein | BGC0002411 | NRP+Polyketide | 23.0 | 26.0 | 181.0 | 9.39e-45 |
| QKM21619.1 | non-ribosomal\_peptide\_synthetase | BGC0002351 | NRP | 25.0 | 25.4 | 182.0 | 9.44e-45 |
| QBQ12464.1 | amino\_acid\_adenylation\_domain-containing\_protein | BGC0002693 | NRP | 25.0 | 25.8 | 182.0 | 9.87e-45 |
| PVC99865.1 | non-ribosomal\_peptide\_synthetase | BGC0002100 | NRP+Other | 25.0 | 25.0 | 182.0 | 9.91e-45 |
| CAD91220.1 | putative\_non-ribosomal\_peptide\_synthetase,\_modules\_1-2 | BGC0000289 | NRP:Glycopeptide+Saccharide:Hybrid/tailoring saccharide | 25.0 | 25.2 | 181.0 | 1e-44 |
| QNH67552.1 | Cip24 | BGC0002108 | NRP | 26.0 | 25.4 | 181.0 | 1.05e-44 |
| AEZ51520.1 | pmxE | BGC0001153 | NRP:Lipopeptide | 24.0 | 22.7 | 181.0 | 1.45e-44 |
| BAX90000.1 | Non-ribosomal\_peptide\_synthetase | BGC0001628 | NRP | 26.0 | 25.5 | 181.0 | 1.71e-44 |
| KZM69124.1 | non-ribosomal\_peptide\_synthetase | BGC0002352 | Other | 26.0 | 24.6 | 180.0 | 1.74e-44 |
| BBA21073.1 | putative\_non-ribosomal\_peptide\_synthetase | BGC0001740 | NRP+Polyketide | 26.0 | 21.9 | 181.0 | 2.18e-44 |
| AHZ20781.1 | non-ribosomal\_peptide\_synthase | BGC0000369 | NRP+Saccharide:Hybrid/tailoring saccharide | 23.0 | 26.1 | 180.0 | 2.28e-44 |
| AIE77059.1 | peptide\_synthetase | BGC0000418 | NRP | 25.0 | 25.1 | 180.0 | 2.95e-44 |
| ABC36785.1 | peptide\_synthetase,\_putative | BGC0000964 | NRP:Cyclic depsipeptide+Polyketide:Trans-AT type I polyketide | 25.0 | 26.1 | 180.0 | 3.76e-44 |
| ABI22131.1 | putative\_non-ribosomal\_peptide\_synthetase | BGC0000422 | NRP | 26.0 | 24.1 | 179.0 | 4.56e-44 |
| CEK23605.1 | Non-ribosomal\_peptide\_synthase\_involved\_in\_xenematides\_synthesis | BGC0001825 | NRP | 24.0 | 22.9 | 179.0 | 5.3e-44 |
| AAZ55898.1 | amino\_acid\_adenylation | BGC0000359 | NRP | 26.0 | 25.7 | 178.0 | 5.47e-44 |
| ABS74205.1 | fengycin\_synthetase\_E | BGC0001095 | NRP | 23.0 | 26.2 | 178.0 | 6.48e-44 |
| AHD05627.1 | putative\_non-ribosomal\_peptide\_ligase\_domain\_protein | BGC0001033 | NRP+Polyketide | 23.0 | 26.0 | 177.0 | 1.09e-43 |
| KJY94240.1 | peptide\_synthetase | BGC0002691 | NRP | 23.0 | 26.1 | 178.0 | 1.1e-43 |
| EAL92291.2 | nonribosomal\_peptide\_synthtease | BGC0000372 | NRP | 23.0 | 24.8 | 178.0 | 1.16e-43 |
| ABB90279.1 | non-ribosomal\_peptide\_synthetase | BGC0001057 | NRP+Polyketide | 25.0 | 22.4 | 177.0 | 1.57e-43 |
| AMK48228.1 | nonribosomal\_peptide\_synthetase | BGC0001351 | NRP | 26.0 | 26.3 | 175.0 | 2.83e-43 |
| AAL33756.1 | putative\_non-ribosomal\_peptide\_synthetase | BGC0000421 | NRP | 24.0 | 25.1 | 175.0 | 3.12e-43 |
| CAE02630.1 | surfactin\_synthetase\_A | BGC0000433 | NRP:Lipopeptide | 24.0 | 20.5 | 177.0 | 3.2e-43 |
| EDY47118.1 | N-(5-amino-5-carboxypentanoyl)-L-cysteinyl-D-\_valine\_synthase | BGC0000319 | NRP:Beta-lactam | 25.0 | 24.9 | 177.0 | 3.24e-43 |
| AIE77058.1 | peptide\_synthetase\_module\_3 | BGC0000418 | NRP | 24.0 | 25.5 | 175.0 | 3.63e-43 |
| BAI23334.1 | putative\_non-ribosomal\_peptide\_synthetase | BGC0000949 | NRP | 24.0 | 25.9 | 175.0 | 4.04e-43 |
| OAL11435.1 | non-ribosomal\_peptide\_synthetase | BGC0001570 | NRP | 25.0 | 25.7 | 175.0 | 4.37e-43 |
| QDQ83032.1 | amino\_acid\_adenylation\_domain-containing\_protein | BGC0002564 | NRP | 24.0 | 25.5 | 176.0 | 4.59e-43 |
| CDF96614.1 | NRPS | BGC0001149 | NRP:Lipopeptide+Saccharide:Hybrid/tailoring saccharide | 24.0 | 26.5 | 176.0 | 6.14e-43 |
| OLZ52457.1 | non-ribosomal\_peptide\_synthetase | BGC0001462 | NRP:Glycopeptide | 24.0 | 24.5 | 176.0 | 7.4e-43 |
| AXF16146.1 | non-ribosomal\_peptide\_synthetase | BGC0002563 | NRP | 25.0 | 25.5 | 176.0 | 7.92e-43 |
| AFD30953.1 | CrmB | BGC0000966 | NRP+Polyketide | 25.0 | 25.8 | 174.0 | 8.31e-43 |
| AJV88376.1 | MfnD | BGC0001214 | NRP | 25.0 | 24.4 | 174.0 | 1.28e-42 |
| ABY66004.1 | type\_II\_beta-Tyr\_adenylation\_domain\_protein | BGC0001008 | Polyketide:Iterative type I polyketide+Polyketide:Enediyne type I polyketide | 25.0 | 25.0 | 173.0 | 1.29e-42 |
| AKP45399.1 | CysK | BGC0001413 | NRP | 26.0 | 20.8 | 175.0 | 1.3e-42 |
| QDF82254.1 | non-ribosomal\_peptide\_synthetase | BGC0001980 | NRP | 25.0 | 25.2 | 174.0 | 1.68e-42 |
| TRX17524.1 | amino\_acid\_adenylation\_domain-containing\_protein | BGC0002329 | NRP | 25.0 | 26.3 | 174.0 | 1.75e-42 |
| AGA37269.1 | NRPS | BGC0000819 | NRP+Alkaloid | 24.0 | 28.0 | 174.0 | 1.81e-42 |
| BAX64246.1 | NRPS | BGC0001623 | NRP+Polyketide | 24.0 | 25.5 | 174.0 | 1.89e-42 |
| QWT72279.1 | non-ribosomal\_peptide\_synthetase | BGC0002430 | NRP+Saccharide | 25.0 | 25.5 | 174.0 | 2.44e-42 |
| EPH46597.1 | putative\_Linear\_gramicidin\_synthase\_subunit\_C | BGC0001519 | NRP+Polyketide | 24.0 | 25.0 | 173.0 | 3.17e-42 |
| AAM80538.1 | StaB | BGC0000290 | NRP:Glycopeptide | 24.0 | 26.0 | 172.0 | 3.65e-42 |
| WP\_051462298.1 | non-ribosomal\_peptide\_synthetase | BGC0001873 | NRP:Lipopeptide | 25.0 | 20.3 | 173.0 | 4.08e-42 |
| BCJ07531.1 | hypothetical\_protein | BGC0002379 | NRP | 26.0 | 23.4 | 171.0 | 4.87e-42 |
| CDE97356.1 | plipastatin\_synthase\_subunit\_C | BGC0001686 | NRP | 22.0 | 24.9 | 172.0 | 6.96e-42 |
| QDJ74274.1 | non-ribosomal\_peptide\_synthetase | BGC0002109 | NRP | 26.0 | 22.5 | 171.0 | 7.27e-42 |
| ACZ55943.1 | non-ribosomal\_peptide\_synthetase | BGC0000302 | NRP | 24.0 | 25.1 | 171.0 | 7.87e-42 |
| QCC62999.1 | BII-rafflesfungin\_nonribosomal\_protein\_synthetase | BGC0001966 | NRP+Polyketide | 25.0 | 23.4 | 172.0 | 9.51e-42 |
| CAG15010.1 | peptide\_synthetase,\_module\_3 | BGC0000441 | NRP | 25.0 | 25.8 | 170.0 | 1.33e-41 |
| ACG60782.1 | NRPS(C/A/PCP/C/A/PCP) | BGC0001058 | NRP:Glycopeptide+Polyketide:Modular type I polyketide+Saccharide:Hybrid/tailoring saccharide | 24.0 | 26.3 | 171.0 | 1.89e-41 |
| ALD82525.1 | non-ribosomal\_peptide\_synthase | BGC0001212 | NRP+Polyketide | 24.0 | 23.0 | 170.0 | 1.92e-41 |
| AMK48225.1 | nonribosomal\_peptide\_synthetase | BGC0001351 | NRP | 27.0 | 20.3 | 169.0 | 2.17e-41 |
| AIG79242.1 | Non-ribosomal\_peptide\_synthetase | BGC0000419 | Saccharide+NRP:Glycopeptide | 25.0 | 25.6 | 168.0 | 3.88e-41 |
| CAN89638.1 | putative\_non-ribosomal\_peptide\_synthetase | BGC0001070 | NRP+Polyketide:Modular type I polyketide+Polyketide:Trans-AT type I polyketide | 24.0 | 26.0 | 167.0 | 6.42e-41 |
| CBF76036.1 | putative\_nonribosomal\_peptide\_synthetase\_(Eurofung) | BGC0001399 | NRP | 25.0 | 24.2 | 169.0 | 7.59e-41 |
| AAX31559.1 | peptide\_synthetase\_3 | BGC0000336 | NRP | 25.0 | 25.6 | 169.0 | 7.68e-41 |
| CCJ67638.1 | TaaC | BGC0000447 | NRP:Lipopeptide | 26.0 | 20.0 | 169.0 | 8.74e-41 |
| CAE53351.1 | non-ribosomal\_peptide\_synthetase | BGC0000440 | NRP:Glycopeptide | 25.0 | 25.8 | 167.0 | 9.08e-41 |
| ATQ39428.1 | cyclosporin\_C\_synthetase | BGC0001565 | NRP | 23.0 | 23.0 | 169.0 | 1.08e-40 |
| ABD65956.1 | nonribosomal\_peptide\_synthetase | BGC0000341 | NRP | 25.0 | 25.2 | 168.0 | 1.23e-40 |
| E0F75\_025360 | amino\_acid\_adenylation\_domain-containing\_protein | BGC0002340 | NRP+Other | 24.0 | 25.2 | 166.0 | 2.22e-40 |
| ABC94347.1 | vicibactin\_biosynthesis\_non-ribosomal\_peptide\_synthase\_protein | BGC0000457 | NRP | 25.0 | 23.8 | 166.0 | 2.4e-40 |
| QCP68971.1 | VatR | BGC0002296 | NRP+Polyketide | 25.0 | 22.4 | 167.0 | 2.58e-40 |
| QYA95682.1 | amino\_acid\_adenylation\_domain-containing\_protein | BGC0002676 | NRP | 25.0 | 22.9 | 166.0 | 4.77e-40 |
| simA |  | BGC0000334 | NRP | 22.0 | 22.9 | 166.0 | 5.4e-40 |
| AQZ69227.1 | hypothetical\_protein | BGC0001635 | NRP+Polyketide | 25.0 | 26.2 | 165.0 | 6.09e-40 |
| CAD91221.1 | putative\_non-ribosomal\_peptide\_synthetase,\_module\_3 | BGC0000289 | NRP:Glycopeptide+Saccharide:Hybrid/tailoring saccharide | 23.0 | 26.1 | 164.0 | 6.2e-40 |
| BAW32333.1 | nonribosomal\_peptide\_synthetase | BGC0001631 | NRP+Polyketide | 25.0 | 25.1 | 166.0 | 6.22e-40 |
| AEA30272.1 | peptide\_synthetase | BGC0000429 | Polyketide+NRP:Cyclic depsipeptide | 25.0 | 22.9 | 166.0 | 6.23e-40 |
| ABA73954.1 | putative\_non-ribosomal\_peptide\_synthetase | BGC0001842 | NRP:Lipopeptide | 23.0 | 25.0 | 166.0 | 6.23e-40 |
| ANZ15840.1 | non-ribosomal\_peptide\_synthase/amino\_acid\_adenylation\_enzyme | BGC0001569 | NRP | 25.0 | 25.2 | 165.0 | 1.06e-39 |
| ADM34138.1 | non-ribosomal\_peptide\_synthetase | BGC0001084 | NRP+Terpene+Alkaloid | 25.0 | 21.0 | 165.0 | 1.09e-39 |
| AFK57220.1 | DidI | BGC0000985 | Polyketide+NRP:Cyclic depsipeptide | 26.0 | 20.2 | 162.0 | 2.23e-39 |
| AJK49766.1 | non-ribosomal\_peptide\_synthase | BGC0002565 | NRP | 24.0 | 25.9 | 164.0 | 2.25e-39 |
| QLY89262.1 | pseudodesmin\_synthetase | BGC0002522 | NRP | 24.0 | 25.1 | 163.0 | 3.13e-39 |
| AFY58523.1 | amino\_acid\_adenylation\_enzyme/thioester\_reductase\_family\_protein | BGC0002411 | NRP+Polyketide | 24.0 | 25.1 | 162.0 | 8.78e-39 |
| ADZ45324.1 | non-ribosomal\_peptide\_synthetase | BGC0001020 | NRP+Polyketide | 25.0 | 24.4 | 159.0 | 2.49e-38 |
| CCJ67646.1 | JagB | BGC0001127 | NRP | 25.0 | 24.8 | 159.0 | 5.25e-38 |
| ESU17760.1 | hypothetical\_protein | BGC0002172 | NRP | 24.0 | 25.4 | 159.0 | 6.56e-38 |
| EAU29302.1 | hypothetical\_protein | BGC0002272 | NRP | 25.0 | 22.3 | 159.0 | 8.23e-38 |
| WP\_064118616.1 | non-ribosomal\_peptide\_synthetase | BGC0002075 | Alkaloid+NRP:Lipopeptide | 24.0 | 25.6 | 157.0 | 1.55e-37 |
| QSV12656.1 | AvmN | BGC0002456 | Polyketide+NRP | 23.0 | 27.1 | 157.0 | 1.62e-37 |
| AGS77308.1 | NRPS\_module\_3 | BGC0001178 | NRP:Glycopeptide | 24.0 | 26.1 | 157.0 | 2.16e-37 |
| AVI26392.1 | putative\_nonribosomal\_peptide\_synthase | BGC0001800 | NRP+Polyketide | 27.0 | 20.9 | 157.0 | 2.74e-37 |
| ABC34305.1 | peptide\_synthetase,\_putative | BGC0000961 | NRP+Polyketide | 24.0 | 24.2 | 154.0 | 1.99e-36 |
| WP\_141576257.1 | non-ribosomal\_peptide\_synthetase | BGC0002686 | NRP | 25.0 | 24.1 | 154.0 | 3.2e-36 |
| WP\_010369425.1 | non-ribosomal\_peptide\_synthetase | BGC0000314 | Polyketide+NRP:Cyclic depsipeptide+Other:Aminocoumarin | 23.0 | 20.5 | 153.0 | 3.56e-36 |
| AYA22335.1 | KerB | BGC0001955 | NRP | 23.0 | 25.6 | 152.0 | 5.02e-36 |
| AAF19811.1 | mtaC | BGC0001024 | NRP+Polyketide:Modular type I polyketide | 24.0 | 20.6 | 149.0 | 3.45e-35 |
| ALV86868.1 | Tlo22 | BGC0001406 | NRP | 23.0 | 23.4 | 150.0 | 4.02e-35 |
| RGP42808.1 | non-ribosomal\_peptide\_synthetase | BGC0002696 | NRP | 25.0 | 23.5 | 150.0 | 4.7e-35 |
| UHJ79951.1 | non-ribosomal\_peptide\_synthetase | BGC0002654 | NRP | 23.0 | 25.4 | 150.0 | 4.92e-35 |
| CBF73453.1 | nonribosomal\_peptide\_synthase,\_putative\_(JCVI) | BGC0001515 | NRP | 24.0 | 23.2 | 149.0 | 6.66e-35 |
| ADH04681.1 | non-ribosomal\_peptide\_synthetase | BGC0001344 | NRP+Polyketide | 24.0 | 26.8 | 148.0 | 6.81e-35 |
| ARS01470.1 | NcmB | BGC0001702 | NRP+Polyketide | 25.0 | 21.0 | 148.0 | 8.28e-35 |
| AQZ71347.1 | hypothetical\_protein | BGC0001635 | NRP+Polyketide | 25.0 | 25.0 | 148.0 | 1.18e-34 |
| QYC40288.1 | A50926\_NRPS,\_module\_3 | BGC0002344 | NRP | 24.0 | 26.2 | 147.0 | 1.76e-34 |
| BAD55612.1 | non-ribosomal\_peptide\_synthetase | BGC0001027 | NRP+Polyketide | 25.0 | 24.8 | 144.0 | 1.35e-33 |
| QRD90553.1 | non-ribosomal\_peptide\_synthetase\_module | BGC0002157 | NRP+Alkaloid | 25.0 | 20.4 | 142.0 | 8.93e-33 |
| EGX96627.1 | non-ribosomal\_peptide\_synthase,\_putative | BGC0002259 | Polyketide+NRP | 23.0 | 25.2 | 142.0 | 1.02e-32 |
| CAJ87590.1 | putative\_peptide\_synthase | BGC0001055 | NRP+Polyketide | 25.0 | 22.5 | 142.0 | 1.06e-32 |
| ALK21569.1 | non-ribosomal\_peptide\_synthetase | BGC0002678 | NRP | 25.0 | 20.9 | 140.0 | 1.95e-32 |
| CBF76038.1 | nonribosomal\_peptide\_synthase,\_putative\_(Eurofung) | BGC0001399 | NRP | 25.0 | 24.9 | 140.0 | 1.96e-32 |
| AFR69331.1 | nonribosomal\_peptide\_synthetase\_SpiA | BGC0001045 | NRP:Cyclic depsipeptide+Polyketide:Modular type I polyketide | 25.0 | 20.9 | 138.0 | 1.07e-31 |
| EJP62835.1 | nonribosomal\_peptide\_synthase,\_putative | BGC0002203 | NRP+Polyketide+Other | 24.0 | 20.3 | 138.0 | 1.96e-31 |
| QPI18726.1 | nonribosomal\_peptide\_synthetase | BGC0002125 | NRP:Cyclic depsipeptide | 24.0 | 26.0 | 136.0 | 3.4e-31 |
| AUS29484.1 | non-ribosomal\_peptide\_synthetase | BGC0002605 | NRP+Polyketide | 24.0 | 22.6 | 136.0 | 4.39e-31 |
| AAO56106.1 | yersiniabactin\_non-ribosomal\_peptide\_synthetase | BGC0002570 | NRP+Polyketide | 24.0 | 22.2 | 136.0 | 5.99e-31 |
| BAH33409.1 | putative\_non-ribosomal\_peptide\_synthetase | BGC0000371 | NRP | 23.0 | 23.4 | 136.0 | 7.3e-31 |
| QPI18723.1 | nonribosomal\_peptide\_synthetase | BGC0002125 | NRP:Cyclic depsipeptide | 24.0 | 23.6 | 134.0 | 2.23e-30 |
| CCA53799.1 | iron\_aquisition\_yersiniabactin\_synthesis\_enzyme | BGC0001801 | NRP | 24.0 | 20.8 | 130.0 | 2.21e-29 |
| WP\_002308266.1 | NRPS,\_A-domain\_Leucine | BGC0002058 | Polyketide+NRP | 22.0 | 22.9 | 129.0 | 5.94e-29 |
| QCY50741.1 | non-ribosomal\_peptide\_synthetase | BGC0002287 | NRP | 22.0 | 22.9 | 129.0 | 5.94e-29 |
| ABL74940.1 | NRPS | BGC0001048 | NRP:Glycopeptide+Polyketide:Modular type I polyketide+Saccharide:Hybrid/tailoring saccharide | 23.0 | 23.3 | 125.0 | 1.22e-27 |
| ARB50207.1 | lysergyl\_peptide\_synthetase\_21 | BGC0001573 | Alkaloid | 24.0 | 20.2 | 123.0 | 6.46e-27 |
| AHZ34242.1 | CipE | BGC0001389 | NRP | 24.0 | 20.6 | 115.0 | 1.75e-24 |
| CCP45167.1 | Peptide\_synthetase\_MbtF\_(peptide\_synthase) | BGC0001021 | NRP+Polyketide | 24.0 | 22.0 | 112.0 | 6.85e-24 |
| AFO59871.1 | three-domain\_carboxylic\_acid\_reductase | BGC0000175 | Polyketide:Trans-AT type I polyketide | 24.0 | 21.5 | 74.0 | 4.11e-12 |
