# Supplementary material for: Alkaliphilic/Alkali-Tolerant Fungi: Molecular, Biochemical, and Biotechnological Aspects
Source: J Fungi (Basel). 2023 Jun 9;9(6):652. doi: 10.3390/jof9060652 (PMC10301932; doi:10.3390/jof9060652)
Supplement: Supplementary file 1 [file jof-09-00652-s001.zip › S2/knownclusterblast/region1/input.path1.gene21_mibig_hits.html]

| MIBiG Protein | Description | MIBiG Cluster | MiBiG Product | % ID | % Coverage | BLAST Score | E-value |
| --- | --- | --- | --- | --- | --- | --- | --- |
| CCT72378.1 | related\_to\_C.carbonum\_toxD\_protein | BGC0001305 | Polyketide | 46.0 | 61.7 | 307.0 | 4.12e-100 |
| BCA42569.1 | trans-acting\_enoylreductase\_GrgB | BGC0002185 | Polyketide | 43.0 | 62.0 | 281.0 | 3.52e-90 |
| EED49870.1 | alcohol\_dehydrogenase,\_putative | BGC0001445 | NRP+Polyketide:Iterative type I polyketide | 40.0 | 62.4 | 251.0 | 1.1e-77 |
| KKP04596.1 | trans\_enoyl-reductase | BGC0002066 | NRP+Polyketide:Iterative type I polyketide | 40.0 | 62.0 | 248.0 | 2.26e-77 |
| QBQ83710.1 | trans-enoyl\_reductase | BGC0002093 | Polyketide+NRP | 39.0 | 64.7 | 244.0 | 1.31e-75 |
| QBK15055.1 | enoyl\_reductase\_TraG | BGC0002197 | Polyketide+NRP | 39.0 | 63.3 | 242.0 | 1.02e-74 |
| ARP51714.1 | NADP-dependent\_dehydrogenase\_/enoyl-reductase | BGC0001741 | NRP+Polyketide | 40.0 | 63.8 | 237.0 | 9.32e-73 |
| iliB |  | BGC0002035 | NRP+Polyketide | 38.0 | 64.6 | 226.0 | 1.82e-68 |
| CEF75882.1 |  | BGC0001600 | Polyketide | 39.0 | 62.2 | 225.0 | 2.7e-68 |
| ATZ45181.1 | Bcboa5 | BGC0001892 | Polyketide | 36.0 | 67.8 | 216.0 | 9.97e-65 |
| QBE85642.1 | BuaC | BGC0001857 | Alkaloid+NRP+Polyketide:Iterative type I polyketide | 34.0 | 62.4 | 215.0 | 1.67e-64 |
| AZZ09612.1 | PvhC | BGC0002304 | Polyketide+NRP | 34.0 | 63.7 | 213.0 | 6.59e-64 |
| ABA02243.1 | dehydrogenase | BGC0000098 | Polyketide | 36.0 | 60.0 | 209.0 | 2.83e-62 |
| BAC20562.1 | enoyl\_reductase | BGC0000039 | Polyketide | 36.0 | 60.6 | 209.0 | 2.99e-62 |
| AAD34554.1 | enoyl\_reductase | BGC0000088 | Polyketide | 36.0 | 61.3 | 209.0 | 3.07e-62 |
| QCS37515.1 | pyiC | BGC0001881 | NRP+Polyketide:Iterative type I polyketide | 36.0 | 64.9 | 209.0 | 5.06e-62 |
| QOG08941.1 | FfsC | BGC0002204 | Polyketide+NRP | 38.0 | 62.7 | 206.0 | 3.59e-61 |
| BBC43187.1 | trans-enoyl\_reductase | BGC0001738 | NRP+Polyketide | 34.0 | 63.5 | 206.0 | 4.39e-61 |
| KAF5858311.1 | Enoyl\_reductase | BGC0002139 | Polyketide | 35.0 | 62.4 | 204.0 | 1.43e-60 |
| CAL69596.1 | hypothetical\_protein | BGC0001049 | NRP+Polyketide:Iterative type I polyketide | 33.0 | 68.5 | 204.0 | 3.35e-60 |
| CBF80481.1 | enoylreductase | BGC0000959 | NRP+Polyketide:Iterative type I polyketide | 35.0 | 59.1 | 202.0 | 7.95e-60 |
| CAO91860.1 | putative\_enoyl\_reductase | BGC0000968 | NRP+Polyketide:Iterative type I polyketide | 34.0 | 62.7 | 201.0 | 2.09e-59 |
| AEO57491.1 | enoylreductase | BGC0001449 | NRP+Alkaloid+Polyketide:Iterative type I polyketide | 35.0 | 66.5 | 201.0 | 4.07e-59 |
| QOJ72665.1 | XenG | BGC0002505 | Polyketide+NRP | 35.0 | 62.0 | 192.0 | 6.62e-56 |
| QHD43131.1 | enoyl\_reductase | BGC0002546 | NRP+Polyketide | 34.0 | 63.3 | 183.0 | 1.98e-52 |
| QBC19713.1 | TwmE | BGC0001954 | NRP+Polyketide | 35.0 | 62.0 | 183.0 | 2.55e-52 |
| XP\_001220461.1 | enoyl\_reducctase\_ | BGC0001182 | NRP+Polyketide:Iterative type I polyketide | 33.0 | 66.0 | 180.0 | 3.24e-51 |
| EHA55861.1 | hypothetical\_protein | BGC0002235 | Polyketide+NRP | 31.0 | 61.3 | 177.0 | 2.1e-50 |
| EAT85330.1 | hypothetical\_protein | BGC0002165 | Polyketide | 32.0 | 61.3 | 144.0 | 4.84e-38 |
| BAQ25464.1 | zinc-binding\_dehydrogenase\_family\_protein | BGC0001264 | Polyketide | 32.0 | 61.8 | 137.0 | 1.51e-35 |
| QJX57336.1 | ChaC | BGC0002538 | Polyketide | 28.0 | 48.1 | 114.0 | 2.1e-27 |
| OPB37946.1 | hypothetical\_protein | BGC0002206 | Polyketide | 33.0 | 36.3 | 80.0 | 6.56e-16 |
| ATY46597.1 | NADPH:quinone\_reductase | BGC0001666 | Polyketide | 29.0 | 35.8 | 64.0 | 9.38e-11 |
| QWF78547.1 | 3-ketoacyl-CoA\_thiolase | BGC0002142 | Polyketide | 32.0 | 35.6 | 59.0 | 1.39e-08 |
| BAO66529.1 | type\_I\_polyketide\_synthase | BGC0000042 | Polyketide | 33.0 | 28.0 | 57.0 | 7.18e-08 |
| WP\_055480219.1 | type\_I\_polyketide\_synthase | BGC0001653 | Polyketide | 29.0 | 36.0 | 56.0 | 9.45e-08 |
| ABI91459.1 | Alcohol\_dehydrogenase,\_zinc-binding\_domain\_protein | BGC0001094 | NRP+Polyketide | 30.0 | 32.9 | 55.0 | 1.14e-07 |
| QSV12662.1 | AvmD | BGC0002456 | Polyketide+NRP | 27.0 | 32.9 | 56.0 | 1.26e-07 |
| QNN81302.1 | IonAVI | BGC0002446 | Polyketide | 31.0 | 26.0 | 55.0 | 2.79e-07 |
| AAZ94389.1 | modular\_polyketide\_synthase | BGC0000040 | Polyketide | 30.0 | 30.9 | 54.0 | 4.8e-07 |
| AKQ22681.1 | malonyl\_CoA-acyl\_carrier\_protein\_transacylase | BGC0001656 | Polyketide | 26.0 | 48.3 | 54.0 | 4.85e-07 |
| AGM05531.1 | type\_I\_polyketide\_synthase | BGC0002098 | Polyketide | 30.0 | 27.1 | 53.0 | 1.09e-06 |
| BCB17033.1 | modular\_polyketide\_synthase | BGC0002523 | NRP | 29.0 | 38.5 | 53.0 | 1.09e-06 |
| QSV12663.1 | AvmE | BGC0002456 | Polyketide+NRP | 29.0 | 34.0 | 52.0 | 1.89e-06 |
| BBA66512.1 | type\_I\_polyketide\_synthase | BGC0001495 | Polyketide | 30.0 | 27.3 | 52.0 | 1.91e-06 |
| ATX68111.1 | malonyl\_CoA-acyl\_carrier\_protein\_transacylase | BGC0001772 | Polyketide | 26.0 | 46.1 | 52.0 | 2.49e-06 |
| QSV12661.1 | AvmC | BGC0002456 | Polyketide+NRP | 26.0 | 48.1 | 51.0 | 4.24e-06 |
| AAF86392.1 | FkbC | BGC0000994 | NRP+Polyketide | 28.0 | 34.9 | 50.0 | 5.56e-06 |
| ADC79618.1 | BafAIII | BGC0000028 | Polyketide:Modular type I polyketide | 32.0 | 23.0 | 50.0 | 5.59e-06 |
| QKV49767.1 | PKS | BGC0002526 | Polyketide | 27.0 | 27.3 | 50.0 | 5.6e-06 |
| ACN69988.1 | polyketide\_synthase | BGC0000079 | Polyketide | 31.0 | 25.5 | 50.0 | 5.64e-06 |
| CAA60462.1 | polyketide\_synthase | BGC0001040 | NRP+Polyketide | 26.0 | 41.6 | 50.0 | 5.69e-06 |
| BCK51633.1 | modular\_polyketide\_synthase | BGC0002520 | Polyketide | 28.0 | 44.1 | 50.0 | 7.42e-06 |
