# Supplementary material for: Alkaliphilic/Alkali-Tolerant Fungi: Molecular, Biochemical, and Biotechnological Aspects
Source: J Fungi (Basel). 2023 Jun 9;9(6):652. doi: 10.3390/jof9060652 (PMC10301932; doi:10.3390/jof9060652)
Supplement: Supplementary file 1 [file jof-09-00652-s001.zip › S2/knownclusterblast/region1/input.path1.gene22_mibig_hits.html]

| MIBiG Protein | Description | MIBiG Cluster | MiBiG Product | % ID | % Coverage | BLAST Score | E-value |
| --- | --- | --- | --- | --- | --- | --- | --- |
| EAU38973.1 | predicted\_protein | BGC0001122 | NRP+Polyketide:Iterative type I polyketide | 41.0 | 107.9 | 287.0 | 2.97e-93 |
| EAL85114.1 | alpha/beta\_hydrolase | BGC0001037 | NRP+Polyketide:Iterative type I polyketide | 41.0 | 112.0 | 284.0 | 5.59e-92 |
| BBQ09586.1 | esterase | BGC0002261 | Polyketide | 40.0 | 109.7 | 276.0 | 1.84e-89 |
| ACS68553.1 | alpha/beta\_hydrolase | BGC0001026 | NRP+Polyketide | 38.0 | 112.0 | 272.0 | 1.42e-87 |
| QJY30861.1 | alpha/beta\_hydrolase | BGC0002539 | Alkaloid | 37.0 | 111.0 | 245.0 | 6.24e-77 |
| EHA55862.1 | dipeptidyl\_aminopeptidase/acylaminoacyl\_peptidase | BGC0002235 | Polyketide+NRP | 38.0 | 111.0 | 244.0 | 1.09e-76 |
| OJJ98487.1 | hypothetical\_protein | BGC0002169 | Polyketide+NRP | 38.0 | 110.5 | 250.0 | 1.26e-76 |
| EPS29078.1 | hypothetical\_protein | BGC0001724 | NRP+Polyketide | 37.0 | 110.2 | 243.0 | 2e-76 |
| EAW09119.1 | conserved\_hypothetical\_protein | BGC0000983 | NRP+Polyketide:Iterative type I polyketide | 37.0 | 109.5 | 236.0 | 1.02e-73 |
| QHD43139.1 | hydrolase | BGC0002546 | NRP+Polyketide | 37.0 | 110.7 | 234.0 | 1.24e-72 |
| QOG08947.1 | FfsE | BGC0002204 | Polyketide+NRP | 37.0 | 112.8 | 232.0 | 6.33e-72 |
| QCS37512.1 | pyiE | BGC0001881 | NRP+Polyketide:Iterative type I polyketide | 35.0 | 116.1 | 231.0 | 2.46e-71 |
| QOJ72659.1 | XenA | BGC0002505 | Polyketide+NRP | 35.0 | 109.7 | 217.0 | 2.83e-66 |
| QXF14596.1 | PydG | BGC0002239 | Polyketide+NRP | 35.0 | 108.2 | 211.0 | 3.41e-64 |
| EAT91808.1 | hypothetical\_protein | BGC0002205 | Polyketide+NRP | 35.0 | 114.1 | 212.0 | 3.62e-64 |
| AEO57489.1 | hydrolase | BGC0001449 | NRP+Alkaloid+Polyketide:Iterative type I polyketide | 31.0 | 117.1 | 198.0 | 2.03e-58 |
| CAA60450.1 |  | BGC0001040 | NRP+Polyketide | 31.0 | 105.4 | 146.0 | 6.97e-40 |
| AXM42923.1 | metal-dependent\_hydrolase | BGC0001940 | Polyketide | 37.0 | 40.7 | 80.0 | 4.58e-16 |
| CAE16558.1 |  | BGC0000196 | Polyketide:Type II polyketide | 31.0 | 36.8 | 59.0 | 4.3e-09 |
