# Supplementary material for: Alkaliphilic/Alkali-Tolerant Fungi: Molecular, Biochemical, and Biotechnological Aspects
Source: J Fungi (Basel). 2023 Jun 9;9(6):652. doi: 10.3390/jof9060652 (PMC10301932; doi:10.3390/jof9060652)
Supplement: Supplementary file 1 [file jof-09-00652-s001.zip › S2/knownclusterblast/region1/input.path1.gene23_mibig_hits.html]

| MIBiG Protein | Description | MIBiG Cluster | MiBiG Product | % ID | % Coverage | BLAST Score | E-value |
| --- | --- | --- | --- | --- | --- | --- | --- |
| cds\_0\_1525 |  | BGC0000334 | NRP | 26.0 | 57.9 | 59.0 | 3.88e-09 |
