# Supplementary material for: Alkaliphilic/Alkali-Tolerant Fungi: Molecular, Biochemical, and Biotechnological Aspects
Source: J Fungi (Basel). 2023 Jun 9;9(6):652. doi: 10.3390/jof9060652 (PMC10301932; doi:10.3390/jof9060652)
Supplement: Supplementary file 1 [file jof-09-00652-s001.zip › S2/knownclusterblast/region1/input.path1.gene24_mibig_hits.html]

| MIBiG Protein | Description | MIBiG Cluster | MiBiG Product | % ID | % Coverage | BLAST Score | E-value |
| --- | --- | --- | --- | --- | --- | --- | --- |
| ATV82114.1 | GMC\_oxidoreductase/oxidase/dehydrogenase | BGC0001909 | Polyketide | 32.0 | 103.9 | 201.0 | 1.23e-56 |
| EIN09539.1 | pyranose\_dehydrogenase | BGC0002213 | Polyketide | 30.0 | 104.9 | 185.0 | 6.87e-51 |
| ctg1\_orf10 |  | BGC0000846 | Other | 27.0 | 101.7 | 187.0 | 7.15e-51 |
| KDM89831.1 | glucose-methanol-choline\_oxidoreductase | BGC0002412 | NRP | 29.0 | 104.9 | 174.0 | 2.44e-47 |
| AEF33092.1 | choline\_dehydrogenase | BGC0001039 | NRP+Polyketide | 30.0 | 100.0 | 173.0 | 3.54e-47 |
| CBF83141.1 | conserved\_hypothetical\_protein | BGC0001722 | Polyketide | 27.0 | 103.9 | 171.0 | 7.65e-46 |
| MCB8905710.1 | GMC\_family\_oxidoreductase\_N-terminal\_domain-containing\_protein | BGC0002340 | NRP+Other | 29.0 | 103.7 | 169.0 | 2.8e-45 |
| BAQ25461.1 | putative\_dehydrogenase | BGC0001264 | Polyketide | 27.0 | 107.3 | 168.0 | 1.3e-44 |
| AAS90088.1 | VBS | BGC0000010 | Polyketide | 27.0 | 103.1 | 153.0 | 3.07e-39 |
| AAS90042.1 | VBS | BGC0000008 | Polyketide | 27.0 | 103.1 | 152.0 | 5.65e-39 |
| BAE71331.1 | versicolorin\_B\_synthase | BGC0000004 | Polyketide | 27.0 | 103.1 | 151.0 | 1.41e-38 |
| AIG62134.1 | patulin\_synthase | BGC0000120 | Polyketide:Iterative type I polyketide | 25.0 | 104.6 | 150.0 | 3.08e-38 |
| AAS90019.1 | VBS | BGC0000007 | Polyketide | 26.0 | 103.1 | 149.0 | 6.41e-38 |
| EEP98515.1 | Glucose-methanol-choline\_oxidoreductase | BGC0002091 | NRP | 26.0 | 103.1 | 147.0 | 1.35e-37 |
| AAS90106.1 | VBS | BGC0000006 | Polyketide | 27.0 | 103.1 | 147.0 | 2.15e-37 |
| AAS90066.1 | VBS | BGC0000009 | Polyketide | 26.0 | 103.1 | 146.0 | 7.07e-37 |
| OJJ97584.1 | hypothetical\_protein | BGC0002229 | Polyketide | 28.0 | 97.8 | 144.0 | 7.24e-37 |
| EHK18384.1 | hypothetical\_protein | BGC0002216 | Terpene | 27.0 | 105.1 | 140.0 | 3.29e-35 |
| BBD84647.1 | putative\_GMC\_oxidoreductase | BGC0001775 | Terpene | 27.0 | 108.0 | 140.0 | 3.31e-35 |
| EAU32818.1 | predicted\_protein | BGC0000160 | Polyketide | 26.0 | 102.0 | 140.0 | 3.37e-35 |
| ACA34720.1 | CtnD | BGC0000894 | Other | 26.0 | 108.7 | 135.0 | 2.71e-33 |
| ALI92648.1 | CitC\_oxidoreductase | BGC0001338 | Polyketide:Iterative type I polyketide | 26.0 | 108.7 | 135.0 | 2.71e-33 |
| ACH72898.1 | AflK | BGC0000011 | Polyketide | 25.0 | 103.9 | 133.0 | 1.76e-32 |
| CAM56763.1 | hypothetical\_protein | BGC0000354 | NRP | 27.0 | 88.6 | 129.0 | 8.03e-32 |
| AJI44177.1 | glucose-methanol-choline\_oxidoreductase | BGC0001193 | NRP | 28.0 | 101.5 | 126.0 | 1.5e-30 |
| CBK62747.1 |  | BGC0001115 | NRP+Polyketide | 24.0 | 104.4 | 125.0 | 4.59e-30 |
| CAD62204.1 | Ata10\_protein | BGC0000873 | Other | 32.0 | 54.0 | 119.0 | 1.98e-28 |
| AET51867.1 | oxidoreductase | BGC0001138 | Other:Nucleoside | 32.0 | 53.5 | 117.0 | 2.08e-27 |
| KNA98285.1 | hypothetical\_protein | BGC0002670 | Other | 25.0 | 104.3 | 116.0 | 5.84e-27 |
| KAF7526514.1 | hypothetical\_protein | BGC0002244 | Polyketide | 24.0 | 102.7 | 107.0 | 2.86e-24 |
