# Supplementary material for: Alkaliphilic/Alkali-Tolerant Fungi: Molecular, Biochemical, and Biotechnological Aspects
Source: J Fungi (Basel). 2023 Jun 9;9(6):652. doi: 10.3390/jof9060652 (PMC10301932; doi:10.3390/jof9060652)
Supplement: Supplementary file 1 [file jof-09-00652-s001.zip › S2/knownclusterblast/region1/input.path1.gene25_mibig_hits.html]

| MIBiG Protein | Description | MIBiG Cluster | MiBiG Product | % ID | % Coverage | BLAST Score | E-value |
| --- | --- | --- | --- | --- | --- | --- | --- |
| EAU31631.1 | predicted\_protein | BGC0002592 | Polyketide | 50.0 | 58.0 | 587.0 | 8.2e-200 |
| EAL89346.1 | extracellular\_dihydrogeodin\_oxidase/laccase,\_putative | BGC0001403 | Polyketide | 48.0 | 60.3 | 566.0 | 1.64e-191 |
| KAF7526517.1 | hypothetical\_protein | BGC0002244 | Polyketide | 46.0 | 58.4 | 520.0 | 9.39e-174 |
| AGO59042.1 | PtaE | BGC0000121 | Polyketide | 42.0 | 60.4 | 482.0 | 5.18e-159 |
| EJP62796.1 | laccase\_2 | BGC0001720 | Polyketide | 34.0 | 59.5 | 321.0 | 8.39e-98 |
| MAA\_10032 | laccase\_Lcc4 | BGC0000337 | NRP | 29.0 | 57.4 | 224.0 | 4.83e-63 |
| AGO59052.1 | PtaK | BGC0000121 | Polyketide | 28.0 | 51.4 | 181.0 | 2.88e-48 |
| ETS82098.1 | hypothetical\_protein | BGC0002161 | Polyketide | 24.0 | 51.0 | 103.0 | 2.19e-22 |
| QKG86291.1 | laccase | BGC0002253 | Polyketide | 24.0 | 40.5 | 89.0 | 8.88e-18 |
| ESU07754.1 | hypothetical\_protein | BGC0002709 | Polyketide | 24.0 | 36.9 | 77.0 | 5.16e-14 |
| XP\_028481812.1 | multi-copper\_oxidase/laccase | BGC0001866 | Polyketide | 23.0 | 35.7 | 73.0 | 9.15e-13 |
| QZA73320.1 | multicopper\_oxidase\_family\_protein | BGC0002385 | Polyketide | 25.0 | 51.9 | 66.0 | 1.87e-10 |
