# Supplementary material for: Alkaliphilic/Alkali-Tolerant Fungi: Molecular, Biochemical, and Biotechnological Aspects
Source: J Fungi (Basel). 2023 Jun 9;9(6):652. doi: 10.3390/jof9060652 (PMC10301932; doi:10.3390/jof9060652)
Supplement: Supplementary file 1 [file jof-09-00652-s001.zip › S2/knownclusterblast/region1/input.path1.gene26_mibig_hits.html]

| MIBiG Protein | Description | MIBiG Cluster | MiBiG Product | % ID | % Coverage | BLAST Score | E-value |
| --- | --- | --- | --- | --- | --- | --- | --- |
| EAU31627.1 | hypothetical\_protein | BGC0002592 | Polyketide | 62.0 | 96.5 | 290.0 | 5.66e-100 |
| EAL89342.1 | glutathione\_S-transferase\_Ure2-like,\_putative | BGC0001403 | Polyketide | 60.0 | 96.5 | 283.0 | 2.99e-97 |
| EJP62797.1 | glutathione-s-transferase | BGC0001720 | Polyketide | 41.0 | 98.2 | 143.0 | 2.93e-42 |
