# Supplementary material for: Alkaliphilic/Alkali-Tolerant Fungi: Molecular, Biochemical, and Biotechnological Aspects
Source: J Fungi (Basel). 2023 Jun 9;9(6):652. doi: 10.3390/jof9060652 (PMC10301932; doi:10.3390/jof9060652)
Supplement: Supplementary file 1 [file jof-09-00652-s001.zip › S2/knownclusterblast/region1/input.path1.gene27_mibig_hits.html]

| MIBiG Protein | Description | MIBiG Cluster | MiBiG Product | % ID | % Coverage | BLAST Score | E-value |
| --- | --- | --- | --- | --- | --- | --- | --- |
| QCL09101.1 | DmxR10 | BGC0002063 | Polyketide:Iterative type I polyketide | 49.0 | 108.8 | 132.0 | 2.08e-40 |
| AGO59045.1 | PtaG | BGC0000121 | Polyketide | 42.0 | 105.9 | 115.0 | 1.15e-33 |
| QBG38883.1 | hypothetical\_protein | BGC0002062 | Polyketide | 44.0 | 102.9 | 112.0 | 4.25e-32 |
| QCF41205.1 | CcxN | BGC0002726 | Polyketide | 36.0 | 105.9 | 98.0 | 1.51e-24 |
