# Supplementary material for: Alkaliphilic/Alkali-Tolerant Fungi: Molecular, Biochemical, and Biotechnological Aspects
Source: J Fungi (Basel). 2023 Jun 9;9(6):652. doi: 10.3390/jof9060652 (PMC10301932; doi:10.3390/jof9060652)
Supplement: Supplementary file 1 [file jof-09-00652-s001.zip › S2/knownclusterblast/region1/input.path1.gene28_mibig_hits.html]

| MIBiG Protein | Description | MIBiG Cluster | MiBiG Product | % ID | % Coverage | BLAST Score | E-value |
| --- | --- | --- | --- | --- | --- | --- | --- |
| KAF7526514.1 | hypothetical\_protein | BGC0002244 | Polyketide | 57.0 | 101.4 | 651.0 | 1.24e-231 |
| AIG62134.1 | patulin\_synthase | BGC0000120 | Polyketide:Iterative type I polyketide | 30.0 | 107.0 | 207.0 | 8.56e-59 |
| EIN09539.1 | pyranose\_dehydrogenase | BGC0002213 | Polyketide | 29.0 | 107.7 | 200.0 | 1.28e-56 |
| AAS90088.1 | VBS | BGC0000010 | Polyketide | 30.0 | 104.1 | 192.0 | 2.38e-53 |
| AAS90019.1 | VBS | BGC0000007 | Polyketide | 30.0 | 104.5 | 191.0 | 4.52e-53 |
| AAS90042.1 | VBS | BGC0000008 | Polyketide | 30.0 | 105.0 | 191.0 | 4.52e-53 |
| BAE71331.1 | versicolorin\_B\_synthase | BGC0000004 | Polyketide | 30.0 | 104.1 | 190.0 | 1.18e-52 |
| AAS90066.1 | VBS | BGC0000009 | Polyketide | 30.0 | 104.0 | 187.0 | 1.49e-51 |
| AAS90106.1 | VBS | BGC0000006 | Polyketide | 29.0 | 104.0 | 187.0 | 2.11e-51 |
| ACH72898.1 | AflK | BGC0000011 | Polyketide | 29.0 | 104.9 | 180.0 | 6.04e-49 |
| CBF83141.1 | conserved\_hypothetical\_protein | BGC0001722 | Polyketide | 27.0 | 110.8 | 176.0 | 1.41e-47 |
| KNA98285.1 | hypothetical\_protein | BGC0002670 | Other | 28.0 | 102.5 | 172.0 | 1.69e-46 |
| AEF33092.1 | choline\_dehydrogenase | BGC0001039 | NRP+Polyketide | 28.0 | 97.7 | 169.0 | 7.3e-46 |
| EAU32818.1 | predicted\_protein | BGC0000160 | Polyketide | 29.0 | 102.9 | 168.0 | 4.76e-45 |
| ATV82114.1 | GMC\_oxidoreductase/oxidase/dehydrogenase | BGC0001909 | Polyketide | 28.0 | 108.1 | 156.0 | 9.72e-41 |
| KDM89831.1 | glucose-methanol-choline\_oxidoreductase | BGC0002412 | NRP | 29.0 | 103.4 | 149.0 | 1.23e-38 |
| AJI44177.1 | glucose-methanol-choline\_oxidoreductase | BGC0001193 | NRP | 28.0 | 100.9 | 148.0 | 2.84e-38 |
| BBD84647.1 | putative\_GMC\_oxidoreductase | BGC0001775 | Terpene | 26.0 | 106.7 | 148.0 | 5.15e-38 |
| EHK18384.1 | hypothetical\_protein | BGC0002216 | Terpene | 30.0 | 66.2 | 134.0 | 2.92e-33 |
| ACA34720.1 | CtnD | BGC0000894 | Other | 31.0 | 64.2 | 133.0 | 1.18e-32 |
| ALI92648.1 | CitC\_oxidoreductase | BGC0001338 | Polyketide:Iterative type I polyketide | 31.0 | 64.2 | 133.0 | 1.18e-32 |
| OJJ97584.1 | hypothetical\_protein | BGC0002229 | Polyketide | 25.0 | 100.2 | 129.0 | 1.07e-31 |
| CAD62204.1 | Ata10\_protein | BGC0000873 | Other | 26.0 | 100.7 | 121.0 | 4.75e-29 |
| MCB8905710.1 | GMC\_family\_oxidoreductase\_N-terminal\_domain-containing\_protein | BGC0002340 | NRP+Other | 25.0 | 104.1 | 115.0 | 4.53e-27 |
| CAM56763.1 | hypothetical\_protein | BGC0000354 | NRP | 28.0 | 56.1 | 97.0 | 3.41e-21 |
