# Supplementary material for: Alkaliphilic/Alkali-Tolerant Fungi: Molecular, Biochemical, and Biotechnological Aspects
Source: J Fungi (Basel). 2023 Jun 9;9(6):652. doi: 10.3390/jof9060652 (PMC10301932; doi:10.3390/jof9060652)
Supplement: Supplementary file 1 [file jof-09-00652-s001.zip › S2/knownclusterblast/region1/input.path1.gene29_mibig_hits.html]

| MIBiG Protein | Description | MIBiG Cluster | MiBiG Product | % ID | % Coverage | BLAST Score | E-value |
| --- | --- | --- | --- | --- | --- | --- | --- |
| AGO59039.1 | PtaJ | BGC0000121 | Polyketide | 51.0 | 52.8 | 457.0 | 8.62e-154 |
| EAU31632.1 | predicted\_protein | BGC0002592 | Polyketide | 48.0 | 53.0 | 434.0 | 1.24e-144 |
| CAP93749.1 |  | BGC0001882 | Polyketide | 50.0 | 50.2 | 433.0 | 2.82e-144 |
| QCL09097.1 | dmxR6 | BGC0002063 | Polyketide:Iterative type I polyketide | 47.0 | 52.7 | 423.0 | 1.89e-140 |
| EAL89345.1 | conserved\_hypothetical\_protein | BGC0001403 | Polyketide | 45.0 | 53.6 | 416.0 | 1.77e-137 |
| QBG38885.1 | Baeyer-Villiger\_monoxygenase | BGC0002062 | Polyketide | 48.0 | 52.1 | 416.0 | 2.06e-137 |
| QCF41203.1 | CcxL | BGC0002726 | Polyketide | 45.0 | 51.3 | 395.0 | 2e-129 |
| CCE31574.1 | uncharacterized\_protein | BGC0001886 | Polyketide | 43.0 | 53.2 | 389.0 | 4.48e-127 |
| AGO59038.1 | PtaI | BGC0000121 | Polyketide | 56.0 | 40.3 | 379.0 | 5.01e-125 |
| KAF7526520.1 | hypothetical\_protein | BGC0002244 | Polyketide | 43.0 | 54.2 | 377.0 | 2.15e-122 |
| ADM34146.1 | hypothetical\_protein | BGC0001084 | NRP+Terpene+Alkaloid | 43.0 | 53.1 | 360.0 | 7.89e-117 |
| PKX92304.1 | hypothetical\_protein | BGC0001988 | Polyketide | 42.0 | 52.0 | 328.0 | 3.85e-104 |
| ACH72894.1 | AflY | BGC0000011 | Polyketide | 30.0 | 52.0 | 206.0 | 8.53e-58 |
| PKY07883.1 | S-adenosyl-L-methionine-dependent\_methyltransferase | BGC0001544 | NRP+Polyketide | 37.0 | 38.4 | 197.0 | 1.83e-56 |
| CCE31571.1 | uncharacterized\_protein | BGC0001886 | Polyketide | 24.0 | 38.2 | 82.0 | 2.54e-16 |
| EAU31629.1 | predicted\_protein | BGC0002592 | Polyketide | 26.0 | 39.8 | 78.0 | 3.75e-15 |
