# Supplementary material for: Alkaliphilic/Alkali-Tolerant Fungi: Molecular, Biochemical, and Biotechnological Aspects
Source: J Fungi (Basel). 2023 Jun 9;9(6):652. doi: 10.3390/jof9060652 (PMC10301932; doi:10.3390/jof9060652)
Supplement: Supplementary file 1 [file jof-09-00652-s001.zip › S2/knownclusterblast/region1/input.path1.gene30_mibig_hits.html]

| MIBiG Protein | Description | MIBiG Cluster | MiBiG Product | % ID | % Coverage | BLAST Score | E-value |
| --- | --- | --- | --- | --- | --- | --- | --- |
| QCL09099.1 | DmxR8 | BGC0002063 | Polyketide:Iterative type I polyketide | 65.0 | 100.0 | 376.0 | 1.17e-131 |
| EAT91807.1 | hypothetical\_protein | BGC0002205 | Polyketide+NRP | 39.0 | 97.2 | 155.0 | 5.88e-45 |
| CBF73447.1 | conserved\_hypothetical\_protein | BGC0001515 | NRP | 40.0 | 87.2 | 154.0 | 9.18e-45 |
| KAF9708865.1 | hypothetical\_protein | BGC0002515 | Polyketide | 36.0 | 94.3 | 151.0 | 1.74e-43 |
| QOG08946.1 | FfsI | BGC0002204 | Polyketide+NRP | 37.0 | 101.1 | 148.0 | 3.89e-42 |
| ESU17759.1 | hypothetical\_protein | BGC0002172 | NRP | 31.0 | 101.1 | 139.0 | 1.32e-38 |
| EHK18432.1 | hypothetical\_protein | BGC0002233 | Polyketide | 36.0 | 102.1 | 136.0 | 1.51e-37 |
| BAJ09787.1 | dehydrogenase | BGC0000146 | Polyketide | 35.0 | 85.4 | 125.0 | 7.25e-34 |
| EHA55863.1 | hypothetical\_protein | BGC0002235 | Polyketide+NRP | 32.0 | 101.1 | 124.0 | 5.21e-33 |
| EHK18427.1 | hypothetical\_protein | BGC0002233 | Polyketide | 32.0 | 103.9 | 112.0 | 9.33e-29 |
