# Supplementary material for: Alkaliphilic/Alkali-Tolerant Fungi: Molecular, Biochemical, and Biotechnological Aspects
Source: J Fungi (Basel). 2023 Jun 9;9(6):652. doi: 10.3390/jof9060652 (PMC10301932; doi:10.3390/jof9060652)
Supplement: Supplementary file 1 [file jof-09-00652-s001.zip › S2/knownclusterblast/region1/input.path1.gene31_mibig_hits.html]

| MIBiG Protein | Description | MIBiG Cluster | MiBiG Product | % ID | % Coverage | BLAST Score | E-value |
| --- | --- | --- | --- | --- | --- | --- | --- |
| QCL09098.1 | DmxR7 | BGC0002063 | Polyketide:Iterative type I polyketide | 65.0 | 96.0 | 296.0 | 2.05e-102 |
| CAP93748.1 |  | BGC0001882 | Polyketide | 61.0 | 94.7 | 273.0 | 3.14e-93 |
| QBG38884.1 | oxidoreductase | BGC0002062 | Polyketide | 58.0 | 96.0 | 269.0 | 4e-91 |
| KAF7526519.1 | hypothetical\_protein | BGC0002244 | Polyketide | 57.0 | 96.0 | 261.0 | 6.15e-88 |
| PKX92292.1 | hypothetical\_protein | BGC0001988 | Polyketide | 54.0 | 95.1 | 242.0 | 1.83e-80 |
| EAL89343.1 | toxin\_biosynthesis\_protein,\_putative | BGC0001403 | Polyketide | 55.0 | 94.7 | 241.0 | 4.88e-80 |
| AGO59035.1 | PtaF | BGC0000121 | Polyketide | 54.0 | 96.4 | 237.0 | 1.06e-78 |
| CCE31577.1 | uncharacterized\_protein | BGC0001886 | Polyketide | 50.0 | 96.4 | 218.0 | 2.53e-71 |
| QCF41207.1 | CcxP | BGC0002726 | Polyketide | 47.0 | 96.0 | 210.0 | 5.73e-68 |
| AAC49202.1 |  | BGC0000152 | Polyketide | 42.0 | 97.8 | 189.0 | 1.23e-59 |
| ACH72895.1 | AflX | BGC0000011 | Polyketide | 41.0 | 96.0 | 188.0 | 3.02e-59 |
| AAS90016.1 | OrdB | BGC0000007 | Polyketide | 43.0 | 96.0 | 185.0 | 3.14e-58 |
| BAE71334.1 | oxidreductase\_B/\_cytochrome\_P450\_monooxygenase | BGC0000004 | Polyketide | 43.0 | 97.3 | 184.0 | 6.28e-58 |
| AAS90038.1 | OrdB | BGC0000008 | Polyketide | 43.0 | 97.3 | 184.0 | 6.28e-58 |
| AAS90109.1 | OrdB | BGC0000006 | Polyketide | 42.0 | 96.0 | 182.0 | 7.12e-57 |
| AAS90084.1 | OrdB | BGC0000010 | Polyketide | 42.0 | 96.0 | 182.0 | 7.12e-57 |
| AAS90064.1 | OrdB | BGC0000009 | Polyketide | 41.0 | 96.0 | 176.0 | 1.36e-54 |
| EAU31628.1 | conserved\_hypothetical\_protein | BGC0002592 | Polyketide | 61.0 | 44.9 | 125.0 | 2.96e-36 |
| ACH72897.1 | AflI | BGC0000011 | Polyketide | 31.0 | 97.8 | 91.0 | 1.91e-21 |
