# Supplementary material for: Alkaliphilic/Alkali-Tolerant Fungi: Molecular, Biochemical, and Biotechnological Aspects
Source: J Fungi (Basel). 2023 Jun 9;9(6):652. doi: 10.3390/jof9060652 (PMC10301932; doi:10.3390/jof9060652)
Supplement: Supplementary file 1 [file jof-09-00652-s001.zip › S2/knownclusterblast/region1/input.path1.gene36_mibig_hits.html]

| MIBiG Protein | Description | MIBiG Cluster | MiBiG Product | % ID | % Coverage | BLAST Score | E-value |
| --- | --- | --- | --- | --- | --- | --- | --- |
| EAA62478.1 | hypothetical\_protein | BGC0002276 | NRP | 63.0 | 88.7 | 1348.0 | 0.0 |
| AGC45618.1 | non-ribosomal\_peptide\_synthetase | BGC0001394 | NRP+Polyketide | 36.0 | 83.2 | 477.0 | 5.94e-145 |
| AAK57184.1 | MxaA | BGC0001022 | NRP+Polyketide | 34.0 | 84.2 | 461.0 | 3.67e-139 |
| AQW44894.1 | non-ribosomal\_peptide\_synthetase | BGC0001737 | NRP+Polyketide | 35.0 | 83.4 | 451.0 | 2.1e-135 |
| AAC44129.1 | saframycin\_Mx1\_synthetase\_A | BGC0002706 | NRP | 36.0 | 76.3 | 438.0 | 3.07e-128 |
| BAP27942.1 | nonribosomal\_peptide\_synthetase | BGC0001085 | NRP+Terpene | 34.0 | 83.4 | 420.0 | 2.51e-122 |
| AOC89001.1 | putative\_nonribosomal\_peptide\_synthetase | BGC0001652 | NRP | 32.0 | 83.1 | 409.0 | 2.3e-120 |
| AAO23334.1 | NcpB | BGC0000397 | NRP | 31.0 | 85.1 | 411.0 | 3.19e-119 |
| AFO85453.1 | non-ribosomal\_peptide\_synthetase | BGC0000391 | NRP | 34.0 | 83.5 | 403.0 | 1.67e-116 |
| ATD51280.1 | nonribosomal\_peptide\_synthase | BGC0001650 | NRP | 34.0 | 77.0 | 398.0 | 2.89e-116 |
| BAY02139.1 | nonribosomal\_protein\_synthetase | BGC0002532 | NRP+Polyketide | 31.0 | 82.5 | 396.0 | 9.47e-116 |
| ACN39015.1 | putative\_nonribosomal\_peptide\_synthetase\_TomB | BGC0000448 | NRP | 32.0 | 82.6 | 392.0 | 3.64e-114 |
| QUF98525.1 | non-ribosomal\_peptide\_synthetase | BGC0002582 | NRP | 31.0 | 83.1 | 382.0 | 1.71e-113 |
| ATP76246.1 | SpuB | BGC0001748 | NRP+Polyketide | 32.0 | 82.8 | 394.0 | 1.76e-113 |
| ABI22133.1 | putative\_non-ribosomal\_peptide\_synthetase | BGC0000422 | NRP | 33.0 | 76.3 | 388.0 | 3.33e-113 |
| AGD80618.1 | non-ribosomal\_peptide\_synthetase | BGC0000394 | NRP | 31.0 | 76.1 | 385.0 | 5.4e-112 |
| CAF32362.1 | putative\_non-ribosomal\_peptide\_synthetase | BGC0000712 | Saccharide | 31.0 | 83.1 | 371.0 | 1.85e-109 |
| BAO84866.1 | putative\_non-ribosomal\_peptide\_synthetase | BGC0000414 | NRP | 31.0 | 76.4 | 370.0 | 6.86e-107 |
| EOY45602.1 | Adenylation\_and\_reductase\_domains\_containing\_protein | BGC0001168 | NRP | 31.0 | 81.6 | 360.0 | 2.02e-106 |
| ACN39727.1 | SibD | BGC0000428 | NRP | 31.0 | 84.0 | 367.0 | 1.26e-105 |
| AHJ31215.1 | Long-chain-fatty-acid--CoA\_ligase | BGC0000430 | NRP+Polyketide:Modular type I polyketide | 30.0 | 85.0 | 369.0 | 4.57e-105 |
| AAK89727.1 | peptide\_synthetase,\_siderophore\_biosynthesis\_protein | BGC0002107 | NRP+Polyketide | 30.0 | 82.7 | 362.0 | 7.63e-104 |
| AAT12283.1 | LtxA | BGC0000384 | NRP | 31.0 | 82.7 | 360.0 | 1.58e-102 |
| ABF87167.1 | non-ribosomal\_peptide\_synthase\_MxcG | BGC0002492 | NRP | 32.0 | 84.6 | 350.0 | 4.12e-100 |
| ATD51278.1 | nonribosomal\_peptide\_synthase | BGC0001650 | NRP | 33.0 | 78.9 | 353.0 | 6.52e-100 |
| AZL87944.1 | aspergillic\_acid\_non-ribosomal\_peptide\_sythetase-like\_(NRPS-like)\_(AsaC) | BGC0002602 | Alkaloid | 30.0 | 77.5 | 342.0 | 8.22e-100 |
| XP\_002373813.1 | NRPS-like\_enzyme,\_putative | BGC0001516 | NRP | 29.0 | 77.4 | 338.0 | 1.92e-98 |
| AAL33758.1 | putative\_non-ribosomal\_peptide\_synthetase | BGC0000421 | NRP | 31.0 | 75.6 | 345.0 | 2.41e-98 |
| AAG31130.1 | MxcG | BGC0001345 | NRP | 30.0 | 84.3 | 341.0 | 5.98e-97 |
| ATY72525.1 | non-ribosomal\_peptide\_synthetase | BGC0001574 | NRP | 32.0 | 84.7 | 339.0 | 1.59e-96 |
| QRK05501.1 | myxochelin\_non-ribosomal\_peptide\_synthetase\_MxcG | BGC0002324 | NRP+Polyketide | 32.0 | 83.2 | 338.0 | 5.92e-96 |
| ATJ04411.1 | NRPS,\_TomB\_binding | BGC0001637 | NRP | 29.0 | 76.3 | 328.0 | 8.83e-93 |
| CDG76959.1 | non-ribosomal\_peptide\_synthetase,\_terminal\_component | BGC0000446 | NRP:Pyrrolobenzodiazepine | 29.0 | 76.4 | 327.0 | 2.91e-92 |
| AEA29644.1 | putative\_nonribosomal\_peptide\_synthetase\_and\_kinurenine\_monooxygenase | BGC0000409 | NRP | 31.0 | 82.0 | 328.0 | 3.54e-92 |
| ABW71853.1 | nonribosomal\_peptide\_synthetase | BGC0000303 | NRP | 32.0 | 77.7 | 321.0 | 3.19e-90 |
| ADQ55475.1 | NRPS | BGC0000350 | NRP:Beta-lactam | 27.0 | 77.1 | 319.0 | 1.03e-89 |
| CBG67541.1 | putative\_non-ribosomal\_peptide\_synthetase | BGC0002367 | NRP | 29.0 | 84.6 | 315.0 | 1.19e-87 |
| CDN62030.1 | Peptide\_synthetase | BGC0001599 | NRP | 29.0 | 83.6 | 310.0 | 2.29e-87 |
| AAD44234.1 | PstB | BGC0000362 | NRP | 29.0 | 78.4 | 313.0 | 3.56e-87 |
| QMW33900.1 | hypothetical\_protein | BGC0002167 | NRP | 27.0 | 85.1 | 301.0 | 4.25e-85 |
| ABY83163.1 | Azi25 | BGC0000960 | NRP+Polyketide | 31.0 | 75.3 | 302.0 | 8.32e-84 |
| CEO59272.1 | hypothetical\_protein | BGC0002278 | Alkaloid+NRP | 28.0 | 83.8 | 296.0 | 1.06e-83 |
| QQO98485.1 | FrzA | BGC0002146 | NRP | 27.0 | 85.0 | 293.0 | 1.09e-82 |
| CAJ77696.1 | MPS2\_protein | BGC0000363 | NRP | 29.0 | 79.0 | 298.0 | 2.53e-82 |
| AEC14349.1 | nonribosomal\_peptide\_synthetase | BGC0000377 | NRP | 25.0 | 82.1 | 283.0 | 2.29e-79 |
| KAF7597140.1 | hypothetical\_protein | BGC0002264 | NRP | 27.0 | 82.6 | 281.0 | 1.65e-78 |
| CAJ77716.1 | Mps2\_protein | BGC0000364 | NRP | 29.0 | 78.6 | 286.0 | 2.28e-78 |
| WP\_019032755.1 | non-ribosomal\_peptide\_synthetase | BGC0001331 | NRP:Cyclic depsipeptide+Polyketide:Modular type I polyketide | 36.0 | 49.5 | 286.0 | 2.59e-78 |
| AAT01807.1 | non-ribosomal\_peptide\_synthetase | BGC0000365 | NRP | 29.0 | 79.5 | 283.0 | 2.19e-77 |
| WP\_053065269.1 | non-ribosomal\_peptide\_synthetase | BGC0001330 | NRP:Cyclic depsipeptide+Polyketide:Modular type I polyketide | 36.0 | 49.3 | 279.0 | 3.2e-76 |
| BBQ09587.1 | PKS-NRPS\_hybrid | BGC0002261 | Polyketide | 28.0 | 80.9 | 279.0 | 4.64e-76 |
| CCP42826.1 | Probable\_peptide\_synthetase\_Nrp\_(peptide\_synthase) | BGC0001627 | NRP | 29.0 | 79.7 | 277.0 | 1.51e-75 |
| ATJ34002.1 | non-ribosomal\_peptide\_synthetase | BGC0001442 | NRP | 29.0 | 79.7 | 273.0 | 1.45e-74 |
| AAU34202.1 | mannopeptimycin\_peptide\_synthetase\_MppA | BGC0000388 | NRP | 35.0 | 48.4 | 274.0 | 1.53e-74 |
| BAW32323.1 | hybrid\_cis-AT\_polyketide\_synthase\_-\_nonribosomal\_peptide\_synthetase | BGC0001630 | NRP+Polyketide | 33.0 | 54.6 | 270.0 | 3.95e-73 |
| AJM89734.1 | PmxB | BGC0001192 | NRP | 34.0 | 48.1 | 266.0 | 4.24e-73 |
| WP\_054234643.1 | non-ribosomal\_peptide\_synthetase | BGC0002014 | NRP+Polyketide | 34.0 | 49.0 | 267.0 | 2.87e-72 |
| AGP37410.1 | peptide\_synthetase | BGC0002386 | NRP+Polyketide | 33.0 | 50.9 | 266.0 | 8.74e-72 |
| AEZ51517.1 | pmxB | BGC0001153 | NRP:Lipopeptide | 33.0 | 48.0 | 262.0 | 1.06e-71 |
| AEZ51520.1 | pmxE | BGC0001153 | NRP:Lipopeptide | 32.0 | 49.8 | 265.0 | 1.78e-71 |
| ACA97580.1 | PmxE | BGC0000408 | NRP | 33.0 | 49.8 | 265.0 | 2.35e-71 |
| AGM16412.1 | paenibacterin\_synthetase\_A | BGC0000400 | NRP | 34.0 | 47.1 | 264.0 | 4.16e-71 |
| ABL74945.1 | NRPS | BGC0001048 | NRP:Glycopeptide+Polyketide:Modular type I polyketide+Saccharide:Hybrid/tailoring saccharide | 34.0 | 48.2 | 263.0 | 6.91e-71 |
| APO47822.1 | non-ribosomal\_peptide\_synthetase | BGC0002653 | NRP | 32.0 | 49.4 | 263.0 | 7.23e-71 |
| AFP73394.1 | FusA | BGC0001268 | NRP+Polyketide | 27.0 | 78.1 | 263.0 | 8.27e-71 |
| AJM89738.1 | PmxE | BGC0001192 | NRP | 32.0 | 49.8 | 262.0 | 1.68e-70 |
| ACA97577.1 | PmxB | BGC0000408 | NRP | 33.0 | 48.0 | 258.0 | 1.97e-70 |
| ANI24100.1 | nonribosomal\_peptide\_synthetase | BGC0001235 | NRP+Polyketide | 34.0 | 48.2 | 259.0 | 8.31e-70 |
| EAL85113.2 | hybrid\_PKS-NRPS\_enzyme | BGC0001037 | NRP+Polyketide:Iterative type I polyketide | 27.0 | 79.1 | 259.0 | 1.4e-69 |
| AAT28740.1 | FUSS | BGC0000064 | NRP+Polyketide | 28.0 | 77.1 | 257.0 | 4.18e-69 |
| ATV95610.1 | NRPS\_A-PCP\_didomain\_protein | BGC0001503 | Polyketide | 32.0 | 47.5 | 244.0 | 4.85e-69 |
| ABQ96384.2 | fusaricidin\_synthetase | BGC0001152 | Polyketide+NRP:Lipopeptide | 32.0 | 47.9 | 257.0 | 5.02e-69 |
| AWI62628.1 | nonribosomal\_peptide\_synthetase | BGC0001822 | NRP | 33.0 | 48.5 | 256.0 | 6.96e-69 |
| WP\_020993844.1 | non-ribosomal\_peptide\_synthetase | BGC0001575 | NRP | 26.0 | 80.1 | 255.0 | 1.53e-68 |
| AEG64698.1 | LpmD | BGC0000379 | NRP | 33.0 | 49.2 | 255.0 | 1.77e-68 |
| AAY89051.1 | hybrid\_nonribosomal\_peptide\_synthetase/polyketide\_synthase | BGC0001069 | NRP+Polyketide:Trans-AT type I polyketide | 33.0 | 47.9 | 255.0 | 1.82e-68 |
| QDA77059.1 | polyketide\_synthase/nonribosomal\_peptide\_synthetase | BGC0002026 | NRP+Polyketide | 34.0 | 47.8 | 254.0 | 3.64e-68 |
| WP\_003981346.1 | non-ribosomal\_peptide\_synthetase | BGC0001813 | NRP | 32.0 | 52.0 | 254.0 | 5.07e-68 |
| QMS47798.1 | JesB | BGC0001629 | NRP:Lipopeptide | 33.0 | 50.8 | 253.0 | 1.34e-67 |
| WP\_041754829.1 | non-ribosomal\_peptide\_synthetase | BGC0001844 | NRP:Lipopeptide | 33.0 | 49.7 | 253.0 | 1.39e-67 |
| AGI89791.1 | Nonribosomal\_peptide\_synthetase | BGC0001792 | NRP | 33.0 | 49.4 | 252.0 | 2.36e-67 |
| AXN93575.1 | PuwA | BGC0001950 | NRP | 32.0 | 49.0 | 251.0 | 2.45e-67 |
| AXN93584.1 | PuwA | BGC0001951 | NRP | 32.0 | 49.0 | 251.0 | 2.45e-67 |
| WP\_013428324.1 | non-ribosomal\_peptide\_synthetase | BGC0001758 | NRP | 32.0 | 51.4 | 251.0 | 3.29e-67 |
| AXA94654.1 | hypothetical\_protein | BGC0002044 | NRP | 32.0 | 49.6 | 248.0 | 4.21e-67 |
| QUJ09167.1 | Lon20 | BGC0002440 | NRP | 33.0 | 47.5 | 251.0 | 5.02e-67 |
| CDG17981.1 | Non-ribosomal\_peptide\_synthetase | BGC0000464 | NRP:Cyclic depsipeptide | 32.0 | 48.6 | 250.0 | 6.33e-67 |
| QPB41097.1 | non-ribosomal\_peptide\_synthetase | BGC0002503 | NRP+Polyketide | 32.0 | 53.0 | 250.0 | 6.81e-67 |
| QPB41096.1 | non-ribosomal\_peptide\_synthetase | BGC0002503 | NRP+Polyketide | 32.0 | 52.9 | 250.0 | 7.32e-67 |
| CBW75453.1 | Non-ribosomal\_peptide\_synthetase\_modules\_(EC\_6.3.2.-) | BGC0002048 | NRP:Cyclic depsipeptide | 33.0 | 51.0 | 250.0 | 7.47e-67 |
| AEF33078.1 | dimodular\_nonribosomal\_peptide\_synthetase | BGC0001039 | NRP+Polyketide | 34.0 | 49.0 | 249.0 | 8.06e-67 |
| BAV56271.1 |  | BGC0001657 | NRP | 33.0 | 47.9 | 250.0 | 9.77e-67 |
| WP\_011146892.1 | non-ribosomal\_peptide\_synthetase | BGC0001641 | NRP | 33.0 | 47.9 | 250.0 | 1.09e-66 |
| ACG60772.1 | NRPS(C/A/PCP/Cy/A/PCP/Cy) | BGC0001058 | NRP:Glycopeptide+Polyketide:Modular type I polyketide+Saccharide:Hybrid/tailoring saccharide | 35.0 | 49.7 | 249.0 | 1.24e-66 |
| CAJ14037.1 | peptide\_synthetase | BGC0000406 | NRP | 33.0 | 52.1 | 247.0 | 1.46e-66 |
| QMS47800.1 | JesC | BGC0001629 | NRP:Lipopeptide | 33.0 | 51.6 | 249.0 | 2.41e-66 |
| ACS68554.1 | hybrid\_PKS-NRPS\_protein | BGC0001026 | NRP+Polyketide | 27.0 | 77.9 | 248.0 | 2.62e-66 |
| CAY48788.1 | putative\_non-ribosomal\_peptide\_synthetase | BGC0001312 | NRP | 33.0 | 48.3 | 248.0 | 2.69e-66 |
| QWP75304.1 | non-ribosomal\_peptide\_synthase | BGC0002126 | NRP:Cyclic depsipeptide | 33.0 | 48.2 | 248.0 | 3.61e-66 |
| AWX24482.1 | non-ribosomal\_peptide\_synthetase | BGC0001695 | NRP | 32.0 | 48.6 | 248.0 | 4.83e-66 |
| AGE11898.1 | nonribosomal\_peptide\_synthetase | BGC0000366 | NRP | 34.0 | 47.8 | 247.0 | 4.95e-66 |
| EAU38971.1 | PKS-NRPS\_hybrid | BGC0001122 | NRP+Polyketide:Iterative type I polyketide | 28.0 | 78.5 | 247.0 | 6.01e-66 |
| QPC57090.1 | polyketide\_synthase-nonribosomal\_peptide\_synthetase | BGC0002230 | Polyketide+NRP | 27.0 | 74.1 | 247.0 | 6.09e-66 |
| OJJ98497.1 | hypothetical\_protein | BGC0002169 | Polyketide+NRP | 27.0 | 77.6 | 246.0 | 6.55e-66 |
| AEH59100.1 | amino\_acid\_adenylation\_domain-containing\_protein/NRPS | BGC0000385 | NRP | 34.0 | 52.5 | 247.0 | 7.16e-66 |
| EED49862.1 | hybrid\_PKS/NRPS\_enzyme,\_putative | BGC0001445 | NRP+Polyketide:Iterative type I polyketide | 27.0 | 78.3 | 247.0 | 7.99e-66 |
| AAF86395.1 | FkbP | BGC0000994 | NRP+Polyketide | 33.0 | 49.1 | 245.0 | 1.06e-65 |
| BBD17759.1 | non-ribosomal\_peptide\_synthetase | BGC0001919 | NRP+Polyketide | 32.0 | 52.6 | 246.0 | 1.11e-65 |
| QPB41098.1 | non-ribosomal\_peptide\_synthetase | BGC0002503 | NRP+Polyketide | 34.0 | 48.2 | 245.0 | 1.15e-65 |
| PHM49485.1 | Amino\_acid\_adenylation | BGC0001131 | NRP | 34.0 | 48.2 | 246.0 | 1.2e-65 |
| AAC44128.1 | saframycin\_Mx1\_synthetase\_B | BGC0002706 | NRP | 34.0 | 46.7 | 245.0 | 1.41e-65 |
| ALG65317.1 | Cal19 | BGC0001297 | NRP | 33.0 | 48.6 | 245.0 | 1.53e-65 |
| QKF54436.1 | nonribosomal\_peptide\_synthetase | BGC0002581 | NRP | 34.0 | 48.2 | 246.0 | 1.69e-65 |
| ABM34278.1 | amino\_acid\_adenylation\_domain\_protein | BGC0002419 | NRP+Polyketide | 33.0 | 48.6 | 243.0 | 1.85e-65 |
| ABS74207.1 | fengycin\_synthetase\_C | BGC0001095 | NRP | 31.0 | 48.2 | 245.0 | 1.97e-65 |
| AHZ34238.1 | CipA | BGC0001389 | NRP | 32.0 | 49.8 | 245.0 | 2.59e-65 |
| AFH75329.1 | nonribosomal\_peptide\_synthetase | BGC0000398 | NRP:Cyclic depsipeptide | 32.0 | 48.0 | 245.0 | 3.32e-65 |
| AYA44686.1 | icosalide\_NRPS | BGC0001833 | NRP:Lipopeptide | 32.0 | 49.3 | 245.0 | 3.44e-65 |
| ctg1\_orf17 |  | BGC0001457 | NRP | 32.0 | 49.3 | 241.0 | 4.95e-65 |
| WP\_018540603.1 | non-ribosomal\_peptide\_synthetase | BGC0001332 | NRP+Polyketide | 33.0 | 47.5 | 241.0 | 5.05e-65 |
| BAV56270.1 |  | BGC0001657 | NRP | 34.0 | 48.7 | 244.0 | 5.07e-65 |
| antaC | NRPS | BGC0001455 | NRP+Polyketide | 32.0 | 53.3 | 244.0 | 6.55e-65 |
| CBW75452.1 | Non-ribosomal\_peptide\_synthetase\_modules\_(EC\_6.3.2.-) | BGC0002048 | NRP:Cyclic depsipeptide | 31.0 | 51.6 | 243.0 | 7.48e-65 |
| BCJ07599.1 | hypothetical\_protein | BGC0002379 | NRP | 32.0 | 50.1 | 240.0 | 9e-65 |
| ATY37608.1 | BreC | BGC0001536 | NRP | 29.0 | 47.9 | 244.0 | 9.1e-65 |
| QIH29228.1 | endopyrrole\_NRPS\_A | BGC0002326 | NRP | 31.0 | 51.6 | 243.0 | 1.06e-64 |
| MCF2151708.1 | Non-ribosomal\_peptide\_synthetase | BGC0002625 | NRP+Polyketide | 32.0 | 49.3 | 243.0 | 1.46e-64 |
| QBA57737.1 | NRPS | BGC0002377 | NRP | 33.0 | 48.6 | 243.0 | 1.51e-64 |
| AEH59099.1 | amino\_acid\_adenylation\_domain-containing\_protein/NRPS | BGC0000385 | NRP | 32.0 | 50.4 | 243.0 | 1.68e-64 |
| ANS62966.1 | non-ribosomal\_peptide\_synthetase | BGC0001567 | NRP | 31.0 | 52.7 | 241.0 | 1.81e-64 |
| WP\_100939443.1 | non-ribosomal\_peptide\_synthetase | BGC0002071 | NRP:Lipopeptide | 33.0 | 48.5 | 243.0 | 1.89e-64 |
| EPS34234.1 | nonribosomal\_peptide\_synthatase-polyketide\_synthase | BGC0002067 | NRP+Polyketide:Iterative type I polyketide | 27.0 | 75.5 | 242.0 | 2.25e-64 |
| CAQ71828.1 | non\_ribosomal\_peptide\_synthase,\_antibiotic\_synthesis;\_contains\_3\_condensation\_domains,\_2\_AMP-acid\_ligases\_II\_domains,\_2\_PP-binding,\_Phosphopantetheine\_attachment\_site | BGC0001189 | NRP | 34.0 | 49.8 | 242.0 | 2.44e-64 |
| NKI69295.1 | amino\_acid\_adenylation\_domain-containing\_protein | BGC0002408 | NRP | 32.0 | 48.4 | 242.0 | 2.74e-64 |
| CCJ67637.1 | TaaB | BGC0000447 | NRP:Lipopeptide | 33.0 | 49.0 | 242.0 | 2.76e-64 |
| EFE73312.1 | nonribosomal\_peptide\_synthetase | BGC0000431 | NRP:Cyclic depsipeptide | 32.0 | 49.1 | 242.0 | 3.32e-64 |
| QUS58939.1 | amino\_acid\_adenylation\_domain-containing\_protein | BGC0002123 | NRP+Polyketide | 31.0 | 49.0 | 241.0 | 3.35e-64 |
| CDG17980.1 | Putative\_Ornithine\_racemase\_(fragment) | BGC0000464 | NRP:Cyclic depsipeptide | 32.0 | 49.0 | 242.0 | 3.45e-64 |
| ADA69239.2 | trans-AT\_hybrid\_polyketide\_synthase-NRPS | BGC0001071 | NRP+Polyketide:Modular type I polyketide+Polyketide:Trans-AT type I polyketide | 31.0 | 49.5 | 242.0 | 3.49e-64 |
| KFL51887.1 | amino\_acid\_adenylation\_protein | BGC0001711 | NRP+Polyketide | 33.0 | 46.9 | 241.0 | 3.59e-64 |
| CAD29799.1 | microcystin\_synthetase | BGC0001015 | NRP+Polyketide | 32.0 | 48.2 | 240.0 | 3.61e-64 |
| WP\_030498974.1 | tyrocidine\_synthase\_3 | BGC0001327 | NRP:Cyclic depsipeptide+Polyketide:Modular type I polyketide | 34.0 | 49.8 | 239.0 | 4.11e-64 |
| QBC75022.1 | non-ribosomal\_peptide\_synthetase | BGC0001968 | NRP | 33.0 | 50.2 | 241.0 | 4.33e-64 |
| UEF20578.1 | nonribosomal\_peptide\_synthetase | BGC0002360 | NRP | 33.0 | 48.2 | 241.0 | 4.76e-64 |
| CDG17982.1 | Non-ribosomal\_peptide\_synthetase | BGC0000464 | NRP:Cyclic depsipeptide | 33.0 | 47.9 | 241.0 | 5.52e-64 |
| ABS74209.1 | fengycin\_synthetase\_A | BGC0001095 | NRP | 31.0 | 48.4 | 241.0 | 5.57e-64 |
| ACO78745.1 | Non-ribosomal\_peptide\_synthase:Amino\_acid\_adenylation | BGC0002433 | NRP | 32.0 | 50.0 | 241.0 | 7.02e-64 |
| AEW31019.1 | plipastatin\_synthetase | BGC0000407 | NRP | 32.0 | 50.2 | 240.0 | 7.36e-64 |
| QEO75075.1 | condensation\_domain-containing\_protein | BGC0002079 | NRP:Cyclic depsipeptide | 33.0 | 48.0 | 241.0 | 7.48e-64 |
| SJZ83675.1 | non-ribosomal\_peptide\_synthase\_domain\_TIGR01720/amino\_acid\_adenylation\_domain-containing\_protein/thioester\_reductase\_domain-containing\_protein | BGC0002660 | NRP | 27.0 | 76.8 | 241.0 | 7.65e-64 |
| AKJ15827.1 | peptide\_synthetase | BGC0002735 | Polyketide+NRP | 33.0 | 49.2 | 240.0 | 9.79e-64 |
| CCJ67648.1 | JagD | BGC0001127 | NRP | 33.0 | 50.1 | 240.0 | 1.05e-63 |
| AEW31021.1 | plipastatin\_synthetase | BGC0000407 | NRP | 32.0 | 50.1 | 239.0 | 1.28e-63 |
| KYC41483.1 | hypothetical\_protein | BGC0002484 | NRP+Polyketide | 31.0 | 47.9 | 237.0 | 1.76e-63 |
| CAJ14039.1 | peptide\_synthetase | BGC0000406 | NRP | 32.0 | 49.4 | 236.0 | 1.83e-63 |
| CUX96954.1 | TmcG | BGC0001829 | NRP+Polyketide | 33.0 | 48.5 | 239.0 | 1.98e-63 |
| BAP05597.1 | calI | BGC0000967 | NRP+Polyketide:Trans-AT type I polyketide | 32.0 | 49.1 | 239.0 | 2.3e-63 |
| BAV57443.1 | NRPS\_(C-A-PCP-TE) | BGC0001818 | NRP | 33.0 | 49.0 | 238.0 | 2.4e-63 |
| QGY73453.1 | Itm21 | BGC0002451 | Polyketide | 33.0 | 52.2 | 239.0 | 2.45e-63 |
| APZ78756.1 | nonribosomal\_peptide\_synthetase | BGC0001423 | NRP:Cyclic depsipeptide+Polyketide:Iterative type I polyketide | 31.0 | 51.7 | 239.0 | 2.59e-63 |
| CAB15186.3 | siderophore\_2,3-dihydroxybenzoate-glycine-threonine\_trimeric\_ester\_bacillibactin\_synthetase | BGC0000309 | NRP | 31.0 | 49.4 | 238.0 | 2.81e-63 |
| AGU50951.1 | putative\_non-ribosomal\_peptide\_synthetase | BGC0002417 | NRP+Polyketide | 32.0 | 48.7 | 236.0 | 2.86e-63 |
| BCD58482.1 | gamma-poly-L-2,4-diaminobutyric\_acid\_synthetase | BGC0002535 | NRP | 32.0 | 48.2 | 237.0 | 2.92e-63 |
| AXA91301.1 | non-ribosomal\_peptide\_synthetase | BGC0002044 | NRP | 30.0 | 59.1 | 238.0 | 3.08e-63 |
| AEO14744.1 | NdaB | BGC0000396 | NRP | 31.0 | 48.0 | 237.0 | 3.25e-63 |
| AXM43052.1 | non-ribosomal\_peptide\_synthetase | BGC0001945 | NRP | 33.0 | 48.3 | 238.0 | 3.43e-63 |
| BAV57451.1 | NRPS\_(A-PCP) | BGC0001818 | NRP | 32.0 | 47.0 | 228.0 | 3.69e-63 |
| BBD17741.1 | non-ribosomal\_peptide\_synthetase | BGC0001918 | NRP+Polyketide | 32.0 | 48.6 | 238.0 | 3.77e-63 |
| ABS74180.1 | bacillomycin\_D\_synthetase\_B | BGC0001090 | Polyketide+NRP:Lipopeptide | 32.0 | 47.5 | 238.0 | 3.93e-63 |
| EWM63002.1 | non-ribosomal\_peptide\_synthetase | BGC0001328 | NRP:Cyclic depsipeptide+Polyketide:Modular type I polyketide | 34.0 | 49.8 | 236.0 | 4.02e-63 |
| WP\_078586793.1 | non-ribosomal\_peptide\_synthetase | BGC0001760 | NRP | 33.0 | 48.6 | 237.0 | 4.23e-63 |
| ABS75232.1 | DhbF | BGC0001185 | NRP:NRP siderophore | 32.0 | 49.5 | 238.0 | 4.89e-63 |
| ACS20360.1 | amino\_acid\_adenylation\_domain\_protein | BGC0002420 | NRP+Polyketide | 31.0 | 48.6 | 235.0 | 4.97e-63 |
| CBL93730.1 | NRPS | BGC0000360 | NRP | 34.0 | 50.0 | 236.0 | 5.6e-63 |
| WP\_004571779.1 | non-ribosomal\_peptide\_synthetase | BGC0001760 | NRP | 33.0 | 48.5 | 234.0 | 5.71e-63 |
| CBJ90082.1 | Non\_Ribosomal\_peptide\_synthetase\_(-succinylbenzoate--CoA\_ligase) | BGC0001132 | NRP | 32.0 | 49.8 | 238.0 | 6.38e-63 |
| SDF67386.1 | non-ribosomal\_peptide\_synthase\_domain\_TIGR01720/amino\_acid\_adenylation\_domain-containing\_protein | BGC0002422 | NRP | 32.0 | 49.3 | 238.0 | 6.59e-63 |
| CDG17985.1 | Putative\_Ornithine\_racemase\_(fragment) | BGC0000464 | NRP:Cyclic depsipeptide | 32.0 | 48.7 | 238.0 | 7.24e-63 |
| QNH85840.1 | BolH | BGC0002327 | NRP | 33.0 | 51.4 | 237.0 | 8.65e-63 |
| AKP45395.1 | CysG | BGC0001413 | NRP | 31.0 | 48.9 | 236.0 | 9.71e-63 |
| ADJ63842.1 | Serobactin\_synthetase | BGC0000424 | NRP:NRP siderophore | 31.0 | 52.7 | 237.0 | 9.86e-63 |
| EPH46597.1 | putative\_Linear\_gramicidin\_synthase\_subunit\_C | BGC0001519 | NRP+Polyketide | 33.0 | 47.7 | 236.0 | 1.19e-62 |
| AUD11994.1 | OrbI | BGC0001721 | NRP | 33.0 | 51.1 | 236.0 | 1.32e-62 |
| CCJ67636.1 | TaaA | BGC0000447 | NRP:Lipopeptide | 30.0 | 50.1 | 236.0 | 1.33e-62 |
| CAR51994.1 | ornibactin\_biosynthesis\_non-ribosomal\_peptide\_synthase | BGC0002569 | NRP | 32.0 | 50.7 | 236.0 | 1.33e-62 |
| AIG26884.1 | NRPS\_domain-containing\_protein | BGC0002432 | NRP | 31.0 | 47.9 | 237.0 | 1.34e-62 |
| AAU34203.1 | mannopeptimycin\_peptide\_synthetase\_MppB | BGC0000388 | NRP | 34.0 | 50.9 | 236.0 | 1.41e-62 |
| AGI87384.1 | Peptide\_synthase | BGC0002358 | Polyketide | 32.0 | 46.4 | 236.0 | 1.47e-62 |
| CDG17987.1 | Putative\_Ornithine\_racemase\_(fragment) | BGC0000464 | NRP:Cyclic depsipeptide | 31.0 | 48.2 | 236.0 | 1.54e-62 |
| CAE15637.1 |  | BGC0001128 | NRP | 31.0 | 47.9 | 236.0 | 1.56e-62 |
| AXG48275.1 | non-ribosomal\_peptide\_synthetase | BGC0002716 | NRP | 31.0 | 47.9 | 236.0 | 1.56e-62 |
| BAF50711.1 | non\_ribosomal\_peptide\_synthetase\_for\_virginiamycin\_S | BGC0001116 | NRP+Polyketide | 32.0 | 51.7 | 236.0 | 1.6e-62 |
| ACY06285.1 | non-ribosomal\_peptide\_synthetase | BGC0001042 | NRP+Polyketide | 34.0 | 50.8 | 236.0 | 1.84e-62 |
| AQH32486.1 | peptide\_synthetase | BGC0001667 | NRP+Polyketide | 31.0 | 47.9 | 234.0 | 2.34e-62 |
| ABV56585.1 | KtzE | BGC0000378 | NRP | 31.0 | 47.0 | 235.0 | 2.34e-62 |
| ABC36785.1 | peptide\_synthetase,\_putative | BGC0000964 | NRP:Cyclic depsipeptide+Polyketide:Trans-AT type I polyketide | 32.0 | 50.5 | 236.0 | 2.44e-62 |
| WP\_012988806.1 | non-ribosomal\_peptide\_synthetase | BGC0002135 | NRP:Lipopeptide | 30.0 | 54.5 | 236.0 | 2.49e-62 |
| ACS20362.1 | amino\_acid\_adenylation\_domain\_protein | BGC0002420 | NRP+Polyketide | 32.0 | 48.5 | 235.0 | 2.6e-62 |
| KYC42612.1 | hypothetical\_protein | BGC0002484 | NRP+Polyketide | 31.0 | 48.3 | 236.0 | 2.77e-62 |
| AAN85512.1 | nonribosomal\_peptide\_synthetase | BGC0001101 | NRP+Polyketide:Modular type I polyketide+Polyketide:Trans-AT type I polyketide | 32.0 | 48.0 | 234.0 | 3.36e-62 |
| CDF96614.1 | NRPS | BGC0001149 | NRP:Lipopeptide+Saccharide:Hybrid/tailoring saccharide | 31.0 | 52.6 | 235.0 | 3.45e-62 |
| CBW54672.1 | non\_ribosomal\_peptide\_synthetase | BGC0000971 | NRP+Polyketide:Modular type I polyketide | 32.0 | 47.3 | 224.0 | 4.22e-62 |
| CDG17986.1 | Non-ribosomal\_peptide\_synthetase | BGC0000464 | NRP:Cyclic depsipeptide | 30.0 | 48.7 | 234.0 | 4.31e-62 |
| AKJ29412.1 | peptide\_synthetase | BGC0001608 | NRP | 31.0 | 50.3 | 235.0 | 4.86e-62 |
| AJF34464.1 | Txo2 | BGC0001207 | NRP | 33.0 | 49.8 | 235.0 | 4.92e-62 |
| AGZ03651.1 | sevB | BGC0000426 | NRP | 30.0 | 48.8 | 234.0 | 4.97e-62 |
| ctg1\_orf1265 |  | BGC0001752 | NRP | 33.0 | 50.6 | 234.0 | 5.35e-62 |
| WA1\_15570 | hypothetical\_protein | BGC0002484 | NRP+Polyketide | 31.0 | 49.4 | 234.0 | 5.49e-62 |
| AEH41794.1 | HrmP | BGC0000374 | NRP:Cyclic depsipeptide | 34.0 | 50.2 | 234.0 | 5.5e-62 |
| NAO96319.1 | amino\_acid\_adenylation\_domain-containing\_protein | BGC0002117 | NRP | 31.0 | 48.8 | 234.0 | 5.58e-62 |
| QNL14922.1 | AptA | BGC0002512 | NRP | 29.0 | 48.9 | 234.0 | 5.61e-62 |
| BAC67536.1 | arthrofactin\_synthetase\_C | BGC0000305 | NRP:Lipopeptide | 32.0 | 51.3 | 234.0 | 6.39e-62 |
| AFJ14794.1 | PlpE | BGC0000403 | NRP | 31.0 | 47.6 | 234.0 | 6.78e-62 |
| APO47825.1 | non-ribosomal\_peptide\_synthetase | BGC0002653 | NRP | 32.0 | 48.0 | 231.0 | 7.74e-62 |
| ATP76244.1 | NdaB | BGC0001705 | NRP+Polyketide | 32.0 | 47.9 | 232.0 | 9.51e-62 |
| AWI62626.1 | nonribosomal\_peptide\_synthetase | BGC0001822 | NRP | 32.0 | 45.9 | 234.0 | 1.07e-61 |
| ALV82356.1 | CDA\_peptide\_synthetase\_I | BGC0001370 | NRP | 32.0 | 48.7 | 234.0 | 1.15e-61 |
| AXN93592.1 | PuwA | BGC0001952 | NRP | 30.0 | 49.0 | 233.0 | 1.15e-61 |
| QRI43520.1 | NRPS/PKS\_hybrid | BGC0002454 | Polyketide | 33.0 | 49.4 | 233.0 | 1.28e-61 |
| WP\_126241403.1 | non-ribosomal\_peptide\_synthetase | BGC0002336 | NRP | 33.0 | 51.4 | 233.0 | 1.38e-61 |
| AAC68816.1 | FK506\_peptide\_synthetase | BGC0000353 | NRP | 32.0 | 52.1 | 232.0 | 1.41e-61 |
| WP\_012408786.1 | non-ribosomal\_peptide\_synthetase | BGC0002061 | NRP:Cyclic depsipeptide+Polyketide:Modular type I polyketide | 30.0 | 50.0 | 233.0 | 1.42e-61 |
| AXN93605.1 | PuwA | BGC0001953 | NRP | 31.0 | 49.4 | 233.0 | 1.52e-61 |
| APU91750.1 | Non-Ribosomal\_Peptide\_Synthetase | BGC0001806 | NRP | 32.0 | 48.3 | 233.0 | 1.62e-61 |
| QIE08736.1 | non-ribosomal\_peptide\_synthetase | BGC0002544 | NRP | 33.0 | 47.9 | 233.0 | 1.72e-61 |
| KON97028.1 | phenylalanine\_racemase | BGC0002122 | NRP | 30.0 | 49.4 | 233.0 | 1.81e-61 |
| AHF21228.1 | TriD | BGC0000449 | NRP | 31.0 | 48.6 | 233.0 | 2.15e-61 |
| ABD65956.1 | nonribosomal\_peptide\_synthetase | BGC0000341 | NRP | 33.0 | 47.9 | 232.0 | 2.16e-61 |
| CAL80824.1 | NRPS\_module\_protein | BGC0000997 | NRP+Polyketide | 31.0 | 51.7 | 230.0 | 2.29e-61 |
| UHJ79951.1 | non-ribosomal\_peptide\_synthetase | BGC0002654 | NRP | 32.0 | 49.4 | 233.0 | 2.5e-61 |
| NAO96318.1 | amino\_acid\_adenylation\_domain-containing\_protein | BGC0002117 | NRP | 31.0 | 49.3 | 232.0 | 2.92e-61 |
| AEZ51516.1 | pmxA | BGC0001153 | NRP:Lipopeptide | 31.0 | 49.4 | 232.0 | 3.23e-61 |
| QMN69933.1 | PsoB | BGC0002521 | NRP | 32.0 | 48.3 | 232.0 | 3.5e-61 |
| ABW17376.1 | PsoB | BGC0000411 | NRP | 32.0 | 49.2 | 232.0 | 3.5e-61 |
| AYJ71720.1 | non-ribosomal\_peptide\_synthetase | BGC0001942 | NRP+Polyketide | 33.0 | 51.5 | 231.0 | 3.81e-61 |
| BBA21073.1 | putative\_non-ribosomal\_peptide\_synthetase | BGC0001740 | NRP+Polyketide | 31.0 | 55.0 | 232.0 | 3.84e-61 |
| ATD51279.1 | nonribosomal\_peptide\_synthase | BGC0001650 | NRP | 34.0 | 51.9 | 231.0 | 4.01e-61 |
| AGE11899.1 | nonribosomal\_peptide\_synthetase | BGC0000366 | NRP | 33.0 | 49.2 | 231.0 | 4.09e-61 |
| AED90003.1 | non-ribosomal\_peptide\_synthetase\_ThaB | BGC0000443 | NRP:Beta-lactam | 30.0 | 49.6 | 232.0 | 4.16e-61 |
| ABA73956.1 | putative\_non-ribosomal\_peptide\_synthetase | BGC0001842 | NRP:Lipopeptide | 33.0 | 48.8 | 232.0 | 4.24e-61 |
| QRG35014.1 | NRPS | BGC0002378 | NRP | 31.0 | 49.8 | 232.0 | 4.27e-61 |
| ANS62967.1 | non-ribosomal\_peptide\_synthase/amino\_acid\_adenylation\_enzyme | BGC0001567 | NRP | 33.0 | 52.6 | 231.0 | 4.35e-61 |
| APZ78729.1 | nonribosomal\_peptide\_synthetase | BGC0001421 | NRP:Cyclic depsipeptide+Polyketide:Iterative type I polyketide | 34.0 | 49.3 | 232.0 | 4.36e-61 |
| DAC80524.1 | peptide\_synthetase | BGC0001841 | NRP+Polyketide | 32.0 | 47.9 | 230.0 | 4.78e-61 |
| BAH22765.1 | nonribosomal\_peptide\_synthetase | BGC0001018 | NRP | 29.0 | 48.9 | 230.0 | 4.79e-61 |
| WP\_018540607.1 | non-ribosomal\_peptide\_synthetase | BGC0001332 | NRP+Polyketide | 34.0 | 47.0 | 231.0 | 4.8e-61 |
| AAY32966.1 | DszC | BGC0001093 | NRP+Polyketide | 32.0 | 49.0 | 231.0 | 5.2e-61 |
| QBG38782.1 | Atr21 | BGC0001975 | NRP | 33.0 | 48.2 | 231.0 | 5.26e-61 |
| QBQ12465.1 | amino\_acid\_adenylation\_domain-containing\_protein | BGC0002693 | NRP | 31.0 | 47.5 | 231.0 | 5.34e-61 |
| ABM34280.1 | amino\_acid\_adenylation\_domain\_protein | BGC0002419 | NRP+Polyketide | 31.0 | 49.5 | 231.0 | 5.51e-61 |
| QTT72092.1 | amino\_acid\_adenylation\_domain-containing\_protein | BGC0002350 | NRP+Polyketide+Saccharide | 33.0 | 49.1 | 231.0 | 5.81e-61 |
| WP\_064118559.1 | non-ribosomal\_peptide\_synthase/polyketide\_synthase | BGC0001509 | NRP | 32.0 | 49.8 | 231.0 | 5.84e-61 |
| BBA84067.1 | type\_I\_polyketide\_synthase | BGC0001649 | Polyketide | 35.0 | 39.4 | 231.0 | 5.9e-61 |
| AQM37584.1 | nonribosomal\_peptide\_synthetase | BGC0001424 | NRP:Cyclic depsipeptide+Polyketide:Iterative type I polyketide | 31.0 | 50.6 | 231.0 | 6.6e-61 |
| UEF20580.1 | nonribosomal\_peptide\_synthetase | BGC0002360 | NRP | 32.0 | 48.2 | 231.0 | 6.94e-61 |
| ABP55169.1 | amino\_acid\_adenylation\_domain | BGC0000150 | NRP+Polyketide:Enediyne type I polyketide | 32.0 | 48.7 | 220.0 | 7.22e-61 |
| AGU50953.1 | putative\_non-ribosomal\_peptide\_synthetase | BGC0002417 | NRP+Polyketide | 31.0 | 48.2 | 230.0 | 7.24e-61 |
| AAC82550.1 | FxbC | BGC0000351 | NRP | 34.0 | 47.7 | 231.0 | 7.41e-61 |
| KPN93063.1 | NupA | BGC0001416 | NRP | 33.0 | 47.6 | 231.0 | 8.28e-61 |
| CBW75451.1 | Non-ribosomal\_peptide\_synthetase\_modules | BGC0002048 | NRP:Cyclic depsipeptide | 32.0 | 48.2 | 231.0 | 8.81e-61 |
| QIH29229.1 | endopyrrole\_NRPS\_B | BGC0002326 | NRP | 32.0 | 48.2 | 231.0 | 8.81e-61 |
| QKF54438.1 | nonribosomal\_peptide\_synthetase | BGC0002581 | NRP | 32.0 | 48.2 | 231.0 | 9.16e-61 |
| AAY91420.2 | non-ribosomal\_peptide\_synthetase\_OfaB | BGC0000399 | NRP:Cyclic depsipeptide | 31.0 | 48.4 | 231.0 | 9.44e-61 |
| CBA63680.1 | nonribosomal\_peptide\_synthetase\_NRPS | BGC0000368 | NRP | 32.0 | 49.0 | 230.0 | 9.71e-61 |
| QWM97320.1 | non-ribosomal\_peptide\_synthetase | BGC0002384 | NRP | 33.0 | 47.7 | 231.0 | 1.03e-60 |
| AHB38515.1 | non-ribosomal\_peptide\_synthetase | BGC0000345 | NRP+Polyketide:Modular type I polyketide | 33.0 | 49.2 | 230.0 | 1.15e-60 |
| ACZ55943.1 | non-ribosomal\_peptide\_synthetase | BGC0000302 | NRP | 30.0 | 49.3 | 228.0 | 1.17e-60 |
| AGM16413.1 | paenibacterin\_synthetase\_B | BGC0000400 | NRP | 31.0 | 49.4 | 230.0 | 1.36e-60 |
| BAH22763.1 | nonribosomal\_peptide\_synthetase | BGC0001018 | NRP | 29.0 | 50.8 | 228.0 | 1.42e-60 |
| UHJ79953.1 | non-ribosomal\_peptide\_synthetase | BGC0002654 | NRP | 32.0 | 48.2 | 230.0 | 1.46e-60 |
| BAY02138.1 | peptide\_synthetase | BGC0002532 | NRP+Polyketide | 32.0 | 48.6 | 227.0 | 1.56e-60 |
| BAX90000.1 | Non-ribosomal\_peptide\_synthetase | BGC0001628 | NRP | 32.0 | 46.8 | 230.0 | 1.57e-60 |
| KFL51883.1 | amino\_acid\_adenylation\_protein | BGC0001711 | NRP+Polyketide | 32.0 | 48.1 | 230.0 | 1.65e-60 |
| AAY37647.1 | Amino\_acid\_adenylation | BGC0000437 | NRP | 33.0 | 47.9 | 230.0 | 1.89e-60 |
| AAF17280.1 | nosC | BGC0001028 | Polyketide+NRP:Cyclic depsipeptide | 32.0 | 49.0 | 229.0 | 1.97e-60 |
| AJV88375.1 | MfnC | BGC0001214 | NRP | 31.0 | 49.0 | 229.0 | 2.05e-60 |
| AJD47484.1 | protein\_PvdD | BGC0002418 | NRP+Polyketide | 32.0 | 48.0 | 229.0 | 2.1e-60 |
| KJY85279.1 | long-chain\_fatty\_acid--CoA\_ligase | BGC0002491 | NRP | 27.0 | 76.8 | 228.0 | 2.1e-60 |
| NPC94426.1 | amino\_acid\_adenylation\_domain-containing\_protein | BGC0002695 | NRP | 31.0 | 49.5 | 229.0 | 2.17e-60 |
| CUX79062.1 | Octapeptin\_synthase\_subunit\_C | BGC0001715 | NRP | 30.0 | 47.9 | 227.0 | 2.23e-60 |
| AXA91302.1 | non-ribosomal\_peptide\_synthetase | BGC0002044 | NRP | 30.0 | 53.7 | 229.0 | 2.27e-60 |
| QUS58937.1 | non-ribosomal\_peptide\_synthetase | BGC0002123 | NRP+Polyketide | 30.0 | 47.9 | 228.0 | 2.48e-60 |
| SDF67417.1 | amino\_acid\_adenylation\_domain-containing\_protein | BGC0002422 | NRP | 31.0 | 50.6 | 229.0 | 2.49e-60 |
| ctg1\_orf1264 |  | BGC0001752 | NRP | 31.0 | 56.9 | 229.0 | 2.84e-60 |
| AJW65406.1 | nonribosomal\_peptide\_synthetase | BGC0001195 | NRP+Polyketide | 32.0 | 47.3 | 228.0 | 3.15e-60 |
| AXN93580.1 | PuwE | BGC0001950 | NRP | 31.0 | 47.9 | 229.0 | 3.33e-60 |
| AXN93589.1 | PuwE | BGC0001951 | NRP | 31.0 | 47.9 | 229.0 | 3.33e-60 |
| BAW27670.1 | NRPS(A-T) | BGC0001764 | NRP | 30.0 | 49.8 | 218.0 | 3.4e-60 |
| AAZ03552.1 | McnC | BGC0000332 | NRP | 30.0 | 48.8 | 229.0 | 3.84e-60 |
| AAY91421.3 | non-ribosomal\_peptide\_synthetase\_OfaC | BGC0000399 | NRP:Cyclic depsipeptide | 30.0 | 51.1 | 229.0 | 3.87e-60 |
| AFH75320.1 | nonribosomal\_peptide\_synthetase | BGC0000425 | NRP:Cyclic depsipeptide | 32.0 | 51.4 | 229.0 | 4.1e-60 |
| QWP75305.1 | non-ribosomal\_peptide\_synthase | BGC0002126 | NRP:Cyclic depsipeptide | 32.0 | 47.6 | 229.0 | 4.11e-60 |
| CAK15815.1 | putative\_non\_ribosomal\_peptide\_synthetase | BGC0000344 | NRP | 32.0 | 48.4 | 229.0 | 4.27e-60 |
| AKJ29410.1 | peptide\_synthetase | BGC0001608 | NRP | 32.0 | 47.1 | 228.0 | 4.46e-60 |
| ABM21572.1 | crpD | BGC0000975 | NRP+Polyketide | 28.0 | 53.3 | 228.0 | 4.52e-60 |
| QNN94286.1 | EmeB | BGC0002555 | NRP+Polyketide | 27.0 | 79.4 | 229.0 | 4.57e-60 |
| ABS74206.1 | fengycin\_synthetase\_D | BGC0001095 | NRP | 29.0 | 49.4 | 228.0 | 4.65e-60 |
| CAY48789.1 | putative\_non-ribosomal\_peptide\_synthetase | BGC0001312 | NRP | 32.0 | 49.0 | 228.0 | 4.73e-60 |
| QED55422.1 | nonribosomal\_peptide\_synthetase | BGC0001984 | NRP | 34.0 | 49.5 | 228.0 | 4.9e-60 |
| ADY76664.1 | non-ribosomal\_peptide\_synthetase | BGC0000950 | NRP:Uridylpeptide+Other:Nucleoside | 32.0 | 48.6 | 223.0 | 5.75e-60 |
| AAZ03551.1 | McnB | BGC0000332 | NRP | 29.0 | 50.5 | 226.0 | 5.86e-60 |
| AUD11993.1 | OrbJ | BGC0001721 | NRP | 28.0 | 65.0 | 227.0 | 6.23e-60 |
| CBF80487.1 | hybrid\_PKS-NRPS\_(Eurofung) | BGC0000959 | NRP+Polyketide:Iterative type I polyketide | 27.0 | 72.5 | 228.0 | 6.32e-60 |
| ALV86868.1 | Tlo22 | BGC0001406 | NRP | 31.0 | 48.4 | 228.0 | 6.6e-60 |
| CAE52334.1 | non-ribosomal\_peptide\_synthase | BGC0001088 | NRP+Polyketide | 31.0 | 50.0 | 227.0 | 7.4e-60 |
| ABD65958.1 | nonribosomal\_peptide\_synthetase | BGC0000341 | NRP | 34.0 | 48.7 | 228.0 | 7.47e-60 |
| AVI26390.1 | polyketide\_synthase\_/\_nonribosomal\_peptide\_synthase\_hybrid | BGC0001800 | NRP+Polyketide | 33.0 | 46.0 | 228.0 | 8.83e-60 |
| ABA73955.1 | putative\_non-ribosomal\_peptide\_synthetase | BGC0001842 | NRP:Lipopeptide | 30.0 | 51.4 | 228.0 | 9.07e-60 |
| QDF82259.1 | non-ribosomal\_peptide\_synthetase | BGC0001980 | NRP | 31.0 | 50.3 | 228.0 | 9.24e-60 |
| AIW82285.1 | PuwH | BGC0001125 | NRP+Polyketide | 30.0 | 47.9 | 225.0 | 1.02e-59 |
| ACC81021.1 | non-ribosomal\_peptide\_synthetase | BGC0001479 | NRP | 30.0 | 47.9 | 227.0 | 1.07e-59 |
| AWS21279.1 | type\_I\_polyketide\_synthase | BGC0001934 | Polyketide | 34.0 | 39.1 | 226.0 | 1.13e-59 |
| AZY91989.1 | polyketide\_synthase | BGC0002022 | Polyketide | 34.0 | 39.1 | 226.0 | 1.13e-59 |
| AKJ29411.1 | peptide\_synthetase | BGC0001608 | NRP | 31.0 | 47.5 | 227.0 | 1.13e-59 |
| AKJ15828.1 | peptide\_synthetase | BGC0002735 | Polyketide+NRP | 31.0 | 48.7 | 226.0 | 1.13e-59 |
| QNL14921.1 | AptB | BGC0002512 | NRP | 29.0 | 48.5 | 224.0 | 1.13e-59 |
| CAK15814.1 | putative\_non-ribosomal\_peptide\_synthetase,\_terminal\_component | BGC0000344 | NRP | 31.0 | 48.2 | 227.0 | 1.16e-59 |
| ATL73036.1 | amino\_acid\_adenylation\_protein | BGC0001807 | NRP+Polyketide | 31.0 | 49.0 | 227.0 | 1.23e-59 |
| AGO86662.1 | equisetin\_synthetase | BGC0001255 | NRP+Polyketide | 28.0 | 71.3 | 227.0 | 1.45e-59 |
| AAC06346.1 | bacitracin\_synthetase\_1 | BGC0000310 | NRP | 31.0 | 48.0 | 227.0 | 1.56e-59 |
| ACC81022.1 | non-ribosomal\_peptide\_synthetase | BGC0001479 | NRP | 29.0 | 49.9 | 224.0 | 1.57e-59 |
| AJD47481.1 | amino\_acid\_adenylation\_domain-containing\_protein | BGC0002418 | NRP+Polyketide | 32.0 | 47.5 | 224.0 | 1.61e-59 |
| ABM34277.1 | amino\_acid\_adenylation\_domain\_protein | BGC0002419 | NRP+Polyketide | 31.0 | 52.9 | 226.0 | 1.61e-59 |
| KPN90376.1 | NupC | BGC0001416 | NRP | 29.0 | 52.3 | 227.0 | 1.74e-59 |
| CAJ18237.2 | non-ribosomal\_peptide\_synthetase\_B | BGC0000354 | NRP | 33.0 | 48.1 | 226.0 | 1.75e-59 |
| QEO75073.1 | condensation\_domain-containing\_protein | BGC0002079 | NRP:Cyclic depsipeptide | 33.0 | 48.6 | 226.0 | 1.77e-59 |
| AAY93355.1 | non-ribosomal\_peptide\_synthetase\_PvdJ | BGC0000413 | NRP | 30.0 | 51.4 | 226.0 | 1.87e-59 |
| BAD55611.1 | putative\_non-ribosomal\_peptide\_synthetase | BGC0001027 | NRP+Polyketide | 31.0 | 47.7 | 226.0 | 1.91e-59 |
| WP\_039806856.1 | non-ribosomal\_peptide\_synthetase | BGC0002001 | NRP+Polyketide | 31.0 | 47.9 | 226.0 | 2e-59 |
| ACO78738.1 | Non-ribosomal\_peptide\_synthase,\_PvdJ(2)-like\_protein | BGC0002433 | NRP | 32.0 | 48.2 | 226.0 | 2.01e-59 |
| QNH67552.1 | Cip24 | BGC0002108 | NRP | 33.0 | 48.5 | 225.0 | 2.1e-59 |
| KYC42747.1 | hypothetical\_protein | BGC0002484 | NRP+Polyketide | 32.0 | 49.0 | 226.0 | 2.16e-59 |
| WA1\_15565 | non-ribosomal\_peptide\_synthetase | BGC0002484 | NRP+Polyketide | 31.0 | 49.1 | 226.0 | 2.47e-59 |
| AXG47007.1 | non-ribosomal\_peptide\_synthetase | BGC0000383 | NRP+Polyketide:Modular type I polyketide | 29.0 | 48.6 | 223.0 | 2.6e-59 |
| ACA97576.1 | PmxA | BGC0000408 | NRP | 31.0 | 51.3 | 226.0 | 2.68e-59 |
| AEU11003.1 | NpnC | BGC0001029 | NRP+Polyketide | 30.0 | 48.4 | 226.0 | 2.69e-59 |
| ABA23460.1 | Amino\_acid\_adenylation | BGC0000427 | NRP | 30.0 | 48.2 | 221.0 | 2.75e-59 |
| AGN74876.1 | nonribosomal\_peptide\_synthetase | BGC0000459 | NRP:Cyclic depsipeptide+Polyketide:Trans-AT type I polyketide | 32.0 | 50.4 | 226.0 | 2.79e-59 |
| ABL74936.1 | NRPS | BGC0001048 | NRP:Glycopeptide+Polyketide:Modular type I polyketide+Saccharide:Hybrid/tailoring saccharide | 31.0 | 47.9 | 225.0 | 3.18e-59 |
| AOA33121.1 | Nonribosomal\_peptide\_synthetase | BGC0001346 | NRP:Cyclic depsipeptide | 31.0 | 50.2 | 225.0 | 3.18e-59 |
| AAZ03554.1 | McnE | BGC0000332 | NRP | 28.0 | 48.9 | 224.0 | 3.27e-59 |
| APZ78680.1 | nonribosomal\_peptide\_synthetase | BGC0001417 | NRP:Cyclic depsipeptide+Polyketide:Iterative type I polyketide | 33.0 | 48.3 | 226.0 | 3.29e-59 |
| DAB41477.1 | nonribosomal\_peptide\_synthetase | BGC0001230 | NRP:Cyclic depsipeptide+Polyketide:Modular type I polyketide | 31.0 | 48.9 | 226.0 | 3.34e-59 |
| AIG26883.1 | NRPS\_domain-containing\_protein | BGC0002432 | NRP | 30.0 | 48.6 | 226.0 | 3.38e-59 |
| CAQ34921.1 | nonribosomal\_peptide\_synthetase | BGC0000986 | NRP+Polyketide | 31.0 | 50.6 | 224.0 | 3.39e-59 |
| QCQ67881.1 | non-ribosomal\_peptide\_synthetase | BGC0002297 | NRP+Polyketide | 31.0 | 48.1 | 224.0 | 3.46e-59 |
| KGA48739.1 | amino\_acid\_adenylation\_domain\_protein | BGC0002413 | NRP | 32.0 | 48.2 | 225.0 | 3.47e-59 |
| APO47826.1 | hypothetical\_protein | BGC0002653 | NRP | 31.0 | 51.4 | 226.0 | 3.54e-59 |
| ctg1\_orf20 |  | BGC0001767 | NRP | 32.0 | 49.2 | 226.0 | 3.56e-59 |
| ATW47208.1 | non-ribosomal\_peptide\_synthetase | BGC0002466 | NRP | 32.0 | 50.2 | 226.0 | 3.57e-59 |
| QDA77045.1 | polyketide\_synthase/nonribosomal\_peptide\_synthetase | BGC0002025 | NRP+Polyketide | 32.0 | 49.0 | 226.0 | 3.8e-59 |
| ctg1\_orf19 |  | BGC0001013 | NRP+Polyketide | 31.0 | 48.4 | 224.0 | 3.87e-59 |
| ABC87508.1 | NRPS\_for\_pipecolate\_incorporation | BGC0001011 | NRP+Polyketide | 31.0 | 48.4 | 224.0 | 3.93e-59 |
| AGC09528.1 | NRPS | BGC0001183 | Polyketide | 31.0 | 49.0 | 225.0 | 4.02e-59 |
| MCC5036786.1 | amino\_acid\_adenylation\_domain-containing\_protein | BGC0002638 | NRP | 33.0 | 48.6 | 225.0 | 4.35e-59 |
| CAM56771.1 |  | BGC0000354 | NRP | 31.0 | 49.8 | 225.0 | 4.53e-59 |
| AAF17281.1 | nosD | BGC0001028 | Polyketide+NRP:Cyclic depsipeptide | 30.0 | 49.5 | 225.0 | 4.63e-59 |
| AXN93581.1 | PuwF-G | BGC0001950 | NRP | 30.0 | 48.2 | 225.0 | 4.82e-59 |
| AXN93590.1 | PuwF-G | BGC0001951 | NRP | 30.0 | 48.2 | 225.0 | 4.82e-59 |
| ABS74179.1 | bacillomycin\_D\_synthetase\_C | BGC0001090 | Polyketide+NRP:Lipopeptide | 29.0 | 48.6 | 225.0 | 4.82e-59 |
| BCJ07532.1 | hypothetical\_protein | BGC0002379 | NRP | 31.0 | 49.0 | 225.0 | 4.83e-59 |
| AIW82282.1 | PuwE | BGC0001125 | NRP+Polyketide | 31.0 | 48.2 | 225.0 | 5.23e-59 |
| AAY37655.1 | Amino\_acid\_adenylation | BGC0000437 | NRP | 32.0 | 50.2 | 225.0 | 5.35e-59 |
| DAC80541.1 | NRPS/PKS | BGC0001840 | NRP+Polyketide | 31.0 | 49.3 | 224.0 | 5.36e-59 |
| QXF14600.1 | PydA | BGC0002239 | Polyketide+NRP | 26.0 | 83.0 | 225.0 | 5.86e-59 |
| DAC80528.1 | peptide\_synthetase | BGC0001878 | NRP+Polyketide | 31.0 | 48.7 | 223.0 | 5.91e-59 |
| AJM89735.1 | PmxA | BGC0001192 | NRP | 32.0 | 48.9 | 225.0 | 6.14e-59 |
| AAG02364.1 | peptide\_synthetase\_NRPS2-1 | BGC0000963 | NRP:Glycopeptide+Polyketide:Modular type I polyketide+Saccharide:Hybrid/tailoring saccharide | 34.0 | 46.1 | 224.0 | 6.36e-59 |
| AGM16414.1 | paenibacterin\_synthetase\_C | BGC0000400 | NRP | 31.0 | 48.9 | 224.0 | 6.99e-59 |
| AAO72425.1 | syringopeptin\_synthetase\_C | BGC0000438 | NRP | 32.0 | 50.2 | 225.0 | 7.05e-59 |
| EFE73313.1 | nonribosomal\_peptide\_synthetase | BGC0000431 | NRP:Cyclic depsipeptide | 31.0 | 47.8 | 224.0 | 7.54e-59 |
| QNH67551.1 | Cip23 | BGC0002108 | NRP | 33.0 | 47.9 | 224.0 | 8e-59 |
| AAO72424.1 | syringopeptin\_synthetase\_B | BGC0000438 | NRP | 30.0 | 50.8 | 224.0 | 8.25e-59 |
| AAR87760.2 | ZmaK | BGC0001059 | NRP+Polyketide | 27.0 | 54.9 | 224.0 | 8.27e-59 |
| ALV86867.1 | Tlo21 | BGC0001406 | NRP | 32.0 | 50.0 | 224.0 | 8.36e-59 |
| CAB53322.1 | putative\_peptide\_synthetase | BGC0000325 | NRP | 31.0 | 50.4 | 224.0 | 9.72e-59 |
| ABI26079.1 | OciC | BGC0000331 | NRP | 28.0 | 47.9 | 223.0 | 9.91e-59 |
| KPN93064.1 | NupB | BGC0001416 | NRP | 30.0 | 50.9 | 224.0 | 1.03e-58 |
| APZ78769.1 | nonribosomal\_peptide\_synthetase | BGC0001425 | NRP:Cyclic depsipeptide+Polyketide:Iterative type I polyketide | 30.0 | 50.2 | 224.0 | 1.04e-58 |
| CAM02313.1 | putative\_non-ribosomal\_peptide\_synthetase | BGC0000349 | NRP | 32.0 | 49.5 | 224.0 | 1.08e-58 |
| QWT72292.1 | putative\_non-ribosomal\_peptide\_synthetase | BGC0002430 | NRP+Saccharide | 34.0 | 48.6 | 224.0 | 1.1e-58 |
| MBE3200466.1 | non-ribosomal\_peptide\_synthetase | BGC0002409 | NRP | 33.0 | 51.0 | 224.0 | 1.13e-58 |
| DAB41478.1 | nonribosomal\_peptide\_synthetase | BGC0001230 | NRP:Cyclic depsipeptide+Polyketide:Modular type I polyketide | 33.0 | 49.2 | 223.0 | 1.17e-58 |
| ARF06222.1 | non-ribosomal\_peptide\_synthetase | BGC0001593 | NRP | 31.0 | 53.1 | 223.0 | 1.24e-58 |
| ACZ55942.1 | non-ribosomal\_peptide\_synthetase | BGC0000302 | NRP | 30.0 | 47.6 | 223.0 | 1.28e-58 |
| ATU31794.1 | NRPS | BGC0001814 | NRP | 33.0 | 46.4 | 224.0 | 1.38e-58 |
| AAZ55899.1 | amino\_acid\_adenylation | BGC0000359 | NRP | 33.0 | 47.5 | 221.0 | 1.49e-58 |
| AGI89789.1 | Nonribosomal\_peptide\_synthetase | BGC0001792 | NRP | 32.0 | 49.8 | 224.0 | 1.52e-58 |
| AHB82072.1 | non\_ribosomal\_peptide\_synthetase/polyketide\_synthase | BGC0001231 | NRP+Polyketide:Modular type I polyketide | 32.0 | 48.6 | 223.0 | 1.55e-58 |
| QBQ12463.1 | amino\_acid\_adenylation\_domain-containing\_protein | BGC0002693 | NRP | 31.0 | 48.2 | 223.0 | 1.75e-58 |
| AFJ23825.1 | WLIP\_synthetase\_B | BGC0001838 | NRP | 30.0 | 48.5 | 223.0 | 1.78e-58 |
| CAG29032.1 | nonribosomal\_peptide\_synthetase\_(modules\_3\_to\_6) | BGC0001023 | NRP+Polyketide:Modular type I polyketide | 33.0 | 48.6 | 223.0 | 1.8e-58 |
| APZ78822.1 | nonribosomal\_peptide\_synthetase | BGC0001429 | NRP:Cyclic depsipeptide+Polyketide:Iterative type I polyketide | 33.0 | 48.6 | 223.0 | 1.8e-58 |
| AIW82283.1 | PuwF | BGC0001125 | NRP+Polyketide | 30.0 | 50.6 | 223.0 | 1.8e-58 |
| AQZ69229.1 | hypothetical\_protein | BGC0001635 | NRP+Polyketide | 31.0 | 55.5 | 223.0 | 1.83e-58 |
| AHZ20773.1 | non-ribosomal\_peptide\_synthase | BGC0000369 | NRP+Saccharide:Hybrid/tailoring saccharide | 30.0 | 47.0 | 223.0 | 2.07e-58 |
| QRG35013.1 | NRPS | BGC0002378 | NRP | 32.0 | 46.9 | 223.0 | 2.08e-58 |
| CAE02630.1 | surfactin\_synthetase\_A | BGC0000433 | NRP:Lipopeptide | 29.0 | 48.0 | 223.0 | 2.21e-58 |
| QMN69934.1 | PsoC | BGC0002521 | NRP | 31.0 | 50.6 | 223.0 | 2.25e-58 |
| ARU08075.1 | mlcM | BGC0001448 | NRP:Lipopeptide:Ca+-dependent lipopeptide | 29.0 | 55.6 | 223.0 | 2.31e-58 |
| EME52974.1 | non-ribosomal\_peptide\_synthetase | BGC0001460 | NRP:Glycopeptide | 31.0 | 48.0 | 213.0 | 2.45e-58 |
| BAX89999.1 | Non-ribosomal\_peptide\_synthetase | BGC0001628 | NRP | 33.0 | 48.2 | 223.0 | 2.48e-58 |
| AFP87549.1 | NrpS | BGC0001135 | NRP | 33.0 | 48.7 | 223.0 | 2.5e-58 |
| MCF2150416.1 | Non-ribosomal\_peptide\_synthetase | BGC0002625 | NRP+Polyketide | 30.0 | 49.0 | 223.0 | 2.56e-58 |
| ATY37588.1 | BogA | BGC0001532 | NRP | 28.0 | 47.9 | 214.0 | 2.56e-58 |
| CAQ71829.1 | non\_ribosomal\_peptide\_synthase,\_antibiotic\_synthesis;\_contains\_4\_condensation\_domains,\_3\_AMP-acid\_ligases\_II\_domains,\_3\_PP-binding,\_Phosphopantetheine\_attachment\_site\_and\_a\_putative\_thioesterase\_domain | BGC0001189 | NRP | 32.0 | 51.7 | 223.0 | 3.01e-58 |
| APZ78795.1 | nonribosomal\_peptide\_synthetase | BGC0001427 | NRP:Cyclic depsipeptide+Polyketide:Iterative type I polyketide | 30.0 | 51.0 | 223.0 | 3.12e-58 |
| MBV7329455.1 | amino\_acid\_adenylation\_domain-containing\_protein | BGC0002131 | Polyketide+NRP:Glycopeptide+Saccharide:Hybrid/tailoring saccharide | 30.0 | 50.1 | 222.0 | 3.31e-58 |
| QWT72279.1 | non-ribosomal\_peptide\_synthetase | BGC0002430 | NRP+Saccharide | 32.0 | 51.4 | 223.0 | 3.51e-58 |
| ATY37591.1 | BogD | BGC0001532 | NRP | 30.0 | 48.6 | 222.0 | 4.14e-58 |
| ANS62968.1 | actinomycin\_synthetase\_II | BGC0001567 | NRP | 32.0 | 48.4 | 222.0 | 4.31e-58 |
| WP\_080679150.1 | non-ribosomal\_peptide\_synthetase | BGC0001228 | NRP:Cyclic depsipeptide | 31.0 | 48.7 | 222.0 | 4.34e-58 |
| BBA20967.1 | nonribosomal\_peptide\_synthetase | BGC0001763 | NRP+Polyketide | 32.0 | 47.1 | 222.0 | 4.59e-58 |
| ASX95241.1 | IlaS | BGC0001620 | NRP+Polyketide | 32.0 | 47.2 | 222.0 | 4.59e-58 |
| ABA73954.1 | putative\_non-ribosomal\_peptide\_synthetase | BGC0001842 | NRP:Lipopeptide | 30.0 | 48.0 | 221.0 | 5.03e-58 |
| QBG38783.1 | Atr22 | BGC0001975 | NRP | 32.0 | 48.1 | 222.0 | 5.1e-58 |
| QYA95681.1 | amino\_acid\_adenylation\_domain-containing\_protein | BGC0002676 | NRP | 31.0 | 48.3 | 222.0 | 5.48e-58 |
| QDJ74273.1 | non-ribosomal\_peptide\_synthetase | BGC0002109 | NRP | 31.0 | 48.0 | 222.0 | 5.71e-58 |
| CBD77746.1 | non-ribosomal\_peptide\_synthetase/polyketide\_synthase | BGC0000974 | NRP+Polyketide | 33.0 | 48.2 | 221.0 | 6.15e-58 |
| OAQ83772.1 | nonribosomal\_peptide\_synthase | BGC0001358 | NRP+Polyketide | 27.0 | 76.0 | 222.0 | 6.31e-58 |
| APZ78846.1 | nonribosomal\_peptide\_synthetase | BGC0001431 | NRP:Cyclic depsipeptide+Polyketide:Iterative type I polyketide | 32.0 | 48.2 | 221.0 | 7.12e-58 |
| AEA30273.1 | peptide\_synthetase | BGC0000429 | Polyketide+NRP:Cyclic depsipeptide | 32.0 | 48.6 | 221.0 | 7.22e-58 |
| WP\_039806852.1 | non-ribosomal\_peptide\_synthetase | BGC0002001 | NRP+Polyketide | 32.0 | 46.7 | 219.0 | 7.23e-58 |
| DAB41484.1 | nonribosomal\_peptide\_synthetase/polyketide\_synthase\_type\_I | BGC0001230 | NRP:Cyclic depsipeptide+Polyketide:Modular type I polyketide | 31.0 | 49.2 | 221.0 | 7.5e-58 |
| ADH04679.1 | non-ribosomal\_peptide\_synthetase | BGC0001344 | NRP+Polyketide | 31.0 | 47.9 | 221.0 | 7.62e-58 |
| QDQ83031.1 | amino\_acid\_adenylation\_domain-containing\_protein | BGC0002564 | NRP | 30.0 | 55.8 | 220.0 | 7.95e-58 |
| QCQ67880.1 | non-ribosomal\_peptide\_synthetase | BGC0002297 | NRP+Polyketide | 30.0 | 48.4 | 221.0 | 8.72e-58 |
| BCJ07600.1 | hypothetical\_protein | BGC0002379 | NRP | 32.0 | 47.8 | 214.0 | 8.88e-58 |
| ANG60379.1 | nonribosomal\_peptide\_synthetase\_BudA | BGC0001434 | NRP | 29.0 | 49.2 | 219.0 | 9.19e-58 |
| AAO62588.1 | peptide\_sythetase | BGC0001016 | NRP+Polyketide | 31.0 | 47.9 | 219.0 | 9.54e-58 |
| CUX79061.1 | Octapeptin\_synthase\_subunit\_B | BGC0001715 | NRP | 30.0 | 48.0 | 221.0 | 9.59e-58 |
| CAM56770.1 |  | BGC0000354 | NRP | 32.0 | 47.0 | 221.0 | 1.02e-57 |
| AFH75322.1 | nonribosomal\_peptide\_synthetase | BGC0000425 | NRP:Cyclic depsipeptide | 30.0 | 51.0 | 221.0 | 1.03e-57 |
| AIE77076.1 | peptide\_synthetase | BGC0000418 | NRP | 31.0 | 48.3 | 211.0 | 1.11e-57 |
| ABV79988.1 | ApnD | BGC0000301 | NRP | 28.0 | 48.2 | 219.0 | 1.21e-57 |
| APZ78809.1 | nonribosomal\_peptide\_synthetase | BGC0001428 | NRP:Cyclic depsipeptide+Polyketide:Iterative type I polyketide | 31.0 | 48.6 | 221.0 | 1.23e-57 |
| WP\_043882190.1 | non-ribosomal\_peptide\_synthetase | BGC0001728 | NRP+Polyketide | 31.0 | 49.4 | 221.0 | 1.35e-57 |
| CCA89328.1 | mixed\_trans-AT\_type\_I\_polyketide\_synthase/nonribosomal\_peptide\_synthetase | BGC0001111 | NRP+Polyketide:Trans-AT type I polyketide | 32.0 | 47.5 | 221.0 | 1.38e-57 |
| ADN26248.1 | peptide\_synthetase | BGC0000951 | NRP | 31.0 | 48.1 | 216.0 | 1.42e-57 |
| ABS90473.1 | NRPS | BGC0001106 | NRP+Polyketide | 37.0 | 36.7 | 220.0 | 1.43e-57 |
| QYA95662.1 | amino\_acid\_adenylation\_domain-containing\_protein | BGC0002676 | NRP | 31.0 | 48.6 | 218.0 | 1.52e-57 |
| AKC91856.1 | nonribosomal\_peptide\_synthetase | BGC0001414 | NRP | 32.0 | 48.8 | 220.0 | 1.59e-57 |
| QTT72098.1 | non-ribosomal\_peptide\_synthetase | BGC0002350 | NRP+Polyketide+Saccharide | 31.0 | 46.1 | 218.0 | 1.8e-57 |
| AXN93582.1 | PuwH | BGC0001950 | NRP | 29.0 | 47.7 | 218.0 | 1.97e-57 |
| AAO62587.1 | peptide\_sythetase | BGC0001016 | NRP+Polyketide | 31.0 | 48.5 | 219.0 | 2e-57 |
| ALP32042.1 | CycB | BGC0001293 | Polyketide | 33.0 | 38.8 | 219.0 | 2e-57 |
| QNL14925.1 | AptD | BGC0002512 | NRP | 30.0 | 48.1 | 219.0 | 2.03e-57 |
| AEI70245.1 | nonribosomal\_peptide\_synthetase\_NRPS | BGC0000401 | NRP | 31.0 | 50.8 | 219.0 | 2.15e-57 |
| AXN93591.1 | PuwH | BGC0001951 | NRP | 29.0 | 47.7 | 218.0 | 2.17e-57 |
| AHH53506.1 | non-ribosomal\_peptide\_synthetase | BGC0000439 | NRP:Lipopeptide:Ca+-dependent lipopeptide | 33.0 | 48.2 | 220.0 | 2.27e-57 |
| ABX37382.1 | amino\_acid\_adenylation\_domain\_protein | BGC0000984 | NRP+Polyketide | 32.0 | 48.6 | 220.0 | 2.3e-57 |
| AJK49757.1 | non-ribosomal\_peptide\_synthase | BGC0002565 | NRP | 32.0 | 48.8 | 219.0 | 2.31e-57 |
| ASA76632.1 | polyketide\_synthase\_non-ribosomal\_peptide\_synthetase\_hybrid | BGC0001751 | NRP+Polyketide | 31.0 | 50.1 | 219.0 | 2.36e-57 |
| AAT09805.1 | NocB | BGC0000395 | NRP | 33.0 | 47.5 | 219.0 | 2.41e-57 |
| RSO11555.1 | non-ribosomal\_peptide\_synthetase | BGC0002637 | NRP | 33.0 | 47.8 | 219.0 | 2.43e-57 |
| APZ78794.1 | nonribosomal\_peptide\_synthetase | BGC0001427 | NRP:Cyclic depsipeptide+Polyketide:Iterative type I polyketide | 33.0 | 37.5 | 219.0 | 2.47e-57 |
| AZH29361.1 | amino\_acid\_adenylation\_domain-containing\_protein | BGC0001843 | NRP | 30.0 | 48.4 | 219.0 | 2.67e-57 |
| QJX57338.1 | ChaA | BGC0002538 | Polyketide | 26.0 | 79.5 | 219.0 | 2.72e-57 |
| AHB82071.1 | non\_ribosomal\_peptide\_synthetase | BGC0001231 | NRP+Polyketide:Modular type I polyketide | 33.0 | 46.1 | 219.0 | 2.75e-57 |
| AHZ34241.1 | CipD | BGC0001389 | NRP | 29.0 | 68.1 | 219.0 | 2.79e-57 |
| AFJ14795.1 | PlpF | BGC0000403 | NRP | 29.0 | 49.0 | 217.0 | 2.92e-57 |
| AAY37654.1 | Amino\_acid\_adenylation | BGC0000437 | NRP | 30.0 | 50.8 | 219.0 | 2.95e-57 |
| AXF16146.1 | non-ribosomal\_peptide\_synthetase | BGC0002563 | NRP | 32.0 | 50.6 | 219.0 | 2.97e-57 |
| ALG65313.1 | Cal23 | BGC0001297 | NRP | 32.0 | 50.6 | 210.0 | 3.08e-57 |
| CAJ46691.1 | non-ribosomal\_peptide\_synthase | BGC0000969 | NRP:Cyclic depsipeptide+Polyketide:Modular type I polyketide | 30.0 | 50.2 | 217.0 | 3.08e-57 |
| ABE35421.1 | Non-ribosomal\_peptide\_synthetase | BGC0002421 | NRP | 28.0 | 65.9 | 218.0 | 3.59e-57 |
| QOJ72663.1 | XenE | BGC0002505 | Polyketide+NRP | 25.0 | 75.7 | 219.0 | 3.59e-57 |
| AFR69334.1 | nonribosomal\_peptide\_synthetase\_SpiDE1 | BGC0001045 | NRP:Cyclic depsipeptide+Polyketide:Modular type I polyketide | 31.0 | 46.7 | 219.0 | 3.62e-57 |
| CCA29203.1 | non-ribosomal\_peptide\_synthetase/polyketide\_synthase | BGC0000955 | NRP+Polyketide:Modular type I polyketide | 30.0 | 50.2 | 219.0 | 3.88e-57 |
| AAF00962.1 | mcyC | BGC0001017 | NRP+Polyketide:Modular type I polyketide | 30.0 | 47.5 | 218.0 | 3.89e-57 |
| CAC48369.1 | peptide\_synthetase | BGC0000311 | NRP | 33.0 | 47.8 | 209.0 | 3.9e-57 |
| QEO74982.1 | omn7 | BGC0002078 | NRP:Cyclic depsipeptide | 29.0 | 59.7 | 219.0 | 4.02e-57 |
| AHH53507.1 | non-ribosomal\_peptide\_synthetase | BGC0000439 | NRP:Lipopeptide:Ca+-dependent lipopeptide | 31.0 | 48.2 | 219.0 | 4.09e-57 |
| AXN93603.1 | PuwH | BGC0001952 | NRP | 31.0 | 49.8 | 217.0 | 4.13e-57 |
| APZ78768.1 | nonribosomal\_peptide\_synthetase | BGC0001425 | NRP:Cyclic depsipeptide+Polyketide:Iterative type I polyketide | 34.0 | 37.3 | 219.0 | 4.28e-57 |
| AAG02358.1 | peptide\_synthetase\_NRPS6 | BGC0000963 | NRP:Glycopeptide+Polyketide:Modular type I polyketide+Saccharide:Hybrid/tailoring saccharide | 31.0 | 46.1 | 217.0 | 4.32e-57 |
| BAW27693.1 | NRPS(C-A-T-TE) | BGC0001764 | NRP | 32.0 | 49.3 | 218.0 | 4.34e-57 |
| AGJ76605.1 | HglB | BGC0000869 | Other | 33.0 | 32.9 | 207.0 | 4.79e-57 |
| AEU11006.1 | NpnB | BGC0001029 | NRP+Polyketide | 31.0 | 46.3 | 219.0 | 4.97e-57 |
| CAE53353.1 | non-ribosomal\_peptide\_synthetase | BGC0000440 | NRP:Glycopeptide | 32.0 | 48.8 | 218.0 | 5.34e-57 |
| ABW17377.1 | PsoC | BGC0000411 | NRP | 30.0 | 50.8 | 218.0 | 6.12e-57 |
| APZ78782.1 | nonribosomal\_peptide\_synthetase | BGC0001426 | NRP:Cyclic depsipeptide+Polyketide:Iterative type I polyketide | 30.0 | 51.0 | 218.0 | 6.43e-57 |
| KPN93065.1 | NunD | BGC0001416 | NRP | 31.0 | 49.0 | 218.0 | 6.59e-57 |
| BAX89998.1 | Non-ribosomal\_peptide\_synthetase | BGC0001628 | NRP | 33.0 | 47.9 | 218.0 | 6.8e-57 |
| AJV88376.1 | MfnD | BGC0001214 | NRP | 32.0 | 46.2 | 217.0 | 7.3e-57 |
| APZ78781.1 | nonribosomal\_peptide\_synthetase | BGC0001426 | NRP:Cyclic depsipeptide+Polyketide:Iterative type I polyketide | 33.0 | 37.2 | 218.0 | 7.42e-57 |
| BAO84868.1 | putative\_non-ribosomal\_peptide\_synthetase | BGC0000414 | NRP | 33.0 | 40.6 | 214.0 | 7.84e-57 |
| APZ78744.1 | nonribosomal\_peptide\_synthetase | BGC0001422 | NRP:Cyclic depsipeptide+Polyketide:Iterative type I polyketide | 30.0 | 50.8 | 218.0 | 8.46e-57 |
| extra\_gene | NRPS/PKS | BGC0002095 | NRP | 32.0 | 48.6 | 218.0 | 8.69e-57 |
| QDQ83032.1 | amino\_acid\_adenylation\_domain-containing\_protein | BGC0002564 | NRP | 31.0 | 50.4 | 218.0 | 8.84e-57 |
| QNH67550.1 | Cip22 | BGC0002108 | NRP | 32.0 | 47.9 | 218.0 | 9.31e-57 |
| ATY37609.1 | BreD | BGC0001536 | NRP | 29.0 | 49.4 | 218.0 | 9.95e-57 |
| CAR51995.1 | ornibactin\_biosynthesis\_non-ribosomal\_peptide\_synthase | BGC0002569 | NRP | 27.0 | 65.5 | 217.0 | 1.08e-56 |
| AHB82058.1 | non\_ribosomal\_peptide\_synthetase | BGC0001019 | NRP+Polyketide:Modular type I polyketide | 32.0 | 47.3 | 218.0 | 1.09e-56 |
| AKA59440.1 | non-ribosomal\_peptide\_synthetase | BGC0001202 | NRP+Polyketide | 31.0 | 48.2 | 207.0 | 1.11e-56 |
| APZ78716.1 | nonribosomal\_peptide\_synthetase | BGC0001420 | NRP:Cyclic depsipeptide+Polyketide:Iterative type I polyketide | 32.0 | 47.1 | 218.0 | 1.11e-56 |
| AIG79224.1 | Non-ribosomal\_peptide\_synthetase/andenylation\_domain | BGC0000419 | Saccharide+NRP:Glycopeptide | 30.0 | 47.9 | 207.0 | 1.2e-56 |
| OKA09664.1 | non-ribosomal\_peptide\_synthetase | BGC0001459 | NRP:Glycopeptide | 31.0 | 48.7 | 207.0 | 1.32e-56 |
| AQH32485.1 | peptide\_synthetase | BGC0001667 | NRP+Polyketide | 30.0 | 50.4 | 217.0 | 1.38e-56 |
| APZ78692.1 | nonribosomal\_peptide\_synthetase | BGC0001418 | NRP:Cyclic depsipeptide+Polyketide:Iterative type I polyketide | 33.0 | 47.1 | 217.0 | 1.47e-56 |
| BAX64247.1 | NRPS | BGC0001623 | NRP+Polyketide | 31.0 | 48.6 | 217.0 | 1.51e-56 |
| ATO51563.1 | non-ribosomal\_peptide\_synthetase | BGC0001796 | NRP | 29.0 | 49.9 | 217.0 | 1.58e-56 |
| CAD17792.1 | probable\_non\_ribosomal\_peptide\_synthetase\_protein | BGC0001363 | NRP+Polyketide | 32.0 | 50.7 | 217.0 | 1.6e-56 |
| CAG15012.1 | peptide\_synthetase,\_module\_7 | BGC0000441 | NRP | 32.0 | 48.6 | 216.0 | 1.61e-56 |
| CCJ67640.1 | TaaE | BGC0000447 | NRP:Lipopeptide | 30.0 | 51.4 | 217.0 | 1.61e-56 |
| ABF87031.1 | non-ribosomal\_peptide\_synthetase/polyketide\_synthase | BGC0000393 | NRP+Polyketide:Modular type I polyketide | 32.0 | 48.2 | 217.0 | 1.73e-56 |
| ABI26077.1 | OciA | BGC0000331 | NRP | 29.0 | 49.4 | 217.0 | 1.79e-56 |
| CAJ96468.1 | non-ribosomal\_peptide\_synthetase | BGC0000330 | NRP:NRP siderophore | 30.0 | 49.8 | 214.0 | 1.83e-56 |
| AAO23333.1 | NcpA | BGC0000397 | NRP | 30.0 | 48.6 | 217.0 | 1.83e-56 |
| BAY02137.1 | amino\_acid\_adenylation\_domain-containing\_protein | BGC0002532 | NRP+Polyketide | 31.0 | 45.7 | 216.0 | 2.09e-56 |
| AVR48535.1 | CusC | BGC0001564 | NRP+Polyketide | 30.0 | 50.4 | 217.0 | 2.2e-56 |
| AEG64696.1 | LpmB | BGC0000379 | NRP | 33.0 | 47.3 | 216.0 | 2.27e-56 |
| ADF88262.1 | mixed\_nonribosomal\_peptide\_synthetase/\_polyketide\_synthase | BGC0000979 | NRP+Polyketide | 29.0 | 51.3 | 215.0 | 2.45e-56 |
| BAP05591.1 | calC | BGC0000967 | NRP+Polyketide:Trans-AT type I polyketide | 31.0 | 49.2 | 216.0 | 2.56e-56 |
| ALV86866.1 | Tlo20 | BGC0001406 | NRP | 31.0 | 50.8 | 216.0 | 2.58e-56 |
| ABX60161.1 | mixed\_NRPS/PKS | BGC0000978 | NRP+Alkaloid+Polyketide:Modular type I polyketide | 29.0 | 51.4 | 216.0 | 2.88e-56 |
| AZH29360.1 | amino\_acid\_adenylation\_domain-containing\_protein | BGC0001843 | NRP | 31.0 | 48.8 | 216.0 | 3e-56 |
| AFH75328.1 | nonribosomal\_peptide\_synthetase | BGC0000398 | NRP:Cyclic depsipeptide | 31.0 | 48.2 | 216.0 | 3.13e-56 |
| RSO11553.1 | non-ribosomal\_peptide\_synthetase | BGC0002637 | NRP | 32.0 | 48.1 | 216.0 | 3.2e-56 |
| BAT51067.1 | type\_I\_polyketide\_synthase | BGC0001296 | Polyketide | 33.0 | 38.0 | 216.0 | 3.24e-56 |
| ACC81024.1 | non-ribosomal\_peptide\_synthetase | BGC0001479 | NRP | 29.0 | 49.3 | 215.0 | 3.29e-56 |
| APZ78704.1 | nonribosomal\_peptide\_synthetase | BGC0001419 | NRP:Cyclic depsipeptide+Polyketide:Iterative type I polyketide | 32.0 | 47.1 | 216.0 | 3.34e-56 |
| AYJ71712.1 | non-ribosomal\_peptide\_synthetase | BGC0001942 | NRP+Polyketide | 30.0 | 48.5 | 206.0 | 3.51e-56 |
| ABI22131.1 | putative\_non-ribosomal\_peptide\_synthetase | BGC0000422 | NRP | 32.0 | 48.2 | 215.0 | 3.62e-56 |
| QTT72106.1 | amino\_acid\_adenylation\_domain-containing\_protein | BGC0002350 | NRP+Polyketide+Saccharide | 36.0 | 35.7 | 216.0 | 3.73e-56 |
| AEI58879.1 | peptide\_synthetase | BGC0000455 | NRP | 30.0 | 47.2 | 206.0 | 3.86e-56 |
| APZ78728.1 | nonribosomal\_peptide\_synthetase | BGC0001421 | NRP:Cyclic depsipeptide+Polyketide:Iterative type I polyketide | 34.0 | 37.5 | 216.0 | 3.86e-56 |
| APZ78743.1 | nonribosomal\_peptide\_synthetase | BGC0001422 | NRP:Cyclic depsipeptide+Polyketide:Iterative type I polyketide | 30.0 | 46.0 | 216.0 | 3.87e-56 |
| QSJ20135.1 | non-ribosomal\_peptide\_synthase/polyketide\_synthase | BGC0002572 | NRP+Polyketide | 31.0 | 48.9 | 216.0 | 3.91e-56 |
| BAP05589.1 | calA | BGC0000967 | NRP+Polyketide:Trans-AT type I polyketide | 35.0 | 36.5 | 216.0 | 4.19e-56 |
| ADF88265.1 | mixed\_nonribosomal\_peptide\_synthetase/\_polyketide\_synthase | BGC0000980 | NRP+Polyketide | 29.0 | 51.3 | 214.0 | 4.27e-56 |
| AAF63833.1 | PstD | BGC0000362 | NRP | 31.0 | 47.6 | 214.0 | 4.3e-56 |
| PHM26612.1 | pvdj | BGC0001130 | NRP+Polyketide | 29.0 | 47.1 | 216.0 | 4.37e-56 |
| ADF88279.1 | mixed\_NRPS/PKS | BGC0000981 | NRP+Polyketide | 29.0 | 51.3 | 215.0 | 4.99e-56 |
| WP\_144411596.1 | non-ribosomal\_peptide\_synthetase | BGC0002001 | NRP+Polyketide | 30.0 | 49.8 | 214.0 | 5.31e-56 |
| QYC40287.1 | A50926\_NRPS,\_modules\_1-2 | BGC0002344 | NRP | 32.0 | 52.4 | 215.0 | 5.37e-56 |
| OKA09424.1 | non-ribosomal\_peptide\_synthetase | BGC0001459 | NRP:Glycopeptide | 32.0 | 46.7 | 215.0 | 5.64e-56 |
| QBA57736.1 | NRPS | BGC0002377 | NRP | 31.0 | 48.2 | 215.0 | 6.17e-56 |
| CAL69889.1 | RhiB\_protein | BGC0001112 | NRP+Polyketide:Trans-AT type I polyketide | 31.0 | 43.6 | 215.0 | 6.3e-56 |
| DAB41479.1 | nonribosomal\_peptide\_synthetase | BGC0001230 | NRP:Cyclic depsipeptide+Polyketide:Modular type I polyketide | 34.0 | 47.9 | 214.0 | 6.58e-56 |
| AAZ55900.1 | non-ribosomal\_peptide\_synthase:Amino\_acid\_adenylation | BGC0000359 | NRP | 32.0 | 50.6 | 215.0 | 7.16e-56 |
| ABX37385.1 | amino\_acid\_adenylation\_domain\_protein | BGC0000984 | NRP+Polyketide | 30.0 | 50.7 | 214.0 | 8.06e-56 |
| AAU39361.1 | lichenysin\_synthase\_LchAC | BGC0000381 | NRP | 29.0 | 47.7 | 213.0 | 8.12e-56 |
| APZ78808.1 | nonribosomal\_peptide\_synthetase | BGC0001428 | NRP:Cyclic depsipeptide+Polyketide:Iterative type I polyketide | 33.0 | 37.2 | 214.0 | 8.81e-56 |
| QGQ63518.1 | nonribosomal\_peptide\_synthetase\_modules\_A | BGC0002548 | NRP | 31.0 | 50.4 | 214.0 | 9.45e-56 |
| AAK81827.1 | peptide\_synthetase | BGC0000326 | NRP | 29.0 | 59.3 | 214.0 | 9.55e-56 |
| AFJ23826.1 | WLIP\_synthetase\_C | BGC0001838 | NRP | 31.0 | 49.2 | 214.0 | 9.57e-56 |
| MBE8994631.1 | amino\_acid\_adenylation\_domain-containing\_protein | BGC0002623 | NRP+Polyketide | 29.0 | 49.3 | 213.0 | 9.88e-56 |
| ADZ24999.1 | non-ribosomal\_peptide\_synthase | BGC0000380 | NRP+Polyketide:Modular type I polyketide | 30.0 | 48.2 | 213.0 | 1.01e-55 |
| KUM80514.1 | hypothetical\_protein | BGC0001562 | NRP | 29.0 | 48.6 | 212.0 | 1.01e-55 |
| ABV99085.1 | thioester\_reductase\_domain | BGC0001007 | Polyketide+NRP | 32.0 | 40.1 | 214.0 | 1.02e-55 |
| QED88055.1 | nonribosomal\_peptide\_synthetase | BGC0001967 | NRP+Polyketide | 32.0 | 48.6 | 214.0 | 1.02e-55 |
| AID65224.1 | nonribosomal\_peptide\_synthetase | BGC0000335 | NRP+Polyketide | 32.0 | 47.9 | 214.0 | 1.06e-55 |
| AAX31557.1 | peptide\_synthetase\_1 | BGC0000336 | NRP | 31.0 | 51.5 | 214.0 | 1.06e-55 |
| BAH43871.1 | truncated\_linear\_pentadecapeptide\_gramicidin\_synthetase\_LgrC | BGC0000367 | NRP | 29.0 | 51.1 | 214.0 | 1.08e-55 |
| QBC75021.1 | non-ribosomal\_peptide\_synthetase | BGC0001968 | NRP | 31.0 | 53.7 | 214.0 | 1.1e-55 |
| ACM79810.1 | ZmaO | BGC0001059 | NRP+Polyketide | 29.0 | 48.3 | 213.0 | 1.11e-55 |
| BAH22762.1 | nonribosomal\_peptide\_synthetase | BGC0001018 | NRP | 30.0 | 47.5 | 212.0 | 1.16e-55 |
| AHZ34240.1 | CipC | BGC0001389 | NRP | 30.0 | 48.1 | 213.0 | 1.16e-55 |
| APZ78755.1 | nonribosomal\_peptide\_synthetase | BGC0001423 | NRP:Cyclic depsipeptide+Polyketide:Iterative type I polyketide | 31.0 | 47.0 | 214.0 | 1.16e-55 |
| QEO74904.1 | AMP-dependent\_synthetase\_and\_ligase | BGC0002588 | Other | 31.0 | 46.6 | 214.0 | 1.17e-55 |
| AIG79243.1 | Non-ribosomal\_peptide\_synthetase | BGC0000419 | Saccharide+NRP:Glycopeptide | 31.0 | 51.0 | 214.0 | 1.21e-55 |
| ACG60782.1 | NRPS(C/A/PCP/C/A/PCP) | BGC0001058 | NRP:Glycopeptide+Polyketide:Modular type I polyketide+Saccharide:Hybrid/tailoring saccharide | 30.0 | 48.7 | 214.0 | 1.24e-55 |
| QLY89262.1 | pseudodesmin\_synthetase | BGC0002522 | NRP | 31.0 | 52.0 | 214.0 | 1.25e-55 |
| AZM51141.1 | non-ribosomal\_peptide\_synthetase | BGC0002702 | NRP | 31.0 | 47.5 | 214.0 | 1.26e-55 |
| ADG27359.1 | peptide\_synthetase | BGC0000296 | NRP | 34.0 | 40.0 | 214.0 | 1.3e-55 |
| CAD17793.1 | probable\_non\_ribosomal\_peptide\_synthetase\_protein | BGC0001363 | NRP+Polyketide | 30.0 | 50.3 | 214.0 | 1.41e-55 |
| AAN32981.1 | BarG | BGC0000962 | NRP+Polyketide:Modular type I polyketide | 29.0 | 49.1 | 214.0 | 1.49e-55 |
| BCD33691.1 | non-ribosomal\_peptide\_synthetase | BGC0002448 | NRP | 30.0 | 49.6 | 213.0 | 1.5e-55 |
| AZF85944.1 | hypothetical\_protein | BGC0001963 | NRP+Polyketide | 31.0 | 50.7 | 211.0 | 1.57e-55 |
| QIE07359.1 | dimodular\_nonribosomal\_peptide\_synthase\_NecA | BGC0002050 | NRP+Polyketide:Trans-AT type I polyketide | 31.0 | 48.2 | 213.0 | 1.58e-55 |
| ABX37383.1 | amino\_acid\_adenylation\_domain\_protein | BGC0000984 | NRP+Polyketide | 31.0 | 50.0 | 214.0 | 1.58e-55 |
| AEW31022.1 | plipastatin\_synthetase | BGC0000407 | NRP | 29.0 | 50.2 | 214.0 | 1.63e-55 |
| AIS24862.1 | dst18 | BGC0001147 | NRP | 32.0 | 50.8 | 211.0 | 1.65e-55 |
| ABG94125.1 | non-ribosomal\_peptide\_synthetase | BGC0000417 | NRP | 30.0 | 57.9 | 213.0 | 1.76e-55 |
| CCA29202.1 | non-ribosomal\_peptide\_synthetase | BGC0000955 | NRP+Polyketide:Modular type I polyketide | 30.0 | 47.1 | 213.0 | 1.77e-55 |
| TRX17524.1 | amino\_acid\_adenylation\_domain-containing\_protein | BGC0002329 | NRP | 31.0 | 49.0 | 214.0 | 1.81e-55 |
| CAJ34381.1 | NRPS\_protein | BGC0000445 | NRP:Cyclic depsipeptide | 31.0 | 46.3 | 213.0 | 1.86e-55 |
| APU91751.1 | Non-Ribosomal\_Peptide\_Synthetase | BGC0001806 | NRP | 30.0 | 47.9 | 214.0 | 1.88e-55 |
| CBL93723.1 | NRPS\_didomain\_A-PCP | BGC0000360 | NRP | 31.0 | 49.1 | 205.0 | 2.02e-55 |
| AAZ55898.1 | amino\_acid\_adenylation | BGC0000359 | NRP | 33.0 | 49.4 | 212.0 | 2.08e-55 |
| AHB82056.1 | non\_ribosomal\_peptide\_synthetase | BGC0001019 | NRP+Polyketide:Modular type I polyketide | 30.0 | 49.0 | 213.0 | 2.26e-55 |
| APZ78834.1 | nonribosomal\_peptide\_synthetase | BGC0001430 | NRP:Cyclic depsipeptide+Polyketide:Iterative type I polyketide | 31.0 | 48.2 | 213.0 | 2.29e-55 |
| APZ78856.1 | nonribosomal\_peptide\_synthetase | BGC0001432 | NRP:Cyclic depsipeptide+Polyketide:Iterative type I polyketide | 32.0 | 48.6 | 213.0 | 2.29e-55 |
| ALK27914.1 | non-ribosomal\_peptide\_synthase | BGC0001233 | NRP | 30.0 | 55.4 | 213.0 | 2.39e-55 |
| OLZ52442.1 | non-ribosomal\_peptide\_synthetase | BGC0001462 | NRP:Glycopeptide | 31.0 | 48.6 | 204.0 | 2.46e-55 |
| AGZ15459.1 | putative\_non-ribosomal\_peptide\_synthetase | BGC0001036 | NRP+Polyketide | 30.0 | 68.8 | 213.0 | 2.55e-55 |
| AQM37583.1 | nonribosomal\_peptide\_synthetase | BGC0001424 | NRP:Cyclic depsipeptide+Polyketide:Iterative type I polyketide | 33.0 | 37.2 | 213.0 | 2.64e-55 |
| ABP57748.1 | DepD | BGC0000993 | NRP:Cyclic depsipeptide+Polyketide:Modular type I polyketide | 31.0 | 50.4 | 213.0 | 2.65e-55 |
| AJY78094.1 | nonribosomal\_peptide\_synthetase | BGC0001902 | NRP+Polyketide | 32.0 | 49.2 | 212.0 | 2.78e-55 |
| QLY89264.1 | pseudodesmin\_synthetase | BGC0002522 | NRP | 30.0 | 49.8 | 213.0 | 2.88e-55 |
| ACZ55945.1 | non-ribosomal\_peptide\_synthetase | BGC0000302 | NRP | 30.0 | 49.4 | 213.0 | 2.9e-55 |
| AHI59108.1 | locillomycin\_synthase\_A | BGC0001005 | NRP+Polyketide | 27.0 | 48.8 | 213.0 | 3.02e-55 |
| EFL06867.1 | predicted\_protein | BGC0000300 | NRP | 32.0 | 47.9 | 213.0 | 3.02e-55 |
| BAH22764.1 | nonribosomal\_peptide\_synthetase | BGC0001018 | NRP | 29.0 | 50.6 | 213.0 | 3.05e-55 |
| OKA09425.1 | non-ribosomal\_peptide\_synthetase | BGC0001459 | NRP:Glycopeptide | 32.0 | 48.2 | 212.0 | 3.32e-55 |
| AMK48227.1 | nonribosomal\_peptide\_synthetas | BGC0001351 | NRP | 32.0 | 51.1 | 212.0 | 3.37e-55 |
| AAY93356.2 | non-ribosomal\_peptide\_synthetase\_PvdI | BGC0000413 | NRP | 31.0 | 48.7 | 213.0 | 3.75e-55 |
| QLY89263.1 | pseudodesmin\_synthetase | BGC0002522 | NRP | 31.0 | 49.4 | 213.0 | 3.94e-55 |
| ATL73045.1 | amino\_acid\_adenylation\_domain\_protein | BGC0001807 | NRP+Polyketide | 30.0 | 50.4 | 210.0 | 4.06e-55 |
| AHB38497.1 | non-ribosomal\_peptide\_synthetase | BGC0000346 | NRP+Polyketide:Modular type I polyketide | 35.0 | 39.6 | 213.0 | 4.08e-55 |
| AGC09519.1 | AMP-dependent\_synthetase/ligase | BGC0001183 | Polyketide | 30.0 | 50.4 | 210.0 | 4.12e-55 |
| AED90002.1 | non-ribosomal\_peptide\_synthetase\_ThaA | BGC0000443 | NRP:Beta-lactam | 31.0 | 47.9 | 213.0 | 4.13e-55 |
| CAG23960.2 | hybrid\_NRPS/PKS\_protein | BGC0001089 | Polyketide+NRP | 29.0 | 47.7 | 213.0 | 4.14e-55 |
| AEW31015.1 | plipastatin\_synthetase | BGC0000407 | NRP | 29.0 | 48.5 | 211.0 | 4.27e-55 |
| AXN93614.1 | PuwF | BGC0001953 | NRP | 29.0 | 51.4 | 212.0 | 4.47e-55 |
| AHB82059.1 | non\_ribosomal\_peptide\_synthetase/polyketide\_synthase | BGC0001019 | NRP+Polyketide:Modular type I polyketide | 30.0 | 48.0 | 212.0 | 4.48e-55 |
| AXN93613.1 | PuwE | BGC0001953 | NRP | 29.0 | 48.4 | 212.0 | 4.61e-55 |
| CBZ42143.1 | putative\_non-ribosomal\_peptide\_synthetase | BGC0001117 | NRP | 32.0 | 46.8 | 212.0 | 4.79e-55 |
| ALD82526.1 | non-ribosomal\_peptide\_synthase | BGC0001212 | NRP+Polyketide | 29.0 | 48.7 | 212.0 | 4.82e-55 |
| QEO74905.1 | condensation\_domain-containing\_protein | BGC0002588 | Other | 33.0 | 47.1 | 212.0 | 4.86e-55 |
| QBE85649.1 | BuaA | BGC0001857 | Alkaloid+NRP+Polyketide:Iterative type I polyketide | 26.0 | 79.1 | 212.0 | 5.04e-55 |
| CCM44336.1 | Nonribosomal\_peptide\_synthetase | BGC0001056 | NRP+Polyketide:Modular type I polyketide+Polyketide:PUFA synthase or related polyketide | 32.0 | 46.4 | 212.0 | 5.11e-55 |
| CAQ43084.1 | non\_ribosomal\_polypeptide\_synthetase | BGC0000970 | NRP+Polyketide:Modular type I polyketide | 29.0 | 48.2 | 211.0 | 5.19e-55 |
| AAK89720.1 | non-ribosomal\_peptide\_synthetase,\_siderophore\_biosynthesis\_protein | BGC0002107 | NRP+Polyketide | 31.0 | 48.4 | 212.0 | 5.33e-55 |
| CEK23605.1 | Non-ribosomal\_peptide\_synthase\_involved\_in\_xenematides\_synthesis | BGC0001825 | NRP | 29.0 | 49.3 | 212.0 | 5.34e-55 |
| QEO75076.1 | AMP-dependent\_synthetase\_and\_ligase | BGC0002079 | NRP:Cyclic depsipeptide | 28.0 | 58.7 | 209.0 | 5.4e-55 |
| AEH41793.1 | HrmO | BGC0000374 | NRP:Cyclic depsipeptide | 32.0 | 48.5 | 212.0 | 5.41e-55 |
| MBE8994632.1 | amino\_acid\_adenylation\_domain-containing\_protein | BGC0002623 | NRP+Polyketide | 29.0 | 50.2 | 212.0 | 5.45e-55 |
| QPI18728.1 | nonribosomal\_peptide\_synthetase | BGC0002125 | NRP:Cyclic depsipeptide | 32.0 | 46.4 | 212.0 | 5.48e-55 |
| BAO84861.1 | putative\_non-ribosomal\_peptide\_synthetase | BGC0000414 | NRP | 29.0 | 48.1 | 210.0 | 5.52e-55 |
| OKA09423.1 | non-ribosomal\_peptide\_synthetase | BGC0001459 | NRP:Glycopeptide | 32.0 | 48.2 | 212.0 | 6.12e-55 |
| DAB41476.1 | nonribosomal\_peptide\_synthetase | BGC0001230 | NRP:Cyclic depsipeptide+Polyketide:Modular type I polyketide | 32.0 | 50.5 | 211.0 | 6.18e-55 |
| KZM69124.1 | non-ribosomal\_peptide\_synthetase | BGC0002352 | Other | 32.0 | 51.2 | 211.0 | 6.2e-55 |
| ADY76684.1 | non-ribosomal\_peptide\_synthetase | BGC0000950 | NRP:Uridylpeptide+Other:Nucleoside | 34.0 | 41.9 | 201.0 | 6.58e-55 |
| AAO56329.1 | non-ribosomal\_peptide\_synthetase\_SyfB | BGC0000435 | NRP | 32.0 | 47.9 | 212.0 | 7.28e-55 |
| BAE98155.1 | putative\_non-ribosomal\_peptide\_synthetase | BGC0000339 | NRP | 33.0 | 45.9 | 211.0 | 7.36e-55 |
| AQI70\_32580 |  | BGC0001561 | NRP | 31.0 | 47.8 | 211.0 | 7.8e-55 |
| APZ78679.1 | nonribosomal\_peptide\_synthetase | BGC0001417 | NRP:Cyclic depsipeptide+Polyketide:Iterative type I polyketide | 33.0 | 36.5 | 211.0 | 7.93e-55 |
| AXN93616.1 | PuwH | BGC0001953 | NRP | 30.0 | 50.3 | 209.0 | 8.52e-55 |
| WP\_012408785.1 | non-ribosomal\_peptide\_synthetase | BGC0002061 | NRP:Cyclic depsipeptide+Polyketide:Modular type I polyketide | 30.0 | 51.5 | 211.0 | 8.66e-55 |
| AGI89790.1 | ATP-dependent\_valine\_adenylase | BGC0001792 | NRP | 31.0 | 49.4 | 211.0 | 8.9e-55 |
| CBA11570.1 | non-ribosomal\_peptide\_synthetase | BGC0001046 | NRP+Polyketide:Modular type I polyketide+Saccharide:Hybrid/tailoring saccharide | 34.0 | 41.5 | 202.0 | 8.93e-55 |
| ACZ55946.1 | non-ribosomal\_peptide\_synthetase | BGC0000302 | NRP | 30.0 | 48.3 | 210.0 | 9.06e-55 |
| ABD65960.1 | nonribosomal\_peptide\_synthetase | BGC0000341 | NRP | 31.0 | 48.2 | 207.0 | 9.19e-55 |
| CAE15497.1 |  | BGC0002286 | NRP | 32.0 | 47.9 | 211.0 | 9.43e-55 |
| ABL74937.1 | NRPS | BGC0001048 | NRP:Glycopeptide+Polyketide:Modular type I polyketide+Saccharide:Hybrid/tailoring saccharide | 31.0 | 48.5 | 209.0 | 9.93e-55 |
| AHZ34239.1 | CipB | BGC0001389 | NRP | 30.0 | 48.2 | 211.0 | 1.06e-54 |
| AGN74886.1 | nonribosomal\_peptide\_synthetase | BGC0000459 | NRP:Cyclic depsipeptide+Polyketide:Trans-AT type I polyketide | 32.0 | 47.6 | 210.0 | 1.08e-54 |
| ABJ97436.1 | MerP | BGC0001012 | NRP+Polyketide | 29.0 | 53.6 | 210.0 | 1.12e-54 |
| QDF82254.1 | non-ribosomal\_peptide\_synthetase | BGC0001980 | NRP | 30.0 | 48.0 | 211.0 | 1.12e-54 |
| AHB82069.1 | non\_ribosomal\_peptide\_synthetase | BGC0001231 | NRP+Polyketide:Modular type I polyketide | 30.0 | 48.7 | 211.0 | 1.14e-54 |
| QVQ62868.1 | nonribosomal\_peptide\_synthase | BGC0002373 | NRP | 28.0 | 69.4 | 211.0 | 1.2e-54 |
| AAG02349.1 | peptide\_synthetase\_NRPS11-10 | BGC0000963 | NRP:Glycopeptide+Polyketide:Modular type I polyketide+Saccharide:Hybrid/tailoring saccharide | 36.0 | 35.7 | 211.0 | 1.33e-54 |
| CBD77749.1 | non-ribosomal\_peptide\_synthetase | BGC0000974 | NRP+Polyketide | 32.0 | 48.2 | 210.0 | 1.38e-54 |
| AGZ15458.1 | putative\_non-ribosomal\_peptide\_synthetase | BGC0001036 | NRP+Polyketide | 31.0 | 46.2 | 210.0 | 1.67e-54 |
| AGN74895.1 | nonribosomal\_peptide\_synthetase/polyketide\_synthase\_hybrid\_protein | BGC0000459 | NRP:Cyclic depsipeptide+Polyketide:Trans-AT type I polyketide | 32.0 | 49.8 | 210.0 | 1.68e-54 |
| QCE43602.1 | nonribosomal\_peptide\_synthetase\_(NRPS),\_subunit\_1 | BGC0001834 | NRP | 29.0 | 48.2 | 210.0 | 1.91e-54 |
| ATY37590.1 | BogC | BGC0001532 | NRP | 28.0 | 48.5 | 210.0 | 2.07e-54 |
| AAZ23078.1 | peptide\_synthetase | BGC0000291 | NRP | 32.0 | 48.5 | 210.0 | 2.1e-54 |
| WP\_245566645.1 | amino\_acid\_adenylation\_domain-containing\_protein | BGC0002467 | NRP | 33.0 | 50.2 | 210.0 | 2.15e-54 |
| ABV79986.1 | ApnB | BGC0000301 | NRP | 29.0 | 48.3 | 208.0 | 2.21e-54 |
| AHZ34243.1 | CipF | BGC0001389 | NRP | 30.0 | 47.7 | 210.0 | 2.25e-54 |
| BAH43870.1 | putative\_linear\_pentadecapeptide\_gramicidin\_synthetase\_LgrB | BGC0000367 | NRP | 29.0 | 47.1 | 210.0 | 2.27e-54 |
| CAA60461.1 | pipecolate\_incorporating\_enzyme | BGC0001040 | NRP+Polyketide | 30.0 | 48.4 | 209.0 | 2.41e-54 |
| ANY58984.1 | non-ribosomal\_synthetase | BGC0001615 | NRP | 29.0 | 48.0 | 209.0 | 2.46e-54 |
| ABC94347.1 | vicibactin\_biosynthesis\_non-ribosomal\_peptide\_synthase\_protein | BGC0000457 | NRP | 29.0 | 49.0 | 209.0 | 2.46e-54 |
| WP\_044618979.1 | non-ribosomal\_peptide\_synthetase | BGC0001791 | NRP | 29.0 | 49.0 | 210.0 | 2.59e-54 |
| EPH46610.1 | putative\_Dimodular\_nonribosomal\_peptide\_synthase | BGC0001519 | NRP+Polyketide | 31.0 | 48.7 | 207.0 | 2.72e-54 |
| BCJ07533.1 | hypothetical\_protein | BGC0002379 | NRP | 30.0 | 51.4 | 209.0 | 2.89e-54 |
| QNL14923.1 | AptC | BGC0002512 | NRP | 29.0 | 49.6 | 209.0 | 2.89e-54 |
| AKJ15826.1 | peptide\_synthetase | BGC0002735 | Polyketide+NRP | 31.0 | 48.3 | 209.0 | 2.9e-54 |
| BAB69700.1 | iturin\_A\_synthetase\_C | BGC0001098 | NRP+Polyketide | 28.0 | 49.6 | 209.0 | 2.91e-54 |
| ABD65957.1 | nonribosomal\_peptide\_synthetase | BGC0000341 | NRP | 31.0 | 47.7 | 210.0 | 2.94e-54 |
| WP\_050383088.1 | non-ribosomal\_peptide\_synthetase | BGC0001451 | NRP | 32.0 | 48.8 | 207.0 | 3.03e-54 |
| CZT62784.1 | Non-ribosomal\_peptide\_synthase,\_involved\_in\_Hassallidin\_biosynthesis | BGC0001614 | NRP | 30.0 | 42.3 | 209.0 | 3.36e-54 |
| QMS47799.1 | JesA | BGC0001629 | NRP:Lipopeptide | 30.0 | 49.1 | 209.0 | 3.7e-54 |
| NHN68325.1 | amino\_acid\_adenylation\_domain-containing\_protein | BGC0002719 | NRP | 30.0 | 47.7 | 209.0 | 3.77e-54 |
| QED55423.1 | nonribosomal\_peptide\_synthetase | BGC0001984 | NRP | 32.0 | 50.4 | 209.0 | 3.87e-54 |
| CAC48362.1 | peptide\_synthetase | BGC0000311 | NRP | 32.0 | 48.3 | 209.0 | 3.95e-54 |
| CCC55922.1 | putative\_non-ribosomal\_peptide\_synthetase | BGC0000973 | NRP+Polyketide:Modular type I polyketide | 31.0 | 51.0 | 207.0 | 4.08e-54 |
| AHD05618.1 | putative\_non-ribosomal\_peptide\_ligase\_domain\_protein | BGC0001033 | NRP+Polyketide | 28.0 | 48.5 | 207.0 | 4.21e-54 |
| EME52989.1 | amino\_acid\_adenylation\_protein | BGC0001460 | NRP:Glycopeptide | 32.0 | 47.1 | 209.0 | 4.55e-54 |
| AKP45399.1 | CysK | BGC0001413 | NRP | 30.0 | 50.2 | 209.0 | 4.69e-54 |
| ABP55493.1 | thioester\_reductase\_domain | BGC0001006 | NRP+Polyketide | 33.0 | 38.9 | 209.0 | 4.77e-54 |
| QSJ20139.1 | non-ribosomal\_peptide\_synthase/polyketide\_synthase | BGC0002572 | NRP+Polyketide | 30.0 | 48.7 | 209.0 | 4.8e-54 |
| KMO93435.1 | NRPS/PKS | BGC0002095 | NRP | 31.0 | 51.0 | 207.0 | 4.94e-54 |
| QKM21620.1 | non-ribosomal\_peptide\_synthetase | BGC0002351 | NRP | 31.0 | 47.4 | 209.0 | 4.96e-54 |
| MBE8994630.1 | amino\_acid\_adenylation\_domain-containing\_protein | BGC0002623 | NRP+Polyketide | 29.0 | 49.5 | 209.0 | 5.15e-54 |
| AHF21229.1 | TriE | BGC0000449 | NRP | 29.0 | 53.1 | 209.0 | 5.84e-54 |
| CAM59606.1 | non-ribosomal\_peptide\_synthetase | BGC0000297 | NRP:Glycopeptide+Polyketide:Other polyketide+Saccharide:Hybrid/tailoring saccharide | 29.0 | 47.7 | 208.0 | 5.91e-54 |
| AAY93445.1 | non-ribosomal\_peptide\_synthetase\_PvdL | BGC0000413 | NRP | 31.0 | 47.1 | 209.0 | 6.09e-54 |
| AAF15891.2 | nosA | BGC0001028 | Polyketide+NRP:Cyclic depsipeptide | 28.0 | 50.9 | 209.0 | 6.11e-54 |
| QEO74981.1 | omn6 | BGC0002078 | NRP:Cyclic depsipeptide | 33.0 | 48.8 | 209.0 | 6.15e-54 |
| CAE52339.1 | Polyketide\_non-ribosomal\_peptide\_synthase | BGC0001088 | NRP+Polyketide | 31.0 | 48.7 | 209.0 | 6.69e-54 |
| ACO78736.1 | Non-ribosomal\_peptide\_synthase,\_PvdD-like\_protein | BGC0002433 | NRP | 30.0 | 49.9 | 208.0 | 6.95e-54 |
| AXG46163.1 | non-ribosomal\_peptide\_synthetase | BGC0002713 | NRP | 29.0 | 49.2 | 207.0 | 6.98e-54 |
| AXN93615.1 | PuwG | BGC0001953 | NRP | 31.0 | 48.4 | 208.0 | 7.05e-54 |
| WP\_019634550.1 | type\_I\_polyketide\_synthase | BGC0001443 | NRP+Polyketide | 33.0 | 47.8 | 207.0 | 7.32e-54 |
| CAE02631.1 | surfactin\_synthetase\_B\_ | BGC0000433 | NRP:Lipopeptide | 29.0 | 47.8 | 208.0 | 7.57e-54 |
| ACM68684.1 | AerB | BGC0000298 | NRP | 29.0 | 46.1 | 207.0 | 7.61e-54 |
| AZM57022.1 | non-ribosomal\_peptide\_synthetase | BGC0002314 | NRP | 30.0 | 50.3 | 208.0 | 7.68e-54 |
| BAC67534.2 | arthrofactin\_synthetase\_A | BGC0000305 | NRP:Lipopeptide | 31.0 | 48.3 | 208.0 | 7.69e-54 |
| CAO91861.1 | PKS-NRPS\_hybrid | BGC0000968 | NRP+Polyketide:Iterative type I polyketide | 26.0 | 76.8 | 208.0 | 7.84e-54 |
| MBE3200467.1 | amino\_acid\_adenylation\_domain-containing\_protein | BGC0002409 | NRP | 34.0 | 43.9 | 199.0 | 7.99e-54 |
| KPN90369.1 | NunE | BGC0001416 | NRP | 29.0 | 48.0 | 208.0 | 8.1e-54 |
| CBJ79916.1 | putative\_Ornithine\_racemase | BGC0001133 | NRP | 29.0 | 49.0 | 208.0 | 8.17e-54 |
| AAK81826.1 | peptide\_synthetase | BGC0000326 | NRP | 35.0 | 38.1 | 208.0 | 8.25e-54 |
| ABC37099.1 | non-ribosomal\_peptide\_synthetase,\_putative | BGC0000386 | NRP:NRP siderophore | 30.0 | 51.0 | 207.0 | 8.6e-54 |
| CAE02633.1 | surfactin\_synthetase\_C\_ | BGC0000433 | NRP:Lipopeptide | 29.0 | 47.4 | 207.0 | 8.99e-54 |
| AAF00961.1 | mcyB | BGC0001017 | NRP+Polyketide:Modular type I polyketide | 29.0 | 48.3 | 207.0 | 1.01e-53 |
| AEG64689.1 | LpmA | BGC0000379 | NRP | 29.0 | 48.4 | 205.0 | 1.01e-53 |
| AEW98135.1 | amino\_acid\_adenylation\_domain-containing\_protein | BGC0002642 | Alkaloid | 29.0 | 50.4 | 204.0 | 1.01e-53 |
| ctg1\_orf13 |  | BGC0001329 | Polyketide+NRP:Cyclic depsipeptide | 32.0 | 51.3 | 206.0 | 1.03e-53 |
| AAY93354.1 | non-ribosomal\_peptide\_synthetase\_PvdD | BGC0000413 | NRP | 31.0 | 49.0 | 208.0 | 1.06e-53 |
| AAX31559.1 | peptide\_synthetase\_3 | BGC0000336 | NRP | 32.0 | 48.9 | 207.0 | 1.09e-53 |
| CAJ88610.1 | putative\_non-ribosomal\_peptide\_synthetase | BGC0000327 | NRP | 33.0 | 50.6 | 206.0 | 1.14e-53 |
| AXN93601.1 | PuwE | BGC0001952 | NRP | 28.0 | 49.6 | 207.0 | 1.24e-53 |
| BCJ07529.1 | hypothetical\_protein | BGC0002379 | NRP | 28.0 | 67.8 | 207.0 | 1.29e-53 |
| CRI73800.1 | loading\_module\_of\_NRPS-PKS | BGC0001215 | NRP | 31.0 | 49.9 | 206.0 | 1.35e-53 |
| AGI89788.1 | Nonribosomal\_peptide\_synthetase | BGC0001792 | NRP | 32.0 | 47.0 | 207.0 | 1.37e-53 |
| AEA30272.1 | peptide\_synthetase | BGC0000429 | Polyketide+NRP:Cyclic depsipeptide | 30.0 | 48.7 | 207.0 | 1.42e-53 |
| QBG38784.1 | Atr23 | BGC0001975 | NRP | 32.0 | 47.7 | 207.0 | 1.42e-53 |
| AAZ23077.1 | peptide\_synthetase | BGC0000291 | NRP | 32.0 | 50.2 | 207.0 | 1.45e-53 |
| BAP16697.1 | nonribosomal\_peptide\_synthetase | BGC0000376 | NRP | 30.0 | 53.7 | 206.0 | 1.53e-53 |
| AAS98786.1 | nonribosomal\_peptide\_synthetase | BGC0001001 | NRP+Polyketide | 29.0 | 48.9 | 206.0 | 1.57e-53 |
| OLZ50899.1 | non-ribosomal\_peptide\_synthetase | BGC0001461 | NRP:Glycopeptide | 30.0 | 48.4 | 198.0 | 1.6e-53 |
| MCC5036783.1 | amino\_acid\_adenylation\_domain-containing\_protein | BGC0002638 | NRP | 32.0 | 47.4 | 205.0 | 1.61e-53 |
| AAU39359.1 | lichenysin\_synthase\_LchAA | BGC0000381 | NRP | 30.0 | 49.1 | 207.0 | 1.72e-53 |
| iliA |  | BGC0002035 | NRP+Polyketide | 26.0 | 72.1 | 207.0 | 1.79e-53 |
| QDJ74275.1 | non-ribosomal\_peptide\_synthetase | BGC0002109 | NRP | 31.0 | 50.2 | 207.0 | 1.89e-53 |
| AGC65516.1 | NRPS/PKS\_hybrid | BGC0001050 | NRP:Lipopeptide+Polyketide:Trans-AT type I polyketide | 31.0 | 49.3 | 207.0 | 1.92e-53 |
| UEF20583.1 | nonribosomal\_peptide\_synthetase | BGC0002360 | NRP | 30.0 | 52.9 | 205.0 | 1.94e-53 |
| ATY37589.1 | BogB | BGC0001532 | NRP | 28.0 | 47.7 | 207.0 | 1.95e-53 |
| OAL11475.1 | non-ribosomal\_peptide\_synthetase\_module | BGC0001570 | NRP | 30.0 | 51.3 | 199.0 | 2.09e-53 |
| AYA22318.1 | KerE | BGC0001955 | NRP | 30.0 | 48.4 | 198.0 | 2.16e-53 |
| UEF20592.1 | nonribosomal\_peptide\_synthetase | BGC0002360 | NRP | 31.0 | 49.0 | 205.0 | 2.19e-53 |
| AIW82284.1 | PuwG | BGC0001125 | NRP+Polyketide | 30.0 | 49.9 | 207.0 | 2.25e-53 |
| AGS77309.1 | NRPS\_modules\_4-6 | BGC0001178 | NRP:Glycopeptide | 31.0 | 46.7 | 207.0 | 2.36e-53 |
| QYI86762.1 | non-ribosomal\_peptide\_synthetase | BGC0002424 | NRP | 32.0 | 54.2 | 207.0 | 2.41e-53 |
| RSO11554.1 | non-ribosomal\_peptide\_synthetase | BGC0002637 | NRP | 32.0 | 46.5 | 207.0 | 2.48e-53 |
| OKJ61999.1 | peptide\_synthetase | BGC0002147 | NRP | 30.0 | 51.7 | 206.0 | 2.5e-53 |
| CAB38518.1 | CDA\_peptide\_synthetase\_I\_(CdaPs1) | BGC0000315 | NRP:Lipopeptide:Ca+-dependent lipopeptide | 31.0 | 48.2 | 207.0 | 2.66e-53 |
| AWN90\_15505 | non-ribosomal\_peptide\_synthetase | BGC0002352 | Other | 31.0 | 51.5 | 206.0 | 2.71e-53 |
| AXF14775.1 | non-ribosomal\_peptide\_synthetase | BGC0002563 | NRP | 31.0 | 49.1 | 206.0 | 2.78e-53 |
| AZF85940.1 | non-ribosomal\_peptide\_synthase | BGC0001963 | NRP+Polyketide | 32.0 | 51.4 | 206.0 | 3e-53 |
| AJW76711.1 | DsaI | BGC0001196 | NRP | 29.0 | 48.2 | 206.0 | 3.26e-53 |
| ACO78737.1 | Non-ribosomal\_peptide\_synthase,\_PvdD/PvdJ-like\_protein | BGC0002433 | NRP | 31.0 | 48.9 | 206.0 | 3.29e-53 |
| AJD47485.1 | PpsD | BGC0002418 | NRP+Polyketide | 32.0 | 46.3 | 206.0 | 3.41e-53 |
| CZT62794.1 | Non-ribosomal\_peptide\_synthase\_involved\_in\_Hassallidin\_biosynthesis | BGC0001614 | NRP | 29.0 | 48.6 | 206.0 | 3.57e-53 |
| UMM61373.1 | Tsk12 | BGC0002661 | NRP | 31.0 | 48.2 | 205.0 | 3.73e-53 |
| AKC91857.1 | nonribosomal\_peptide\_synthetase | BGC0001414 | NRP | 30.0 | 49.2 | 205.0 | 3.88e-53 |
| AEA30274.1 | peptide\_synthetase | BGC0000429 | Polyketide+NRP:Cyclic depsipeptide | 30.0 | 50.0 | 206.0 | 4.03e-53 |
| AVI26393.1 | nonribosomal\_peptide\_synthase | BGC0001800 | NRP+Polyketide | 30.0 | 48.2 | 206.0 | 4.06e-53 |
| WP\_012408783.1 | non-ribosomal\_peptide\_synthetase | BGC0002061 | NRP:Cyclic depsipeptide+Polyketide:Modular type I polyketide | 29.0 | 49.5 | 206.0 | 4.16e-53 |
| ABV56604.1 | adenylation\_domain\_protein | BGC0000378 | NRP | 32.0 | 45.8 | 197.0 | 4.18e-53 |
| QYA95682.1 | amino\_acid\_adenylation\_domain-containing\_protein | BGC0002676 | NRP | 30.0 | 48.7 | 206.0 | 4.24e-53 |
| ABR67749.1 | CmnF | BGC0000316 | NRP | 32.0 | 49.4 | 204.0 | 4.42e-53 |
| AAC06348.1 | bacitracin\_synthetase\_3 | BGC0000310 | NRP | 30.0 | 48.2 | 206.0 | 4.49e-53 |
| QKF54435.2 | nonribosomal\_peptide\_synthetase | BGC0002581 | NRP | 31.0 | 49.1 | 206.0 | 4.51e-53 |
| AFJ14793.1 | PlpD | BGC0000403 | NRP | 28.0 | 52.8 | 205.0 | 4.85e-53 |
| ADY76667.1 | non-ribosomal\_peptide\_synthetase | BGC0000950 | NRP:Uridylpeptide+Other:Nucleoside | 29.0 | 47.5 | 202.0 | 4.91e-53 |
| DAC76736.1 | non-ribosomal\_peptide\_synthetase | BGC0001885 | NRP+Polyketide | 32.0 | 39.8 | 205.0 | 5.08e-53 |
| QYA95680.1 | amino\_acid\_adenylation\_domain-containing\_protein | BGC0002676 | NRP | 30.0 | 49.8 | 206.0 | 5.29e-53 |
| AJW76710.1 | DsaH | BGC0001196 | NRP | 31.0 | 46.6 | 205.0 | 5.45e-53 |
| ABR67744.1 | CmnA | BGC0000316 | NRP | 31.0 | 49.7 | 205.0 | 5.67e-53 |
| QKF54440.2 | P450+A+T | BGC0002581 | NRP | 30.0 | 52.9 | 204.0 | 5.91e-53 |
| WP\_153044786.1 | non-ribosomal\_peptide\_synthetase | BGC0001826 | NRP | 30.0 | 49.3 | 204.0 | 5.92e-53 |
| PVC99865.1 | non-ribosomal\_peptide\_synthetase | BGC0002100 | NRP+Other | 31.0 | 54.9 | 205.0 | 6.87e-53 |
| OLZ52457.1 | non-ribosomal\_peptide\_synthetase | BGC0001462 | NRP:Glycopeptide | 32.0 | 46.7 | 205.0 | 7.04e-53 |
| AZM58102.1 | non-ribosomal\_peptide\_synthetase | BGC0002314 | NRP | 32.0 | 48.6 | 205.0 | 7.08e-53 |
| AFH75330.1 | nonribosomal\_peptide\_synthetase | BGC0000398 | NRP:Cyclic depsipeptide | 29.0 | 51.0 | 205.0 | 7.38e-53 |
| CBZ42140.1 | non-ribosomal\_peptide\_synthetase | BGC0001117 | NRP | 30.0 | 48.1 | 202.0 | 7.66e-53 |
| AKJ15829.1 | peptide\_synthetase | BGC0002735 | Polyketide+NRP | 30.0 | 47.9 | 204.0 | 8e-53 |
| EME52988.1 | amino\_acid\_adenylation\_protein | BGC0001460 | NRP:Glycopeptide | 31.0 | 48.2 | 204.0 | 8.02e-53 |
| WP\_050383082.1 | non-ribosomal\_peptide\_synthetase | BGC0001451 | NRP | 32.0 | 48.6 | 205.0 | 8.49e-53 |
| AAD44233.1 | PstA | BGC0000362 | NRP | 29.0 | 45.7 | 205.0 | 8.76e-53 |
| BAW32324.1 | nonribosomal\_peptide\_synthetase | BGC0001630 | NRP+Polyketide | 31.0 | 45.9 | 205.0 | 8.9e-53 |
| BAZ95823.1 | PKS-NRPS\_hybrid\_cpaA | BGC0001563 | NRP+Polyketide | 27.0 | 73.2 | 205.0 | 9.13e-53 |
| BAK26562.1 | PKS-NRPS\_hybrid | BGC0000977 | NRP+Polyketide | 24.0 | 77.8 | 205.0 | 9.15e-53 |
| ABS90476.1 | NRPS | BGC0001106 | NRP+Polyketide | 31.0 | 49.6 | 203.0 | 9.17e-53 |
| AZM50111.1 | non-ribosomal\_peptide\_synthetase | BGC0002702 | NRP | 32.0 | 46.3 | 205.0 | 9.56e-53 |
| WP\_051700112.1 | non-ribosomal\_peptide\_synthetase | BGC0001368 | NRP | 32.0 | 49.8 | 203.0 | 9.75e-53 |
| CAA11796.1 | PCZA363.5 | BGC0000322 | NRP | 31.0 | 49.4 | 204.0 | 1.07e-52 |
| OLZ52456.1 | non-ribosomal\_peptide\_synthetase | BGC0001462 | NRP:Glycopeptide | 31.0 | 50.2 | 204.0 | 1.07e-52 |
| APZ78691.1 | nonribosomal\_peptide\_synthetase | BGC0001418 | NRP:Cyclic depsipeptide+Polyketide:Iterative type I polyketide | 33.0 | 36.2 | 204.0 | 1.1e-52 |
| ABW17375.1 | PsoA | BGC0000411 | NRP | 31.0 | 47.7 | 204.0 | 1.19e-52 |
| AAZ03550.1 | McnA | BGC0000332 | NRP | 29.0 | 46.6 | 202.0 | 1.2e-52 |
| AAV66110.2 | fusaridione\_A\_synthetase | BGC0000992 | NRP+Polyketide | 26.0 | 72.1 | 204.0 | 1.21e-52 |
| AOA33122.1 | Nonribosomal\_peptide\_synthetase | BGC0001346 | NRP:Cyclic depsipeptide | 30.0 | 49.9 | 204.0 | 1.24e-52 |
| AKA59447.1 | non-ribosomal\_peptide\_synthetase | BGC0001203 | NRP+Polyketide | 34.0 | 36.7 | 204.0 | 1.26e-52 |
| ADG27358.1 | peptide\_synthetase | BGC0000296 | NRP | 30.0 | 48.3 | 204.0 | 1.35e-52 |
| WP\_050383094.1 | non-ribosomal\_peptide\_synthetase | BGC0001451 | NRP | 32.0 | 47.9 | 203.0 | 1.37e-52 |
| QCQ67879.1 | non-ribosomal\_peptide\_synthetase | BGC0002297 | NRP+Polyketide | 33.0 | 37.2 | 204.0 | 1.39e-52 |
| APZ78715.1 | nonribosomal\_peptide\_synthetase | BGC0001420 | NRP:Cyclic depsipeptide+Polyketide:Iterative type I polyketide | 33.0 | 36.2 | 204.0 | 1.45e-52 |
| CAA11794.1 | PCZA363.3 | BGC0000322 | NRP | 32.0 | 47.5 | 204.0 | 1.47e-52 |
| BAD55612.1 | non-ribosomal\_peptide\_synthetase | BGC0001027 | NRP+Polyketide | 32.0 | 49.2 | 203.0 | 1.48e-52 |
| CAD29794.1 | peptide\_synthetase | BGC0001015 | NRP+Polyketide | 28.0 | 47.5 | 204.0 | 1.53e-52 |
| UMM61371.1 | Tsk10 | BGC0002661 | NRP | 34.0 | 36.6 | 204.0 | 1.54e-52 |
| CAE53352.1 | non-ribosomal\_peptide\_synthetase | BGC0000440 | NRP:Glycopeptide | 32.0 | 47.1 | 204.0 | 1.6e-52 |
| ALV82388.1 | CDA\_peptide\_synthetase\_III | BGC0001370 | NRP | 32.0 | 48.8 | 204.0 | 1.7e-52 |
| AIE77058.1 | peptide\_synthetase\_module\_3 | BGC0000418 | NRP | 32.0 | 46.2 | 202.0 | 1.74e-52 |
| AAX31558.1 | peptide\_synthetase\_2 | BGC0000336 | NRP | 33.0 | 48.6 | 204.0 | 1.8e-52 |
| AIE77060.1 | peptide\_synthetase\_module\_7 | BGC0000418 | NRP | 31.0 | 48.9 | 203.0 | 1.85e-52 |
| AHZ20784.1 | non-ribosomal\_peptide\_synthase | BGC0000369 | NRP+Saccharide:Hybrid/tailoring saccharide | 29.0 | 48.1 | 204.0 | 1.85e-52 |
| AJV88377.1 | MfnE | BGC0001214 | NRP | 31.0 | 50.9 | 204.0 | 2.03e-52 |
| QED55421.1 | nonribosomal\_peptide\_synthetase | BGC0001984 | NRP | 30.0 | 48.6 | 204.0 | 2.07e-52 |
| AHY86403.1 | non\_ribosomal\_peptide\_synthetase | BGC0000329 | NRP | 32.0 | 45.3 | 203.0 | 2.09e-52 |
| AID65222.1 | putative\_aspartate\_racemase | BGC0000335 | NRP+Polyketide | 32.0 | 47.7 | 204.0 | 2.12e-52 |
| CCC55927.1 | hypothetical\_protein | BGC0000973 | NRP+Polyketide:Modular type I polyketide | 34.0 | 37.4 | 202.0 | 2.15e-52 |
| QWT72291.1 | amino\_acid\_adenylation\_domain-containing\_protein | BGC0002430 | NRP+Saccharide | 30.0 | 48.3 | 201.0 | 2.32e-52 |
| UEF20591.1 | nonribosomal\_peptide\_synthetase | BGC0002360 | NRP | 31.0 | 46.7 | 202.0 | 2.38e-52 |
| QYC40290.1 | A50926\_NRPS,\_module\_7 | BGC0002344 | NRP | 31.0 | 49.1 | 203.0 | 2.45e-52 |
| CAJ34367.1 | NRPS\_protein | BGC0000445 | NRP:Cyclic depsipeptide | 30.0 | 49.1 | 195.0 | 2.46e-52 |
| AHH25592.1 | NRPS | BGC0000957 | NRP+Polyketide | 31.0 | 49.5 | 199.0 | 2.59e-52 |
| QPI18726.1 | nonribosomal\_peptide\_synthetase | BGC0002125 | NRP:Cyclic depsipeptide | 33.0 | 48.3 | 201.0 | 2.76e-52 |
| AAM80537.1 | StaC | BGC0000290 | NRP:Glycopeptide | 29.0 | 52.6 | 203.0 | 2.77e-52 |
| ABV56588.1 | KtzH | BGC0000378 | NRP | 37.0 | 37.7 | 203.0 | 2.94e-52 |
| QVQ62856.1 | nonribosomal\_peptide\_synthase | BGC0002373 | NRP | 30.0 | 48.9 | 195.0 | 2.97e-52 |
| AIG79242.1 | Non-ribosomal\_peptide\_synthetase | BGC0000419 | Saccharide+NRP:Glycopeptide | 32.0 | 46.2 | 201.0 | 3.07e-52 |
| CEK23367.1 | putative\_Ornithine\_racemase | BGC0001716 | NRP | 29.0 | 49.4 | 203.0 | 3.17e-52 |
| BAE98162.1 | putative\_non-ribosomal\_peptide\_synthetase | BGC0000339 | NRP | 30.0 | 50.5 | 195.0 | 3.43e-52 |
| BAI63283.1 | putative\_non-ribosomal\_peptide\_synthetase | BGC0000434 | NRP | 31.0 | 49.4 | 194.0 | 3.48e-52 |
| CAD29798.1 | peptide\_synthetase | BGC0001015 | NRP+Polyketide | 28.0 | 48.2 | 202.0 | 3.59e-52 |
| OLZ50885.1 | non-ribosomal\_peptide\_synthetase | BGC0001461 | NRP:Glycopeptide | 32.0 | 46.6 | 203.0 | 3.64e-52 |
| ADN43685.1 | PKS-NRPS | BGC0001136 | NRP+Polyketide:Iterative type I polyketide | 26.0 | 72.9 | 203.0 | 3.66e-52 |
| QGQ63519.1 | nonribosomal\_peptide\_synthetase\_modules\_B | BGC0002548 | NRP | 29.0 | 47.1 | 203.0 | 3.7e-52 |
| QST87270.1 | amino\_acid\_adenylation\_domain-containing\_protein | BGC0002572 | NRP+Polyketide | 31.0 | 36.3 | 203.0 | 4.12e-52 |
| ABS74205.1 | fengycin\_synthetase\_E | BGC0001095 | NRP | 28.0 | 48.1 | 201.0 | 4.25e-52 |
| APZ78703.1 | nonribosomal\_peptide\_synthetase | BGC0001419 | NRP:Cyclic depsipeptide+Polyketide:Iterative type I polyketide | 32.0 | 36.2 | 202.0 | 4.34e-52 |
| APZ78845.1 | nonribosomal\_peptide\_synthetase | BGC0001431 | NRP:Cyclic depsipeptide+Polyketide:Iterative type I polyketide | 32.0 | 36.8 | 202.0 | 4.34e-52 |
| AHZ34242.1 | CipE | BGC0001389 | NRP | 30.0 | 49.4 | 202.0 | 4.35e-52 |
| ABI22132.1 | putative\_non-ribosomal\_peptide\_synthetase | BGC0000422 | NRP | 30.0 | 46.9 | 201.0 | 4.6e-52 |
| QBQ12464.1 | amino\_acid\_adenylation\_domain-containing\_protein | BGC0002693 | NRP | 30.0 | 46.7 | 202.0 | 4.64e-52 |
| AAY91419.3 | non-ribosomal\_peptide\_synthetase\_OfaA | BGC0000399 | NRP:Cyclic depsipeptide | 31.0 | 47.8 | 202.0 | 4.69e-52 |
| AYA22334.1 | KerC | BGC0001955 | NRP | 32.0 | 46.5 | 202.0 | 4.78e-52 |
| KYC42613.1 | non-ribosomal\_peptide\_synthetase | BGC0002484 | NRP+Polyketide | 28.0 | 50.2 | 202.0 | 5.04e-52 |
| CBZ42146.1 | putative\_non-ribosomal\_peptide\_synthetase | BGC0001117 | NRP | 31.0 | 48.4 | 202.0 | 5.05e-52 |
| WP\_099111429.1 | non-ribosomal\_peptide\_synthetase | BGC0001826 | NRP | 29.0 | 48.6 | 201.0 | 5.07e-52 |
| AAC06347.1 | bacitracin\_synthetase\_2 | BGC0000310 | NRP | 29.0 | 48.5 | 202.0 | 5.31e-52 |
| AIG79240.1 | Hypothetical\_protein | BGC0000419 | Saccharide+NRP:Glycopeptide | 30.0 | 49.3 | 202.0 | 5.54e-52 |
| CAD91211.1 | putative\_non-ribosomal\_peptide\_synthetase,\_module\_7 | BGC0000289 | NRP:Glycopeptide+Saccharide:Hybrid/tailoring saccharide | 29.0 | 58.0 | 202.0 | 5.56e-52 |
| AJD77023.1 | IkaA | BGC0001435 | NRP+Polyketide:Iterative type I polyketide | 31.0 | 48.9 | 202.0 | 5.76e-52 |
| NHN68324.1 | amino\_acid\_adenylation\_domain-containing\_protein | BGC0002719 | NRP | 31.0 | 47.9 | 202.0 | 5.8e-52 |
| AIE77057.1 | peptide\_synthetase | BGC0000418 | NRP | 31.0 | 47.5 | 202.0 | 6.04e-52 |
| AHD05679.1 | putative\_non-ribosomal\_peptide\_ligase/\_polyketide\_synthase\_hybrid | BGC0000402 | NRP | 28.0 | 50.2 | 202.0 | 6.25e-52 |
| ABS74208.1 | fengycin\_synthetase\_B | BGC0001095 | NRP | 27.0 | 47.9 | 202.0 | 6.93e-52 |
| ALK27916.1 | non-ribosomal\_peptide\_synthase | BGC0001233 | NRP | 30.0 | 50.7 | 202.0 | 7.82e-52 |
| QTT72111.1 | non-ribosomal\_peptide\_synthetase | BGC0002350 | NRP+Polyketide+Saccharide | 32.0 | 48.8 | 193.0 | 7.93e-52 |
| BAH43765.1 | tyrocidine\_synthetase\_II | BGC0000452 | NRP | 28.0 | 53.3 | 202.0 | 7.96e-52 |
| CAD91220.1 | putative\_non-ribosomal\_peptide\_synthetase,\_modules\_1-2 | BGC0000289 | NRP:Glycopeptide+Saccharide:Hybrid/tailoring saccharide | 31.0 | 51.0 | 201.0 | 8.05e-52 |
| EYT83459.1 | hypothetical\_protein | BGC0001213 | Polyketide | 33.0 | 31.8 | 188.0 | 8.39e-52 |
| MCC5036785.1 | amino\_acid\_adenylation\_domain-containing\_protein | BGC0002638 | NRP | 33.0 | 46.4 | 202.0 | 8.55e-52 |
| AFJ20782.1 | nonribosomal\_peptide\_synthetase | BGC0002300 | NRP | 31.0 | 47.1 | 201.0 | 8.58e-52 |
| AJQ95678.1 | polyketide\_synthase\_modules-related\_protein | BGC0002046 | NRP+Polyketide:Trans-AT type I polyketide | 29.0 | 48.4 | 202.0 | 8.61e-52 |
| AXN93602.1 | PuwF-G | BGC0001952 | NRP | 30.0 | 47.6 | 202.0 | 8.96e-52 |
| ABW00331.1 | amino\_acid\_adenylation\_domain | BGC0000333 | NRP | 31.0 | 47.7 | 202.0 | 9.35e-52 |
| ABL86391.1 | hybrid\_polyketide\_synthase\_and\_nonribosomal\_peptide\_synthetase | BGC0000999 | NRP+Polyketide | 29.0 | 50.2 | 201.0 | 9.96e-52 |
| AAF19811.1 | mtaC | BGC0001024 | NRP+Polyketide:Modular type I polyketide | 29.0 | 47.1 | 200.0 | 1.01e-51 |
| CBJ79915.1 | putative\_Phenylalanine\_racemase\_(ATP-hydrolyzing) | BGC0001133 | NRP | 29.0 | 48.3 | 201.0 | 1.08e-51 |
| AGD80616.1 | amino\_acid\_adenylation\_domain-containing\_protein | BGC0000394 | NRP | 34.0 | 41.3 | 198.0 | 1.08e-51 |
| AAF99707.2 | syringopeptin\_synthetase | BGC0000438 | NRP | 30.0 | 50.2 | 201.0 | 1.16e-51 |
| AEW31020.1 | plipastatin\_synthetase | BGC0000407 | NRP | 27.0 | 48.7 | 201.0 | 1.2e-51 |
| ALG65341.1 | Var6 | BGC0002416 | NRP+Polyketide | 30.0 | 50.2 | 201.0 | 1.2e-51 |
| QPI18729.1 | nonribosomal\_peptide\_synthetase | BGC0002125 | NRP:Cyclic depsipeptide | 33.0 | 48.2 | 201.0 | 1.31e-51 |
| QCQ67875.1 | hybrid\_peptide\_synthetase/polyketide\_synthase | BGC0002297 | NRP+Polyketide | 27.0 | 47.8 | 201.0 | 1.36e-51 |
| QVQ62855.1 | nonribosomal\_peptide\_synthase | BGC0002373 | NRP | 30.0 | 49.2 | 201.0 | 1.37e-51 |
| AAK81824.1 | peptide\_synthetase | BGC0000326 | NRP | 31.0 | 46.1 | 201.0 | 1.4e-51 |
| AFA26384.1 | polyketide\_synthase\_A | BGC0001874 | NRP+Polyketide | 24.0 | 77.6 | 201.0 | 1.41e-51 |
| BAC67535.1 | arthrofactin\_synthetase\_B | BGC0000305 | NRP:Lipopeptide | 30.0 | 50.6 | 201.0 | 1.45e-51 |
| ABO15860.1 | polyketide\_synthase | BGC0000130 | Polyketide | 32.0 | 41.6 | 201.0 | 1.45e-51 |
| AGS77310.1 | NRPS\_module\_7 | BGC0001178 | NRP:Glycopeptide | 30.0 | 56.4 | 200.0 | 1.67e-51 |
| CAJ96470.1 | non-ribosomal\_peptide\_synthetase | BGC0000330 | NRP:NRP siderophore | 30.0 | 46.3 | 200.0 | 1.77e-51 |
| AAT01806.1 | non-ribosomal\_peptide\_synthetase | BGC0000365 | NRP | 30.0 | 46.3 | 201.0 | 1.78e-51 |
| CAB38517.1 | CDA\_peptide\_synthetase\_II\_(CdaPs2) | BGC0000315 | NRP:Lipopeptide:Ca+-dependent lipopeptide | 30.0 | 47.9 | 201.0 | 1.82e-51 |
| AAT09804.1 | NocA | BGC0000395 | NRP | 32.0 | 47.5 | 201.0 | 1.82e-51 |
| CCE88377.1 | non-ribosomal\_peptide\_synthetase/polyketide\_synthase | BGC0001034 | NRP+Polyketide:Modular type I polyketide | 33.0 | 41.7 | 201.0 | 1.87e-51 |
| AAG02343.1 | peptide\_synthetase\_NRPS12 | BGC0000963 | NRP:Glycopeptide+Polyketide:Modular type I polyketide+Saccharide:Hybrid/tailoring saccharide | 31.0 | 48.8 | 192.0 | 1.94e-51 |
| KON97029.1 | phenylalanine\_racemase | BGC0002122 | NRP | 29.0 | 46.7 | 199.0 | 1.96e-51 |
| AEW95634.1 | non-ribosomal\_peptide\_synthetase | BGC0002697 | NRP+Polyketide | 31.0 | 50.6 | 200.0 | 1.99e-51 |
| ABV79987.1 | ApnC | BGC0000301 | NRP | 29.0 | 47.1 | 200.0 | 2.07e-51 |
| ALG65318.1 | Cal18 | BGC0001297 | NRP | 32.0 | 52.5 | 200.0 | 2.08e-51 |
| AEO14743.1 | NdaA | BGC0000396 | NRP | 32.0 | 37.7 | 200.0 | 2.11e-51 |
| AAK89731.2 | siderophore\_biosynthesis\_protein | BGC0002107 | NRP+Polyketide | 29.0 | 50.0 | 199.0 | 2.15e-51 |
| ABF85931.1 | non-ribosomal\_peptide\_synthase/polyketide\_synthase\_Ta1 | BGC0001025 | NRP+Polyketide:Trans-AT type I polyketide | 29.0 | 51.3 | 201.0 | 2.16e-51 |
| DMH02\_027320 | amino\_acid\_adenylation\_domain-containing\_protein | BGC0002638 | NRP | 35.0 | 40.0 | 197.0 | 2.37e-51 |
| EDY47118.1 | N-(5-amino-5-carboxypentanoyl)-L-cysteinyl-D-\_valine\_synthase | BGC0000319 | NRP:Beta-lactam | 30.0 | 48.4 | 200.0 | 2.41e-51 |
| KKP04599.1 | Non-ribosomal\_peptide\_synthetase\_-\_Polyketide\_synthase | BGC0002066 | NRP+Polyketide:Iterative type I polyketide | 25.0 | 77.5 | 200.0 | 2.45e-51 |
| BAH43764.1 | tyrocidine\_synthetase\_I | BGC0000452 | NRP | 29.0 | 46.7 | 198.0 | 2.54e-51 |
| QIE08737.1 | non-ribosomal\_peptide\_synthetase | BGC0002544 | NRP | 32.0 | 46.5 | 200.0 | 2.62e-51 |
| AEG64697.1 | LpmC | BGC0000379 | NRP | 32.0 | 47.1 | 200.0 | 2.74e-51 |
| AET98906.1 | putative\_non-ribosomal\_peptide\_synthetase | BGC0000415 | NRP | 33.0 | 42.4 | 200.0 | 2.78e-51 |
| AEI58867.1 | peptide\_synthetase | BGC0000455 | NRP | 31.0 | 48.1 | 199.0 | 2.88e-51 |
| CBF76038.1 | nonribosomal\_peptide\_synthase,\_putative\_(Eurofung) | BGC0001399 | NRP | 26.0 | 75.3 | 199.0 | 2.94e-51 |
| CZT62785.1 | Non-ribosomal\_peptide\_synthase,\_involved\_in\_Hassallidin\_biosynthesis | BGC0001614 | NRP | 31.0 | 46.1 | 200.0 | 2.94e-51 |
| AAL15600.1 | SimH | BGC0000270 | Polyketide | 30.0 | 48.8 | 197.0 | 3.02e-51 |
| AAK06804.1 | Tyroxyl-AMP-forming\_enzyme | BGC0001072 | Saccharide+Polyketide:Modular type I polyketide+Polyketide:Type II polyketide+Other:Aminocoumarin | 30.0 | 48.8 | 197.0 | 3.21e-51 |
| CAC48360.1 | peptide\_synthetase | BGC0000311 | NRP | 33.0 | 48.6 | 199.0 | 3.93e-51 |
| CAE53350.1 | non-ribosomal\_peptide\_synthetase | BGC0000440 | NRP:Glycopeptide | 32.0 | 47.5 | 199.0 | 4.14e-51 |
| QRN75755.1 | Amino\_acid\_adenylation\_domain\_protein | BGC0002114 | NRP+Polyketide | 30.0 | 50.1 | 199.0 | 4.2e-51 |
| CAA11795.1 | PCZA363.4 | BGC0000322 | NRP | 31.0 | 46.3 | 199.0 | 4.26e-51 |
| CAD55498.1 | CDA\_peptide\_synthetase\_III\_(CdaPs3) | BGC0000315 | NRP:Lipopeptide:Ca+-dependent lipopeptide | 32.0 | 48.6 | 199.0 | 4.56e-51 |
| AAF08797.1 | MycC | BGC0001103 | NRP+Polyketide | 27.0 | 49.5 | 199.0 | 4.75e-51 |
| ABS90470.1 | NRPS/PKS | BGC0001106 | NRP+Polyketide | 31.0 | 53.0 | 199.0 | 4.81e-51 |
| WP\_100939442.1 | non-ribosomal\_peptide\_synthetase | BGC0002071 | NRP:Lipopeptide | 30.0 | 49.9 | 199.0 | 5.17e-51 |
| AGS77307.1 | NRPS\_modules\_1-2 | BGC0001178 | NRP:Glycopeptide | 29.0 | 47.9 | 199.0 | 5.46e-51 |
| QBA57741.1 | NRPS | BGC0002377 | NRP | 31.0 | 48.2 | 191.0 | 5.47e-51 |
| WP\_064118561.1 | non-ribosomal\_peptide\_synthetase | BGC0001509 | NRP | 30.0 | 50.2 | 199.0 | 5.55e-51 |
| BAH04162.1 | trsJ | BGC0000450 | NRP | 32.0 | 47.0 | 199.0 | 6.33e-51 |
| WP\_051700105.1 | non-ribosomal\_peptide\_synthetase | BGC0001368 | NRP | 30.0 | 53.7 | 197.0 | 7.24e-51 |
| ACM79812.1 | ZmaQ | BGC0001059 | NRP+Polyketide | 30.0 | 48.2 | 198.0 | 7.4e-51 |
| DAD54574.1 | NRPS-like\_oxidoreductase | BGC0002256 | NRP+Other | 24.0 | 78.6 | 196.0 | 7.91e-51 |
| ARU08074.1 | mlcL | BGC0001448 | NRP:Lipopeptide:Ca+-dependent lipopeptide | 31.0 | 50.0 | 199.0 | 7.91e-51 |
| QNL34618.1 | SteC | BGC0002092 | NRP:Cyclic depsipeptide | 29.0 | 52.3 | 199.0 | 7.99e-51 |
| ACZ55944.1 | non-ribosomal\_peptide\_synthetase | BGC0000302 | NRP | 28.0 | 47.6 | 198.0 | 8.14e-51 |
| ANZ15839.1 | peptide\_synthetase\_ScpsB | BGC0001569 | NRP | 31.0 | 50.6 | 198.0 | 8.24e-51 |
| QTT72097.1 | amino\_acid\_adenylation\_domain-containing\_protein | BGC0002350 | NRP+Polyketide+Saccharide | 33.0 | 45.3 | 198.0 | 8.32e-51 |
| QDJ74274.1 | non-ribosomal\_peptide\_synthetase | BGC0002109 | NRP | 32.0 | 43.8 | 196.0 | 8.5e-51 |
| RSO11556.1 | non-ribosomal\_peptide\_synthetase | BGC0002637 | NRP | 32.0 | 48.0 | 198.0 | 8.88e-51 |
| CAF05649.1 | TubD\_protein | BGC0001053 | NRP+Polyketide | 30.0 | 48.4 | 198.0 | 9.26e-51 |
| CAC11137.1 | NikP1\_protein | BGC0000876 | Other | 29.0 | 51.6 | 192.0 | 9.39e-51 |
| CZT62792.1 | non-ribosomal\_peptide\_synthase\_involved\_in\_Hassallidin\_biosynthesis | BGC0001614 | NRP | 29.0 | 48.0 | 198.0 | 9.64e-51 |
| CUX79060.1 | Octapeptin\_synthase\_subunit\_A | BGC0001715 | NRP | 29.0 | 50.6 | 198.0 | 9.69e-51 |
| NKI69296.1 | amino\_acid\_adenylation\_domain-containing\_protein | BGC0002408 | NRP | 26.0 | 65.8 | 197.0 | 1.04e-50 |
| AFY58519.1 | amino\_acid\_adenylation\_enzyme/thioester\_reductase\_family\_protein,thioester\_reductase-like\_protein | BGC0002411 | NRP+Polyketide | 35.0 | 26.8 | 197.0 | 1.17e-50 |
| ABP73646.1 | SalB | BGC0000145 | NRP+Polyketide | 31.0 | 48.9 | 191.0 | 1.17e-50 |
| AFK57219.1 | DidH | BGC0000985 | Polyketide+NRP:Cyclic depsipeptide | 31.0 | 50.6 | 197.0 | 1.21e-50 |
| AAO62582.1 | polyketide\_synthase\_peptide\_sythetase\_fusion\_protein | BGC0001016 | NRP+Polyketide | 27.0 | 51.1 | 198.0 | 1.21e-50 |
| CBL93716.1 | NRPS\_A-domain | BGC0000360 | NRP | 32.0 | 41.5 | 188.0 | 1.23e-50 |
| CAG15009.1 | peptide\_synthetase,\_module\_1-2 | BGC0000441 | NRP | 32.0 | 47.2 | 197.0 | 1.24e-50 |
| AHH53508.1 | non-ribosomal\_peptide\_synthetase | BGC0000439 | NRP:Lipopeptide:Ca+-dependent lipopeptide | 31.0 | 51.3 | 197.0 | 1.35e-50 |
| QPI18723.1 | nonribosomal\_peptide\_synthetase | BGC0002125 | NRP:Cyclic depsipeptide | 33.0 | 48.2 | 196.0 | 1.56e-50 |
| CAJ77715.1 | Mps1\_protein | BGC0000364 | NRP | 31.0 | 46.0 | 197.0 | 1.59e-50 |
| AZM50110.1 | non-ribosomal\_peptide\_synthetase | BGC0002702 | NRP | 33.0 | 46.5 | 197.0 | 1.62e-50 |
| CAD91221.1 | putative\_non-ribosomal\_peptide\_synthetase,\_module\_3 | BGC0000289 | NRP:Glycopeptide+Saccharide:Hybrid/tailoring saccharide | 30.0 | 50.6 | 196.0 | 1.62e-50 |
| ABO15888.1 | polyketide\_synthase | BGC0000132 | Polyketide | 31.0 | 42.0 | 197.0 | 1.62e-50 |
| UKO95756.1 | amino\_acid\_adenylation\_domain-containing\_protein | BGC0002632 | NRP | 29.0 | 47.7 | 197.0 | 1.71e-50 |
| AAS47562.1 | mixed\_type\_I\_polyketide\_synthase\_-\_peptide\_synthetase | BGC0001108 | NRP+Polyketide:Trans-AT type I polyketide | 30.0 | 51.0 | 197.0 | 1.83e-50 |
| ctg1\_orf8 |  | BGC0001109 | NRP+Polyketide | 30.0 | 51.0 | 197.0 | 1.83e-50 |
| BAP05590.1 | calB | BGC0000967 | NRP+Polyketide:Trans-AT type I polyketide | 29.0 | 47.1 | 197.0 | 1.85e-50 |
| AAM80536.1 | StaD | BGC0000290 | NRP:Glycopeptide | 32.0 | 48.8 | 197.0 | 1.96e-50 |
| AKJ15896.1 | non\_ribosomal\_peptide\_synthetase | BGC0002735 | Polyketide+NRP | 31.0 | 47.9 | 195.0 | 1.99e-50 |
| AEI58865.1 | peptide\_synthetase | BGC0000455 | NRP | 31.0 | 47.9 | 197.0 | 2.02e-50 |
| ABP53497.1 | NRPS\_(ACP-P) | BGC0001041 | NRP+Polyketide | 31.0 | 48.2 | 190.0 | 2.11e-50 |
| QGY73448.1 | Itm16 | BGC0002451 | Polyketide | 31.0 | 47.8 | 196.0 | 2.15e-50 |
| AFY58523.1 | amino\_acid\_adenylation\_enzyme/thioester\_reductase\_family\_protein | BGC0002411 | NRP+Polyketide | 28.0 | 48.1 | 197.0 | 2.19e-50 |
| CAD91212.1 | putative\_non-ribosomal\_peptide\_synthetase,\_modules\_4-6 | BGC0000289 | NRP:Glycopeptide+Saccharide:Hybrid/tailoring saccharide | 33.0 | 46.6 | 197.0 | 2.2e-50 |
| QOE83925.1 | linear\_gramicidin\_synthase\_subunit\_B | BGC0002051 | NRP | 29.0 | 48.9 | 190.0 | 2.35e-50 |
| BCD33690.1 | non-ribosomal\_peptide\_synthetase | BGC0002448 | NRP | 33.0 | 46.3 | 197.0 | 2.4e-50 |
| DAC76731.1 | methionyl-tRNA\_formyltransferase | BGC0001885 | NRP+Polyketide | 31.0 | 50.1 | 196.0 | 2.41e-50 |
| AQH32484.1 | peptide\_synthetase | BGC0001667 | NRP+Polyketide | 31.0 | 37.3 | 197.0 | 2.53e-50 |
| TXC99989.1 | non-ribosomal\_peptide\_synthetase | BGC0001877 | Polyketide | 30.0 | 50.2 | 195.0 | 2.59e-50 |
| ACU36654.1 | amino\_acid\_adenylation\_domain\_protein | BGC0000392 | NRP | 32.0 | 49.3 | 197.0 | 2.73e-50 |
| MBA0053739.1 | acyltransferase\_domain-containing\_protein | BGC0002096 | Polyketide | 32.0 | 42.2 | 196.0 | 2.74e-50 |
| ADH04678.1 | non-ribosomal\_peptide\_synthetase | BGC0001344 | NRP+Polyketide | 35.0 | 36.7 | 196.0 | 2.75e-50 |
| QBK15049.1 | PKS-NRPS\_hybrid\_TraA | BGC0002197 | Polyketide+NRP | 26.0 | 68.9 | 197.0 | 2.84e-50 |
| AIE77059.1 | peptide\_synthetase | BGC0000418 | NRP | 31.0 | 46.5 | 197.0 | 2.87e-50 |
| CAG15011.1 | peptide\_synthetase,\_module\_4-6 | BGC0000441 | NRP | 32.0 | 47.1 | 197.0 | 2.88e-50 |
| AAY37653.1 | Amino\_acid\_adenylation | BGC0000437 | NRP | 29.0 | 50.6 | 197.0 | 3.07e-50 |
| AFV27434.1 | indigoidine\_synthase | BGC0000375 | NRP | 32.0 | 40.2 | 196.0 | 3.13e-50 |
| EME52990.1 | amino\_acid\_adenylation\_protein | BGC0001460 | NRP:Glycopeptide | 31.0 | 48.5 | 196.0 | 3.49e-50 |
| AQZ69228.1 | hypothetical\_protein | BGC0001635 | NRP+Polyketide | 31.0 | 46.7 | 196.0 | 3.66e-50 |
| BBA21071.1 | putative\_non-ribosomal\_peptide\_synthetase | BGC0001740 | NRP+Polyketide | 31.0 | 48.1 | 195.0 | 3.72e-50 |
| QBQ83704.1 | polyketide\_synthase-nonribosomal\_peptide\_synthetase | BGC0002093 | Polyketide+NRP | 26.0 | 74.4 | 196.0 | 3.78e-50 |
| CAC22144.1 | CpkC;\_Polyketide\_synthase\_module\_5 | BGC0000038 | Polyketide:Modular type I polyketide | 30.0 | 37.7 | 196.0 | 3.79e-50 |
| ATY69584.1 | hybrid\_nonribosomal\_peptide\_synthetase/type\_I\_polyketide\_synthase | BGC0001823 | NRP+Polyketide | 30.0 | 48.2 | 196.0 | 3.9e-50 |
| AAG02359.1 | peptide\_synthetase\_NRPS5-4-3 | BGC0000963 | NRP:Glycopeptide+Polyketide:Modular type I polyketide+Saccharide:Hybrid/tailoring saccharide | 32.0 | 45.3 | 196.0 | 4.29e-50 |
| OLZ50886.1 | non-ribosomal\_peptide\_synthetase | BGC0001461 | NRP:Glycopeptide | 30.0 | 48.2 | 196.0 | 4.48e-50 |
| AYA22333.1 | KerD | BGC0001955 | NRP | 30.0 | 48.2 | 196.0 | 4.48e-50 |
| ADA82585.1 | hybrid\_trans-AT\_polyketide\_synthase\_-\_nonribosomal\_peptide\_synthetase | BGC0001110 | NRP+Polyketide:Trans-AT type I polyketide | 30.0 | 49.1 | 196.0 | 4.48e-50 |
| QGU18619.1 | polyketide\_synthase/non-ribosomal\_peptide\_synthetase | BGC0002365 | Other+Polyketide | 31.0 | 49.4 | 196.0 | 4.58e-50 |
| AZM57023.1 | non-ribosomal\_peptide\_synthetase | BGC0002314 | NRP | 31.0 | 46.4 | 195.0 | 4.83e-50 |
| AHI59110.1 | locillomycin\_synthase\_C | BGC0001005 | NRP+Polyketide | 28.0 | 47.9 | 196.0 | 4.95e-50 |
| QDF82255.1 | non-ribosomal\_peptide\_synthetase | BGC0001980 | NRP | 30.0 | 49.8 | 196.0 | 5.06e-50 |
| QBF51786.1 | Nrps | BGC0001856 | Polyketide:Modular type I polyketide | 30.0 | 51.8 | 194.0 | 5.44e-50 |
| AAS47564.1 | mixed\_type\_I\_polyketide\_synthase/nonribosomal\_peptide\_synthetase | BGC0001108 | NRP+Polyketide:Trans-AT type I polyketide | 30.0 | 48.4 | 196.0 | 5.68e-50 |
| ctg1\_orf6 |  | BGC0001109 | NRP+Polyketide | 30.0 | 48.4 | 196.0 | 5.68e-50 |
| CAD29797.1 | peptide\_synthetase | BGC0001015 | NRP+Polyketide | 33.0 | 37.2 | 196.0 | 5.77e-50 |
| QUJ09166.1 | Lon19 | BGC0002440 | NRP | 28.0 | 49.1 | 195.0 | 6.56e-50 |
| AKQ22680.1 | malonyl\_CoA-acyl\_carrier\_protein\_transacylase | BGC0001656 | Polyketide | 29.0 | 47.1 | 196.0 | 6.83e-50 |
| AAV97877.1 | OnnI | BGC0001105 | NRP+Polyketide:Trans-AT type I polyketide | 29.0 | 48.6 | 196.0 | 6.88e-50 |
| ACC81023.1 | non-ribosomal\_peptide\_synthetase | BGC0001479 | NRP | 29.0 | 47.2 | 195.0 | 7.27e-50 |
| ADH01485.1 | putative\_mixed\_polyketide\_synthase/non-ribosomal\_peptide\_synthetase | BGC0000995 | NRP+Polyketide | 30.0 | 50.4 | 195.0 | 7.4e-50 |
| AGN11881.1 | tstDEF | BGC0001114 | NRP+Polyketide | 31.0 | 51.1 | 196.0 | 7.41e-50 |
| ARU08063.1 | mlcA | BGC0001448 | NRP:Lipopeptide:Ca+-dependent lipopeptide | 28.0 | 49.8 | 193.0 | 8.06e-50 |
| UHJ79948.1 | non-ribosomal\_peptide\_synthetase | BGC0002654 | NRP | 30.0 | 49.1 | 195.0 | 8.1e-50 |
| ADH04680.1 | hybrid\_polyketide\_synthase/non-ribosomal\_peptide\_synthetase | BGC0001344 | NRP+Polyketide | 29.0 | 52.8 | 195.0 | 8.23e-50 |
| WP\_013310343.1 | non-ribosomal\_peptide\_synthetase | BGC0001728 | NRP+Polyketide | 28.0 | 48.0 | 194.0 | 8.39e-50 |
| AYA22335.1 | KerB | BGC0001955 | NRP | 31.0 | 46.3 | 193.0 | 8.42e-50 |
| QNL34616.1 | SteA | BGC0002092 | NRP:Cyclic depsipeptide | 30.0 | 47.9 | 195.0 | 8.58e-50 |
| ABV79985.1 | ApnA | BGC0000301 | NRP | 28.0 | 47.0 | 195.0 | 8.66e-50 |
| SCO70310.1 | Type\_I\_polyketide\_synthase | BGC0001433 | Polyketide:Modular type I polyketide | 30.0 | 40.8 | 195.0 | 8.75e-50 |
| EFL06865.1 | hypothetical\_protein | BGC0000300 | NRP | 31.0 | 46.3 | 195.0 | 9.13e-50 |
| AJF34463.1 | Txo1 | BGC0001207 | NRP | 35.0 | 36.9 | 195.0 | 9.44e-50 |
| BAH43766.1 | tyrocidine\_synthetase\_III | BGC0000452 | NRP | 27.0 | 49.1 | 195.0 | 9.45e-50 |
| mycF | polyketide\_synthase | BGC0002055 | NRP+Polyketide:Trans-AT type I polyketide | 28.0 | 47.6 | 195.0 | 9.88e-50 |
| KJY94239.1 | thioester\_reductase | BGC0002691 | NRP | 29.0 | 47.3 | 194.0 | 9.91e-50 |
| AOZ21320.1 | SulM | BGC0001790 | NRP | 30.0 | 47.1 | 195.0 | 1.02e-49 |
| AHZ20774.1 | non-ribosomal\_peptide\_synthase | BGC0000369 | NRP+Saccharide:Hybrid/tailoring saccharide | 29.0 | 42.1 | 195.0 | 1.09e-49 |
| mycH | polyketide\_synthase | BGC0002055 | NRP+Polyketide:Trans-AT type I polyketide | 28.0 | 49.8 | 195.0 | 1.25e-49 |
| AIC32693.1 | FR9DEF | BGC0001113 | NRP+Polyketide | 30.0 | 50.4 | 195.0 | 1.28e-49 |
| AWI62629.1 | nonribosomal\_peptide\_synthetase | BGC0001822 | NRP | 33.0 | 36.5 | 194.0 | 1.3e-49 |
| QBC75023.1 | non-ribosomal\_peptide\_synthetase | BGC0001968 | NRP | 33.0 | 40.5 | 194.0 | 1.3e-49 |
| APZ78855.1 | nonribosomal\_peptide\_synthetase | BGC0001432 | NRP:Cyclic depsipeptide+Polyketide:Iterative type I polyketide | 29.0 | 46.0 | 194.0 | 1.35e-49 |
| QUS58938.1 | amino\_acid\_adenylation\_domain-containing\_protein | BGC0002123 | NRP+Polyketide | 29.0 | 47.7 | 194.0 | 1.42e-49 |
| KYQ85937.1 | hypothetical\_protein | BGC0002437 | NRP | 29.0 | 48.2 | 194.0 | 1.42e-49 |
| AKC54422.1 | fumosorinone\_biosynthesis\_polyketide\_synthase | BGC0001218 | NRP+Polyketide | 26.0 | 72.4 | 194.0 | 1.49e-49 |
| NAO96320.1 | amino\_acid\_adenylation\_domain-containing\_protein | BGC0002117 | NRP | 29.0 | 48.7 | 194.0 | 1.53e-49 |
| ANZ15840.1 | non-ribosomal\_peptide\_synthase/amino\_acid\_adenylation\_enzyme | BGC0001569 | NRP | 31.0 | 49.6 | 194.0 | 1.54e-49 |
| ARU08073.1 | mlcK | BGC0001448 | NRP:Lipopeptide:Ca+-dependent lipopeptide | 30.0 | 48.2 | 194.0 | 1.78e-49 |
| CCJ67639.1 | TaaD | BGC0000447 | NRP:Lipopeptide | 29.0 | 47.9 | 194.0 | 1.84e-49 |
| AAM80539.1 | StaA | BGC0000290 | NRP:Glycopeptide | 32.0 | 47.1 | 194.0 | 1.91e-49 |
| BS329\_14150 | non-ribosomal\_peptide\_synthetase | BGC0001462 | NRP:Glycopeptide | 30.0 | 47.1 | 194.0 | 1.92e-49 |
| AHD05678.1 | nonribosomal\_peptide\_ligase\_subunit | BGC0000402 | NRP | 28.0 | 49.1 | 194.0 | 2.07e-49 |
| CAG29031.1 | nonribosomal\_peptide\_synthetase\_(modules\_1\_and\_2) | BGC0001023 | NRP+Polyketide:Modular type I polyketide | 31.0 | 36.5 | 194.0 | 2.34e-49 |
| APZ78821.1 | nonribosomal\_peptide\_synthetase | BGC0001429 | NRP:Cyclic depsipeptide+Polyketide:Iterative type I polyketide | 31.0 | 36.5 | 194.0 | 2.34e-49 |
| QYC40289.1 | A50926\_NRPS,\_modules\_4-5-6 | BGC0002344 | NRP | 33.0 | 46.8 | 194.0 | 2.56e-49 |
| BAI63288.1 | putative\_non-ribosomal\_peptide\_synthetase | BGC0000434 | NRP | 34.0 | 42.4 | 193.0 | 2.86e-49 |
| RAT94091.1 | NRPS | BGC0001469 | NRP | 29.0 | 48.5 | 192.0 | 2.93e-49 |
| QMN69932.1 | PsoA | BGC0002521 | NRP | 30.0 | 47.4 | 193.0 | 3.35e-49 |
| QSJ20140.1 | amino\_acid\_adenylation\_domain-containing\_protein | BGC0002572 | NRP+Polyketide | 27.0 | 49.8 | 192.0 | 3.47e-49 |
| QYC40288.1 | A50926\_NRPS,\_module\_3 | BGC0002344 | NRP | 29.0 | 51.7 | 191.0 | 3.49e-49 |
| PHM26613.1 | pyoverdine\_synthetase\_D | BGC0001130 | NRP+Polyketide | 29.0 | 49.4 | 193.0 | 3.5e-49 |
| BAB69699.1 | iturin\_A\_synthetase\_B | BGC0001098 | NRP+Polyketide | 29.0 | 49.4 | 193.0 | 3.58e-49 |
| BAX64244.1 | NRPS | BGC0001623 | NRP+Polyketide | 30.0 | 51.7 | 193.0 | 3.72e-49 |
| AET98916.1 | putative\_non-ribosomal\_peptide\_synthetase | BGC0000415 | NRP | 30.0 | 49.4 | 186.0 | 3.88e-49 |
| CAF05647.1 | TubB\_protein | BGC0001053 | NRP+Polyketide | 34.0 | 36.2 | 192.0 | 4.35e-49 |
| AIG79241.1 | Hypothetical\_protein | BGC0000419 | Saccharide+NRP:Glycopeptide | 31.0 | 46.3 | 193.0 | 4.4e-49 |
| CAG23957.2 | hybrid\_NRPS/PKS\_protein | BGC0001089 | Polyketide+NRP | 29.0 | 46.4 | 193.0 | 4.63e-49 |
| ABD14711.1 | cesA | BGC0000320 | NRP:Cyclic depsipeptide | 28.0 | 46.3 | 192.0 | 5.5e-49 |
| ALV82384.1 | CDA\_peptide\_synthetase\_II | BGC0001370 | NRP | 32.0 | 47.7 | 192.0 | 5.63e-49 |
| BS330\_28385 | non-ribosomal\_peptide\_synthetase | BGC0001461 | NRP:Glycopeptide | 30.0 | 47.4 | 192.0 | 5.72e-49 |
| AHZ34232.1 | CifA | BGC0000323 | NRP:Lipopeptide | 30.0 | 48.6 | 192.0 | 7.06e-49 |
| OTA20325.1 | peptide\_synthase | BGC0001824 | NRP | 29.0 | 47.1 | 192.0 | 7.51e-49 |
| AXG49819.1 | hybrid\_non-ribosomal\_peptide\_synthetase/type\_I\_polyketide\_synthase | BGC0000383 | NRP+Polyketide:Modular type I polyketide | 29.0 | 51.5 | 192.0 | 7.66e-49 |
| AOA33123.1 | Nonribosomal\_peptide\_synthetase | BGC0001346 | NRP:Cyclic depsipeptide | 29.0 | 47.7 | 192.0 | 8.79e-49 |
| EFL02193.1 | amino\_acid\_adenylation\_domain-containing\_protein | BGC0000996 | NRP+Polyketide:Iterative type I polyketide | 32.0 | 41.8 | 192.0 | 8.86e-49 |
| BCJ07531.1 | hypothetical\_protein | BGC0002379 | NRP | 32.0 | 41.2 | 190.0 | 8.94e-49 |
| BAP82667.1 | non-ribosomal\_peptide\_synthetase\_A-domain\_containing\_protein | BGC0001148 | NRP+RiPP | 31.0 | 41.1 | 186.0 | 9.37e-49 |
| AZM51140.1 | non-ribosomal\_peptide\_synthetase | BGC0002702 | NRP | 29.0 | 46.1 | 191.0 | 9.71e-49 |
| CAC48361.1 | peptide\_synthetase | BGC0000311 | NRP | 31.0 | 46.3 | 192.0 | 1e-48 |
| AAY37650.1 | Amino\_acid\_adenylation | BGC0000437 | NRP | 28.0 | 52.3 | 185.0 | 1e-48 |
| AAG06715.1 | probable\_non-ribosomal\_peptide\_synthetase | BGC0002037 | NRP | 30.0 | 48.4 | 191.0 | 1.07e-48 |
| AEP18655.1 | WAPS2 | BGC0000461 | NRP | 34.0 | 37.2 | 192.0 | 1.12e-48 |
| AAO56328.1 | non-ribosomal\_peptide\_synthetase\_SyfA | BGC0000435 | NRP | 30.0 | 49.0 | 191.0 | 1.22e-48 |
| ATP76239.1 | NdaF | BGC0001705 | NRP+Polyketide | 27.0 | 51.2 | 191.0 | 1.26e-48 |
| CAL17541.1 | peptide\_synthetase,\_putative | BGC0002465 | NRP | 29.0 | 47.1 | 191.0 | 1.27e-48 |
| CCM44337.1 | Nonribosomal\_peptide\_synthetase | BGC0001056 | NRP+Polyketide:Modular type I polyketide+Polyketide:PUFA synthase or related polyketide | 31.0 | 48.6 | 191.0 | 1.34e-48 |
| AMK48228.1 | nonribosomal\_peptide\_synthetase | BGC0001351 | NRP | 31.0 | 45.9 | 189.0 | 1.38e-48 |
| WP\_006051170.1 | non-ribosomal\_peptide\_synthetase | BGC0001999 | NRP | 31.0 | 50.3 | 191.0 | 1.41e-48 |
| ADL64235.1 | aureusimine\_non-ribosomal\_peptide\_synthetase | BGC0000308 | NRP | 25.0 | 76.6 | 191.0 | 1.42e-48 |
| CBF73453.1 | nonribosomal\_peptide\_synthase,\_putative\_(JCVI) | BGC0001515 | NRP | 26.0 | 75.8 | 191.0 | 1.47e-48 |
| ABP57749.1 | DepE | BGC0000993 | NRP:Cyclic depsipeptide+Polyketide:Modular type I polyketide | 30.0 | 46.3 | 191.0 | 1.59e-48 |
| ABM21571.1 | crpC | BGC0000975 | NRP+Polyketide | 32.0 | 36.4 | 191.0 | 1.63e-48 |
| AEI58866.1 | peptide\_synthetase | BGC0000455 | NRP | 30.0 | 47.5 | 191.0 | 1.73e-48 |
| AAR87759.1 | ZmaJ | BGC0001059 | NRP+Polyketide | 28.0 | 41.3 | 182.0 | 1.74e-48 |
| DMA15\_34345 | non-ribosomal\_peptide\_synthetase | BGC0002314 | NRP | 32.0 | 46.4 | 190.0 | 1.75e-48 |
| QCF28926.1 | type\_I\_polyketide\_synthase | BGC0002308 | Alkaloid+Polyketide | 31.0 | 37.3 | 191.0 | 1.77e-48 |
| AAG29780.1 | peptide\_synthetase-like\_protein | BGC0000833 | Saccharide:Hybrid/tailoring saccharide+Other:Aminocoumarin | 29.0 | 49.1 | 184.0 | 1.92e-48 |
| ALG65336.1 | Var3 | BGC0002416 | NRP+Polyketide | 29.0 | 45.9 | 190.0 | 1.95e-48 |
| UKO95748.1 | amino\_acid\_adenylation\_domain-containing\_protein | BGC0002632 | NRP | 30.0 | 47.3 | 191.0 | 2.02e-48 |
| WP\_082191961.1 | non-ribosomal\_peptide\_synthetase | BGC0001451 | NRP | 31.0 | 47.9 | 191.0 | 2.07e-48 |
| CAQ46279.1 | putative\_enterobactin\_synthetase\_component\_F | BGC0002689 | NRP | 31.0 | 48.2 | 189.0 | 2.31e-48 |
| AAW03326.1 | CtaC | BGC0000982 | NRP+Polyketide | 29.0 | 47.3 | 189.0 | 2.35e-48 |
| CCJ67638.1 | TaaC | BGC0000447 | NRP:Lipopeptide | 29.0 | 51.0 | 190.0 | 2.81e-48 |
| AAM80538.1 | StaB | BGC0000290 | NRP:Glycopeptide | 31.0 | 45.9 | 189.0 | 2.85e-48 |
| MCC5036784.1 | amino\_acid\_adenylation\_domain-containing\_protein | BGC0002638 | NRP | 31.0 | 47.1 | 189.0 | 2.97e-48 |
| BCK51628.1 | non-ribosomal\_peptide\_synthetase | BGC0002520 | Polyketide | 30.0 | 48.5 | 187.0 | 3.04e-48 |
| ALG65342.1 | Var7 | BGC0002416 | NRP+Polyketide | 29.0 | 49.8 | 190.0 | 3.24e-48 |
| ADN26251.1 | ATP-dependent\_adenylase | BGC0000951 | NRP | 31.0 | 42.0 | 181.0 | 3.29e-48 |
| AIW58892.1 | non-ribosomal\_peptide\_synthetase | BGC0001582 | NRP | 33.0 | 36.7 | 190.0 | 3.55e-48 |
| KJY94240.1 | peptide\_synthetase | BGC0002691 | NRP | 29.0 | 47.5 | 190.0 | 3.8e-48 |
| AFP87523.1 | type\_I\_polyketide\_synthase | BGC0001159 | NRP+Polyketide:Modular type I polyketide | 36.0 | 33.0 | 189.0 | 4.08e-48 |
| NSC23530.1 | amino\_acid\_adenylation\_domain-containing\_protein | BGC0002359 | NRP | 31.0 | 46.8 | 189.0 | 4.4e-48 |
| ATP76243.1 | NdaA | BGC0001705 | NRP+Polyketide | 30.0 | 36.7 | 189.0 | 4.42e-48 |
| AZM51139.1 | non-ribosomal\_peptide\_synthetase | BGC0002702 | NRP | 30.0 | 46.5 | 189.0 | 4.8e-48 |
| CAJ77695.1 | MPS1\_protein | BGC0000363 | NRP | 29.0 | 50.2 | 189.0 | 4.91e-48 |
| QNL34617.1 | SteB | BGC0002092 | NRP:Cyclic depsipeptide | 30.0 | 51.5 | 189.0 | 5.23e-48 |
| AGZ15460.1 | putative\_non-ribosomal\_peptide\_synthetase | BGC0001036 | NRP+Polyketide | 33.0 | 39.4 | 189.0 | 5.58e-48 |
| WP\_039806850.1 | non-ribosomal\_peptide\_synthetase | BGC0002001 | NRP+Polyketide | 29.0 | 50.6 | 189.0 | 5.78e-48 |
| ATU31795.1 | NRPS | BGC0001814 | NRP | 32.0 | 48.3 | 189.0 | 5.84e-48 |
| AGZ03650.1 | sevA | BGC0000426 | NRP | 27.0 | 59.5 | 187.0 | 5.96e-48 |
| AAU39360.1 | lichenysin\_synthase\_LchAB | BGC0000381 | NRP | 29.0 | 49.8 | 189.0 | 6.52e-48 |
| CAJ46692.1 | non-ribosomal\_peptide\_synthase | BGC0000969 | NRP:Cyclic depsipeptide+Polyketide:Modular type I polyketide | 35.0 | 37.4 | 189.0 | 6.69e-48 |
| AAP92491.1 | nonribosomal\_peptide\_synthetase | BGC0000458 | NRP | 29.0 | 47.6 | 189.0 | 6.79e-48 |
| AZH23822.1 | MgiJ | BGC0001971 | NRP+Polyketide | 31.0 | 37.9 | 188.0 | 6.99e-48 |
| AHZ34233.1 | CifB | BGC0000323 | NRP:Lipopeptide | 29.0 | 48.2 | 189.0 | 7.28e-48 |
| BBA21068.1 | putative\_non-ribosomal\_peptide\_synthetase | BGC0001740 | NRP+Polyketide | 29.0 | 47.5 | 189.0 | 7.3e-48 |
| CAD32904.2 | non-ribosomal\_peptide\_synthetase\_A | BGC0000354 | NRP | 28.0 | 49.8 | 187.0 | 7.45e-48 |
| AGU50950.1 | putative\_non-ribosomal\_peptide\_synthetase | BGC0002417 | NRP+Polyketide | 29.0 | 46.7 | 189.0 | 7.62e-48 |
| AMK48226.1 | nonribosomal\_peptide\_synthetase | BGC0001351 | NRP | 28.0 | 61.4 | 189.0 | 7.62e-48 |
| EFE73308.1 | non-ribosomal\_peptide\_synthetase | BGC0000431 | NRP:Cyclic depsipeptide | 28.0 | 49.6 | 187.0 | 7.63e-48 |
| ctg3\_18 |  | BGC0001853 | NRP+Polyketide:Modular type I polyketide | 33.0 | 42.2 | 189.0 | 8.99e-48 |
| QGY73445.1 | Itm13 | BGC0002451 | Polyketide | 33.0 | 40.8 | 189.0 | 9.6e-48 |
| AKA59436.1 | non-ribosomal\_peptide\_synthetase | BGC0001202 | NRP+Polyketide | 31.0 | 53.2 | 188.0 | 9.89e-48 |
| QIE07364.1 | polyketide\_synthase\_NecE | BGC0002050 | NRP+Polyketide:Trans-AT type I polyketide | 30.0 | 48.7 | 188.0 | 1.03e-47 |
| QKM21619.1 | non-ribosomal\_peptide\_synthetase | BGC0002351 | NRP | 31.0 | 47.5 | 188.0 | 1.09e-47 |
| AFK57215.1 | DidD | BGC0000985 | Polyketide+NRP:Cyclic depsipeptide | 35.0 | 37.4 | 188.0 | 1.15e-47 |
| EPH46598.1 | putative\_Dimodular\_nonribosomal\_peptide\_synthase | BGC0001519 | NRP+Polyketide | 29.0 | 48.4 | 187.0 | 1.17e-47 |
| ATX68112.1 | malonyl\_CoA-acyl\_carrier\_protein\_transacylase | BGC0001772 | Polyketide | 28.0 | 46.6 | 188.0 | 1.22e-47 |
| AJI44167.1 | long-chain-fatty-acid-CoA\_ligase | BGC0001193 | NRP | 30.0 | 52.8 | 187.0 | 1.29e-47 |
| CAL17540.1 | peptide\_synthetase,\_putative | BGC0002465 | NRP | 29.0 | 48.9 | 187.0 | 1.38e-47 |
| QCP68976.1 | VatQ | BGC0002296 | NRP+Polyketide | 28.0 | 47.6 | 187.0 | 1.59e-47 |
| RAT94090.1 | NRPS | BGC0001469 | NRP | 28.0 | 48.4 | 188.0 | 1.65e-47 |
| AHD05621.1 | non-ribosomal\_peptide\_ligase\_domain\_protein | BGC0001033 | NRP+Polyketide | 29.0 | 36.3 | 187.0 | 1.76e-47 |
| AAP92496.1 | nonribosomal\_peptide\_synthetase | BGC0000458 | NRP | 30.0 | 52.4 | 186.0 | 1.77e-47 |
| CAJ96472.1 | non-ribosomal\_peptide\_synthetase | BGC0000330 | NRP:NRP siderophore | 31.0 | 51.3 | 187.0 | 1.85e-47 |
| ACM68690.1 | AerG1 | BGC0000298 | NRP | 28.0 | 48.9 | 186.0 | 1.87e-47 |
| KPN90374.1 | NunB1 | BGC0001416 | NRP | 29.0 | 49.4 | 181.0 | 2.08e-47 |
| AFH75321.1 | nonribosomal\_peptide\_synthetase | BGC0000425 | NRP:Cyclic depsipeptide | 29.0 | 50.6 | 187.0 | 2.2e-47 |
| AQX14441.1 | EM5400\_NRPS\_scaffold | BGC0001671 | NRP | 28.0 | 47.6 | 187.0 | 2.36e-47 |
| QIE07362.1 | polyketide\_synthase\_NecC | BGC0002050 | NRP+Polyketide:Trans-AT type I polyketide | 28.0 | 48.3 | 187.0 | 2.42e-47 |
| AGD80623.1 | non-ribosomal\_peptide\_synthetase | BGC0000394 | NRP | 31.0 | 46.5 | 187.0 | 2.43e-47 |
| ACU71638.1 | peptide\_synthetase-like\_protein | BGC0001154 | Other | 31.0 | 46.3 | 180.0 | 2.44e-47 |
| CAD89774.1 | MelC\_protein | BGC0001010 | NRP+Polyketide:Modular type I polyketide | 29.0 | 48.2 | 186.0 | 2.85e-47 |
| QPI18727.1 | nonribosomal\_peptide\_synthetase | BGC0002125 | NRP:Cyclic depsipeptide | 31.0 | 47.1 | 187.0 | 3.16e-47 |
| AET98905.1 | putative\_non-ribosomal\_peptide\_synthetase | BGC0000415 | NRP | 31.0 | 37.5 | 187.0 | 3.21e-47 |
| BAI63289.1 | putative\_non-ribosomal\_peptide\_synthetase | BGC0000434 | NRP | 33.0 | 36.5 | 187.0 | 3.21e-47 |
| BAH04173.1 | putative\_non-ribosomal\_peptide\_synthetase | BGC0000450 | NRP | 29.0 | 49.4 | 180.0 | 3.33e-47 |
| WP\_052165466.1 | non-ribosomal\_peptide\_synthetase | BGC0001327 | NRP:Cyclic depsipeptide+Polyketide:Modular type I polyketide | 34.0 | 36.9 | 186.0 | 3.44e-47 |
| AAO62586.1 | peptide\_sythetase | BGC0001016 | NRP+Polyketide | 30.0 | 36.9 | 186.0 | 4.02e-47 |
| AQZ26587.1 | obafluorin\_dimodular\_nonribosomal\_peptide\_synthetase | BGC0001437 | NRP | 30.0 | 47.8 | 186.0 | 4.27e-47 |
| AJK49766.1 | non-ribosomal\_peptide\_synthase | BGC0002565 | NRP | 31.0 | 48.4 | 186.0 | 4.44e-47 |
| ARR97036.1 | SphC | BGC0001780 | NRP | 29.0 | 48.6 | 186.0 | 4.84e-47 |
| CBL93718.1 | NRPS\_didomain\_PCP-C | BGC0000360 | NRP | 30.0 | 50.2 | 186.0 | 4.93e-47 |
| QWM97319.1 | non-ribosomal\_peptide\_synthetase | BGC0002384 | NRP | 33.0 | 37.2 | 186.0 | 5.19e-47 |
| AAG02355.1 | peptide\_synthetase\_NRPS9-8 | BGC0000963 | NRP:Glycopeptide+Polyketide:Modular type I polyketide+Saccharide:Hybrid/tailoring saccharide | 29.0 | 51.0 | 186.0 | 6.11e-47 |
| KFL51885.1 | amino\_acid\_adenylation\_protein | BGC0001711 | NRP+Polyketide | 29.0 | 48.4 | 185.0 | 6.35e-47 |
| QTT72100.1 | non-ribosomal\_peptide\_synthetase | BGC0002350 | NRP+Polyketide+Saccharide | 31.0 | 50.3 | 184.0 | 6.97e-47 |
| APD26279.1 | PtmA | BGC0001726 | NRP+Polyketide | 30.0 | 49.3 | 186.0 | 7.24e-47 |
| EWM63005.1 | linear\_gramicidin\_synthetase\_LgrC | BGC0001328 | NRP:Cyclic depsipeptide+Polyketide:Modular type I polyketide | 34.0 | 36.9 | 184.0 | 7.93e-47 |
| QTT72101.1 | non-ribosomal\_peptide\_synthetase | BGC0002350 | NRP+Polyketide+Saccharide | 29.0 | 51.0 | 185.0 | 7.97e-47 |
| TXD00259.1 | amino\_acid\_adenylation\_domain-containing\_protein | BGC0001877 | Polyketide | 30.0 | 39.5 | 184.0 | 8e-47 |
| BAP16693.1 | nonribosomal\_peptide\_synthetase | BGC0000376 | NRP | 30.0 | 49.3 | 184.0 | 8.16e-47 |
| AAN65224.1 | peptide\_synthetase-like\_protein | BGC0000832 | Saccharide:Hybrid/tailoring saccharide+Other:Aminocoumarin | 29.0 | 48.6 | 179.0 | 9.16e-47 |
| WP\_064118560.1 | non-ribosomal\_peptide\_synthetase | BGC0001509 | NRP | 29.0 | 50.8 | 185.0 | 1.05e-46 |
| UMM61372.1 | Tsk11 | BGC0002661 | NRP | 29.0 | 51.0 | 184.0 | 1.05e-46 |
| CBL93720.1 | NRPS\_didomain\_PCP-A | BGC0000360 | NRP | 29.0 | 50.2 | 182.0 | 1.09e-46 |
| AGN74885.1 | nonribosomal\_peptide\_synthetase | BGC0000459 | NRP:Cyclic depsipeptide+Polyketide:Trans-AT type I polyketide | 33.0 | 37.3 | 185.0 | 1.11e-46 |
| WP\_068925909.1 | non-ribosomal\_peptide\_synthetase | BGC0002688 | NRP | 31.0 | 50.6 | 185.0 | 1.11e-46 |
| AFU82614.1 | mixed\_NRPS\_PKS | BGC0000998 | NRP+Polyketide | 30.0 | 52.1 | 185.0 | 1.13e-46 |
| AEP18656.1 | WAPS1 | BGC0000461 | NRP | 30.0 | 47.6 | 185.0 | 1.18e-46 |
| AAG02356.1 | peptide\_synthetase\_NRPS7 | BGC0000963 | NRP:Glycopeptide+Polyketide:Modular type I polyketide+Saccharide:Hybrid/tailoring saccharide | 31.0 | 50.2 | 183.0 | 1.22e-46 |
| APZ78833.1 | nonribosomal\_peptide\_synthetase | BGC0001430 | NRP:Cyclic depsipeptide+Polyketide:Iterative type I polyketide | 29.0 | 46.6 | 185.0 | 1.24e-46 |
| BAE98156.1 | putative\_non-ribosomal\_peptide\_synthetase | BGC0000339 | NRP | 33.0 | 36.2 | 185.0 | 1.25e-46 |
| BAH04161.1 | putative\_non-ribosomal\_peptide\_synthetase | BGC0000450 | NRP | 31.0 | 37.5 | 185.0 | 1.25e-46 |
| AAF00958.1 | mcyE | BGC0001017 | NRP+Polyketide:Modular type I polyketide | 27.0 | 51.3 | 185.0 | 1.3e-46 |
| AAF08795.1 | MycA | BGC0001103 | NRP+Polyketide | 28.0 | 49.2 | 185.0 | 1.34e-46 |
| AYJ71721.1 | non-ribosomal\_peptide\_synthetase | BGC0001942 | NRP+Polyketide | 28.0 | 48.7 | 184.0 | 1.46e-46 |
| AED90004.1 | non-ribosomal\_peptide\_synthetase\_ThaC1 | BGC0000443 | NRP:Beta-lactam | 28.0 | 48.9 | 178.0 | 1.52e-46 |
| ACS20359.1 | amino\_acid\_adenylation\_domain\_protein | BGC0002420 | NRP+Polyketide | 29.0 | 49.8 | 184.0 | 1.53e-46 |
| WP\_013184322.1 | non-ribosomal\_peptide\_synthetase | BGC0001692 | NRP | 29.0 | 49.5 | 184.0 | 1.62e-46 |
| AYA22336.1 | KerA | BGC0001955 | NRP | 30.0 | 47.2 | 184.0 | 1.78e-46 |
| AAK81825.1 | peptide\_synthetase | BGC0000326 | NRP | 30.0 | 46.4 | 184.0 | 1.8e-46 |
| BGRAMDRAFT\_RS22640 | amino\_acid\_adenylation\_domain-containing\_protein | BGC0001999 | NRP | 28.0 | 54.4 | 183.0 | 1.81e-46 |
| QCX41944.1 | Amc7 | BGC0001957 | Polyketide | 30.0 | 48.9 | 181.0 | 2e-46 |
| QHW08555.1 | non-ribosomal\_peptide\_synthetase | BGC0002054 | Polyketide+NRP+Saccharide | 30.0 | 48.9 | 181.0 | 2e-46 |
| CCJ67646.1 | JagB | BGC0001127 | NRP | 30.0 | 49.4 | 184.0 | 2.1e-46 |
| QWM97862.1 | hybrid\_non-ribosomal\_peptide\_synthetase/type\_I\_polyketide\_synthase | BGC0002434 | Polyketide+NRP | 29.0 | 49.8 | 184.0 | 2.17e-46 |
| AFD30953.1 | CrmB | BGC0000966 | NRP+Polyketide | 29.0 | 49.0 | 182.0 | 2.67e-46 |
| AJK49758.1 | non-ribosomal\_peptide\_synthase | BGC0002565 | NRP | 30.0 | 47.9 | 184.0 | 2.73e-46 |
| CEK23364.1 | putative\_Phenylalanine\_racemase\_(ATP-hydrolyzing) | BGC0001716 | NRP | 26.0 | 49.0 | 184.0 | 2.97e-46 |
| FIS9431\_RS32925 | non-ribosomal\_peptide\_synthetase | BGC0001467 | NRP:Cyclic depsipeptide+Polyketide:Modular type I polyketide | 31.0 | 38.7 | 183.0 | 3.02e-46 |
| QUJ09168.1 | Lon21 | BGC0002440 | NRP | 31.0 | 49.1 | 183.0 | 3.11e-46 |
| QEO75077.1 | condensation\_domain-containing\_protein | BGC0002079 | NRP:Cyclic depsipeptide | 30.0 | 50.6 | 183.0 | 3.26e-46 |
| QXJ26485.1 | amino\_acid\_adenylation\_domain-containing\_protein | BGC0002370 | NRP | 30.0 | 47.8 | 181.0 | 3.4e-46 |
| ATJ34005.1 | adenylation\_domain-containing\_protein | BGC0001442 | NRP | 30.0 | 41.3 | 175.0 | 3.46e-46 |
| AGS77308.1 | NRPS\_module\_3 | BGC0001178 | NRP:Glycopeptide | 30.0 | 46.2 | 182.0 | 3.56e-46 |
| AAF00960.1 | mcyA | BGC0001017 | NRP+Polyketide:Modular type I polyketide | 32.0 | 36.6 | 183.0 | 3.57e-46 |
| QNS30807.1 | hybrid\_non-ribosomal\_peptide\_synthetase/type\_I\_polyketide\_syn-thase | BGC0002509 | NRP | 29.0 | 50.1 | 183.0 | 3.74e-46 |
| CBG75492.1 | putative\_NRPS/siderophore\_biosynthesis\_protein | BGC0000423 | NRP | 30.0 | 47.9 | 183.0 | 4.38e-46 |
| WP\_013310342.1 | non-ribosomal\_peptide\_synthetase | BGC0001728 | NRP+Polyketide | 27.0 | 49.8 | 183.0 | 4.79e-46 |
| AHD05615.1 | putative\_non-ribosomal\_peptide\_ligase/\_polyketide\_synthase\_hybrid | BGC0001033 | NRP+Polyketide | 26.0 | 48.7 | 183.0 | 4.82e-46 |
| KFL51886.1 | amino\_acid\_adenylation\_protein | BGC0001711 | NRP+Polyketide | 27.0 | 48.9 | 183.0 | 4.83e-46 |
| BAG23199.1 | putative\_type-I\_PKS | BGC0002673 | Polyketide+Alkaloid | 32.0 | 37.4 | 182.0 | 5.33e-46 |
| WP\_028678148.1 | non-ribosomal\_peptide\_synthetase | BGC0001228 | NRP:Cyclic depsipeptide | 32.0 | 36.7 | 182.0 | 6.41e-46 |
| AIZ66879.1 | nonribosomal\_peptide\_synthetase | BGC0002666 | NRP+Alkaloid | 28.0 | 49.4 | 182.0 | 6.5e-46 |
| AAL33756.1 | putative\_non-ribosomal\_peptide\_synthetase | BGC0000421 | NRP | 28.0 | 48.2 | 181.0 | 7e-46 |
| AWI62627.1 | nonribosomal\_peptide\_synthetase | BGC0001822 | NRP | 29.0 | 50.2 | 182.0 | 7.12e-46 |
| AAF08796.1 | MycB | BGC0001103 | NRP+Polyketide | 28.0 | 50.0 | 182.0 | 7.34e-46 |
| AKC91849.1 | nonribosomal\_peptide\_synthetase | BGC0001414 | NRP | 32.0 | 37.3 | 182.0 | 7.75e-46 |
| BAX64246.1 | NRPS | BGC0001623 | NRP+Polyketide | 31.0 | 46.2 | 182.0 | 7.97e-46 |
| AUD08663.1 | iPKS-NRPS | BGC0001553 | NRP+Polyketide | 30.0 | 50.2 | 182.0 | 8.56e-46 |
| QCP68971.1 | VatR | BGC0002296 | NRP+Polyketide | 29.0 | 36.9 | 182.0 | 8.58e-46 |
| ADZ24989.1 | prolin\_adenylation\_protein | BGC0000380 | NRP+Polyketide:Modular type I polyketide | 29.0 | 42.4 | 174.0 | 8.79e-46 |
| QCP68969.1 | VatN | BGC0002296 | NRP+Polyketide | 30.0 | 37.2 | 182.0 | 9.33e-46 |
| AFY58521.1 | amino\_acid\_adenylation\_enzyme/thioester\_reductase\_family\_protein | BGC0002411 | NRP+Polyketide | 28.0 | 47.5 | 181.0 | 9.38e-46 |
| QGA70148.1 | nonribosomal\_peptide\_synthetase | BGC0002293 | NRP | 34.0 | 36.5 | 182.0 | 1.07e-45 |
| BAP16689.1 | nonribosomal\_peptide\_synthetase | BGC0000376 | NRP | 30.0 | 50.8 | 181.0 | 1.19e-45 |
| DAD54486.1 | trans-acyltransferase\_polyketide\_synthase | BGC0002059 | NRP+Polyketide:Trans-AT type I polyketide | 28.0 | 52.8 | 182.0 | 1.34e-45 |
| MQQ32958.1 | amino\_acid\_adenylation\_domain-containing\_protein | BGC0002518 | NRP | 28.0 | 47.5 | 181.0 | 1.46e-45 |
| CCJ67647.1 | JagC | BGC0001127 | NRP | 29.0 | 46.8 | 181.0 | 1.52e-45 |
| AWO77084.1 | hybrid\_non-ribosomal\_peptide\_synthetase/type\_I\_polyketide\_synthase | BGC0001556 | NRP+Polyketide | 28.0 | 51.2 | 181.0 | 1.95e-45 |
| QCP68975.1 | VatS | BGC0002296 | NRP+Polyketide | 30.0 | 38.1 | 180.0 | 2.2e-45 |
| CAI94718.1 | putative\_CoA\_ligase | BGC0000141 | Polyketide | 31.0 | 49.0 | 180.0 | 2.3e-45 |
| ABL74940.1 | NRPS | BGC0001048 | NRP:Glycopeptide+Polyketide:Modular type I polyketide+Saccharide:Hybrid/tailoring saccharide | 34.0 | 41.7 | 181.0 | 2.38e-45 |
| BAG17643.1 | putative\_NRPS-type-I\_PKS\_fusion\_protein | BGC0001043 | NRP+Polyketide | 29.0 | 47.5 | 181.0 | 2.51e-45 |
| AHA12079.1 | polyketide\_synthase\_type\_1 | BGC0001172 | NRP+Polyketide:Modular type I polyketide | 30.0 | 40.0 | 180.0 | 2.58e-45 |
| AXA20090.1 | hybrid\_trans-AT\_PKS/NRPS\_LgaA | BGC0001646 | NRP+Polyketide | 28.0 | 47.4 | 181.0 | 2.61e-45 |
| CBG70279.1 | thaxtomin\_synthetase\_A | BGC0002089 | NRP | 32.0 | 36.5 | 180.0 | 2.63e-45 |
| BAB69698.1 | iturin\_A\_synthetase\_A | BGC0001098 | NRP+Polyketide | 27.0 | 48.7 | 181.0 | 2.69e-45 |
| QGQ63520.1 | nonribosomal\_peptide\_synthetase\_modules\_C | BGC0002548 | NRP | 28.0 | 47.8 | 180.0 | 3.14e-45 |
| ABS74181.1 | bacillomycin\_D\_synthetase\_A\_ | BGC0001090 | Polyketide+NRP:Lipopeptide | 27.0 | 48.7 | 180.0 | 3.53e-45 |
| AGC65514.1 | TtcB | BGC0001876 | NRP | 29.0 | 49.0 | 180.0 | 3.73e-45 |
| ATY69551.1 | hybrid\_nonribosomal\_peptide\_synthetase/type\_I\_polyketide\_synthase | BGC0001611 | NRP+Polyketide | 27.0 | 55.6 | 179.0 | 5.02e-45 |
| ATY37592.1 | BogE | BGC0001532 | NRP | 28.0 | 48.1 | 179.0 | 5.16e-45 |
| AAZ23076.1 | peptide\_synthetase | BGC0000291 | NRP | 29.0 | 49.8 | 179.0 | 6.28e-45 |
| BAP05596.1 | calH | BGC0000967 | NRP+Polyketide:Trans-AT type I polyketide | 28.0 | 47.9 | 179.0 | 6.34e-45 |
| WP\_084702182.1 | non-ribosomal\_peptide\_synthetase | BGC0001211 | NRP | 33.0 | 39.4 | 179.0 | 6.78e-45 |
| EFL06866.1 | predicted\_protein | BGC0000300 | NRP | 30.0 | 47.8 | 178.0 | 7.5e-45 |
| AAF67501.2 | peptide\_synthetase-like\_protein | BGC0000834 | Saccharide:Hybrid/tailoring saccharide+Other:Aminocoumarin | 29.0 | 49.8 | 173.0 | 7.67e-45 |
| AAW49318.1 | thaxtomin\_synthetase\_A | BGC0000444 | NRP | 31.0 | 37.7 | 178.0 | 7.84e-45 |
| QED88054.1 | nonribosomal\_peptide\_synthetase | BGC0001967 | NRP+Polyketide | 33.0 | 37.0 | 179.0 | 8.27e-45 |
| AZM57024.1 | non-ribosomal\_peptide\_synthetase | BGC0002314 | NRP | 32.0 | 48.0 | 179.0 | 8.28e-45 |
| AZH23792.1 | MgcJ | BGC0001970 | NRP+Polyketide | 31.0 | 37.9 | 178.0 | 8.63e-45 |
| CCC55921.1 | non-ribosomal\_peptide\_synthetase/polyketide\_synthase\_hybrid\_protein | BGC0000973 | NRP+Polyketide:Modular type I polyketide | 29.0 | 53.9 | 179.0 | 9.02e-45 |
| AHD05614.1 | putative\_non-ribosomal\_peptide\_ligase/\_polyketide\_synthase\_hybrid | BGC0001033 | NRP+Polyketide | 29.0 | 48.1 | 179.0 | 9.08e-45 |
| QDX19369.1 | NRPS(A-Ox-T-TE-Tau) | BGC0002295 | NRP+Saccharide | 31.0 | 39.4 | 178.0 | 9.66e-45 |
| CAD70195.1 | non-ribosomal\_peptide\_synthetase | BGC0001047 | NRP+Polyketide | 31.0 | 49.1 | 179.0 | 1.06e-44 |
| ABY66004.1 | type\_II\_beta-Tyr\_adenylation\_domain\_protein | BGC0001008 | Polyketide:Iterative type I polyketide+Polyketide:Enediyne type I polyketide | 30.0 | 52.4 | 177.0 | 1.15e-44 |
| CAF05648.1 | TubC\_protein | BGC0001053 | NRP+Polyketide | 33.0 | 37.3 | 178.0 | 1.2e-44 |
| MBX9445647.1 | amino\_acid\_adenylation\_domain-containing\_protein | BGC0002414 | NRP | 28.0 | 47.9 | 178.0 | 1.24e-44 |
| CBG70278.1 | thaxtomin\_synthetase\_B | BGC0002089 | NRP | 33.0 | 36.9 | 177.0 | 1.39e-44 |
| CAN89633.1 | putative\_hybrid\_non-ribosomal\_peptide\_synthetase/polyketide\_synthase | BGC0001070 | NRP+Polyketide:Modular type I polyketide+Polyketide:Trans-AT type I polyketide | 30.0 | 49.0 | 177.0 | 1.46e-44 |
| AHD05677.1 | nonribosomal\_peptide\_ligase\_subunit | BGC0000402 | NRP | 27.0 | 48.7 | 178.0 | 1.57e-44 |
| BAP16699.1 | nonribosomal\_peptide\_synthetase | BGC0000376 | NRP | 31.0 | 41.5 | 177.0 | 1.71e-44 |
| ALJ49909.1 | TlmI | BGC0001237 | Polyketide | 28.0 | 51.0 | 176.0 | 2.03e-44 |
| WP\_010369428.1 | non-ribosomal\_peptide\_synthetase | BGC0000314 | Polyketide+NRP:Cyclic depsipeptide+Other:Aminocoumarin | 28.0 | 47.9 | 177.0 | 2.06e-44 |
| SDF67357.1 | amino\_acid\_adenylation\_domain-containing\_protein | BGC0002422 | NRP | 28.0 | 45.6 | 176.0 | 2.08e-44 |
| ctg4\_5 |  | BGC0002017 | NRP | 29.0 | 56.0 | 177.0 | 2.14e-44 |
| AHD05616.1 | putative\_non-ribosomal\_peptide\_ligase\_domain\_protein | BGC0001033 | NRP+Polyketide | 28.0 | 36.3 | 177.0 | 2.16e-44 |
| CAQ34918.1 | nonribosomal\_peptide\_synthetase/\_polyketide\_synthase | BGC0000986 | NRP+Polyketide | 27.0 | 51.7 | 177.0 | 2.17e-44 |
| ATG32076.1 | nonribosomal\_peptide\_synthetase | BGC0001750 | NRP+Polyketide | 32.0 | 38.2 | 177.0 | 2.23e-44 |
| AQZ69227.1 | hypothetical\_protein | BGC0001635 | NRP+Polyketide | 31.0 | 46.3 | 177.0 | 2.37e-44 |
| BBC83957.1 | nonribosomal\_peptide\_synthetase | BGC0001636 | NRP | 29.0 | 48.8 | 177.0 | 2.77e-44 |
| ADZ24995.1 | non-ribosomal\_peptide\_synthase/polyketide\_synthase | BGC0000380 | NRP+Polyketide:Modular type I polyketide | 27.0 | 48.7 | 177.0 | 2.86e-44 |
| CCP45167.1 | Peptide\_synthetase\_MbtF\_(peptide\_synthase) | BGC0001021 | NRP+Polyketide | 32.0 | 41.3 | 176.0 | 3.1e-44 |
| QBM78313.1 | non-ribosomal\_peptide\_synthatase | BGC0002542 | Polyketide+NRP | 28.0 | 49.0 | 176.0 | 3.89e-44 |
| EWS95122.1 | hypothetical\_protein | BGC0000306 | NRP:Lipopeptide | 33.0 | 38.1 | 177.0 | 3.94e-44 |
| CAG15010.1 | peptide\_synthetase,\_module\_3 | BGC0000441 | NRP | 29.0 | 46.3 | 175.0 | 4.06e-44 |
| AAW49319.1 | thaxtomin\_synthetase\_B | BGC0000444 | NRP | 33.0 | 37.4 | 176.0 | 4.22e-44 |
| KDM89832.1 | peptide\_synthetase | BGC0002412 | NRP | 27.0 | 46.1 | 175.0 | 4.71e-44 |
| RLV64601.1 | polyketide\_synthase\_of\_type\_I | BGC0001845 | Polyketide+NRP+Other:Aminocoumarin | 30.0 | 48.9 | 176.0 | 5.22e-44 |
| CCJ67645.1 | JagA | BGC0001127 | NRP | 30.0 | 49.0 | 176.0 | 5.97e-44 |
| CAD89775.1 | MelD\_protein | BGC0001010 | NRP+Polyketide:Modular type I polyketide | 32.0 | 37.4 | 176.0 | 6.66e-44 |
| MBE3202942.1 | non-ribosomal\_peptide\_synthetase | BGC0002410 | NRP | 29.0 | 52.1 | 175.0 | 8.49e-44 |
| BAH33409.1 | putative\_non-ribosomal\_peptide\_synthetase | BGC0000371 | NRP | 30.0 | 50.0 | 176.0 | 9.32e-44 |
| EWS95124.1 | hypothetical\_protein | BGC0000306 | NRP:Lipopeptide | 32.0 | 42.0 | 175.0 | 1.23e-43 |
| DAC76734.1 | type\_I\_polyketide\_synthase/non-ribosomal\_peptide\_synthetase | BGC0001885 | NRP+Polyketide | 32.0 | 42.0 | 175.0 | 1.36e-43 |
| SDF67478.1 | Phosphopantetheine\_attachment\_site | BGC0002422 | NRP | 27.0 | 48.8 | 174.0 | 1.37e-43 |
| BAF50720.1 | hybrid\_non\_ribosomal\_peptide\_synthetase-polyketide\_synthase | BGC0001116 | NRP+Polyketide | 31.0 | 40.5 | 175.0 | 1.38e-43 |
| AFO59871.1 | three-domain\_carboxylic\_acid\_reductase | BGC0000175 | Polyketide:Trans-AT type I polyketide | 27.0 | 74.1 | 174.0 | 1.47e-43 |
| ABA70582.1 | alpha-aminoadypil-cysteinyl-valine\_synthetase | BGC0000404 | NRP | 29.0 | 49.0 | 175.0 | 1.57e-43 |
| ABR12615.1 | ACV\_synthetase | BGC0000405 | NRP:Beta-lactam | 29.0 | 49.0 | 175.0 | 1.57e-43 |
| CAL69597.1 | PKS-NRPS | BGC0001049 | NRP+Polyketide:Iterative type I polyketide | 25.0 | 66.2 | 175.0 | 1.61e-43 |
| CAJ21198.2 | non-ribosomal\_peptide\_synthetase | BGC0000297 | NRP:Glycopeptide+Polyketide:Other polyketide+Saccharide:Hybrid/tailoring saccharide | 29.0 | 47.5 | 174.0 | 1.76e-43 |
| AXG47411.1 | hybrid\_non-ribosomal\_peptide\_synthetase/type\_I\_polyketide\_synthase | BGC0002715 | NRP+Polyketide | 26.0 | 47.4 | 174.0 | 2.07e-43 |
| ALK27915.1 | non-ribosomal\_peptide\_synthase | BGC0001233 | NRP | 28.0 | 51.5 | 174.0 | 2.33e-43 |
| PLB34720.1 | polyketide\_synthase | BGC0002749 | NRP+Polyketide | 28.0 | 47.7 | 174.0 | 2.35e-43 |
| AIW82277.1 | PuwA | BGC0001125 | NRP+Polyketide | 32.0 | 37.4 | 174.0 | 2.48e-43 |
| AHI59109.1 | locillomycin\_synthase\_B | BGC0001005 | NRP+Polyketide | 24.0 | 47.9 | 174.0 | 2.72e-43 |
| CAE53351.1 | non-ribosomal\_peptide\_synthetase | BGC0000440 | NRP:Glycopeptide | 29.0 | 46.3 | 172.0 | 2.82e-43 |
| QDG75035.1 | mixed\_type\_I\_polyketide\_synthase\_-\_peptide\_synthetase | BGC0002068 | NRP+Polyketide | 29.0 | 48.6 | 174.0 | 3.07e-43 |
| AQV04224.1 | SwnK | BGC0001793 | NRP+Polyketide | 28.0 | 47.5 | 174.0 | 3.08e-43 |
| WP\_054234617.1 | non-ribosomal\_peptide\_synthetase | BGC0002014 | NRP+Polyketide | 30.0 | 37.5 | 174.0 | 3.09e-43 |
| simA |  | BGC0000334 | NRP | 31.0 | 39.4 | 174.0 | 3.22e-43 |
| AXA20091.1 | hybrid\_trans-AT\_PKS/NRPS\_LgaB | BGC0001646 | NRP+Polyketide | 28.0 | 47.9 | 174.0 | 3.91e-43 |
| ADH04660.1 | TugD | BGC0001342 | NRP+Polyketide | 32.0 | 36.4 | 174.0 | 3.93e-43 |
| WP\_010369430.1 | non-ribosomal\_peptide\_synthetase | BGC0000314 | Polyketide+NRP:Cyclic depsipeptide+Other:Aminocoumarin | 28.0 | 49.8 | 173.0 | 4.47e-43 |
| QEO74983.1 | omn8 | BGC0002078 | NRP:Cyclic depsipeptide | 29.0 | 49.6 | 173.0 | 5.1e-43 |
| ABI26078.1 | OciB | BGC0000331 | NRP | 30.0 | 38.5 | 173.0 | 6.41e-43 |
| RGP42808.1 | non-ribosomal\_peptide\_synthetase | BGC0002696 | NRP | 29.0 | 49.4 | 172.0 | 8.19e-43 |
| AAQ59905.1 | synthetase\_CbsF | BGC0002680 | NRP | 29.0 | 47.3 | 172.0 | 9.65e-43 |
| ACN39014.1 | putative\_nonribosomal\_peptide\_synthetase\_TomA | BGC0000448 | NRP | 29.0 | 48.2 | 167.0 | 9.93e-43 |
| AJI44176.1 | nonribosomal\_peptide\_synthetase | BGC0001193 | NRP | 29.0 | 48.2 | 171.0 | 1.08e-42 |
| ACN39728.1 | SibE | BGC0000428 | NRP | 27.0 | 50.0 | 166.0 | 1.08e-42 |
| AAN85522.1 | hybrid\_nonribosomal\_peptide\_synthetase\_/\_polyketide\_synthase | BGC0001101 | NRP+Polyketide:Modular type I polyketide+Polyketide:Trans-AT type I polyketide | 30.0 | 41.7 | 172.0 | 1.09e-42 |
| CDG12864.1 | non-ribosomal\_peptide\_synthetase | BGC0001415 | NRP+Polyketide | 26.0 | 49.5 | 172.0 | 1.13e-42 |
| AJW76709.1 | DsaG | BGC0001196 | NRP | 29.0 | 54.6 | 172.0 | 1.23e-42 |
| CAJ34375.1 | NRPS | BGC0000445 | NRP:Cyclic depsipeptide | 33.0 | 36.1 | 172.0 | 1.31e-42 |
| ANG60380.1 | nonribosomal\_peptide\_synthetase\_BudB | BGC0001434 | NRP | 31.0 | 37.2 | 171.0 | 1.53e-42 |
| EFY95969.1 | polyketide\_synthase | BGC0002270 | NRP+Polyketide | 27.0 | 47.2 | 171.0 | 1.57e-42 |
| ALG65319.1 | Cal17 | BGC0001297 | NRP | 33.0 | 36.9 | 171.0 | 1.59e-42 |
| AGK13426.1 | enterochelin\_sythetase\_component\_F\_(Serine-activating\_enzyme)\_(Seryl-AMP\_ligase) | BGC0002528 | NRP | 30.0 | 49.7 | 171.0 | 1.68e-42 |
| AEE88284.1 | CurF | BGC0000976 | NRP+Polyketide:Modular type I polyketide | 27.0 | 50.3 | 171.0 | 1.73e-42 |
| AAT70101.1 | CurF | BGC0001165 | NRP+Polyketide:Modular type I polyketide | 27.0 | 50.3 | 171.0 | 1.73e-42 |
| CAJ96471.1 | non-ribosomal\_peptide\_synthetase | BGC0000330 | NRP:NRP siderophore | 28.0 | 50.6 | 170.0 | 1.81e-42 |
| AKQ52532.1 | nonribosomal\_peptide\_synthetase | BGC0002533 | NRP+Polyketide | 27.0 | 49.3 | 171.0 | 1.83e-42 |
| QNH68024.1 | PfpA | BGC0002268 | Polyketide+NRP | 25.0 | 69.7 | 171.0 | 1.84e-42 |
| MBD2892722.1 | D-alanine--D-alanyl\_carrier\_protein\_ligase | BGC0002718 | NRP | 32.0 | 40.6 | 171.0 | 1.84e-42 |
| ABC35796.1 | NRPS-PKS\_hybrid | BGC0001102 | NRP+Polyketide:Modular type I polyketide+Polyketide:Trans-AT type I polyketide | 30.0 | 38.9 | 171.0 | 1.84e-42 |
| AQX14497.1 | monobactam\_NRPS\_scaffold\_4 | BGC0001672 | NRP | 25.0 | 47.9 | 171.0 | 2.05e-42 |
| EPH46596.1 | putative\_Linear\_gramicidin\_synthase\_subunit\_C | BGC0001519 | NRP+Polyketide | 30.0 | 37.3 | 171.0 | 2.87e-42 |
| AQH32481.1 | hybrid\_polyketide\_synthase/peptide\_synthetase | BGC0001667 | NRP+Polyketide | 25.0 | 51.8 | 171.0 | 3.06e-42 |
| AAP92505.1 | nonribosomal\_peptide\_synthetase | BGC0000458 | NRP | 30.0 | 50.2 | 165.0 | 3.15e-42 |
| WP\_141576257.1 | non-ribosomal\_peptide\_synthetase | BGC0002686 | NRP | 30.0 | 46.8 | 171.0 | 3.16e-42 |
| E0F75\_025360 | amino\_acid\_adenylation\_domain-containing\_protein | BGC0002340 | NRP+Other | 29.0 | 48.5 | 169.0 | 3.62e-42 |
| ALD82525.1 | non-ribosomal\_peptide\_synthase | BGC0001212 | NRP+Polyketide | 31.0 | 38.2 | 169.0 | 4.51e-42 |
| AFD30954.1 | CrmA | BGC0000966 | NRP+Polyketide | 29.0 | 45.1 | 170.0 | 4.67e-42 |
| AEC14346.1 | nonribosomal\_peptide\_synthetase | BGC0000377 | NRP | 24.0 | 46.7 | 170.0 | 4.76e-42 |
| ARR97038.1 | SphE | BGC0001780 | NRP | 29.0 | 49.0 | 170.0 | 4.83e-42 |
| ATQ39428.1 | cyclosporin\_C\_synthetase | BGC0001565 | NRP | 27.0 | 49.4 | 170.0 | 4.85e-42 |
| RLV71193.1 | non-ribosomal\_peptide\_synthetase | BGC0001846 | NRP+Saccharide:Hybrid/tailoring saccharide | 30.0 | 46.9 | 170.0 | 5.17e-42 |
| ABM34276.1 | amino\_acid\_adenylation\_domain\_protein | BGC0002419 | NRP+Polyketide | 30.0 | 51.7 | 169.0 | 5.21e-42 |
| BBG06551.1 | AMP\_binding\_enzyme | BGC0001925 | Alkaloid | 29.0 | 42.5 | 163.0 | 5.87e-42 |
| ABA59547.1 | NRPS | BGC0000453 | NRP:Cyclic depsipeptide | 29.0 | 46.9 | 169.0 | 6.86e-42 |
| WP\_053065270.1 | non-ribosomal\_peptide\_synthetase | BGC0001330 | NRP:Cyclic depsipeptide+Polyketide:Modular type I polyketide | 32.0 | 38.0 | 169.0 | 6.95e-42 |
| WP\_078857609.1 | non-ribosomal\_peptide\_synthetase | BGC0001368 | NRP | 30.0 | 48.1 | 164.0 | 7.59e-42 |
| DAC80525.1 | malonyl\_CoA-acyl\_carrier\_protein\_transacylase | BGC0001841 | NRP+Polyketide | 30.0 | 49.3 | 169.0 | 8.42e-42 |
| ABY83142.1 | Azi3 | BGC0000960 | NRP+Polyketide | 29.0 | 49.0 | 168.0 | 8.46e-42 |
| AJO72702.1 | Acyl-CoA\_ligase/oxidoreductase | BGC0001381 | Polyketide | 27.0 | 82.6 | 168.0 | 9.27e-42 |
| CBJ90358.1 | putative\_Peptide\_synthetase | BGC0000465 | NRP | 31.0 | 36.5 | 169.0 | 9.46e-42 |
| ABW70809.1 | PchE | BGC0002475 | NRP | 29.0 | 48.7 | 167.0 | 1.22e-41 |
| CAJ34374.1 | NRPS\_protein | BGC0000445 | NRP:Cyclic depsipeptide | 31.0 | 42.2 | 168.0 | 1.41e-41 |
| AAF62881.1 | EpoB | BGC0000991 | NRP+Polyketide | 31.0 | 36.6 | 167.0 | 1.61e-41 |
| EJK79843.1 | amino\_acid\_adenylation\_enzyme/thioester\_reductase\_family\_protein | BGC0000436 | NRP | 28.0 | 53.5 | 168.0 | 1.63e-41 |
| QCE43603.1 | nonribosomal\_peptide\_synthetase\_(NRPS),\_subunit\_2 | BGC0001834 | NRP | 28.0 | 46.5 | 168.0 | 1.73e-41 |
| AAZ23075.1 | peptide\_synthetase | BGC0000291 | NRP | 32.0 | 39.3 | 168.0 | 2.29e-41 |
| AKJ70942.1 | non-ribosomal\_peptide\_synthetase | BGC0002611 | NRP | 29.0 | 48.1 | 168.0 | 2.33e-41 |
| MBN3579113.1 | amino\_acid\_adenylation\_domain-containing\_protein | BGC0002613 | NRP+Polyketide | 26.0 | 48.9 | 167.0 | 2.77e-41 |
| QCX41915.1 | Mhr9 | BGC0001956 | Polyketide | 30.0 | 49.1 | 165.0 | 2.81e-41 |
| QHD26315.1 | non-ribosomal\_peptide\_synthetase | BGC0002479 | Polyketide+NRP+Saccharide | 30.0 | 49.1 | 165.0 | 2.81e-41 |
| WP\_050383084.1 | non-ribosomal\_peptide\_synthetase | BGC0001451 | NRP | 32.0 | 36.5 | 167.0 | 3.15e-41 |
| AQM58286.1 | non-ribosomal\_peptide\_synthase | BGC0001816 | NRP+Polyketide | 29.0 | 49.8 | 167.0 | 3.21e-41 |
| WP\_051700111.1 | non-ribosomal\_peptide\_synthetase | BGC0001368 | NRP | 32.0 | 46.6 | 166.0 | 3.52e-41 |
| EET76303.1 | AMP-binding\_enzyme | BGC0002685 | NRP | 29.0 | 54.2 | 166.0 | 3.76e-41 |
| ABD14712.1 | cesB | BGC0000320 | NRP:Cyclic depsipeptide | 27.0 | 41.1 | 167.0 | 4.24e-41 |
| ACM79806.1 | ZmaB | BGC0001059 | NRP+Polyketide | 26.0 | 48.7 | 166.0 | 4.71e-41 |
| AJD47483.1 | amino\_acid\_adenylation\_domain-containing\_protein | BGC0002418 | NRP+Polyketide | 29.0 | 48.2 | 165.0 | 5.1e-41 |
| KUM80513.1 | hypothetical\_protein | BGC0001562 | NRP | 29.0 | 47.1 | 166.0 | 5.52e-41 |
| AID65225.1 | nonribosomal\_peptide\_synthetase | BGC0000335 | NRP+Polyketide | 27.0 | 47.6 | 166.0 | 5.71e-41 |
| ABC34137.1 | peptide\_synthetase,\_putative | BGC0000961 | NRP+Polyketide | 29.0 | 41.4 | 160.0 | 6e-41 |
| ACB46193.1 | nonribosomal\_peptide\_synthetase | BGC0000989 | NRP+Polyketide | 31.0 | 36.6 | 166.0 | 6.33e-41 |
| ACG60776.1 | NRPS(AL/ACP/C/A/PCP/C/A) | BGC0001058 | NRP:Glycopeptide+Polyketide:Modular type I polyketide+Saccharide:Hybrid/tailoring saccharide | 31.0 | 44.1 | 166.0 | 7.34e-41 |
| AHD05617.1 | putative\_non-ribosomal\_peptide\_ligase\_domain\_protein | BGC0001033 | NRP+Polyketide | 26.0 | 49.4 | 165.0 | 7.79e-41 |
| AAF26925.1 | nonribosomal\_peptide\_synthetase | BGC0000988 | NRP+Polyketide | 31.0 | 36.6 | 165.0 | 8.32e-41 |
| EFG10344.1 | Non-ribosomal\_peptide\_synthetase | BGC0000373 | NRP | 28.0 | 47.6 | 164.0 | 9.96e-41 |
| XP\_003044554.1 | uncharacterized\_protein | BGC0001768 | NRP | 28.0 | 48.6 | 166.0 | 1.16e-40 |
| AXG46164.1 | non-ribosomal\_peptide\_synthetase | BGC0002713 | NRP | 30.0 | 37.7 | 165.0 | 1.22e-40 |
| ABC36450.1 | peptide\_synthetase-like\_protein | BGC0000386 | NRP:NRP siderophore | 31.0 | 41.6 | 165.0 | 1.35e-40 |
| CAC17500.1 | putative\_non-ribosomal\_peptide\_synthase | BGC0000324 | NRP | 31.0 | 38.9 | 165.0 | 1.37e-40 |
| ADB12489.1 | EpoB | BGC0000990 | NRP+Polyketide | 31.0 | 36.6 | 164.0 | 1.44e-40 |
| BAD55613.1 | putative\_non-ribosomal\_peptide\_synthetase | BGC0001027 | NRP+Polyketide | 28.0 | 46.6 | 164.0 | 1.47e-40 |
| QWT72293.1 | non-ribosomal\_peptide\_synthetase | BGC0002430 | NRP+Saccharide | 28.0 | 49.0 | 165.0 | 1.58e-40 |
| CAD70194.1 | non-ribosomal\_peptide\_synthetase | BGC0001047 | NRP+Polyketide | 29.0 | 49.2 | 164.0 | 1.76e-40 |
| CEK23365.1 | conserved\_hypothetical\_protein | BGC0001716 | NRP | 27.0 | 41.5 | 164.0 | 1.85e-40 |
| NAO96317.1 | amino\_acid\_adenylation\_domain-containing\_protein | BGC0002117 | NRP | 28.0 | 48.5 | 163.0 | 2.13e-40 |
| AGA37267.1 | NRPS | BGC0000816 | NRP+Alkaloid | 24.0 | 78.5 | 164.0 | 2.69e-40 |
| WP\_004571777.1 | non-ribosomal\_peptide\_synthetase | BGC0001760 | NRP | 32.0 | 42.1 | 164.0 | 2.98e-40 |
| CAL80821.1 | sylD-like\_NRPS/PKS | BGC0000997 | NRP+Polyketide | 28.0 | 48.6 | 164.0 | 3.22e-40 |
| AZH23819.1 | MgiR | BGC0001971 | NRP+Polyketide | 31.0 | 33.0 | 164.0 | 3.27e-40 |
| QXJ21807.1 | amino\_acid\_adenylation\_domain-containing\_protein | BGC0002370 | NRP | 27.0 | 50.3 | 164.0 | 3.53e-40 |
| ACK77757.1 | putative\_indigoidine\_synthase\_IndC | BGC0000727 | Saccharide | 29.0 | 36.9 | 163.0 | 3.84e-40 |
| AJQ95677.1 | polyketide\_synthase\_modules-related\_protein | BGC0002046 | NRP+Polyketide:Trans-AT type I polyketide | 28.0 | 50.4 | 164.0 | 4.08e-40 |
| BAW32334.1 | hybrid\_cis-AT\_polyketide\_synthase\_-\_nonribosomal\_peptide\_synthetase | BGC0001631 | NRP+Polyketide | 27.0 | 48.9 | 164.0 | 4.21e-40 |
| CBJ89761.1 | Non-ribosomal\_peptide\_synthase\_involved\_in\_xenocoumacin\_synthesis | BGC0001054 | NRP+Polyketide:Modular type I polyketide | 26.0 | 50.8 | 161.0 | 5.98e-40 |
| BAF50727.1 | hybrid\_polyketide\_synthase-non\_ribosomal\_peptide\_synthetase | BGC0001116 | NRP+Polyketide | 27.0 | 49.9 | 163.0 | 6.01e-40 |
| CAQ18839.1 | hybrid\_polyketide\_synthase/nonribosomal\_polypetide\_synthetase | BGC0000954 | NRP+Polyketide:Modular type I polyketide | 31.0 | 37.8 | 163.0 | 6.76e-40 |
| WP\_019032753.1 | non-ribosomal\_peptide\_synthetase | BGC0001331 | NRP:Cyclic depsipeptide+Polyketide:Modular type I polyketide | 32.0 | 38.0 | 162.0 | 7.13e-40 |
| AMM63162.1 | AniA | BGC0001371 | NRP | 28.0 | 47.2 | 163.0 | 7.92e-40 |
| ABE35422.1 | Non-ribosomal\_peptide\_synthase | BGC0002421 | NRP | 28.0 | 49.6 | 162.0 | 8.91e-40 |
| QCC62999.1 | BII-rafflesfungin\_nonribosomal\_protein\_synthetase | BGC0001966 | NRP+Polyketide | 28.0 | 49.3 | 162.0 | 1.07e-39 |
| CCA89326.1 | mixed\_trans-AT\_type\_I\_polyketide\_synthase/nonribosomal\_peptide\_synthetase | BGC0001111 | NRP+Polyketide:Trans-AT type I polyketide | 29.0 | 47.9 | 162.0 | 1.08e-39 |
| CBJ90359.1 | putative\_Peptide\_synthetase | BGC0000465 | NRP | 30.0 | 36.5 | 161.0 | 1.6e-39 |
| AFY58522.1 | non-ribosomal\_peptide\_synthase/amino\_acid\_adenylation\_enzyme | BGC0002411 | NRP+Polyketide | 26.0 | 47.1 | 161.0 | 1.81e-39 |
| QEO75074.1 | condensation\_domain-containing\_protein | BGC0002079 | NRP:Cyclic depsipeptide | 29.0 | 45.0 | 160.0 | 1.99e-39 |
| WP\_010369425.1 | non-ribosomal\_peptide\_synthetase | BGC0000314 | Polyketide+NRP:Cyclic depsipeptide+Other:Aminocoumarin | 27.0 | 48.1 | 160.0 | 2.37e-39 |
| WP\_081656241.1 | non-ribosomal\_peptide\_synthetase | BGC0001467 | NRP:Cyclic depsipeptide+Polyketide:Modular type I polyketide | 30.0 | 40.5 | 161.0 | 2.37e-39 |
| ARR97039.1 | SphF | BGC0001780 | NRP | 27.0 | 41.2 | 160.0 | 2.92e-39 |
| ACN69986.1 | proline\_adenyltransferase | BGC0000079 | Polyketide | 31.0 | 41.2 | 154.0 | 3.01e-39 |
| BAH43869.1 | linear\_pentadecapeptide\_gramicidin\_synthetase\_LgrA | BGC0000367 | NRP | 26.0 | 47.4 | 160.0 | 3.06e-39 |
| AQV04230.1 | SwnK | BGC0001794 | NRP+Polyketide | 26.0 | 48.1 | 160.0 | 4.19e-39 |
| ADH04641.1 | TgaC | BGC0001051 | NRP+Polyketide:Modular type I polyketide | 30.0 | 45.0 | 160.0 | 5.22e-39 |
| QGZ36672.1 | amino\_acid\_adenylation\_domain-containing\_protein | BGC0002082 | NRP+Polyketide | 28.0 | 48.5 | 160.0 | 6.86e-39 |
| AQZ71347.1 | hypothetical\_protein | BGC0001635 | NRP+Polyketide | 30.0 | 45.9 | 159.0 | 6.97e-39 |
| ABV56582.1 | KtzB | BGC0000378 | NRP | 28.0 | 40.8 | 153.0 | 9.48e-39 |
| QBA57735.1 | NRPS | BGC0002377 | NRP | 32.0 | 37.7 | 159.0 | 9.55e-39 |
| AAS92545.1 | SirP | BGC0001044 | NRP | 27.0 | 48.6 | 159.0 | 1.17e-38 |
| AAK89719.2 | peptide\_synthetase,\_siderophore\_biosynthesis\_protein | BGC0002107 | NRP+Polyketide | 27.0 | 50.2 | 159.0 | 1.25e-38 |
| CAD89778.1 | MelG\_protein | BGC0001010 | NRP+Polyketide:Modular type I polyketide | 29.0 | 38.1 | 158.0 | 1.35e-38 |
| QXJ21808.1 | amino\_acid\_adenylation\_domain-containing\_protein | BGC0002370 | NRP | 30.0 | 38.6 | 159.0 | 1.39e-38 |
| ADC79642.1 | TamD | BGC0001052 | NRP+Polyketide:Modular type I polyketide | 28.0 | 48.6 | 157.0 | 1.72e-38 |
| CCA53804.1 | pyochelin\_synthetase\_F | BGC0001801 | NRP | 31.0 | 36.7 | 158.0 | 1.83e-38 |
| EFL06864.1 | non-ribosomal\_peptide\_synthetase | BGC0000300 | NRP | 28.0 | 46.8 | 157.0 | 1.92e-38 |
| ABP57745.1 | DepA | BGC0000993 | NRP:Cyclic depsipeptide+Polyketide:Modular type I polyketide | 29.0 | 47.5 | 157.0 | 2.29e-38 |
| EEP98516.1 | Peptide\_synthetase | BGC0002091 | NRP | 25.0 | 46.0 | 157.0 | 2.55e-38 |
| AEF16059.1 | non-ribosomal\_peptide\_synthetase | BGC0000953 | Saccharide:Aminoglycoside | 30.0 | 41.4 | 155.0 | 2.56e-38 |
| QDG75033.1 | mixed\_type\_I\_polyketide\_synthase\_-\_peptide\_synthetase | BGC0002068 | NRP+Polyketide | 28.0 | 48.2 | 158.0 | 2.62e-38 |
| DAC80529.1 | malonyl\_CoA-acyl\_carrier\_protein\_transacylase | BGC0001878 | NRP+Polyketide | 30.0 | 41.8 | 157.0 | 3.93e-38 |
| QRN75754.1 | Polyketide\_synthase | BGC0002114 | NRP+Polyketide | 29.0 | 42.3 | 157.0 | 5.37e-38 |
| AVV61987.1 | putative\_non-ribosomal\_peptide\_synthetase | BGC0001477 | NRP+Polyketide:Modular type I polyketide | 28.0 | 48.4 | 155.0 | 6.56e-38 |
| ABC39418.1 | dihydroaeruginoic\_acid\_synthetase | BGC0000964 | NRP:Cyclic depsipeptide+Polyketide:Trans-AT type I polyketide | 28.0 | 47.9 | 156.0 | 6.8e-38 |
| CBF77087.1 | conserved\_hypothetical\_protein | BGC0001679 | NRP | 28.0 | 49.4 | 156.0 | 6.82e-38 |
| AXM42949.1 | hybrid\_type\_1\_PKS/NRPS | BGC0001941 | NRP+Polyketide | 30.0 | 36.4 | 156.0 | 6.87e-38 |
| AZH23788.1 | MgcR | BGC0001970 | NRP+Polyketide | 27.0 | 38.5 | 156.0 | 8.4e-38 |
| ABB69752.1 | PlaP4 | BGC0000654 | Terpene+Saccharide:Hybrid/tailoring saccharide | 30.0 | 42.4 | 151.0 | 9.56e-38 |
| ESU15173.1 | hypothetical\_protein | BGC0002186 | NRP+Polyketide | 27.0 | 48.8 | 156.0 | 9.68e-38 |
| AFK57221.1 | DidJ | BGC0000985 | Polyketide+NRP:Cyclic depsipeptide | 31.0 | 32.8 | 155.0 | 1.02e-37 |
| EJK79842.1 | amino\_acid\_adenylation\_enzyme/thioester\_reductase\_family\_protein | BGC0000436 | NRP | 27.0 | 48.4 | 155.0 | 1.03e-37 |
| AGE11891.1 | nonribosomal\_peptide\_synthetase | BGC0000366 | NRP | 28.0 | 47.0 | 155.0 | 1.15e-37 |
| ANG60381.1 | nonribosomal\_peptide\_synthetase\_BudC | BGC0001434 | NRP | 30.0 | 36.6 | 155.0 | 1.29e-37 |
| QUS58936.1 | non-ribosomal\_peptide\_synthetase | BGC0002123 | NRP+Polyketide | 27.0 | 47.9 | 155.0 | 1.52e-37 |
| EKJ70673.1 | hypothetical\_protein | BGC0002188 | NRP+Polyketide | 28.0 | 46.9 | 155.0 | 1.82e-37 |
| AXM42952.1 | non-ribosomal\_peptide\_synthetase | BGC0001941 | NRP+Polyketide | 32.0 | 30.7 | 154.0 | 2.06e-37 |
| AGN74892.1 | nonribosomal\_peptide\_synthetase/polyketide\_synthase\_hybrid\_protein | BGC0000459 | NRP:Cyclic depsipeptide+Polyketide:Trans-AT type I polyketide | 31.0 | 41.4 | 155.0 | 2.19e-37 |
| QRD93053.1 | putative\_nonribosomal\_peptide\_synthase | BGC0002160 | NRP | 29.0 | 42.9 | 155.0 | 2.36e-37 |
| DAC80540.1 | nrps | BGC0001840 | NRP+Polyketide | 27.0 | 48.2 | 154.0 | 2.65e-37 |
| AAW03330.1 | CtaG | BGC0000982 | NRP+Polyketide | 33.0 | 29.9 | 154.0 | 2.7e-37 |
| AAF19815.1 | mtaG | BGC0001024 | NRP+Polyketide:Modular type I polyketide | 32.0 | 30.0 | 154.0 | 2.71e-37 |
| QXJ21809.1 | amino\_acid\_adenylation\_domain-containing\_protein | BGC0002370 | NRP | 31.0 | 35.1 | 154.0 | 3.32e-37 |
| BAU50944.1 | nonribosomal\_peptide\_synthetase | BGC0001379 | NRP | 30.0 | 48.6 | 150.0 | 3.88e-37 |
| AHD05627.1 | putative\_non-ribosomal\_peptide\_ligase\_domain\_protein | BGC0001033 | NRP+Polyketide | 27.0 | 49.1 | 153.0 | 4.16e-37 |
| CBJ90288.1 | Peptide\_synthetase | BGC0000416 | NRP | 28.0 | 36.6 | 153.0 | 4.29e-37 |
| WP\_013184318.1 | non-ribosomal\_peptide\_synthetase | BGC0001692 | NRP | 28.0 | 36.6 | 153.0 | 4.29e-37 |
| ACS20358.1 | amino\_acid\_adenylation\_domain\_protein | BGC0002420 | NRP+Polyketide | 27.0 | 47.1 | 153.0 | 5e-37 |
| CAJ76290.1 | putative\_non-ribosomal\_peptide\_synthase | BGC0000972 | NRP+Polyketide:Modular type I polyketide+Polyketide:Trans-AT type I polyketide | 25.0 | 50.2 | 153.0 | 5.23e-37 |
| ALG65340.1 | Var5 | BGC0002416 | NRP+Polyketide | 28.0 | 47.9 | 152.0 | 6e-37 |
| AOC89000.1 | putative\_nonribosomal\_peptide\_synthetase | BGC0001652 | NRP | 29.0 | 46.7 | 149.0 | 6.15e-37 |
| AQZ42163.1 | putative\_nonribosomal\_peptide\_synthase | BGC0001820 | NRP | 26.0 | 48.8 | 153.0 | 6.58e-37 |
| CBJ90289.1 | peptide\_synthetase | BGC0000416 | NRP | 28.0 | 36.6 | 153.0 | 6.6e-37 |
| WP\_013184319.1 | non-ribosomal\_peptide\_synthetase | BGC0001692 | NRP | 28.0 | 36.6 | 153.0 | 6.6e-37 |
| CAC17499.1 | putative\_non-ribosomal\_peptide\_synthase | BGC0000324 | NRP | 27.0 | 48.6 | 153.0 | 6.95e-37 |
| ALK21570.1 | non-ribosomal\_peptide\_synthetase | BGC0002678 | NRP | 28.0 | 36.9 | 152.0 | 8.24e-37 |
| CAD15508.1 | polyketide\_synthase/non-ribosomal\_peptide\_synthetase | BGC0001014 | NRP:NRP siderophore+Polyketide:Modular type I polyketide+Polyketide:Iterative type I polyketide | 30.0 | 36.5 | 153.0 | 8.4e-37 |
| CEK23366.1 | putative\_Ornithine\_racemase | BGC0001716 | NRP | 27.0 | 46.3 | 152.0 | 1.04e-36 |
| QIW91877.1 | NRPS | BGC0002543 | NRP | 27.0 | 49.1 | 152.0 | 1.19e-36 |
| ADD82940.1 | Bat2 | BGC0001099 | NRP+Polyketide:Modular type I polyketide+Polyketide:Trans-AT type I polyketide | 27.0 | 47.9 | 152.0 | 1.2e-36 |
| OAL11436.1 | hypothetical\_protein | BGC0001570 | NRP | 29.0 | 51.3 | 149.0 | 1.25e-36 |
| AQX14499.1 | monobactam\_NRPS\_scaffold\_1 | BGC0001672 | NRP | 24.0 | 48.2 | 152.0 | 1.32e-36 |
| CDG12865.1 | non-ribosomal\_peptide\_synthetase | BGC0001415 | NRP+Polyketide | 27.0 | 47.1 | 152.0 | 1.61e-36 |
| WP\_051462298.1 | non-ribosomal\_peptide\_synthetase | BGC0001873 | NRP:Lipopeptide | 26.0 | 49.0 | 152.0 | 1.64e-36 |
| AEC14348.1 | nonribosomal\_peptide\_synthetase | BGC0000377 | NRP | 27.0 | 47.5 | 151.0 | 2.86e-36 |
| CAJ76298.1 | putative\_hybrid\_polyketide-non-ribosomal\_peptide\_synthetase | BGC0000972 | NRP+Polyketide:Modular type I polyketide+Polyketide:Trans-AT type I polyketide | 26.0 | 48.2 | 151.0 | 3.06e-36 |
| ctg1\_orf18 |  | BGC0001329 | Polyketide+NRP:Cyclic depsipeptide | 33.0 | 33.4 | 143.0 | 3.06e-36 |
| ABW70808.1 | PchF | BGC0002475 | NRP | 31.0 | 36.1 | 150.0 | 3.19e-36 |
| CAA79245.2 | enniatin\_synthetase | BGC0000342 | NRP | 29.0 | 37.4 | 150.0 | 3.98e-36 |
| ARO38317.1 | nonribosomal\_peptide\_synthetase | BGC0001560 | NRP+Polyketide | 27.0 | 47.4 | 150.0 | 4.54e-36 |
| AGZ15455.1 | non-ribosomal\_peptide\_synthetase | BGC0001036 | NRP+Polyketide | 29.0 | 41.6 | 145.0 | 5.94e-36 |
| CDE97356.1 | plipastatin\_synthase\_subunit\_C | BGC0001686 | NRP | 24.0 | 48.6 | 150.0 | 6.35e-36 |
| ABO15844.1 | amino\_acid\_adenyltransferase | BGC0000130 | Polyketide | 29.0 | 39.6 | 143.0 | 8.08e-36 |
| ABB90279.1 | non-ribosomal\_peptide\_synthetase | BGC0001057 | NRP+Polyketide | 24.0 | 72.5 | 149.0 | 8.15e-36 |
| CAJ45639.1 | vanchrobactin\_non\_ribosomal\_peptide\_synthetase | BGC0000454 | NRP | 27.0 | 43.7 | 149.0 | 8.73e-36 |
| ADQ74618.1 | amino\_acid\_adenylation\_protein | BGC0000921 | Polyketide+NRP+Other:Shikimate-derived | 28.0 | 48.7 | 145.0 | 9.92e-36 |
| ORC16618.1 | hypothetical\_protein | BGC0001341 | NRP | 27.0 | 43.5 | 149.0 | 1.2e-35 |
| AAC83657.1 | pyochelin\_synthetase | BGC0000412 | NRP | 28.0 | 37.3 | 149.0 | 1.24e-35 |
| AAQ90177.1 | putative\_acyl-CoA\_synthetase | BGC0000128 | Polyketide | 28.0 | 41.8 | 143.0 | 1.66e-35 |
| DAB41917.1 | ArzO\_-\_NRPS\_(Cy,\_A,\_Ox,\_PCP) | BGC0001884 | NRP+Polyketide | 28.0 | 36.5 | 148.0 | 1.81e-35 |
| BAY02129.1 | barbamide\_biosynthesis\_protein\_BarG | BGC0002532 | NRP+Polyketide | 26.0 | 47.0 | 147.0 | 1.92e-35 |
| ABS75103.1 | non-ribosomal\_peptide\_synthetase | BGC0002641 | NRP | 24.0 | 48.7 | 148.0 | 2.07e-35 |
| AXG22420.1 | proline\_adenyltransferase | BGC0002024 | Polyketide | 30.0 | 40.1 | 142.0 | 3.11e-35 |
| XP\_020058100.1 | uncharacterized\_protein | BGC0001220 | NRP | 25.0 | 50.6 | 147.0 | 4.01e-35 |
| AWS21276.1 | amino\_acid\_adenyltransferase | BGC0001934 | Polyketide | 29.0 | 41.2 | 141.0 | 5.1e-35 |
| AZY92000.1 | proline\_adenylation\_protein | BGC0002022 | Polyketide | 29.0 | 41.2 | 141.0 | 5.1e-35 |
| CBJ90287.1 | peptide\_synthetase | BGC0000416 | NRP | 28.0 | 36.1 | 146.0 | 7.6e-35 |
| WP\_013184317.1 | non-ribosomal\_peptide\_synthetase | BGC0001692 | NRP | 28.0 | 36.1 | 146.0 | 7.6e-35 |
| KIA75458.1 | hypothetical\_protein | BGC0002208 | NRP | 26.0 | 54.2 | 146.0 | 8.26e-35 |
| EAL89049.1 | nonribosomal\_peptide\_synthetase | BGC0000355 | NRP | 27.0 | 50.6 | 146.0 | 8.28e-35 |
| QKW60393.1 | amino\_acid\_adenylation\_domain-containing\_protein | BGC0002288 | NRP | 27.0 | 48.4 | 145.0 | 8.76e-35 |
| ACZ66258.1 | APS1 | BGC0000304 | NRP | 26.0 | 49.1 | 146.0 | 1.13e-34 |
| OJJ99913.1 | hypothetical\_protein | BGC0002225 | Terpene | 27.0 | 49.1 | 145.0 | 1.18e-34 |
| ADI59531.1 | CorI | BGC0001091 | NRP+Polyketide | 28.0 | 42.4 | 145.0 | 1.42e-34 |
| ABK36076.1 | nonribosomal\_peptide\_synthetase | BGC0001502 | NRP | 29.0 | 47.0 | 145.0 | 1.53e-34 |
| ctg1\_orf10 |  | BGC0000321 | NRP | 27.0 | 53.2 | 145.0 | 1.6e-34 |
| DAD54576.1 | NRPS-like\_tryptophan\_epimerase | BGC0002256 | NRP+Other | 26.0 | 50.6 | 144.0 | 2.14e-34 |
| ACR33075.1 | Proline\_adenylation\_protein | BGC0000017 | Alkaloid+Polyketide:Modular type I polyketide | 25.0 | 42.3 | 140.0 | 3.07e-34 |
| UKO95761.1 | amino\_acid\_adenylation\_domain-containing\_protein | BGC0002632 | NRP | 25.0 | 43.1 | 140.0 | 3.19e-34 |
| AMK48225.1 | nonribosomal\_peptide\_synthetase | BGC0001351 | NRP | 27.0 | 46.6 | 143.0 | 3.89e-34 |
| WP\_069848004.1 | non-ribosomal\_peptide\_synthetase | BGC0002472 | NRP | 28.0 | 37.5 | 144.0 | 3.96e-34 |
| WP\_035121683.1 | non-ribosomal\_peptide\_synthetase | BGC0002624 | NRP+Polyketide | 28.0 | 37.7 | 143.0 | 4.75e-34 |
| EAL89046.1 | nonribosomal\_peptide\_synthetase | BGC0000355 | NRP | 29.0 | 52.6 | 143.0 | 5.12e-34 |
| EPE34341.1 | non-ribosomal\_peptide\_synthetase | BGC0001035 | Polyketide+NRP | 27.0 | 48.9 | 144.0 | 5.98e-34 |
| WP\_157358234.1 | SDR\_family\_NAD(P)-dependent\_oxidoreductase | BGC0002011 | Polyketide | 29.0 | 42.4 | 143.0 | 7.34e-34 |
| DAB41661.1 | nonribosomal\_peptide\_synthetase | BGC0001585 | Alkaloid | 26.0 | 50.4 | 142.0 | 9.99e-34 |
| UMP03490.1 | NmvB | BGC0002649 | NRP+Polyketide | 28.0 | 47.4 | 141.0 | 1.26e-33 |
| QIE08738.1 | non-ribosomal\_peptide\_synthetase | BGC0002544 | NRP | 28.0 | 46.5 | 141.0 | 1.48e-33 |
| QPP19367.1 | PenP | BGC0002501 | Alkaloid | 28.0 | 48.6 | 142.0 | 1.53e-33 |
| BAI23334.1 | putative\_non-ribosomal\_peptide\_synthetase | BGC0000949 | NRP | 26.0 | 49.8 | 141.0 | 1.85e-33 |
| AOZ21316.1 | SulI | BGC0001790 | NRP | 27.0 | 49.3 | 141.0 | 1.86e-33 |
| QKW60392.1 | amino\_acid\_adenylation\_domain-containing\_protein | BGC0002288 | NRP | 27.0 | 49.8 | 141.0 | 1.86e-33 |
| CAJ76292.1 | putative\_non-ribosomal\_peptide\_synthase | BGC0000972 | NRP+Polyketide:Modular type I polyketide+Polyketide:Trans-AT type I polyketide | 28.0 | 41.0 | 141.0 | 2.65e-33 |
| AFR69331.1 | nonribosomal\_peptide\_synthetase\_SpiA | BGC0001045 | NRP:Cyclic depsipeptide+Polyketide:Modular type I polyketide | 25.0 | 47.8 | 141.0 | 2.73e-33 |
| CBK62746.1 |  | BGC0001115 | NRP+Polyketide | 25.0 | 46.9 | 140.0 | 4.39e-33 |
| XP\_002379984.1 | nonribosomal\_peptide\_synthase,\_putative | BGC0001621 | NRP | 27.0 | 51.7 | 140.0 | 5.23e-33 |
| ANY94451.1 | A-domain\_type\_II\_peptide\_synthetase | BGC0001584 | Polyketide | 32.0 | 31.1 | 139.0 | 6.71e-33 |
| CBF87069.1 | nonribosomal\_peptide\_synthase,\_putative\_(Eurofung) | BGC0001290 | NRP | 26.0 | 48.3 | 140.0 | 6.8e-33 |
| CCP45171.1 | Phenyloxazoline\_synthase\_MbtB\_(phenyloxazoline\_synthetase) | BGC0001021 | NRP+Polyketide | 26.0 | 47.9 | 139.0 | 7.22e-33 |
| QGJ79675.1 | Polyketide\_synthase | BGC0002552 | Polyketide | 29.0 | 42.4 | 140.0 | 8.4e-33 |
| CAP93139.1 | cyclic\_hydrophobic\_tetrapeptide | BGC0000357 | NRP:Cyclic depsipeptide | 28.0 | 42.3 | 140.0 | 8.78e-33 |
| AXG46165.1 | non-ribosomal\_peptide\_synthetase | BGC0002713 | NRP | 29.0 | 36.4 | 139.0 | 8.88e-33 |
| EPH46593.1 | putative\_Bacitracin\_synthase\_3 | BGC0001519 | NRP+Polyketide | 28.0 | 41.7 | 135.0 | 9.28e-33 |
| CBF76036.1 | putative\_nonribosomal\_peptide\_synthetase\_(Eurofung) | BGC0001399 | NRP | 26.0 | 47.9 | 139.0 | 9.42e-33 |
| EAL92291.2 | nonribosomal\_peptide\_synthtease | BGC0000372 | NRP | 25.0 | 48.2 | 139.0 | 1.21e-32 |
| AAD24881.1 | putative\_acyl-CoA\_synthetase | BGC0000127 | Polyketide | 28.0 | 40.6 | 134.0 | 1.48e-32 |
| BAH23995.1 | nonribosomal\_peptide\_synthetase | BGC0000356 | NRP+Alkaloid | 27.0 | 54.5 | 139.0 | 1.59e-32 |
| WP\_069848010.1 | non-ribosomal\_peptide\_synthetase | BGC0002472 | NRP | 26.0 | 42.6 | 139.0 | 1.75e-32 |
| BAW27672.1 | NRPS(A-T-C) | BGC0001764 | NRP | 29.0 | 41.4 | 137.0 | 2.03e-32 |
| OJJ96433.1 | hypothetical\_protein | BGC0002226 | NRP | 25.0 | 47.4 | 138.0 | 2.82e-32 |
| AGN74898.1 | nonribosomal\_peptide\_synthetase | BGC0000459 | NRP:Cyclic depsipeptide+Polyketide:Trans-AT type I polyketide | 28.0 | 43.4 | 136.0 | 2.85e-32 |
| WP\_051700122.1 | non-ribosomal\_peptide\_synthetase | BGC0001368 | NRP | 27.0 | 52.2 | 137.0 | 2.89e-32 |
| AIT38302.1 | LstE | BGC0000382 | NRP | 30.0 | 36.0 | 137.0 | 3.04e-32 |
| WP\_030185025.1 | thioester\_reductase\_domain-containing\_protein | BGC0001813 | NRP | 30.0 | 32.7 | 137.0 | 3.04e-32 |
| AET79184.1 | lysergyl\_peptide\_synthetase\_subunit\_1 | BGC0001241 | Terpene | 27.0 | 48.3 | 137.0 | 4.11e-32 |
| CCE30225.1 | non-ribosomal\_peptide\_synthetase | BGC0002232 | Alkaloid | 27.0 | 48.3 | 137.0 | 4.11e-32 |
| AGC83576.1 | NRPS | BGC0000818 | NRP | 25.0 | 50.1 | 137.0 | 4.71e-32 |
| CRG85572.1 | nonribosomal\_peptide\_synthase,\_putative | BGC0001402 | NRP | 26.0 | 50.2 | 137.0 | 5.78e-32 |
| CAD15513.1 | non-ribosomal\_peptide\_synthetase | BGC0001014 | NRP:NRP siderophore+Polyketide:Modular type I polyketide+Polyketide:Iterative type I polyketide | 29.0 | 36.7 | 137.0 | 5.93e-32 |
| ABC34483.1 | nonribosomal\_peptide\_synthetase,\_putative | BGC0000961 | NRP+Polyketide | 26.0 | 47.7 | 136.0 | 6.91e-32 |
| ctg1\_orf00001 |  | BGC0000901 | Other | 27.0 | 48.9 | 136.0 | 9.01e-32 |
| AQX14493.1 | monobactam\_NRPS\_scaffold\_2 | BGC0001672 | NRP | 22.0 | 48.7 | 136.0 | 9.09e-32 |
| AAY42398.1 | Nonribosomal\_peptide\_synthetase | BGC0001000 | NRP:Lipopeptide+Polyketide:Modular type I polyketide | 26.0 | 38.3 | 136.0 | 9.42e-32 |
| QYA95659.1 | amino\_acid\_adenylation\_domain-containing\_protein | BGC0002676 | NRP | 27.0 | 46.5 | 135.0 | 1.23e-31 |
| AAF01762.1 | AM-toxin\_synthetase | BGC0001261 | NRP | 26.0 | 53.0 | 136.0 | 1.26e-31 |
| AAC82549.1 | FxbB | BGC0000351 | NRP | 28.0 | 45.2 | 135.0 | 1.46e-31 |
| XP\_020057667.1 | uncharacterized\_protein | BGC0001718 | NRP | 26.0 | 49.6 | 135.0 | 2.3e-31 |
| RLV71192.1 | non-ribosomal\_peptide\_synthetase | BGC0001846 | NRP+Saccharide:Hybrid/tailoring saccharide | 26.0 | 47.9 | 134.0 | 3.33e-31 |
| ARS01470.1 | NcmB | BGC0001702 | NRP+Polyketide | 30.0 | 36.5 | 134.0 | 3.42e-31 |
| QYA95657.1 | amino\_acid\_adenylation\_domain-containing\_protein | BGC0002676 | NRP | 29.0 | 41.8 | 130.0 | 3.53e-31 |
| CAP96445.1 | NRPS | BGC0000420 | NRP | 27.0 | 49.6 | 134.0 | 4.21e-31 |
| KMO93436.1 | NRPS/PKS | BGC0002095 | NRP | 29.0 | 41.7 | 129.0 | 5.96e-31 |
| WP\_141576286.1 | non-ribosomal\_peptide\_synthetase | BGC0002686 | NRP | 28.0 | 47.0 | 133.0 | 6.01e-31 |
| MBA0053730.1 | D-alanine--poly(phosphoribitol)\_ligase | BGC0002096 | Polyketide | 28.0 | 36.2 | 129.0 | 6.05e-31 |
| CBF87869.1 | nonribosomal\_peptide\_synthase,\_putative\_(Eurofung) | BGC0001699 | NRP | 26.0 | 50.1 | 134.0 | 6.64e-31 |
| ALU98443.1 | hypothetical\_protein | BGC0001397 | NRP+Polyketide | 29.0 | 43.6 | 132.0 | 7.16e-31 |
| AAL06681.1 | A-domain\_type\_II\_peptide\_synthetase | BGC0000965 | Polyketide:Iterative type I polyketide+Polyketide:Enediyne type I polyketide | 32.0 | 30.4 | 132.0 | 9.65e-31 |
| ABX71111.1 | Lct28 | BGC0000238 | Polyketide | 29.0 | 41.8 | 129.0 | 1.04e-30 |
| ESU17760.1 | hypothetical\_protein | BGC0002172 | NRP | 25.0 | 49.1 | 133.0 | 1.2e-30 |
| QRD90553.1 | non-ribosomal\_peptide\_synthetase\_module | BGC0002157 | NRP+Alkaloid | 26.0 | 49.4 | 132.0 | 1.28e-30 |
| AGA37269.1 | NRPS | BGC0000819 | NRP+Alkaloid | 23.0 | 77.0 | 132.0 | 1.65e-30 |
| UPA71912.1 | tyrocidine\_synthase\_3 | BGC0002636 | Polyketide | 28.0 | 42.8 | 128.0 | 1.88e-30 |
| ABV97151.1 | AMP-dependent\_synthetase\_and\_ligase | BGC0000137 | Polyketide | 27.0 | 52.1 | 132.0 | 2.52e-30 |
| ctg1\_orf4 |  | BGC0001329 | Polyketide+NRP:Cyclic depsipeptide | 32.0 | 23.8 | 131.0 | 2.63e-30 |
| MBD2892727.1 | Phenyloxazoline\_synthase\_MbtB | BGC0002718 | NRP | 27.0 | 46.3 | 130.0 | 3.11e-30 |
| WP\_024483797.1 | non-ribosomal\_peptide\_synthetase | BGC0002002 | NRP | 26.0 | 48.9 | 128.0 | 4.16e-30 |
| AKU20507.1 | polyketide\_synthase | BGC0002687 | Polyketide+NRP | 27.0 | 37.1 | 131.0 | 4.21e-30 |
| MAA\_10043 | non-ribosomal\_peptide\_synthetase | BGC0000337 | NRP | 26.0 | 48.7 | 131.0 | 4.51e-30 |
| AMQ36132.1 | PsyA | BGC0002617 | NRP | 26.0 | 56.4 | 130.0 | 8.06e-30 |
| WP\_063738219.1 | amino\_acid\_adenylation\_domain-containing\_protein | BGC0002010 | NRP+Polyketide | 27.0 | 42.7 | 126.0 | 8.77e-30 |
| QMW30133.1 | hypothetical\_protein | BGC0002248 | Terpene+NRP | 26.0 | 47.0 | 129.0 | 8.79e-30 |
| AAS98783.1 | polyketide\_synthase/nonribosomal\_peptide\_synthase\_hybrid | BGC0001001 | NRP+Polyketide | 31.0 | 23.3 | 129.0 | 1.6e-29 |
| AOE23579.1 | FoxBIII | BGC0001598 | NRP+Polyketide | 32.0 | 23.6 | 128.0 | 1.7e-29 |
| AEA29643.1 | putative\_nonribosomal\_peptide\_synthetase | BGC0000409 | NRP | 26.0 | 41.9 | 126.0 | 2e-29 |
| BAL15726.1 | NRPS | BGC0000432 | NRP | 29.0 | 48.8 | 126.0 | 2.16e-29 |
| EAW16180.1 | nonribosomal\_peptide\_synthase,\_putative | BGC0000293 | NRP | 27.0 | 47.5 | 128.0 | 2.45e-29 |
| AGK15447.1 | Non-ribosomal\_peptide\_synthetase,\_with\_condensation,\_AMP\_binding\_and\_thioesterase\_modules | BGC0002529 | NRP | 25.0 | 46.9 | 128.0 | 2.5e-29 |
| AFP87519.1 | proline\_adenyltransferase | BGC0001159 | NRP+Polyketide:Modular type I polyketide | 27.0 | 45.1 | 125.0 | 2.73e-29 |
| AAY42397.1 | Nonribosomal\_peptide\_synthetase | BGC0001000 | NRP:Lipopeptide+Polyketide:Modular type I polyketide | 26.0 | 39.2 | 128.0 | 3.51e-29 |
| ADY16697.1 | TqaA | BGC0001142 | NRP | 24.0 | 58.6 | 128.0 | 3.63e-29 |
| EGX96627.1 | non-ribosomal\_peptide\_synthase,\_putative | BGC0002259 | Polyketide+NRP | 27.0 | 43.1 | 128.0 | 3.72e-29 |
| AGC65515.1 | TtcC | BGC0001876 | NRP | 28.0 | 36.9 | 127.0 | 3.78e-29 |
| EXU96269.1 | nonribosomal\_peptide\_synthetase,\_serinocyclin\_synthetase\_NPS1 | BGC0001240 | NRP | 25.0 | 52.2 | 128.0 | 3.96e-29 |
| XP\_011325838.1 | hypothetical\_protein | BGC0001545 | NRP | 25.0 | 49.4 | 127.0 | 4.22e-29 |
| AQZ37113.1 | polyketide\_synthase | BGC0001511 | Polyketide | 28.0 | 42.4 | 127.0 | 4.84e-29 |
| AET79177.1 | lysergyl\_peptide\_synthetase\_subunit\_3 | BGC0001241 | Terpene | 26.0 | 49.1 | 127.0 | 4.97e-29 |
| CCE30237.1 | related\_to\_AM-toxin\_synthetase\_(AMT) | BGC0002232 | Alkaloid | 26.0 | 49.1 | 127.0 | 4.97e-29 |
| ATY46587.1 | polyketide\_synthase | BGC0001666 | Polyketide | 27.0 | 46.3 | 127.0 | 8.4e-29 |
| QIQ51365.1 | hypothetical\_protein | BGC0002199 | Alkaloid | 27.0 | 48.2 | 126.0 | 9.63e-29 |
| ABW71852.1 | nonribosomal\_peptide\_synthetase | BGC0000303 | NRP | 27.0 | 49.4 | 124.0 | 1.05e-28 |
| CAJ87590.1 | putative\_peptide\_synthase | BGC0001055 | NRP+Polyketide | 27.0 | 36.5 | 126.0 | 1.18e-28 |
| CCE28989.1 | non-ribosomal\_peptide\_synthetase | BGC0001365 | NRP | 27.0 | 47.5 | 126.0 | 1.18e-28 |
| QVK45120.1 | non-ribosomal\_peptide\_synthetase | BGC0002438 | Alkaloid | 27.0 | 48.5 | 126.0 | 1.19e-28 |
| EAU29302.1 | hypothetical\_protein | BGC0002272 | NRP | 27.0 | 47.5 | 126.0 | 1.24e-28 |
| BAO66533.1 | nonribosomal\_peptide\_synthase | BGC0000042 | Polyketide | 27.0 | 42.7 | 122.0 | 1.32e-28 |
| ADM34138.1 | non-ribosomal\_peptide\_synthetase | BGC0001084 | NRP+Terpene+Alkaloid | 24.0 | 49.9 | 125.0 | 1.6e-28 |
| CCP20047.1 | divK\_protein | BGC0001119 | Polyketide:Modular type I polyketide | 28.0 | 43.2 | 125.0 | 1.9e-28 |
| EHK22005.1 | putative\_non-ribosomal\_peptide\_synthetase\_GliP | BGC0001609 | NRP | 25.0 | 49.0 | 125.0 | 2.03e-28 |
| AFL68053.1 | amino\_acid\_adenylation\_enzyme/thioester\_reductase\_family\_protein | BGC0001524 | NRP+Polyketide | 24.0 | 49.2 | 125.0 | 2.09e-28 |
| AAM54075.1 | polyketide\_synthase | BGC0000020 | Polyketide | 28.0 | 42.4 | 125.0 | 2.46e-28 |
| EHA53213.1 | D-alanine-poly(phosphoribitol)\_ligase\_subunit\_1 | BGC0002158 | NRP+Polyketide | 26.0 | 51.2 | 125.0 | 2.49e-28 |
| AAG05690.1 | AmbE | BGC0000287 | NRP | 27.0 | 36.6 | 124.0 | 4.65e-28 |
| EJP62835.1 | nonribosomal\_peptide\_synthase,\_putative | BGC0002203 | NRP+Polyketide+Other | 26.0 | 49.3 | 124.0 | 5.56e-28 |
| BAE56606.1 |  | BGC0001123 | NRP | 26.0 | 48.9 | 123.0 | 9.38e-28 |
| UHJ79952.1 | non-ribosomal\_peptide\_synthetase | BGC0002654 | NRP | 33.0 | 23.6 | 121.0 | 9.92e-28 |
| AQM58287.1 | non-ribosomal\_peptide\_synthase | BGC0001816 | NRP+Polyketide | 24.0 | 52.6 | 123.0 | 1.09e-27 |
| ctg1\_orf000000 |  | BGC0000901 | Other | 25.0 | 51.0 | 122.0 | 1.53e-27 |
| CCM44334.1 | Thioester\_reductase | BGC0001056 | NRP+Polyketide:Modular type I polyketide+Polyketide:PUFA synthase or related polyketide | 28.0 | 31.0 | 117.0 | 1.82e-27 |
| AXB34356.1 | non-ribosomal\_peptide\_synthetase | BGC0002415 | NRP | 25.0 | 42.6 | 122.0 | 1.95e-27 |
| ADM46356.1 | polyketide\_synthase | BGC0000106 | Polyketide | 28.0 | 42.6 | 122.0 | 2.86e-27 |
| AGZ20183.1 | non-ribosomal\_peptide\_synthetase | BGC0002618 | Terpene | 26.0 | 53.2 | 121.0 | 3.51e-27 |
| QIE07123.1 | OvmK1 | BGC0001719 | Polyketide | 28.0 | 43.2 | 120.0 | 3.76e-27 |
| AAC83656.1 | dihydroaeruginoic\_acid\_synthetase | BGC0000412 | NRP | 28.0 | 36.0 | 120.0 | 4.48e-27 |
| BAG84247.1 | putative\_L-prolyl-AMP\_ligase | BGC0000257 | Polyketide | 27.0 | 40.0 | 118.0 | 6.57e-27 |
| AUS29484.1 | non-ribosomal\_peptide\_synthetase | BGC0002605 | NRP+Polyketide | 26.0 | 41.6 | 120.0 | 6.67e-27 |
| ABA59548.1 | NRPS | BGC0000453 | NRP:Cyclic depsipeptide | 26.0 | 49.7 | 120.0 | 7.44e-27 |
| AGQ43600.1 | HC-toxin\_synthetase | BGC0001166 | NRP | 24.0 | 51.3 | 120.0 | 8.35e-27 |
| AHA12086.1 | amino\_acid\_adenyltransferase | BGC0001172 | NRP+Polyketide:Modular type I polyketide | 25.0 | 35.7 | 116.0 | 8.97e-27 |
| BAE60013.1 |  | BGC0001518 | Terpene | 27.0 | 44.0 | 119.0 | 1.27e-26 |
| ACO94489.1 | putative\_AMP-dependent\_acyl-CoA\_synthetase/ligase | BGC0000097 | Polyketide:Modular type I polyketide | 26.0 | 42.7 | 116.0 | 1.4e-26 |
| AAO56106.1 | yersiniabactin\_non-ribosomal\_peptide\_synthetase | BGC0002570 | NRP+Polyketide | 27.0 | 36.1 | 119.0 | 1.56e-26 |
| ABK36691.1 | dimodular\_nonribosomal\_peptide\_synthetase | BGC0001502 | NRP | 28.0 | 40.2 | 116.0 | 1.61e-26 |
| BAC76476.1 | multifunctional\_polyketide-peptide\_synthase\_LkcA | BGC0001100 | NRP+Polyketide | 25.0 | 55.3 | 119.0 | 1.85e-26 |
| WP\_003060229.1 | type\_I\_polyketide\_synthase | BGC0002009 | Polyketide | 27.0 | 50.6 | 119.0 | 1.86e-26 |
| CQR60497.1 | Polyketide\_synthase,\_type\_I,\_modules:\_loading,\_1,\_2\_and\_3 | BGC0001287 | Polyketide | 28.0 | 43.1 | 119.0 | 1.89e-26 |
| AMK48234.1 | salicylate-AMP\_ligase | BGC0001351 | NRP | 28.0 | 43.1 | 116.0 | 2.07e-26 |
| ORC16617.1 | hypothetical\_protein | BGC0001341 | NRP | 26.0 | 48.5 | 118.0 | 2.88e-26 |
| CBJ89771.1 | Non-ribosomal\_peptide\_synthase\_involved\_in\_Xenocoumacin\_synthesis | BGC0001054 | NRP+Polyketide:Modular type I polyketide | 25.0 | 39.3 | 118.0 | 2.88e-26 |
| CAJ76286.1 | putative\_non-ribosomal\_peptide\_synthetase | BGC0000972 | NRP+Polyketide:Modular type I polyketide+Polyketide:Trans-AT type I polyketide | 27.0 | 41.6 | 118.0 | 3.02e-26 |
| AUS29494.1 | non-ribosomal\_peptide\_synthetase | BGC0001030 | NRP+Polyketide | 26.0 | 48.6 | 118.0 | 3.39e-26 |
| CBJ82073.1 | hypothetical\_protein | BGC0001872 | Polyketide | 26.0 | 30.4 | 113.0 | 3.52e-26 |
| TXD00261.1 | AMP-binding\_protein | BGC0001877 | Polyketide | 29.0 | 41.2 | 118.0 | 3.67e-26 |
| ACI30655.1 | BEAS\_beauvericin\_nonribosomal\_cyclodepsipeptide\_synthetase | BGC0000313 | NRP | 24.0 | 50.7 | 118.0 | 3.92e-26 |
| ACO94461.1 | putative\_AMP-dependent\_acyl-CoA\_synthetase/ligase | BGC0000029 | Polyketide:Modular type I polyketide | 26.0 | 42.6 | 115.0 | 4.42e-26 |
| ALA09365.1 | AMP-dependent\_synthetase\_and\_ligase | BGC0001303 | Polyketide | 29.0 | 29.4 | 114.0 | 5.25e-26 |
| ONK09689.1 | Beta-ketoacyl-acyl-carrier-protein\_synthase\_I | BGC0001647 | Polyketide | 29.0 | 39.2 | 117.0 | 6.43e-26 |
| BAE98151.1 | putative\_AMP-binding\_ligase | BGC0000339 | NRP | 30.0 | 43.9 | 114.0 | 7.52e-26 |
| AET79179.1 | lysergyl\_peptide\_synthetase\_subunit\_2 | BGC0001241 | Terpene | 25.0 | 50.4 | 116.0 | 1.1e-25 |
| CCE30235.1 | related\_to\_non-ribosomal\_peptide\_synthetase | BGC0002232 | Alkaloid | 25.0 | 50.4 | 116.0 | 1.1e-25 |
| AQX36215.1 | siderophore\_synthetase | BGC0001527 | Other | 25.0 | 46.3 | 115.0 | 1.88e-25 |
| KIA75688.1 | nonribosomal\_peptide\_synthase | BGC0002242 | NRP | 24.0 | 59.0 | 115.0 | 2.05e-25 |
| BCK51633.1 | modular\_polyketide\_synthase | BGC0002520 | Polyketide | 28.0 | 43.2 | 115.0 | 2.12e-25 |
| AEH41789.1 | HrmK | BGC0000374 | NRP:Cyclic depsipeptide | 28.0 | 42.4 | 112.0 | 3.16e-25 |
| ACF35445.1 | mbcAI | BGC0000090 | Polyketide | 28.0 | 42.9 | 115.0 | 3.75e-25 |
| AXG47410.1 | non-ribosomal\_peptide\_synthetase | BGC0002715 | NRP+Polyketide | 25.0 | 36.2 | 114.0 | 5.25e-25 |
| CCA53799.1 | iron\_aquisition\_yersiniabactin\_synthesis\_enzyme | BGC0001801 | NRP | 27.0 | 40.6 | 113.0 | 8e-25 |
| ESU05146.1 | hypothetical\_protein | BGC0002178 | NRP | 25.0 | 48.2 | 112.0 | 9.31e-25 |
| SAI82901.1 | HrnJ;\_Putative\_AMP-dependent\_acyl-CoA\_synthetase/ligase;\_AMP-binding\_enzyme;\_Pfam00501 | BGC0002101 | Polyketide | 25.0 | 42.3 | 110.0 | 1.03e-24 |
| QKG86306.1 | non-ribosomal\_peptide\_synthetase | BGC0002254 | Polyketide | 24.0 | 50.4 | 113.0 | 1.22e-24 |
| DAC74137.1 | AMP-dependent\_synthetase | BGC0002019 | Terpene | 26.0 | 41.6 | 109.0 | 1.85e-24 |
| AET79183.1 | lysergyl\_peptide\_synthetase\_subunit\_1 | BGC0001241 | Terpene | 25.0 | 50.4 | 112.0 | 2.31e-24 |
| CCE30226.1 | non-ribosomal\_peptide\_synthetase | BGC0002232 | Alkaloid | 25.0 | 50.4 | 112.0 | 2.31e-24 |
| ATY69590.1 | adenylation\_protein | BGC0001823 | NRP+Polyketide | 28.0 | 41.2 | 109.0 | 2.33e-24 |
| ADZ45321.1 | amino\_acid\_adenylation\_and\_condensation\_domain-containing\_protein | BGC0001020 | NRP+Polyketide | 26.0 | 57.2 | 111.0 | 2.48e-24 |
| MBN3579112.1 | amino\_acid\_adenylation\_domain-containing\_protein | BGC0002613 | NRP+Polyketide | 25.0 | 36.4 | 112.0 | 2.66e-24 |
| AQM58288.1 | non-ribosomal\_peptide\_synthase | BGC0001816 | NRP+Polyketide | 25.0 | 50.7 | 111.0 | 3.38e-24 |
| ABP55217.1 | AMP-dependent\_synthetase\_and\_ligase | BGC0000142 | Polyketide | 28.0 | 29.4 | 109.0 | 3.57e-24 |
| KAF7597139.1 | hypothetical\_protein | BGC0002264 | NRP | 27.0 | 49.4 | 111.0 | 3.77e-24 |
| AZC86156.1 | peramine\_synthetase | BGC0002166 | NRP | 28.0 | 34.8 | 111.0 | 3.78e-24 |
| AUS29499.1 | non-ribosomal\_peptide\_synthetase | BGC0002607 | NRP+Polyketide | 24.0 | 50.6 | 111.0 | 4.45e-24 |
| WP\_015031692.1 | type\_I\_polyketide\_synthase | BGC0001819 | Polyketide | 26.0 | 47.5 | 111.0 | 4.7e-24 |
| XP\_001217690.1 | hypothetical\_protein | BGC0001517 | NRP | 25.0 | 47.9 | 111.0 | 4.74e-24 |
| CAI94682.1 | putative\_polyketide\_synthase | BGC0000141 | Polyketide | 29.0 | 42.0 | 111.0 | 5.45e-24 |
| UOH28374.1 | AceN | BGC0002149 | NRP+Terpene | 25.0 | 51.3 | 110.0 | 8.45e-24 |
| AHH25595.1 | PKS | BGC0000957 | NRP+Polyketide | 27.0 | 37.7 | 110.0 | 8.53e-24 |
| AAC01710.1 | RifA | BGC0000136 | Polyketide | 26.0 | 51.4 | 108.0 | 2.72e-23 |
| AAO06916.1 | GdmAI | BGC0000066 | Polyketide | 26.0 | 43.1 | 108.0 | 2.81e-23 |
| QTX15956.1 | nonribosomal\_peptide\_synthase | BGC0002598 | Polyketide | 24.0 | 50.6 | 108.0 | 2.96e-23 |
| AAY28225.1 | HbmAI | BGC0000074 | Polyketide | 26.0 | 43.1 | 108.0 | 3.68e-23 |
| ABB86408.1 | GelA | BGC0000067 | Polyketide | 26.0 | 43.1 | 107.0 | 6.32e-23 |
| UHY14129.1 | PKS\_I | BGC0002671 | Polyketide | 27.0 | 43.4 | 106.0 | 1.36e-22 |
| ANY57892.1 | PenN | BGC0001372 | Terpene | 23.0 | 51.2 | 105.0 | 2.18e-22 |
| AWH12671.1 | RmpA1 | BGC0001759 | Polyketide | 26.0 | 54.9 | 105.0 | 2.22e-22 |
| BAP34707.1 | AMP-dependent\_synthetase\_and\_ligase | BGC0000078 | Polyketide | 28.0 | 30.4 | 103.0 | 2.33e-22 |
| AAR12528.1 | non-ribosomal\_peptide\_synthetase | BGC0002468 | NRP | 24.0 | 48.6 | 104.0 | 3.32e-22 |
| QSV12656.1 | AvmN | BGC0002456 | Polyketide+NRP | 28.0 | 36.2 | 104.0 | 4.56e-22 |
| CEF75881.1 |  | BGC0001600 | Polyketide | 30.0 | 20.5 | 103.0 | 5.9e-22 |
| BAL15718.1 | NRPS | BGC0000432 | NRP | 28.0 | 41.6 | 102.0 | 6.03e-22 |
| ACR78148.1 | BSLS | BGC0000312 | NRP | 24.0 | 47.7 | 104.0 | 6.56e-22 |
| QES95474.1 | type\_I\_polyketide\_synthase | BGC0002453 | Polyketide | 27.0 | 44.3 | 104.0 | 7.02e-22 |
| QTT72113.1 | type\_I\_polyketide\_synthase | BGC0002350 | NRP+Polyketide+Saccharide | 27.0 | 54.9 | 103.0 | 8.33e-22 |
| ARB50207.1 | lysergyl\_peptide\_synthetase\_21 | BGC0001573 | Alkaloid | 25.0 | 53.1 | 103.0 | 1.15e-21 |
| AUS29489.1 | non-ribosomal\_peptide\_synthetase | BGC0002606 | NRP+Polyketide | 25.0 | 43.0 | 103.0 | 1.31e-21 |
| CAE17553.1 | acyl\_CoA\_ligase | BGC0000210 | Polyketide:Type II polyketide+Saccharide:Oligosaccharide | 27.0 | 37.7 | 100.0 | 1.66e-21 |
| EYT83445.1 | Triostin\_synthetase\_I | BGC0001213 | Polyketide | 26.0 | 43.3 | 100.0 | 1.77e-21 |
| QBF51769.1 | type\_I\_polyketide\_synthase | BGC0001856 | Polyketide:Modular type I polyketide | 28.0 | 42.6 | 102.0 | 1.83e-21 |
| AAF86393.1 | FkbB | BGC0000994 | NRP+Polyketide | 26.0 | 38.3 | 102.0 | 2.12e-21 |
| AMQ36134.1 | PsyC | BGC0002617 | NRP | 24.0 | 51.8 | 100.0 | 5.44e-21 |
| CCC55917.1 | putative\_acyl\_CoA\_ligase | BGC0000973 | NRP+Polyketide:Modular type I polyketide | 28.0 | 44.0 | 99.0 | 6.2e-21 |
| ACN64830.1 | PokM3 | BGC0001061 | Polyketide:Iterative type I polyketide+Polyketide:Type II polyketide+Saccharide:Hybrid/tailoring saccharide | 26.0 | 43.5 | 98.0 | 1.36e-20 |
| CAC17498.1 | putative\_AMP-binding\_ligase | BGC0000324 | NRP | 28.0 | 45.8 | 97.0 | 3.2e-20 |
| AKQ52531.1 | nonribosomal\_peptide\_synthetase | BGC0002533 | NRP+Polyketide | 26.0 | 32.5 | 99.0 | 3.4e-20 |
| AIS24844.1 | dst22 | BGC0001147 | NRP | 27.0 | 43.8 | 97.0 | 3.63e-20 |
| BAE06845.2 | peramine\_synthetase | BGC0002164 | NRP | 25.0 | 38.1 | 98.0 | 4.83e-20 |
| ARB50206.1 | lysergyl\_peptide\_synthetase\_2 | BGC0001573 | Alkaloid | 23.0 | 49.8 | 98.0 | 5.11e-20 |
| ABM91454.1 | lysergyl\_peptide\_synthetase\_LpsB | BGC0000348 | NRP | 24.0 | 49.4 | 97.0 | 6.73e-20 |
| QGA70083.1 | ATP-dependent\_ligase | BGC0002517 | Polyketide | 24.0 | 39.8 | 96.0 | 7.76e-20 |
| WP\_141576284.1 | AMP-binding\_protein | BGC0002686 | NRP | 27.0 | 41.6 | 94.0 | 2.61e-19 |
| KDQ70107.1 | Triostin\_synthetase\_I | BGC0001444 | Other:shikimate derived | 28.0 | 43.7 | 93.0 | 7.14e-19 |
| AAC68815.1 | FK506\_polyketide\_synthase | BGC0000353 | NRP | 26.0 | 37.5 | 94.0 | 1.04e-18 |
| CAQ52626.1 | type\_I\_polyketide\_synthase,\_loading\_module\_and\_modules\_1-3 | BGC0001066 | Polyketide:Modular type I polyketide | 26.0 | 43.4 | 93.0 | 1.77e-18 |
| AAQ59159.1 | 2,3-dihydroxybenzoate-AMP\_ligase | BGC0002679 | NRP | 24.0 | 42.7 | 91.0 | 2.64e-18 |
| AFV52187.1 | acyl-CoA\_synthetase | BGC0000081 | NRP+Polyketide:Iterative type I polyketide+Polyketide:Enediyne type I polyketide | 27.0 | 42.9 | 91.0 | 2.84e-18 |
| AEI70243.1 | 2,3-dihydroxybenzoate-AMP\_ligase | BGC0000401 | NRP | 25.0 | 42.4 | 91.0 | 3.63e-18 |
| QMS79067.1 | nonribosomal\_peptide\_synthetase\_12 | BGC0002198 | NRP | 22.0 | 49.8 | 92.0 | 4.68e-18 |
| CAA60460.1 | polyketide\_synthase | BGC0001040 | NRP+Polyketide | 25.0 | 37.1 | 92.0 | 5.29e-18 |
| AFV30247.1 | polyketide\_synthase | BGC0000075 | Polyketide | 26.0 | 42.3 | 91.0 | 6.75e-18 |
| NPC94428.1 | (2,3-dihydroxybenzoyl)adenylate\_synthase | BGC0002695 | NRP | 27.0 | 30.7 | 89.0 | 1.45e-17 |
| ACA34358.1 | acyl-CoA\_ligase | BGC0001152 | Polyketide+NRP:Lipopeptide | 27.0 | 29.3 | 87.0 | 3.15e-17 |
| MBD2892724.1 | 2,3-dihydroxybenzoate-AMP\_ligase | BGC0002718 | NRP | 26.0 | 37.4 | 87.0 | 3.31e-17 |
| ACZ65474.1 | palmitoyl-CoA\_synthetase | BGC0000140 | Polyketide | 28.0 | 38.8 | 87.0 | 5.34e-17 |
| AHH25585.1 | AMP-dependent\_synthetase\_and\_ligase | BGC0000957 | NRP+Polyketide | 25.0 | 39.3 | 87.0 | 1.13e-16 |
| AAM77987.1 | adenylate\_ligase | BGC0000112 | Polyketide:Iterative type I polyketide+Polyketide:Enediyne type I polyketide | 26.0 | 43.8 | 85.0 | 1.9e-16 |
| BAL15732.1 | NRPS | BGC0000432 | NRP | 27.0 | 45.9 | 84.0 | 5.81e-16 |
| AAQ84158.1 | PlmJK | BGC0000123 | Polyketide | 28.0 | 24.9 | 84.0 | 7.79e-16 |
| KZM73517.1 | hypothetical\_protein | BGC0000632 | Terpene+Saccharide | 26.0 | 37.3 | 83.0 | 9.7e-16 |
| BAH33406.1 | 2,3-dihydroxybenzoate-AMP\_ligase | BGC0000371 | NRP | 27.0 | 37.3 | 82.0 | 1.32e-15 |
| ACU36660.1 | AMP-dependent\_synthetase\_and\_ligase | BGC0000392 | NRP | 26.0 | 43.2 | 82.0 | 1.33e-15 |
| AAZ55904.1 | 2,3-dihydroxybenzoate-AMP\_ligase | BGC0000359 | NRP | 29.0 | 24.9 | 81.0 | 2.93e-15 |
| MAA\_10036 | nonribosomal\_peptide\_synthase\_GliP-like,\_putative | BGC0000337 | NRP | 25.0 | 22.8 | 82.0 | 3.89e-15 |
| ADC79614.1 | BafY | BGC0000028 | Polyketide:Modular type I polyketide | 27.0 | 42.9 | 81.0 | 4.65e-15 |
| AEM06018.2 | hypothetical\_protein | BGC0000966 | NRP+Polyketide | 26.0 | 43.7 | 79.0 | 1.17e-14 |
| AFD30957.1 | CrmE | BGC0000966 | NRP+Polyketide | 26.0 | 43.7 | 79.0 | 1.48e-14 |
| RGP42811.1 | 2,3-dihydroxybenzoate-AMP\_ligase | BGC0002696 | NRP | 26.0 | 37.3 | 79.0 | 1.6e-14 |
| WP\_051729283.1 | AMP-binding\_protein | BGC0002137 | Polyketide | 25.0 | 37.1 | 79.0 | 2.43e-14 |
| AGN74880.1 | 3-hydroxypicolinic\_acid:AMP\_ligase | BGC0000459 | NRP:Cyclic depsipeptide+Polyketide:Trans-AT type I polyketide | 26.0 | 42.0 | 78.0 | 3.54e-14 |
| ASZ00147.1 | polyketide\_synthase | BGC0001785 | Polyketide | 28.0 | 26.6 | 79.0 | 4.92e-14 |
| ADI58632.1 | 5-Enolpyruvylshikimate-3-phosphate\_synthase/CHC-CoA\_ligase | BGC0000187 | Polyketide:Type II polyketide | 26.0 | 40.1 | 77.0 | 7.85e-14 |
| AAN65228.1 | amide\_synthetase | BGC0000832 | Saccharide:Hybrid/tailoring saccharide+Other:Aminocoumarin | 27.0 | 31.1 | 76.0 | 1.04e-13 |
| BBF25314.1 | NRPS-like\_oxidoreductase | BGC0001923 | Terpene+Polyketide | 25.0 | 34.3 | 77.0 | 1.42e-13 |
| AXG47413.1 | salicylate\_synthase | BGC0002715 | NRP+Polyketide | 24.0 | 42.0 | 74.0 | 6.88e-13 |
| AET13875.1 | epichloenin\_A\_synthetase | BGC0001250 | NRP | 26.0 | 30.9 | 75.0 | 7.19e-13 |
| AET13879.1 | epichloenin\_A\_synthetase | BGC0001251 | NRP | 26.0 | 30.9 | 75.0 | 7.19e-13 |
| CCB53266.1 | non-ribosomal\_peptide\_synthetase | BGC0001393 | NRP | 22.0 | 35.2 | 74.0 | 1.3e-12 |
| AGY30675.1 | Ann3 | BGC0001298 | Polyketide | 24.0 | 37.1 | 72.0 | 1.6e-12 |
| AAG29784.2 | amide\_synthetase | BGC0000833 | Saccharide:Hybrid/tailoring saccharide+Other:Aminocoumarin | 26.0 | 31.7 | 72.0 | 1.67e-12 |
| ABY66018.1 | CoA\_ligase | BGC0001008 | Polyketide:Iterative type I polyketide+Polyketide:Enediyne type I polyketide | 25.0 | 42.8 | 72.0 | 1.75e-12 |
| QEO75071.1 | AMP-dependent\_synthetase\_and\_ligase | BGC0002079 | NRP:Cyclic depsipeptide | 25.0 | 38.2 | 72.0 | 1.79e-12 |
| KIA75587.1 | NRPS-like\_enzyme | BGC0002209 | Polyketide | 23.0 | 70.5 | 72.0 | 3.71e-12 |
| APZ78718.1 | benzoate-CoA\_ligase\_family\_protein | BGC0001420 | NRP:Cyclic depsipeptide+Polyketide:Iterative type I polyketide | 26.0 | 24.4 | 71.0 | 3.71e-12 |
| AAF67505.1 | amide\_synthetase | BGC0000834 | Saccharide:Hybrid/tailoring saccharide+Other:Aminocoumarin | 25.0 | 36.3 | 71.0 | 3.82e-12 |
| CBF80711.1 | TdiA\_[Source:UniProtKB/TrEMBL;Acc:A7XRY0] | BGC0000442 | NRP | 24.0 | 36.9 | 71.0 | 7.84e-12 |
| CCA53797.1 | Long-chain-fatty-acid--CoA\_ligase | BGC0001801 | NRP | 25.0 | 43.6 | 69.0 | 2.68e-11 |
| EET76301.1 | putative\_(2,3-dihydroxybenzoyl)adenylate\_synthase | BGC0002685 | NRP | 23.0 | 30.4 | 69.0 | 2.86e-11 |
| AIE54254.1 | Pau34 | BGC0001732 | Other | 25.0 | 40.3 | 68.0 | 3.09e-11 |
| CAJ45636.1 | 2,3-dihydroxybenzoate-AMP\_ligase | BGC0000454 | NRP | 25.0 | 42.8 | 68.0 | 3.59e-11 |
| WP\_005009579.1 | AMP-binding\_protein | BGC0002473 | NRP | 22.0 | 42.6 | 66.0 | 1.46e-10 |
| EAU35432.1 | predicted\_protein | BGC0002734 | Polyketide | 25.0 | 32.5 | 66.0 | 2.14e-10 |
| AIE54201.1 | Pau34 | BGC0001731 | Other | 25.0 | 40.4 | 66.0 | 2.16e-10 |
| QIZ24104.1 | type\_I\_polyketide\_synthase | BGC0002540 | Polyketide | 24.0 | 53.8 | 66.0 | 4.15e-10 |
| OJF16272.1 | AceP1 | BGC0001491 | Polyketide | 24.0 | 42.0 | 64.0 | 9.2e-10 |
| CCA65703.1 | anthranilate-CoA\_ligase | BGC0001343 | Polyketide | 23.0 | 32.7 | 63.0 | 1.24e-09 |
| EDT06082.1 | AMP-dependent\_synthetase\_and\_ligase | BGC0001897 | Polyketide | 25.0 | 45.3 | 62.0 | 3.2e-09 |
| ADX66470.1 | ScnS0 | BGC0000108 | Polyketide | 25.0 | 51.7 | 62.0 | 4.72e-09 |
| AGD80622.1 | AMP-dependent\_synthetase\_and\_ligase | BGC0000394 | NRP | 27.0 | 34.3 | 61.0 | 5.9e-09 |
| AQT01384.1 | SgnS0 | BGC0001690 | Polyketide | 24.0 | 51.0 | 61.0 | 1.39e-08 |
| AAG04385.1 | probable\_coenzyme\_A\_ligase | BGC0000922 | Other | 24.0 | 37.6 | 59.0 | 3.39e-08 |
| PLB46276.1 | acetyl-CoA\_synthetase-like\_protein | BGC0001712 | Other | 23.0 | 34.9 | 58.0 | 8.05e-08 |
| KFA69336.1 | hypothetical\_protein | BGC0001626 | Polyketide | 24.0 | 28.0 | 57.0 | 1.43e-07 |
| CAD62194.1 | Ata18\_protein | BGC0000873 | Other | 25.0 | 41.2 | 54.0 | 1.16e-06 |
| EAU36089.1 | predicted\_protein | BGC0002273 | NRP | 26.0 | 24.7 | 53.0 | 2.73e-06 |
| BAV19380.1 | NRPS-like\_enzyme | BGC0001390 | NRP+Polyketide | 22.0 | 34.2 | 52.0 | 6.34e-06 |
