# Supplementary material for: Alkaliphilic/Alkali-Tolerant Fungi: Molecular, Biochemical, and Biotechnological Aspects
Source: J Fungi (Basel). 2023 Jun 9;9(6):652. doi: 10.3390/jof9060652 (PMC10301932; doi:10.3390/jof9060652)
Supplement: Supplementary file 1 [file jof-09-00652-s001.zip › S2/knownclusterblast/region1/input.path1.gene37_mibig_hits.html]

| MIBiG Protein | Description | MIBiG Cluster | MiBiG Product | % ID | % Coverage | BLAST Score | E-value |
| --- | --- | --- | --- | --- | --- | --- | --- |
| OAG05543.1 | cytochrome\_P450 | BGC0002211 | Polyketide | 56.0 | 95.0 | 595.0 | 3.52e-211 |
| GAP90688.2 | putative\_cytochrome\_P450 | BGC0002651 | Terpene | 41.0 | 93.0 | 383.0 | 5.37e-128 |
| AIA58898.1 | putative\_cytochrome\_P450 | BGC0001141 | Polyketide:Iterative type I polyketide | 43.0 | 94.6 | 363.0 | 3.38e-120 |
| BAV32149.1 | cytochrome\_P450\_monooxygenase | BGC0001373 | Polyketide | 41.0 | 94.6 | 361.0 | 3.01e-119 |
| AIA58895.1 | putative\_cytochrome\_P450 | BGC0001141 | Polyketide:Iterative type I polyketide | 40.0 | 94.2 | 345.0 | 3.72e-113 |
| AIA58896.1 | putative\_cytochrome\_P450 | BGC0001141 | Polyketide:Iterative type I polyketide | 40.0 | 94.0 | 337.0 | 4.46e-110 |
| EMD93706.1 | hypothetical\_protein | BGC0002245 | Terpene | 38.0 | 96.5 | 330.0 | 5.09e-107 |
| APY21860.1 | FmsC | BGC0001659 | Terpene | 38.0 | 95.4 | 315.0 | 2.18e-101 |
| AUS29483.1 | cytochrome\_P450\_monooxygenase | BGC0002605 | NRP+Polyketide | 36.0 | 94.8 | 308.0 | 1.25e-98 |
| AUS29488.1 | cytochrome\_P450\_monooxygenase | BGC0002606 | NRP+Polyketide | 35.0 | 94.8 | 305.0 | 1.13e-97 |
| AUS29498.1 | cytochrome\_P450\_monooxygenase | BGC0002607 | NRP+Polyketide | 35.0 | 95.6 | 301.0 | 5.64e-96 |
| AUS29493.1 | cytochrome\_P450\_monooxygenase | BGC0001030 | NRP+Polyketide | 35.0 | 95.9 | 298.0 | 8.73e-95 |
| QGW49100.1 | putative\_cytochrome\_P450 | BGC0002731 | Polyketide | 37.0 | 94.6 | 294.0 | 4.1e-94 |
| DAD54578.1 | putative\_tryptophan\_6-hydroxylase | BGC0002256 | NRP+Other | 36.0 | 94.2 | 295.0 | 1.21e-93 |
| AZQ56743.1 | cytochrome\_P450 | BGC0001969 | Terpene | 35.0 | 97.7 | 292.0 | 9.49e-93 |
| CAP86416.1 |  | BGC0002320 | Terpene | 36.0 | 93.2 | 283.0 | 2.04e-89 |
| AAK33073.1 | cytochrome\_P450 | BGC0001278 | Terpene | 34.0 | 91.7 | 284.0 | 2.27e-89 |
| OSS48490.1 | hypothetical\_protein | BGC0002194 | Polyketide | 35.0 | 98.6 | 275.0 | 1e-85 |
| BAX01960.1 | trichodiene\_oxygenase | BGC0001811 | Terpene | 34.0 | 95.2 | 273.0 | 2.26e-85 |
| XP\_001213595.1 | hypothetical\_protein | BGC0001475 | Terpene | 36.0 | 89.6 | 273.0 | 3.61e-85 |
| BAT32890.1 | stellatic\_acid\_synthase | BGC0002610 | Terpene | 35.0 | 103.5 | 273.0 | 4.71e-85 |
| QPP19371.1 | Pen10 | BGC0002501 | Alkaloid | 33.0 | 94.6 | 268.0 | 1.34e-83 |
| AAK33083.1 | putative\_cytochrome\_P450 | BGC0001277 | Terpene | 34.0 | 95.6 | 268.0 | 2.65e-83 |
| CBF77085.1 | cytochrome\_P450,\_putative\_(Eurofung) | BGC0001679 | NRP | 34.0 | 94.0 | 266.0 | 8.37e-83 |
| ATZ56108.1 | Bcbot3 | BGC0000631 | Terpene | 35.0 | 94.4 | 264.0 | 2.57e-81 |
| QMW30128.1 | hypothetical\_protein | BGC0002248 | Terpene+NRP | 35.0 | 90.1 | 273.0 | 2.72e-81 |
| XP\_001826052.1 |  | BGC0001995 | Terpene | 34.0 | 101.2 | 262.0 | 4.44e-81 |
| AAK53577.1 | trichodiene\_oxygenase | BGC0000930 | Terpene | 33.0 | 95.2 | 261.0 | 1.68e-80 |
| AEO57488.1 | p450 | BGC0001449 | NRP+Alkaloid+Polyketide:Iterative type I polyketide | 34.0 | 96.9 | 259.0 | 9.78e-80 |
| PLB46285.1 | cytochrome\_P450 | BGC0001712 | Other | 36.0 | 96.3 | 257.0 | 4.87e-79 |
| ADM79460.1 | P450\_protein | BGC0001266 | Polyketide | 31.0 | 106.6 | 235.0 | 2.57e-70 |
| QBC75447.1 | MacH | BGC0002615 | Terpene | 34.0 | 92.1 | 233.0 | 4.87e-70 |
| ATZ56106.1 | Bcbot1 | BGC0000631 | Terpene | 30.0 | 93.8 | 233.0 | 6.81e-70 |
| QBK15048.1 | cytochrome\_P450\_ClaJ | BGC0002196 | Polyketide | 33.0 | 85.5 | 226.0 | 2.5e-68 |
| EHA22197.1 | hypothetical\_protein | BGC0000170 | Polyketide | 33.0 | 93.8 | 226.0 | 1.73e-67 |
| AAS89997.1 | CypA | BGC0000007 | Polyketide | 31.0 | 94.4 | 224.0 | 6.1e-67 |
| EAU36102.1 | predicted\_protein | BGC0002273 | NRP | 32.0 | 73.3 | 216.0 | 6.46e-65 |
| GAP90685.1 | putative\_benzoate\_4-monooxygenase\_cytochrome | BGC0002651 | Terpene | 31.0 | 95.2 | 219.0 | 1.18e-64 |
| AAS90045.1 | CypA | BGC0000009 | Polyketide | 28.0 | 98.8 | 213.0 | 2.04e-62 |
| BAV32152.1 | cytochrome\_P450\_monooxygenase | BGC0001373 | Polyketide | 30.0 | 100.8 | 205.0 | 2.72e-59 |
| OJJ99917.1 | hypothetical\_protein | BGC0002225 | Terpene | 30.0 | 90.1 | 204.0 | 2.75e-59 |
| QTX15957.1 | cytochrome\_P450\_monooxygenase | BGC0002598 | Polyketide | 33.0 | 68.9 | 198.0 | 4.56e-58 |
| EAU36094.1 | predicted\_protein | BGC0002273 | NRP | 32.0 | 79.5 | 194.0 | 1.77e-56 |
| KIA75687.1 | hypothetical\_protein | BGC0002242 | NRP | 29.0 | 93.0 | 187.0 | 1.35e-52 |
| AGA37280.1 | P450\_monooxygenase | BGC0000819 | NRP+Alkaloid | 28.0 | 92.6 | 180.0 | 4.48e-50 |
| ANY57880.1 | PenB | BGC0001372 | Terpene | 30.0 | 83.2 | 174.0 | 2.06e-48 |
| KAF7597145.1 | hypothetical\_protein | BGC0002264 | NRP | 28.0 | 90.3 | 169.0 | 2.61e-46 |
| CEO59279.1 | Putative\_Benzoate\_4-monooxygenase\_cytochrome\_P450 | BGC0002278 | Alkaloid+NRP | 28.0 | 90.1 | 163.0 | 5.31e-44 |
| BAJ09784.1 | P450 | BGC0000146 | Polyketide | 27.0 | 84.7 | 150.0 | 1.07e-39 |
| ARB50204.1 | clavine\_oxidase | BGC0001573 | Alkaloid | 25.0 | 88.8 | 149.0 | 5.67e-39 |
| EIN09534.1 | cytochrome\_P450 | BGC0002213 | Polyketide | 28.0 | 89.7 | 145.0 | 9.96e-38 |
| ACD39751.1 | cytochrome\_P450 | BGC0000076 | Polyketide | 27.0 | 94.4 | 145.0 | 1.68e-37 |
| ACD39760.1 | cytochrome\_P450 | BGC0000077 | Polyketide | 27.0 | 94.4 | 145.0 | 1.68e-37 |
| ABV57820.1 | cytochrome\_P450\_monooxygenase | BGC0001267 | Terpene | 27.0 | 89.4 | 142.0 | 1.7e-36 |
| AET79191.1 | elymoclavine\_monooxygenase | BGC0001241 | Terpene | 25.0 | 90.9 | 142.0 | 2.21e-36 |
| CCE30234.1 | related\_to\_trichodiene\_oxygenase\_cytochrome\_P450 | BGC0002232 | Alkaloid | 25.0 | 90.9 | 142.0 | 2.21e-36 |
| XP\_023093493.1 |  | BGC0001995 | Terpene | 28.0 | 88.4 | 137.0 | 8.56e-35 |
| BAD83682.1 | cytochrome\_P-450 | BGC0000012 | Polyketide | 27.0 | 89.7 | 131.0 | 9.87e-33 |
| ATZ56105.1 | Bcbot4 | BGC0000631 | Terpene | 28.0 | 89.4 | 130.0 | 4.01e-32 |
| APY21861.1 | FmsD | BGC0001659 | Terpene | 26.0 | 96.3 | 128.0 | 1.19e-31 |
| BAK26559.1 | cytochrome\_P450 | BGC0000977 | NRP+Polyketide | 27.0 | 88.8 | 127.0 | 2.12e-31 |
| AMQ36133.1 | PsyB | BGC0002617 | NRP | 27.0 | 88.2 | 124.0 | 2.3e-30 |
| BAQ25465.1 | cytochrome\_P450 | BGC0001264 | Polyketide | 26.0 | 89.4 | 124.0 | 3.68e-30 |
| BAV32150.1 | cytochrome\_P450\_monooxygenase | BGC0001373 | Polyketide | 24.0 | 89.2 | 124.0 | 4.98e-30 |
| ATZ52741.1 | Bcaba2 | BGC0001893 | Other | 24.0 | 94.6 | 124.0 | 5.24e-30 |
| EAT85331.1 | hypothetical\_protein | BGC0002165 | Polyketide | 26.0 | 90.7 | 123.0 | 6.28e-30 |
| GAP90687.1 | putative\_cytochrome\_P450 | BGC0002651 | Terpene | 24.0 | 82.8 | 120.0 | 4.12e-29 |
| PLB46284.1 | cytochrome\_P450 | BGC0001712 | Other | 25.0 | 96.9 | 119.0 | 2.81e-28 |
| AAS90014.1 | CypX | BGC0000007 | Polyketide | 26.0 | 88.8 | 118.0 | 4.11e-28 |
| BAE60010.1 |  | BGC0001518 | Terpene | 24.0 | 90.3 | 118.0 | 4.25e-28 |
| AAS90107.1 | CypX | BGC0000006 | Polyketide | 26.0 | 89.2 | 117.0 | 7.5e-28 |
| AAS90036.1 | CypX | BGC0000008 | Polyketide | 26.0 | 89.2 | 117.0 | 7.5e-28 |
| AAS90082.1 | CypX | BGC0000010 | Polyketide | 26.0 | 89.7 | 117.0 | 7.5e-28 |
| QKG86308.1 | NotG | BGC0002254 | Polyketide | 26.0 | 89.0 | 117.0 | 9.44e-28 |
| BAE71332.1 | cytochrome\_P450\_monooxygenase | BGC0000004 | Polyketide | 26.0 | 89.2 | 115.0 | 2.49e-27 |
| AJG44382.1 | MpaDE' | BGC0002619 | Polyketide | 27.0 | 93.4 | 117.0 | 2.98e-27 |
| EPS29067.1 | hypothetical\_protein | BGC0001724 | NRP+Polyketide | 26.0 | 93.8 | 115.0 | 3.48e-27 |
| CBF73446.1 | cytochrome\_P450,\_putative\_(Eurofung) | BGC0001515 | NRP | 25.0 | 91.7 | 115.0 | 4.87e-27 |
| ADM34140.1 | P450 | BGC0001084 | NRP+Terpene+Alkaloid | 28.0 | 87.2 | 113.0 | 1.88e-26 |
| AAS90062.1 | CypX | BGC0000009 | Polyketide | 25.0 | 89.2 | 112.0 | 4.91e-26 |
| BBQ09591.1 | P450\_monooxygenase | BGC0002261 | Polyketide | 26.0 | 89.4 | 111.0 | 8.95e-26 |
| PKY07888.1 | cytochrome\_P450 | BGC0001544 | NRP+Polyketide | 25.0 | 94.4 | 111.0 | 9.82e-26 |
| AAK33070.1 | trichothecene\_C-15\_hydroxylase | BGC0001278 | Terpene | 25.0 | 91.1 | 110.0 | 1.93e-25 |
| MAA\_10044 | benzoate\_4-monooxygenase\_cytochrome\_P450 | BGC0000337 | NRP | 27.0 | 92.1 | 110.0 | 1.94e-25 |
| AAS90010.1 | AvnA | BGC0000007 | Polyketide | 25.0 | 92.1 | 109.0 | 3.59e-25 |
| AGC83578.1 | P450\_monooxygenase | BGC0000818 | NRP | 26.0 | 87.8 | 109.0 | 3.6e-25 |
| AAC49196.1 | putative\_p450\_monooxygenase | BGC0000152 | Polyketide | 25.0 | 95.0 | 107.0 | 1.69e-24 |
| EMD93703.1 | hypothetical\_protein | BGC0002245 | Terpene | 25.0 | 95.2 | 107.0 | 2.27e-24 |
| BAD29972.1 | P450\_monooxygenase\_1 | BGC0000676 | Terpene | 25.0 | 89.2 | 105.0 | 6.45e-24 |
| KIA75457.1 | CND5p | BGC0002208 | NRP | 25.0 | 79.5 | 105.0 | 1.02e-23 |
| BAI44339.1 | P450 | BGC0000677 | Terpene | 27.0 | 72.9 | 104.0 | 1.28e-23 |
| ACH72911.1 | AflV | BGC0000011 | Polyketide | 27.0 | 90.5 | 103.0 | 2.01e-23 |
| OJJ98490.1 | hypothetical\_protein | BGC0002169 | Polyketide+NRP | 25.0 | 84.7 | 103.0 | 2.29e-23 |
| ACH72905.1 | AflG | BGC0000011 | Polyketide | 25.0 | 94.2 | 103.0 | 3.17e-23 |
| DAD54579.1 | FscF | BGC0002256 | NRP+Other | 24.0 | 91.3 | 103.0 | 4.98e-23 |
| AAK53580.1 | isotrichodermin\_C-15\_hydroxylase | BGC0000930 | Terpene | 24.0 | 83.0 | 102.0 | 5.26e-23 |
| BAH23996.1 | cytochrome\_P450 | BGC0000356 | NRP+Alkaloid | 26.0 | 84.9 | 103.0 | 5.49e-23 |
| BBE36500.1 | putative\_cytochrome\_P450 | BGC0001528 | Terpene | 25.0 | 94.2 | 102.0 | 5.85e-23 |
| QQO98482.1 | FrzC | BGC0002146 | NRP | 24.0 | 90.9 | 102.0 | 7.18e-23 |
| KAF7597161.1 | hypothetical\_protein | BGC0002646 | Polyketide | 27.0 | 81.2 | 101.0 | 8.9e-23 |
| BAX01965.1 | isotrichodermin\_C-15\_hydroxylase | BGC0001811 | Terpene | 24.0 | 83.0 | 102.0 | 9.45e-23 |
| AAS90058.1 | AvnA | BGC0000009 | Polyketide | 25.0 | 93.0 | 101.0 | 1.29e-22 |
| BAO10618.1 | cytochrome\_P450\_monooxygenase | BGC0001262 | Polyketide | 24.0 | 89.6 | 101.0 | 2e-22 |
| EAU32824.1 | predicted\_protein | BGC0000160 | Polyketide | 40.0 | 21.5 | 92.0 | 5.06e-22 |
| QPP19376.1 | Pen14 | BGC0002501 | Alkaloid | 23.0 | 88.2 | 100.0 | 6.05e-22 |
| BBE36501.1 | putative\_cytochrome\_P450 | BGC0001528 | Terpene | 25.0 | 92.6 | 100.0 | 6.55e-22 |
| AAK33080.1 | putative\_trichothecene\_C-15\_hydroxylase | BGC0001277 | Terpene | 24.0 | 83.0 | 99.0 | 7.31e-22 |
| OJJ99918.1 | hypothetical\_protein | BGC0002225 | Terpene | 23.0 | 92.3 | 97.0 | 5e-21 |
| BAV32164.1 | cytochrome\_P450\_monooxygenase | BGC0001373 | Polyketide | 24.0 | 96.1 | 96.0 | 9.85e-21 |
| ATQ39434.1 | cytochrome\_P450 | BGC0001565 | NRP | 24.0 | 89.6 | 94.0 | 3.59e-20 |
| ACZ66253.1 | APS7 | BGC0000304 | NRP | 22.0 | 102.1 | 94.0 | 3.77e-20 |
| WP\_003598550.1 | cytochrome\_P450 | BGC0001991 | Polyketide | 26.0 | 86.5 | 88.0 | 5.91e-18 |
| KJA16715.1 | hypothetical\_protein | BGC0002246 | Terpene | 24.0 | 92.8 | 81.0 | 6.4e-16 |
| CCD48293.1 | similar\_to\_cytochrome\_P450\_monooxygenase | BGC0002217 | Terpene | 22.0 | 92.8 | 81.0 | 1.13e-15 |
| CAO98851.1 | cytochrome\_P450\_monooxygenase\_AufH | BGC0000023 | Polyketide:Modular type I polyketide | 29.0 | 49.3 | 79.0 | 2.81e-15 |
| EAT86859.2 | hypothetical\_protein | BGC0001858 | Polyketide | 29.0 | 31.7 | 79.0 | 3.28e-15 |
| TXD00003.1 | cytochrome\_P450 | BGC0001877 | Polyketide | 24.0 | 83.2 | 77.0 | 1.15e-14 |
| AAC49192.1 | putative\_p450\_monooxygenase | BGC0000152 | Polyketide | 24.0 | 81.6 | 74.0 | 1e-13 |
| EEF48745.1 | cytochrome\_P450,\_putative | BGC0002393 | Terpene | 29.0 | 43.7 | 74.0 | 1.01e-13 |
| WP\_062148882.1 | cytochrome\_P450 | BGC0002013 | RiPP | 31.0 | 38.7 | 74.0 | 1.13e-13 |
| ADI59538.1 | CorO | BGC0001091 | NRP+Polyketide | 33.0 | 36.8 | 73.0 | 1.87e-13 |
| QLM00038.1 | cytochrome\_P450 | BGC0002257 | Polyketide | 21.0 | 93.0 | 73.0 | 2.87e-13 |
| OJJ99915.1 | hypothetical\_protein | BGC0002225 | Terpene | 25.0 | 65.2 | 72.0 | 6.96e-13 |
| AAY42399.1 | Cytochrome\_P450\_monooxygenase | BGC0001000 | NRP:Lipopeptide+Polyketide:Modular type I polyketide | 24.0 | 83.8 | 71.0 | 1.02e-12 |
| CBF82292.1 | cytochrome\_P450,\_putative\_(Eurofung) | BGC0002180 | Polyketide | 30.0 | 30.6 | 71.0 | 1.72e-12 |
| CYP719A21 |  | BGC0001325 | Alkaloid | 31.0 | 36.2 | 69.0 | 4.69e-12 |
| XP\_037497855.1 | LOW\_QUALITY\_PROTEIN:\_premnaspirodiene\_oxygenase-like | BGC0002724 | Terpene | 29.0 | 35.6 | 69.0 | 6.83e-12 |
| QGY73452.1 | Itm20 | BGC0002451 | Polyketide | 29.0 | 33.7 | 64.0 | 1.73e-10 |
| AVY05511.1 | cytochrome\_P450\_monooxygenase | BGC0001571 | Terpene | 25.0 | 40.6 | 64.0 | 2.17e-10 |
| XP\_020057670.1 | uncharacterized\_protein | BGC0001718 | NRP | 30.0 | 31.3 | 62.0 | 7.97e-10 |
| QQO98481.1 | FrzL | BGC0002146 | NRP | 26.0 | 42.2 | 58.0 | 1.27e-08 |
| CAL69892.1 | RhiH\_protein | BGC0001112 | NRP+Polyketide:Trans-AT type I polyketide | 25.0 | 33.7 | 57.0 | 2.83e-08 |
| AAC01737.2 | putative\_cytochrome\_P450\_monooxygenase | BGC0000136 | Polyketide | 22.0 | 69.4 | 54.0 | 1.78e-07 |
| AEW95633.1 | cytochrome\_P450\_protein | BGC0002697 | NRP+Polyketide | 22.0 | 80.1 | 54.0 | 1.91e-07 |
| BBB04330.1 | cytochrome\_P450 | BGC0001717 | NRP | 26.0 | 30.9 | 54.0 | 2.12e-07 |
| ANY94454.1 | P450\_hydroxylase | BGC0001584 | Polyketide | 24.0 | 69.2 | 52.0 | 7.23e-07 |
| BBU37365.1 | P450\_monooxygenase | BGC0002525 | Polyketide | 24.0 | 77.0 | 52.0 | 1.17e-06 |
| UMP03496.1 | NmvG | BGC0002649 | NRP+Polyketide | 22.0 | 69.4 | 49.0 | 6.53e-06 |
