# Supplementary material for: Alkaliphilic/Alkali-Tolerant Fungi: Molecular, Biochemical, and Biotechnological Aspects
Source: J Fungi (Basel). 2023 Jun 9;9(6):652. doi: 10.3390/jof9060652 (PMC10301932; doi:10.3390/jof9060652)
Supplement: Supplementary file 1 [file jof-09-00652-s001.zip › S2/knownclusterblast/region1/input.path1.gene38_mibig_hits.html]

| MIBiG Protein | Description | MIBiG Cluster | MiBiG Product | % ID | % Coverage | BLAST Score | E-value |
| --- | --- | --- | --- | --- | --- | --- | --- |
| EAA36369.1 | hypothetical\_protein | BGC0002729 | Polyketide | 50.0 | 100.8 | 234.0 | 2.26e-77 |
| OAG05542.1 | aflatoxin\_biosynthesis\_ketoreductase\_nor-1 | BGC0002211 | Polyketide | 49.0 | 100.0 | 216.0 | 2.5e-70 |
| EHA52504.1 | aflatoxin\_biosynthesis\_ketoreductase\_nor-1 | BGC0001749 | Polyketide | 44.0 | 100.0 | 202.0 | 1e-64 |
| AAS90000.1 | Nor-1 | BGC0000007 | Polyketide | 39.0 | 100.0 | 164.0 | 2.03e-49 |
| AAS90110.1 | Nor-1 | BGC0000006 | Polyketide | 38.0 | 100.0 | 162.0 | 8.07e-49 |
| AAS90070.1 | Nor-1 | BGC0000010 | Polyketide | 38.0 | 100.0 | 162.0 | 8.07e-49 |
| AAS90023.1 | Nor-1 | BGC0000008 | Polyketide | 38.0 | 100.0 | 162.0 | 1.14e-48 |
| BAE71315.1 | norsolorinic\_acid\_reductase | BGC0000004 | Polyketide | 38.0 | 100.0 | 160.0 | 4.52e-48 |
| ACZ66256.1 | APS10 | BGC0000304 | NRP | 37.0 | 100.4 | 157.0 | 2.83e-47 |
| AAS90048.1 | Nor-1 | BGC0000009 | Polyketide | 37.0 | 100.0 | 157.0 | 4.89e-47 |
| AAC49194.1 | putative\_ketoreductase | BGC0000152 | Polyketide | 35.0 | 94.0 | 139.0 | 7.62e-40 |
| ACH72908.1 | AflD | BGC0000011 | Polyketide | 34.0 | 96.8 | 132.0 | 3.86e-37 |
| ADI24951.1 | GsfK | BGC0000070 | Polyketide:Iterative type I polyketide | 36.0 | 103.6 | 130.0 | 1.58e-36 |
| ACA34721.1 | CtnE | BGC0000894 | Other | 28.0 | 81.9 | 59.0 | 4.33e-10 |
| ALI92649.1 | CitE\_dehydrogenase | BGC0001338 | Polyketide:Iterative type I polyketide | 28.0 | 81.9 | 58.0 | 1.2e-09 |
| ACP19359.1 | SaqN | BGC0000267 | Polyketide:Type II polyketide+Saccharide:Oligosaccharide | 29.0 | 91.9 | 52.0 | 1.62e-07 |
