# Supplementary material for: Alkaliphilic/Alkali-Tolerant Fungi: Molecular, Biochemical, and Biotechnological Aspects
Source: J Fungi (Basel). 2023 Jun 9;9(6):652. doi: 10.3390/jof9060652 (PMC10301932; doi:10.3390/jof9060652)
Supplement: Supplementary file 1 [file jof-09-00652-s001.zip › S2/knownclusterblast/region1/input.path1.gene39_mibig_hits.html]

| MIBiG Protein | Description | MIBiG Cluster | MiBiG Product | % ID | % Coverage | BLAST Score | E-value |
| --- | --- | --- | --- | --- | --- | --- | --- |
| CAB38583.1 | probable\_anthranilate\_phosphoribotransferase\_(trpD2) | BGC0000315 | NRP:Lipopeptide:Ca+-dependent lipopeptide | 36.0 | 81.3 | 138.0 | 5.06e-37 |
| ALV86861.1 | Tlo15 | BGC0001406 | NRP | 32.0 | 94.2 | 133.0 | 3.69e-35 |
| ALL53315.1 | anthranilate\_phosphoribosyltransferase | BGC0001903 | Other | 32.0 | 81.1 | 132.0 | 1.04e-34 |
| ATU31821.1 | anthranilate\_phosphoribosyltransferase | BGC0001814 | NRP | 30.0 | 96.1 | 124.0 | 2.25e-31 |
| PVD00184.1 | anthranilate\_phosphoribosyltransferase | BGC0002100 | NRP+Other | 34.0 | 76.5 | 121.0 | 1.25e-30 |
| QUJ09152.1 | Lon5 | BGC0002440 | NRP | 32.0 | 98.8 | 119.0 | 6.02e-30 |
| APZ79628.1 | FisI6 | BGC0001595 | Alkaloid | 34.0 | 75.7 | 119.0 | 7.5e-30 |
| APZ79572.1 | FimI6 | BGC0001594 | Alkaloid | 34.0 | 75.7 | 117.0 | 2.7e-29 |
| AHB62782.1 | anthranilate\_phosphoribosyltransferase\_(TrpD) | BGC0001126 | Terpene+Alkaloid | 34.0 | 75.7 | 117.0 | 3.71e-29 |
| APZ79605.1 | FilI6 | BGC0001501 | Alkaloid | 34.0 | 75.7 | 117.0 | 3.71e-29 |
| APB62268.1 | anthranilate\_phosphoribosyltransferase | BGC0001612 | Alkaloid | 34.0 | 75.7 | 117.0 | 3.71e-29 |
| AHI58830.1 | anthranilate\_phosphoribosyltransferase\_(TrpD) | BGC0000668 | Terpene+Alkaloid | 36.0 | 63.3 | 112.0 | 3.19e-27 |
