# Supplementary material for: Alkaliphilic/Alkali-Tolerant Fungi: Molecular, Biochemical, and Biotechnological Aspects
Source: J Fungi (Basel). 2023 Jun 9;9(6):652. doi: 10.3390/jof9060652 (PMC10301932; doi:10.3390/jof9060652)
Supplement: Supplementary file 1 [file jof-09-00652-s001.zip › S2/knownclusterblast/region1/input.path1.gene40_mibig_hits.html]

| MIBiG Protein | Description | MIBiG Cluster | MiBiG Product | % ID | % Coverage | BLAST Score | E-value |
| --- | --- | --- | --- | --- | --- | --- | --- |
| ADI58673.1 | hypothetical\_protein | BGC0000187 | Polyketide:Type II polyketide | 32.0 | 81.8 | 246.0 | 1.61e-72 |
| ADB23376.1 | glycerol-3-phosphate\_dehydrogenase | BGC0001062 | Polyketide | 30.0 | 80.5 | 207.0 | 1.68e-58 |
| QVV57701.1 | anaerobic\_glycerol-3-phosphate\_dehydrogenase\_subunit\_A | BGC0002338 | Polyketide | 25.0 | 76.5 | 129.0 | 2.43e-31 |
