# Supplementary material for: Alkaliphilic/Alkali-Tolerant Fungi: Molecular, Biochemical, and Biotechnological Aspects
Source: J Fungi (Basel). 2023 Jun 9;9(6):652. doi: 10.3390/jof9060652 (PMC10301932; doi:10.3390/jof9060652)
Supplement: Supplementary file 1 [file jof-09-00652-s001.zip › S2/knownclusterblast/region1/input.path1.gene42_mibig_hits.html]

| MIBiG Protein | Description | MIBiG Cluster | MiBiG Product | % ID | % Coverage | BLAST Score | E-value |
| --- | --- | --- | --- | --- | --- | --- | --- |
| BBA21054.1 | putative\_glycerol\_kinase | BGC0001740 | NRP+Polyketide | 48.0 | 97.8 | 449.0 | 5.2e-154 |
| ADI58674.1 | hypothetical\_protein | BGC0000187 | Polyketide:Type II polyketide | 33.0 | 100.8 | 221.0 | 1.3e-65 |
