# Supplementary material for: Alkaliphilic/Alkali-Tolerant Fungi: Molecular, Biochemical, and Biotechnological Aspects
Source: J Fungi (Basel). 2023 Jun 9;9(6):652. doi: 10.3390/jof9060652 (PMC10301932; doi:10.3390/jof9060652)
Supplement: Supplementary file 1 [file jof-09-00652-s001.zip › S2/knownclusterblast/region1/input.path1.gene43_mibig_hits.html]

| MIBiG Protein | Description | MIBiG Cluster | MiBiG Product | % ID | % Coverage | BLAST Score | E-value |
| --- | --- | --- | --- | --- | --- | --- | --- |
| ADC45555.1 | sugar\_transporter | BGC0000093 | Polyketide | 29.0 | 70.1 | 143.0 | 2.48e-36 |
| EED57519.1 | MFS\_glucose\_transporter,\_putative | BGC0001446 | Polyketide:Iterative type I polyketide | 27.0 | 76.0 | 135.0 | 1.94e-33 |
| XP\_023094066.1 |  | BGC0001996 | Other | 26.0 | 73.2 | 102.0 | 1.49e-22 |
| FAC38\_15 |  | BGC0002198 | NRP | 25.0 | 79.4 | 100.0 | 1.35e-21 |
| BAV32173.1 | putative\_MFS\_sugar\_transporter | BGC0001373 | Polyketide | 22.0 | 74.6 | 90.0 | 2.18e-18 |
