# Supplementary material for: Alkaliphilic/Alkali-Tolerant Fungi: Molecular, Biochemical, and Biotechnological Aspects
Source: J Fungi (Basel). 2023 Jun 9;9(6):652. doi: 10.3390/jof9060652 (PMC10301932; doi:10.3390/jof9060652)
Supplement: Supplementary file 1 [file jof-09-00652-s001.zip › S2/knownclusterblast/region1/input.path1.gene44_mibig_hits.html]

| MIBiG Protein | Description | MIBiG Cluster | MiBiG Product | % ID | % Coverage | BLAST Score | E-value |
| --- | --- | --- | --- | --- | --- | --- | --- |
| ABV99087.1 | Xaa-Pro\_aminopeptidase | BGC0001007 | Polyketide+NRP | 31.0 | 89.8 | 139.0 | 6.92e-36 |
| ABP55495.1 | Xaa-Pro\_aminopeptidase | BGC0001006 | NRP+Polyketide | 32.0 | 66.2 | 138.0 | 1.3e-35 |
| AUV64161.1 | aminopeptidase | BGC0002436 | Other | 27.0 | 101.7 | 136.0 | 5.93e-35 |
