# Supplementary material for: Alkaliphilic/Alkali-Tolerant Fungi: Molecular, Biochemical, and Biotechnological Aspects
Source: J Fungi (Basel). 2023 Jun 9;9(6):652. doi: 10.3390/jof9060652 (PMC10301932; doi:10.3390/jof9060652)
Supplement: Supplementary file 1 [file jof-09-00652-s001.zip › S2/knownclusterblast/region1/input.path1.gene46_mibig_hits.html]

| MIBiG Protein | Description | MIBiG Cluster | MiBiG Product | % ID | % Coverage | BLAST Score | E-value |
| --- | --- | --- | --- | --- | --- | --- | --- |
| EAU35432.1 | predicted\_protein | BGC0002734 | Polyketide | 37.0 | 102.9 | 623.0 | 9.53e-208 |
| ESK96610.1 | hypothetical\_protein | BGC0002212 | Polyketide | 38.0 | 103.3 | 580.0 | 6.72e-191 |
| KIA75587.1 | NRPS-like\_enzyme | BGC0002209 | Polyketide | 36.0 | 107.9 | 566.0 | 3.56e-185 |
| BBF25314.1 | NRPS-like\_oxidoreductase | BGC0001923 | Terpene+Polyketide | 35.0 | 103.3 | 542.0 | 2.53e-176 |
| BAV19380.1 | NRPS-like\_enzyme | BGC0001390 | NRP+Polyketide | 34.0 | 102.6 | 522.0 | 6.5e-169 |
| KFA69336.1 | hypothetical\_protein | BGC0001626 | Polyketide | 33.0 | 105.0 | 503.0 | 1.74e-161 |
| ALI92655.1 | CitS\_citrinin\_polyketide\_synthase | BGC0001338 | Polyketide:Iterative type I polyketide | 36.0 | 39.3 | 220.0 | 2.04e-58 |
| AWM95789.1 | non-reduciing\_polyketide\_synthase\_methylorcinaldehyde\_synthase | BGC0001827 | Polyketide | 34.0 | 40.9 | 218.0 | 1.15e-57 |
| OPB37950.1 | hypothetical\_protein | BGC0002206 | Polyketide | 34.0 | 40.1 | 212.0 | 1.08e-55 |
| QBK15044.1 | clavatol\_synthase\_ClaF | BGC0002196 | Polyketide | 34.0 | 39.6 | 210.0 | 3.28e-55 |
| AGN71604.1 | conidial\_yellow\_pigment\_biosynthesis\_polyketide\_synthase | BGC0000027 | Polyketide:Iterative type I polyketide | 32.0 | 41.9 | 209.0 | 1.04e-54 |
| EAU31923.1 | hypothetical\_protein | BGC0002267 | Polyketide | 33.0 | 41.5 | 203.0 | 7.23e-53 |
| ADH01663.1 | putative\_polyketide\_synthase\_PKS3 | BGC0000099 | Polyketide | 32.0 | 39.2 | 199.0 | 9.06e-52 |
| QCO93110.1 | polyketide\_synthase | BGC0001976 | Terpene | 32.0 | 41.0 | 190.0 | 1.01e-48 |
| AMJ52084.1 | lijE | BGC0002255 | Polyketide | 30.0 | 44.3 | 189.0 | 1.34e-48 |
| CBF83139.1 | polyketide\_synthase,\_putative\_(JCVI) | BGC0001722 | Polyketide | 31.0 | 42.0 | 187.0 | 4.95e-48 |
| EHA28237.1 | hypothetical\_protein | BGC0001143 | Polyketide | 30.0 | 44.8 | 187.0 | 5.35e-48 |
| EAA65602.1 | hypothetical\_protein | BGC0000022 | Polyketide | 33.0 | 39.1 | 181.0 | 8.34e-46 |
| CCE67070.1 | polyketide\_synthase | BGC0001242 | Polyketide | 28.0 | 52.1 | 179.0 | 1.77e-45 |
| AUW31047.1 | PKS-like\_protein | BGC0002483 | Polyketide | 31.0 | 35.7 | 157.0 | 4.06e-42 |
| KFH44362.1 | Conidial\_yellow\_pigment\_biosynthesis\_polyketide\_synthase-like\_protein | BGC0002190 | Polyketide | 31.0 | 41.5 | 159.0 | 5.84e-39 |
| QRK05501.1 | myxochelin\_non-ribosomal\_peptide\_synthetase\_MxcG | BGC0002324 | NRP+Polyketide | 24.0 | 98.1 | 118.0 | 1.83e-26 |
| AAG31130.1 | MxcG | BGC0001345 | NRP | 24.0 | 97.0 | 110.0 | 3.29e-24 |
| BAO84866.1 | putative\_non-ribosomal\_peptide\_synthetase | BGC0000414 | NRP | 23.0 | 94.1 | 95.0 | 3.09e-19 |
| AAC44129.1 | saframycin\_Mx1\_synthetase\_A | BGC0002706 | NRP | 23.0 | 89.6 | 92.0 | 2.39e-18 |
| BAZ95823.1 | PKS-NRPS\_hybrid\_cpaA | BGC0001563 | NRP+Polyketide | 25.0 | 50.6 | 81.0 | 8.5e-15 |
| ARP51711.1 | PKS-NRPS\_hybrid\_protein | BGC0001741 | NRP+Polyketide | 24.0 | 48.6 | 72.0 | 2.5e-12 |
| QBK15049.1 | PKS-NRPS\_hybrid\_TraA | BGC0002197 | Polyketide+NRP | 23.0 | 42.2 | 70.0 | 1.26e-11 |
| EDU47082.1 | lovastatin\_nonaketide\_synthase | BGC0002250 | Polyketide+NRP | 25.0 | 36.7 | 66.0 | 2.47e-10 |
| QXF14600.1 | PydA | BGC0002239 | Polyketide+NRP | 21.0 | 52.5 | 65.0 | 5.58e-10 |
| QJD55556.1 | acetyl-CoA\_ligase | BGC0001909 | Polyketide | 30.0 | 20.8 | 59.0 | 2.13e-08 |
| EAT85332.2 | hypothetical\_protein | BGC0002165 | Polyketide | 24.0 | 34.6 | 57.0 | 1.2e-07 |
| AAY28225.1 | HbmAI | BGC0000074 | Polyketide | 27.0 | 30.5 | 56.0 | 2.84e-07 |
| AAO06916.1 | GdmAI | BGC0000066 | Polyketide | 27.0 | 30.3 | 56.0 | 3.72e-07 |
| ABB86408.1 | GelA | BGC0000067 | Polyketide | 27.0 | 30.3 | 56.0 | 3.72e-07 |
| CCP45172.1 | Bifunctional\_enzyme\_MbtA:\_salicyl-AMP\_ligase\_(SAL-AMP\_ligase)\_+\_salicyl-S-ArCP\_synthetase | BGC0001021 | NRP+Polyketide | 28.0 | 30.0 | 53.0 | 1.62e-06 |
