# Supplementary material for: Alkaliphilic/Alkali-Tolerant Fungi: Molecular, Biochemical, and Biotechnological Aspects
Source: J Fungi (Basel). 2023 Jun 9;9(6):652. doi: 10.3390/jof9060652 (PMC10301932; doi:10.3390/jof9060652)
Supplement: Supplementary file 1 [file jof-09-00652-s001.zip › S2/knownclusterblast/region1/input.path1.gene47_mibig_hits.html]

| MIBiG Protein | Description | MIBiG Cluster | MiBiG Product | % ID | % Coverage | BLAST Score | E-value |
| --- | --- | --- | --- | --- | --- | --- | --- |
| CAL80830.1 | dehydrogenase-related\_protein | BGC0000997 | NRP+Polyketide | 43.0 | 95.9 | 191.0 | 2.34e-59 |
| KDN80075.1 | 3-ketoacyl-ACP\_reductase | BGC0001074 | Saccharide+Polyketide | 42.0 | 96.9 | 181.0 | 2.46e-55 |
| ALJ49935.1 | TtmK | BGC0001236 | Polyketide | 40.0 | 96.6 | 175.0 | 3.06e-53 |
| CAG44632.1 | SriL03.9 | BGC0000712 | Saccharide | 39.0 | 95.9 | 159.0 | 1.01e-46 |
| QTA30590.1 | short\_chain\_dehydrogenase | BGC0002143 | Polyketide | 39.0 | 95.9 | 156.0 | 9.94e-46 |
| AWH12904.1 | KR\_domain-containing\_protein | BGC0001784 | Polyketide | 35.0 | 100.0 | 137.0 | 3.31e-38 |
| ATZ45194.1 | Bcboa17 | BGC0001892 | Polyketide | 33.0 | 96.6 | 132.0 | 1.22e-36 |
| QGA70091.1 | putative\_oxidoreductase | BGC0002517 | Polyketide | 37.0 | 88.7 | 127.0 | 1.34e-34 |
| WP\_099049898.1 | SDR\_family\_NAD(P)-dependent\_oxidoreductase | BGC0002002 | NRP | 40.0 | 63.4 | 115.0 | 3.56e-30 |
| ABI75134.1 | short-chain\_alcohol\_dehydrogenase | BGC0000887 | Alkaloid | 35.0 | 84.6 | 109.0 | 2.89e-28 |
| ABI75108.1 | short-chain\_alcohol\_dehydrogenase | BGC0000188 | Alkaloid | 39.0 | 64.7 | 105.0 | 8.13e-27 |
| EDY47129.1 | clavaldehyde\_dehydrogenase | BGC0000845 | Other:Non-NRP beta-lactam | 39.0 | 69.2 | 100.0 | 8.31e-25 |
| KJA16714.1 | hypothetical\_protein | BGC0002246 | Terpene | 33.0 | 95.9 | 94.0 | 6.14e-22 |
| QQW45476.1 | short-chain\_dehydrogenase/reductase\_CalM' | BGC0002168 | Polyketide | 29.0 | 96.2 | 92.0 | 4.01e-21 |
| BBM05072.1 | putative\_oxidoreductase | BGC0002170 | Polyketide | 29.0 | 96.2 | 92.0 | 4.01e-21 |
| AMJ52082.1 | lijC | BGC0002255 | Polyketide | 37.0 | 64.4 | 90.0 | 1.06e-20 |
| ATZ56112.1 | Bcbot7 | BGC0000631 | Terpene | 31.0 | 102.7 | 89.0 | 3.32e-20 |
| AAG06718.1 | probable\_short\_chain\_dehydrogenase | BGC0002037 | NRP | 32.0 | 87.0 | 86.0 | 3.6e-19 |
| KAF7526513.1 | hypothetical\_protein | BGC0002244 | Polyketide | 33.0 | 81.5 | 86.0 | 5.05e-19 |
| AHZ61852.1 | short-chain\_dehydrogenase | BGC0000240 | Polyketide:Type II polyketide+Saccharide:Hybrid/tailoring saccharide | 33.0 | 70.5 | 81.0 | 9.26e-18 |
| ABP54641.1 | short-chain\_dehydrogenase/reductase\_SDR | BGC0000241 | Polyketide:Type II polyketide+Saccharide:Hybrid/tailoring saccharide | 33.0 | 70.5 | 82.0 | 9.37e-18 |
| AKT74300.1 | TxnC4 | BGC0002141 | Polyketide | 35.0 | 67.8 | 81.0 | 1.23e-17 |
| ABO15851.1 | oxidoreductase | BGC0000130 | Polyketide | 32.0 | 84.6 | 81.0 | 2.45e-17 |
| CAB15190.2 | 2,3-dihydro-2,3-dihydroxybenzoate\_dehydrogenase | BGC0000309 | NRP | 32.0 | 63.7 | 80.0 | 3.26e-17 |
| ABB69757.1 | PlaU | BGC0000654 | Terpene+Saccharide:Hybrid/tailoring saccharide | 31.0 | 64.0 | 79.0 | 6.56e-17 |
| EAA36368.1 | short-chain\_dehydrogenase/reductase | BGC0002729 | Polyketide | 34.0 | 65.8 | 79.0 | 9.07e-17 |
| KGA48900.1 | 2,3-dihydro-2,3-dihydroxybenzoate\_dehydrogenase | BGC0002413 | NRP | 31.0 | 60.6 | 78.0 | 1.9e-16 |
| APR73624.1 | dehydrogenase | BGC0001625 | Polyketide | 32.0 | 81.2 | 78.0 | 1.99e-16 |
| AAP11945.1 | Short\_chain\_dehydrogenase | BGC0000612 | RiPP:Thiopeptide | 29.0 | 65.4 | 78.0 | 2.17e-16 |
| WP\_010369414.1 | SDR\_family\_oxidoreductase | BGC0000314 | Polyketide+NRP:Cyclic depsipeptide+Other:Aminocoumarin | 31.0 | 64.0 | 77.0 | 2.32e-16 |
| ADE34504.1 | ssfK | BGC0000269 | Polyketide:Type II polyketide+Saccharide:Hybrid/tailoring saccharide | 32.0 | 66.4 | 77.0 | 3.34e-16 |
| AFN69430.1 | ElxO | BGC0000509 | RiPP:Lanthipeptide | 29.0 | 72.3 | 77.0 | 3.44e-16 |
| AAA65202.1 | daunorubicin-doxorubicin\_polyketide\_synthase | BGC0000218 | Polyketide | 32.0 | 85.6 | 76.0 | 9.32e-16 |
| AAL15583.1 | Sim5 | BGC0000270 | Polyketide | 32.0 | 68.5 | 75.0 | 2.76e-15 |
| AAK06787.1 | putative\_ketoreductase\_SimA6 | BGC0001072 | Saccharide+Polyketide:Modular type I polyketide+Polyketide:Type II polyketide+Other:Aminocoumarin | 32.0 | 68.5 | 75.0 | 2.76e-15 |
| OOH83075.1 | 3-oxoacyl-ACP\_reductase | BGC0001987 | Polyketide | 29.0 | 64.7 | 74.0 | 5.71e-15 |
| AAQ08926.1 | putative\_3-ketoacyl-ACP\_reductase | BGC0000224 | Polyketide:Type II polyketide | 34.0 | 63.0 | 74.0 | 6.05e-15 |
| QBK46640.1 | HrsK3 | BGC0001960 | Polyketide | 33.0 | 72.6 | 74.0 | 7.1e-15 |
| WP\_003598534.1 | SDR\_family\_oxidoreductase | BGC0001991 | Polyketide | 31.0 | 61.0 | 73.0 | 7.95e-15 |
| CAJ34364.1 | NAD\_or\_NADP\_oxidoreductase | BGC0000445 | NRP:Cyclic depsipeptide | 32.0 | 64.7 | 73.0 | 1.01e-14 |
| ABB52548.1 | 3-oxoacyl-(acyl-carrier-protein)-reductase | BGC0000047 | Polyketide | 26.0 | 74.3 | 73.0 | 1.12e-14 |
| EHA52498.1 | hypothetical\_protein | BGC0001749 | Polyketide | 29.0 | 89.4 | 73.0 | 1.27e-14 |
| ATJ00771.1 | ketoacyl\_reductase | BGC0001568 | Polyketide | 32.0 | 74.3 | 73.0 | 1.33e-14 |
| ARD70870.1 | Polyketide\_synthesis,\_ketoreductase | BGC0001693 | Polyketide | 33.0 | 71.6 | 73.0 | 1.33e-14 |
| BAI70380.1 | short\_chain\_dehydrogenase | BGC0000896 | Other | 31.0 | 71.6 | 73.0 | 1.43e-14 |
| BAF85845.1 | putative\_oxidoreductase | BGC0000109 | Polyketide | 30.0 | 64.4 | 72.0 | 1.66e-14 |
| ARD70866.1 | Short-chain\_dehydrogenase/reductase | BGC0001693 | Polyketide | 31.0 | 68.5 | 72.0 | 1.94e-14 |
| CAD18969.1 | putative\_oxidoreductase | BGC0000847 | Other:Non-NRP beta-lactam | 32.0 | 62.3 | 72.0 | 2.43e-14 |
| AAC18111.1 | ketoreductase | BGC0000225 | Polyketide | 33.0 | 73.6 | 72.0 | 2.8e-14 |
| EHK18435.1 | hypothetical\_protein | BGC0002233 | Polyketide | 32.0 | 64.7 | 72.0 | 3.33e-14 |
| ANC94959.1 | AlmK | BGC0001396 | Polyketide | 26.0 | 74.3 | 71.0 | 3.94e-14 |
| AGZ20196.1 | short\_chain\_dehydrogenase | BGC0002618 | Terpene | 32.0 | 66.1 | 71.0 | 5.22e-14 |
| BAL90266.1 | putative\_short-chain\_dehydrogenase | BGC0002021 | Polyketide | 30.0 | 84.6 | 71.0 | 5.25e-14 |
| BBC20651.1 | enoyl-(acyl\_carrier\_protein)\_reductase | BGC0001917 | Polyketide | 30.0 | 65.1 | 71.0 | 5.39e-14 |
| AQZ26590.1 | 2,3-dihydro-2,3-dihydroxybenzoate\_dehydrogenase | BGC0001437 | NRP | 30.0 | 62.7 | 71.0 | 5.97e-14 |
| AEW95637.1 | hypothetical\_protein | BGC0002697 | NRP+Polyketide | 36.0 | 63.4 | 71.0 | 1.16e-13 |
| AMX23331.1 | putative\_acyl\_carrier\_protein | BGC0001500 | Polyketide | 32.0 | 66.4 | 70.0 | 1.33e-13 |
| OWA25242.1 | ketoacyl\_reductase | BGC0001438 | Polyketide+Saccharide:Hybrid/tailoring saccharide | 33.0 | 71.2 | 69.0 | 2.18e-13 |
| KDN80050.1 | ketoacyl\_reductase | BGC0001074 | Saccharide+Polyketide | 32.0 | 75.0 | 69.0 | 2.21e-13 |
| CAG23976.1 | 3-oxoacyl-(acyl\_carrier\_protein)\_reductase | BGC0000176 | Polyketide | 29.0 | 63.7 | 69.0 | 2.48e-13 |
| CAH10174.1 | ChaL\_protein | BGC0000207 | Polyketide | 32.0 | 60.3 | 69.0 | 2.51e-13 |
| AJS09392.1 | Ketoacyl-(acyl-carrier-protein)\_reductase | BGC0001150 | Polyketide:Type II polyketide+Polyketide:Type III polyketide | 28.0 | 80.8 | 69.0 | 2.57e-13 |
| CAP12610.1 | dehydrogenase | BGC0000219 | Polyketide:Type II polyketide+Saccharide:Hybrid/tailoring saccharide | 31.0 | 64.4 | 69.0 | 2.98e-13 |
| AAO65349.1 | putative\_ketoreductase | BGC0000236 | Polyketide | 33.0 | 68.5 | 69.0 | 3.01e-13 |
| WP\_016640238.1 | 3-oxoacyl-ACP\_reductase\_FabG | BGC0002000 | Polyketide | 31.0 | 64.4 | 69.0 | 3.01e-13 |
| ADE22326.1 | dehydrogenase | BGC0000065 | Polyketide:Iterative type I polyketide | 28.0 | 74.7 | 69.0 | 3.25e-13 |
| AQW35064.1 | Polyketide\_C-9\_ketoreductase | BGC0001675 | Polyketide | 31.0 | 67.1 | 69.0 | 4.15e-13 |
| AHN91930.1 | short-chain\_dehydrogenase/reductase\_SDR | BGC0000340 | NRP | 29.0 | 61.3 | 68.0 | 4.51e-13 |
| ABL09970.1 | oxidoreductase | BGC0000197 | Polyketide:Type II polyketide+Saccharide:Hybrid/tailoring saccharide | 32.0 | 62.7 | 69.0 | 5.74e-13 |
| AEE65483.1 | short-chain\_dehydrogenase/reductase\_SDR | BGC0000223 | Polyketide:Type II polyketide | 31.0 | 70.2 | 68.0 | 6.01e-13 |
| AAZ55906.1 | 2,3-dihydro-2,3-dihydroxybenzoate\_dehydrogenase;\_RBL00455 | BGC0000359 | NRP | 30.0 | 64.4 | 68.0 | 7.54e-13 |
| AYU66237.1 | TjhC3 | BGC0002461 | Polyketide | 33.0 | 69.5 | 68.0 | 7.63e-13 |
| AVO00812.1 | May13 | BGC0001661 | Polyketide | 33.0 | 76.0 | 68.0 | 7.71e-13 |
| BAA84591.1 | reductase | BGC0000025 | Polyketide | 30.0 | 54.8 | 67.0 | 7.79e-13 |
| AAS79466.1 | putative\_post-PKS\_ketoreductase | BGC0000035 | Polyketide | 25.0 | 74.3 | 67.0 | 8.95e-13 |
| CAC44199.1 | ketoacyl\_reductase | BGC0000194 | Polyketide:Type II polyketide | 30.0 | 65.1 | 67.0 | 1.04e-12 |
| BAC79042.1 | keto\_reductase\_(KR) | BGC0000245 | Polyketide | 29.0 | 86.3 | 67.0 | 1.04e-12 |
| CAA09652.1 | polyketide\_ketoreductase | BGC0000227 | Polyketide:Type II polyketide | 32.0 | 69.2 | 67.0 | 1.17e-12 |
| CBA63665.1 | 2,3-dihydro-2,3-dihydroxybenzoate\_dehydrogenase | BGC0000368 | NRP | 28.0 | 64.4 | 67.0 | 1.21e-12 |
| AAO39097.1 | AdmC | BGC0000956 | NRP:Beta-lactam+Polyketide:Type II polyketide | 30.0 | 67.1 | 67.0 | 1.24e-12 |
| QVQ68802.1 | mmyTIII | BGC0002129 | Polyketide | 33.0 | 65.1 | 67.0 | 1.25e-12 |
| AAF73457.1 | putative\_aklaviketone\_reductase | BGC0000193 | Polyketide | 33.0 | 61.6 | 67.0 | 1.51e-12 |
| AHL24460.1 | short-chain\_dehydrogenase/reductase\_SDR | BGC0000806 | Saccharide | 32.0 | 64.7 | 67.0 | 1.74e-12 |
| QFS19040.1 | OxyM\_family\_protein | BGC0002506 | Polyketide | 32.0 | 61.0 | 66.0 | 2.46e-12 |
| AGO50613.1 | ketoreductase | BGC0000229 | Polyketide:Type II polyketide+Saccharide:Hybrid/tailoring saccharide | 31.0 | 70.5 | 66.0 | 2.62e-12 |
| ABP54645.1 | short-chain\_dehydrogenase/reductase\_SDR | BGC0000241 | Polyketide:Type II polyketide+Saccharide:Hybrid/tailoring saccharide | 32.0 | 70.2 | 66.0 | 2.62e-12 |
| OKI81335.1 | short-chain\_dehydrogenase | BGC0002478 | Polyketide | 29.0 | 75.0 | 66.0 | 2.64e-12 |
| WP\_051892705.1 | 7-ketoreductase | BGC0001851 | Polyketide:Type II polyketide+Saccharide:Hybrid/tailoring saccharide | 29.0 | 72.9 | 66.0 | 2.68e-12 |
| MUL41457.1 | SDR\_family\_oxidoreductase | BGC0002045 | Polyketide:Type II polyketide | 33.0 | 67.1 | 66.0 | 2.84e-12 |
| AAG23281.1 | probable\_keto\_acyl\_reductase | BGC0000148 | Polyketide | 30.0 | 71.6 | 66.0 | 3.35e-12 |
| AAK57528.1 | PgaD | BGC0000262 | Polyketide:Type II polyketide+Saccharide:Hybrid/tailoring saccharide | 31.0 | 70.5 | 66.0 | 3.57e-12 |
| WP\_051808697.1 | 7-ketoreductase | BGC0001852 | Polyketide:Type II polyketide+Saccharide:Hybrid/tailoring saccharide | 29.0 | 70.9 | 66.0 | 3.64e-12 |
| AAL24452.1 | RdmJ | BGC0000265 | Polyketide | 29.0 | 71.9 | 66.0 | 3.68e-12 |
| BAB69696.1 |  | BGC0001098 | NRP+Polyketide | 26.0 | 64.7 | 66.0 | 4.85e-12 |
| QFS19054.1 | ketoreductase | BGC0002506 | Polyketide | 33.0 | 64.4 | 66.0 | 5.05e-12 |
| WP\_008704360.1 | SDR\_family\_oxidoreductase | BGC0001575 | NRP | 27.0 | 80.8 | 65.0 | 5.93e-12 |
| AHD25940.1 | putative\_ketoreductase | BGC0000208 | Polyketide | 33.0 | 64.0 | 65.0 | 6.67e-12 |
| AXL88813.1 | ketoacyl\_reductase | BGC0001895 | Polyketide | 33.0 | 65.4 | 65.0 | 6.67e-12 |
| MCG7203804.1 | SDR\_family\_oxidoreductase | BGC0000248 | Polyketide | 31.0 | 63.4 | 65.0 | 7.55e-12 |
| ADG86325.1 | ketoreductase | BGC0000190 | Polyketide | 33.0 | 64.0 | 65.0 | 7.9e-12 |
| SCF06487.1 | Short-chain\_dehydrogenase | BGC0002435 | Other | 28.0 | 60.3 | 64.0 | 9.58e-12 |
| QED93096.1 | short-chain\_dehydrogenase/reductase\_SDR | BGC0002556 | Alkaloid | 33.0 | 59.6 | 64.0 | 1.03e-11 |
| UPN68084.1 | reductase | BGC0002672 | Polyketide | 31.0 | 70.9 | 64.0 | 1.21e-11 |
| MCG7203803.1 | 3-oxoacyl-ACP\_reductase | BGC0000248 | Polyketide | 29.0 | 70.2 | 64.0 | 1.22e-11 |
| ABS74183.1 | YxjF | BGC0001090 | Polyketide+NRP:Lipopeptide | 26.0 | 64.7 | 64.0 | 1.22e-11 |
| AFO85456.1 | reductase/oxidase | BGC0000391 | NRP | 29.0 | 64.4 | 64.0 | 1.48e-11 |
| ACR12253.1 | 2,3-dihydro-2,3-dihydroxybenzoate\_dehydrogenase | BGC0000451 | NRP | 27.0 | 65.1 | 64.0 | 1.56e-11 |
| ADB02846.1 | AzicD | BGC0000202 | Polyketide | 29.0 | 71.6 | 64.0 | 1.59e-11 |
| NHN68323.1 | SDR\_family\_oxidoreductase | BGC0002719 | NRP | 33.0 | 70.2 | 64.0 | 1.64e-11 |
| EDY42534.1 | monensin\_polyketide\_synthase\_ketoacyl\_reductase | BGC0000212 | Polyketide:Type II polyketide | 31.0 | 66.4 | 64.0 | 1.66e-11 |
| QOP59273.1 | keto-reductase | BGC0002504 | Polyketide | 32.0 | 70.5 | 64.0 | 1.66e-11 |
| AHL46733.1 | ketoreductase | BGC0001179 | Polyketide:Type II polyketide | 28.0 | 73.6 | 64.0 | 1.82e-11 |
| AHA81975.1 | Ketoreductase | BGC0000199 | Polyketide:Type II polyketide+Saccharide:Hybrid/tailoring saccharide | 29.0 | 69.2 | 64.0 | 2.12e-11 |
| AUV64165.1 | short-chain\_dehydrogenase | BGC0002436 | Other | 28.0 | 69.5 | 63.0 | 2.14e-11 |
| BAL90288.1 | putative\_short-chain\_dehydrogenase | BGC0002021 | Polyketide | 31.0 | 73.3 | 64.0 | 2.23e-11 |
| AHZ61856.1 | short-chain\_dehydrogenase/reductase | BGC0000240 | Polyketide:Type II polyketide+Saccharide:Hybrid/tailoring saccharide | 31.0 | 72.3 | 64.0 | 2.25e-11 |
| AAL15603.1 | SimJ1 | BGC0000270 | Polyketide | 29.0 | 66.8 | 64.0 | 2.51e-11 |
| AAK06807.1 | putative\_3-keto-acyl-reductase\_SimD2 | BGC0001072 | Saccharide+Polyketide:Modular type I polyketide+Polyketide:Type II polyketide+Other:Aminocoumarin | 29.0 | 66.8 | 64.0 | 2.51e-11 |
| WP\_018891734.1 | SDR\_family\_oxidoreductase | BGC0001558 | Polyketide | 31.0 | 60.3 | 63.0 | 2.53e-11 |
| ATJ00769.1 | C-7\_ketoreductase | BGC0001568 | Polyketide | 31.0 | 60.3 | 63.0 | 2.67e-11 |
| BAO98807.1 | putative\_ketoreductase | BGC0001002 | NRP+Polyketide | 32.0 | 40.4 | 63.0 | 2.85e-11 |
| ADE34491.1 | ssfU | BGC0000269 | Polyketide:Type II polyketide+Saccharide:Hybrid/tailoring saccharide | 32.0 | 64.7 | 63.0 | 3.05e-11 |
| PPQ57492.1 | ketoacyl\_reductase | BGC0002016 | Polyketide | 31.0 | 68.5 | 63.0 | 3.05e-11 |
| AEI98650.1 | CtcG | BGC0000209 | Polyketide | 32.0 | 70.5 | 63.0 | 3.08e-11 |
| QXJ26486.1 | mycofactocin-coupled\_SDR\_family\_oxidoreductase | BGC0002370 | NRP | 28.0 | 74.7 | 63.0 | 3.43e-11 |
| OKJ61997.1 | short-chain\_dehydrogenase | BGC0002147 | NRP | 26.0 | 84.6 | 63.0 | 3.63e-11 |
| WP\_030957358.1 | 9-ketoreductase | BGC0001852 | Polyketide:Type II polyketide+Saccharide:Hybrid/tailoring saccharide | 29.0 | 72.3 | 63.0 | 4.14e-11 |
| QTA30588.1 | SDR\_family\_NAD(P)-dependent\_oxidoreductase | BGC0002143 | Polyketide | 30.0 | 68.5 | 63.0 | 4.14e-11 |
| CAJ42322.1 | ketoreductase | BGC0000273 | Polyketide:Type II polyketide+Saccharide:Hybrid/tailoring saccharide | 29.0 | 62.3 | 63.0 | 4.65e-11 |
| AQW35069.1 | ketoreductase | BGC0001675 | Polyketide | 29.0 | 63.4 | 62.0 | 5.36e-11 |
| CAG14968.1 | ketoreductase | BGC0000253 | Polyketide:Type II polyketide | 31.0 | 70.9 | 62.0 | 5.57e-11 |
| ARK36158.1 | ketoacyl\_reductase | BGC0001723 | Polyketide | 31.0 | 70.9 | 62.0 | 5.57e-11 |
| QED90618.1 | ketoacyl\_reductase | BGC0002081 | Polyketide | 32.0 | 69.2 | 62.0 | 5.93e-11 |
| PAU45554.1 | beta-ketoacyl-ACP\_reductase | BGC0002138 | Polyketide | 30.0 | 65.8 | 62.0 | 7.06e-11 |
| ARO44671.1 | ketoreductase | BGC0001769 | Polyketide | 28.0 | 74.7 | 62.0 | 7.62e-11 |
| MBW8699686.1 | putative\_ketoacyl\_reductase | BGC0002140 | Polyketide | 28.0 | 74.7 | 62.0 | 7.62e-11 |
| OKI81342.1 | ketoacyl\_reductase | BGC0002478 | Polyketide | 32.0 | 65.1 | 62.0 | 7.62e-11 |
| ADE22303.1 | putative\_short-chain\_dehydrogenase/reductase\_SDR | BGC0000279 | Polyketide | 31.0 | 63.0 | 61.0 | 9.91e-11 |
| CAH10175.1 | ChaZ\_protein | BGC0000207 | Polyketide | 30.0 | 64.4 | 62.0 | 1.03e-10 |
| BBA97253.1 | putative\_ketoreductase | BGC0002383 | Polyketide | 31.0 | 68.5 | 62.0 | 1.03e-10 |
| BAW27600.1 | putative\_short-chain\_dehydrogenase | BGC0001547 | Terpene | 27.0 | 87.3 | 61.0 | 1.36e-10 |
| WP\_020275098.1 | SDR\_family\_NAD(P)-dependent\_oxidoreductase | BGC0002012 | Polyketide | 31.0 | 64.4 | 61.0 | 1.9e-10 |
| QDQ37882.1 | ketoreductase | BGC0001979 | Polyketide | 30.0 | 69.2 | 61.0 | 2.57e-10 |
| ANY57964.1 | MtcE\_3-ketoacyl-ACP\_reductase | BGC0001369 | Polyketide | 29.0 | 66.8 | 60.0 | 3.18e-10 |
| ABO15849.1 | oxidoreductase | BGC0000130 | Polyketide | 34.0 | 46.2 | 60.0 | 3.45e-10 |
| QDG00823.1 | polyketide\_ketoreductase | BGC0002028 | Polyketide | 32.0 | 64.7 | 60.0 | 3.45e-10 |
| CBH32080.1 | putative\_polyketide\_ketoreductase | BGC0000211 | Polyketide | 33.0 | 64.0 | 60.0 | 3.48e-10 |
| BAV17002.1 | putative\_ketoreductase | BGC0001384 | Polyketide | 28.0 | 74.7 | 60.0 | 3.48e-10 |
| CCH32748.1 | Ketoreductase | BGC0002070 | Polyketide | 29.0 | 70.9 | 60.0 | 4.63e-10 |
| ABL09955.1 | ketoreductase | BGC0000197 | Polyketide:Type II polyketide+Saccharide:Hybrid/tailoring saccharide | 29.0 | 70.5 | 60.0 | 4.71e-10 |
| ANY57970.1 | Short\_chain\_dehydrogenase | BGC0001369 | Polyketide | 28.0 | 65.8 | 59.0 | 5.62e-10 |
| QQZ01599.1 | short-chain\_dehydrogenase/reductase\_SDR | BGC0002498 | Other | 30.0 | 66.4 | 59.0 | 5.67e-10 |
| ACX83621.1 | keto\_reductase | BGC0000221 | Polyketide | 29.0 | 70.5 | 59.0 | 6.37e-10 |
| AAB36565.1 | ketoreductase | BGC0000234 | Polyketide | 28.0 | 68.5 | 59.0 | 6.53e-10 |
| CBH32818.1 | putative\_ketoreductase | BGC0000263 | Polyketide | 30.0 | 63.0 | 59.0 | 8.61e-10 |
| AJW65399.1 | oxidoreductase | BGC0001195 | NRP+Polyketide | 29.0 | 65.8 | 59.0 | 1.05e-09 |
| BCP96887.1 | short-chain\_dehydrogenase | BGC0002614 | NRP+Polyketide | 27.0 | 85.3 | 59.0 | 1.11e-09 |
| EHM27508.1 | short-chain\_dehydrogenase/reductase\_SDR | BGC0000235 | Polyketide | 31.0 | 68.5 | 59.0 | 1.12e-09 |
| AAP69586.1 | putative\_ketoreductase | BGC0000226 | Polyketide | 26.0 | 84.6 | 59.0 | 1.15e-09 |
| WP\_031147019.1 | 9-ketoreductase | BGC0001851 | Polyketide:Type II polyketide+Saccharide:Hybrid/tailoring saccharide | 30.0 | 69.5 | 59.0 | 1.16e-09 |
| WP\_037817190.1 | 3-oxoacyl-ACP\_reductase\_FabG | BGC0002137 | Polyketide | 31.0 | 70.5 | 59.0 | 1.16e-09 |
| OKI59859.1 | ketoacyl\_reductase | BGC0002477 | Polyketide | 31.0 | 68.5 | 59.0 | 1.16e-09 |
| QNL10616.1 | Ketoacyl\_reductase | BGC0002514 | Polyketide | 29.0 | 68.2 | 59.0 | 1.16e-09 |
| KDN80052.1 | ketoreductase | BGC0001074 | Saccharide+Polyketide | 30.0 | 60.3 | 58.0 | 1.34e-09 |
| SCN11974.1 | short-chain\_dehydrogenase/reductase\_SDR | BGC0001580 | Polyketide | 30.0 | 62.7 | 58.0 | 1.45e-09 |
| CAM34348.1 | putative\_short-chain\_dehydrogenase/reductase\_SDR | BGC0000242 | Polyketide | 31.0 | 65.1 | 58.0 | 1.55e-09 |
| ACP19356.1 | SaqD | BGC0000267 | Polyketide:Type II polyketide+Saccharide:Oligosaccharide | 30.0 | 63.0 | 58.0 | 2.01e-09 |
| CAH10114.1 | putative\_ketoreducatse | BGC0000268 | Polyketide | 29.0 | 69.2 | 57.0 | 2.99e-09 |
| CAM34370.1 | putative\_3-oxoacyl-ACP\_reductase | BGC0000242 | Polyketide | 32.0 | 64.7 | 57.0 | 3.4e-09 |
| AAF23366.1 | PhaB | BGC0000866 | Other | 27.0 | 65.1 | 57.0 | 3.43e-09 |
| CAK50783.1 | ketoreductase | BGC0000247 | Polyketide:Type II polyketide+Saccharide:Oligosaccharide | 30.0 | 59.6 | 57.0 | 3.46e-09 |
| POM23765.1 | putative\_ketoacyl\_reductase | BGC0002369 | Polyketide | 29.0 | 67.5 | 57.0 | 3.85e-09 |
| AEE65465.1 | ketoreductase | BGC0000223 | Polyketide:Type II polyketide | 27.0 | 75.0 | 57.0 | 5.2e-09 |
| TRO56980.1 | SDR\_family\_NAD(P)-dependent\_oxidoreductase | BGC0002361 | Polyketide+Saccharide | 30.0 | 69.5 | 57.0 | 5.44e-09 |
| AVW82957.1 | short\_chain\_dehydrognase | BGC0002040 | Other | 27.0 | 68.5 | 56.0 | 5.55e-09 |
| MQS11296.1 | SDR\_family\_oxidoreductase | BGC0002282 | Other | 27.0 | 68.5 | 56.0 | 5.55e-09 |
| QTK22480.1 | short-chain\_dehydrogenase | BGC0002462 | Saccharide | 27.0 | 68.5 | 56.0 | 5.55e-09 |
| ADG39439.1 | AllS | BGC0000886 | Other | 35.0 | 34.9 | 56.0 | 8.71e-09 |
| AHL46698.1 | ketoreductase | BGC0001177 | Polyketide:Type II polyketide | 29.0 | 71.2 | 56.0 | 9.54e-09 |
| SEG87456.1 | ketoreductase | BGC0002712 | Polyketide | 29.0 | 64.7 | 56.0 | 1.06e-08 |
| ACI88864.1 | AlnP\_ketoreductase | BGC0000195 | Polyketide:Type II polyketide | 28.0 | 69.5 | 56.0 | 1.31e-08 |
| QBA57739.1 | NAD(P)-dependent\_oxidoreductase | BGC0002377 | NRP | 27.0 | 70.2 | 56.0 | 1.41e-08 |
| AAD13539.1 | reductase\_homolog | BGC0000239 | Polyketide:Type II polyketide+Saccharide:Hybrid/tailoring saccharide | 28.0 | 71.2 | 54.0 | 3.15e-08 |
| WP\_040253449.1 | SDR\_family\_NAD(P)-dependent\_oxidoreductase | BGC0001596 | Polyketide | 28.0 | 64.0 | 54.0 | 3.22e-08 |
| AIE54248.1 | PauY28 | BGC0001732 | Other | 25.0 | 67.8 | 54.0 | 4.07e-08 |
| AFJ52673.1 | C-9\_ketoreductase | BGC0001073 | NRP+Polyketide | 30.0 | 63.7 | 54.0 | 4.25e-08 |
| QHZ32176.1 | putative\_ketoacyl\_reductase | BGC0002047 | Polyketide | 28.0 | 63.4 | 54.0 | 4.25e-08 |
| QTA30612.1 | sorbitol\_utilization\_protein\_SOU2 | BGC0002143 | Polyketide | 29.0 | 62.7 | 54.0 | 5.05e-08 |
| QTA30592.1 | 3-phenylpropionate-dihydrodiol/cinnamic\_acid-dihydrodiol\_dehydrogenase | BGC0002143 | Polyketide | 29.0 | 76.4 | 54.0 | 6.1e-08 |
| AHX24712.1 | 3-oxoacyl-ACP\_reductase | BGC0000200 | Polyketide:Type II polyketide+Saccharide:Hybrid/tailoring saccharide | 30.0 | 64.7 | 53.0 | 6.87e-08 |
| ACA34721.1 | CtnE | BGC0000894 | Other | 30.0 | 63.0 | 53.0 | 8.4e-08 |
| AAG30255.1 | NADPH-dependent\_acetoacetyl\_CoA\_reductase | BGC0000867 | Other | 28.0 | 44.2 | 52.0 | 8.86e-08 |
| ALI92649.1 | CitE\_dehydrogenase | BGC0001338 | Polyketide:Iterative type I polyketide | 30.0 | 63.0 | 53.0 | 1.23e-07 |
| AIE54195.1 | Pau28 | BGC0001731 | Other | 25.0 | 67.8 | 52.0 | 1.81e-07 |
| BAO98802.1 | putative\_reductase | BGC0001002 | NRP+Polyketide | 27.0 | 88.7 | 52.0 | 2.31e-07 |
| BAJ07854.1 | putative\_ketoreductase | BGC0000232 | Polyketide | 30.0 | 63.4 | 51.0 | 3.4e-07 |
| ADI71446.1 | putative\_ketoreductase | BGC0000203 | Polyketide | 27.0 | 68.2 | 51.0 | 4.57e-07 |
| QDA77039.1 | short-chain\_dehydrogenase/reductase\_SDR | BGC0002025 | NRP+Polyketide | 29.0 | 61.3 | 51.0 | 4.65e-07 |
| ADD83005.1 | PtnO7 | BGC0001156 | Terpene | 26.0 | 66.4 | 50.0 | 7.44e-07 |
| CAC37883.1 | CpkI;\_Nicotinamide-dependent\_dehydrogenase | BGC0000038 | Polyketide:Modular type I polyketide | 30.0 | 74.0 | 50.0 | 8.44e-07 |
| ACM68688.1 | AerF | BGC0000298 | NRP | 27.0 | 64.7 | 49.0 | 1.53e-06 |
| antaM | 3-oxoacyl-ACP\_reductase | BGC0001455 | NRP+Polyketide | 28.0 | 67.1 | 48.0 | 3.64e-06 |
| ABW96534.1 | putative\_dehydrogenase | BGC0000159 | Polyketide:Modular type I polyketide | 33.0 | 41.8 | 48.0 | 4.18e-06 |
