# Supplementary material for: Alkaliphilic/Alkali-Tolerant Fungi: Molecular, Biochemical, and Biotechnological Aspects
Source: J Fungi (Basel). 2023 Jun 9;9(6):652. doi: 10.3390/jof9060652 (PMC10301932; doi:10.3390/jof9060652)
Supplement: Supplementary file 1 [file jof-09-00652-s001.zip › S2/knownclusterblast/region1/input.path1.gene48_mibig_hits.html]

| MIBiG Protein | Description | MIBiG Cluster | MiBiG Product | % ID | % Coverage | BLAST Score | E-value |
| --- | --- | --- | --- | --- | --- | --- | --- |
| KIA75592.1 | hypothetical\_protein | BGC0002209 | Polyketide | 40.0 | 59.3 | 128.0 | 3.79e-33 |
| CDM31316.1 | FAD-binding,\_type\_2 | BGC0000667 | Terpene | 39.0 | 48.1 | 81.0 | 1.25e-16 |
| QMS79053.1 | bifunctional\_solanapyrone\_synthase | BGC0002198 | NRP | 52.0 | 22.8 | 75.0 | 1.09e-14 |
| OAG05541.1 | FAD-binding\_domain-containing\_protein | BGC0002211 | Polyketide | 55.0 | 22.8 | 72.0 | 1.5e-13 |
| XP\_659391.1 | hypothetical\_protein | BGC0001998 | Polyketide | 30.0 | 59.6 | 70.0 | 5.14e-13 |
| ARP51712.1 | FAD\_oxidoreductase | BGC0001741 | NRP+Polyketide | 39.0 | 38.5 | 70.0 | 6.49e-13 |
| QGW49096.1 | putative\_FAD-binding\_oxidoreductase | BGC0002731 | Polyketide | 33.0 | 56.4 | 66.0 | 9.24e-12 |
| BAE62224.1 |  | BGC0002237 | Polyketide | 44.0 | 22.4 | 64.0 | 3.9e-11 |
| ANY57959.1 | MtcA\_FAD-linked\_oxidase | BGC0001369 | Polyketide | 42.0 | 21.5 | 57.0 | 9.14e-09 |
| AAM97372.1 | RubI | BGC0000266 | Polyketide | 34.0 | 39.4 | 57.0 | 9.78e-09 |
| ABB90284.1 | isoamyl\_alcohol\_oxidase | BGC0001057 | NRP+Polyketide | 25.0 | 61.2 | 56.0 | 3.23e-08 |
| WP\_018960025.1 | FAD-binding\_protein | BGC0002010 | NRP+Polyketide | 39.0 | 27.2 | 54.0 | 6.83e-08 |
| AAM33667.1 | putative\_FAD-binding\_oxidoreductase | BGC0000230 | Polyketide:Type II polyketide | 39.0 | 22.8 | 52.0 | 3.89e-07 |
| ACB37752.1 | dehydrogenase | BGC0000162 | Polyketide | 31.0 | 34.6 | 51.0 | 7.2e-07 |
| ABM91450.1 | putative\_oxidoreductase | BGC0000348 | NRP | 41.0 | 28.2 | 51.0 | 7.78e-07 |
| AIG62142.1 | isoamyl\_alcohol\_oxidase | BGC0000120 | Polyketide:Iterative type I polyketide | 41.0 | 23.4 | 51.0 | 1.01e-06 |
| EAL94104.1 | FAD\_binding\_oxidoreductase\_CpoX1 | BGC0000811 | Alkaloid | 43.0 | 23.7 | 50.0 | 1.4e-06 |
| XP\_007301603.1 | FAD-binding\_domain-containing\_protein | BGC0001617 | Terpene | 32.0 | 32.4 | 50.0 | 2.4e-06 |
| BBE36455.1 | oxidase | BGC0001922 | Polyketide | 38.0 | 20.2 | 49.0 | 2.84e-06 |
| ABP55175.1 | FAD\_linked\_oxidase\_domain\_protein | BGC0000150 | NRP+Polyketide:Enediyne type I polyketide | 34.0 | 21.5 | 49.0 | 2.91e-06 |
| ATY46582.1 | FAD-linked\_oxidase\_domain\_protein | BGC0001666 | Polyketide | 41.0 | 22.8 | 49.0 | 3.01e-06 |
| KFH44377.1 | 6-hydroxy-D-nicotine\_oxidase-like\_protein | BGC0002190 | Polyketide | 43.0 | 20.8 | 49.0 | 3.19e-06 |
| EAT85327.2 | hypothetical\_protein | BGC0002165 | Polyketide | 42.0 | 20.8 | 47.0 | 9.18e-06 |
| ANY57886.1 | PenH | BGC0001372 | Terpene | 34.0 | 39.7 | 48.0 | 9.91e-06 |
