# Supplementary material for: Alkaliphilic/Alkali-Tolerant Fungi: Molecular, Biochemical, and Biotechnological Aspects
Source: J Fungi (Basel). 2023 Jun 9;9(6):652. doi: 10.3390/jof9060652 (PMC10301932; doi:10.3390/jof9060652)
Supplement: Supplementary file 1 [file jof-09-00652-s001.zip › S2/knownclusterblast/region1/input.path1.gene50_mibig_hits.html]

| MIBiG Protein | Description | MIBiG Cluster | MiBiG Product | % ID | % Coverage | BLAST Score | E-value |
| --- | --- | --- | --- | --- | --- | --- | --- |
| EAU35437.1 | conserved\_hypothetical\_protein | BGC0002734 | Polyketide | 41.0 | 114.1 | 503.0 | 3.18e-170 |
| EAU38788.1 | conserved\_hypothetical\_protein | BGC0000161 | Polyketide:Iterative type I polyketide | 23.0 | 96.2 | 119.0 | 6.22e-28 |
| AEO12714.1 | flavin\_monooxygenase | BGC0001334 | Alkaloid | 33.0 | 31.2 | 97.0 | 9.94e-21 |
| WP\_078870630.1 | FAD-dependent\_oxidoreductase | BGC0002137 | Polyketide | 33.0 | 31.6 | 91.0 | 6.53e-19 |
| OWA01622.1 | oxygenase | BGC0001439 | Polyketide+Saccharide:Hybrid/tailoring saccharide | 30.0 | 31.1 | 90.0 | 2.14e-18 |
| AFU65903.1 | DacO2 | BGC0000216 | Polyketide | 31.0 | 33.4 | 88.0 | 8.96e-18 |
| AGI62225.1 | AbeX1-like\_monooxygenase | BGC0001335 | Alkaloid | 32.0 | 33.8 | 87.0 | 1.17e-17 |
| CEO59274.1 | Putative\_FAD\_binding\_domain\_protein | BGC0002278 | Alkaloid+NRP | 26.0 | 42.8 | 86.0 | 3.34e-17 |
| AHE14649.1 | FAD-binding\_monooxygenase | BGC0001336 | Alkaloid | 33.0 | 28.3 | 84.0 | 1.45e-16 |
| BAQ25478.1 | monooxygenase | BGC0001288 | Polyketide | 34.0 | 29.5 | 84.0 | 2.08e-16 |
| AAZ78335.1 | OxyL | BGC0000254 | Polyketide | 33.0 | 30.8 | 78.0 | 1.31e-14 |
| WP\_020636836.1 | FAD-dependent\_monooxygenase | BGC0002011 | Polyketide | 30.0 | 29.7 | 77.0 | 2.64e-14 |
| QGJ79664.1 | Epoxidase | BGC0002552 | Polyketide | 30.0 | 29.7 | 77.0 | 2.64e-14 |
| ADQ55479.1 | FAD-binding\_monooxygenase | BGC0000350 | NRP:Beta-lactam | 33.0 | 27.8 | 72.0 | 7.57e-13 |
| AQZ37098.1 | epoxidase | BGC0001511 | Polyketide | 28.0 | 29.7 | 71.0 | 2.4e-12 |
| WP\_018891727.1 | FAD-dependent\_oxidoreductase | BGC0001558 | Polyketide | 29.0 | 33.6 | 68.0 | 1.77e-11 |
| ATJ00776.1 | FAD-dependent\_oxidoreductase | BGC0001568 | Polyketide | 29.0 | 33.6 | 68.0 | 1.77e-11 |
| AKG47162.1 | HysC | BGC0001223 | Other | 30.0 | 28.3 | 65.0 | 1.25e-10 |
| BAF47693.1 | monooxygenase | BGC0000825 | Alkaloid | 29.0 | 29.1 | 63.0 | 5.11e-10 |
| AAA83424.1 | RdmE | BGC0000265 | Polyketide | 27.0 | 33.6 | 62.0 | 1.16e-09 |
| KDN80045.1 | FAD-dependent\_oxidoreductase | BGC0001074 | Saccharide+Polyketide | 27.0 | 33.6 | 62.0 | 1.52e-09 |
| EFG04594.1 | Staurosporine\_biosynthesis\_monooxygenase\_StaC | BGC0000826 | Alkaloid | 31.0 | 28.3 | 61.0 | 2.72e-09 |
| ABD59214.1 | InkE | BGC0000813 | Alkaloid | 27.0 | 30.8 | 58.0 | 1.83e-08 |
| ACN29721.1 | monooxygenase | BGC0000814 | Alkaloid | 27.0 | 30.8 | 58.0 | 1.83e-08 |
| WP\_091316344.1 | FAD-dependent\_monooxygenase | BGC0002007 | Terpene | 26.0 | 29.1 | 57.0 | 4.71e-08 |
| CAO98853.1 | monooxygenase\_AufJ | BGC0000023 | Polyketide:Modular type I polyketide | 28.0 | 29.1 | 56.0 | 1.24e-07 |
| AJO72705.1 | Monooxygenase | BGC0001381 | Polyketide | 24.0 | 55.8 | 52.0 | 1.63e-06 |
| AAM97366.1 | RubP | BGC0000266 | Polyketide | 23.0 | 33.4 | 51.0 | 2.69e-06 |
| AVO00807.1 | May8 | BGC0001661 | Polyketide | 28.0 | 28.3 | 50.0 | 6.02e-06 |
