# Supplementary material for: Alkaliphilic/Alkali-Tolerant Fungi: Molecular, Biochemical, and Biotechnological Aspects
Source: J Fungi (Basel). 2023 Jun 9;9(6):652. doi: 10.3390/jof9060652 (PMC10301932; doi:10.3390/jof9060652)
Supplement: Supplementary file 1 [file jof-09-00652-s001.zip › S2/knownclusterblast/region1/input.path1.gene51_mibig_hits.html]

| MIBiG Protein | Description | MIBiG Cluster | MiBiG Product | % ID | % Coverage | BLAST Score | E-value |
| --- | --- | --- | --- | --- | --- | --- | --- |
| EAU31622.1 | conserved\_hypothetical\_protein | BGC0002592 | Polyketide | 47.0 | 101.8 | 185.0 | 8.06e-56 |
| EAL89337.1 | O-methyltransferase,\_putative | BGC0001403 | Polyketide | 43.0 | 101.4 | 174.0 | 1.37e-51 |
