# Supplementary material for: Alkaliphilic/Alkali-Tolerant Fungi: Molecular, Biochemical, and Biotechnological Aspects
Source: J Fungi (Basel). 2023 Jun 9;9(6):652. doi: 10.3390/jof9060652 (PMC10301932; doi:10.3390/jof9060652)
Supplement: Supplementary file 1 [file jof-09-00652-s001.zip › S2/knownclusterblast/region1/input.path1.gene55_mibig_hits.html]

| MIBiG Protein | Description | MIBiG Cluster | MiBiG Product | % ID | % Coverage | BLAST Score | E-value |
| --- | --- | --- | --- | --- | --- | --- | --- |
| ACR44939.1 | 8-amino-7-oxononanoate\_synthase | BGC0001238 | Other | 39.0 | 78.0 | 236.0 | 2.31e-72 |
| ACR44942.1 | 8-amino-7-oxononanoate\_synthase | BGC0001239 | Other | 39.0 | 78.0 | 236.0 | 2.31e-72 |
| AGM05528.1 | 5-aminolevulinate\_synthase | BGC0002098 | Polyketide | 32.0 | 82.4 | 139.0 | 2.27e-36 |
| AAX98209.1 | 5-aminolevulinate\_synthase | BGC0000052 | Polyketide | 33.0 | 62.1 | 135.0 | 1.2e-34 |
| CAH08161.1 | putative\_8-amino-7-oxononanoate\_synthase | BGC0000948 | Other | 27.0 | 70.9 | 132.0 | 6.14e-34 |
| APZ78719.1 | 8-amino-7-oxononanoate\_synthase | BGC0001420 | NRP:Cyclic depsipeptide+Polyketide:Iterative type I polyketide | 29.0 | 77.0 | 125.0 | 2.61e-30 |
| BAG84248.1 | putative\_polyketide\_synthase | BGC0000257 | Polyketide | 26.0 | 70.3 | 102.0 | 1.53e-22 |
| MBA5221219.1 | aminotransferase\_class\_I/II-fold\_pyridoxal\_phosphate-dependent\_enzyme | BGC0002090 | NRP+Polyketide:Modular type I polyketide | 24.0 | 85.2 | 100.0 | 8.65e-22 |
| CAA16183.1 | polyketide\_synthase | BGC0001063 | NRP+Polyketide | 25.0 | 70.9 | 93.0 | 2.47e-19 |
| AHF22854.1 | MarL | BGC0000091 | Polyketide | 25.0 | 76.8 | 92.0 | 4.33e-19 |
