# Supplementary material for: Alkaliphilic/Alkali-Tolerant Fungi: Molecular, Biochemical, and Biotechnological Aspects
Source: J Fungi (Basel). 2023 Jun 9;9(6):652. doi: 10.3390/jof9060652 (PMC10301932; doi:10.3390/jof9060652)
Supplement: Supplementary file 1 [file jof-09-00652-s001.zip › S2/knownclusterblast/region1/input.path1.gene59_mibig_hits.html]

| MIBiG Protein | Description | MIBiG Cluster | MiBiG Product | % ID | % Coverage | BLAST Score | E-value |
| --- | --- | --- | --- | --- | --- | --- | --- |
| AGZ20204.1 | negative\_transcriptional\_regulator\_A | BGC0002618 | Terpene | 27.0 | 55.7 | 59.0 | 1.51e-09 |
| QMW33901.1 | hypothetical\_protein | BGC0002167 | NRP | 27.0 | 81.0 | 57.0 | 1.15e-08 |
| QQO98486.1 | FrzB | BGC0002146 | NRP | 40.0 | 21.3 | 54.0 | 8.79e-08 |
| AGA37264.1 | negative\_regulator | BGC0000816 | NRP+Alkaloid | 38.0 | 23.0 | 52.0 | 4.96e-07 |
