# Supplementary material for: Alkaliphilic/Alkali-Tolerant Fungi: Molecular, Biochemical, and Biotechnological Aspects
Source: J Fungi (Basel). 2023 Jun 9;9(6):652. doi: 10.3390/jof9060652 (PMC10301932; doi:10.3390/jof9060652)
Supplement: Supplementary file 1 [file jof-09-00652-s001.zip › S2/knownclusterblast/region1/input.path1.gene61_mibig_hits.html]

| MIBiG Protein | Description | MIBiG Cluster | MiBiG Product | % ID | % Coverage | BLAST Score | E-value |
| --- | --- | --- | --- | --- | --- | --- | --- |
| ACZ57546.1 | predicted\_MFS\_transporter | BGC0000046 | Polyketide:Iterative type I polyketide | 41.0 | 90.1 | 402.0 | 5.69e-134 |
| BAZ95819.1 | cpaN1\_MFS\_transporter | BGC0001563 | NRP+Polyketide | 39.0 | 92.8 | 381.0 | 1.82e-125 |
| KIA75590.1 | MFS\_transporter | BGC0002209 | Polyketide | 37.0 | 88.9 | 349.0 | 7.06e-114 |
| AIG62136.1 | MFS\_transporter | BGC0000120 | Polyketide:Iterative type I polyketide | 35.0 | 93.3 | 327.0 | 6.63e-105 |
| CBF82297.1 | MFS\_transporter,\_putative\_(AFU\_orthologue;\_AFUA\_5G10340) | BGC0002180 | Polyketide | 36.0 | 88.4 | 325.0 | 3.18e-104 |
| BAE60006.1 |  | BGC0001518 | Terpene | 31.0 | 83.1 | 225.0 | 6.19e-66 |
| BAE71313.1 | putative\_ABC\_transporter | BGC0000004 | Polyketide | 32.0 | 82.4 | 220.0 | 1.09e-64 |
| AAS90046.1 | AflT | BGC0000009 | Polyketide | 33.0 | 86.2 | 219.0 | 5.03e-64 |
| AAS90069.1 | AflT | BGC0000010 | Polyketide | 32.0 | 86.8 | 218.0 | 2.06e-63 |
| CAP96439.1 | Transporter | BGC0000420 | NRP | 31.0 | 80.1 | 217.0 | 3.84e-63 |
| AAS89998.1 | AflT | BGC0000007 | Polyketide | 31.0 | 86.4 | 212.0 | 1.95e-61 |
| BAZ95831.1 | MFS\_transporter\_cpaI | BGC0001563 | NRP+Polyketide | 30.0 | 93.3 | 212.0 | 2.94e-61 |
| EDU47089.1 | MFS\_gliotoxin\_efflux\_transporter\_GliA | BGC0002250 | Polyketide+NRP | 31.0 | 78.8 | 211.0 | 1.1e-60 |
| AAS90092.1 | AflT | BGC0000006 | Polyketide | 31.0 | 86.4 | 207.0 | 1.02e-59 |
| AAS90021.1 | AflT | BGC0000008 | Polyketide | 30.0 | 86.4 | 206.0 | 2.75e-59 |
| OPB37947.1 | MFS\_efflux\_pump | BGC0002206 | Polyketide | 32.0 | 78.0 | 206.0 | 5.38e-59 |
| DAB41650.1 | MFS\_transporter | BGC0001583 | Polyketide | 32.0 | 81.1 | 206.0 | 1.23e-58 |
| BAV69308.1 | PrhG | BGC0001729 | Polyketide+Terpene | 29.0 | 75.0 | 204.0 | 7.42e-58 |
| ACZ66257.1 | APS11 | BGC0000304 | NRP | 30.0 | 94.7 | 203.0 | 9.87e-58 |
| AAD34558.1 | unknown | BGC0000088 | Polyketide | 29.0 | 87.1 | 191.0 | 1.07e-53 |
| XP\_028481817.1 | MFS\_transporter | BGC0001866 | Polyketide | 29.0 | 74.8 | 187.0 | 3.46e-52 |
| ABA02247.1 | efflux\_pump | BGC0000098 | Polyketide | 29.0 | 80.6 | 187.0 | 5.35e-52 |
| BAC20568.1 | efflux\_pump | BGC0000039 | Polyketide | 31.0 | 78.0 | 186.0 | 8.84e-52 |
| AHV78251.1 | ResE | BGC0001246 | Polyketide | 28.0 | 89.4 | 184.0 | 7.53e-51 |
| ACD39756.1 | major\_facilitator\_superfamily\_transporter | BGC0000076 | Polyketide | 28.0 | 86.2 | 181.0 | 7.05e-50 |
| ACD39765.1 | major\_facilitator\_superfamily\_transporter | BGC0000077 | Polyketide | 28.0 | 86.2 | 181.0 | 7.05e-50 |
| PIB01159.1 | putative\_HC-toxin\_efflux\_carrier\_TOXA | BGC0001541 | Polyketide | 28.0 | 78.5 | 179.0 | 6.73e-49 |
| CBF76046.1 | conserved\_hypothetical\_protein | BGC0001399 | NRP | 28.0 | 70.5 | 176.0 | 2.09e-48 |
| OJJ98488.1 | hypothetical\_protein | BGC0002169 | Polyketide+NRP | 28.0 | 81.5 | 175.0 | 1.4e-47 |
| XP\_001798920.1 | MFS\_transporter | BGC0001865 | Polyketide:Iterative type I polyketide | 30.0 | 70.7 | 175.0 | 1.59e-47 |
| QQW45466.1 | major\_facilitator\_superfamily\_transporter\_CalB' | BGC0002168 | Polyketide | 26.0 | 79.5 | 174.0 | 5.92e-47 |
| BBM05083.1 | putative\_transporter | BGC0002170 | Polyketide | 26.0 | 79.5 | 174.0 | 5.92e-47 |
| EAU31926.1 | conserved\_hypothetical\_protein | BGC0002267 | Polyketide | 27.0 | 88.9 | 171.0 | 3.92e-46 |
| AAM94765.1 | CalT1 | BGC0000033 | Polyketide | 27.0 | 83.8 | 159.0 | 6.03e-42 |
| EAQ86390.1 | hypothetical\_protein | BGC0001405 | Polyketide | 29.0 | 66.5 | 157.0 | 3.13e-41 |
| ADU85991.1 | putative\_membrane\_transport\_protein | BGC0000165 | Polyketide:Modular type I polyketide | 27.0 | 84.8 | 147.0 | 8.41e-38 |
| QKG86294.1 | MFS\_general\_substrate\_transporter | BGC0002253 | Polyketide | 27.0 | 73.2 | 147.0 | 3.38e-37 |
| OSS48493.1 | hypothetical\_protein | BGC0002194 | Polyketide | 27.0 | 72.8 | 141.0 | 1.68e-35 |
| EAU36749.1 | predicted\_protein | BGC0000292 | NRP | 28.0 | 71.8 | 137.0 | 5.67e-35 |
| EAU38977.1 | predicted\_protein | BGC0001122 | NRP+Polyketide:Iterative type I polyketide | 27.0 | 81.3 | 137.0 | 2.75e-34 |
| QOG08945.1 | FfsH | BGC0002204 | Polyketide+NRP | 26.0 | 95.2 | 134.0 | 2.6e-33 |
| AGN71625.1 | putative\_HC-toxin\_efflux\_carrier\_TOXA | BGC0000027 | Polyketide:Iterative type I polyketide | 25.0 | 78.5 | 119.0 | 1.31e-28 |
| AMY15059.1 | MFS\_transporter | BGC0001339 | Polyketide:Iterative type I polyketide | 28.0 | 63.0 | 109.0 | 7.67e-25 |
| CAC44196.1 | putative\_actinorhodin\_transporter | BGC0000194 | Polyketide:Type II polyketide | 25.0 | 89.1 | 100.0 | 6.08e-22 |
| AAQ08939.1 | putative\_membrane\_transporter | BGC0000224 | Polyketide:Type II polyketide | 25.0 | 85.4 | 98.0 | 3.66e-21 |
| AAA67509.1 | tetracenomycin\_C\_resistance\_and\_export\_protein | BGC0000275 | Polyketide:Type II polyketide | 25.0 | 79.2 | 86.0 | 1.98e-17 |
| ARS01481.1 | NcmH | BGC0001702 | NRP+Polyketide | 24.0 | 80.4 | 86.0 | 2.01e-17 |
| ALD83683.1 | multidrug\_efflux\_protein | BGC0001300 | Polyketide | 23.0 | 88.7 | 82.0 | 3.03e-16 |
| AGH68910.1 | MFS-like\_transporter | BGC0001083 | Terpene+Polyketide:Type III polyketide | 25.0 | 81.5 | 82.0 | 5.61e-16 |
| CAM56779.1 | Mem2 | BGC0000354 | NRP | 23.0 | 73.4 | 73.0 | 3.73e-13 |
| AEA60653.1 | transporter | BGC0000892 | Other | 28.0 | 37.0 | 66.0 | 5.94e-11 |
| AQW35028.1 | Multidrug\_MFS\_transporter | BGC0001675 | Polyketide | 24.0 | 86.2 | 66.0 | 8.18e-11 |
| BAN59741.1 | MFS\_transporter | BGC0001075 | Terpene+Polyketide | 22.0 | 79.2 | 65.0 | 9.44e-11 |
| WP\_016640227.1 | DHA2\_family\_efflux\_MFS\_transporter\_permease\_subunit | BGC0002000 | Polyketide | 23.0 | 79.4 | 65.0 | 9.63e-11 |
| ADB02838.1 | AzicY | BGC0000202 | Polyketide | 30.0 | 31.9 | 62.0 | 1.04e-09 |
| ALI92656.1 | MRR1\_Major\_Facilitator\_Superfamily\_(MFS)\_protein | BGC0001338 | Polyketide:Iterative type I polyketide | 32.0 | 22.6 | 57.0 | 2.54e-08 |
| BAV56014.1 | MFS\_transporter | BGC0001597 | Polyketide | 23.0 | 82.2 | 55.0 | 1.81e-07 |
| PLB34719.1 | major\_facilitator\_superfamily\_domain-containing\_protein | BGC0002749 | NRP+Polyketide | 28.0 | 21.3 | 52.0 | 1.67e-06 |
