# Supplementary material for: Alkaliphilic/Alkali-Tolerant Fungi: Molecular, Biochemical, and Biotechnological Aspects
Source: J Fungi (Basel). 2023 Jun 9;9(6):652. doi: 10.3390/jof9060652 (PMC10301932; doi:10.3390/jof9060652)
Supplement: Supplementary file 1 [file jof-09-00652-s001.zip › S2/knownclusterblast/region1/input.path1.gene62_mibig_hits.html]

| MIBiG Protein | Description | MIBiG Cluster | MiBiG Product | % ID | % Coverage | BLAST Score | E-value |
| --- | --- | --- | --- | --- | --- | --- | --- |
| BDA39138.1 | short\_chain\_dehydrogenase/reductase | BGC0001398 | RiPP | 35.0 | 103.1 | 134.0 | 5.22e-37 |
| XP\_028481816.1 | short\_chain\_dehydrogenase/reductase | BGC0001866 | Polyketide | 32.0 | 111.6 | 107.0 | 9.47e-27 |
| QKG86292.1 | enoyl\_reductase | BGC0002253 | Polyketide | 33.0 | 106.5 | 109.0 | 1.24e-26 |
| MCG7203804.1 | SDR\_family\_oxidoreductase | BGC0000248 | Polyketide | 32.0 | 83.3 | 89.0 | 2.15e-20 |
| WP\_008704360.1 | SDR\_family\_oxidoreductase | BGC0001575 | NRP | 28.0 | 91.8 | 88.0 | 4.59e-20 |
| AJI44188.1 | hypothetical\_protein | BGC0001193 | NRP | 32.0 | 93.2 | 86.0 | 2.44e-19 |
| BAI70380.1 | short\_chain\_dehydrogenase | BGC0000896 | Other | 30.0 | 71.8 | 86.0 | 4.16e-19 |
| BAL90266.1 | putative\_short-chain\_dehydrogenase | BGC0002021 | Polyketide | 29.0 | 90.1 | 82.0 | 3.89e-18 |
| ABY83179.1 | Azi41 | BGC0000960 | NRP+Polyketide | 30.0 | 93.2 | 82.0 | 5.64e-18 |
| APR73624.1 | dehydrogenase | BGC0001625 | Polyketide | 34.0 | 69.4 | 79.0 | 7.89e-17 |
| AAL15603.1 | SimJ1 | BGC0000270 | Polyketide | 30.0 | 91.8 | 78.0 | 2.67e-16 |
| AAK06807.1 | putative\_3-keto-acyl-reductase\_SimD2 | BGC0001072 | Saccharide+Polyketide:Modular type I polyketide+Polyketide:Type II polyketide+Other:Aminocoumarin | 30.0 | 91.8 | 78.0 | 2.67e-16 |
| AEE65483.1 | short-chain\_dehydrogenase/reductase\_SDR | BGC0000223 | Polyketide:Type II polyketide | 31.0 | 70.7 | 76.0 | 6.01e-16 |
| ANY57970.1 | Short\_chain\_dehydrogenase | BGC0001369 | Polyketide | 30.0 | 90.8 | 76.0 | 1.24e-15 |
| WP\_018891734.1 | SDR\_family\_oxidoreductase | BGC0001558 | Polyketide | 29.0 | 91.2 | 75.0 | 1.61e-15 |
| CAI47656.1 | putative\_tobramycin/apramycin\_oxidoreductase\_3 | BGC0000692 | Saccharide | 31.0 | 83.3 | 75.0 | 1.73e-15 |
| ATJ00769.1 | C-7\_ketoreductase | BGC0001568 | Polyketide | 29.0 | 91.2 | 75.0 | 1.73e-15 |
| AXM42926.1 | ketoacyl\_reductase | BGC0001940 | Polyketide | 29.0 | 92.5 | 74.0 | 4.09e-15 |
| ABS75236.1 | DhbA | BGC0001185 | NRP:NRP siderophore | 26.0 | 92.2 | 73.0 | 9.35e-15 |
| QNH67547.1 | Cip19 | BGC0002108 | NRP | 28.0 | 79.6 | 73.0 | 1.15e-14 |
| EHM27499.1 | putative\_short\_chain\_oxidoreductase | BGC0000235 | Polyketide | 30.0 | 74.8 | 72.0 | 1.31e-14 |
| FAC38\_10 |  | BGC0002198 | NRP | 33.0 | 74.1 | 73.0 | 1.6e-14 |
| OKI59868.1 | short-chain\_dehydrogenase | BGC0002477 | Polyketide | 30.0 | 74.8 | 72.0 | 1.79e-14 |
| KDN80052.1 | ketoreductase | BGC0001074 | Saccharide+Polyketide | 28.0 | 91.2 | 72.0 | 2.01e-14 |
| CAA09651.1 |  | BGC0000227 | Polyketide:Type II polyketide | 28.0 | 89.8 | 72.0 | 2.19e-14 |
| ALG65315.1 | Cal21 | BGC0001297 | NRP | 26.0 | 93.2 | 72.0 | 2.2e-14 |
| AAP69586.1 | putative\_ketoreductase | BGC0000226 | Polyketide | 28.0 | 86.7 | 72.0 | 3.4e-14 |
| AMX23331.1 | putative\_acyl\_carrier\_protein | BGC0001500 | Polyketide | 29.0 | 84.7 | 71.0 | 5.31e-14 |
| QBA57739.1 | NAD(P)-dependent\_oxidoreductase | BGC0002377 | NRP | 25.0 | 93.2 | 71.0 | 5.54e-14 |
| CAI94720.1 | putative\_oxyacyl-(acyl\_carrier\_protein)\_reductase | BGC0000141 | Polyketide | 28.0 | 78.9 | 71.0 | 5.6e-14 |
| BBE36453.1 | dehydrogenase | BGC0001922 | Polyketide | 33.0 | 58.2 | 71.0 | 8.79e-14 |
| CBG67532.1 | putative\_oxidoreductase | BGC0002367 | NRP | 26.0 | 84.4 | 71.0 | 1.03e-13 |
| CEO59270.1 | Putative\_2-deoxy-D-gluconate\_3-dehydrogenase | BGC0002278 | Alkaloid+NRP | 26.0 | 91.8 | 70.0 | 1.27e-13 |
| AKT74300.1 | TxnC4 | BGC0002141 | Polyketide | 27.0 | 81.0 | 69.0 | 1.74e-13 |
| QBG38776.1 | Atr15 | BGC0001975 | NRP | 26.0 | 78.6 | 69.0 | 2.64e-13 |
| EDY42546.1 | ketoreductase | BGC0000212 | Polyketide:Type II polyketide | 28.0 | 91.8 | 69.0 | 3.62e-13 |
| ADE34491.1 | ssfU | BGC0000269 | Polyketide:Type II polyketide+Saccharide:Hybrid/tailoring saccharide | 24.0 | 86.4 | 68.0 | 5.73e-13 |
| AAF81727.1 | putative\_ketoreductase\_EncD | BGC0000220 | Polyketide:Type II polyketide | 27.0 | 86.4 | 68.0 | 6.05e-13 |
| QIC03941.1 | EncD | BGC0002366 | Polyketide | 27.0 | 86.4 | 68.0 | 6.05e-13 |
| AAZ55906.1 | 2,3-dihydro-2,3-dihydroxybenzoate\_dehydrogenase;\_RBL00455 | BGC0000359 | NRP | 29.0 | 90.5 | 68.0 | 7.72e-13 |
| QDQ37876.1 | ketoreductase | BGC0001979 | Polyketide | 27.0 | 80.6 | 66.0 | 2.1e-12 |
| AGN71606.1 | ketoreductase\_ | BGC0000027 | Polyketide:Iterative type I polyketide | 28.0 | 68.4 | 67.0 | 2.12e-12 |
| BAU98027.1 | dehydrogenase | BGC0001386 | Polyketide | 31.0 | 70.7 | 66.0 | 2.29e-12 |
| XP\_004252844.1 | uncharacterized\_protein\_LOC101268370 | BGC0002404 | Other | 33.0 | 69.4 | 66.0 | 2.94e-12 |
| AAC18111.1 | ketoreductase | BGC0000225 | Polyketide | 26.0 | 92.2 | 66.0 | 4.03e-12 |
| OOH83075.1 | 3-oxoacyl-ACP\_reductase | BGC0001987 | Polyketide | 27.0 | 90.5 | 66.0 | 4.16e-12 |
| ARF06204.1 | short-chain\_dehydrogenase/reductase\_SDR | BGC0001593 | NRP | 27.0 | 77.2 | 66.0 | 4.5e-12 |
| PPQ57492.1 | ketoacyl\_reductase | BGC0002016 | Polyketide | 25.0 | 98.3 | 66.0 | 4.96e-12 |
| MCG7203803.1 | 3-oxoacyl-ACP\_reductase | BGC0000248 | Polyketide | 26.0 | 89.5 | 65.0 | 9.16e-12 |
| QXJ26486.1 | mycofactocin-coupled\_SDR\_family\_oxidoreductase | BGC0002370 | NRP | 24.0 | 94.2 | 65.0 | 1.04e-11 |
| AAF73457.1 | putative\_aklaviketone\_reductase | BGC0000193 | Polyketide | 28.0 | 93.5 | 64.0 | 1.32e-11 |
| PVC99863.1 | 3-oxoacyl-ACP\_reductase\_FabG | BGC0002100 | NRP+Other | 25.0 | 91.2 | 64.0 | 1.48e-11 |
| CAG38717.1 | putative\_dehydrogenase | BGC0000708 | Saccharide | 28.0 | 76.9 | 64.0 | 1.79e-11 |
| CAA09652.1 | polyketide\_ketoreductase | BGC0000227 | Polyketide:Type II polyketide | 26.0 | 92.2 | 64.0 | 1.87e-11 |
| AME18007.1 | oxidoreductase | BGC0001378 | Polyketide:Enediyne type I polyketide | 28.0 | 68.0 | 64.0 | 1.99e-11 |
| WP\_037817190.1 | 3-oxoacyl-ACP\_reductase\_FabG | BGC0002137 | Polyketide | 26.0 | 91.5 | 64.0 | 2.29e-11 |
| AEI98650.1 | CtcG | BGC0000209 | Polyketide | 28.0 | 86.7 | 64.0 | 2.32e-11 |
| OKJ61997.1 | short-chain\_dehydrogenase | BGC0002147 | NRP | 28.0 | 75.2 | 63.0 | 2.73e-11 |
| CAP12610.1 | dehydrogenase | BGC0000219 | Polyketide:Type II polyketide+Saccharide:Hybrid/tailoring saccharide | 28.0 | 92.9 | 63.0 | 3.08e-11 |
| AHZ61852.1 | short-chain\_dehydrogenase | BGC0000240 | Polyketide:Type II polyketide+Saccharide:Hybrid/tailoring saccharide | 30.0 | 67.3 | 63.0 | 3.47e-11 |
| CAH10175.1 | ChaZ\_protein | BGC0000207 | Polyketide | 26.0 | 93.2 | 62.0 | 5.73e-11 |
| QBK46640.1 | HrsK3 | BGC0001960 | Polyketide | 28.0 | 69.0 | 62.0 | 5.73e-11 |
| QHZ32176.1 | putative\_ketoacyl\_reductase | BGC0002047 | Polyketide | 27.0 | 92.5 | 62.0 | 5.73e-11 |
| WP\_016640238.1 | 3-oxoacyl-ACP\_reductase\_FabG | BGC0002000 | Polyketide | 26.0 | 92.9 | 62.0 | 7.77e-11 |
| ADB02846.1 | AzicD | BGC0000202 | Polyketide | 26.0 | 91.8 | 62.0 | 1.01e-10 |
| QDG00823.1 | polyketide\_ketoreductase | BGC0002028 | Polyketide | 25.0 | 90.8 | 62.0 | 1.04e-10 |
| CAC44199.1 | ketoacyl\_reductase | BGC0000194 | Polyketide:Type II polyketide | 25.0 | 84.7 | 62.0 | 1.05e-10 |
| CAB15190.2 | 2,3-dihydro-2,3-dihydroxybenzoate\_dehydrogenase | BGC0000309 | NRP | 26.0 | 96.6 | 62.0 | 1.05e-10 |
| QFS19054.1 | ketoreductase | BGC0002506 | Polyketide | 26.0 | 87.4 | 62.0 | 1.09e-10 |
| WP\_010369414.1 | SDR\_family\_oxidoreductase | BGC0000314 | Polyketide+NRP:Cyclic depsipeptide+Other:Aminocoumarin | 26.0 | 90.8 | 61.0 | 1.2e-10 |
| AHY06369.1 | 3-oxoacyl-ACP\_reductase | BGC0002496 | NRP | 27.0 | 90.8 | 61.0 | 1.26e-10 |
| AQW35064.1 | Polyketide\_C-9\_ketoreductase | BGC0001675 | Polyketide | 25.0 | 85.7 | 61.0 | 1.44e-10 |
| AMK92578.1 | oxidoreductase | BGC0001377 | Polyketide | 27.0 | 68.0 | 61.0 | 1.45e-10 |
| AHL46733.1 | ketoreductase | BGC0001179 | Polyketide:Type II polyketide | 26.0 | 86.7 | 61.0 | 1.56e-10 |
| AHG26146.1 | quinone\_reductase | BGC0000812 | Alkaloid | 33.0 | 42.9 | 61.0 | 1.72e-10 |
| AAO39097.1 | AdmC | BGC0000956 | NRP:Beta-lactam+Polyketide:Type II polyketide | 27.0 | 75.2 | 61.0 | 1.73e-10 |
| CCH32748.1 | Ketoreductase | BGC0002070 | Polyketide | 27.0 | 92.2 | 61.0 | 1.9e-10 |
| AAM33668.1 | putative\_3-oxoacyl-ACP\_reductase | BGC0000230 | Polyketide:Type II polyketide | 28.0 | 84.0 | 61.0 | 2.34e-10 |
| QOP59273.1 | keto-reductase | BGC0002504 | Polyketide | 26.0 | 90.8 | 61.0 | 2.62e-10 |
| AVO00812.1 | May13 | BGC0001661 | Polyketide | 26.0 | 90.8 | 61.0 | 2.64e-10 |
| ABP54641.1 | short-chain\_dehydrogenase/reductase\_SDR | BGC0000241 | Polyketide:Type II polyketide+Saccharide:Hybrid/tailoring saccharide | 30.0 | 66.7 | 61.0 | 2.66e-10 |
| WP\_040253449.1 | SDR\_family\_NAD(P)-dependent\_oxidoreductase | BGC0001596 | Polyketide | 26.0 | 92.2 | 61.0 | 2.69e-10 |
| MUL41457.1 | SDR\_family\_oxidoreductase | BGC0002045 | Polyketide:Type II polyketide | 29.0 | 68.7 | 60.0 | 2.93e-10 |
| AFJ52671.1 | ketoreductase | BGC0001073 | NRP+Polyketide | 28.0 | 92.2 | 60.0 | 3.48e-10 |
| EDY42534.1 | monensin\_polyketide\_synthase\_ketoacyl\_reductase | BGC0000212 | Polyketide:Type II polyketide | 25.0 | 93.5 | 60.0 | 3.54e-10 |
| KDN80050.1 | ketoacyl\_reductase | BGC0001074 | Saccharide+Polyketide | 27.0 | 86.4 | 60.0 | 3.54e-10 |
| ANY58988.1 | short-chain\_dehydrogenase | BGC0001615 | NRP | 23.0 | 91.8 | 60.0 | 4.49e-10 |
| AAO65349.1 | putative\_ketoreductase | BGC0000236 | Polyketide | 27.0 | 91.2 | 60.0 | 4.79e-10 |
| AAK57528.1 | PgaD | BGC0000262 | Polyketide:Type II polyketide+Saccharide:Hybrid/tailoring saccharide | 27.0 | 92.2 | 60.0 | 4.79e-10 |
| OKI81335.1 | short-chain\_dehydrogenase | BGC0002478 | Polyketide | 28.0 | 67.3 | 59.0 | 5.06e-10 |
| ADG86311.1 | 3-ketoacyl\_ACP-reductase | BGC0000190 | Polyketide | 25.0 | 81.0 | 59.0 | 5.66e-10 |
| CAH10174.1 | ChaL\_protein | BGC0000207 | Polyketide | 27.0 | 91.8 | 59.0 | 5.66e-10 |
| CAJ34364.1 | NAD\_or\_NADP\_oxidoreductase | BGC0000445 | NRP:Cyclic depsipeptide | 27.0 | 90.8 | 59.0 | 6.65e-10 |
| ACN38371.1 | short-chain\_dehydrogenase/reductase | BGC0000714 | Saccharide | 27.0 | 79.6 | 59.0 | 7.6e-10 |
| AAL15583.1 | Sim5 | BGC0000270 | Polyketide | 25.0 | 90.5 | 59.0 | 1.19e-09 |
| AAK06787.1 | putative\_ketoreductase\_SimA6 | BGC0001072 | Saccharide+Polyketide:Modular type I polyketide+Polyketide:Type II polyketide+Other:Aminocoumarin | 25.0 | 90.5 | 59.0 | 1.19e-09 |
| BAJ07858.1 | putative\_ketoacyl\_reductase | BGC0000232 | Polyketide | 28.0 | 70.7 | 58.0 | 1.51e-09 |
| BAV17002.1 | putative\_ketoreductase | BGC0001384 | Polyketide | 25.0 | 89.8 | 58.0 | 1.6e-09 |
| QNL10616.1 | Ketoacyl\_reductase | BGC0002514 | Polyketide | 25.0 | 89.8 | 58.0 | 1.6e-09 |
| AUI41032.1 | 3-oxoacyl-(acyl-carrier\_protein)\_reductase | BGC0001512 | Polyketide | 26.0 | 90.8 | 58.0 | 2.08e-09 |
| OKI81342.1 | ketoacyl\_reductase | BGC0002478 | Polyketide | 25.0 | 92.2 | 58.0 | 2.17e-09 |
| EDY47129.1 | clavaldehyde\_dehydrogenase | BGC0000845 | Other:Non-NRP beta-lactam | 26.0 | 68.0 | 57.0 | 2.62e-09 |
| BCD52387.1 | short-chain\_dehydrogenase/reductase\_SptI | BGC0002537 | Polyketide+Terpene | 26.0 | 69.7 | 58.0 | 2.64e-09 |
| AFO85456.1 | reductase/oxidase | BGC0000391 | NRP | 26.0 | 90.8 | 57.0 | 2.67e-09 |
| AFJ52673.1 | C-9\_ketoreductase | BGC0001073 | NRP+Polyketide | 27.0 | 93.2 | 57.0 | 2.92e-09 |
| TRO56980.1 | SDR\_family\_NAD(P)-dependent\_oxidoreductase | BGC0002361 | Polyketide+Saccharide | 25.0 | 90.8 | 57.0 | 3.04e-09 |
| AAZ78333.1 | OxyJ | BGC0000254 | Polyketide | 24.0 | 91.8 | 57.0 | 4.01e-09 |
| CAH10114.1 | putative\_ketoreducatse | BGC0000268 | Polyketide | 24.0 | 89.5 | 57.0 | 4.1e-09 |
| AWH12904.1 | KR\_domain-containing\_protein | BGC0001784 | Polyketide | 24.0 | 89.1 | 57.0 | 4.87e-09 |
| UPN68084.1 | reductase | BGC0002672 | Polyketide | 25.0 | 91.2 | 57.0 | 5.29e-09 |
| AXL88813.1 | ketoacyl\_reductase | BGC0001895 | Polyketide | 25.0 | 92.2 | 57.0 | 5.37e-09 |
| ADI71446.1 | putative\_ketoreductase | BGC0000203 | Polyketide | 25.0 | 92.2 | 56.0 | 7.19e-09 |
| AHA81975.1 | Ketoreductase | BGC0000199 | Polyketide:Type II polyketide+Saccharide:Hybrid/tailoring saccharide | 27.0 | 91.8 | 56.0 | 9.27e-09 |
| AEE65465.1 | ketoreductase | BGC0000223 | Polyketide:Type II polyketide | 25.0 | 92.5 | 56.0 | 9.63e-09 |
| ACX83621.1 | keto\_reductase | BGC0000221 | Polyketide | 27.0 | 66.7 | 56.0 | 9.7e-09 |
| CBZ42141.1 | Acyl-Co\_Adehydrogenase | BGC0001117 | NRP | 24.0 | 84.7 | 56.0 | 1.08e-08 |
| AAD13539.1 | reductase\_homolog | BGC0000239 | Polyketide:Type II polyketide+Saccharide:Hybrid/tailoring saccharide | 26.0 | 86.4 | 56.0 | 1.31e-08 |
| AHD25940.1 | putative\_ketoreductase | BGC0000208 | Polyketide | 24.0 | 93.9 | 56.0 | 1.32e-08 |
| ARD70866.1 | Short-chain\_dehydrogenase/reductase | BGC0001693 | Polyketide | 26.0 | 67.3 | 55.0 | 1.52e-08 |
| AAZ23052.1 | probable\_dehydrogenase | BGC0000291 | NRP | 24.0 | 95.9 | 56.0 | 1.64e-08 |
| ALJ99852.1 | FlsE | BGC0001904 | Polyketide | 25.0 | 99.3 | 55.0 | 1.75e-08 |
| ABP54645.1 | short-chain\_dehydrogenase/reductase\_SDR | BGC0000241 | Polyketide:Type II polyketide+Saccharide:Hybrid/tailoring saccharide | 25.0 | 86.1 | 55.0 | 1.76e-08 |
| ATJ00771.1 | ketoacyl\_reductase | BGC0001568 | Polyketide | 26.0 | 86.4 | 55.0 | 1.76e-08 |
| QLQ36629.1 | SDR\_family\_NAD(P)-dependent\_oxidoreductase | BGC0002097 | NRP+Polyketide:Type II polyketide+Saccharide:Hybrid/tailoring saccharide | 24.0 | 91.8 | 55.0 | 2.38e-08 |
| AYU66237.1 | TjhC3 | BGC0002461 | Polyketide | 25.0 | 91.5 | 55.0 | 2.38e-08 |
| AAQ08912.1 | putative\_3-ketoacyl-ACP\_reductase | BGC0000224 | Polyketide:Type II polyketide | 25.0 | 81.0 | 55.0 | 2.46e-08 |
| EHM27508.1 | short-chain\_dehydrogenase/reductase\_SDR | BGC0000235 | Polyketide | 24.0 | 89.5 | 54.0 | 3.09e-08 |
| CBH32818.1 | putative\_ketoreductase | BGC0000263 | Polyketide | 25.0 | 76.2 | 54.0 | 3.2e-08 |
| OKI59859.1 | ketoacyl\_reductase | BGC0002477 | Polyketide | 24.0 | 89.5 | 54.0 | 3.2e-08 |
| ABQ04140.1 | short-chain\_dehydrogenase/reductase\_SDR | BGC0000838 | Polyketide | 26.0 | 80.6 | 54.0 | 3.77e-08 |
| OKI59856.1 | dehydrogenase | BGC0002477 | Polyketide | 25.0 | 68.4 | 54.0 | 4.11e-08 |
| AHZ61856.1 | short-chain\_dehydrogenase/reductase | BGC0000240 | Polyketide:Type II polyketide+Saccharide:Hybrid/tailoring saccharide | 25.0 | 83.3 | 54.0 | 4.32e-08 |
| QTA30588.1 | SDR\_family\_NAD(P)-dependent\_oxidoreductase | BGC0002143 | Polyketide | 26.0 | 83.7 | 54.0 | 4.32e-08 |
| ACI88864.1 | AlnP\_ketoreductase | BGC0000195 | Polyketide:Type II polyketide | 24.0 | 87.4 | 54.0 | 4.38e-08 |
| CAL80830.1 | dehydrogenase-related\_protein | BGC0000997 | NRP+Polyketide | 25.0 | 84.7 | 54.0 | 4.8e-08 |
| BAB72043.1 | AknA | BGC0000191 | Polyketide | 24.0 | 93.2 | 54.0 | 5.81e-08 |
| WP\_020275098.1 | SDR\_family\_NAD(P)-dependent\_oxidoreductase | BGC0002012 | Polyketide | 25.0 | 91.2 | 54.0 | 5.81e-08 |
| AAL24452.1 | RdmJ | BGC0000265 | Polyketide | 26.0 | 86.4 | 54.0 | 5.93e-08 |
| ANW12115.1 | putative\_dehydrogenase/reductase | BGC0001374 | Other | 27.0 | 78.2 | 54.0 | 6.31e-08 |
| BAP81863.1 | AndI | BGC0002612 | Terpene | 24.0 | 69.4 | 54.0 | 6.88e-08 |
| C5F59\_12930 | dehydrogenase | BGC0002016 | Polyketide | 29.0 | 55.4 | 53.0 | 7.13e-08 |
| AAF70104.1 | AknA | BGC0000192 | Polyketide | 24.0 | 93.2 | 53.0 | 7.83e-08 |
| ACU59350.1 | short-chain\_dehydrogenase/reductase\_SDR | BGC0000839 | Other | 25.0 | 68.0 | 53.0 | 9.18e-08 |
| AIL50168.1 | putative\_ketoreductase | BGC0000213 | Polyketide:Type II polyketide | 28.0 | 77.2 | 53.0 | 9.47e-08 |
| SCN11974.1 | short-chain\_dehydrogenase/reductase\_SDR | BGC0001580 | Polyketide | 25.0 | 90.8 | 53.0 | 9.83e-08 |
| QDQ37882.1 | ketoreductase | BGC0001979 | Polyketide | 26.0 | 82.0 | 53.0 | 1.05e-07 |
| AKT74293.1 | TxnC2 | BGC0002141 | Polyketide | 25.0 | 93.9 | 53.0 | 1.05e-07 |
| EHM27511.1 | putative\_short\_chain\_dehydrogenase | BGC0000235 | Polyketide | 24.0 | 68.4 | 52.0 | 1.35e-07 |
| ARO44671.1 | ketoreductase | BGC0001769 | Polyketide | 24.0 | 89.8 | 52.0 | 1.42e-07 |
| MBW8699686.1 | putative\_ketoacyl\_reductase | BGC0002140 | Polyketide | 24.0 | 89.8 | 52.0 | 1.42e-07 |
| AAF23366.1 | PhaB | BGC0000866 | Other | 25.0 | 75.2 | 52.0 | 1.72e-07 |
| AGO50613.1 | ketoreductase | BGC0000229 | Polyketide:Type II polyketide+Saccharide:Hybrid/tailoring saccharide | 26.0 | 92.5 | 52.0 | 1.91e-07 |
| WP\_031147019.1 | 9-ketoreductase | BGC0001851 | Polyketide:Type II polyketide+Saccharide:Hybrid/tailoring saccharide | 26.0 | 84.4 | 52.0 | 1.91e-07 |
| XP\_007301854.1 | NAD-P-binding\_protein | BGC0001617 | Terpene | 29.0 | 70.1 | 52.0 | 2.22e-07 |
| CAQ64702.1 | probable\_glucose\_dehydrogenase | BGC0000087 | Polyketide | 29.0 | 70.1 | 52.0 | 2.35e-07 |
| AJS09392.1 | Ketoacyl-(acyl-carrier-protein)\_reductase | BGC0001150 | Polyketide:Type II polyketide+Polyketide:Type III polyketide | 26.0 | 68.7 | 52.0 | 2.78e-07 |
| ABW96534.1 | putative\_dehydrogenase | BGC0000159 | Polyketide:Modular type I polyketide | 24.0 | 91.5 | 50.0 | 5.29e-07 |
| CAG14968.1 | ketoreductase | BGC0000253 | Polyketide:Type II polyketide | 25.0 | 92.5 | 50.0 | 6.19e-07 |
| ARK36158.1 | ketoacyl\_reductase | BGC0001723 | Polyketide | 25.0 | 92.5 | 50.0 | 6.19e-07 |
| POM23765.1 | putative\_ketoacyl\_reductase | BGC0002369 | Polyketide | 25.0 | 92.5 | 50.0 | 6.19e-07 |
| CBH32080.1 | putative\_polyketide\_ketoreductase | BGC0000211 | Polyketide | 24.0 | 76.2 | 50.0 | 6.23e-07 |
| AAB36565.1 | ketoreductase | BGC0000234 | Polyketide | 23.0 | 91.2 | 50.0 | 6.34e-07 |
| BAP34750.1 | dehydrogenase | BGC0000078 | Polyketide | 24.0 | 79.6 | 49.0 | 1.4e-06 |
| AAG23281.1 | probable\_keto\_acyl\_reductase | BGC0000148 | Polyketide | 27.0 | 59.5 | 49.0 | 1.96e-06 |
| WP\_030957358.1 | 9-ketoreductase | BGC0001852 | Polyketide:Type II polyketide+Saccharide:Hybrid/tailoring saccharide | 25.0 | 84.4 | 48.0 | 3.65e-06 |
| OBR09785.1 | Short\_chain\_dehydrogenase | BGC0002429 | Terpene+Polyketide | 25.0 | 57.5 | 48.0 | 3.79e-06 |
| ABL09955.1 | ketoreductase | BGC0000197 | Polyketide:Type II polyketide+Saccharide:Hybrid/tailoring saccharide | 24.0 | 92.9 | 48.0 | 4.9e-06 |
| PYC67523.1 | NAD(P)-dependent\_oxidoreductase | BGC0001473 | RiPP:Thiopeptide | 26.0 | 68.4 | 47.0 | 9.25e-06 |
