# Supplementary material for: Alkaliphilic/Alkali-Tolerant Fungi: Molecular, Biochemical, and Biotechnological Aspects
Source: J Fungi (Basel). 2023 Jun 9;9(6):652. doi: 10.3390/jof9060652 (PMC10301932; doi:10.3390/jof9060652)
Supplement: Supplementary file 1 [file jof-09-00652-s001.zip › S2/knownclusterblast/region1/input.path1.gene7_mibig_hits.html]

| MIBiG Protein | Description | MIBiG Cluster | MiBiG Product | % ID | % Coverage | BLAST Score | E-value |
| --- | --- | --- | --- | --- | --- | --- | --- |
| ESU07753.1 | hypothetical\_protein | BGC0002709 | Polyketide | 28.0 | 77.1 | 116.0 | 4.61e-28 |
| QKG86292.1 | enoyl\_reductase | BGC0002253 | Polyketide | 34.0 | 47.8 | 98.0 | 2.35e-21 |
