# Supplementary material for: Alkaliphilic/Alkali-Tolerant Fungi: Molecular, Biochemical, and Biotechnological Aspects
Source: J Fungi (Basel). 2023 Jun 9;9(6):652. doi: 10.3390/jof9060652 (PMC10301932; doi:10.3390/jof9060652)
Supplement: Supplementary file 1 [file jof-09-00652-s001.zip › S2/knownclusterblast/region1/input.path1.gene9_mibig_hits.html]

| MIBiG Protein | Description | MIBiG Cluster | MiBiG Product | % ID | % Coverage | BLAST Score | E-value |
| --- | --- | --- | --- | --- | --- | --- | --- |
| AAU93813.2 | LipN | BGC0000054 | Polyketide | 31.0 | 63.8 | 82.0 | 7.66e-17 |
| CAP12611.1 | lipase | BGC0000219 | Polyketide:Type II polyketide+Saccharide:Hybrid/tailoring saccharide | 33.0 | 61.3 | 81.0 | 1.08e-16 |
| APR73630.1 | esterase | BGC0001625 | Polyketide | 41.0 | 30.4 | 76.0 | 7.61e-15 |
| CCT69238.1 | uncharacterized\_protein | BGC0000030 | Polyketide | 25.0 | 63.3 | 70.0 | 8.37e-13 |
| BAK64648.1 | putative\_thioesterase | BGC0000135 | Polyketide | 30.0 | 61.1 | 67.0 | 3.69e-12 |
| BAU98033.1 | esterase | BGC0001386 | Polyketide | 40.0 | 27.6 | 67.0 | 7.43e-12 |
| BAV56003.1 | esterase | BGC0001597 | Polyketide | 35.0 | 36.4 | 67.0 | 8.17e-12 |
| ACB01058.1 | esterase/lipase | BGC0002470 | Other | 35.0 | 28.4 | 58.0 | 4.7e-09 |
| AWH12653.1 | Rmp2 | BGC0001759 | Polyketide | 35.0 | 25.4 | 53.0 | 2.5e-07 |
