# Supplementary material for: Alkaliphilic/Alkali-Tolerant Fungi: Molecular, Biochemical, and Biotechnological Aspects
Source: J Fungi (Basel). 2023 Jun 9;9(6):652. doi: 10.3390/jof9060652 (PMC10301932; doi:10.3390/jof9060652)
Supplement: Supplementary file 1 [file jof-09-00652-s001.zip › S2/knownclusterblast/region2/input.path1.gene30_mibig_hits.html]

| MIBiG Protein | Description | MIBiG Cluster | MiBiG Product | % ID | % Coverage | BLAST Score | E-value |
| --- | --- | --- | --- | --- | --- | --- | --- |
| KJA16713.1 | hypothetical\_protein | BGC0002246 | Terpene | 41.0 | 94.0 | 196.0 | 1.43e-59 |
| WP\_027148179.1 | NAD(P)-dependent\_alcohol\_dehydrogenase | BGC0001480 | Other | 37.0 | 96.9 | 171.0 | 4.17e-50 |
| WP\_234353270.1 | SDR\_family\_NAD(P)-dependent\_oxidoreductase | BGC0001537 | Polyketide | 31.0 | 94.0 | 99.0 | 3.89e-22 |
| TXD00025.1 | SDR\_family\_NAD(P)-dependent\_oxidoreductase | BGC0001877 | Polyketide | 30.0 | 100.3 | 91.0 | 1.92e-19 |
| AVX51108.1 | nysC | BGC0001709 | Polyketide | 36.0 | 74.4 | 89.0 | 1.15e-18 |
| AVV61984.1 | type\_I\_modular\_polyketide\_synthase | BGC0001477 | NRP+Polyketide:Modular type I polyketide | 30.0 | 94.0 | 85.0 | 2.07e-17 |
| RAT98527.1 | trans-acyltransferase\_polyketide\_synthase | BGC0001470 | Polyketide:Trans-AT type I polyketide | 27.0 | 95.2 | 84.0 | 2.83e-17 |
| QBK46646.1 | HrsP2 | BGC0001960 | Polyketide | 27.0 | 96.9 | 82.0 | 3.45e-17 |
| QBF51759.1 | type\_I\_polyketide\_synthase | BGC0001856 | Polyketide:Modular type I polyketide | 30.0 | 101.4 | 84.0 | 5.08e-17 |
| AFV30250.1 | polyketide\_synthase | BGC0000075 | Polyketide | 30.0 | 96.0 | 83.0 | 6.35e-17 |
| CCE88378.1 | polyketide\_synthase | BGC0001034 | NRP+Polyketide:Modular type I polyketide | 28.0 | 94.6 | 81.0 | 2.77e-16 |
| QKG20163.1 | type\_I\_polyketide\_synthase | BGC0002124 | Polyketide | 30.0 | 93.5 | 81.0 | 5.23e-16 |
| AAQ82565.1 | FscB | BGC0000034 | NRP+Polyketide | 28.0 | 94.0 | 80.0 | 9.43e-16 |
| AAZ94389.1 | modular\_polyketide\_synthase | BGC0000040 | Polyketide | 30.0 | 93.5 | 78.0 | 2.96e-15 |
| ATY46597.1 | NADPH:quinone\_reductase | BGC0001666 | Polyketide | 30.0 | 92.0 | 76.0 | 5.02e-15 |
| UPA71925.1 | crotonyl-CoA\_reductase | BGC0002636 | Polyketide | 29.0 | 88.6 | 76.0 | 1.01e-14 |
| UHY14126.1 | PKS\_I | BGC0002671 | Polyketide | 27.0 | 93.5 | 76.0 | 1.6e-14 |
| WP\_018960016.1 | crotonyl-CoA\_carboxylase/reductase | BGC0002010 | NRP+Polyketide | 28.0 | 101.4 | 74.0 | 2.47e-14 |
| AAF62880.1 | EpoA | BGC0000991 | NRP+Polyketide | 26.0 | 96.3 | 75.0 | 2.68e-14 |
| QBG82518.1 | Polyketide\_synthase | BGC0002587 | Polyketide | 38.0 | 59.7 | 75.0 | 2.87e-14 |
| ACB46192.1 | polyketide\_synthase | BGC0000989 | NRP+Polyketide | 26.0 | 96.3 | 74.0 | 4.78e-14 |
| BAC76492.1 | lankamycin\_synthase\_LkmAII | BGC0000085 | Polyketide | 33.0 | 70.2 | 74.0 | 7.14e-14 |
| AFJ52692.1 | enoyl\_reductase | BGC0001073 | NRP+Polyketide | 33.0 | 67.6 | 71.0 | 2.54e-13 |
| QLQ36607.1 | zinc-binding\_dehydrogenase | BGC0002097 | NRP+Polyketide:Type II polyketide+Saccharide:Hybrid/tailoring saccharide | 35.0 | 63.6 | 71.0 | 3.41e-13 |
| AAF26919.1 | polyketide\_synthase | BGC0000988 | NRP+Polyketide | 25.0 | 96.3 | 71.0 | 4.82e-13 |
| BAD08373.1 | polyketide\_synthase\_modules\_1-3 | BGC0000167 | Polyketide | 27.0 | 93.5 | 71.0 | 7.34e-13 |
| AAD28446.1 | unknown | BGC0000915 | Other:Aminocoumarin | 28.0 | 97.7 | 70.0 | 8.27e-13 |
| BAG85026.1 | putative\_polyketide\_synthase | BGC0000086 | Polyketide | 28.0 | 100.9 | 70.0 | 1.3e-12 |
| CAQ64686.1 | lasalocid\_modular\_polyketide\_synthase | BGC0000087 | Polyketide | 28.0 | 100.9 | 70.0 | 1.3e-12 |
| ATL73033.1 | type\_I\_modular\_polyketide\_synthase | BGC0001807 | NRP+Polyketide | 29.0 | 93.5 | 70.0 | 1.31e-12 |
| ABC84457.1 | NigAII | BGC0000114 | Polyketide:Modular type I polyketide | 28.0 | 93.5 | 69.0 | 2.89e-12 |
| ctg1\_13 |  | BGC0001931 | Polyketide | 28.0 | 96.3 | 67.0 | 1.69e-11 |
| UHH90025.1 | VicP1 | BGC0002634 | Polyketide+NRP+Other | 27.0 | 93.5 | 66.0 | 4.06e-11 |
| AAD03047.1 | type\_I\_polyketide\_synthase | BGC0000041 | Polyketide | 29.0 | 99.7 | 65.0 | 5.17e-11 |
| simG |  | BGC0000334 | NRP | 24.0 | 102.6 | 64.0 | 9.08e-11 |
| ACF35445.1 | mbcAI | BGC0000090 | Polyketide | 28.0 | 97.2 | 64.0 | 9.6e-11 |
| ctg1\_orf29 |  | BGC0000096 | Polyketide | 31.0 | 59.7 | 61.0 | 9.09e-10 |
| AEH42474.1 | polyketide\_synthase | BGC0000032 | Polyketide | 30.0 | 67.6 | 58.0 | 8.37e-09 |
| AEH42491.1 | polyketide\_synthase | BGC0000032 | Polyketide | 30.0 | 65.3 | 57.0 | 1.55e-08 |
| EJP62832.1 | polyketide\_synthase,\_putative | BGC0002203 | NRP+Polyketide+Other | 24.0 | 100.6 | 56.0 | 3.5e-08 |
| AAK19883.1 | soraphen\_polyketide\_synthase\_A | BGC0000147 | Polyketide:Modular type I polyketide | 26.0 | 104.3 | 54.0 | 1.5e-07 |
| EHA28239.1 | hypothetical\_protein | BGC0001143 | Polyketide | 28.0 | 58.8 | 53.0 | 2.28e-07 |
| AAA79984.2 | soraphen\_polyketide\_synthase\_B | BGC0000147 | Polyketide:Modular type I polyketide | 25.0 | 100.6 | 51.0 | 1.88e-06 |
