# Supplementary material for: Alkaliphilic/Alkali-Tolerant Fungi: Molecular, Biochemical, and Biotechnological Aspects
Source: J Fungi (Basel). 2023 Jun 9;9(6):652. doi: 10.3390/jof9060652 (PMC10301932; doi:10.3390/jof9060652)
Supplement: Supplementary file 1 [file jof-09-00652-s001.zip › S2/knownclusterblast/region2/input.path1.gene35_mibig_hits.html]

| MIBiG Protein | Description | MIBiG Cluster | MiBiG Product | % ID | % Coverage | BLAST Score | E-value |
| --- | --- | --- | --- | --- | --- | --- | --- |
| CBL93711.1 | Serine/threonine\_kinase | BGC0000360 | NRP | 32.0 | 22.2 | 67.0 | 7.7e-11 |
| EFG07991.1 | Putative\_serine-threonine\_protein\_kinase | BGC0000843 | Other:Non-NRP beta-lactam | 30.0 | 22.2 | 59.0 | 1.31e-08 |
| CAA09628.1 | putative\_serine-threonine\_protein\_kinase | BGC0000227 | Polyketide:Type II polyketide | 32.0 | 22.2 | 59.0 | 1.38e-08 |
