# Supplementary material for: Alkaliphilic/Alkali-Tolerant Fungi: Molecular, Biochemical, and Biotechnological Aspects
Source: J Fungi (Basel). 2023 Jun 9;9(6):652. doi: 10.3390/jof9060652 (PMC10301932; doi:10.3390/jof9060652)
Supplement: Supplementary file 1 [file jof-09-00652-s001.zip › S2/knownclusterblast/region2/input.path1.gene40_mibig_hits.html]

| MIBiG Protein | Description | MIBiG Cluster | MiBiG Product | % ID | % Coverage | BLAST Score | E-value |
| --- | --- | --- | --- | --- | --- | --- | --- |
| BAE65964.1 |  | BGC0002236 | Polyketide | 32.0 | 71.2 | 159.0 | 3.21e-44 |
| KIA75591.1 | O-methyltransferase | BGC0002209 | Polyketide | 33.0 | 59.0 | 150.0 | 7.79e-41 |
| PIB02398.1 | 6-hydroxytryprostatin\_B\_O-methyltransferase | BGC0001541 | Polyketide | 33.0 | 66.4 | 154.0 | 1.35e-40 |
| BBC83956.1 | putative\_O-methyltransferase | BGC0001636 | NRP | 44.0 | 39.1 | 145.0 | 6.02e-39 |
| ARU80379.1 | cercosporin\_toxin\_biosynthesis\_protein | BGC0001542 | Polyketide | 36.0 | 48.9 | 136.0 | 1.94e-34 |
| XP\_748078.1 | O-methyltransferase,\_putative | BGC0001990 | Other | 32.0 | 60.4 | 122.0 | 2.31e-30 |
| PIB02404.1 | 6-hydroxytryprostatin\_B\_O-methyltransferase | BGC0001541 | Polyketide | 29.0 | 72.7 | 122.0 | 2.46e-30 |
| KAF9708864.1 | hypothetical\_protein | BGC0002515 | Polyketide | 38.0 | 42.9 | 120.0 | 8.18e-30 |
| BAJ09788.1 | O-methyltransferase | BGC0000146 | Polyketide | 37.0 | 42.9 | 119.0 | 2.74e-29 |
| XP\_001798919.1 | O-methyltransferase | BGC0001865 | Polyketide:Iterative type I polyketide | 33.0 | 53.5 | 117.0 | 7.95e-29 |
| ARU80381.1 | O-methyltransferase | BGC0001542 | Polyketide | 32.0 | 54.0 | 115.0 | 6.12e-28 |
| QVK45114.1 | O-methyltransferase | BGC0002438 | Alkaloid | 31.0 | 42.0 | 90.0 | 3.34e-19 |
| EAU31622.1 | conserved\_hypothetical\_protein | BGC0002592 | Polyketide | 35.0 | 39.8 | 87.0 | 2.78e-18 |
| EAL89337.1 | O-methyltransferase,\_putative | BGC0001403 | Polyketide | 32.0 | 39.8 | 86.0 | 8.81e-18 |
| CAP12604.1 | C3\_O-methyltransferase | BGC0000219 | Polyketide:Type II polyketide+Saccharide:Hybrid/tailoring saccharide | 35.0 | 36.0 | 84.0 | 1.71e-17 |
| ERF77225.1 | hypothetical\_protein | BGC0002215 | Polyketide | 31.0 | 38.8 | 81.0 | 3.26e-16 |
| OWA01605.1 | hypothetical\_protein | BGC0001439 | Polyketide+Saccharide:Hybrid/tailoring saccharide | 28.0 | 37.4 | 74.0 | 3.92e-14 |
| DAB41656.1 | methyltransferase | BGC0001585 | Alkaloid | 32.0 | 36.7 | 69.0 | 2.27e-12 |
| QIQ51366.1 | hypothetical\_protein | BGC0002199 | Alkaloid | 31.0 | 36.2 | 64.0 | 7.19e-11 |
| BAP16697.1 | nonribosomal\_peptide\_synthetase | BGC0000376 | NRP | 29.0 | 27.1 | 60.0 | 3.04e-09 |
| QKI29064.1 | methyltransferase | BGC0002130 | RiPP:Lanthipeptide | 31.0 | 24.7 | 52.0 | 7.45e-07 |
| ADG27351.1 | methyltransferase | BGC0000296 | NRP | 31.0 | 36.7 | 49.0 | 7.2e-06 |
