# Supplementary material for: Alkaliphilic/Alkali-Tolerant Fungi: Molecular, Biochemical, and Biotechnological Aspects
Source: J Fungi (Basel). 2023 Jun 9;9(6):652. doi: 10.3390/jof9060652 (PMC10301932; doi:10.3390/jof9060652)
Supplement: Supplementary file 1 [file jof-09-00652-s001.zip › S2/knownclusterblast/region2/input.path1.gene41_mibig_hits.html]

| MIBiG Protein | Description | MIBiG Cluster | MiBiG Product | % ID | % Coverage | BLAST Score | E-value |
| --- | --- | --- | --- | --- | --- | --- | --- |
| EAU36966.1 | predicted\_protein | BGC0002274 | NRP | 43.0 | 95.4 | 645.0 | 5.17e-219 |
| AQM58276.1 | hypothetical\_protein | BGC0001816 | NRP+Polyketide | 41.0 | 95.4 | 629.0 | 1.38e-212 |
| EAU36089.1 | predicted\_protein | BGC0002273 | NRP | 41.0 | 95.1 | 592.0 | 4.45e-198 |
| CBF80711.1 | TdiA\_[Source:UniProtKB/TrEMBL;Acc:A7XRY0] | BGC0000442 | NRP | 41.0 | 97.3 | 579.0 | 7.47e-193 |
| EAU39346.1 | conserved\_hypothetical\_protein | BGC0002271 | NRP | 41.0 | 92.3 | 567.0 | 3.2e-188 |
| PLB46276.1 | acetyl-CoA\_synthetase-like\_protein | BGC0001712 | Other | 40.0 | 96.7 | 553.0 | 8.14e-183 |
| EAU31031.1 | predicted\_protein | BGC0002193 | Other | 38.0 | 95.5 | 544.0 | 1.7e-179 |
| CBF82791.1 | NRPS-like\_enzyme,\_putative\_(JCVI) | BGC0001668 | NRP | 39.0 | 90.0 | 535.0 | 7.02e-176 |
| BCN28621.1 | non-ribosomal\_peptide\_synthetase | BGC0002441 | Polyketide+NRP | 39.0 | 95.2 | 498.0 | 5.41e-162 |
| EAU36837.1 | predicted\_protein | BGC0002348 | NRP | 36.0 | 95.4 | 493.0 | 6.33e-161 |
| AHN91924.1 | EchA | BGC0000340 | NRP | 38.0 | 92.5 | 496.0 | 9.93e-161 |
| QDJ74293.1 | peptide\_synthetase | BGC0002109 | NRP | 36.0 | 95.1 | 469.0 | 5.89e-151 |
| AFB76152.1 | atromentin\_synthetase | BGC0002277 | NRP | 34.0 | 89.5 | 430.0 | 1.44e-135 |
| AAN32979.1 | BarE | BGC0000962 | NRP+Polyketide:Modular type I polyketide | 28.0 | 66.1 | 232.0 | 1.17e-62 |
| CAG23957.2 | hybrid\_NRPS/PKS\_protein | BGC0001089 | Polyketide+NRP | 25.0 | 72.0 | 214.0 | 1.79e-56 |
| ABM21569.1 | crpA | BGC0000975 | NRP+Polyketide | 26.0 | 66.2 | 209.0 | 3.63e-55 |
| antaC | NRPS | BGC0001455 | NRP+Polyketide | 30.0 | 62.4 | 206.0 | 3.56e-54 |
| ATP76245.1 | SpuA | BGC0001748 | NRP+Polyketide | 28.0 | 56.6 | 203.0 | 2.88e-53 |
| WP\_010639240.1 | non-ribosomal\_peptide\_synthetase | BGC0000958 | NRP:Cyclic depsipeptide+Polyketide:Modular type I polyketide | 29.0 | 63.0 | 190.0 | 6.97e-49 |
| ABD14712.1 | cesB | BGC0000320 | NRP:Cyclic depsipeptide | 26.0 | 59.7 | 187.0 | 4.88e-48 |
| AKA54626.1 | NRPS | BGC0001216 | NRP+Polyketide | 28.0 | 62.3 | 187.0 | 6.64e-48 |
| AHJ31217.1 | Long-chain-fatty-acid--CoA\_ligase | BGC0000430 | NRP+Polyketide:Modular type I polyketide | 27.0 | 56.7 | 185.0 | 1.01e-47 |
| WP\_081656241.1 | non-ribosomal\_peptide\_synthetase | BGC0001467 | NRP:Cyclic depsipeptide+Polyketide:Modular type I polyketide | 27.0 | 62.4 | 186.0 | 1.08e-47 |
| BBD17741.1 | non-ribosomal\_peptide\_synthetase | BGC0001918 | NRP+Polyketide | 29.0 | 58.5 | 178.0 | 4.74e-45 |
| ADH04681.1 | non-ribosomal\_peptide\_synthetase | BGC0001344 | NRP+Polyketide | 25.0 | 67.3 | 176.0 | 9.04e-45 |
| AEU11005.1 | NpnA | BGC0001029 | NRP+Polyketide | 27.0 | 56.6 | 177.0 | 1.12e-44 |
| AWX24482.1 | non-ribosomal\_peptide\_synthetase | BGC0001695 | NRP | 31.0 | 45.6 | 171.0 | 7.25e-43 |
| AHB82051.1 | polyketide\_synthase | BGC0001019 | NRP+Polyketide:Modular type I polyketide | 26.0 | 60.6 | 164.0 | 1e-40 |
| AEW95634.1 | non-ribosomal\_peptide\_synthetase | BGC0002697 | NRP+Polyketide | 27.0 | 75.2 | 161.0 | 6.34e-40 |
| ABV56587.1 | KtzG | BGC0000378 | NRP | 26.0 | 65.4 | 160.0 | 1.29e-39 |
| AIZ66879.1 | nonribosomal\_peptide\_synthetase | BGC0002666 | NRP+Alkaloid | 28.0 | 68.9 | 160.0 | 1.31e-39 |
| CAF05650.1 | TubE\_protein | BGC0001053 | NRP+Polyketide | 24.0 | 76.5 | 157.0 | 6.27e-39 |
| AAN32981.1 | BarG | BGC0000962 | NRP+Polyketide:Modular type I polyketide | 26.0 | 81.0 | 157.0 | 1.88e-38 |
| AHB82062.1 | polyketide\_synthase | BGC0001231 | NRP+Polyketide:Modular type I polyketide | 27.0 | 47.0 | 146.0 | 3.39e-35 |
| QWT72293.1 | non-ribosomal\_peptide\_synthetase | BGC0002430 | NRP+Saccharide | 26.0 | 75.9 | 144.0 | 2.1e-34 |
| ABA23460.1 | Amino\_acid\_adenylation | BGC0000427 | NRP | 24.0 | 70.5 | 137.0 | 1.02e-32 |
| ANY58984.1 | non-ribosomal\_synthetase | BGC0001615 | NRP | 27.0 | 43.0 | 137.0 | 2.19e-32 |
| QRI43520.1 | NRPS/PKS\_hybrid | BGC0002454 | Polyketide | 27.0 | 77.6 | 135.0 | 1.28e-31 |
| AAO72425.1 | syringopeptin\_synthetase\_C | BGC0000438 | NRP | 26.0 | 81.2 | 132.0 | 7.51e-31 |
| AAY37655.1 | Amino\_acid\_adenylation | BGC0000437 | NRP | 26.0 | 81.3 | 131.0 | 1.7e-30 |
| AAS47562.1 | mixed\_type\_I\_polyketide\_synthase\_-\_peptide\_synthetase | BGC0001108 | NRP+Polyketide:Trans-AT type I polyketide | 25.0 | 67.0 | 121.0 | 1.95e-27 |
| ctg1\_orf8 |  | BGC0001109 | NRP+Polyketide | 25.0 | 67.0 | 121.0 | 1.95e-27 |
| CAQ34921.1 | nonribosomal\_peptide\_synthetase | BGC0000986 | NRP+Polyketide | 23.0 | 71.4 | 118.0 | 1.22e-26 |
| CAJ34382.1 | NRPS\_protein | BGC0000445 | NRP:Cyclic depsipeptide | 27.0 | 42.8 | 117.0 | 3.51e-26 |
| QMS47800.1 | JesC | BGC0001629 | NRP:Lipopeptide | 26.0 | 87.3 | 115.0 | 1.57e-25 |
| ALK27916.1 | non-ribosomal\_peptide\_synthase | BGC0001233 | NRP | 26.0 | 61.2 | 114.0 | 4.21e-25 |
| QMN69934.1 | PsoC | BGC0002521 | NRP | 29.0 | 44.1 | 113.0 | 7.37e-25 |
| QGZ36672.1 | amino\_acid\_adenylation\_domain-containing\_protein | BGC0002082 | NRP+Polyketide | 24.0 | 69.0 | 113.0 | 7.85e-25 |
| AAF17281.1 | nosD | BGC0001028 | Polyketide+NRP:Cyclic depsipeptide | 23.0 | 67.4 | 112.0 | 8.91e-25 |
| WP\_064118616.1 | non-ribosomal\_peptide\_synthetase | BGC0002075 | Alkaloid+NRP:Lipopeptide | 24.0 | 52.6 | 111.0 | 1.59e-24 |
| extra\_gene | NRPS/PKS | BGC0002095 | NRP | 27.0 | 48.1 | 111.0 | 2.02e-24 |
| AVI26393.1 | nonribosomal\_peptide\_synthase | BGC0001800 | NRP+Polyketide | 26.0 | 45.5 | 111.0 | 2.9e-24 |
| WP\_012408786.1 | non-ribosomal\_peptide\_synthetase | BGC0002061 | NRP:Cyclic depsipeptide+Polyketide:Modular type I polyketide | 23.0 | 75.8 | 110.0 | 6.05e-24 |
| AFV52199.1 | acyl-ACP\_synthetase | BGC0000081 | NRP+Polyketide:Iterative type I polyketide+Polyketide:Enediyne type I polyketide | 27.0 | 56.1 | 108.0 | 7.95e-24 |
| QCP68969.1 | VatN | BGC0002296 | NRP+Polyketide | 28.0 | 28.1 | 109.0 | 1e-23 |
| CDG17982.1 | Non-ribosomal\_peptide\_synthetase | BGC0000464 | NRP:Cyclic depsipeptide | 24.0 | 73.6 | 108.0 | 1.99e-23 |
| CDG17987.1 | Putative\_Ornithine\_racemase\_(fragment) | BGC0000464 | NRP:Cyclic depsipeptide | 24.0 | 74.2 | 108.0 | 2.62e-23 |
| QKF54438.1 | nonribosomal\_peptide\_synthetase | BGC0002581 | NRP | 26.0 | 53.6 | 107.0 | 3.34e-23 |
| WP\_051206796.1 | LLM\_class\_flavin-dependent\_oxidoreductase | BGC0002624 | NRP+Polyketide | 33.0 | 21.6 | 107.0 | 3.93e-23 |
| ACM79812.1 | ZmaQ | BGC0001059 | NRP+Polyketide | 22.0 | 54.5 | 107.0 | 5.15e-23 |
| QWP75304.1 | non-ribosomal\_peptide\_synthase | BGC0002126 | NRP:Cyclic depsipeptide | 26.0 | 46.0 | 106.0 | 6.96e-23 |
| UEF20580.1 | nonribosomal\_peptide\_synthetase | BGC0002360 | NRP | 25.0 | 54.5 | 106.0 | 9.92e-23 |
| AYA44686.1 | icosalide\_NRPS | BGC0001833 | NRP:Lipopeptide | 28.0 | 43.8 | 106.0 | 1.02e-22 |
| ABW17377.1 | PsoC | BGC0000411 | NRP | 28.0 | 44.4 | 105.0 | 2.23e-22 |
| AHH25585.1 | AMP-dependent\_synthetase\_and\_ligase | BGC0000957 | NRP+Polyketide | 31.0 | 31.5 | 104.0 | 2.35e-22 |
| AAQ84158.1 | PlmJK | BGC0000123 | Polyketide | 30.0 | 31.6 | 103.0 | 5.53e-22 |
| QVV57688.1 | KR\_domain-containing\_protein | BGC0002338 | Polyketide | 31.0 | 21.0 | 103.0 | 5.8e-22 |
| AOA33123.1 | Nonribosomal\_peptide\_synthetase | BGC0001346 | NRP:Cyclic depsipeptide | 26.0 | 60.5 | 103.0 | 6.29e-22 |
| AED90003.1 | non-ribosomal\_peptide\_synthetase\_ThaB | BGC0000443 | NRP:Beta-lactam | 31.0 | 28.7 | 103.0 | 6.75e-22 |
| KZM73517.1 | hypothetical\_protein | BGC0000632 | Terpene+Saccharide | 24.0 | 57.2 | 101.0 | 8.49e-22 |
| AHZ34233.1 | CifB | BGC0000323 | NRP:Lipopeptide | 27.0 | 43.2 | 102.0 | 1.19e-21 |
| AXA91301.1 | non-ribosomal\_peptide\_synthetase | BGC0002044 | NRP | 26.0 | 52.5 | 101.0 | 2.45e-21 |
| ctg4\_1 |  | BGC0002017 | NRP | 26.0 | 48.0 | 99.0 | 3.34e-21 |
| WP\_100939443.1 | non-ribosomal\_peptide\_synthetase | BGC0002071 | NRP:Lipopeptide | 28.0 | 43.0 | 100.0 | 4.63e-21 |
| AAY91421.3 | non-ribosomal\_peptide\_synthetase\_OfaC | BGC0000399 | NRP:Cyclic depsipeptide | 26.0 | 72.8 | 100.0 | 5.97e-21 |
| QLY89264.1 | pseudodesmin\_synthetase | BGC0002522 | NRP | 26.0 | 44.3 | 100.0 | 7.63e-21 |
| AJF34464.1 | Txo2 | BGC0001207 | NRP | 24.0 | 69.4 | 100.0 | 8.01e-21 |
| AHD25943.1 | putative\_acyl-CoA\_ligase/oxygenase\_fusion\_protein | BGC0000208 | Polyketide | 25.0 | 51.0 | 97.0 | 2.06e-20 |
| SDF67296.1 | Acyl-CoA\_synthetase\_(AMP-forming)/AMP-acid\_ligase\_II | BGC0002422 | NRP | 25.0 | 44.9 | 98.0 | 2.12e-20 |
| CAY48789.1 | putative\_non-ribosomal\_peptide\_synthetase | BGC0001312 | NRP | 26.0 | 54.4 | 99.0 | 2.26e-20 |
| AAO56329.1 | non-ribosomal\_peptide\_synthetase\_SyfB | BGC0000435 | NRP | 28.0 | 53.0 | 99.0 | 2.36e-20 |
| CAE52339.1 | Polyketide\_non-ribosomal\_peptide\_synthase | BGC0001088 | NRP+Polyketide | 25.0 | 57.1 | 99.0 | 2.38e-20 |
| AGC65515.1 | TtcC | BGC0001876 | NRP | 23.0 | 80.0 | 98.0 | 2.83e-20 |
| QKM21620.1 | non-ribosomal\_peptide\_synthetase | BGC0002351 | NRP | 26.0 | 70.7 | 98.0 | 4.06e-20 |
| NHN68325.1 | amino\_acid\_adenylation\_domain-containing\_protein | BGC0002719 | NRP | 24.0 | 78.6 | 98.0 | 4.06e-20 |
| BAX64247.1 | NRPS | BGC0001623 | NRP+Polyketide | 24.0 | 71.1 | 97.0 | 4.76e-20 |
| AAM77987.1 | adenylate\_ligase | BGC0000112 | Polyketide:Iterative type I polyketide+Polyketide:Enediyne type I polyketide | 25.0 | 54.4 | 94.0 | 1.45e-19 |
| CAD17793.1 | probable\_non\_ribosomal\_peptide\_synthetase\_protein | BGC0001363 | NRP+Polyketide | 27.0 | 44.3 | 96.0 | 1.58e-19 |
| BCD33691.1 | non-ribosomal\_peptide\_synthetase | BGC0002448 | NRP | 23.0 | 51.8 | 94.0 | 4.57e-19 |
| AHY86403.1 | non\_ribosomal\_peptide\_synthetase | BGC0000329 | NRP | 25.0 | 78.1 | 94.0 | 5.35e-19 |
| ALD83704.1 | tAT\_polyketide\_synthase | BGC0001299 | Polyketide | 30.0 | 22.1 | 94.0 | 5.55e-19 |
| CAF05651.1 | TubF\_protein | BGC0001053 | NRP+Polyketide | 27.0 | 25.3 | 94.0 | 7.39e-19 |
| QDF82259.1 | non-ribosomal\_peptide\_synthetase | BGC0001980 | NRP | 28.0 | 42.8 | 92.0 | 1.81e-18 |
| QGQ63520.1 | nonribosomal\_peptide\_synthetase\_modules\_C | BGC0002548 | NRP | 24.0 | 61.5 | 92.0 | 2.86e-18 |
| BAC67536.1 | arthrofactin\_synthetase\_C | BGC0000305 | NRP:Lipopeptide | 28.0 | 42.8 | 92.0 | 3.11e-18 |
| ADH04682.1 | polyketide\_synthase | BGC0001344 | NRP+Polyketide | 27.0 | 31.8 | 91.0 | 4.94e-18 |
| CCJ67640.1 | TaaE | BGC0000447 | NRP:Lipopeptide | 25.0 | 46.4 | 91.0 | 5.4e-18 |
| BAX64244.1 | NRPS | BGC0001623 | NRP+Polyketide | 23.0 | 77.0 | 90.0 | 8.35e-18 |
| ABA73956.1 | putative\_non-ribosomal\_peptide\_synthetase | BGC0001842 | NRP:Lipopeptide | 26.0 | 44.2 | 90.0 | 9.05e-18 |
| KPN90376.1 | NupC | BGC0001416 | NRP | 24.0 | 67.7 | 90.0 | 1.24e-17 |
| OWA01623.1 | acyl--CoA\_ligase | BGC0001439 | Polyketide+Saccharide:Hybrid/tailoring saccharide | 26.0 | 61.4 | 88.0 | 1.6e-17 |
| AHZ34243.1 | CipF | BGC0001389 | NRP | 25.0 | 59.7 | 89.0 | 1.6e-17 |
| AFJ23826.1 | WLIP\_synthetase\_C | BGC0001838 | NRP | 26.0 | 45.2 | 87.0 | 5.89e-17 |
| WP\_064118559.1 | non-ribosomal\_peptide\_synthase/polyketide\_synthase | BGC0001509 | NRP | 26.0 | 44.2 | 87.0 | 6.12e-17 |
| QNL34618.1 | SteC | BGC0002092 | NRP:Cyclic depsipeptide | 26.0 | 43.3 | 87.0 | 6.12e-17 |
| AWI62629.1 | nonribosomal\_peptide\_synthetase | BGC0001822 | NRP | 25.0 | 28.9 | 87.0 | 6.81e-17 |
| AXG47411.1 | hybrid\_non-ribosomal\_peptide\_synthetase/type\_I\_polyketide\_synthase | BGC0002715 | NRP+Polyketide | 24.0 | 48.9 | 87.0 | 7.75e-17 |
| AFH75322.1 | nonribosomal\_peptide\_synthetase | BGC0000425 | NRP:Cyclic depsipeptide | 24.0 | 51.0 | 87.0 | 8.11e-17 |
| ATV95617.1 | CoA\_ligase | BGC0001503 | Polyketide | 24.0 | 60.6 | 85.0 | 1.18e-16 |
| ADE34495.1 | ssfL1 | BGC0000269 | Polyketide:Type II polyketide+Saccharide:Hybrid/tailoring saccharide | 25.0 | 57.1 | 84.0 | 2e-16 |
| BCJ07529.1 | hypothetical\_protein | BGC0002379 | NRP | 25.0 | 50.3 | 85.0 | 2.96e-16 |
| CAE17553.1 | acyl\_CoA\_ligase | BGC0000210 | Polyketide:Type II polyketide+Saccharide:Oligosaccharide | 25.0 | 50.7 | 84.0 | 3.33e-16 |
| AAT09805.1 | NocB | BGC0000395 | NRP | 24.0 | 58.9 | 85.0 | 3.54e-16 |
| AAC06348.1 | bacitracin\_synthetase\_3 | BGC0000310 | NRP | 23.0 | 59.8 | 85.0 | 4.09e-16 |
| BAG84248.1 | putative\_polyketide\_synthase | BGC0000257 | Polyketide | 24.0 | 57.7 | 84.0 | 4.85e-16 |
| CAK15814.1 | putative\_non-ribosomal\_peptide\_synthetase,\_terminal\_component | BGC0000344 | NRP | 28.0 | 54.4 | 84.0 | 5.27e-16 |
| CDF96614.1 | NRPS | BGC0001149 | NRP:Lipopeptide+Saccharide:Hybrid/tailoring saccharide | 22.0 | 46.9 | 84.0 | 6.37e-16 |
| AFU65902.1 | DacH | BGC0000216 | Polyketide | 26.0 | 53.8 | 82.0 | 7.69e-16 |
| AFH75330.1 | nonribosomal\_peptide\_synthetase | BGC0000398 | NRP:Cyclic depsipeptide | 22.0 | 60.5 | 83.0 | 1.56e-15 |
| ARF06222.1 | non-ribosomal\_peptide\_synthetase | BGC0001593 | NRP | 23.0 | 75.7 | 82.0 | 1.65e-15 |
| CAJ87594.1 | 2,3-dihydroxybenzoate-AMP\_ligase | BGC0001055 | NRP+Polyketide | 25.0 | 57.9 | 81.0 | 1.77e-15 |
| BAW27693.1 | NRPS(C-A-T-TE) | BGC0001764 | NRP | 24.0 | 73.1 | 82.0 | 2.82e-15 |
| WP\_069848012.1 | salicylate\_synthase | BGC0002472 | NRP | 27.0 | 41.1 | 81.0 | 4.27e-15 |
| MBN3579113.1 | amino\_acid\_adenylation\_domain-containing\_protein | BGC0002613 | NRP+Polyketide | 22.0 | 67.4 | 81.0 | 5.93e-15 |
| CAA73130.1 | Irp5\_protein | BGC0000467 | NRP | 24.0 | 57.7 | 79.0 | 7.15e-15 |
| AZF85930.1 | AMP-binding\_protein | BGC0001963 | NRP+Polyketide | 22.0 | 69.5 | 79.0 | 8.72e-15 |
| MBD2892724.1 | 2,3-dihydroxybenzoate-AMP\_ligase | BGC0002718 | NRP | 27.0 | 50.1 | 79.0 | 9.79e-15 |
| ADG27359.1 | peptide\_synthetase | BGC0000296 | NRP | 29.0 | 27.7 | 80.0 | 1.03e-14 |
| AAZ55904.1 | 2,3-dihydroxybenzoate-AMP\_ligase | BGC0000359 | NRP | 25.0 | 57.2 | 78.0 | 1.74e-14 |
| BAE98151.1 | putative\_AMP-binding\_ligase | BGC0000339 | NRP | 26.0 | 46.8 | 77.0 | 2.9e-14 |
| QDG75033.1 | mixed\_type\_I\_polyketide\_synthase\_-\_peptide\_synthetase | BGC0002068 | NRP+Polyketide | 25.0 | 57.7 | 79.0 | 3.1e-14 |
| QVQ68786.1 | mmyL | BGC0002129 | Polyketide | 26.0 | 35.0 | 77.0 | 3.67e-14 |
| AAY89051.1 | hybrid\_nonribosomal\_peptide\_synthetase/polyketide\_synthase | BGC0001069 | NRP+Polyketide:Trans-AT type I polyketide | 22.0 | 57.9 | 78.0 | 4e-14 |
| ACB12556.1 | Fum10 | BGC0000063 | Polyketide | 25.0 | 47.4 | 77.0 | 4.19e-14 |
| ACR11042.1 | Enterobactin\_synthetase\_component\_E/2,3-dihydroxybenzoate-AMP\_ligase | BGC0000451 | NRP | 24.0 | 44.9 | 77.0 | 5.22e-14 |
| CZT62785.1 | Non-ribosomal\_peptide\_synthase,\_involved\_in\_Hassallidin\_biosynthesis | BGC0001614 | NRP | 26.0 | 32.9 | 76.0 | 1.95e-13 |
| ADE34493.1 | ssfL2 | BGC0000269 | Polyketide:Type II polyketide+Saccharide:Hybrid/tailoring saccharide | 25.0 | 51.5 | 75.0 | 2.07e-13 |
| CCC55917.1 | putative\_acyl\_CoA\_ligase | BGC0000973 | NRP+Polyketide:Modular type I polyketide | 31.0 | 25.7 | 75.0 | 2.22e-13 |
| EFE73313.1 | nonribosomal\_peptide\_synthetase | BGC0000431 | NRP:Cyclic depsipeptide | 27.0 | 29.5 | 76.0 | 2.62e-13 |
| QCF28941.1 | fatty\_acyl-AMP\_ligase | BGC0002308 | Alkaloid+Polyketide | 24.0 | 36.0 | 74.0 | 3.11e-13 |
| AKQ52532.1 | nonribosomal\_peptide\_synthetase | BGC0002533 | NRP+Polyketide | 21.0 | 43.0 | 75.0 | 4.51e-13 |
| AAX98208.1 | amide\_synthetase | BGC0000052 | Polyketide | 27.0 | 38.2 | 73.0 | 6.11e-13 |
| AET98914.1 | putative\_AMP-binding\_ligase | BGC0000415 | NRP | 24.0 | 47.5 | 73.0 | 8.19e-13 |
| AGE11892.1 | 2,3-dihydroxybenzoate-AMP\_ligase | BGC0000366 | NRP | 24.0 | 45.0 | 73.0 | 9.12e-13 |
| ADB92576.1 | Ccb2 | BGC0001225 | NRP | 24.0 | 59.0 | 72.0 | 1.5e-12 |
| BCK51633.1 | modular\_polyketide\_synthase | BGC0002520 | Polyketide | 25.0 | 58.9 | 72.0 | 2.33e-12 |
| AKA59448.1 | polyketide\_synthase | BGC0001203 | NRP+Polyketide | 28.0 | 20.9 | 71.0 | 4.73e-12 |
| AEA35021.1 | hypothetical\_protein | BGC0002502 | Polyketide | 27.0 | 34.8 | 69.0 | 1.07e-11 |
| AHB38498.1 | polyketide\_synthase | BGC0000346 | NRP+Polyketide:Modular type I polyketide | 27.0 | 21.0 | 70.0 | 1.41e-11 |
| BAC87906.1 | probable\_acinetobactin\_biosynthesis\_protein | BGC0000294 | NRP | 23.0 | 56.9 | 69.0 | 1.79e-11 |
| ABW70812.1 | PchD | BGC0002475 | NRP | 30.0 | 21.0 | 69.0 | 1.83e-11 |
| EPS29069.1 | hypothetical\_protein | BGC0001724 | NRP+Polyketide | 22.0 | 62.4 | 69.0 | 2e-11 |
| AQZ26587.1 | obafluorin\_dimodular\_nonribosomal\_peptide\_synthetase | BGC0001437 | NRP | 24.0 | 61.4 | 69.0 | 2.4e-11 |
| AAO56101.1 | yersiniabactin\_synthetase,\_salicylate\_ligase\_component | BGC0002570 | NRP+Polyketide | 25.0 | 48.5 | 68.0 | 2.99e-11 |
| AGC65516.1 | NRPS/PKS\_hybrid | BGC0001050 | NRP:Lipopeptide+Polyketide:Trans-AT type I polyketide | 24.0 | 53.9 | 68.0 | 4.58e-11 |
| WP\_005009579.1 | AMP-binding\_protein | BGC0002473 | NRP | 23.0 | 57.1 | 67.0 | 7.31e-11 |
| QQZ01621.1 | long-chain\_fatty\_acid--CoA\_ligase | BGC0002497 | Other | 27.0 | 36.8 | 66.0 | 8.9e-11 |
| CAC17498.1 | putative\_AMP-binding\_ligase | BGC0000324 | NRP | 29.0 | 32.9 | 66.0 | 1.27e-10 |
| CAP20363.1 | equibactin\_siderophore\_biosynthetic\_protein | BGC0000347 | NRP | 22.0 | 57.1 | 65.0 | 2.77e-10 |
| AAN74813.2 | Fum10p | BGC0000062 | Polyketide | 26.0 | 32.9 | 64.0 | 3.81e-10 |
| OKA09423.1 | non-ribosomal\_peptide\_synthetase | BGC0001459 | NRP:Glycopeptide | 24.0 | 51.9 | 65.0 | 5.03e-10 |
| EGX96627.1 | non-ribosomal\_peptide\_synthase,\_putative | BGC0002259 | Polyketide+NRP | 22.0 | 57.7 | 65.0 | 5.18e-10 |
| AFX60341.1 | polyketide\_synthase | BGC0001032 | NRP+Polyketide | 21.0 | 25.8 | 65.0 | 5.21e-10 |
| ADD82992.1 | PtnA3 | BGC0001156 | Terpene | 25.0 | 44.3 | 62.0 | 1.45e-09 |
| AAV66110.2 | fusaridione\_A\_synthetase | BGC0000992 | NRP+Polyketide | 24.0 | 55.0 | 63.0 | 1.98e-09 |
| AAY93356.2 | non-ribosomal\_peptide\_synthetase\_PvdI | BGC0000413 | NRP | 25.0 | 36.3 | 62.0 | 2.58e-09 |
| ACG60772.1 | NRPS(C/A/PCP/Cy/A/PCP/Cy) | BGC0001058 | NRP:Glycopeptide+Polyketide:Modular type I polyketide+Saccharide:Hybrid/tailoring saccharide | 26.0 | 33.5 | 62.0 | 4.31e-09 |
| ACO31297.1 | PtmA3 | BGC0001140 | Terpene | 25.0 | 43.8 | 60.0 | 1.01e-08 |
| AFX60318.1 | polyketide\_synthase | BGC0001031 | NRP+Polyketide | 21.0 | 26.7 | 60.0 | 1.75e-08 |
| CAI94706.1 | putative\_aminocoumarin\_ligase | BGC0000141 | Polyketide | 26.0 | 22.2 | 59.0 | 1.75e-08 |
| CCC21111.1 | putative\_peptide\_synthetase | BGC0000171 | Polyketide:Modular type I polyketide | 26.0 | 38.0 | 56.0 | 1.08e-07 |
| EJP62835.1 | nonribosomal\_peptide\_synthase,\_putative | BGC0002203 | NRP+Polyketide+Other | 22.0 | 63.1 | 56.0 | 1.98e-07 |
| AAC45927.1 | 2,3-dihydroxybenzoate-AMP\_ligase | BGC0002494 | NRP | 22.0 | 51.1 | 54.0 | 8.46e-07 |
| CBF76036.1 | putative\_nonribosomal\_peptide\_synthetase\_(Eurofung) | BGC0001399 | NRP | 23.0 | 56.9 | 54.0 | 9.53e-07 |
| BAK26562.1 | PKS-NRPS\_hybrid | BGC0000977 | NRP+Polyketide | 23.0 | 57.2 | 54.0 | 9.91e-07 |
| AIW58892.1 | non-ribosomal\_peptide\_synthetase | BGC0001582 | NRP | 23.0 | 54.4 | 53.0 | 1.77e-06 |
