# Supplementary material for: Alkaliphilic/Alkali-Tolerant Fungi: Molecular, Biochemical, and Biotechnological Aspects
Source: J Fungi (Basel). 2023 Jun 9;9(6):652. doi: 10.3390/jof9060652 (PMC10301932; doi:10.3390/jof9060652)
Supplement: Supplementary file 1 [file jof-09-00652-s001.zip › S2/knownclusterblast/region2/input.path1.gene42_mibig_hits.html]

| MIBiG Protein | Description | MIBiG Cluster | MiBiG Product | % ID | % Coverage | BLAST Score | E-value |
| --- | --- | --- | --- | --- | --- | --- | --- |
| ACH72896.1 | AflO | BGC0000011 | Polyketide | 40.0 | 98.2 | 176.0 | 2.42e-53 |
| AAS90103.1 | OmtB | BGC0000006 | Polyketide | 40.0 | 99.1 | 172.0 | 6.21e-52 |
| AAS90034.1 | OmtB | BGC0000008 | Polyketide | 40.0 | 99.1 | 172.0 | 6.21e-52 |
| BAE71328.1 | o-methyltransferase\_B | BGC0000004 | Polyketide | 40.0 | 99.1 | 172.0 | 8.74e-52 |
| AAS90060.1 | OmtB | BGC0000009 | Polyketide | 41.0 | 93.3 | 172.0 | 8.74e-52 |
| AAS90012.1 | OmtB | BGC0000007 | Polyketide | 40.0 | 99.1 | 172.0 | 1.23e-51 |
| AAS90080.1 | OmtB | BGC0000010 | Polyketide | 40.0 | 99.1 | 169.0 | 9.56e-51 |
| EAU39349.1 | predicted\_protein | BGC0002271 | NRP | 37.0 | 98.2 | 159.0 | 8.22e-47 |
| PKY07885.1 | S-adenosyl-L-methionine-dependent\_methyltransferase | BGC0001544 | NRP+Polyketide | 37.0 | 94.2 | 152.0 | 4.15e-44 |
| BAE62227.1 |  | BGC0002237 | Polyketide | 37.0 | 97.3 | 149.0 | 5.37e-43 |
| EPS34232.1 | o-methyl\_transferase | BGC0002067 | NRP+Polyketide:Iterative type I polyketide | 40.0 | 93.8 | 149.0 | 1.05e-42 |
| ESU08485.1 | hypothetical\_protein | BGC0002428 | Terpene+Polyketide | 35.0 | 87.5 | 143.0 | 8.8e-41 |
| BBB04332.1 | methyltransferase | BGC0001717 | NRP | 35.0 | 100.4 | 140.0 | 1.98e-39 |
| KKP04593.1 | O-methyl\_transferase | BGC0002066 | NRP+Polyketide:Iterative type I polyketide | 39.0 | 87.5 | 136.0 | 8.06e-38 |
| API82661.1 | putative\_O-methyltransferase | BGC0001677 | Polyketide | 37.0 | 101.3 | 123.0 | 5.18e-33 |
| API82668.1 | putative\_O-methyltransferase | BGC0002616 | Polyketide | 37.0 | 101.3 | 123.0 | 5.18e-33 |
| CDM36722.1 | O-methyltransferase,\_caffeic\_acid-type | BGC0001360 | Polyketide | 36.0 | 101.3 | 120.0 | 4.69e-32 |
| ADI24956.1 | GsfD | BGC0000070 | Polyketide:Iterative type I polyketide | 35.0 | 82.6 | 113.0 | 1.88e-29 |
| AUW31187.1 | putative\_O-methyltransferase | BGC0001489 | Polyketide | 31.0 | 104.5 | 113.0 | 2.76e-29 |
| ACZ66252.1 | APS6 | BGC0000304 | NRP | 35.0 | 68.3 | 102.0 | 2.89e-25 |
| QPP19361.1 | Pen4-1 | BGC0002501 | Alkaloid | 31.0 | 94.6 | 94.0 | 2.96e-22 |
| QPP19362.1 | PenK | BGC0002501 | Alkaloid | 31.0 | 94.6 | 94.0 | 5.49e-22 |
| EHA55876.1 | hypothetical\_protein | BGC0002235 | Polyketide+NRP | 35.0 | 44.6 | 70.0 | 1.52e-13 |
| QYA95664.1 | hypothetical\_protein | BGC0002676 | NRP | 31.0 | 74.6 | 64.0 | 1.06e-11 |
