# Supplementary material for: Alkaliphilic/Alkali-Tolerant Fungi: Molecular, Biochemical, and Biotechnological Aspects
Source: J Fungi (Basel). 2023 Jun 9;9(6):652. doi: 10.3390/jof9060652 (PMC10301932; doi:10.3390/jof9060652)
Supplement: Supplementary file 1 [file jof-09-00652-s001.zip › S2/knownclusterblast/region2/input.path1.gene50_mibig_hits.html]

| MIBiG Protein | Description | MIBiG Cluster | MiBiG Product | % ID | % Coverage | BLAST Score | E-value |
| --- | --- | --- | --- | --- | --- | --- | --- |
| AIG62134.1 | patulin\_synthase | BGC0000120 | Polyketide:Iterative type I polyketide | 29.0 | 96.9 | 204.0 | 6.02e-57 |
| EIN09539.1 | pyranose\_dehydrogenase | BGC0002213 | Polyketide | 29.0 | 98.6 | 191.0 | 1.02e-52 |
| AAS90019.1 | VBS | BGC0000007 | Polyketide | 28.0 | 100.2 | 184.0 | 8.31e-50 |
| AAS90088.1 | VBS | BGC0000010 | Polyketide | 28.0 | 100.5 | 182.0 | 2.92e-49 |
| BAE71331.1 | versicolorin\_B\_synthase | BGC0000004 | Polyketide | 28.0 | 100.5 | 181.0 | 1.02e-48 |
| AAS90066.1 | VBS | BGC0000009 | Polyketide | 28.0 | 100.5 | 181.0 | 1.36e-48 |
| AAS90042.1 | VBS | BGC0000008 | Polyketide | 28.0 | 100.5 | 181.0 | 1.4e-48 |
| EAU32818.1 | predicted\_protein | BGC0000160 | Polyketide | 29.0 | 96.6 | 178.0 | 4.83e-48 |
| AAS90106.1 | VBS | BGC0000006 | Polyketide | 27.0 | 99.7 | 179.0 | 6.67e-48 |
| ACA34720.1 | CtnD | BGC0000894 | Other | 27.0 | 104.1 | 171.0 | 2.6e-45 |
| ALI92648.1 | CitC\_oxidoreductase | BGC0001338 | Polyketide:Iterative type I polyketide | 27.0 | 104.1 | 171.0 | 2.6e-45 |
| BBD84647.1 | putative\_GMC\_oxidoreductase | BGC0001775 | Terpene | 27.0 | 99.4 | 170.0 | 4.62e-45 |
| ACH72898.1 | AflK | BGC0000011 | Polyketide | 26.0 | 96.6 | 168.0 | 3.69e-44 |
| EHK18384.1 | hypothetical\_protein | BGC0002216 | Terpene | 26.0 | 101.1 | 166.0 | 7.49e-44 |
| BAQ25461.1 | putative\_dehydrogenase | BGC0001264 | Polyketide | 25.0 | 102.8 | 162.0 | 2.07e-42 |
| ctg1\_orf10 |  | BGC0000846 | Other | 29.0 | 82.2 | 163.0 | 3.4e-42 |
| KAF7526514.1 | hypothetical\_protein | BGC0002244 | Polyketide | 28.0 | 96.6 | 158.0 | 5.15e-41 |
| ATV82114.1 | GMC\_oxidoreductase/oxidase/dehydrogenase | BGC0001909 | Polyketide | 26.0 | 101.1 | 145.0 | 2.46e-36 |
| EEP98515.1 | Glucose-methanol-choline\_oxidoreductase | BGC0002091 | NRP | 28.0 | 47.1 | 81.0 | 1e-15 |
| CAD62204.1 | Ata10\_protein | BGC0000873 | Other | 29.0 | 49.8 | 80.0 | 2.05e-15 |
| AEF33092.1 | choline\_dehydrogenase | BGC0001039 | NRP+Polyketide | 28.0 | 41.3 | 79.0 | 3.82e-15 |
| AET51867.1 | oxidoreductase | BGC0001138 | Other:Nucleoside | 27.0 | 52.9 | 68.0 | 1.77e-11 |
| CAM56763.1 | hypothetical\_protein | BGC0000354 | NRP | 27.0 | 50.2 | 54.0 | 3.59e-07 |
