# Supplementary figures and images for: Alkaliphilic/Alkali-Tolerant Fungi: Molecular, Biochemical, and Biotechnological Aspects
Source: J Fungi (Basel). 2023 Jun 9;9(6):652. doi: 10.3390/jof9060652 (PMC10301932; doi:10.3390/jof9060652)

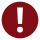

Supplement: Supplementary file 1 [file jof-09-00652-s001.zip › S2/images/bacteria_about.png]

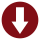

Supplement: Supplementary file 1 [file jof-09-00652-s001.zip › S2/images/bacteria_download.png]

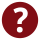

Supplement: Supplementary file 1 [file jof-09-00652-s001.zip › S2/images/bacteria_help.png]

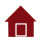

Supplement: Supplementary file 1 [file jof-09-00652-s001.zip › S2/images/bacteria_home.png]

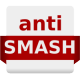

Supplement: Supplementary file 1 [file jof-09-00652-s001.zip › S2/images/bacteria_logo.png]

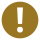

Supplement: Supplementary file 1 [file jof-09-00652-s001.zip › S2/images/fungi_about.png]

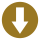

Supplement: Supplementary file 1 [file jof-09-00652-s001.zip › S2/images/fungi_download.png]

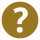

Supplement: Supplementary file 1 [file jof-09-00652-s001.zip › S2/images/fungi_help.png]

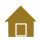

Supplement: Supplementary file 1 [file jof-09-00652-s001.zip › S2/images/fungi_home.png]

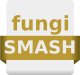

Supplement: Supplementary file 1 [file jof-09-00652-s001.zip › S2/images/fungi_logo.png]

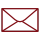

Supplement: Supplementary file 1 [file jof-09-00652-s001.zip › S2/images/mail.png]

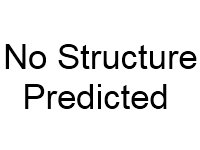

Supplement: Supplementary file 1 [file jof-09-00652-s001.zip › S2/images/nostructure_icon.png]
